# Evolutionary shifts in gene expression decoupled from gene duplication across functionally distinct spider silk glands

**DOI:** 10.1038/s41598-017-07388-1

**Published:** 2017-08-21

**Authors:** Thomas H. Clarke, Jessica E. Garb, Robert A. Haney, R. Crystal Chaw, Cheryl Y. Hayashi, Nadia A. Ayoub

**Affiliations:** 1 Department of Biology, Washington and Lee University, Lexington, VA 24450 USA; 2 Department of Biology, University of California, Riverside, CA 92521 USA; 3 J. Craig Venter Institute, Rockville, MD 28050 USA; 4 Department of Biological Sciences, University of Massachusetts Lowell, Lowell, MA 01854 USA; 5 Department of Neurology, Oregon Health & Science University, Portland, OR 97239 USA; 6 Division of Invertebrate Zoology, American Museum of Natural History, New York, NY 10024 USA

## Abstract

Spider silk synthesis is an emerging model for the evolution of tissue-specific gene expression and the role of gene duplication in functional novelty, but its potential has not been fully realized. Accordingly, we quantified transcript (mRNA) abundance in seven silk gland types and three non-silk gland tissues for three cobweb-weaving spider species. Evolutionary analyses based on expression levels of thousands of homologous transcripts and phylogenetic reconstruction of 605 gene families demonstrated conservation of expression for each gland type among species. Despite serial homology of all silk glands, the expression profiles of the glue-forming aggregate glands were divergent from fiber-forming glands. Also surprising was our finding that shifts in gene expression among silk gland types were not necessarily coupled with gene duplication, even though silk-specific genes belong to multi-paralog gene families. Our results challenge widely accepted models of tissue specialization and significantly advance efforts to replicate silk-based high-performance biomaterials.

## Introduction

**Figure 1:**
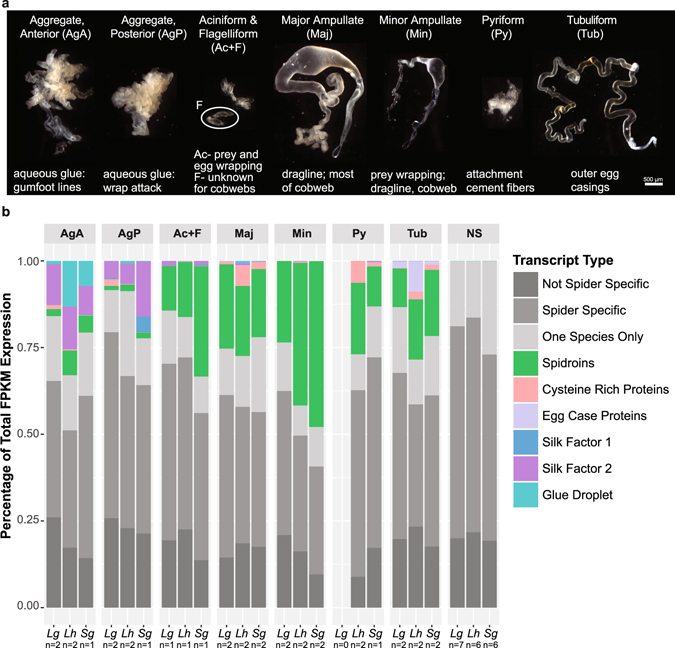
Spider silk gland sampling and proportion of total expression in spider tissues due to known silk structural protein encoding transcripts. (**a**) Representative images of silk gland types sampled (images from *L*. *geometricus*). Functions of each glands’ silk secretions also shown^31, 32, 51, 63^. Flagelliform glands synthesize the axial fiber of the capture spiral in orb-web weavers, but have an unknown function in cobweb weavers. Flagelliform glands are approximately the same size as aciniform glands in *Latrodectus* and *Steatoda*; the single pair of flagelliform glands was included with the multitude of aciniform glands in which they were nestled (Ac + F). (**b**) Proportion of total mean FPKM (fragments aligned per thousand base pairs per million aligned fragments) shown for transcripts classified according to BLASTX homology to known spider silk structural proteins (colors, see Supplementary File 1); only a spider non-silk structural protein (light grey); or to a protein found outside of spiders (dark grey, see Supplementary File 2). Transcripts without a BLASTX alignment to published proteins and that did not align with any other species’ transcripts using BlastClust are shown as “One Species Only” (lightest grey). *Lg* = *Latrodectus geometricus*, *Lh* = *L*. *hesperus*, *Sg* = *Steatoda grossa*. n = number of biological replicates for each tissue type (see also Supplementary Fig. S1). Multiple individuals were combined to generate sufficient RNA for each replicate.

Spiders (Araneae) owe their ecological success as keystone predators^1, 2^ in large part to their usage of silk^3–5^. Orb-web and cobweb weaving spiders (Araneoidea) possess multiple morphologically distinct gland types, each synthesizing a task-specific fiber or glue^6^ (Fig. 1). The material properties of spider silks are impressive, including draglines that can rival the tensile strength of steel, capture spiral filaments that can extend 300%^7, 8^, and glues that surpass synthetic glue in maintaining adhesiveness across a range of environmental conditions^9^. Because spider silks are primarily composed of proteins, they are prime targets for developing biomimetic materials through recombinant technology for industrial and medical use^10–12^. Attempts to spin artificial silks have improved in recent years, but knowledge of the molecular processes underlying silk production remains limited.

Spider silk synthesis also presents a valuable model for studying the evolution of tissue-specific gene expression and the role of gene duplication in functional novelty. Thus far, transcriptome evolution in specialized tissues has primarily been examined in mammals^13–19^. These studies largely found that gene expression profiles of thousands of orthologous genes were most similar among homologous tissues from different species, supporting a long held assumption that conserved patterns of gene expression “underlie tissue identity in mammals”^16^. However, one study found that different tissues tend to group within species^18^, questioning this basic premise. Another analysis suggested that it is not whole transcriptomes, but the expression of select sets of genes that explain mammalian tissue specialization^15^. These patterns should be examined in non-mammalians. An additional limitation of these studies is they exclusively relied on expression of strictly orthologous genes (derived from speciation), though sequence and expression divergence of paralogous genes (derived from gene duplication) are critical components of evolving new tissue functions^20, 21^.

Spider silk glands are serial homologs (duplicated anatomical units that develop at different positions within the spider’s abdomen)^22^ that have diverged in morphology and function over evolutionary time^23^ (Fig. 1a). Expression divergence is thought to be an important component of functional divergence for spider silk glands^24^. Specifically, the spidroin gene family encodes structural constituents of fibers and glues; different spidroin paralogs are primarily expressed in different gland types in association with divergent fiber functions^6, 24–26^, suggesting that the spidroin gene family co-evolved with glandular specialization^27^. For instance, tubuliform silk glands likely develop from aciniform silk glands^23, 28^ and the tubuliform spidroin (*TuSp*) and aciniform spidroin (*AcSp*) are sister paralogs^29^. The importance of gene duplication for spider silk synthesis is further supported by the finding that thousands of non-spidroin genes with silk gland specific expression come from larger than average gene families^30^. However, the relationship among morphological specialization of silk glands, gene duplication, and gene expression evolution has only been investigated for spidroins.

Here, we address these issues by profiling gene expression patterns across the different silk glands and non-silk gland tissues of three cobweb weaving spider species (Theridiidae). We focus on theridiid spiders because they belong to the Araneoidea, which possess the most diverse silk gland types for spiders. Additionally, theridiid spiders have recently specialized silk glands: the two pairs of glue-forming aggregate glands have diverged in morphology and likely function^31, 32^ (Fig. 1a). Our goals were to characterize all proteins and molecular processes contributing to silk synthesis at a gland-specific level and to test predicted patterns of transcriptome evolution for specialized tissues. First, if conserved gene expression networks are important for silk gland specialization, we predict that homologous silk gland types of the three species should group together based on gene expression levels, rather than different tissues grouping by species. Second, because silk glands are serial homologs, we predict they would group together to the exclusion of other tissue types. Furthermore, if gene expression evolution is largely neutral, similar to mammalian tissues^14, 19^, then patterns of shared gene expression among different silk gland types should reflect the evolutionary addition of those gland types. Because gene duplication is important for silk-specific functions, we suspected that orthologous genes may not capture these predicted patterns. We thus examined expression levels of (1) all orthologous genes, and (2) families of orthologs and paralags that included genes more abundantly expressed in silk glands than other tissues. We also reconstructed evolutionary shifts in gene expression among tissue types for hundreds of gene families to test if shifts between silk gland types were more likely to occur after gene duplication, as expected based on the patterns of gene expression and molecular evolution of the spidroin gene family. Our results expand models of the evolution of tissue specialization and will improve efforts to artificially recreate spider silks.

## Results

### Silk structural protein-encoding gene families differentiate glue-forming from fiber-forming gland types

We measured transcript abundance in the individual silk gland and non-silk gland tissues, including the cephalothorax (fused head-body), venom glands, and ovaries of adult females, using high-throughput sequencing of RNA (see Figs 1 and S1, & Clarke *et al*.^30^ for tissue sampling, library construction, sequencing, and transcriptome assembly). We sequenced two biological replicates for most tissue types in each of our focal species, *Latrodectus hesperus*, *L*. *geometricus*, and *Steatoda grossa*, resulting in over 1 billion high quality, non-ribosomal sequence reads (Supplementary Fig. S1).

**Figure 2:**
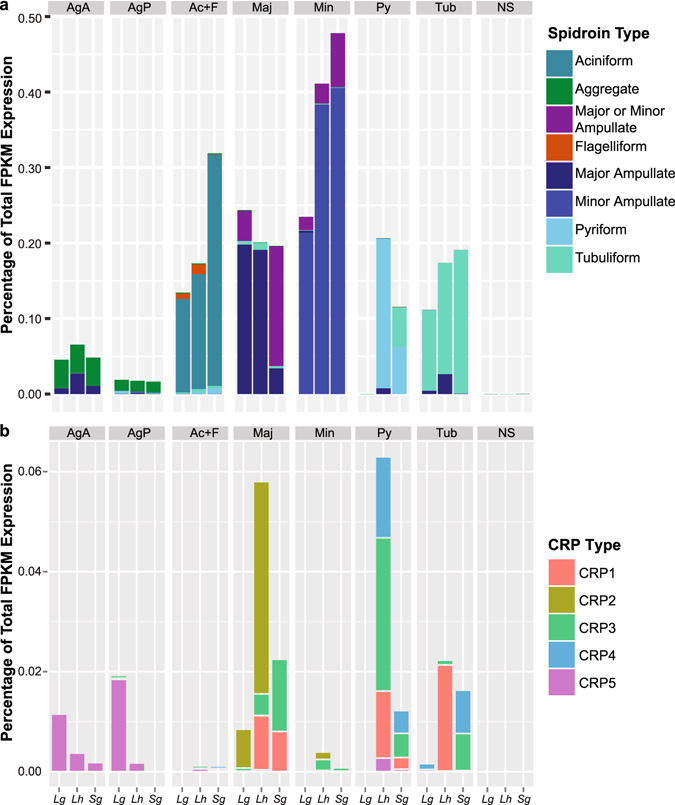
Proportion of total expression assigned to transcripts classified as a spidroin or CRP. **(a)** Spidroin classification was based on the transcript’s top BLASTX alignments to published spidroins or their membership in a BlastClust cluster with such a transcript (see Supplementary File 1). **(b)** Homologs of the five Cysteine Rich Proteins (CRPs) identified by Pham *et al*.^40^ were identified by best BLASTP match with an e-value <1e-5 (see Supplementary File 1). Gland and species abbreviations, and sample sizes, as in Fig. 1.

The specialized functions of the silk glands have largely been ascribed to a handful of known silk structural proteins^24, 25^ (Figs 1b and 2). We confirmed that the five gland types known to synthesize fibers in cobweb weavers express at least one spidroin paralog (10–50% of total mean FPKM, fragments aligned per thousand base pairs per million aligned fragments). Each of these gland types expresses the expected paralog in each of our three species (e.g. *AcSp* is almost exclusively expressed in aciniform glands, *PySp* in pyriform glands, etc.; Fig. 2a). Some paralogs are expressed in multiple gland types (e.g. major ampullate spidroin-encoding genes, *MaSp1* and *MaSp2*, have highest expression in major ampullate glands but are also expressed in tubuliform and anterior aggregate glands of *L*. *hesperus*) confirming previous indications that not every spidroin paralog is restricted to a single gland type^33–36^. Both types of glue-producing aggregate glands are clearly distinguished from the fiber-forming glands by the paucity of spidroin production (Fig. 1b), even the aggregate-specific spidroin, *AgSp1* (formerly ASG2^37, 38^; Fig. 2a).

Other proteins known to form structural constituents of fibers include the Egg Case Proteins (ECP 1 & 2)^39^ and Cysteine Rich Proteins (CRPs)^40^. We found ECPs were almost exclusively expressed in tubuliform glands, as expected, but CRPs, formerly only described from major ampullate silk^36, 40^, were expressed in each of the individual gland types (Fig. 1b) and are far more diverse than previously recognized. We identified ~50 unique transcripts with BLASTX homology to one of five published CRPs (Supplementary File 1). Some have silk gland type specificity, such as CRP2 homologs in major amplullate glands, CRP4 homologs in pyriform and tubuliform glands, and CRP5 homologs in aggregate glands. Homologs of CRP1 and CRP3 were instead found in most of the silk glands (Fig. 2b).

Constituents of the aqueous glue synthesized in aggregate glands by cobweb weavers include Aggregate Silk Factors (AgSF) 1 & 2 (found in the connection joints of webs and in the fiber-glue composite used to wrap prey)^41^ and aqueous glue droplet peptides (SCP) 1 & 2 (coat fibers of the cobweb)^42^. We found transcripts for homologs of each of these proteins in aggregate glands of all three species, but the relative abundance of these transcripts differs between the anterior and posterior aggregate glands (Fig. 1b and Supplementary File 1). Furthermore, each aggregate gland type expresses different AgSF2 paralogs, of which there are up to 10 within an individual species (Supplementary File 1).

### Expression patterns of orthologous genes differentiate some but not all tissue types

**Figure 3:**
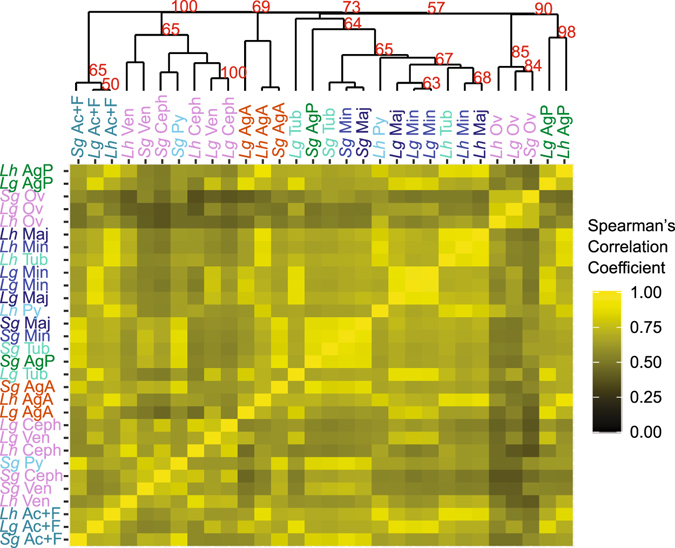
Correlation among multiple tissues for three spider species based on expression of 6550 ortholog groups. Heat maps show pairwise Spearman’s correlation coefficients based on the mean FPKM of orthologs identified with OrthoMCL. Correlation coefficients were used to hierarchically cluster the libraries (bootstrap proportions ≥50% shown). Silk gland and species abbreviations, and sample sizes, as in Fig. 1. Non-silk tissues are cephalothoraxes (Ceph), ovaries (Ov), and venom glands (Ven). There were two biological replicates for each non-silk tissue, except *Lh* Ven (n = 3).

Measuring gene expression evolution for strictly orthologous genes is straightforward compared to paralogous genes and formed the basis for transcriptome evolution studies of mammalian tissues^13–19^. We similarly identified 6550 1:1:1 orthologs among our three cobweb weavers using OrthoMCL^43^. We calculated pairwise Spearman’s correlation coefficients based on normalized FPKM of each ortholog among tissues for all three species (Fig. 3). Hierarchical clustering found statistical support for some homologous tissue groupings across species, including a well-supported cluster of aciniform plus flagelliform (Ac + F) glands, a well-supported cluster of ovaries, and a weakly supported cluster of anterior aggregate glands (Fig. 3). In contrast, clusters of non-homologous tissues were also found, including one that intermingled venom glands and cephalothoraxes of all three species and also included *S*. *grossa’s* pyriform glands. Furthermore, the major ampullate, minor ampullate, and tubuliform glands of *L*. *hesperus* grouped together as did the same set of silk glands for *S*. *grossa*. Correlation coefficients of different tissue types within and among species were generally high (min = 0.64; average = 0.71; Fig. 3), suggesting that these orthologous genes are generally expressed throughout the body of all three species. Thus, strictly orthologous genes may not be the best set of genes to understand the evolution of tissue specialization, at least not for spider silk glands.

### Suites of transcripts differentiate silk glands and coordinate expression

**Figure 4:**
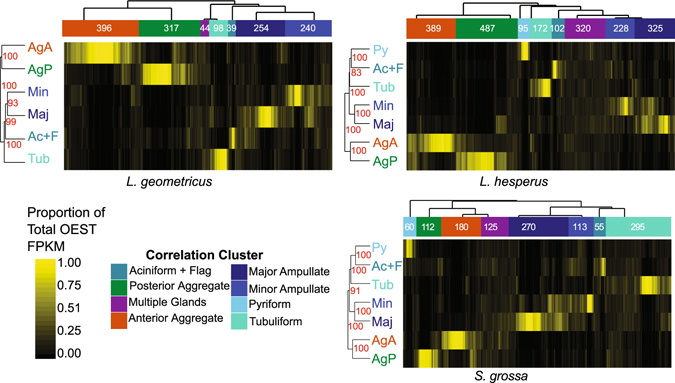
Expression patterns of over-expressed silk transcripts (OESTs). Within each focal species, transcripts significantly more abundant in silk glands than non-silk gland tissues (FDR <0.05) were identified with edgeR. The common dispersions (CV^2^) were 1.56 (*L*. *geometricus*), 1.55 (*L*. *hesperus*), 1.49 (*S*. *grossa*). OESTs were heirarchically clustered based on 1 – Spearman’s rho. Pairwise Spearman’s correlation coefficients between each OEST were calculated based on the proportion of the OEST’s mean FPKM in each of the seven silk gland types. Seven (*L*. *geometricus*) or eight (*L*. *hesperus*, *S*. *grossa*) clusters were identified and classified to an individual gland type or to mulitple glands according to majority of expression of all OESTs in the cluster (top dendrograms). The silk gland types were also heirarchically clustered according to 1 – Spearman’s rho, with correlation coefficients calculated from the proportion of OEST expression in each gland type (side dendrograms). Gland abbreviations and sample sizes as in Fig. 1.

**Figure 5:**
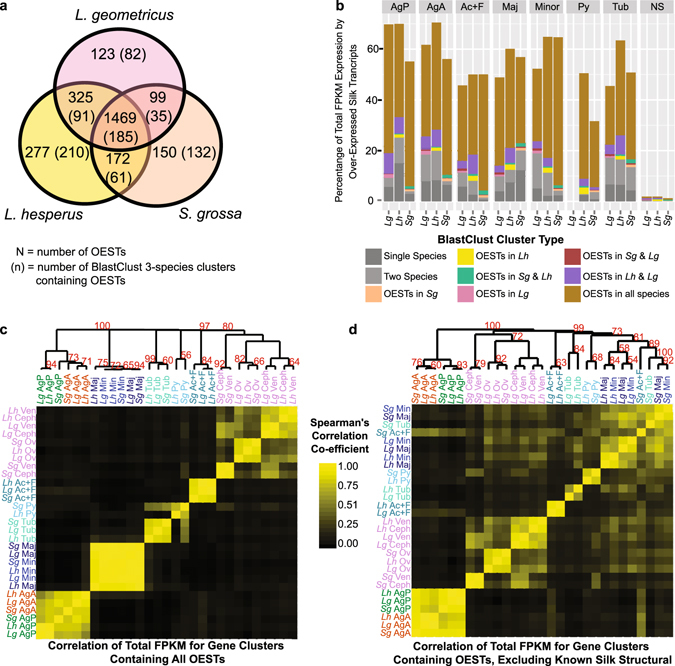
Conservation of expression patterns of over-expressed silk transcripts (OESTs) among cobweb weaving spider species. (**a**) OESTs with homologs in all three spider species were identified by clustering transcripts with BlastClust^30^ and are referred to as BlastClust 3-species clusters. Venn Diagram indicates BlastClust 3-species clusters that contained an OEST from just one species, two species, or all three species. (**b**) Proportion of total mean FPKM in each of the silk gland and non-silk (NS) tissues attributed to OESTs in BlastClust 3-species clusters (colors), OESTs with no homologs in the other two species (dark gray), and OESTs with a homolog in only one other species (light gray). **(c** and **d)** Heat maps of pairwise Spearman’s correlation coefficients based on the summed mean FPKM of BlastClust 3-species clusters containing OESTs between each tissue type sampled from the three focal species. Correlation coefficients were used for heirarchical clustering (bootstrap proportions ≥50% shown). In (**c**) all OEST-containing clusters are included. In (**d**) the clusters containing known silk structural protein-encoding transcripts have been excluded. Tissue and gland abbreviations, and sample sizes, as in Figs 1 and 3.

To identify a more suitable set of genes for examining the evolution of tissue-specific gene expression we performed statistical comparisons with edgeR^44^ based on RSEM^45^ estimated read counts. Transcripts within each species that were significantly more abundant in silk glands than non-silk gland tissues (FDR < 0.05) are referred to as over-expressed silk transcripts or OESTs (Fig. 4). Pooling silk gland types was necessary since a few of the individual silk gland types lacked a biological replicate (Figs 1b and S1). Pooling also allowed us to identify those transcripts that were consistently, even if lowly, expressed across multiple silk gland types that were not expressed in non-silk gland tissues. Comparing individual gland types to non-silk gland tissues identified those transcripts that were exclusively yet lowly expressed in that gland type in addition to most of the OESTs, but did not capture transcripts consistently but lowly expressed in multiple gland types (Supplementary Fig. S2 and Supplementary File 2). The OESTs make up 30–75% of the total expression in each gland type (Fig. 5b), typically doubling the proportion of expression compared to the known silk structural transcripts alone (compare Figs 5b to 1b).

To identify OESTs with silk gland type-restricted expression or with expression in multiple gland types, we grouped OESTs within species using hierarchical clustering (Fig. 4). Clustering based on pairwise Spearman’s correlation coefficients tended to place an OEST in the gland with its highest expression, with few OESTs falling in the “multiple glands” cluster even if expressed in more than one gland type (Fig. 4). For OESTs assigned to the aciniform plus flagelliform (Ac + F) cluster, we cannot determine if they are specialized to aciniform or flagelliform or have shared expression. However, due to the larger contribution of aciniform glands to the total RNA (Fig. 1a), most of these OESTs are likely to be primarily expressed in aciniform glands.

Functions enriched in OESTs (as defined by GO Terms) include trans-membrane export, oxidation-reduction, translation, peptidase activity, and peptidase inhibition (Supplementary File 3). Slightly more than half of the 66 (39) enriched GO Terms across all three species were found in individual silk gland types at a rate divergent from the number of GO Terms assigned to the silk gland type (Supplementary Fig. S3). Exported OESTs (possess a signal peptide) make up a large fraction of expression in silk glands (Supplementary Fig. S4). The GO Terms associated with exported OESTs are either the terms directly involved with extra-cellular activity or indicate functions potentially involved with silk processing. The latter include peptidase inhibitors and peptidase activity in the posterior aggregate gland, lipid metabolism in the major and minor ampullate glands, or proteolysis in all of the glands (Supplementary File 3).

Our hierarchical clustering strategy resulted in consistent groupings of the silk gland types across species (Fig. 4), which was supported by Principal Component Analysis (PCA) (Supplementary Fig. S5). For instance, the two types of aggregate glands always grouped in the hierarchical clustering and the first PC, and this was to the exclusion of the remaining gland types. The second PC differentiated the two types of aggregate glands. Major and minor ampullate glands also consistently grouped together. In the two species for which pyriform glands were sampled, pyriform and aciniform plus flagelliform glands grouped.

### OEST expression patterns reflect a mixture of coordinated expression among individual silk gland types, evolutionarily conserved expression levels, and rapid sequence evolution

The consistent grouping of silk gland types based on expression patterns of species-specific OESTs (Fig. 4) could result from conserved expression levels of homologous genes or could reflect coordinated expression of the gland types regardless of the homology of the individual transcripts. Ortholog groups identified with OrthoMCL^43^ captured only a fraction of the transcripts likely important for silk synthesis: only 357 of the 6550 ortholog groups contained at least one OEST and only 12% of the OESTs homologous to known silk structural encoding genes were included in an ortholog group (Supplementary Fig. S6). Thus, we expanded our analyses to include paralogous gene families by identifying groups of homologous transcripts with representatives in each of our three cobweb weaving species using BlastClust (detailed in Clarke *et al*.^30^). The majority of OESTs were members of these 3-species clusters (54.9% of all OESTs; *L*. *hesperus*: 53.9%, *L*. *geometricus*: 55.8%, *S*. *grossa*: 57.8%). Approximately 31% of the OESTs were members of 185 3-species clusters in which an OEST from all three species was represented (Fig. 5a). However, these OESTs make up a majority of the OEST expression (50–91%) of each silk gland type (Fig. 5b). The considerable proportion of expression from OESTs that were not members of 3-species clusters (3–36%) may reflect rapidly evolving genes, especially in the *L*. *hesperus* posterior aggregate glands where small glue peptides may be common^32^ and for which it might be difficult to detect cross-species homologs.

There was also a sizable proportion of expression from those OESTs in 3-species clusters with OEST representatives from the two *Latrodectus* species but not *Steatoda* (5–15%, Fig. 5b), suggesting that expression levels reflect evolutionary history to some extent. Hierarchical clustering of pairwise Spearman correlation coefficients of the total FPKM due to the BlastClust 3-species clusters containing at least one OEST among tissue types and species further supported this interpretation (Fig. 5c,d). When known silk structural transcripts (e.g. spidroins, ECPs, SCPs, CRPs, AgSFs) were included in correlation and hierarchical clustering analyses, most silk gland types and non-silk gland tissues formed tissue-specific clusters with sub-clusters reflecting species relationships (Fig. 5c). Exceptions were the major and minor ampullate silk glands, which formed a single intermingled group and the *Steatoda* posterior aggregate gland, which grouped with the anterior aggregate glands (Fig. 5c). Removing the known silk structural genes from the correlation and clustering analysis changed the patterns somewhat, but the patterns still reflected evolutionary history, albeit to a lesser extent. Specifically, the two types of aggregate glands formed separate groups and each further grouped according to species relationships (Fig. 5d). *Latrodectus* aciniform plus flagelliform, *Latrodectus* major and minor ampullate, and *Latrodectus* tubuliform also formed three distinct groups. In contrast, *Steatoda* aciniform plus flagelliform and *Steatoda* tubuliform grouped with *Steatoda* major and minor ampullate glands in their own sub-cluster. These results suggest that silk structural genes (primarily spidroins) account for the conserved gland-specific expression of most fiber-forming glands, but that there are numerous additional homologous transcripts with conserved expression in pyriform (attachment cement) and both types of aggregate glands (aqueous glues).

Groupings among gland types when considering expression levels of BlastClust 3-species clusters (Fig. 5c,d) differed somewhat from the groupings based on within-species OESTs (Fig. 4). The two types of aggregate glands strongly grouped with each other and to the exclusion of all the fiber forming glands in both types of analyses. The major and minor ampullate glands also grouped in both types of analyses, suggesting the gland types coordinate expression of individual transcripts and draw on the same gene families in all three species. However, for the remaining fiber-forming gland types there was weak grouping among subsets of gland types for the FPKM of BlastClust 3-species clusters, suggesting that the consistent grouping of aciniform plus flagelliform with pyriform glands based on within-species OESTs (Fig. 4) reflects coordinated expression of those gland types within species rather than conserved expression of homologous transcripts.

### Silk gland specific expression evolutionarily derived from expression in non-silk glands or multiple silk glands and decoupled from gene duplication

**Figure 6:**
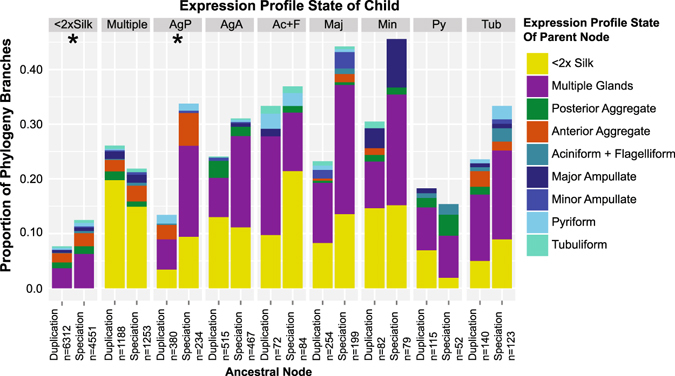
Derivation of silk gland specific expression. Classification of branches in phylogenies inferred for 605 BlastClust clusters containing representatives from all three cobweb weaving species and at least one transcript with expression levels 2-fold higher in silk glands than non-silk glands (see Fig. 7 for exemplar trees and Supplementary File 5 for all phylogenies). Each branch was classified according to whether or not there was a change in expression state between the ancestral node and the descendant. Maximum likelihood ancestral FPKM was determined for each gland type independently. If the mean FPKM was more than 2-fold greater in silk glands than non-silk glands, the node was assigned to the silk gland type with the majority of the expression, or to “multiple” glands if no majority (Supplementary File 4). Branches deriving from duplication and speciation nodes are shown seperately (n = number of nodes). Asterisks (*) denote significant differences in changing expression state between duplication and speciation nodes with Fisher’s exact test after Bonferroni multiple test correction. Fisher’s exact test is appropriate here due to the imbalance in number of speciation and duplication nodes and limited instances of changing expression states for some tissues.

To further explore evolution of expression levels, we mapped ancestral expression states onto individual gene trees (Supplementary Files 4 and 5). Instead of attempting to use statistical comparisons of ancestral expression levels to infer ancestral OESTs, we used 2-fold higher mean silk gland FPKM versus non-silk gland FPKM as a proxy for silk-gland specific expression. Within the 605 phylogenies inferred from high quality alignments that contained at least one transcript with 2-fold higher FPKM in silk glands than non-silk glands, we found most (83%) have at least one branch with an altered expression profile. Nevertheless, expression profiles tend to be strongly conserved with 86% of all branches maintaining the ancestral expression pattern (Fig. 6). Nodes inferred to have just changed to a majority of expression in one silk gland type most often descended from one of two ancestral conditions: not silk specific (e.g. <2-fold higher transcript abundance in silk gland than non-silk gland tissues) or expression in multiple silk gland types (Fig. 6). Switching the majority of expression between individual silk gland types is rare, except for between the two types of aggregate glands and from major ampullate to minor ampullate (Fig. 6). There was also considerable switching from posterior aggregate to pyriform, and from pyriform to aciniform (plus flagelliform), but this may be a by-product of not sampling pyriform glands in *L*. *geometricus*. Regardless, even for the aggregate glands the switches are not balanced: 4.9% of nodes with majority of expression in posterior aggregate glands derive from ancestral nodes with majority expression in anterior aggregate glands, but only 2.4% show the reverse pattern.

Our expectation based on the spidroin diversification model^24–26, 29^ was for expression shifts to a different silk gland type to occur more frequently after gene duplication than speciation. Surprisingly, we found that the probability of changing expression states after duplication or speciation nodes is usually similar (Fig. 6). When they do differ, as for posterior aggregate glands and the loss of silk-specific expression, the switch is significantly more likely to happen after a speciation event (Fig. 6).

**Figure 7:**
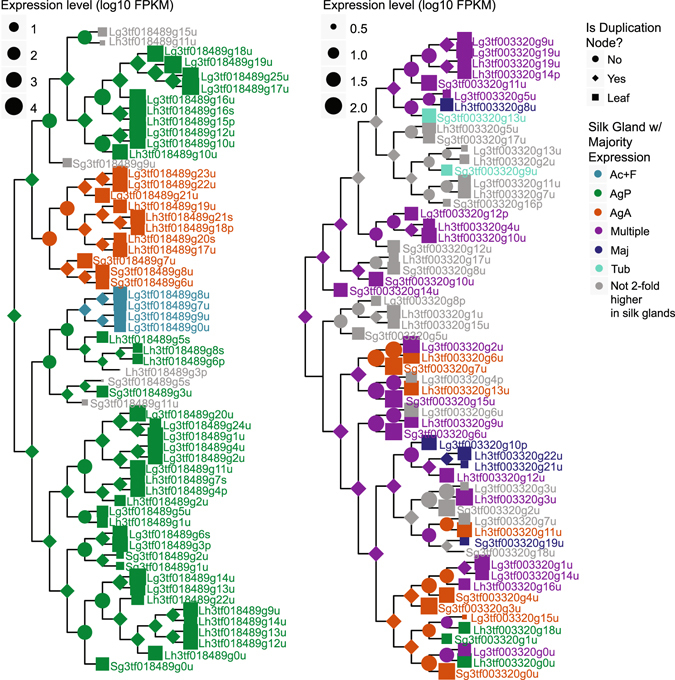
Exemplar gene trees demonstrate the reconstruction of ancestral expression states. Left tree contains homologs of the PXCC family of proteins, which are highly expressed in venom glands of spitting spiders^64^. Transcripts in the right tree encode homologs of transmembrane transport proteins. Branch lengths are ultrametric. For branch lengths reflecting molecular distances see Supplementary File 5. Horizontal width of symbols represents expression level.

We also tested for a non-random distribution of silk-gland specific expression using the D-statistic introduced in Fritz & Purvis^46^ (Supplementary File 6). If the spidroin model of diversification is common we would expect numerous gene families to include clades of silk-gland type restricted transcripts (D-statistic consistent with non-random evolution). We only found 171 gene trees containing transcripts with majority expression in more than one gland type (e.g. Fig. 7). Of these, approximately half (47%) have at least one gland type with a non-random (e.g. clustered) distribution, such as homologs of PXCC family proteins (Fig. 7). In the remaining gene families the evolution of silk-gland type specificity appears to be random, such as for the gene family containing homologs of transmembrane transport proteins (Fig. 7).

## Discussion

Here we provide the first comprehensive quantification of gene expression patterns in the functionally distinct spider silk gland types to examine the extent to which tissue specialization is driven by expression evolution. Our comparative analyses among three species of cobweb weavers also have applied importance in revealing the molecular contributors required to produce high-performance fibers and glues.

First, we propose that a number of the transcripts we identified as more abundant in silk glands than non-silk gland tissues (OESTs) are previously undocumented constituents of silk fibers and glues. OESTs make up the same or more of the transcriptional profile as known structural constituents (e.g. spidroins), most have gland-type restricted expression (Fig. 4), and many are likely exported out of the cell. While the exported OESTs with functionally validated homologs may be involved in silk processing, the 209 with no known homolog outside of spiders are excellent candidates for new constituents of silk fibers and glues. Proteomic analyses of major ampullate fibers in *L*. *hesperus* found 15 of 35 identified proteins had no published homolog or no functional information^35, 36^, further supporting this conclusion. It is also possible that some of the 209 new proteins are chaperones that assist with spidroin folding or aggregation.

In conjunction with additional proteomic analyses of other fiber types and glues, the OESTs can be used to identify the best suite of candidates for recombinant silk production, especially the aqueous glues synthesized in aggregate glands and placed on cobweb gumfoot lines that are used to capture walking prey. These glues are mixtures of glycoproteins, small peptides, and other organic and inorganic molecules, but the identities of the proteins are currently unknown^9, 32^. Our analyses suggest that aggregate glands draw on a more complex set of gene families for protein composition than do the fiber-forming glands (Figs 4 and 5), and aggregate glands have a distinct set of silk processing functions (Supplementary File 3). Despite the potential for greater complexity of protein composition than the fibers, the glues are especially promising candidates for biomimetic applications as they take on their final properties outside of the spider with minimal processing^32^, unlike the fibers, which require chemical and mechanical processing to spin from liquid dope.

Second, we provide the first transcriptome-level evolutionary analyses of gene expression in spider silk glands and one of the few comparative studies in a non-mammalian system^47^. Although gene expression can vary based on environmental factors, as suggested by differences in amino acid content of major ampullate fibers in spiders fed different prey^48, 49^, our results demonstrate a high degree of evolutionarily conserved expression patterns for homologous silk gland types based on the correlation of expression levels of whole gene families (Fig. 5) and ancestral expression state reconstruction in gene trees (Fig. 6). However, in contrast to analyses of gene expression in mammalian tissues, we found little support for the grouping of homologous silk gland types based on expression levels of strict orthologs (Fig. 3), consistent with single copy genes having a limited role in functional specialization of spider silk glands. Our findings are also consistent with recent work that suggests that conserved tissue-specific gene expression is a property of a subset of genes, rather than the entire transcriptome^15^.

Our results support several models for the evolution of gland-specific expression. Gene duplication and expression divergence of the spidroin gene family has been proposed to co-evolve with silk gland differentiation^27^. We found this model has limited application to only a few, generally highly expressed, gene families (e.g. Fig. 7, left). Across gene families that do not include known silk structural genes, we found very limited switching of over-expression in one gland type to another and that when this does occur it is equally or more likely to happen after speciation than gene duplication (Fig. 6). Furthermore, it appears that silk glands may need to draw on certain classes of proteins, but that any member of the gene family may serve equally well (e.g. transmembrane transport proteins, Fig. 7, right). One caveat is that we do not know the timing of duplication events that precede the common ancestor of our three cobweb weaving species, all of which possess all the gland types. Thus, duplication of a silk gland specific gene followed by expression divergence in newly evolved gland types may be more common than our study design can capture.

In 2004, Khaitovich *et al*.^19^ proposed that gene expression evolution was largely neutral and could reflect the evolutionary origin of specialized tissues or organs. We found little support for this model with spider silk glands. For instance, tubuliform glands develop from aciniform glands^28^, but these gland types did not group together in any analyses of correlated expression patterns (Figs 3, 4 and 5). Another interesting case was the aggregate glands, which are the most recent evolutionary additions to the spider silk gland toolkit found only in araneoid spiders. We expected these gland types to group with fiber-forming glands to the exclusion of other tissue types in all analyses of correlated expression levels, but did not find that pattern for all orthologs (Fig. 3) or for gene families containing OESTs (Fig. 5c,d). The lack of silk gland grouping based on ortholog expression levels likely results from the limited contribution of these single copy genes to silk gland specialization, but the analysis of multi-copy gene families (Fig. 5c,d) suggests that gene expression in most types of silk glands may evolve largely independent of other types.

Nevertheless, aggregate gland expression was partially consistent with the expectation that gene expression patterns reflect the evolutionary history of tissue specialization. For instance, the two types of aggregate glands grouped together based on correlated gene expression levels within each species (Fig. 4) and total expression levels of gene families containing OESTs (Fig. 5c,d). Also, the two types of aggregate glands in cobweb weavers are transcriptionally distinct (Figs 3, 4 and 5), as expected given their recent morphological and functional specialization relative to orb-web weavers (Fig. 1a)^31, 32^. Intriguingly, a number of transcripts with posterior aggregate-specific expression are derived from ancestral nodes inferred to have anterior aggregate-specific expression (Fig. 6). This pattern is consistent with Khaitovich *et al*.’s model^19^, which would propose that derivation of posterior aggregate-specific expression coincided with morphological divergence of this gland type. However, this interpretation is refuted by our finding that most switches of expression to posterior aggregate glands occurred after speciation of one of our three cobweb weavers while morphological divergence of the posterior aggregate glands, inferred from changes in spigot morphology, almost certainly happened in the common ancestor of Theridiidae^50^. Instead, we propose that the similar functions of the two types of aggregate glands (glue formation) necessitate coordinated expression and co-evolution.

Further evidence that correlated expression patterns among silk gland types reflects coordinated functions rather than the evolutionary addition of gland types comes from the major and minor ampullate glands. These gland types are documented in most true spiders (Araneomorphae), yet they grouped together within each species based on shared expression of individual transcripts (Fig. 4) and the correlation of expression levels of gene families (Fig. 5c,d). In cobweb weavers, major ampullate fibers form the primary dragline and most of the cobweb, while minor ampullate silks are the most abundant fibers used to wrap prey^51^. However, the use of major ampullate silk likely coincides with the use of minor ampullate silk. For instance, some major ampullate fibers have been found wrapped around prey^51^ and minor ampullate silks form the inner fiber of gumfoot lines, which are subsequently covered with major ampullate fibers and aggregate glue droplets^52^. Thus, it appears that major and minor ampullate glands perform tightly integrated functions, necessitating coordinated gene expression of these two gland types.

In conclusion, we have dramatically increased our understanding of spider silk genetics. Because the expression patterns are broadly conserved among the different gland types across our three species, they are likely important for spider silk synthesis. Moreover, our results support multiple function-driven models of gene expression evolution in specialized tissues.

## Methods

### Tissue Sampling

Up to 29 adult female individuals were used for each species per tissue replicate (8–10 more typical) to obtain sufficient RNA for library construction. Tissues were sampled from the same sets of individuals as detailed in Supplementary Table S1 of Clarke *et al*.^30^. Individuals were arbitrarily assigned to either replicate 1 or 2.

### Transcript Annotation

High-quality transcripts from our previous assemblies (TSA: GBJM0000000, GBJN00000000, GBJQ00000000) were annotated by homology to published proteins using BLASTX. We defined a transcript as spider-specific if there were no BLASTX alignments to a UniProt sequence outside the Araneae with an e-value <1e-5. A transcript was defined as encoding a known silk structural protein if it had a best BLASTX match ≤1e-5 to a UniProt defined spider silk protein, it had a best BlastP match to a Cysteine Rich Protein (CRP)^40^, or it was in the BlastClust cluster with such a transcript (Supplementary File 1).

GO Terms were assigned to transcripts based on their best UniProt homologs^53^ (Supplementary File 3). GoTerm enrichment in silk glands was determined using GoSeq.^54^, which accounts for length bias in RNA-seq. We identified 66 GO Terms^55^ enriched in the OESTs combined from all three species compared to all of the transcripts with >1 FPKM in at least one tissue using GoSeq.^54^ (Supplementary File 3). Similar enrichment analyses on each species individually identified only 43 total GO Terms enriched for the OESTs, of which only 7 were shared among all three species, suggesting that combining the species’ OESTs is required for sufficient power to detect conserved functions in the silk glands (Supplementary File 3). Signal peptides were identified by SignalP v 4.1^56^ for predicted M-started proteins.

### Expression Analyses

Transcript abundance was estimated by aligning processed (adapters, low quality sequences, rRNA sequences, and reads lacking a pair removed)^30^ raw paired-end sequence reads from each species tissue-specific library to our transcriptomes using RSEM^45^. RSEM provides an estimate of the number of sequence reads that originated from a given transcript, accounting for the possibility that a single read could align to multiple transcripts. Transcripts significantly over-expressed in the silk glands were identified by comparing all silk gland libraries to all non-silk gland libraries in each of the three species using edgeR on RSEM’s expected read counts normalized with the Trimmed Mean of M (TMM) method^57^. Transcripts with <1 count per million reads in every library were removed. The biological coefficient of variation among samples was approximated by the common dispersion parameter in edgeR’s negative binomial model, followed by estimation of transcript-wise dispersion parameters. Transcripts that were both over-represented in the silk libraries and had an FDR <0.05 were classified as Over-Expressed Silk Transcripts (OESTs).

We also compared each silk gland type to non-silk gland tissues using EdgeR (Supplementary File 2). When no biological replicates were available for a silk gland type we set the dispersion to 0.4 to approximate the biological coefficient of variation. Transcripts ≥2-fold higher in silk glands than other tissues were also identified by EdgeR.

For comparisons of expression levels among transcripts and species, we used RSEM calculated fragments per RSEM defined “effective” kilobase per million aligned reads (FPKM) for each transcript. FPKM for each transcript in each libary was normalized by multiplying by the library-specific normalization factor identified with TMM. Replicate tissues, when available, had high Spearman’s correlation coefficients based on normalized FPKMs of all transcripts (Supplementary Fig. S7 and Supplementary File 7). Remaining analyses were thus based on the mean FPKM for each transcript in each tissue (average of the normalized FPKM for the transcript in each tissue-specific library associated with the species).

To identify OESTs with silk gland type-restricted expression or with expression in multiple gland types, we grouped within species OESTs using hierarchical clustering. Distances for hierarchical clustering were 1 – Spearman’s rho, with pairwise Spearman’s correlation coefficients calculated from the proportion of mean FPKM of the transcripts in each of the seven silk gland types (Fig. 4). We split the hierarchical clusters into the seven or eight largest groups (number of silk gland types assayed plus one) and assigned the group to an individual gland type if the summed expression of all the OESTs in the cluster was >50% due to that gland type. Otherwise, the cluster was designated as “multiple glands”.

We also hierarchically clustered the individual silk gland types by the pairwise Spearman’s correlation coefficients calculated from the proportion of expression of the OESTs in each gland type. Bootstrap support for clusters were computed by the boot.phylo function in the APE R package^58^ with Spearman’s correlation-based hierarchical clustering with 1000 replicates. Principal Components Analysis was performed on the mean FPKM values of all species OESTs in the silk gland types and the non-silk tissues in R using the prcomp function.

### Evolutionary analyses of expression patterns

Homologous transcripts (orthologs and paralogs) were identified using BlastClust as detailed in Clarke *et al*.^30^. Evolutionary expression analyses were restricted to BlastClust clusters with a representative from each species (BlastClust 3-species clusters). We also identified 1:1:1 orthologs among the three species using OrthoMCL with default parameters^43^. To compare expression levels of homologous transcripts among species, we summed the edgeR normalized FPKM within each tissue-type of every transcript in each BlastClust 3-species cluster in a species-specific manner. Pairwise Spearman’s correlation coefficients between each species-tissue combination were calculated. Transcript abundance of 1:1:1 orthologous transcripts was calculated by re-aligning raw reads to only these sequences. The resulting FPKMs of each ortholog were normalized by edgeR (TMM) and used to calculate pairwise Spearman’s correlation coefficients.

For ancestral expression state analyses we focused on the 630 BlastClust 3-species clusters with at least one transcript in each species that was 2-fold higher in silk glands than non-silk glands and mean FPKM >1 in at least one tissue. We added tick homologs to these BlastClust clusters when they could be identified as described in Clarke *et al*.^30^.Transcripts from the clusters were aligned, trimmed of low information positions using trimAl^59^, and removed if the remaining positions were >25% gaps. The phylogenies of the remaining transcripts were inferred with TreeBeST (see Clarke *et al*.^30^). Since silk glands evolved in spiders after the divergence of tick and spiders, for those phylogenies with tick homologs, the trees were sub-divided at each tick–spider speciation node, from which we used only the spider descendants, resulting in 605 gene trees. For each transcript remaining in the phylogenies, we coded expression patterns as the mean FPKM within each silk gland type and non-silk gland tissue. We inferred the maximum likelihood ancestral states of each tissue type independently, assuming a Brownian motion model of evolution^60^, using PHYTOOLS^61^ in R. Ancestral nodes with FPKM 2-fold higher in silk glands than non silk glands were further classified to individual gland types: if an ancestral node had >50% of its FPKM in one silk gland type it was assigned to that type, otherwise it was assigned to “multiple glands”.

We tested if changes in expression patterns were non-randomly associated with speciation versus duplication events for each of the gland types using Fisher’s exact test in R and assigned significance after correcting the p-value using Bonferroni multiple test correction. We also examined whether silk-over expression is non-randomly concentrated within each phylogeny inferred above using the D-statistic^46^ implemented in the Caper R package^62^. Because the D-statistic can only be computed for binary characters, each transcript and internal node was assigned as either 2-fold higher in silk or not. The D-statistic was recalculated seven times for each tree classifying the transcripts or nodes as majority expression in a single silk gland type or not.

### Data Availability

RSEM estimated read counts and normalized FPKM are available through the Gene Expression Omnibus, with links to raw reads in the Short Read Archive (GEO series GSE95367).

## Electronic supplementary material

Supplementary Figures

Supplementary File 1

Supplementary File 2

Supplementary File 3

Supplementary File 4

Supplementary File 5

Supplementary File 6

Supplementary File 7

Supplementary File 8
