# Supplementary material for: Evolutionary shifts in gene expression decoupled from gene duplication across functionally distinct spider silk glands
Source: Sci Rep. 2017 Aug 21;7:8393. doi: 10.1038/s41598-017-07388-1 (PMC5566633; doi:10.1038/s41598-017-07388-1)
Supplement: Supplementary file 1 — Supplementary Figures [file 41598_2017_7388_MOESM1_ESM.pdf]

Supplementary figures for

Evolutionary shifts in gene expression decoupled from gene duplication across functionally distinct spider silk glands

Thomas H. Clarke, Jessica E. Garb, Robert A. Haney, R. Crystal Chaw, Cheryl Y. Hayashi, Nadia A. Ayoub

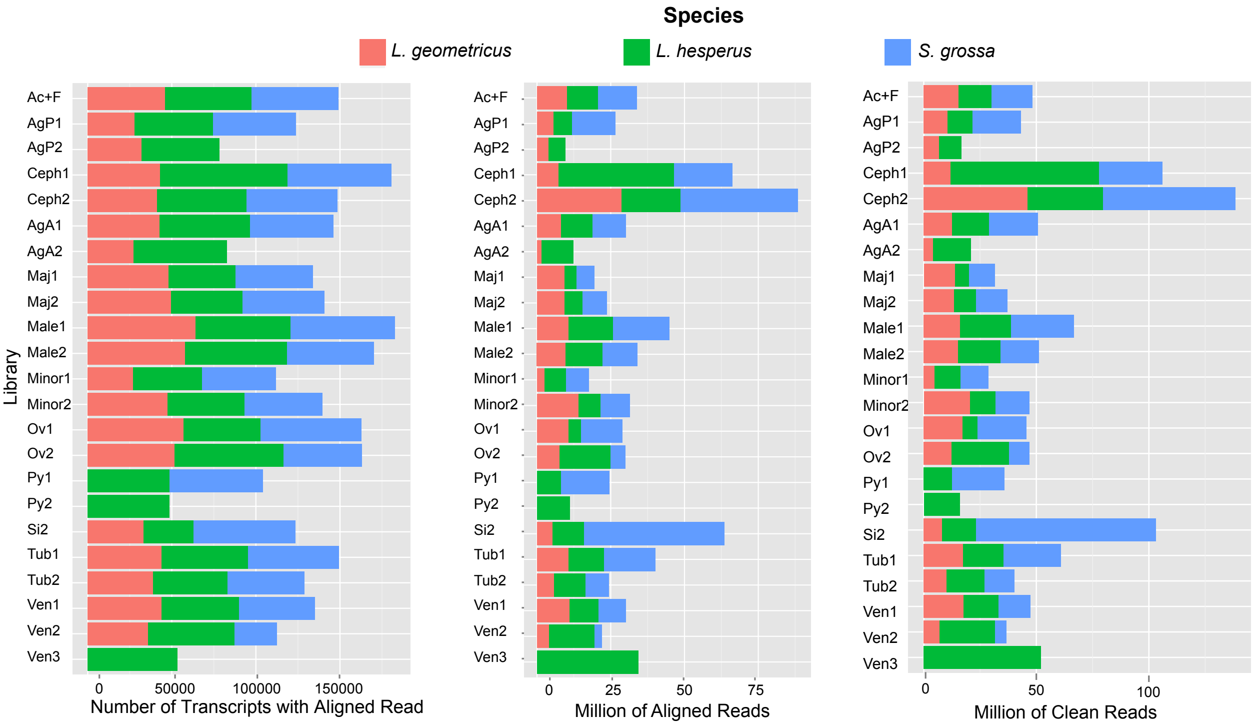

Supplementary Figure S1. **Sequencing depth in three cobweb weaving species in different tissue-specific RNA-seq libraries**. Left panel: In each library, the number of transcripts for which RSEM mapped at least one pair of reads. Transcripts assembled in Clarke et al.[1] and RSEM run with default parameters. Middle panel: The number of high quality non-ribosomal reads for each library obtained from paired end Illumina sequencing that aligned to a transcript. Right panel: The number of high quality non-ribosomal reads for each library obtained from paired end Illumina sequencing. Libraries derive from silk gland tissues (Ac+F=Aciniform and Flagelliform, AgP=Posterior Aggregate, AgA=Anterior Aggregate, Maj = Major Ampullate, Minor=Minor Ampullate, Py = Pyriform, Si = all silk glands combined, Tub=Tubuliform) and non-silk gland tissues (Ceph=Cephalothorax, Ov = Ovary, Ven=Venom) obtained from dissections of adult females, and from whole body adult males (Male).

Note that anterior aggregate glands referenced in this study are equivalent to the “flagelliform” glands referenced in Clarke et al.[1]. This study followed gland nomenclature in Jeffery et al.[2], in which only one pair of aggregate glands was identified. Subsequently, we performed a more extensive search of the silk glands of our target species because of multiple reports in the literature of two pairs of aggregate glands in araneoid spiders, including cobweb weavers[3–5]. We found a pair of glands morphologically similar to orb-weaver flagelliform glands, but approximately the same size as aciniform glands (see Figure 1 in main manuscript). The glands named “flagelliform” by Jeffery et al. were morphologically similar to the description of anterior aggregate glands in black widows and both pairs of aggregate glands in orb-weavers[4,5].

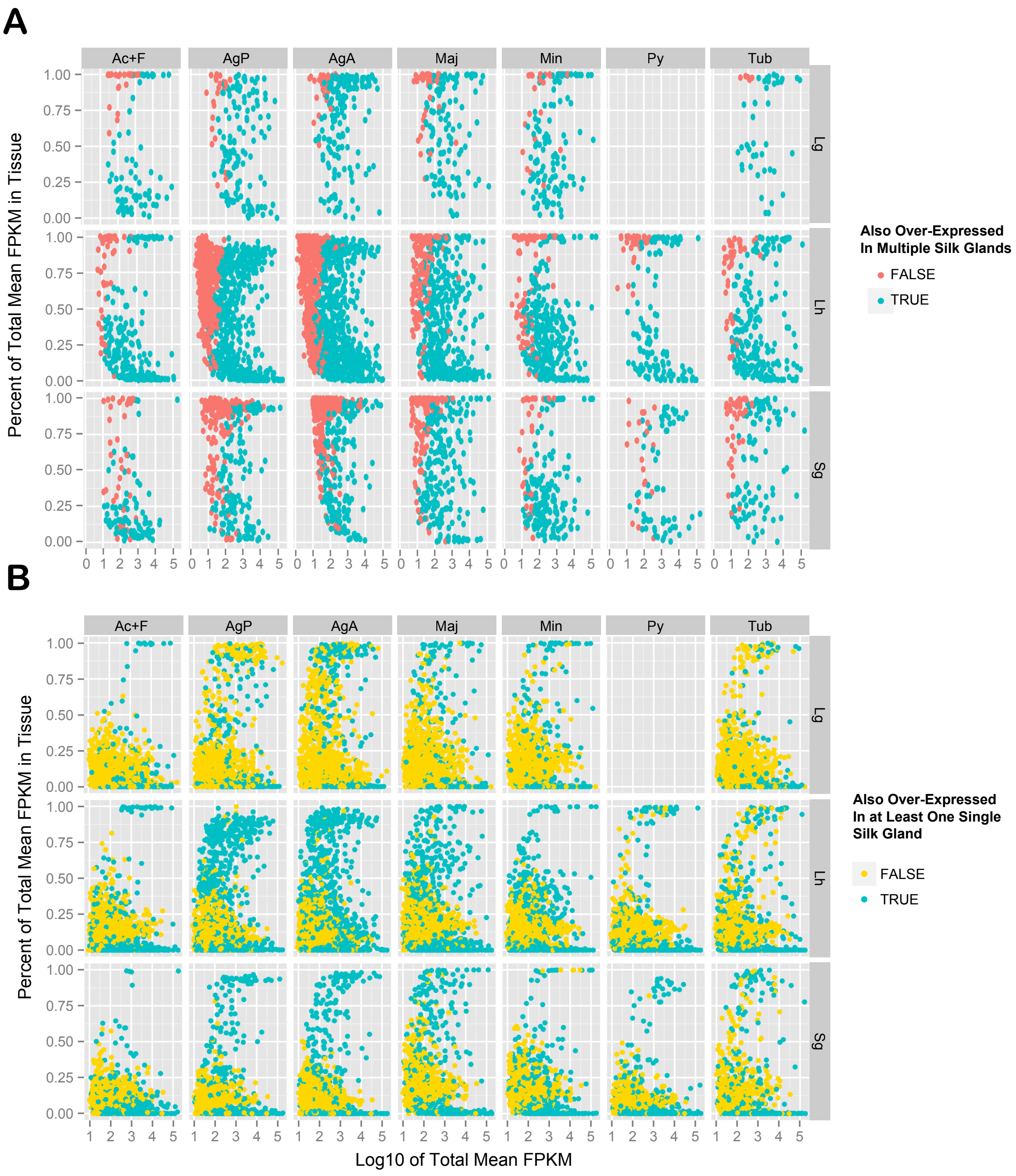

Supplementary Figure S2. **Comparison of transcripts identified as over-expressed within a single gland type versus all of the silk glands combined.** Transcripts more abundant in silk glands were identified by edgeR for each species by comparing the non-silk gland tissues (cepholathorax, ovary, venom) to either each of the silk gland types individually (single silk gland, **A**) or all of the silk glands at once (multiple silk gland, **B**). Transcripts only found to be over-expressed in the single gland comparisons (red points, **A**) were generally lowly and almost exclusively expressed in the single gland compared to transcripts found over-expressed in multiple gland comparison as well (blue points in **A** & **B**). Ac+F=Aciniform and Flagelliform, AgP=Posterior Aggregate, AgA=Anterior Aggregate, Maj = Major Ampullate, Minor=Minor Ampullate, Py = Pyriform, Tub=Tubuliform

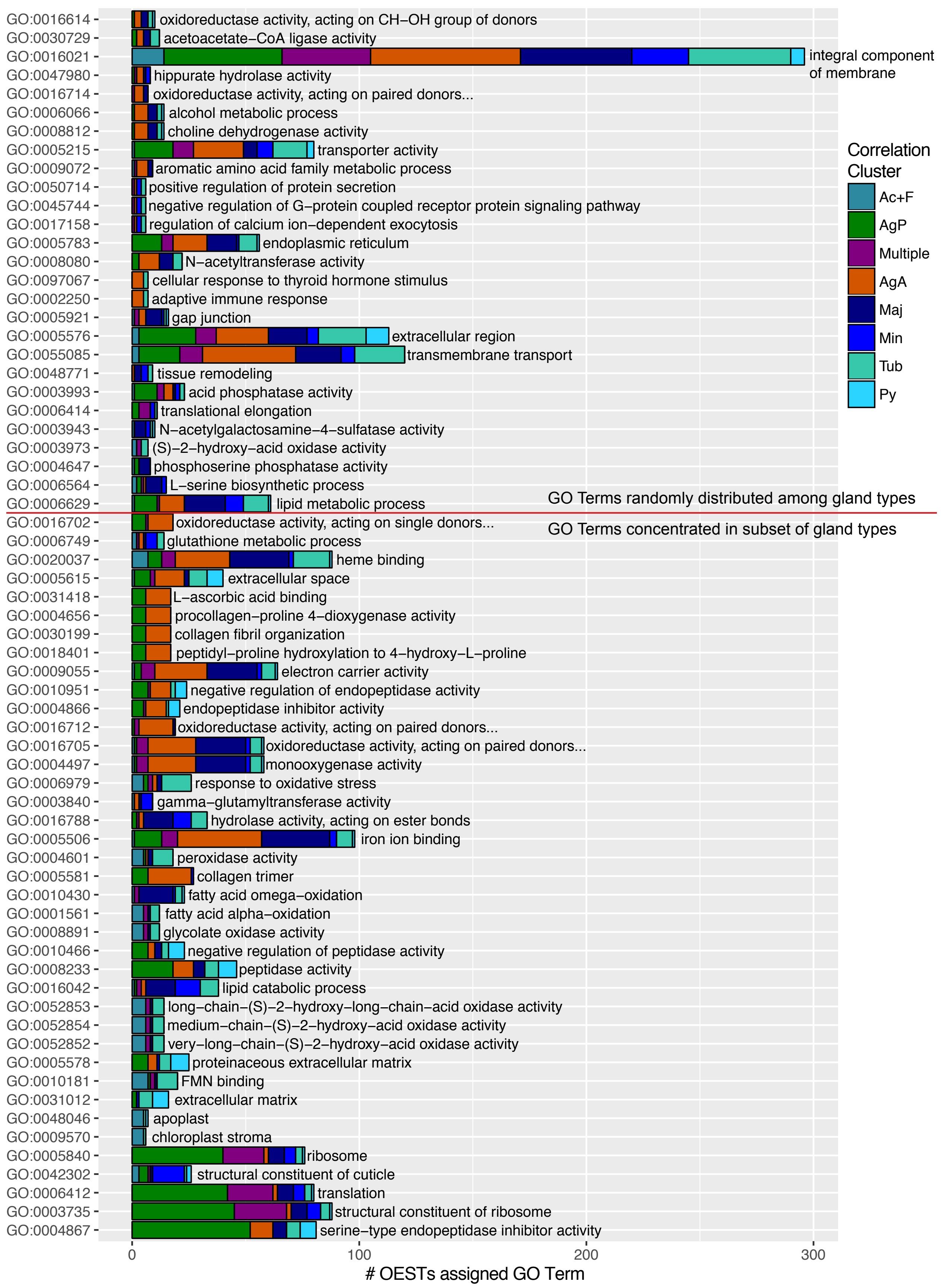
GO:0016614 oxidoreductase activity, acting on CH−OH group of donors
GO:0030729 acetoacetate−CoA ligase activity
GO:0016021 integral component of membrane
GO:0047980 hippurate hydrolase activity
GO:0016714 oxidoreductase activity, acting on paired donors...
GO:0006066 alcohol metabolic process
GO:0008812 choline dehydrogenase activity
GO:0005215 transporter activity
GO:0009072 aromatic amino acid family metabolic process
GO:0050714 positive regulation of protein secretion
GO:0045744 negative regulation of G−protein coupled receptor protein signaling pathway
GO:0017158 regulation of calcium ion−dependent exocytosis
GO:0005783 endoplasmic reticulum
GO:0008080 N−acetyltransferase activity
GO:0097067 cellular response to thyroid hormone stimulus
GO:0002250 adaptive immune response
GO:0005921 gap junction
GO:0005576 extracellular region
GO:0055085 transmembrane transport
GO:0048771 tissue remodeling
GO:0003993 acid phosphatase activity
GO:0006414 translational elongation
GO:0003943 N−acetylgalactosamine−4−sulfatase activity
GO:0003973 (S)−2−hydroxy−acid oxidase activity
GO:0004647 phosphoserine phosphatase activity
GO:0006564 L−serine biosynthetic process
GO:0006629 lipid metabolic process
GO Terms randomly distributed among gland types
GO Terms concentrated in subset of gland types
GO:0016702 oxidoreductase activity, acting on single donors...
GO:0006749 glutathione metabolic process
GO:0020037 heme binding
GO:0005615 extracellular space
GO:0031418 L−ascorbic acid binding
GO:0004656 procollagen−proline 4−dioxygenase activity
GO:0030199 collagen fibril organization
GO:0018401 peptidyl−proline hydroxylation to 4−hydroxy−L−proline
GO:0009055 electron carrier activity
GO:0010951 negative regulation of endopeptidase activity
GO:0004866 endopeptidase inhibitor activity
GO:0016712 oxidoreductase activity, acting on paired donors...
GO:0016705 oxidoreductase activity, acting on paired donors...
GO:0004497 monooxygenase activity
GO:0006979 response to oxidative stress
GO:0003840 gamma−glutamyltransferase activity
GO:0016788 hydrolase activity, acting on ester bonds
GO:0005506 iron ion binding
GO:0004601 peroxidase activity
GO:0005581 collagen trimer
GO:0010430 fatty acid omega−oxidation
GO:0001561 fatty acid alpha−oxidation
GO:0008891 glycolate oxidase activity
GO:0010466 negative regulation of peptidase activity
GO:0008233 peptidase activity
GO:0016042 lipid catabolic process
GO:0052853 long−chain−(S)−2−hydroxy−long−chain−acid oxidase activity
GO:0052854 medium−chain−(S)−2−hydroxy−acid oxidase activity
GO:0052852 very−long−chain−(S)−2−hydroxy−acid oxidase activity
GO:0005578 proteinaceous extracellular matrix
GO:0010181 FMN binding
GO:0031012 extracellular matrix
GO:0048046 apoplast
GO:0009570 chloroplast stroma
GO:0005840 ribosome
GO:0042302 structural constituent of cuticle
GO:0006412 translation
GO:0003735 structural constituent of ribosome
GO:0004867 serine−type endopeptidase inhibitor activity
0
100
200
300
OESTs assigned GO Term
Correlation Cluster
Ac+F
AgP
Multiple
AgA
Maj
Min
Tub
Py

Supplementary Figure S3. **Number of OESTs assigned a GO Term that was enriched in OESTs compared to all transcripts.** OESTs are color-coded according to the silk gland type assigned in correlation clustering (see Fig 4). GO terms are ranked from bottom to top by the p-value associated with a chi-square test comparing the observed distribution of a GO Term in the different silk gland types to the expected distribution based on the total number of GO Terms assigned to each silk gland type. P-values below the red line are less than the Bonferroni corrected alpha. See Supplementary File 6 for full list of GO Term assignments.

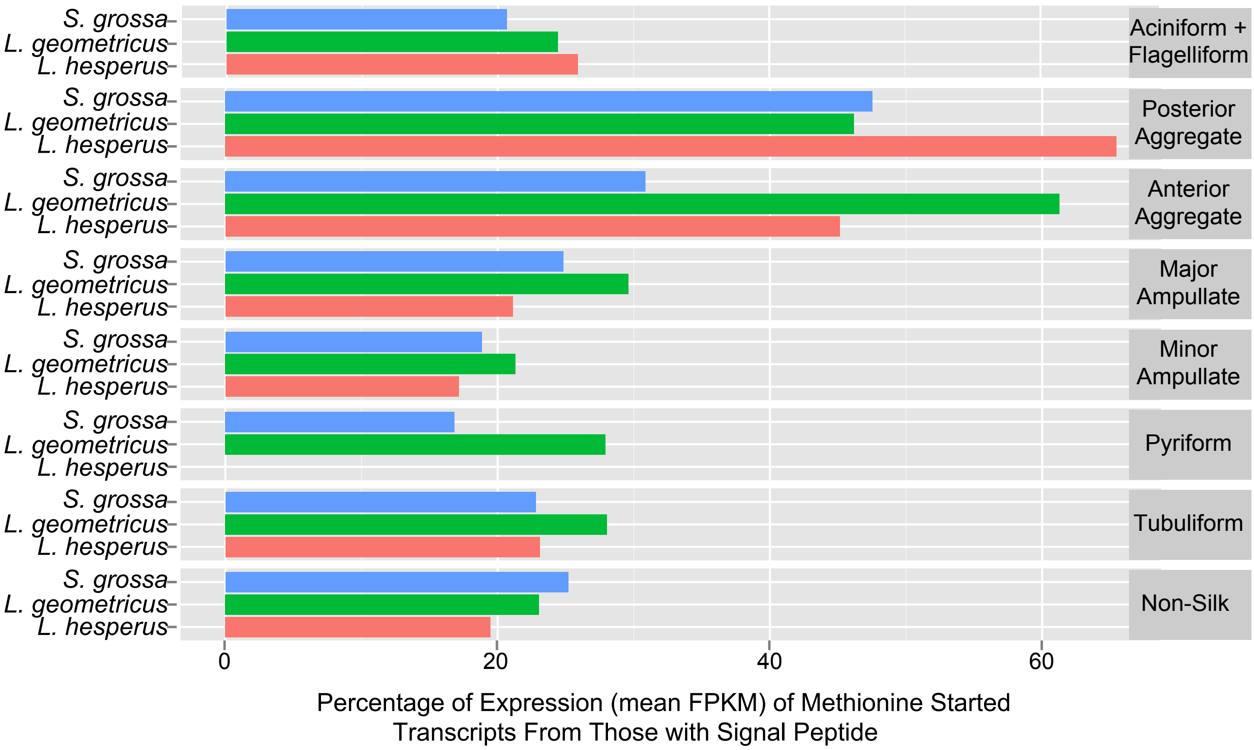

Supplementary Figure S4. **Proportion of expression devoted to transcripts predicted to encode signal peptides.** The average percentage of FPKM in each silk gland type from transcripts whose translation starts with a methionine that had a signal peptide as identified by SignalP (v4.1[6]). Because silk fibers and glues function outside of the spider we expected that structural proteins and any proteins associated with processing these structural constituents would have signal peptides to direct their extracellular transport. Consistent with this expectation, we found that of transcripts predicted to encode complete proteins, OESTs are more likely to include a signal peptide than all the other proteins, a pattern that is consistent across all the silk gland types (Fisher's Exact Test, FDR < 0.001 for all OEST clusters identified in Fig 4). OESTs assigned to the pyriform glands have the highest proportion of signal peptides (54% versus 30-43% of OESTs assigned to other silk gland types and 11% in OESTs assigned to multiple glands), but overall aggregate glands devote more of their expression to proteins containing signal peptides.

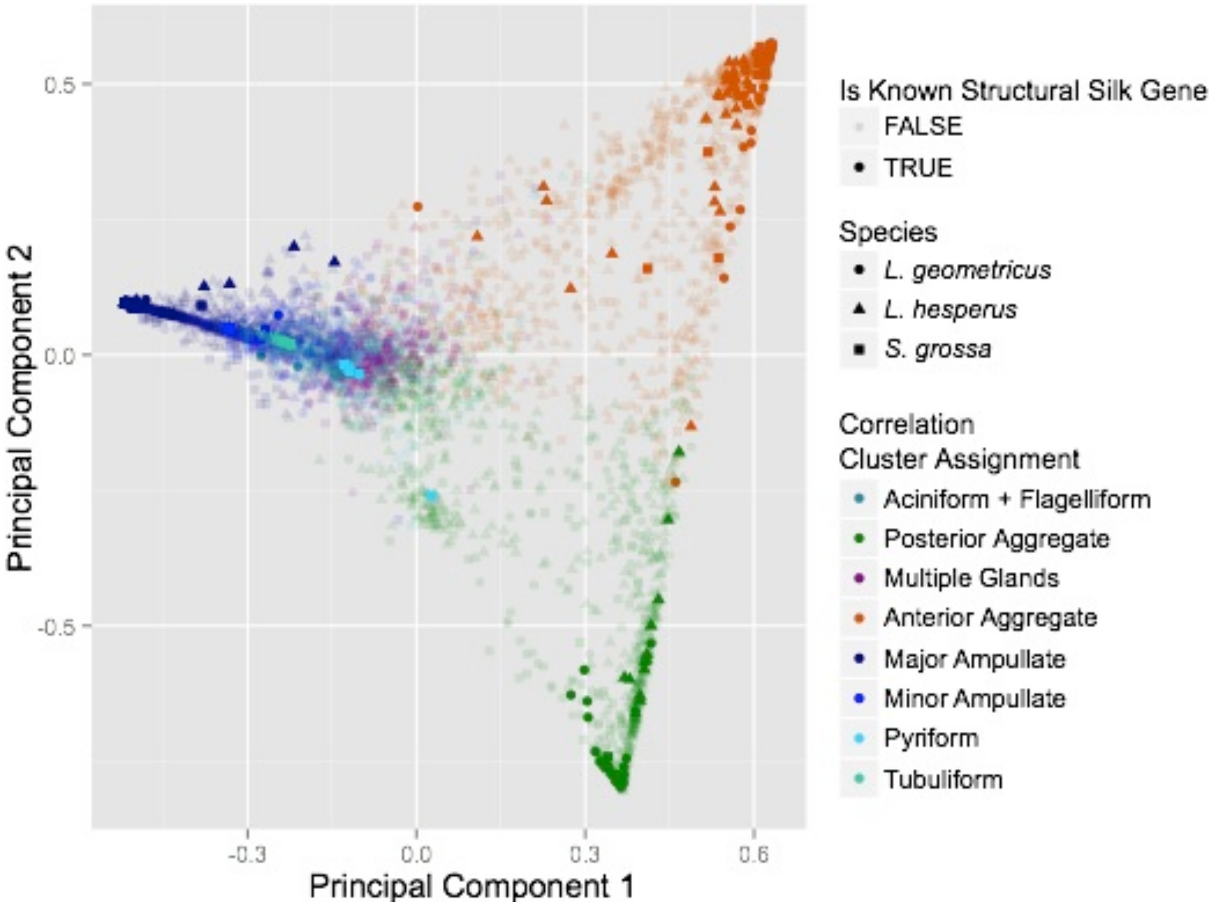

Supplementary Fig S5. **Principle Components Analysis of all three species' OESTs based on their proportion of expression in the seven silk gland types.** OESTs are color-coded according to the silk gland type assigned in correlation clustering (see Fig 4). Shapes represent species and bolded points are homologous to known silk structural encoding genes (see Supplementary File 1).

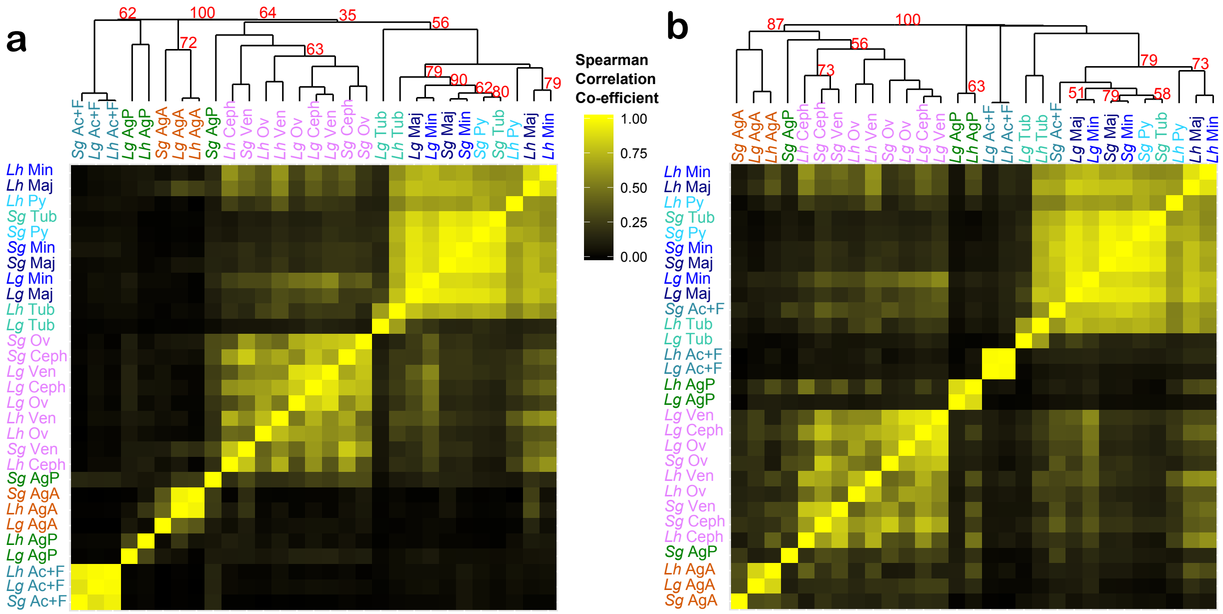

Supplementary Figure S6. **Correlation between species and tissue-specific RNA-seq libraries based on expression of 1:1:1 orthologs over-expressed in silk glands.** Heat maps show pairwise Spearman's correlation coefficients based on the normalized FPKM of orthologs identified with OrthoMCL. Orthologous groups (N=357) contained at least one OEST. Distances used to heirarchically cluster the libraries were 1-Spearman's rho (bootstrap proportions ≥50% shown). In (a) orthologous groups containing any OEST are included. In (b) the ortholog groups composed of known silk structural protein-encoding transcripts have been excluded (N=314). Despite weak correlations here, the 70 orthologous groups entirely composed of OESTs likely represent highly conserved members of silk synthesis pathways, especially the 26 in which every member has a majority of expression in the same gland type (Supplementary File 2). Abbreviations and sample sizes as in Fig 1 and 3 of main manuscrpt.

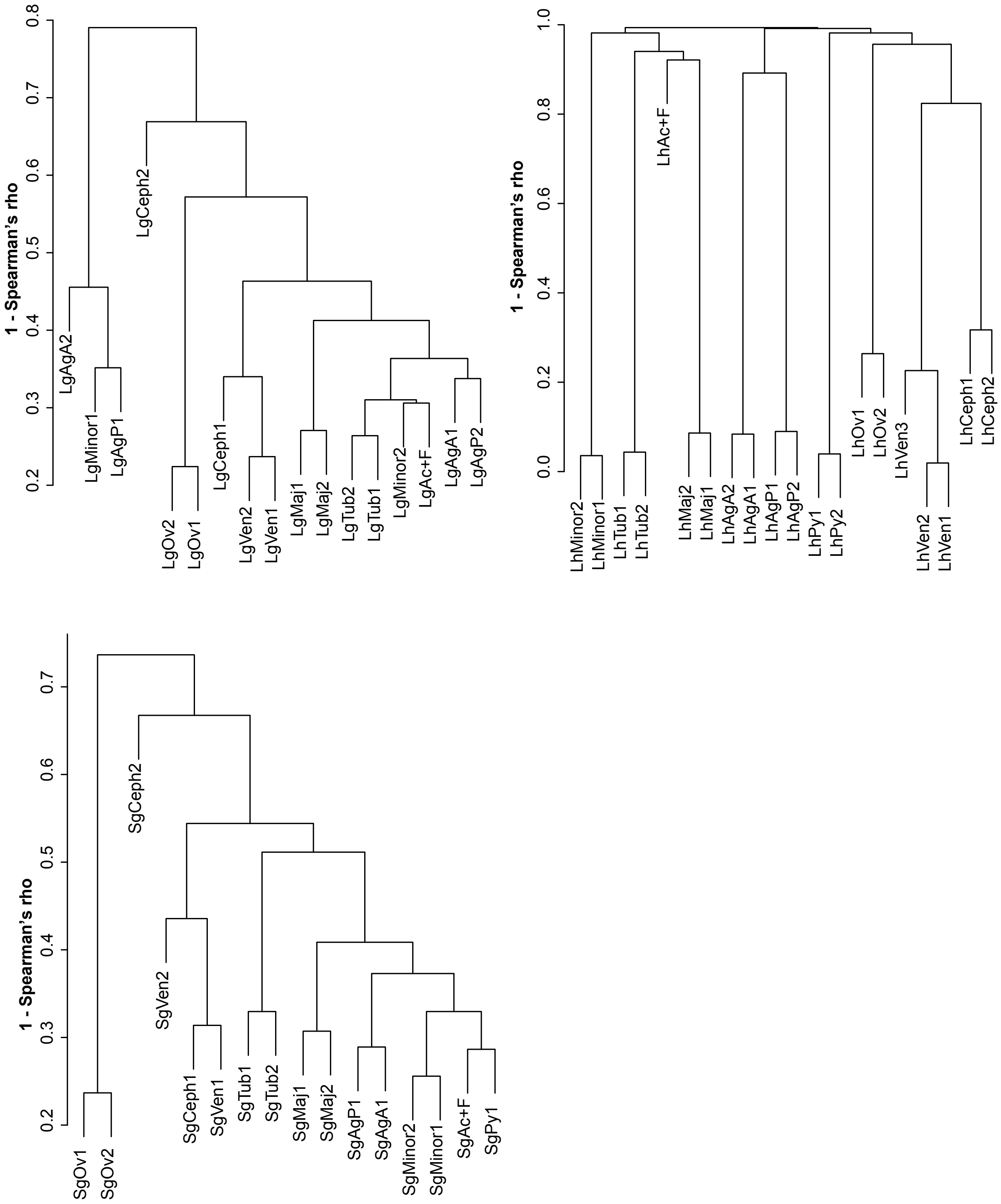

Supplementary Figure S7. **Hierarchical clustering of each tissue-specific RNA-seq library.** Pairwise Spearman's rank correlation coefficients were computed based on the normalized FPKM of all transcripts. See Supplementary File 7 for plots of normalized FPKM between each tissue-specific replicate.

References

1. Clarke, T. H., Garb, J. E., Hayashi, C. Y., Arensburger, P. & Ayoub, N. A. Spider transcriptomes identify ancient large-scale gene duplication event potentially important in silk gland evolution. *Genome Biol. Evol.* **7,** 1856–1857 (2015).
2. Jeffery, F. *et al.* Microdissection of black widow spider silk-producing glands. *J. Vis. Exp. JoVE* (2011). doi:10.3791/2382
3. Kovoor, J. J. in *Ecophysiology of spiders.* (ed. Nentwig, W.) 159–186 (Springer-Verlag, 1987).
4. Kovoor, J. J. Données histochimiques sur les glandes séricigènes de la veuve noire *Latrodectus mactans* Fabr. (Araneae, Theridiidae). *Ann. Sc. Nat. Zool.* **12,** 63–87 (1977).
5. Townley, M. A. & Tillinghast, E. K. in *Spider Ecophysiology* (ed. Nentwig, W.) 283–302 (Springer Berlin Heidelberg, 2013).
6. Petersen, T. N., Brunak, S. ren, von Heijne, G. & Nielsen, H. SignalP 4.0: discriminating signal peptides from transmembrane regions. *Nat. Methods* **8,** 785–6 (2011).
