# Supplementary material for: Evolutionary shifts in gene expression decoupled from gene duplication across functionally distinct spider silk glands
Source: Sci Rep. 2017 Aug 21;7:8393. doi: 10.1038/s41598-017-07388-1 (PMC5566633; doi:10.1038/s41598-017-07388-1)
Supplement: Supplementary file 6 — Supplementary File 5 [file 41598_2017_7388_MOESM6_ESM.pdf]

Supplementary file 5 for

Evolutionary shifts in gene expression decoupled from gene duplication across functionally distinct spider silk glands

Thomas H. Clarke, Jessica E. Garb, Robert A. Haney, R. Crystal Chaw, Cheryl Y. Hayashi, Nadia A. Ayoub

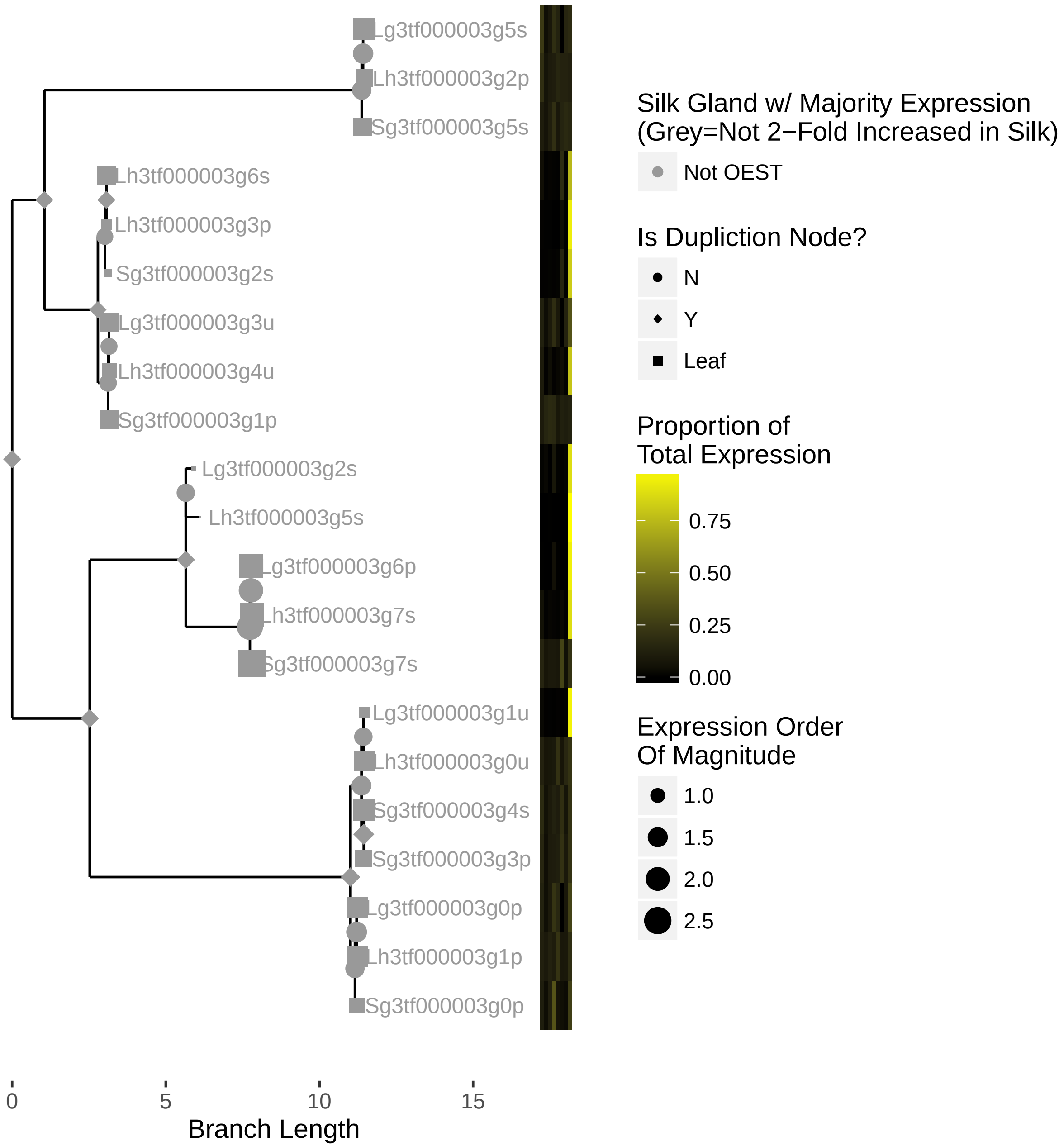
Lg3tf000003g5s
Lh3tf000003g2p
Sg3tf000003g5s
Lh3tf000003g6s
Lh3tf000003g3p
Sg3tf000003g2s
Lg3tf000003g3u
Lh3tf000003g4u
Sg3tf000003g1p
Lg3tf000003g2s
Lh3tf000003g5s
Lg3tf000003g6p
Lh3tf000003g7s
Sg3tf000003g7s
Lg3tf000003g1u
Lh3tf000003g0u
Sg3tf000003g4s
Sg3tf000003g3p
Lg3tf000003g0p
Lh3tf000003g1p
Sg3tf000003g0p
0
5
10
15
Branch Length
Silk Gland w/ Majority Expression
(Grey=Not 2−Fold Increased in Silk)
Not OEST
Is Dupliction Node?
N
Y
Leaf
Proportion of
Total Expression
0.75
0.50
0.25
0.00
Expression Order
Of Magnitude
1.0
1.5
2.0
2.5

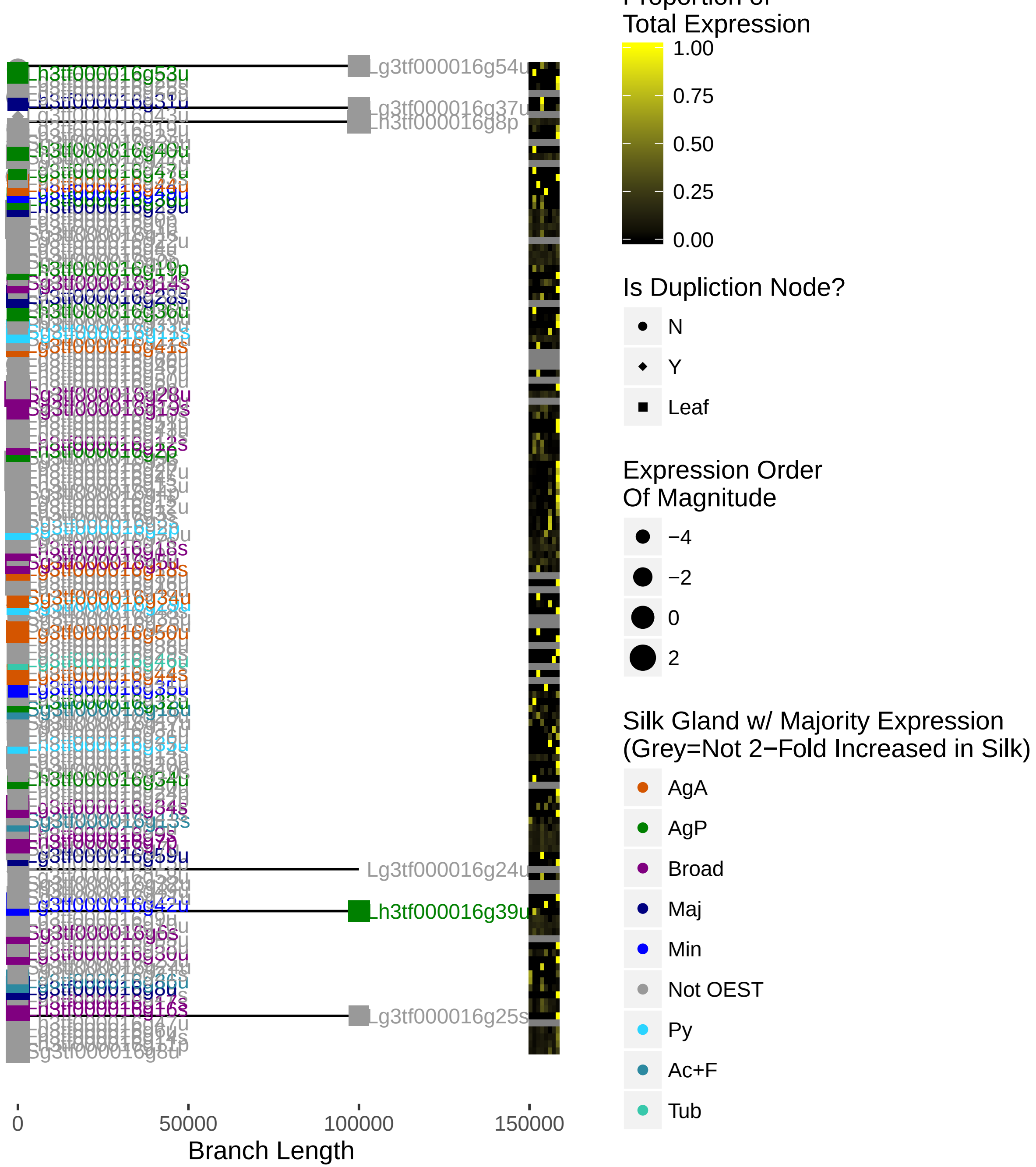

Total Expression
1.00
0.75
0.50
0.25
0.00
Is Dupliction Node?
N
Y
Leaf
Expression Order
Of Magnitude
−4
−2
0
2
Silk Gland w/ Majority Expression
(Grey=Not 2−Fold Increased in Silk)
AgA
AgP
Broad
Maj
Min
Not OEST
Py
Ac+F
Tub
Lg3tf000016g54u
Lg3tf000016g37u
Lh3tf000016g8p
Lg3tf000016g24u
Lh3tf000016g39u
Lg3tf000016g25s
Lh3tf000016g53u
Lh3tf000016g40u
Lh3tf000016g29u
Lh3tf000016g19p
Sg3tf000016g14s
Lh3tf000016g36u
Lg3tf000016g41s
Sg3tf000016g28u
Sg3tf000016g19s
Lh3tf000016g34u
Lg3tf000016g34s
Lg3tf000016g59u
Sg3tf000016g6s
0
50000
100000
150000
Branch Length

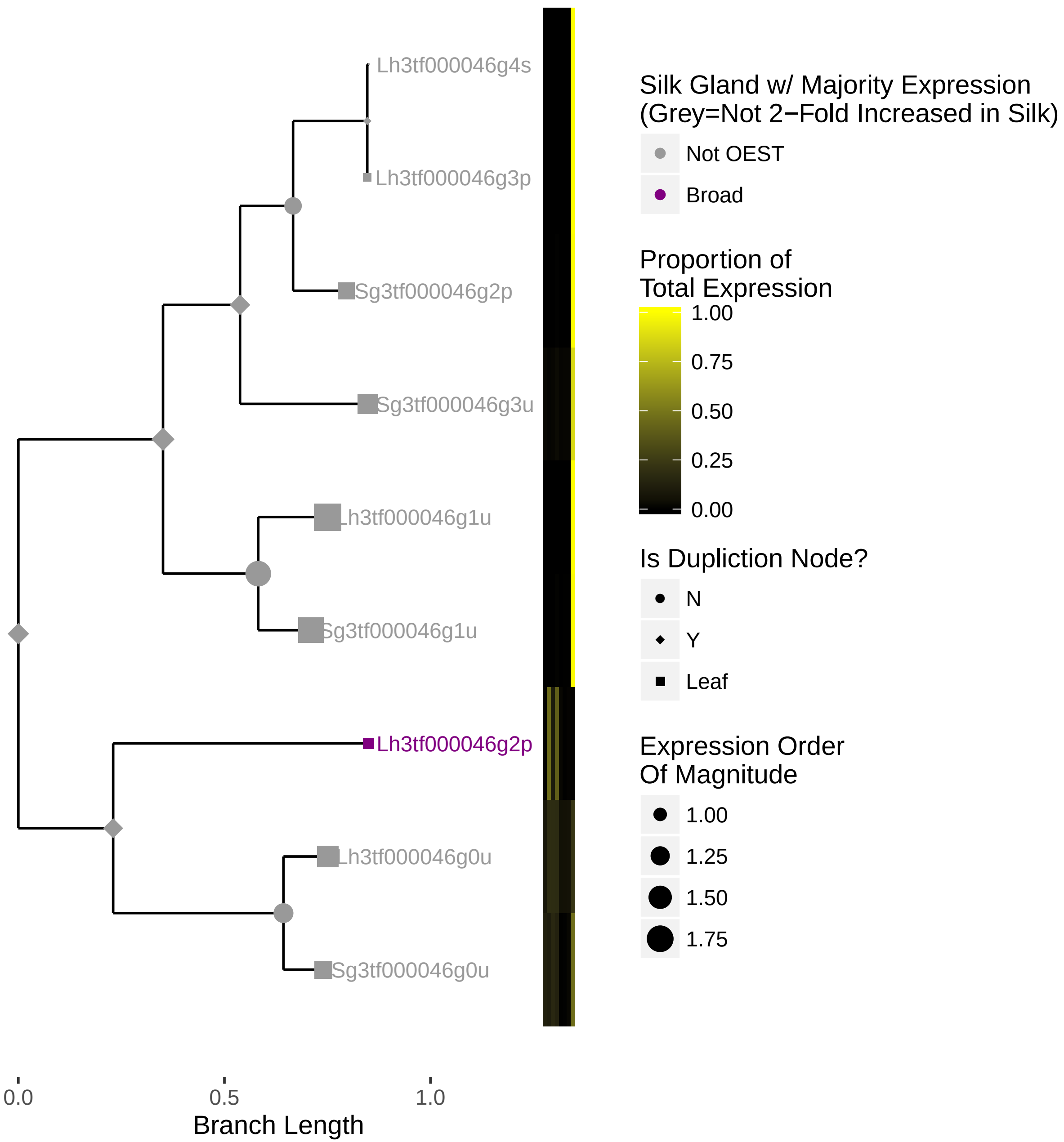

Lh3tf000046g4s
Lh3tf000046g3p
Sg3tf000046g2p
Sg3tf000046g3u
Lh3tf000046g1u
Sg3tf000046g1u
Lh3tf000046g2p
Lh3tf000046g0u
Sg3tf000046g0u
0.0
0.5
1.0
Branch Length
Silk Gland w/ Majority Expression
(Grey=Not 2−Fold Increased in Silk)
Not OEST
Broad
Proportion of
Total Expression
1.00
0.75
0.50
0.25
0.00
Is Dupliction Node?
N
Y
Leaf
Expression Order
Of Magnitude
1.00
1.25
1.50
1.75

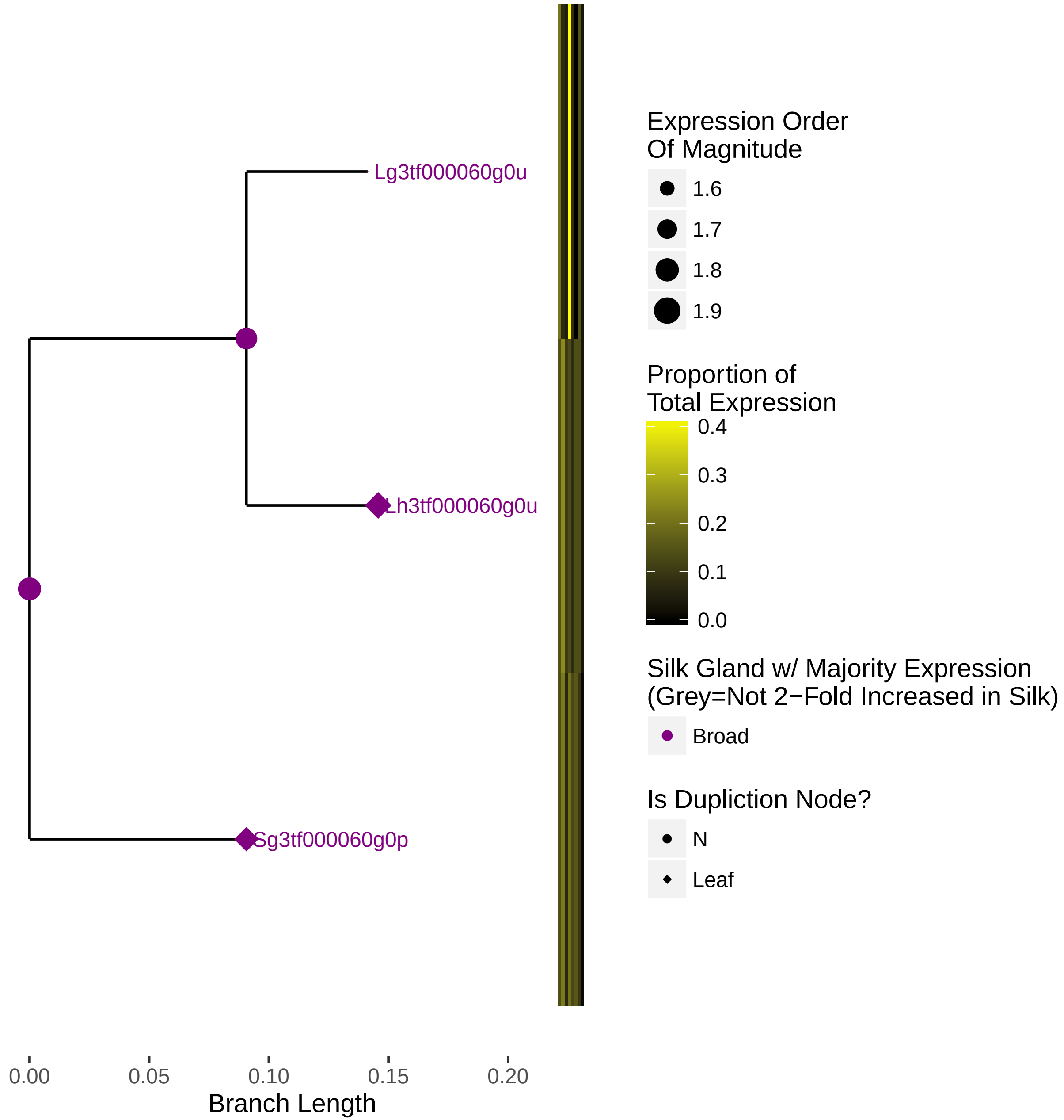
Lg3tf000060g0u
Lh3tf000060g0u
Sg3tf000060g0p
0.00
0.05
0.10
0.15
0.20
Branch Length
Expression Order
Of Magnitude
1.6
1.7
1.8
1.9
Proportion of
Total Expression
0.4
0.3
0.2
0.1
0.0
Silk Gland w/ Majority Expression
(Grey=Not 2−Fold Increased in Silk)
Broad
Is Dupliction Node?
N
Leaf

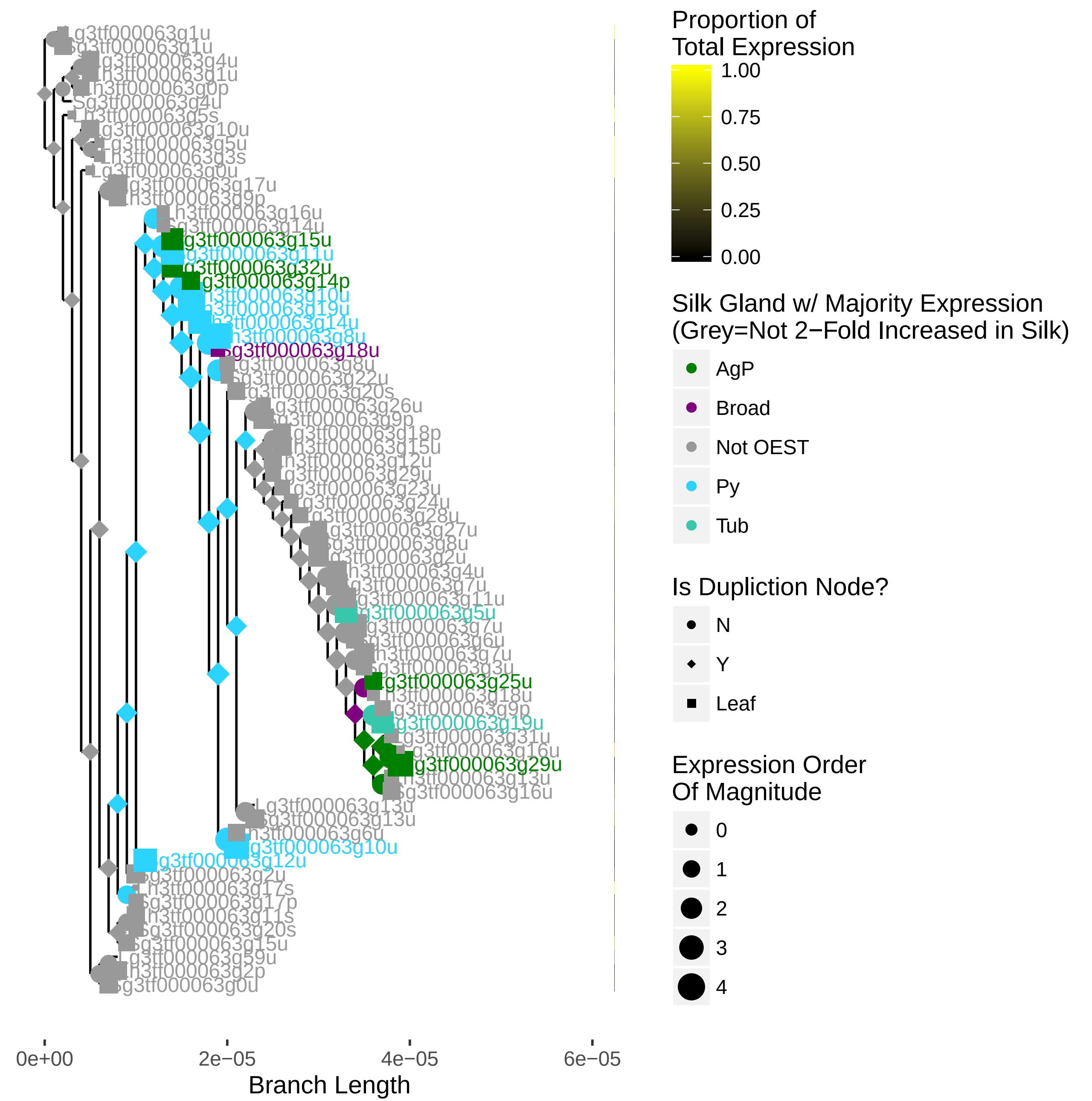

Proportion of Total Expression
1.00
0.75
0.50
0.25
0.00
Silk Gland w/ Majority Expression (Grey=Not 2−Fold Increased in Silk)
AgP
Broad
Not OEST
Py
Tub
Is Dupliction Node?
N
Y
Leaf
Expression Order Of Magnitude
0
1
2
3
4
0e+00
2e−05
4e−05
6e−05
Branch Length

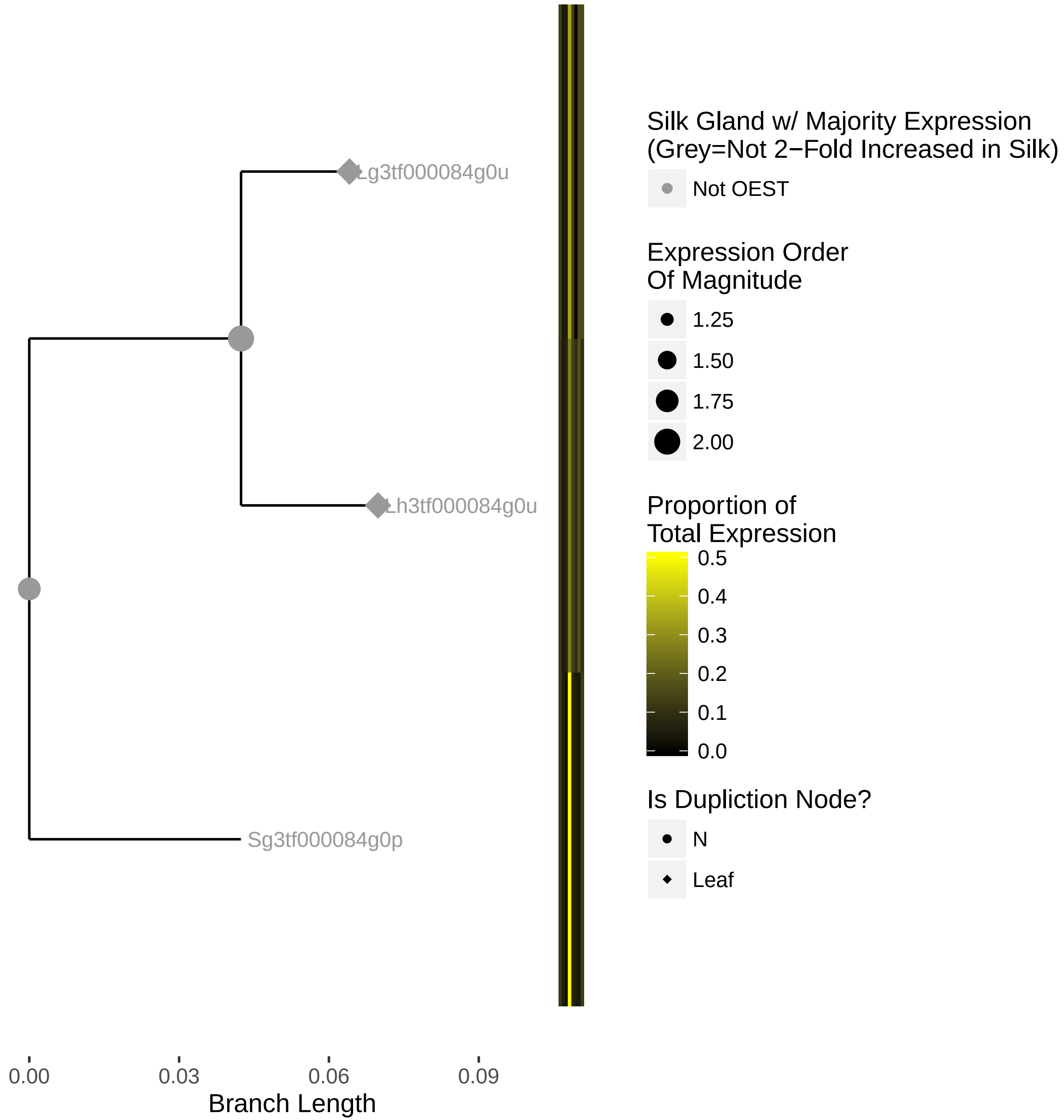

Lg3tf000084g0u
Lh3tf000084g0u
Sg3tf000084g0p
0.00
0.03
0.06
0.09
Branch Length
Silk Gland w/ Majority Expression
(Grey=Not 2−Fold Increased in Silk)
Not OEST
Expression Order
Of Magnitude
1.25
1.50
1.75
2.00
Proportion of
Total Expression
0.5
0.4
0.3
0.2
0.1
0.0
Is Dupliction Node?
N
Leaf

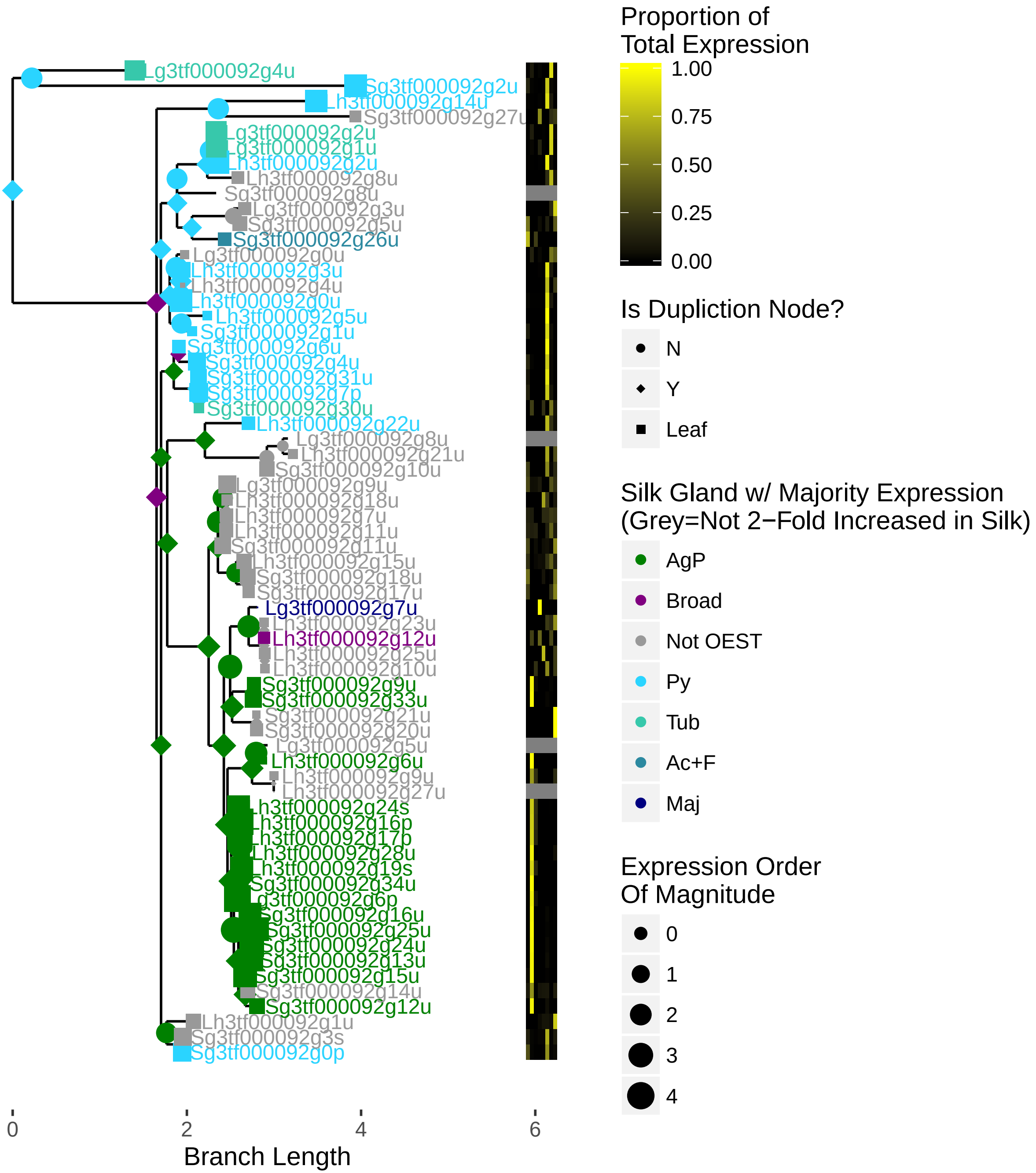

Lg3tf000092g4u
Sg3tf000092g2u
Lh3tf000092g14u
Sg3tf000092g27u
Lg3tf000092g2u
Lg3tf000092g1u
Lh3tf000092g2u
Lh3tf000092g8u
Sg3tf000092g8u
Lg3tf000092g3u
Sg3tf000092g5u
Sg3tf000092g26u
Lg3tf000092g0u
Lh3tf000092g3u
Lh3tf000092g4u
Lh3tf000092g0u
Lh3tf000092g5u
Sg3tf000092g1u
Sg3tf000092g6u
Sg3tf000092g4u
Sg3tf000092g31u
Sg3tf000092g7p
Sg3tf000092g30u
Lh3tf000092g22u
Lg3tf000092g8u
Lh3tf000092g21u
Sg3tf000092g10u
Lg3tf000092g9u
Lh3tf000092g18u
Lh3tf000092g7u
Lh3tf000092g11u
Sg3tf000092g11u
Lh3tf000092g15u
Sg3tf000092g18u
Sg3tf000092g17u
Lg3tf000092g7u
Lh3tf000092g23u
Lh3tf000092g12u
Lh3tf000092g25u
Lh3tf000092g10u
Sg3tf000092g9u
Sg3tf000092g33u
Sg3tf000092g21u
Sg3tf000092g20u
Lg3tf000092g5u
Lh3tf000092g6u
Lh3tf000092g9u
Lh3tf000092g27u
h3tf000092g24s
Lh3tf000092g16p
Lh3tf000092g17p
Lh3tf000092g28u
Lh3tf000092g19s
Sg3tf000092g34u
g3tf000092g6p
Sg3tf000092g16u
Sg3tf000092g25u
Sg3tf000092g24u
Sg3tf000092g13u
Sg3tf000092g15u
Sg3tf000092g14u
Sg3tf000092g12u
Lh3tf000092g1u
Sg3tf000092g3s
Sg3tf000092g0p
0
2
4
6
Branch Length
Proportion of
Total Expression
1.00
0.75
0.50
0.25
0.00
Is Dupliction Node?
N
Y
Leaf
Silk Gland w/ Majority Expression
(Grey=Not 2−Fold Increased in Silk)
AgP
Broad
Not OEST
Py
Tub
Ac+F
Maj
Expression Order
Of Magnitude
0
1
2
3
4

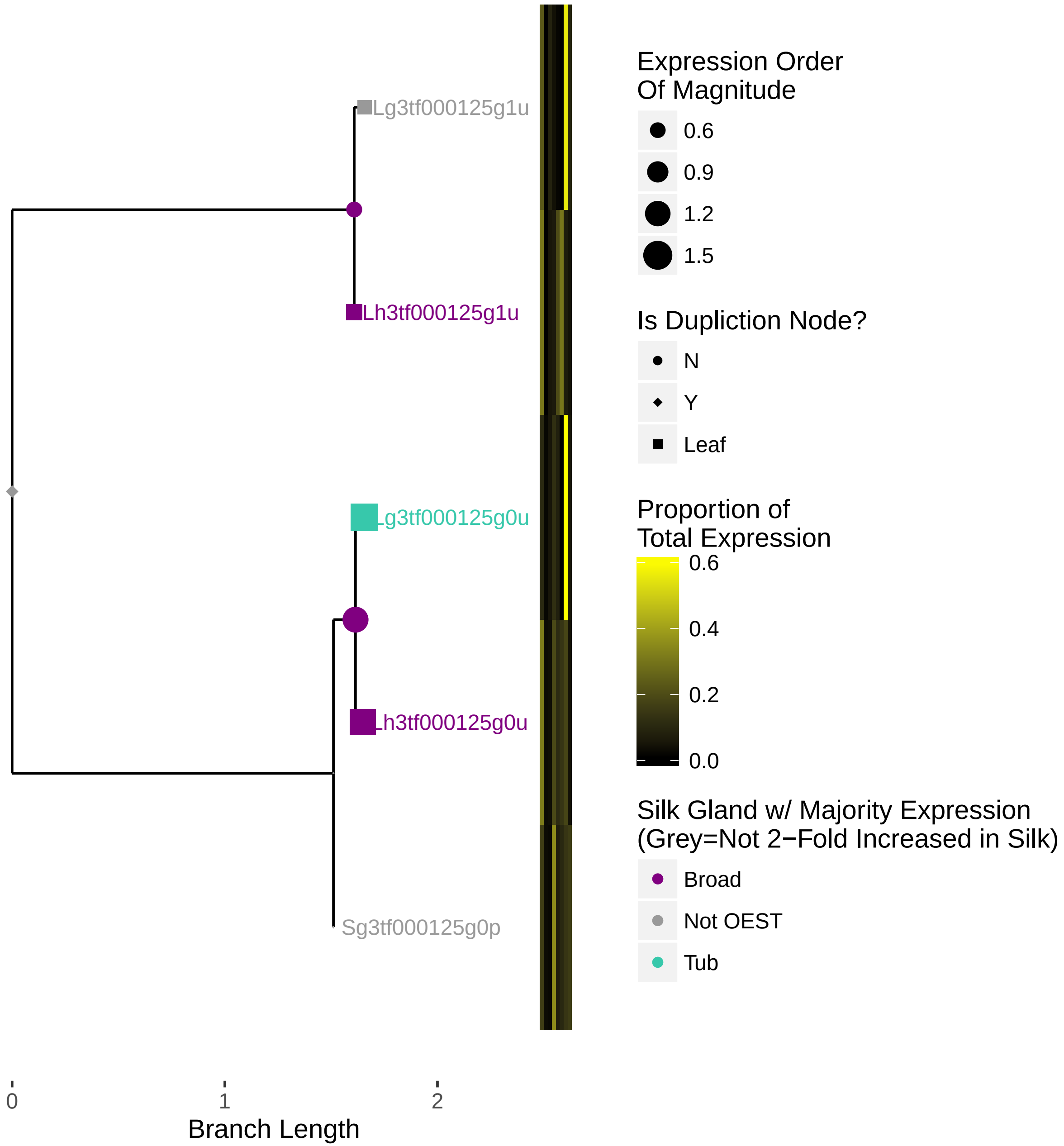
Lg3tf000125g1u
Lh3tf000125g1u
Lg3tf000125g0u
Lh3tf000125g0u
Sg3tf000125g0p
0
1
2
Branch Length
Expression Order
Of Magnitude
0.6
0.9
1.2
1.5
Is Dupliction Node?
N
Y
Leaf
Proportion of
Total Expression
0.6
0.4
0.2
0.0
Silk Gland w/ Majority Expression
(Grey=Not 2−Fold Increased in Silk)
Broad
Not OEST
Tub

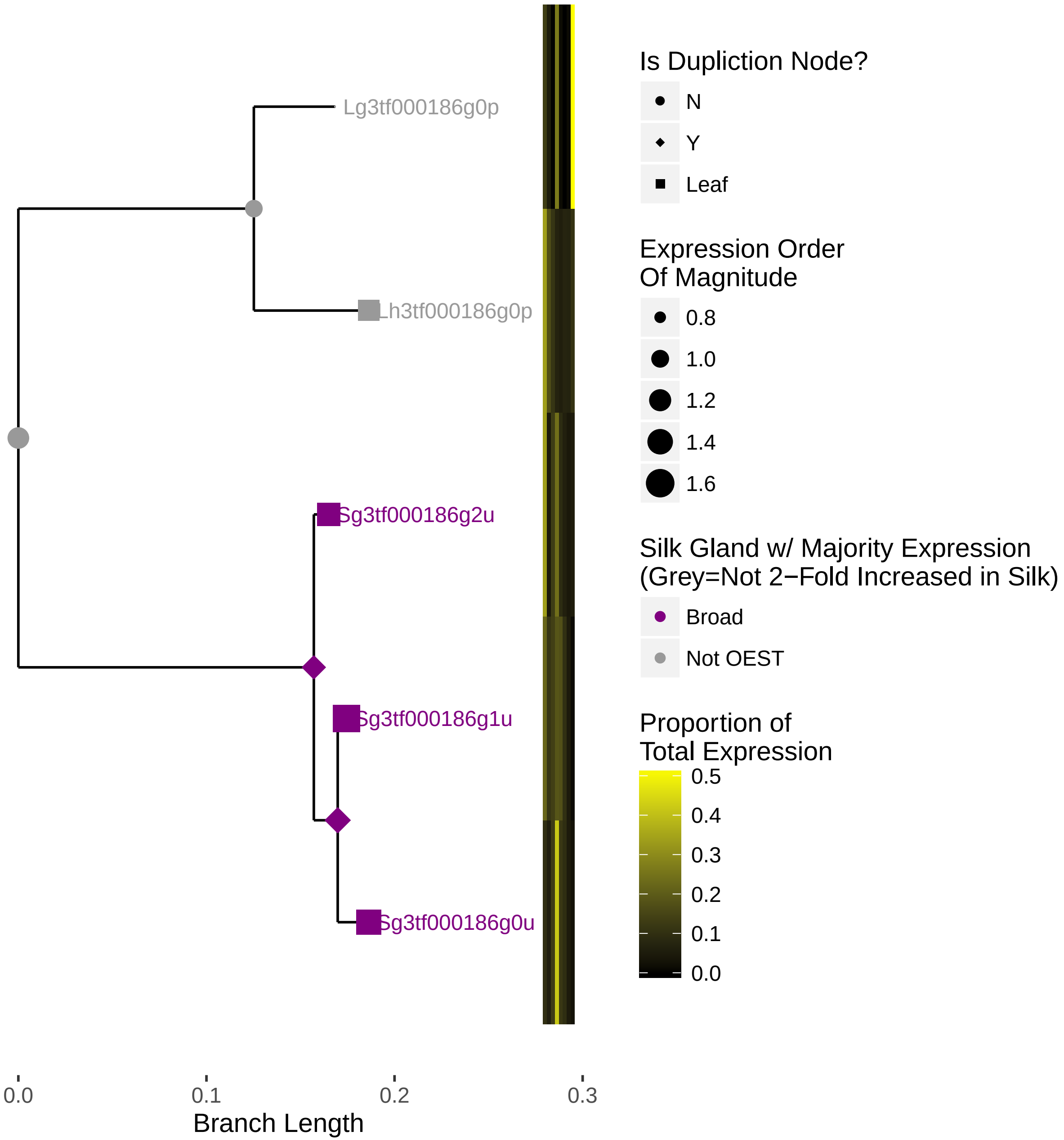
Lg3tf000186g0p
Lh3tf000186g0p
Sg3tf000186g2u
Sg3tf000186g1u
Sg3tf000186g0u
0.0
0.1
0.2
0.3
Branch Length
Is Dupliction Node?
N
Y
Leaf
Expression Order
Of Magnitude
0.8
1.0
1.2
1.4
1.6
Silk Gland w/ Majority Expression
(Grey=Not 2−Fold Increased in Silk)
Broad
Not OEST
Proportion of
Total Expression
0.5
0.4
0.3
0.2
0.1
0.0

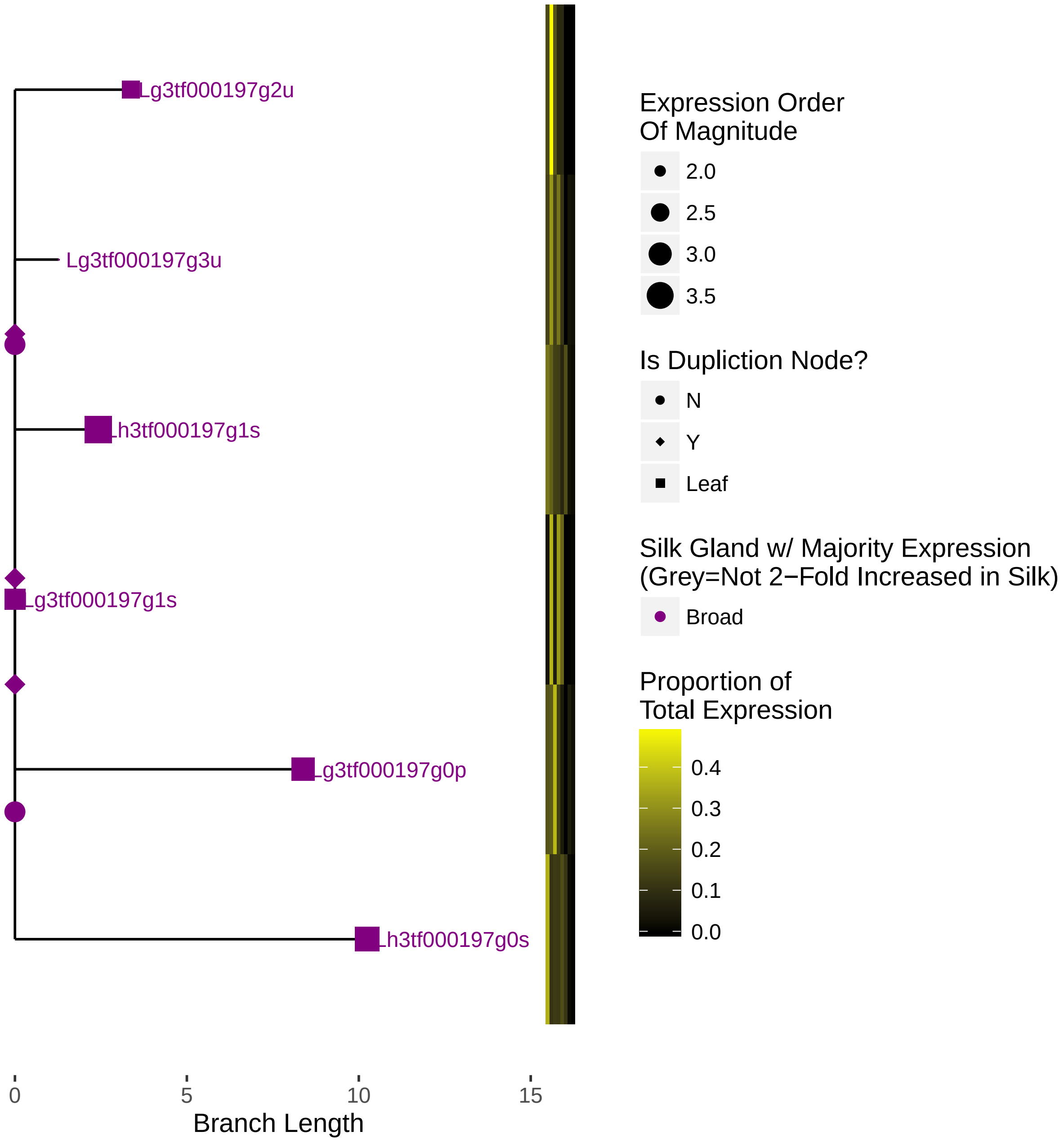
Lg3tf000197g2u
Lg3tf000197g3u
Lh3tf000197g1s
Lg3tf000197g1s
Lg3tf000197g0p
Lh3tf000197g0s
0
5
10
15
Branch Length
Expression Order
Of Magnitude
2.0
2.5
3.0
3.5
Is Dupliction Node?
N
Y
Leaf
Silk Gland w/ Majority Expression
(Grey=Not 2−Fold Increased in Silk)
Broad
Proportion of
Total Expression
0.4
0.3
0.2
0.1
0.0

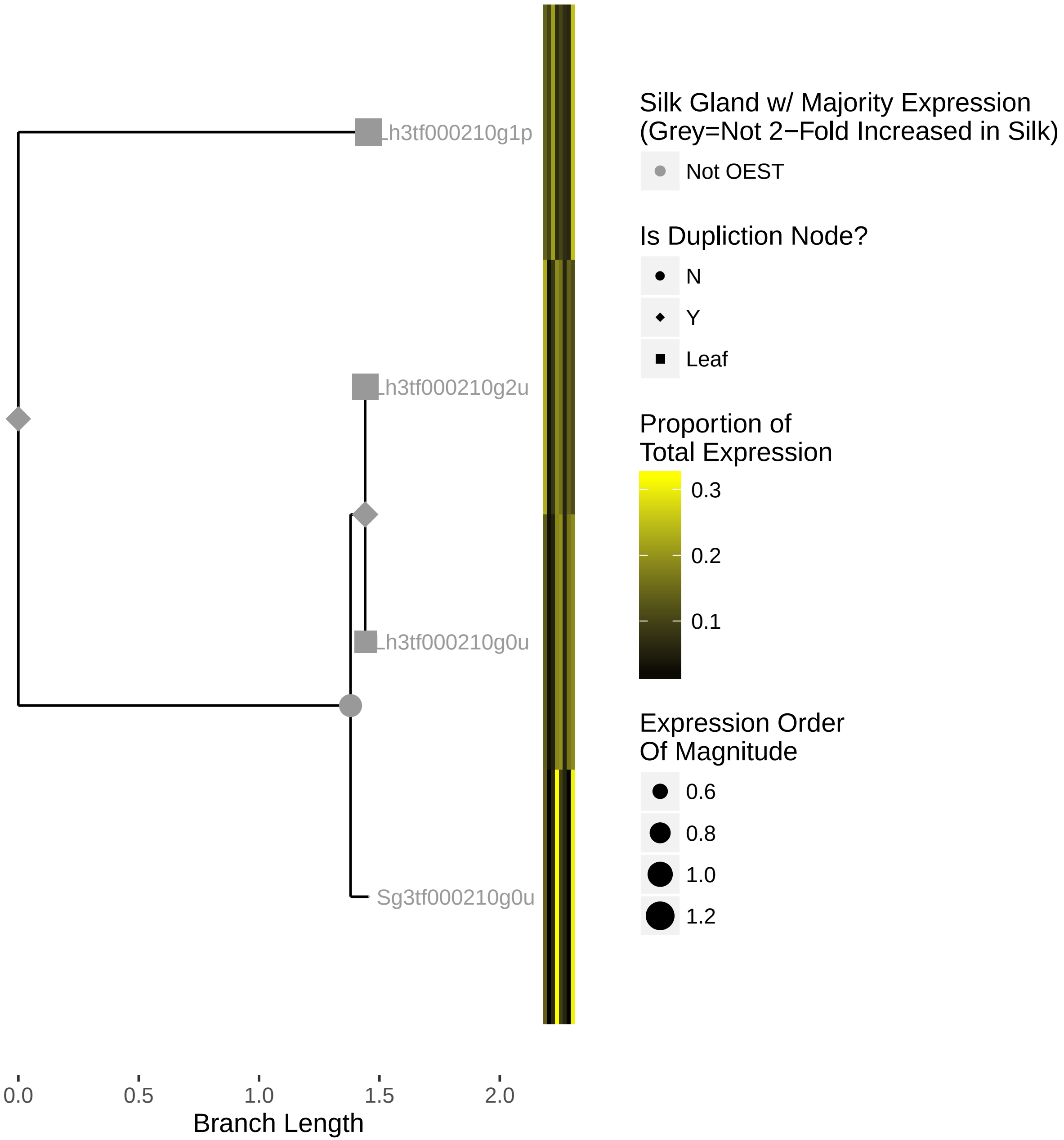
Lh3tf000210g1p
Lh3tf000210g2u
Lh3tf000210g0u
Sg3tf000210g0u
0.0
0.5
1.0
1.5
2.0
Branch Length
Silk Gland w/ Majority Expression
(Grey=Not 2−Fold Increased in Silk)
Not OEST
Is Dupliction Node?
N
Y
Leaf
Proportion of
Total Expression
0.3
0.2
0.1
Expression Order
Of Magnitude
0.6
0.8
1.0
1.2

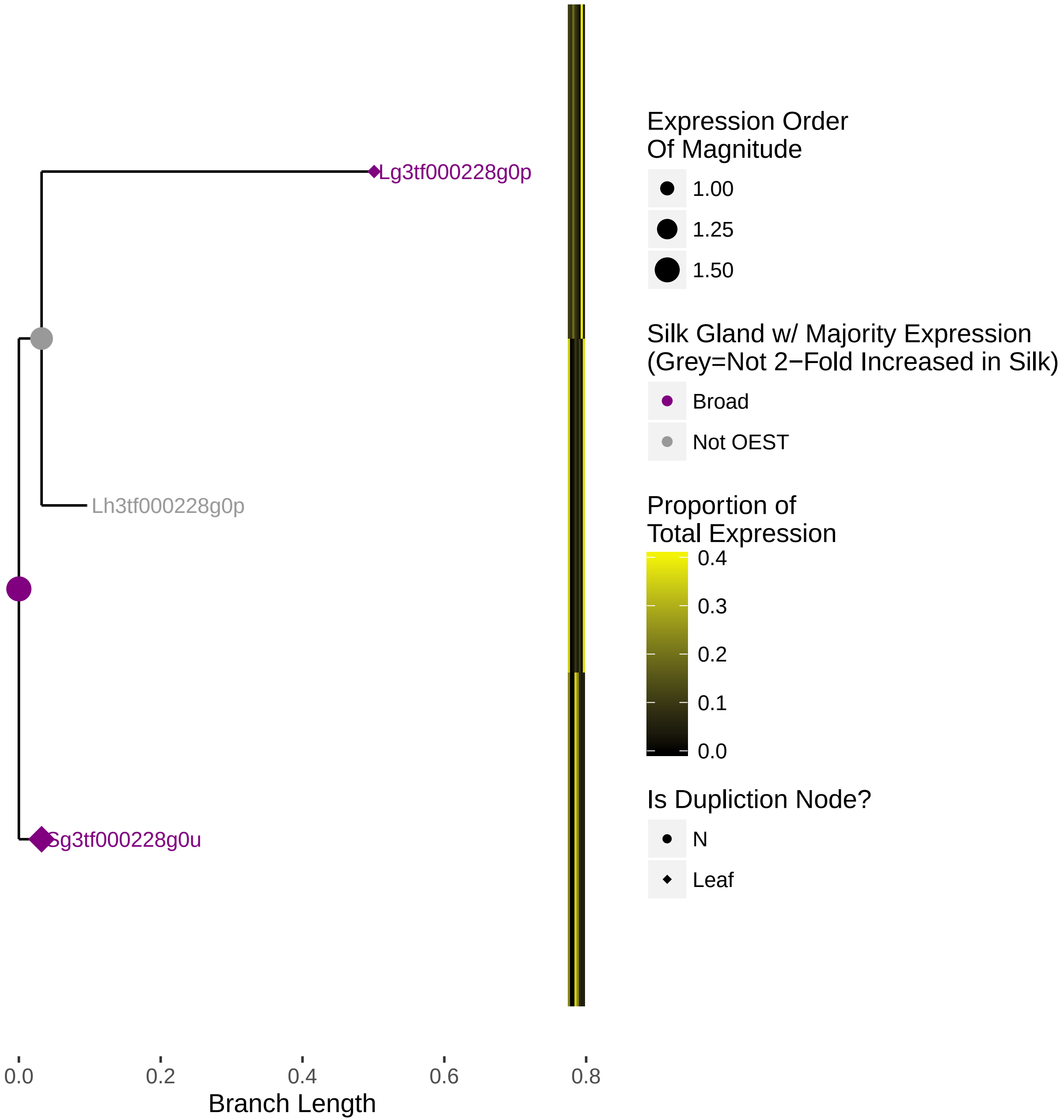
Lg3tf000228g0p
Lh3tf000228g0p
Sg3tf000228g0u
Expression Order
Of Magnitude
1.00
1.25
1.50
Silk Gland w/ Majority Expression
(Grey=Not 2−Fold Increased in Silk)
Broad
Not OEST
Proportion of
Total Expression
0.4
0.3
0.2
0.1
0.0
Is Dupliction Node?
N
Leaf
0.0
0.2
0.4
0.6
0.8
Branch Length

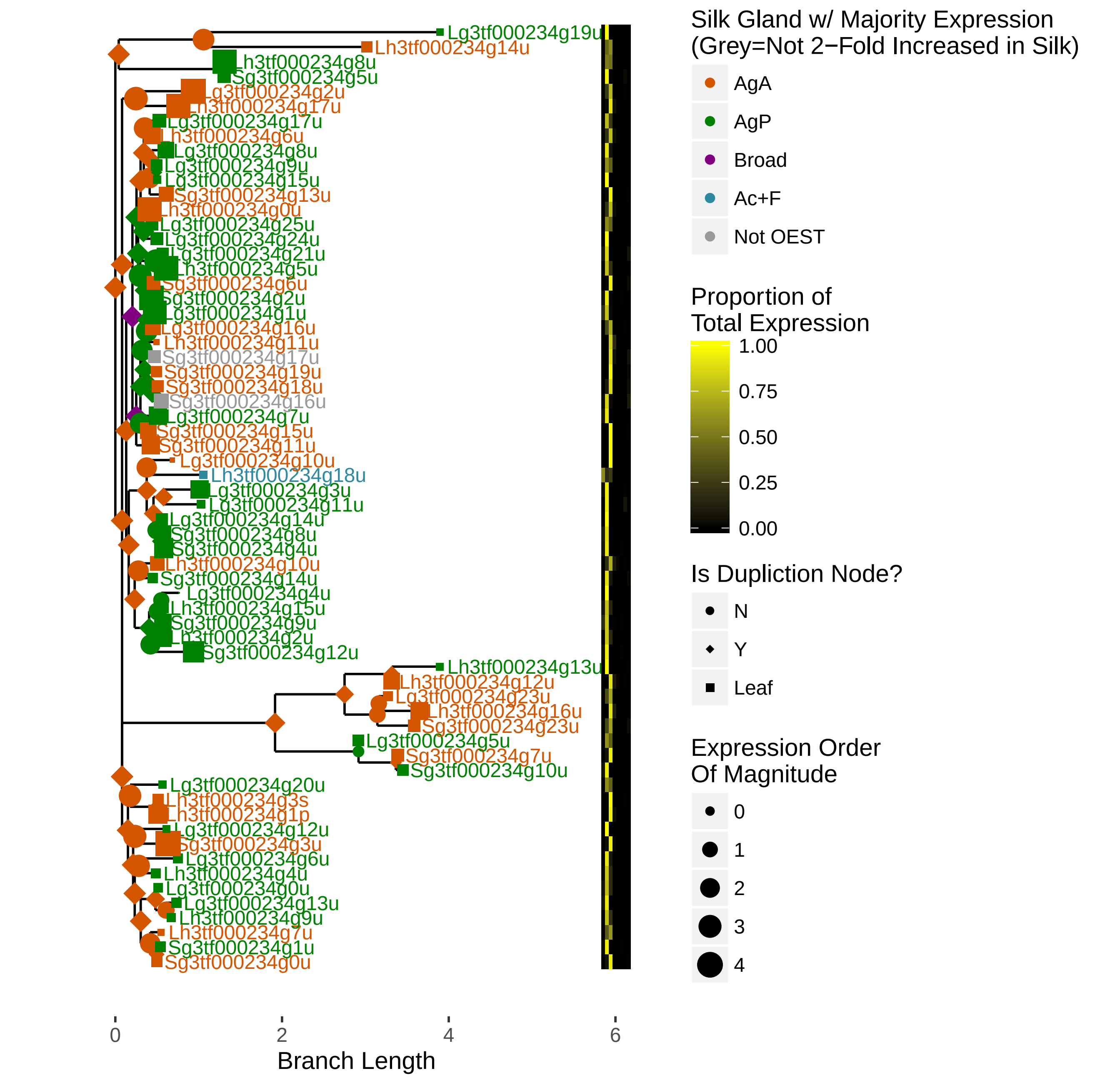

Lg3tf000234g19u
Lh3tf000234g14u
h3tf000234g8u
Sg3tf000234g5u
g3tf000234g2u
h3tf000234g17u
Lg3tf000234g17u
Lh3tf000234g6u
Lg3tf000234g8u
Lg3tf000234g9u
Lg3tf000234g15u
Sg3tf000234g13u
Lh3tf000234g0u
Lg3tf000234g25u
Lg3tf000234g24u
Lg3tf000234g21u
Lh3tf000234g5u
Sg3tf000234g6u
Sg3tf000234g2u
Lg3tf000234g1u
Lg3tf000234g16u
Lh3tf000234g11u
Sg3tf000234g17u
Sg3tf000234g19u
Sg3tf000234g18u
Sg3tf000234g16u
Lg3tf000234g7u
Sg3tf000234g15u
Sg3tf000234g11u
Lg3tf000234g10u
Lh3tf000234g18u
Lg3tf000234g3u
Lg3tf000234g11u
Lg3tf000234g14u
Sg3tf000234g8u
Sg3tf000234g4u
Lh3tf000234g10u
Sg3tf000234g14u
Lg3tf000234g4u
Lh3tf000234g15u
Sg3tf000234g9u
Lh3tf000234g2u
Sg3tf000234g12u
Lh3tf000234g13u
Lh3tf000234g12u
Lg3tf000234g23u
Lh3tf000234g16u
Sg3tf000234g23u
Lg3tf000234g5u
Sg3tf000234g7u
Sg3tf000234g10u
Lg3tf000234g20u
Lh3tf000234g3s
Lh3tf000234g1p
Lg3tf000234g12u
Sg3tf000234g3u
Lg3tf000234g6u
Lh3tf000234g4u
Lg3tf000234g0u
Lg3tf000234g13u
Lh3tf000234g9u
Lh3tf000234g7u
Sg3tf000234g1u
Sg3tf000234g0u
0
2
4
6
Branch Length
Silk Gland w/ Majority Expression
(Grey=Not 2−Fold Increased in Silk)
AgA
AgP
Broad
Ac+F
Not OEST
Proportion of
Total Expression
1.00
0.75
0.50
0.25
0.00
Is Dupliction Node?
N
Y
Leaf
Expression Order
Of Magnitude
0
1
2
3
4

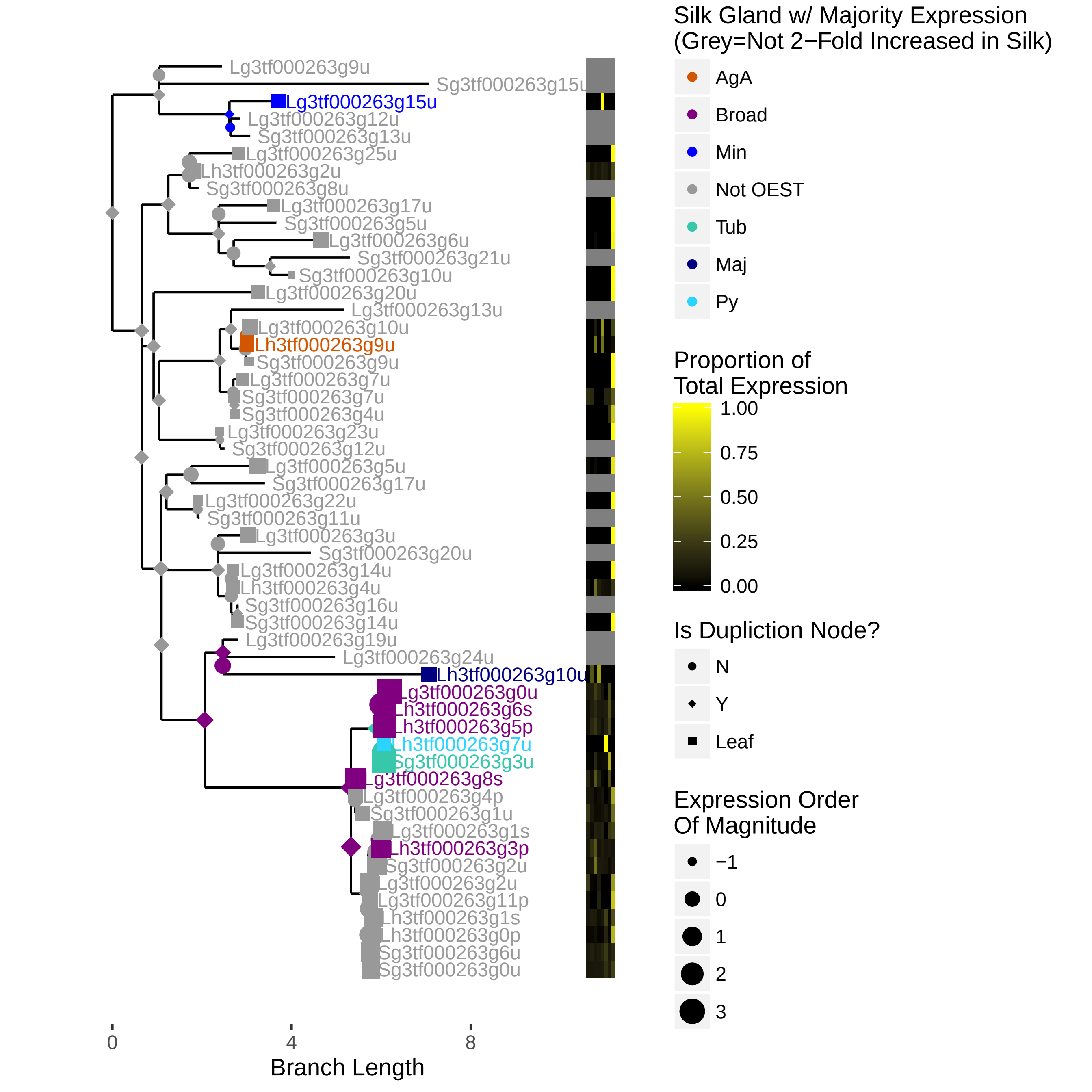

Lg3tf000263g9u
Sg3tf000263g15u
Lg3tf000263g15u
Lg3tf000263g12u
Sg3tf000263g13u
Lg3tf000263g25u
Lh3tf000263g2u
Sg3tf000263g8u
Lg3tf000263g17u
Sg3tf000263g5u
Lg3tf000263g6u
Sg3tf000263g21u
Sg3tf000263g10u
Lg3tf000263g20u
Lg3tf000263g13u
Lg3tf000263g10u
Lh3tf000263g9u
Sg3tf000263g9u
Lg3tf000263g7u
Sg3tf000263g7u
Sg3tf000263g4u
Lg3tf000263g23u
Sg3tf000263g12u
Lg3tf000263g5u
Sg3tf000263g17u
Lg3tf000263g22u
Sg3tf000263g11u
Lg3tf000263g3u
Sg3tf000263g20u
Lg3tf000263g14u
Lh3tf000263g4u
Sg3tf000263g16u
Sg3tf000263g14u
Lg3tf000263g19u
Lg3tf000263g24u
Lh3tf000263g10u
g3tf000263g0u
h3tf000263g6s
h3tf000263g5p
h3tf000263g7u
Sg3tf000263g3u
g3tf000263g8s
Lg3tf000263g4p
Sg3tf000263g1u
g3tf000263g1s
h3tf000263g3p
Sg3tf000263g2u
g3tf000263g2u
Lg3tf000263g11p
Lh3tf000263g1s
Lh3tf000263g0p
Sg3tf000263g6u
Sg3tf000263g0u
0
4
8
Branch Length
Silk Gland w/ Majority Expression
(Grey=Not 2−Fold Increased in Silk)
AgA
Broad
Min
Not OEST
Tub
Maj
Py
Proportion of
Total Expression
1.00
0.75
0.50
0.25
0.00
Is Dupliction Node?
N
Y
Leaf
Expression Order
Of Magnitude
−1
0
1
2
3

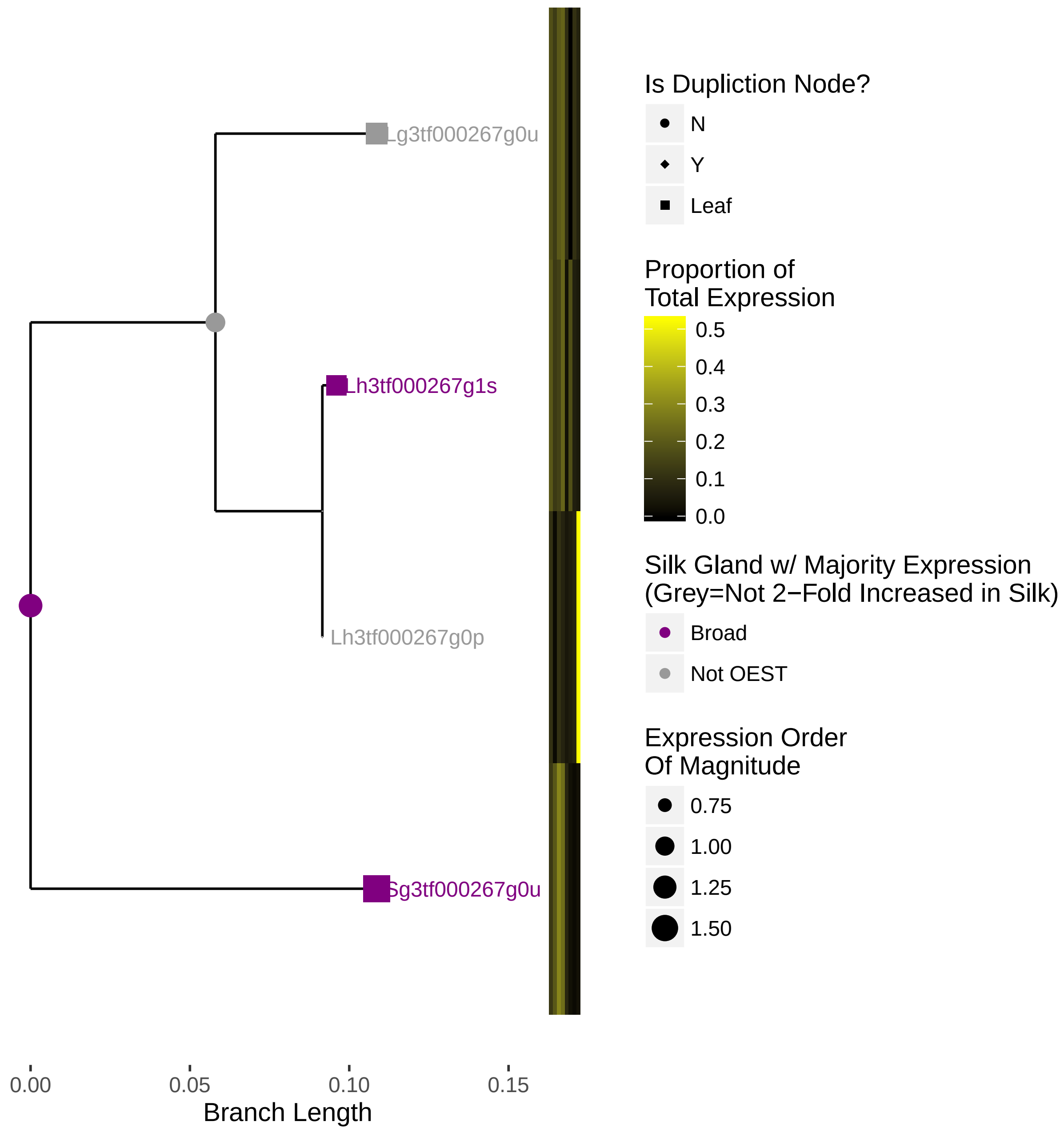

Lg3tf000267g0u
Lh3tf000267g1s
Lh3tf000267g0p
Sg3tf000267g0u
0.00
0.05
0.10
0.15
Branch Length
Is Dupliction Node?
N
Y
Leaf
Proportion of
Total Expression
0.5
0.4
0.3
0.2
0.1
0.0
Silk Gland w/ Majority Expression
(Grey=Not 2−Fold Increased in Silk)
Broad
Not OEST
Expression Order
Of Magnitude
0.75
1.00
1.25
1.50

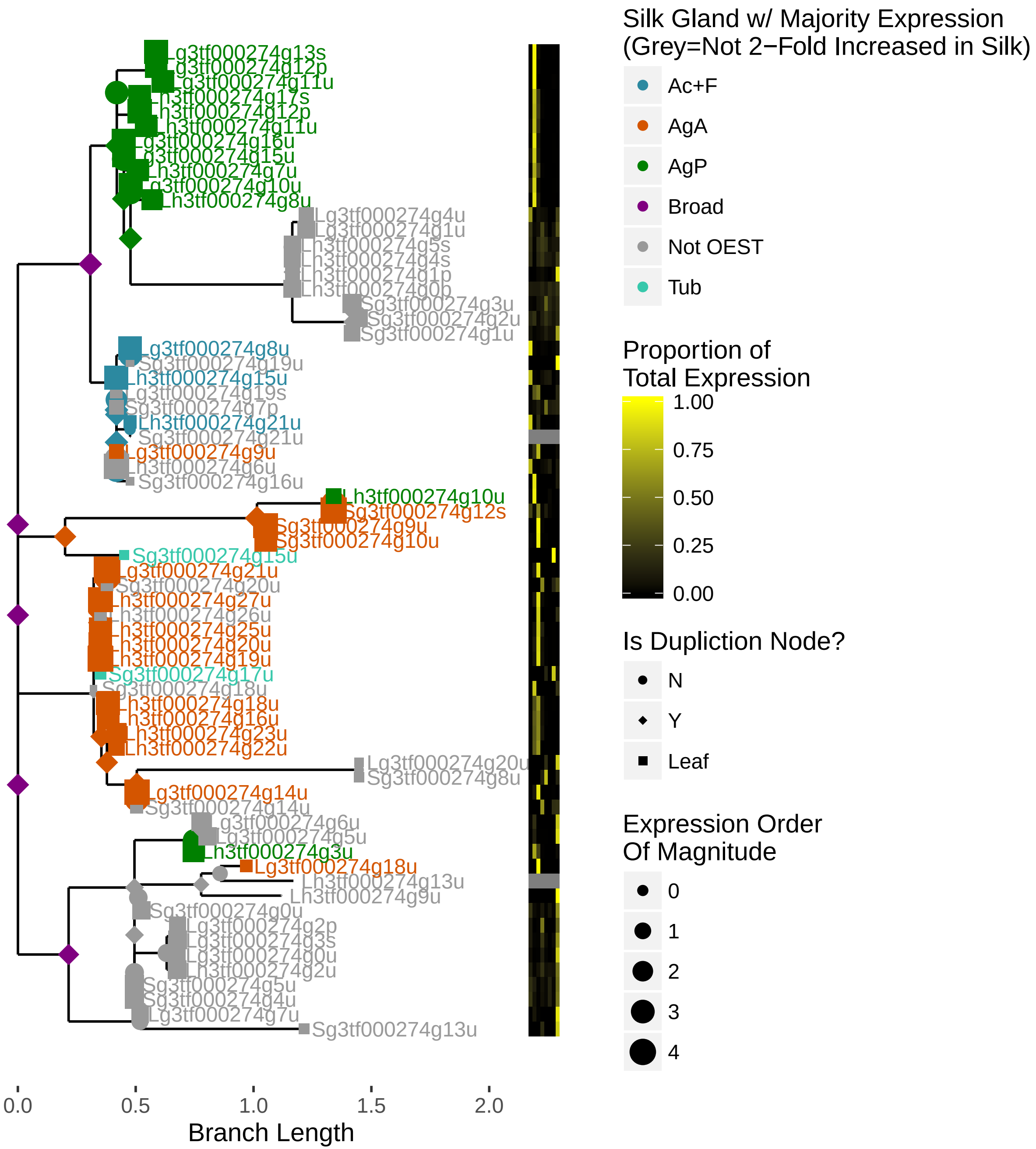

Silk Gland w/ Majority Expression
(Grey=Not 2−Fold Increased in Silk)
Ac+F
AgA
AgP
Broad
Not OEST
Tub
Proportion of
Total Expression
1.00
0.75
0.50
0.25
0.00
Is Dupliction Node?
N
Y
Leaf
Expression Order
Of Magnitude
0
1
2
3
4
Lg3tf000274g13s
Lg3tf000274g12p
Lg3tf000274g11u
Lh3tf000274g17s
Lh3tf000274g12p
Lh3tf000274g11u
Lg3tf000274g16u
Lg3tf000274g15u
Lh3tf000274g7u
Lg3tf000274g10u
Lh3tf000274g8u
Lg3tf000274g4u
Lg3tf000274g1u
Lh3tf000274g5s
Lh3tf000274g4s
Lh3tf000274g1p
Lh3tf000274g0p
Sg3tf000274g3u
Sg3tf000274g2u
Sg3tf000274g1u
Lg3tf000274g8u
Sg3tf000274g19u
Lh3tf000274g15u
Lg3tf000274g19s
Sg3tf000274g7p
Lh3tf000274g21u
Sg3tf000274g21u
Lg3tf000274g9u
Lh3tf000274g6u
Sg3tf000274g16u
Lh3tf000274g10u
Sg3tf000274g12s
Sg3tf000274g9u
Sg3tf000274g10u
Sg3tf000274g15u
Lg3tf000274g21u
Sg3tf000274g20u
Lh3tf000274g27u
Lh3tf000274g26u
Lh3tf000274g25u
Lh3tf000274g20u
Lh3tf000274g19u
Sg3tf000274g17u
Sg3tf000274g18u
Lh3tf000274g18u
Lh3tf000274g16u
Lh3tf000274g23u
Lh3tf000274g22u
Lg3tf000274g20u
Sg3tf000274g8u
Lg3tf000274g14u
Sg3tf000274g14u
Lg3tf000274g6u
Lg3tf000274g5u
Lh3tf000274g3u
Lg3tf000274g18u
Lh3tf000274g13u
Lh3tf000274g9u
Sg3tf000274g0u
Lg3tf000274g2p
Lg3tf000274g3s
Lg3tf000274g0u
Lh3tf000274g2u
Sg3tf000274g5u
Sg3tf000274g4u
Lg3tf000274g7u
Sg3tf000274g13u
0.0
0.5
1.0
1.5
2.0
Branch Length

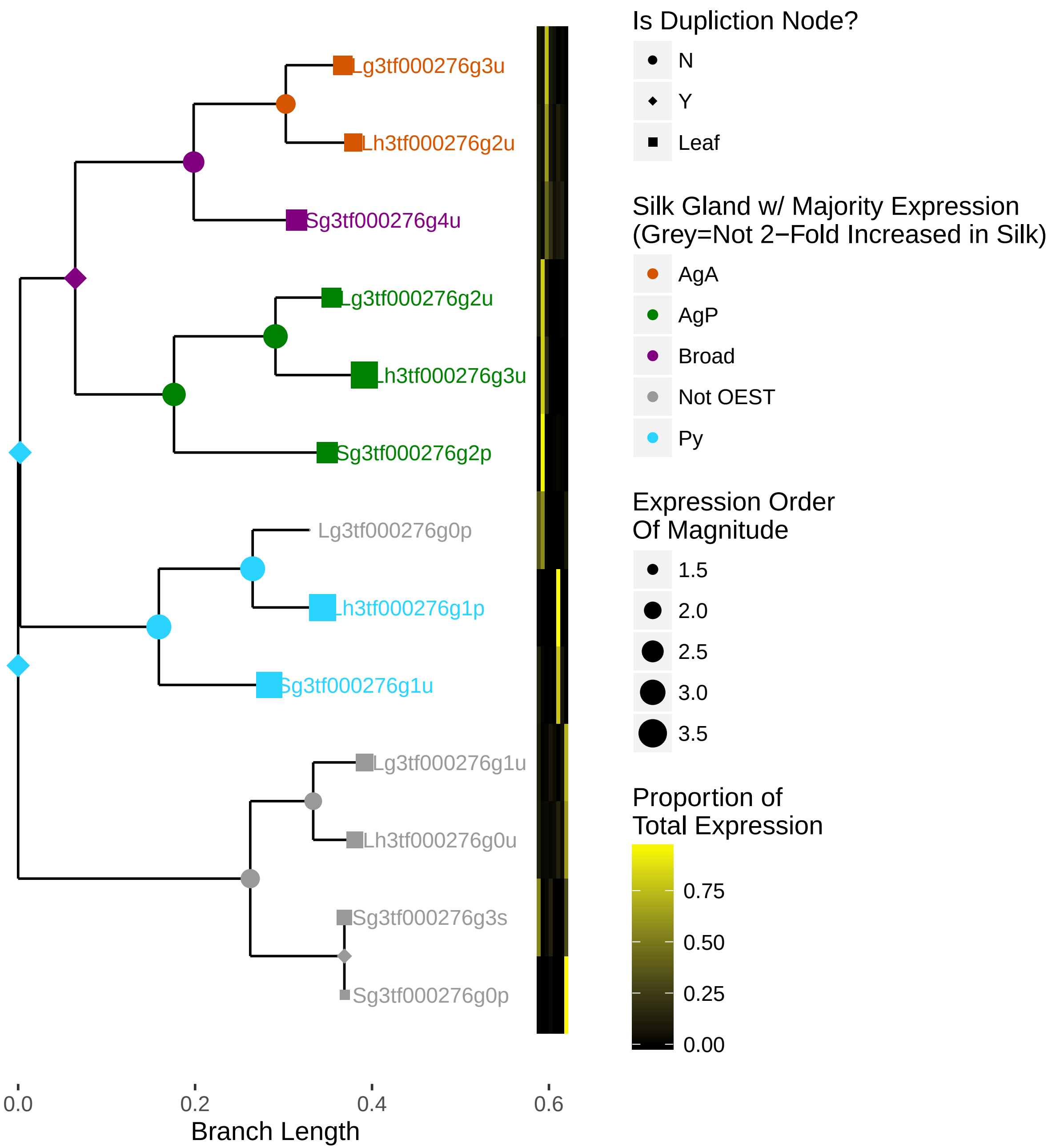

Is Dupliction Node?
N
Y
Leaf
Silk Gland w/ Majority Expression
(Grey=Not 2−Fold Increased in Silk)
AgA
AgP
Broad
Not OEST
Py
Expression Order
Of Magnitude
1.5
2.0
2.5
3.0
3.5
Proportion of
Total Expression
0.75
0.50
0.25
0.00
Lg3tf000276g3u
Lh3tf000276g2u
Sg3tf000276g4u
Lg3tf000276g2u
Lh3tf000276g3u
Sg3tf000276g2p
Lg3tf000276g0p
Lh3tf000276g1p
Sg3tf000276g1u
Lg3tf000276g1u
Lh3tf000276g0u
Sg3tf000276g3s
Sg3tf000276g0p
0.0
0.2
0.4
0.6
Branch Length

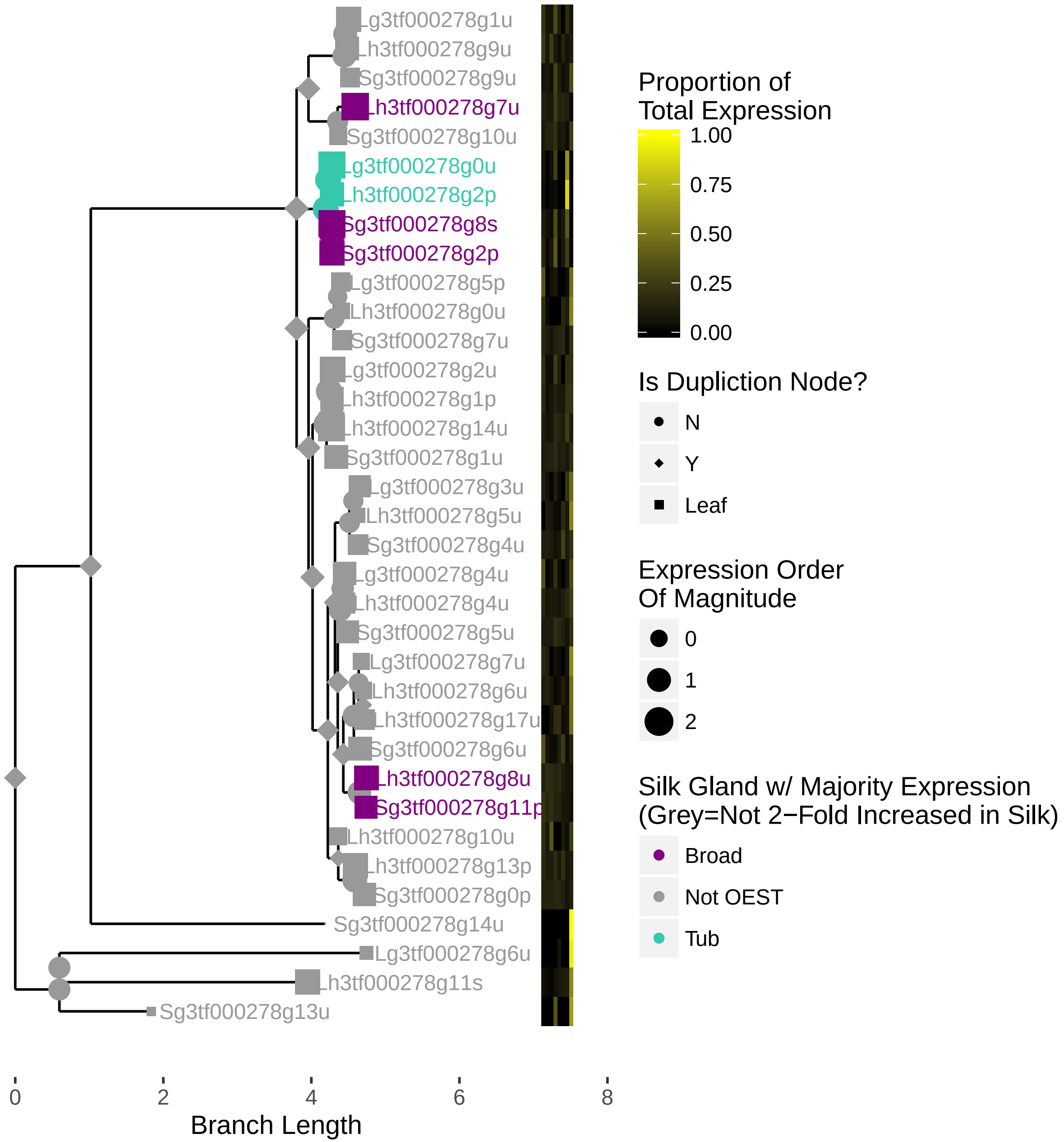

Lg3tf000278g1u
Lh3tf000278g9u
Sg3tf000278g9u
Lh3tf000278g7u
Sg3tf000278g10u
Lg3tf000278g0u
Lh3tf000278g2p
Sg3tf000278g8s
Sg3tf000278g2p
Lg3tf000278g5p
Lh3tf000278g0u
Sg3tf000278g7u
Lg3tf000278g2u
Lh3tf000278g1p
Lh3tf000278g14u
Sg3tf000278g1u
Lg3tf000278g3u
Lh3tf000278g5u
Sg3tf000278g4u
Lg3tf000278g4u
Lh3tf000278g4u
Sg3tf000278g5u
Lg3tf000278g7u
Lh3tf000278g6u
Lh3tf000278g17u
Sg3tf000278g6u
Lh3tf000278g8u
Sg3tf000278g11p
Lh3tf000278g10u
Lh3tf000278g13p
Sg3tf000278g0p
Sg3tf000278g14u
Lg3tf000278g6u
Lh3tf000278g11s
Sg3tf000278g13u
0
2
4
6
8
Branch Length
Proportion of
Total Expression
1.00
0.75
0.50
0.25
0.00
Is Dupliction Node?
N
Y
Leaf
Expression Order
Of Magnitude
0
1
2
Silk Gland w/ Majority Expression
(Grey=Not 2−Fold Increased in Silk)
Broad
Not OEST
Tub

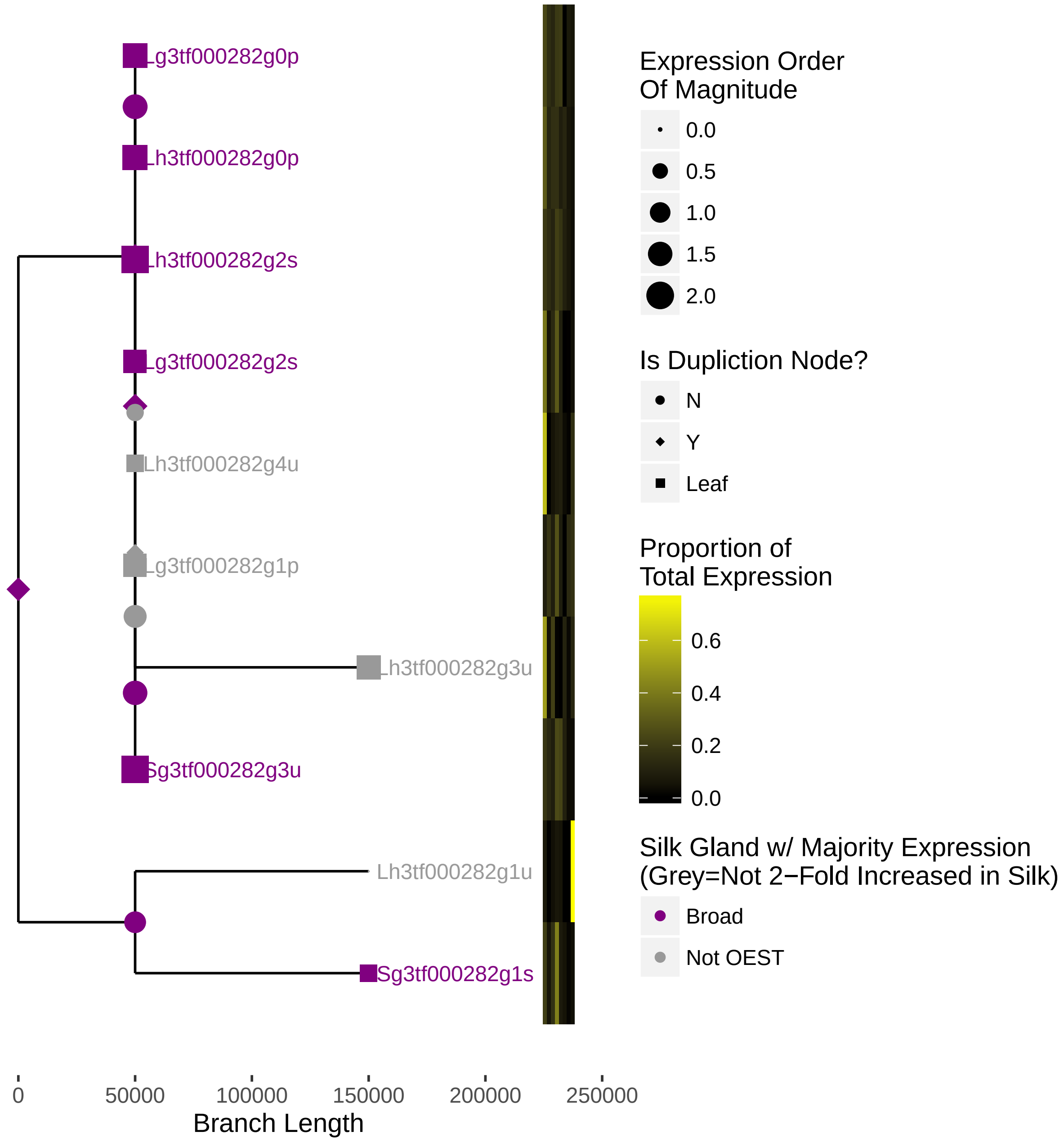

Lg3tf000282g0p
Lh3tf000282g0p
Lh3tf000282g2s
Lg3tf000282g2s
Lh3tf000282g4u
Lg3tf000282g1p
Lh3tf000282g3u
Sg3tf000282g3u
Lh3tf000282g1u
Sg3tf000282g1s
0
50000
100000
150000
200000
250000
Branch Length
Expression Order Of Magnitude
0.0
0.5
1.0
1.5
2.0
Is Dupliction Node?
N
Y
Leaf
Proportion of Total Expression
0.6
0.4
0.2
0.0
Silk Gland w/ Majority Expression (Grey=Not 2−Fold Increased in Silk)
Broad
Not OEST

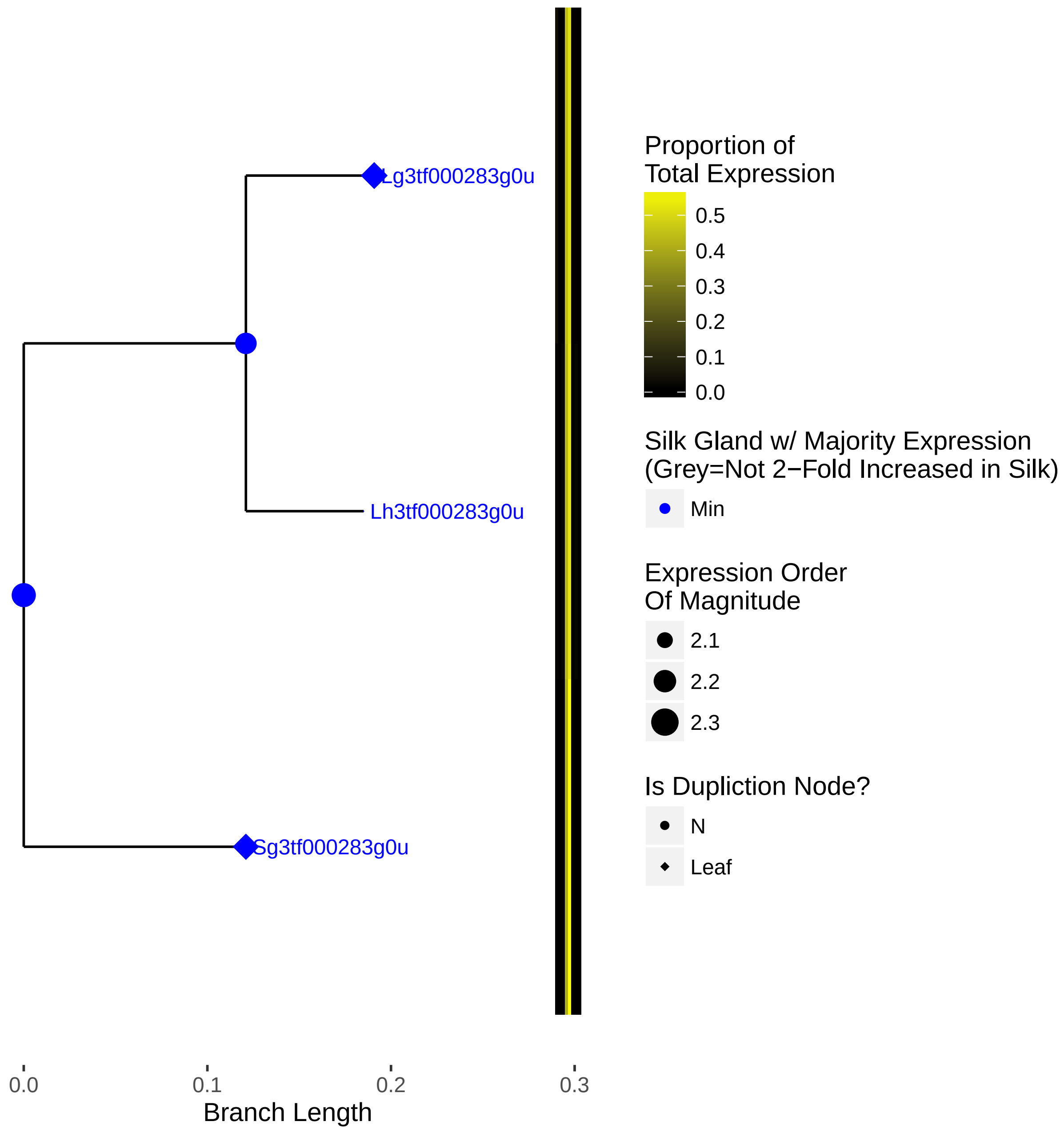

Lg3tf000283g0u
Lh3tf000283g0u
Sg3tf000283g0u
0.0
0.1
0.2
0.3
Branch Length
Proportion of
Total Expression
0.5
0.4
0.3
0.2
0.1
0.0
Silk Gland w/ Majority Expression
(Grey=Not 2−Fold Increased in Silk)
Min
Expression Order
Of Magnitude
2.1
2.2
2.3
Is Dupliction Node?
N
Leaf

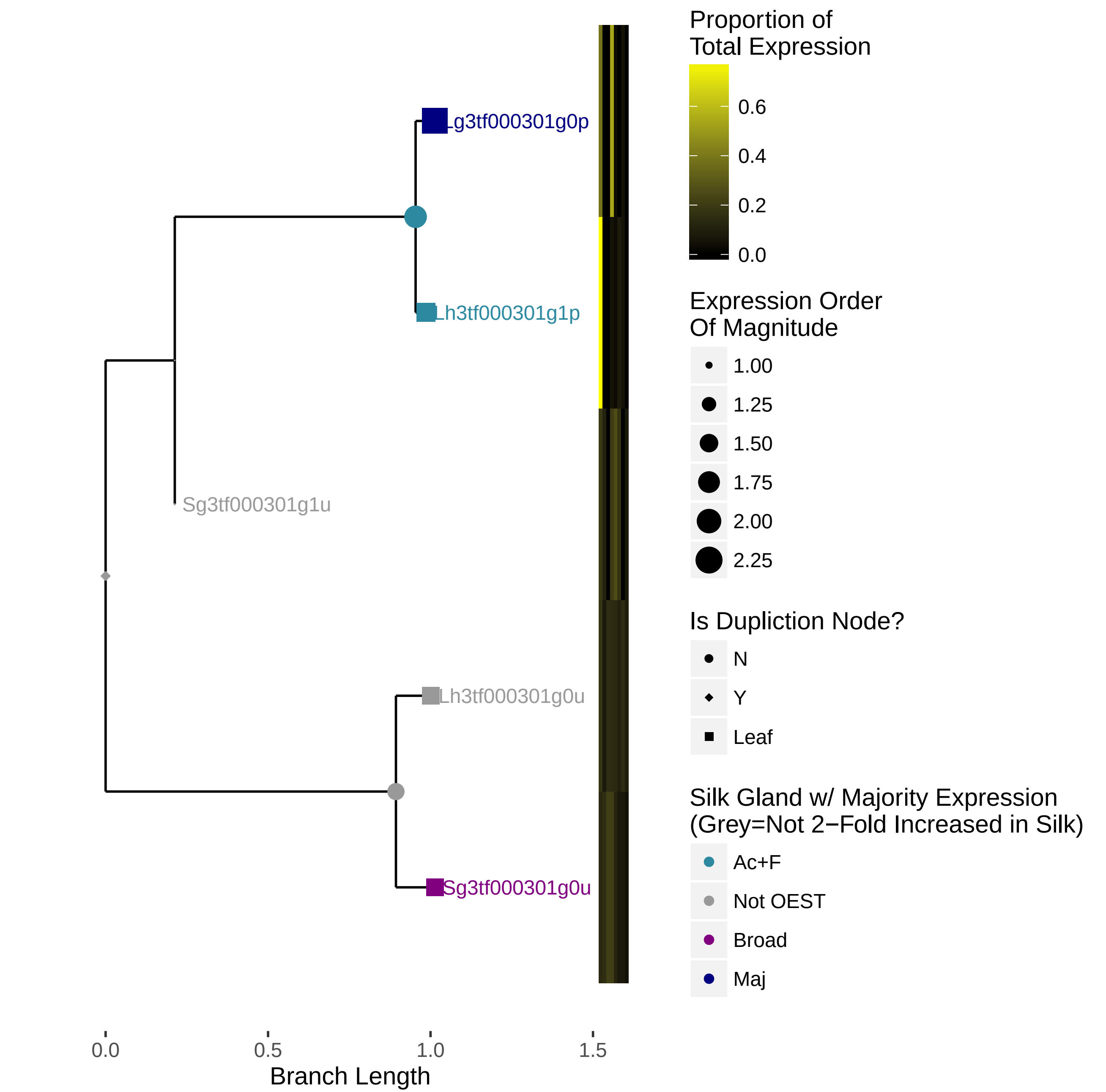

Lg3tf000301g0p
Lh3tf000301g1p
Sg3tf000301g1u
Lh3tf000301g0u
Sg3tf000301g0u
0.0
0.5
1.0
1.5
Branch Length
Proportion of
Total Expression
0.6
0.4
0.2
0.0
Expression Order
Of Magnitude
1.00
1.25
1.50
1.75
2.00
2.25
Is Dupliction Node?
N
Y
Leaf
Silk Gland w/ Majority Expression
(Grey=Not 2−Fold Increased in Silk)
Ac+F
Not OEST
Broad
Maj

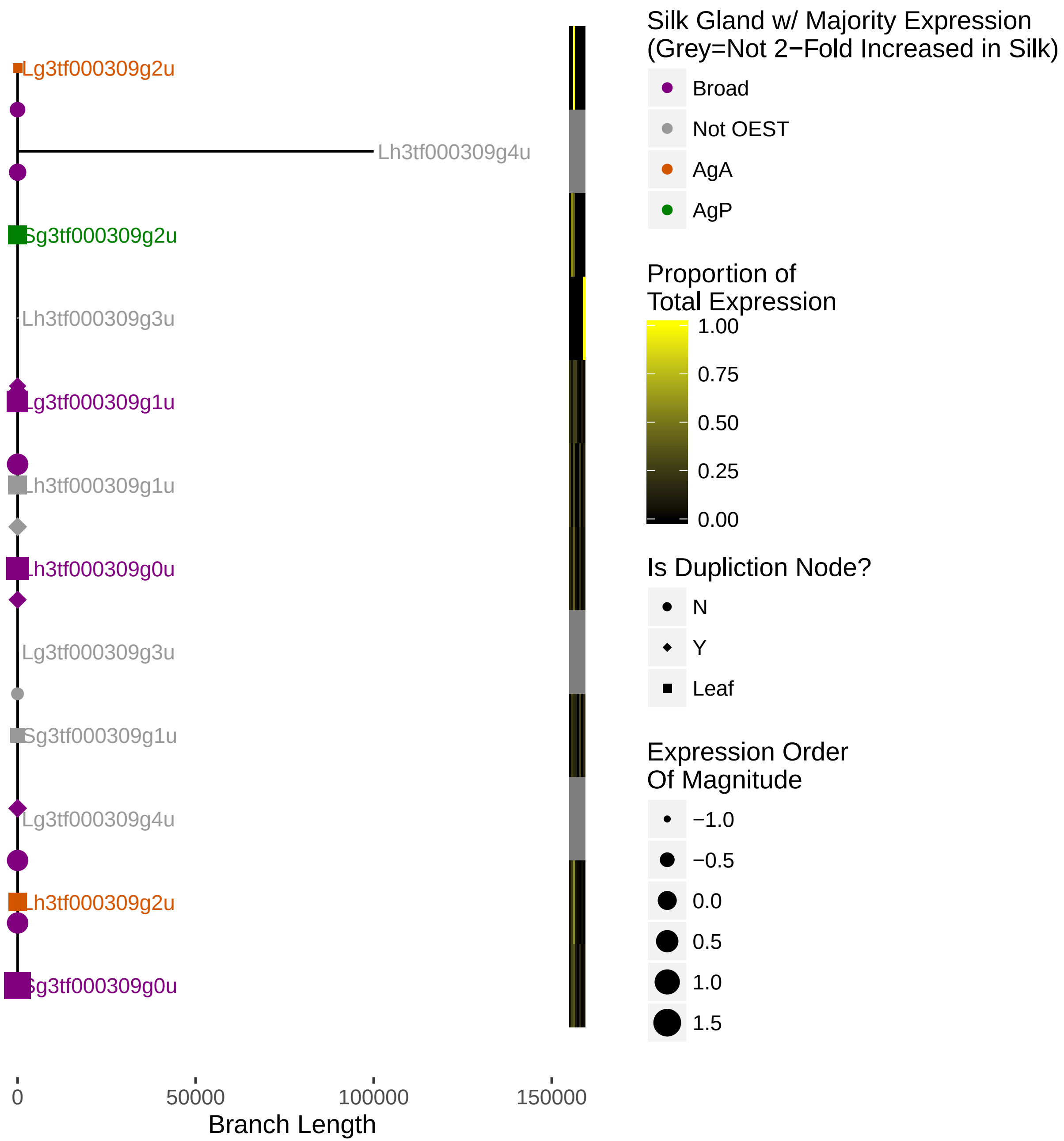

Lg3tf000309g2u
Lh3tf000309g4u
Sg3tf000309g2u
Lh3tf000309g3u
Lg3tf000309g1u
Lh3tf000309g1u
Lh3tf000309g0u
Lg3tf000309g3u
Sg3tf000309g1u
Lg3tf000309g4u
Lh3tf000309g2u
Sg3tf000309g0u
0
50000
100000
150000
Branch Length
Silk Gland w/ Majority Expression
(Grey=Not 2−Fold Increased in Silk)
Broad
Not OEST
AgA
AgP
Proportion of
Total Expression
1.00
0.75
0.50
0.25
0.00
Is Dupliction Node?
N
Y
Leaf
Expression Order
Of Magnitude
−1.0
−0.5
0.0
0.5
1.0
1.5

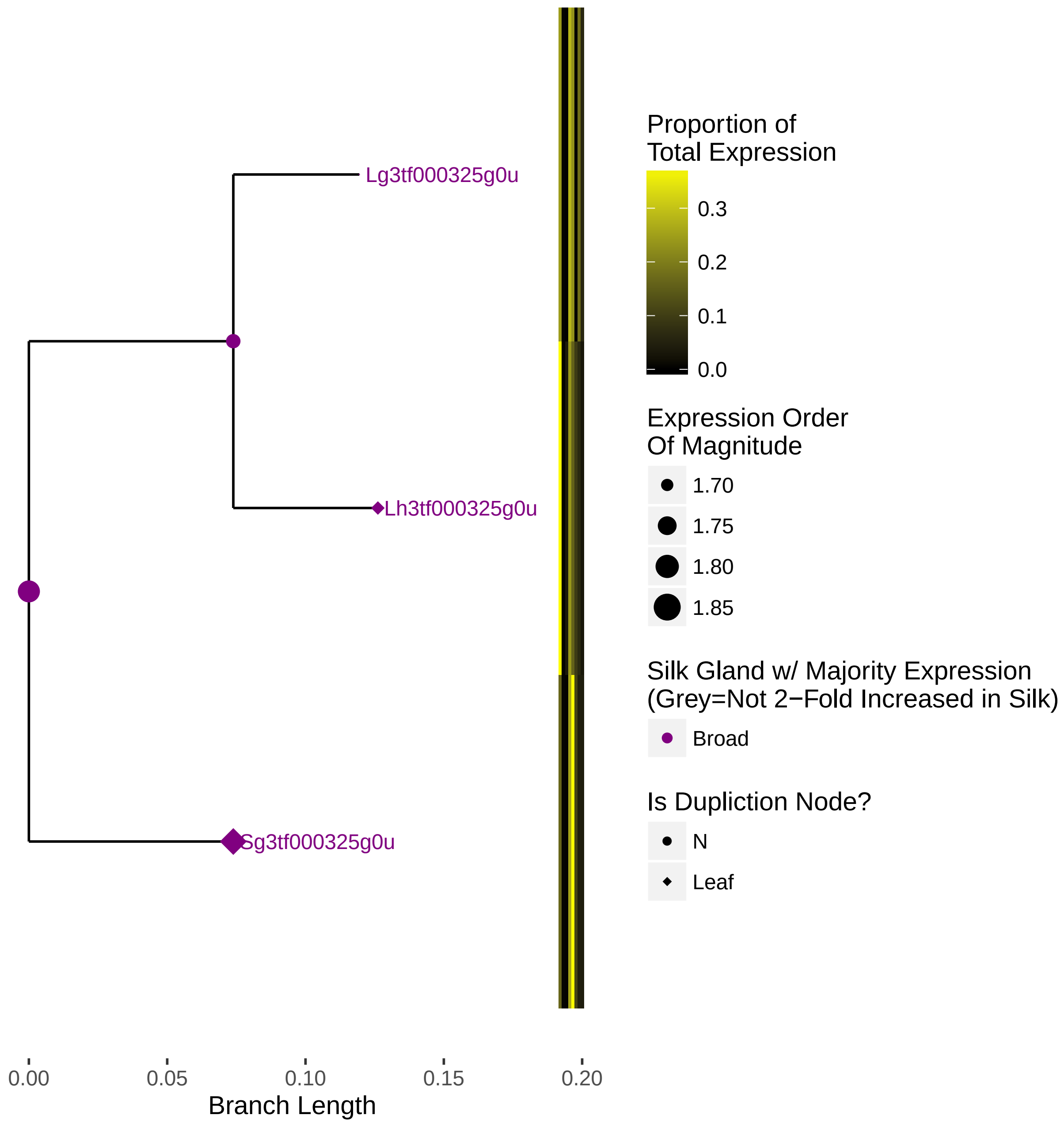

Lg3tf000325g0u
Lh3tf000325g0u
Sg3tf000325g0u
0.00
0.05
0.10
0.15
0.20
Branch Length
Proportion of Total Expression
0.3
0.2
0.1
0.0
Expression Order Of Magnitude
1.70
1.75
1.80
1.85
Silk Gland w/ Majority Expression (Grey=Not 2−Fold Increased in Silk)
Broad
Is Dupliction Node?
N
Leaf

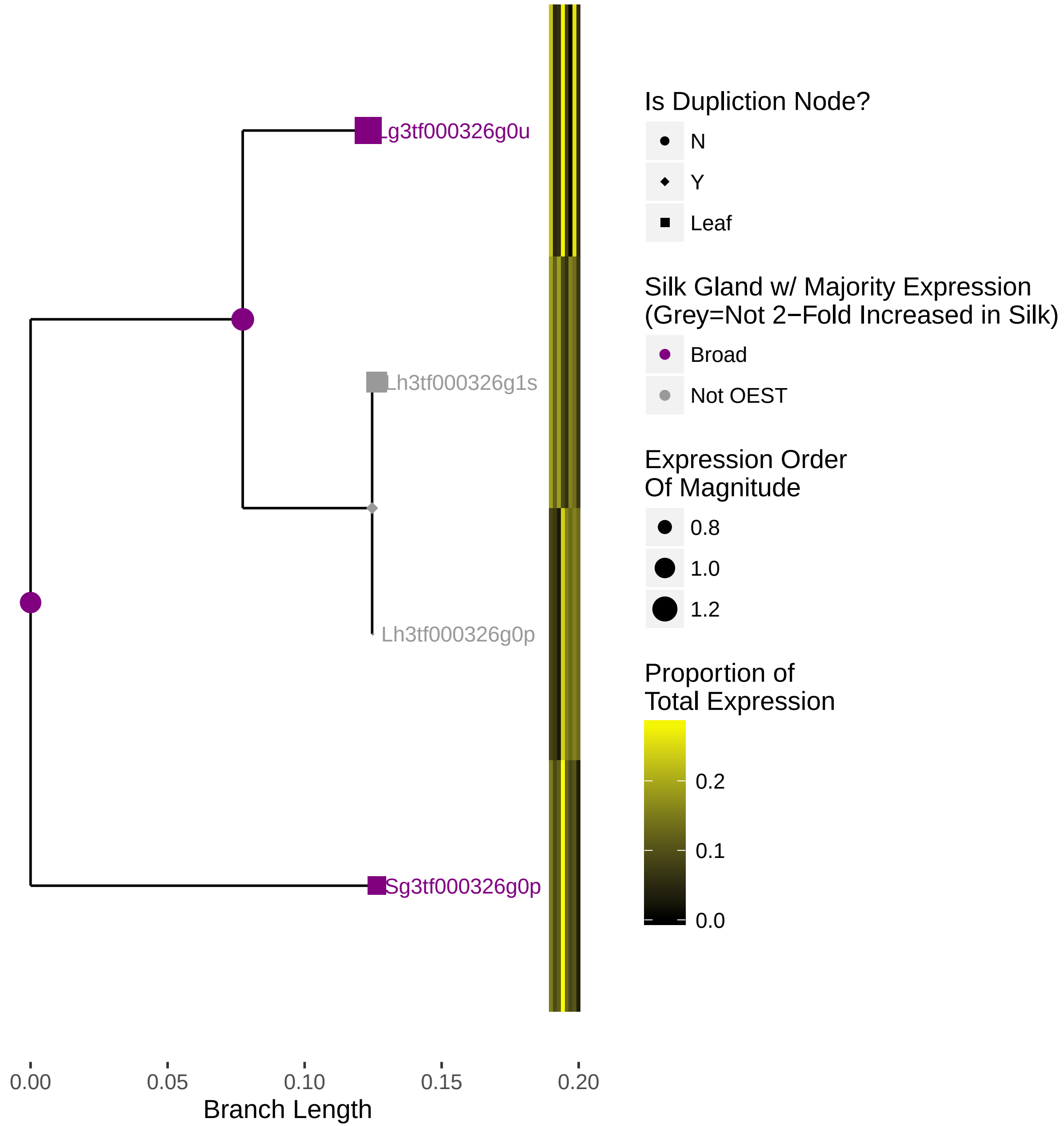

Lg3tf000326g0u
Lh3tf000326g1s
Lh3tf000326g0p
Sg3tf000326g0p
0.00
0.05
0.10
0.15
0.20
Branch Length
Is Dupliction Node?
N
Y
Leaf
Silk Gland w/ Majority Expression
(Grey=Not 2−Fold Increased in Silk)
Broad
Not OEST
Expression Order
Of Magnitude
0.8
1.0
1.2
Proportion of
Total Expression
0.2
0.1
0.0

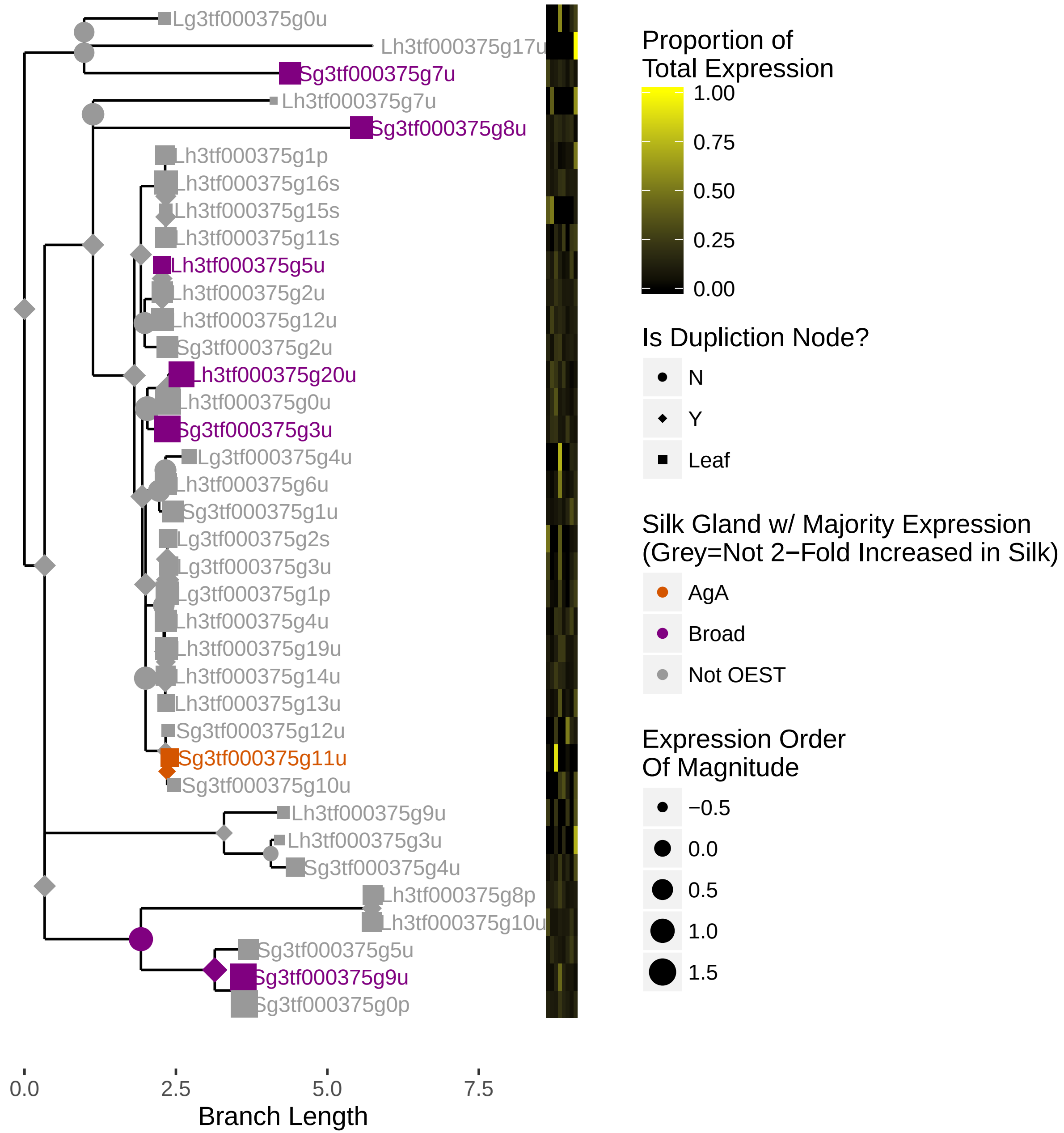

Lg3tf000375g0u
Lh3tf000375g17u
Sg3tf000375g7u
Lh3tf000375g7u
Sg3tf000375g8u
Lh3tf000375g1p
Lh3tf000375g16s
Lh3tf000375g15s
Lh3tf000375g11s
Lh3tf000375g5u
Lh3tf000375g2u
Lh3tf000375g12u
Sg3tf000375g2u
Lh3tf000375g20u
Lh3tf000375g0u
Sg3tf000375g3u
Lg3tf000375g4u
Lh3tf000375g6u
Sg3tf000375g1u
Lg3tf000375g2s
Lg3tf000375g3u
Lg3tf000375g1p
Lh3tf000375g4u
Lh3tf000375g19u
Lh3tf000375g14u
Lh3tf000375g13u
Sg3tf000375g12u
Sg3tf000375g11u
Sg3tf000375g10u
Lh3tf000375g9u
Lh3tf000375g3u
Sg3tf000375g4u
Lh3tf000375g8p
Lh3tf000375g10u
Sg3tf000375g5u
Sg3tf000375g9u
Sg3tf000375g0p
0.0
2.5
5.0
7.5
Branch Length
Proportion of
Total Expression
1.00
0.75
0.50
0.25
0.00
Is Dupliction Node?
N
Y
Leaf
Silk Gland w/ Majority Expression
(Grey=Not 2−Fold Increased in Silk)
AgA
Broad
Not OEST
Expression Order
Of Magnitude
−0.5
0.0
0.5
1.0
1.5

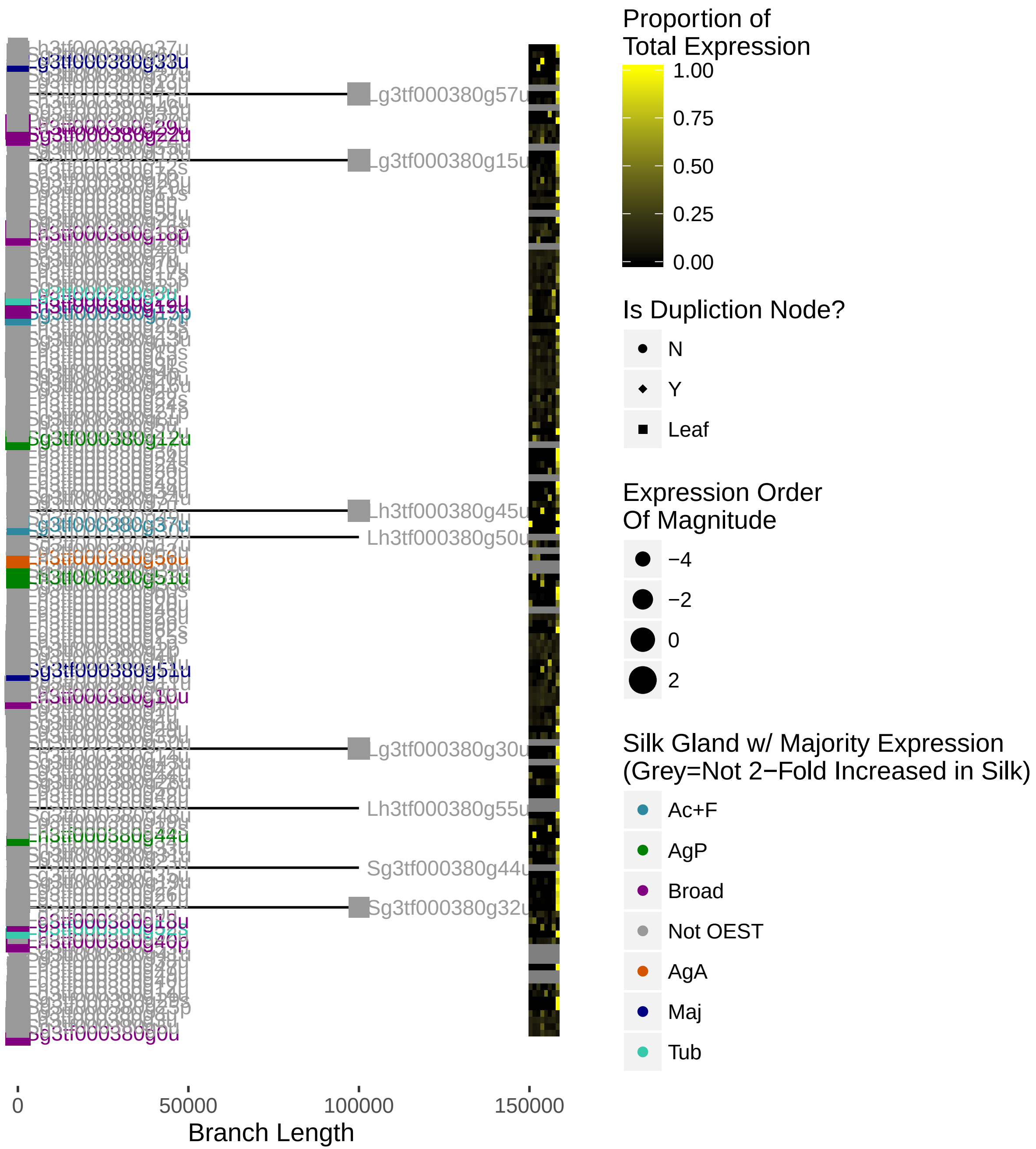

Proportion of Total Expression
1.00
0.75
0.50
0.25
0.00
Is Dupliction Node?
N
Y
Leaf
Expression Order Of Magnitude
−4
−2
0
2
Silk Gland w/ Majority Expression (Grey=Not 2−Fold Increased in Silk)
Ac+F
AgP
Broad
Not OEST
AgA
Maj
Tub
Lg3tf000380g57u
Lg3tf000380g15u
Lh3tf000380g45u
Lh3tf000380g50u
Lg3tf000380g30u
Lh3tf000380g55u
Sg3tf000380g44u
Sg3tf000380g32u
Sg3tf000380g22u
Sg3tf000380g12u
Lh3tf000380g51u
Sg3tf000380g51u
Sg3tf000380g0u
0
50000
100000
150000
Branch Length

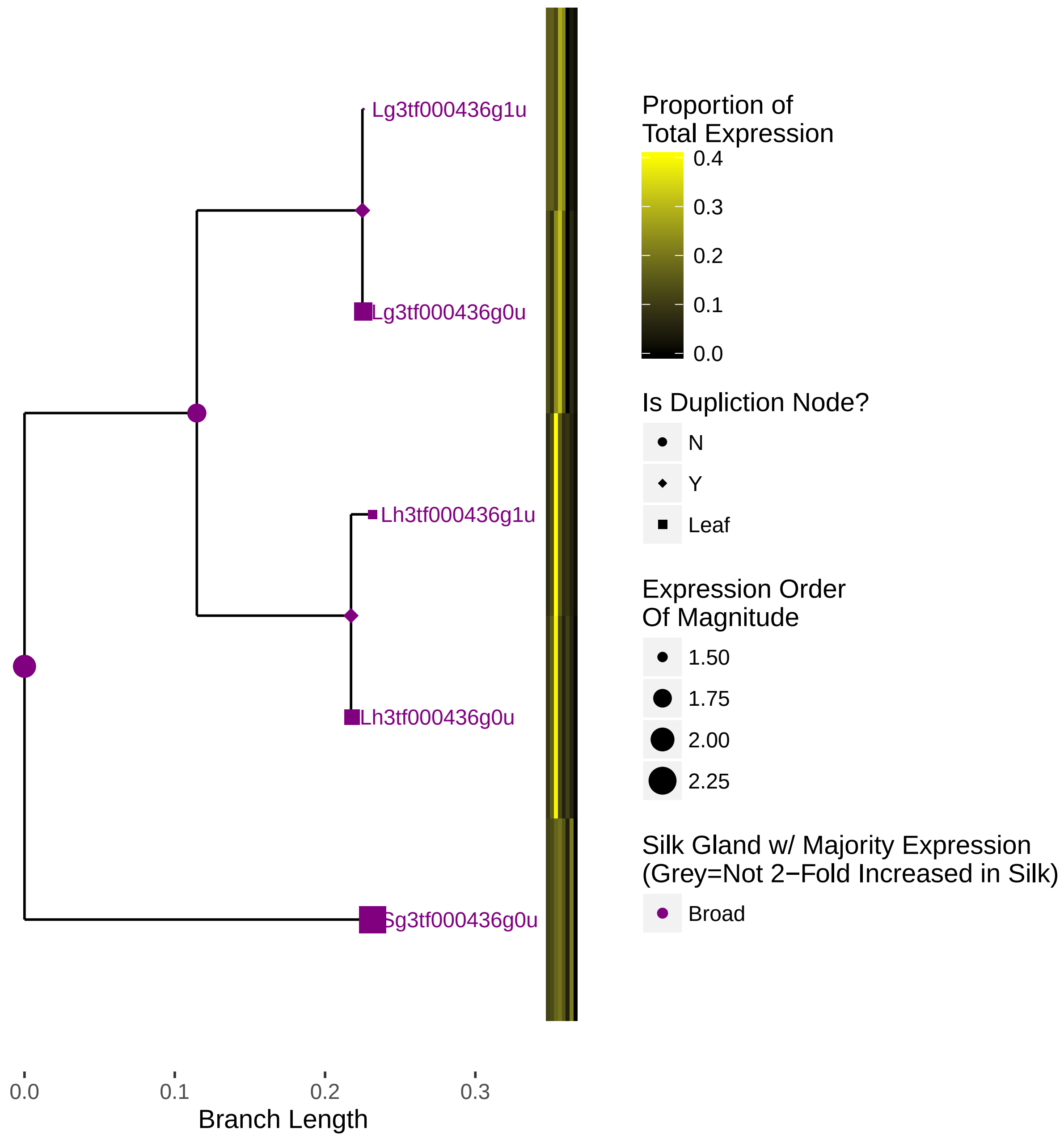

Lg3tf000436g1u
Lg3tf000436g0u
Lh3tf000436g1u
Lh3tf000436g0u
Sg3tf000436g0u
0.0
0.1
0.2
0.3
Branch Length
Proportion of
Total Expression
0.4
0.3
0.2
0.1
0.0
Is Dupliction Node?
N
Y
Leaf
Expression Order
Of Magnitude
1.50
1.75
2.00
2.25
Silk Gland w/ Majority Expression
(Grey=Not 2−Fold Increased in Silk)
Broad

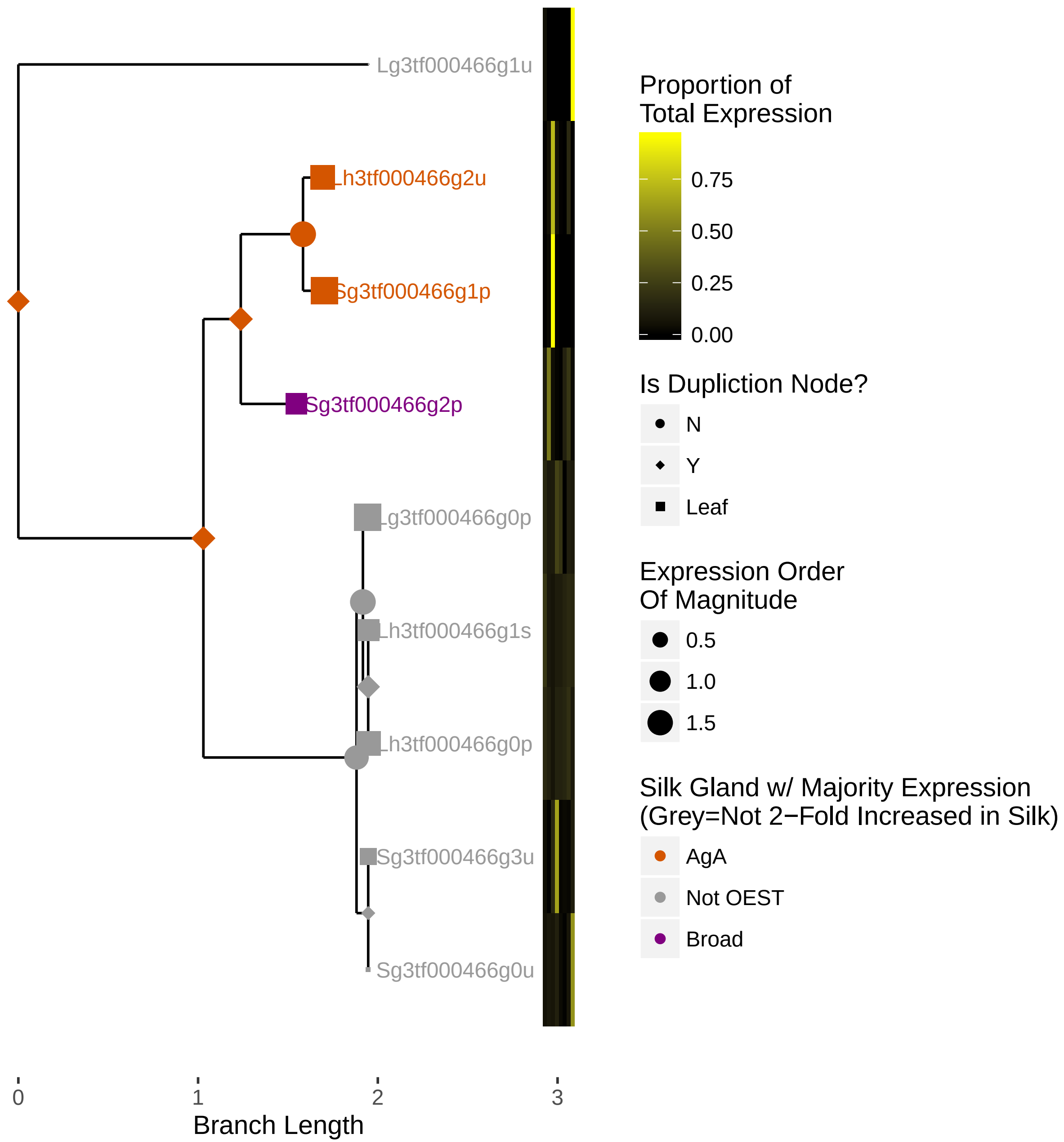

Lg3tf000466g1u
Lh3tf000466g2u
Sg3tf000466g1p
Sg3tf000466g2p
Lg3tf000466g0p
Lh3tf000466g1s
Lh3tf000466g0p
Sg3tf000466g3u
Sg3tf000466g0u
0
1
2
3
Branch Length
Proportion of
Total Expression
0.75
0.50
0.25
0.00
Is Dupliction Node?
N
Y
Leaf
Expression Order
Of Magnitude
0.5
1.0
1.5
Silk Gland w/ Majority Expression
(Grey=Not 2−Fold Increased in Silk)
AgA
Not OEST
Broad

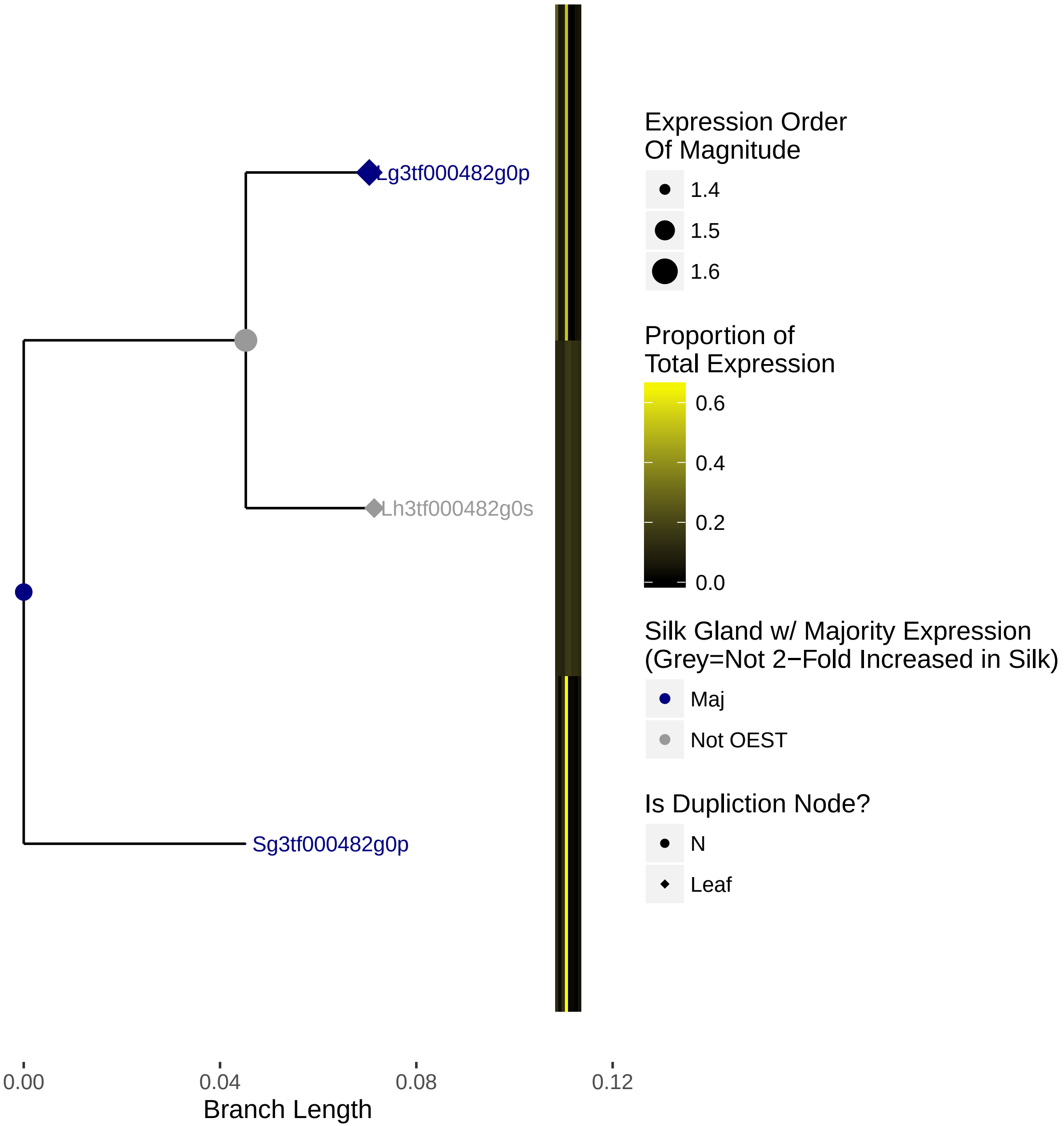
Lg3tf000482g0p
Lh3tf000482g0s
Sg3tf000482g0p
Expression Order
Of Magnitude
1.4
1.5
1.6
Proportion of
Total Expression
0.6
0.4
0.2
0.0
Silk Gland w/ Majority Expression
(Grey=Not 2−Fold Increased in Silk)
Maj
Not OEST
Is Dupliction Node?
N
Leaf
0.00
0.04
0.08
0.12
Branch Length

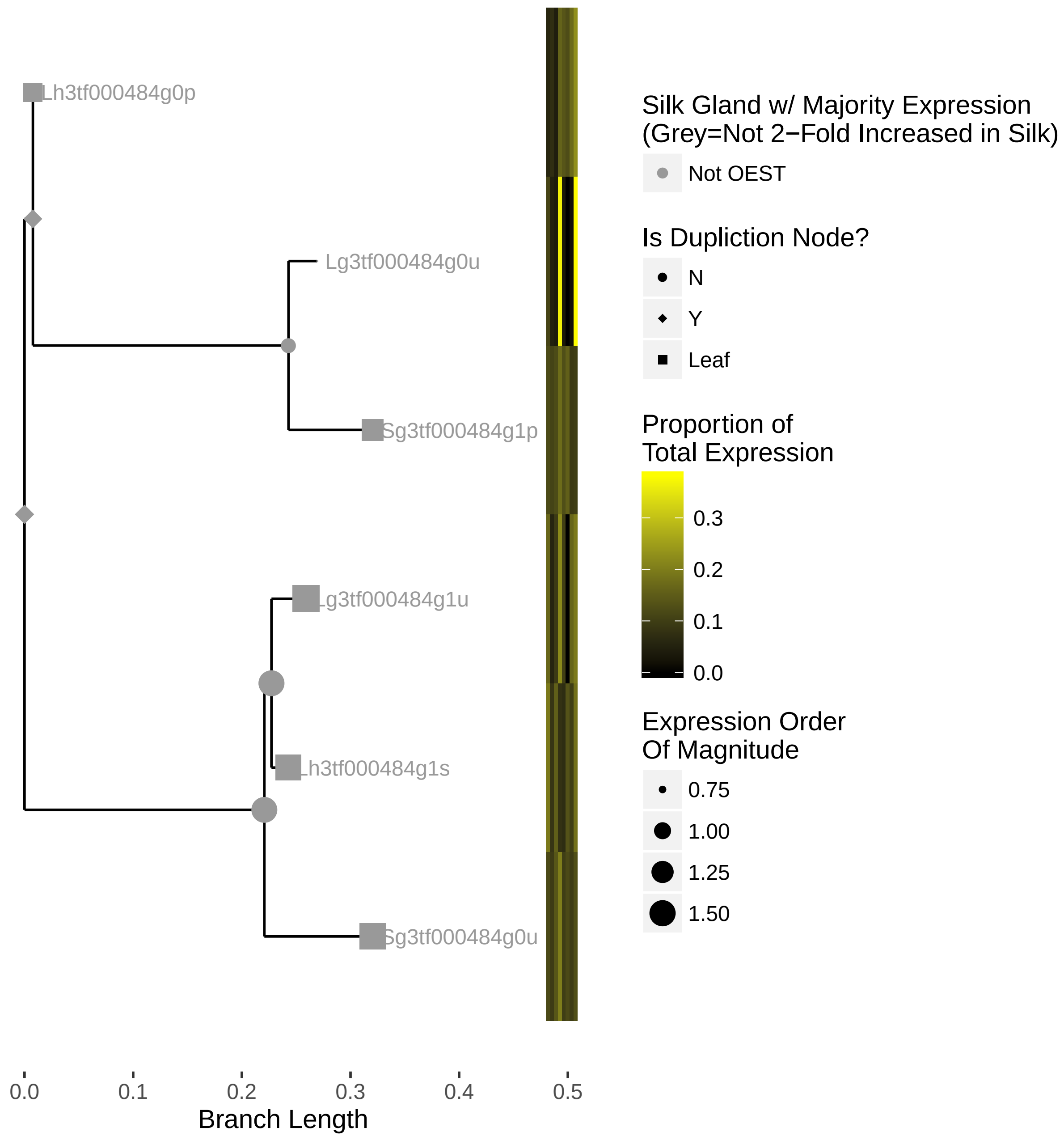

Lh3tf000484g0p
Lg3tf000484g0u
Sg3tf000484g1p
Lg3tf000484g1u
Lh3tf000484g1s
Sg3tf000484g0u
0.0
0.1
0.2
0.3
0.4
0.5
Branch Length
Silk Gland w/ Majority Expression
(Grey=Not 2−Fold Increased in Silk)
Not OEST
Is Dupliction Node?
N
Y
Leaf
Proportion of
Total Expression
0.3
0.2
0.1
0.0
Expression Order
Of Magnitude
0.75
1.00
1.25
1.50

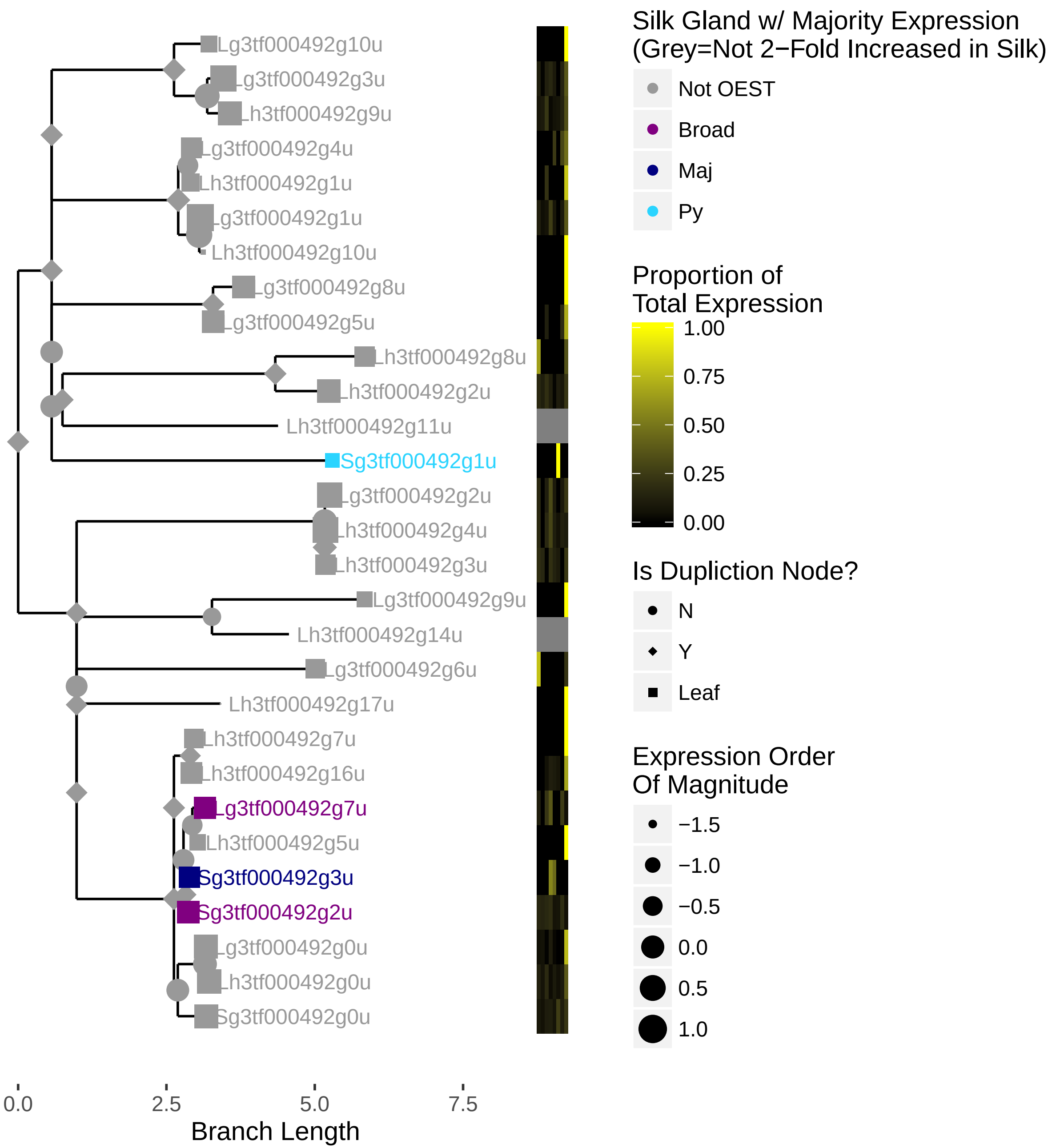

Lg3tf000492g10u
Lg3tf000492g3u
Lh3tf000492g9u
Lg3tf000492g4u
Lh3tf000492g1u
Lg3tf000492g1u
Lh3tf000492g10u
Lg3tf000492g8u
Lg3tf000492g5u
Lh3tf000492g8u
Lh3tf000492g2u
Lh3tf000492g11u
Sg3tf000492g1u
Lg3tf000492g2u
Lh3tf000492g4u
Lh3tf000492g3u
Lg3tf000492g9u
Lh3tf000492g14u
Lg3tf000492g6u
Lh3tf000492g17u
Lh3tf000492g7u
Lh3tf000492g16u
Lg3tf000492g7u
Lh3tf000492g5u
Sg3tf000492g3u
Sg3tf000492g2u
Lg3tf000492g0u
Lh3tf000492g0u
Sg3tf000492g0u
0.0
2.5
5.0
7.5
Branch Length
Silk Gland w/ Majority Expression
(Grey=Not 2−Fold Increased in Silk)
Not OEST
Broad
Maj
Py
Proportion of
Total Expression
1.00
0.75
0.50
0.25
0.00
Is Dupliction Node?
N
Y
Leaf
Expression Order
Of Magnitude
−1.5
−1.0
−0.5
0.0
0.5
1.0

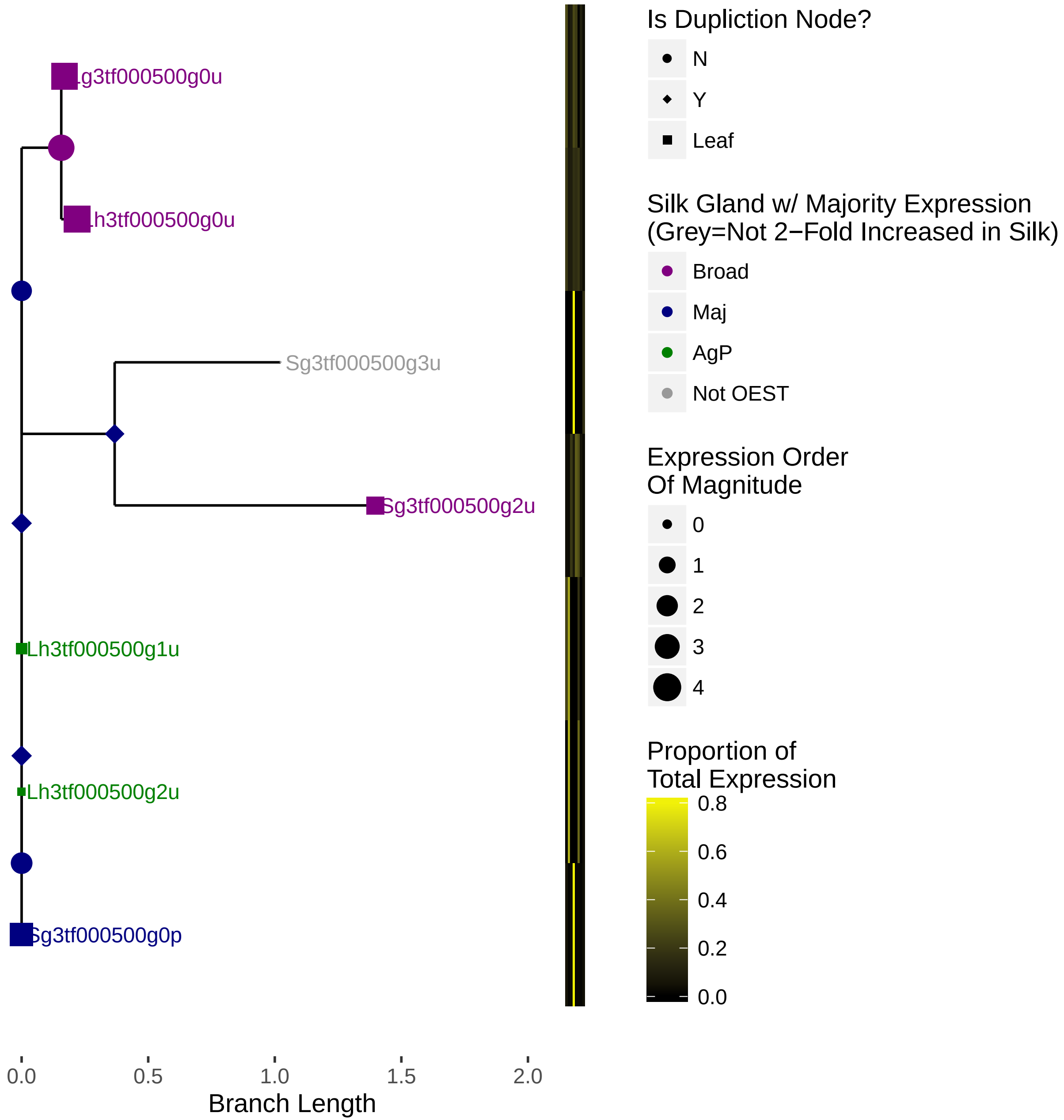

.g3tf000500g0u
.h3tf000500g0u
Sg3tf000500g3u
Sg3tf000500g2u
Lh3tf000500g1u
Lh3tf000500g2u
Sg3tf000500g0p
0.0
0.5
1.0
1.5
2.0
Branch Length
Is Dupliction Node?
N
Y
Leaf
Silk Gland w/ Majority Expression
(Grey=Not 2−Fold Increased in Silk)
Broad
Maj
AgP
Not OEST
Expression Order
Of Magnitude
0
1
2
3
4
Proportion of
Total Expression
0.8
0.6
0.4
0.2
0.0

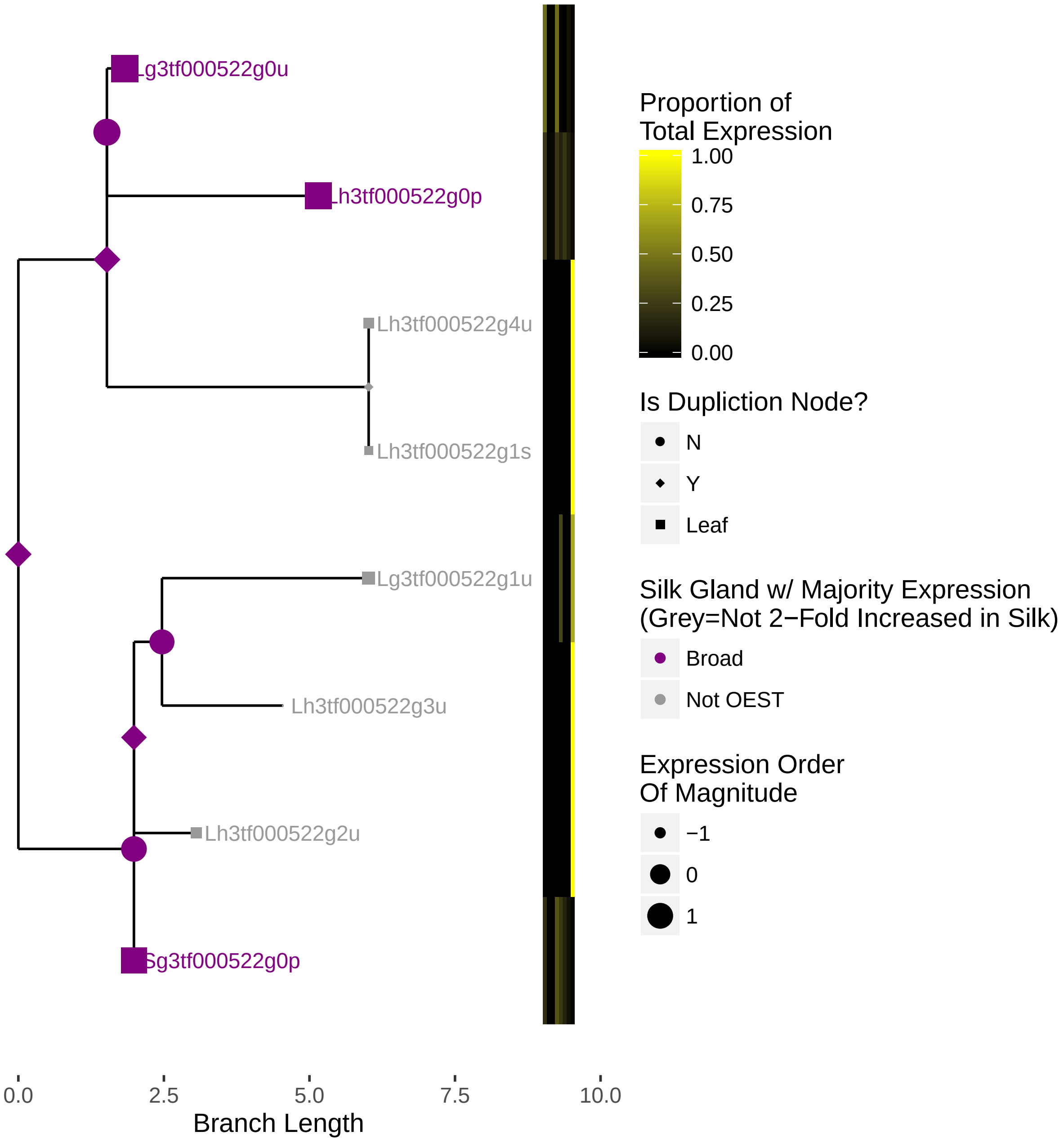

Lg3tf000522g0u
Lh3tf000522g0p
Lh3tf000522g4u
Lh3tf000522g1s
Lg3tf000522g1u
Lh3tf000522g3u
Lh3tf000522g2u
Sg3tf000522g0p
0.0
2.5
5.0
7.5
10.0
Branch Length
Proportion of Total Expression
1.00
0.75
0.50
0.25
0.00
Is Dupliction Node?
N
Y
Leaf
Silk Gland w/ Majority Expression (Grey=Not 2−Fold Increased in Silk)
Broad
Not OEST
Expression Order Of Magnitude
−1
0
1

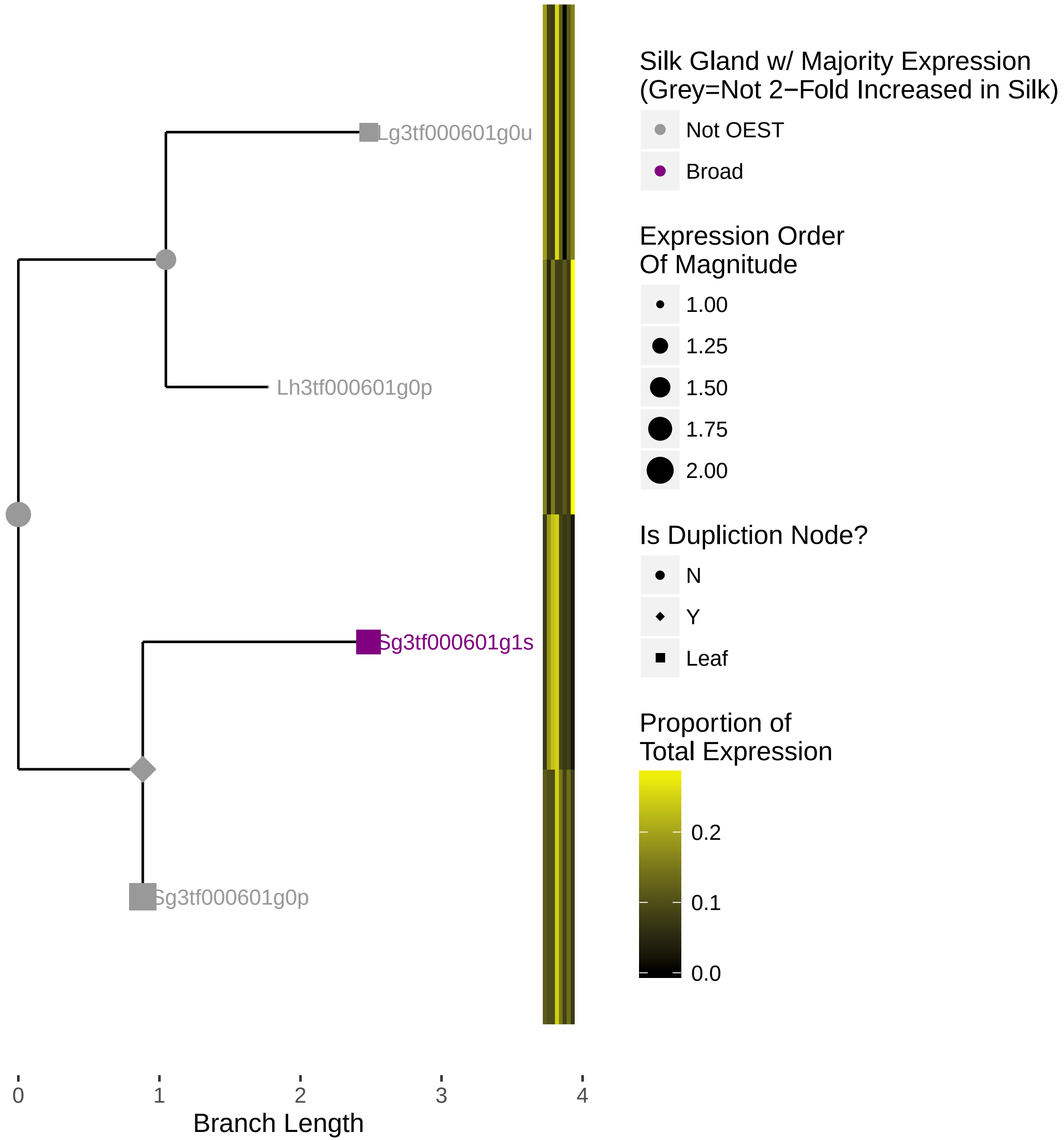

Lg3tf000601g0u
Lh3tf000601g0p
Sg3tf000601g1s
Sg3tf000601g0p
Silk Gland w/ Majority Expression
(Grey=Not 2−Fold Increased in Silk)
Not OEST
Broad
Expression Order
Of Magnitude
1.00
1.25
1.50
1.75
2.00
Is Dupliction Node?
N
Y
Leaf
Proportion of
Total Expression
0.2
0.1
0.0
0
1
2
3
4
Branch Length

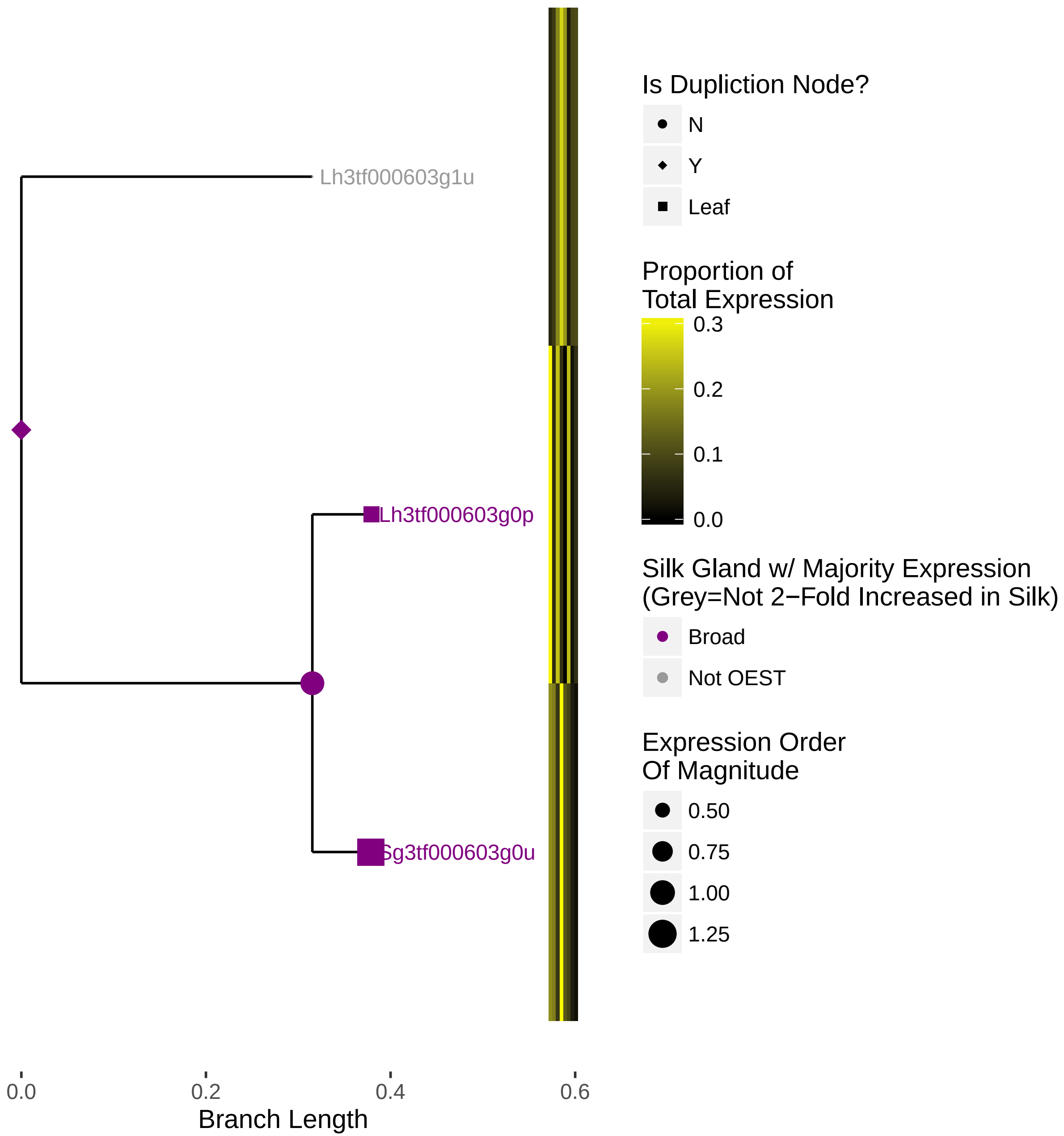
Lh3tf000603g1u
Lh3tf000603g0p
Sg3tf000603g0u
0.0
0.2
0.4
0.6
Branch Length
Is Dupliction Node?
N
Y
Leaf
Proportion of
Total Expression
0.3
0.2
0.1
0.0
Silk Gland w/ Majority Expression
(Grey=Not 2−Fold Increased in Silk)
Broad
Not OEST
Expression Order
Of Magnitude
0.50
0.75
1.00
1.25

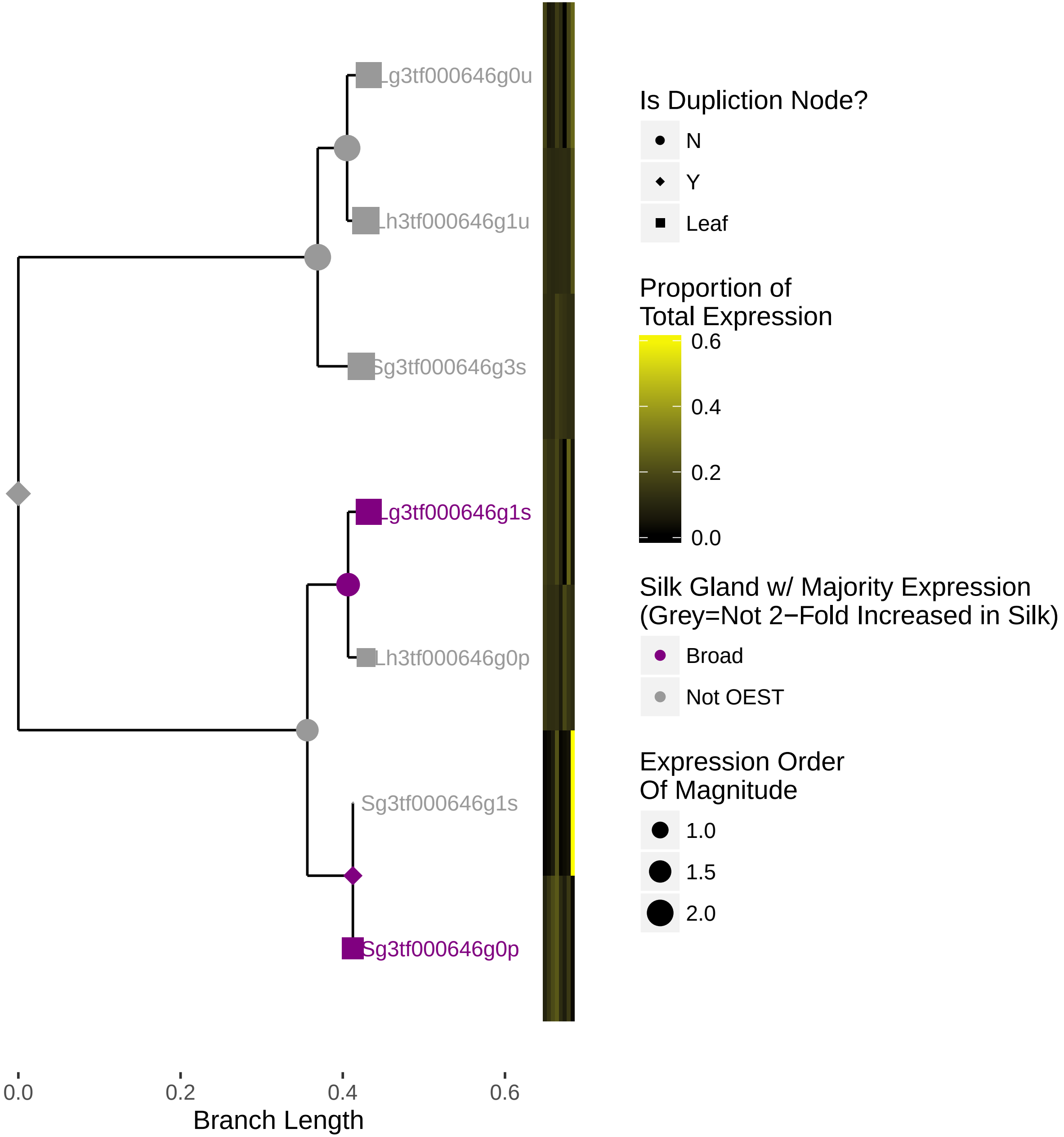
Lg3tf000646g0u
Lh3tf000646g1u
Sg3tf000646g3s
Lg3tf000646g1s
Lh3tf000646g0p
Sg3tf000646g1s
Sg3tf000646g0p
0.0
0.2
0.4
0.6
Branch Length
Is Dupliction Node?
N
Y
Leaf
Proportion of
Total Expression
0.6
0.4
0.2
0.0
Silk Gland w/ Majority Expression
(Grey=Not 2−Fold Increased in Silk)
Broad
Not OEST
Expression Order
Of Magnitude
1.0
1.5
2.0

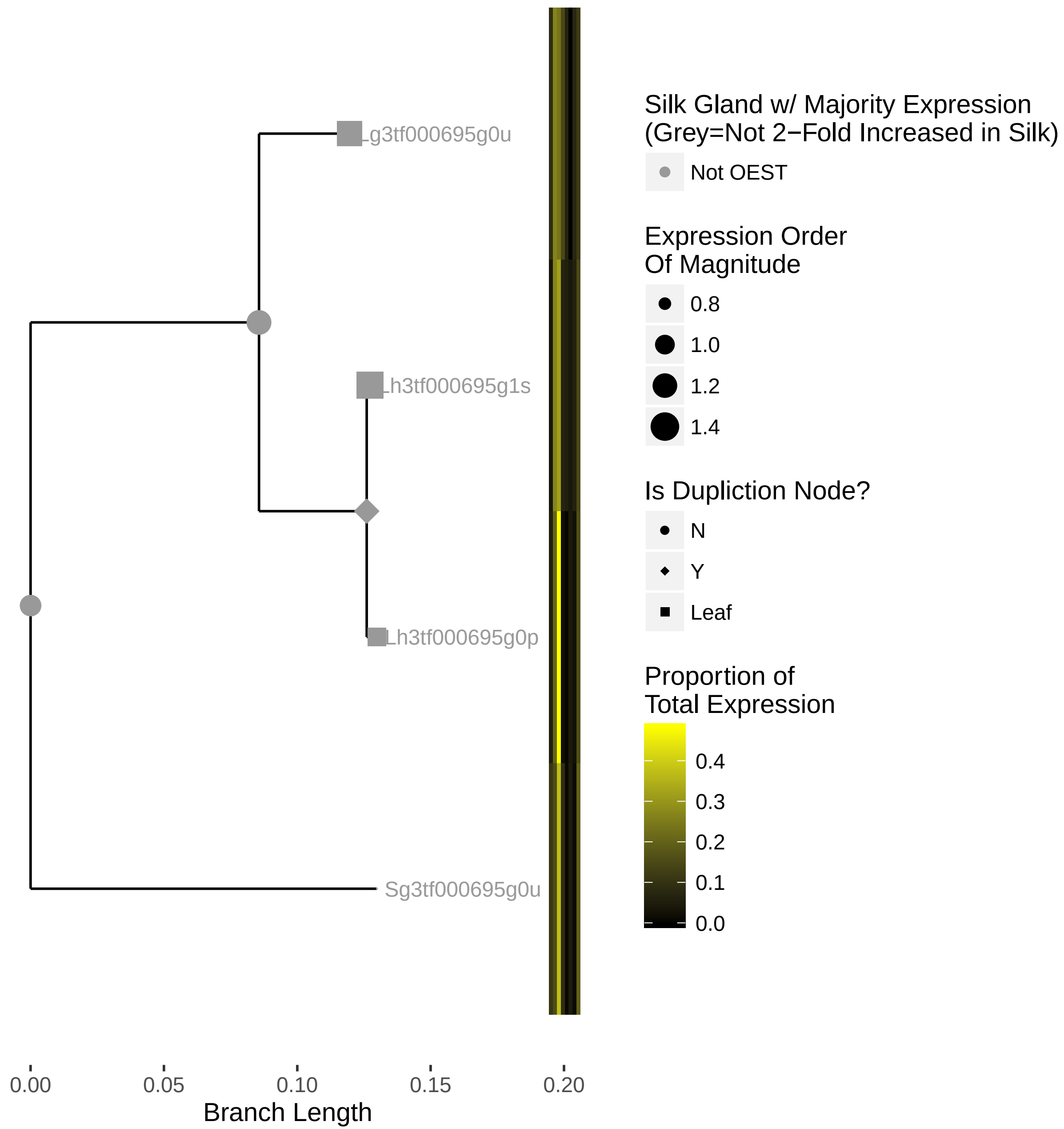

Lg3tf000695g0u
Lh3tf000695g1s
Lh3tf000695g0p
Sg3tf000695g0u
0.00
0.05
0.10
0.15
0.20
Branch Length
Silk Gland w/ Majority Expression
(Grey=Not 2−Fold Increased in Silk)
Not OEST
Expression Order
Of Magnitude
0.8
1.0
1.2
1.4
Is Dupliction Node?
N
Y
Leaf
Proportion of
Total Expression
0.4
0.3
0.2
0.1
0.0

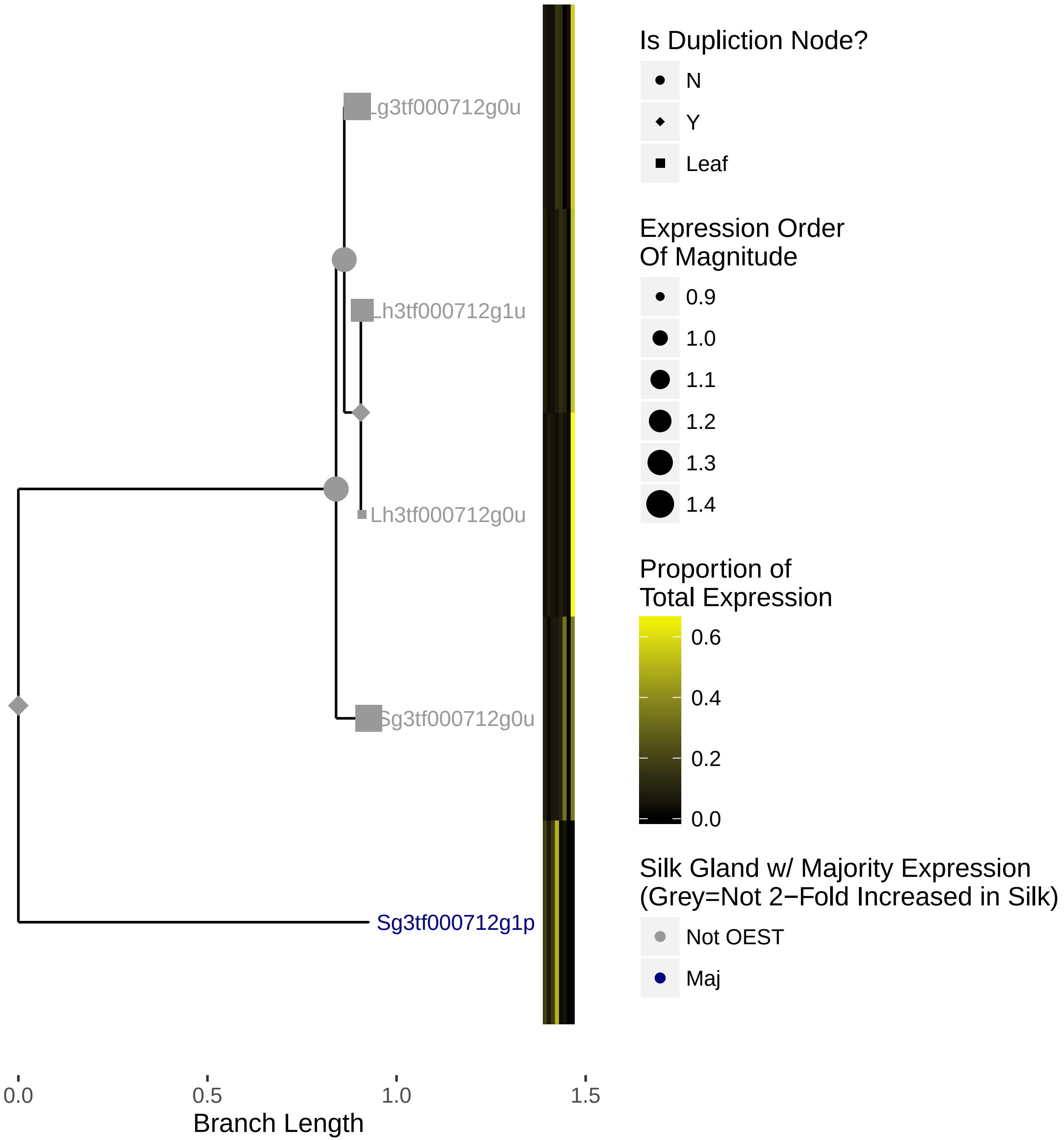

Lg3tf000712g0u
Lh3tf000712g1u
Lh3tf000712g0u
Sg3tf000712g0u
Sg3tf000712g1p
0.0
0.5
1.0
1.5
Branch Length
Is Dupliction Node?
N
Y
Leaf
Expression Order
Of Magnitude
0.9
1.0
1.1
1.2
1.3
1.4
Proportion of
Total Expression
0.6
0.4
0.2
0.0
Silk Gland w/ Majority Expression
(Grey=Not 2−Fold Increased in Silk)
Not OEST
Maj

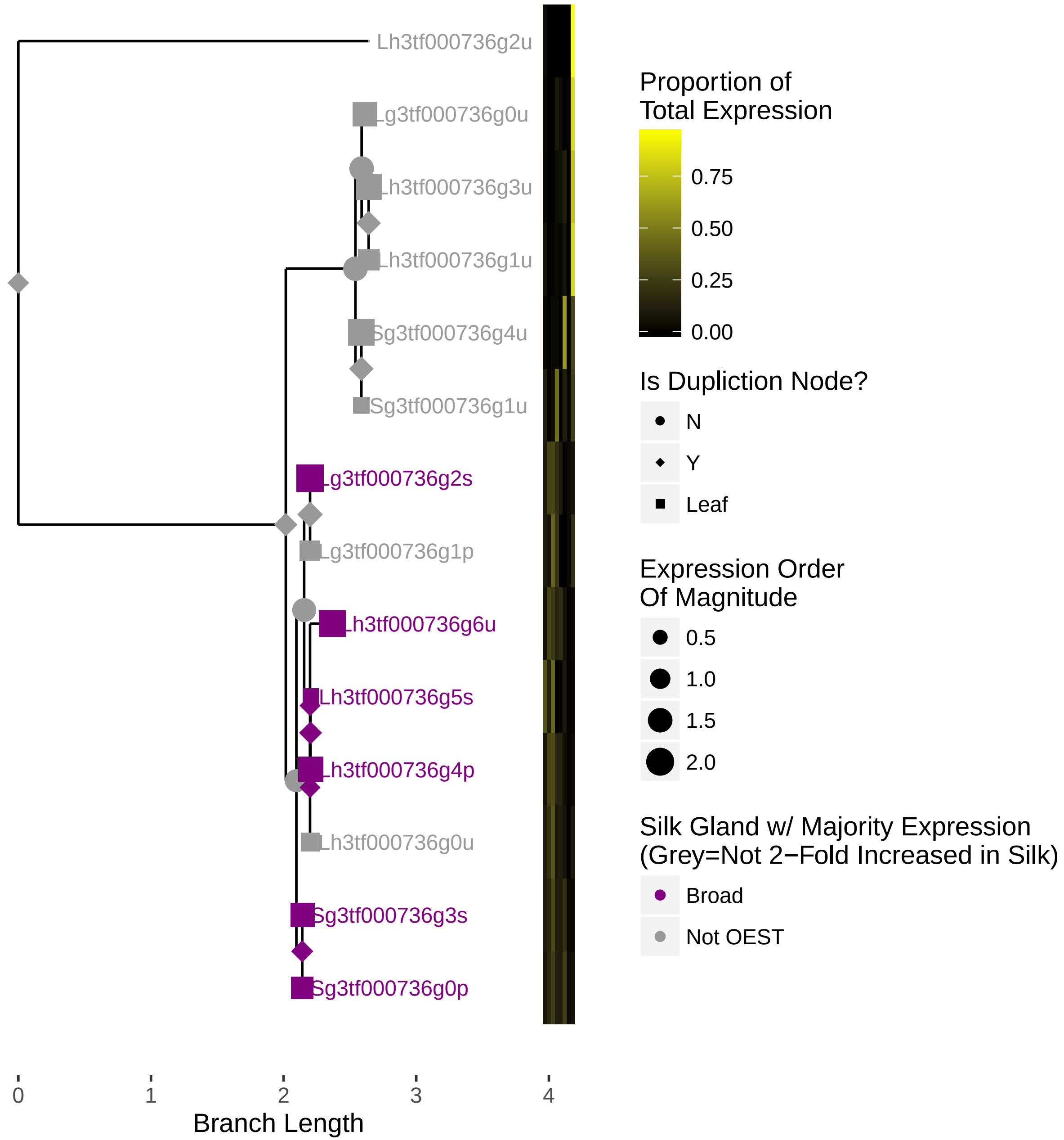

Lh3tf000736g2u
Lg3tf000736g0u
Lh3tf000736g3u
Lh3tf000736g1u
Sg3tf000736g4u
Sg3tf000736g1u
Lg3tf000736g2s
Lg3tf000736g1p
Lh3tf000736g6u
Lh3tf000736g5s
Lh3tf000736g4p
Lh3tf000736g0u
Sg3tf000736g3s
Sg3tf000736g0p
0
1
2
3
4
Branch Length
Proportion of
Total Expression
0.75
0.50
0.25
0.00
Is Dupliction Node?
N
Y
Leaf
Expression Order
Of Magnitude
0.5
1.0
1.5
2.0
Silk Gland w/ Majority Expression
(Grey=Not 2−Fold Increased in Silk)
Broad
Not OEST

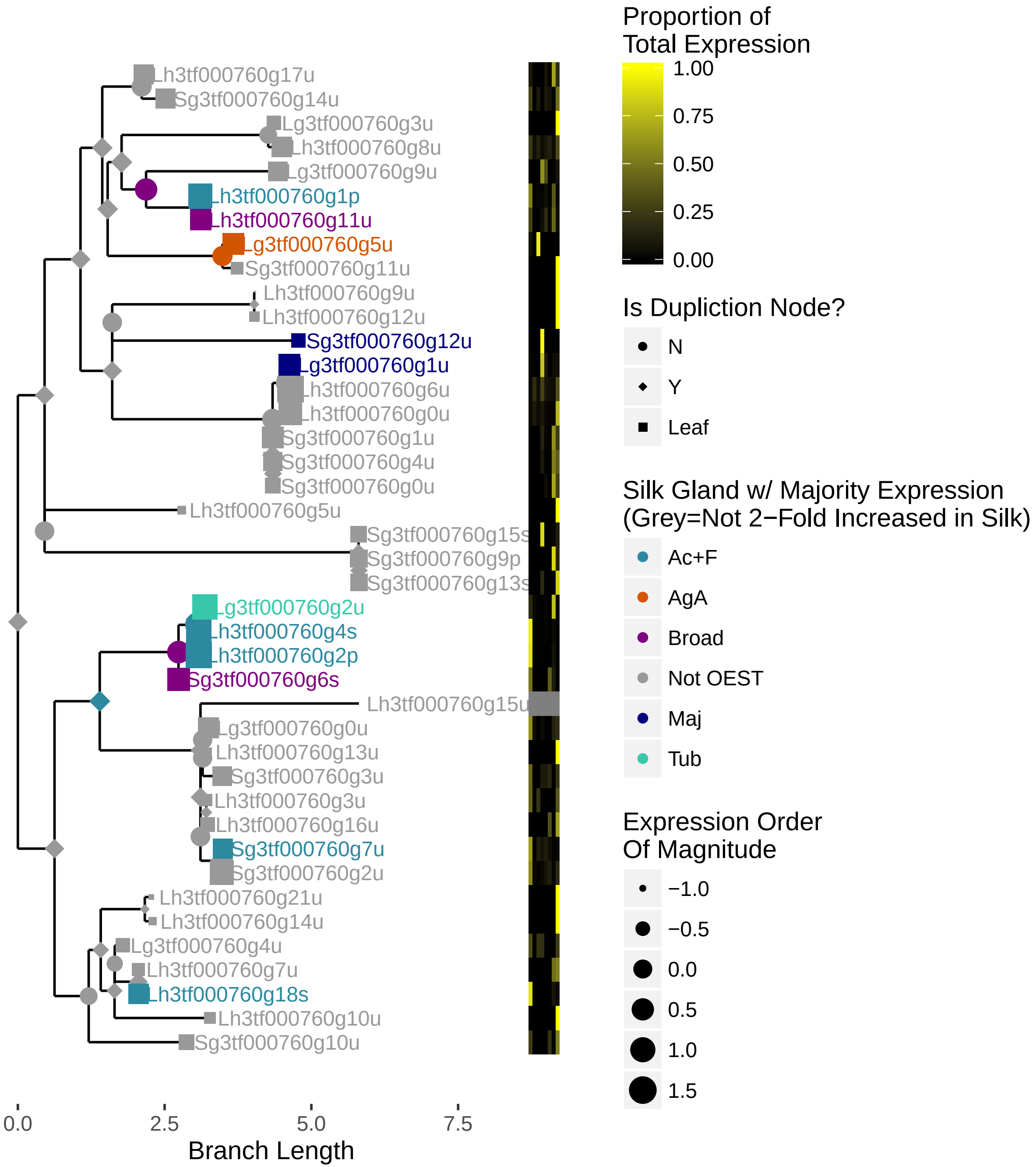

Proportion of Total Expression
1.00
0.75
0.50
0.25
0.00
Is Dupliction Node?
N
Y
Leaf
Silk Gland w/ Majority Expression (Grey=Not 2−Fold Increased in Silk)
Ac+F
AgA
Broad
Not OEST
Maj
Tub
Expression Order Of Magnitude
−1.0
−0.5
0.0
0.5
1.0
1.5
Lh3tf000760g17u
Sg3tf000760g14u
Lg3tf000760g3u
Lh3tf000760g8u
Lg3tf000760g9u
Lh3tf000760g1p
Lh3tf000760g11u
Lg3tf000760g5u
Sg3tf000760g11u
Lh3tf000760g9u
Lh3tf000760g12u
Sg3tf000760g12u
Lg3tf000760g1u
Lh3tf000760g6u
Lh3tf000760g0u
Sg3tf000760g1u
Sg3tf000760g4u
Sg3tf000760g0u
Lh3tf000760g5u
Sg3tf000760g15s
Sg3tf000760g9p
Sg3tf000760g13s
Lg3tf000760g2u
Lh3tf000760g4s
Lh3tf000760g2p
Sg3tf000760g6s
Lh3tf000760g15u
Lg3tf000760g0u
Lh3tf000760g13u
Sg3tf000760g3u
Lh3tf000760g3u
Lh3tf000760g16u
Sg3tf000760g7u
Sg3tf000760g2u
Lh3tf000760g21u
Lh3tf000760g14u
Lg3tf000760g4u
Lh3tf000760g7u
Lh3tf000760g18s
Lh3tf000760g10u
Sg3tf000760g10u
0.0
2.5
5.0
7.5
Branch Length

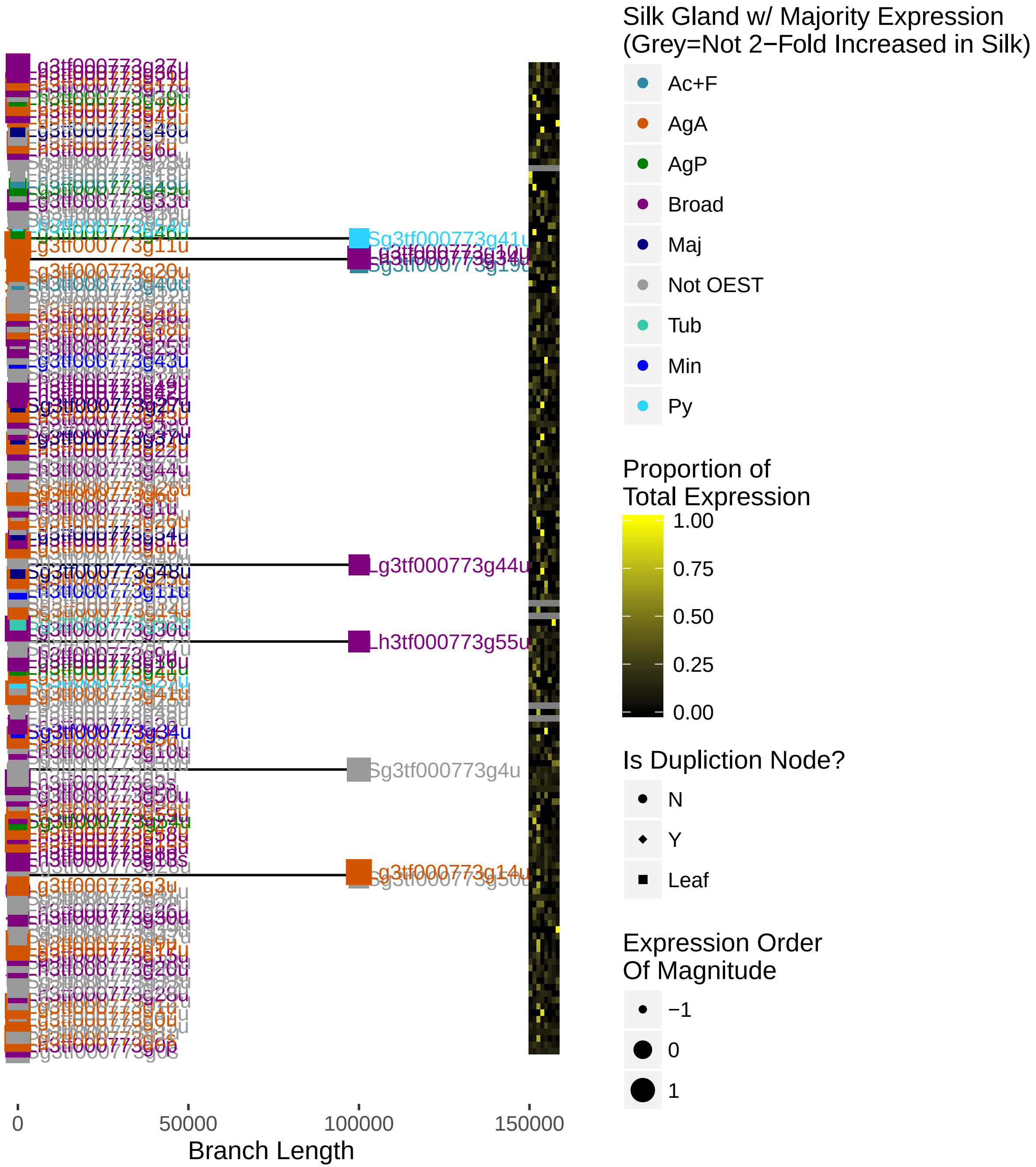
Silk Gland w/ Majority Expression
(Grey=Not 2−Fold Increased in Silk)
Ac+F
AgA
AgP
Broad
Maj
Not OEST
Tub
Min
Py
Proportion of
Total Expression
1.00
0.75
0.50
0.25
0.00
Is Dupliction Node?
N
Y
Leaf
Expression Order
Of Magnitude
−1
0
1
Sg3tf000773g41u
Lg3tf000773g44u
Lh3tf000773g55u
Sg3tf000773g4u
0
50000
100000
150000
Branch Length

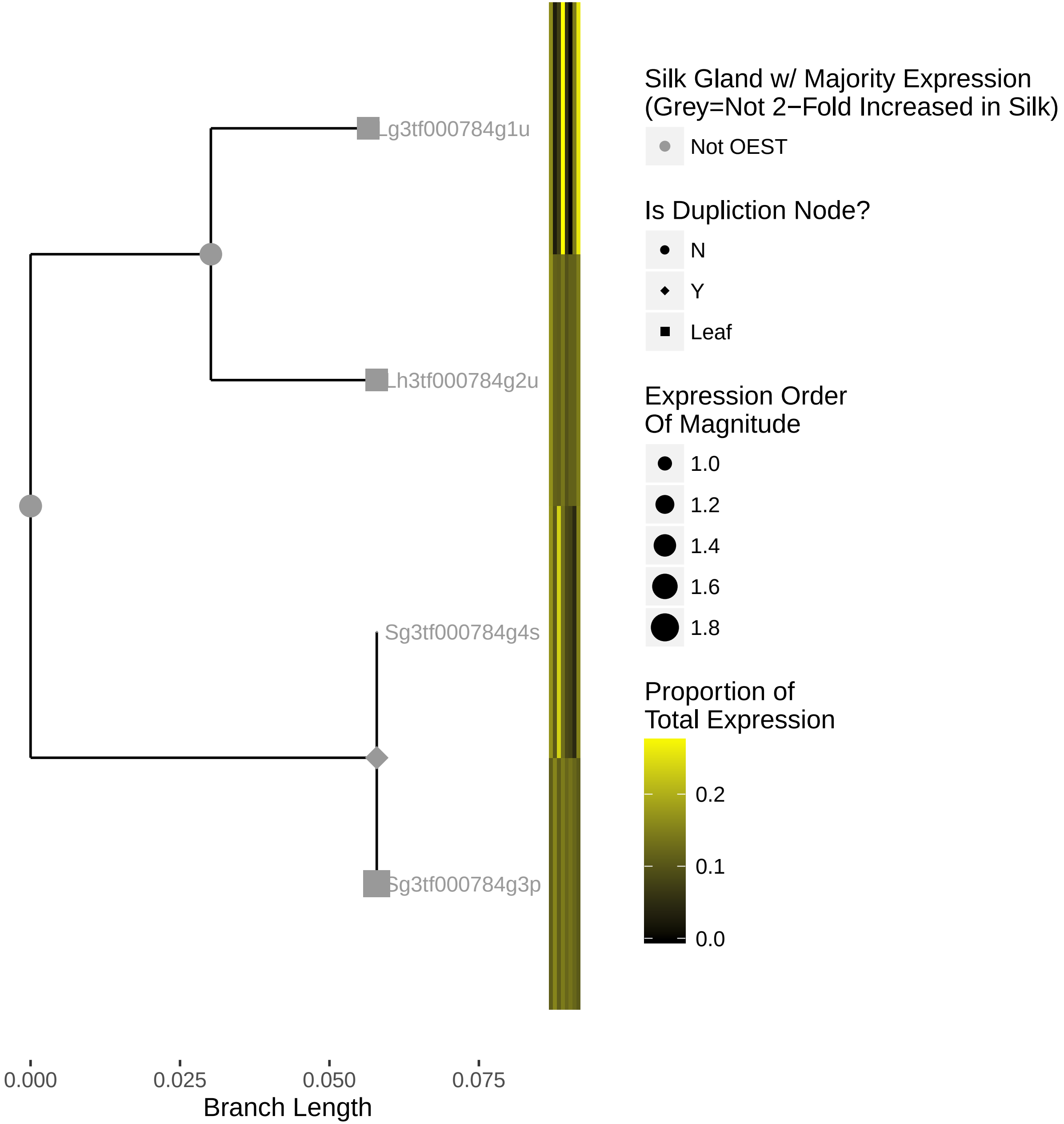

Lg3tf000784g1u
Lh3tf000784g2u
Sg3tf000784g4s
Sg3tf000784g3p
0.000
0.025
0.050
0.075
Branch Length
Silk Gland w/ Majority Expression
(Grey=Not 2−Fold Increased in Silk)
Not OEST
Is Dupliction Node?
N
Y
Leaf
Expression Order
Of Magnitude
1.0
1.2
1.4
1.6
1.8
Proportion of
Total Expression
0.2
0.1
0.0

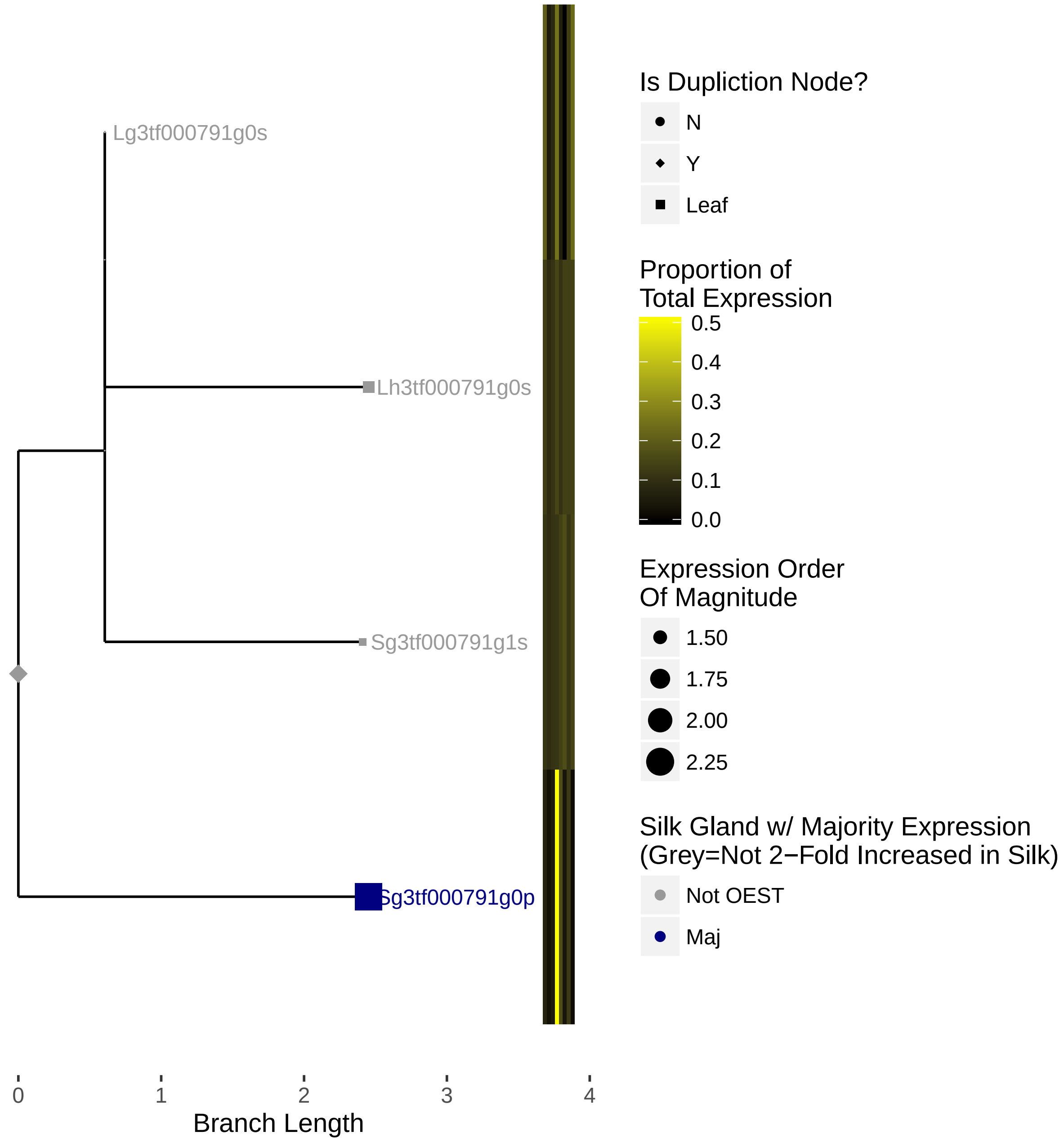

Lg3tf000791g0s
Lh3tf000791g0s
Sg3tf000791g1s
Sg3tf000791g0p
0
1
2
3
4
Branch Length
Is Dupliction Node?
N
Y
Leaf
Proportion of
Total Expression
0.5
0.4
0.3
0.2
0.1
0.0
Expression Order
Of Magnitude
1.50
1.75
2.00
2.25
Silk Gland w/ Majority Expression
(Grey=Not 2−Fold Increased in Silk)
Not OEST
Maj

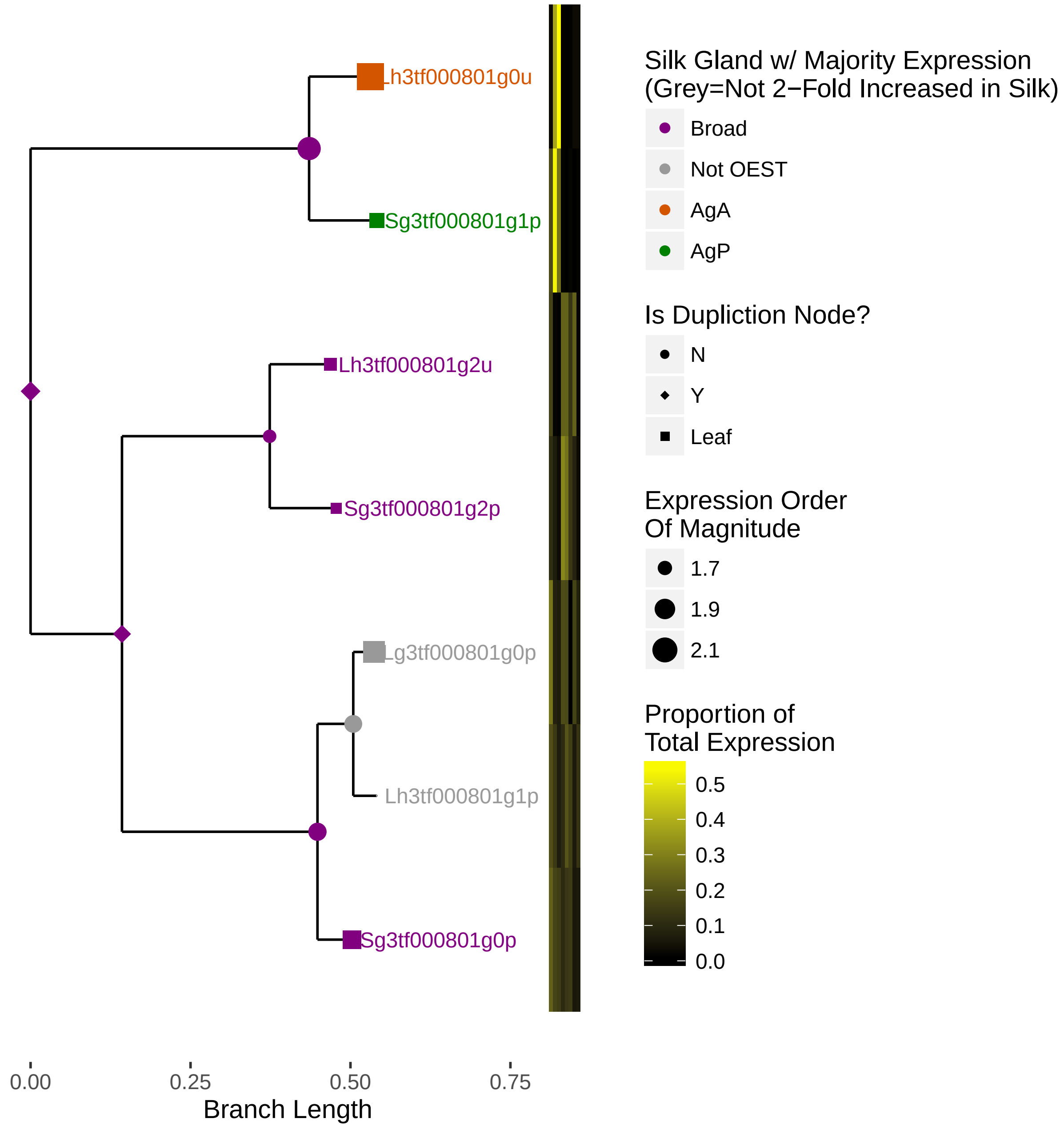

Lh3tf000801g0u
Sg3tf000801g1p
Lh3tf000801g2u
Sg3tf000801g2p
Lg3tf000801g0p
Lh3tf000801g1p
Sg3tf000801g0p
0.00
0.25
0.50
0.75
Branch Length
Silk Gland w/ Majority Expression
(Grey=Not 2−Fold Increased in Silk)
Broad
Not OEST
AgA
AgP
Is Dupliction Node?
N
Y
Leaf
Expression Order
Of Magnitude
1.7
1.9
2.1
Proportion of
Total Expression
0.5
0.4
0.3
0.2
0.1
0.0

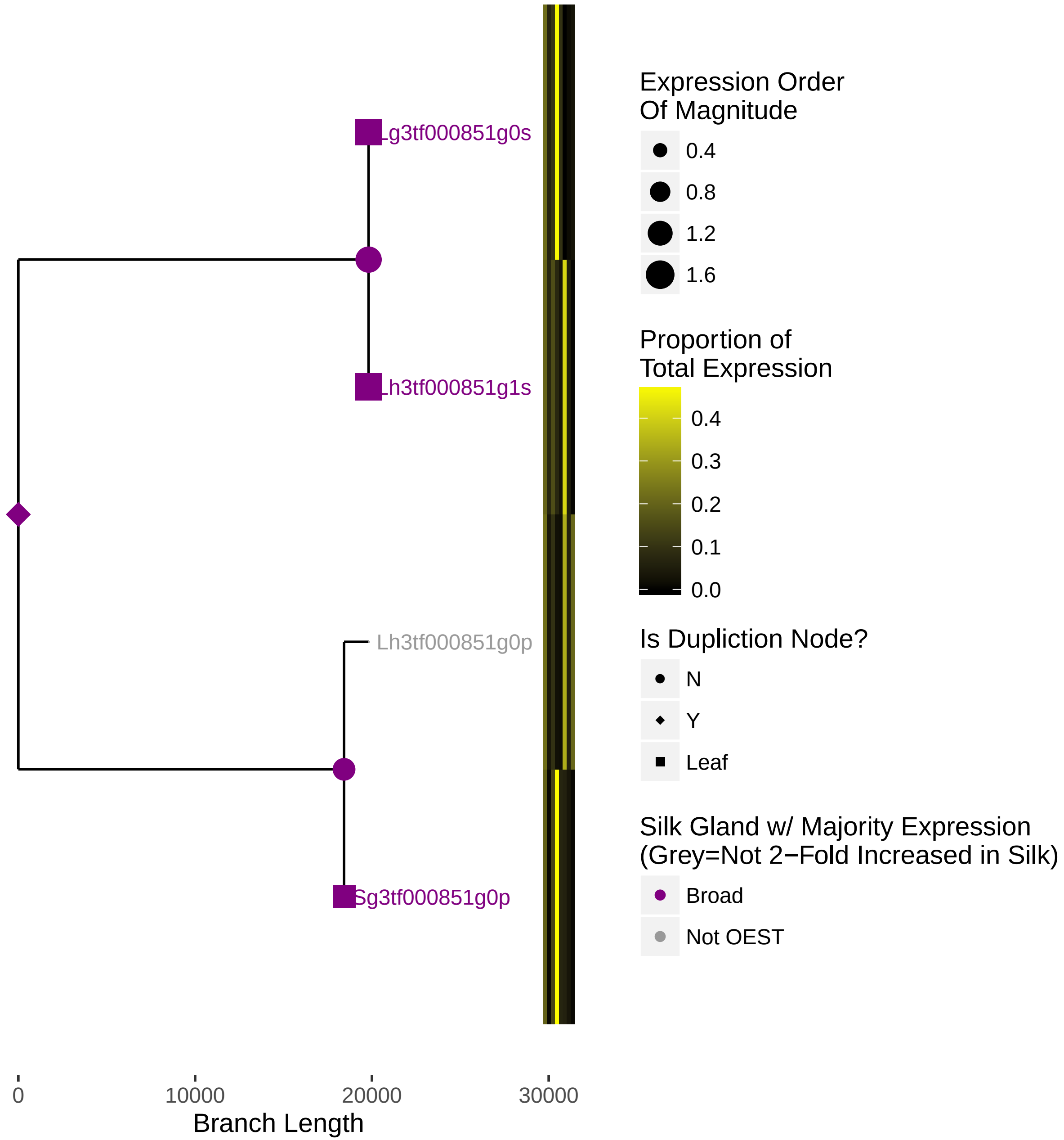

Lg3tf000851g0s
Lh3tf000851g1s
Lh3tf000851g0p
Sg3tf000851g0p
0
10000
20000
30000
Branch Length
Expression Order
Of Magnitude
0.4
0.8
1.2
1.6
Proportion of
Total Expression
0.4
0.3
0.2
0.1
0.0
Is Dupliction Node?
N
Y
Leaf
Silk Gland w/ Majority Expression
(Grey=Not 2−Fold Increased in Silk)
Broad
Not OEST

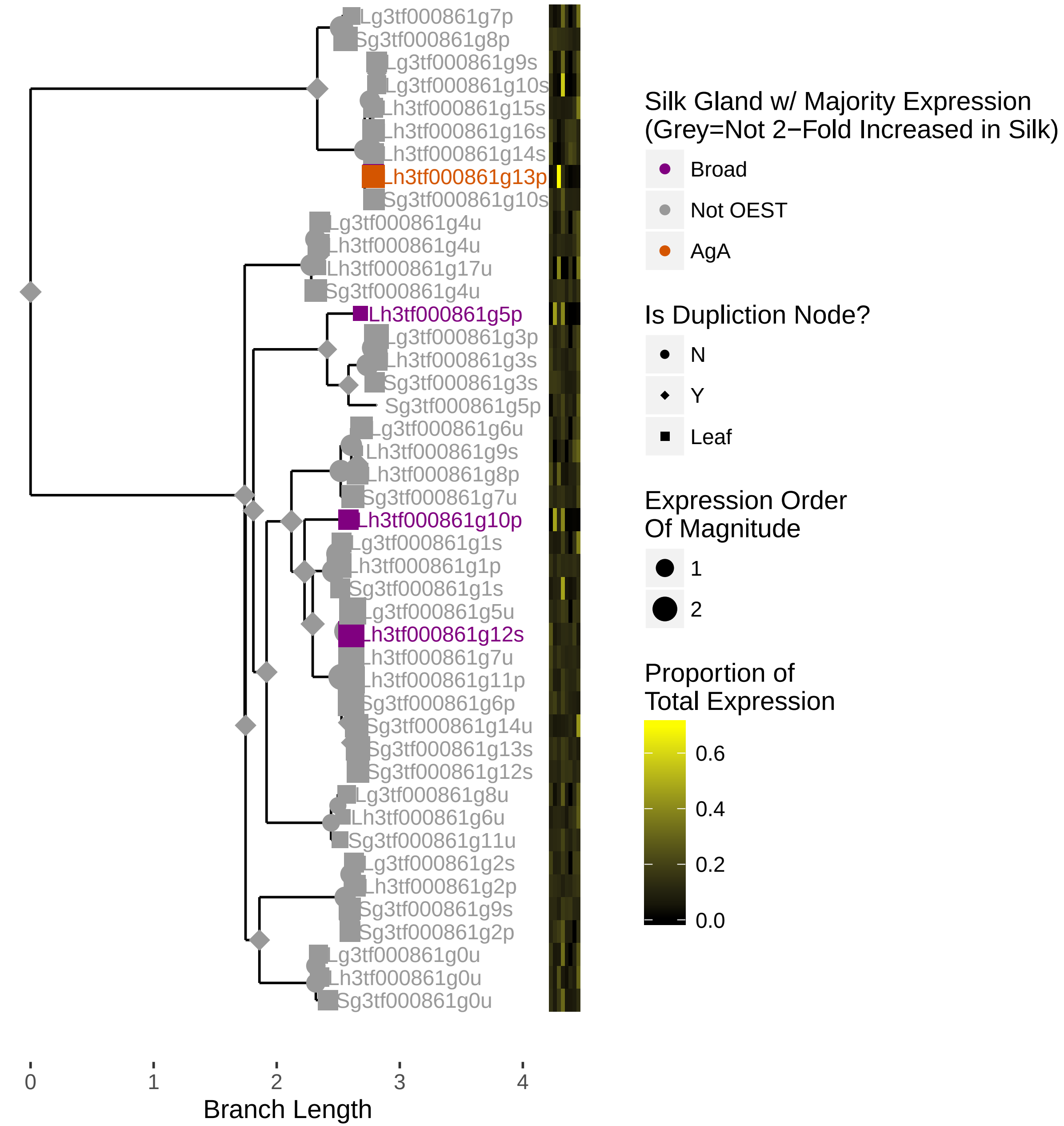

Lg3tf000861g7p
Sg3tf000861g8p
Lg3tf000861g9s
Lg3tf000861g10s
Lh3tf000861g15s
Lh3tf000861g16s
Lh3tf000861g14s
Lh3tf000861g13p
Sg3tf000861g10s
Lg3tf000861g4u
Lh3tf000861g4u
Lh3tf000861g17u
Sg3tf000861g4u
Lh3tf000861g5p
Lg3tf000861g3p
Lh3tf000861g3s
Sg3tf000861g3s
Sg3tf000861g5p
Lg3tf000861g6u
Lh3tf000861g9s
Lh3tf000861g8p
Sg3tf000861g7u
Lh3tf000861g10p
Lg3tf000861g1s
Lh3tf000861g1p
Sg3tf000861g1s
Lg3tf000861g5u
Lh3tf000861g12s
Lh3tf000861g7u
Lh3tf000861g11p
Sg3tf000861g6p
Sg3tf000861g14u
Sg3tf000861g13s
Sg3tf000861g12s
Lg3tf000861g8u
Lh3tf000861g6u
Sg3tf000861g11u
Lg3tf000861g2s
Lh3tf000861g2p
Sg3tf000861g9s
Sg3tf000861g2p
Lg3tf000861g0u
Lh3tf000861g0u
Sg3tf000861g0u
0
1
2
3
4
Branch Length
Silk Gland w/ Majority Expression
(Grey=Not 2−Fold Increased in Silk)
Broad
Not OEST
AgA
Is Dupliction Node?
N
Y
Leaf
Expression Order
Of Magnitude
1
2
Proportion of
Total Expression
0.6
0.4
0.2
0.0

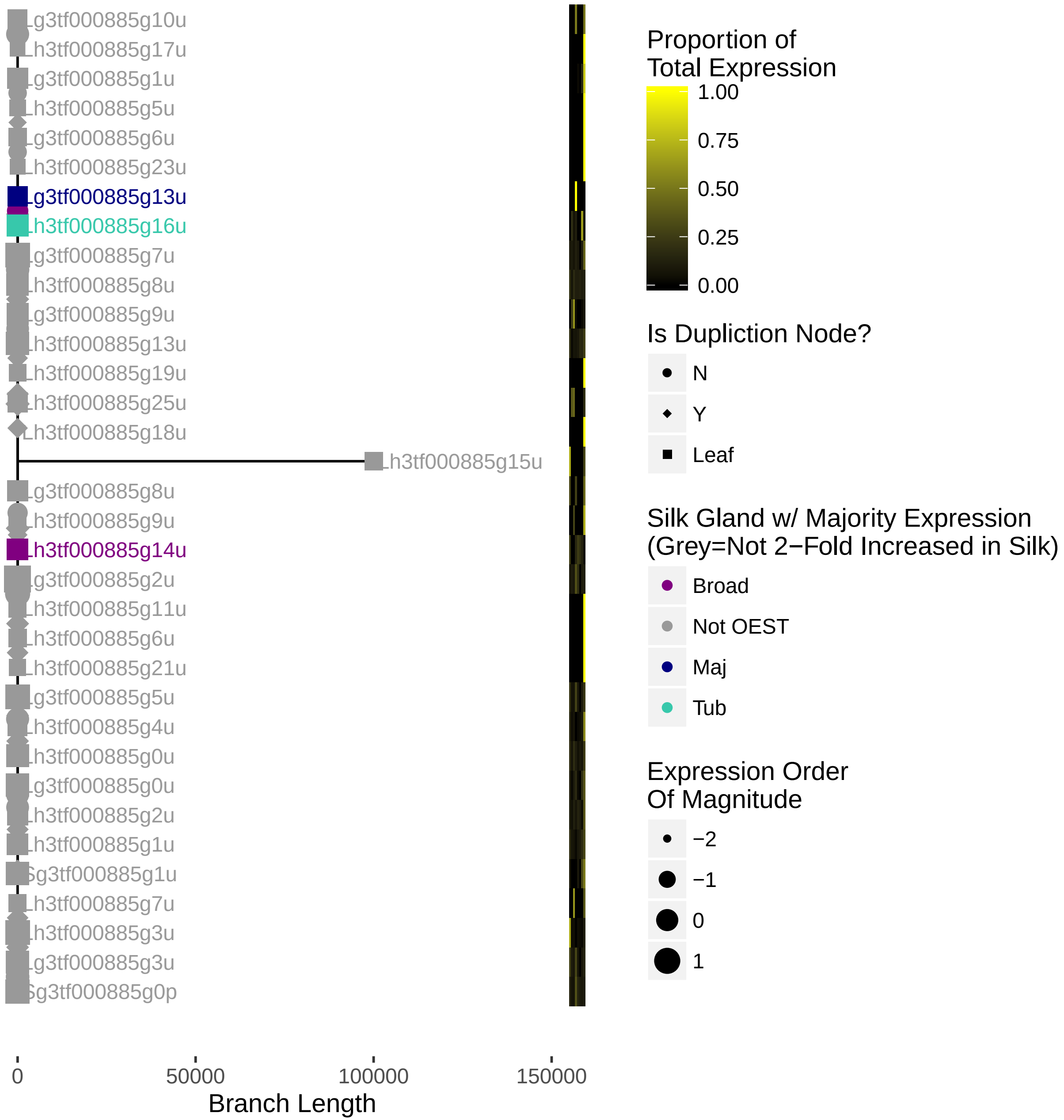

Lg3tf000885g10u
Lh3tf000885g17u
Lg3tf000885g1u
Lh3tf000885g5u
Lg3tf000885g6u
Lh3tf000885g23u
Lg3tf000885g13u
Lh3tf000885g16u
Lg3tf000885g7u
Lh3tf000885g8u
Lg3tf000885g9u
Lh3tf000885g13u
Lh3tf000885g19u
Lh3tf000885g25u
Lh3tf000885g18u
Lh3tf000885g15u
Lg3tf000885g8u
Lh3tf000885g9u
Lh3tf000885g14u
Lg3tf000885g2u
Lh3tf000885g11u
Lh3tf000885g6u
Lh3tf000885g21u
Lg3tf000885g5u
Lh3tf000885g4u
Lh3tf000885g0u
Lg3tf000885g0u
Lh3tf000885g2u
Lh3tf000885g1u
Sg3tf000885g1u
Lh3tf000885g7u
Lh3tf000885g3u
Lg3tf000885g3u
Sg3tf000885g0p
0
50000
100000
150000
Branch Length
Proportion of Total Expression
1.00
0.75
0.50
0.25
0.00
Is Dupliction Node?
N
Y
Leaf
Silk Gland w/ Majority Expression (Grey=Not 2−Fold Increased in Silk)
Broad
Not OEST
Maj
Tub
Expression Order Of Magnitude
−2
−1
0
1

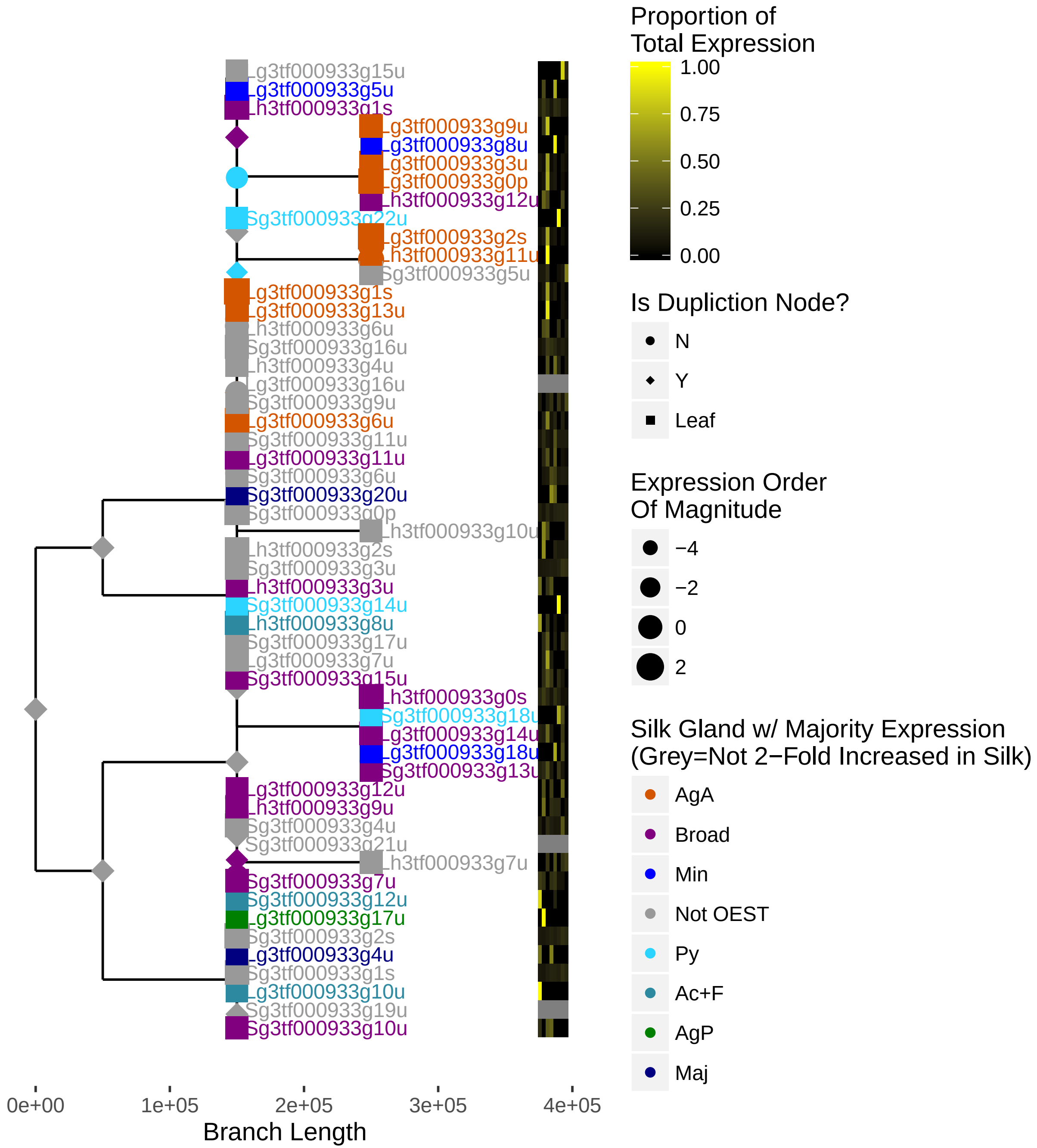

Proportion of Total Expression
1.00
0.75
0.50
0.25
0.00
Is Duplicion Node?
N
Y
Leaf
Expression Order Of Magnitude
−4
−2
0
2
Silk Gland w/ Majority Expression (Grey=Not 2−Fold Increased in Silk)
AgA
Broad
Min
Not OEST
Py
Ac+F
AgP
Maj
g3tf000933g15u
g3tf000933g5u
h3tf000933g1s
g3tf000933g9u
g3tf000933g8u
g3tf000933g3u
g3tf000933g0p
h3tf000933g12u
Sg3tf000933g22u
g3tf000933g2s
h3tf000933g11u
Sg3tf000933g5u
g3tf000933g1s
g3tf000933g13u
h3tf000933g6u
Sg3tf000933g16u
h3tf000933g4u
g3tf000933g16u
Sg3tf000933g9u
g3tf000933g6u
Sg3tf000933g11u
g3tf000933g11u
Sg3tf000933g6u
Sg3tf000933g20u
Sg3tf000933g0p
h3tf000933g10u
h3tf000933g2s
Sg3tf000933g3u
h3tf000933g3u
Sg3tf000933g14u
h3tf000933g8u
Sg3tf000933g17u
g3tf000933g7u
Sg3tf000933g15u
h3tf000933g0s
Sg3tf000933g18u
g3tf000933g14u
g3tf000933g18u
Sg3tf000933g13u
g3tf000933g12u
h3tf000933g9u
Sg3tf000933g4u
Sg3tf000933g21u
h3tf000933g7u
Sg3tf000933g7u
Sg3tf000933g12u
g3tf000933g17u
Sg3tf000933g2s
g3tf000933g4u
Sg3tf000933g1s
g3tf000933g10u
Sg3tf000933g19u
Sg3tf000933g10u
0e+00
1e+05
2e+05
3e+05
4e+05
Branch Length

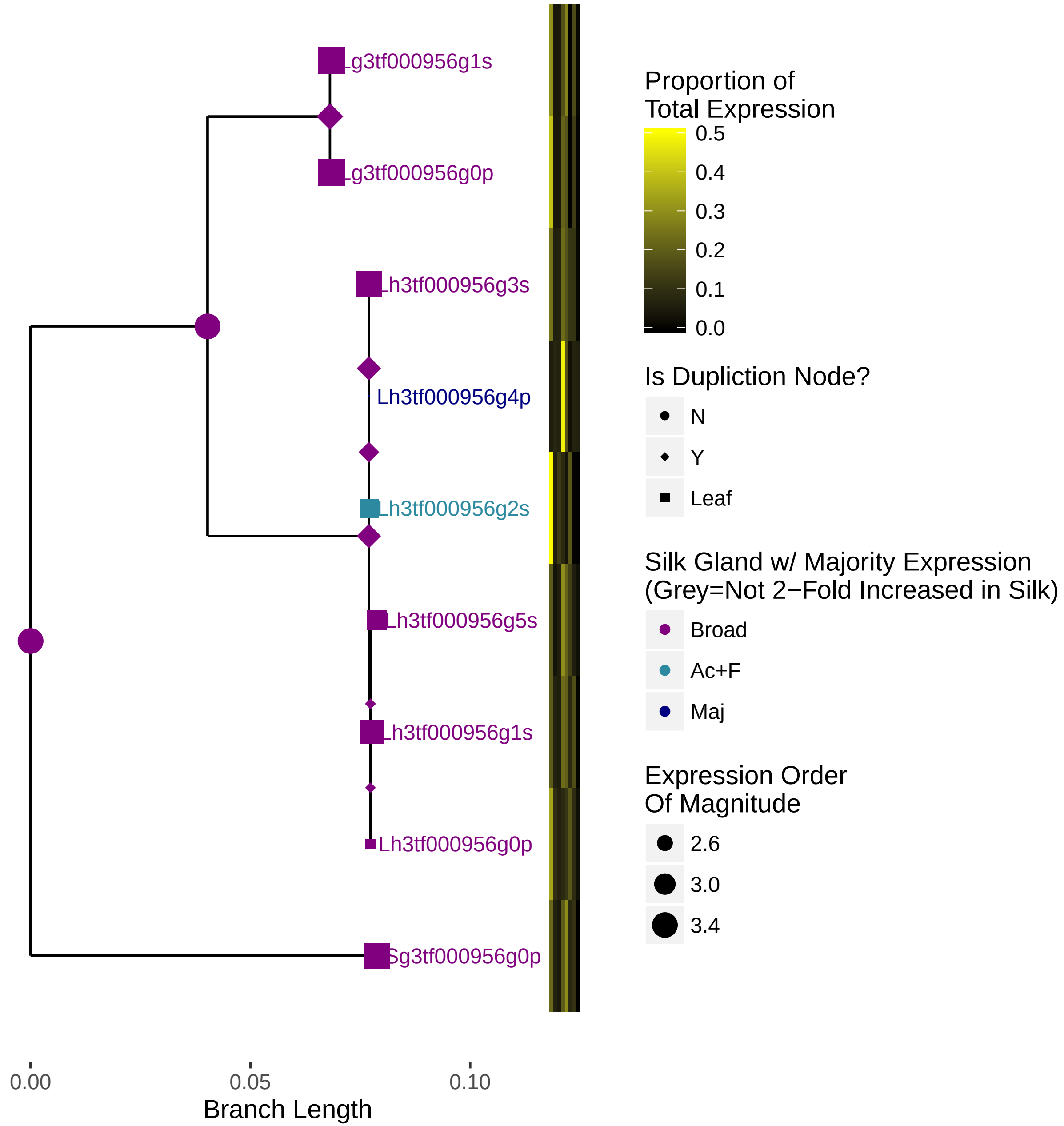
Lg3tf000956g1s
Lg3tf000956g0p
Lh3tf000956g3s
Lh3tf000956g4p
Lh3tf000956g2s
Lh3tf000956g5s
Lh3tf000956g1s
Lh3tf000956g0p
Sg3tf000956g0p
0.00
0.05
0.10
Branch Length
Proportion of
Total Expression
0.5
0.4
0.3
0.2
0.1
0.0
Is Dupliction Node?
N
Y
Leaf
Silk Gland w/ Majority Expression
(Grey=Not 2−Fold Increased in Silk)
Broad
Ac+F
Maj
Expression Order
Of Magnitude
2.6
3.0
3.4

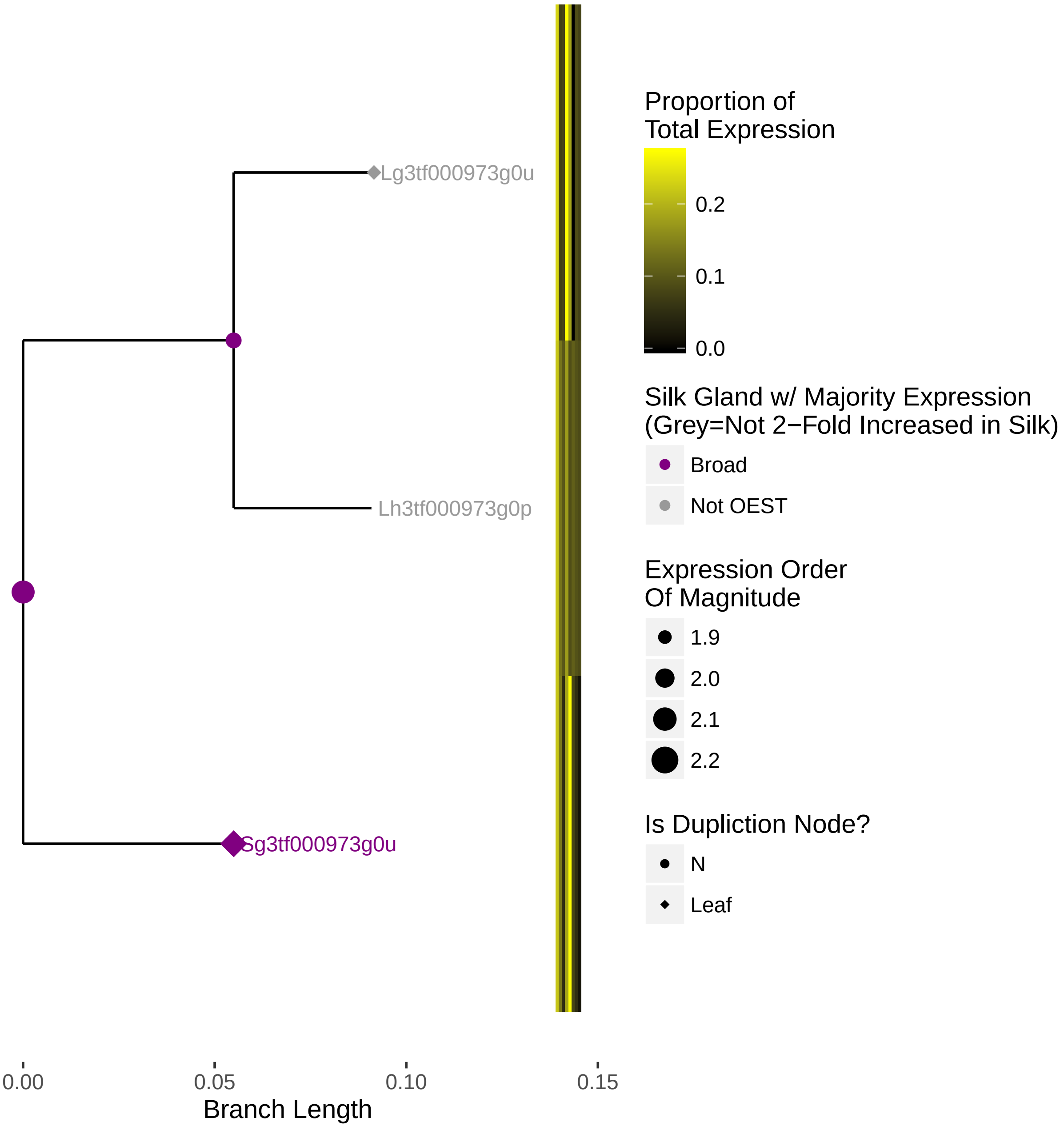

Lg3tf000973g0u
Lh3tf000973g0p
Sg3tf000973g0u
0.00
0.05
0.10
0.15
Branch Length
Proportion of
Total Expression
0.2
0.1
0.0
Silk Gland w/ Majority Expression
(Grey=Not 2−Fold Increased in Silk)
Broad
Not OEST
Expression Order
Of Magnitude
1.9
2.0
2.1
2.2
Is Dupliction Node?
N
Leaf

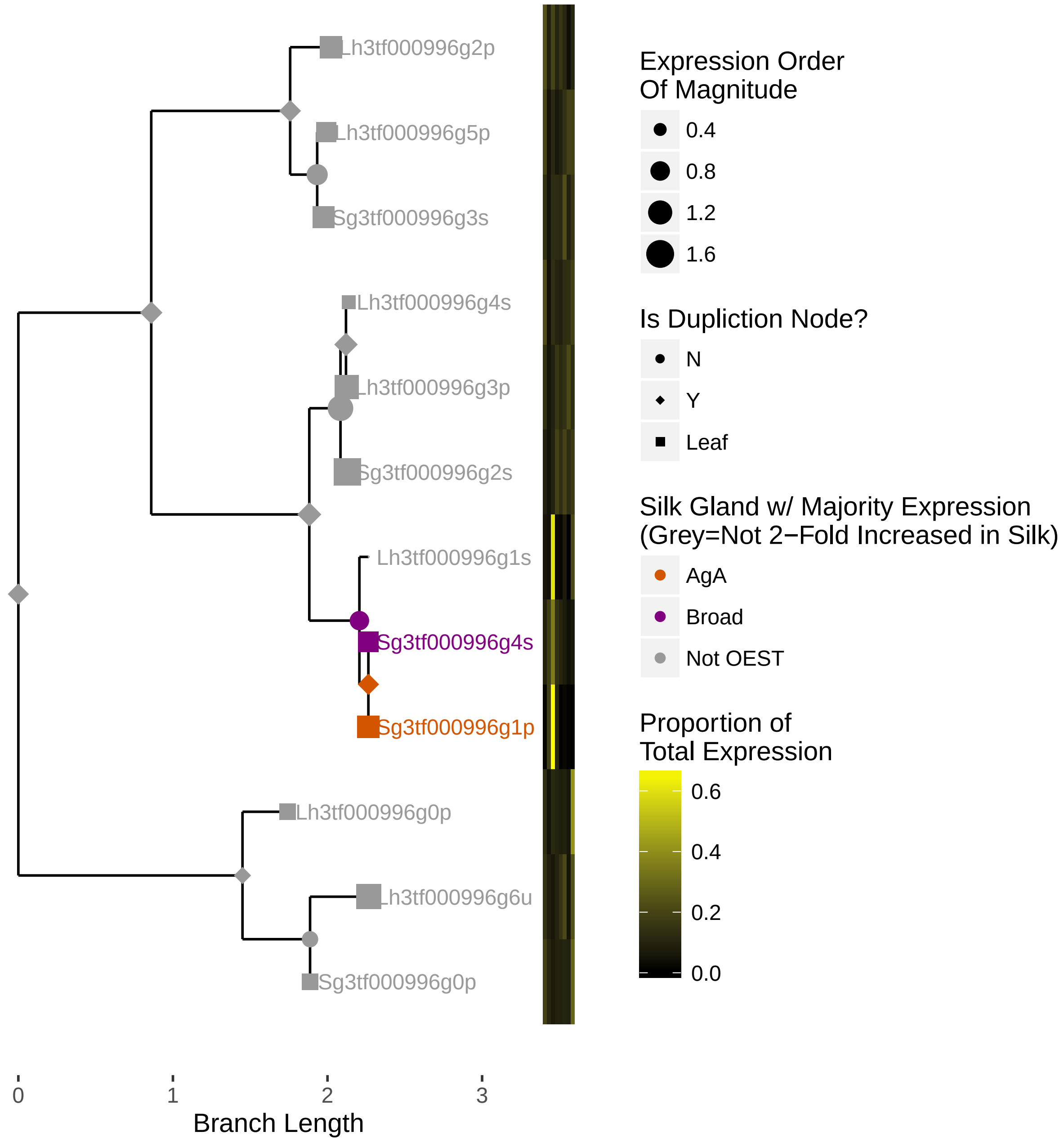

Lh3tf000996g2p
Lh3tf000996g5p
Sg3tf000996g3s
Lh3tf000996g4s
Lh3tf000996g3p
Sg3tf000996g2s
Lh3tf000996g1s
Sg3tf000996g4s
Sg3tf000996g1p
Lh3tf000996g0p
Lh3tf000996g6u
Sg3tf000996g0p
0
1
2
3
Branch Length
Expression Order Of Magnitude
0.4
0.8
1.2
1.6
Is Dupliction Node?
N
Y
Leaf
Silk Gland w/ Majority Expression (Grey=Not 2−Fold Increased in Silk)
AgA
Broad
Not OEST
Proportion of Total Expression
0.6
0.4
0.2
0.0

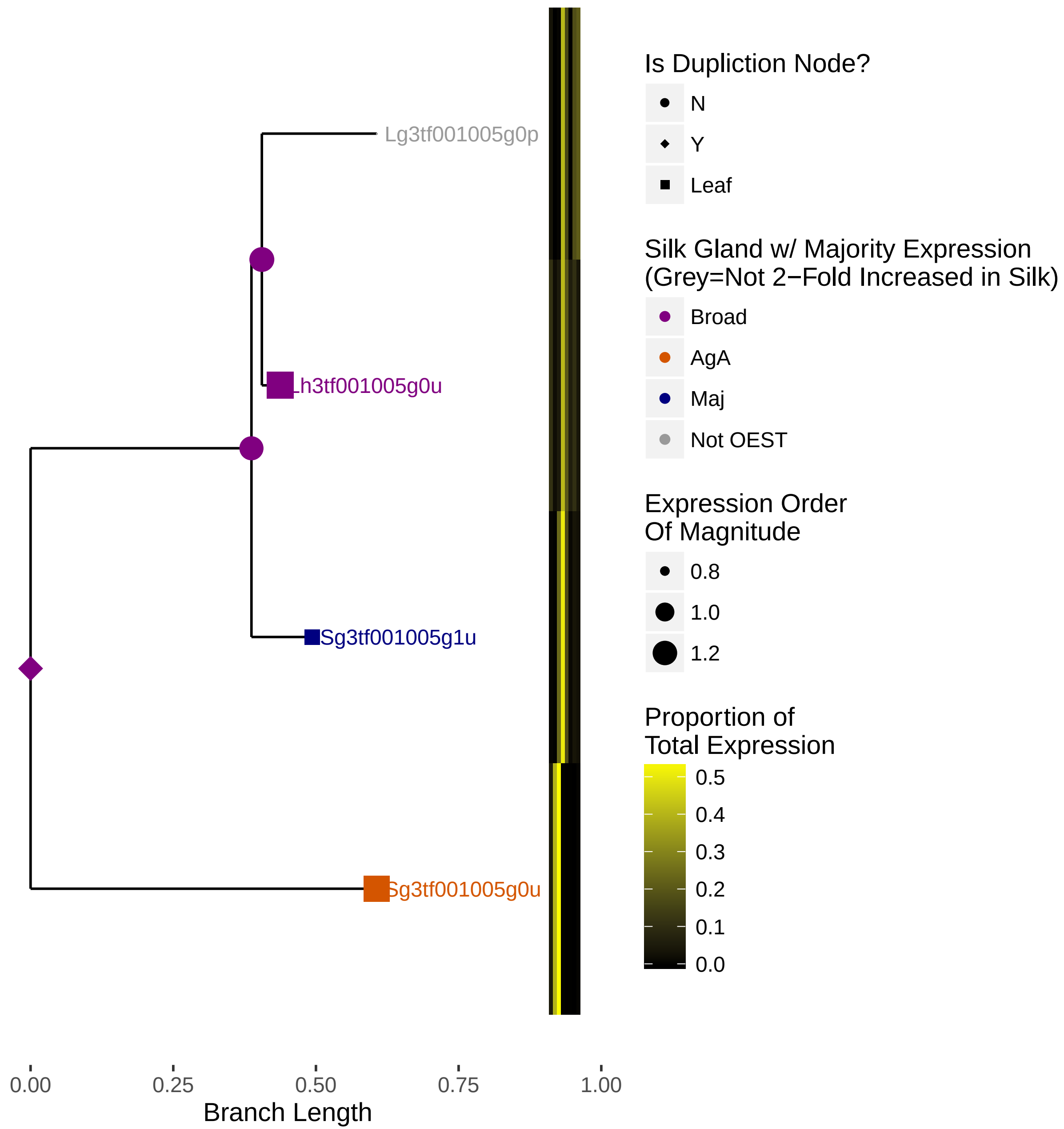

Lg3tf001005g0p
Lh3tf001005g0u
Sg3tf001005g1u
Sg3tf001005g0u
Is Dupliction Node?
N
Y
Leaf
Silk Gland w/ Majority Expression
(Grey=Not 2−Fold Increased in Silk)
Broad
AgA
Maj
Not OEST
Expression Order
Of Magnitude
0.8
1.0
1.2
Proportion of
Total Expression
0.5
0.4
0.3
0.2
0.1
0.0
0.00
0.25
0.50
0.75
1.00
Branch Length

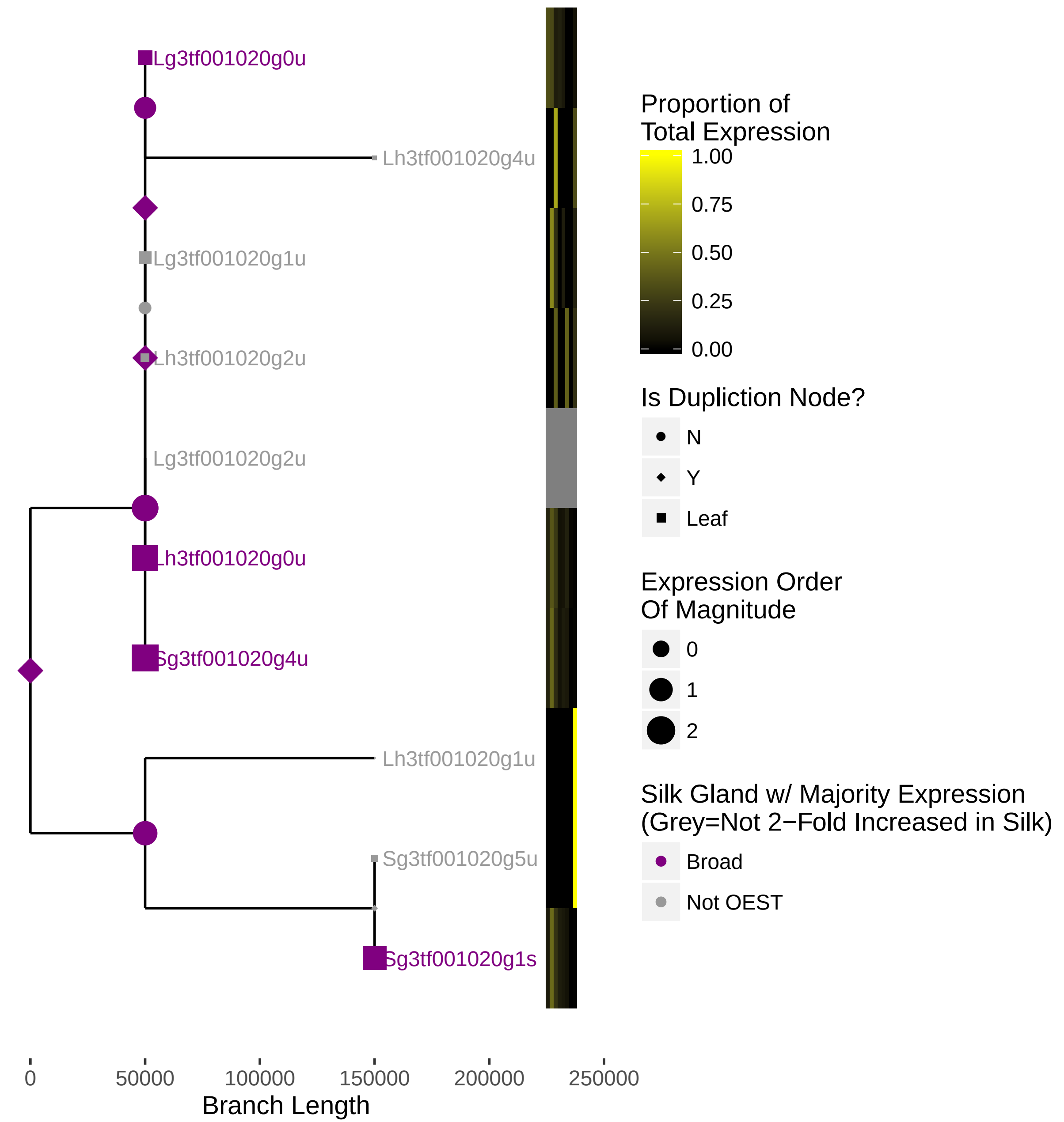

Lg3tf001020g0u
Lh3tf001020g4u
Lg3tf001020g1u
Lh3tf001020g2u
Lg3tf001020g2u
Lh3tf001020g0u
Sg3tf001020g4u
Lh3tf001020g1u
Sg3tf001020g5u
Sg3tf001020g1s
0
50000
100000
150000
200000
250000
Branch Length
Proportion of
Total Expression
1.00
0.75
0.50
0.25
0.00
Is Dupliction Node?
N
Y
Leaf
Expression Order
Of Magnitude
0
1
2
Silk Gland w/ Majority Expression
(Grey=Not 2−Fold Increased in Silk)
Broad
Not OEST

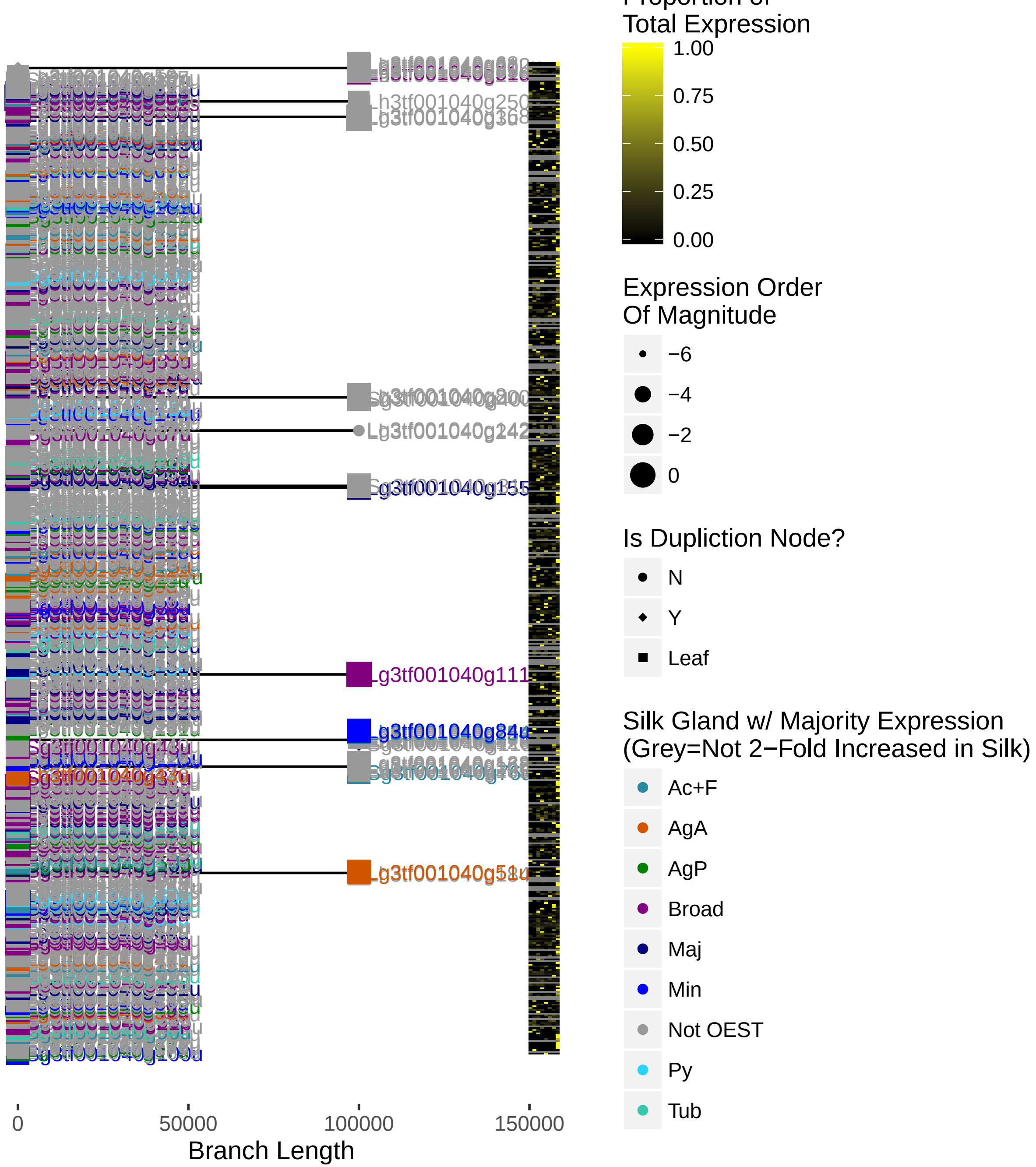

Total Expression
1.00
0.75
0.50
0.25
0.00
Expression Order
Of Magnitude
−6
−4
−2
0
Is Dupliction Node?
N
Y
Leaf
Silk Gland w/ Majority Expression
(Grey=Not 2−Fold Increased in Silk)
Ac+F
AgA
AgP
Broad
Maj
Min
Not OEST
Py
Tub
h3tf001040g250
Lg3tf001040g111
0
50000
100000
150000
Branch Length

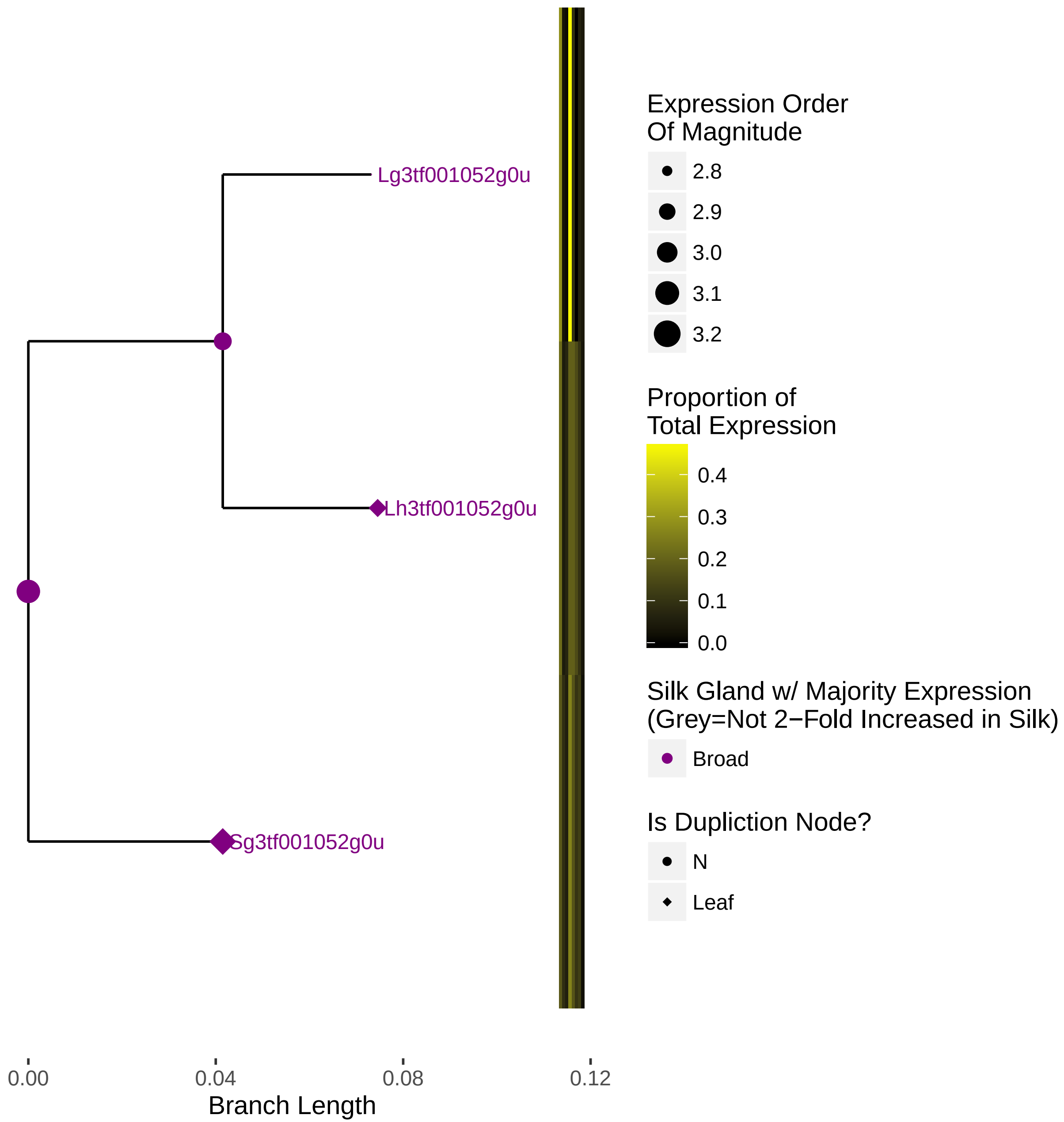

Lg3tf001052g0u
Lh3tf001052g0u
Sg3tf001052g0u
Expression Order
Of Magnitude
2.8
2.9
3.0
3.1
3.2
Proportion of
Total Expression
0.4
0.3
0.2
0.1
0.0
Silk Gland w/ Majority Expression
(Grey=Not 2−Fold Increased in Silk)
Broad
Is Dupliction Node?
N
Leaf
0.00
0.04
0.08
0.12
Branch Length

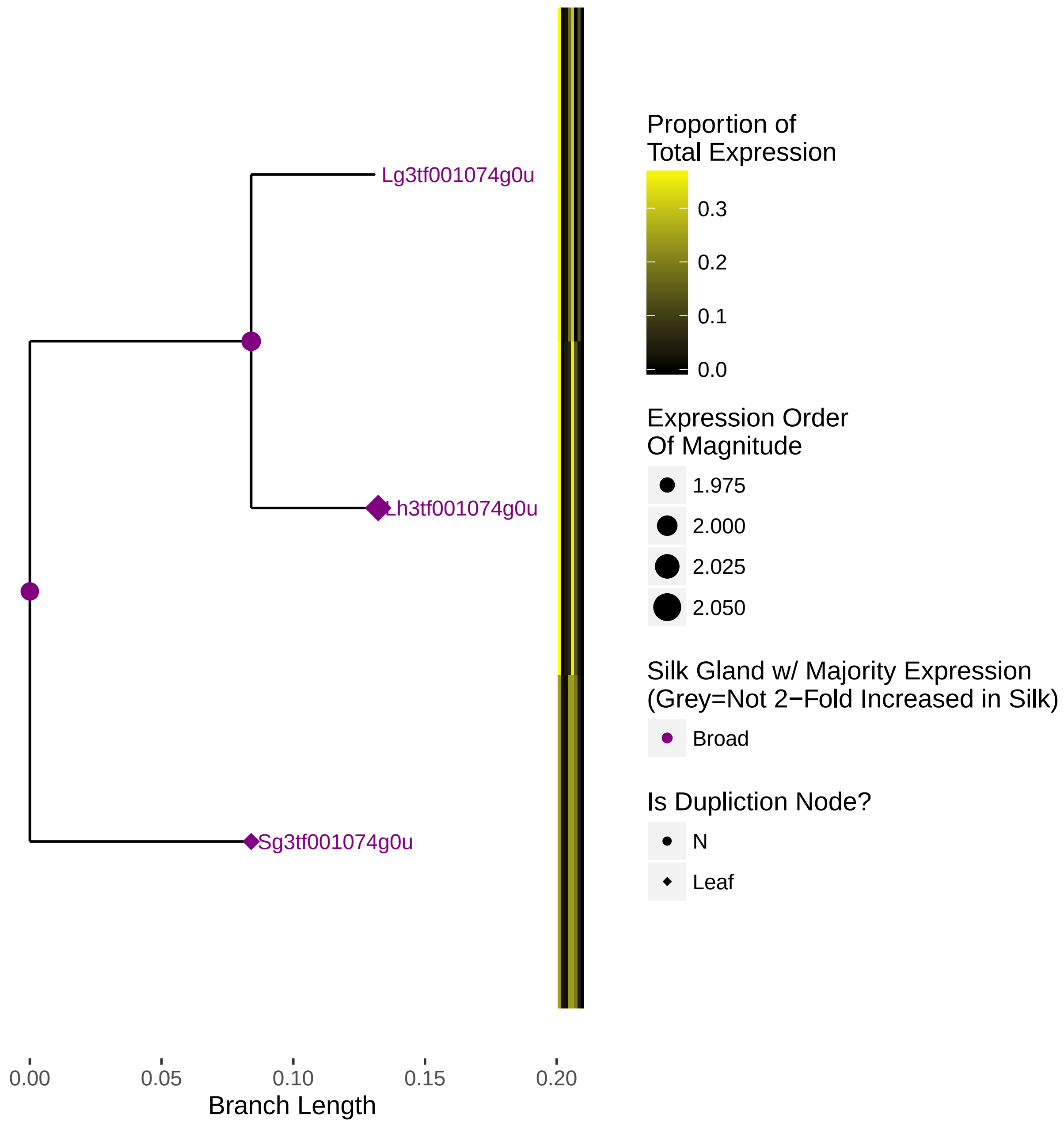

Lg3tf001074g0u
Lh3tf001074g0u
Sg3tf001074g0u
0.00
0.05
0.10
0.15
0.20
Branch Length
Proportion of Total Expression
0.3
0.2
0.1
0.0
Expression Order Of Magnitude
1.975
2.000
2.025
2.050
Silk Gland w/ Majority Expression (Grey=Not 2−Fold Increased in Silk)
Broad
Is Dupliction Node?
N
Leaf

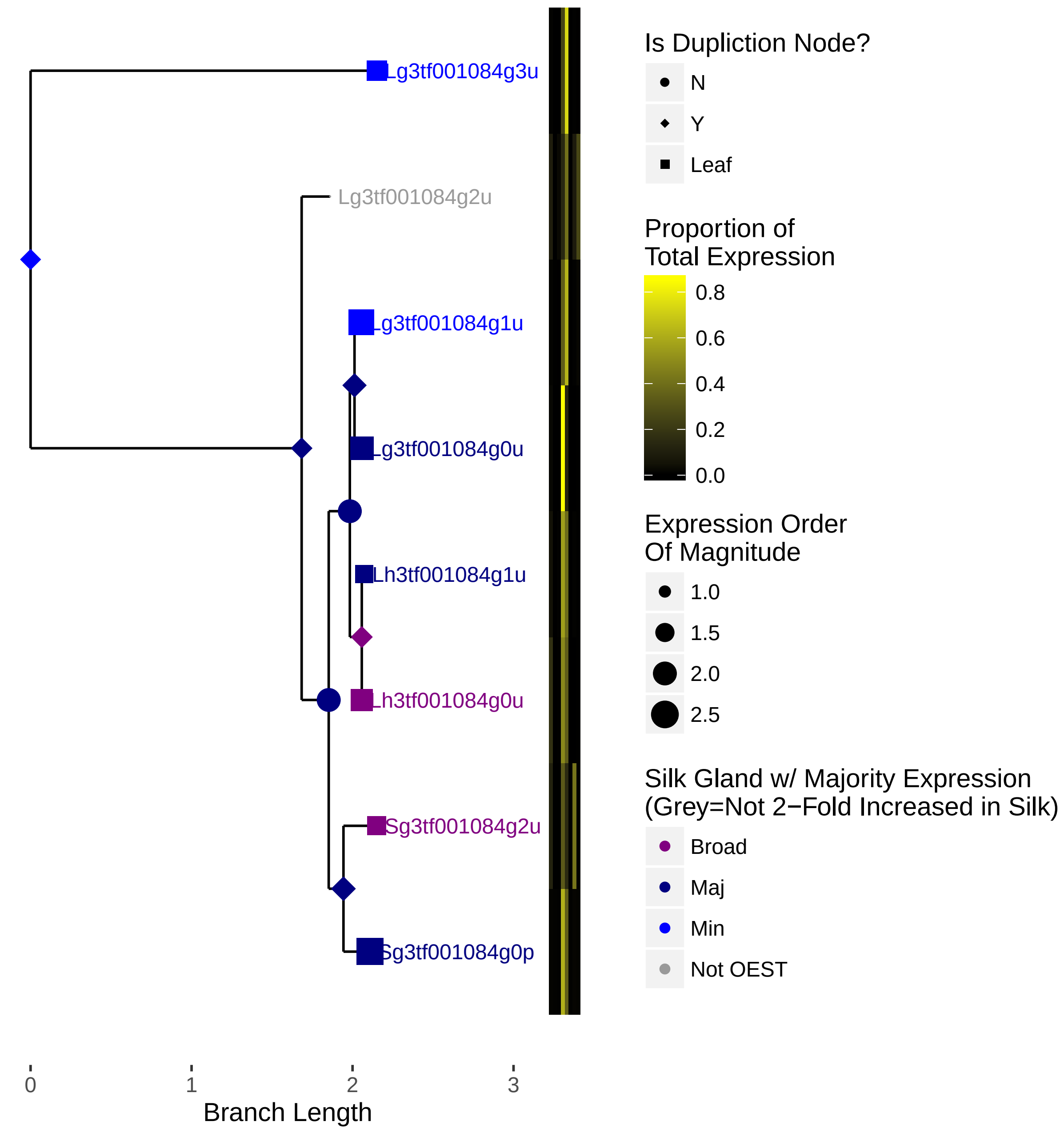
Lg3tf001084g3u
Lg3tf001084g2u
Lg3tf001084g1u
Lg3tf001084g0u
Lh3tf001084g1u
Lh3tf001084g0u
Sg3tf001084g2u
Sg3tf001084g0p
0
1
2
3
Branch Length
Is Dupliction Node?
N
Y
Leaf
Proportion of
Total Expression
0.8
0.6
0.4
0.2
0.0
Expression Order
Of Magnitude
1.0
1.5
2.0
2.5
Silk Gland w/ Majority Expression
(Grey=Not 2−Fold Increased in Silk)
Broad
Maj
Min
Not OEST

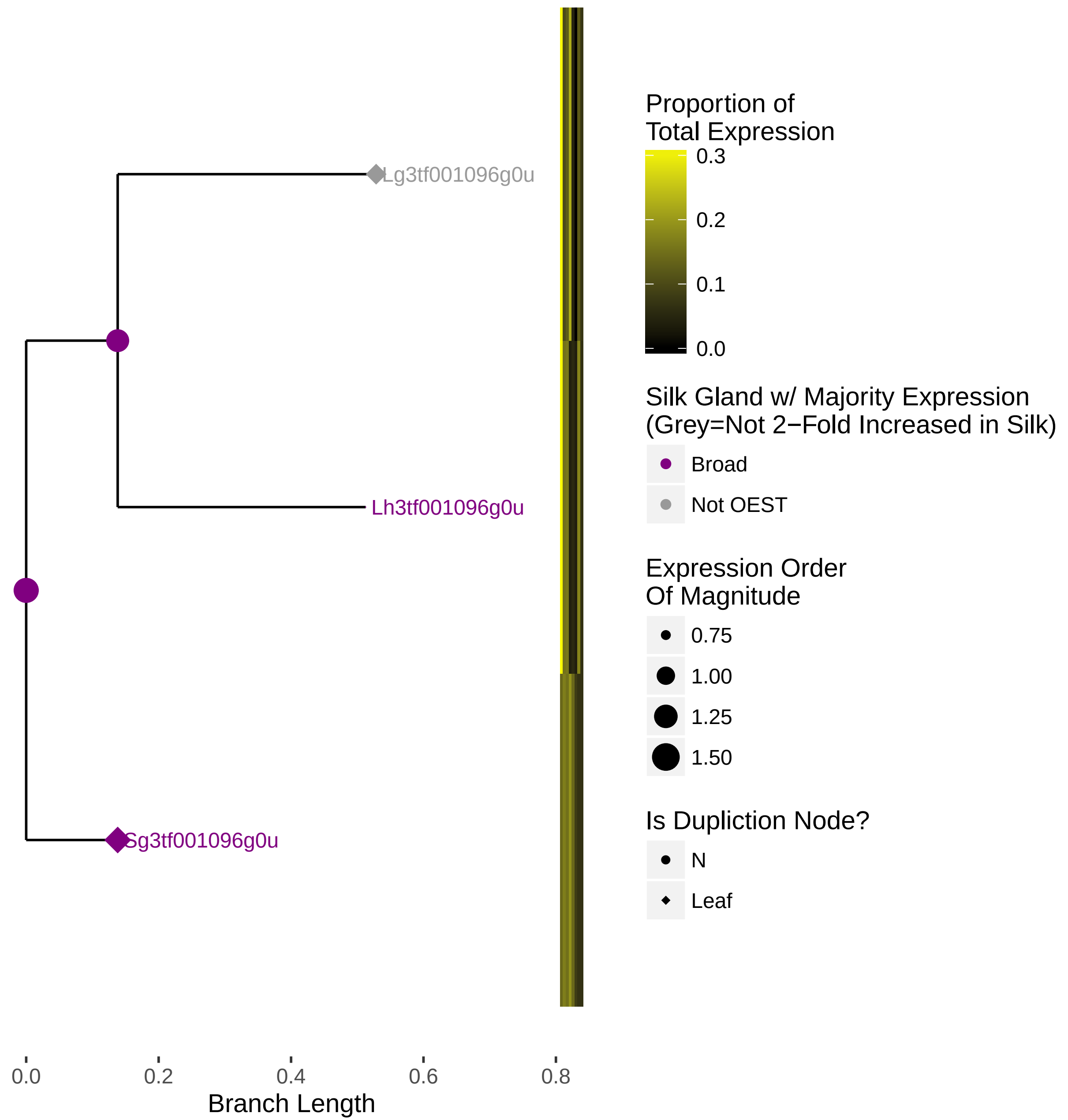

Lg3tf001096g0u
Lh3tf001096g0u
Sg3tf001096g0u
0.0
0.2
0.4
0.6
0.8
Branch Length
Proportion of
Total Expression
0.3
0.2
0.1
0.0
Silk Gland w/ Majority Expression
(Grey=Not 2−Fold Increased in Silk)
Broad
Not OEST
Expression Order
Of Magnitude
0.75
1.00
1.25
1.50
Is Dupliction Node?
N
Leaf

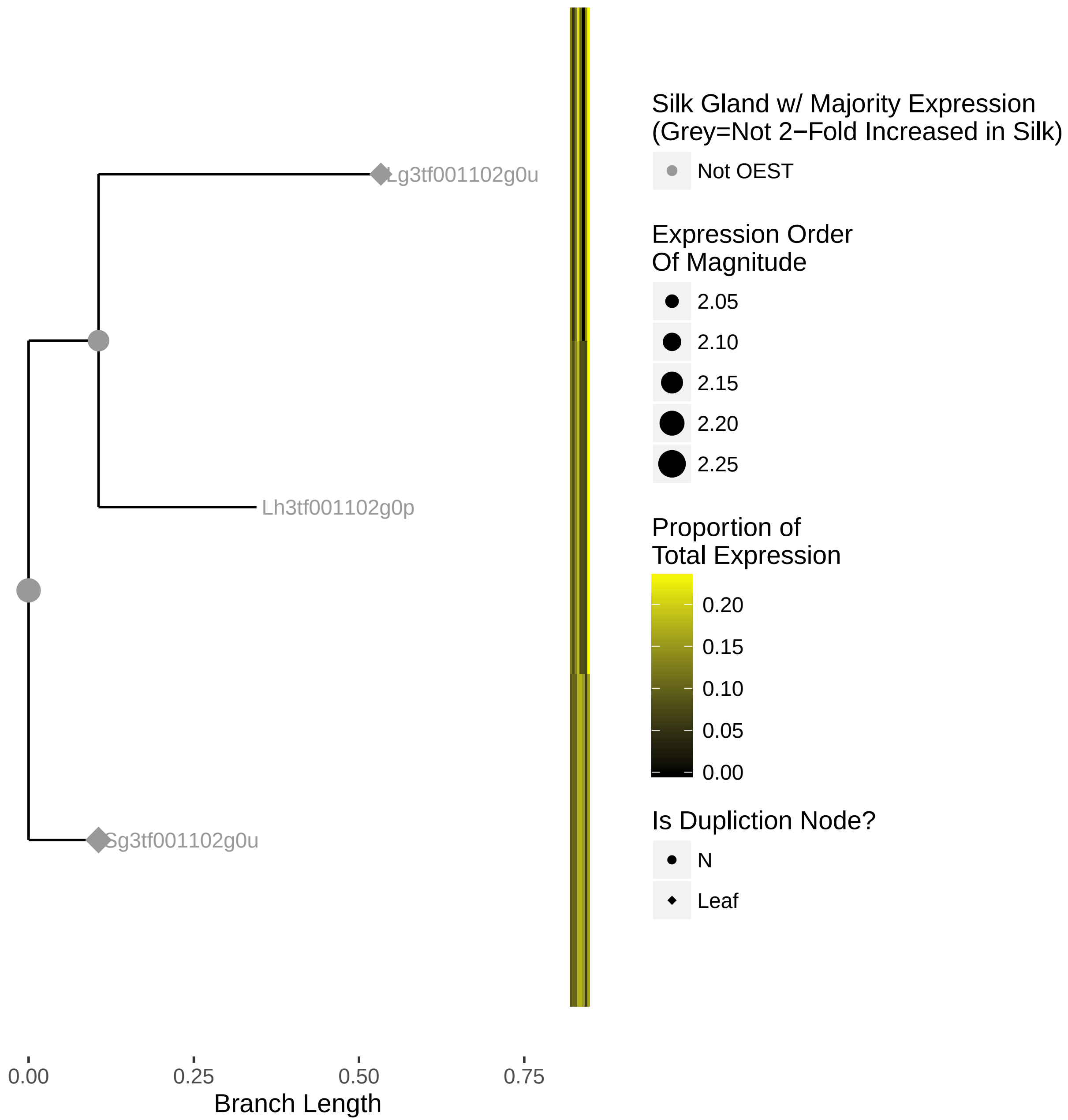
Lg3tf001102g0u
Lh3tf001102g0p
Sg3tf001102g0u
0.00
0.25
0.50
0.75
Branch Length
Silk Gland w/ Majority Expression
(Grey=Not 2−Fold Increased in Silk)
Not OEST
Expression Order
Of Magnitude
2.05
2.10
2.15
2.20
2.25
Proportion of
Total Expression
0.20
0.15
0.10
0.05
0.00
Is Dupliction Node?
N
Leaf

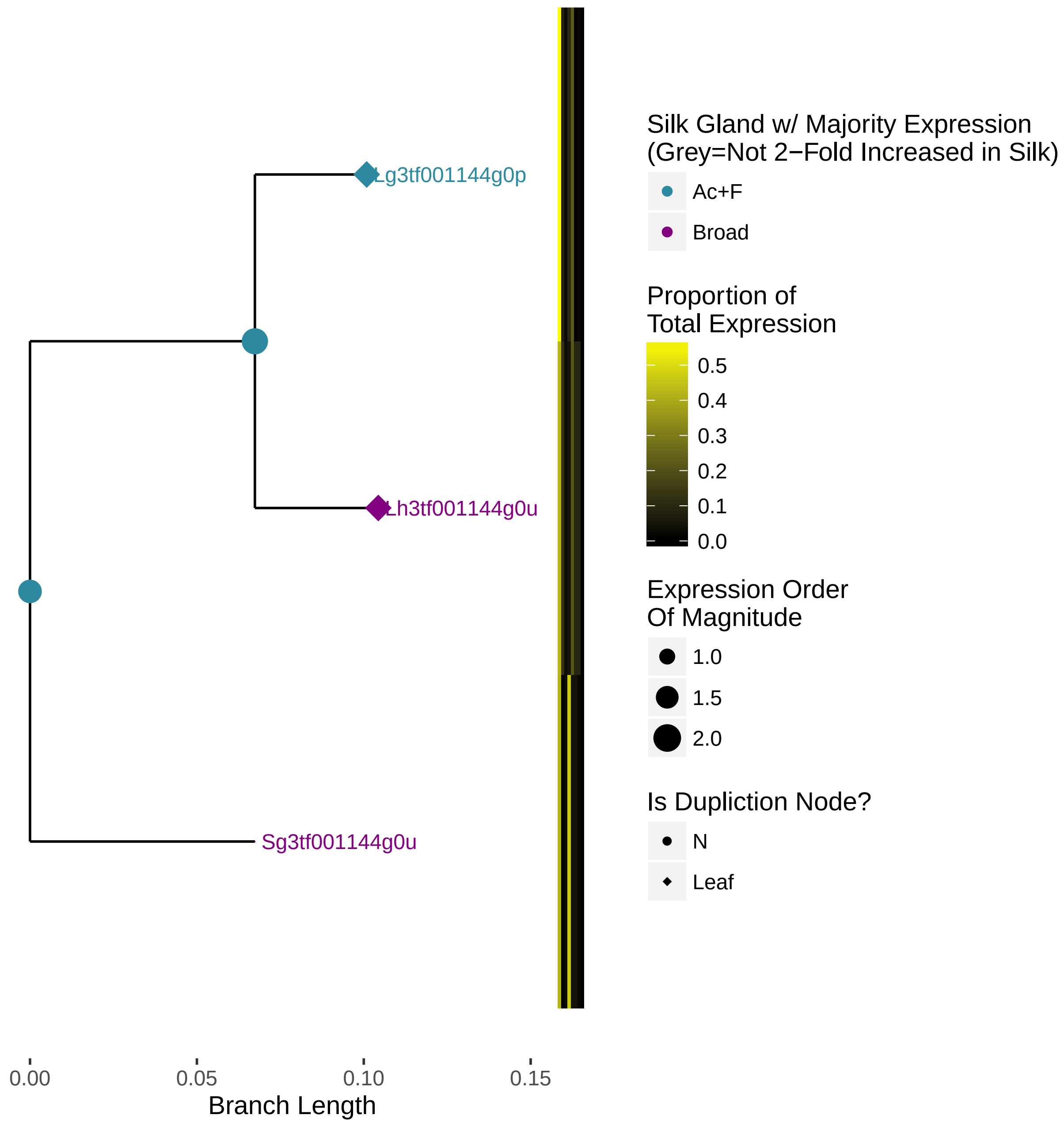

Lg3tf001144g0p
Lh3tf001144g0u
Sg3tf001144g0u
0.00
0.05
0.10
0.15
Branch Length
Silk Gland w/ Majority Expression
(Grey=Not 2−Fold Increased in Silk)
Ac+F
Broad
Proportion of
Total Expression
0.5
0.4
0.3
0.2
0.1
0.0
Expression Order
Of Magnitude
1.0
1.5
2.0
Is Dupliction Node?
N
Leaf

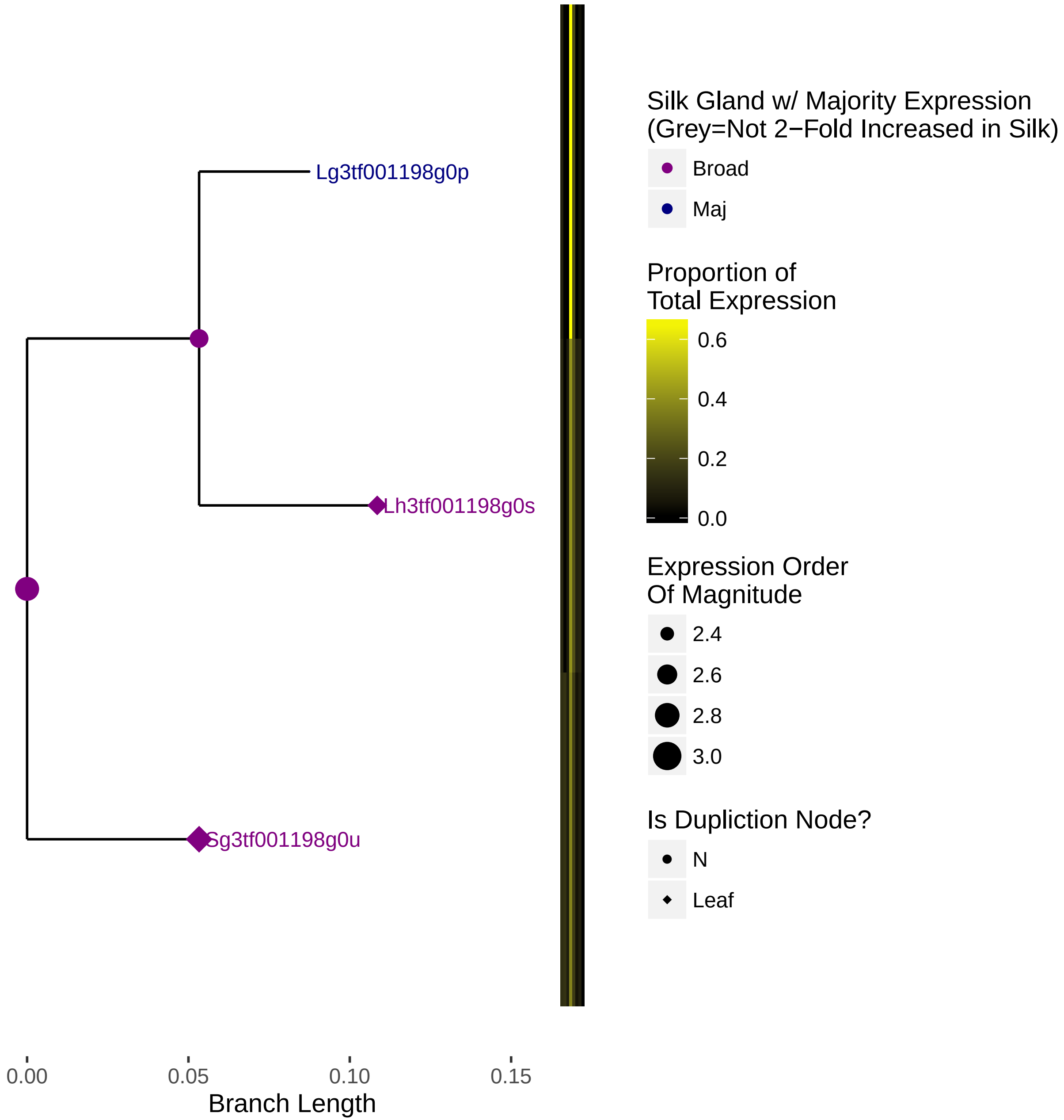
Lg3tf001198g0p
Lh3tf001198g0s
Sg3tf001198g0u
0.00
0.05
0.10
0.15
Branch Length
Silk Gland w/ Majority Expression
(Grey=Not 2−Fold Increased in Silk)
Broad
Maj
Proportion of
Total Expression
0.6
0.4
0.2
0.0
Expression Order
Of Magnitude
2.4
2.6
2.8
3.0
Is Dupliction Node?
N
Leaf

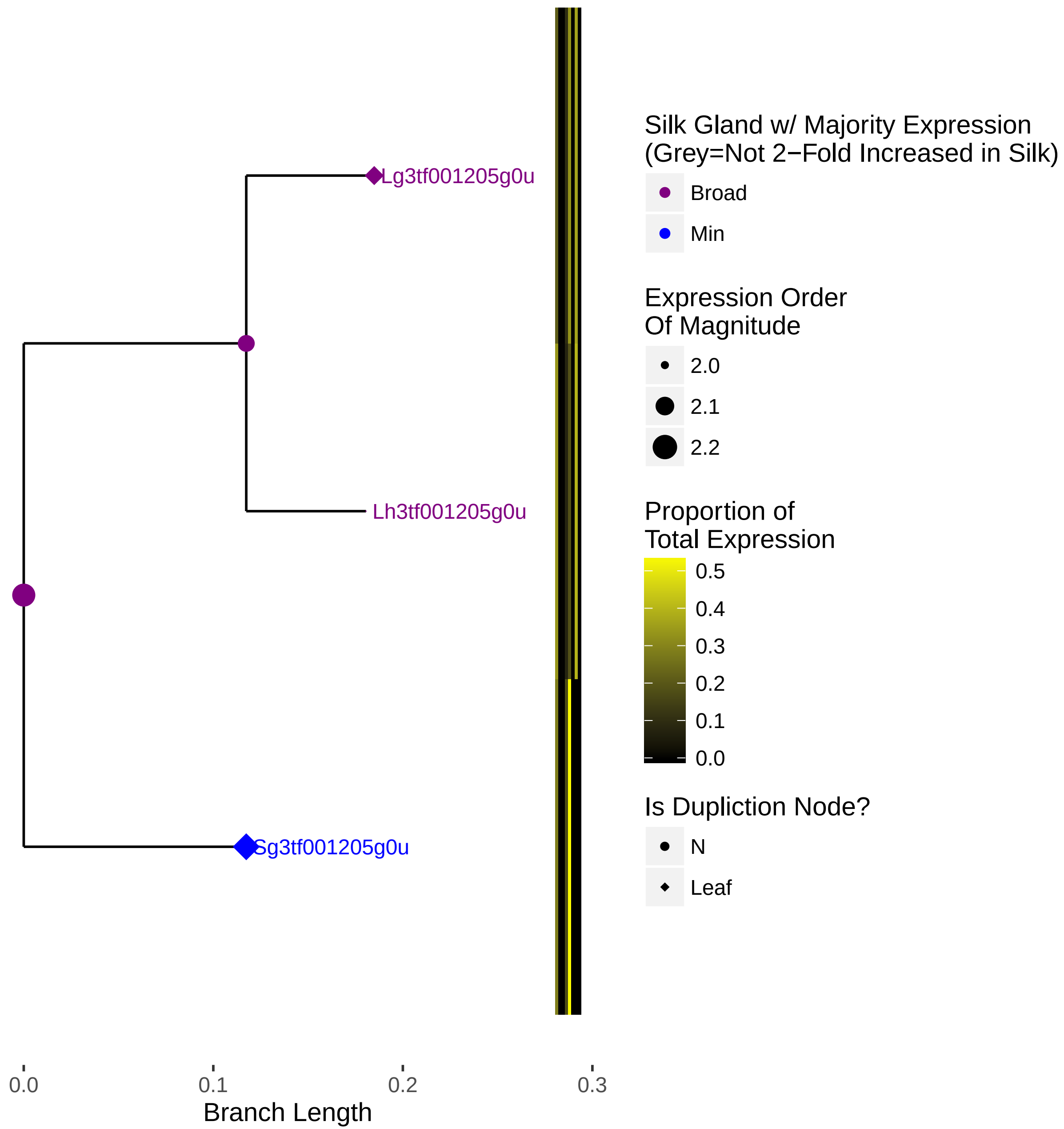

Lg3tf001205g0u
Lh3tf001205g0u
Sg3tf001205g0u
0.0
0.1
0.2
0.3
Branch Length
Silk Gland w/ Majority Expression
(Grey=Not 2−Fold Increased in Silk)
Broad
Min
Expression Order
Of Magnitude
2.0
2.1
2.2
Proportion of
Total Expression
0.5
0.4
0.3
0.2
0.1
0.0
Is Dupliction Node?
N
Leaf

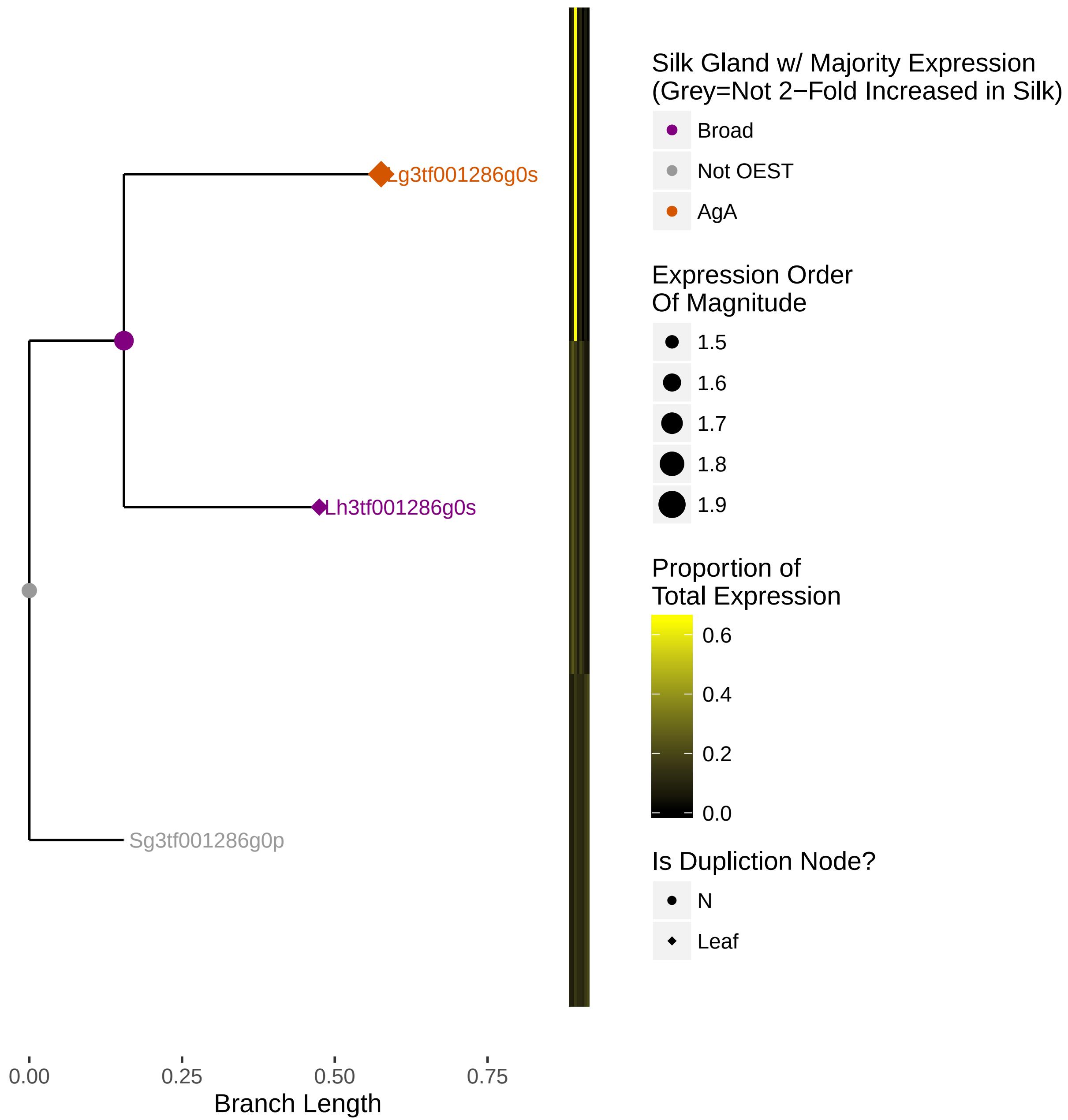
Lg3tf001286g0s
Lh3tf001286g0s
Sg3tf001286g0p
0.00
0.25
0.50
0.75
Branch Length
Silk Gland w/ Majority Expression
(Grey=Not 2−Fold Increased in Silk)
Broad
Not OEST
AgA
Expression Order
Of Magnitude
1.5
1.6
1.7
1.8
1.9
Proportion of
Total Expression
0.6
0.4
0.2
0.0
Is Dupliction Node?
N
Leaf

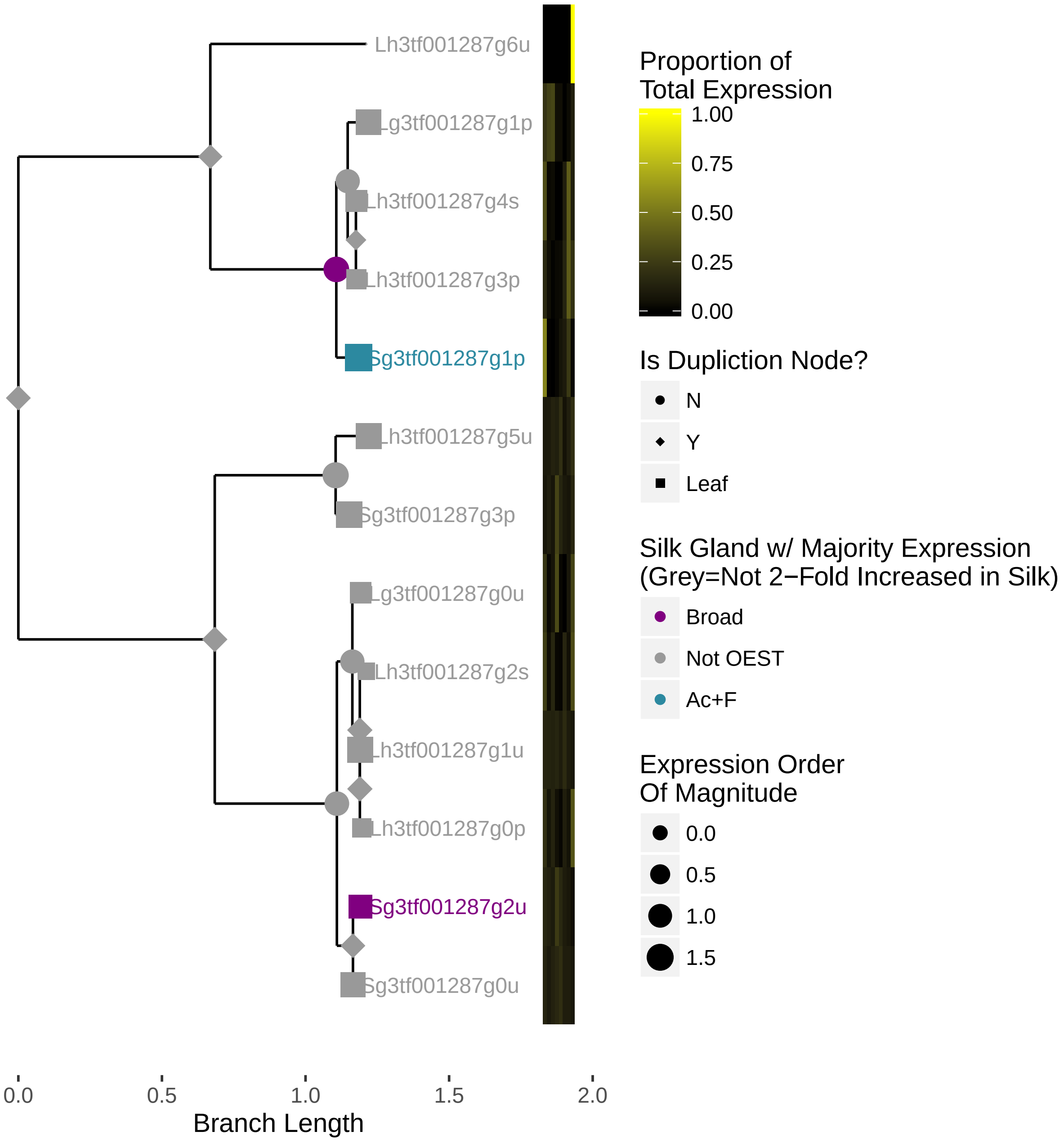

Lh3tf001287g6u
Lg3tf001287g1p
Lh3tf001287g4s
Lh3tf001287g3p
Sg3tf001287g1p
Lh3tf001287g5u
Sg3tf001287g3p
Lg3tf001287g0u
Lh3tf001287g2s
Lh3tf001287g1u
Lh3tf001287g0p
Sg3tf001287g2u
Sg3tf001287g0u
0.0
0.5
1.0
1.5
2.0
Branch Length
Proportion of Total Expression
1.00
0.75
0.50
0.25
0.00
Is Dupliction Node?
N
Y
Leaf
Silk Gland w/ Majority Expression (Grey=Not 2−Fold Increased in Silk)
Broad
Not OEST
Ac+F
Expression Order Of Magnitude
0.0
0.5
1.0
1.5

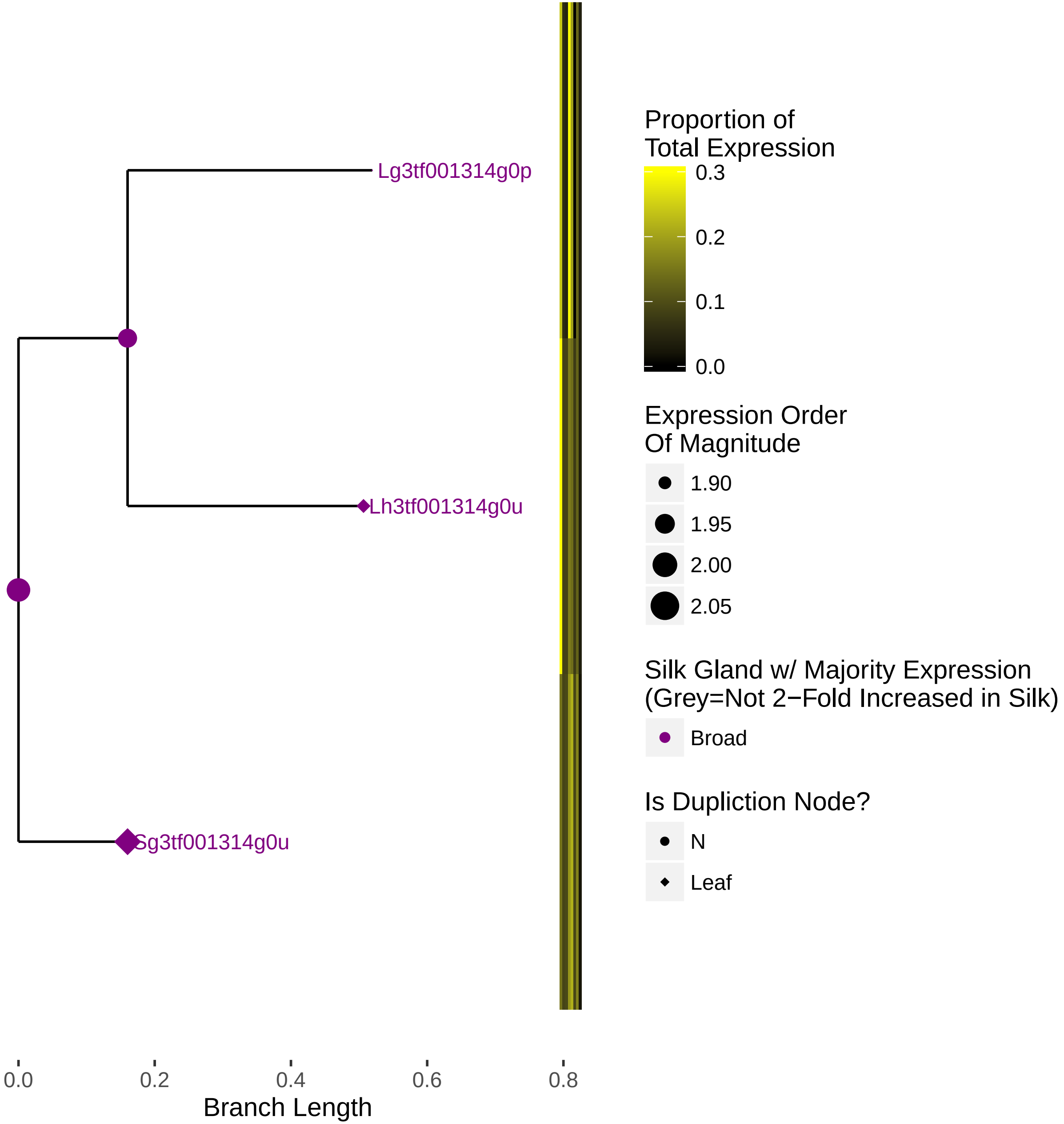
Lg3tf001314g0p
Lh3tf001314g0u
Sg3tf001314g0u
0.0
0.2
0.4
0.6
0.8
Branch Length
Proportion of
Total Expression
0.3
0.2
0.1
0.0
Expression Order
Of Magnitude
1.90
1.95
2.00
2.05
Silk Gland w/ Majority Expression
(Grey=Not 2−Fold Increased in Silk)
Broad
Is Dupliction Node?
N
Leaf

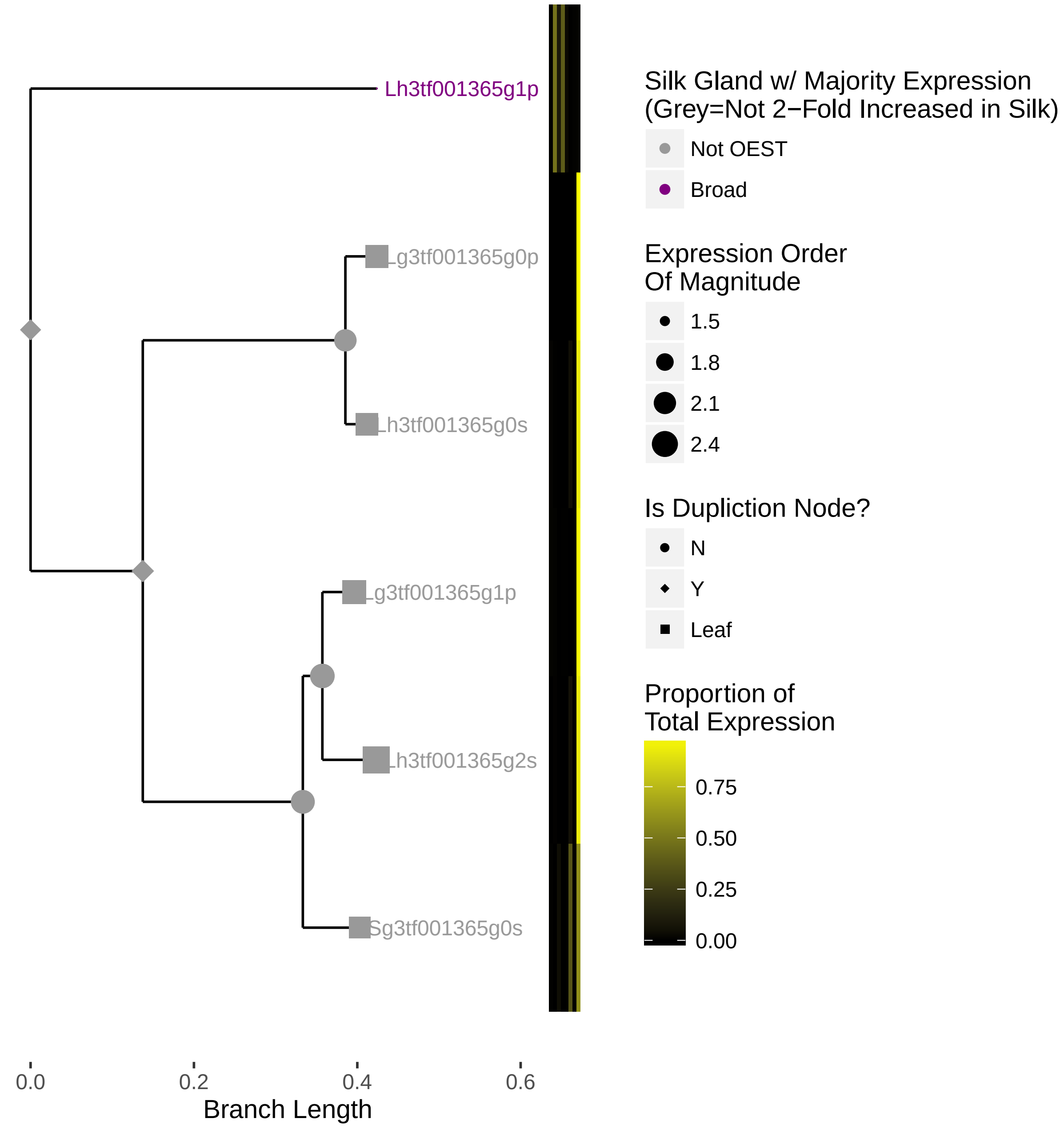

Lh3tf001365g1p
Lg3tf001365g0p
Lh3tf001365g0s
Lg3tf001365g1p
Lh3tf001365g2s
Sg3tf001365g0s
0.0
0.2
0.4
0.6
Branch Length
Silk Gland w/ Majority Expression
(Grey=Not 2−Fold Increased in Silk)
Not OEST
Broad
Expression Order
Of Magnitude
1.5
1.8
2.1
2.4
Is Dupliction Node?
N
Y
Leaf
Proportion of
Total Expression
0.75
0.50
0.25
0.00

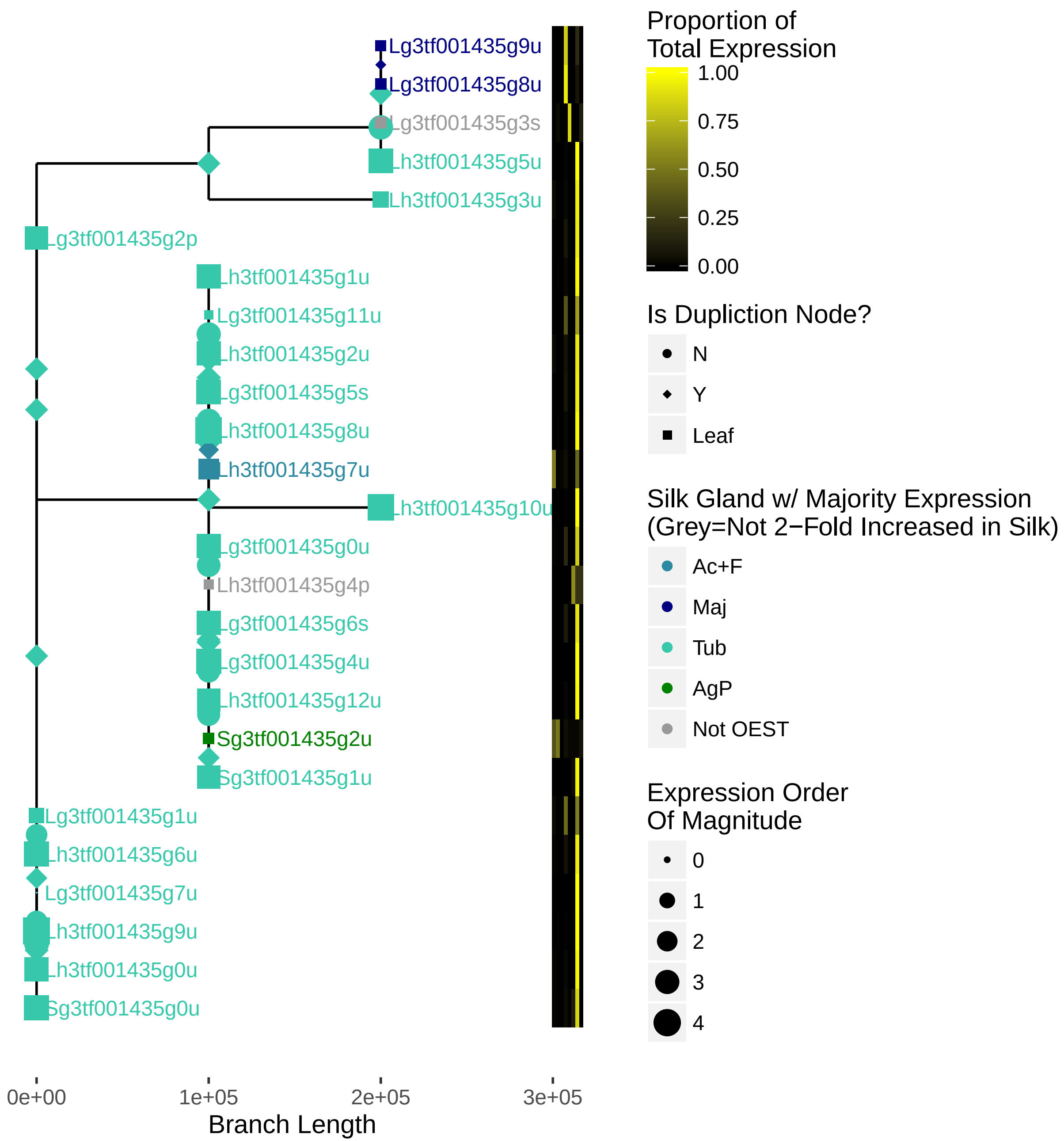

Lg3tf001435g9u
Lg3tf001435g8u
g3tf001435g3s
h3tf001435g5u
Lh3tf001435g3u
g3tf001435g2p
h3tf001435g1u
Lg3tf001435g11u
h3tf001435g2u
g3tf001435g5s
h3tf001435g8u
Lh3tf001435g7u
h3tf001435g10u
g3tf001435g0u
Lh3tf001435g4p
g3tf001435g6s
g3tf001435g4u
h3tf001435g12u
Sg3tf001435g2u
Sg3tf001435g1u
Lg3tf001435g1u
h3tf001435g6u
Lg3tf001435g7u
h3tf001435g9u
h3tf001435g0u
Sg3tf001435g0u
0e+00
1e+05
2e+05
3e+05
Branch Length
Proportion of
Total Expression
1.00
0.75
0.50
0.25
0.00
Is Dupliction Node?
N
Y
Leaf
Silk Gland w/ Majority Expression
(Grey=Not 2−Fold Increased in Silk)
Ac+F
Maj
Tub
AgP
Not OEST
Expression Order
Of Magnitude
0
1
2
3
4

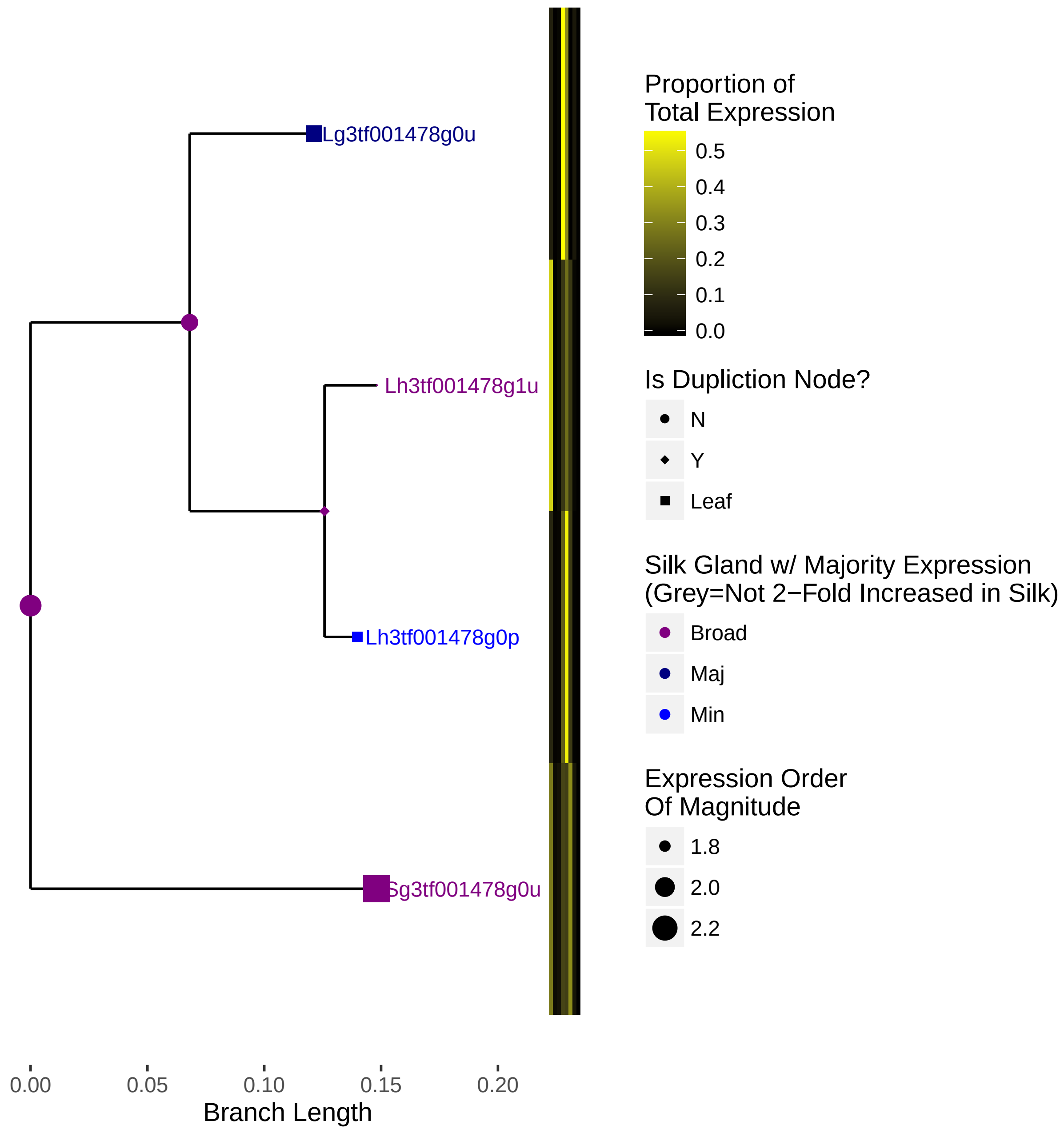

Lg3tf001478g0u
Lh3tf001478g1u
Lh3tf001478g0p
Sg3tf001478g0u
0.00
0.05
0.10
0.15
0.20
Branch Length
Proportion of
Total Expression
0.5
0.4
0.3
0.2
0.1
0.0
Is Dupliction Node?
N
Y
Leaf
Silk Gland w/ Majority Expression
(Grey=Not 2−Fold Increased in Silk)
Broad
Maj
Min
Expression Order
Of Magnitude
1.8
2.0
2.2

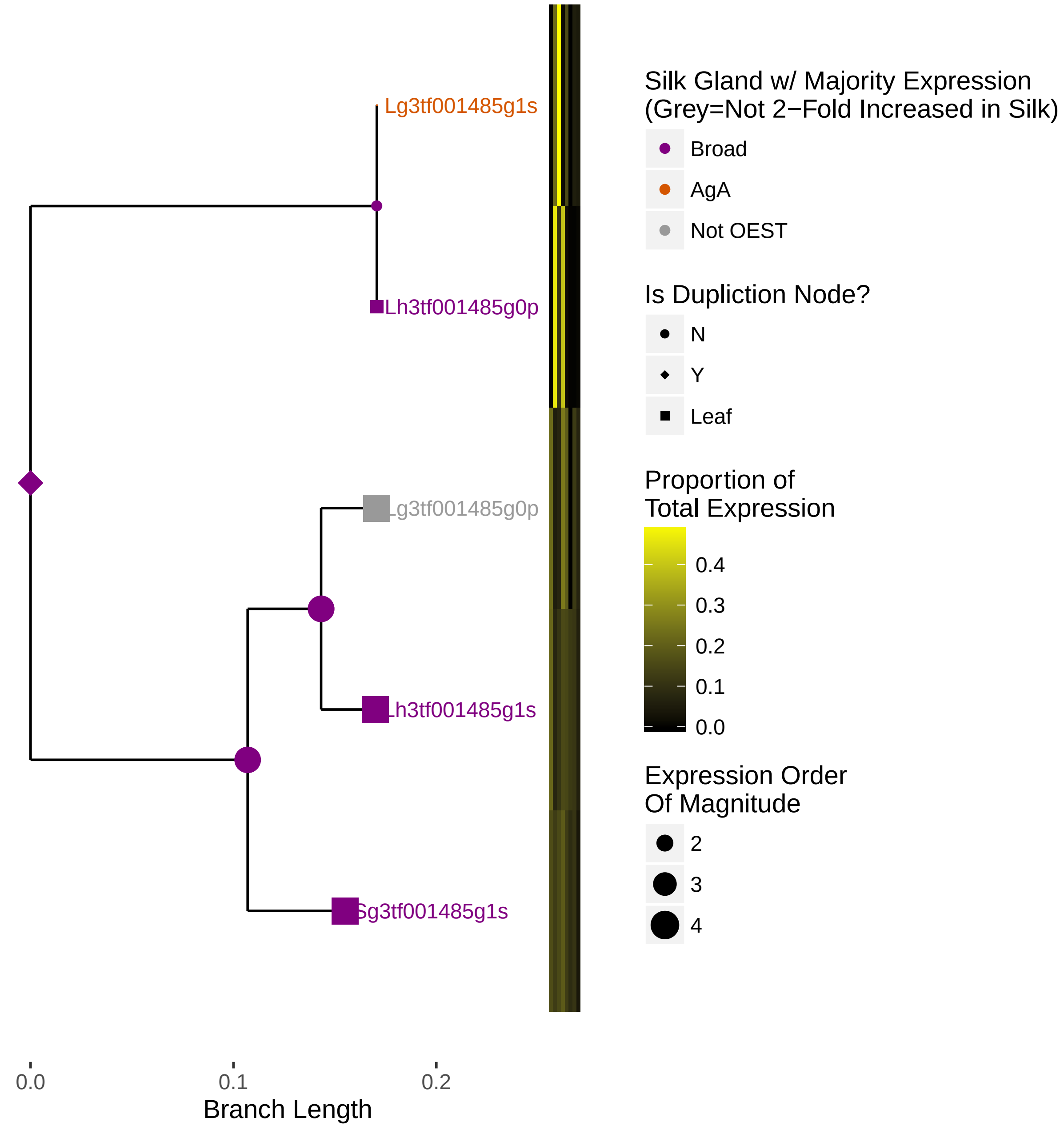

Lg3tf001485g1s
Lh3tf001485g0p
Lg3tf001485g0p
Lh3tf001485g1s
Sg3tf001485g1s
Silk Gland w/ Majority Expression
(Grey=Not 2−Fold Increased in Silk)
Broad
AgA
Not OEST
Is Dupliction Node?
N
Y
Leaf
Proportion of
Total Expression
0.4
0.3
0.2
0.1
0.0
Expression Order
Of Magnitude
2
3
4
0.0
0.1
0.2
Branch Length

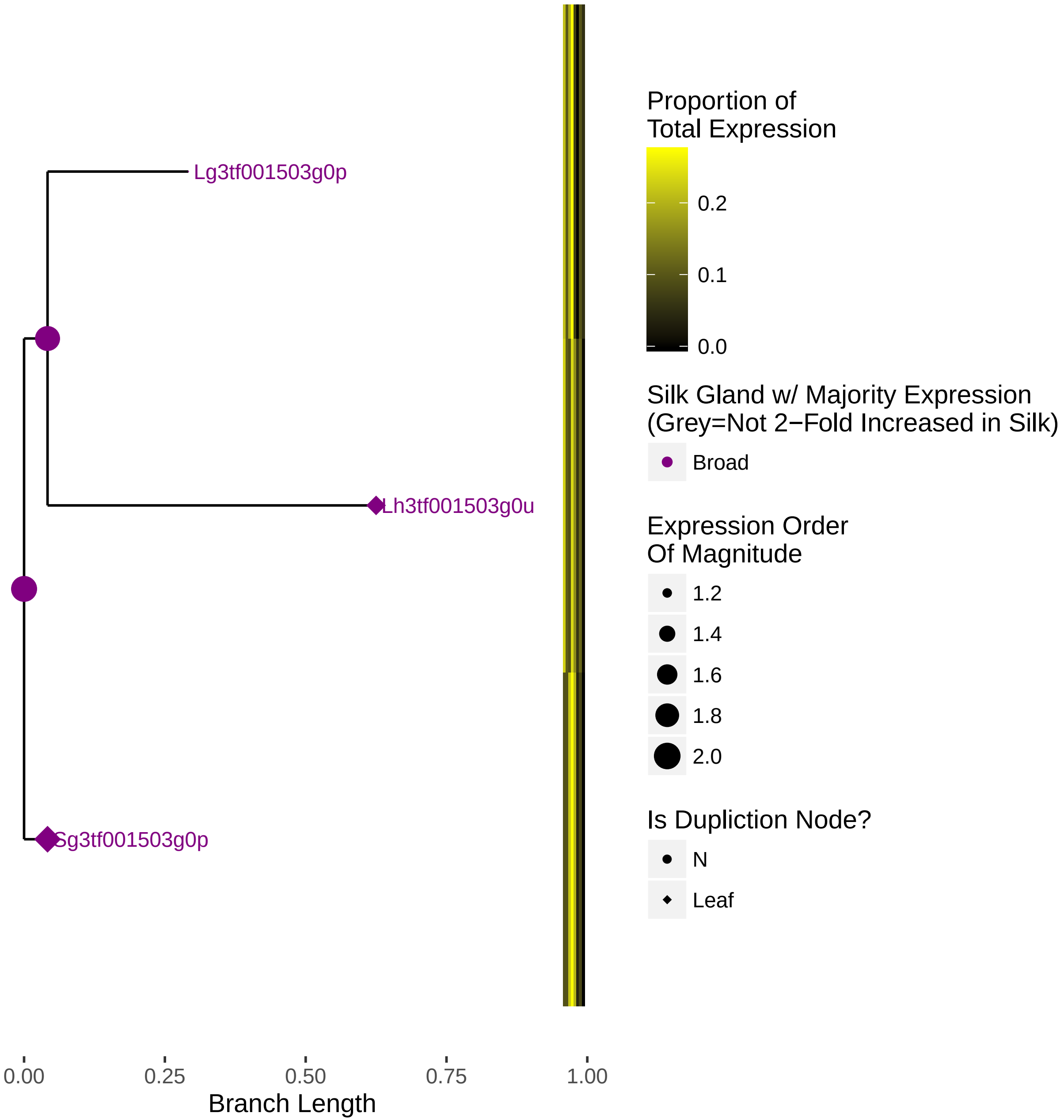
Lg3tf001503g0p
Lh3tf001503g0u
Sg3tf001503g0p
0.00
0.25
0.50
0.75
1.00
Branch Length
Proportion of Total Expression
0.2
0.1
0.0
Silk Gland w/ Majority Expression (Grey=Not 2−Fold Increased in Silk)
Broad
Expression Order Of Magnitude
1.2
1.4
1.6
1.8
2.0
Is Dupliction Node?
N
Leaf

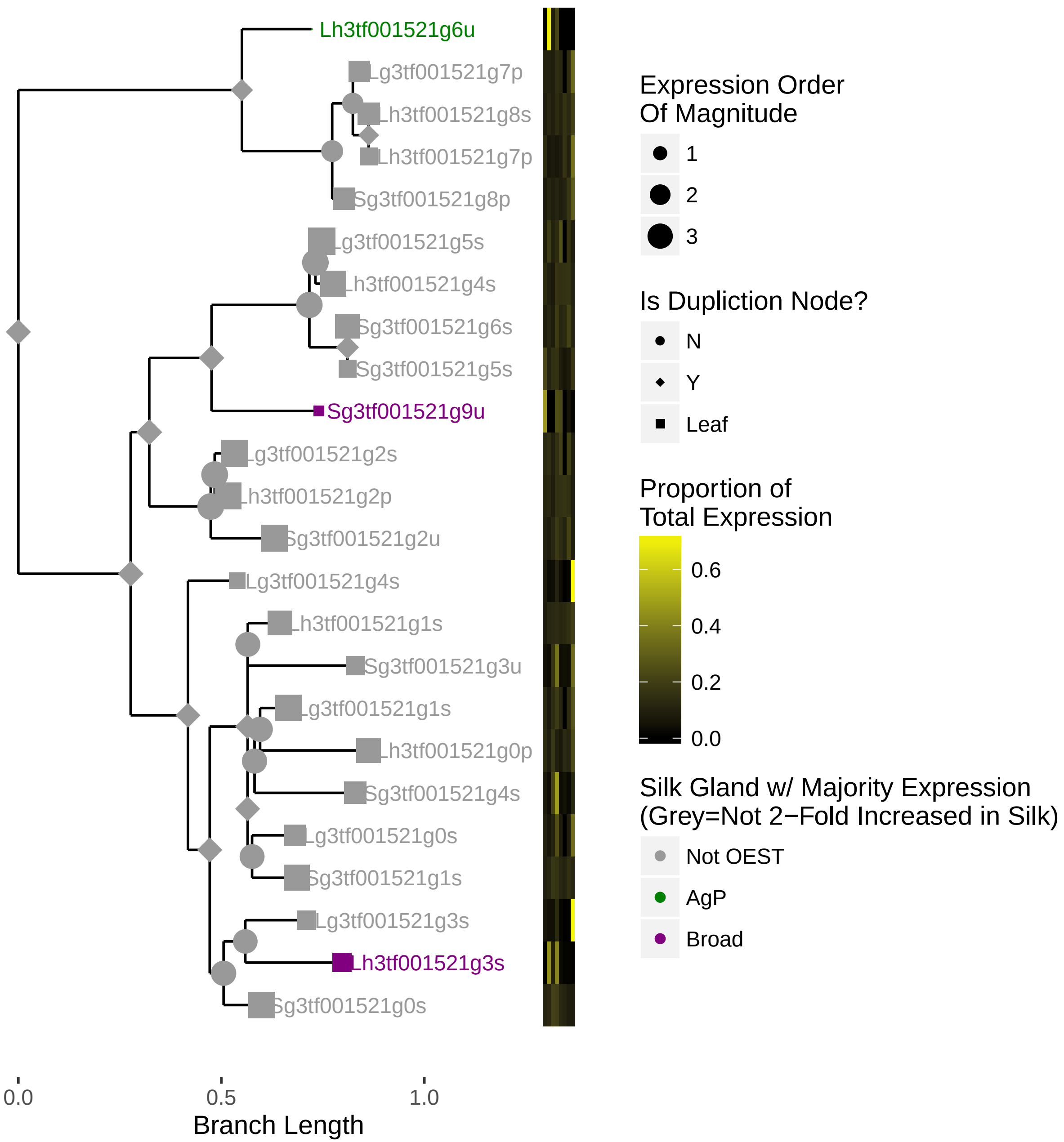

Lh3tf001521g6u
Lg3tf001521g7p
Lh3tf001521g8s
Lh3tf001521g7p
Sg3tf001521g8p
Lg3tf001521g5s
Lh3tf001521g4s
Sg3tf001521g6s
Sg3tf001521g5s
Sg3tf001521g9u
Lg3tf001521g2s
Lh3tf001521g2p
Sg3tf001521g2u
Lg3tf001521g4s
Lh3tf001521g1s
Sg3tf001521g3u
Lg3tf001521g1s
Lh3tf001521g0p
Sg3tf001521g4s
Lg3tf001521g0s
Sg3tf001521g1s
Lg3tf001521g3s
Lh3tf001521g3s
Sg3tf001521g0s
0.0
0.5
1.0
Branch Length
Expression Order Of Magnitude
1
2
3
Is Dupliction Node?
N
Y
Leaf
Proportion of Total Expression
0.6
0.4
0.2
0.0
Silk Gland w/ Majority Expression (Grey=Not 2−Fold Increased in Silk)
Not OEST
AgP
Broad

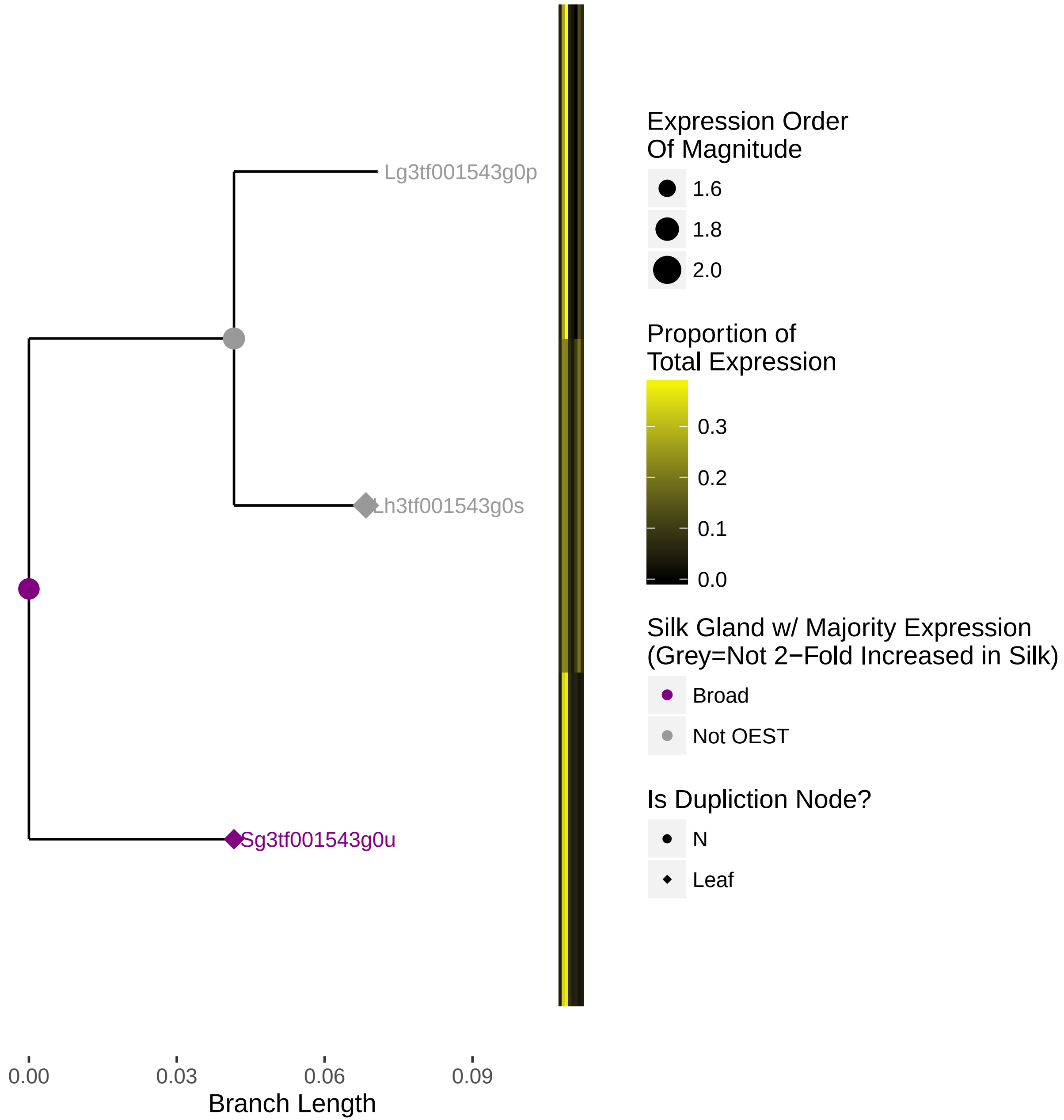
Lg3tf001543g0p
Lh3tf001543g0s
Sg3tf001543g0u
0.00
0.03
0.06
0.09
Branch Length
Expression Order
Of Magnitude
1.6
1.8
2.0
Proportion of
Total Expression
0.3
0.2
0.1
0.0
Silk Gland w/ Majority Expression
(Grey=Not 2−Fold Increased in Silk)
Broad
Not OEST
Is Dupliction Node?
N
Leaf

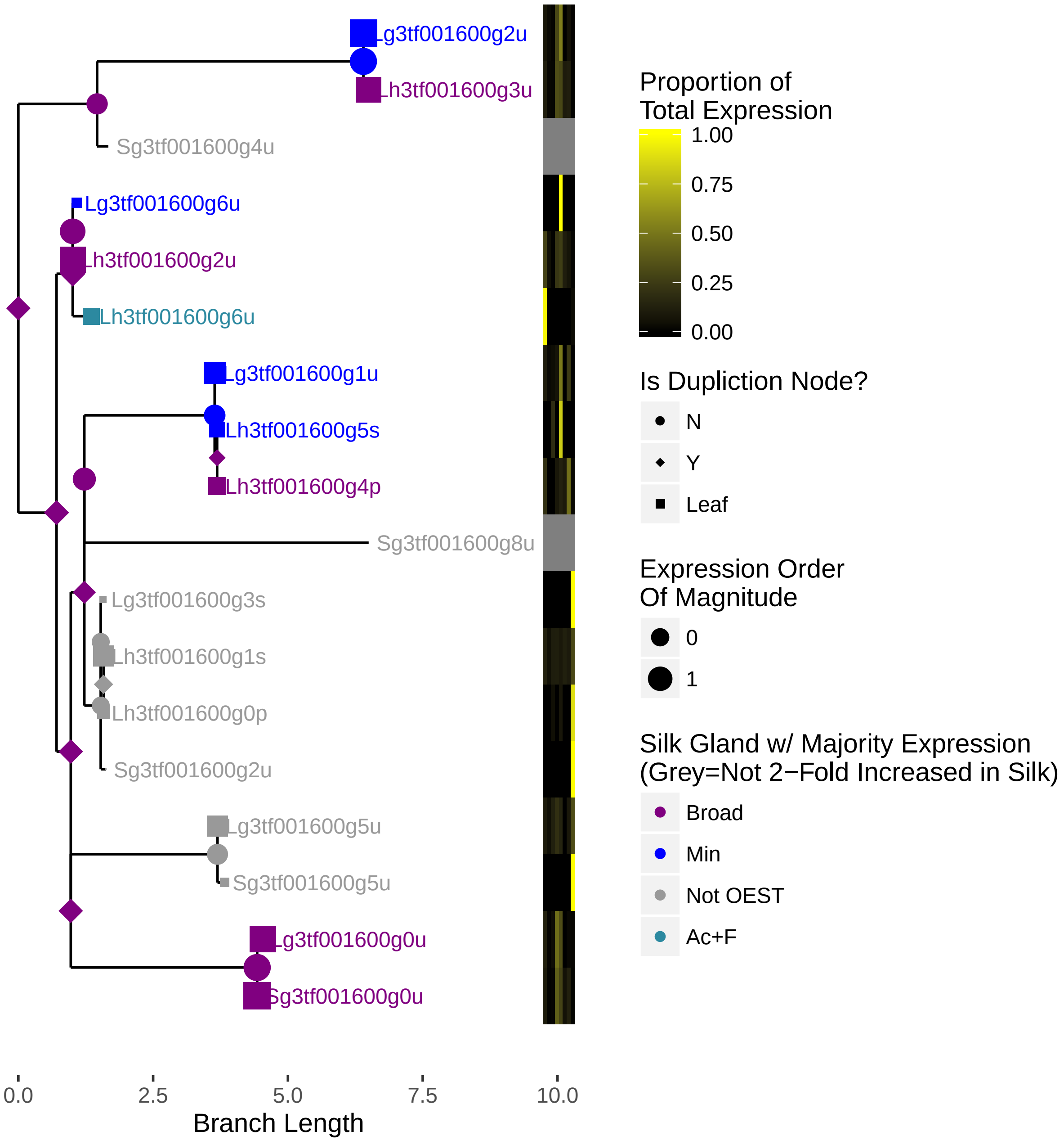
Lg3tf001600g2u
Lh3tf001600g3u
Sg3tf001600g4u
Lg3tf001600g6u
Lh3tf001600g2u
Lh3tf001600g6u
Lg3tf001600g1u
Lh3tf001600g5s
Lh3tf001600g4p
Sg3tf001600g8u
Lg3tf001600g3s
Lh3tf001600g1s
Lh3tf001600g0p
Sg3tf001600g2u
Lg3tf001600g5u
Sg3tf001600g5u
Lg3tf001600g0u
Sg3tf001600g0u
0.0
2.5
5.0
7.5
10.0
Branch Length
Proportion of Total Expression
1.00
0.75
0.50
0.25
0.00
Is Dupliction Node?
N
Y
Leaf
Expression Order Of Magnitude
0
1
Silk Gland w/ Majority Expression (Grey=Not 2−Fold Increased in Silk)
Broad
Min
Not OEST
Ac+F

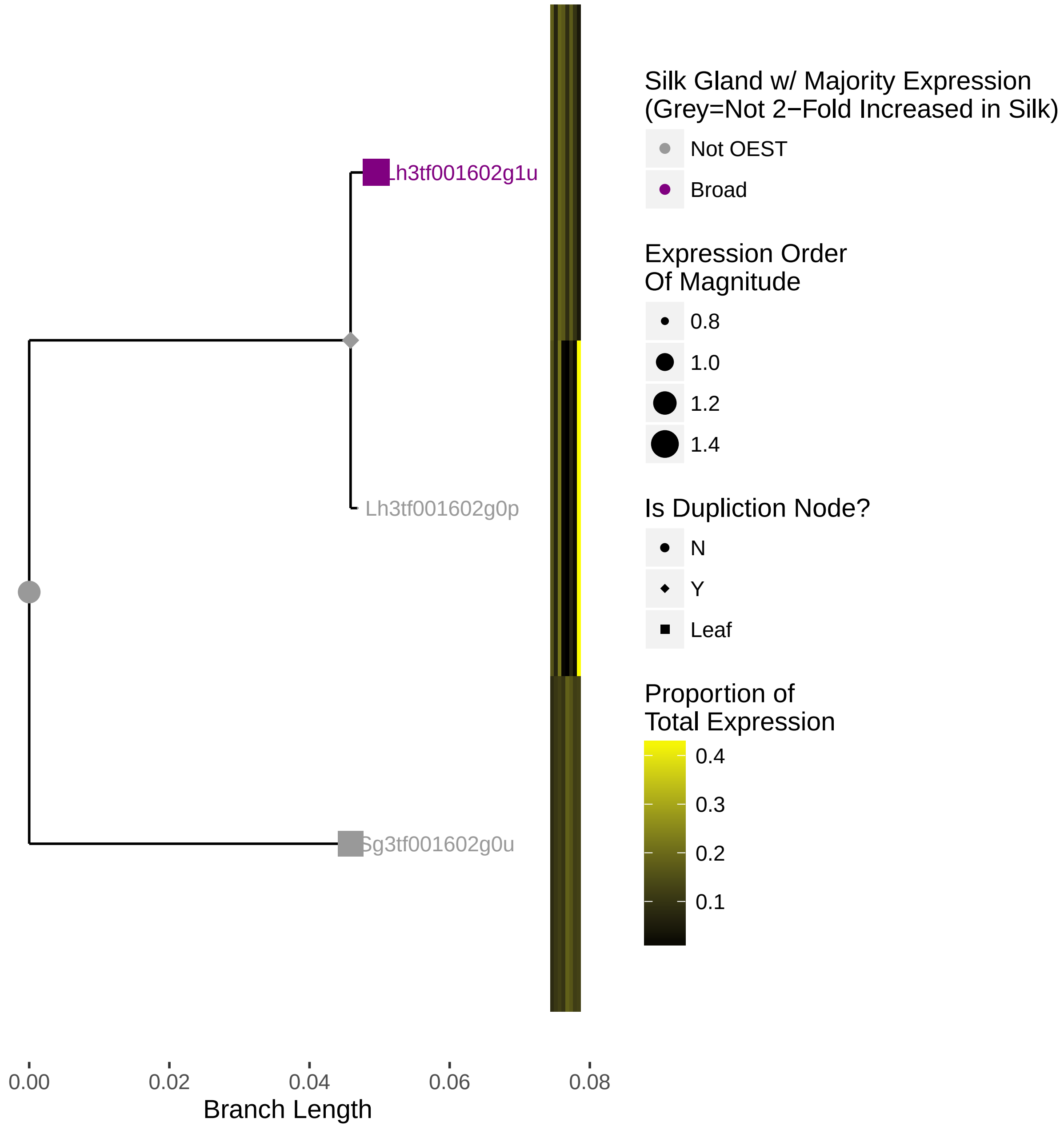
h3tf001602g1u
Lh3tf001602g0p
g3tf001602g0u
0.00
0.02
0.04
0.06
0.08
Branch Length
Silk Gland w/ Majority Expression
(Grey=Not 2−Fold Increased in Silk)
Not OEST
Broad
Expression Order
Of Magnitude
0.8
1.0
1.2
1.4
Is Dupliction Node?
N
Y
Leaf
Proportion of
Total Expression
0.4
0.3
0.2
0.1

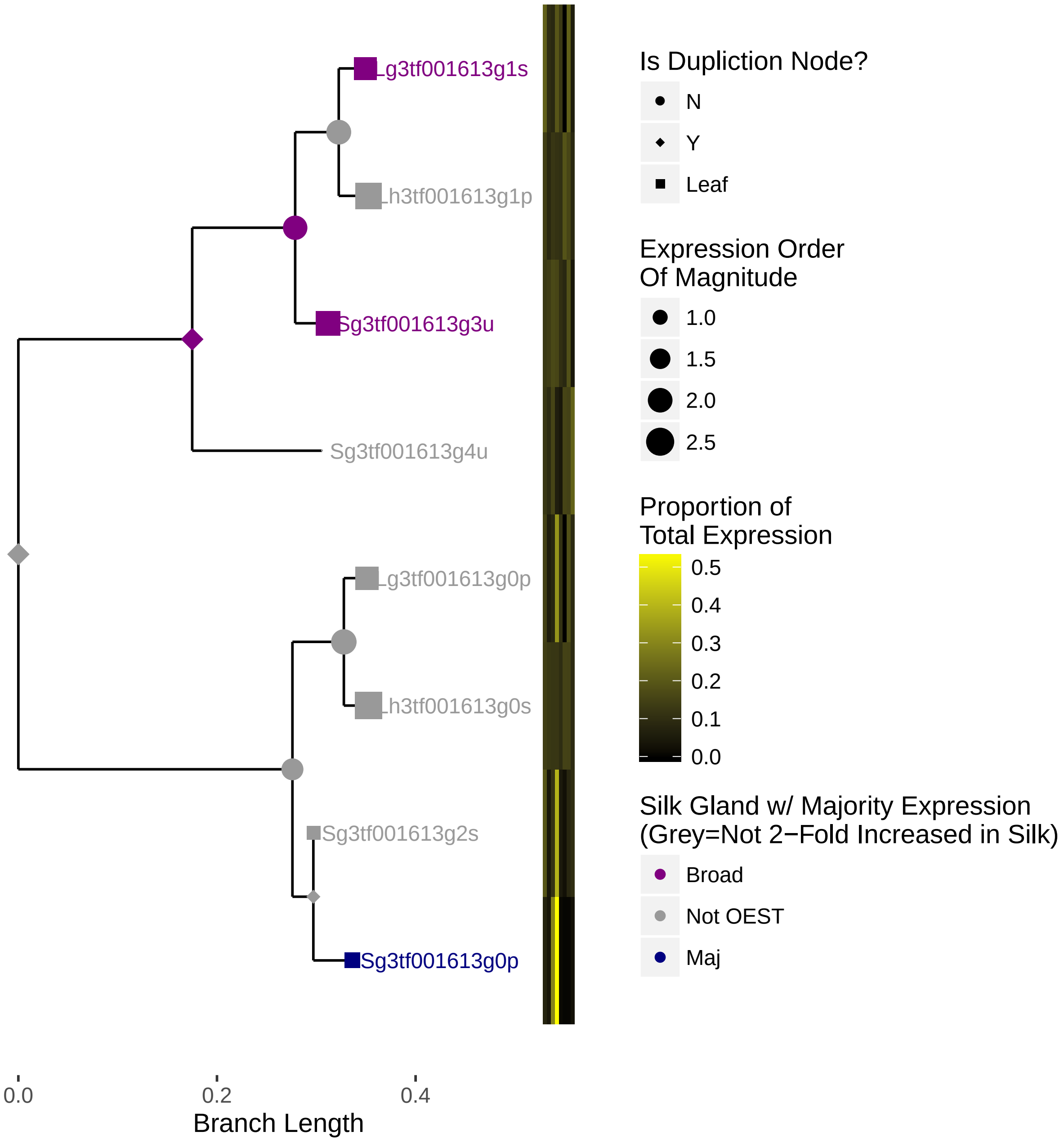

Lg3tf001613g1s
Lh3tf001613g1p
Sg3tf001613g3u
Sg3tf001613g4u
Lg3tf001613g0p
Lh3tf001613g0s
Sg3tf001613g2s
Sg3tf001613g0p
0.0
0.2
0.4
Branch Length
Is Dupliction Node?
N
Y
Leaf
Expression Order Of Magnitude
1.0
1.5
2.0
2.5
Proportion of Total Expression
0.5
0.4
0.3
0.2
0.1
0.0
Silk Gland w/ Majority Expression (Grey=Not 2−Fold Increased in Silk)
Broad
Not OEST
Maj

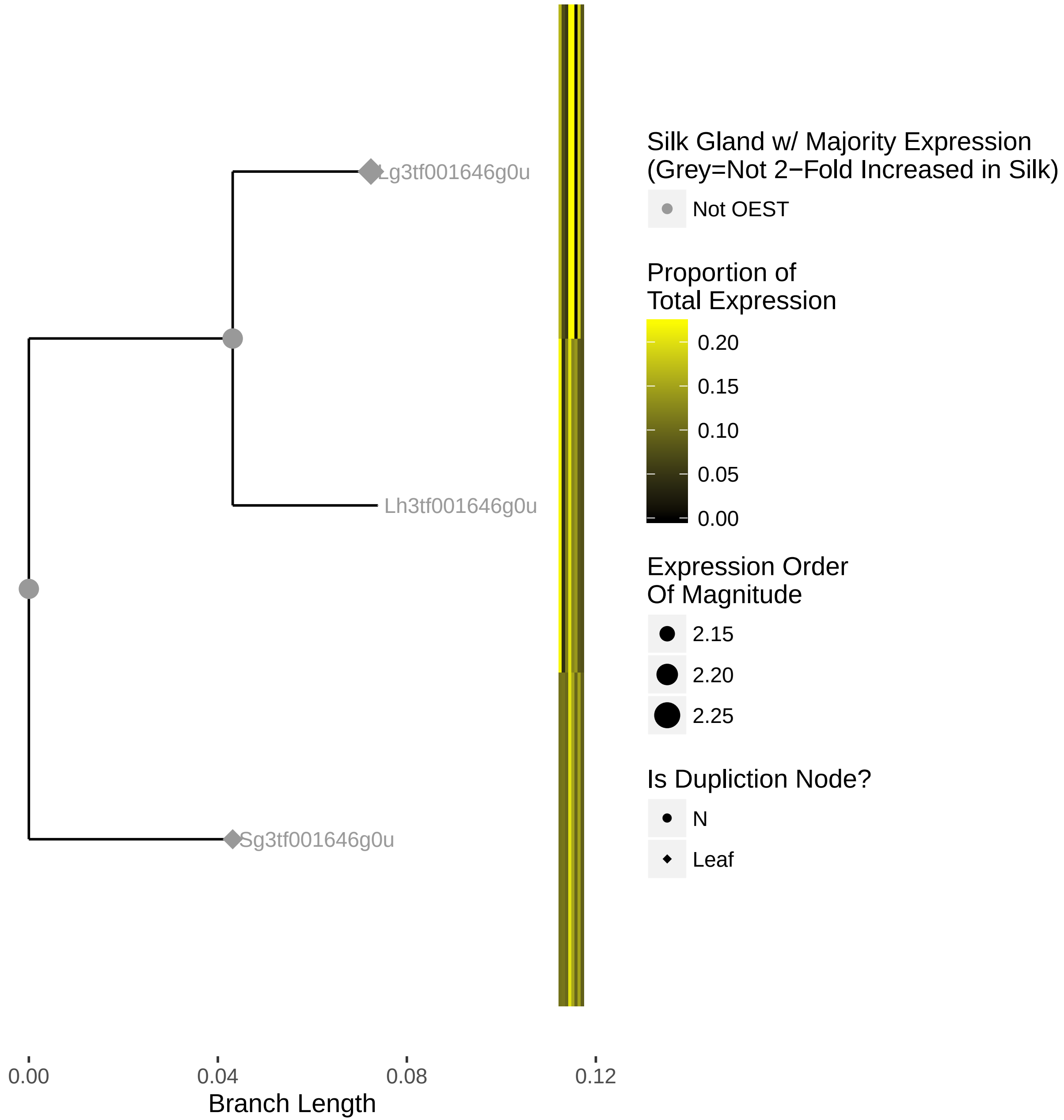

Lg3tf001646g0u
Lh3tf001646g0u
Sg3tf001646g0u
0.00
0.04
0.08
0.12
Branch Length
Silk Gland w/ Majority Expression
(Grey=Not 2−Fold Increased in Silk)
Not OEST
Proportion of
Total Expression
0.20
0.15
0.10
0.05
0.00
Expression Order
Of Magnitude
2.15
2.20
2.25
Is Dupliction Node?
N
Leaf

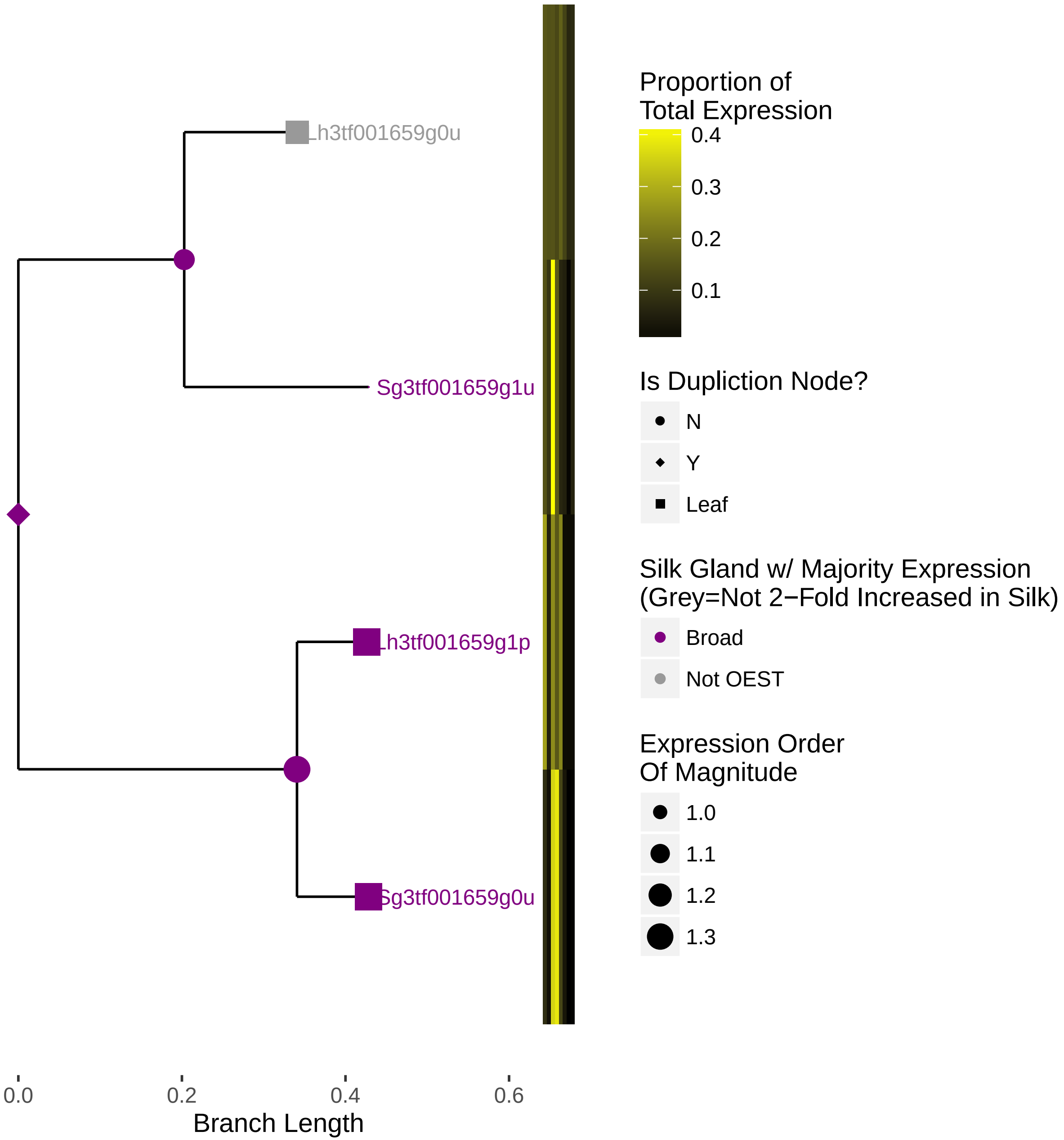

Lh3tf001659g0u
Sg3tf001659g1u
Lh3tf001659g1p
Sg3tf001659g0u
0.0
0.2
0.4
0.6
Branch Length
Proportion of
Total Expression
0.4
0.3
0.2
0.1
Is Dupliction Node?
N
Y
Leaf
Silk Gland w/ Majority Expression
(Grey=Not 2−Fold Increased in Silk)
Broad
Not OEST
Expression Order
Of Magnitude
1.0
1.1
1.2
1.3

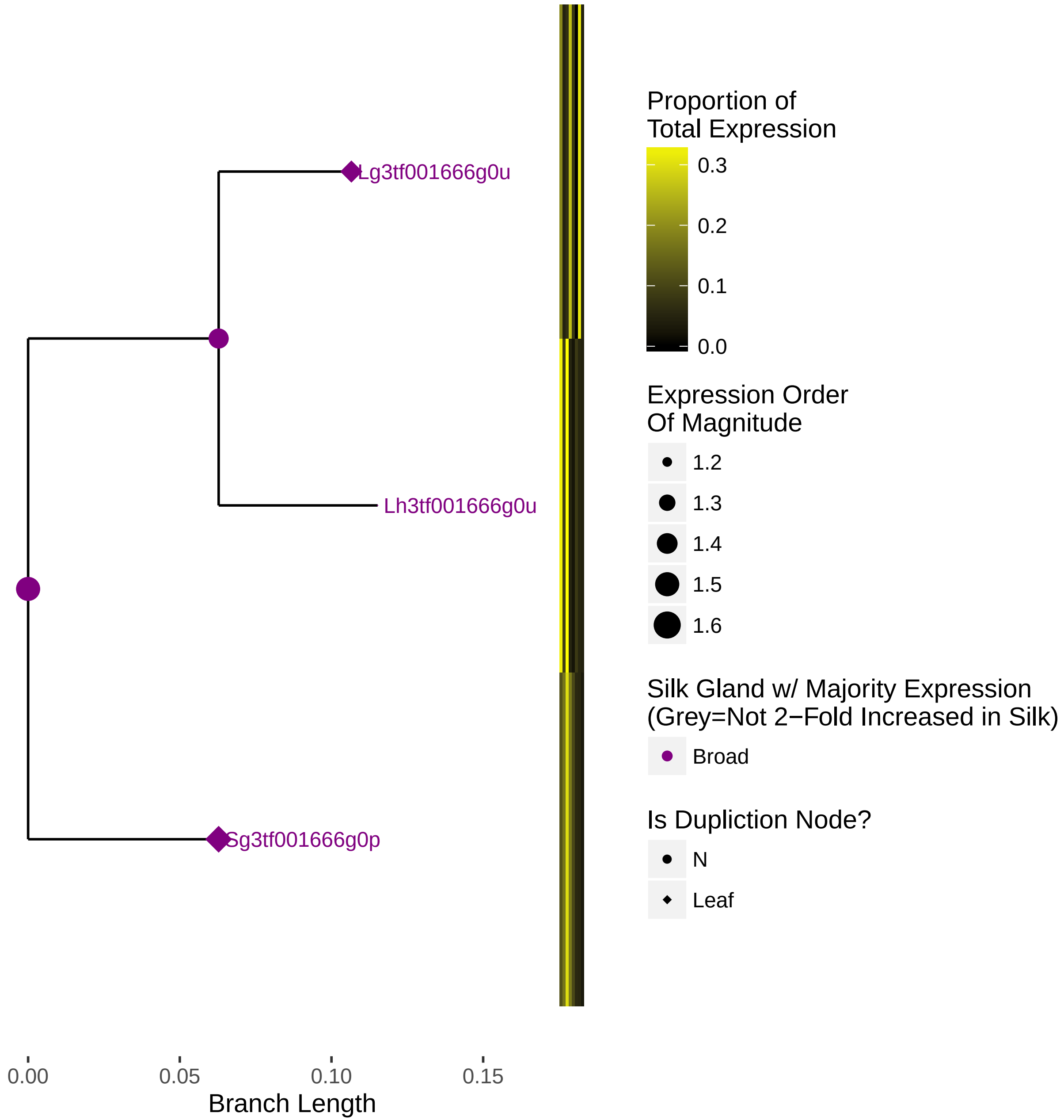

Lg3tf001666g0u
Lh3tf001666g0u
Sg3tf001666g0p
0.00
0.05
0.10
0.15
Branch Length
Proportion of
Total Expression
0.3
0.2
0.1
0.0
Expression Order
Of Magnitude
1.2
1.3
1.4
1.5
1.6
Silk Gland w/ Majority Expression
(Grey=Not 2−Fold Increased in Silk)
Broad
Is Dupliction Node?
N
Leaf

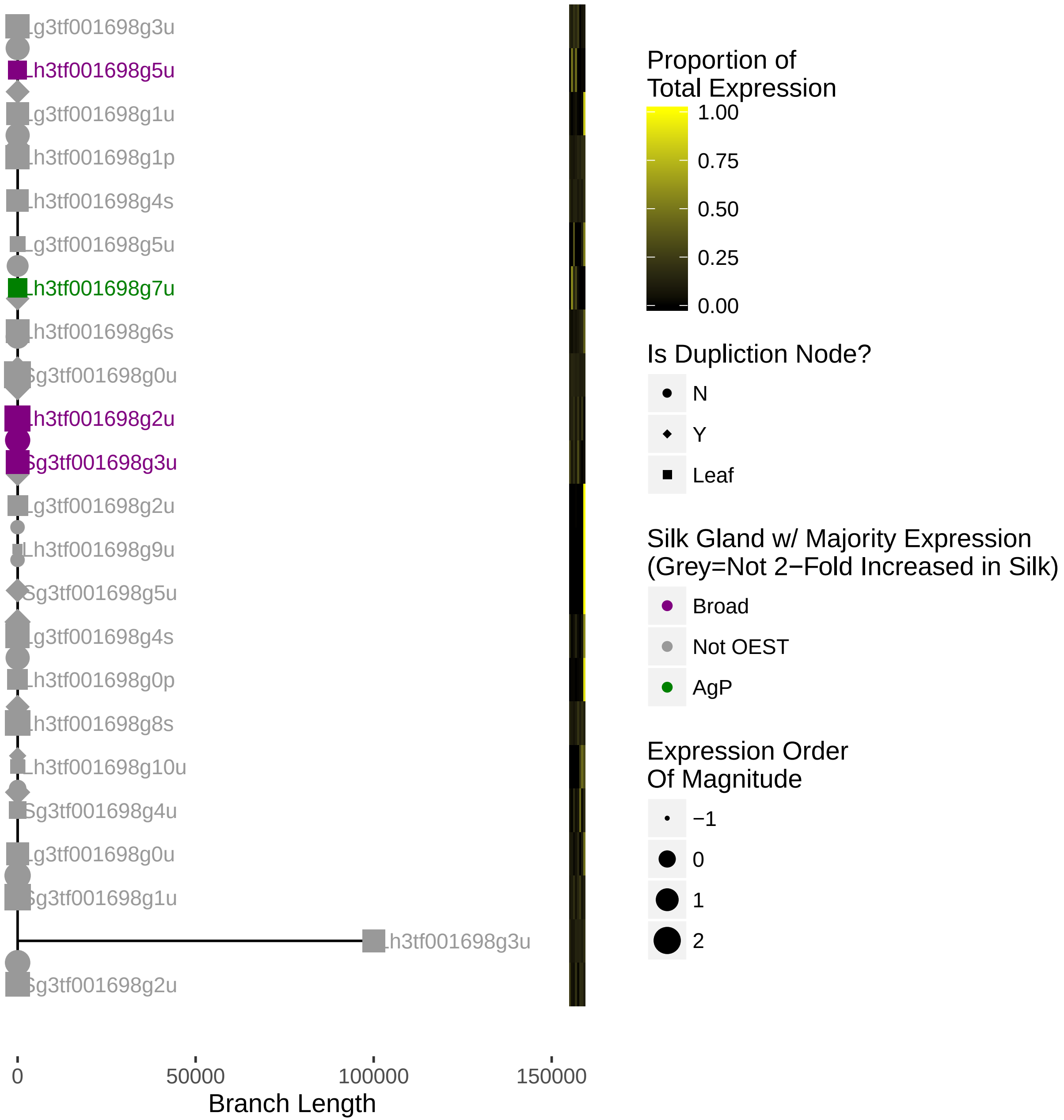

Lg3tf001698g3u
Lh3tf001698g5u
Lg3tf001698g1u
Lh3tf001698g1p
Lh3tf001698g4s
Lg3tf001698g5u
Lh3tf001698g7u
Lh3tf001698g6s
Sg3tf001698g0u
Lh3tf001698g2u
Sg3tf001698g3u
Lg3tf001698g2u
Lh3tf001698g9u
Sg3tf001698g5u
Lg3tf001698g4s
Lh3tf001698g0p
Lh3tf001698g8s
Lh3tf001698g10u
Sg3tf001698g4u
Lg3tf001698g0u
Sg3tf001698g1u
Lh3tf001698g3u
Sg3tf001698g2u
0
50000
100000
150000
Branch Length
Proportion of
Total Expression
1.00
0.75
0.50
0.25
0.00
Is Dupliction Node?
N
Y
Leaf
Silk Gland w/ Majority Expression
(Grey=Not 2−Fold Increased in Silk)
Broad
Not OEST
AgP
Expression Order
Of Magnitude
−1
0
1
2

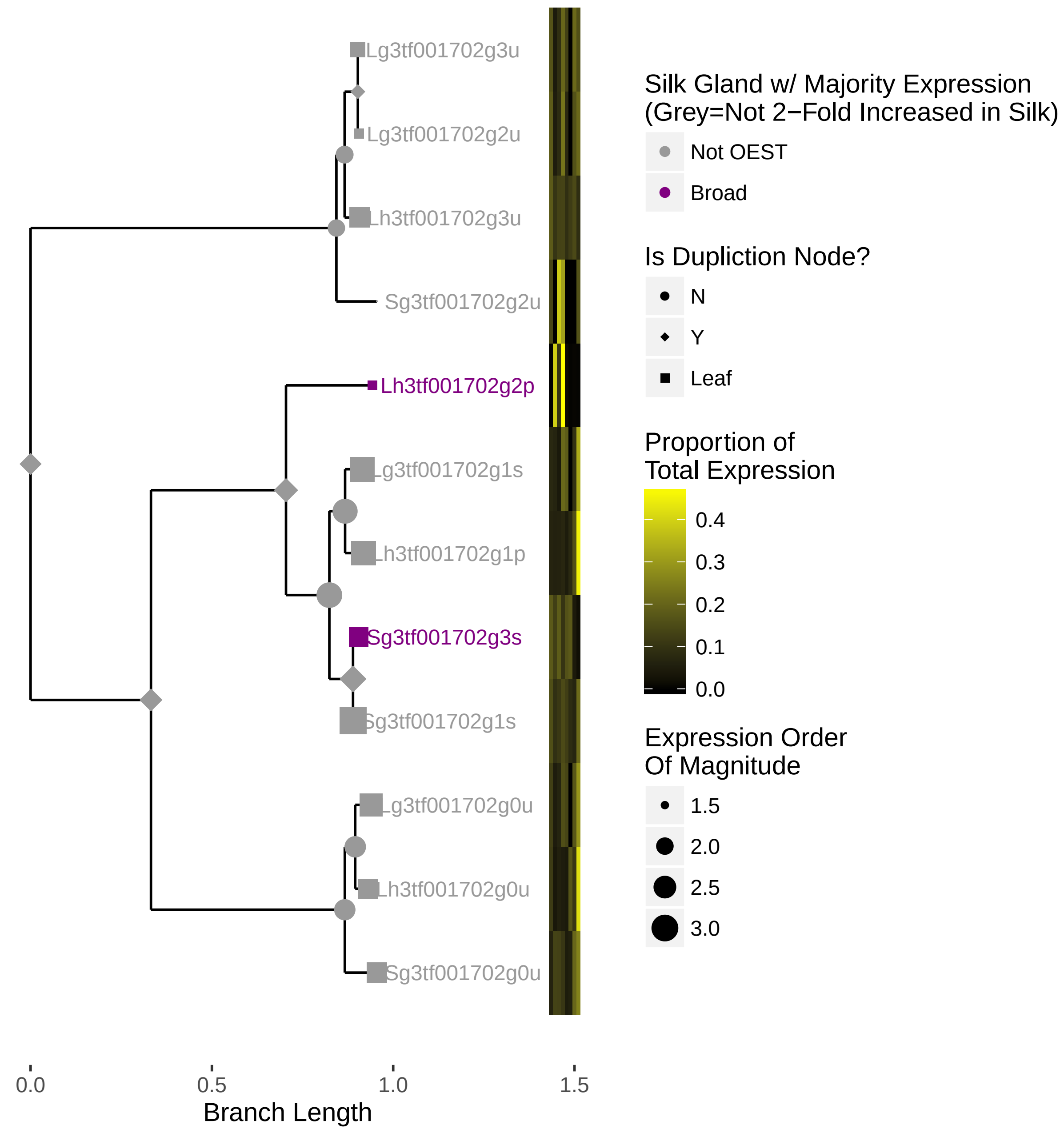

Lg3tf001702g3u
Lg3tf001702g2u
Lh3tf001702g3u
Sg3tf001702g2u
Lh3tf001702g2p
Lg3tf001702g1s
Lh3tf001702g1p
Sg3tf001702g3s
Sg3tf001702g1s
Lg3tf001702g0u
Lh3tf001702g0u
Sg3tf001702g0u
0.0
0.5
1.0
1.5
Branch Length
Silk Gland w/ Majority Expression
(Grey=Not 2−Fold Increased in Silk)
Not OEST
Broad
Is Dupliction Node?
N
Y
Leaf
Proportion of
Total Expression
0.4
0.3
0.2
0.1
0.0
Expression Order
Of Magnitude
1.5
2.0
2.5
3.0

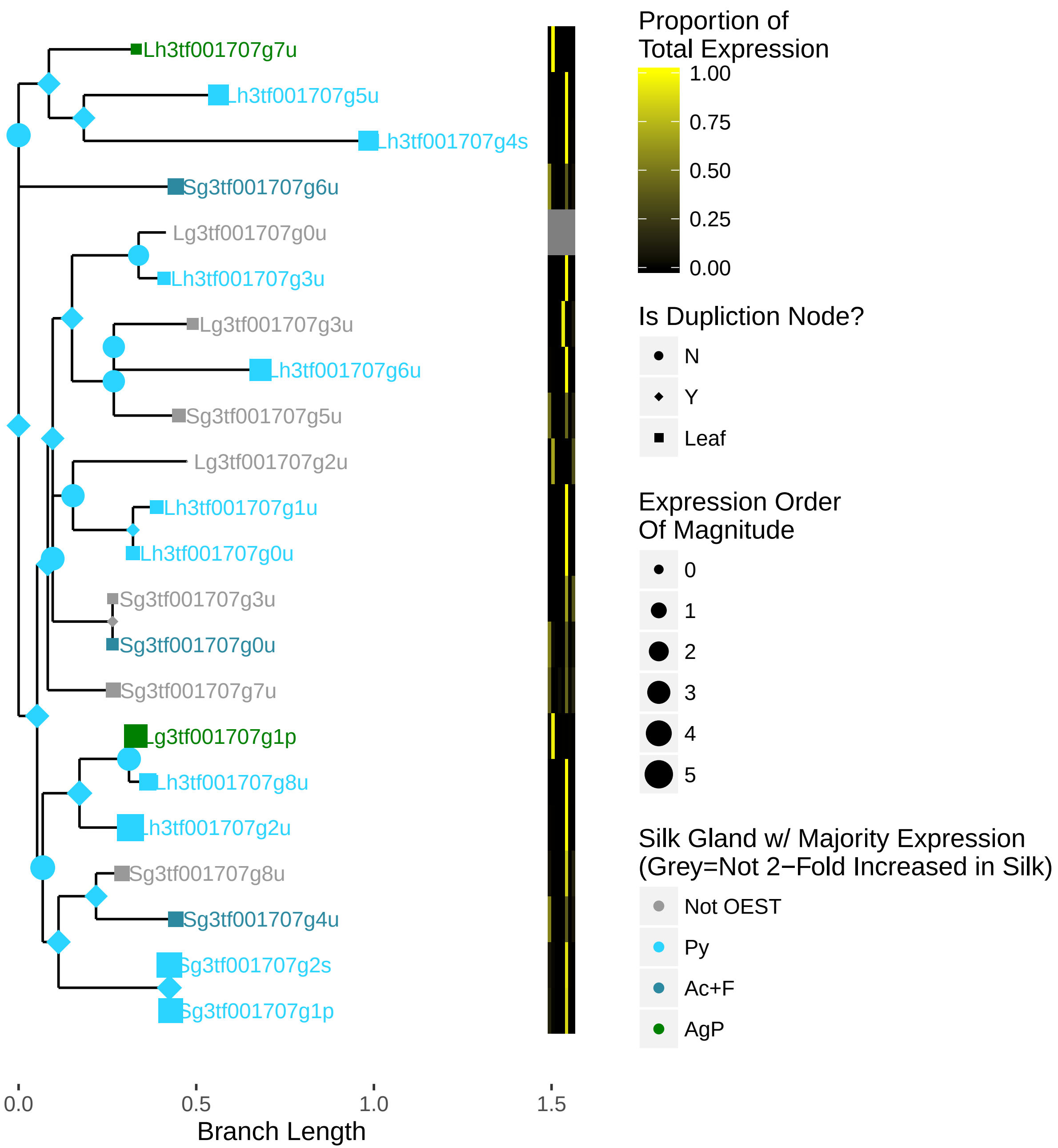

Lh3tf001707g7u
h3tf001707g5u
h3tf001707g4s
Sg3tf001707g6u
Lg3tf001707g0u
Lh3tf001707g3u
Lg3tf001707g3u
h3tf001707g6u
Sg3tf001707g5u
Lg3tf001707g2u
Lh3tf001707g1u
Lh3tf001707g0u
Sg3tf001707g3u
Sg3tf001707g0u
Sg3tf001707g7u
g3tf001707g1p
h3tf001707g8u
h3tf001707g2u
Sg3tf001707g8u
Sg3tf001707g4u
g3tf001707g2s
g3tf001707g1p
0.0
0.5
1.0
1.5
Branch Length
Proportion of Total Expression
1.00
0.75
0.50
0.25
0.00
Is Dupliction Node?
N
Y
Leaf
Expression Order Of Magnitude
0
1
2
3
4
5
Silk Gland w/ Majority Expression (Grey=Not 2−Fold Increased in Silk)
Not OEST
Py
Ac+F
AgP

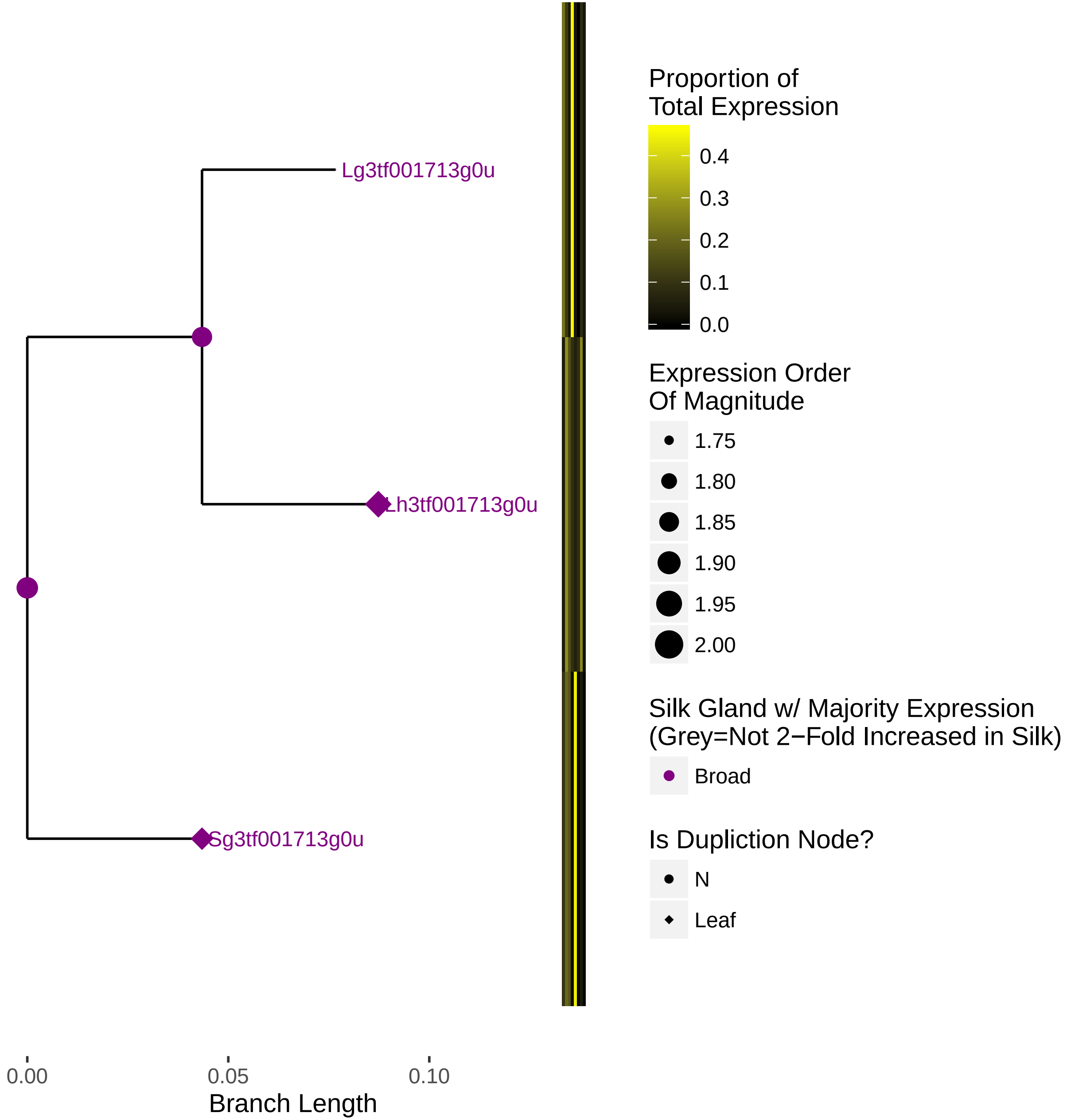

Lg3tf001713g0u
Lh3tf001713g0u
Sg3tf001713g0u
Proportion of
Total Expression
0.4
0.3
0.2
0.1
0.0
Expression Order
Of Magnitude
1.75
1.80
1.85
1.90
1.95
2.00
Silk Gland w/ Majority Expression
(Grey=Not 2−Fold Increased in Silk)
Broad
Is Dupliction Node?
N
Leaf
0.00
0.05
0.10
Branch Length

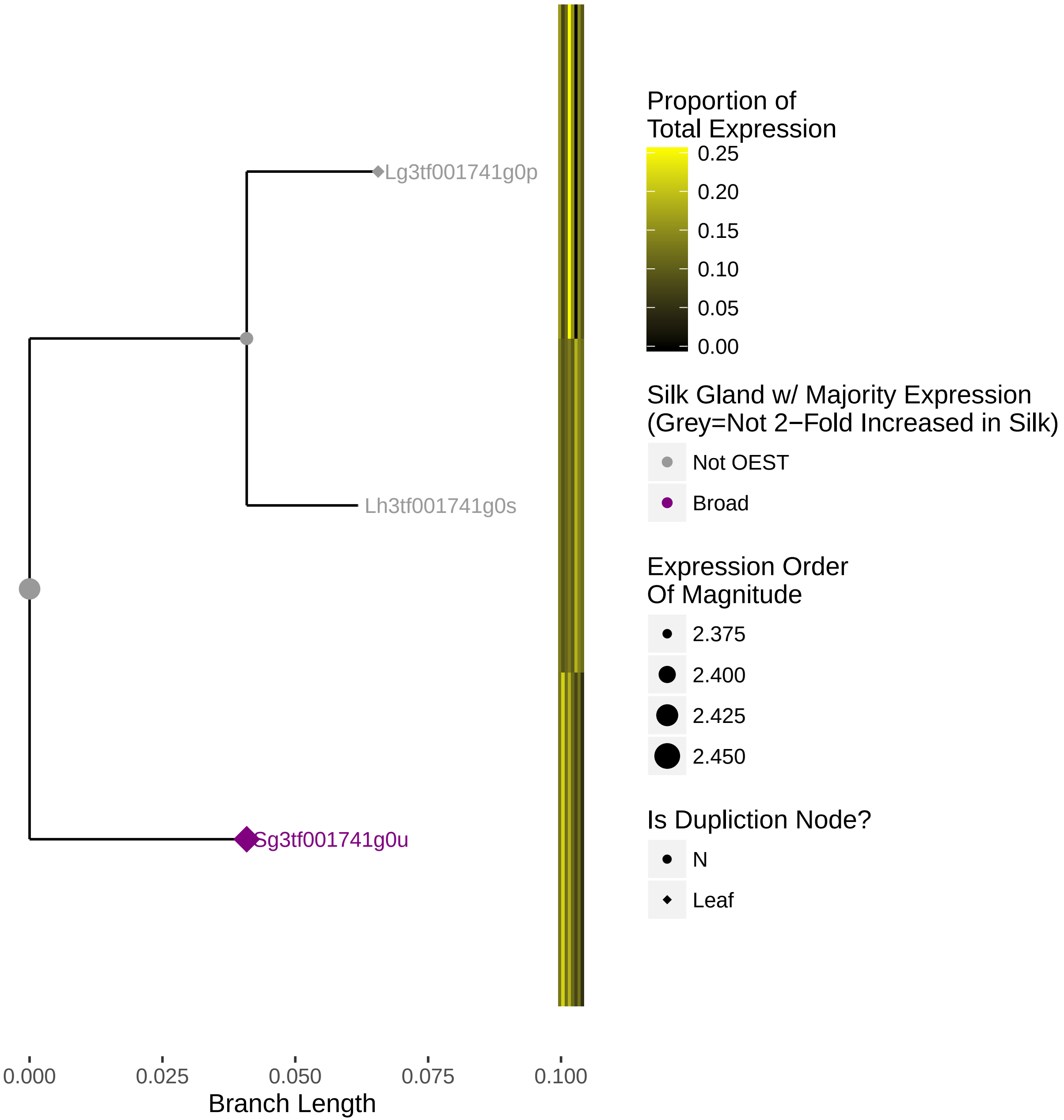

Lg3tf001741g0p
Lh3tf001741g0s
Sg3tf001741g0u
0.000
0.025
0.050
0.075
0.100
Branch Length
Proportion of Total Expression
0.25
0.20
0.15
0.10
0.05
0.00
Silk Gland w/ Majority Expression (Grey=Not 2−Fold Increased in Silk)
Not OEST
Broad
Expression Order Of Magnitude
2.375
2.400
2.425
2.450
Is Dupliction Node?
N
Leaf

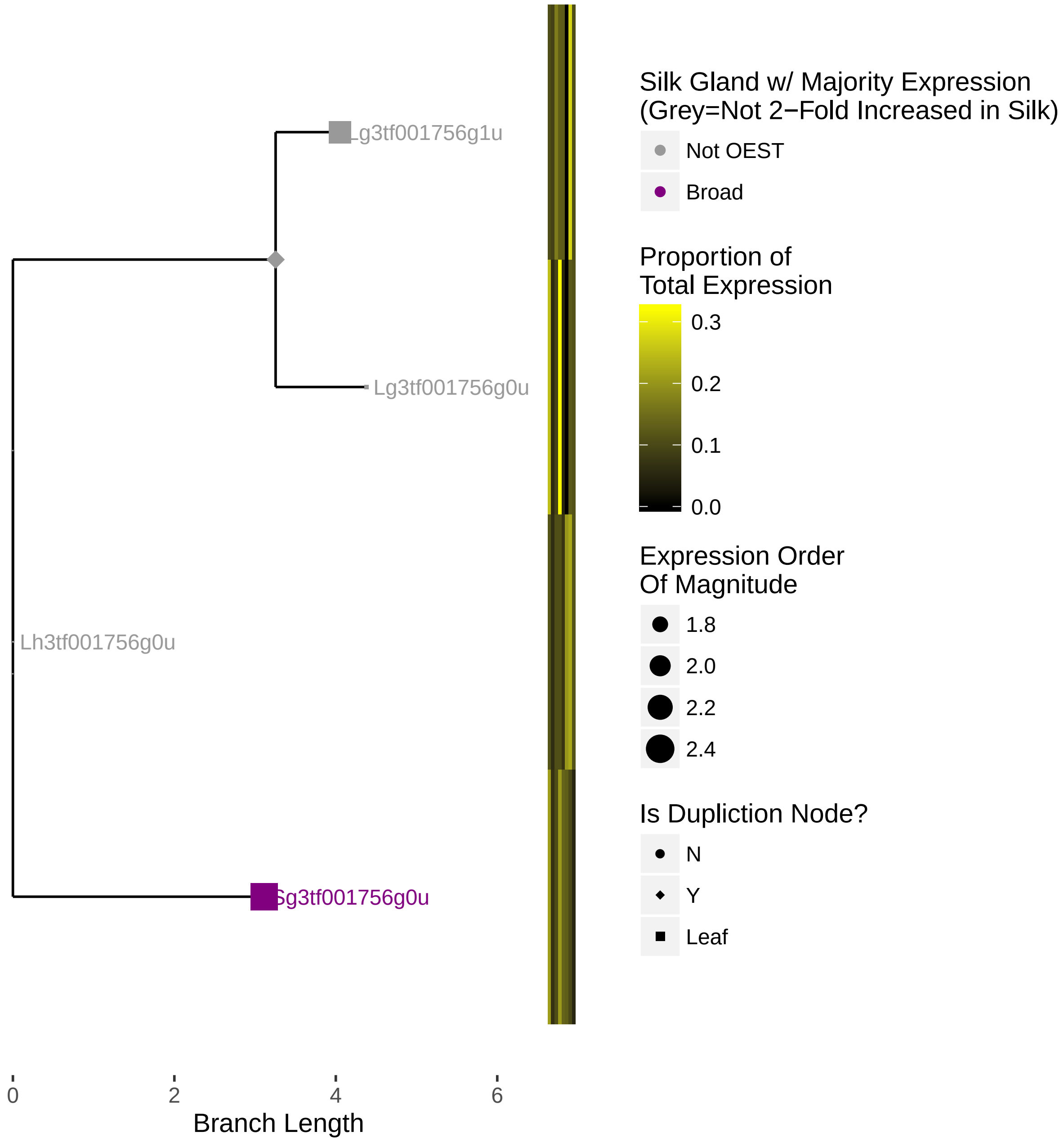

Lg3tf001756g1u
Lg3tf001756g0u
Lh3tf001756g0u
Sg3tf001756g0u
0
2
4
6
Branch Length
Silk Gland w/ Majority Expression
(Grey=Not 2−Fold Increased in Silk)
Not OEST
Broad
Proportion of
Total Expression
0.3
0.2
0.1
0.0
Expression Order
Of Magnitude
1.8
2.0
2.2
2.4
Is Dupliction Node?
N
Y
Leaf

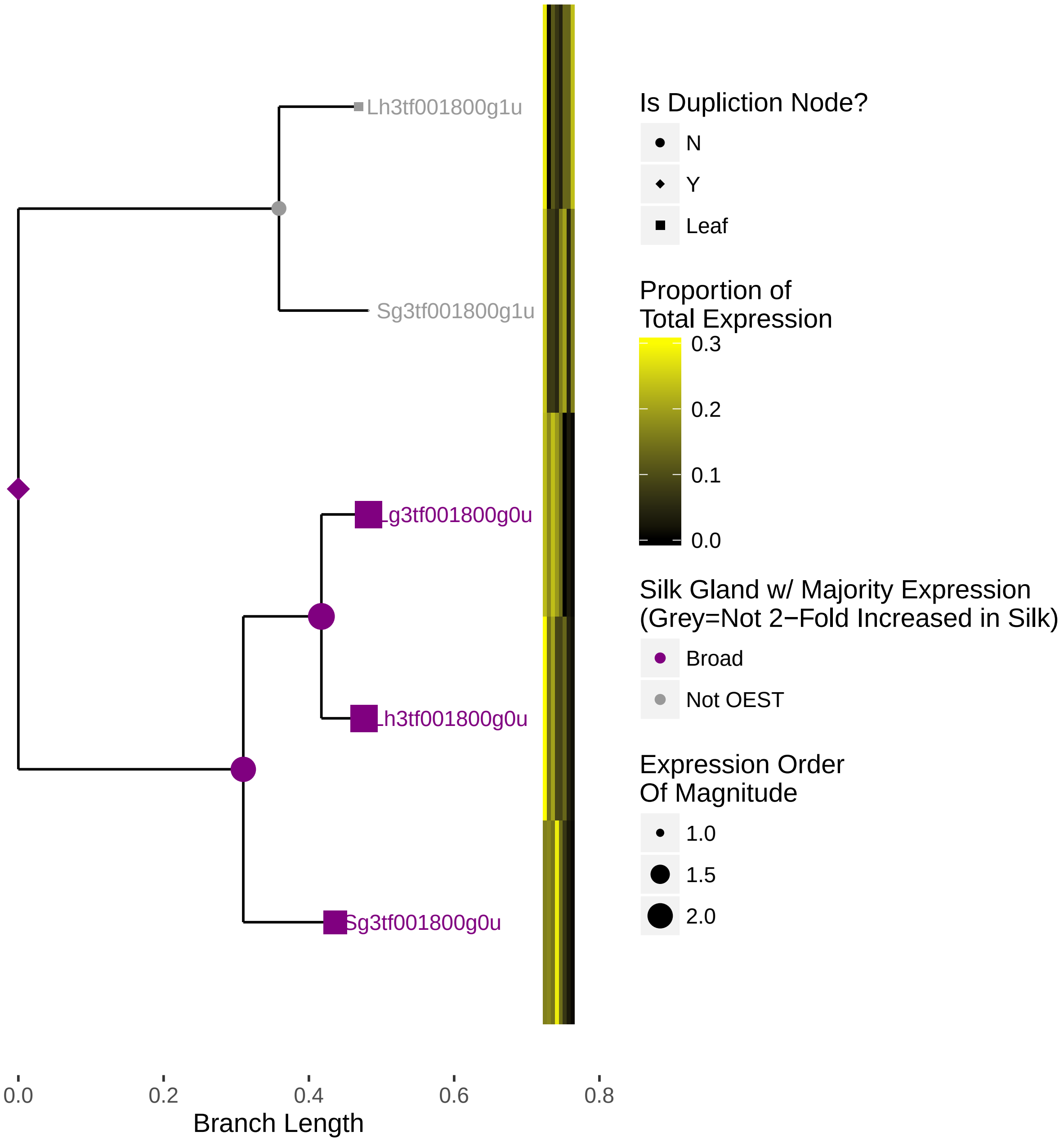

Lh3tf001800g1u
Sg3tf001800g1u
Lg3tf001800g0u
Lh3tf001800g0u
Sg3tf001800g0u
0.0
0.2
0.4
0.6
0.8
Branch Length
Is Dupliction Node?
N
Y
Leaf
Proportion of Total Expression
0.3
0.2
0.1
0.0
Silk Gland w/ Majority Expression (Grey=Not 2−Fold Increased in Silk)
Broad
Not OEST
Expression Order Of Magnitude
1.0
1.5
2.0

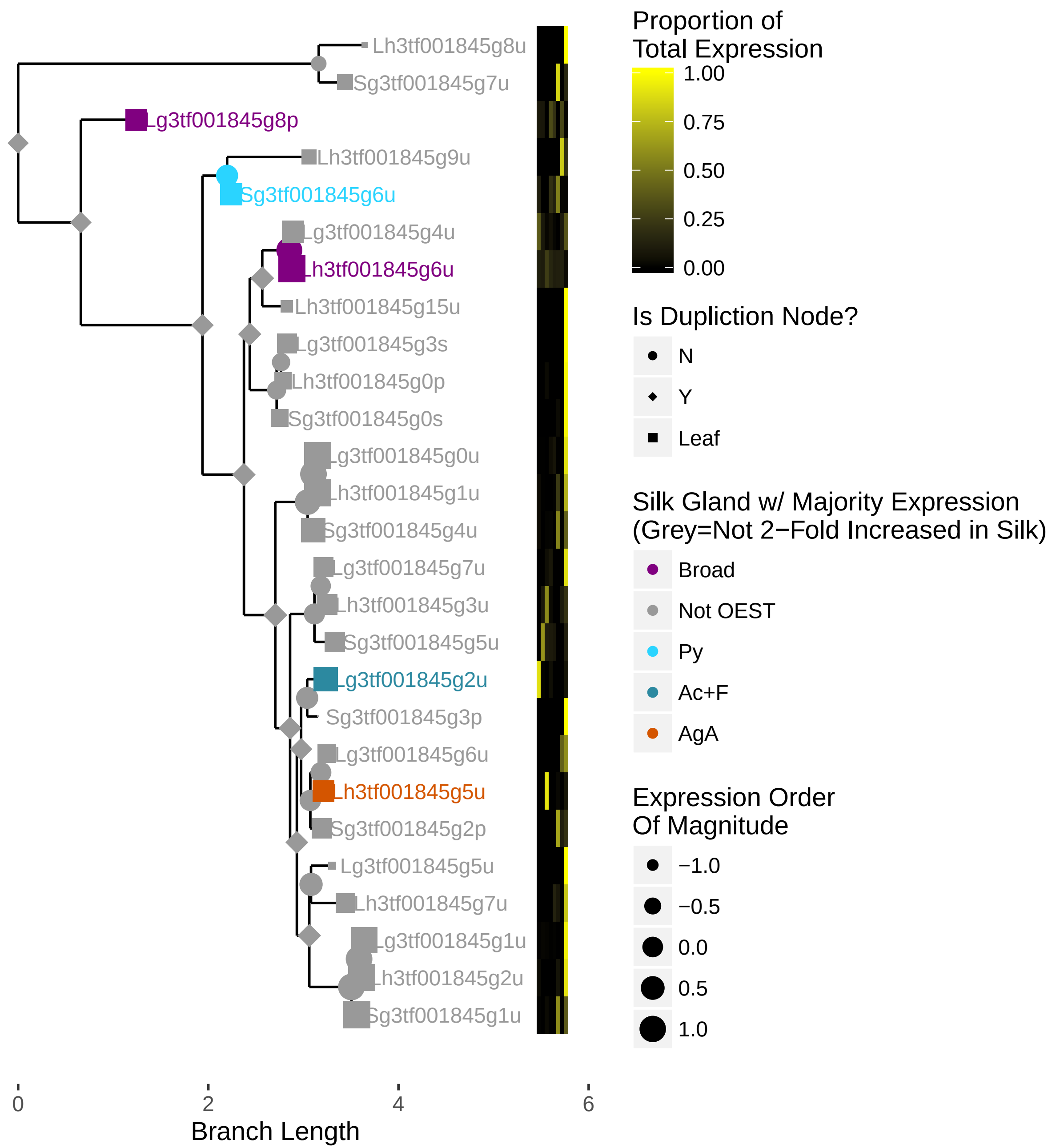

Lh3tf001845g8u
Sg3tf001845g7u
Lg3tf001845g8p
Lh3tf001845g9u
Sg3tf001845g6u
Lg3tf001845g4u
Lh3tf001845g6u
Lh3tf001845g15u
Lg3tf001845g3s
Lh3tf001845g0p
Sg3tf001845g0s
Lg3tf001845g0u
Lh3tf001845g1u
Sg3tf001845g4u
Lg3tf001845g7u
Lh3tf001845g3u
Sg3tf001845g5u
Lg3tf001845g2u
Sg3tf001845g3p
Lg3tf001845g6u
Lh3tf001845g5u
Sg3tf001845g2p
Lg3tf001845g5u
Lh3tf001845g7u
Lg3tf001845g1u
Lh3tf001845g2u
Sg3tf001845g1u
0
2
4
6
Branch Length
Proportion of Total Expression
1.00
0.75
0.50
0.25
0.00
Is Dupliction Node?
N
Y
Leaf
Silk Gland w/ Majority Expression (Grey=Not 2−Fold Increased in Silk)
Broad
Not OEST
Py
Ac+F
AgA
Expression Order Of Magnitude
−1.0
−0.5
0.0
0.5
1.0

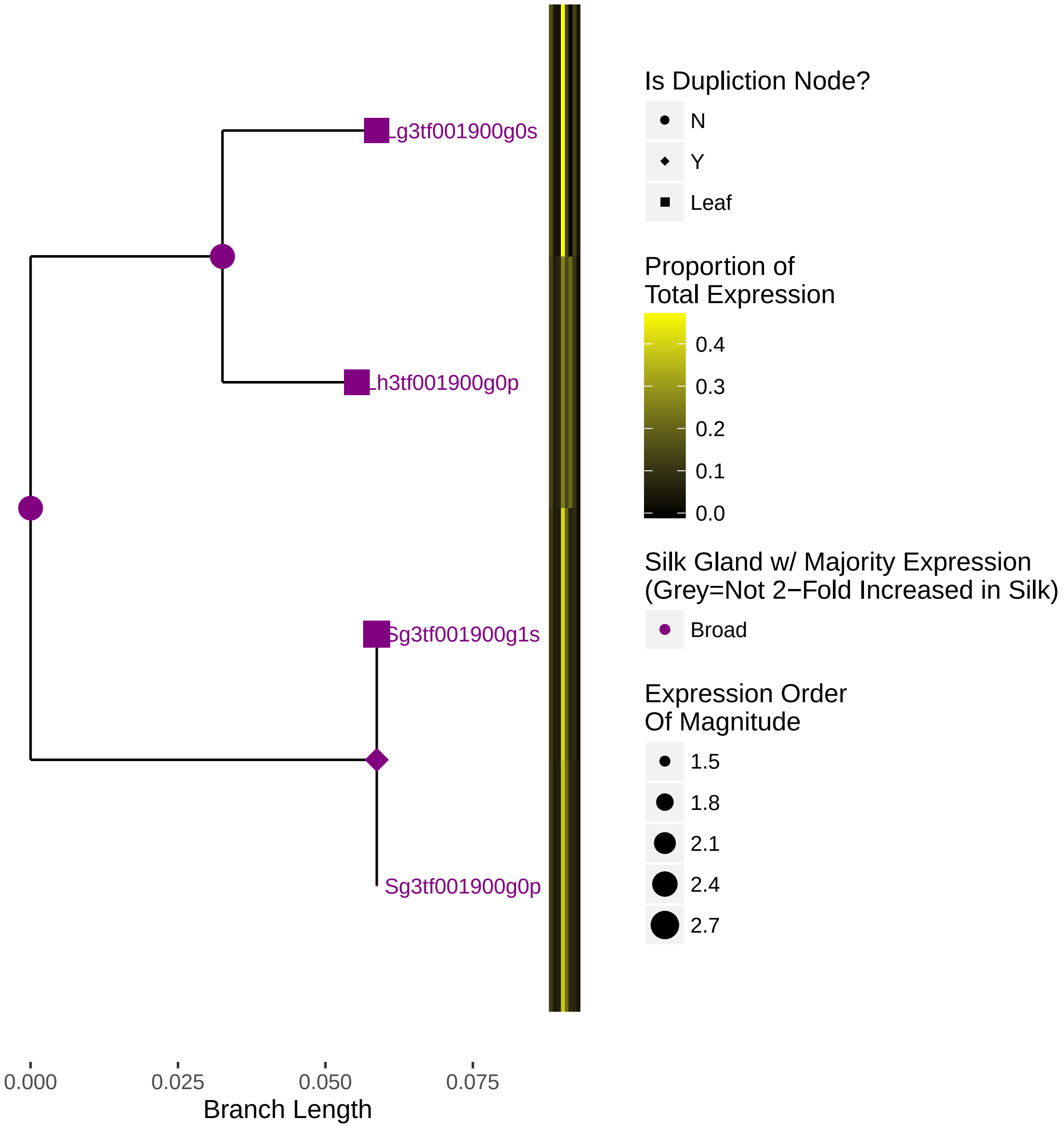
Lg3tf001900g0s
Lh3tf001900g0p
Sg3tf001900g1s
Sg3tf001900g0p
0.000
0.025
0.050
0.075
Branch Length
Is Dupliction Node?
N
Y
Leaf
Proportion of
Total Expression
0.4
0.3
0.2
0.1
0.0
Silk Gland w/ Majority Expression
(Grey=Not 2−Fold Increased in Silk)
Broad
Expression Order
Of Magnitude
1.5
1.8
2.1
2.4
2.7

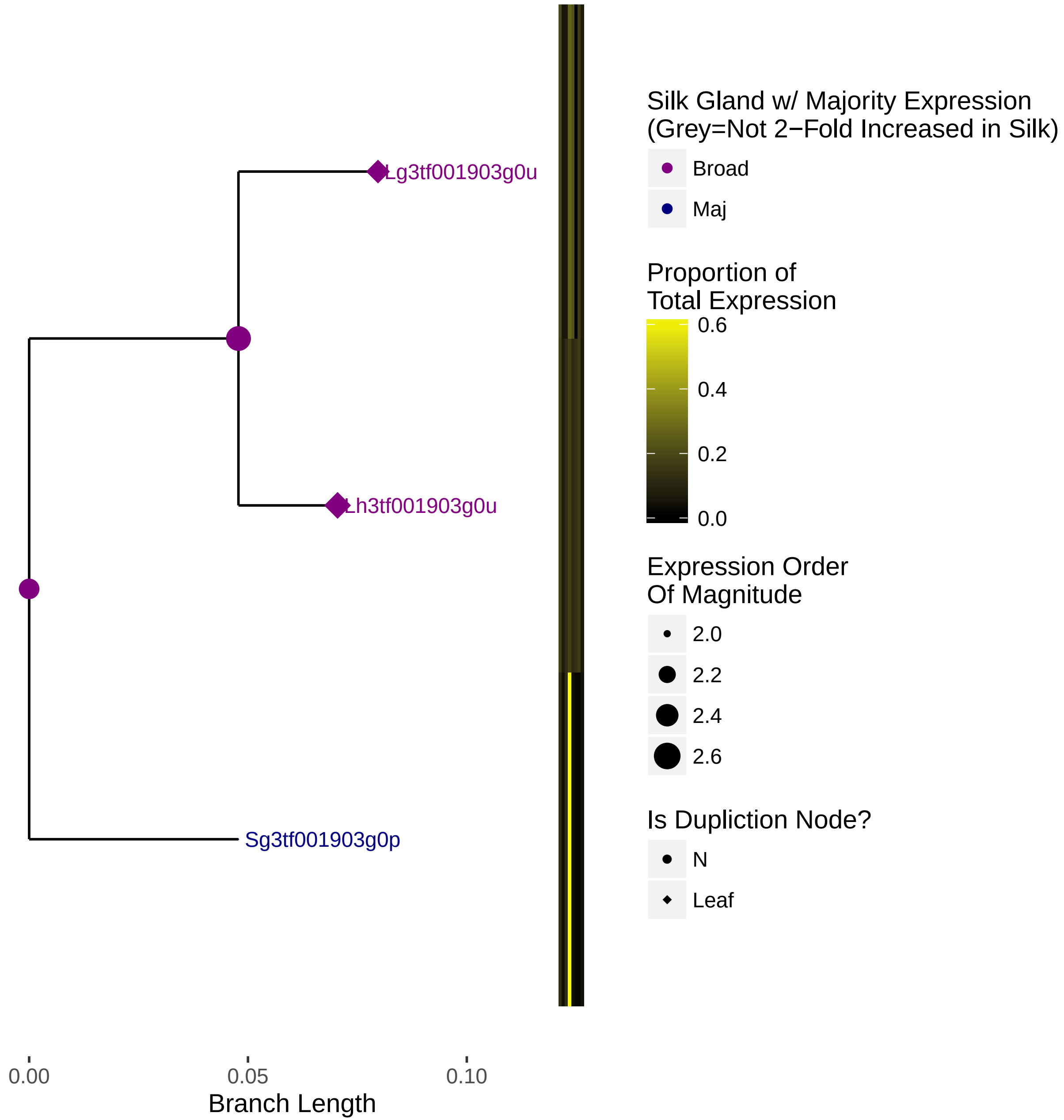
Lg3tf001903g0u
Lh3tf001903g0u
Sg3tf001903g0p
0.00
0.05
0.10
Branch Length
Silk Gland w/ Majority Expression
(Grey=Not 2−Fold Increased in Silk)
Broad
Maj
Proportion of
Total Expression
0.6
0.4
0.2
0.0
Expression Order
Of Magnitude
2.0
2.2
2.4
2.6
Is Dupliction Node?
N
Leaf

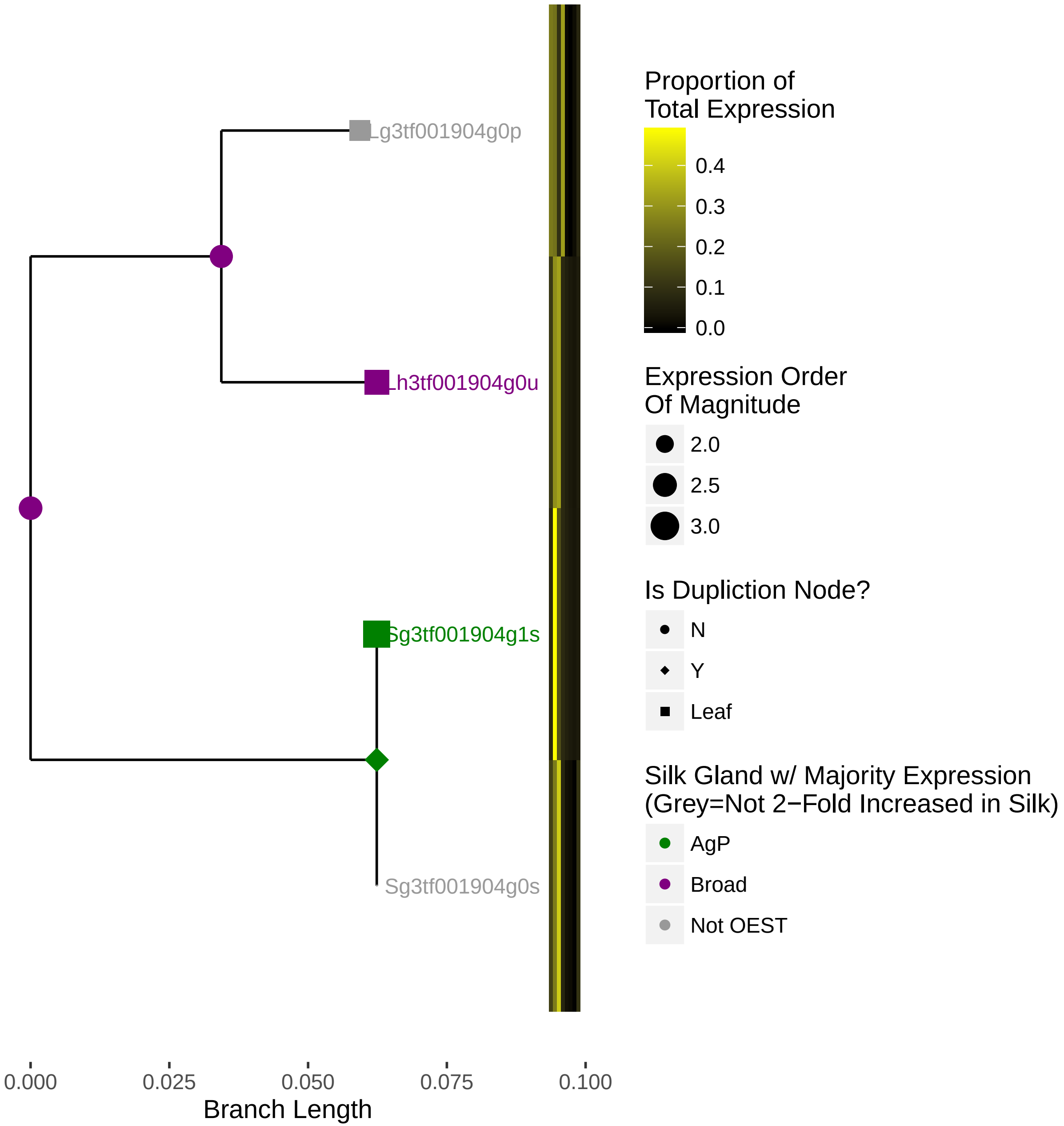

Lg3tf001904g0p
Lh3tf001904g0u
Sg3tf001904g1s
Sg3tf001904g0s
0.000
0.025
0.050
0.075
0.100
Branch Length
Proportion of
Total Expression
0.4
0.3
0.2
0.1
0.0
Expression Order
Of Magnitude
2.0
2.5
3.0
Is Dupliction Node?
N
Y
Leaf
Silk Gland w/ Majority Expression
(Grey=Not 2−Fold Increased in Silk)
AgP
Broad
Not OEST

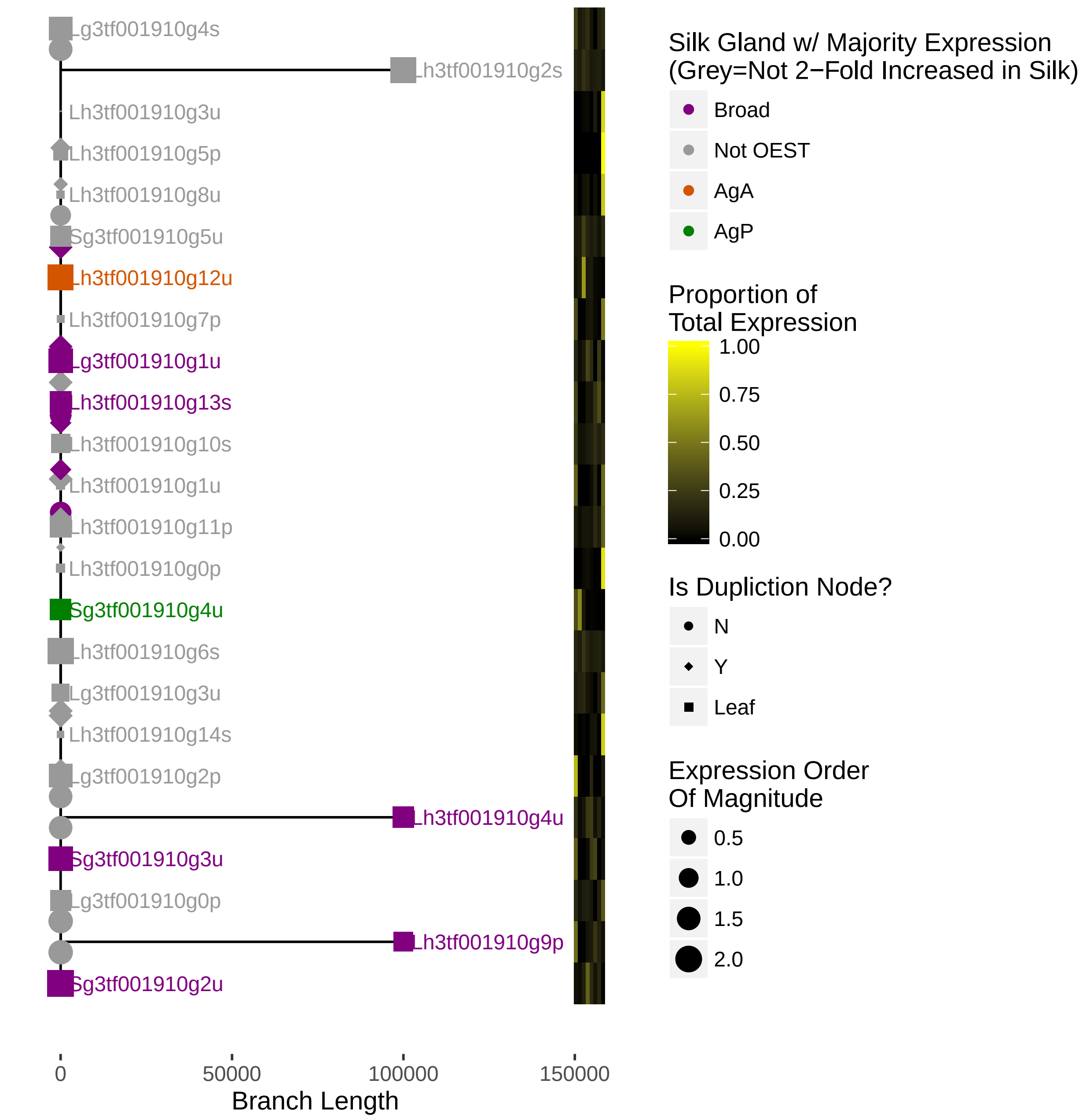

Lg3tf001910g4s
Lh3tf001910g2s
Lh3tf001910g3u
Lh3tf001910g5p
Lh3tf001910g8u
Sg3tf001910g5u
Lh3tf001910g12u
Lh3tf001910g7p
Lg3tf001910g1u
Lh3tf001910g13s
Lh3tf001910g10s
Lh3tf001910g1u
Lh3tf001910g11p
Lh3tf001910g0p
Sg3tf001910g4u
Lh3tf001910g6s
Lg3tf001910g3u
Lh3tf001910g14s
Lg3tf001910g2p
Lh3tf001910g4u
Sg3tf001910g3u
Lg3tf001910g0p
Lh3tf001910g9p
Sg3tf001910g2u
0
50000
100000
150000
Branch Length
Silk Gland w/ Majority Expression
(Grey=Not 2−Fold Increased in Silk)
Broad
Not OEST
AgA
AgP
Proportion of
Total Expression
1.00
0.75
0.50
0.25
0.00
Is Dupliction Node?
N
Y
Leaf
Expression Order
Of Magnitude
0.5
1.0
1.5
2.0

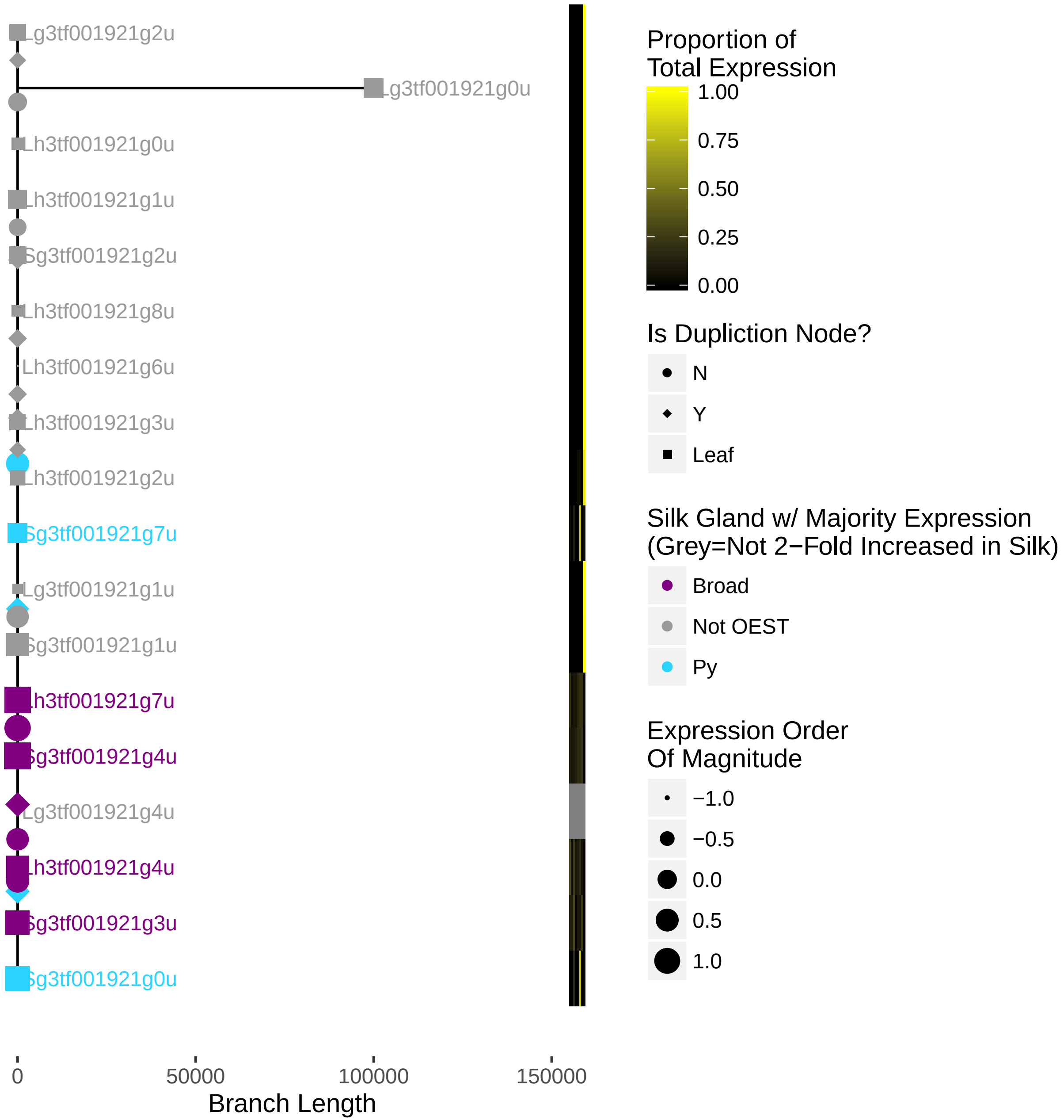

Lg3tf001921g2u
Lg3tf001921g0u
Lh3tf001921g0u
Lh3tf001921g1u
Sg3tf001921g2u
Lh3tf001921g8u
Lh3tf001921g6u
Lh3tf001921g3u
Lh3tf001921g2u
Sg3tf001921g7u
Lg3tf001921g1u
Sg3tf001921g1u
h3tf001921g7u
g3tf001921g4u
Lg3tf001921g4u
h3tf001921g4u
g3tf001921g3u
g3tf001921g0u
0
50000
100000
150000
Branch Length
Proportion of
Total Expression
1.00
0.75
0.50
0.25
0.00
Is Dupliction Node?
N
Y
Leaf
Silk Gland w/ Majority Expression
(Grey=Not 2−Fold Increased in Silk)
Broad
Not OEST
Py
Expression Order
Of Magnitude
−1.0
−0.5
0.0
0.5
1.0

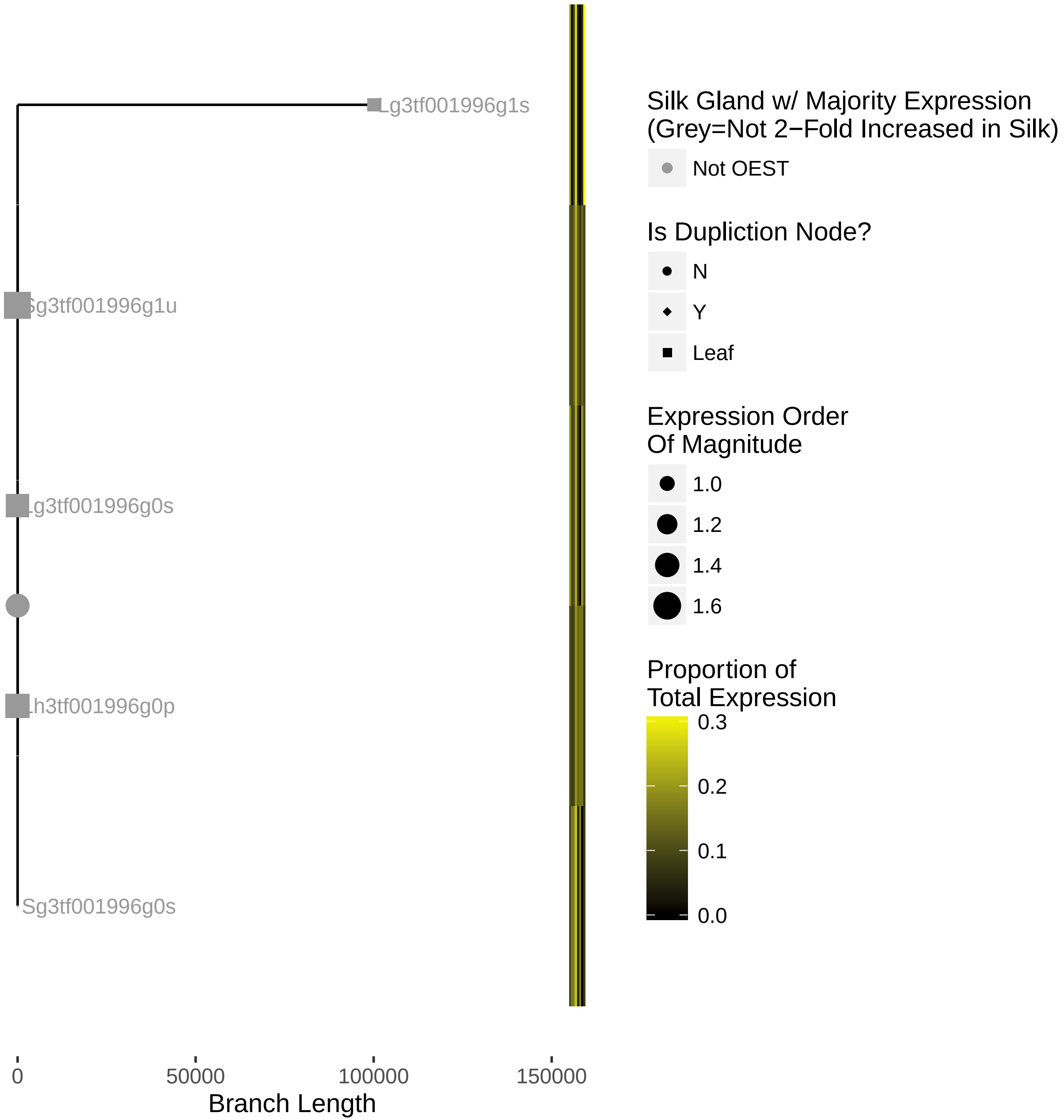
Lg3tf001996g1s
Sg3tf001996g1u
Lg3tf001996g0s
Lh3tf001996g0p
Sg3tf001996g0s
0
50000
100000
150000
Branch Length
Silk Gland w/ Majority Expression
(Grey=Not 2−Fold Increased in Silk)
Not OEST
Is Dupliction Node?
N
Y
Leaf
Expression Order
Of Magnitude
1.0
1.2
1.4
1.6
Proportion of
Total Expression
0.3
0.2
0.1
0.0

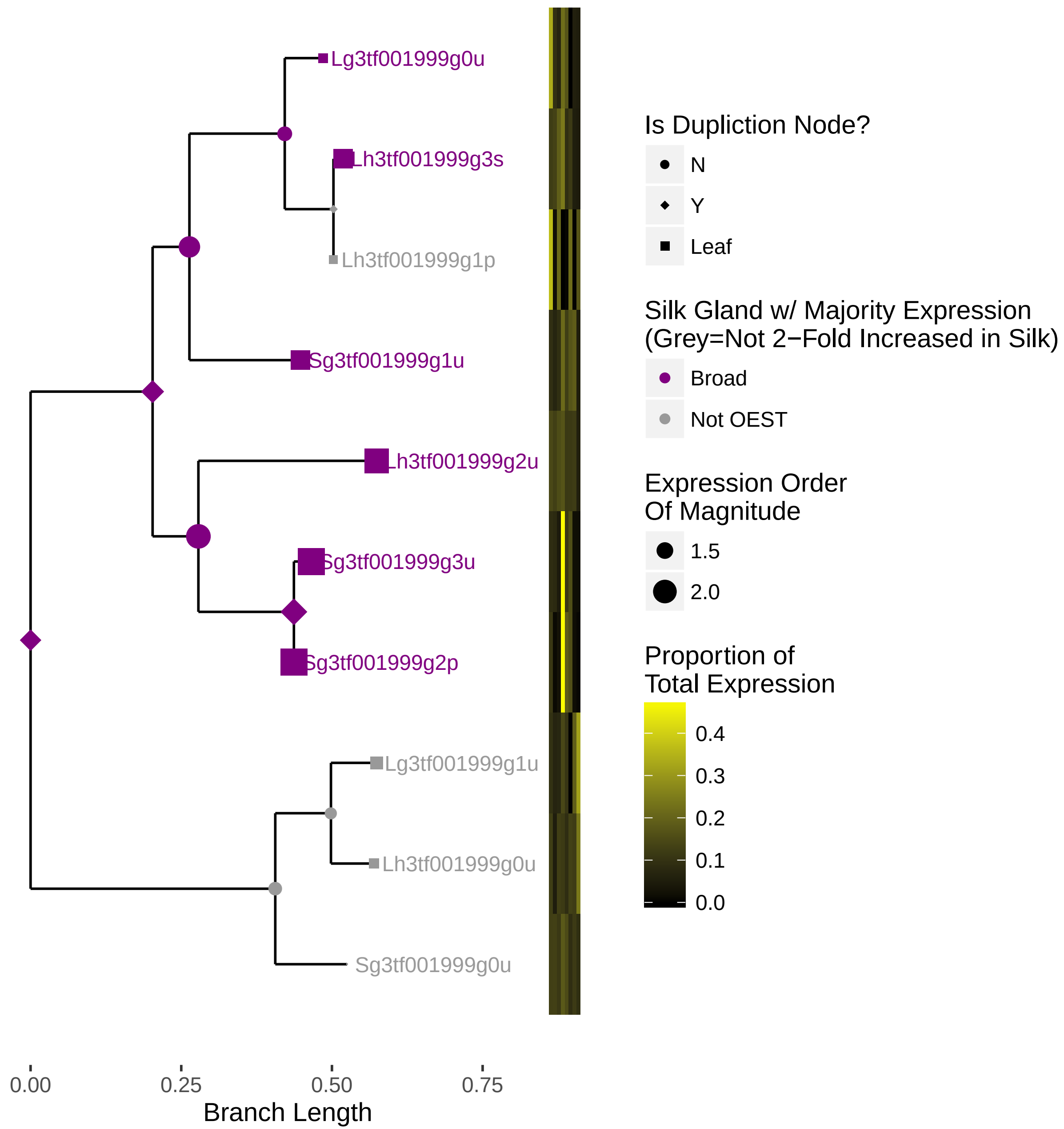

Lg3tf001999g0u
Lh3tf001999g3s
Lh3tf001999g1p
Sg3tf001999g1u
Lh3tf001999g2u
Sg3tf001999g3u
Sg3tf001999g2p
Lg3tf001999g1u
Lh3tf001999g0u
Sg3tf001999g0u
0.00
0.25
0.50
0.75
Branch Length
Is Dupliction Node?
N
Y
Leaf
Silk Gland w/ Majority Expression
(Grey=Not 2−Fold Increased in Silk)
Broad
Not OEST
Expression Order
Of Magnitude
1.5
2.0
Proportion of
Total Expression
0.4
0.3
0.2
0.1
0.0

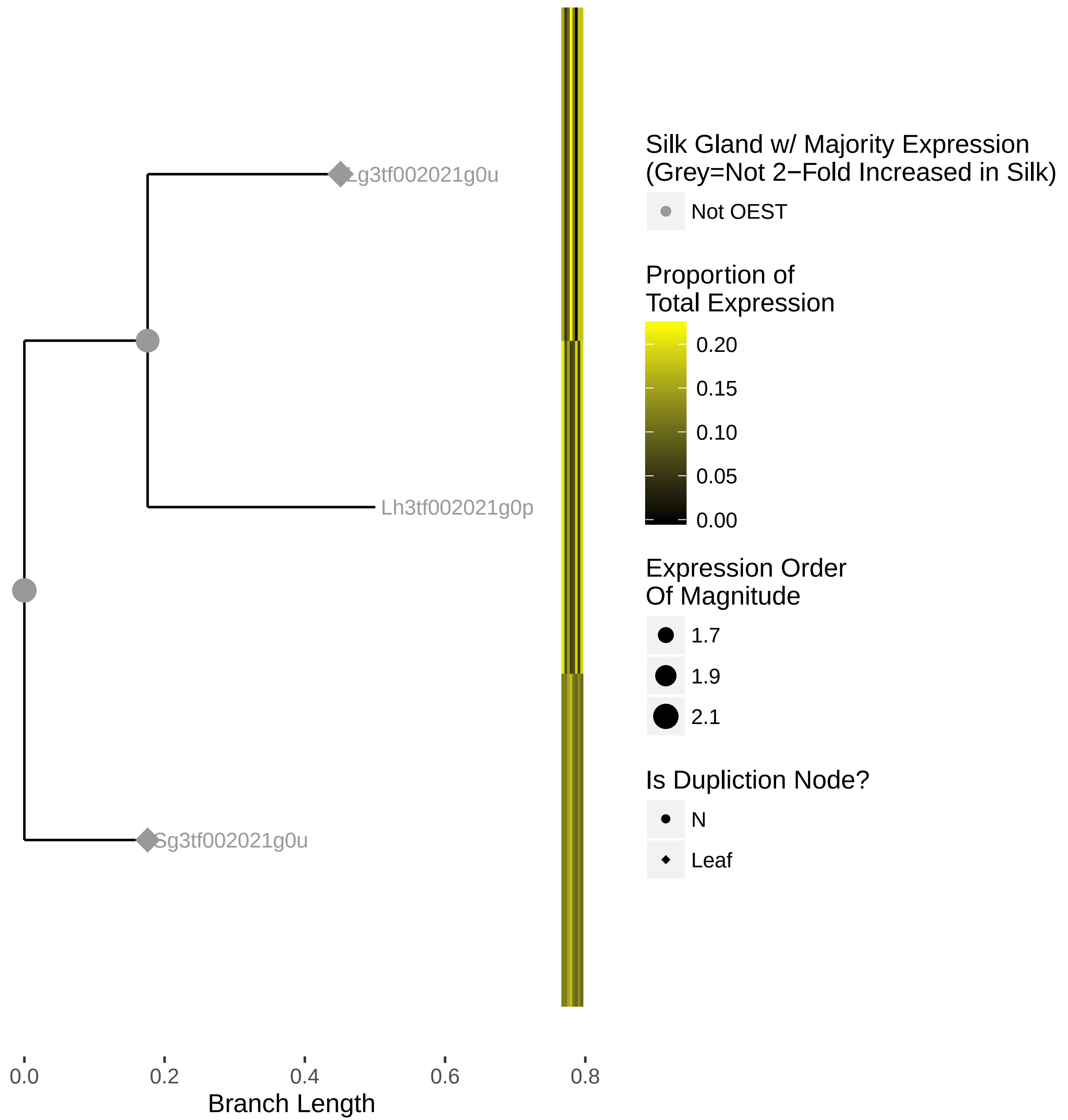

Lg3tf002021g0u
Lh3tf002021g0p
Sg3tf002021g0u
Silk Gland w/ Majority Expression
(Grey=Not 2−Fold Increased in Silk)
Not OEST
Proportion of
Total Expression
0.20
0.15
0.10
0.05
0.00
Expression Order
Of Magnitude
1.7
1.9
2.1
Is Dupliction Node?
N
Leaf
0.0
0.2
0.4
0.6
0.8
Branch Length

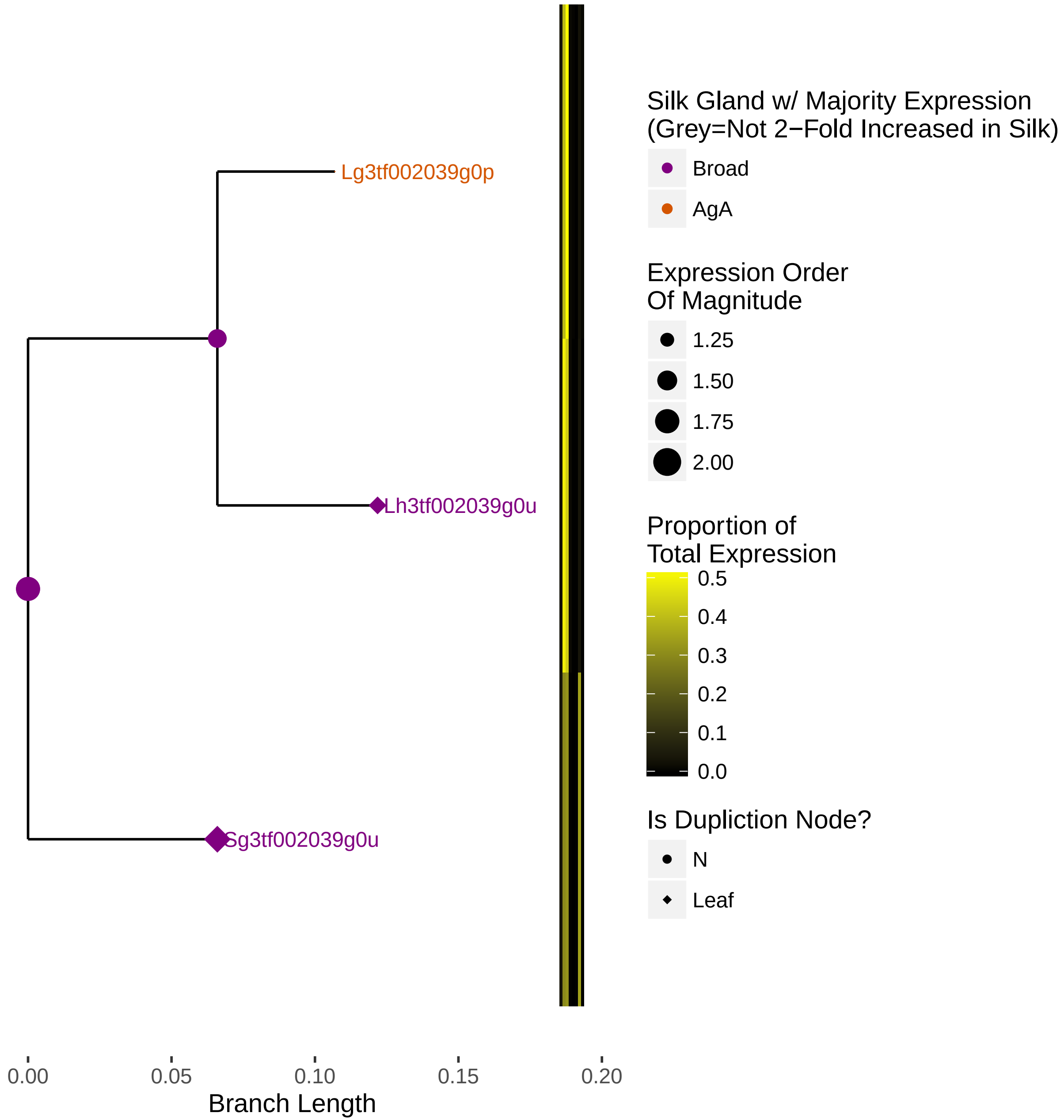
Lg3tf002039g0p
Lh3tf002039g0u
Sg3tf002039g0u
0.00
0.05
0.10
0.15
0.20
Branch Length
Silk Gland w/ Majority Expression
(Grey=Not 2−Fold Increased in Silk)
Broad
AgA
Expression Order
Of Magnitude
1.25
1.50
1.75
2.00
Proportion of
Total Expression
0.5
0.4
0.3
0.2
0.1
0.0
Is Dupliction Node?
N
Leaf

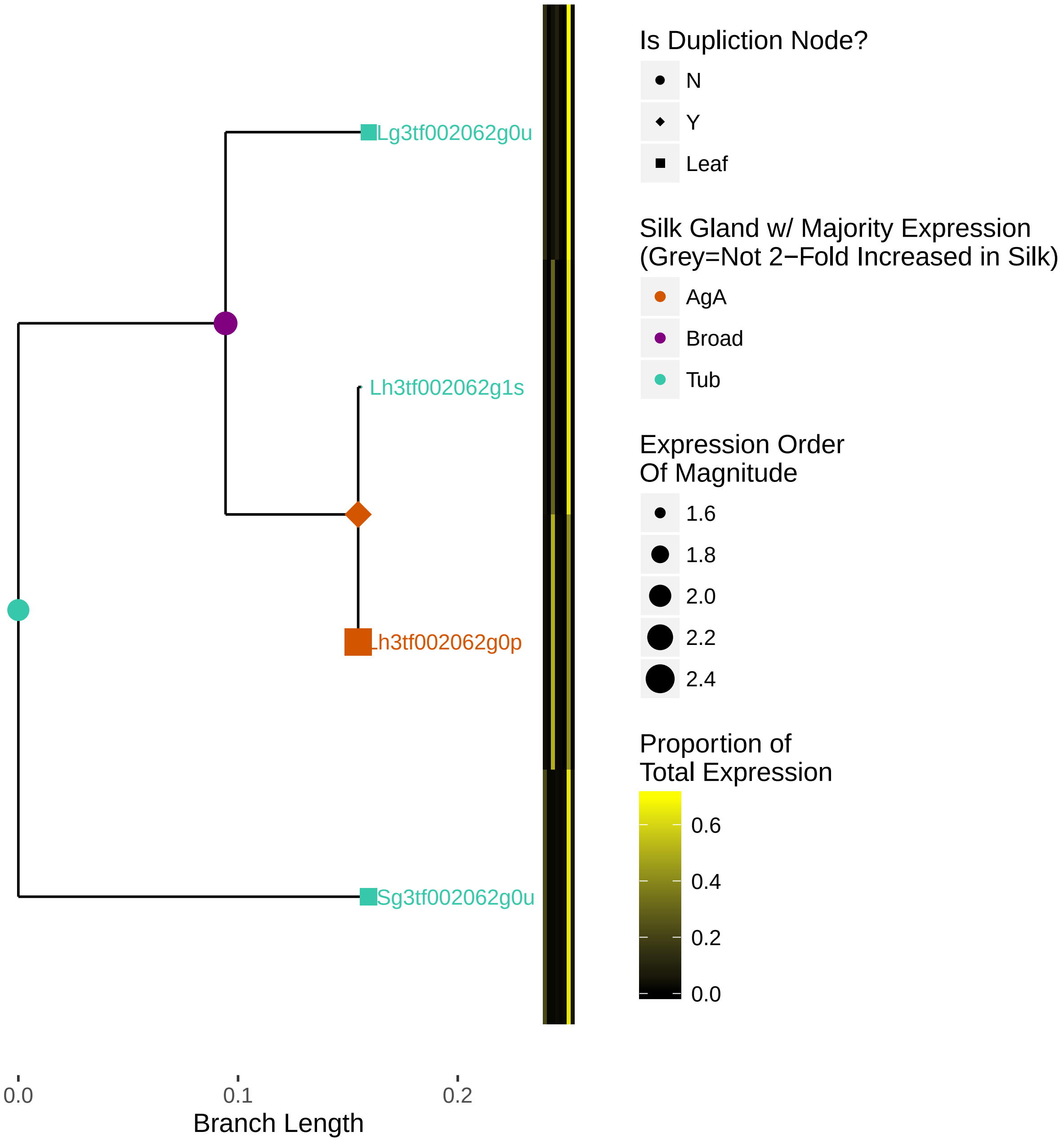

Lg3tf002062g0u
Lh3tf002062g1s
Lh3tf002062g0p
Sg3tf002062g0u
0.0
0.1
0.2
Branch Length
Is Dupliction Node?
N
Y
Leaf
Silk Gland w/ Majority Expression
(Grey=Not 2−Fold Increased in Silk)
AgA
Broad
Tub
Expression Order
Of Magnitude
1.6
1.8
2.0
2.2
2.4
Proportion of
Total Expression
0.6
0.4
0.2
0.0

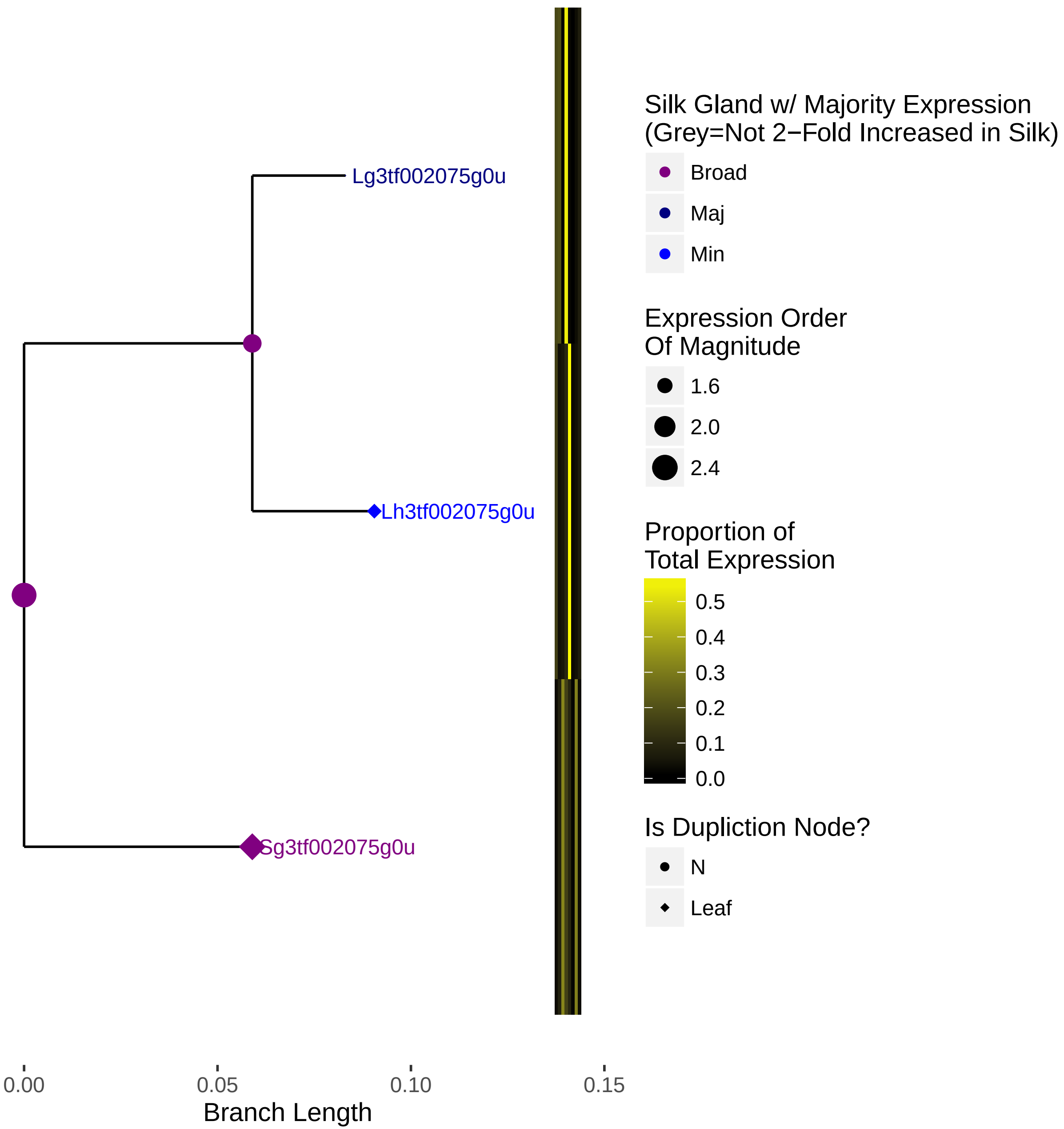

Lg3tf002075g0u
Lh3tf002075g0u
Sg3tf002075g0u
Silk Gland w/ Majority Expression
(Grey=Not 2−Fold Increased in Silk)
Broad
Maj
Min
Expression Order
Of Magnitude
1.6
2.0
2.4
Proportion of
Total Expression
0.5
0.4
0.3
0.2
0.1
0.0
Is Dupliction Node?
N
Leaf
0.00
0.05
0.10
0.15
Branch Length

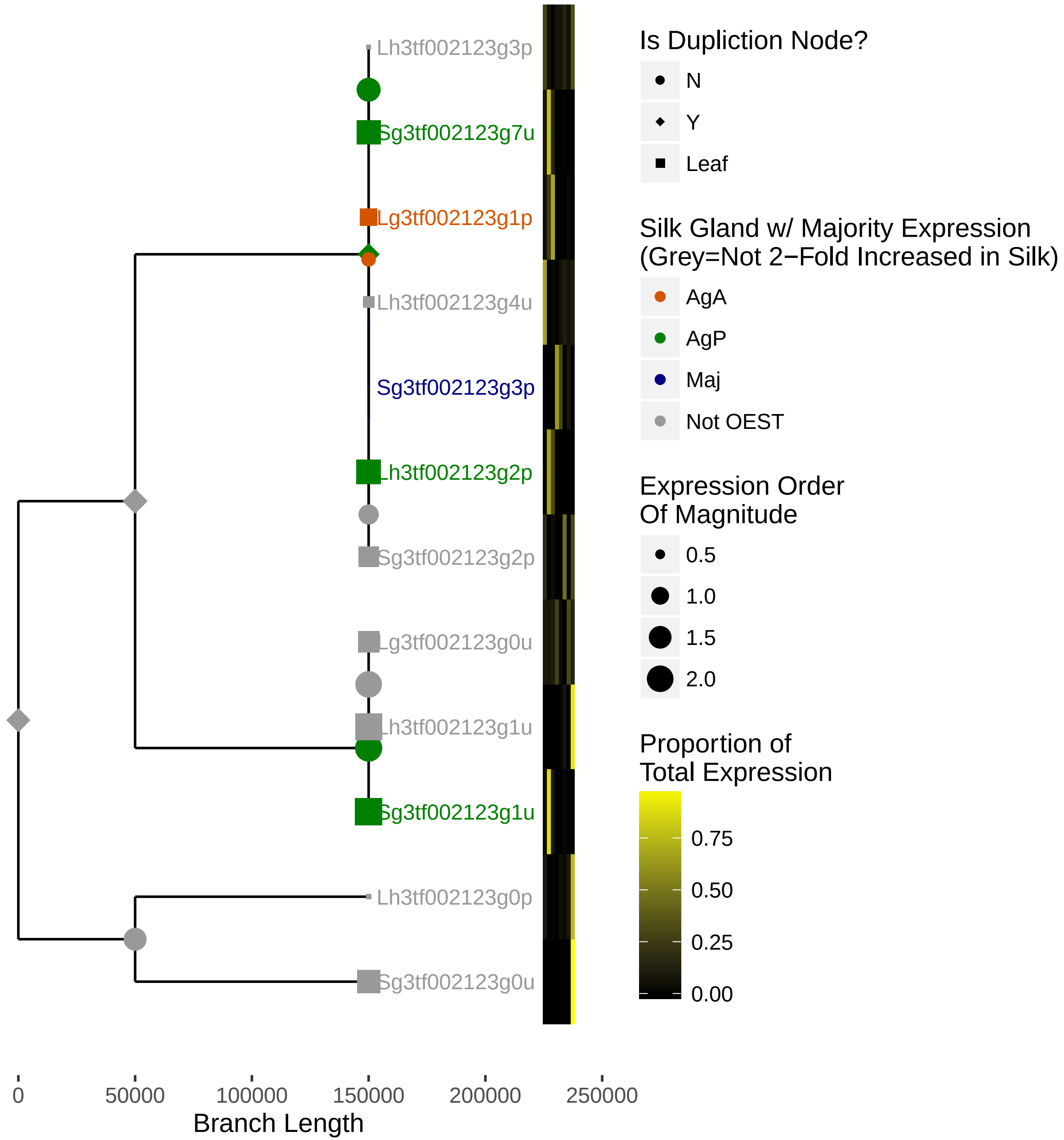

Lh3tf002123g3p
Sg3tf002123g7u
Lg3tf002123g1p
Lh3tf002123g4u
Sg3tf002123g3p
Lh3tf002123g2p
Sg3tf002123g2p
Lg3tf002123g0u
Lh3tf002123g1u
Sg3tf002123g1u
Lh3tf002123g0p
Sg3tf002123g0u
0
50000
100000
150000
200000
250000
Branch Length
Is Dupliction Node?
N
Y
Leaf
Silk Gland w/ Majority Expression
(Grey=Not 2−Fold Increased in Silk)
AgA
AgP
Maj
Not OEST
Expression Order
Of Magnitude
0.5
1.0
1.5
2.0
Proportion of
Total Expression
0.75
0.50
0.25
0.00

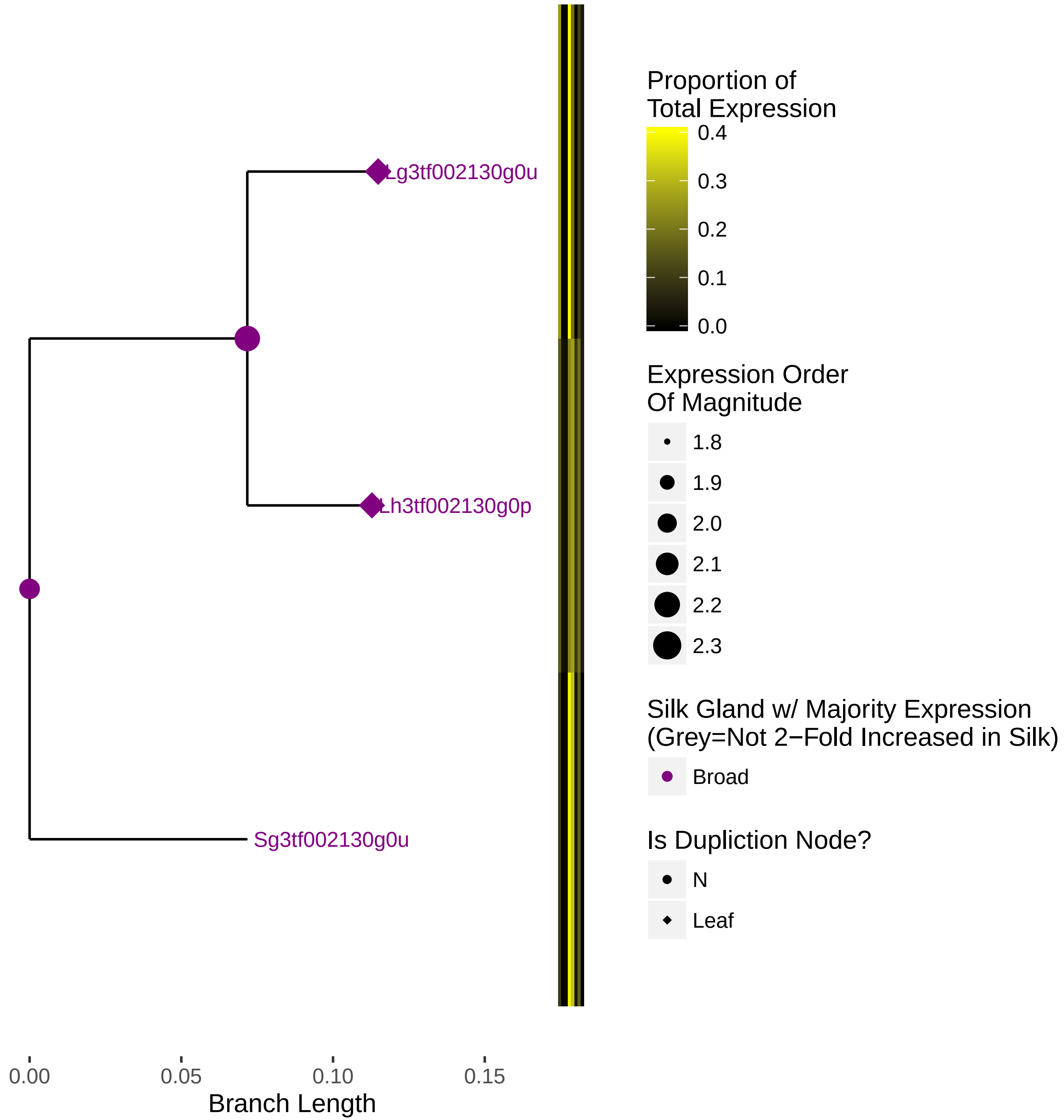

Lg3tf002130g0u
Lh3tf002130g0p
Sg3tf002130g0u
0.00
0.05
0.10
0.15
Branch Length
Proportion of Total Expression
0.4
0.3
0.2
0.1
0.0
Expression Order Of Magnitude
1.8
1.9
2.0
2.1
2.2
2.3
Silk Gland w/ Majority Expression (Grey=Not 2−Fold Increased in Silk)
Broad
Is Dupliction Node?
N
Leaf

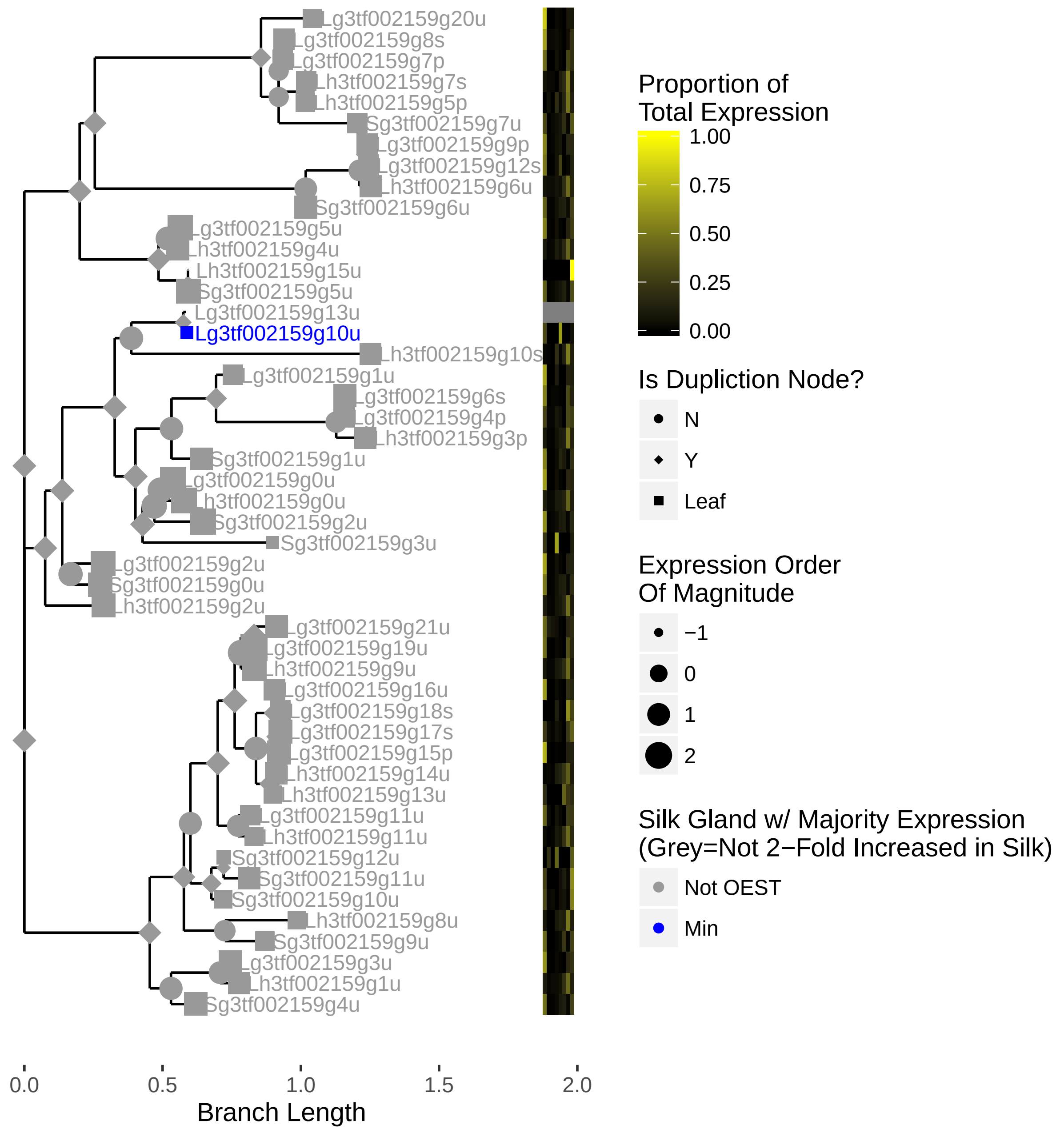

Lg3tf002159g20u
Lg3tf002159g8s
Lg3tf002159g7p
Lh3tf002159g7s
Lh3tf002159g5p
Sg3tf002159g7u
Lg3tf002159g9p
Lg3tf002159g12s
Lh3tf002159g6u
Sg3tf002159g6u
Lg3tf002159g5u
Lh3tf002159g4u
Lh3tf002159g15u
Sg3tf002159g5u
Lg3tf002159g13u
Lg3tf002159g10u
Lh3tf002159g10s
Lg3tf002159g1u
Lg3tf002159g6s
Lg3tf002159g4p
Lh3tf002159g3p
Sg3tf002159g1u
Lg3tf002159g0u
Lh3tf002159g0u
Sg3tf002159g2u
Sg3tf002159g3u
Lg3tf002159g2u
Sg3tf002159g0u
Lh3tf002159g2u
Lg3tf002159g21u
Lg3tf002159g19u
Lh3tf002159g9u
Lg3tf002159g16u
Lg3tf002159g18s
Lg3tf002159g17s
Lg3tf002159g15p
Lh3tf002159g14u
Lh3tf002159g13u
Lg3tf002159g11u
Lh3tf002159g11u
Sg3tf002159g12u
Sg3tf002159g11u
Sg3tf002159g10u
Lh3tf002159g8u
Sg3tf002159g9u
Lg3tf002159g3u
Lh3tf002159g1u
Sg3tf002159g4u
0.0
0.5
1.0
1.5
2.0
Branch Length
Proportion of
Total Expression
1.00
0.75
0.50
0.25
0.00
Is Dupliction Node?
N
Y
Leaf
Expression Order
Of Magnitude
−1
0
1
2
Silk Gland w/ Majority Expression
(Grey=Not 2−Fold Increased in Silk)
Not OEST
Min

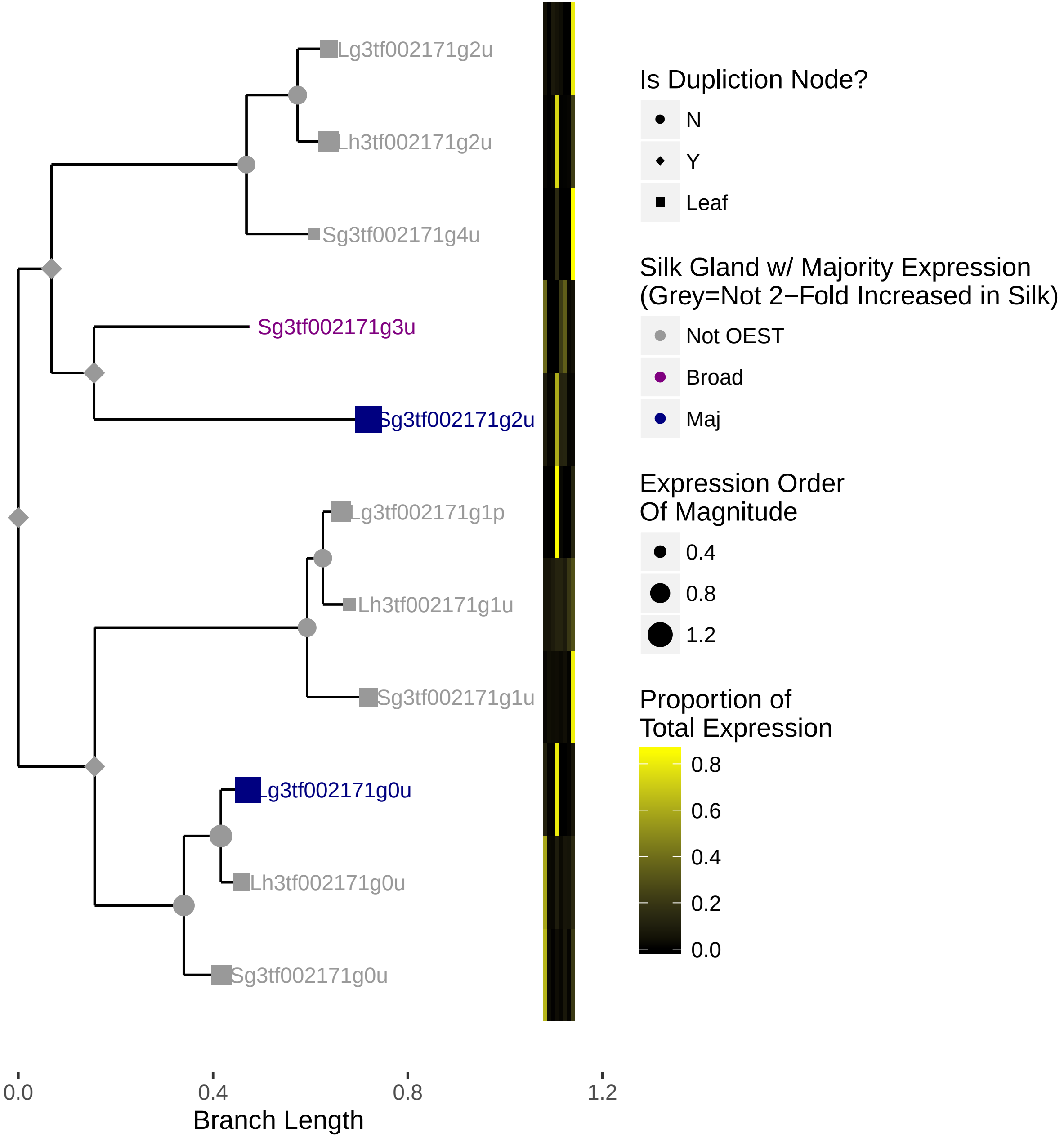

Lg3tf002171g2u
Lh3tf002171g2u
Sg3tf002171g4u
Sg3tf002171g3u
Sg3tf002171g2u
Lg3tf002171g1p
Lh3tf002171g1u
Sg3tf002171g1u
Lg3tf002171g0u
Lh3tf002171g0u
Sg3tf002171g0u
0.0
0.4
0.8
1.2
Branch Length
Is Dupliction Node?
N
Y
Leaf
Silk Gland w/ Majority Expression
(Grey=Not 2−Fold Increased in Silk)
Not OEST
Broad
Maj
Expression Order
Of Magnitude
0.4
0.8
1.2
Proportion of
Total Expression
0.8
0.6
0.4
0.2
0.0

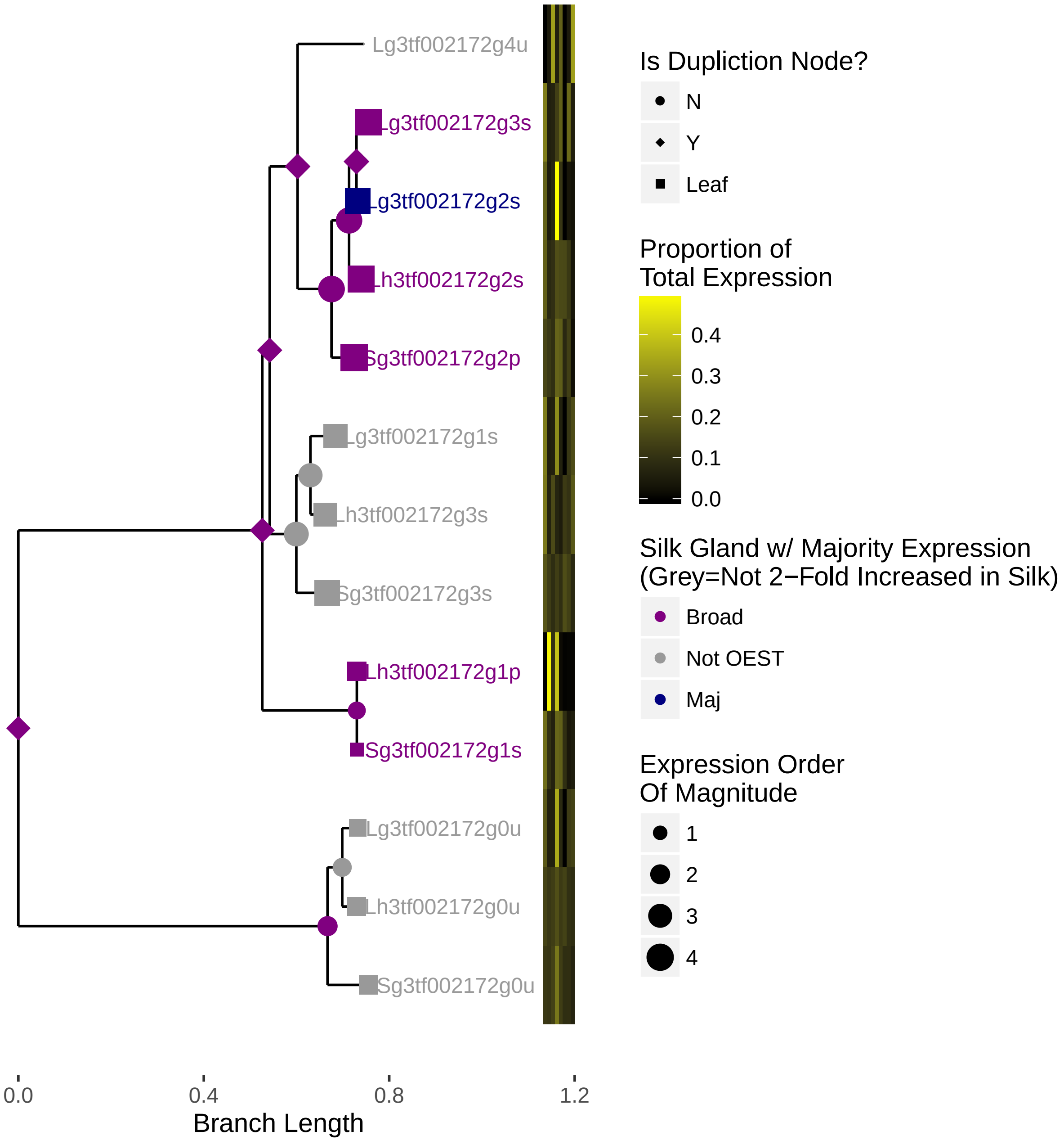

Lg3tf002172g4u
g3tf002172g3s
g3tf002172g2s
h3tf002172g2s
g3tf002172g2p
g3tf002172g1s
h3tf002172g3s
g3tf002172g3s
Lh3tf002172g1p
Sg3tf002172g1s
Lg3tf002172g0u
Lh3tf002172g0u
Sg3tf002172g0u
0.0
0.4
0.8
1.2
Branch Length
Is Dupliction Node?
N
Y
Leaf
Proportion of
Total Expression
0.4
0.3
0.2
0.1
0.0
Silk Gland w/ Majority Expression
(Grey=Not 2−Fold Increased in Silk)
Broad
Not OEST
Maj
Expression Order
Of Magnitude
1
2
3
4

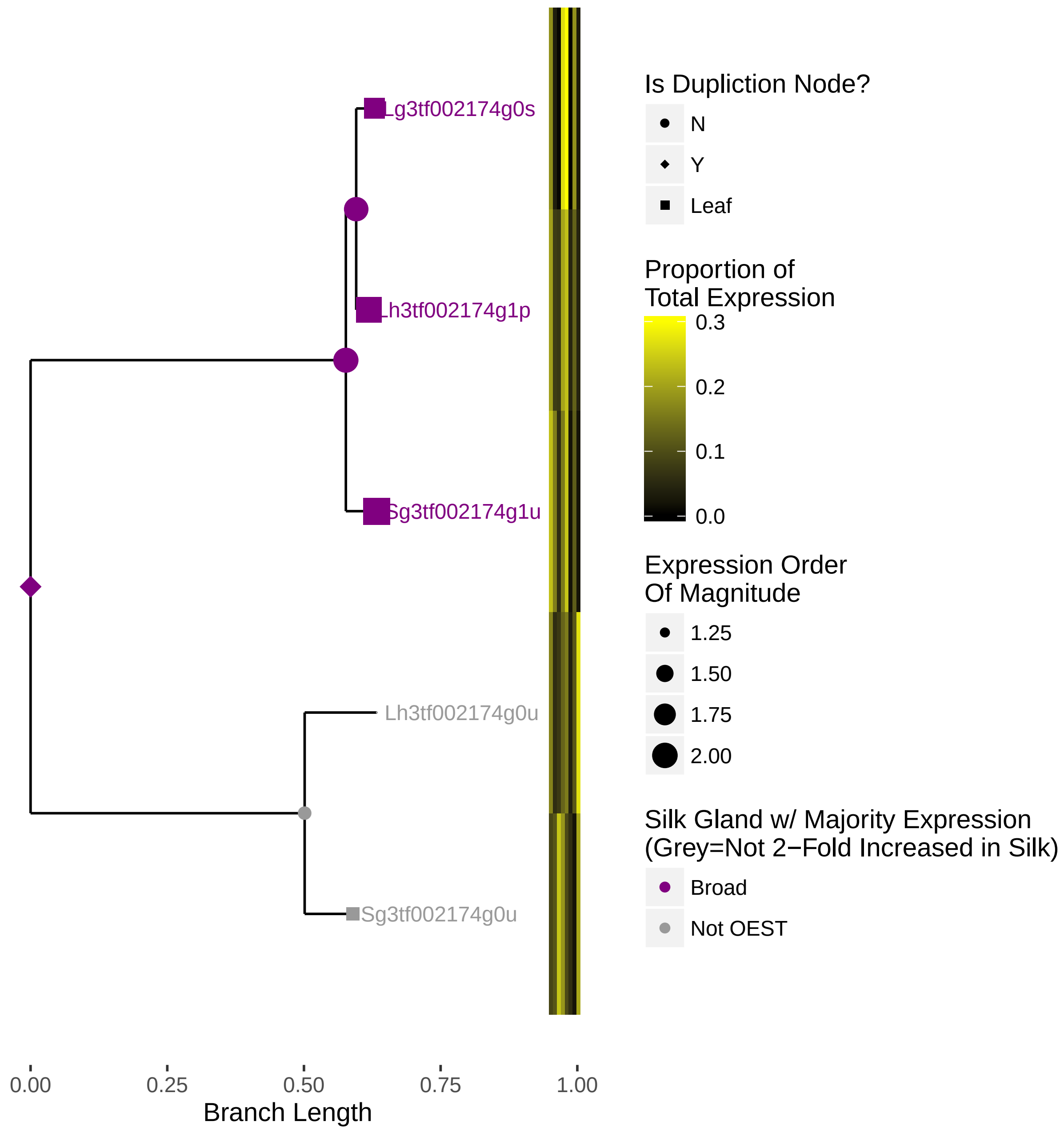

Lg3tf002174g0s
Lh3tf002174g1p
Sg3tf002174g1u
Lh3tf002174g0u
Sg3tf002174g0u
0.00
0.25
0.50
0.75
1.00
Branch Length
Is Dupliction Node?
N
Y
Leaf
Proportion of Total Expression
0.3
0.2
0.1
0.0
Expression Order Of Magnitude
1.25
1.50
1.75
2.00
Silk Gland w/ Majority Expression (Grey=Not 2−Fold Increased in Silk)
Broad
Not OEST

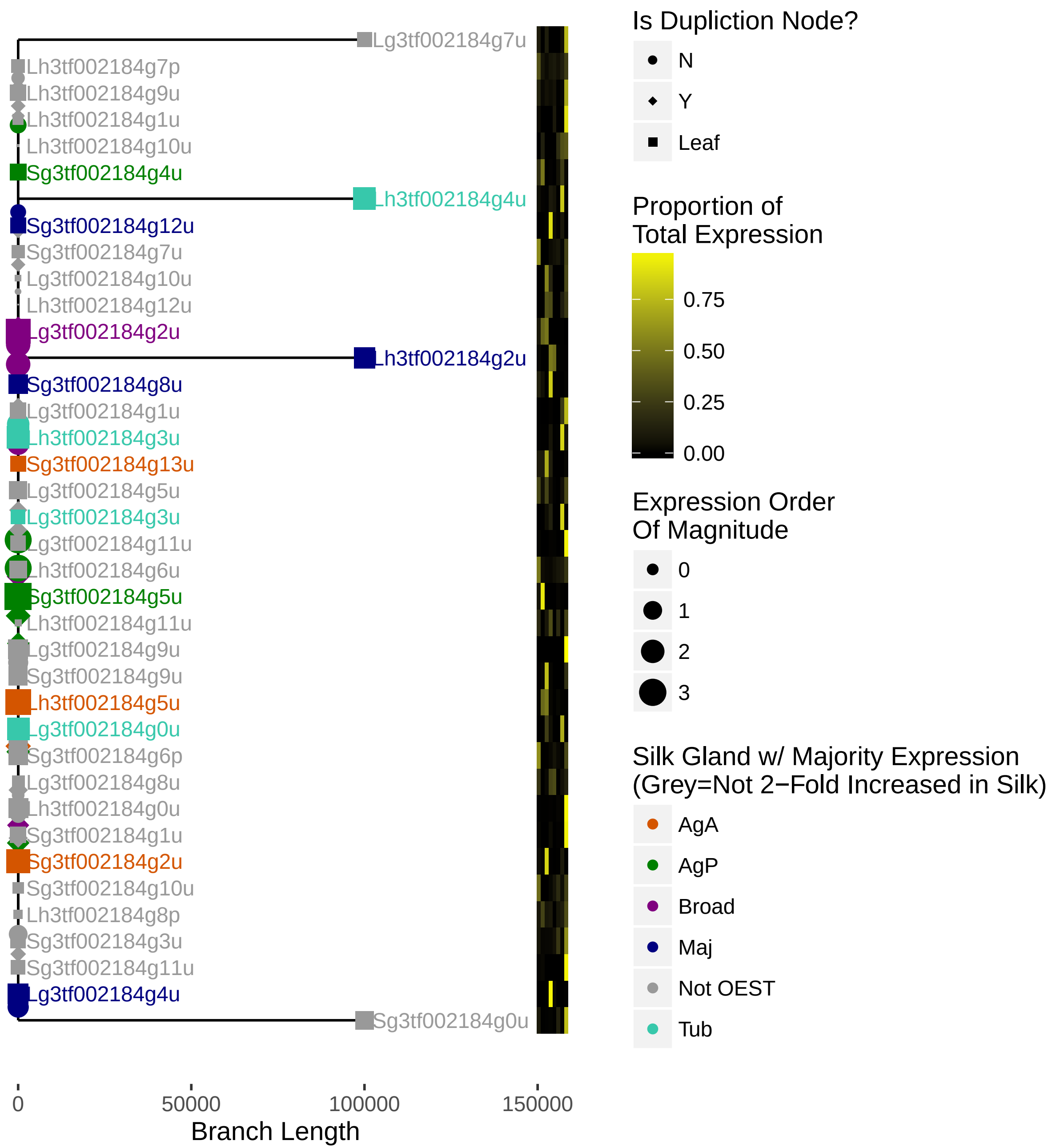
Lg3tf002184g7u
Lh3tf002184g7p
Lh3tf002184g9u
Lh3tf002184g1u
Lh3tf002184g10u
Sg3tf002184g4u
Lh3tf002184g4u
Sg3tf002184g12u
Sg3tf002184g7u
Lg3tf002184g10u
Lh3tf002184g12u
Lg3tf002184g2u
Lh3tf002184g2u
Sg3tf002184g8u
Lg3tf002184g1u
Lh3tf002184g3u
Sg3tf002184g13u
Lg3tf002184g5u
Lg3tf002184g3u
Lg3tf002184g11u
Lh3tf002184g6u
Sg3tf002184g5u
Lh3tf002184g11u
Lg3tf002184g9u
Sg3tf002184g9u
Lh3tf002184g5u
Lg3tf002184g0u
Sg3tf002184g6p
Lg3tf002184g8u
Lh3tf002184g0u
Sg3tf002184g1u
Sg3tf002184g2u
Sg3tf002184g10u
Lh3tf002184g8p
Sg3tf002184g3u
Sg3tf002184g11u
Lg3tf002184g4u
Sg3tf002184g0u
0
50000
100000
150000
Branch Length
Is Dupliction Node?
N
Y
Leaf
Proportion of Total Expression
0.75
0.50
0.25
0.00
Expression Order Of Magnitude
0
1
2
3
Silk Gland w/ Majority Expression (Grey=Not 2−Fold Increased in Silk)
AgA
AgP
Broad
Maj
Not OEST
Tub

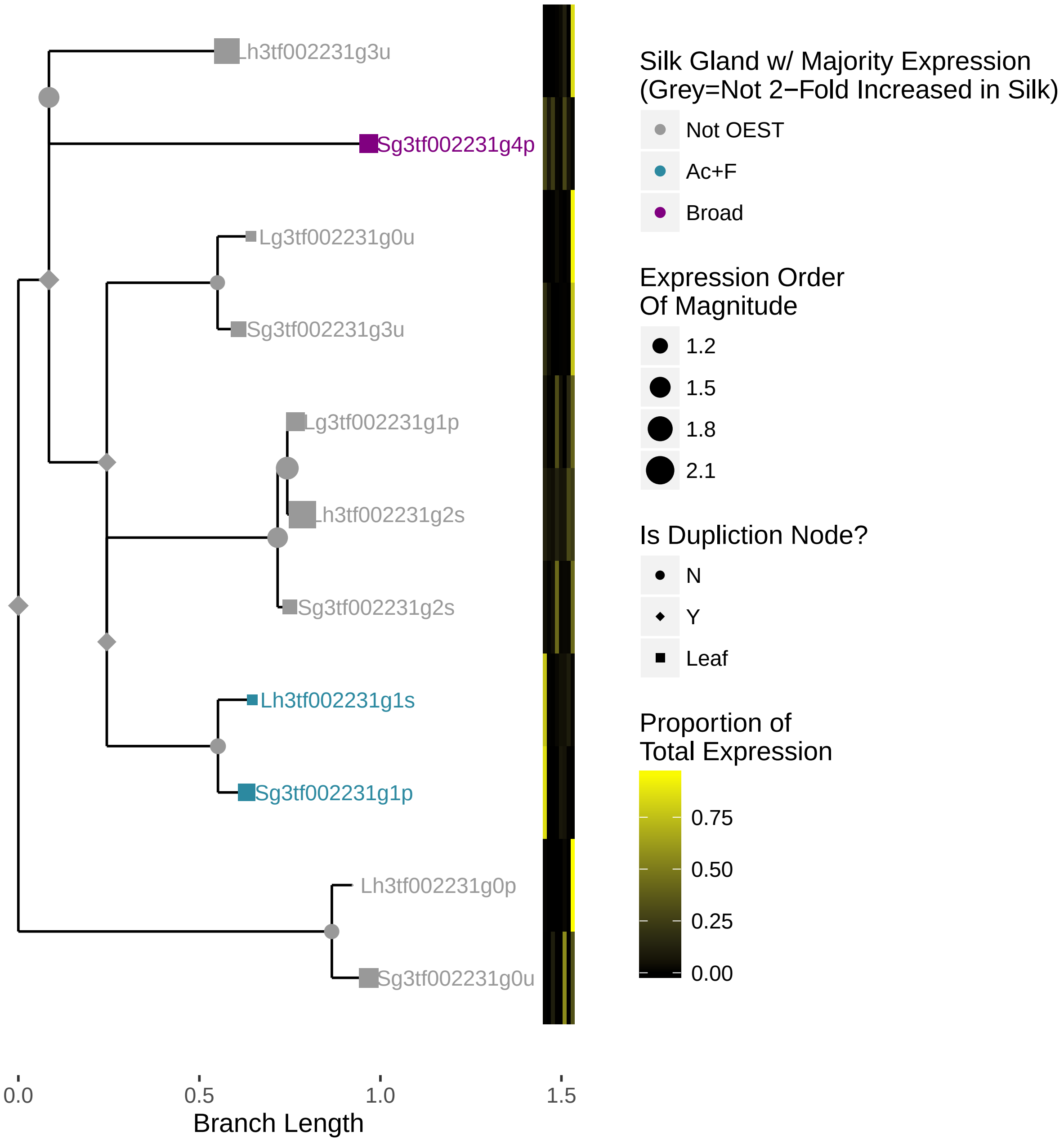

Lh3tf002231g3u
Sg3tf002231g4p
Lg3tf002231g0u
Sg3tf002231g3u
Lg3tf002231g1p
Lh3tf002231g2s
Sg3tf002231g2s
Lh3tf002231g1s
Sg3tf002231g1p
Lh3tf002231g0p
Sg3tf002231g0u
0.0
0.5
1.0
1.5
Branch Length
Silk Gland w/ Majority Expression (Grey=Not 2−Fold Increased in Silk)
Not OEST
Ac+F
Broad
Expression Order Of Magnitude
1.2
1.5
1.8
2.1
Is Dupliction Node?
N
Y
Leaf
Proportion of Total Expression
0.75
0.50
0.25
0.00

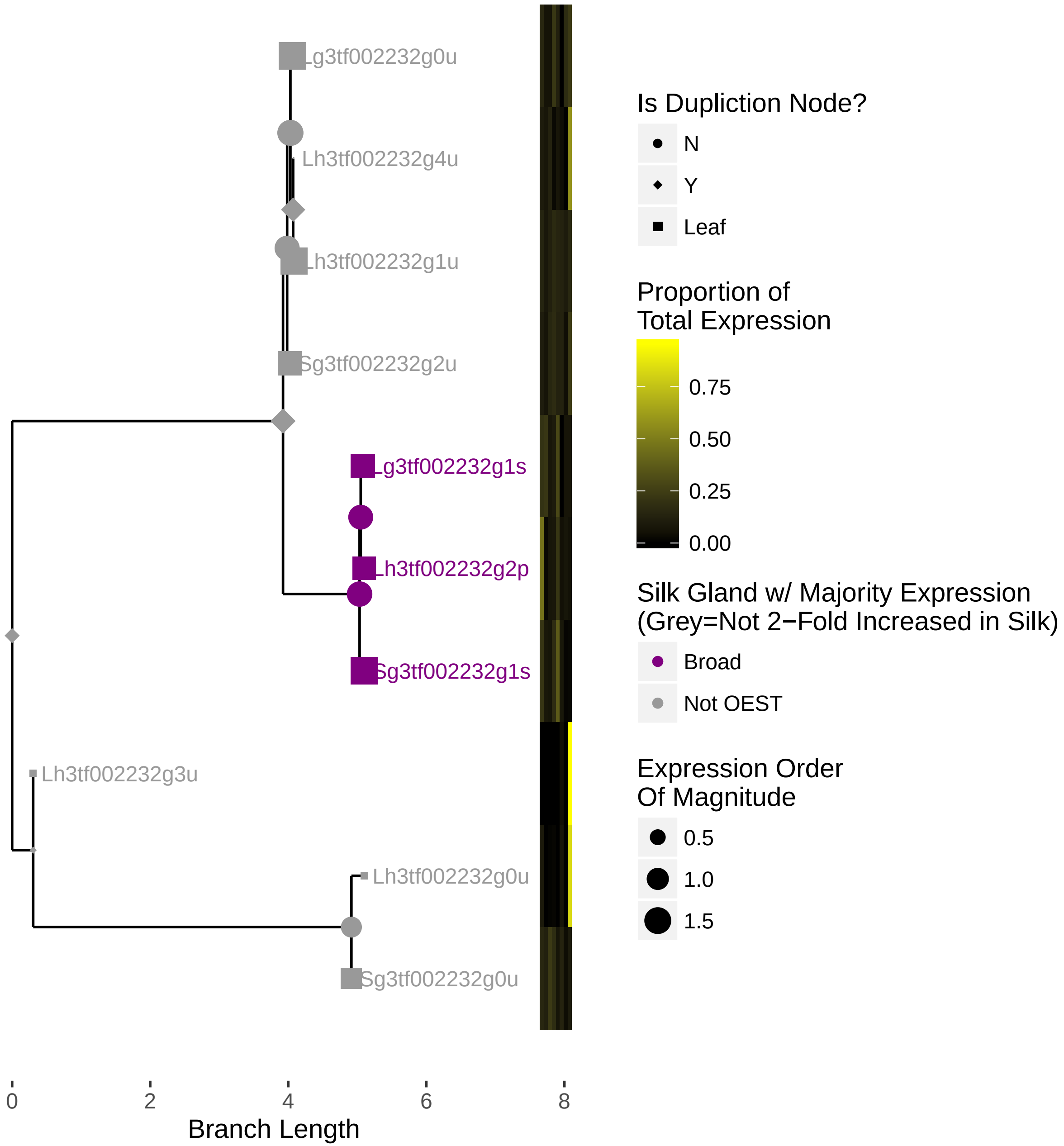

Lg3tf002232g0u
Lh3tf002232g4u
Lh3tf002232g1u
Sg3tf002232g2u
Lg3tf002232g1s
Lh3tf002232g2p
Sg3tf002232g1s
Lh3tf002232g3u
Lh3tf002232g0u
Sg3tf002232g0u
0
2
4
6
8
Branch Length
Is Dupliction Node?
N
Y
Leaf
Proportion of Total Expression
0.75
0.50
0.25
0.00
Silk Gland w/ Majority Expression (Grey=Not 2−Fold Increased in Silk)
Broad
Not OEST
Expression Order Of Magnitude
0.5
1.0
1.5

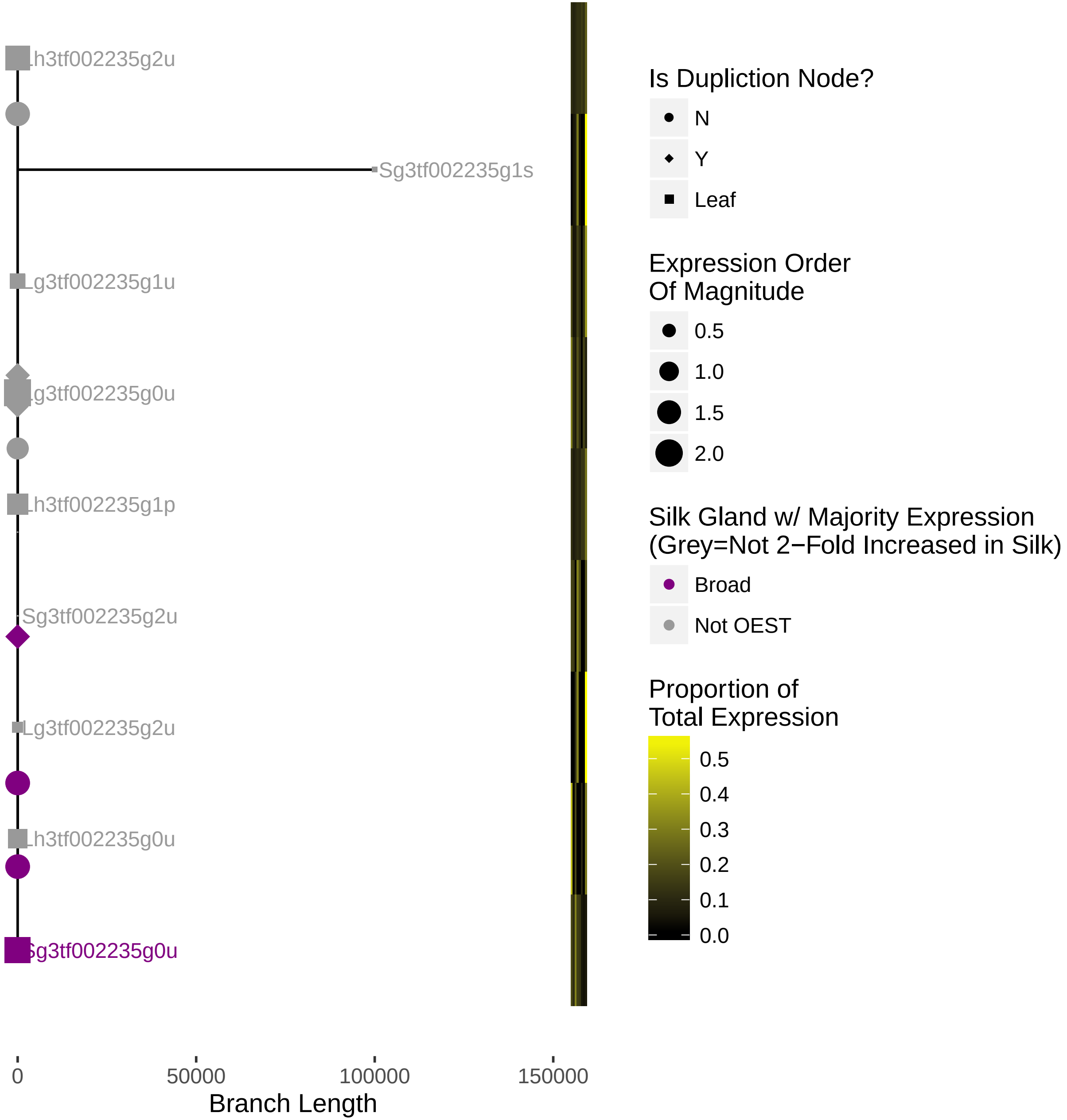

Lh3tf002235g2u
Sg3tf002235g1s
Lg3tf002235g1u
Lg3tf002235g0u
Lh3tf002235g1p
Sg3tf002235g2u
Lg3tf002235g2u
Lh3tf002235g0u
Sg3tf002235g0u
0
50000
100000
150000
Branch Length
Is Dupliction Node?
N
Y
Leaf
Expression Order
Of Magnitude
0.5
1.0
1.5
2.0
Silk Gland w/ Majority Expression
(Grey=Not 2−Fold Increased in Silk)
Broad
Not OEST
Proportion of
Total Expression
0.5
0.4
0.3
0.2
0.1
0.0

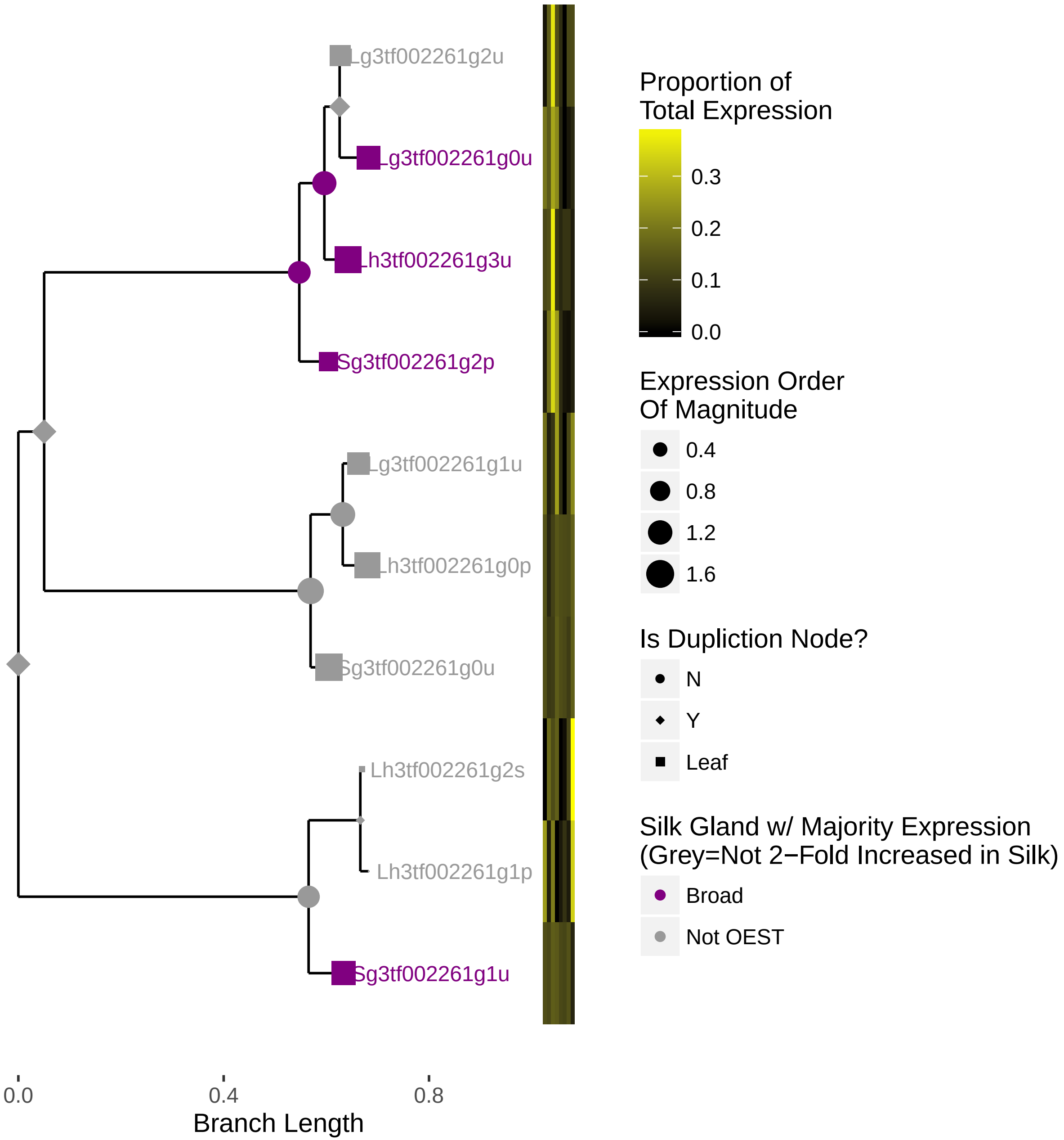

Lg3tf002261g2u
Lg3tf002261g0u
Lh3tf002261g3u
Sg3tf002261g2p
Lg3tf002261g1u
Lh3tf002261g0p
Sg3tf002261g0u
Lh3tf002261g2s
Lh3tf002261g1p
Sg3tf002261g1u
0.0
0.4
0.8
Branch Length
Proportion of Total Expression
0.3
0.2
0.1
0.0
Expression Order Of Magnitude
0.4
0.8
1.2
1.6
Is Dupliction Node?
N
Y
Leaf
Silk Gland w/ Majority Expression (Grey=Not 2−Fold Increased in Silk)
Broad
Not OEST

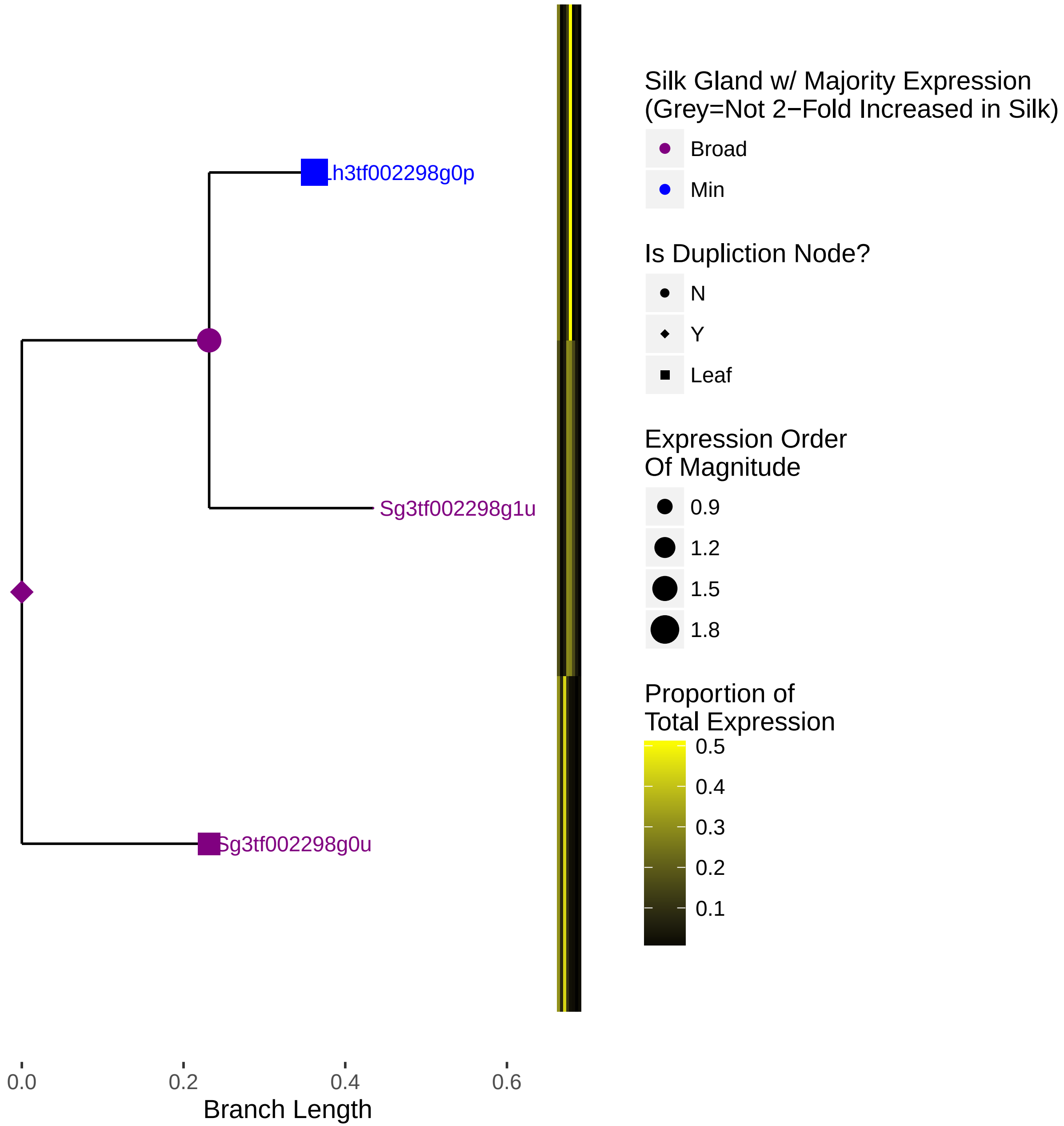

Lh3tf002298g0p
Sg3tf002298g1u
Sg3tf002298g0u
0.0
0.2
0.4
0.6
Branch Length
Silk Gland w/ Majority Expression
(Grey=Not 2−Fold Increased in Silk)
Broad
Min
Is Dupliction Node?
N
Y
Leaf
Expression Order
Of Magnitude
0.9
1.2
1.5
1.8
Proportion of
Total Expression
0.5
0.4
0.3
0.2
0.1

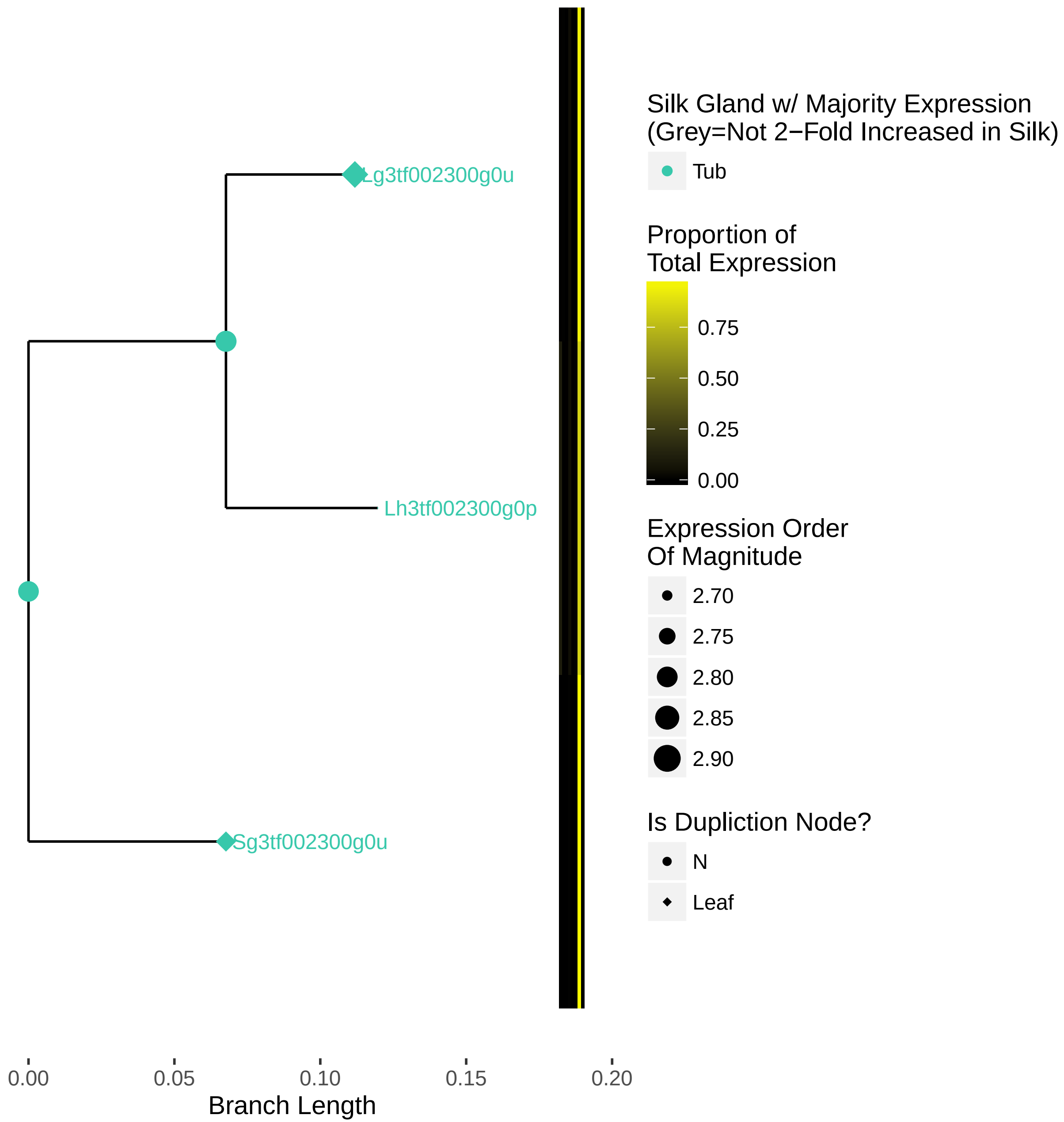

Lg3tf002300g0u
Lh3tf002300g0p
Sg3tf002300g0u
Silk Gland w/ Majority Expression
(Grey=Not 2−Fold Increased in Silk)
Tub
Proportion of
Total Expression
0.75
0.50
0.25
0.00
Expression Order
Of Magnitude
2.70
2.75
2.80
2.85
2.90
Is Dupliction Node?
N
Leaf
0.00
0.05
0.10
0.15
0.20
Branch Length

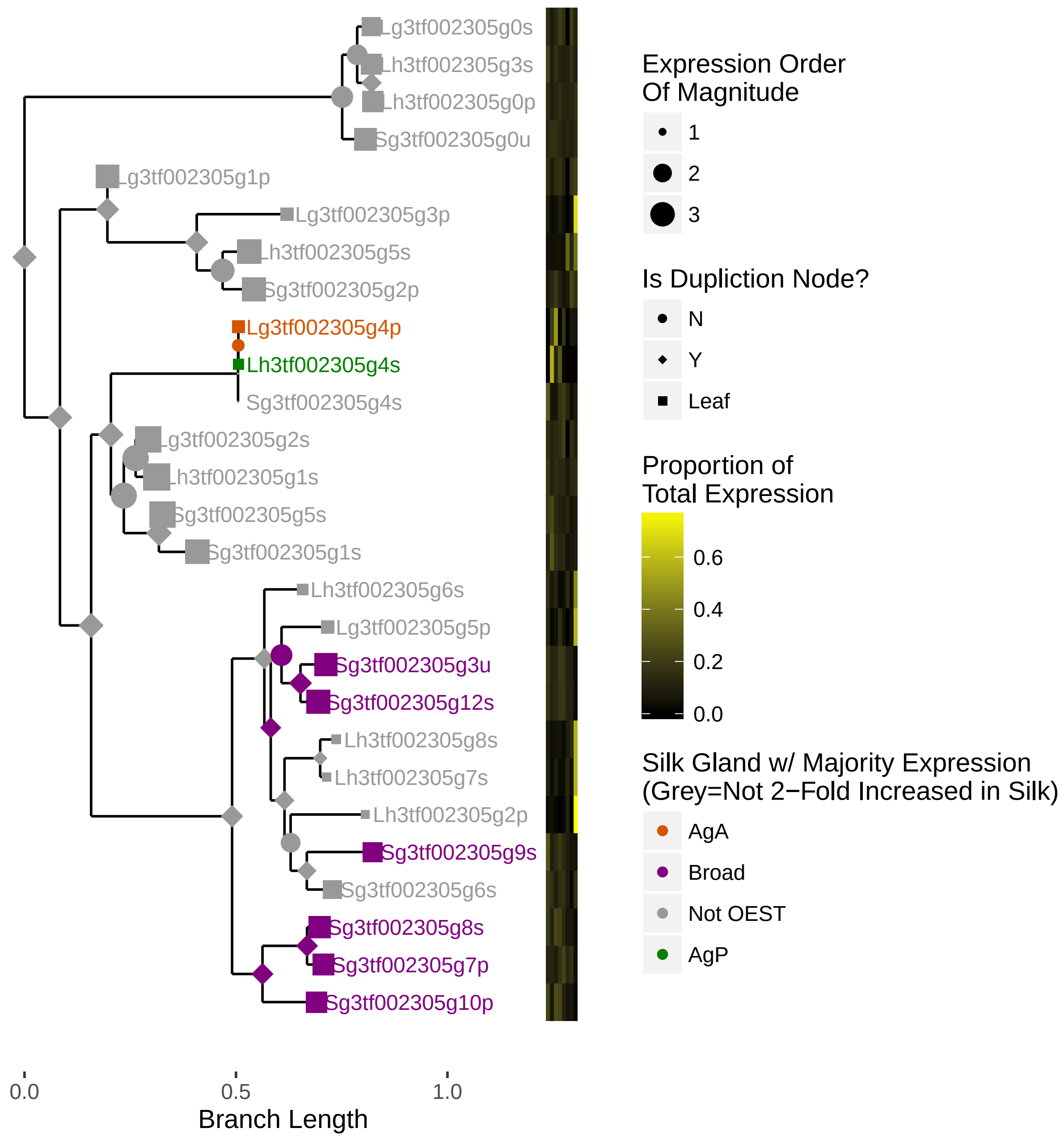

Lg3tf002305g0s
Lh3tf002305g3s
Lh3tf002305g0p
Sg3tf002305g0u
Lg3tf002305g1p
Lg3tf002305g3p
Lh3tf002305g5s
Sg3tf002305g2p
Lg3tf002305g4p
Lh3tf002305g4s
Sg3tf002305g4s
Lg3tf002305g2s
Lh3tf002305g1s
Sg3tf002305g5s
Sg3tf002305g1s
Lh3tf002305g6s
Lg3tf002305g5p
Sg3tf002305g3u
Sg3tf002305g12s
Lh3tf002305g8s
Lh3tf002305g7s
Lh3tf002305g2p
Sg3tf002305g9s
Sg3tf002305g6s
Sg3tf002305g8s
Sg3tf002305g7p
Sg3tf002305g10p
0.0
0.5
1.0
Branch Length
Expression Order Of Magnitude
1
2
3
Is Duplicion Node?
N
Y
Leaf
Proportion of Total Expression
0.6
0.4
0.2
0.0
Silk Gland w/ Majority Expression (Grey=Not 2−Fold Increased in Silk)
AgA
Broad
Not OEST
AgP

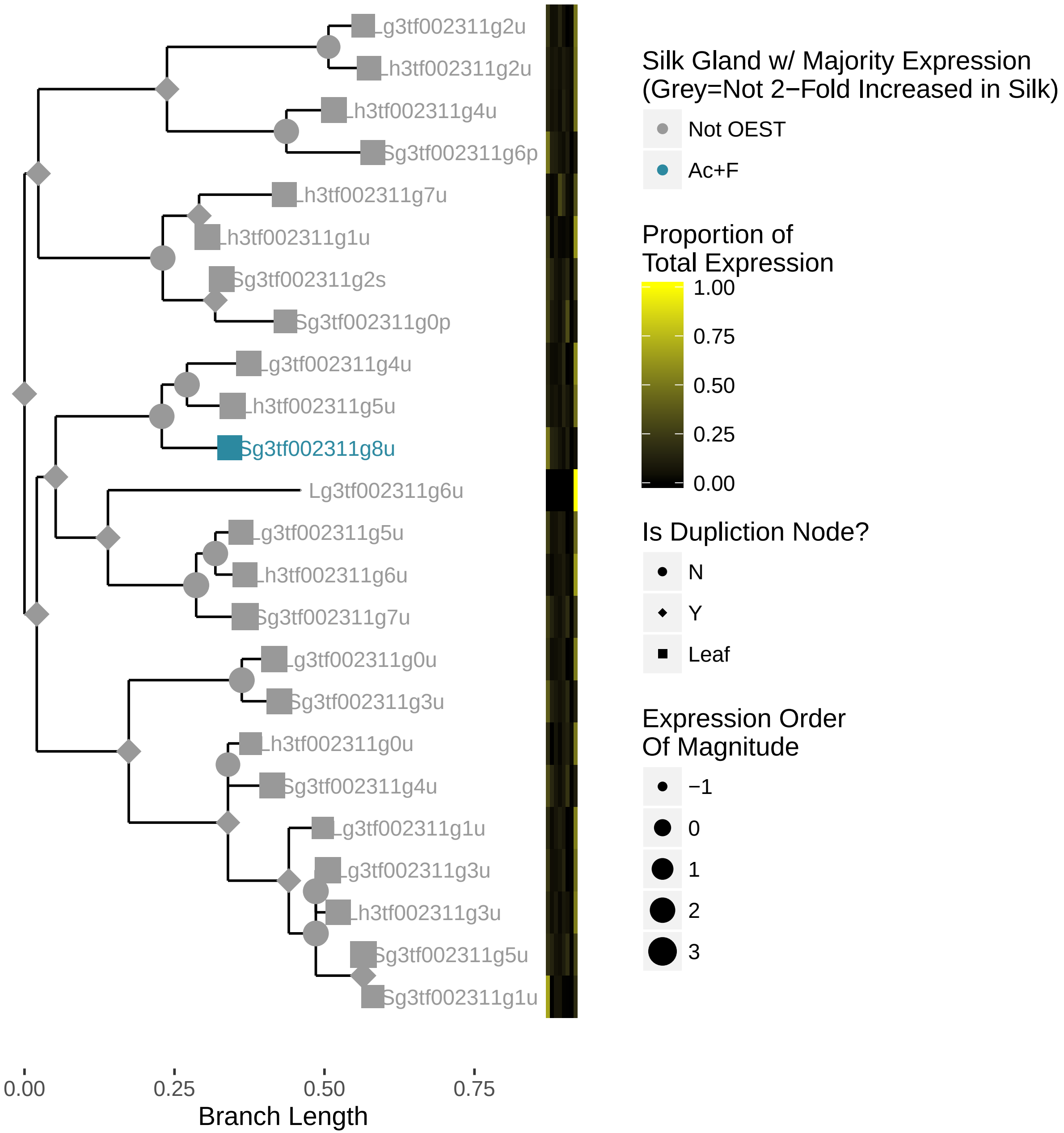
Lg3tf002311g2u
Lh3tf002311g2u
Lh3tf002311g4u
Sg3tf002311g6p
Lh3tf002311g7u
Lh3tf002311g1u
Sg3tf002311g2s
Sg3tf002311g0p
Lg3tf002311g4u
Lh3tf002311g5u
Sg3tf002311g8u
Lg3tf002311g6u
Lg3tf002311g5u
Lh3tf002311g6u
Sg3tf002311g7u
Lg3tf002311g0u
Sg3tf002311g3u
Lh3tf002311g0u
Sg3tf002311g4u
Lg3tf002311g1u
Lg3tf002311g3u
Lh3tf002311g3u
Sg3tf002311g5u
Sg3tf002311g1u
0.00
0.25
0.50
0.75
Branch Length
Silk Gland w/ Majority Expression
(Grey=Not 2−Fold Increased in Silk)
Not OEST
Ac+F
Proportion of
Total Expression
1.00
0.75
0.50
0.25
0.00
Is Dupliction Node?
N
Y
Leaf
Expression Order
Of Magnitude
−1
0
1
2
3

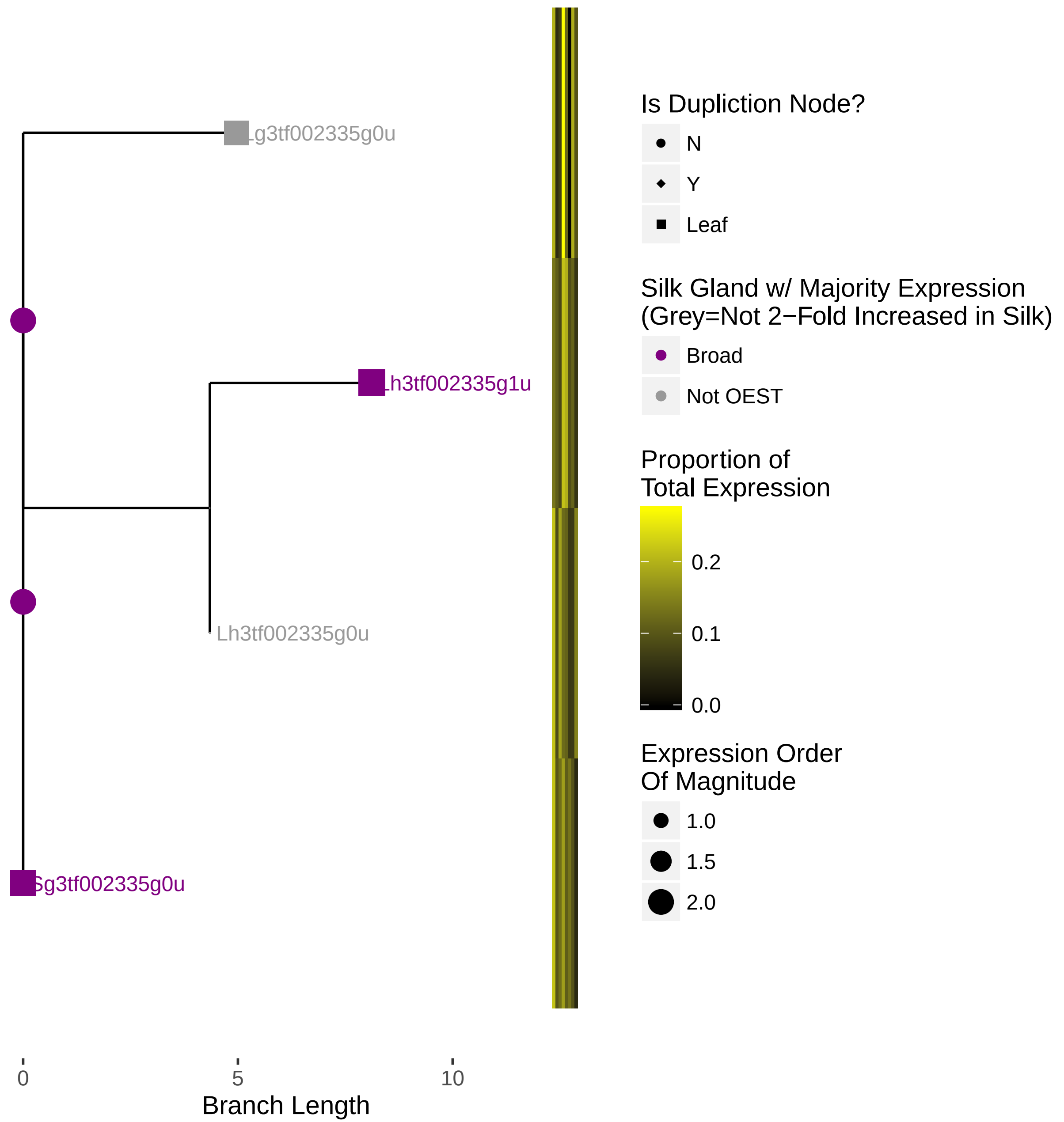
Lg3tf002335g0u
Lh3tf002335g1u
Lh3tf002335g0u
Sg3tf002335g0u
0
5
10
Branch Length
Is Dupliction Node?
N
Y
Leaf
Silk Gland w/ Majority Expression
(Grey=Not 2−Fold Increased in Silk)
Broad
Not OEST
Proportion of
Total Expression
0.2
0.1
0.0
Expression Order
Of Magnitude
1.0
1.5
2.0

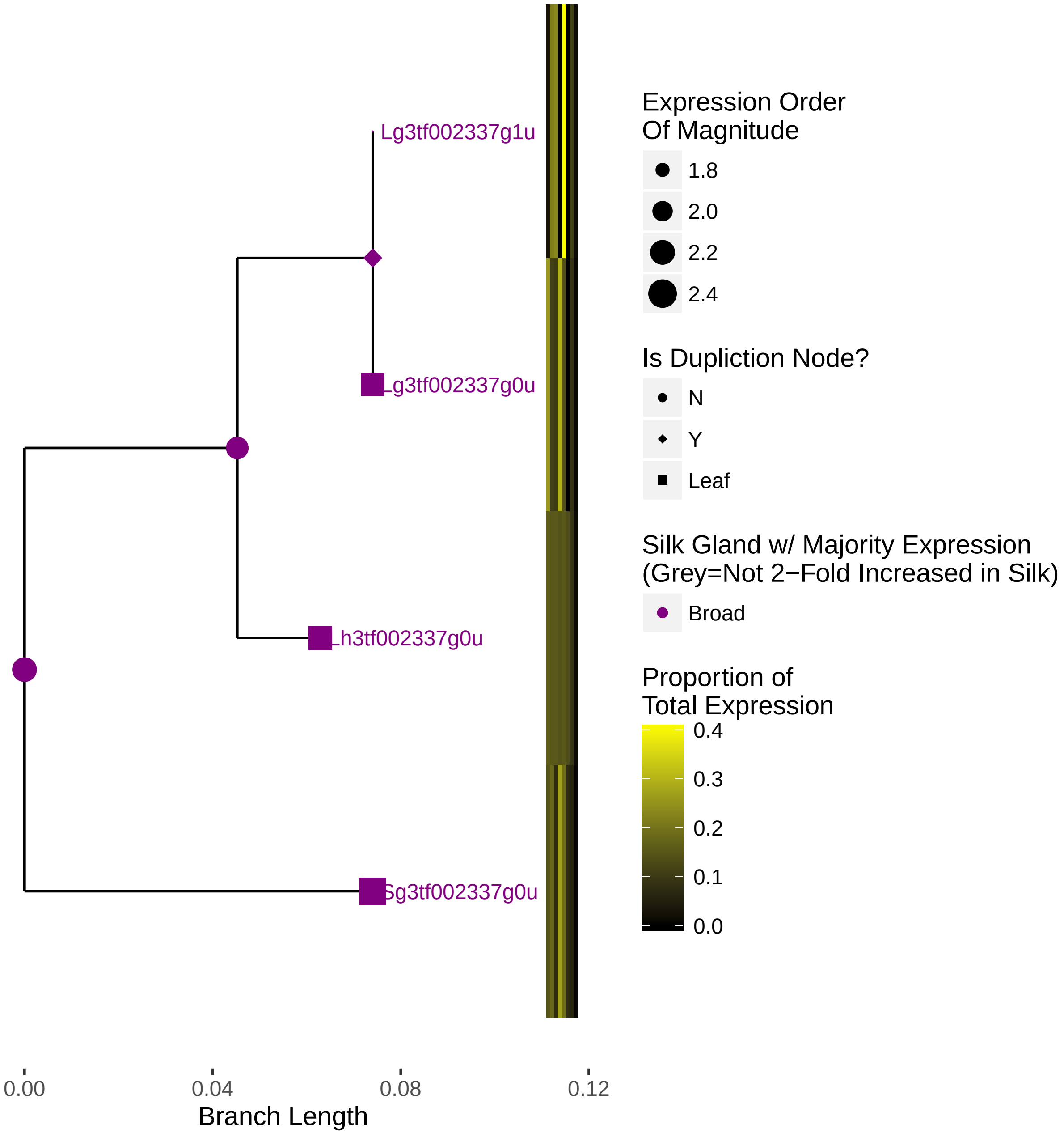

Lg3tf002337g1u
Lg3tf002337g0u
Lh3tf002337g0u
Sg3tf002337g0u
Expression Order
Of Magnitude
1.8
2.0
2.2
2.4
Is Dupliction Node?
N
Y
Leaf
Silk Gland w/ Majority Expression
(Grey=Not 2−Fold Increased in Silk)
Broad
Proportion of
Total Expression
0.4
0.3
0.2
0.1
0.0
0.00
0.04
0.08
0.12
Branch Length

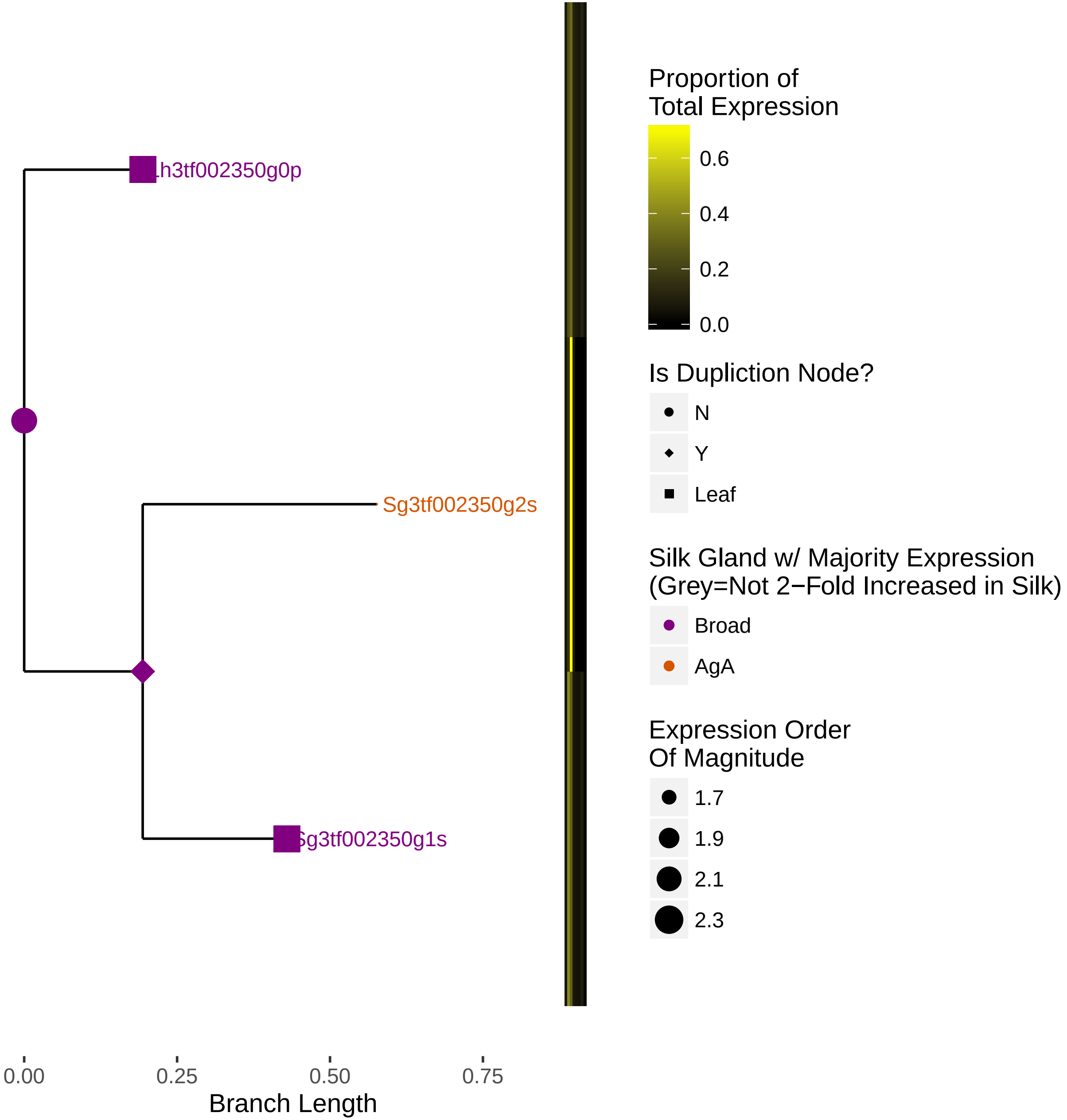

Proportion of
Total Expression
0.6
0.4
0.2
0.0
Is Dupliction Node?
N
Y
Leaf
Silk Gland w/ Majority Expression
(Grey=Not 2−Fold Increased in Silk)
Broad
AgA
Expression Order
Of Magnitude
1.7
1.9
2.1
2.3
h3tf002350g0p
Sg3tf002350g2s
g3tf002350g1s
0.00
0.25
0.50
0.75
Branch Length

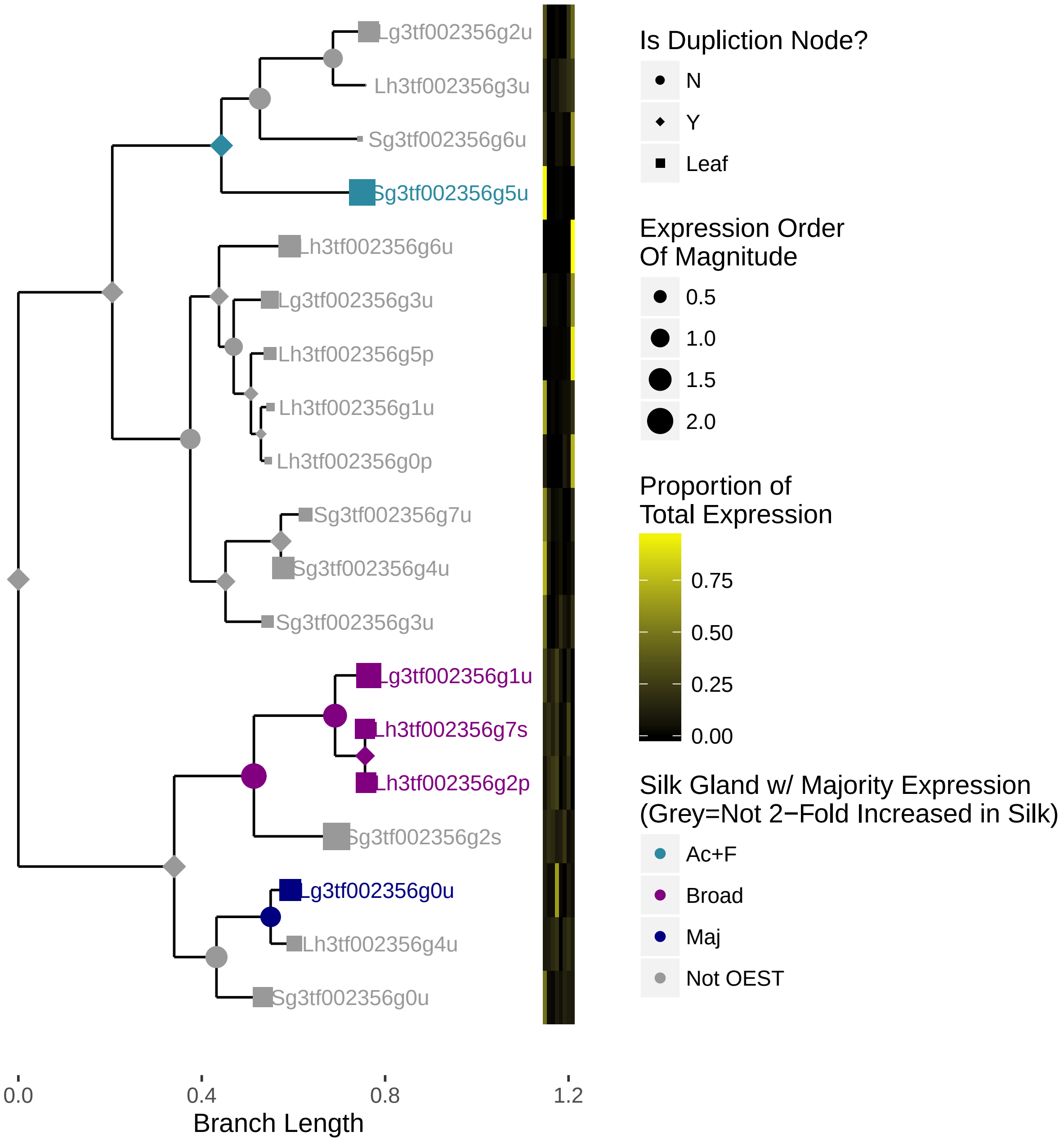

Lg3tf002356g2u
Lh3tf002356g3u
Sg3tf002356g6u
Sg3tf002356g5u
Lh3tf002356g6u
Lg3tf002356g3u
Lh3tf002356g5p
Lh3tf002356g1u
Lh3tf002356g0p
Sg3tf002356g7u
Sg3tf002356g4u
Sg3tf002356g3u
Lg3tf002356g1u
Lh3tf002356g7s
Lh3tf002356g2p
Sg3tf002356g2s
Lg3tf002356g0u
Lh3tf002356g4u
Sg3tf002356g0u
0.0
0.4
0.8
1.2
Branch Length
Is Dupliction Node?
N
Y
Leaf
Expression Order Of Magnitude
0.5
1.0
1.5
2.0
Proportion of Total Expression
0.75
0.50
0.25
0.00
Silk Gland w/ Majority Expression (Grey=Not 2−Fold Increased in Silk)
Ac+F
Broad
Maj
Not OEST

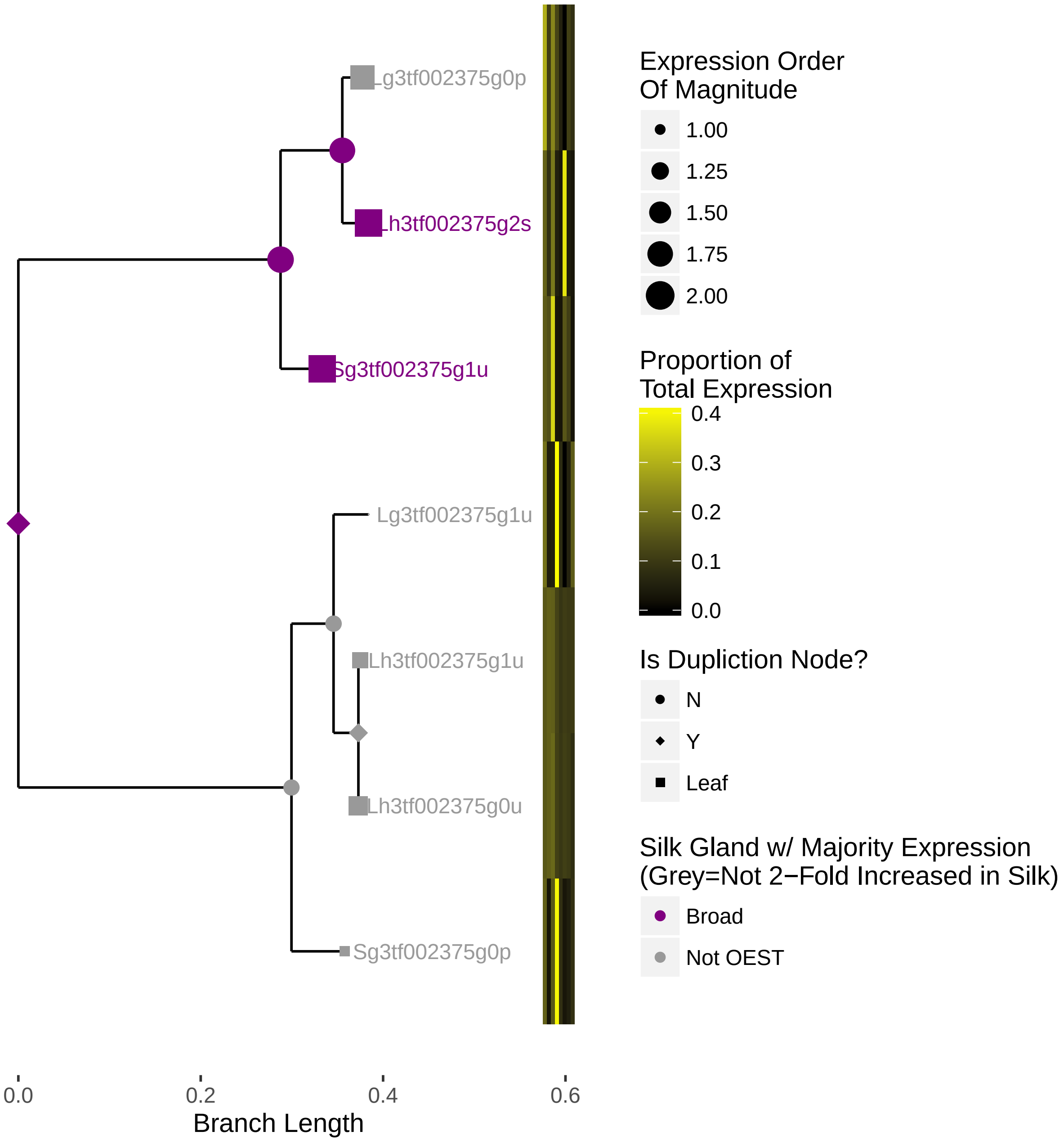
Lg3tf002375g0p
Lh3tf002375g2s
Sg3tf002375g1u
Lg3tf002375g1u
Lh3tf002375g1u
Lh3tf002375g0u
Sg3tf002375g0p
0.0
0.2
0.4
0.6
Branch Length
Expression Order Of Magnitude
1.00
1.25
1.50
1.75
2.00
Proportion of Total Expression
0.4
0.3
0.2
0.1
0.0
Is Dupliction Node?
N
Y
Leaf
Silk Gland w/ Majority Expression (Grey=Not 2−Fold Increased in Silk)
Broad
Not OEST

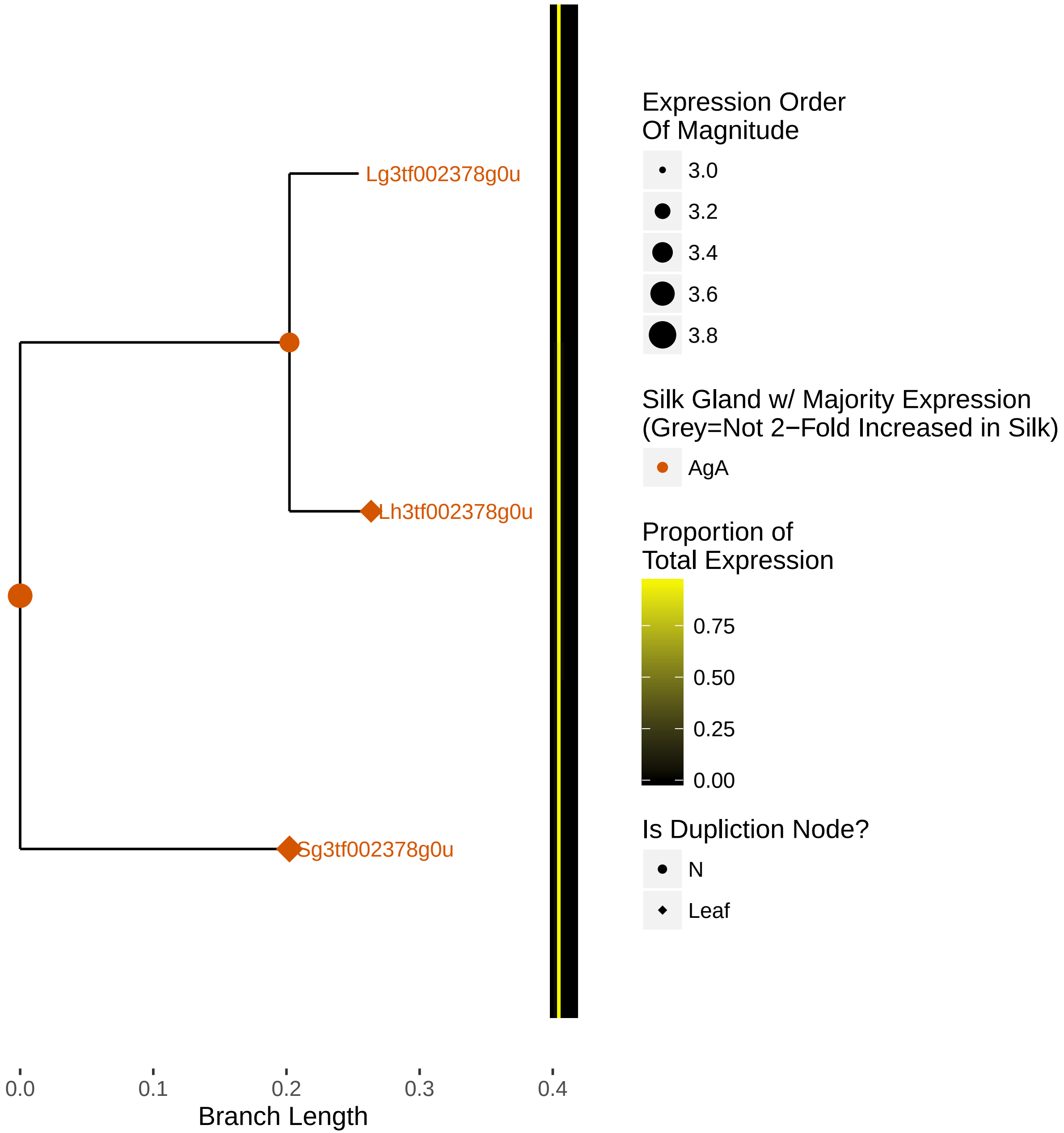
Lg3tf002378g0u
Lh3tf002378g0u
Sg3tf002378g0u
0.0
0.1
0.2
0.3
0.4
Branch Length
Expression Order
Of Magnitude
3.0
3.2
3.4
3.6
3.8
Silk Gland w/ Majority Expression
(Grey=Not 2−Fold Increased in Silk)
AgA
Proportion of
Total Expression
0.75
0.50
0.25
0.00
Is Dupliction Node?
N
Leaf

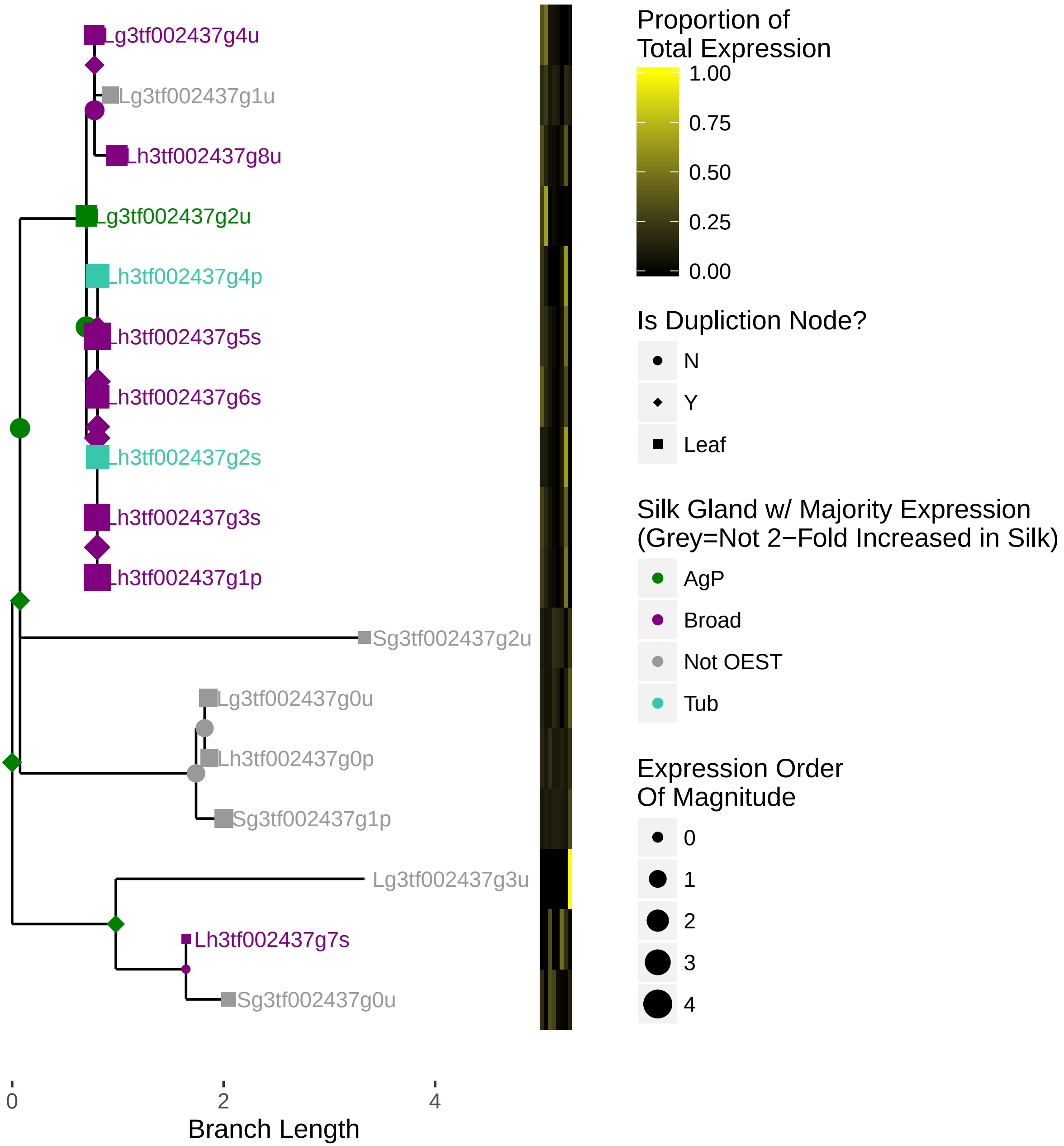
Lg3tf002437g4u
Lg3tf002437g1u
Lh3tf002437g8u
Lg3tf002437g2u
Lh3tf002437g4p
Lh3tf002437g5s
Lh3tf002437g6s
Lh3tf002437g2s
Lh3tf002437g3s
Lh3tf002437g1p
Sg3tf002437g2u
Lg3tf002437g0u
Lh3tf002437g0p
Sg3tf002437g1p
Lg3tf002437g3u
Lh3tf002437g7s
Sg3tf002437g0u
0
2
4
Branch Length
Proportion of Total Expression
1.00
0.75
0.50
0.25
0.00
Is Dupliction Node?
N
Y
Leaf
Silk Gland w/ Majority Expression (Grey=Not 2−Fold Increased in Silk)
AgP
Broad
Not OEST
Tub
Expression Order Of Magnitude
0
1
2
3
4

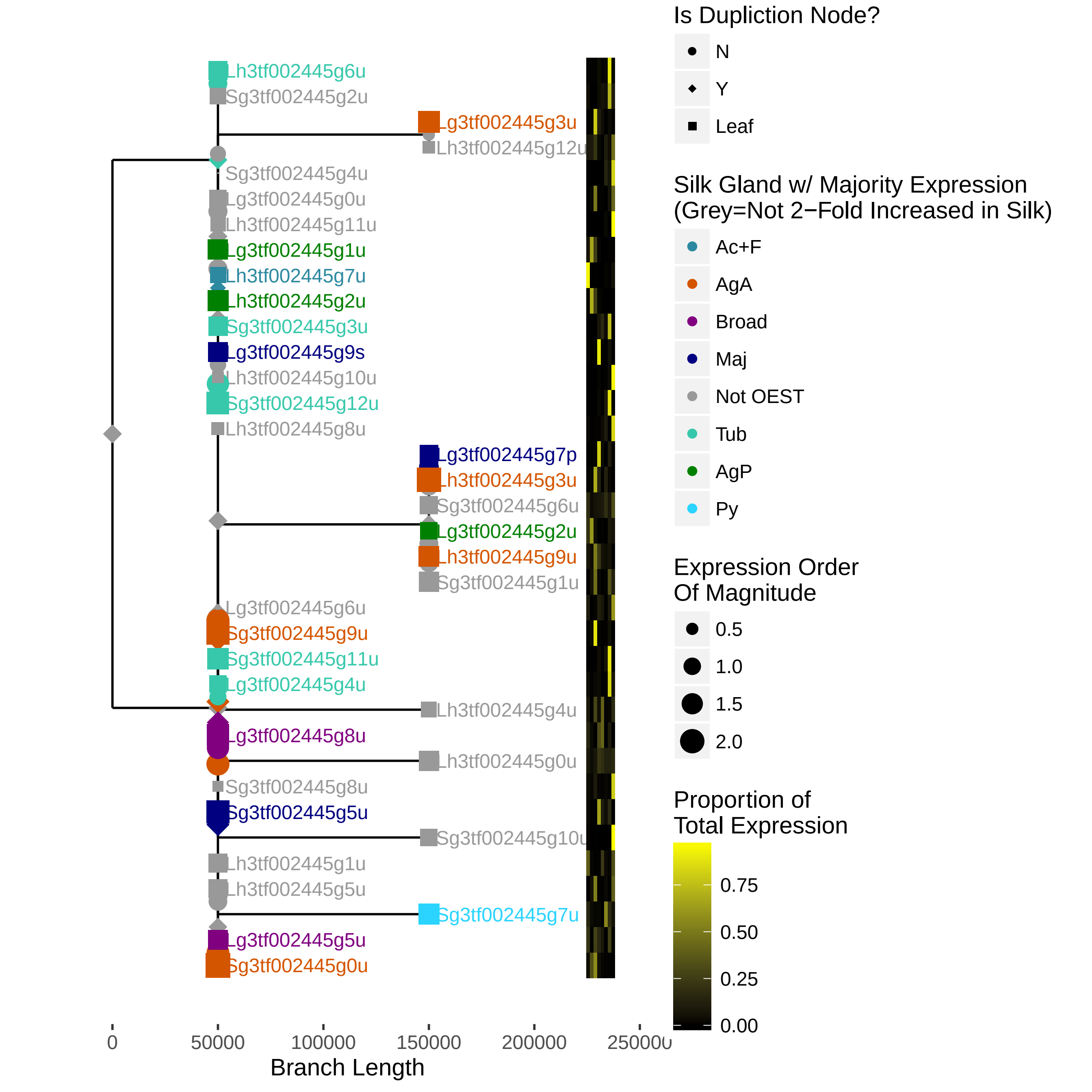
Is Dupliction Node?
N
Y
Leaf
Silk Gland w/ Majority Expression
(Grey=Not 2−Fold Increased in Silk)
Ac+F
AgA
Broad
Maj
Not OEST
Tub
AgP
Py
Expression Order
Of Magnitude
0.5
1.0
1.5
2.0
Proportion of
Total Expression
0.75
0.50
0.25
0.00
Lh3tf002445g6u
Sg3tf002445g2u
Lg3tf002445g3u
Lh3tf002445g12u
Sg3tf002445g4u
Lg3tf002445g0u
Lh3tf002445g11u
Lg3tf002445g1u
Lh3tf002445g7u
Lh3tf002445g2u
Sg3tf002445g3u
Lg3tf002445g9s
Lh3tf002445g10u
Sg3tf002445g12u
Lh3tf002445g8u
Lg3tf002445g7p
Lh3tf002445g3u
Sg3tf002445g6u
Lg3tf002445g2u
Lh3tf002445g9u
Sg3tf002445g1u
Lg3tf002445g6u
Sg3tf002445g9u
Sg3tf002445g11u
Lg3tf002445g4u
Lh3tf002445g4u
Lg3tf002445g8u
Lh3tf002445g0u
Sg3tf002445g8u
Sg3tf002445g5u
Sg3tf002445g10u
Lh3tf002445g1u
Lh3tf002445g5u
Sg3tf002445g7u
Lg3tf002445g5u
Sg3tf002445g0u
0
50000
100000
150000
200000
250000
Branch Length

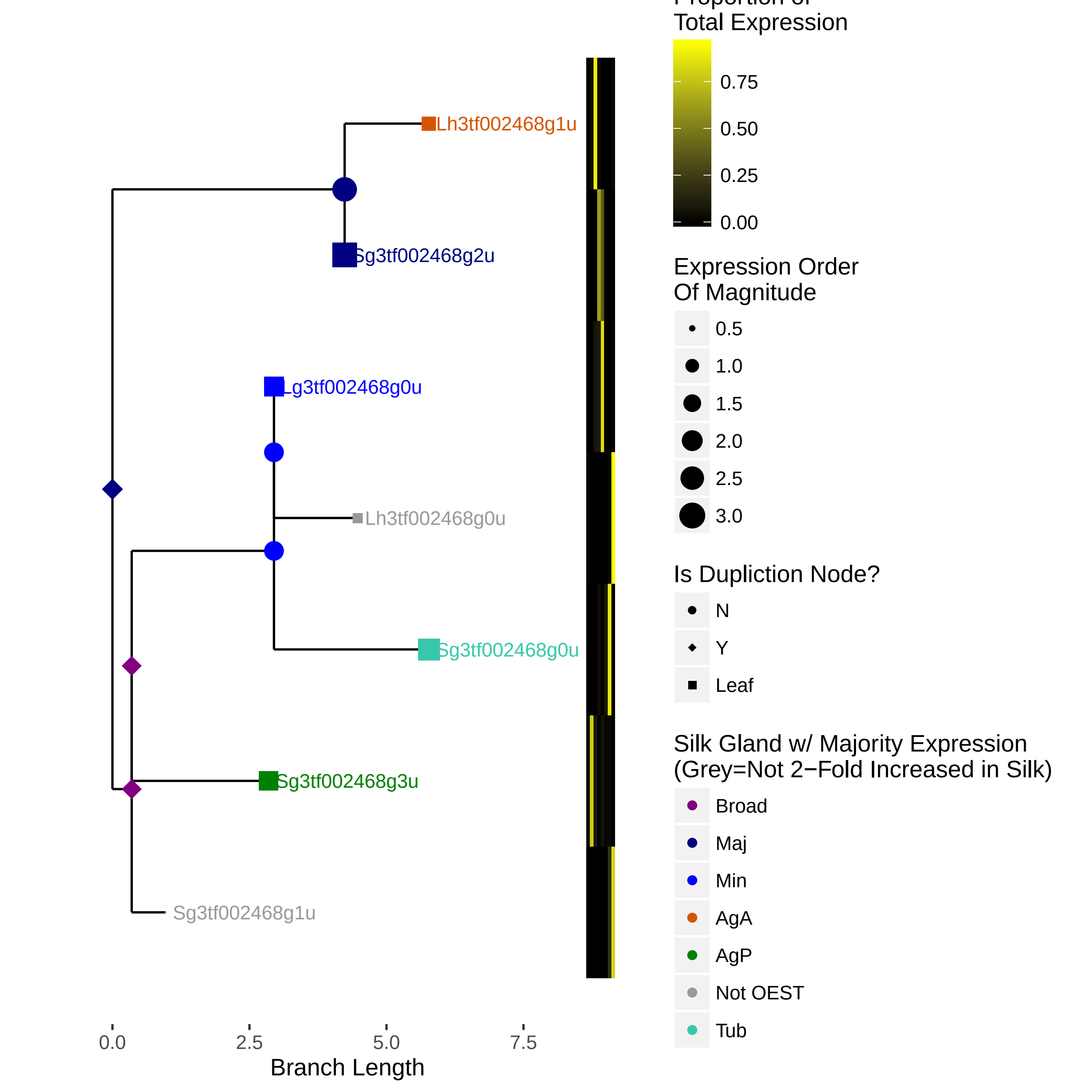

Proportion of
Total Expression
0.75
0.50
0.25
0.00
Expression Order
Of Magnitude
0.5
1.0
1.5
2.0
2.5
3.0
Is Dupliction Node?
N
Y
Leaf
Silk Gland w/ Majority Expression
(Grey=Not 2−Fold Increased in Silk)
Broad
Maj
Min
AgA
AgP
Not OEST
Tub
Lh3tf002468g1u
Sg3tf002468g2u
Lg3tf002468g0u
Lh3tf002468g0u
Sg3tf002468g0u
Sg3tf002468g3u
Sg3tf002468g1u
0.0
2.5
5.0
7.5
Branch Length

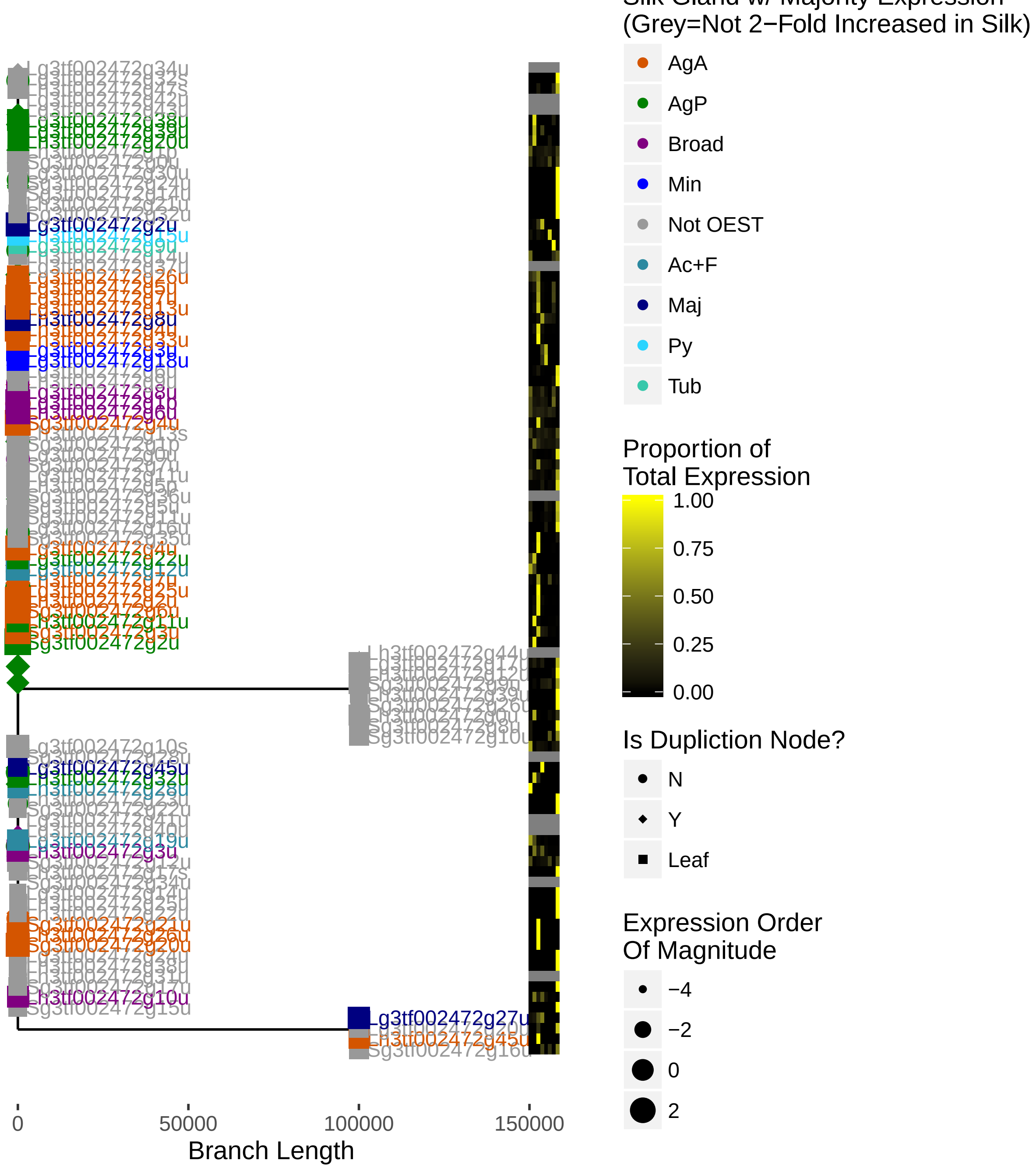
(Grey=Not 2−Fold Increased in Silk)
AgA
AgP
Broad
Min
Not OEST
Ac+F
Maj
Py
Tub
Proportion of
Total Expression
1.00
0.75
0.50
0.25
0.00
Is Dupliction Node?
N
Y
Leaf
Expression Order
Of Magnitude
−4
−2
0
2
Lg3tf002472g2u
Lh3tf002472g8u
Lg3tf002472g18u
Lh3tf002472g6u
Sg3tf002472g4u
Sg3tf002472g2u
Lg3tf002472g45u
Lh3tf002472g3u
Lh3tf002472g10u
Sg3tf002472g15u
Lg3tf002472g27u
Lh3tf002472g45u
Sg3tf002472g16u
Sg3tf002472g10u
0
50000
100000
150000
Branch Length

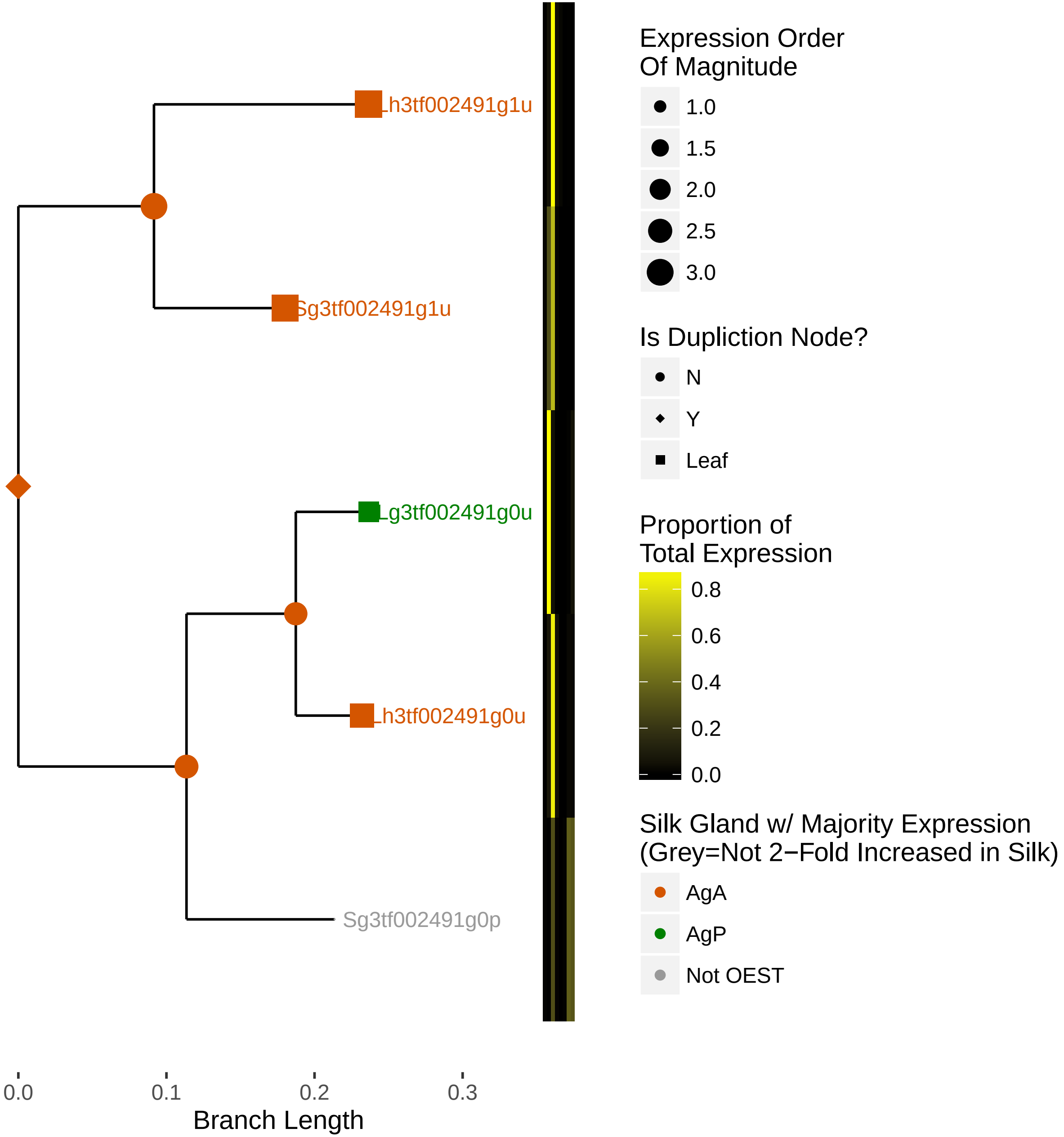

h3tf002491g1u
g3tf002491g1u
Lg3tf002491g0u
h3tf002491g0u
Sg3tf002491g0p
0.0
0.1
0.2
0.3
Branch Length
Expression Order
Of Magnitude
1.0
1.5
2.0
2.5
3.0
Is Dupliction Node?
N
Y
Leaf
Proportion of
Total Expression
0.8
0.6
0.4
0.2
0.0
Silk Gland w/ Majority Expression
(Grey=Not 2−Fold Increased in Silk)
AgA
AgP
Not OEST

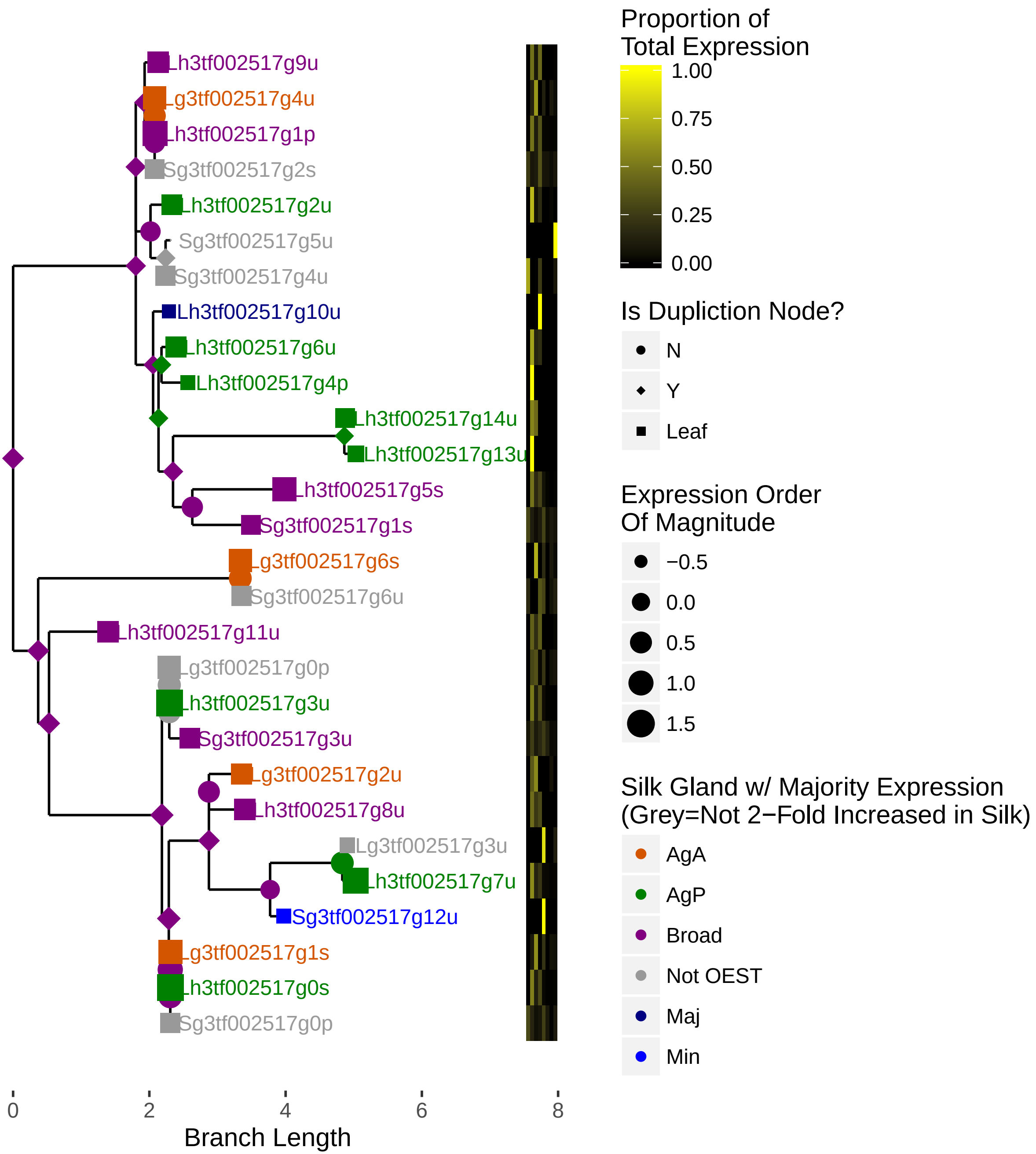
Lh3tf002517g9u
Lg3tf002517g4u
Lh3tf002517g1p
Sg3tf002517g2s
Lh3tf002517g2u
Sg3tf002517g5u
Sg3tf002517g4u
Lh3tf002517g10u
Lh3tf002517g6u
Lh3tf002517g4p
Lh3tf002517g14u
Lh3tf002517g13u
Lh3tf002517g5s
Sg3tf002517g1s
Lg3tf002517g6s
Sg3tf002517g6u
Lh3tf002517g11u
Lg3tf002517g0p
Lh3tf002517g3u
Sg3tf002517g3u
Lg3tf002517g2u
Lh3tf002517g8u
Lg3tf002517g3u
Lh3tf002517g7u
Sg3tf002517g12u
Lg3tf002517g1s
Lh3tf002517g0s
Sg3tf002517g0p
0
2
4
6
8
Branch Length
Proportion of
Total Expression
1.00
0.75
0.50
0.25
0.00
Is Dupliction Node?
N
Y
Leaf
Expression Order
Of Magnitude
−0.5
0.0
0.5
1.0
1.5
Silk Gland w/ Majority Expression
(Grey=Not 2−Fold Increased in Silk)
AgA
AgP
Broad
Not OEST
Maj
Min

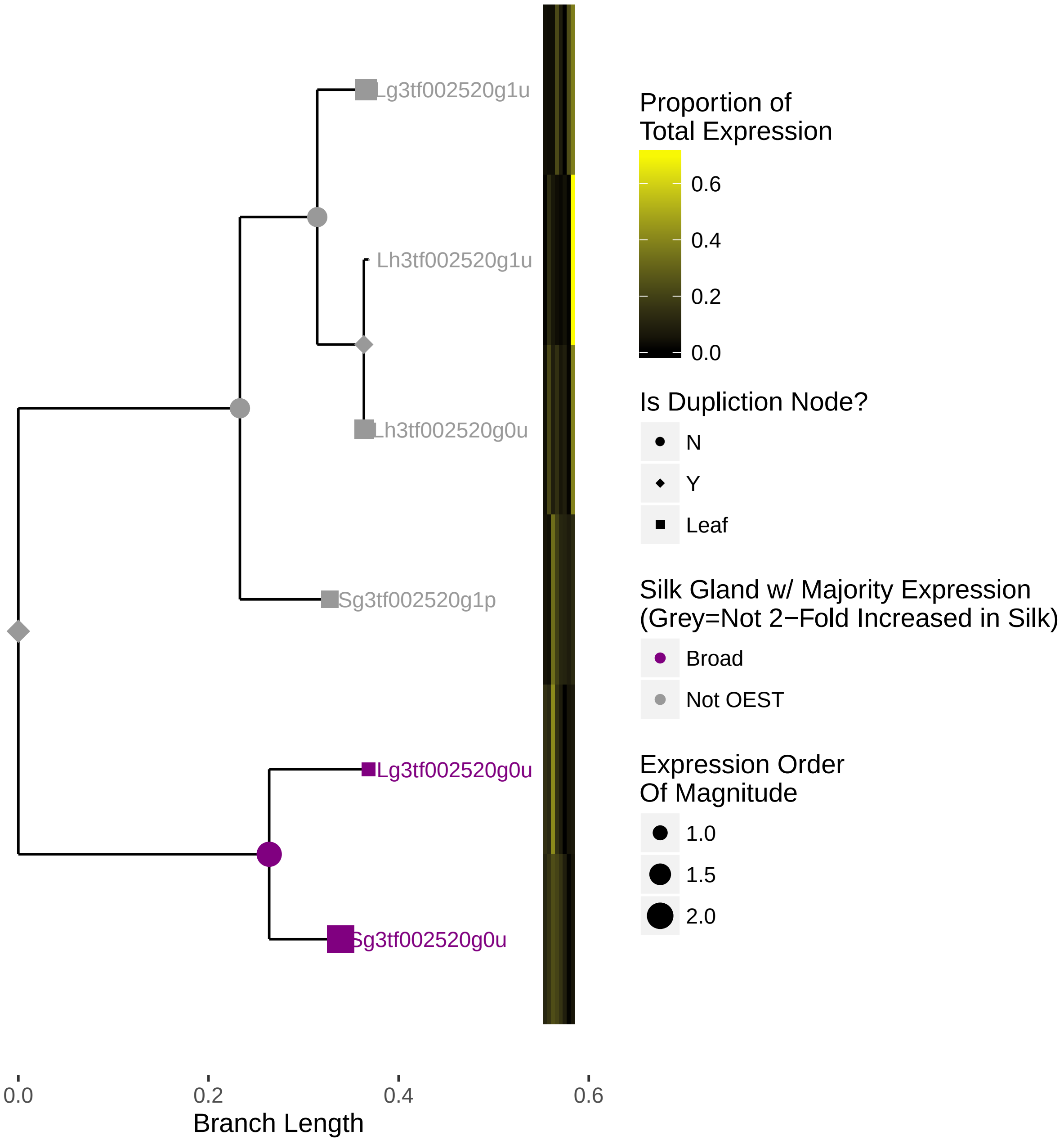

Lg3tf002520g1u
Lh3tf002520g1u
Lh3tf002520g0u
Sg3tf002520g1p
Lg3tf002520g0u
Sg3tf002520g0u
0.0
0.2
0.4
0.6
Branch Length
Proportion of Total Expression
0.6
0.4
0.2
0.0
Is Dupliction Node?
N
Y
Leaf
Silk Gland w/ Majority Expression (Grey=Not 2−Fold Increased in Silk)
Broad
Not OEST
Expression Order Of Magnitude
1.0
1.5
2.0

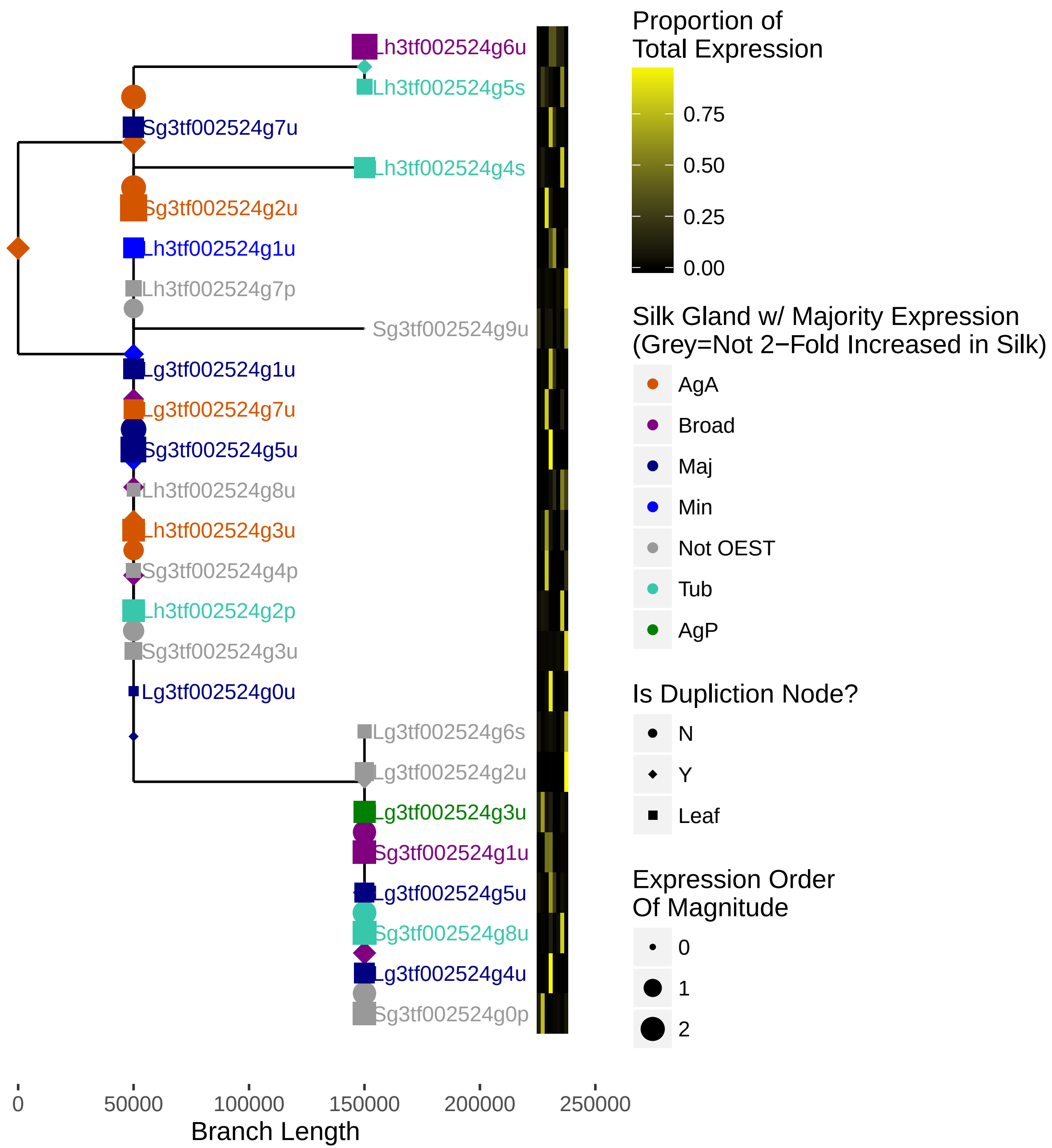
Lh3tf002524g6u
Lh3tf002524g5s
Sg3tf002524g7u
Lh3tf002524g4s
Sg3tf002524g2u
Lh3tf002524g1u
Lh3tf002524g7p
Sg3tf002524g9u
Lg3tf002524g1u
Lg3tf002524g7u
Sg3tf002524g5u
Lh3tf002524g8u
Lh3tf002524g3u
Sg3tf002524g4p
Lh3tf002524g2p
Sg3tf002524g3u
Lg3tf002524g0u
Lg3tf002524g6s
Lg3tf002524g2u
Lg3tf002524g3u
Sg3tf002524g1u
Lg3tf002524g5u
Sg3tf002524g8u
Lg3tf002524g4u
Sg3tf002524g0p
0
50000
100000
150000
200000
250000
Branch Length
Proportion of Total Expression
0.75
0.50
0.25
0.00
Silk Gland w/ Majority Expression (Grey=Not 2−Fold Increased in Silk)
AgA
Broad
Maj
Min
Not OEST
Tub
AgP
Is Dupliction Node?
N
Y
Leaf
Expression Order Of Magnitude
0
1
2

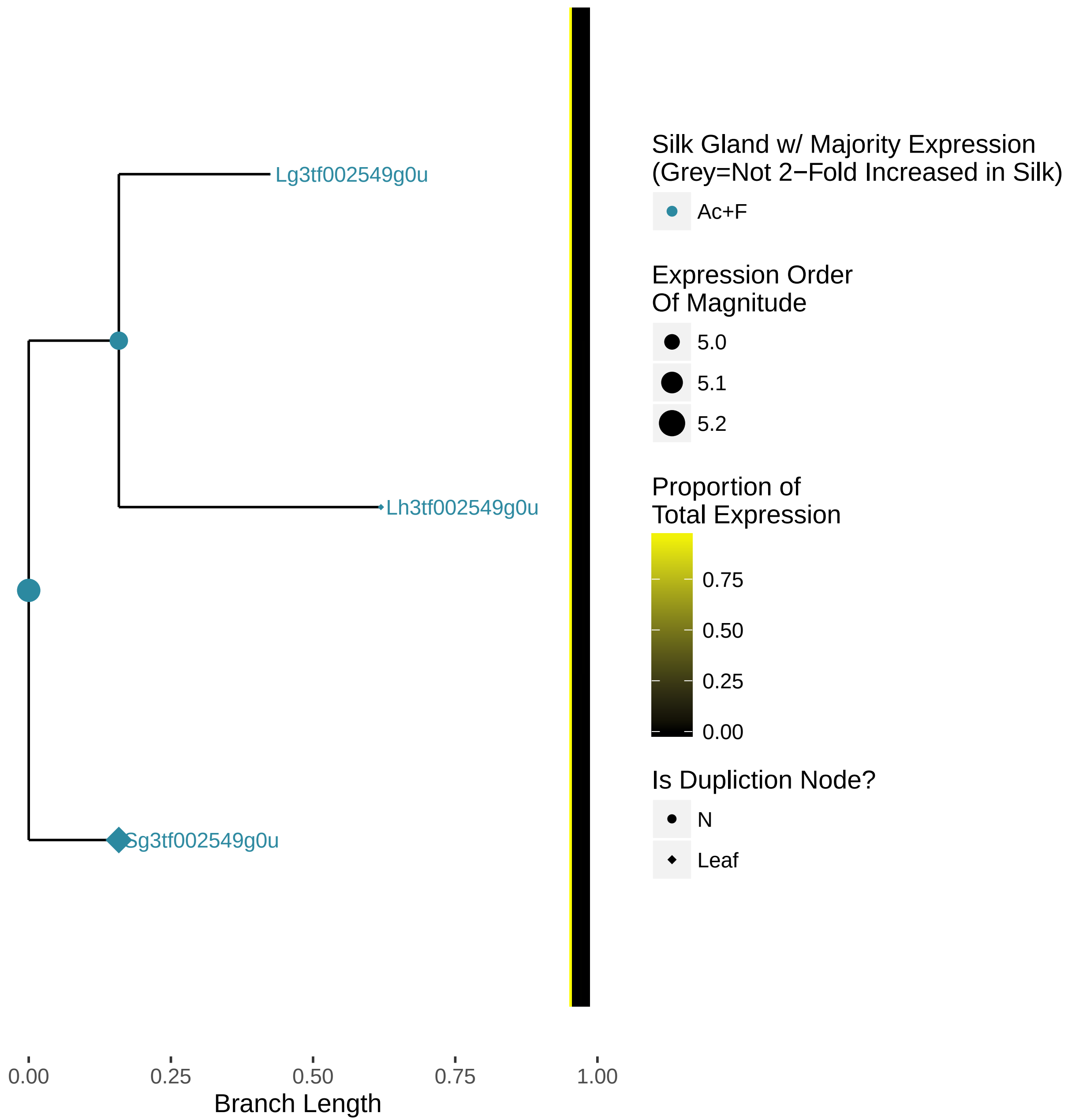

Lg3tf002549g0u
Lh3tf002549g0u
Sg3tf002549g0u
0.00
0.25
0.50
0.75
1.00
Branch Length
Silk Gland w/ Majority Expression
(Grey=Not 2−Fold Increased in Silk)
Ac+F
Expression Order
Of Magnitude
5.0
5.1
5.2
Proportion of
Total Expression
0.75
0.50
0.25
0.00
Is Dupliction Node?
N
Leaf

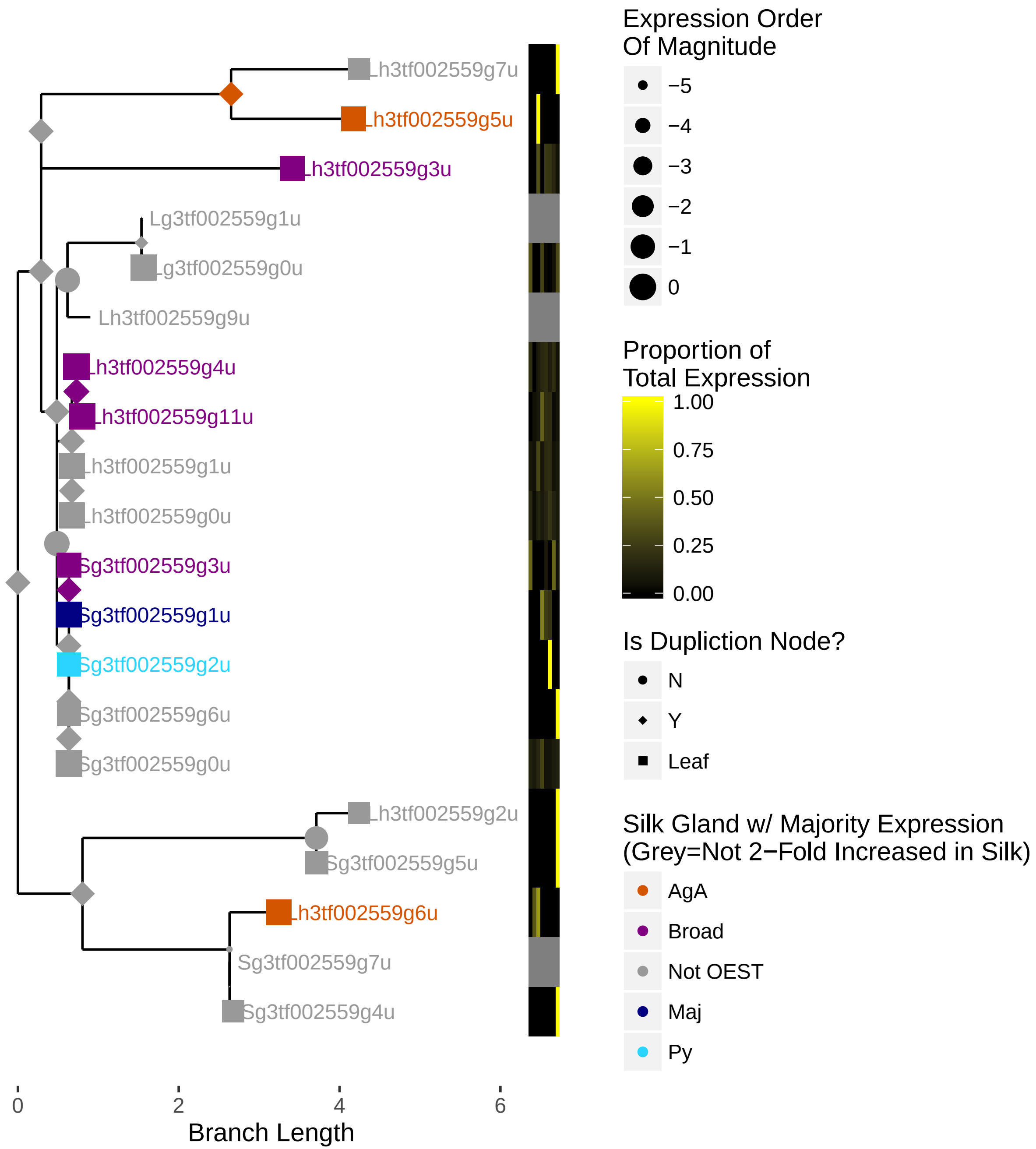

Lh3tf002559g7u
Lh3tf002559g5u
h3tf002559g3u
Lg3tf002559g1u
g3tf002559g0u
Lh3tf002559g9u
h3tf002559g4u
h3tf002559g11u
h3tf002559g1u
h3tf002559g0u
Sg3tf002559g3u
Sg3tf002559g1u
Sg3tf002559g2u
Sg3tf002559g6u
Sg3tf002559g0u
Lh3tf002559g2u
Sg3tf002559g5u
h3tf002559g6u
Sg3tf002559g7u
Sg3tf002559g4u
0
2
4
6
Branch Length
Expression Order
Of Magnitude
−5
−4
−3
−2
−1
0
Proportion of
Total Expression
1.00
0.75
0.50
0.25
0.00
Is Dupliction Node?
N
Y
Leaf
Silk Gland w/ Majority Expression
(Grey=Not 2−Fold Increased in Silk)
AgA
Broad
Not OEST
Maj
Py

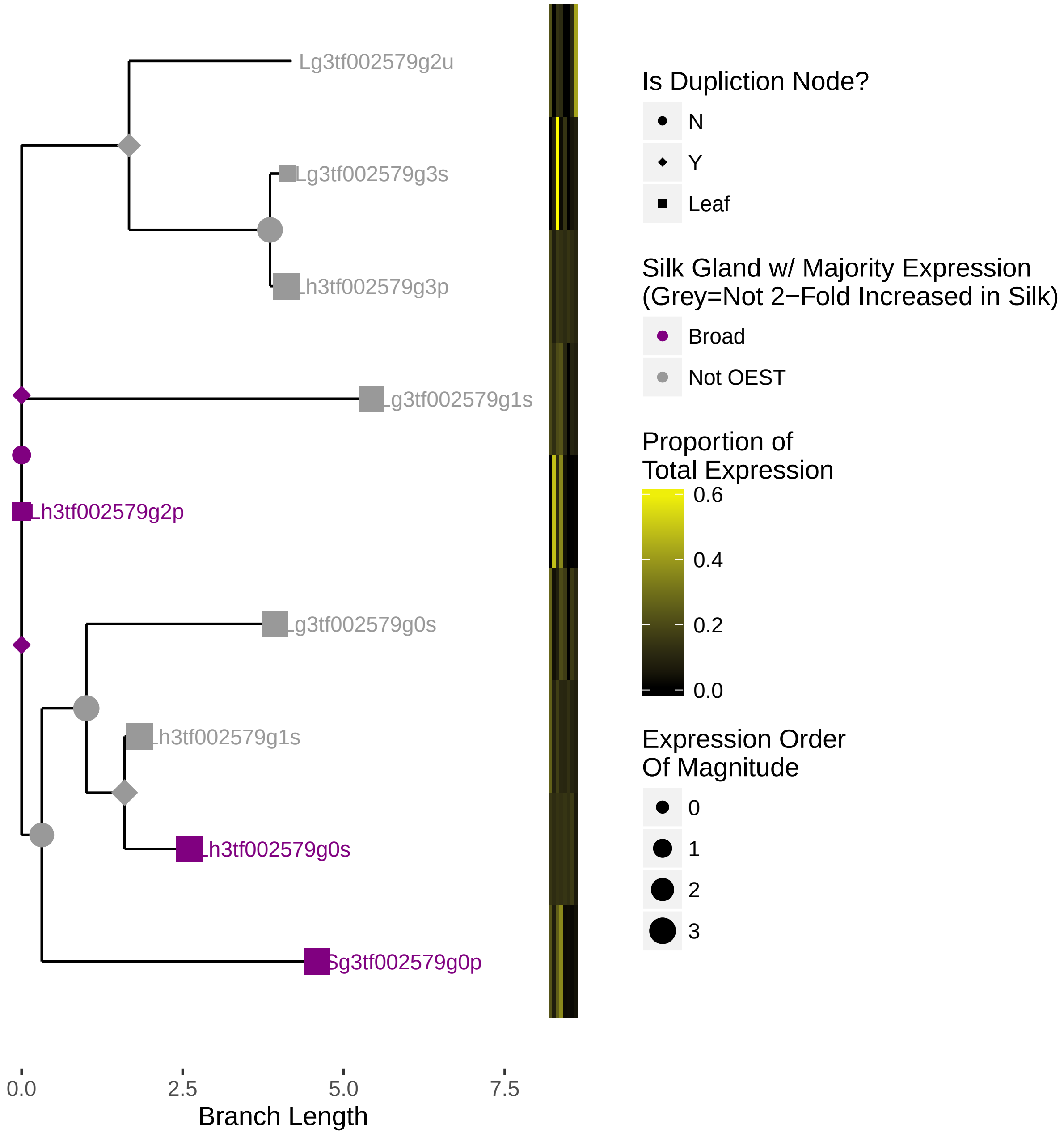

Lg3tf002579g2u
Lg3tf002579g3s
Lh3tf002579g3p
Lg3tf002579g1s
Lh3tf002579g2p
Lg3tf002579g0s
Lh3tf002579g1s
Lh3tf002579g0s
Sg3tf002579g0p
0.0
2.5
5.0
7.5
Branch Length
Is Dupliction Node?
N
Y
Leaf
Silk Gland w/ Majority Expression
(Grey=Not 2−Fold Increased in Silk)
Broad
Not OEST
Proportion of
Total Expression
0.6
0.4
0.2
0.0
Expression Order
Of Magnitude
0
1
2
3

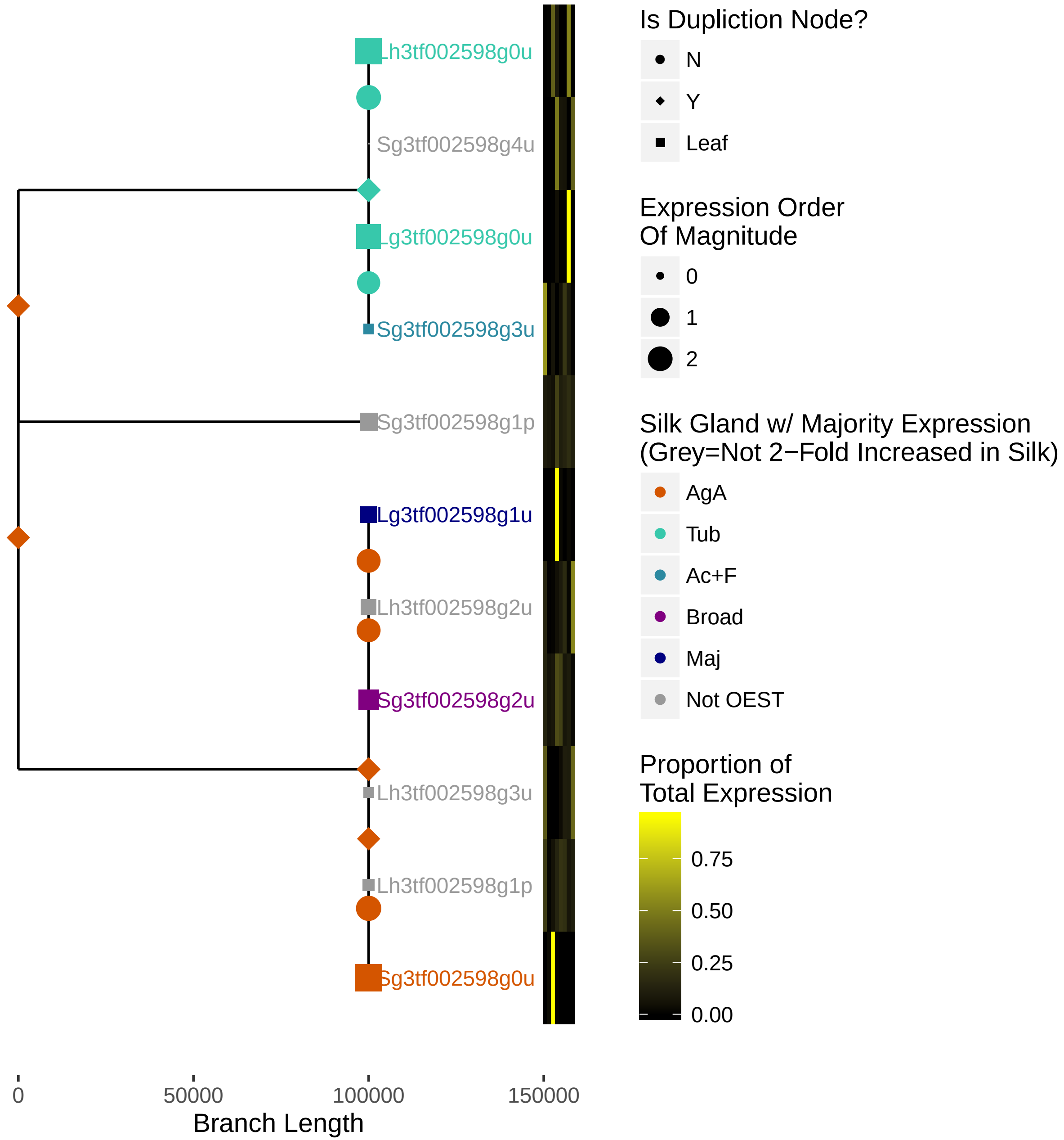
Lh3tf002598g0u
Sg3tf002598g4u
Lg3tf002598g0u
Sg3tf002598g3u
Sg3tf002598g1p
Lg3tf002598g1u
Lh3tf002598g2u
Sg3tf002598g2u
Lh3tf002598g3u
Lh3tf002598g1p
Sg3tf002598g0u
0
50000
100000
150000
Branch Length
Is Dupliction Node?
N
Y
Leaf
Expression Order Of Magnitude
0
1
2
Silk Gland w/ Majority Expression (Grey=Not 2−Fold Increased in Silk)
AgA
Tub
Ac+F
Broad
Maj
Not OEST
Proportion of Total Expression
0.75
0.50
0.25
0.00

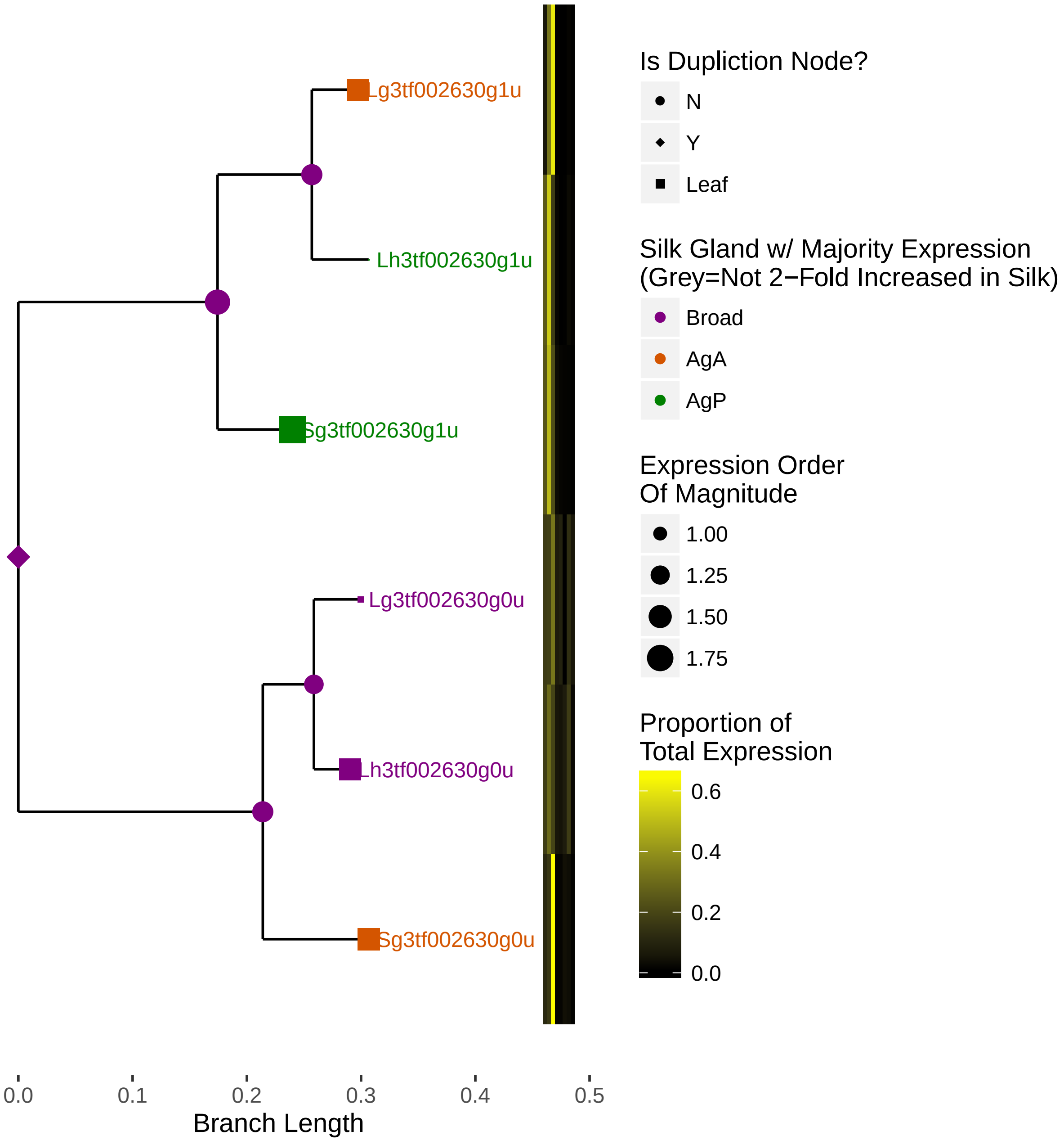
Lg3tf002630g1u
Lh3tf002630g1u
Sg3tf002630g1u
Lg3tf002630g0u
Lh3tf002630g0u
Sg3tf002630g0u
0.0
0.1
0.2
0.3
0.4
0.5
Branch Length
Is Dupliction Node?
N
Y
Leaf
Silk Gland w/ Majority Expression
(Grey=Not 2−Fold Increased in Silk)
Broad
AgA
AgP
Expression Order
Of Magnitude
1.00
1.25
1.50
1.75
Proportion of
Total Expression
0.6
0.4
0.2
0.0

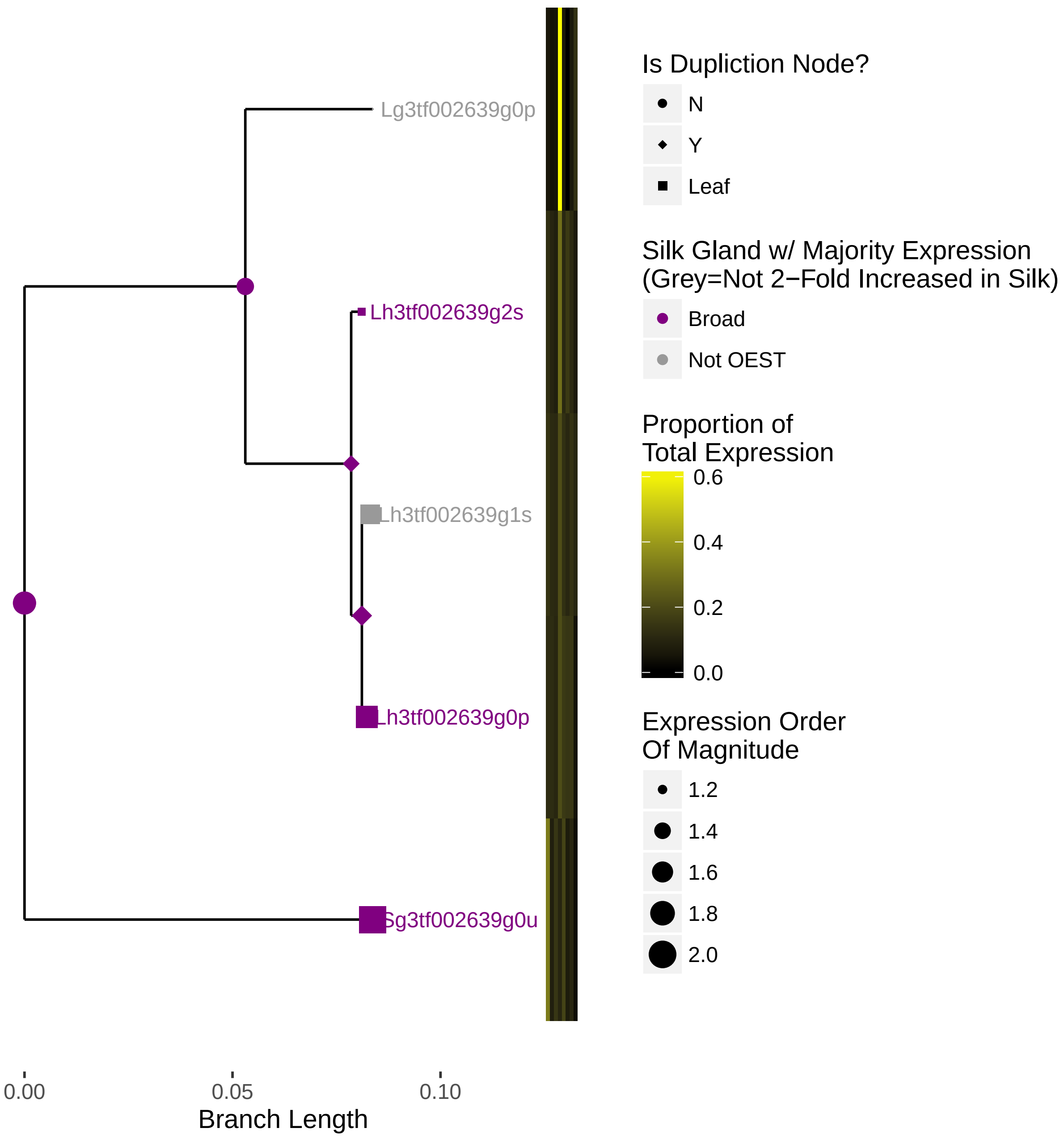

Lg3tf002639g0p
Lh3tf002639g2s
Lh3tf002639g1s
Lh3tf002639g0p
Sg3tf002639g0u
0.00
0.05
0.10
Branch Length
Is Dupliction Node?
N
Y
Leaf
Silk Gland w/ Majority Expression
(Grey=Not 2−Fold Increased in Silk)
Broad
Not OEST
Proportion of
Total Expression
0.6
0.4
0.2
0.0
Expression Order
Of Magnitude
1.2
1.4
1.6
1.8
2.0

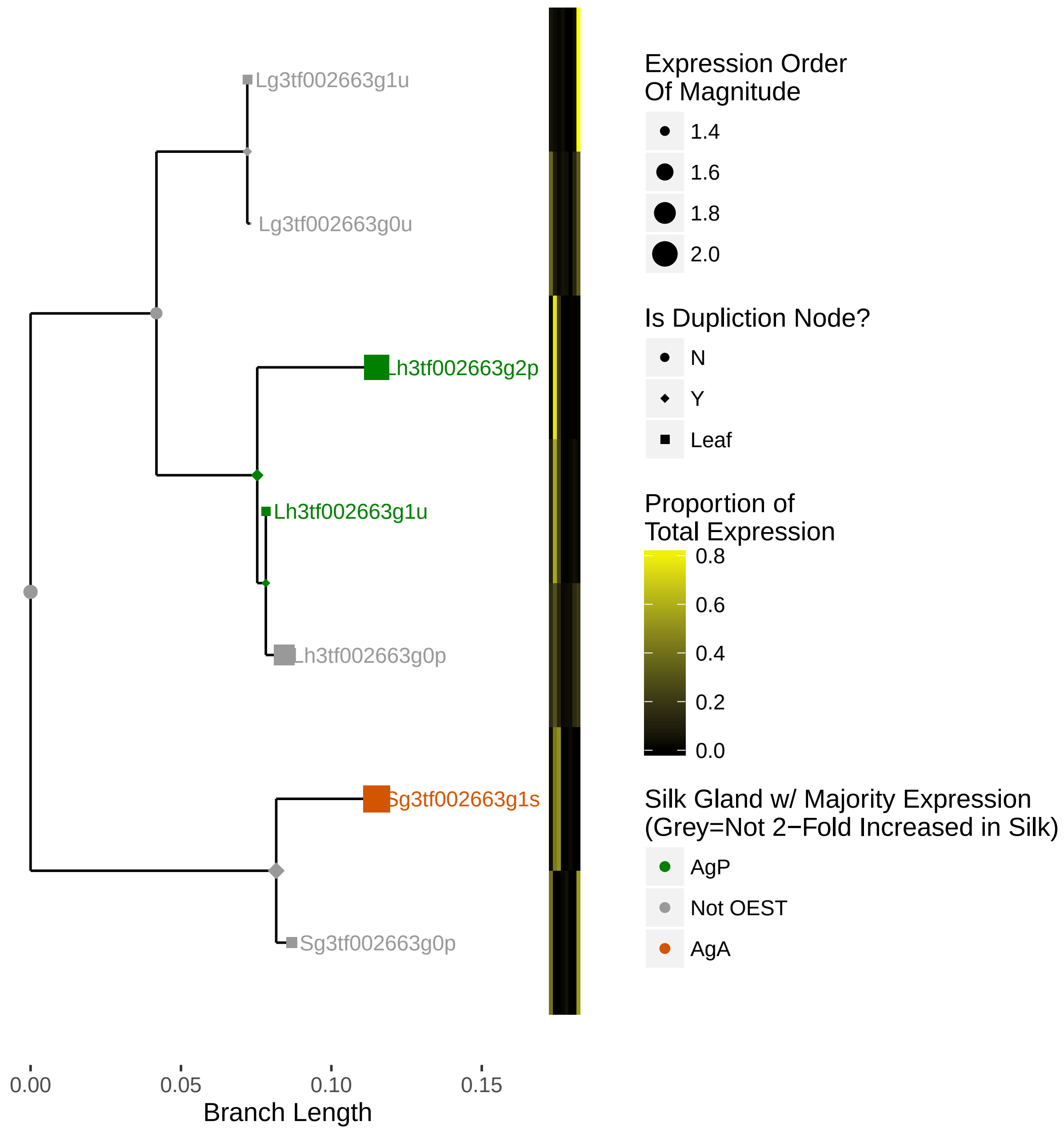

Lg3tf002663g1u
Lg3tf002663g0u
h3tf002663g2p
Lh3tf002663g1u
Lh3tf002663g0p
Sg3tf002663g1s
Sg3tf002663g0p
0.00
0.05
0.10
0.15
Branch Length
Expression Order Of Magnitude
1.4
1.6
1.8
2.0
Is Dupliction Node?
N
Y
Leaf
Proportion of Total Expression
0.8
0.6
0.4
0.2
0.0
Silk Gland w/ Majority Expression (Grey=Not 2−Fold Increased in Silk)
AgP
Not OEST
AgA

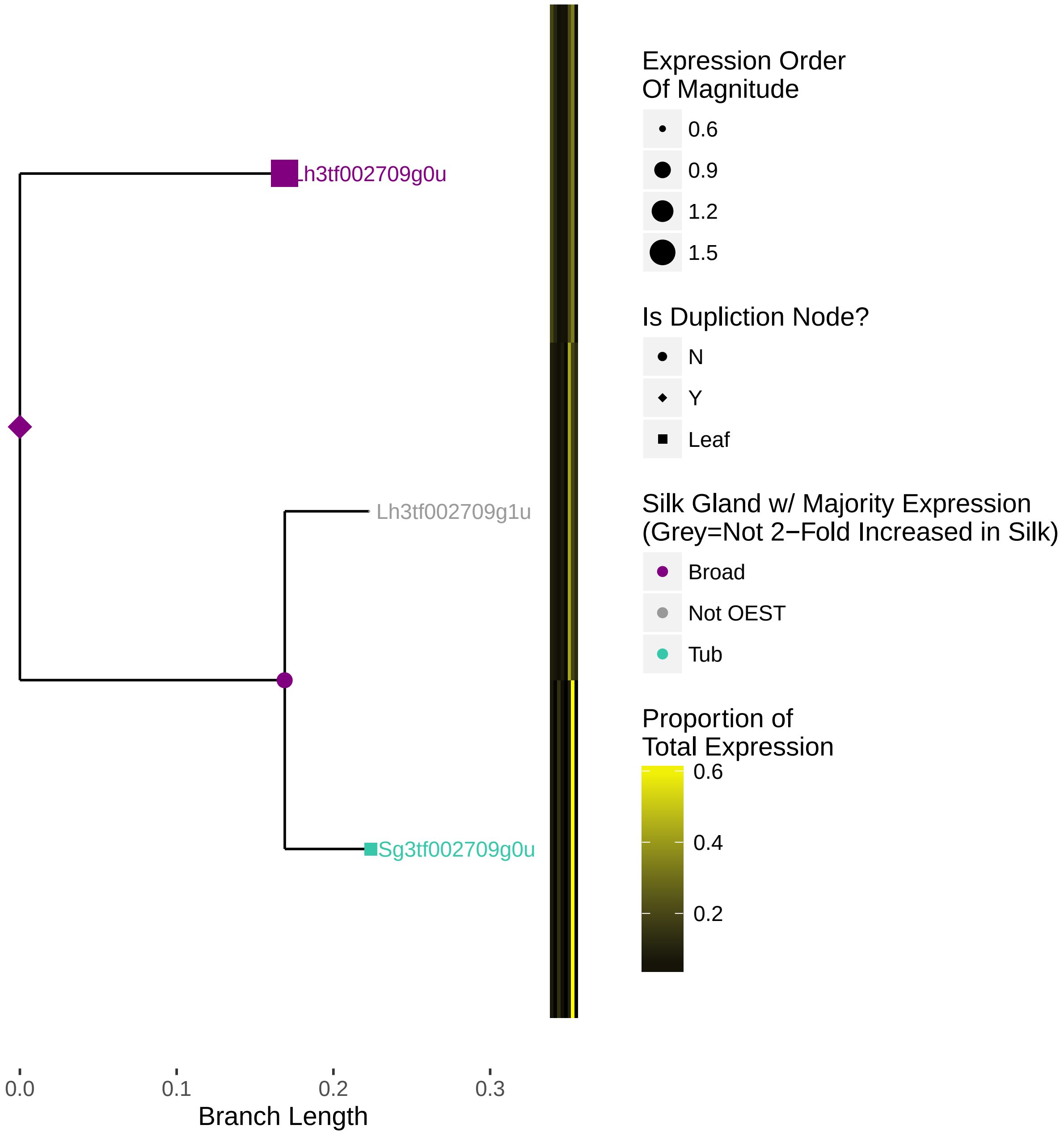

Lh3tf002709g0u
Lh3tf002709g1u
Sg3tf002709g0u
0.0
0.1
0.2
0.3
Branch Length
Expression Order Of Magnitude
0.6
0.9
1.2
1.5
Is Dupliction Node?
N
Y
Leaf
Silk Gland w/ Majority Expression (Grey=Not 2−Fold Increased in Silk)
Broad
Not OEST
Tub
Proportion of Total Expression
0.6
0.4
0.2

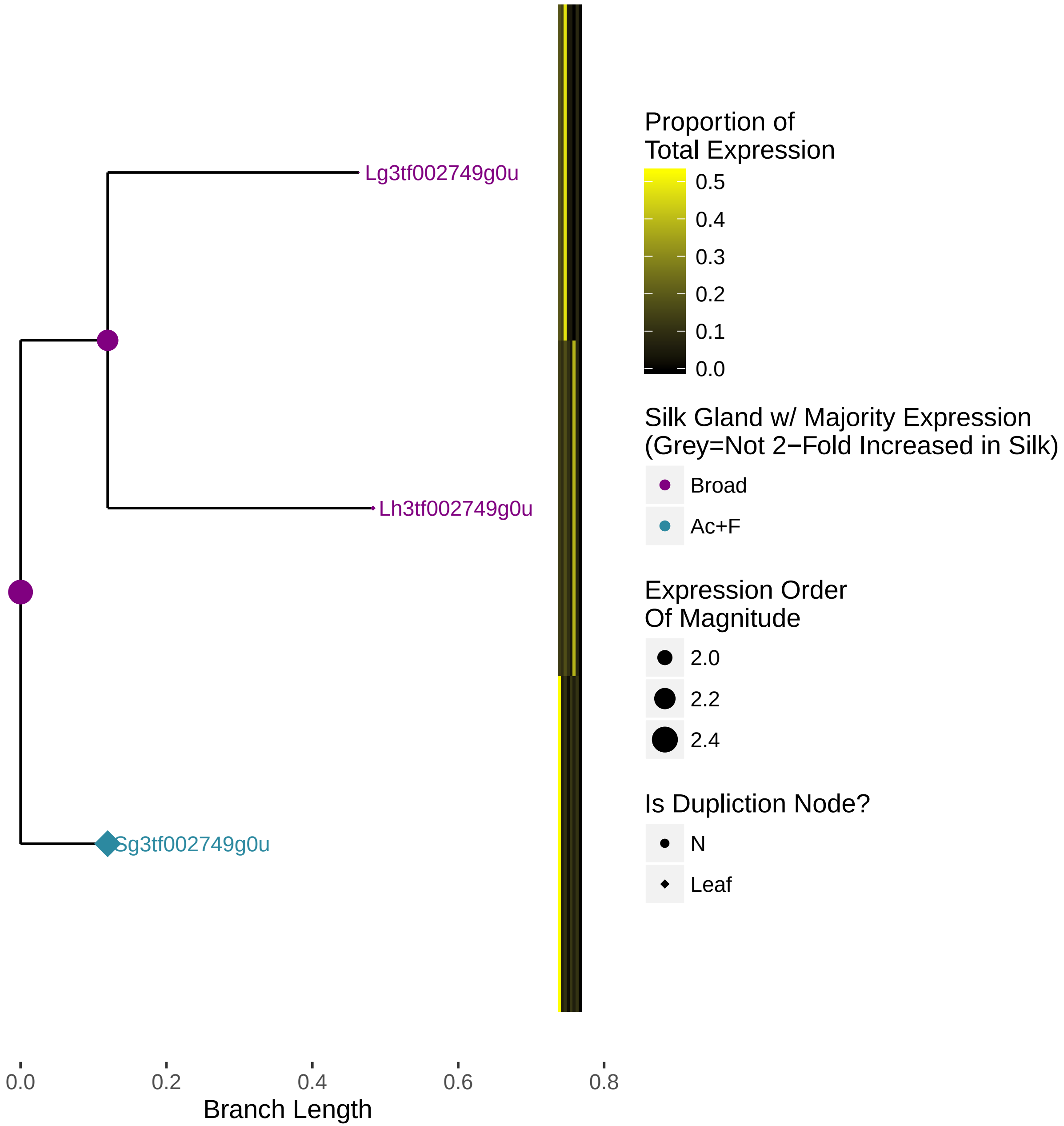

Lg3tf002749g0u
Lh3tf002749g0u
Sg3tf002749g0u
Proportion of
Total Expression
0.5
0.4
0.3
0.2
0.1
0.0
Silk Gland w/ Majority Expression
(Grey=Not 2−Fold Increased in Silk)
Broad
Ac+F
Expression Order
Of Magnitude
2.0
2.2
2.4
Is Dupliction Node?
N
Leaf
0.0
0.2
0.4
0.6
0.8
Branch Length

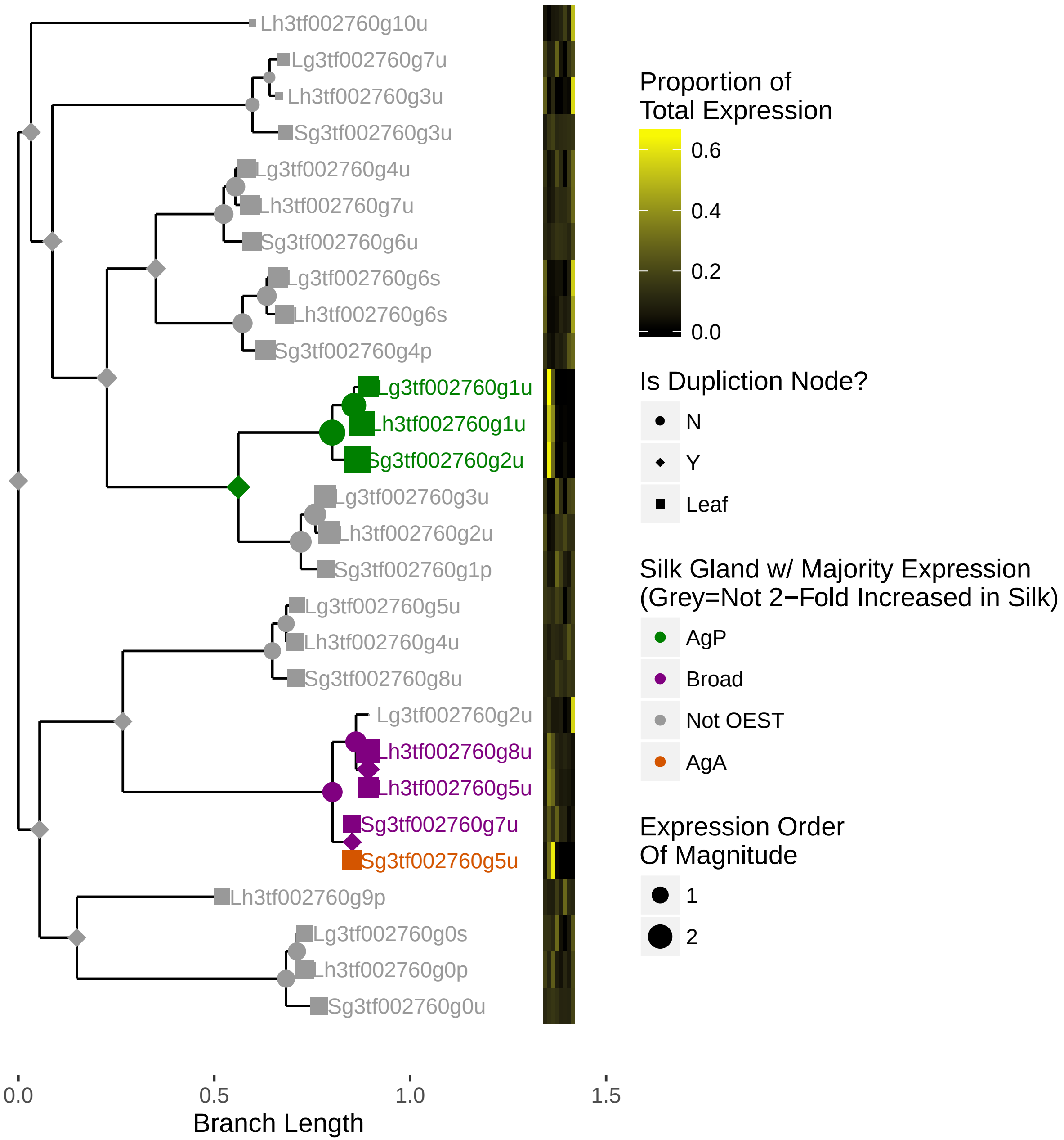

Lh3tf002760g10u
Lg3tf002760g7u
Lh3tf002760g3u
Sg3tf002760g3u
Lg3tf002760g4u
Lh3tf002760g7u
Sg3tf002760g6u
Lg3tf002760g6s
Lh3tf002760g6s
Sg3tf002760g4p
Lg3tf002760g1u
Lh3tf002760g1u
Sg3tf002760g2u
Lg3tf002760g3u
Lh3tf002760g2u
Sg3tf002760g1p
Lg3tf002760g5u
Lh3tf002760g4u
Sg3tf002760g8u
Lg3tf002760g2u
Lh3tf002760g8u
Lh3tf002760g5u
Sg3tf002760g7u
Sg3tf002760g5u
Lh3tf002760g9p
Lg3tf002760g0s
Lh3tf002760g0p
Sg3tf002760g0u
0.0
0.5
1.0
1.5
Branch Length
Proportion of
Total Expression
0.6
0.4
0.2
0.0
Is Dupliction Node?
N
Y
Leaf
Silk Gland w/ Majority Expression
(Grey=Not 2−Fold Increased in Silk)
AgP
Broad
Not OEST
AgA
Expression Order
Of Magnitude
1
2

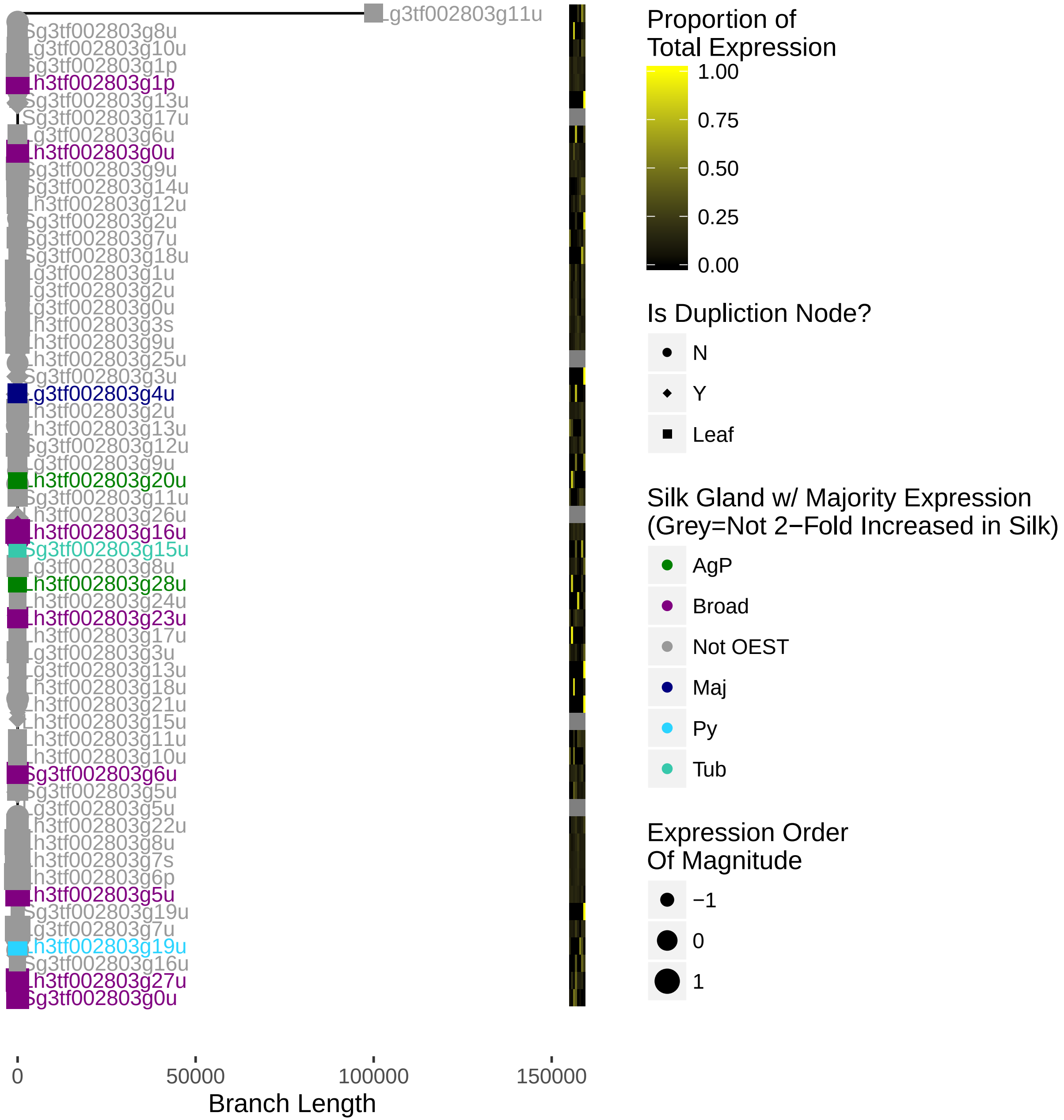

Lg3tf002803g11u
Sg3tf002803g8u
Lg3tf002803g10u
Sg3tf002803g1p
Lh3tf002803g1p
Sg3tf002803g13u
Sg3tf002803g17u
Lg3tf002803g6u
Lh3tf002803g0u
Sg3tf002803g9u
Sg3tf002803g14u
Lh3tf002803g12u
Sg3tf002803g2u
Sg3tf002803g7u
Sg3tf002803g18u
Lg3tf002803g1u
Lg3tf002803g2u
Lg3tf002803g0u
Lh3tf002803g3s
Lh3tf002803g9u
Lh3tf002803g25u
Sg3tf002803g3u
Lg3tf002803g4u
Lh3tf002803g2u
Lh3tf002803g13u
Sg3tf002803g12u
Lg3tf002803g9u
Lh3tf002803g20u
Sg3tf002803g11u
Lh3tf002803g26u
Lh3tf002803g16u
Sg3tf002803g15u
Lg3tf002803g8u
Lh3tf002803g28u
Lh3tf002803g24u
Lh3tf002803g23u
Lh3tf002803g17u
Lg3tf002803g3u
Lg3tf002803g13u
Lh3tf002803g18u
Lh3tf002803g21u
Lh3tf002803g15u
Lh3tf002803g11u
Lh3tf002803g10u
Sg3tf002803g6u
Sg3tf002803g5u
Lg3tf002803g5u
Lh3tf002803g22u
Lh3tf002803g8u
Lh3tf002803g7s
Lh3tf002803g6p
Lh3tf002803g5u
Sg3tf002803g19u
Lg3tf002803g7u
Lh3tf002803g19u
Sg3tf002803g16u
Lh3tf002803g27u
Sg3tf002803g0u
0
50000
100000
150000
Branch Length
Proportion of Total Expression
1.00
0.75
0.50
0.25
0.00
Is Dupliction Node?
N
Y
Leaf
Silk Gland w/ Majority Expression (Grey=Not 2−Fold Increased in Silk)
AgP
Broad
Not OEST
Maj
Py
Tub
Expression Order Of Magnitude
−1
0
1

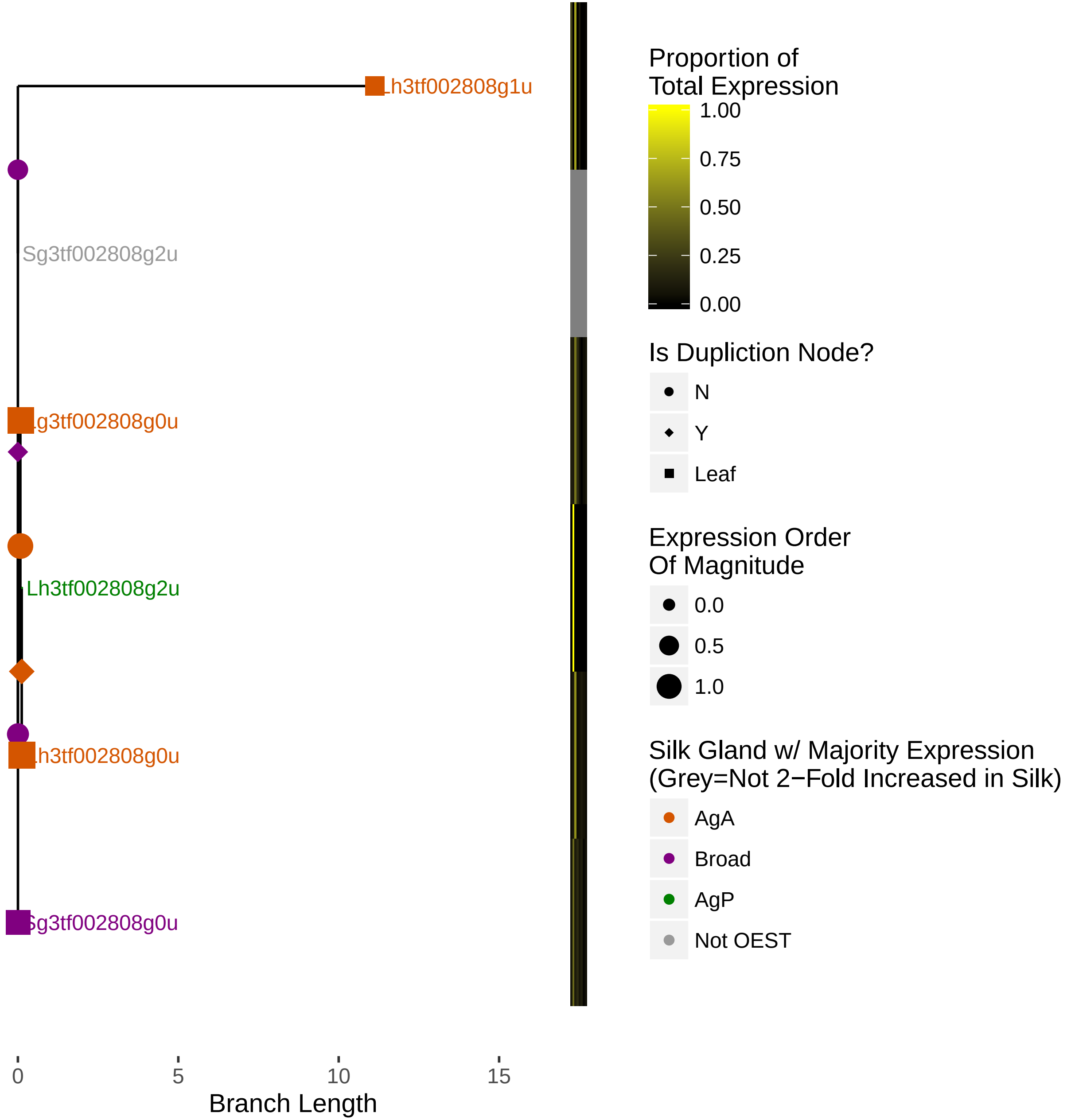

h3tf002808g1u
Sg3tf002808g2u
g3tf002808g0u
Lh3tf002808g2u
h3tf002808g0u
g3tf002808g0u
0
5
10
15
Branch Length
Proportion of
Total Expression
1.00
0.75
0.50
0.25
0.00
Is Dupliction Node?
N
Y
Leaf
Expression Order
Of Magnitude
0.0
0.5
1.0
Silk Gland w/ Majority Expression
(Grey=Not 2−Fold Increased in Silk)
AgA
Broad
AgP
Not OEST

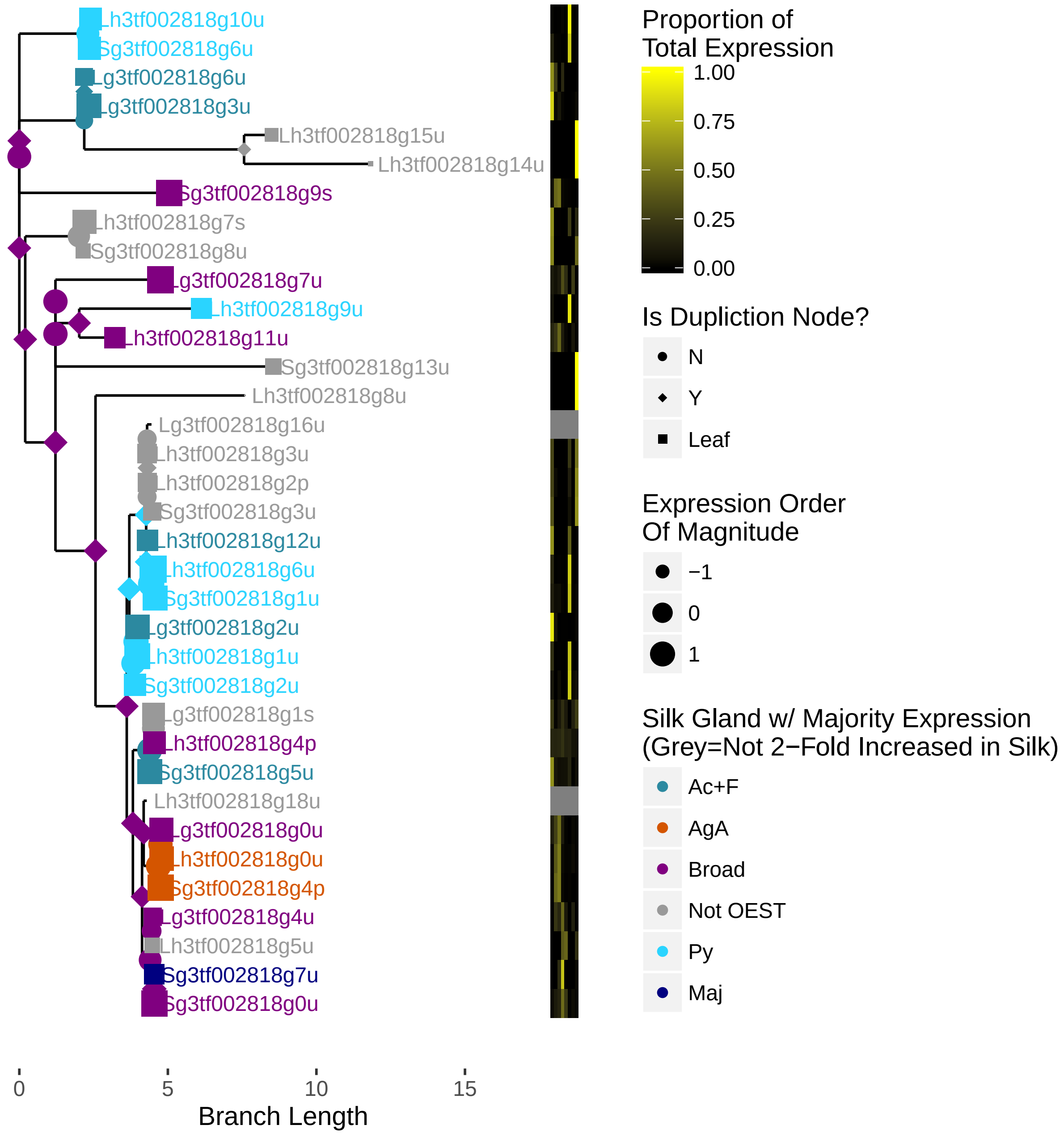

Lh3tf002818g10u
Sg3tf002818g6u
Lg3tf002818g6u
Lg3tf002818g3u
Lh3tf002818g15u
Lh3tf002818g14u
Sg3tf002818g9s
Lh3tf002818g7s
Sg3tf002818g8u
Lg3tf002818g7u
Lh3tf002818g9u
Lh3tf002818g11u
Sg3tf002818g13u
Lh3tf002818g8u
Lg3tf002818g16u
Lh3tf002818g3u
Lh3tf002818g2p
Sg3tf002818g3u
Lh3tf002818g12u
Lh3tf002818g6u
Sg3tf002818g1u
Lg3tf002818g2u
Lh3tf002818g1u
Sg3tf002818g2u
Lg3tf002818g1s
Lh3tf002818g4p
Sg3tf002818g5u
Lh3tf002818g18u
Lg3tf002818g0u
Lh3tf002818g0u
Sg3tf002818g4p
Lg3tf002818g4u
Lh3tf002818g5u
Sg3tf002818g7u
Sg3tf002818g0u
0
5
10
15
Branch Length
Proportion of Total Expression
1.00
0.75
0.50
0.25
0.00
Is Dupliction Node?
N
Y
Leaf
Expression Order Of Magnitude
−1
0
1
Silk Gland w/ Majority Expression (Grey=Not 2−Fold Increased in Silk)
Ac+F
AgA
Broad
Not OEST
Py
Maj

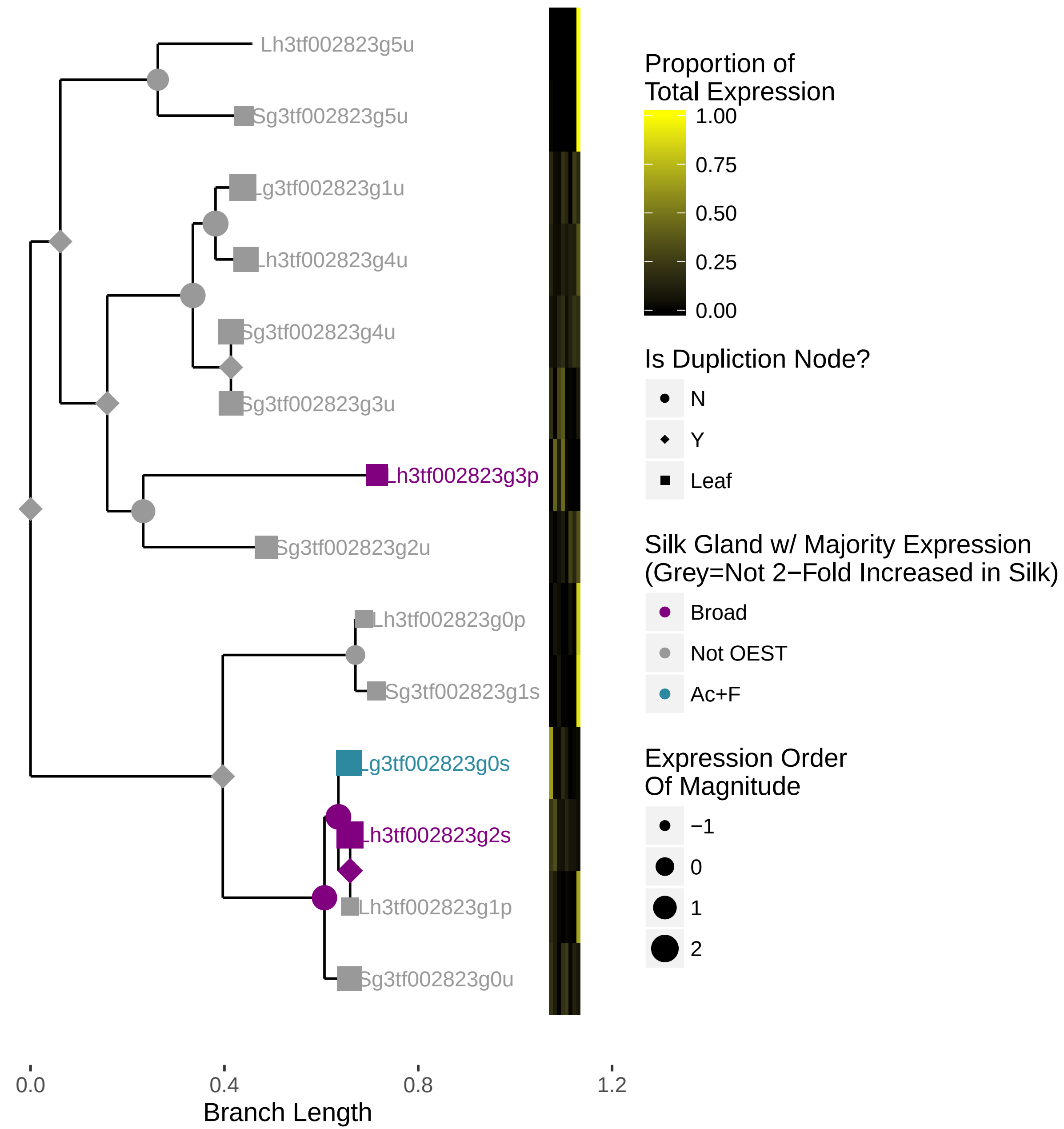

Lh3tf002823g5u
Sg3tf002823g5u
Lg3tf002823g1u
Lh3tf002823g4u
Sg3tf002823g4u
Sg3tf002823g3u
Lh3tf002823g3p
Sg3tf002823g2u
Lh3tf002823g0p
Sg3tf002823g1s
Lg3tf002823g0s
Lh3tf002823g2s
Lh3tf002823g1p
Sg3tf002823g0u
0.0
0.4
0.8
1.2
Branch Length
Proportion of
Total Expression
1.00
0.75
0.50
0.25
0.00
Is Dupliction Node?
N
Y
Leaf
Silk Gland w/ Majority Expression
(Grey=Not 2−Fold Increased in Silk)
Broad
Not OEST
Ac+F
Expression Order
Of Magnitude
−1
0
1
2

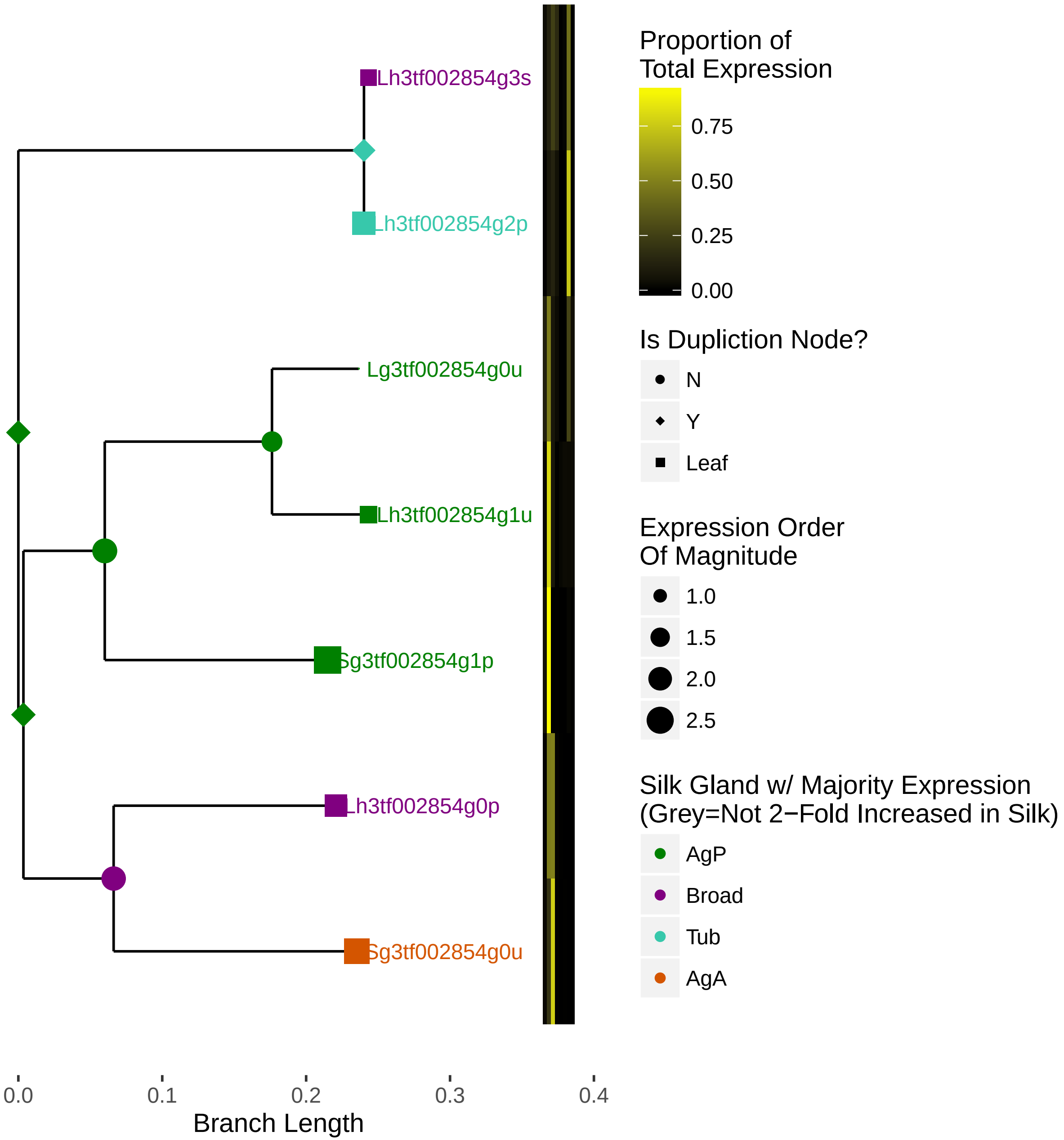
Lh3tf002854g3s
Lh3tf002854g2p
Lg3tf002854g0u
Lh3tf002854g1u
Sg3tf002854g1p
Lh3tf002854g0p
Sg3tf002854g0u
0.0
0.1
0.2
0.3
0.4
Branch Length
Proportion of Total Expression
0.75
0.50
0.25
0.00
Is Dupliction Node?
N
Y
Leaf
Expression Order Of Magnitude
1.0
1.5
2.0
2.5
Silk Gland w/ Majority Expression (Grey=Not 2−Fold Increased in Silk)
AgP
Broad
Tub
AgA

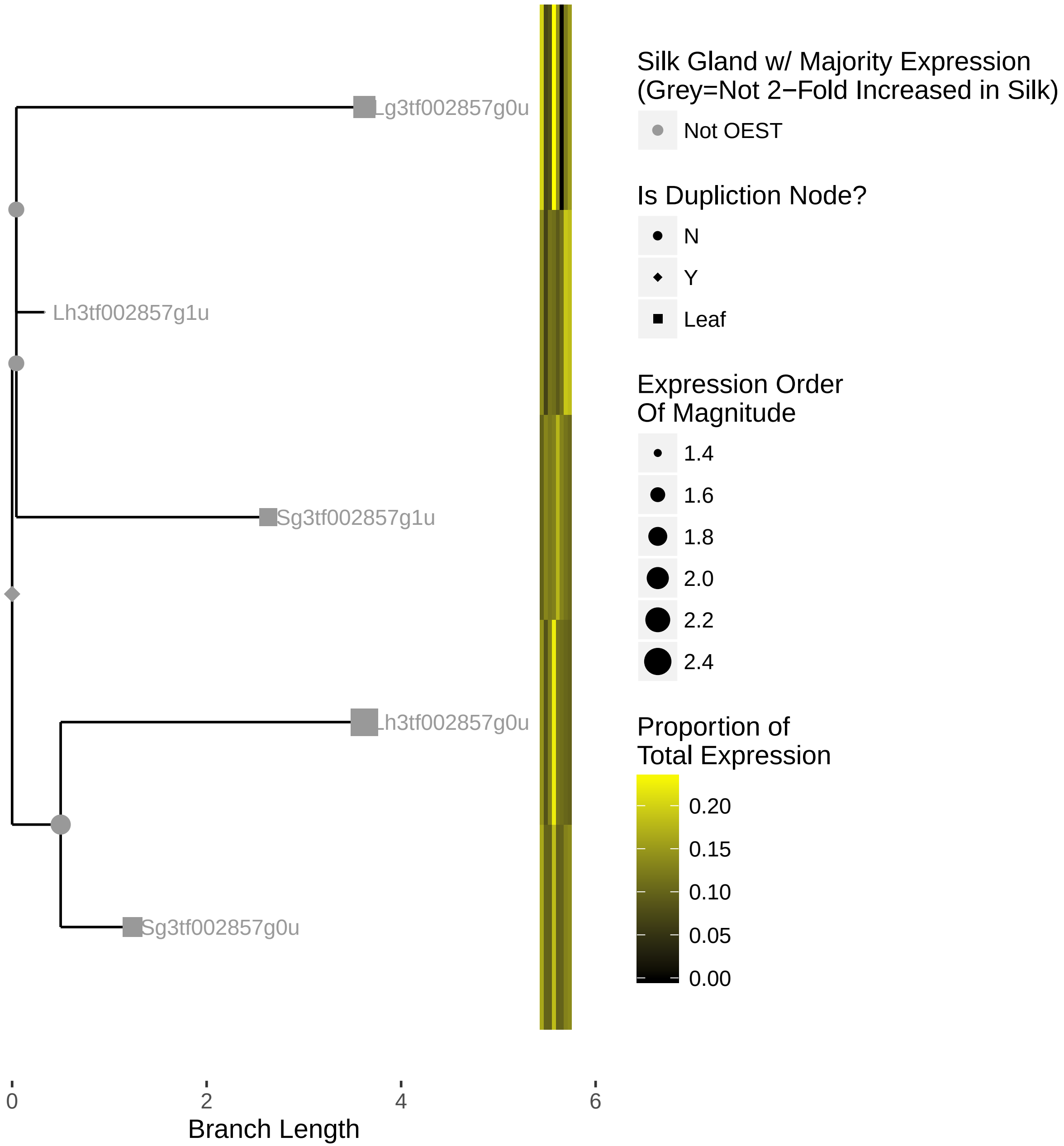
Lg3tf002857g0u
Lh3tf002857g1u
Sg3tf002857g1u
Lh3tf002857g0u
Sg3tf002857g0u
0
2
4
6
Branch Length
Silk Gland w/ Majority Expression
(Grey=Not 2−Fold Increased in Silk)
Not OEST
Is Dupliction Node?
N
Y
Leaf
Expression Order
Of Magnitude
1.4
1.6
1.8
2.0
2.2
2.4
Proportion of
Total Expression
0.20
0.15
0.10
0.05
0.00

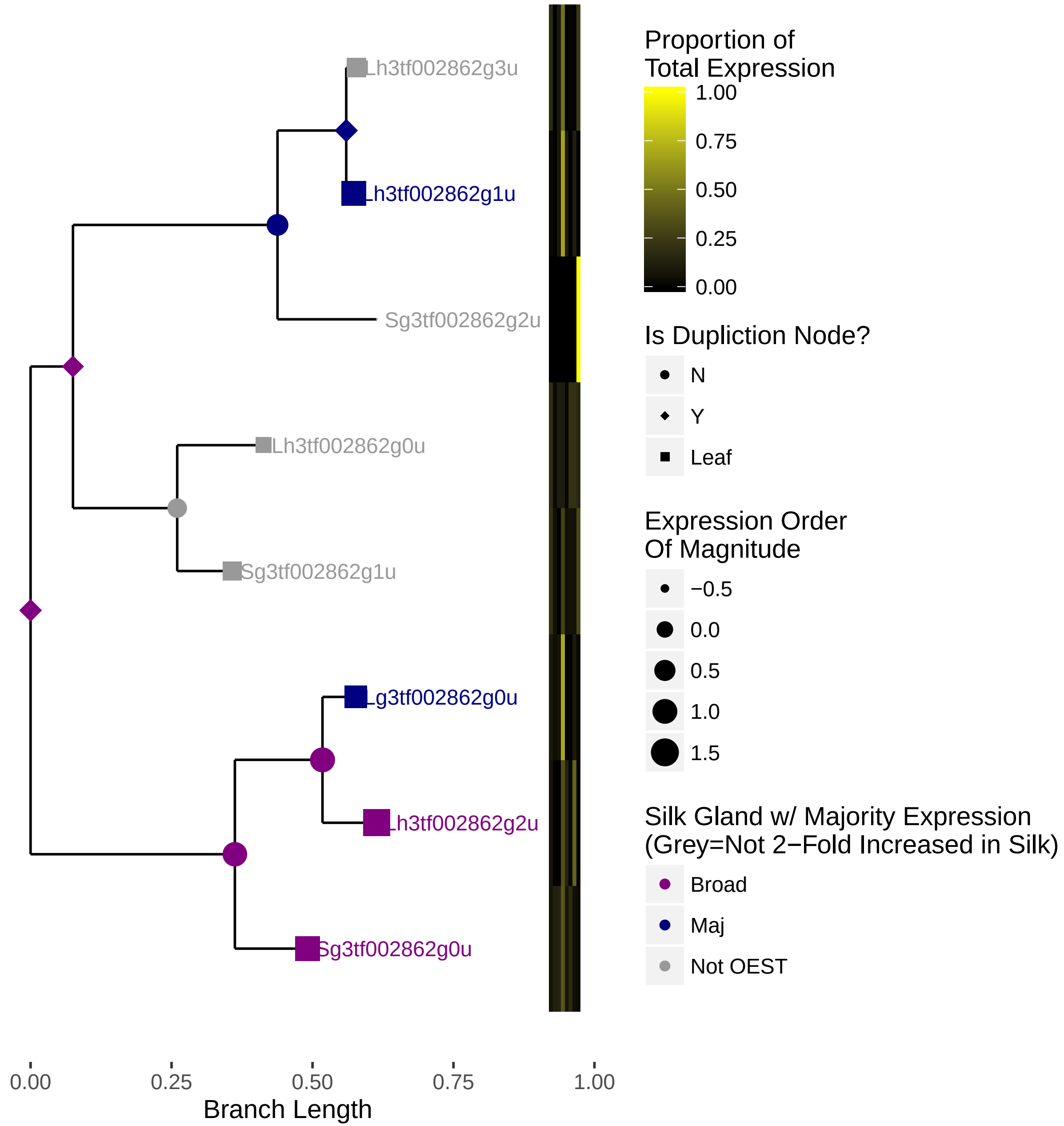

Lh3tf002862g3u
Lh3tf002862g1u
Sg3tf002862g2u
Lh3tf002862g0u
Sg3tf002862g1u
Lg3tf002862g0u
Lh3tf002862g2u
Sg3tf002862g0u
0.00
0.25
0.50
0.75
1.00
Branch Length
Proportion of
Total Expression
1.00
0.75
0.50
0.25
0.00
Is Dupliction Node?
N
Y
Leaf
Expression Order
Of Magnitude
−0.5
0.0
0.5
1.0
1.5
Silk Gland w/ Majority Expression
(Grey=Not 2−Fold Increased in Silk)
Broad
Maj
Not OEST

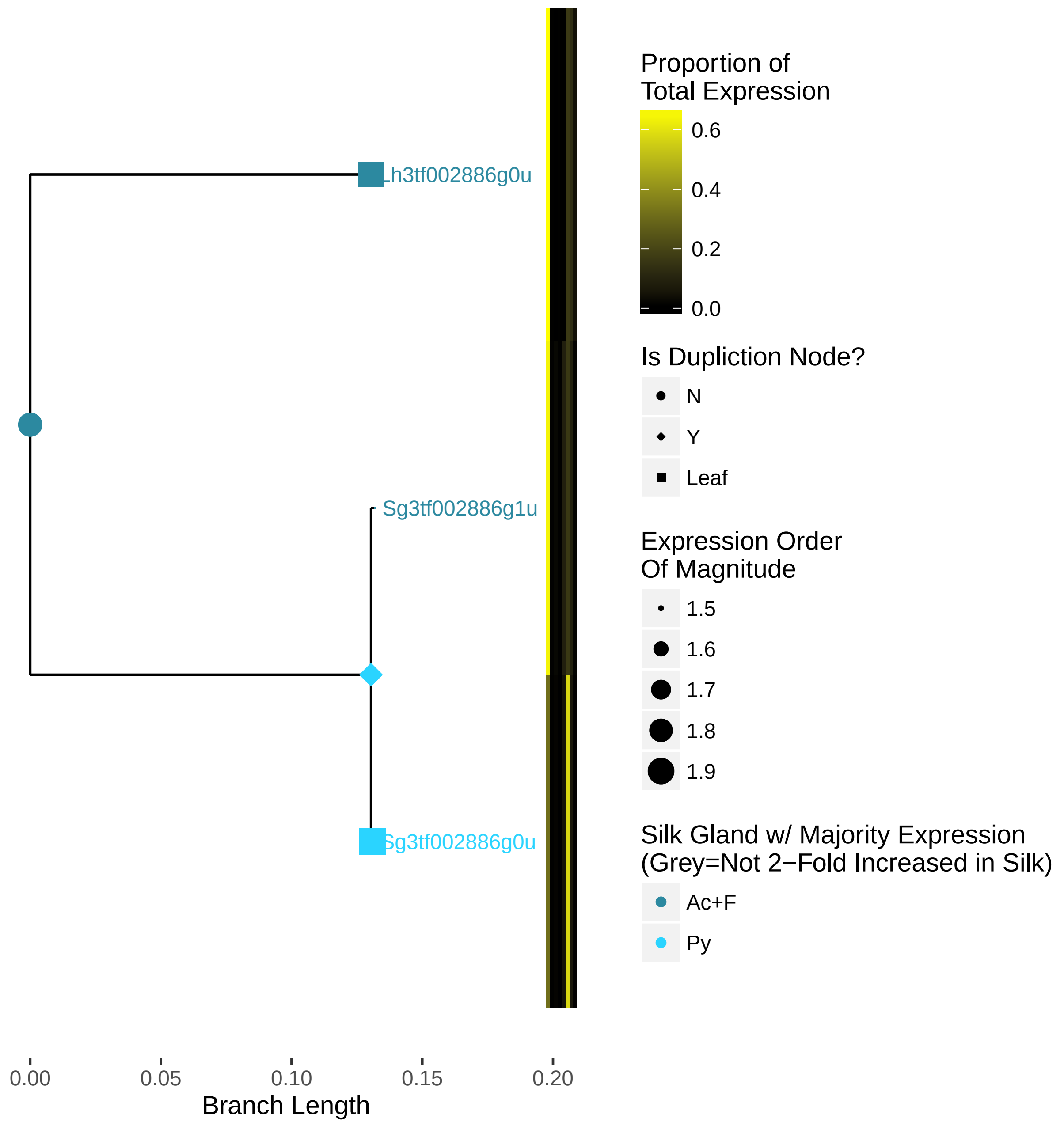

Lh3tf002886g0u
Sg3tf002886g1u
Sg3tf002886g0u
0.00
0.05
0.10
0.15
0.20
Branch Length
Proportion of
Total Expression
0.6
0.4
0.2
0.0
Is Dupliction Node?
N
Y
Leaf
Expression Order
Of Magnitude
1.5
1.6
1.7
1.8
1.9
Silk Gland w/ Majority Expression
(Grey=Not 2−Fold Increased in Silk)
Ac+F
Py

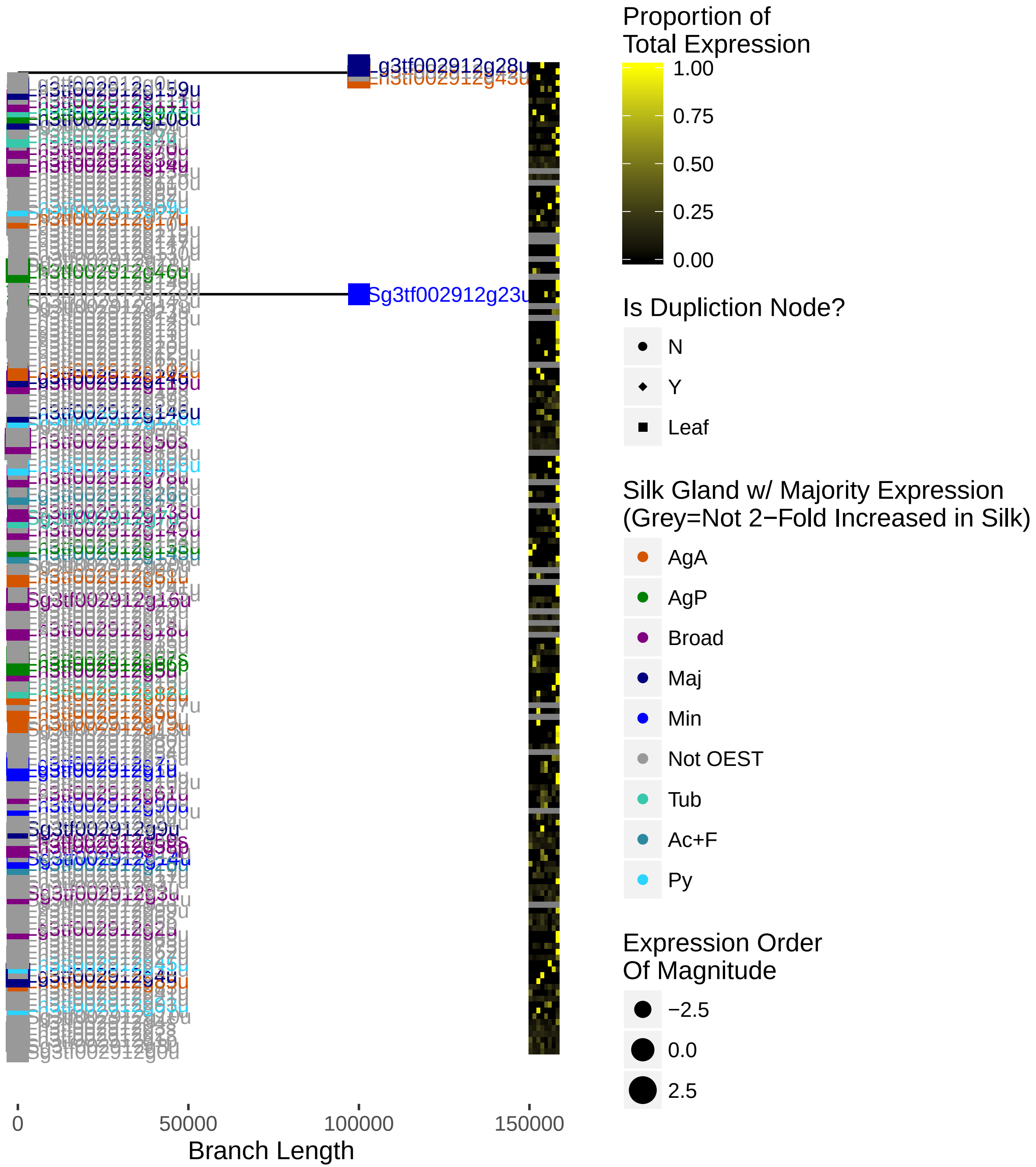

Proportion of Total Expression
1.00
0.75
0.50
0.25
0.00
Is Dupliction Node?
N
Y
Leaf
Silk Gland w/ Majority Expression (Grey=Not 2−Fold Increased in Silk)
AgA
AgP
Broad
Maj
Min
Not OEST
Tub
Ac+F
Py
Expression Order Of Magnitude
−2.5
0.0
2.5
Sg3tf002912g23u
Sg3tf002912g16u
Sg3tf002912g9u
Sg3tf002912g3u
Sg3tf002912g0u
0
50000
100000
150000
Branch Length

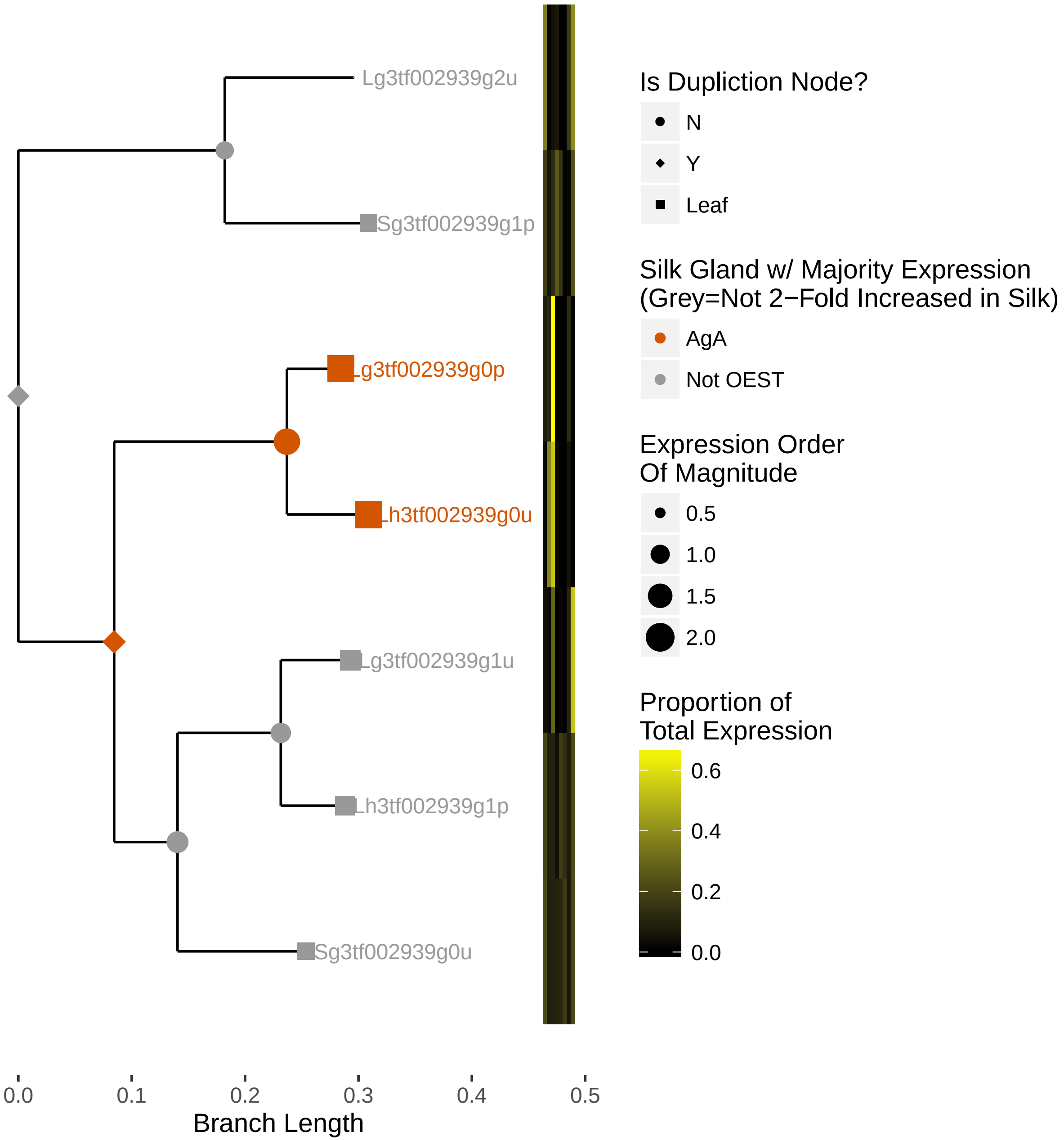

Lg3tf002939g2u
Sg3tf002939g1p
Lg3tf002939g0p
Lh3tf002939g0u
Lg3tf002939g1u
Lh3tf002939g1p
Sg3tf002939g0u
0.0
0.1
0.2
0.3
0.4
0.5
Branch Length
Is Dupliction Node?
N
Y
Leaf
Silk Gland w/ Majority Expression
(Grey=Not 2−Fold Increased in Silk)
AgA
Not OEST
Expression Order
Of Magnitude
0.5
1.0
1.5
2.0
Proportion of
Total Expression
0.6
0.4
0.2
0.0

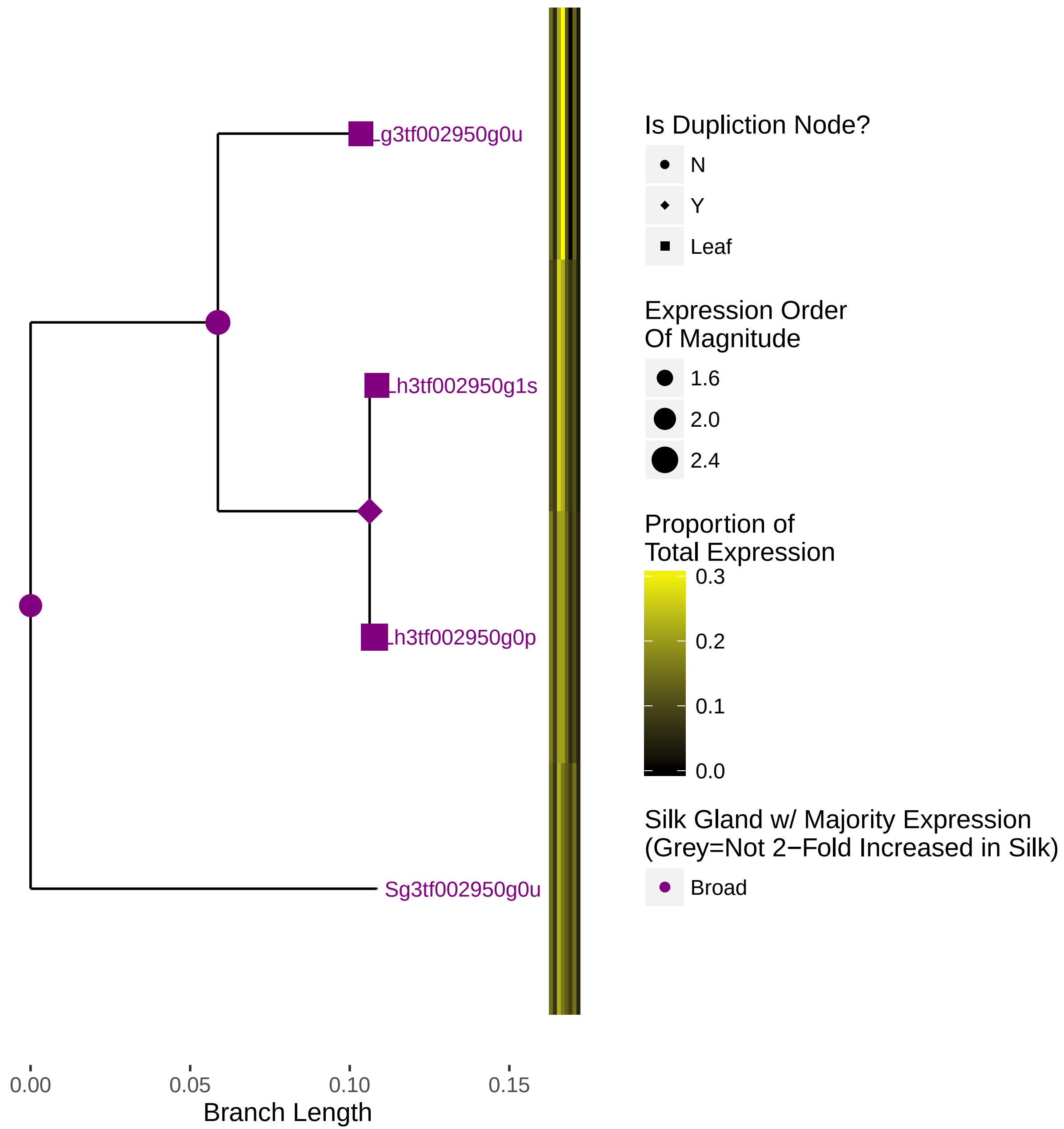

Lg3tf002950g0u
Lh3tf002950g1s
Lh3tf002950g0p
Sg3tf002950g0u
0.00
0.05
0.10
0.15
Branch Length
Is Dupliction Node?
N
Y
Leaf
Expression Order
Of Magnitude
1.6
2.0
2.4
Proportion of
Total Expression
0.3
0.2
0.1
0.0
Silk Gland w/ Majority Expression
(Grey=Not 2−Fold Increased in Silk)
Broad

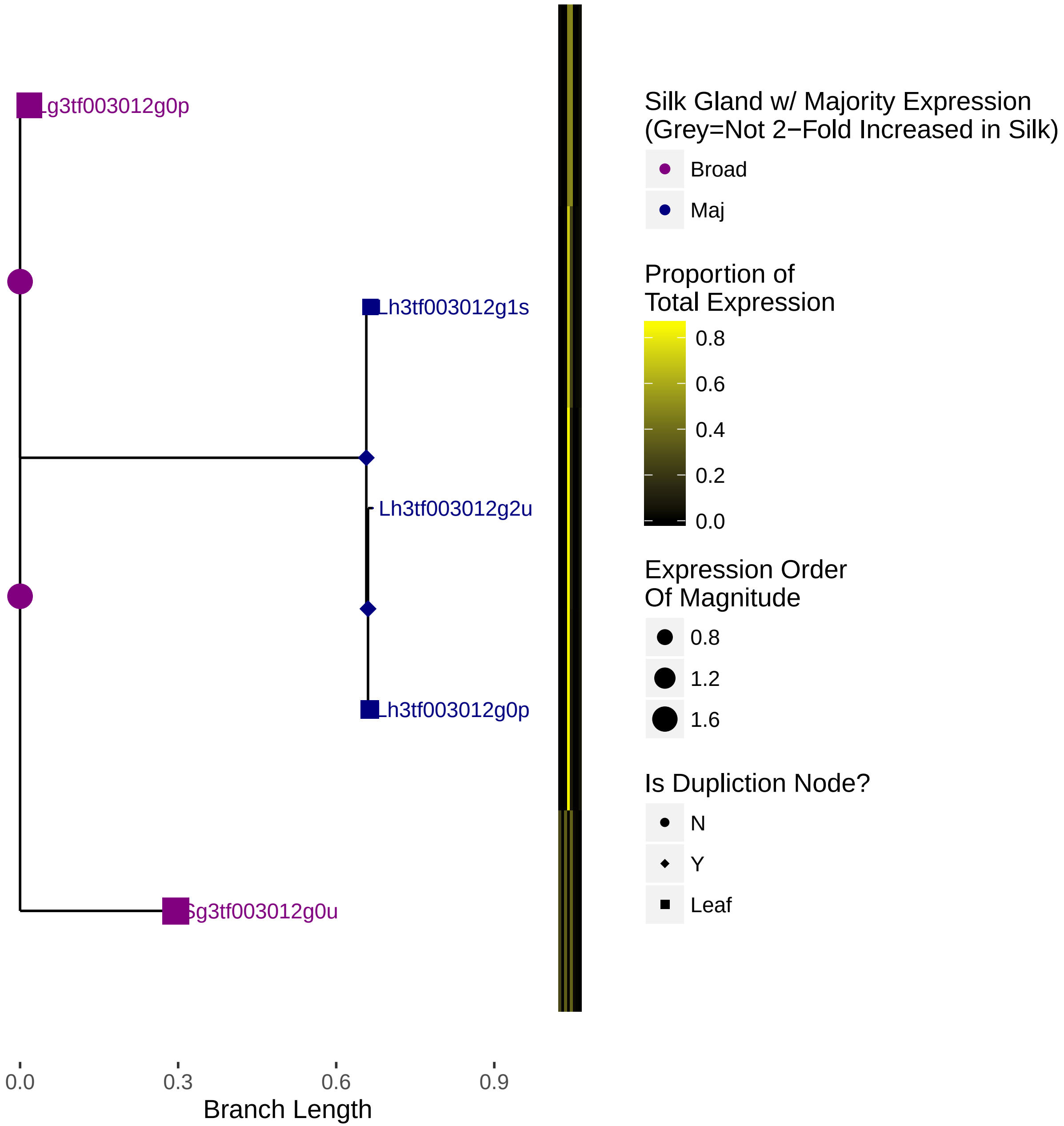

Lg3tf003012g0p
Lh3tf003012g1s
Lh3tf003012g2u
Lh3tf003012g0p
Sg3tf003012g0u
0.0
0.3
0.6
0.9
Branch Length
Silk Gland w/ Majority Expression
(Grey=Not 2−Fold Increased in Silk)
Broad
Maj
Proportion of
Total Expression
0.8
0.6
0.4
0.2
0.0
Expression Order
Of Magnitude
0.8
1.2
1.6
Is Dupliction Node?
N
Y
Leaf

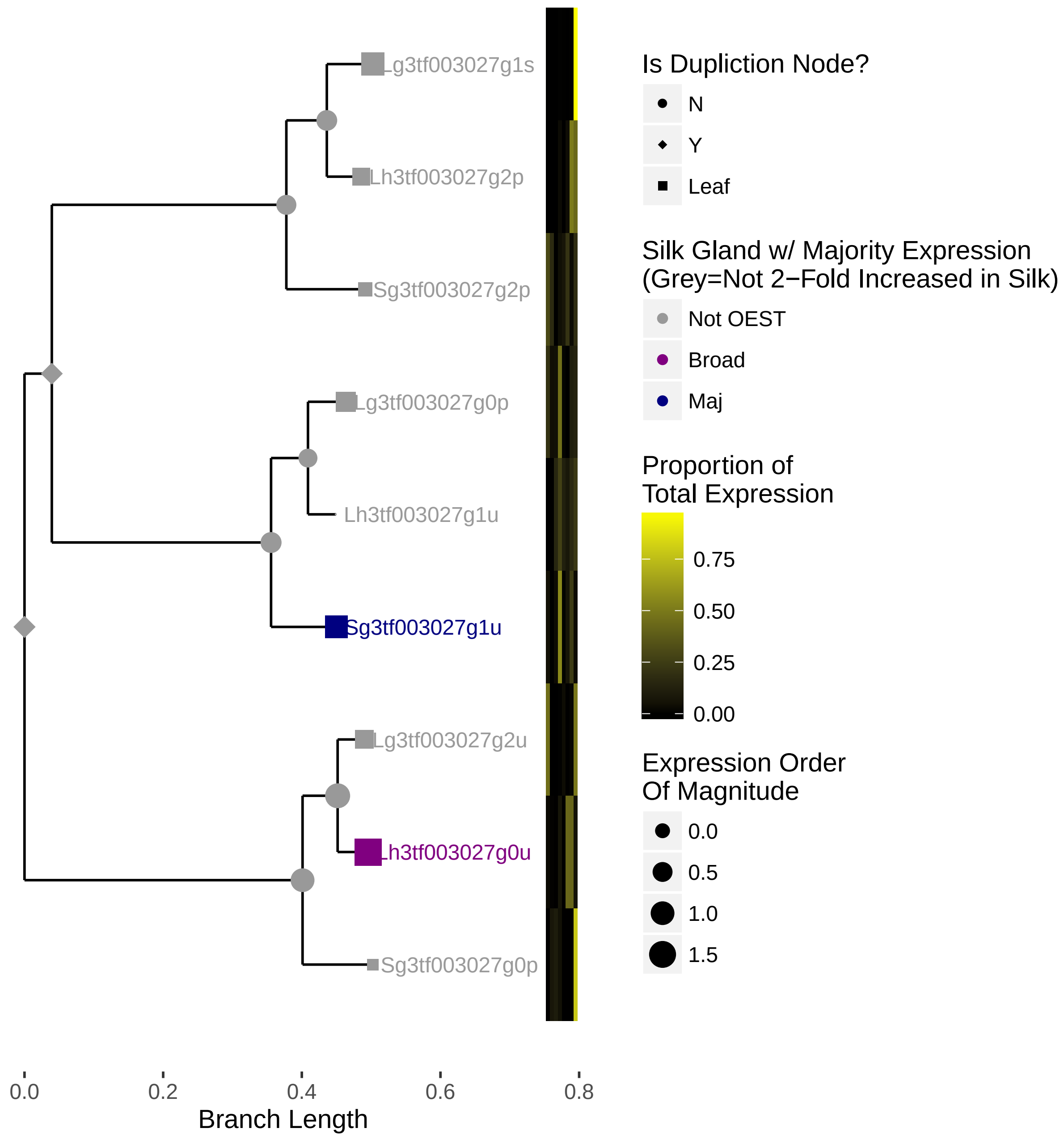
Lg3tf003027g1s
Lh3tf003027g2p
Sg3tf003027g2p
Lg3tf003027g0p
Lh3tf003027g1u
Sg3tf003027g1u
Lg3tf003027g2u
Lh3tf003027g0u
Sg3tf003027g0p
0.0
0.2
0.4
0.6
0.8
Branch Length
Is Dupliction Node?
N
Y
Leaf
Silk Gland w/ Majority Expression
(Grey=Not 2−Fold Increased in Silk)
Not OEST
Broad
Maj
Proportion of
Total Expression
0.75
0.50
0.25
0.00
Expression Order
Of Magnitude
0.0
0.5
1.0
1.5

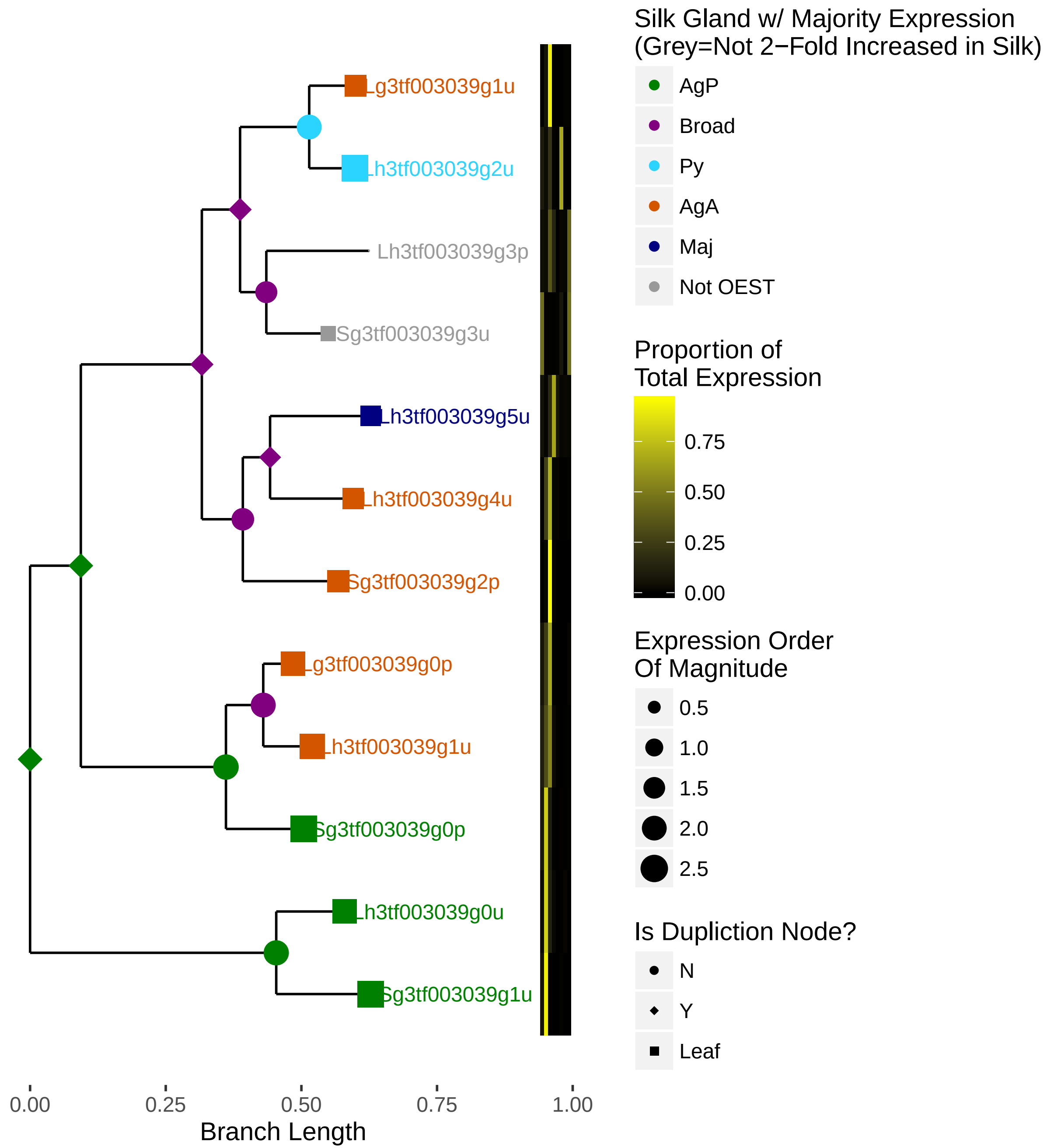

Silk Gland w/ Majority Expression
(Grey=Not 2−Fold Increased in Silk)
AgP
Broad
Py
AgA
Maj
Not OEST
Proportion of
Total Expression
0.75
0.50
0.25
0.00
Expression Order
Of Magnitude
0.5
1.0
1.5
2.0
2.5
Is Dupliction Node?
N
Y
Leaf
Lg3tf003039g1u
Lh3tf003039g2u
Lh3tf003039g3p
Sg3tf003039g3u
Lh3tf003039g5u
Lh3tf003039g4u
Sg3tf003039g2p
Lg3tf003039g0p
Lh3tf003039g1u
Sg3tf003039g0p
Lh3tf003039g0u
Sg3tf003039g1u
0.00
0.25
0.50
0.75
1.00
Branch Length

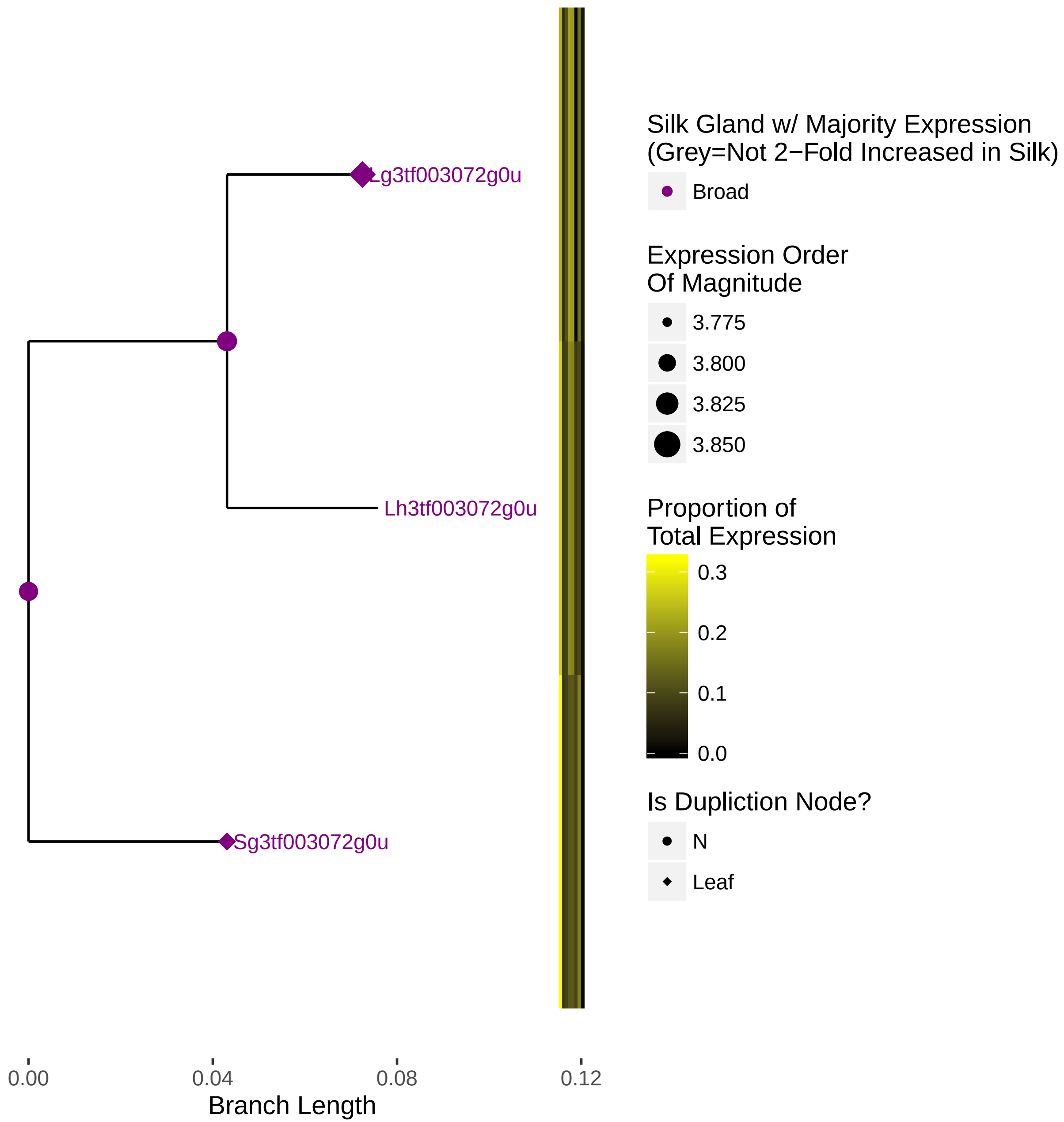

Lg3tf003072g0u
Lh3tf003072g0u
Sg3tf003072g0u
0.00
0.04
0.08
0.12
Branch Length
Silk Gland w/ Majority Expression
(Grey=Not 2−Fold Increased in Silk)
Broad
Expression Order
Of Magnitude
3.775
3.800
3.825
3.850
Proportion of
Total Expression
0.3
0.2
0.1
0.0
Is Dupliction Node?
N
Leaf

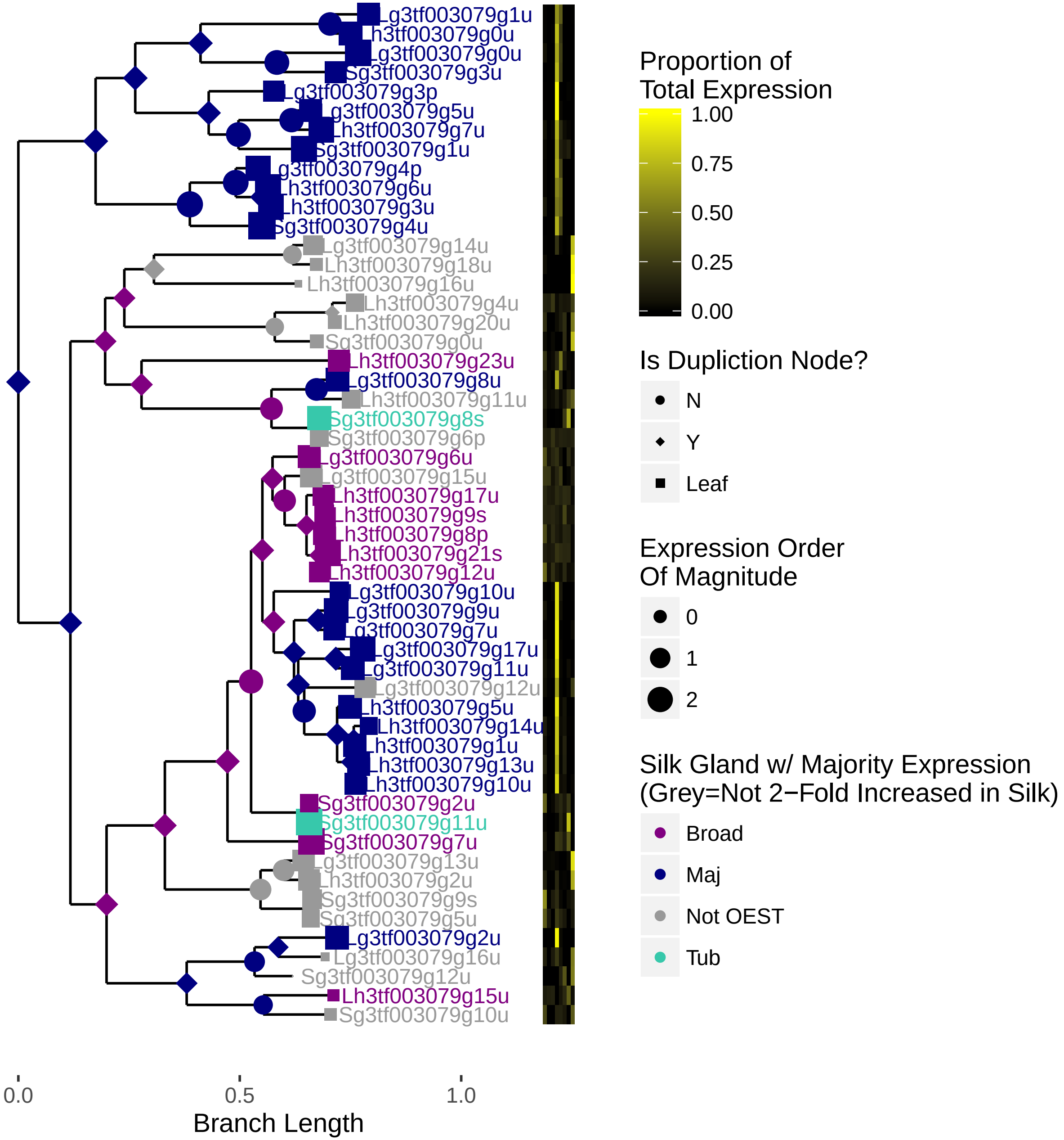

Lg3tf003079g1u
h3tf003079g0u
Lg3tf003079g0u
Sg3tf003079g3u
Lg3tf003079g3p
g3tf003079g5u
Lh3tf003079g7u
Sg3tf003079g1u
g3tf003079g4p
Lh3tf003079g6u
Lh3tf003079g3u
Sg3tf003079g4u
Lg3tf003079g14u
Lh3tf003079g18u
Lh3tf003079g16u
Lh3tf003079g4u
Lh3tf003079g20u
Sg3tf003079g0u
Lh3tf003079g23u
Lg3tf003079g8u
Lh3tf003079g11u
Sg3tf003079g8s
Sg3tf003079g6p
Lg3tf003079g6u
Lg3tf003079g15u
Lh3tf003079g17u
Lh3tf003079g9s
Lh3tf003079g8p
Lh3tf003079g21s
Lh3tf003079g12u
Lg3tf003079g10u
Lg3tf003079g9u
g3tf003079g7u
Lg3tf003079g17u
Lg3tf003079g11u
Lg3tf003079g12u
Lh3tf003079g5u
Lh3tf003079g14u
h3tf003079g1u
Lh3tf003079g13u
Lh3tf003079g10u
Sg3tf003079g2u
Sg3tf003079g11u
Sg3tf003079g7u
Lg3tf003079g13u
Lh3tf003079g2u
Sg3tf003079g9s
Sg3tf003079g5u
Lg3tf003079g2u
Lg3tf003079g16u
Sg3tf003079g12u
Lh3tf003079g15u
Sg3tf003079g10u
0.0
0.5
1.0
Branch Length
Proportion of Total Expression
1.00
0.75
0.50
0.25
0.00
Is Dupliction Node?
N
Y
Leaf
Expression Order Of Magnitude
0
1
2
Silk Gland w/ Majority Expression (Grey=Not 2−Fold Increased in Silk)
Broad
Maj
Not OEST
Tub

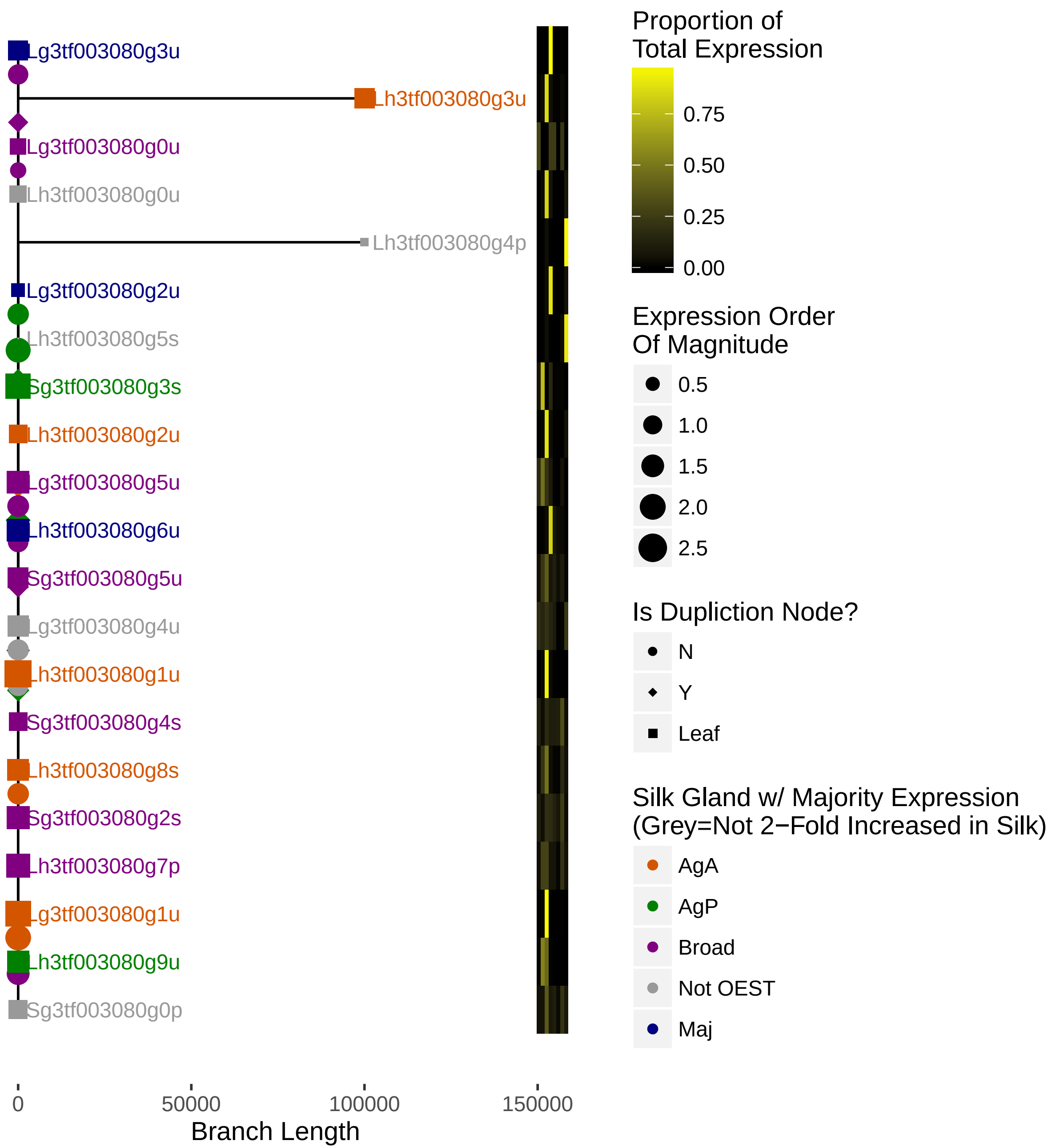

Lg3tf003080g3u
Lh3tf003080g3u
Lg3tf003080g0u
Lh3tf003080g0u
Lh3tf003080g4p
Lg3tf003080g2u
Lh3tf003080g5s
Sg3tf003080g3s
Lh3tf003080g2u
Lg3tf003080g5u
Lh3tf003080g6u
Sg3tf003080g5u
Lg3tf003080g4u
Lh3tf003080g1u
Sg3tf003080g4s
Lh3tf003080g8s
Sg3tf003080g2s
Lh3tf003080g7p
Lg3tf003080g1u
Lh3tf003080g9u
Sg3tf003080g0p
0
50000
100000
150000
Branch Length
Proportion of
Total Expression
0.75
0.50
0.25
0.00
Expression Order
Of Magnitude
0.5
1.0
1.5
2.0
2.5
Is Dupliction Node?
N
Y
Leaf
Silk Gland w/ Majority Expression
(Grey=Not 2−Fold Increased in Silk)
AgA
AgP
Broad
Not OEST
Maj

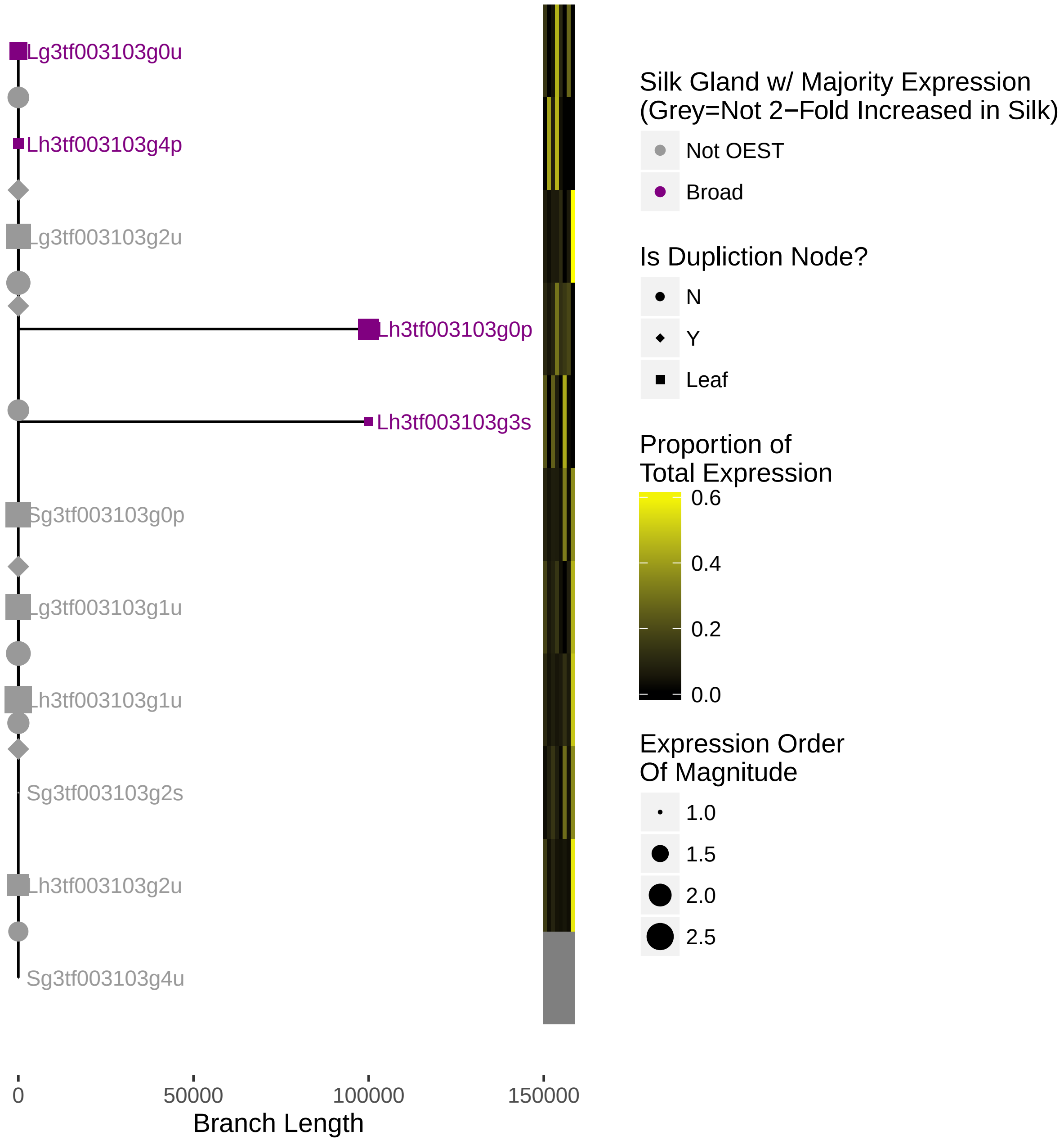
Lg3tf003103g0u
Lh3tf003103g4p
Lg3tf003103g2u
Lh3tf003103g0p
Lh3tf003103g3s
Sg3tf003103g0p
Lg3tf003103g1u
Lh3tf003103g1u
Sg3tf003103g2s
Lh3tf003103g2u
Sg3tf003103g4u
0
50000
100000
150000
Branch Length
Silk Gland w/ Majority Expression
(Grey=Not 2−Fold Increased in Silk)
Not OEST
Broad
Is Dupliction Node?
N
Y
Leaf
Proportion of
Total Expression
0.6
0.4
0.2
0.0
Expression Order
Of Magnitude
1.0
1.5
2.0
2.5

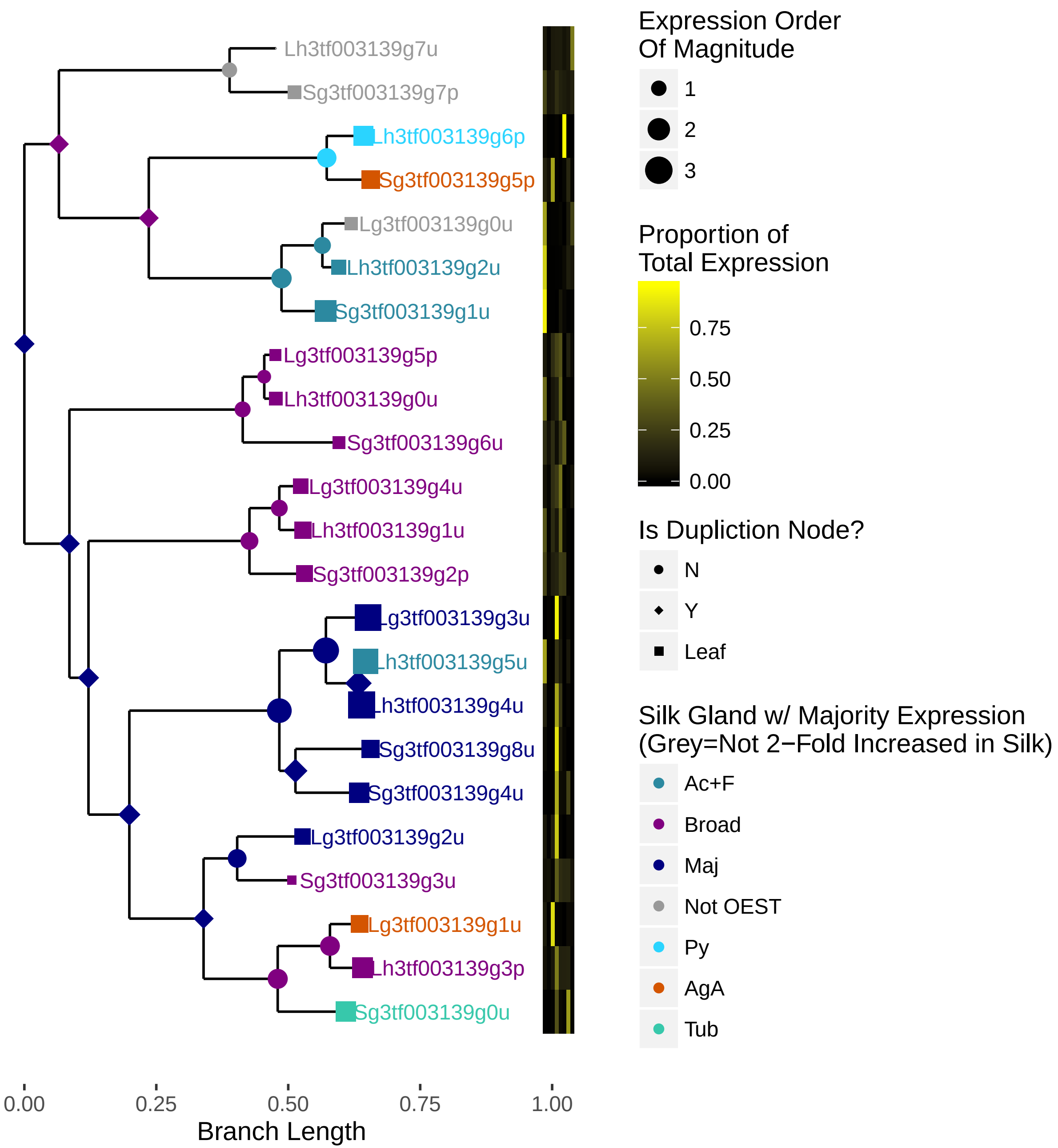

Lh3tf003139g7u
Sg3tf003139g7p
Lh3tf003139g6p
Sg3tf003139g5p
Lg3tf003139g0u
Lh3tf003139g2u
Sg3tf003139g1u
Lg3tf003139g5p
Lh3tf003139g0u
Sg3tf003139g6u
Lg3tf003139g4u
Lh3tf003139g1u
Sg3tf003139g2p
Lg3tf003139g3u
Lh3tf003139g5u
Lh3tf003139g4u
Sg3tf003139g8u
Sg3tf003139g4u
Lg3tf003139g2u
Sg3tf003139g3u
Lg3tf003139g1u
Lh3tf003139g3p
Sg3tf003139g0u
0.00
0.25
0.50
0.75
1.00
Branch Length
Expression Order Of Magnitude
1
2
3
Proportion of Total Expression
0.75
0.50
0.25
0.00
Is Dupliction Node?
N
Y
Leaf
Silk Gland w/ Majority Expression (Grey=Not 2−Fold Increased in Silk)
Ac+F
Broad
Maj
Not OEST
Py
AgA
Tub

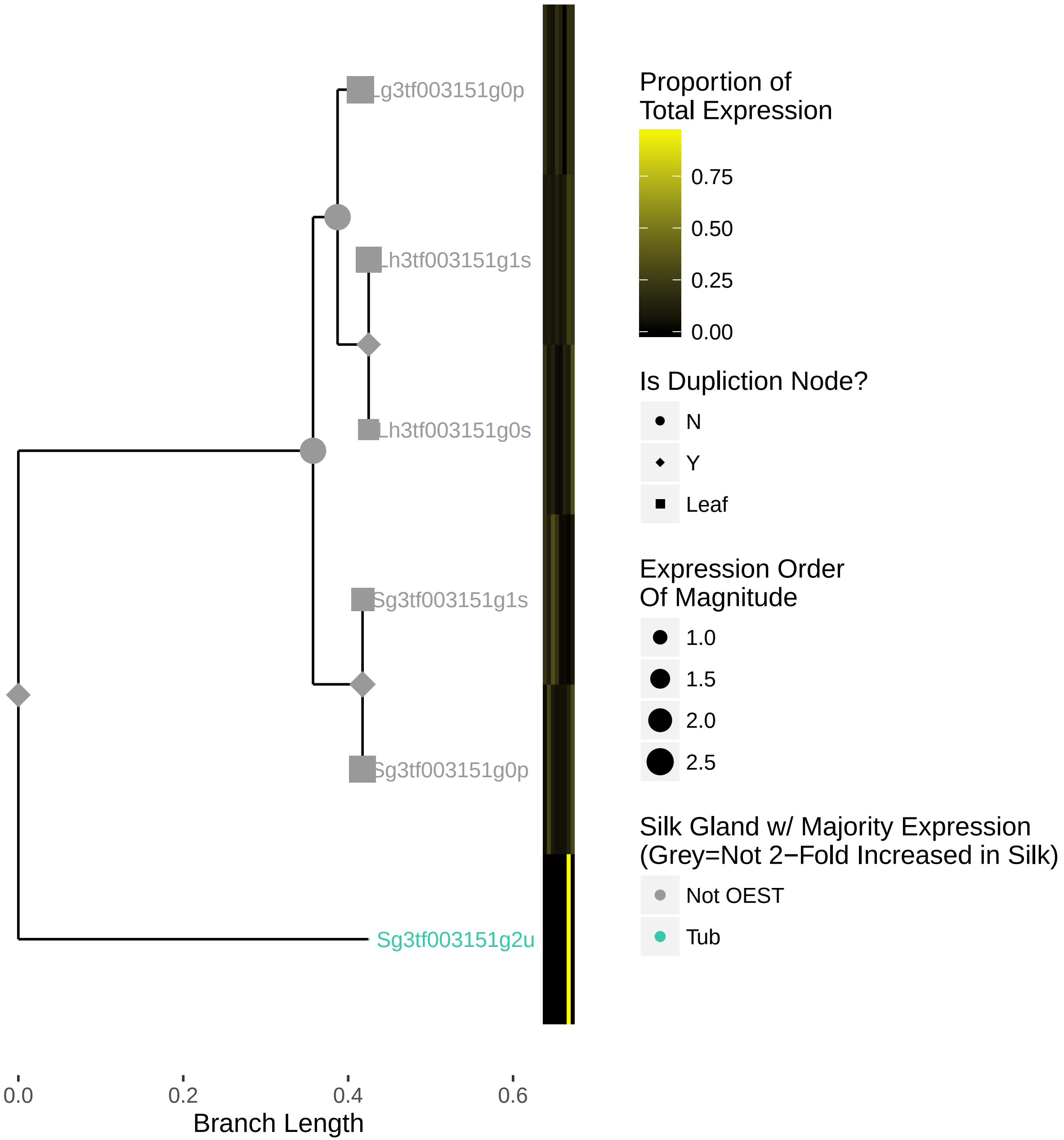

Lg3tf003151g0p
Lh3tf003151g1s
Lh3tf003151g0s
Sg3tf003151g1s
Sg3tf003151g0p
Sg3tf003151g2u
0.0
0.2
0.4
0.6
Branch Length
Proportion of
Total Expression
0.75
0.50
0.25
0.00
Is Dupliction Node?
N
Y
Leaf
Expression Order
Of Magnitude
1.0
1.5
2.0
2.5
Silk Gland w/ Majority Expression
(Grey=Not 2−Fold Increased in Silk)
Not OEST
Tub

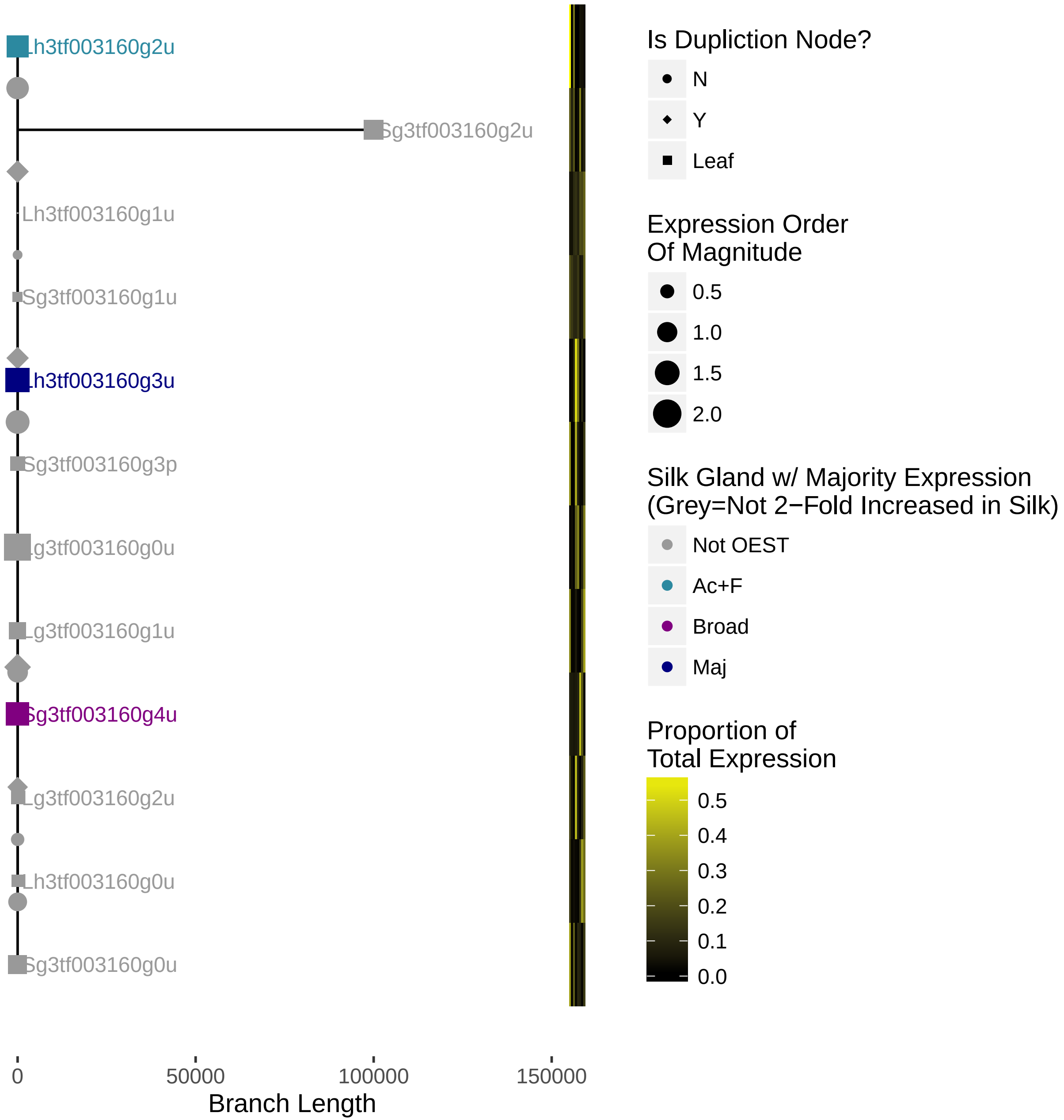

Lh3tf003160g2u
Sg3tf003160g2u
Lh3tf003160g1u
Sg3tf003160g1u
Lh3tf003160g3u
Sg3tf003160g3p
Lg3tf003160g0u
Lg3tf003160g1u
Sg3tf003160g4u
Lg3tf003160g2u
Lh3tf003160g0u
Sg3tf003160g0u
0
50000
100000
150000
Branch Length
Is Dupliction Node?
N
Y
Leaf
Expression Order Of Magnitude
0.5
1.0
1.5
2.0
Silk Gland w/ Majority Expression (Grey=Not 2−Fold Increased in Silk)
Not OEST
Ac+F
Broad
Maj
Proportion of Total Expression
0.5
0.4
0.3
0.2
0.1
0.0

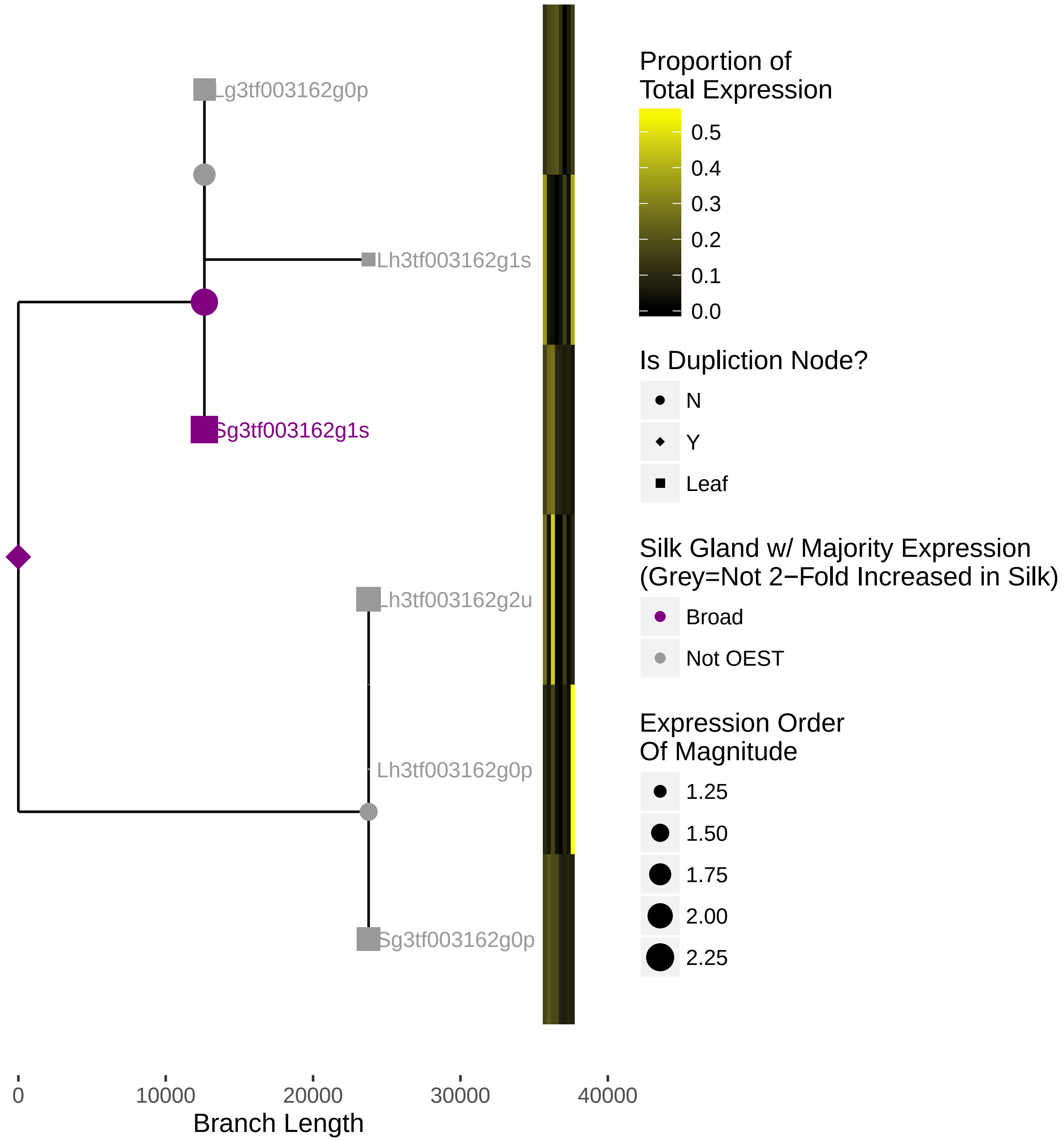

Lg3tf003162g0p
Lh3tf003162g1s
Sg3tf003162g1s
Lh3tf003162g2u
Lh3tf003162g0p
Sg3tf003162g0p
0
10000
20000
30000
40000
Branch Length
Proportion of
Total Expression
0.5
0.4
0.3
0.2
0.1
0.0
Is Dupliction Node?
N
Y
Leaf
Silk Gland w/ Majority Expression
(Grey=Not 2−Fold Increased in Silk)
Broad
Not OEST
Expression Order
Of Magnitude
1.25
1.50
1.75
2.00
2.25

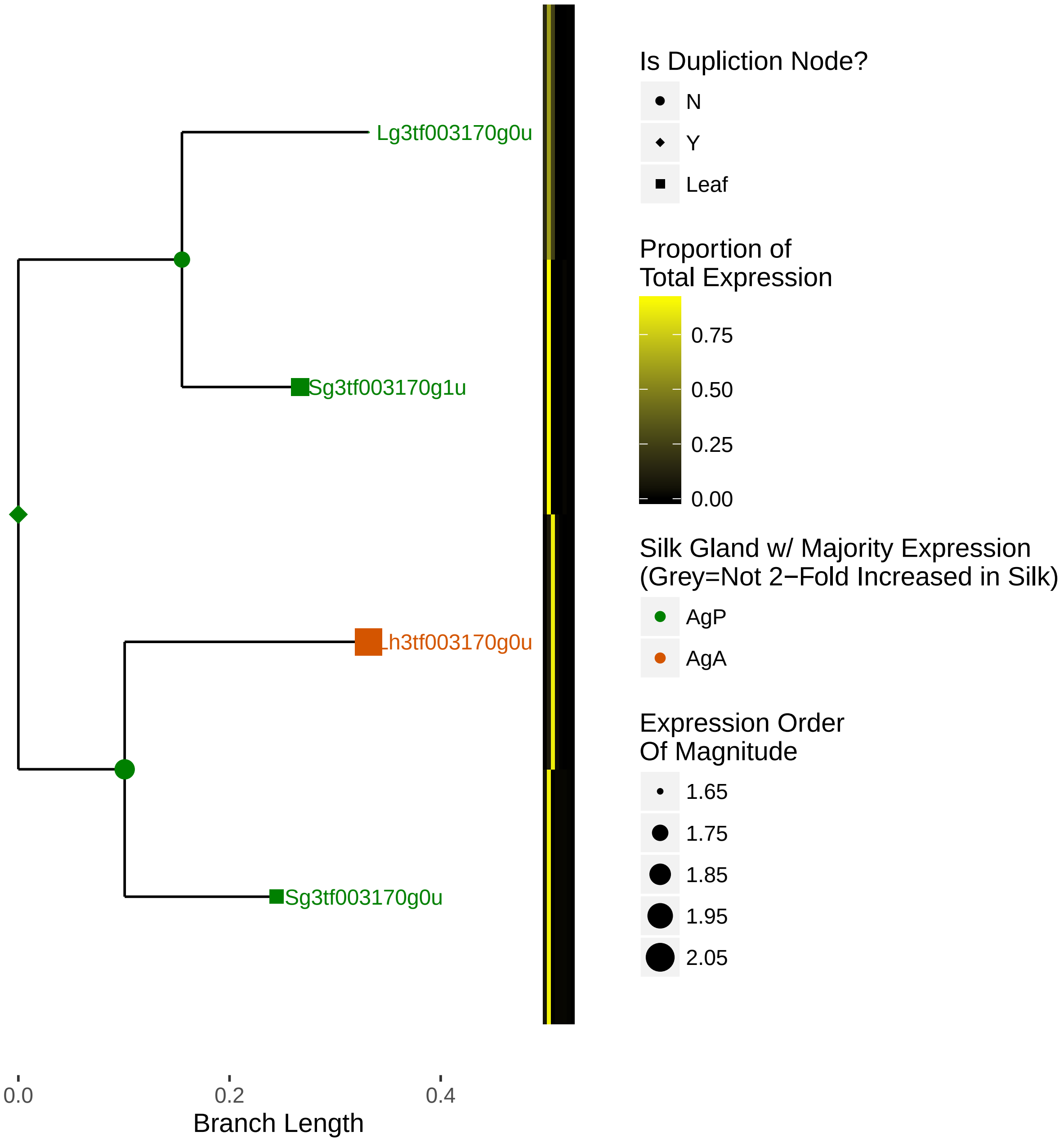
Lg3tf003170g0u
Sg3tf003170g1u
Lh3tf003170g0u
Sg3tf003170g0u
0.0
0.2
0.4
Branch Length
Is Dupliction Node?
N
Y
Leaf
Proportion of
Total Expression
0.75
0.50
0.25
0.00
Silk Gland w/ Majority Expression
(Grey=Not 2−Fold Increased in Silk)
AgP
AgA
Expression Order
Of Magnitude
1.65
1.75
1.85
1.95
2.05

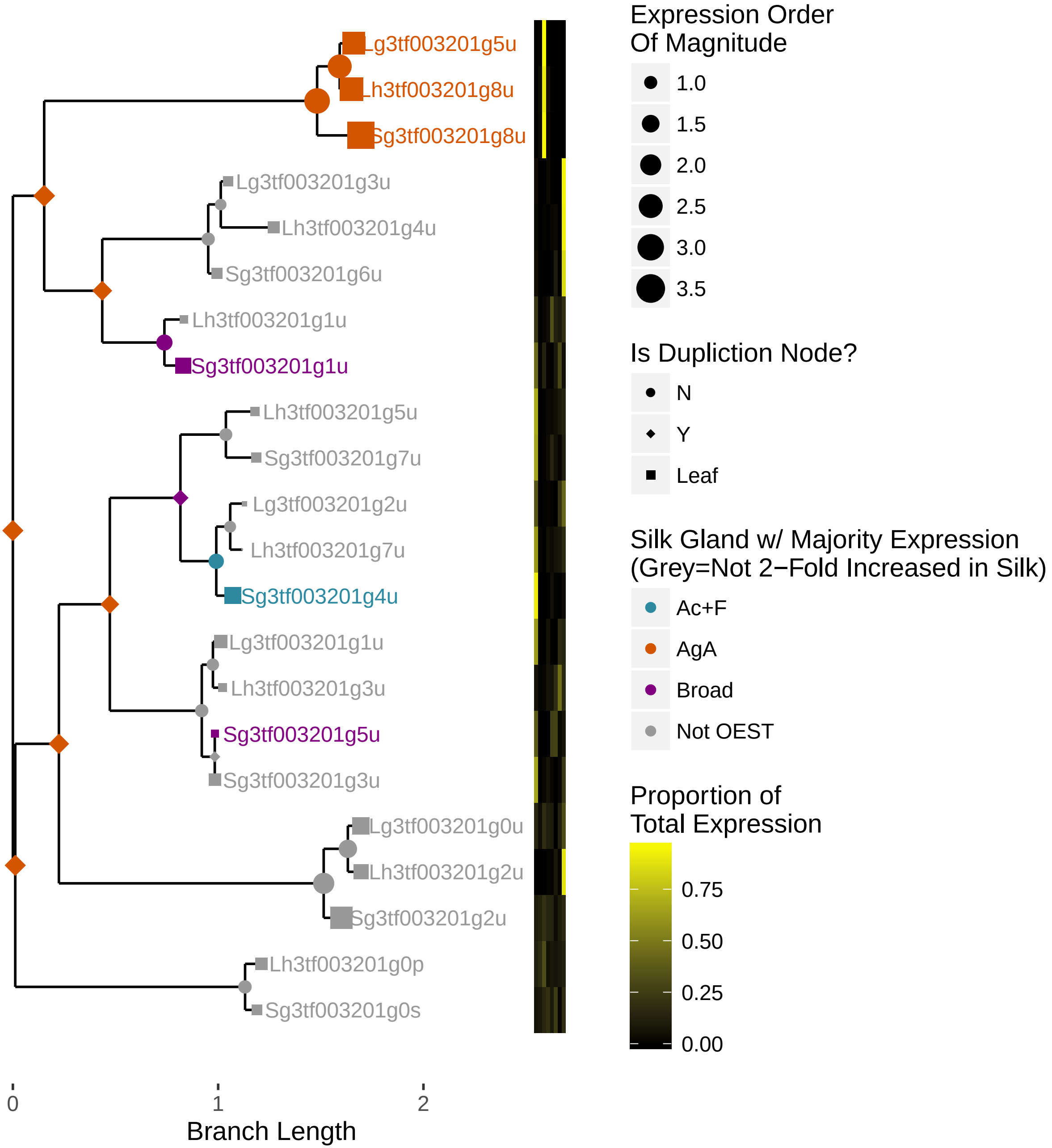

Lg3tf003201g5u
Lh3tf003201g8u
Sg3tf003201g8u
Lg3tf003201g3u
Lh3tf003201g4u
Sg3tf003201g6u
Lh3tf003201g1u
Sg3tf003201g1u
Lh3tf003201g5u
Sg3tf003201g7u
Lg3tf003201g2u
Lh3tf003201g7u
Sg3tf003201g4u
Lg3tf003201g1u
Lh3tf003201g3u
Sg3tf003201g5u
Sg3tf003201g3u
Lg3tf003201g0u
Lh3tf003201g2u
Sg3tf003201g2u
Lh3tf003201g0p
Sg3tf003201g0s
0
1
2
Branch Length
Expression Order Of Magnitude
1.0
1.5
2.0
2.5
3.0
3.5
Is Dupliction Node?
N
Y
Leaf
Silk Gland w/ Majority Expression (Grey=Not 2−Fold Increased in Silk)
Ac+F
AgA
Broad
Not OEST
Proportion of Total Expression
0.75
0.50
0.25
0.00

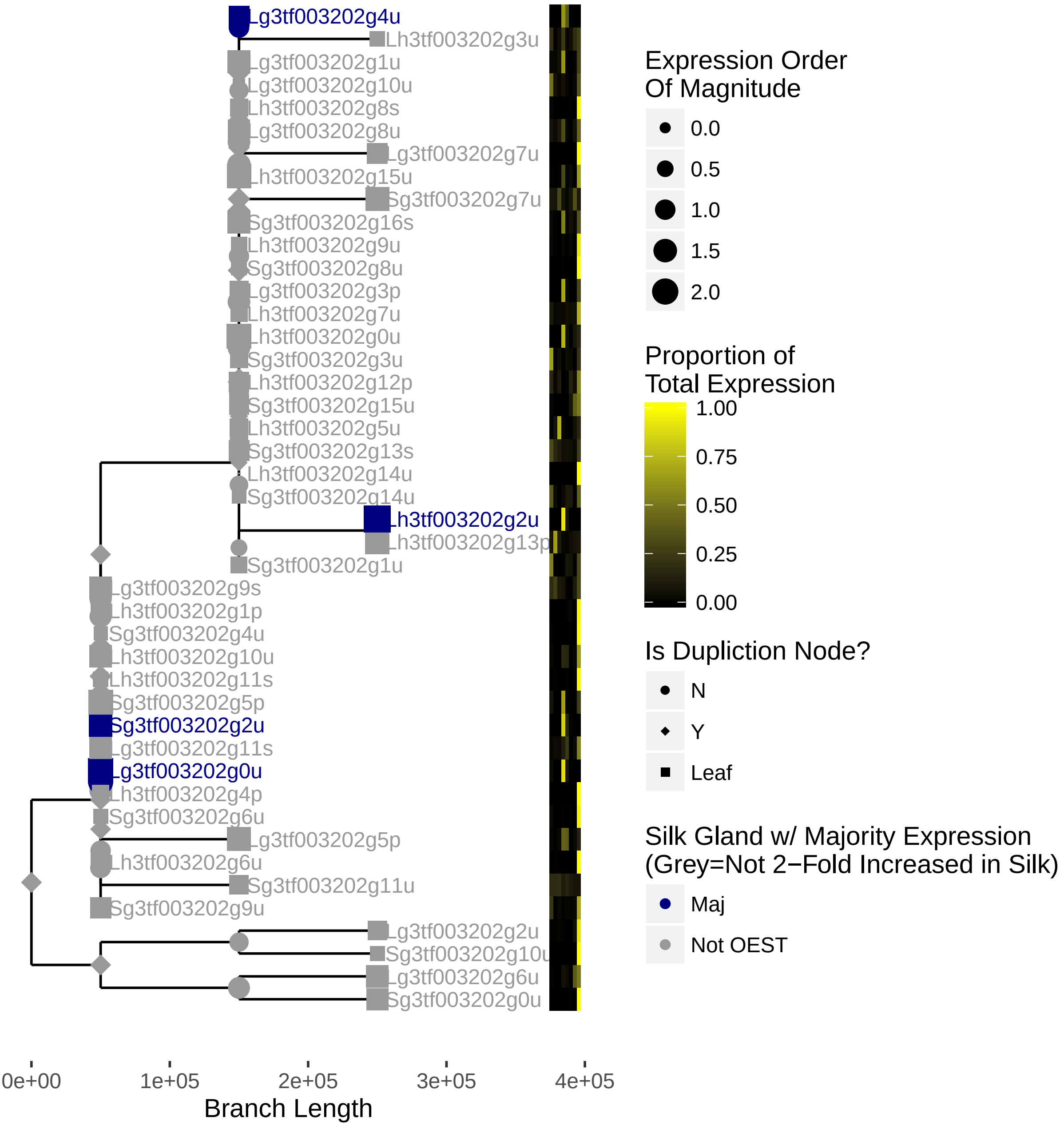
Lg3tf003202g4u
Lh3tf003202g3u
Lg3tf003202g1u
Lg3tf003202g10u
Lh3tf003202g8s
Lg3tf003202g8u
Lg3tf003202g7u
Lh3tf003202g15u
Sg3tf003202g7u
Sg3tf003202g16s
Lh3tf003202g9u
Sg3tf003202g8u
Lg3tf003202g3p
Lh3tf003202g7u
Lh3tf003202g0u
Sg3tf003202g3u
Lh3tf003202g12p
Sg3tf003202g15u
Lh3tf003202g5u
Sg3tf003202g13s
Lh3tf003202g14u
Sg3tf003202g14u
Lh3tf003202g2u
Lh3tf003202g13p
Sg3tf003202g1u
Lg3tf003202g9s
Lh3tf003202g1p
Sg3tf003202g4u
Lh3tf003202g10u
Lh3tf003202g11s
Sg3tf003202g5p
Sg3tf003202g2u
Lg3tf003202g11s
Lg3tf003202g0u
Lh3tf003202g4p
Sg3tf003202g6u
Lg3tf003202g5p
Lh3tf003202g6u
Sg3tf003202g11u
Sg3tf003202g9u
Lg3tf003202g2u
Sg3tf003202g10u
Lg3tf003202g6u
Sg3tf003202g0u
0e+00
1e+05
2e+05
3e+05
4e+05
Branch Length
Expression Order
Of Magnitude
0.0
0.5
1.0
1.5
2.0
Proportion of
Total Expression
1.00
0.75
0.50
0.25
0.00
Is Dupliction Node?
N
Y
Leaf
Silk Gland w/ Majority Expression
(Grey=Not 2−Fold Increased in Silk)
Maj
Not OEST

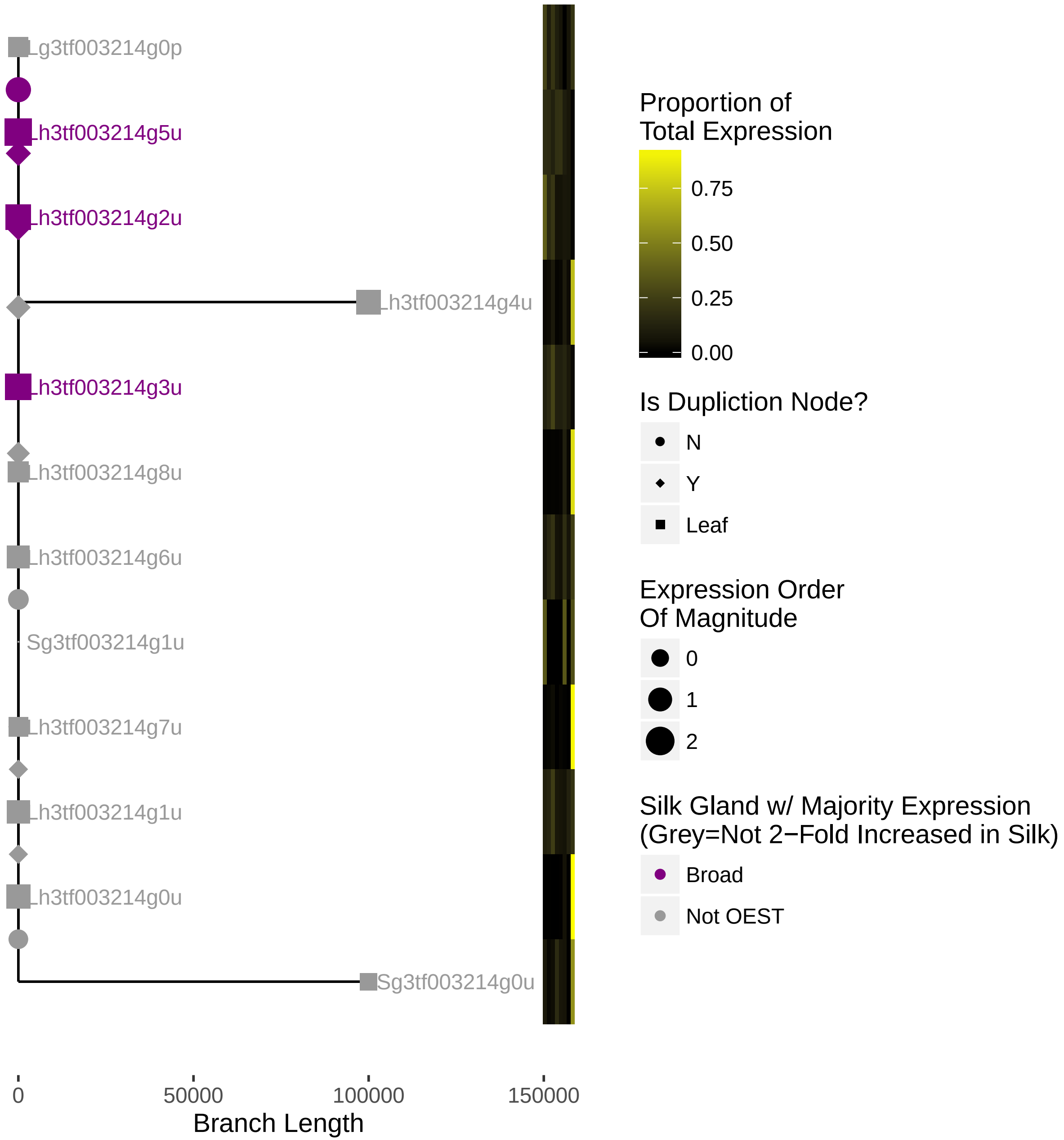

Lg3tf003214g0p
Lh3tf003214g5u
Lh3tf003214g2u
Lh3tf003214g4u
Lh3tf003214g3u
Lh3tf003214g8u
Lh3tf003214g6u
Sg3tf003214g1u
Lh3tf003214g7u
Lh3tf003214g1u
Lh3tf003214g0u
Sg3tf003214g0u
0
50000
100000
150000
Branch Length
Proportion of
Total Expression
0.75
0.50
0.25
0.00
Is Dupliction Node?
N
Y
Leaf
Expression Order
Of Magnitude
0
1
2
Silk Gland w/ Majority Expression
(Grey=Not 2−Fold Increased in Silk)
Broad
Not OEST

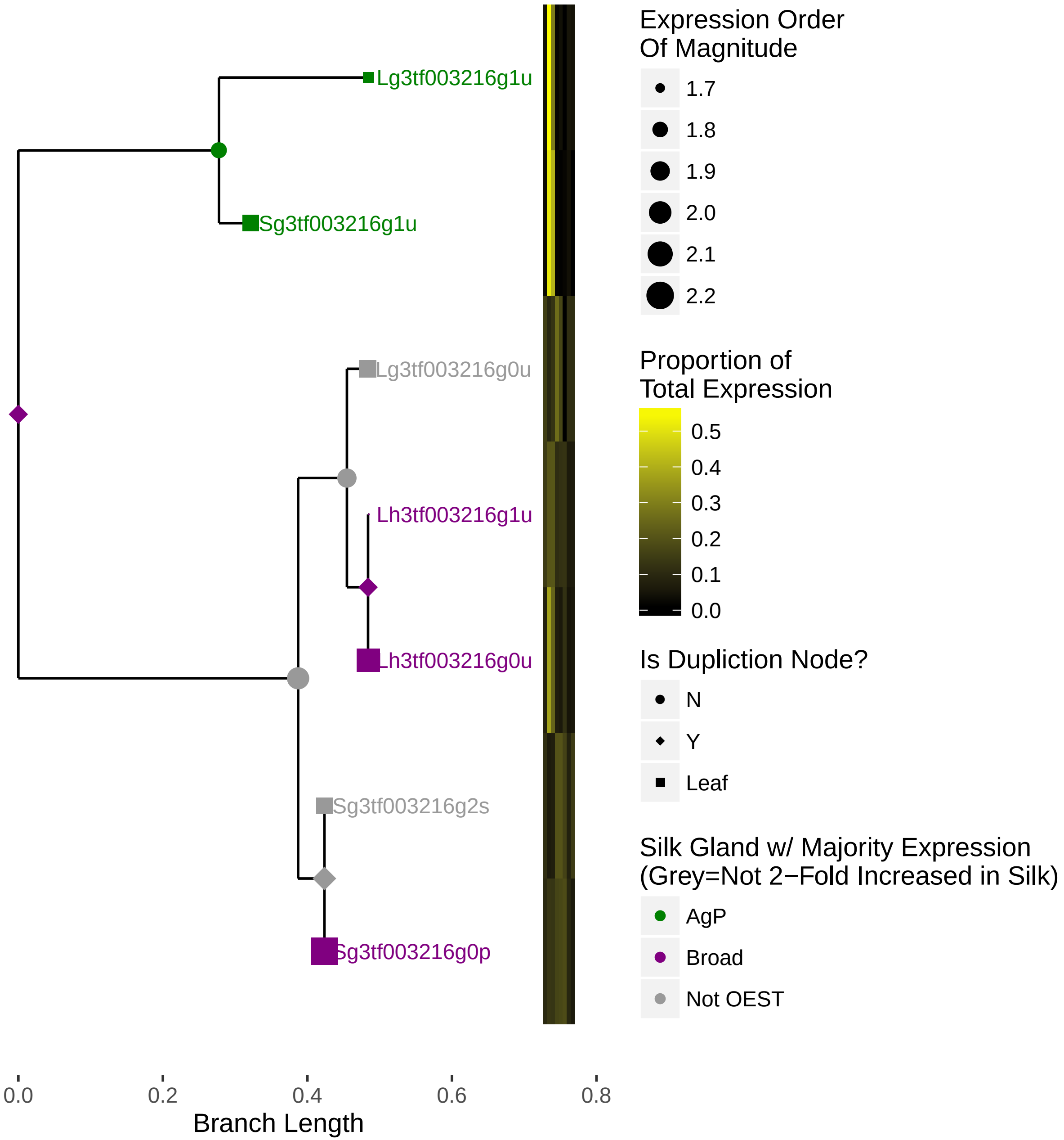

Lg3tf003216g1u
Sg3tf003216g1u
Lg3tf003216g0u
Lh3tf003216g1u
Lh3tf003216g0u
Sg3tf003216g2s
Sg3tf003216g0p
0.0
0.2
0.4
0.6
0.8
Branch Length
Expression Order Of Magnitude
1.7
1.8
1.9
2.0
2.1
2.2
Proportion of Total Expression
0.5
0.4
0.3
0.2
0.1
0.0
Is Dupliction Node?
N
Y
Leaf
Silk Gland w/ Majority Expression (Grey=Not 2−Fold Increased in Silk)
AgP
Broad
Not OEST

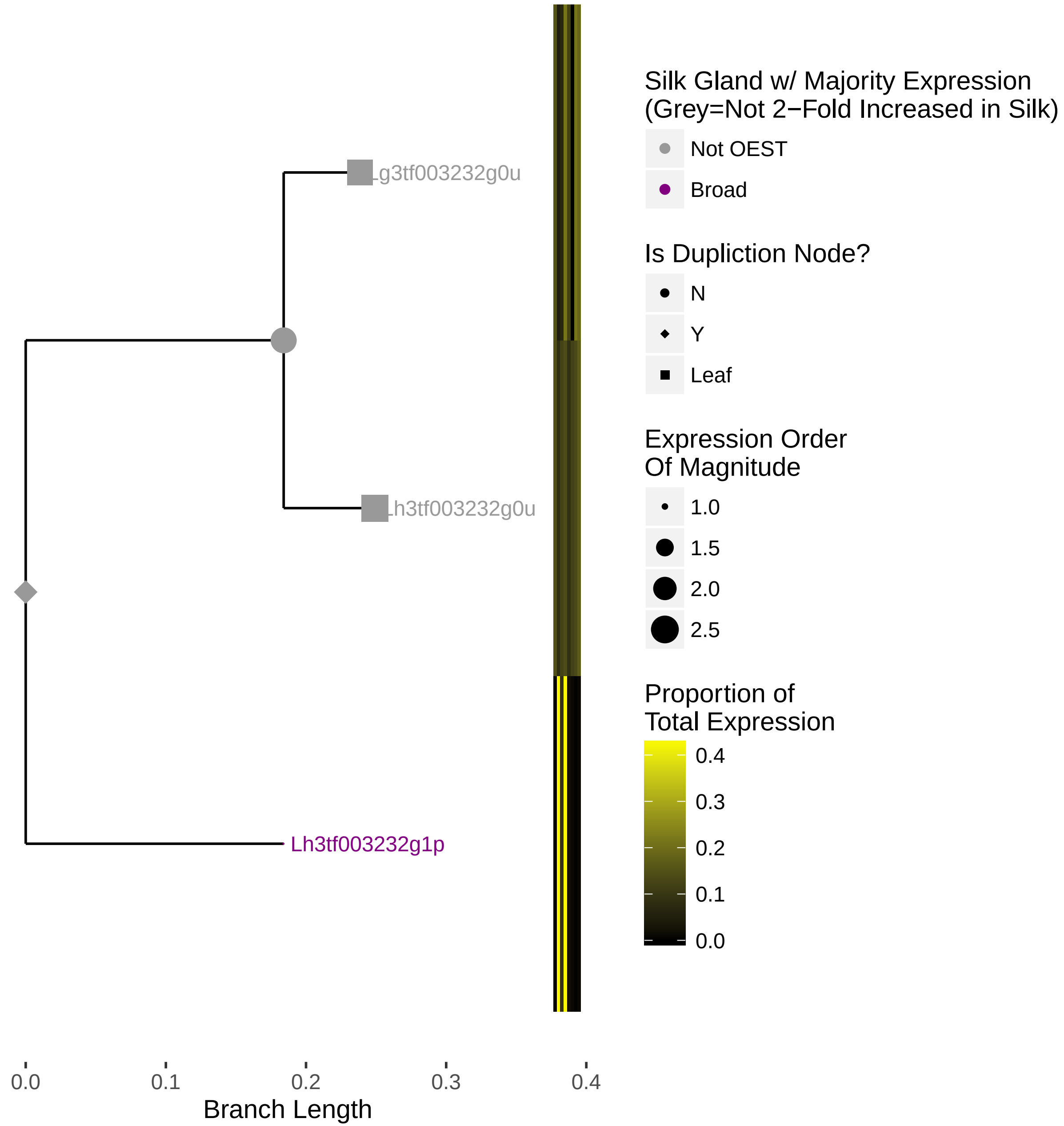

Lg3tf003232g0u
Lh3tf003232g0u
Lh3tf003232g1p
0.0
0.1
0.2
0.3
0.4
Branch Length
Silk Gland w/ Majority Expression
(Grey=Not 2−Fold Increased in Silk)
Not OEST
Broad
Is Dupliction Node?
N
Y
Leaf
Expression Order
Of Magnitude
1.0
1.5
2.0
2.5
Proportion of
Total Expression
0.4
0.3
0.2
0.1
0.0

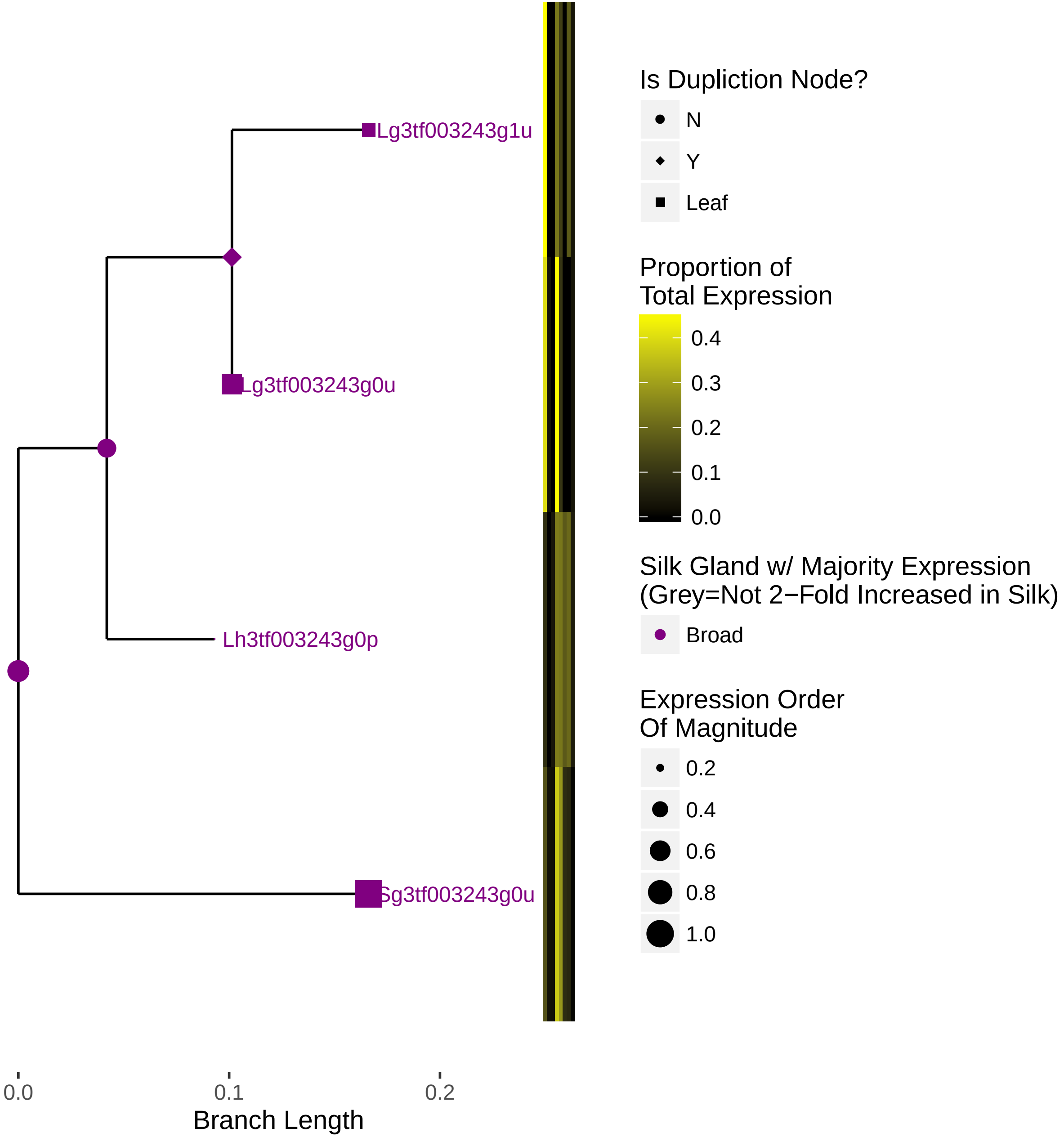

Lg3tf003243g1u
Lg3tf003243g0u
Lh3tf003243g0p
Sg3tf003243g0u
0.0
0.1
0.2
Branch Length
Is Dupliction Node?
N
Y
Leaf
Proportion of
Total Expression
0.4
0.3
0.2
0.1
0.0
Silk Gland w/ Majority Expression
(Grey=Not 2−Fold Increased in Silk)
Broad
Expression Order
Of Magnitude
0.2
0.4
0.6
0.8
1.0

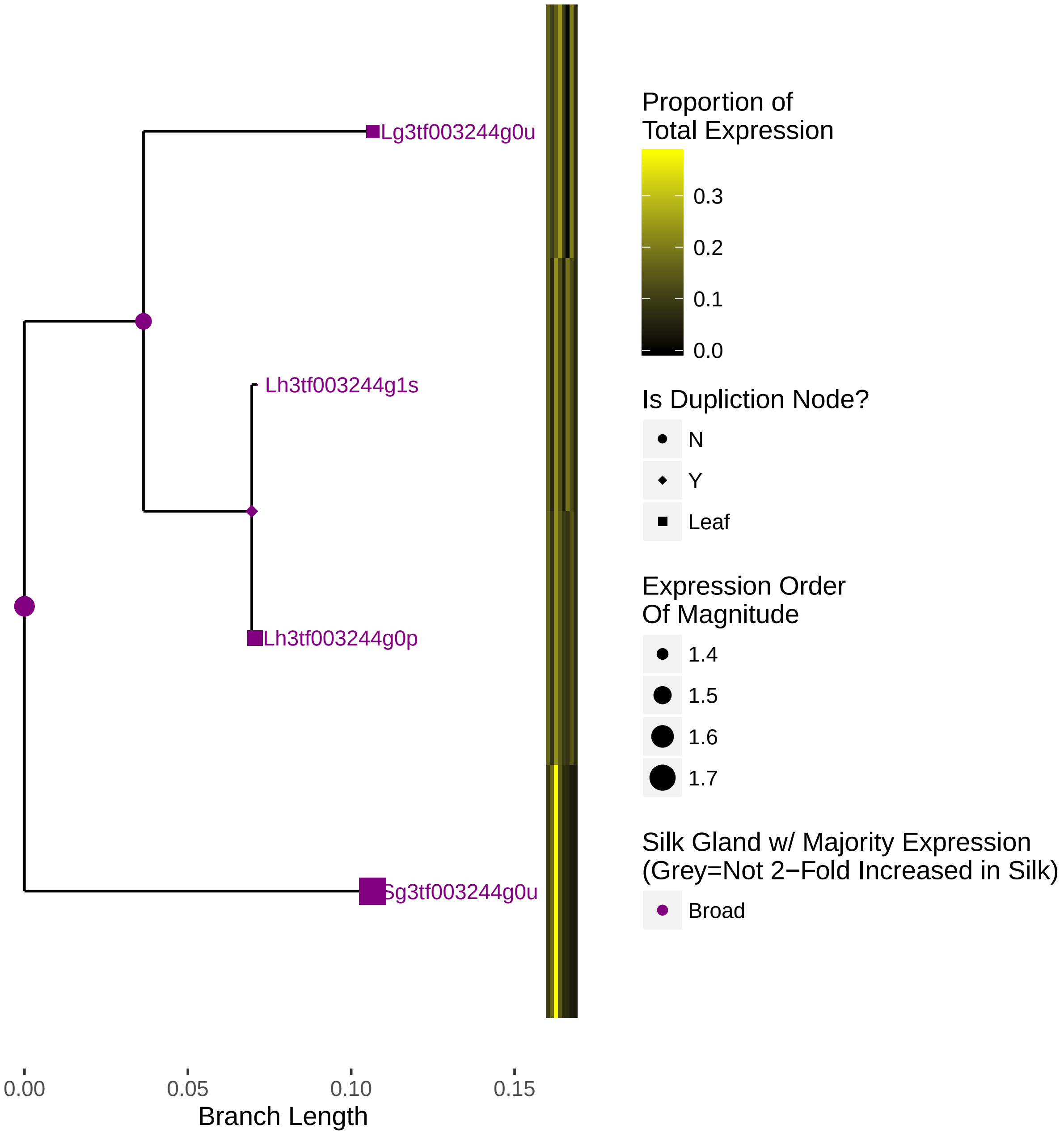

Lg3tf003244g0u
Lh3tf003244g1s
Lh3tf003244g0p
Sg3tf003244g0u
0.00
0.05
0.10
0.15
Branch Length
Proportion of Total Expression
0.3
0.2
0.1
0.0
Is Dupliction Node?
N
Y
Leaf
Expression Order Of Magnitude
1.4
1.5
1.6
1.7
Silk Gland w/ Majority Expression (Grey=Not 2−Fold Increased in Silk)
Broad

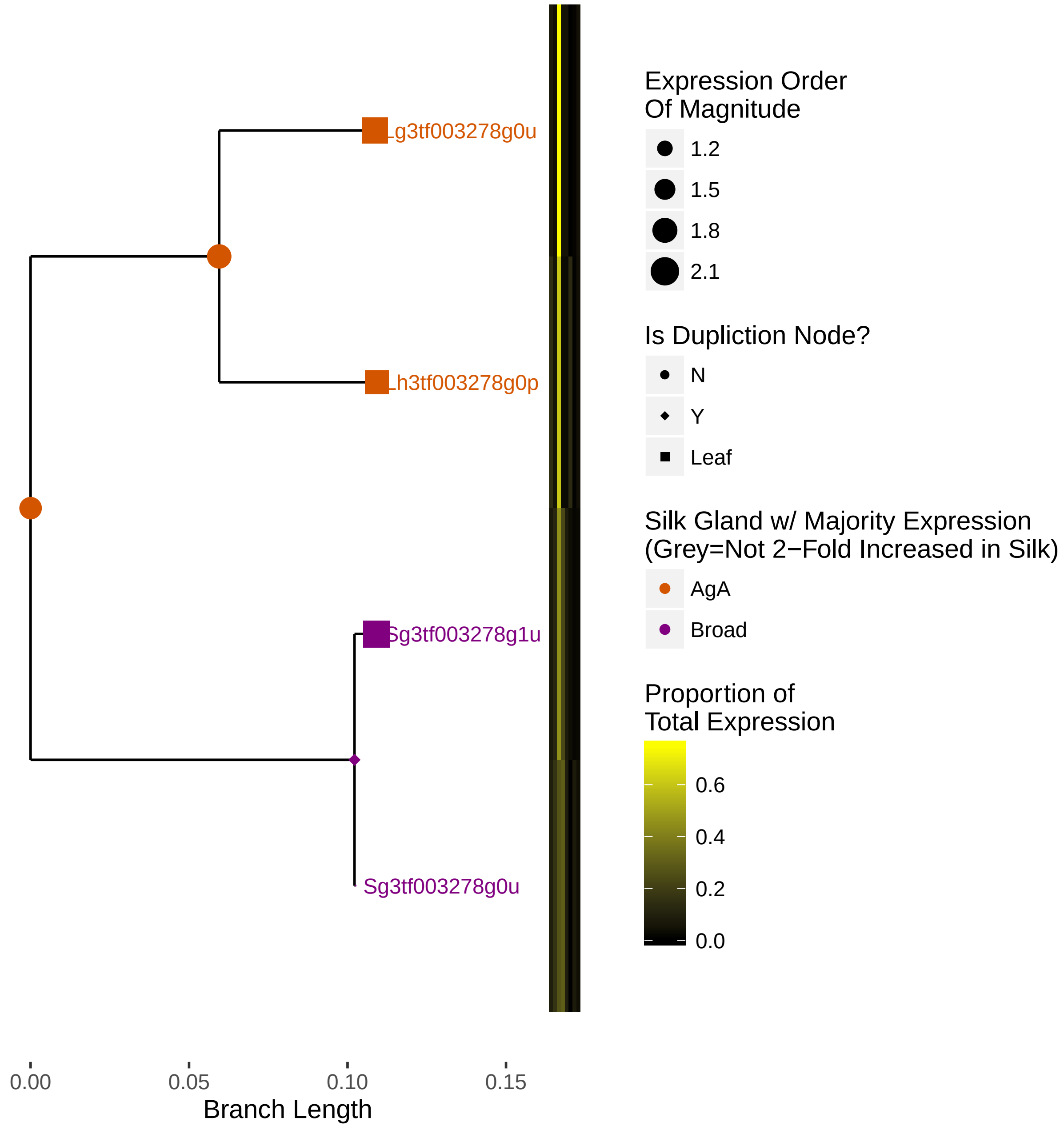

Lg3tf003278g0u
Lh3tf003278g0p
Sg3tf003278g1u
Sg3tf003278g0u
0.00
0.05
0.10
0.15
Branch Length
Expression Order
Of Magnitude
1.2
1.5
1.8
2.1
Is Dupliction Node?
N
Y
Leaf
Silk Gland w/ Majority Expression
(Grey=Not 2−Fold Increased in Silk)
AgA
Broad
Proportion of
Total Expression
0.6
0.4
0.2
0.0

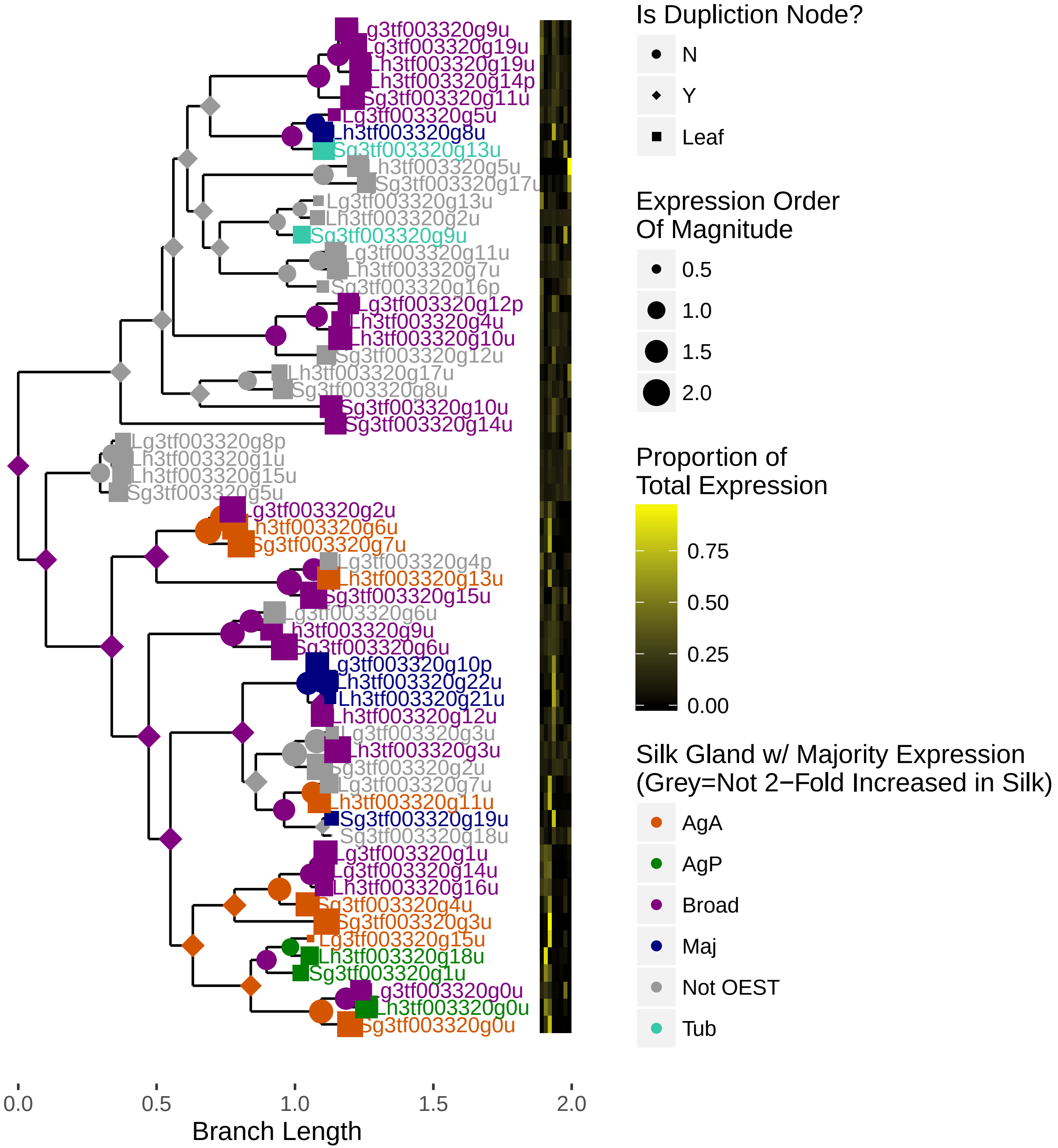
g3tf003320g9u
Lg3tf003320g19u
Lh3tf003320g19u
Lh3tf003320g14p
Sg3tf003320g11u
Lg3tf003320g5u
Lh3tf003320g8u
Sg3tf003320g13u
h3tf003320g5u
Sg3tf003320g17u
Lg3tf003320g13u
Lh3tf003320g2u
Sg3tf003320g9u
Lg3tf003320g11u
Lh3tf003320g7u
Sg3tf003320g16p
Lg3tf003320g12p
Lh3tf003320g4u
Lh3tf003320g10u
Sg3tf003320g12u
Lh3tf003320g17u
Sg3tf003320g8u
Sg3tf003320g10u
Sg3tf003320g14u
Lg3tf003320g8p
Lh3tf003320g1u
Lh3tf003320g15u
Sg3tf003320g5u
g3tf003320g2u
h3tf003320g6u
Sg3tf003320g7u
Lg3tf003320g4p
Lh3tf003320g13u
Sg3tf003320g15u
g3tf003320g6u
h3tf003320g9u
Sg3tf003320g6u
g3tf003320g10p
Lh3tf003320g22u
Lh3tf003320g21u
Lh3tf003320g12u
Lg3tf003320g3u
Lh3tf003320g3u
Sg3tf003320g2u
Lg3tf003320g7u
Lh3tf003320g11u
Sg3tf003320g19u
Sg3tf003320g18u
Lg3tf003320g1u
Lg3tf003320g14u
Lh3tf003320g16u
Sg3tf003320g4u
Sg3tf003320g3u
Lg3tf003320g15u
Lh3tf003320g18u
Sg3tf003320g1u
g3tf003320g0u
h3tf003320g0u
Sg3tf003320g0u
0.0
0.5
1.0
1.5
2.0
Branch Length
Is Dupliction Node?
N
Y
Leaf
Expression Order Of Magnitude
0.5
1.0
1.5
2.0
Proportion of Total Expression
0.75
0.50
0.25
0.00
Silk Gland w/ Majority Expression (Grey=Not 2−Fold Increased in Silk)
AgA
AgP
Broad
Maj
Not OEST
Tub

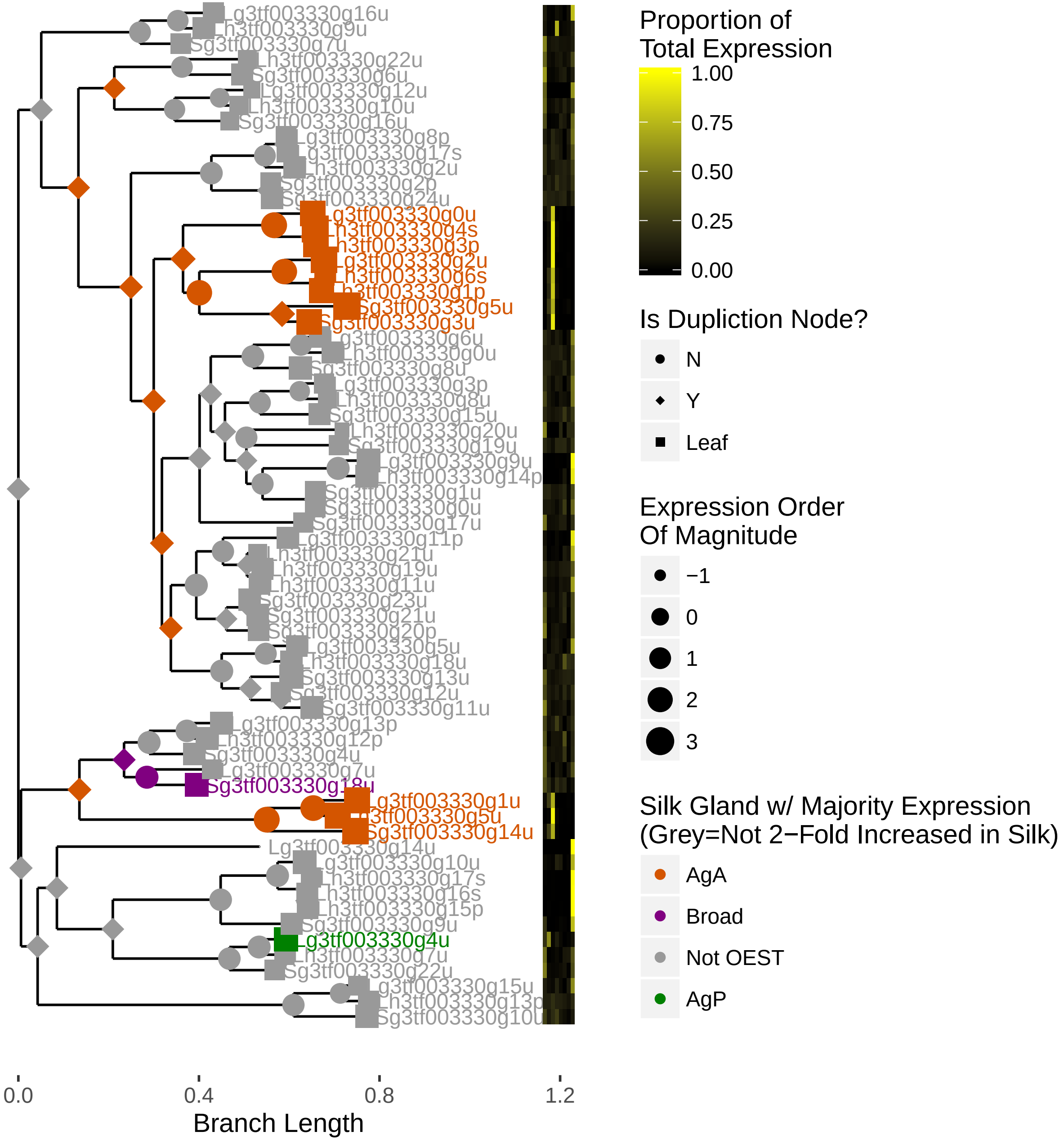

Lg3tf003330g16u
Lh3tf003330g9u
Sg3tf003330g7u
Lh3tf003330g22u
Sg3tf003330g6u
Lg3tf003330g12u
Lh3tf003330g10u
Sg3tf003330g16u
Lg3tf003330g8p
Lg3tf003330g17s
Lh3tf003330g2u
Sg3tf003330g2p
Sg3tf003330g24u
g3tf003330g0u
h3tf003330g4s
h3tf003330g3p
g3tf003330g2u
Lh3tf003330g6s
h3tf003330g1p
Sg3tf003330g5u
Sg3tf003330g3u
g3tf003330g6u
Lh3tf003330g0u
Sg3tf003330g8u
Lg3tf003330g3p
Lh3tf003330g8u
Sg3tf003330g15u
Lh3tf003330g20u
Sg3tf003330g19u
Lg3tf003330g9u
Lh3tf003330g14p
Sg3tf003330g1u
Sg3tf003330g0u
Sg3tf003330g17u
Lg3tf003330g11p
Lh3tf003330g21u
Lh3tf003330g19u
Lh3tf003330g11u
Sg3tf003330g23u
Sg3tf003330g21u
Sg3tf003330g20p
Lg3tf003330g5u
Lh3tf003330g18u
Sg3tf003330g13u
Sg3tf003330g12u
Sg3tf003330g11u
Lg3tf003330g13p
Lh3tf003330g12p
Sg3tf003330g4u
Lg3tf003330g7u
Sg3tf003330g18u
Lg3tf003330g1u
3tf003330g5u
Sg3tf003330g14u
Lg3tf003330g14u
g3tf003330g10u
Lh3tf003330g17s
Lh3tf003330g16s
Lh3tf003330g15p
Sg3tf003330g9u
Lg3tf003330g4u
Lh3tf003330g7u
Sg3tf003330g22u
g3tf003330g15u
Lh3tf003330g13p
Sg3tf003330g10u
0.0
0.4
0.8
1.2
Branch Length
Proportion of Total Expression
1.00
0.75
0.50
0.25
0.00
Is Dupliction Node?
N
Y
Leaf
Expression Order Of Magnitude
−1
0
1
2
3
Silk Gland w/ Majority Expression (Grey=Not 2−Fold Increased in Silk)
AgA
Broad
Not OEST
AgP

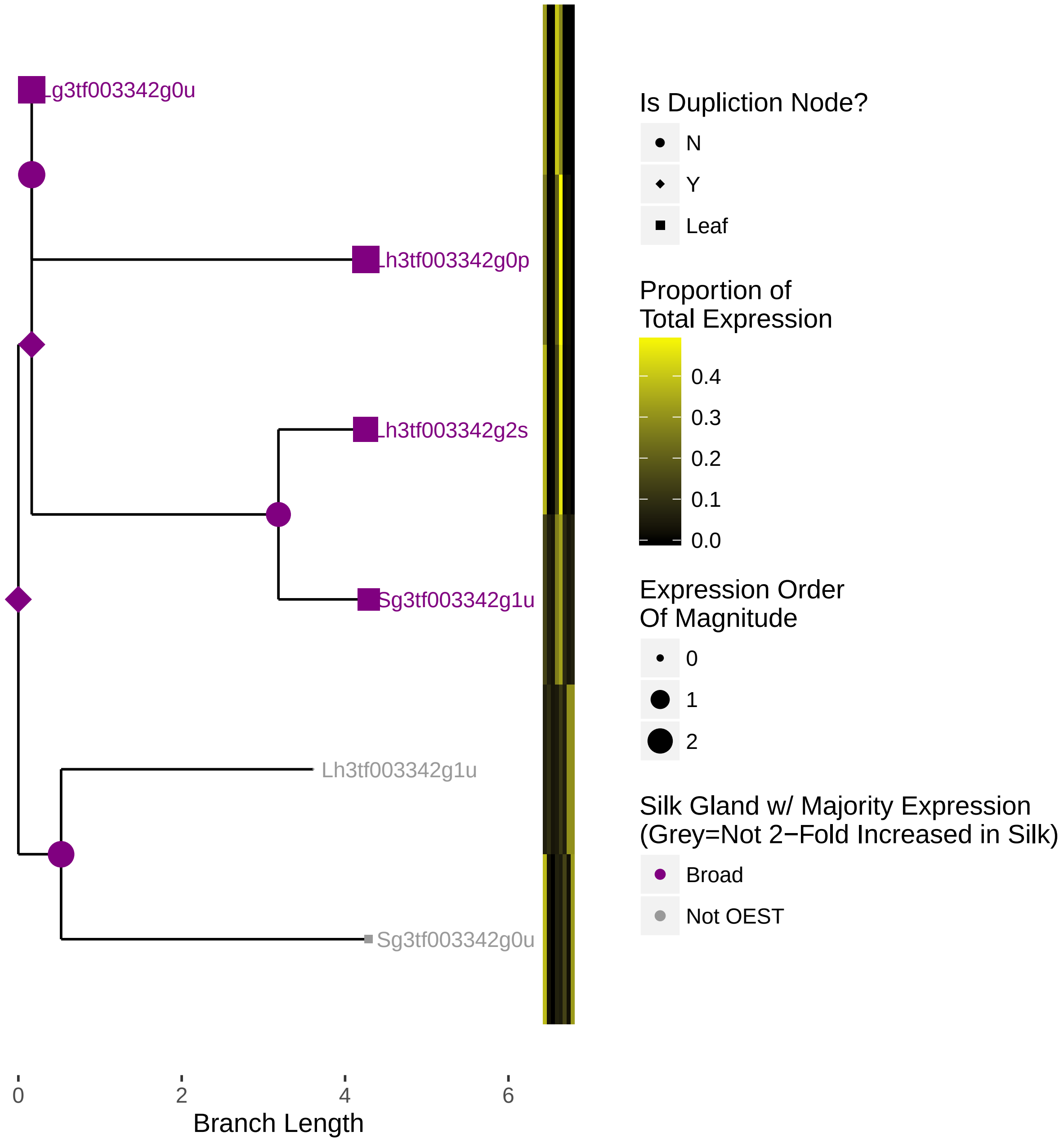
Lg3tf003342g0u
Lh3tf003342g0p
Lh3tf003342g2s
Sg3tf003342g1u
Lh3tf003342g1u
Sg3tf003342g0u
0
2
4
6
Branch Length
Is Dupliction Node?
N
Y
Leaf
Proportion of
Total Expression
0.4
0.3
0.2
0.1
0.0
Expression Order
Of Magnitude
0
1
2
Silk Gland w/ Majority Expression
(Grey=Not 2−Fold Increased in Silk)
Broad
Not OEST

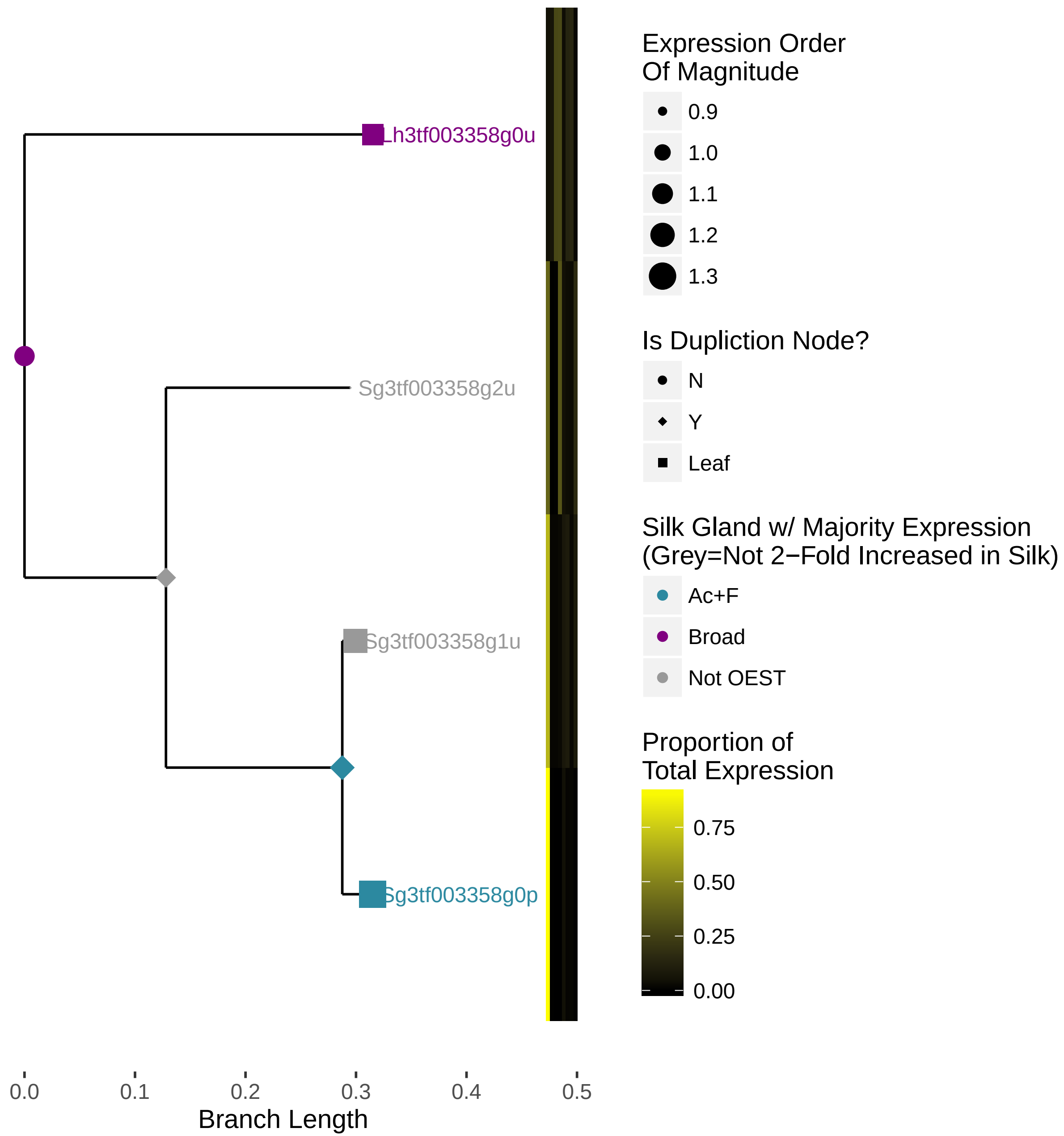

Lh3tf003358g0u
Sg3tf003358g2u
Sg3tf003358g1u
Sg3tf003358g0p
0.0
0.1
0.2
0.3
0.4
0.5
Branch Length
Expression Order
Of Magnitude
0.9
1.0
1.1
1.2
1.3
Is Dupliction Node?
N
Y
Leaf
Silk Gland w/ Majority Expression
(Grey=Not 2−Fold Increased in Silk)
Ac+F
Broad
Not OEST
Proportion of
Total Expression
0.75
0.50
0.25
0.00

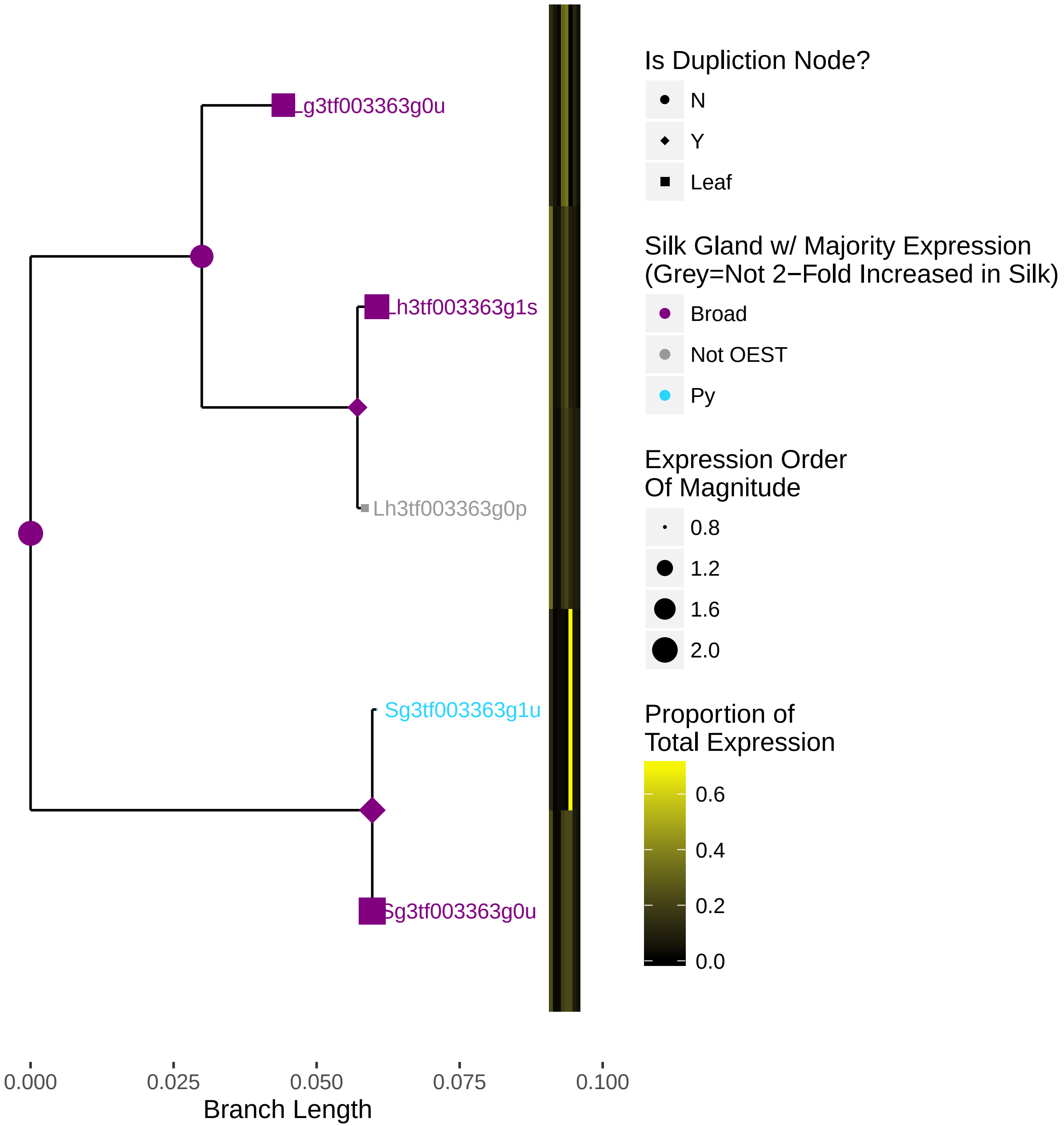
Lg3tf003363g0u
Lh3tf003363g1s
Lh3tf003363g0p
Sg3tf003363g1u
Sg3tf003363g0u
0.000
0.025
0.050
0.075
0.100
Branch Length
Is Dupliction Node?
N
Y
Leaf
Silk Gland w/ Majority Expression
(Grey=Not 2−Fold Increased in Silk)
Broad
Not OEST
Py
Expression Order
Of Magnitude
0.8
1.2
1.6
2.0
Proportion of
Total Expression
0.6
0.4
0.2
0.0

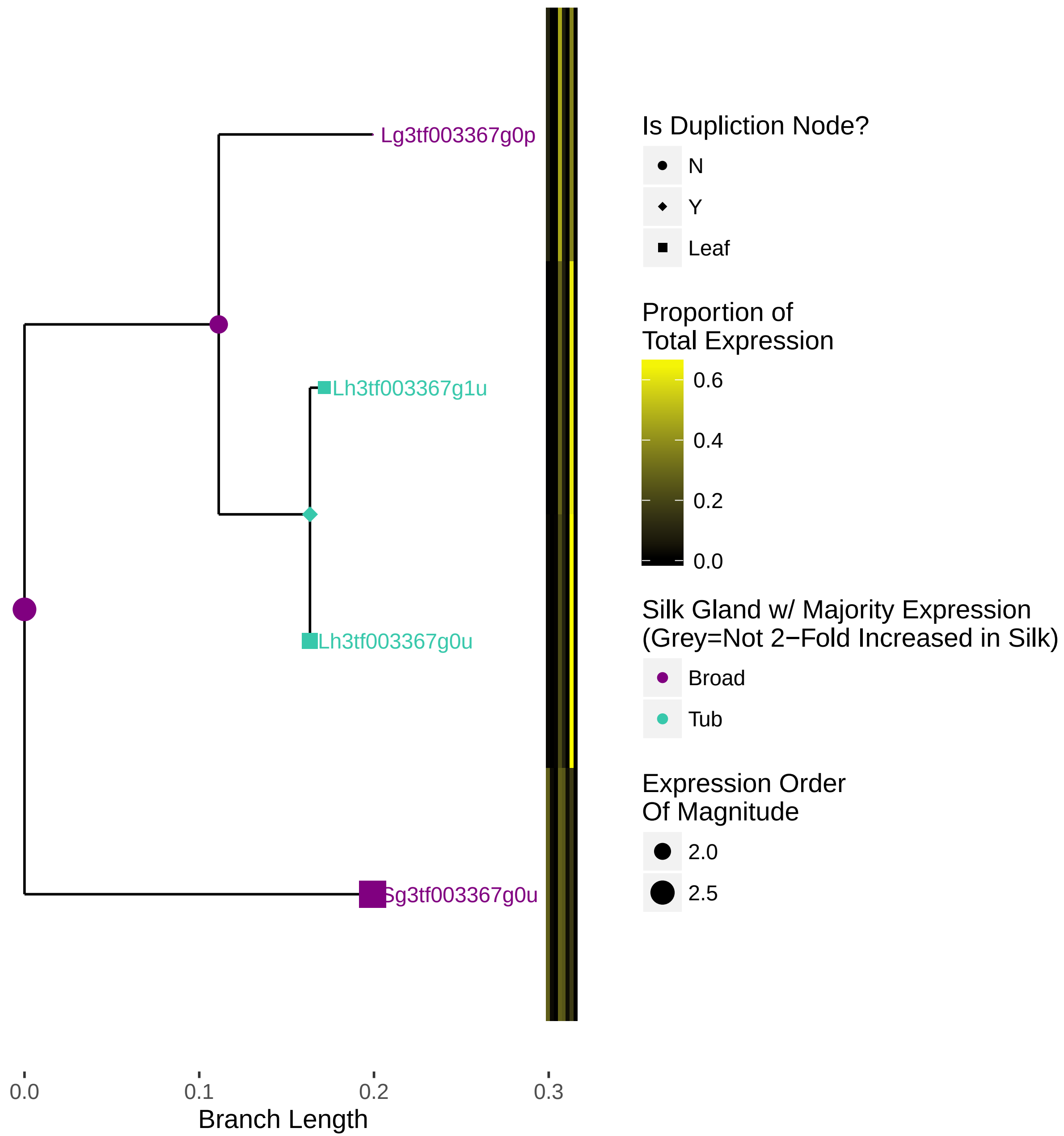

Lg3tf003367g0p
Lh3tf003367g1u
Lh3tf003367g0u
Sg3tf003367g0u
0.0
0.1
0.2
0.3
Branch Length
Is Dupliction Node?
N
Y
Leaf
Proportion of
Total Expression
0.6
0.4
0.2
0.0
Silk Gland w/ Majority Expression
(Grey=Not 2−Fold Increased in Silk)
Broad
Tub
Expression Order
Of Magnitude
2.0
2.5

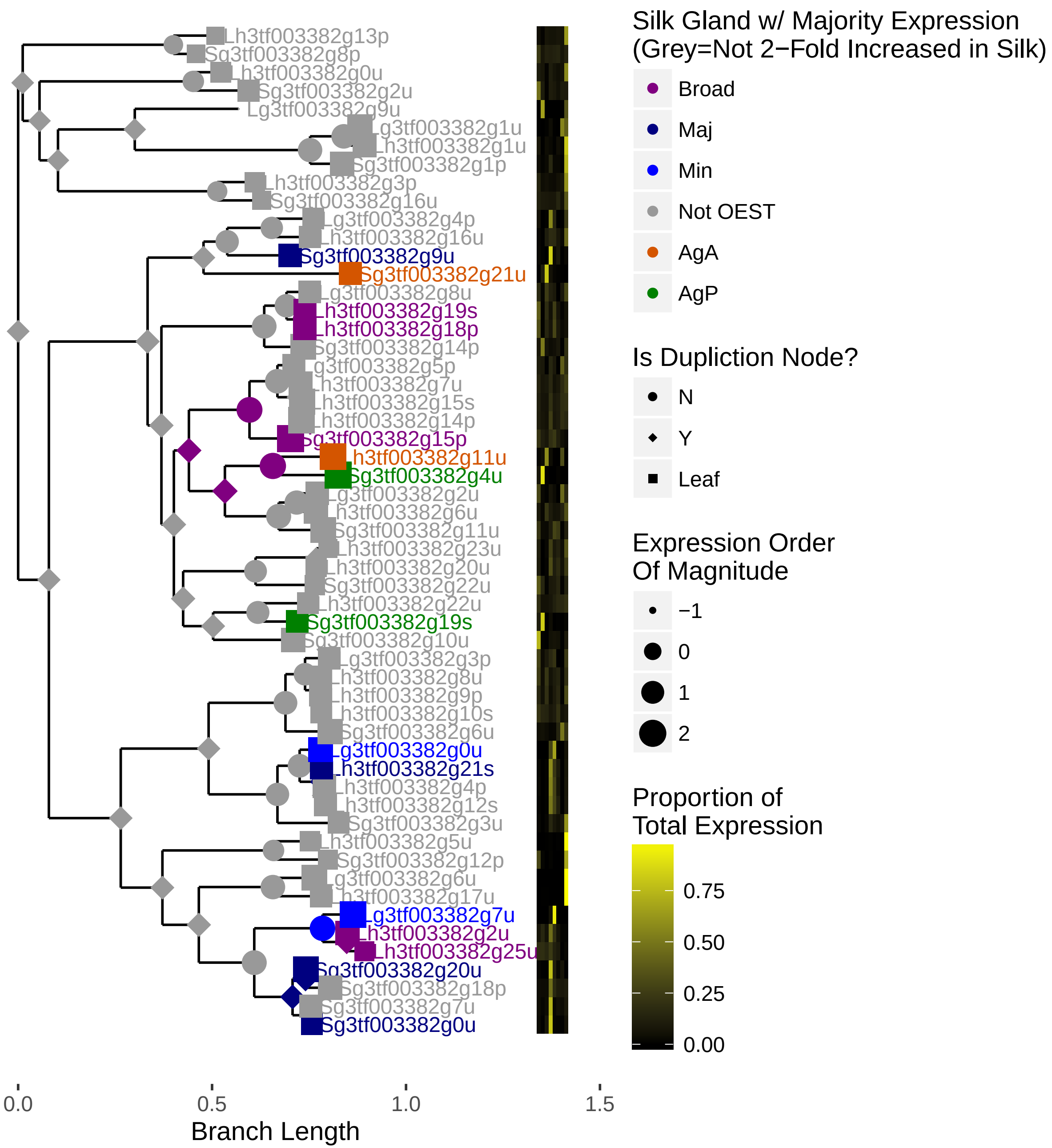
Lh3tf003382g13p
Sg3tf003382g8p
h3tf003382g0u
Sg3tf003382g2u
Lg3tf003382g9u
g3tf003382g1u
Lh3tf003382g1u
Sg3tf003382g1p
Lh3tf003382g3p
Sg3tf003382g16u
g3tf003382g4p
h3tf003382g16u
Sg3tf003382g9u
Sg3tf003382g21u
g3tf003382g8u
h3tf003382g19s
h3tf003382g18p
Sg3tf003382g14p
g3tf003382g5p
h3tf003382g7u
h3tf003382g15s
h3tf003382g14p
g3tf003382g15p
h3tf003382g11u
Sg3tf003382g4u
g3tf003382g2u
h3tf003382g6u
Sg3tf003382g11u
h3tf003382g23u
h3tf003382g20u
Sg3tf003382g22u
h3tf003382g22u
Sg3tf003382g19s
Sg3tf003382g10u
g3tf003382g3p
h3tf003382g8u
h3tf003382g9p
h3tf003382g10s
Sg3tf003382g6u
g3tf003382g0u
h3tf003382g21s
h3tf003382g4p
h3tf003382g12s
Sg3tf003382g3u
h3tf003382g5u
Sg3tf003382g12p
g3tf003382g6u
h3tf003382g17u
g3tf003382g7u
h3tf003382g2u
h3tf003382g25u
g3tf003382g20u
Sg3tf003382g18p
Sg3tf003382g7u
Sg3tf003382g0u
0.0
0.5
1.0
1.5
Branch Length
Silk Gland w/ Majority Expression
(Grey=Not 2−Fold Increased in Silk)
Broad
Maj
Min
Not OEST
AgA
AgP
Is Dupliction Node?
N
Y
Leaf
Expression Order
Of Magnitude
−1
0
1
2
Proportion of
Total Expression
0.75
0.50
0.25
0.00

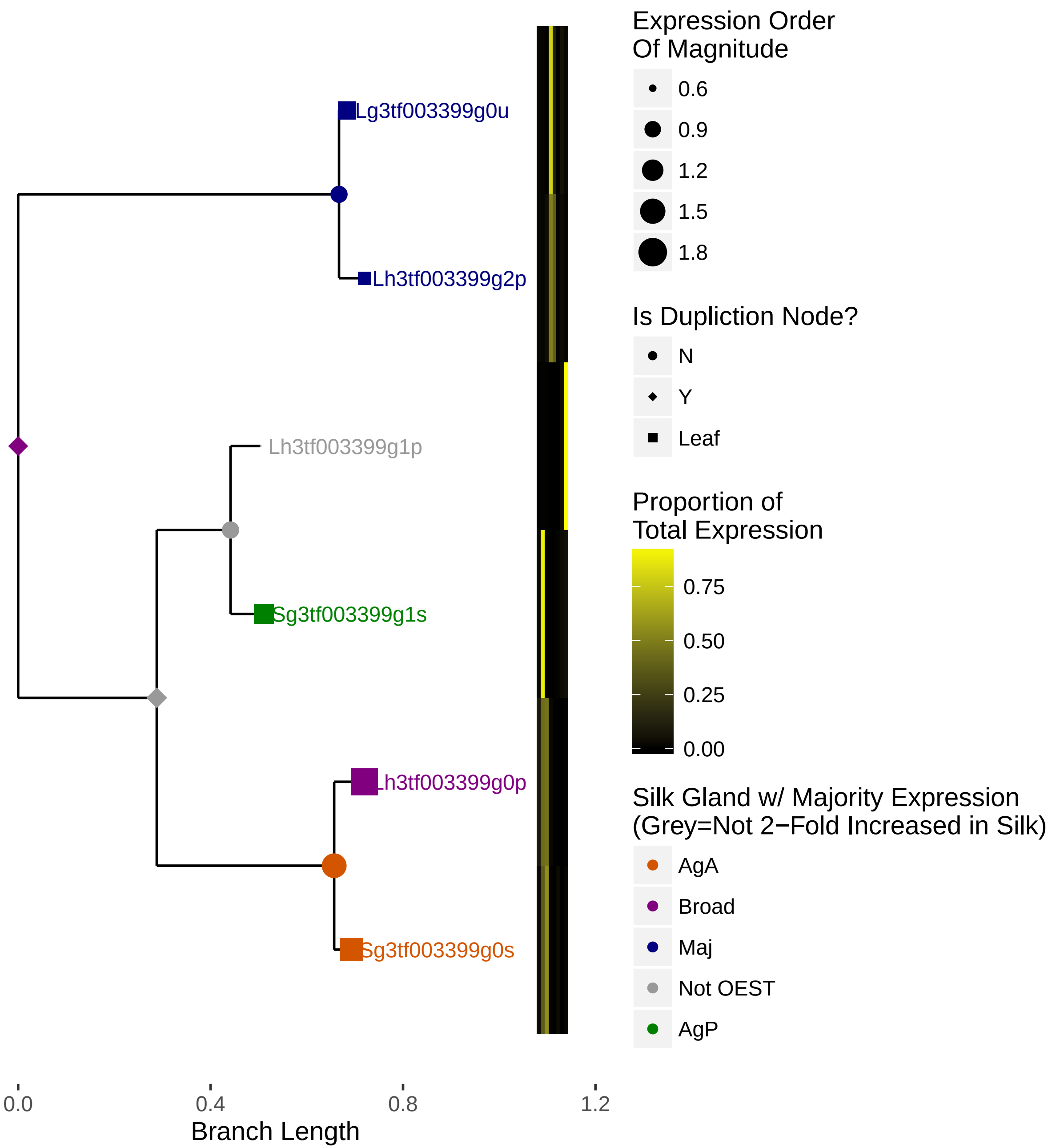

Lg3tf003399g0u
Lh3tf003399g2p
Lh3tf003399g1p
Sg3tf003399g1s
Lh3tf003399g0p
Sg3tf003399g0s
0.0
0.4
0.8
1.2
Branch Length
Expression Order
Of Magnitude
0.6
0.9
1.2
1.5
1.8
Is Dupliction Node?
N
Y
Leaf
Proportion of
Total Expression
0.75
0.50
0.25
0.00
Silk Gland w/ Majority Expression
(Grey=Not 2−Fold Increased in Silk)
AgA
Broad
Maj
Not OEST
AgP

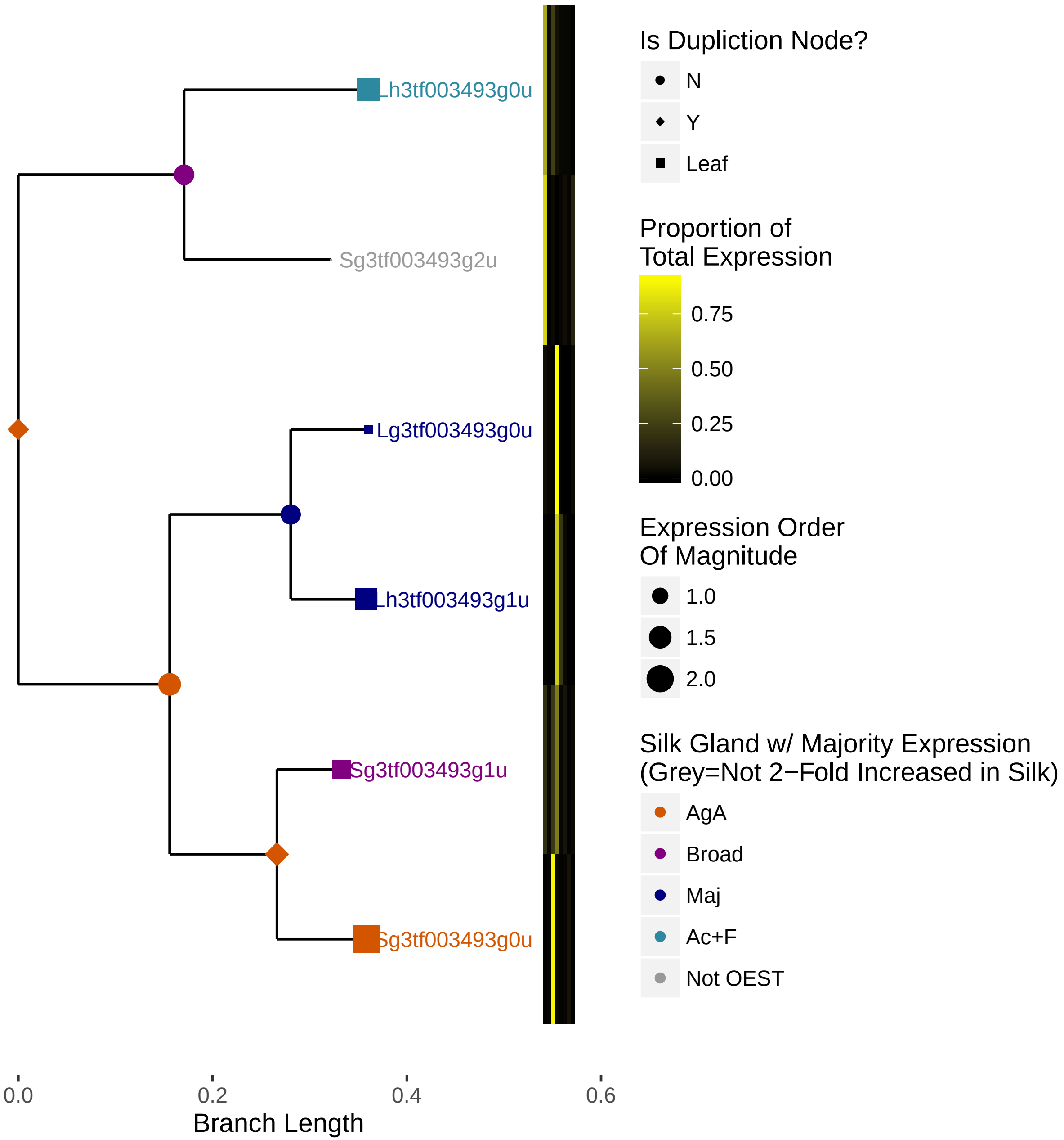

Lh3tf003493g0u
Sg3tf003493g2u
Lg3tf003493g0u
Lh3tf003493g1u
Sg3tf003493g1u
Sg3tf003493g0u
0.0
0.2
0.4
0.6
Branch Length
Is Dupliction Node?
N
Y
Leaf
Proportion of
Total Expression
0.75
0.50
0.25
0.00
Expression Order
Of Magnitude
1.0
1.5
2.0
Silk Gland w/ Majority Expression
(Grey=Not 2−Fold Increased in Silk)
AgA
Broad
Maj
Ac+F
Not OEST

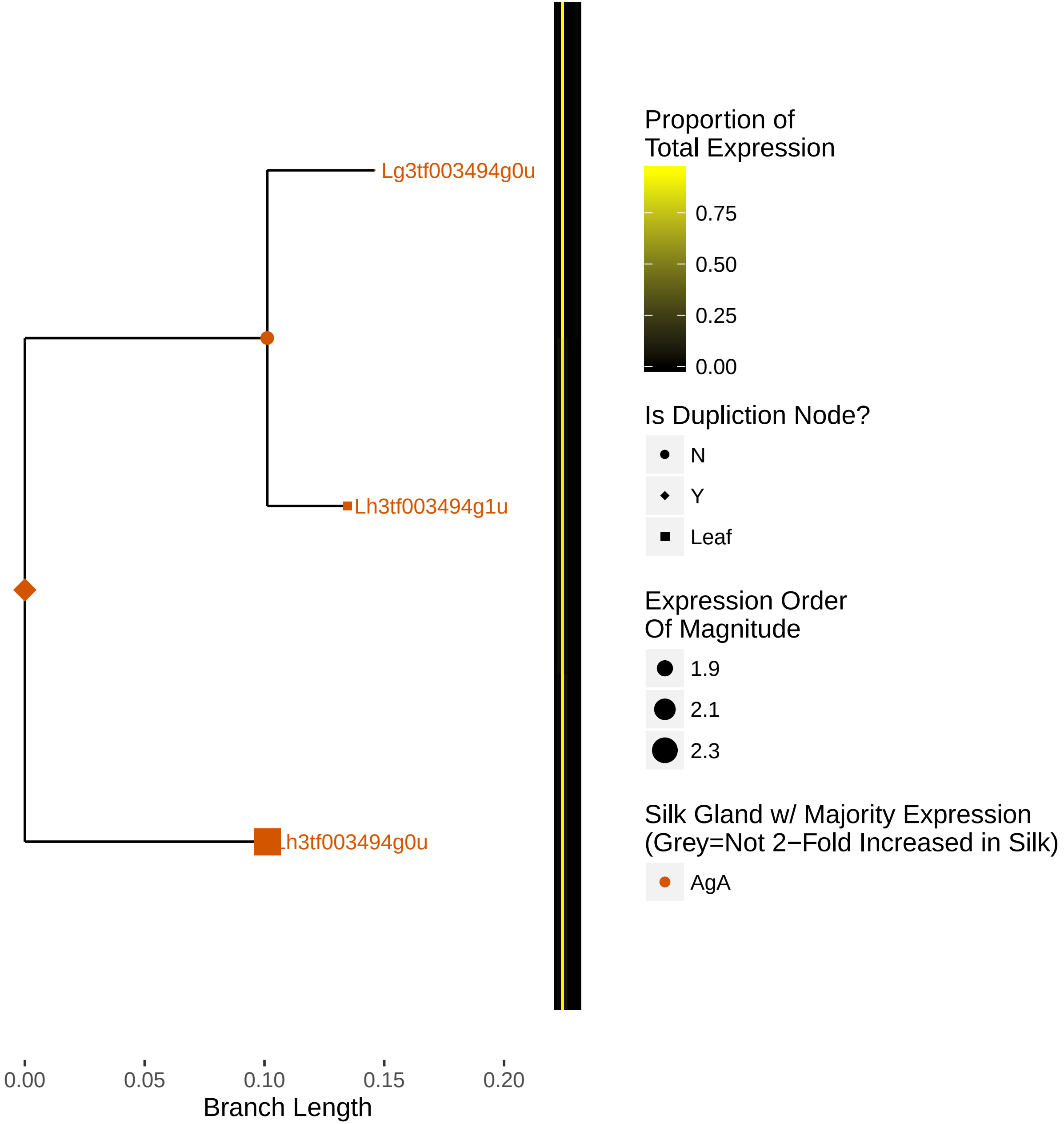

Lg3tf003494g0u
Lh3tf003494g1u
Lh3tf003494g0u
0.00
0.05
0.10
0.15
0.20
Branch Length
Proportion of
Total Expression
0.75
0.50
0.25
0.00
Is Dupliction Node?
N
Y
Leaf
Expression Order
Of Magnitude
1.9
2.1
2.3
Silk Gland w/ Majority Expression
(Grey=Not 2−Fold Increased in Silk)
AgA

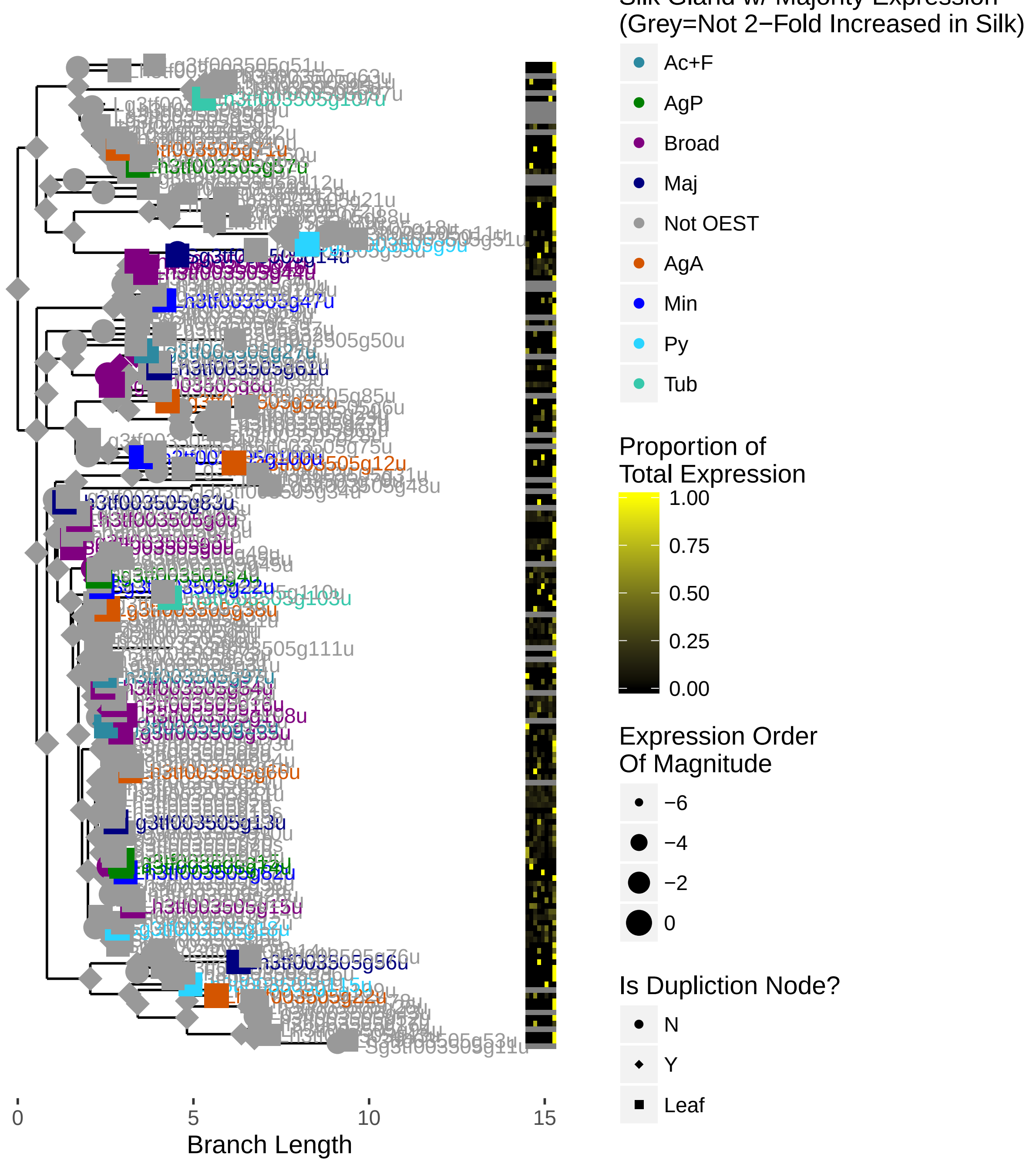

(Grey=Not 2−Fold Increased in Silk)
Ac+F
AgP
Broad
Maj
Not OEST
AgA
Min
Py
Tub
Proportion of
Total Expression
1.00
0.75
0.50
0.25
0.00
Expression Order
Of Magnitude
−6
−4
−2
0
Is Dupliction Node?
N
Y
Leaf
0
5
10
15
Branch Length

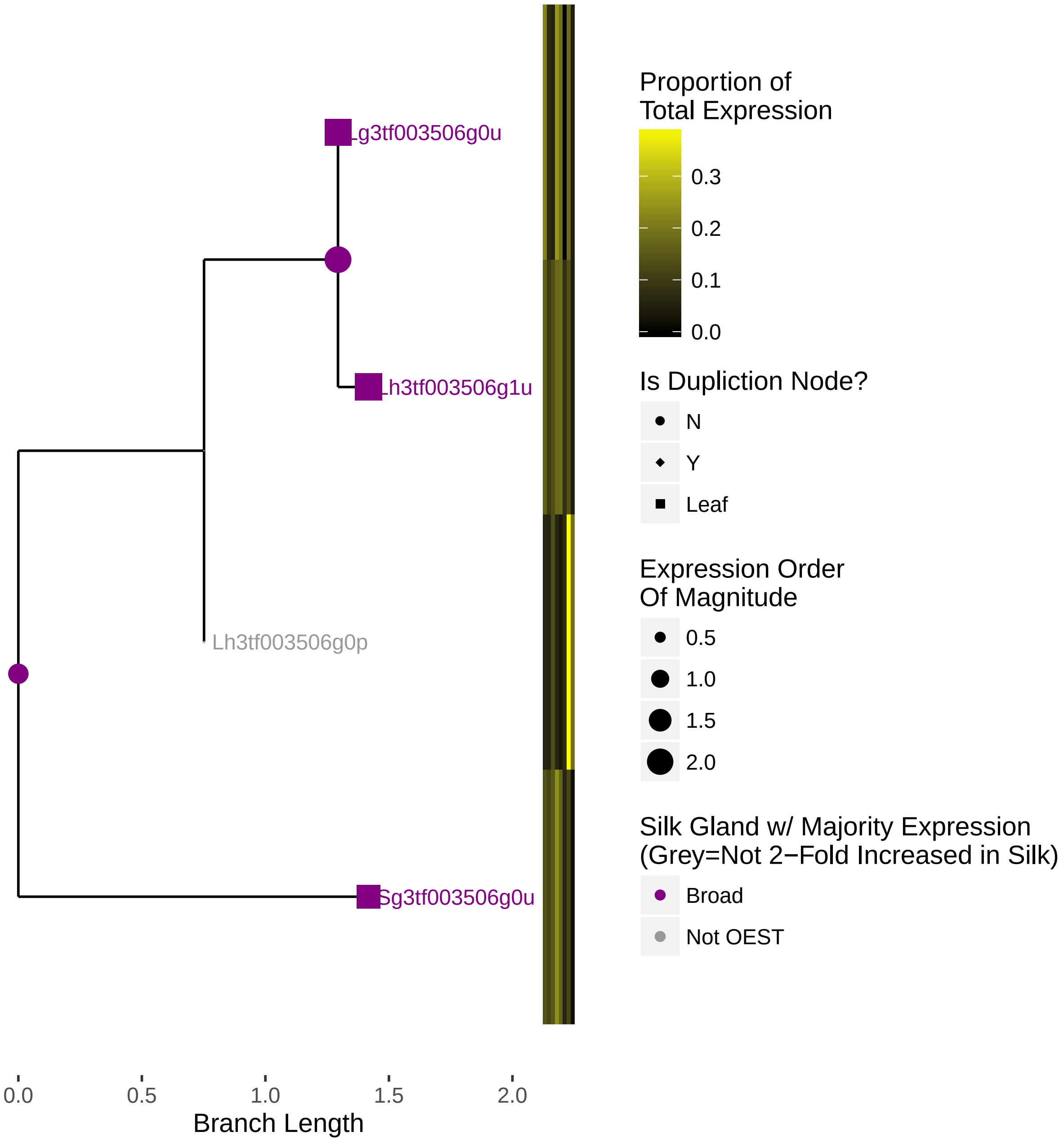

Lg3tf003506g0u
Lh3tf003506g1u
Lh3tf003506g0p
Sg3tf003506g0u
0.0
0.5
1.0
1.5
2.0
Branch Length
Proportion of
Total Expression
0.3
0.2
0.1
0.0
Is Dupliction Node?
N
Y
Leaf
Expression Order
Of Magnitude
0.5
1.0
1.5
2.0
Silk Gland w/ Majority Expression
(Grey=Not 2−Fold Increased in Silk)
Broad
Not OEST

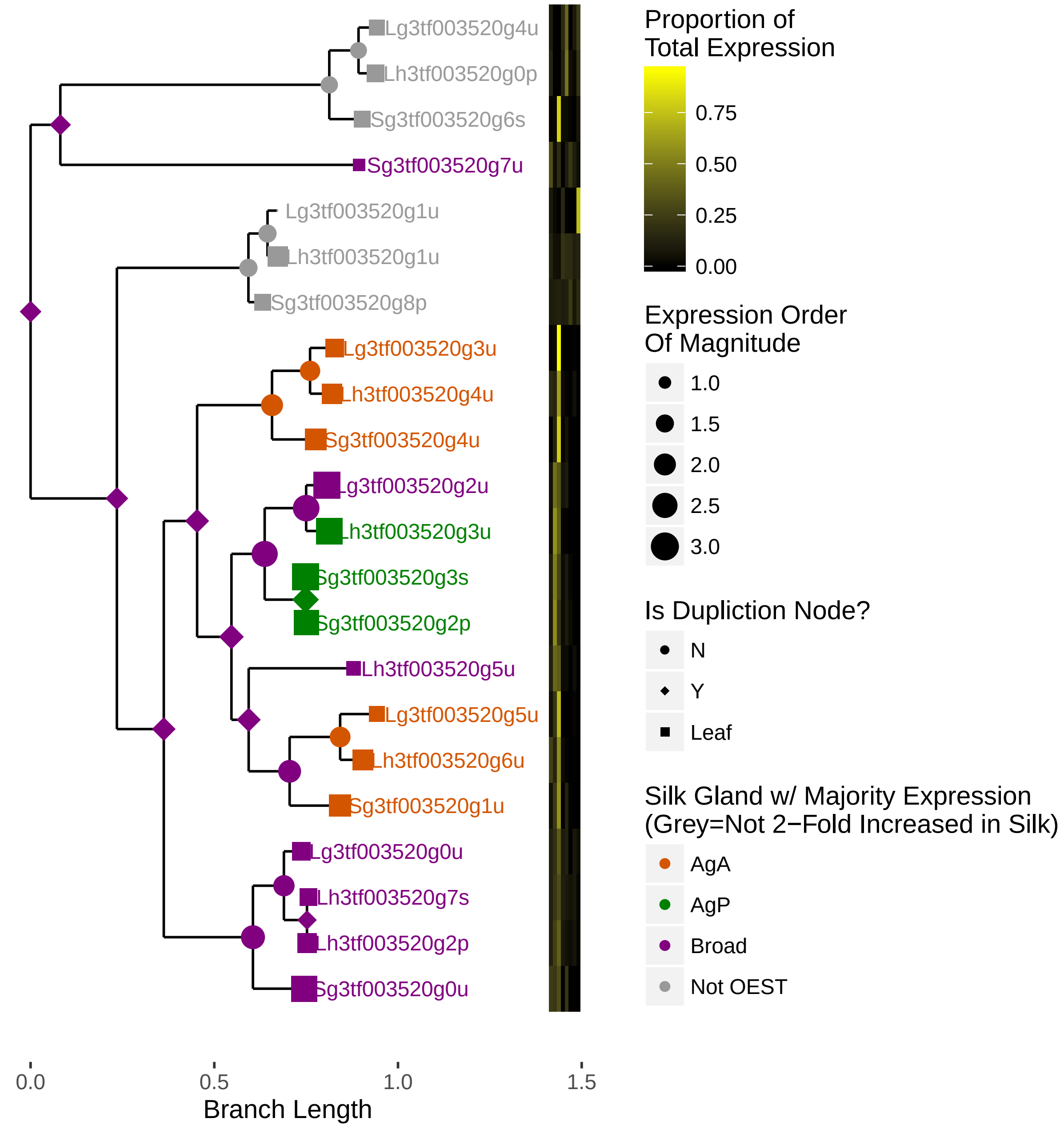

Lg3tf003520g4u
Lh3tf003520g0p
Sg3tf003520g6s
Sg3tf003520g7u
Lg3tf003520g1u
Lh3tf003520g1u
Sg3tf003520g8p
Lg3tf003520g3u
Lh3tf003520g4u
Sg3tf003520g4u
g3tf003520g2u
h3tf003520g3u
Sg3tf003520g3s
Sg3tf003520g2p
Lh3tf003520g5u
Lg3tf003520g5u
Lh3tf003520g6u
Sg3tf003520g1u
Lg3tf003520g0u
Lh3tf003520g7s
Lh3tf003520g2p
Sg3tf003520g0u
0.0
0.5
1.0
1.5
Branch Length
Proportion of
Total Expression
0.75
0.50
0.25
0.00
Expression Order
Of Magnitude
1.0
1.5
2.0
2.5
3.0
Is Dupliction Node?
N
Y
Leaf
Silk Gland w/ Majority Expression
(Grey=Not 2−Fold Increased in Silk)
AgA
AgP
Broad
Not OEST

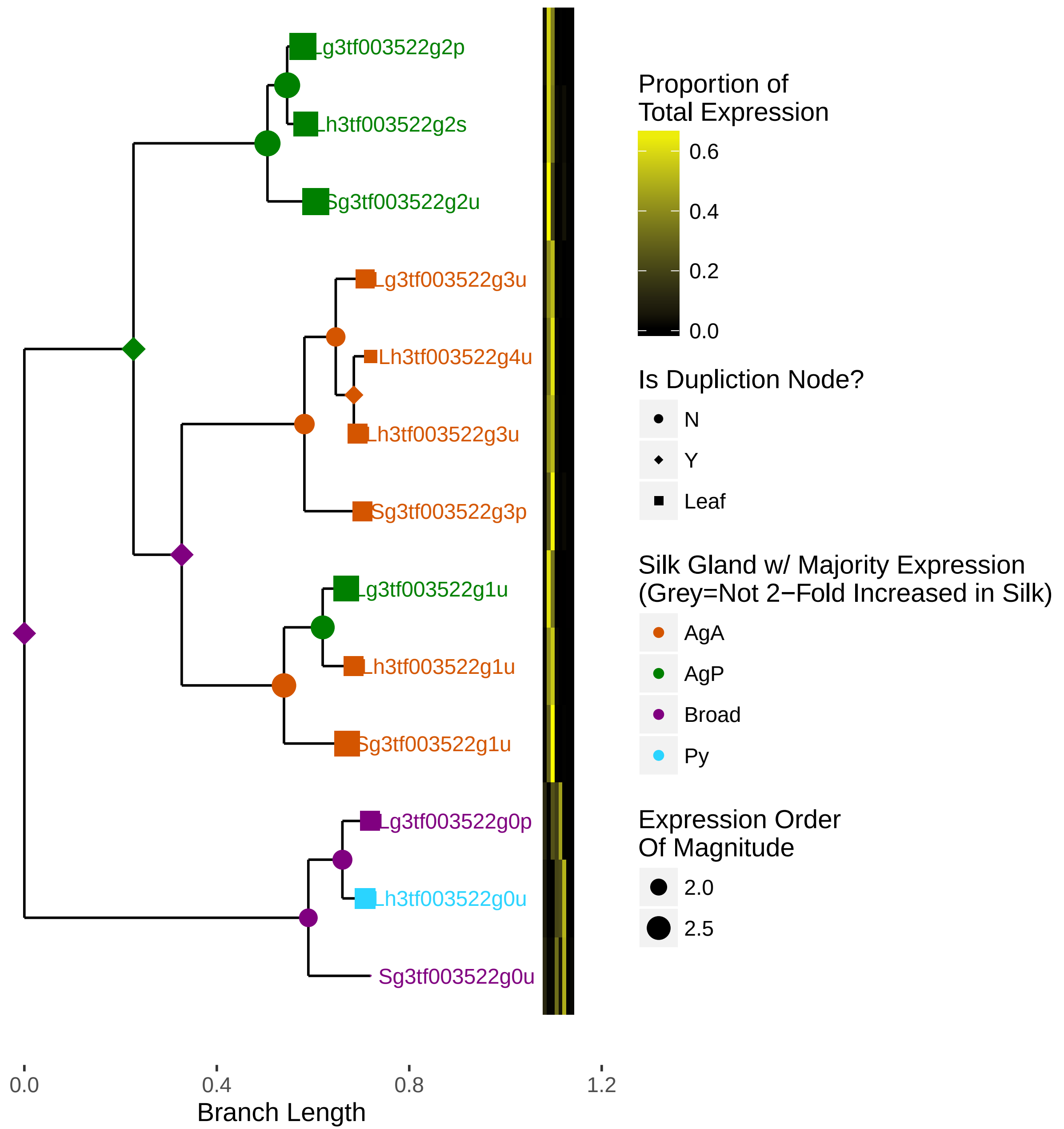
Lg3tf003522g2p
Lh3tf003522g2s
Sg3tf003522g2u
Lg3tf003522g3u
Lh3tf003522g4u
Lh3tf003522g3u
Sg3tf003522g3p
Lg3tf003522g1u
Lh3tf003522g1u
Sg3tf003522g1u
Lg3tf003522g0p
Lh3tf003522g0u
Sg3tf003522g0u
0.0
0.4
0.8
1.2
Branch Length
Proportion of
Total Expression
0.6
0.4
0.2
0.0
Is Dupliction Node?
N
Y
Leaf
Silk Gland w/ Majority Expression
(Grey=Not 2−Fold Increased in Silk)
AgA
AgP
Broad
Py
Expression Order
Of Magnitude
2.0
2.5

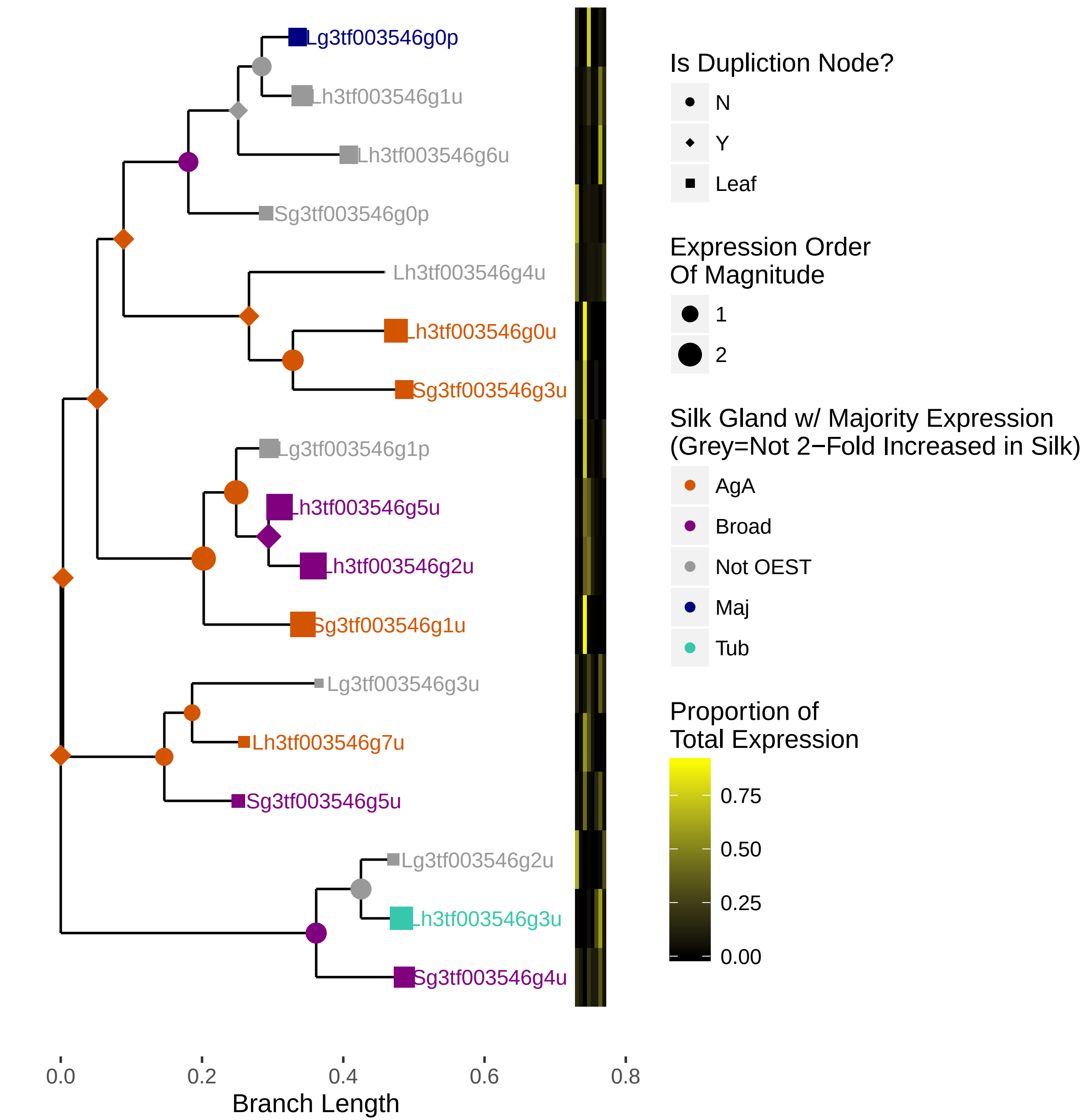

Lg3tf003546g0p
Lh3tf003546g1u
Lh3tf003546g6u
Sg3tf003546g0p
Lh3tf003546g4u
Lh3tf003546g0u
Sg3tf003546g3u
Lg3tf003546g1p
Lh3tf003546g5u
Lh3tf003546g2u
Sg3tf003546g1u
Lg3tf003546g3u
Lh3tf003546g7u
Sg3tf003546g5u
Lg3tf003546g2u
Lh3tf003546g3u
Sg3tf003546g4u
0.0
0.2
0.4
0.6
0.8
Branch Length
Is Dupliction Node?
N
Y
Leaf
Expression Order Of Magnitude
1
2
Silk Gland w/ Majority Expression (Grey=Not 2−Fold Increased in Silk)
AgA
Broad
Not OEST
Maj
Tub
Proportion of Total Expression
0.75
0.50
0.25
0.00

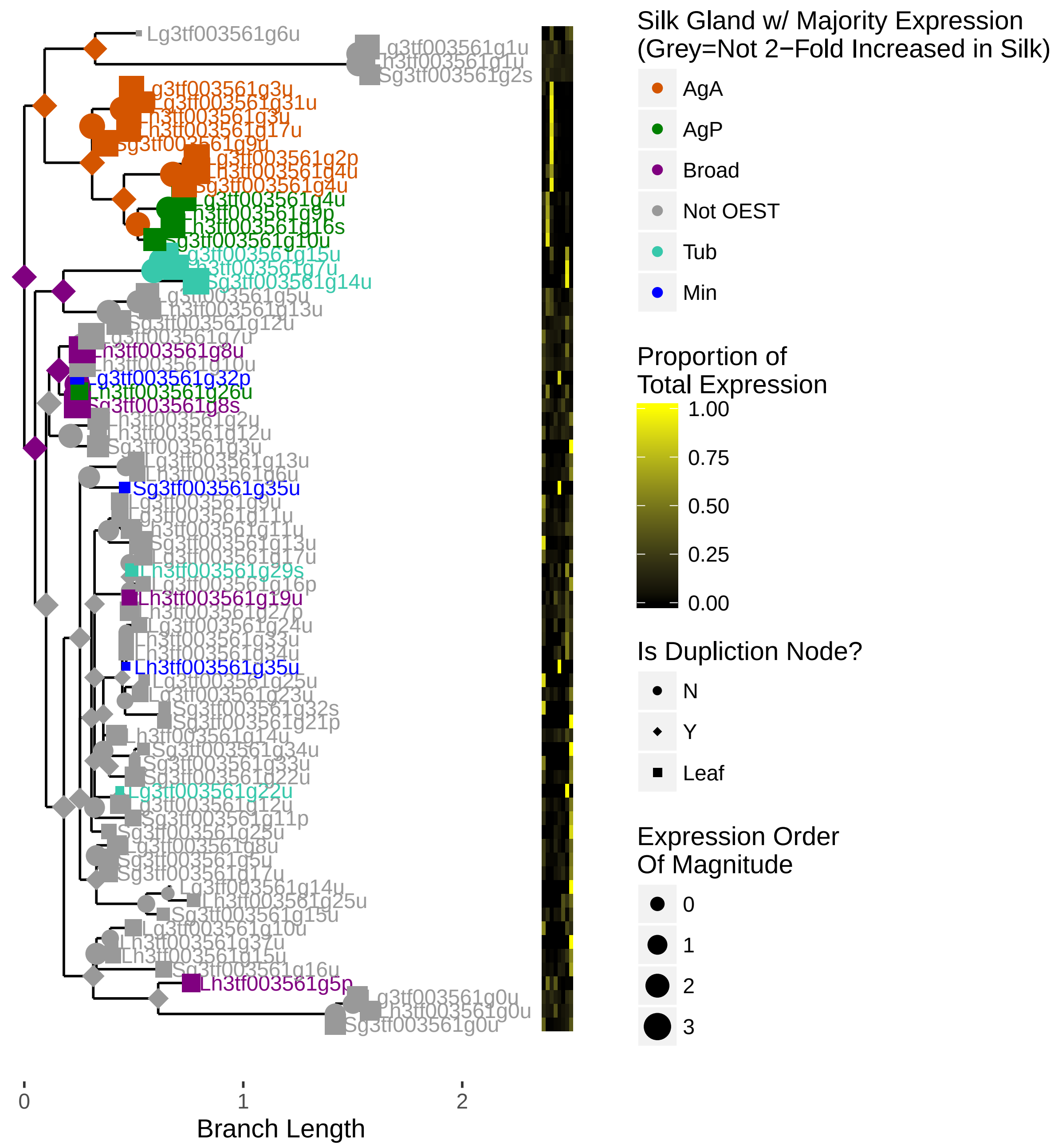

Lg3tf003561g6u
Sg3tf003561g2s
Lg3tf003561g31u
Lh3tf003561g17u
Sg3tf003561g4u
Lh3tf003561g16s
Sg3tf003561g14u
Lh3tf003561g13u
Sg3tf003561g12u
Lh3tf003561g26u
Lh3tf003561g12u
Sg3tf003561g3u
Lg3tf003561g13u
Sg3tf003561g35u
Lh3tf003561g29s
Lh3tf003561g19u
Lg3tf003561g24u
Lh3tf003561g33u
Lh3tf003561g34u
Lh3tf003561g35u
Lg3tf003561g25u
Lg3tf003561g23u
Sg3tf003561g32s
Sg3tf003561g21p
Sg3tf003561g34u
Sg3tf003561g33u
Sg3tf003561g22u
Lg3tf003561g22u
Sg3tf003561g11p
Sg3tf003561g25u
Lg3tf003561g8u
Sg3tf003561g5u
Sg3tf003561g17u
Lg3tf003561g14u
Lh3tf003561g25u
Sg3tf003561g15u
Lg3tf003561g10u
Lh3tf003561g37u
Lh3tf003561g15u
Sg3tf003561g16u
Lh3tf003561g5p
Lh3tf003561g0u
Sg3tf003561g0u
0
1
2
Branch Length
Silk Gland w/ Majority Expression
(Grey=Not 2−Fold Increased in Silk)
AgA
AgP
Broad
Not OEST
Tub
Min
Proportion of
Total Expression
1.00
0.75
0.50
0.25
0.00
Is Dupliction Node?
N
Y
Leaf
Expression Order
Of Magnitude
0
1
2
3

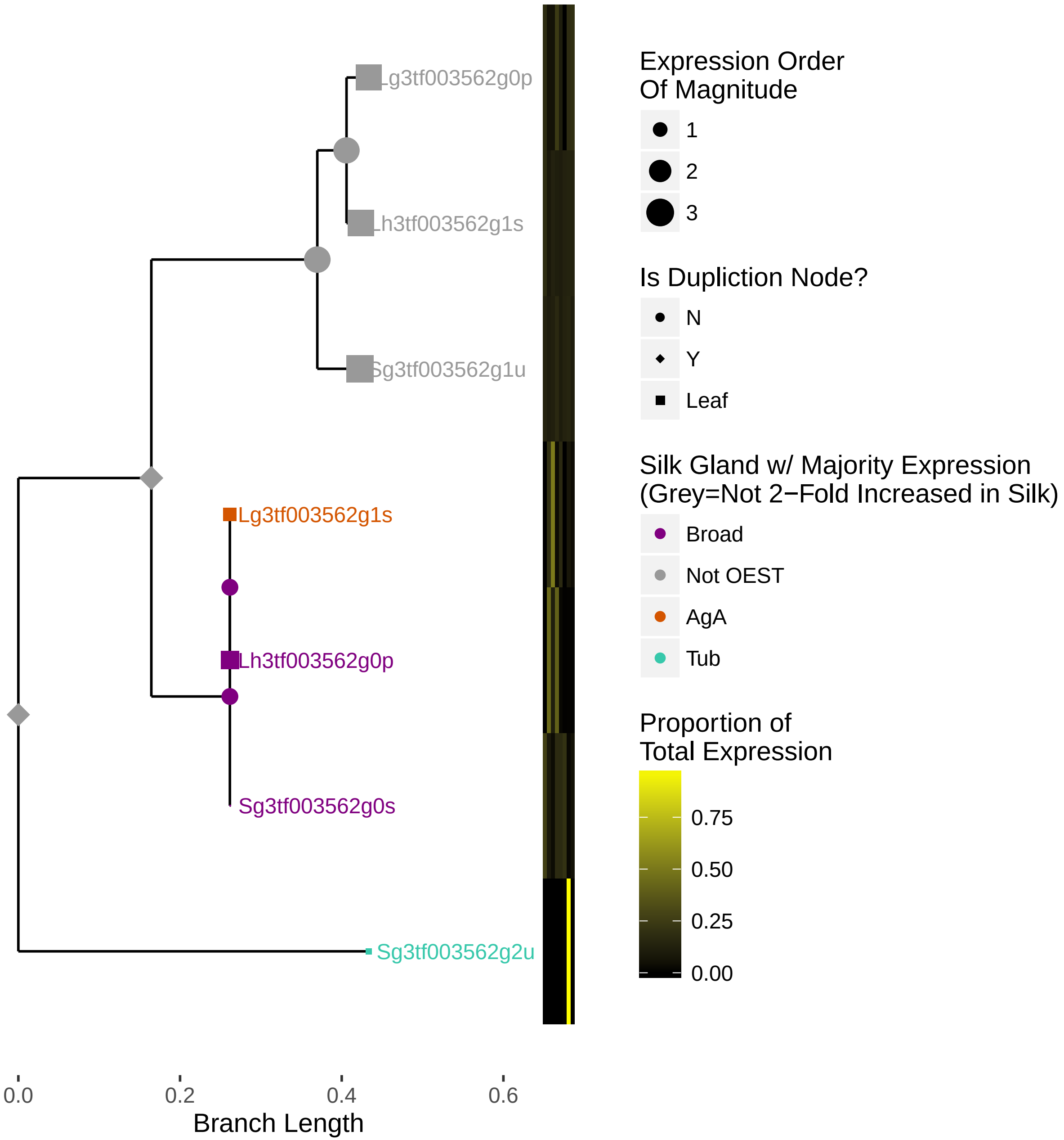

Lg3tf003562g0p
Lh3tf003562g1s
Sg3tf003562g1u
Lg3tf003562g1s
Lh3tf003562g0p
Sg3tf003562g0s
Sg3tf003562g2u
0.0
0.2
0.4
0.6
Branch Length
Expression Order
Of Magnitude
1
2
3
Is Dupliction Node?
N
Y
Leaf
Silk Gland w/ Majority Expression
(Grey=Not 2−Fold Increased in Silk)
Broad
Not OEST
AgA
Tub
Proportion of
Total Expression
0.75
0.50
0.25
0.00

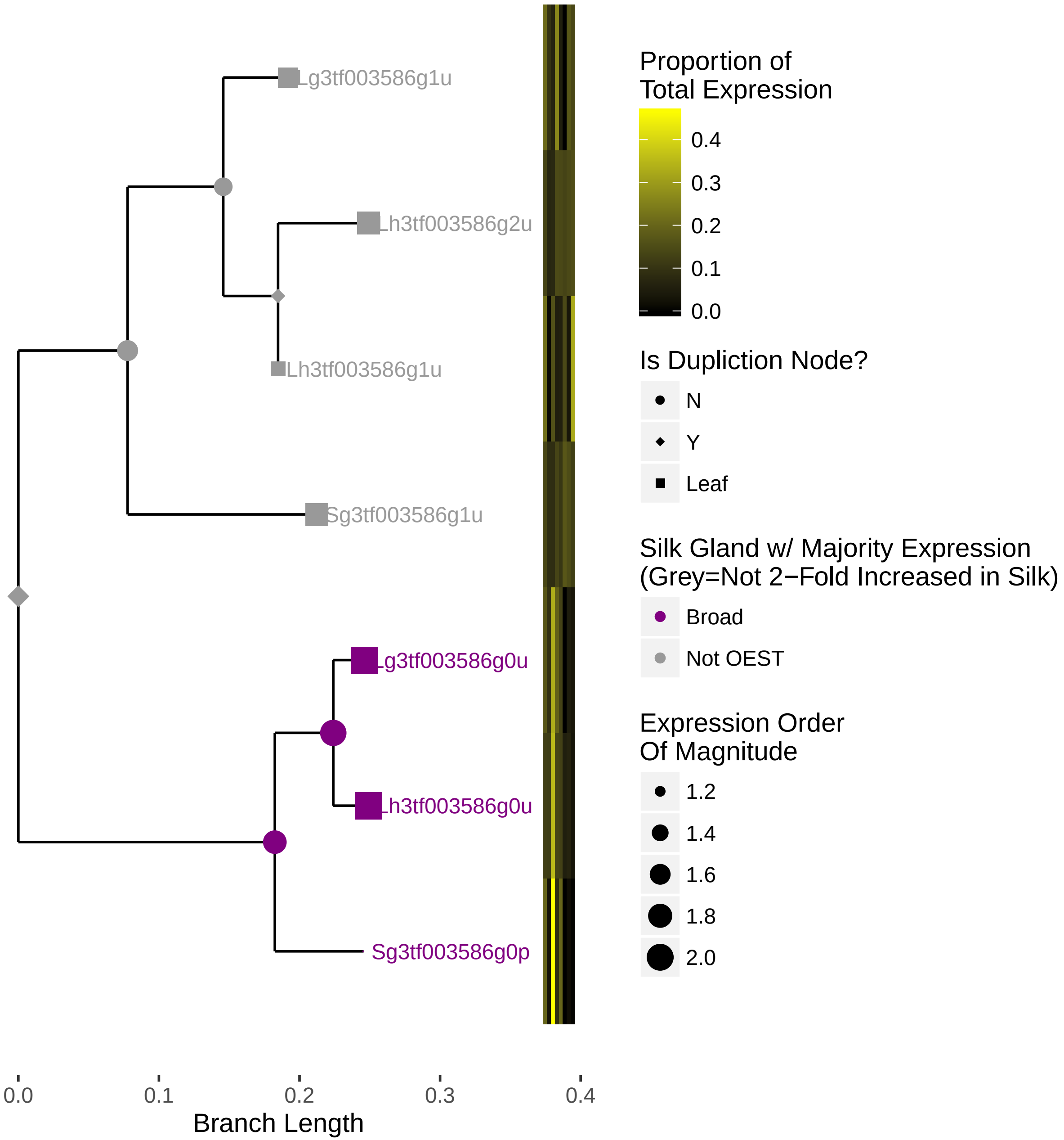
Lg3tf003586g1u
Lh3tf003586g2u
Lh3tf003586g1u
Sg3tf003586g1u
Lg3tf003586g0u
Lh3tf003586g0u
Sg3tf003586g0p
0.0
0.1
0.2
0.3
0.4
Branch Length
Proportion of
Total Expression
0.4
0.3
0.2
0.1
0.0
Is Dupliction Node?
N
Y
Leaf
Silk Gland w/ Majority Expression
(Grey=Not 2−Fold Increased in Silk)
Broad
Not OEST
Expression Order
Of Magnitude
1.2
1.4
1.6
1.8
2.0

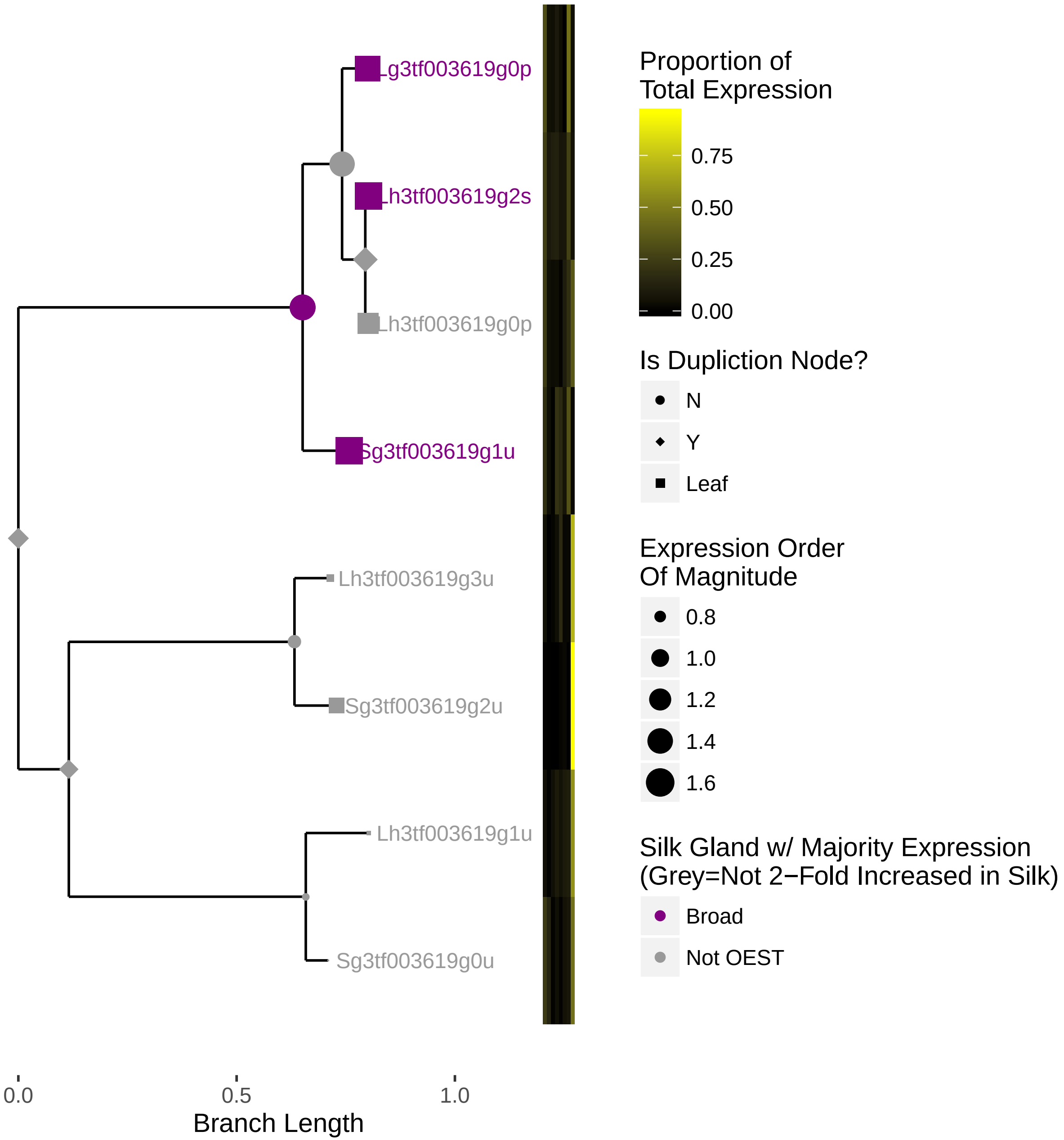

Lg3tf003619g0p
Lh3tf003619g2s
Lh3tf003619g0p
Sg3tf003619g1u
Lh3tf003619g3u
Sg3tf003619g2u
Lh3tf003619g1u
Sg3tf003619g0u
0.0
0.5
1.0
Branch Length
Proportion of
Total Expression
0.75
0.50
0.25
0.00
Is Dupliction Node?
N
Y
Leaf
Expression Order
Of Magnitude
0.8
1.0
1.2
1.4
1.6
Silk Gland w/ Majority Expression
(Grey=Not 2−Fold Increased in Silk)
Broad
Not OEST

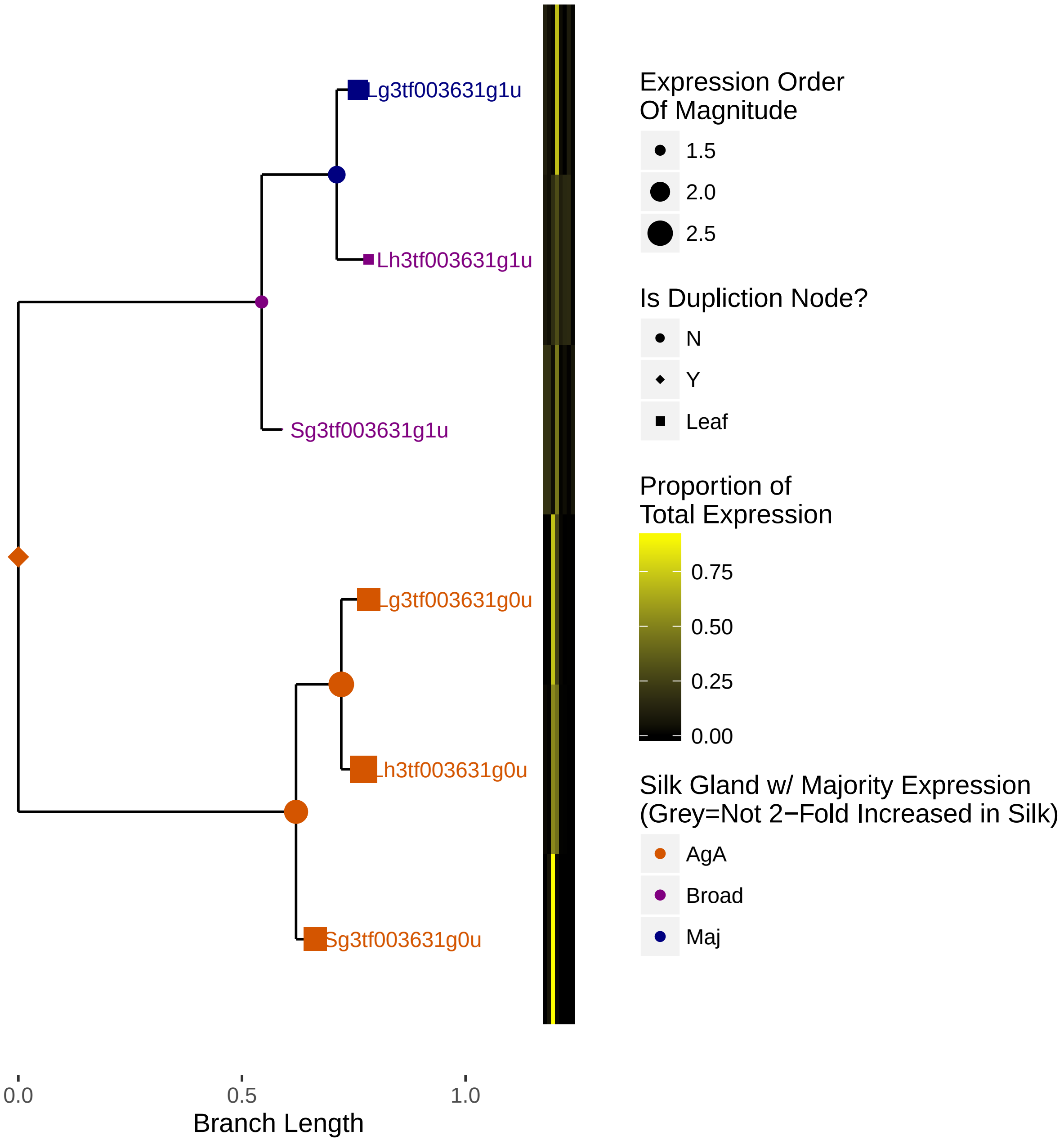
Lg3tf003631g1u
Lh3tf003631g1u
Sg3tf003631g1u
Lg3tf003631g0u
Lh3tf003631g0u
Sg3tf003631g0u
0.0
0.5
1.0
Branch Length
Expression Order
Of Magnitude
1.5
2.0
2.5
Is Dupliction Node?
N
Y
Leaf
Proportion of
Total Expression
0.75
0.50
0.25
0.00
Silk Gland w/ Majority Expression
(Grey=Not 2−Fold Increased in Silk)
AgA
Broad
Maj

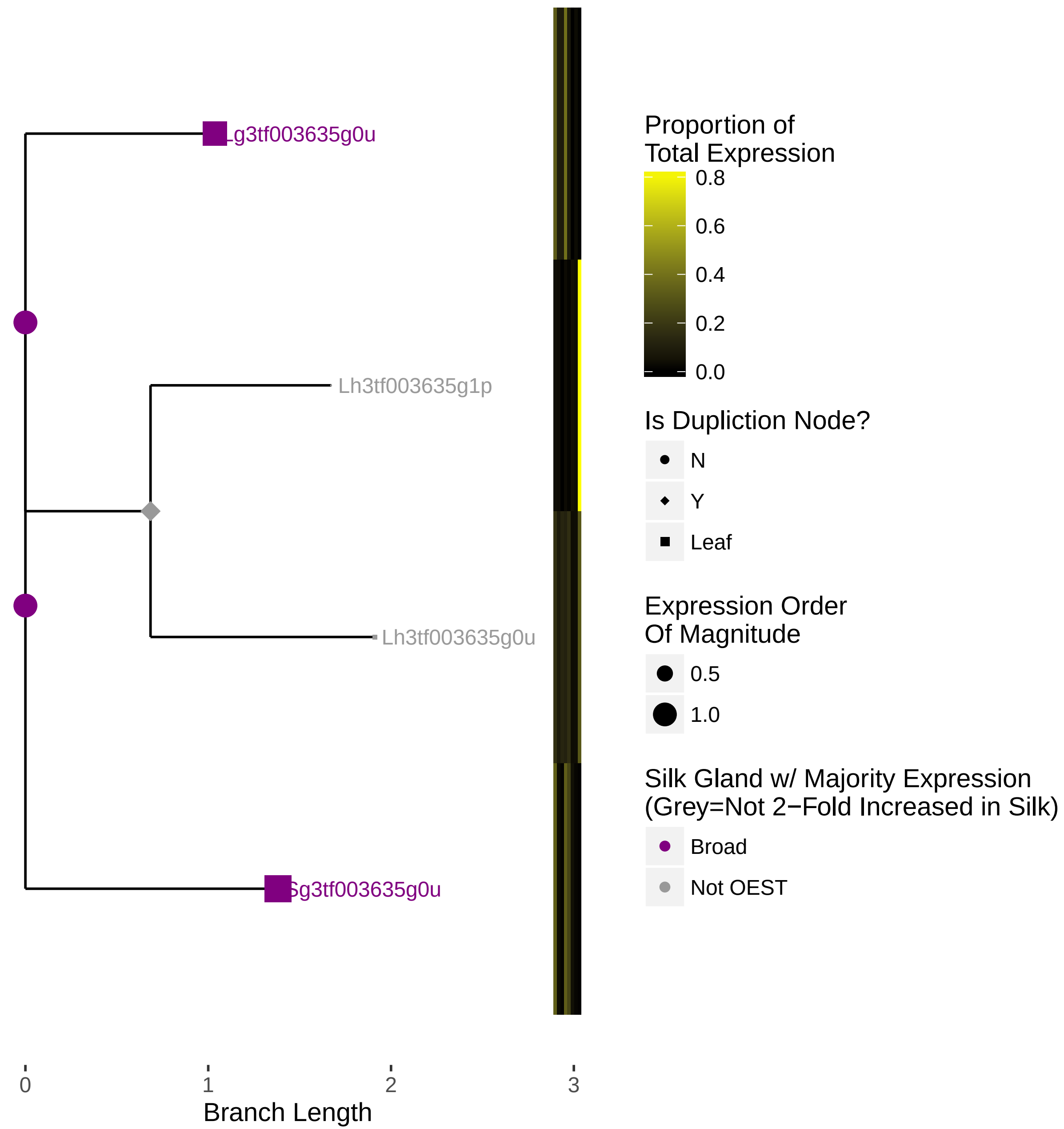

Lg3tf003635g0u
Lh3tf003635g1p
Lh3tf003635g0u
Sg3tf003635g0u
0
1
2
3
Branch Length
Proportion of Total Expression
0.8
0.6
0.4
0.2
0.0
Is Dupliction Node?
N
Y
Leaf
Expression Order Of Magnitude
0.5
1.0
Silk Gland w/ Majority Expression (Grey=Not 2−Fold Increased in Silk)
Broad
Not OEST

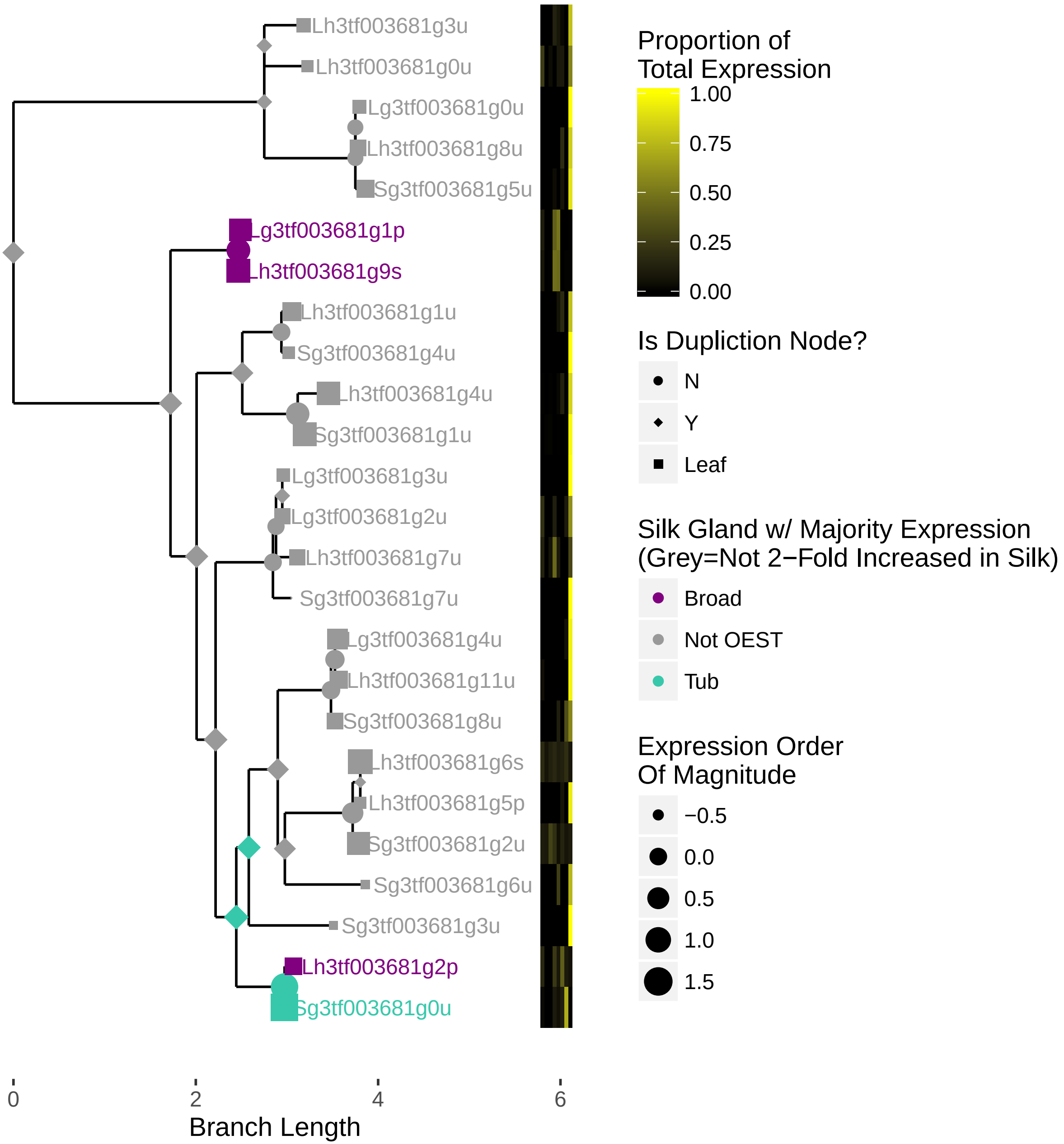
Lh3tf003681g3u
Lh3tf003681g0u
Lg3tf003681g0u
Lh3tf003681g8u
Sg3tf003681g5u
Lg3tf003681g1p
Lh3tf003681g9s
Lh3tf003681g1u
Sg3tf003681g4u
Lh3tf003681g4u
Sg3tf003681g1u
Lg3tf003681g3u
Lg3tf003681g2u
Lh3tf003681g7u
Sg3tf003681g7u
Lg3tf003681g4u
Lh3tf003681g11u
Sg3tf003681g8u
Lh3tf003681g6s
Lh3tf003681g5p
Sg3tf003681g2u
Sg3tf003681g6u
Sg3tf003681g3u
Lh3tf003681g2p
Sg3tf003681g0u
0
2
4
6
Branch Length
Proportion of
Total Expression
1.00
0.75
0.50
0.25
0.00
Is Dupliction Node?
N
Y
Leaf
Silk Gland w/ Majority Expression
(Grey=Not 2−Fold Increased in Silk)
Broad
Not OEST
Tub
Expression Order
Of Magnitude
−0.5
0.0
0.5
1.0
1.5

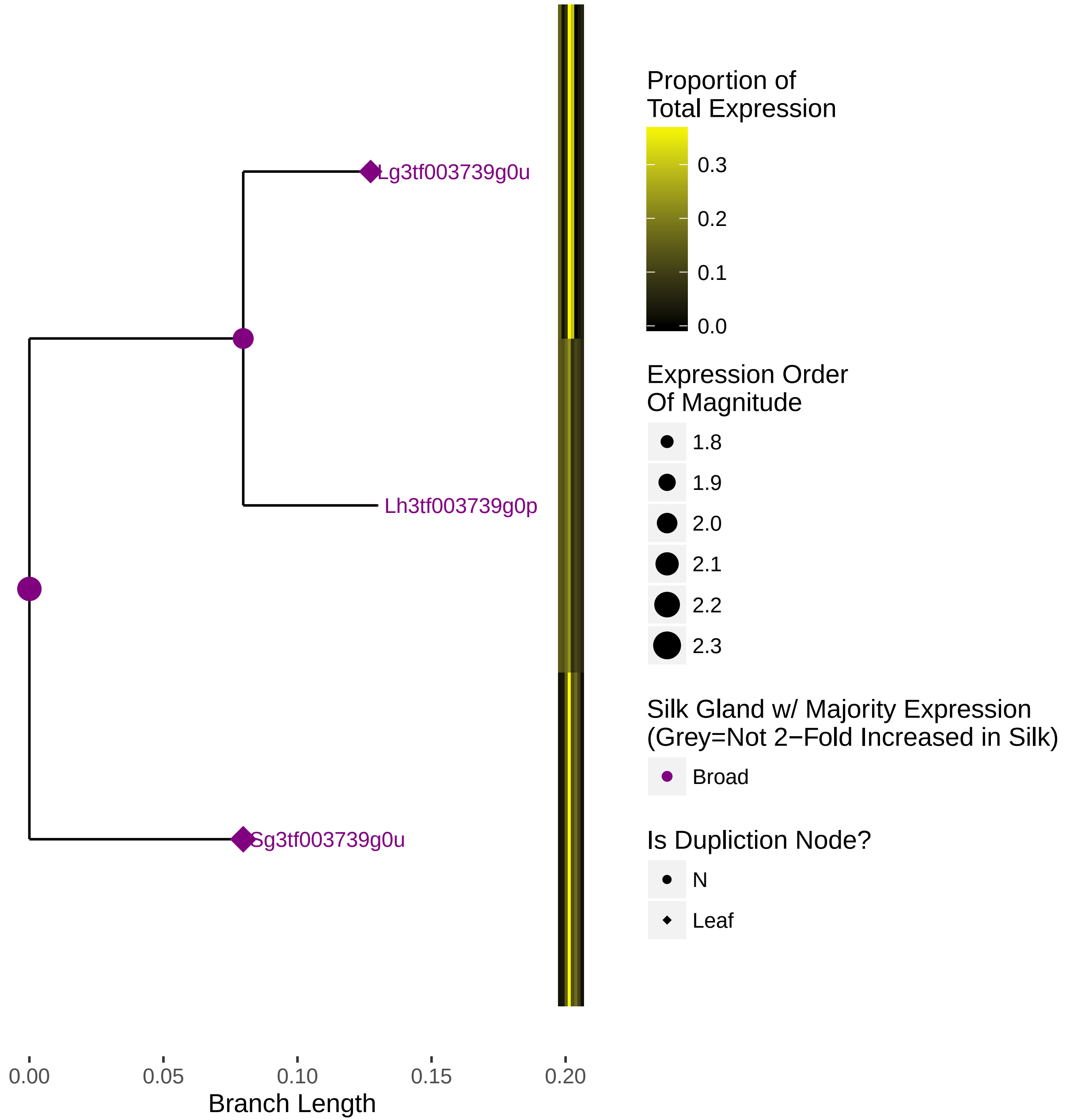
Lg3tf003739g0u
Lh3tf003739g0p
Sg3tf003739g0u
0.00
0.05
0.10
0.15
0.20
Branch Length
Proportion of
Total Expression
0.3
0.2
0.1
0.0
Expression Order
Of Magnitude
1.8
1.9
2.0
2.1
2.2
2.3
Silk Gland w/ Majority Expression
(Grey=Not 2−Fold Increased in Silk)
Broad
Is Dupliction Node?
N
Leaf

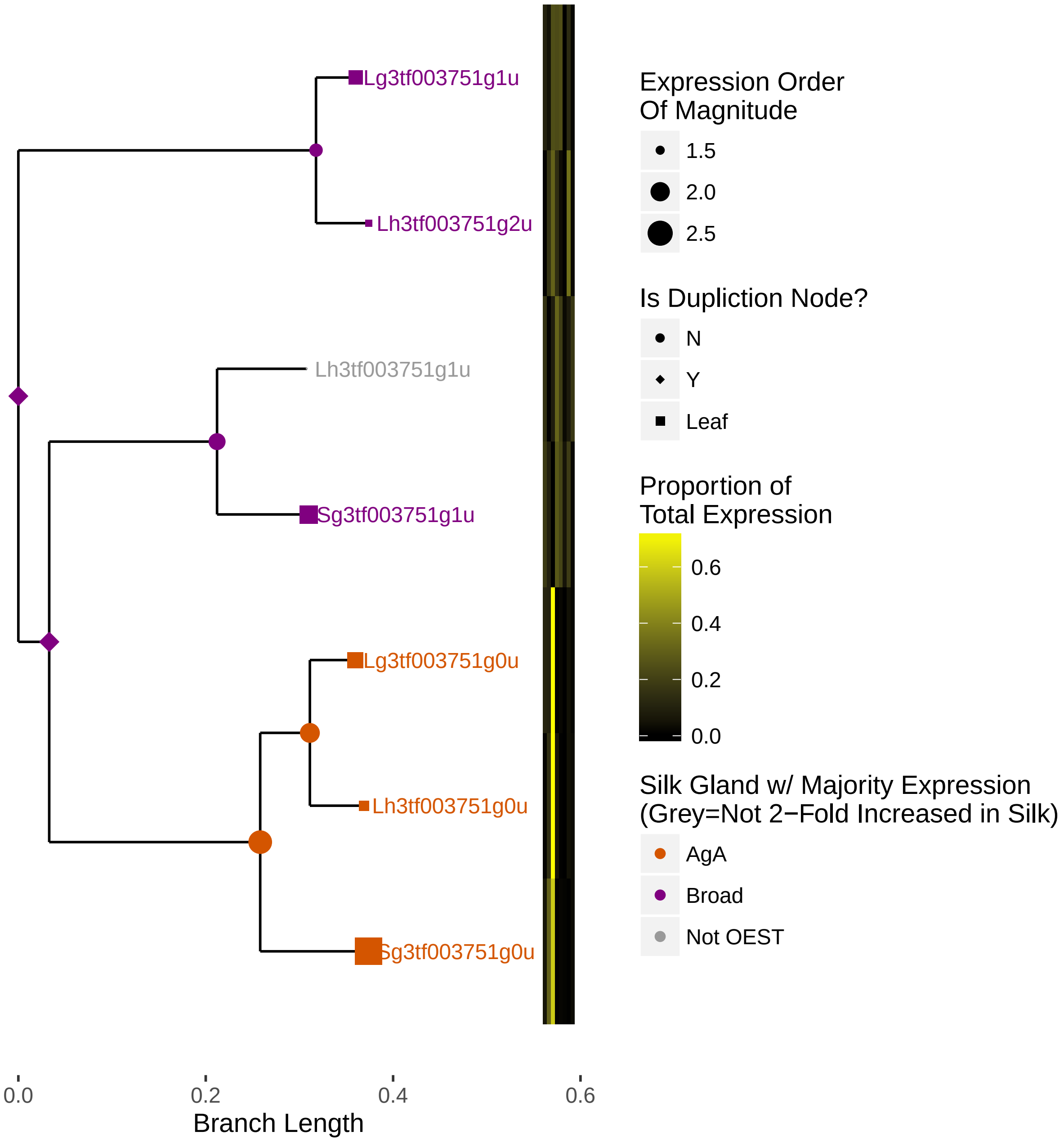
Lg3tf003751g1u
Lh3tf003751g2u
Lh3tf003751g1u
Sg3tf003751g1u
Lg3tf003751g0u
Lh3tf003751g0u
Sg3tf003751g0u
0.0
0.2
0.4
0.6
Branch Length
Expression Order
Of Magnitude
1.5
2.0
2.5
Is Dupliction Node?
N
Y
Leaf
Proportion of
Total Expression
0.6
0.4
0.2
0.0
Silk Gland w/ Majority Expression
(Grey=Not 2−Fold Increased in Silk)
AgA
Broad
Not OEST

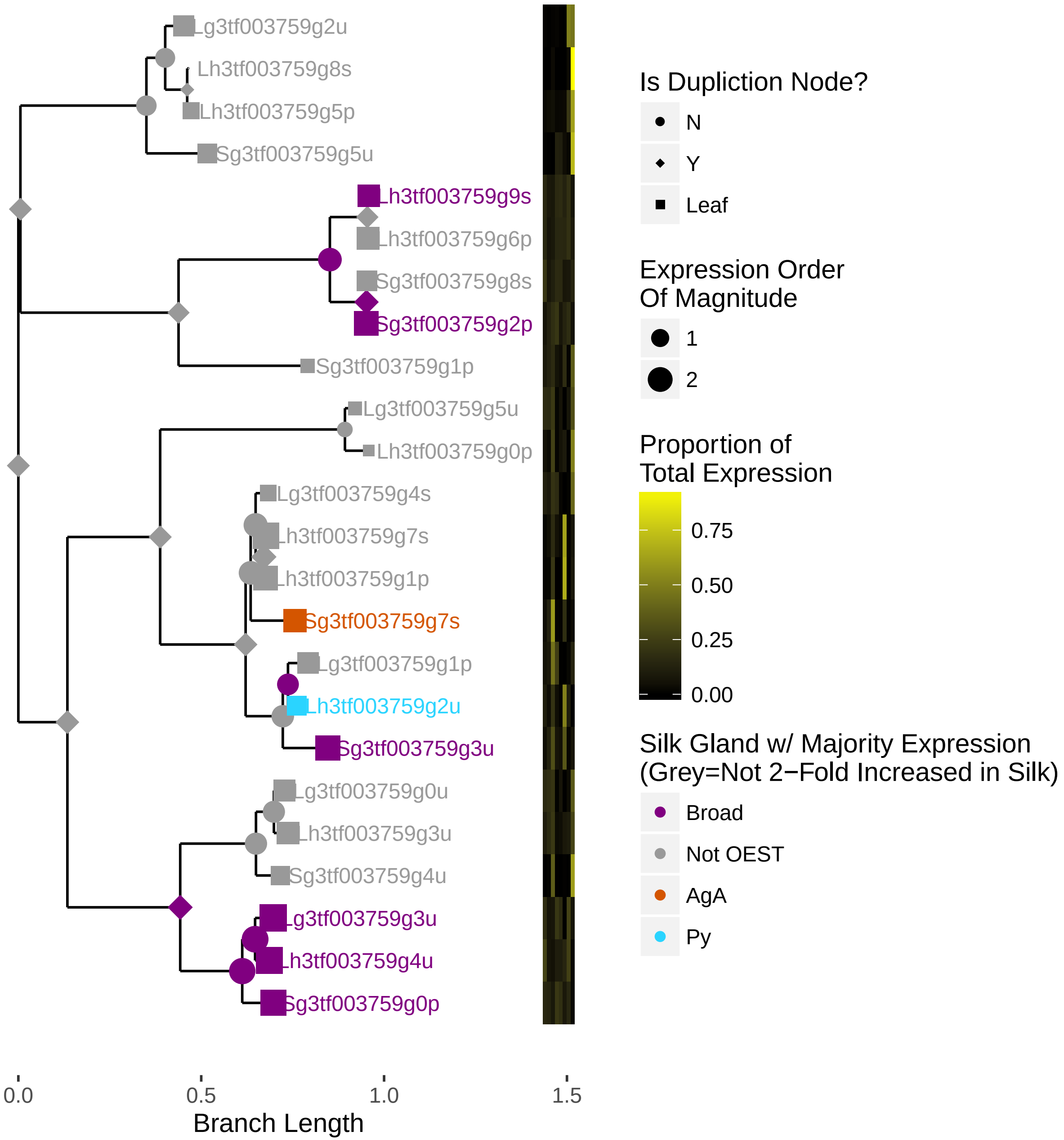

g3tf003759g2u
Lh3tf003759g8s
Lh3tf003759g5p
Sg3tf003759g5u
h3tf003759g9s
h3tf003759g6p
Sg3tf003759g8s
Sg3tf003759g2p
Sg3tf003759g1p
Lg3tf003759g5u
Lh3tf003759g0p
Lg3tf003759g4s
h3tf003759g7s
h3tf003759g1p
Sg3tf003759g7s
g3tf003759g1p
h3tf003759g2u
Sg3tf003759g3u
Lg3tf003759g0u
Lh3tf003759g3u
Sg3tf003759g4u
g3tf003759g3u
h3tf003759g4u
Sg3tf003759g0p
0.0
0.5
1.0
1.5
Branch Length
Is Dupliction Node?
N
Y
Leaf
Expression Order Of Magnitude
1
2
Proportion of Total Expression
0.75
0.50
0.25
0.00
Silk Gland w/ Majority Expression (Grey=Not 2−Fold Increased in Silk)
Broad
Not OEST
AgA
Py

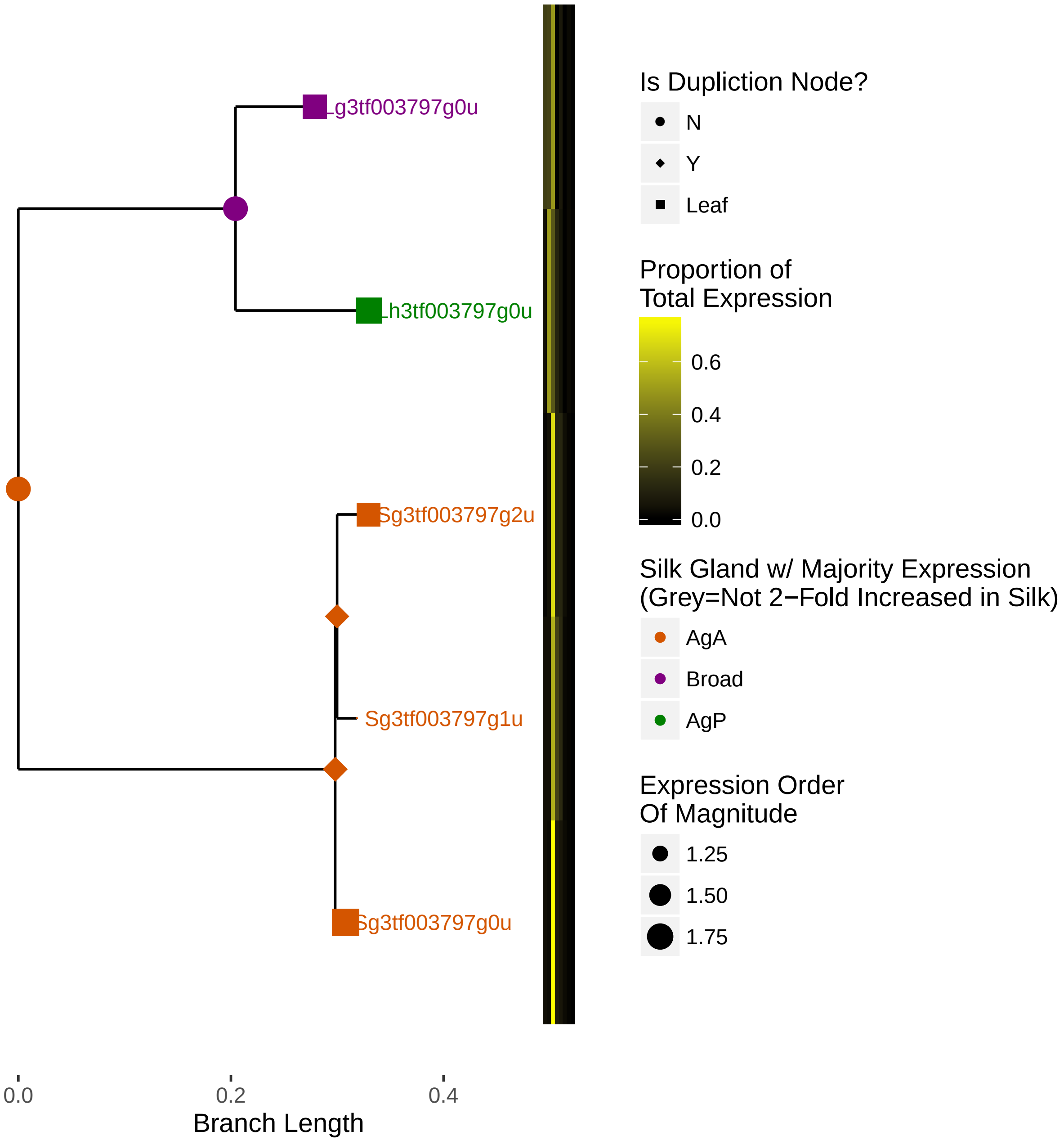

Lg3tf003797g0u
Lh3tf003797g0u
Sg3tf003797g2u
Sg3tf003797g1u
Sg3tf003797g0u
0.0
0.2
0.4
Branch Length
Is Dupliction Node?
N
Y
Leaf
Proportion of
Total Expression
0.6
0.4
0.2
0.0
Silk Gland w/ Majority Expression
(Grey=Not 2−Fold Increased in Silk)
AgA
Broad
AgP
Expression Order
Of Magnitude
1.25
1.50
1.75

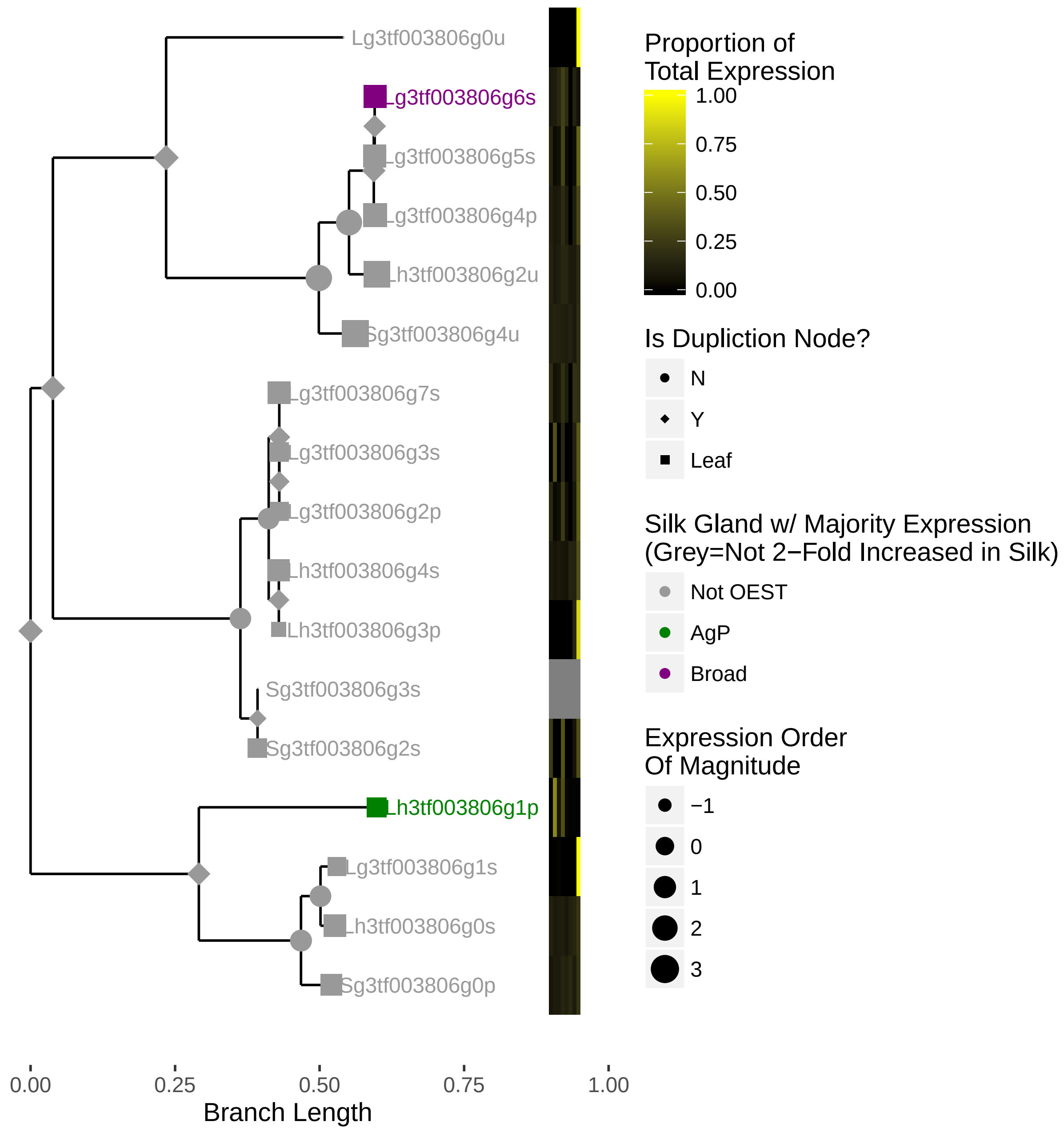

Lg3tf003806g0u
Lg3tf003806g6s
Lg3tf003806g5s
Lg3tf003806g4p
Lh3tf003806g2u
Sg3tf003806g4u
Lg3tf003806g7s
Lg3tf003806g3s
Lg3tf003806g2p
Lh3tf003806g4s
Lh3tf003806g3p
Sg3tf003806g3s
Sg3tf003806g2s
Lh3tf003806g1p
Lg3tf003806g1s
Lh3tf003806g0s
Sg3tf003806g0p
0.00
0.25
0.50
0.75
1.00
Branch Length
Proportion of Total Expression
1.00
0.75
0.50
0.25
0.00
Is Dupliction Node?
N
Y
Leaf
Silk Gland w/ Majority Expression (Grey=Not 2−Fold Increased in Silk)
Not OEST
AgP
Broad
Expression Order Of Magnitude
−1
0
1
2
3

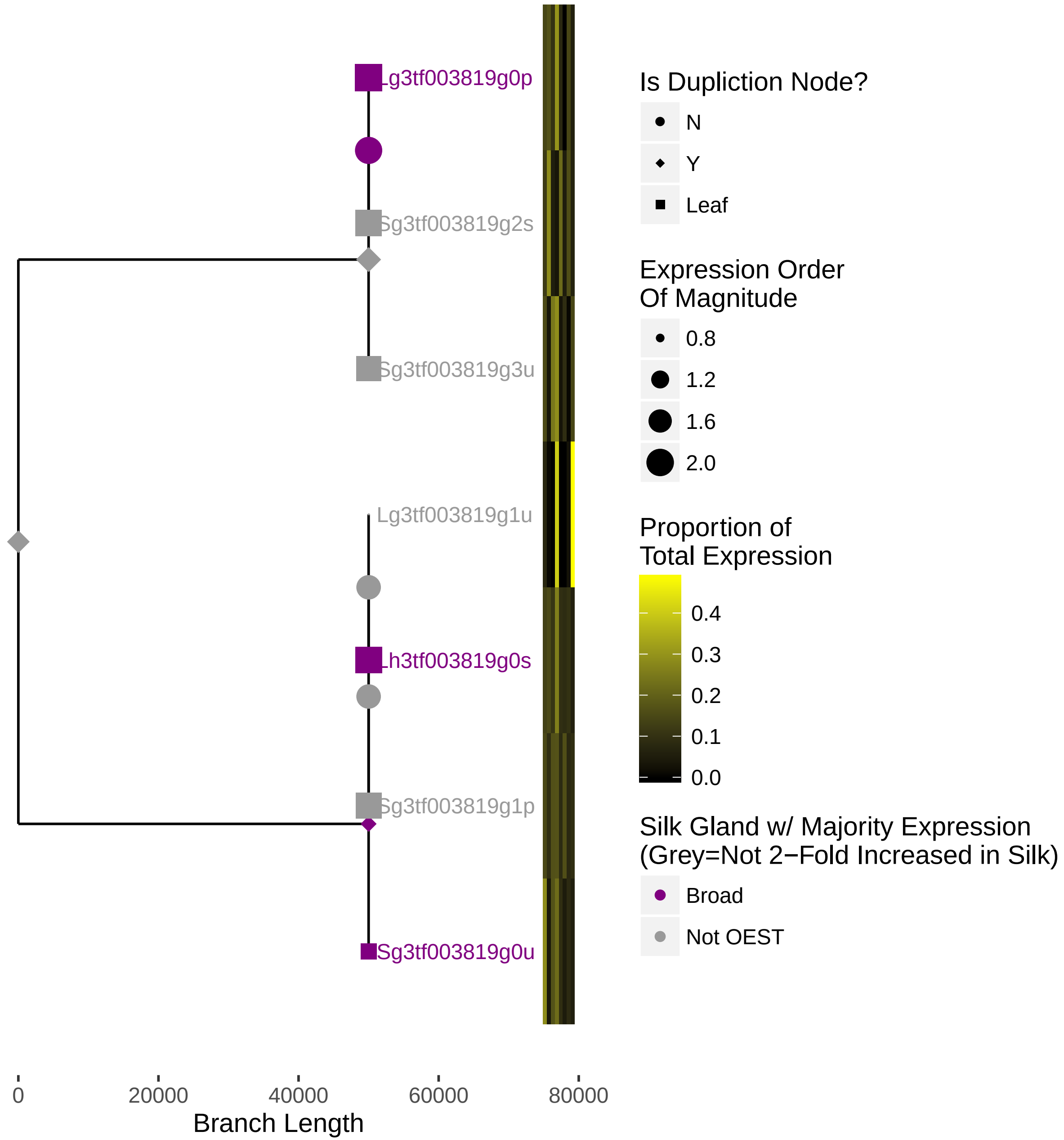

Lg3tf003819g0p
Sg3tf003819g2s
Sg3tf003819g3u
Lg3tf003819g1u
Lh3tf003819g0s
Sg3tf003819g1p
Sg3tf003819g0u
0
20000
40000
60000
80000
Branch Length
Is Dupliction Node?
N
Y
Leaf
Expression Order Of Magnitude
0.8
1.2
1.6
2.0
Proportion of Total Expression
0.4
0.3
0.2
0.1
0.0
Silk Gland w/ Majority Expression (Grey=Not 2−Fold Increased in Silk)
Broad
Not OEST

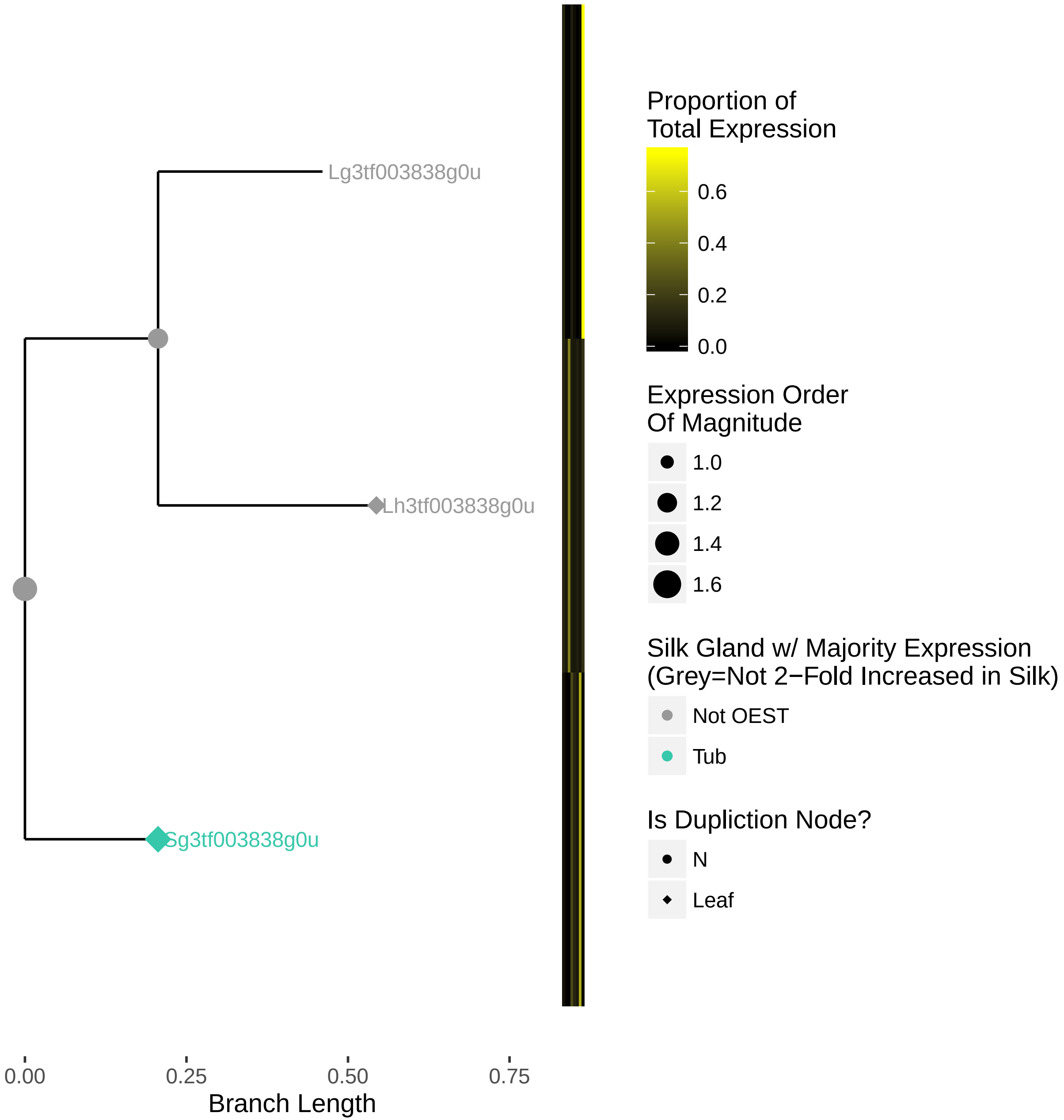

Lg3tf003838g0u
Lh3tf003838g0u
Sg3tf003838g0u
0.00
0.25
0.50
0.75
Branch Length
Proportion of
Total Expression
0.6
0.4
0.2
0.0
Expression Order
Of Magnitude
1.0
1.2
1.4
1.6
Silk Gland w/ Majority Expression
(Grey=Not 2−Fold Increased in Silk)
Not OEST
Tub
Is Dupliction Node?
N
Leaf

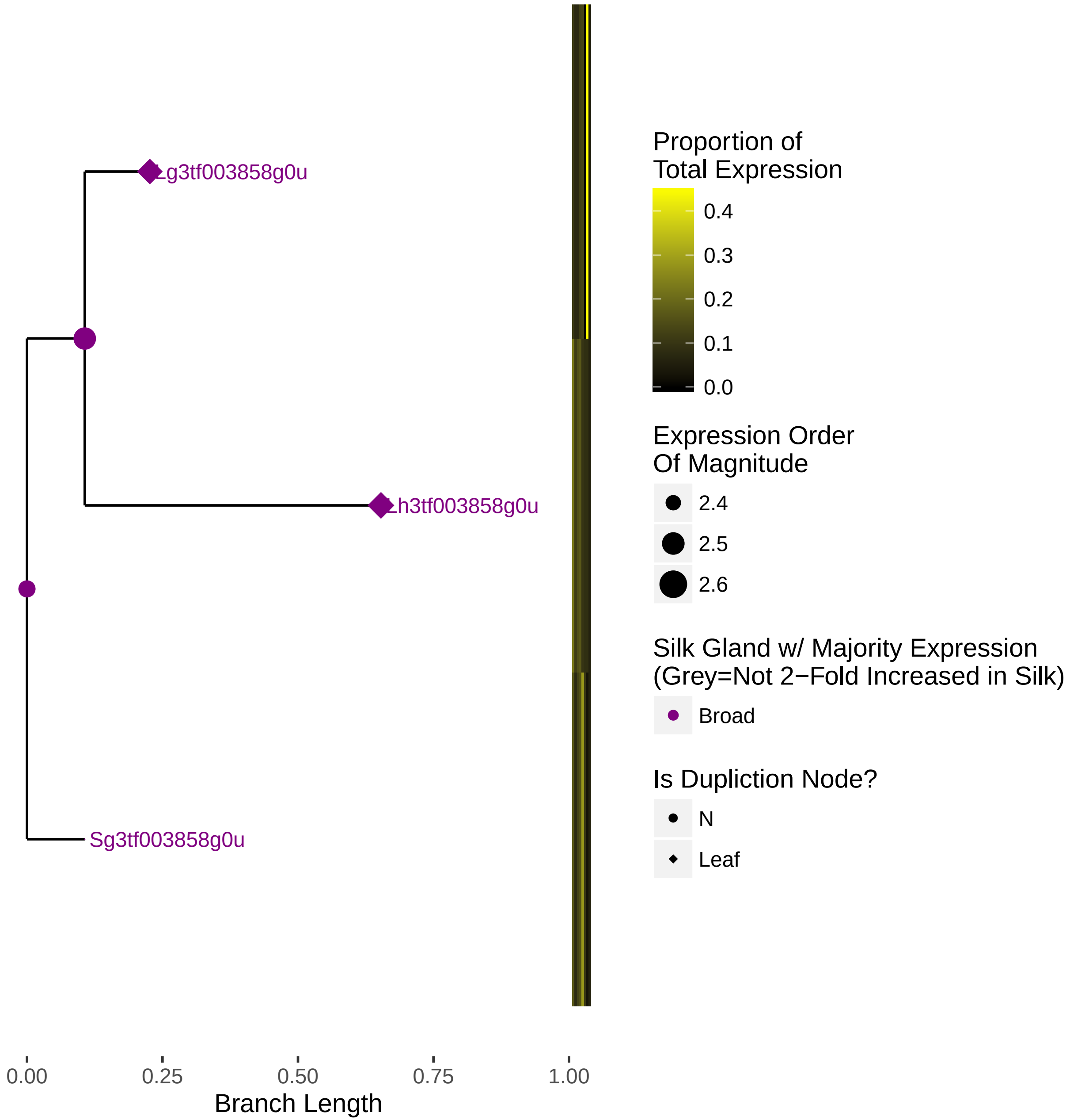

Lg3tf003858g0u
Lh3tf003858g0u
Sg3tf003858g0u
0.00
0.25
0.50
0.75
1.00
Branch Length
Proportion of
Total Expression
0.4
0.3
0.2
0.1
0.0
Expression Order
Of Magnitude
2.4
2.5
2.6
Silk Gland w/ Majority Expression
(Grey=Not 2−Fold Increased in Silk)
Broad
Is Dupliction Node?
N
Leaf

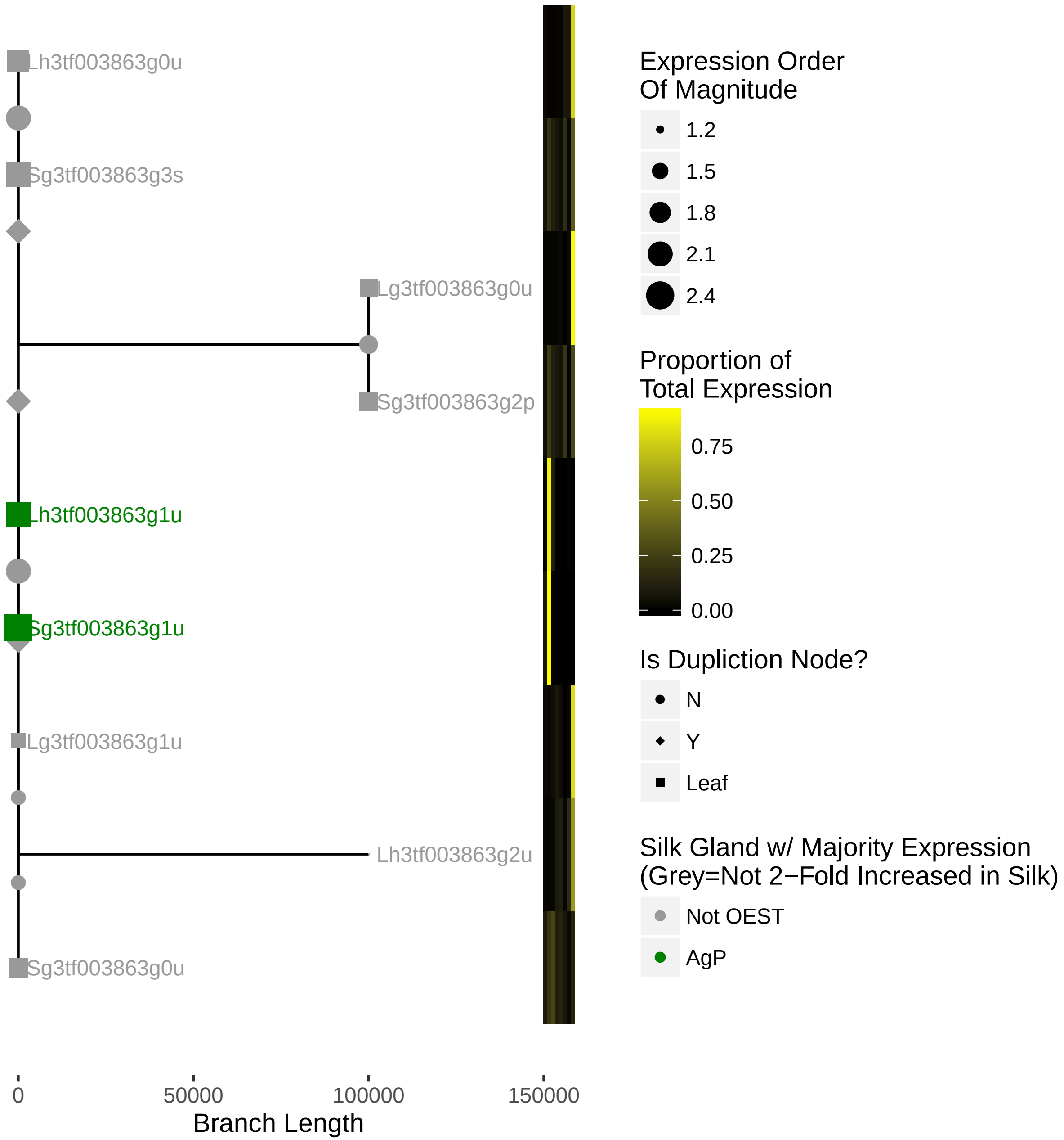
Lh3tf003863g0u
Sg3tf003863g3s
Lg3tf003863g0u
Sg3tf003863g2p
Lh3tf003863g1u
Sg3tf003863g1u
Lg3tf003863g1u
Lh3tf003863g2u
Sg3tf003863g0u
0
50000
100000
150000
Branch Length
Expression Order Of Magnitude
1.2
1.5
1.8
2.1
2.4
Proportion of Total Expression
0.75
0.50
0.25
0.00
Is Dupliction Node?
N
Y
Leaf
Silk Gland w/ Majority Expression (Grey=Not 2−Fold Increased in Silk)
Not OEST
AgP

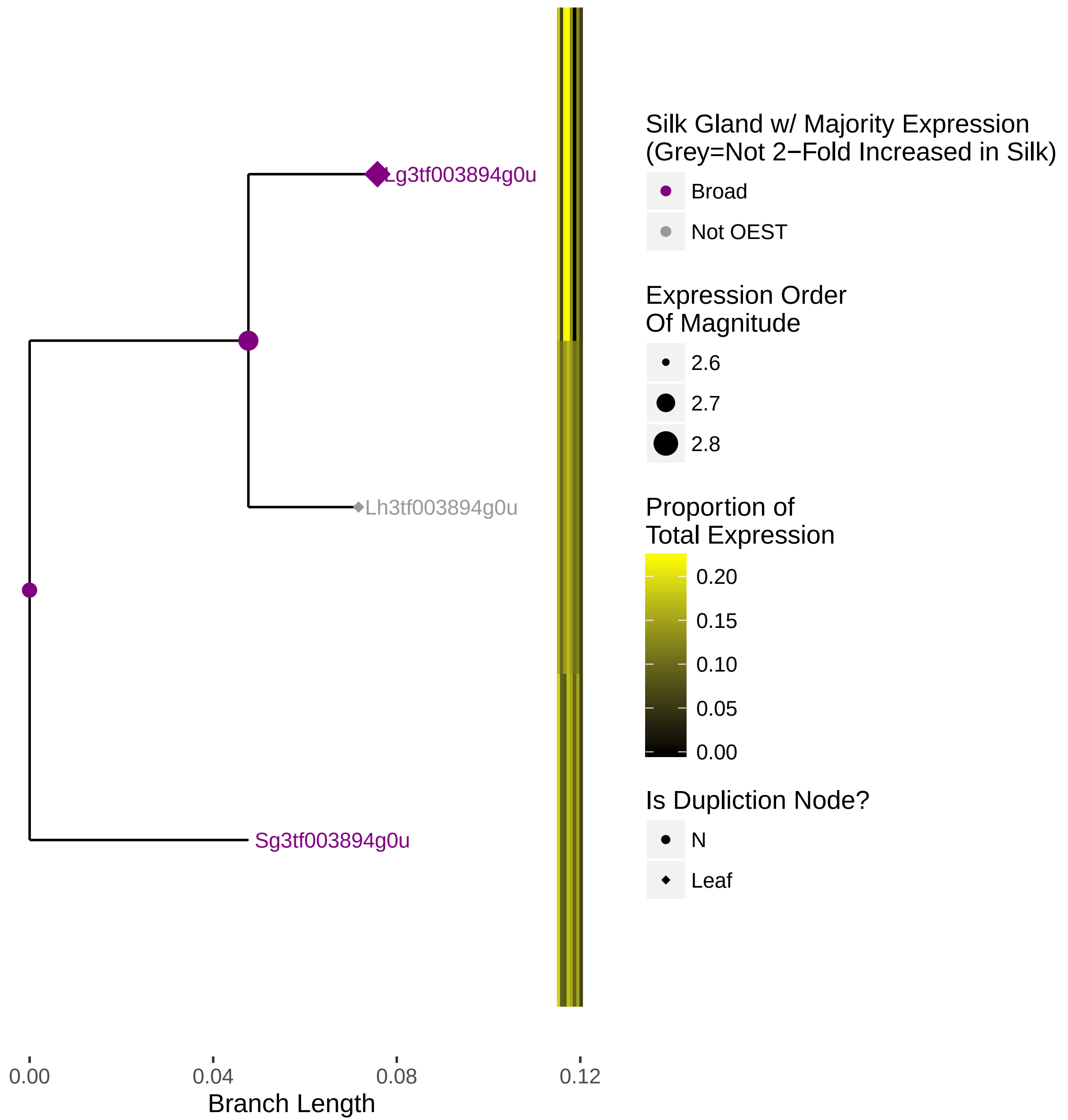

Lg3tf003894g0u
Lh3tf003894g0u
Sg3tf003894g0u
Silk Gland w/ Majority Expression
(Grey=Not 2−Fold Increased in Silk)
Broad
Not OEST
Expression Order
Of Magnitude
2.6
2.7
2.8
Proportion of
Total Expression
0.20
0.15
0.10
0.05
0.00
Is Dupliction Node?
N
Leaf
0.00
0.04
0.08
0.12
Branch Length

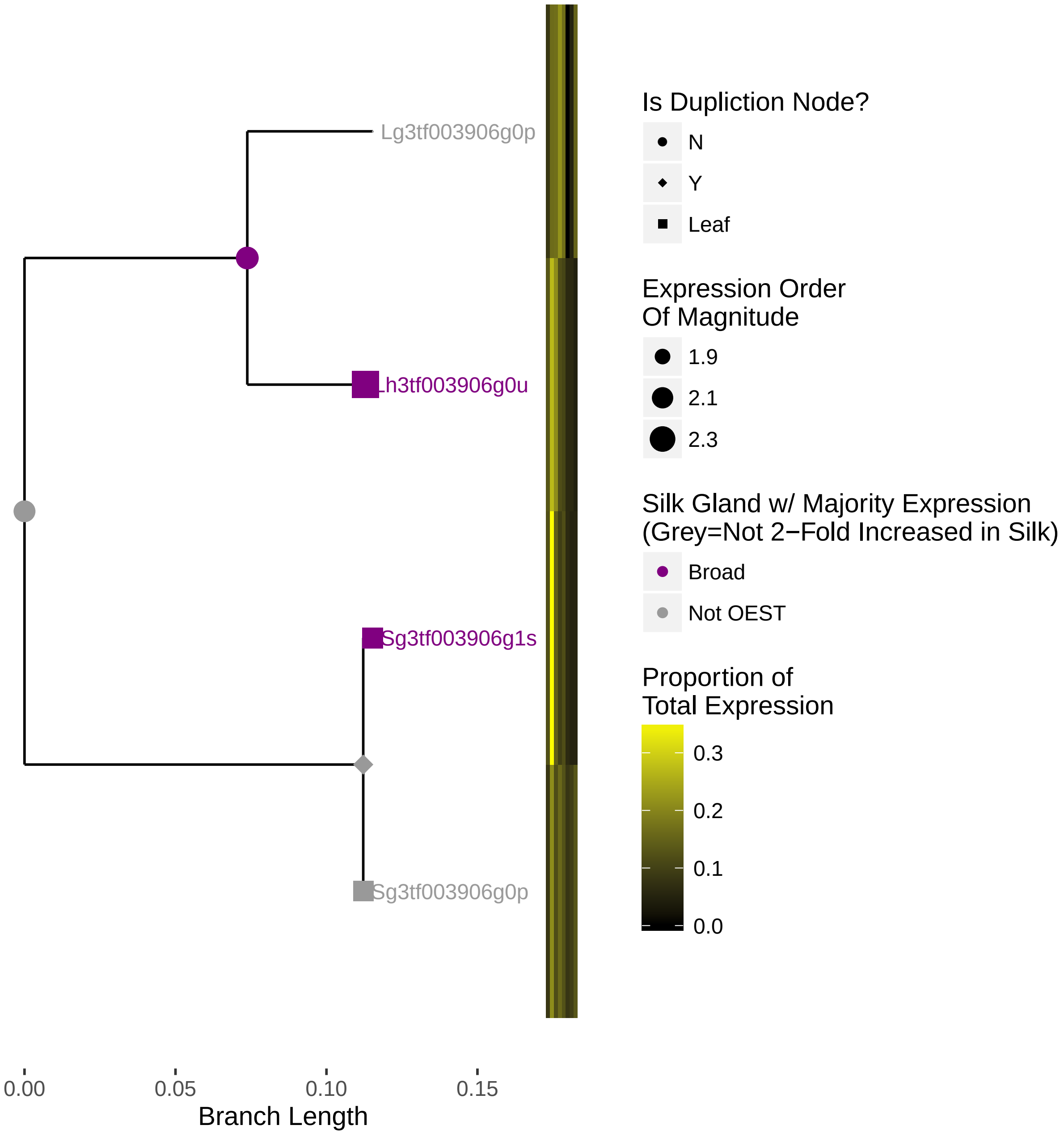

Lg3tf003906g0p
Lh3tf003906g0u
Sg3tf003906g1s
Sg3tf003906g0p
0.00
0.05
0.10
0.15
Branch Length
Is Dupliction Node?
N
Y
Leaf
Expression Order Of Magnitude
1.9
2.1
2.3
Silk Gland w/ Majority Expression (Grey=Not 2−Fold Increased in Silk)
Broad
Not OEST
Proportion of Total Expression
0.3
0.2
0.1
0.0

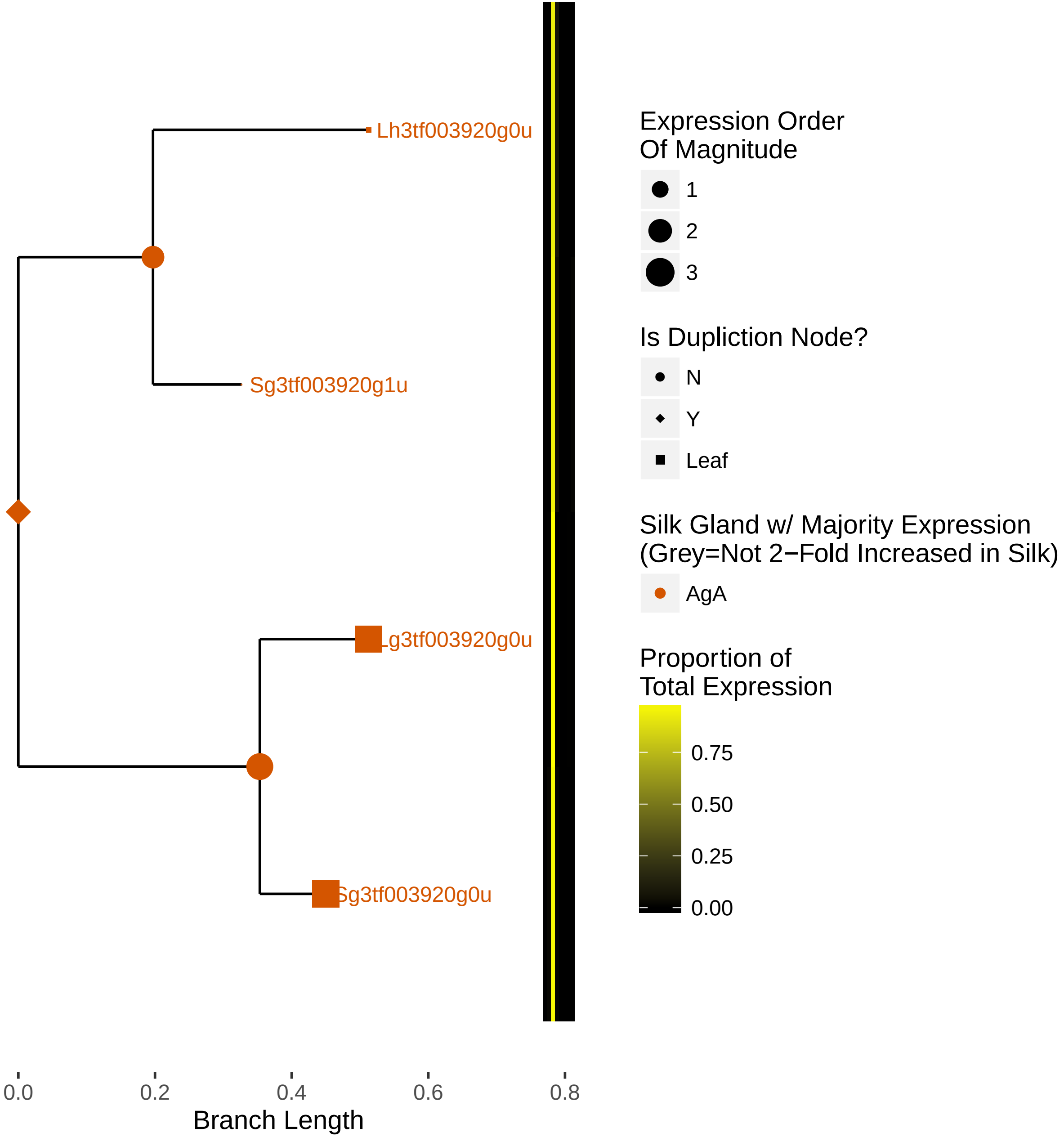

Lh3tf003920g0u
Sg3tf003920g1u
g3tf003920g0u
g3tf003920g0u
0.0
0.2
0.4
0.6
0.8
Branch Length
Expression Order
Of Magnitude
1
2
3
Is Dupliction Node?
N
Y
Leaf
Silk Gland w/ Majority Expression
(Grey=Not 2−Fold Increased in Silk)
AgA
Proportion of
Total Expression
0.75
0.50
0.25
0.00

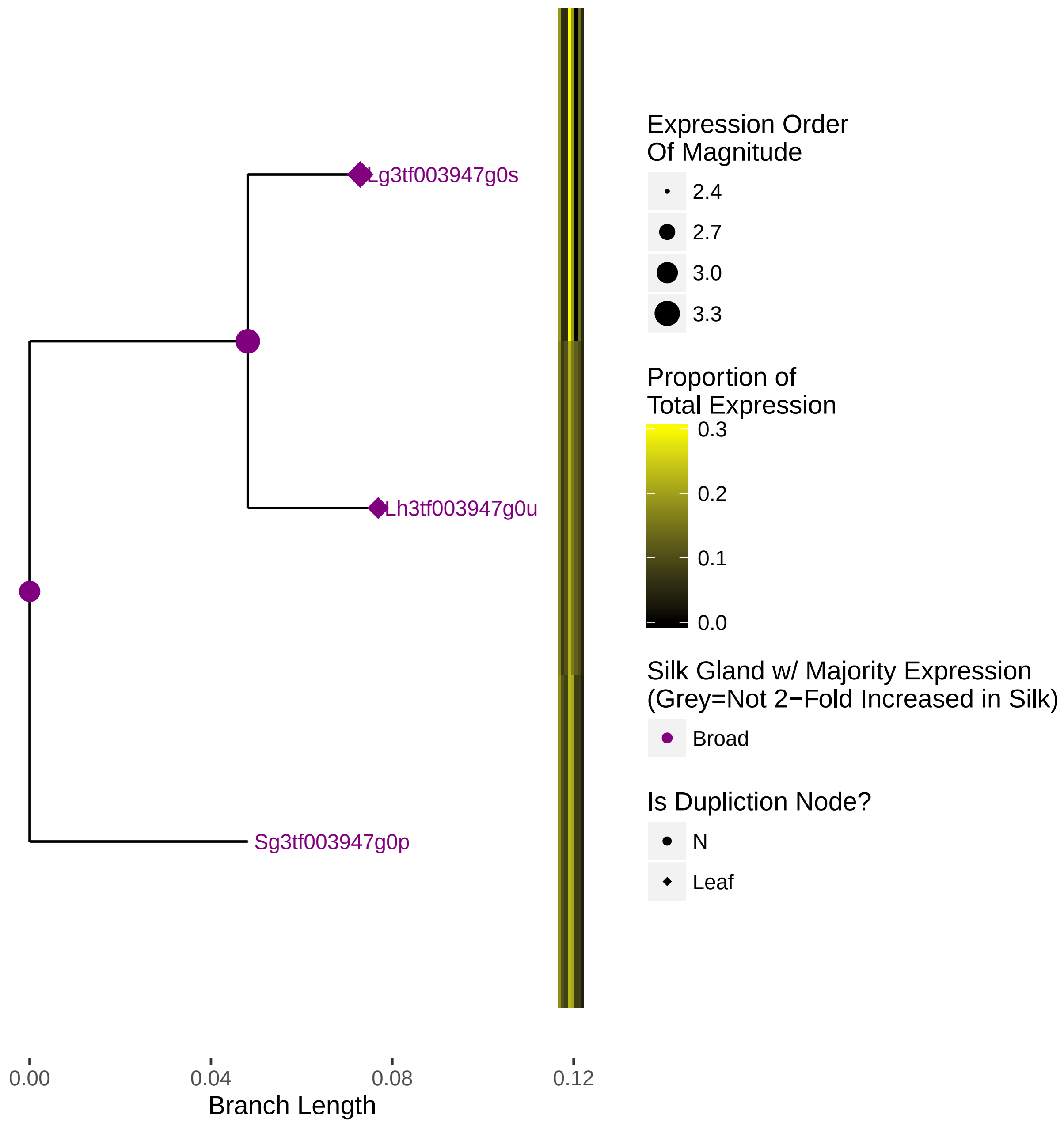
Lg3tf003947g0s
Lh3tf003947g0u
Sg3tf003947g0p
0.00
0.04
0.08
0.12
Branch Length
Expression Order Of Magnitude
2.4
2.7
3.0
3.3
Proportion of Total Expression
0.3
0.2
0.1
0.0
Silk Gland w/ Majority Expression (Grey=Not 2−Fold Increased in Silk)
Broad
Is Dupliction Node?
N
Leaf

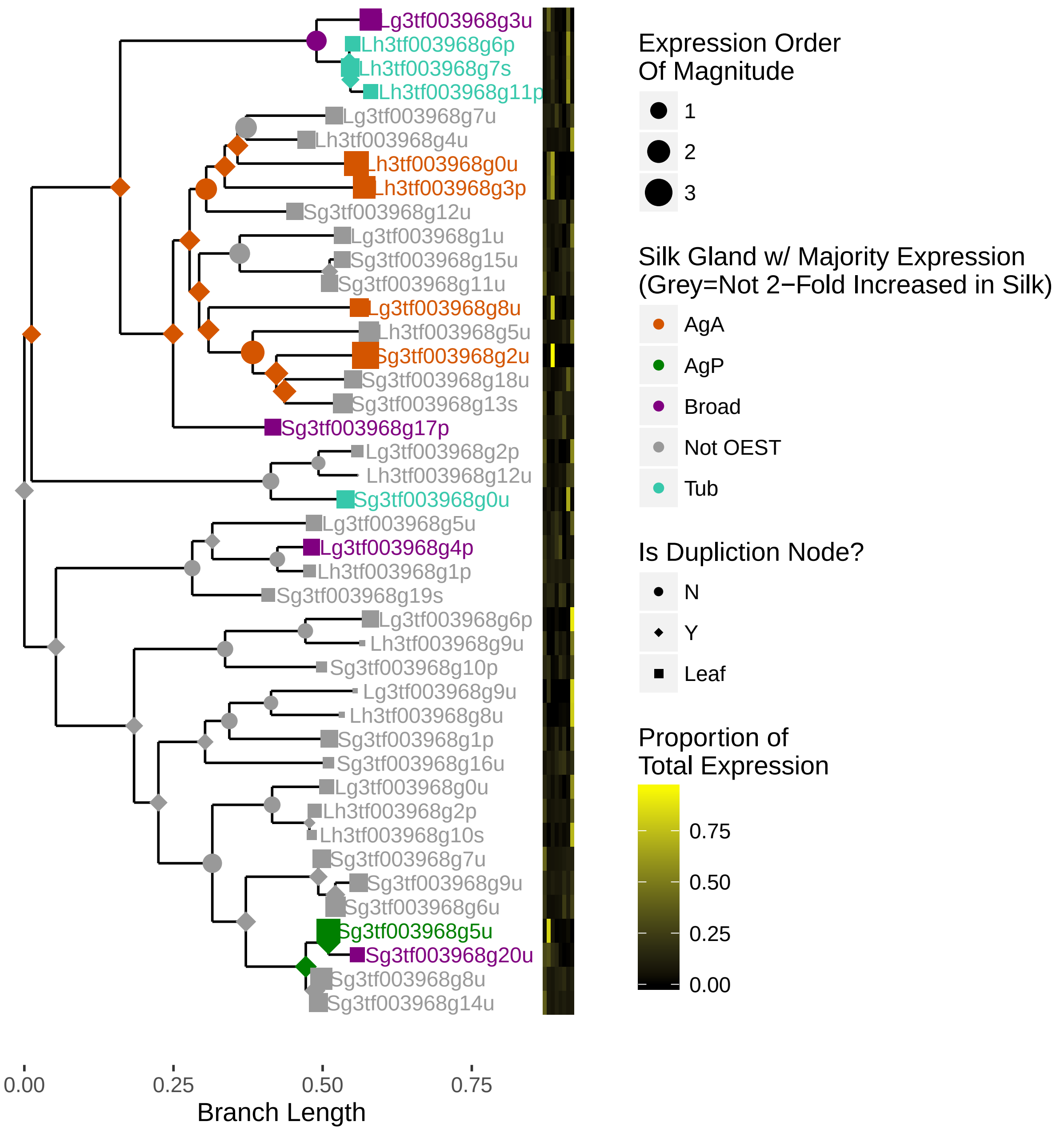

Lg3tf003968g3u
Lh3tf003968g6p
Lh3tf003968g7s
Lh3tf003968g11p
Lg3tf003968g7u
Lh3tf003968g4u
Lh3tf003968g0u
Lh3tf003968g3p
Sg3tf003968g12u
Lg3tf003968g1u
Sg3tf003968g15u
Sg3tf003968g11u
Lg3tf003968g8u
Lh3tf003968g5u
Sg3tf003968g2u
Sg3tf003968g18u
Sg3tf003968g13s
Sg3tf003968g17p
Lg3tf003968g2p
Lh3tf003968g12u
Sg3tf003968g0u
Lg3tf003968g5u
Lg3tf003968g4p
Lh3tf003968g1p
Sg3tf003968g19s
Lg3tf003968g6p
Lh3tf003968g9u
Sg3tf003968g10p
Lg3tf003968g9u
Lh3tf003968g8u
Sg3tf003968g1p
Sg3tf003968g16u
Lg3tf003968g0u
Lh3tf003968g2p
Lh3tf003968g10s
Sg3tf003968g7u
Sg3tf003968g9u
Sg3tf003968g6u
Sg3tf003968g5u
Sg3tf003968g20u
Sg3tf003968g8u
Sg3tf003968g14u
0.00
0.25
0.50
0.75
Branch Length
Expression Order Of Magnitude
1
2
3
Silk Gland w/ Majority Expression (Grey=Not 2−Fold Increased in Silk)
AgA
AgP
Broad
Not OEST
Tub
Is Dupliction Node?
N
Y
Leaf
Proportion of Total Expression
0.75
0.50
0.25
0.00

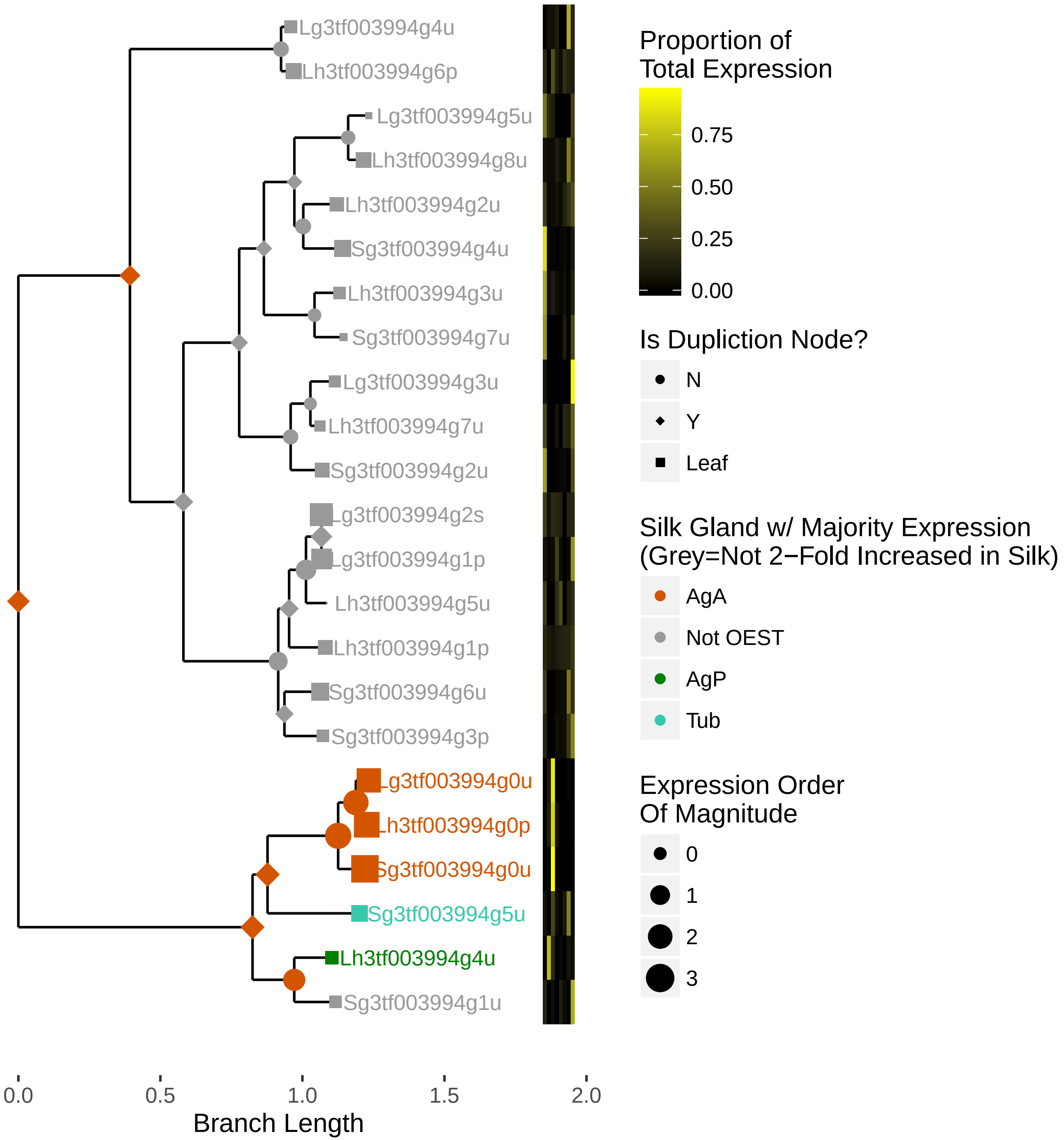

Lg3tf003994g4u
Lh3tf003994g6p
Lg3tf003994g5u
Lh3tf003994g8u
Lh3tf003994g2u
Sg3tf003994g4u
Lh3tf003994g3u
Sg3tf003994g7u
Lg3tf003994g3u
Lh3tf003994g7u
Sg3tf003994g2u
g3tf003994g2s
g3tf003994g1p
Lh3tf003994g5u
Lh3tf003994g1p
Sg3tf003994g6u
Sg3tf003994g3p
g3tf003994g0u
h3tf003994g0p
g3tf003994g0u
Sg3tf003994g5u
Lh3tf003994g4u
Sg3tf003994g1u
0.0
0.5
1.0
1.5
2.0
Branch Length
Proportion of
Total Expression
0.75
0.50
0.25
0.00
Is Dupliction Node?
N
Y
Leaf
Silk Gland w/ Majority Expression
(Grey=Not 2−Fold Increased in Silk)
AgA
Not OEST
AgP
Tub
Expression Order
Of Magnitude
0
1
2
3

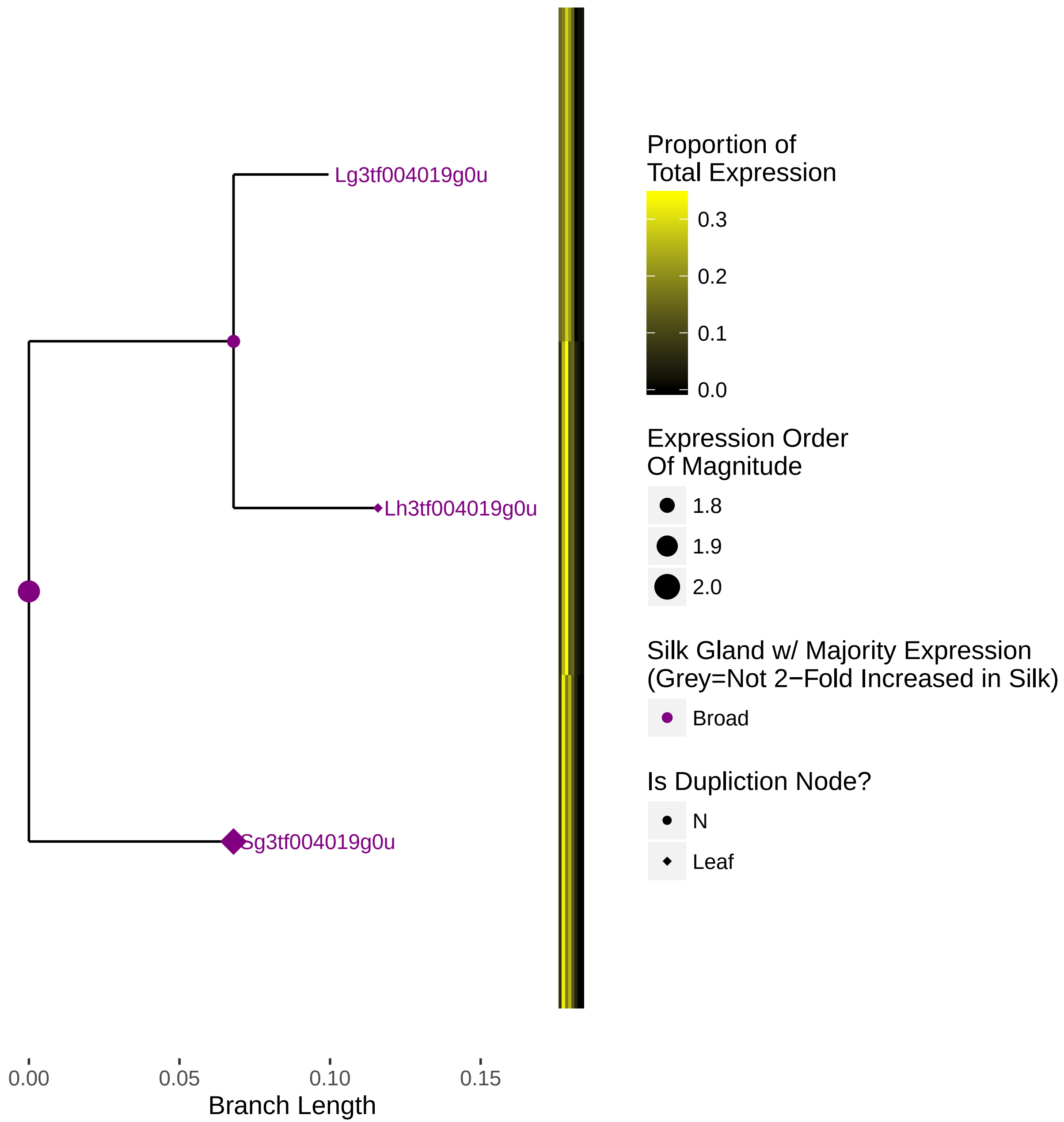

Lg3tf004019g0u
Lh3tf004019g0u
Sg3tf004019g0u
0.00
0.05
0.10
0.15
Branch Length
Proportion of Total Expression
0.3
0.2
0.1
0.0
Expression Order Of Magnitude
1.8
1.9
2.0
Silk Gland w/ Majority Expression (Grey=Not 2−Fold Increased in Silk)
Broad
Is Dupliction Node?
N
Leaf

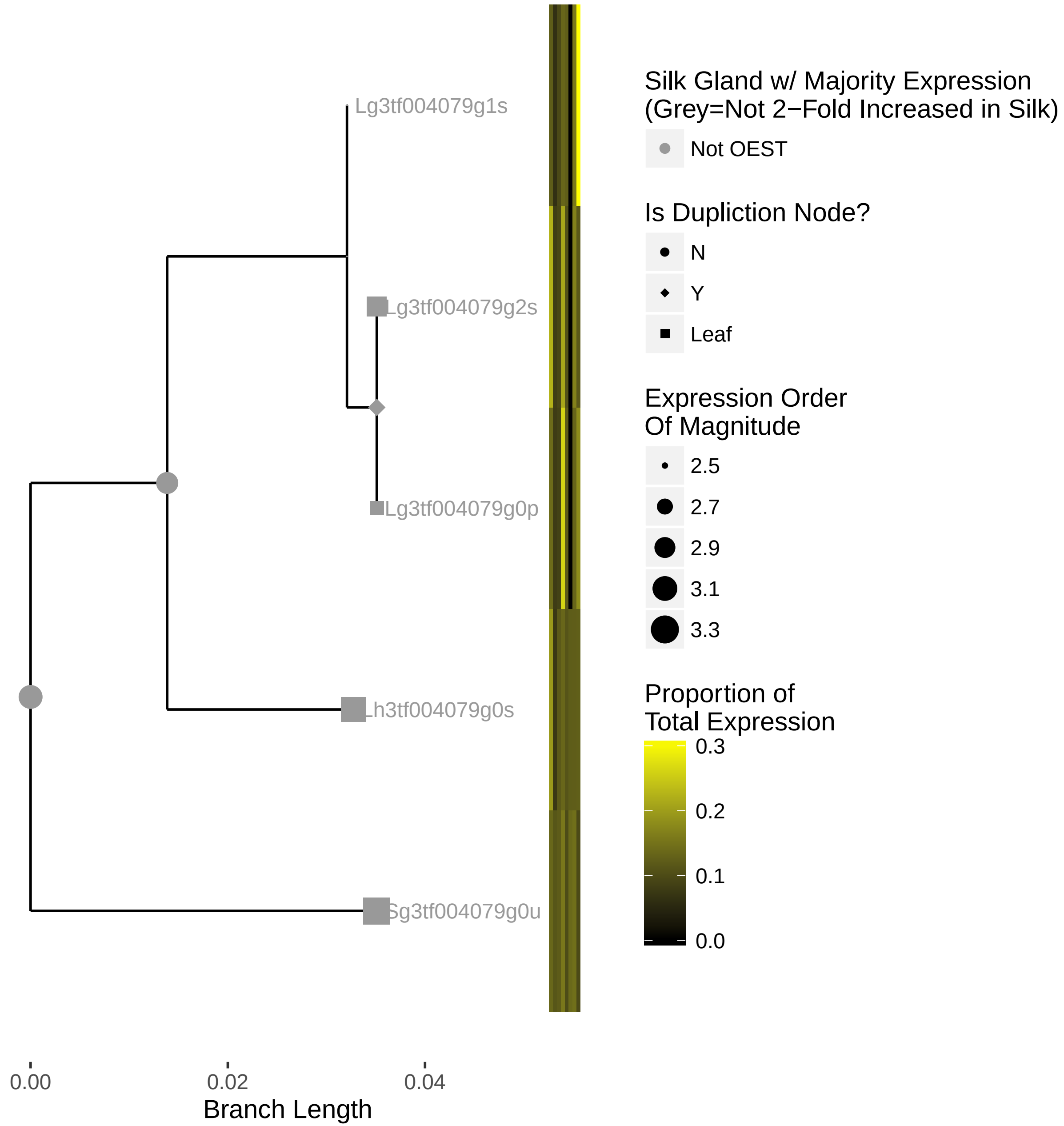
Lg3tf004079g1s
Lg3tf004079g2s
Lg3tf004079g0p
Lh3tf004079g0s
Sg3tf004079g0u
0.00
0.02
0.04
Branch Length
Silk Gland w/ Majority Expression
(Grey=Not 2−Fold Increased in Silk)
Not OEST
Is Dupliction Node?
N
Y
Leaf
Expression Order
Of Magnitude
2.5
2.7
2.9
3.1
3.3
Proportion of
Total Expression
0.3
0.2
0.1
0.0

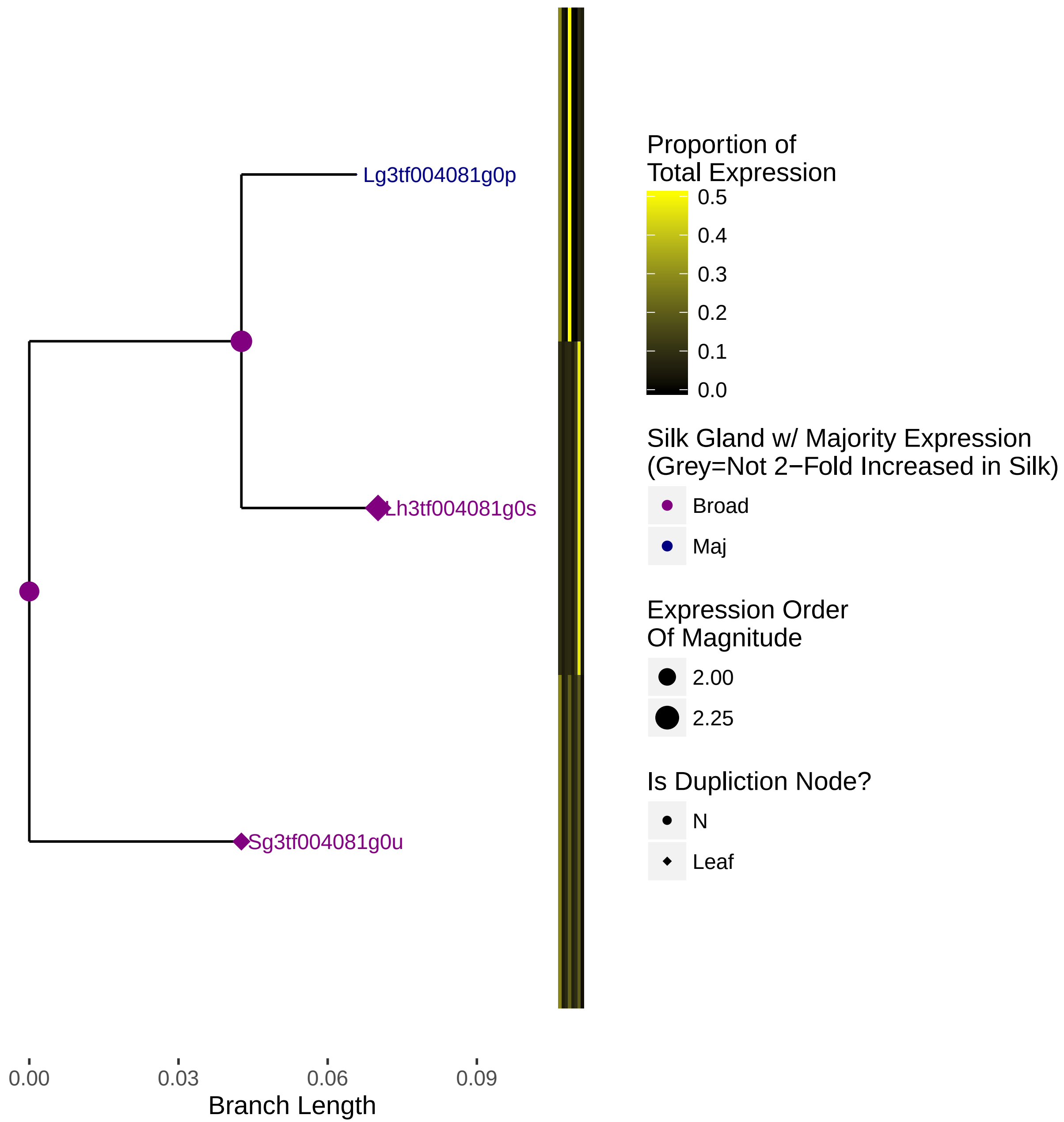

Lg3tf004081g0p
Lh3tf004081g0s
Sg3tf004081g0u
Proportion of
Total Expression
0.5
0.4
0.3
0.2
0.1
0.0
Silk Gland w/ Majority Expression
(Grey=Not 2−Fold Increased in Silk)
Broad
Maj
Expression Order
Of Magnitude
2.00
2.25
Is Dupliction Node?
N
Leaf
0.00
0.03
0.06
0.09
Branch Length

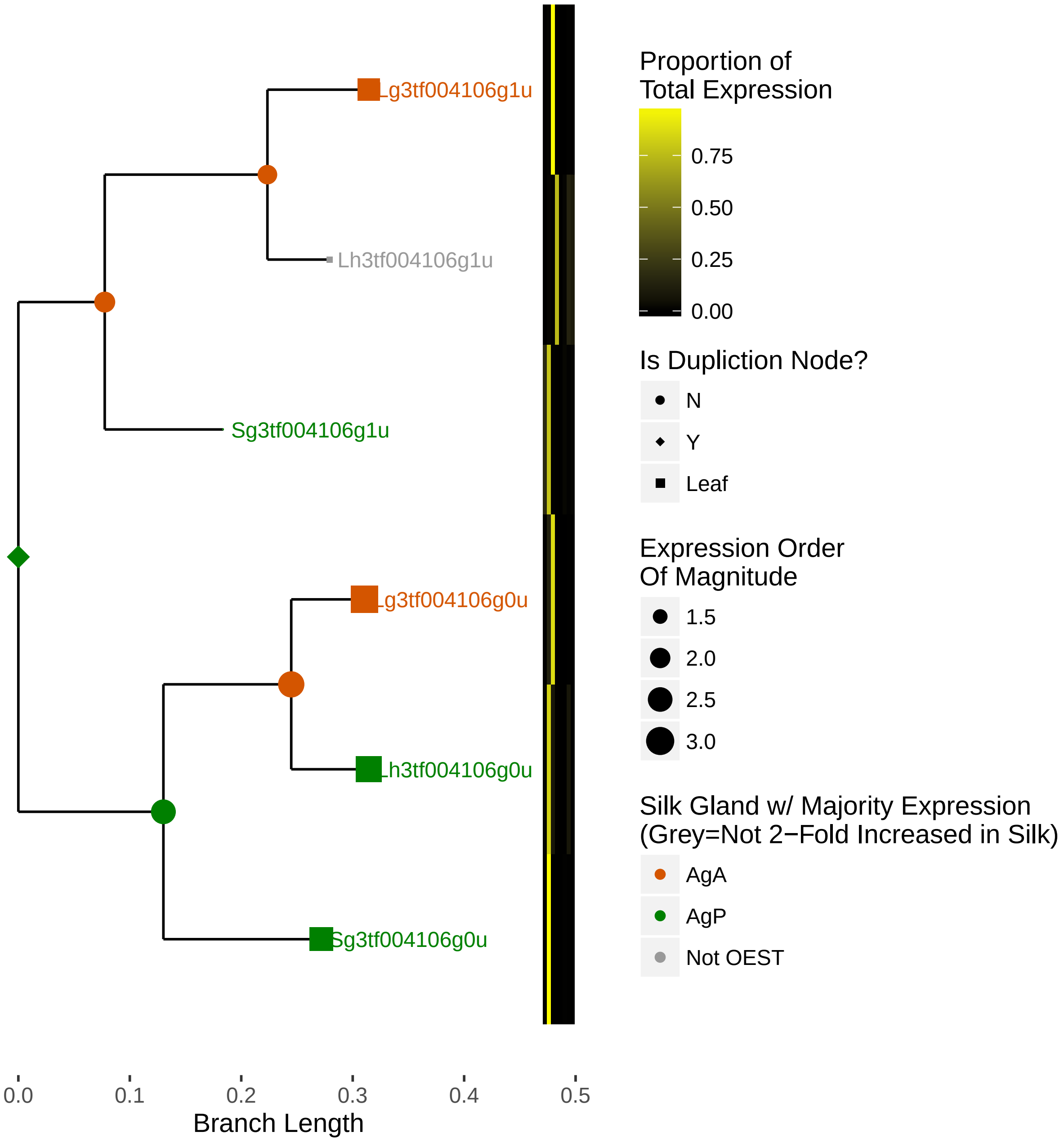

Lg3tf004106g1u
Lh3tf004106g1u
Sg3tf004106g1u
Lg3tf004106g0u
Lh3tf004106g0u
Sg3tf004106g0u
0.0
0.1
0.2
0.3
0.4
0.5
Branch Length
Proportion of
Total Expression
0.75
0.50
0.25
0.00
Is Dupliction Node?
N
Y
Leaf
Expression Order
Of Magnitude
1.5
2.0
2.5
3.0
Silk Gland w/ Majority Expression
(Grey=Not 2−Fold Increased in Silk)
AgA
AgP
Not OEST

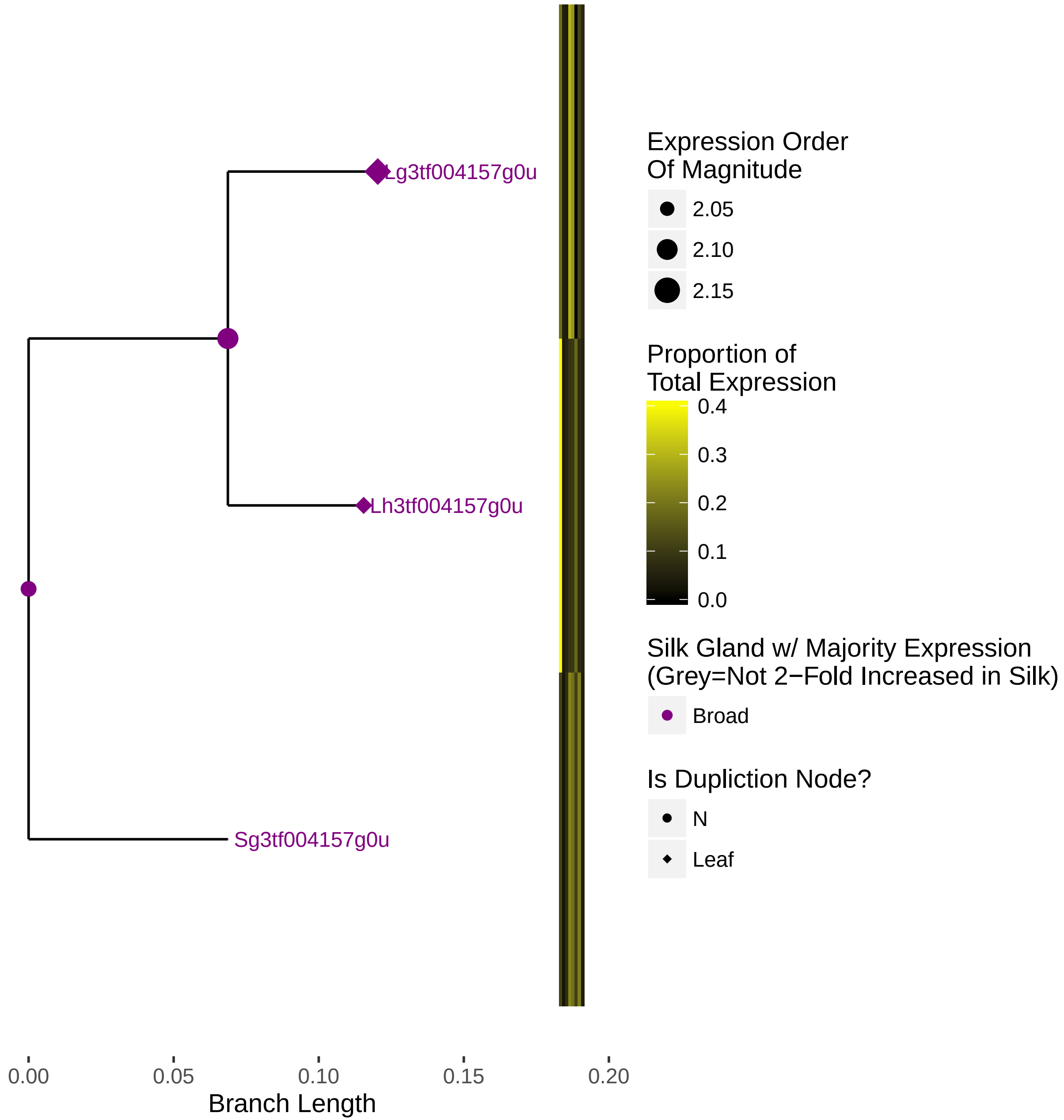

Lg3tf004157g0u
Lh3tf004157g0u
Sg3tf004157g0u
0.00
0.05
0.10
0.15
0.20
Branch Length
Expression Order
Of Magnitude
2.05
2.10
2.15
Proportion of
Total Expression
0.4
0.3
0.2
0.1
0.0
Silk Gland w/ Majority Expression
(Grey=Not 2−Fold Increased in Silk)
Broad
Is Dupliction Node?
N
Leaf

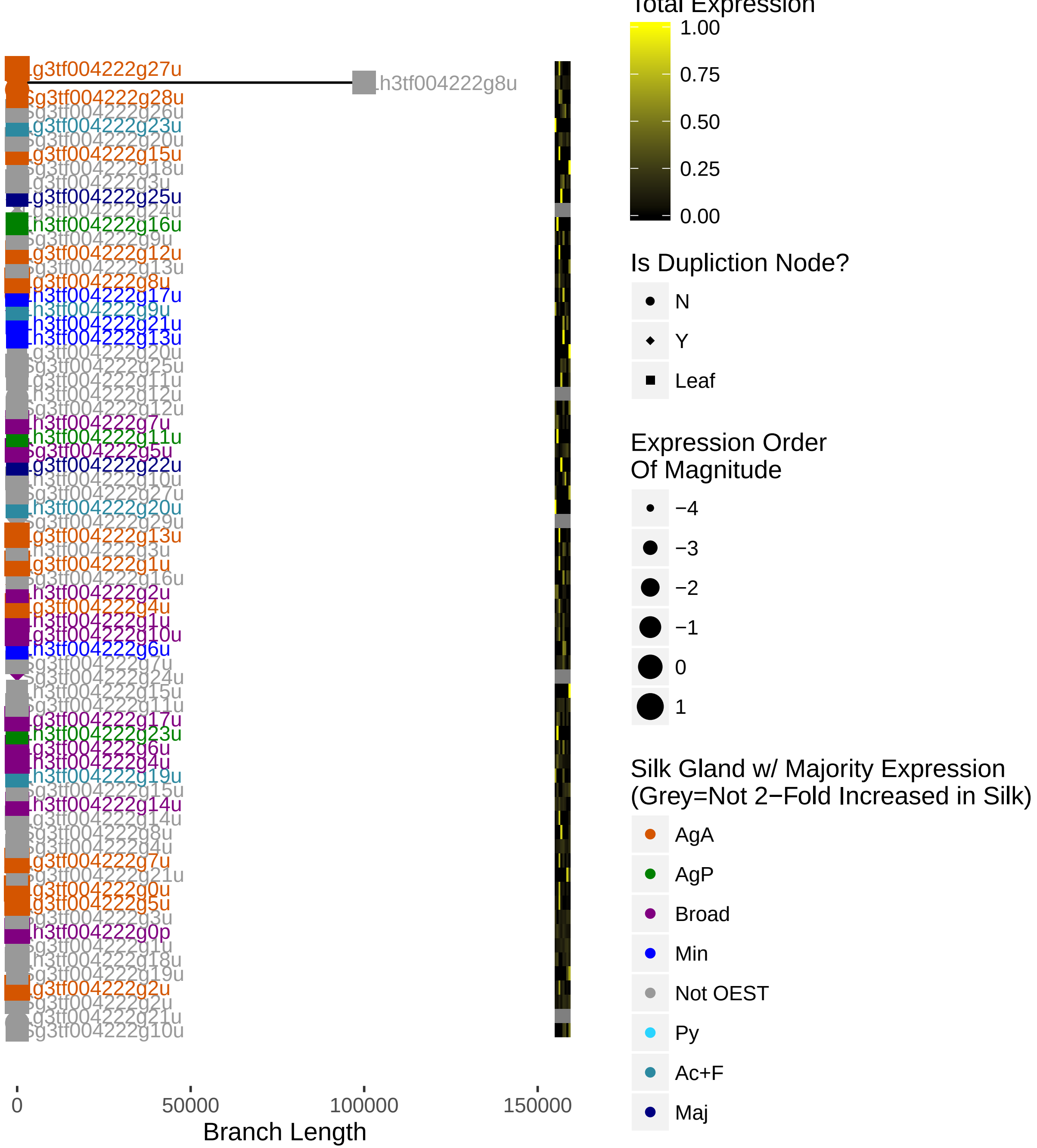

h3tf004222g8u
0
50000
100000
150000
Branch Length
Total Expression
1.00
0.75
0.50
0.25
0.00
Is Dupliction Node?
N
Y
Leaf
Expression Order
Of Magnitude
−4
−3
−2
−1
0
1
Silk Gland w/ Majority Expression
(Grey=Not 2−Fold Increased in Silk)
AgA
AgP
Broad
Min
Not OEST
Py
Ac+F
Maj

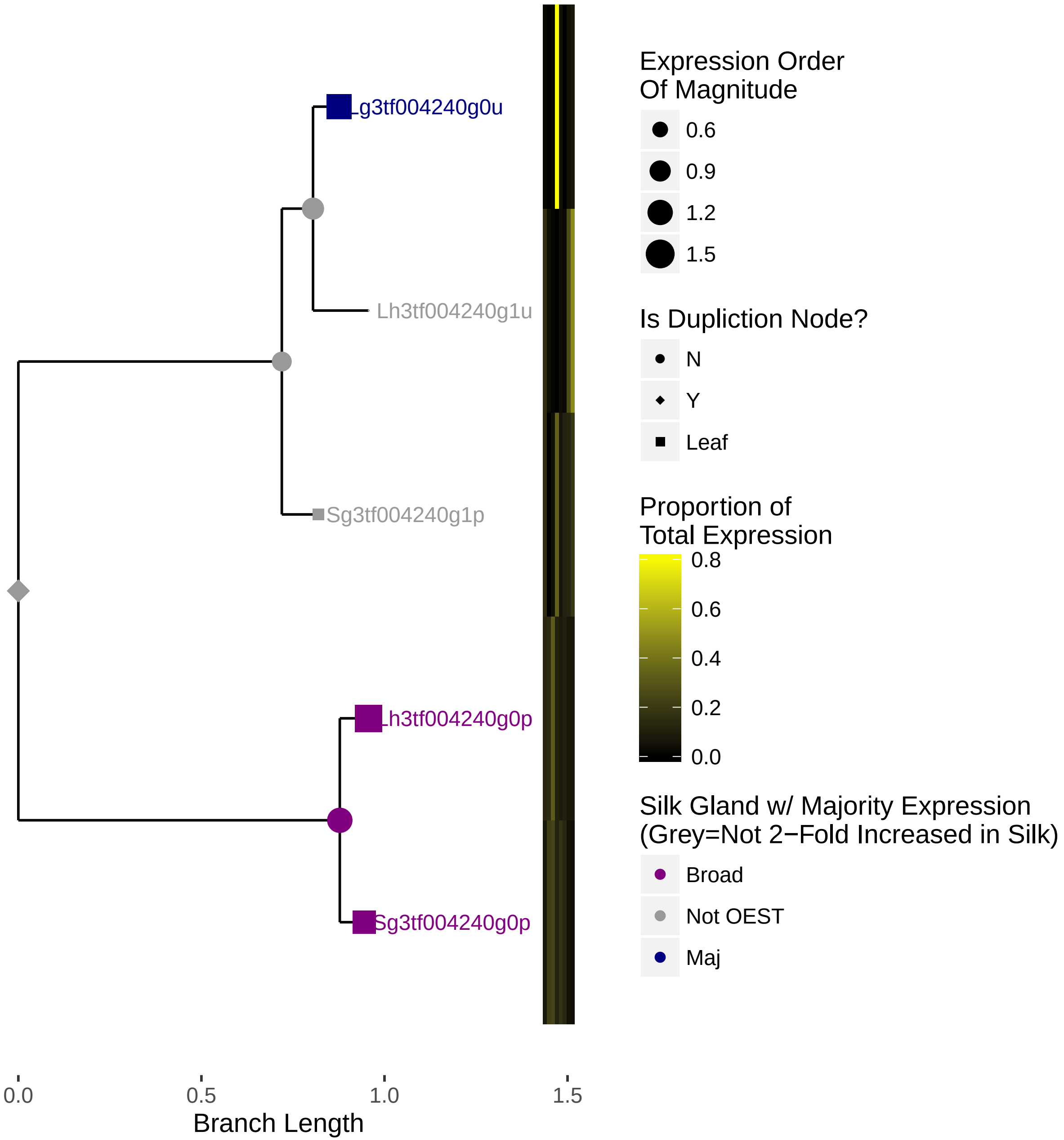

Lg3tf004240g0u
Lh3tf004240g1u
Sg3tf004240g1p
Lh3tf004240g0p
Sg3tf004240g0p
0.0
0.5
1.0
1.5
Branch Length
Expression Order Of Magnitude
0.6
0.9
1.2
1.5
Is Dupliction Node?
N
Y
Leaf
Proportion of Total Expression
0.8
0.6
0.4
0.2
0.0
Silk Gland w/ Majority Expression (Grey=Not 2−Fold Increased in Silk)
Broad
Not OEST
Maj

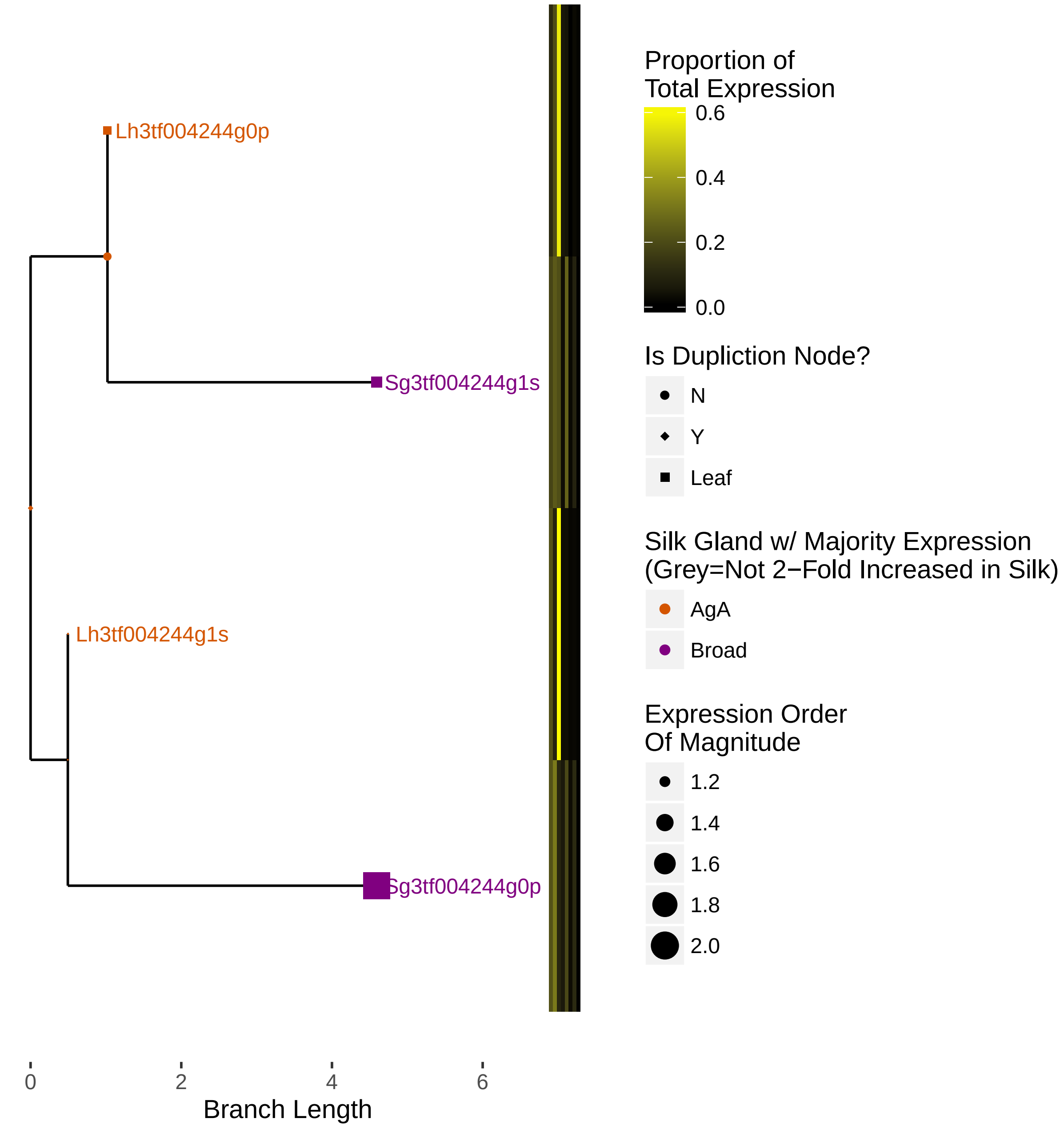

Lh3tf004244g0p
Sg3tf004244g1s
Lh3tf004244g1s
Sg3tf004244g0p
0
2
4
6
Branch Length
Proportion of
Total Expression
0.6
0.4
0.2
0.0
Is Dupliction Node?
N
Y
Leaf
Silk Gland w/ Majority Expression
(Grey=Not 2−Fold Increased in Silk)
AgA
Broad
Expression Order
Of Magnitude
1.2
1.4
1.6
1.8
2.0

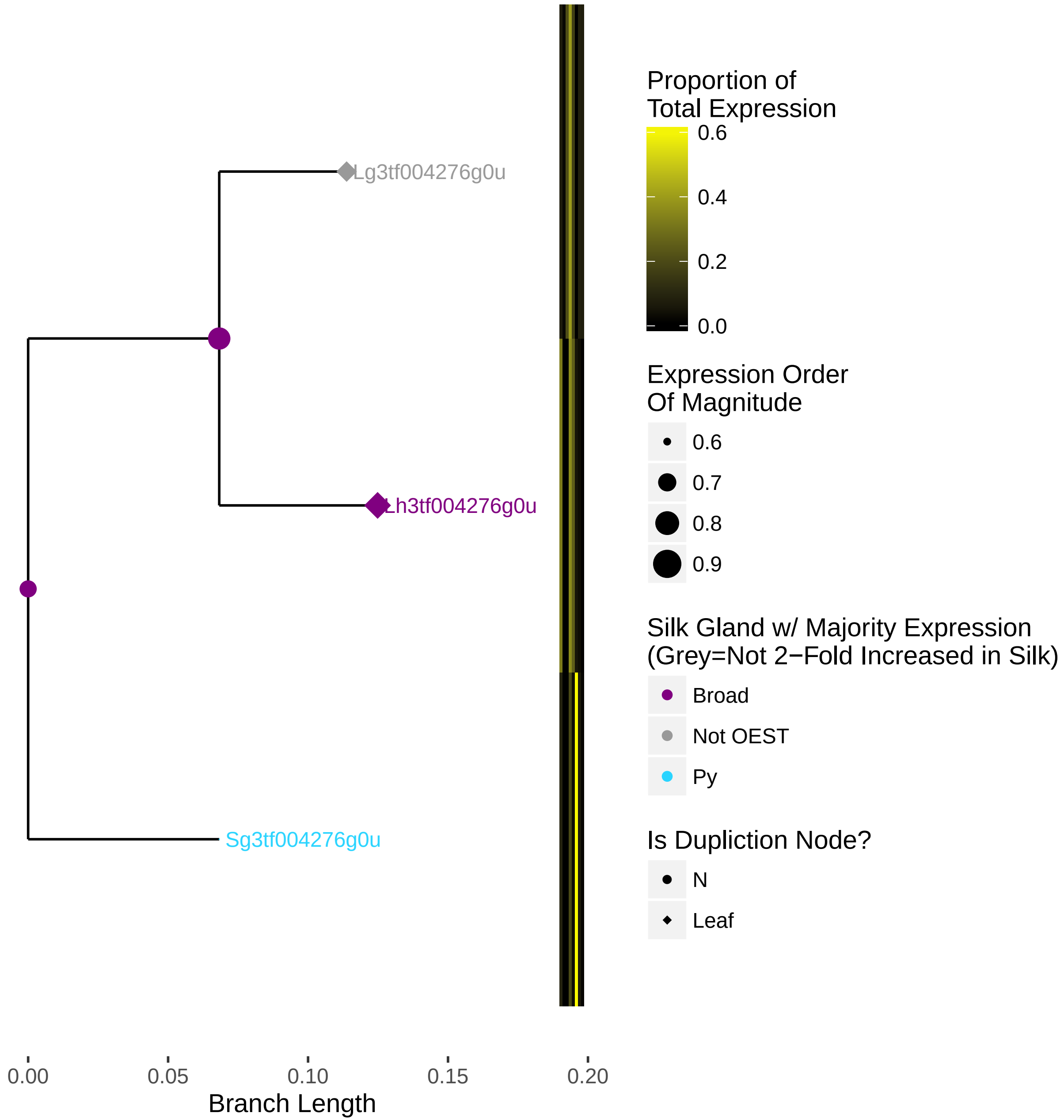

Lg3tf004276g0u
Lh3tf004276g0u
Sg3tf004276g0u
0.00
0.05
0.10
0.15
0.20
Branch Length
Proportion of Total Expression
0.6
0.4
0.2
0.0
Expression Order Of Magnitude
0.6
0.7
0.8
0.9
Silk Gland w/ Majority Expression (Grey=Not 2−Fold Increased in Silk)
Broad
Not OEST
Py
Is Dupliction Node?
N
Leaf

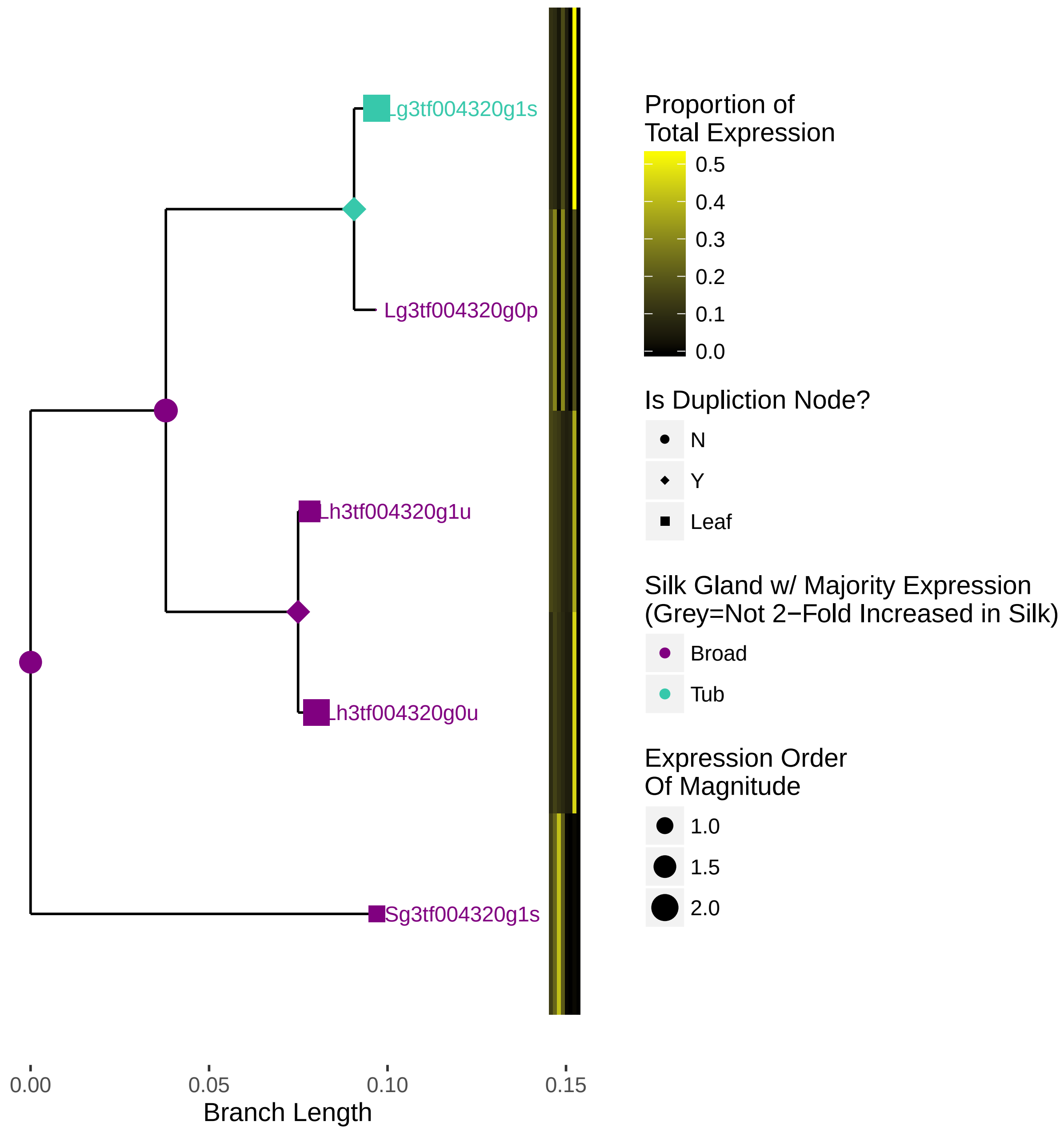

Lg3tf004320g1s
Lg3tf004320g0p
Lh3tf004320g1u
Lh3tf004320g0u
Sg3tf004320g1s
0.00
0.05
0.10
0.15
Branch Length
Proportion of
Total Expression
0.5
0.4
0.3
0.2
0.1
0.0
Is Dupliction Node?
N
Y
Leaf
Silk Gland w/ Majority Expression
(Grey=Not 2−Fold Increased in Silk)
Broad
Tub
Expression Order
Of Magnitude
1.0
1.5
2.0

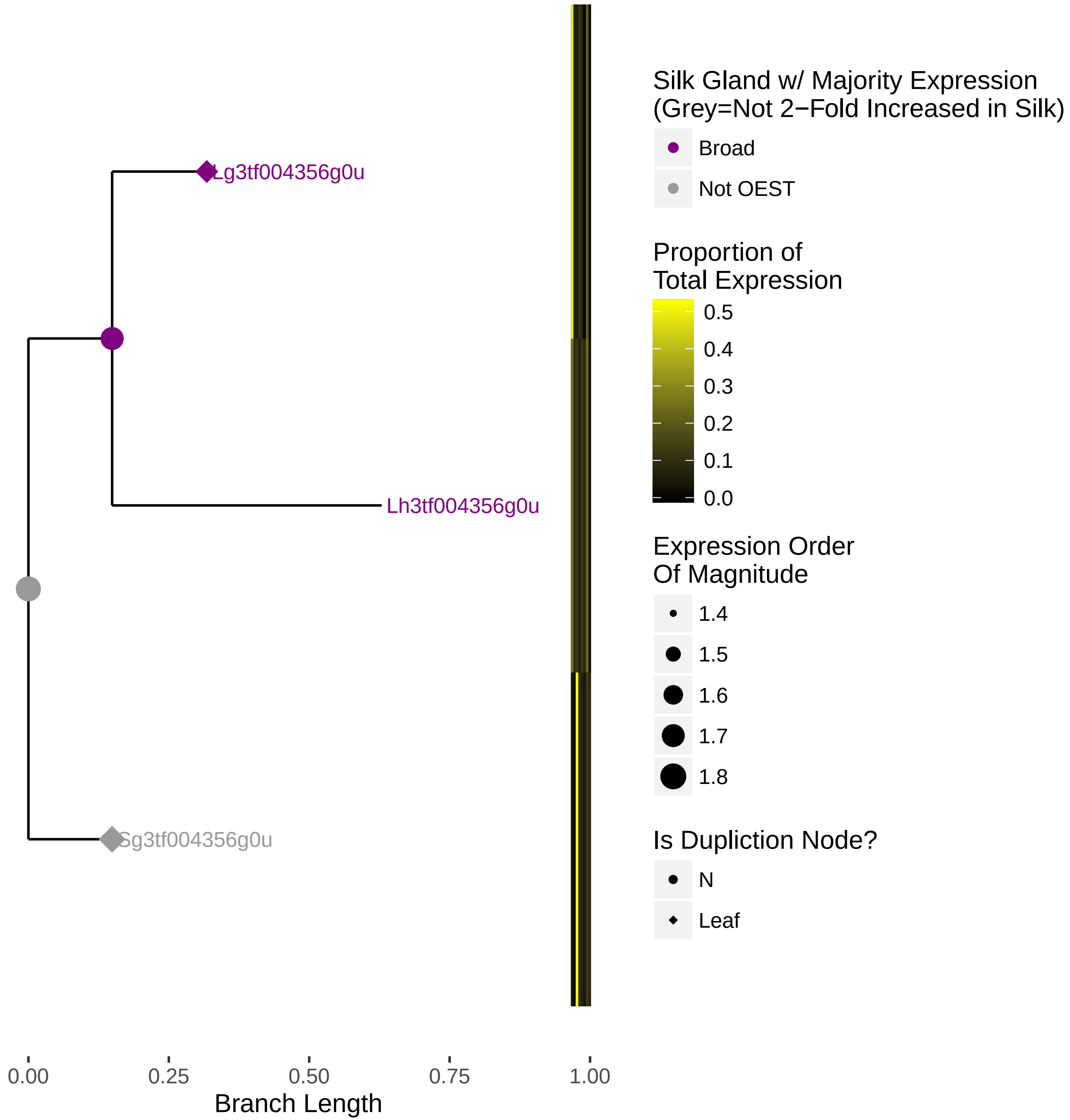

Lg3tf004356g0u
Lh3tf004356g0u
Sg3tf004356g0u
0.00
0.25
0.50
0.75
1.00
Branch Length
Silk Gland w/ Majority Expression
(Grey=Not 2−Fold Increased in Silk)
Broad
Not OEST
Proportion of
Total Expression
0.5
0.4
0.3
0.2
0.1
0.0
Expression Order
Of Magnitude
1.4
1.5
1.6
1.7
1.8
Is Dupliction Node?
N
Leaf

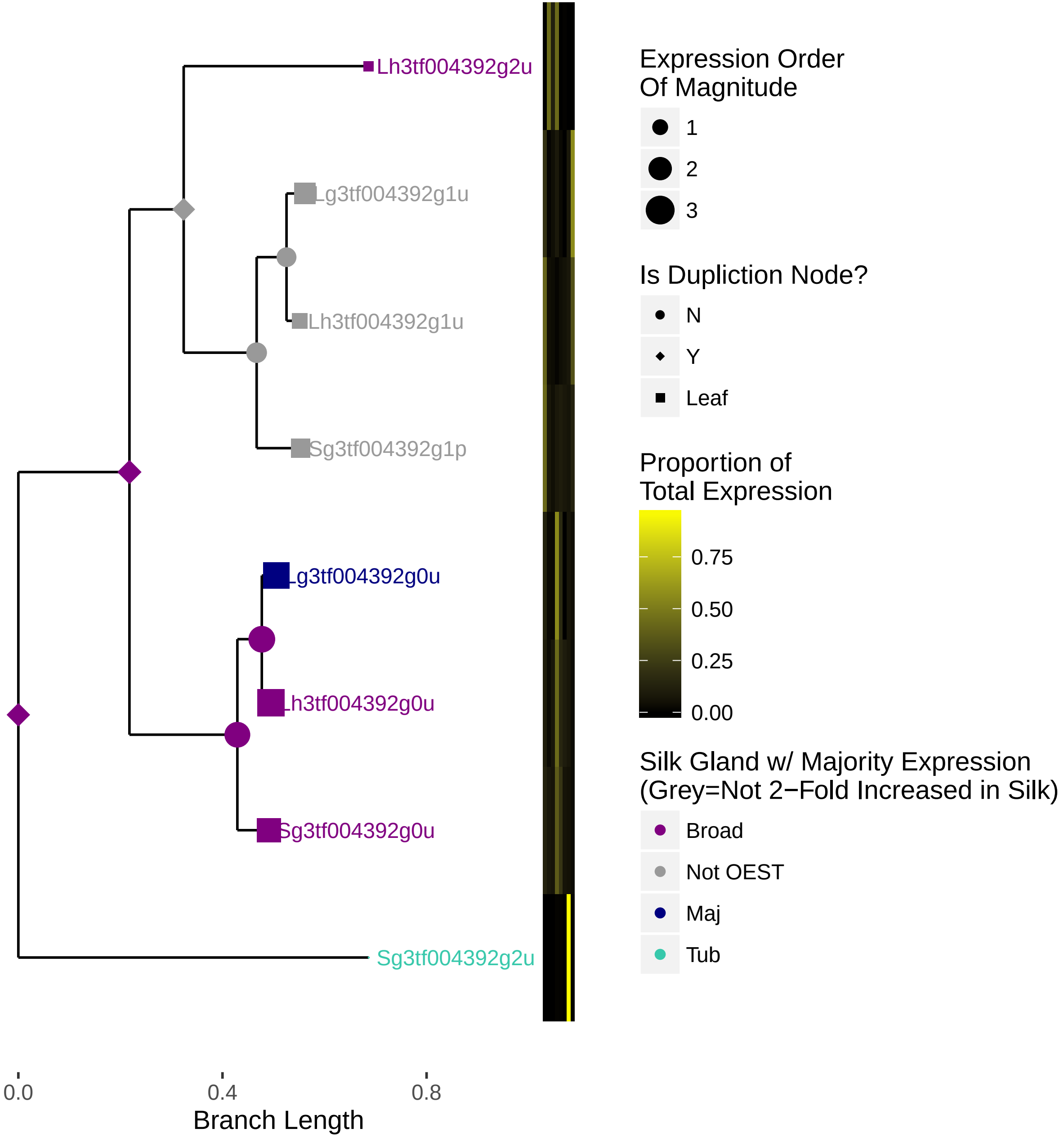
Lh3tf004392g2u
Lg3tf004392g1u
Lh3tf004392g1u
Sg3tf004392g1p
Lg3tf004392g0u
h3tf004392g0u
Sg3tf004392g0u
Sg3tf004392g2u
0.0
0.4
0.8
Branch Length
Expression Order
Of Magnitude
1
2
3
Is Dupliction Node?
N
Y
Leaf
Proportion of
Total Expression
0.75
0.50
0.25
0.00
Silk Gland w/ Majority Expression
(Grey=Not 2−Fold Increased in Silk)
Broad
Not OEST
Maj
Tub

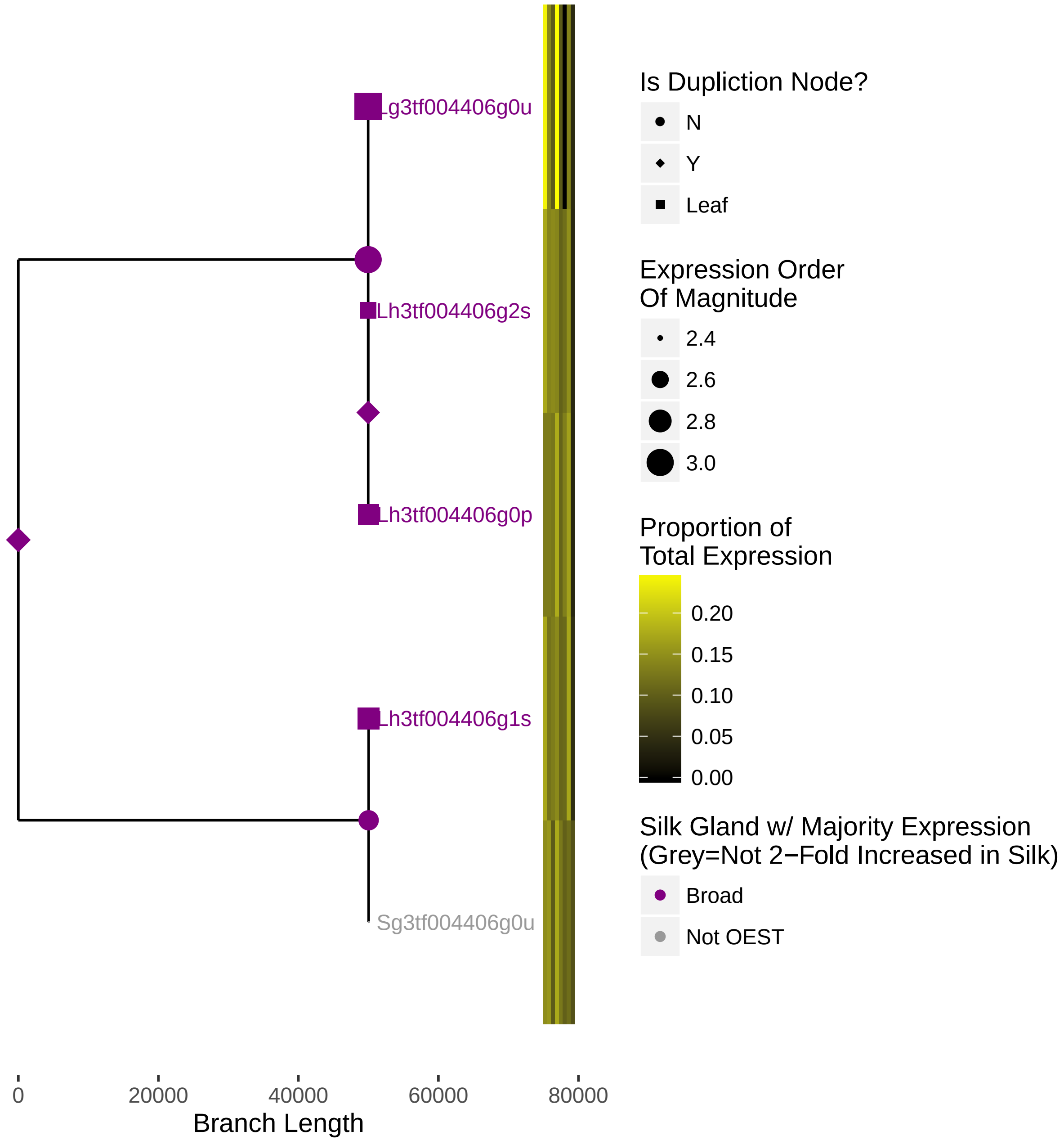

Lg3tf004406g0u
Lh3tf004406g2s
Lh3tf004406g0p
Lh3tf004406g1s
Sg3tf004406g0u
Is Dupliction Node?
N
Y
Leaf
Expression Order
Of Magnitude
2.4
2.6
2.8
3.0
Proportion of
Total Expression
0.20
0.15
0.10
0.05
0.00
Silk Gland w/ Majority Expression
(Grey=Not 2−Fold Increased in Silk)
Broad
Not OEST
0
20000
40000
60000
80000
Branch Length

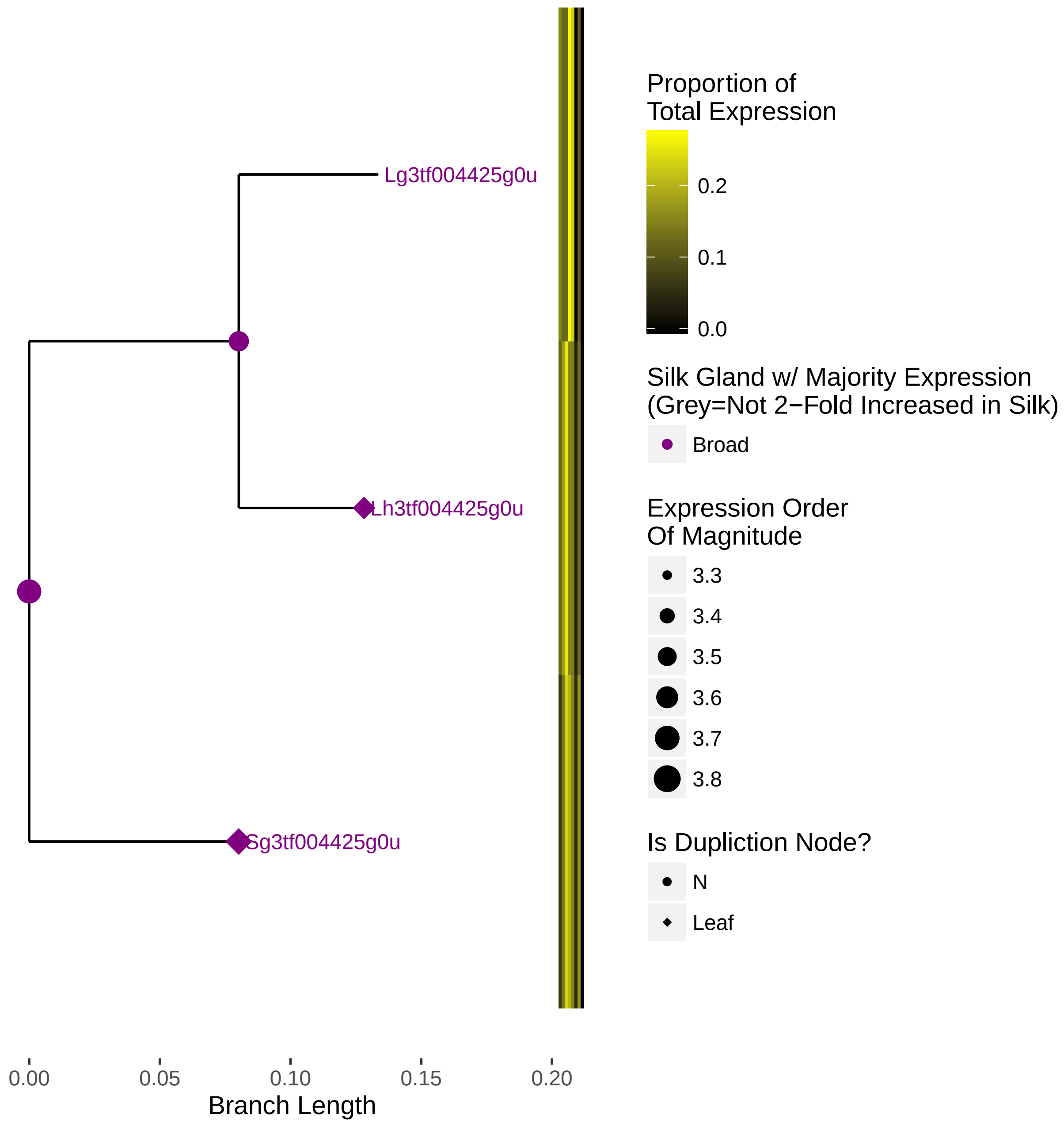

Lg3tf004425g0u
Lh3tf004425g0u
Sg3tf004425g0u
0.00
0.05
0.10
0.15
0.20
Branch Length
Proportion of
Total Expression
0.2
0.1
0.0
Silk Gland w/ Majority Expression
(Grey=Not 2−Fold Increased in Silk)
Broad
Expression Order
Of Magnitude
3.3
3.4
3.5
3.6
3.7
3.8
Is Dupliction Node?
N
Leaf

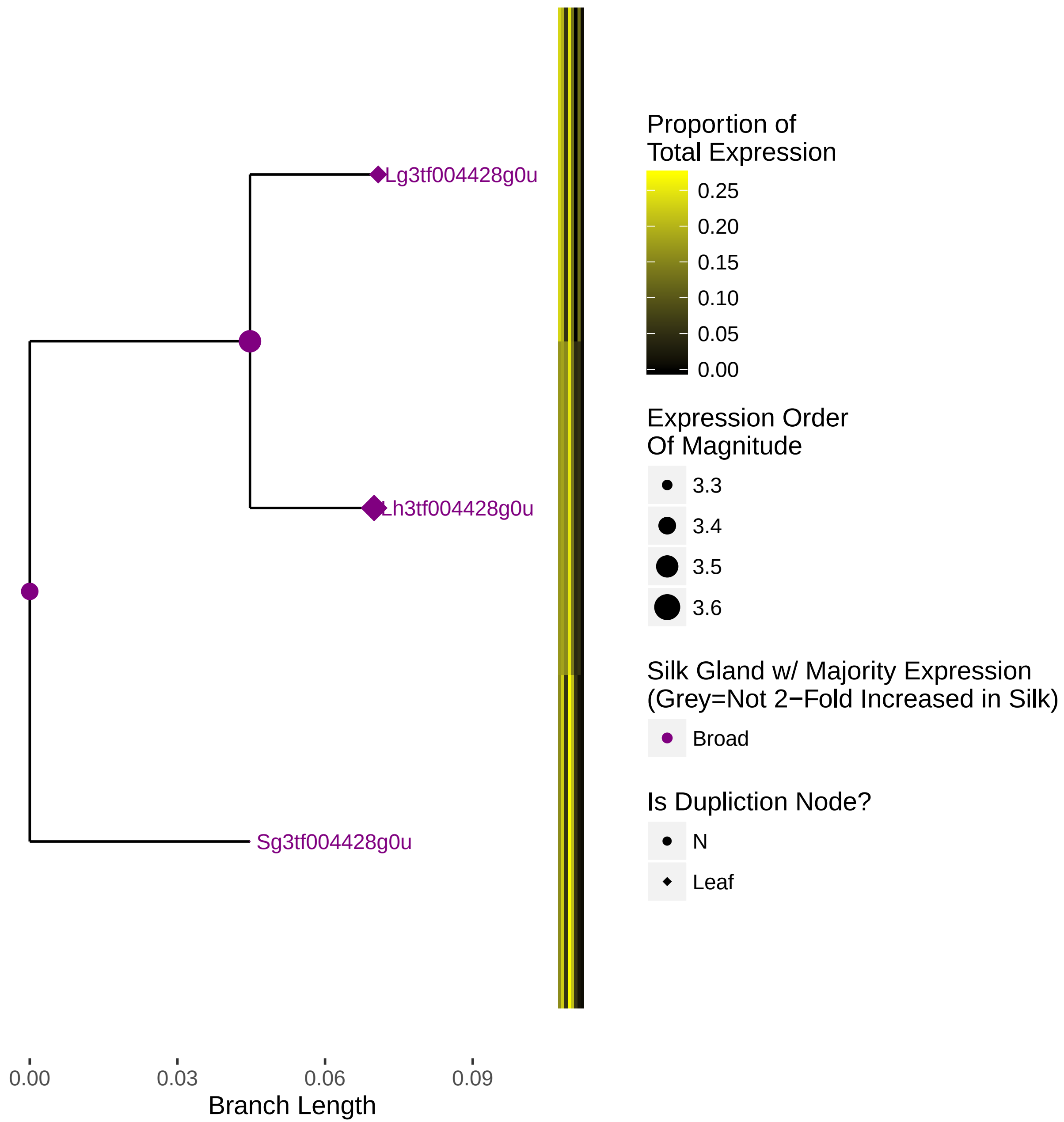

Lg3tf004428g0u
Lh3tf004428g0u
Sg3tf004428g0u
0.00
0.03
0.06
0.09
Branch Length
Proportion of
Total Expression
0.25
0.20
0.15
0.10
0.05
0.00
Expression Order
Of Magnitude
3.3
3.4
3.5
3.6
Silk Gland w/ Majority Expression
(Grey=Not 2−Fold Increased in Silk)
Broad
Is Dupliction Node?
N
Leaf

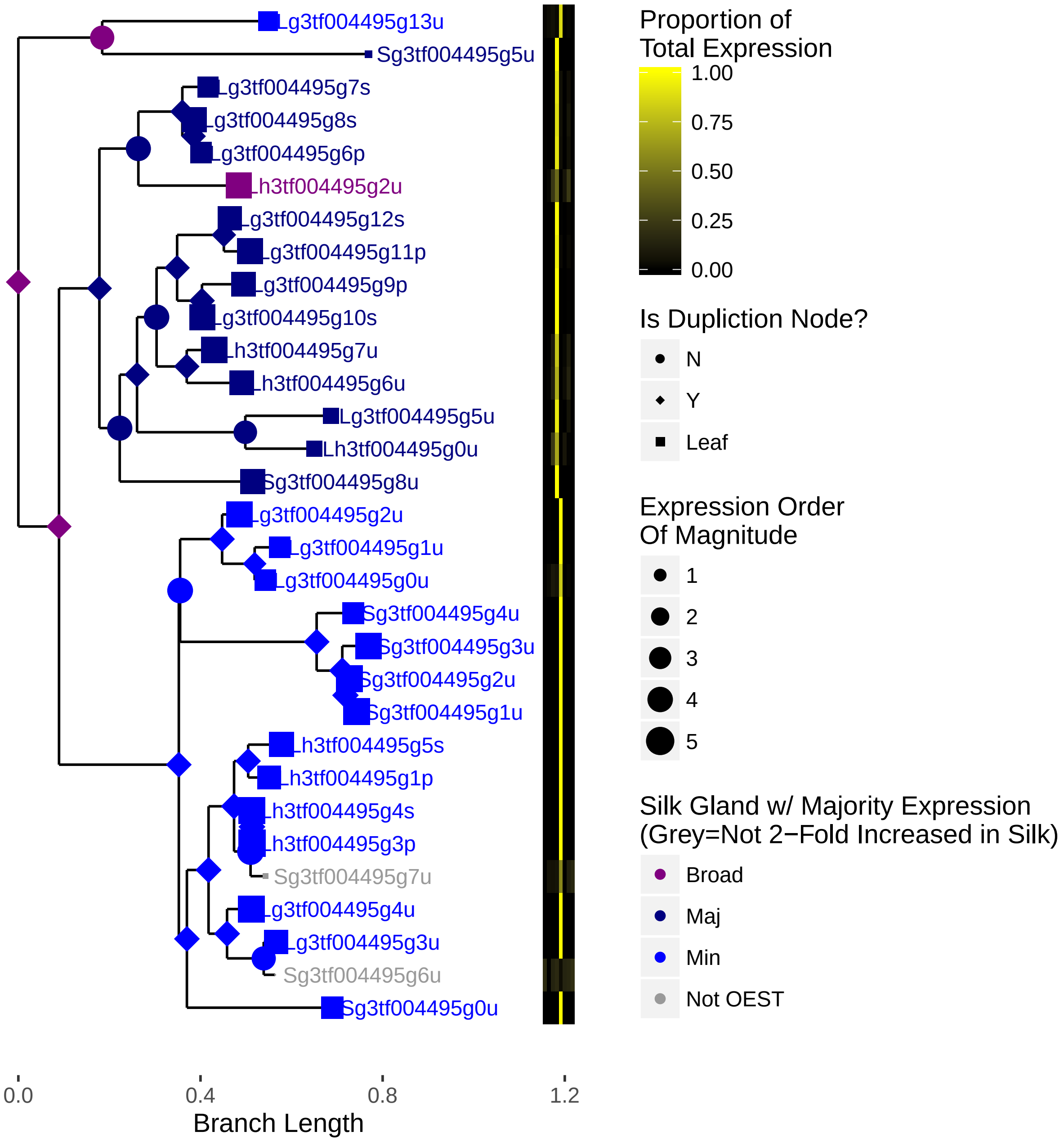
Lg3tf004495g13u
Sg3tf004495g5u
Lg3tf004495g7s
Lg3tf004495g8s
Lg3tf004495g6p
Lh3tf004495g2u
Lg3tf004495g12s
Lg3tf004495g11p
Lg3tf004495g9p
Lg3tf004495g10s
Lh3tf004495g7u
Lh3tf004495g6u
Lg3tf004495g5u
Lh3tf004495g0u
Sg3tf004495g8u
Lg3tf004495g2u
Lg3tf004495g1u
Lg3tf004495g0u
Sg3tf004495g4u
Sg3tf004495g3u
Sg3tf004495g2u
Sg3tf004495g1u
Lh3tf004495g5s
Lh3tf004495g1p
Lh3tf004495g4s
Lh3tf004495g3p
Sg3tf004495g7u
Lg3tf004495g4u
Lg3tf004495g3u
Sg3tf004495g6u
Sg3tf004495g0u
0.0
0.4
0.8
1.2
Branch Length
Proportion of Total Expression
1.00
0.75
0.50
0.25
0.00
Is Dupliction Node?
N
Y
Leaf
Expression Order Of Magnitude
1
2
3
4
5
Silk Gland w/ Majority Expression (Grey=Not 2−Fold Increased in Silk)
Broad
Maj
Min
Not OEST

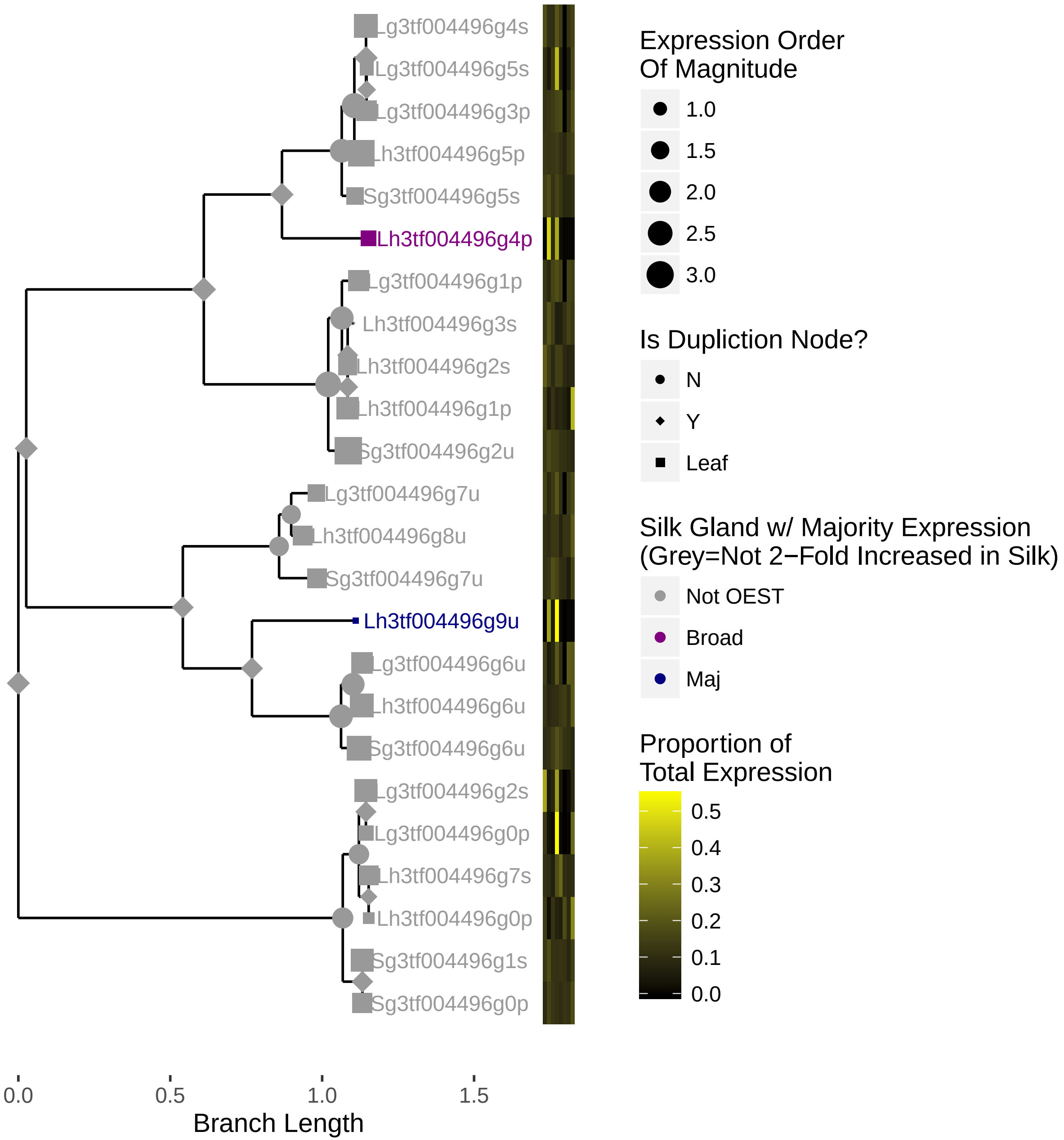

Lg3tf004496g4s
Lg3tf004496g5s
Lg3tf004496g3p
Lh3tf004496g5p
Sg3tf004496g5s
Lh3tf004496g4p
Lg3tf004496g1p
Lh3tf004496g3s
Lh3tf004496g2s
Lh3tf004496g1p
Sg3tf004496g2u
Lg3tf004496g7u
Lh3tf004496g8u
Sg3tf004496g7u
Lh3tf004496g9u
Lg3tf004496g6u
Lh3tf004496g6u
Sg3tf004496g6u
Lg3tf004496g2s
Lg3tf004496g0p
Lh3tf004496g7s
Lh3tf004496g0p
Sg3tf004496g1s
Sg3tf004496g0p
0.0
0.5
1.0
1.5
Branch Length
Expression Order Of Magnitude
1.0
1.5
2.0
2.5
3.0
Is Dupliction Node?
N
Y
Leaf
Silk Gland w/ Majority Expression (Grey=Not 2−Fold Increased in Silk)
Not OEST
Broad
Maj
Proportion of Total Expression
0.5
0.4
0.3
0.2
0.1
0.0

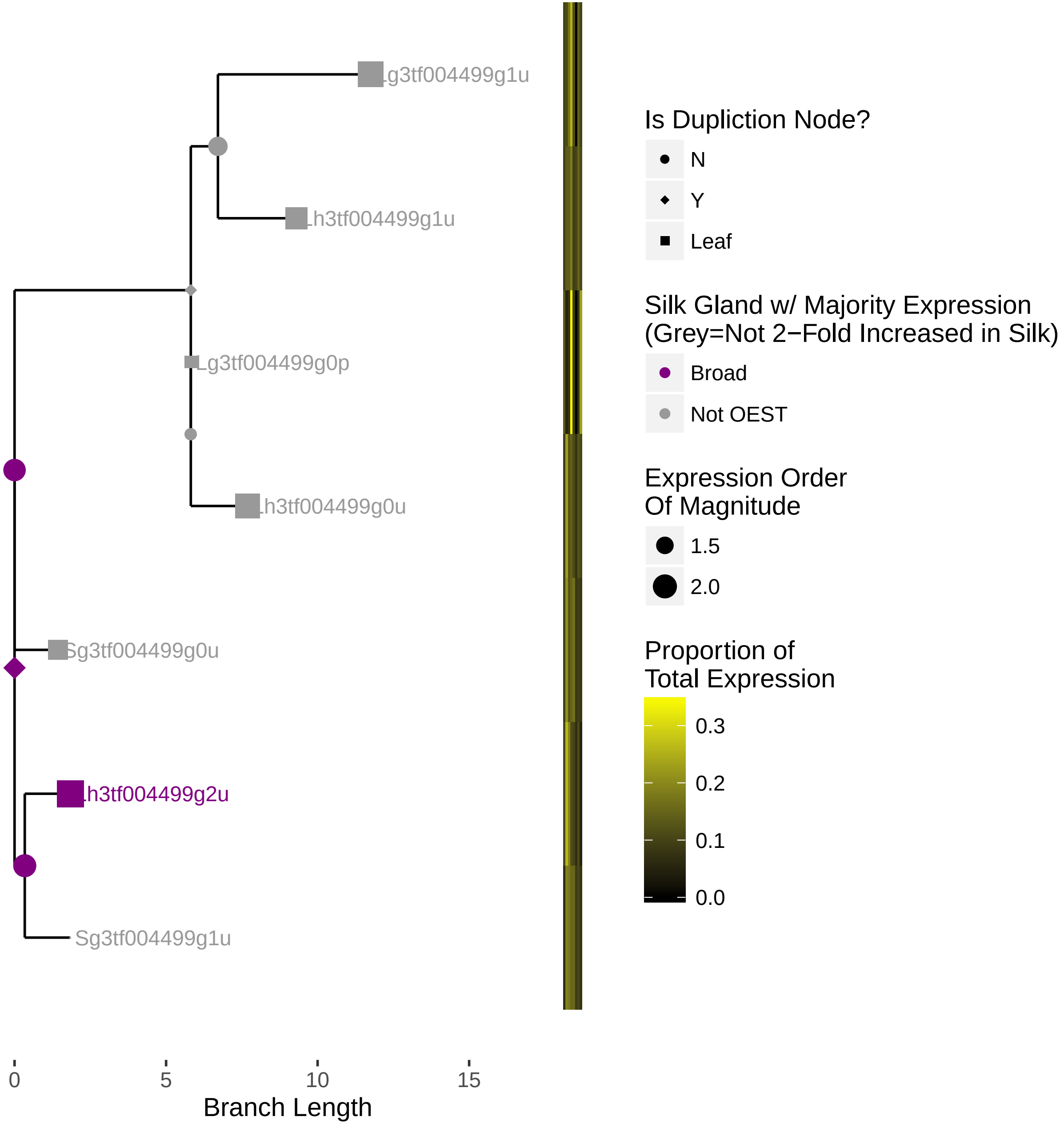

Lg3tf004499g1u
Lh3tf004499g1u
Lg3tf004499g0p
Lh3tf004499g0u
Sg3tf004499g0u
Lh3tf004499g2u
Sg3tf004499g1u
0
5
10
15
Branch Length
Is Dupliction Node?
N
Y
Leaf
Silk Gland w/ Majority Expression
(Grey=Not 2−Fold Increased in Silk)
Broad
Not OEST
Expression Order
Of Magnitude
1.5
2.0
Proportion of
Total Expression
0.3
0.2
0.1
0.0

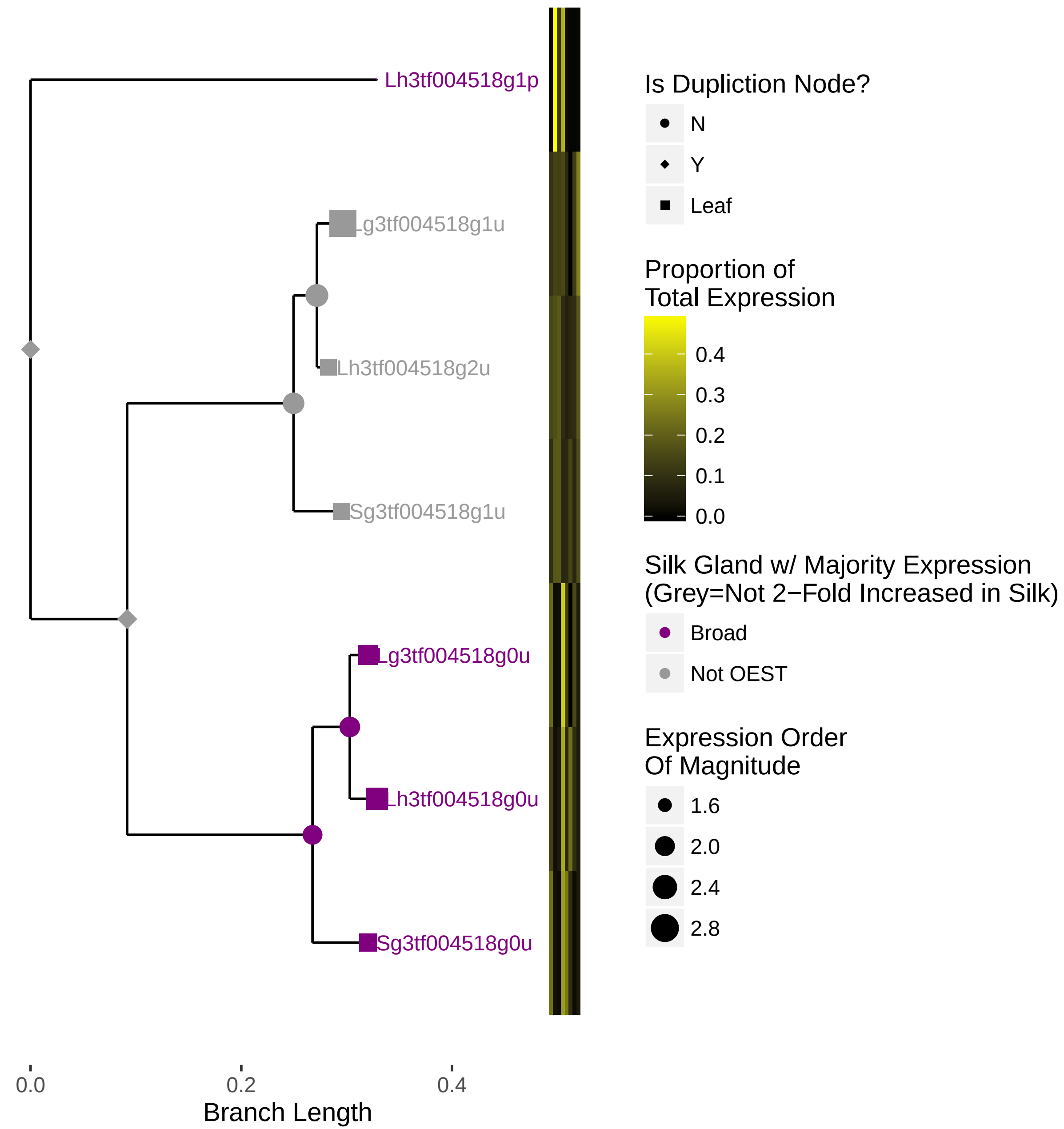
Lh3tf004518g1p
Lg3tf004518g1u
Lh3tf004518g2u
Sg3tf004518g1u
Lg3tf004518g0u
Lh3tf004518g0u
Sg3tf004518g0u
0.0
0.2
0.4
Branch Length
Is Dupliction Node?
N
Y
Leaf
Proportion of
Total Expression
0.4
0.3
0.2
0.1
0.0
Silk Gland w/ Majority Expression
(Grey=Not 2−Fold Increased in Silk)
Broad
Not OEST
Expression Order
Of Magnitude
1.6
2.0
2.4
2.8

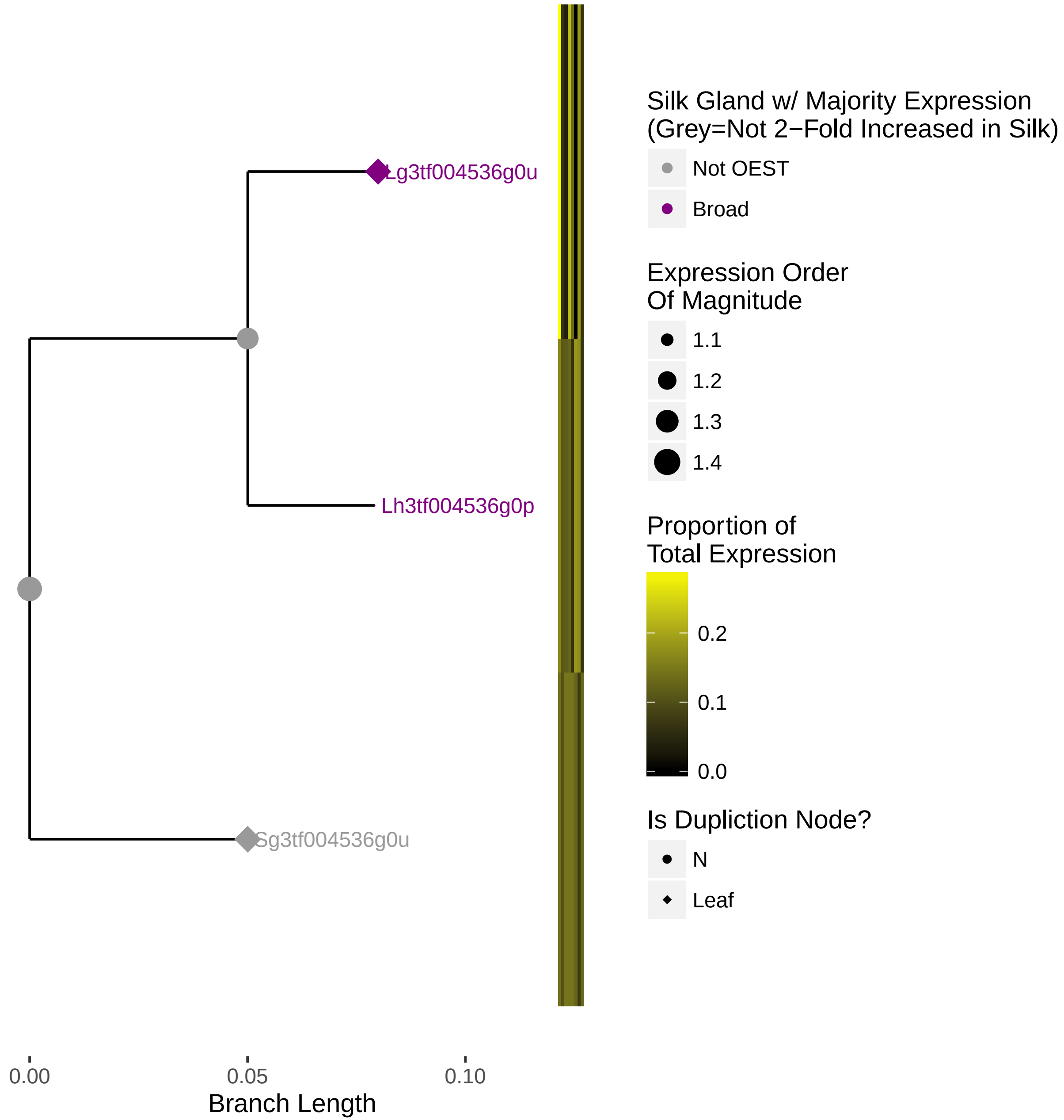

Lg3tf004536g0u
Lh3tf004536g0p
Sg3tf004536g0u
0.00
0.05
0.10
Branch Length
Silk Gland w/ Majority Expression
(Grey=Not 2−Fold Increased in Silk)
Not OEST
Broad
Expression Order
Of Magnitude
1.1
1.2
1.3
1.4
Proportion of
Total Expression
0.2
0.1
0.0
Is Dupliction Node?
N
Leaf

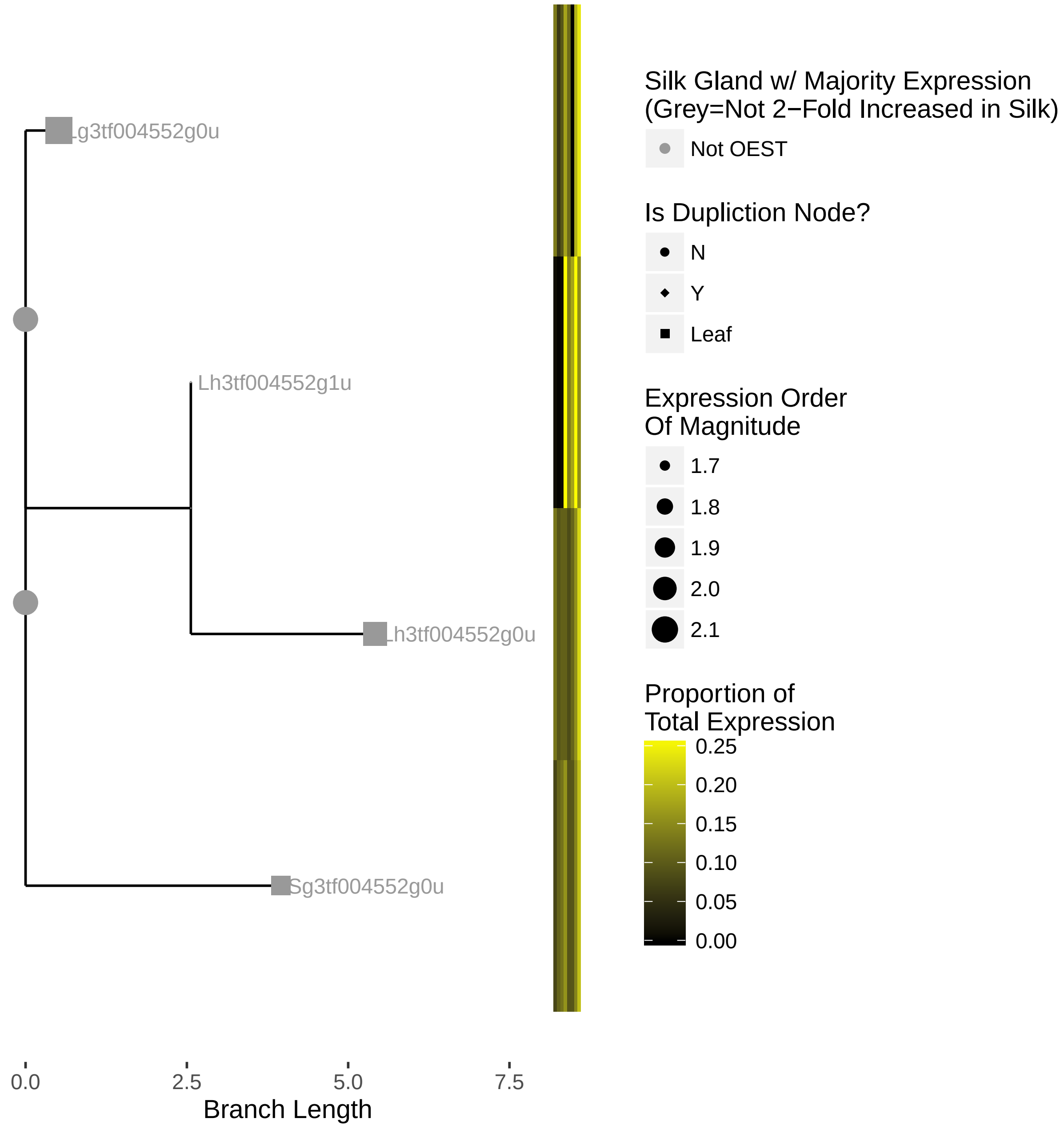

Lg3tf004552g0u
Lh3tf004552g1u
Lh3tf004552g0u
Sg3tf004552g0u
0.0
2.5
5.0
7.5
Branch Length
Silk Gland w/ Majority Expression
(Grey=Not 2−Fold Increased in Silk)
Not OEST
Is Dupliction Node?
N
Y
Leaf
Expression Order
Of Magnitude
1.7
1.8
1.9
2.0
2.1
Proportion of
Total Expression
0.25
0.20
0.15
0.10
0.05
0.00

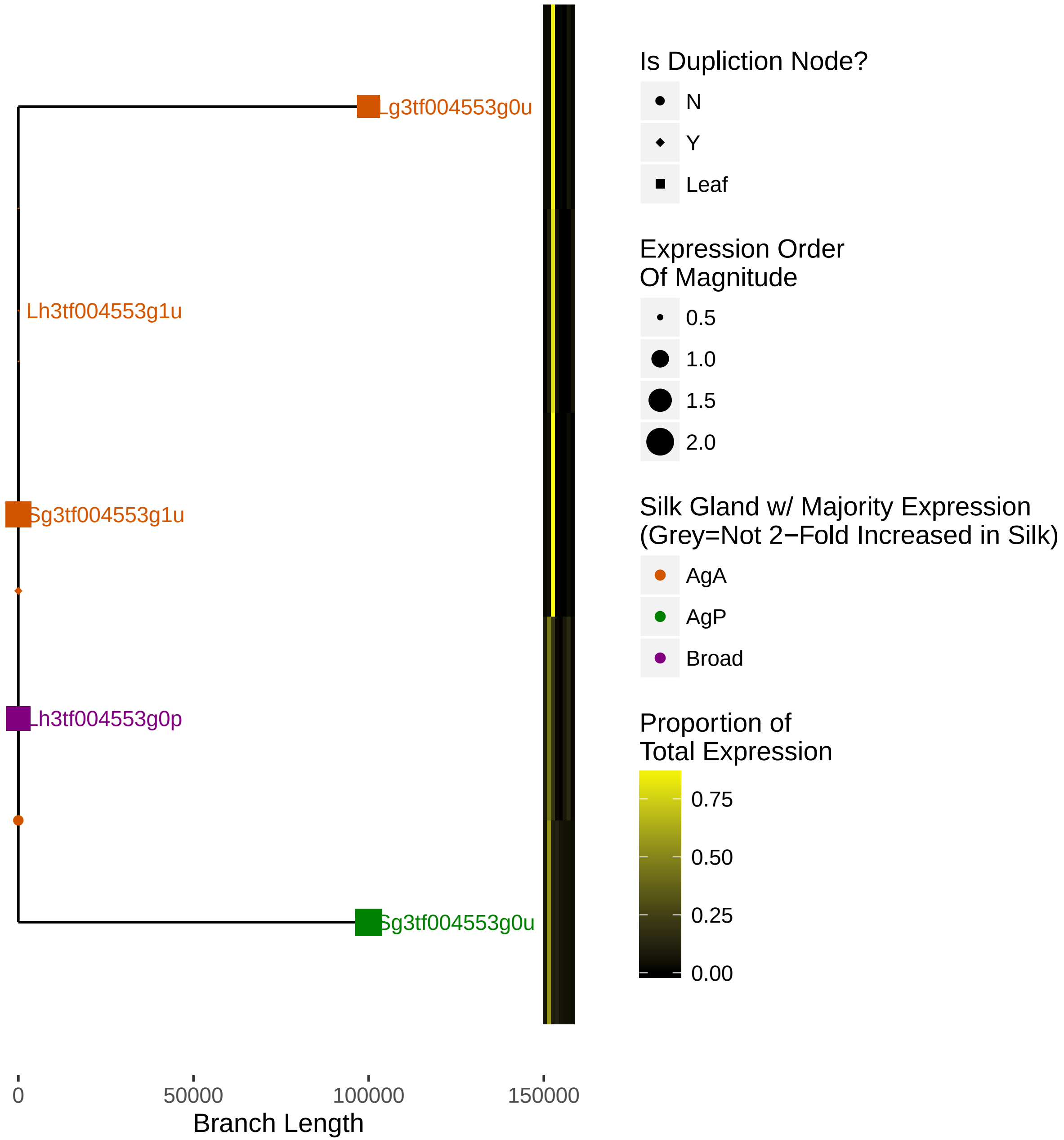

Lg3tf004553g0u
Lh3tf004553g1u
Sg3tf004553g1u
Lh3tf004553g0p
Sg3tf004553g0u
0
50000
100000
150000
Branch Length
Is Dupliction Node?
N
Y
Leaf
Expression Order Of Magnitude
0.5
1.0
1.5
2.0
Silk Gland w/ Majority Expression (Grey=Not 2−Fold Increased in Silk)
AgA
AgP
Broad
Proportion of Total Expression
0.75
0.50
0.25
0.00

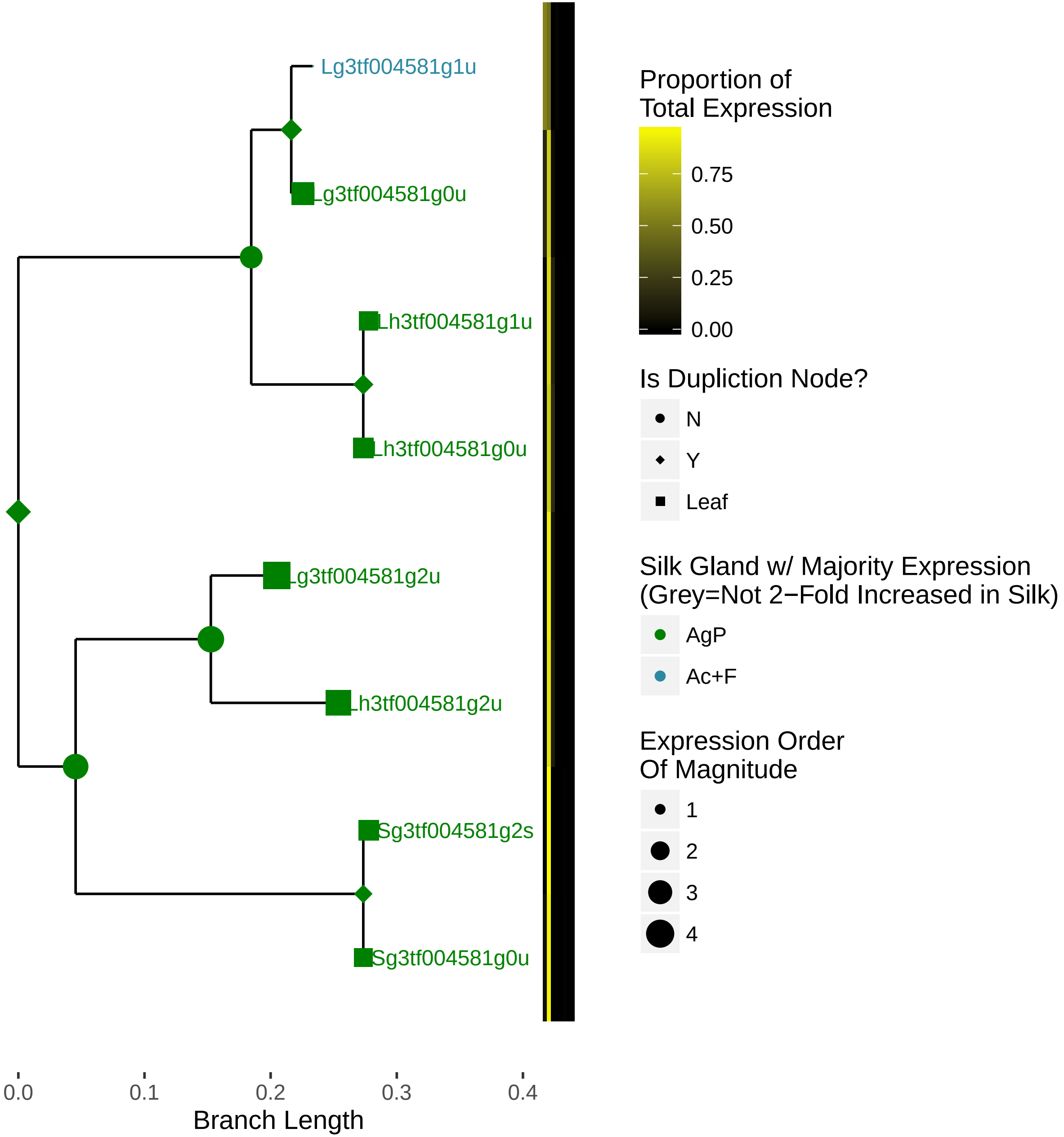

Lg3tf004581g1u
Lg3tf004581g0u
Lh3tf004581g1u
Lh3tf004581g0u
Lg3tf004581g2u
Lh3tf004581g2u
Sg3tf004581g2s
Sg3tf004581g0u
0.0
0.1
0.2
0.3
0.4
Branch Length
Proportion of Total Expression
0.75
0.50
0.25
0.00
Is Dupliction Node?
N
Y
Leaf
Silk Gland w/ Majority Expression (Grey=Not 2−Fold Increased in Silk)
AgP
Ac+F
Expression Order Of Magnitude
1
2
3
4

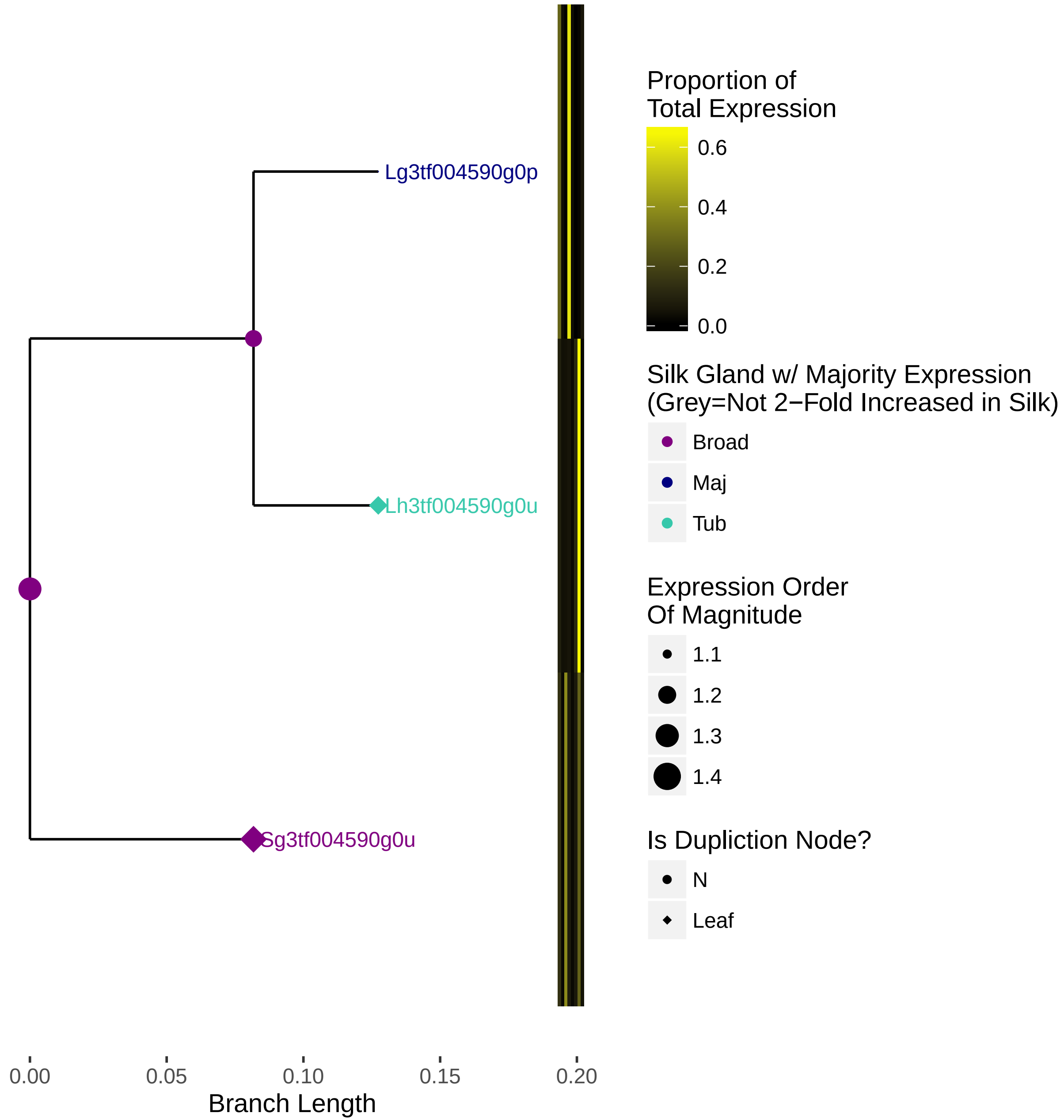

Lg3tf004590g0p
Lh3tf004590g0u
Sg3tf004590g0u
0.00
0.05
0.10
0.15
0.20
Branch Length
Proportion of Total Expression
0.6
0.4
0.2
0.0
Silk Gland w/ Majority Expression (Grey=Not 2−Fold Increased in Silk)
Broad
Maj
Tub
Expression Order Of Magnitude
1.1
1.2
1.3
1.4
Is Dupliction Node?
N
Leaf

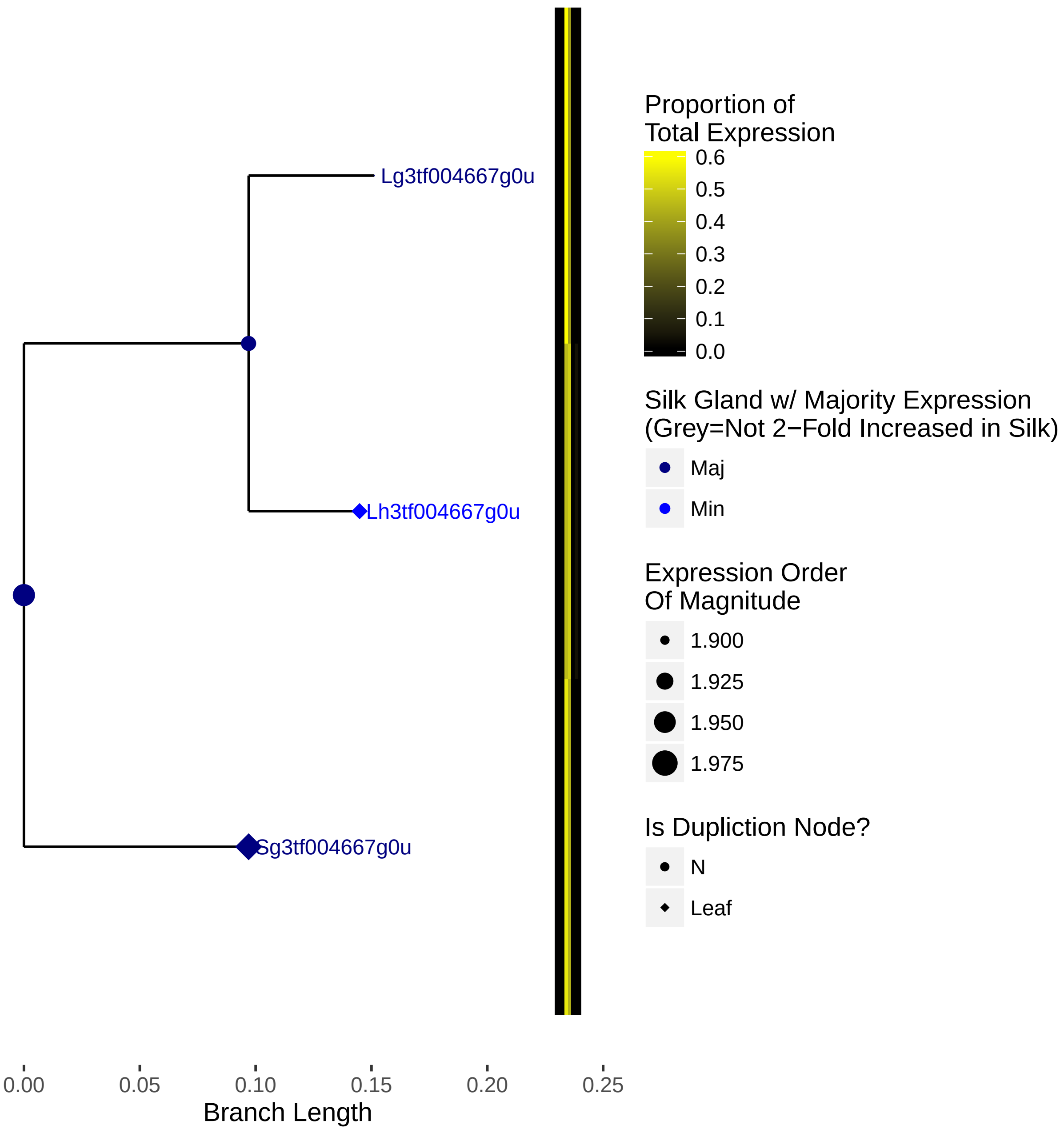

Lg3tf004667g0u
Lh3tf004667g0u
Sg3tf004667g0u
Proportion of
Total Expression
0.6
0.5
0.4
0.3
0.2
0.1
0.0
Silk Gland w/ Majority Expression
(Grey=Not 2−Fold Increased in Silk)
Maj
Min
Expression Order
Of Magnitude
1.900
1.925
1.950
1.975
Is Dupliction Node?
N
Leaf
0.00
0.05
0.10
0.15
0.20
0.25
Branch Length

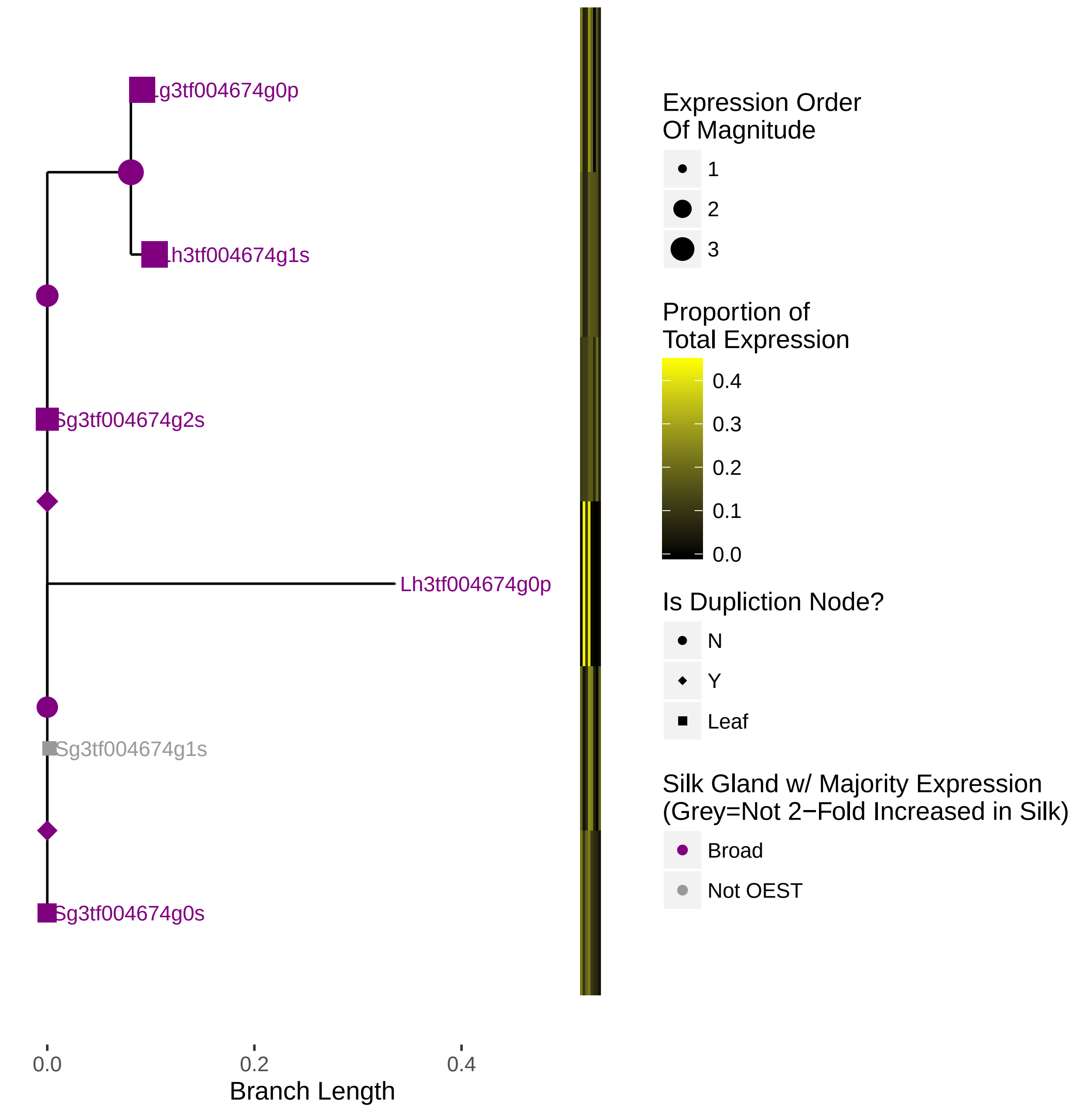

g3tf004674g0p
h3tf004674g1s
Sg3tf004674g2s
Lh3tf004674g0p
Sg3tf004674g1s
Sg3tf004674g0s
0.0
0.2
0.4
Branch Length
Expression Order
Of Magnitude
1
2
3
Proportion of
Total Expression
0.4
0.3
0.2
0.1
0.0
Is Dupliction Node?
N
Y
Leaf
Silk Gland w/ Majority Expression
(Grey=Not 2−Fold Increased in Silk)
Broad
Not OEST

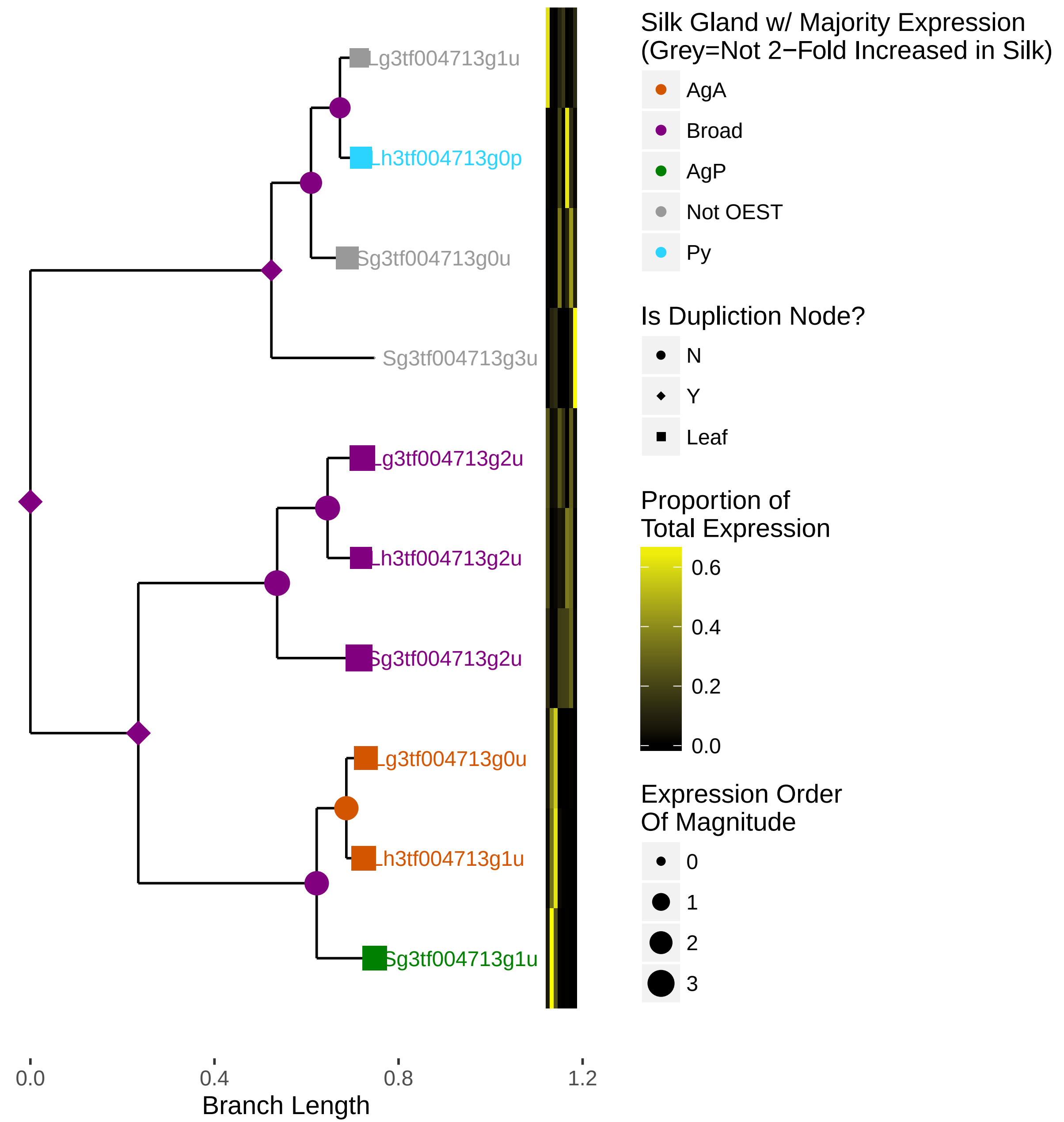

Lg3tf004713g1u
Lh3tf004713g0p
Sg3tf004713g0u
Sg3tf004713g3u
Lg3tf004713g2u
Lh3tf004713g2u
Sg3tf004713g2u
Lg3tf004713g0u
Lh3tf004713g1u
Sg3tf004713g1u
0.0
0.4
0.8
1.2
Branch Length
Silk Gland w/ Majority Expression
(Grey=Not 2−Fold Increased in Silk)
AgA
Broad
AgP
Not OEST
Py
Is Dupliction Node?
N
Y
Leaf
Proportion of
Total Expression
0.6
0.4
0.2
0.0
Expression Order
Of Magnitude
0
1
2
3

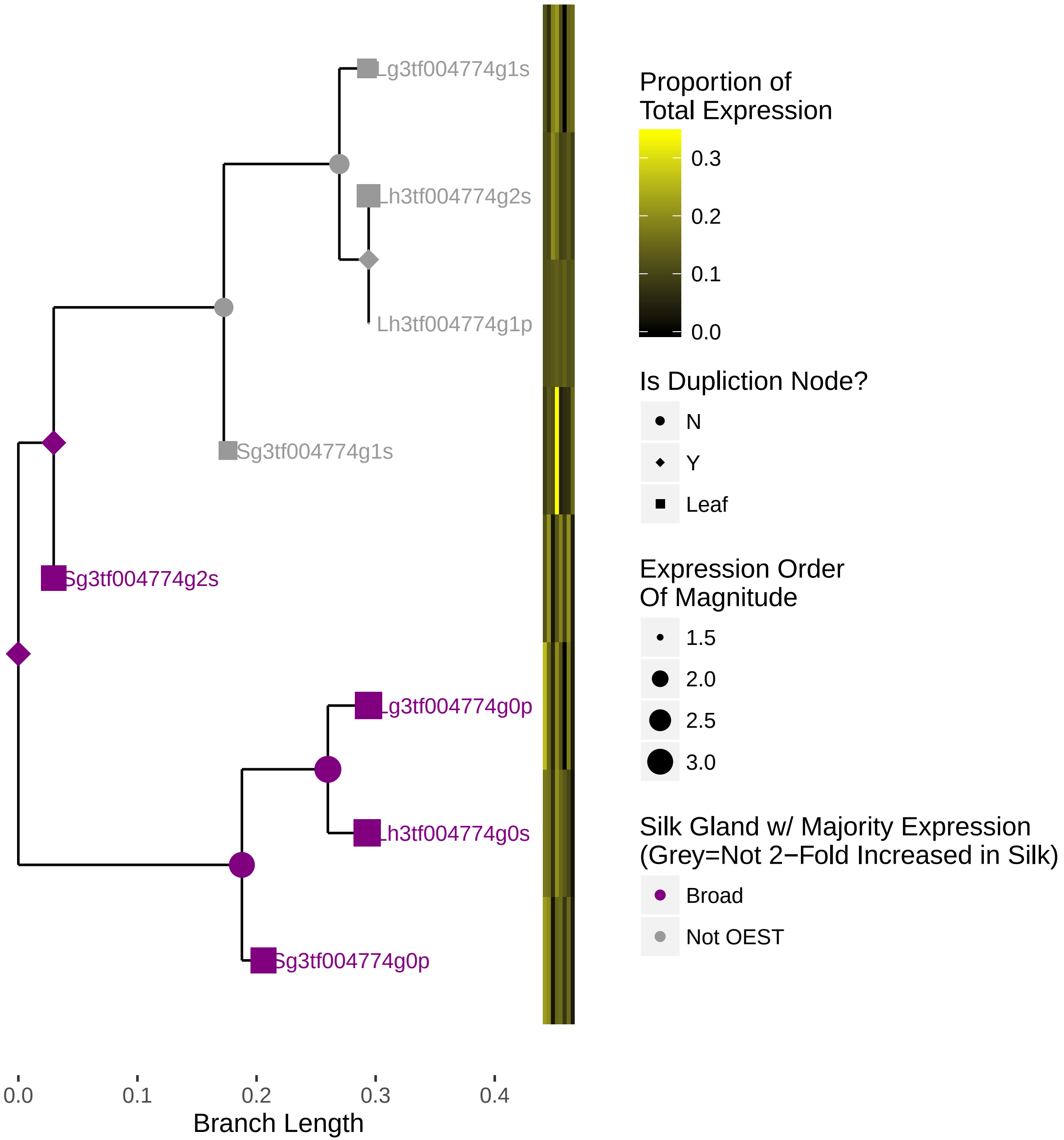
Lg3tf004774g1s
Lh3tf004774g2s
Lh3tf004774g1p
Sg3tf004774g1s
Sg3tf004774g2s
Lg3tf004774g0p
Lh3tf004774g0s
Sg3tf004774g0p
0.0
0.1
0.2
0.3
0.4
Branch Length
Proportion of
Total Expression
0.3
0.2
0.1
0.0
Is Dupliction Node?
N
Y
Leaf
Expression Order
Of Magnitude
1.5
2.0
2.5
3.0
Silk Gland w/ Majority Expression
(Grey=Not 2−Fold Increased in Silk)
Broad
Not OEST

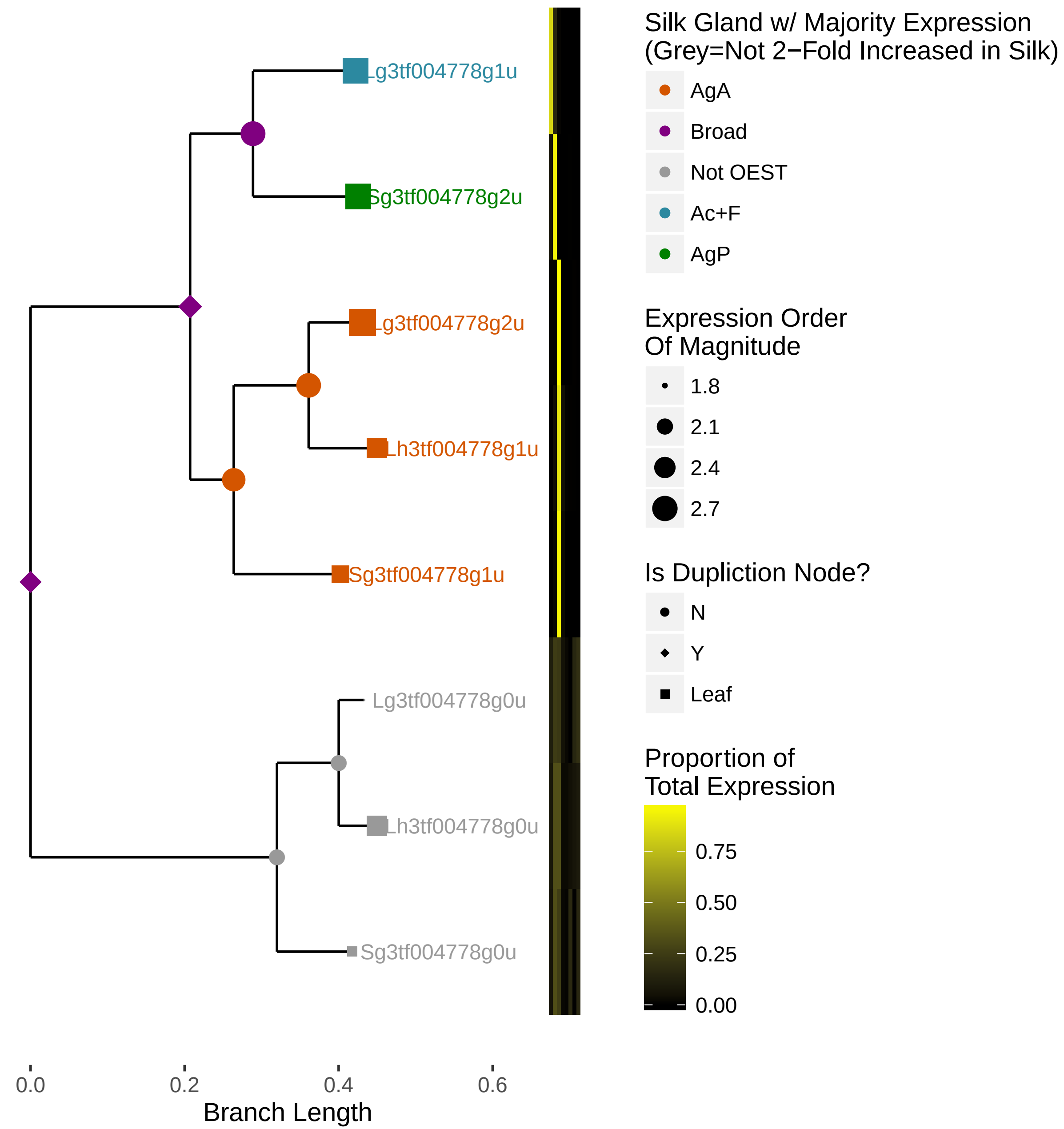
Lg3tf004778g1u
Sg3tf004778g2u
Lg3tf004778g2u
Lh3tf004778g1u
Sg3tf004778g1u
Lg3tf004778g0u
Lh3tf004778g0u
Sg3tf004778g0u
0.0
0.2
0.4
0.6
Branch Length
Silk Gland w/ Majority Expression
(Grey=Not 2−Fold Increased in Silk)
AgA
Broad
Not OEST
Ac+F
AgP
Expression Order
Of Magnitude
1.8
2.1
2.4
2.7
Is Dupliction Node?
N
Y
Leaf
Proportion of
Total Expression
0.75
0.50
0.25
0.00

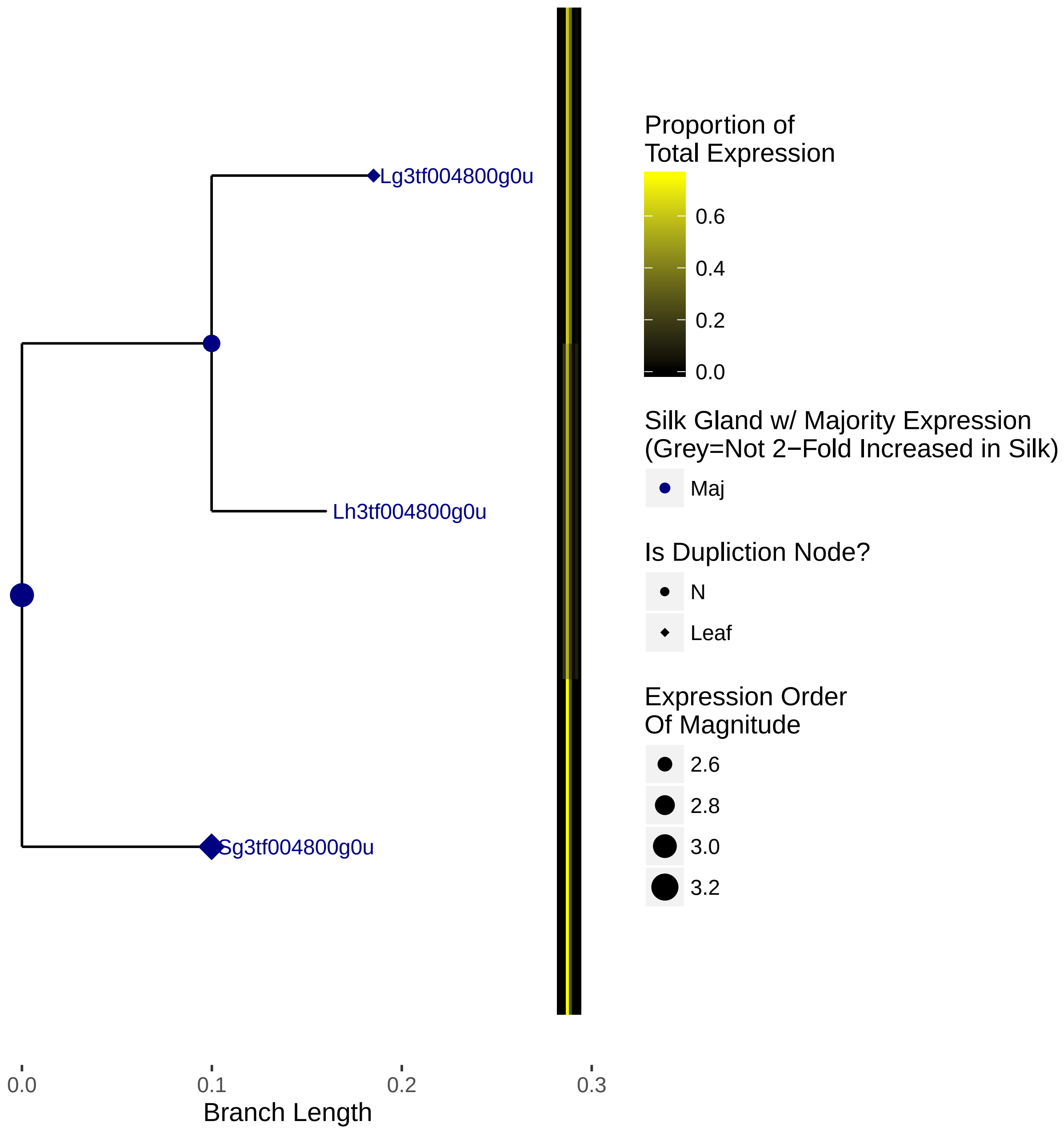

Lg3tf004800g0u
Lh3tf004800g0u
Sg3tf004800g0u
0.0
0.1
0.2
0.3
Branch Length
Proportion of
Total Expression
0.6
0.4
0.2
0.0
Silk Gland w/ Majority Expression
(Grey=Not 2−Fold Increased in Silk)
Maj
Is Dupliction Node?
N
Leaf
Expression Order
Of Magnitude
2.6
2.8
3.0
3.2

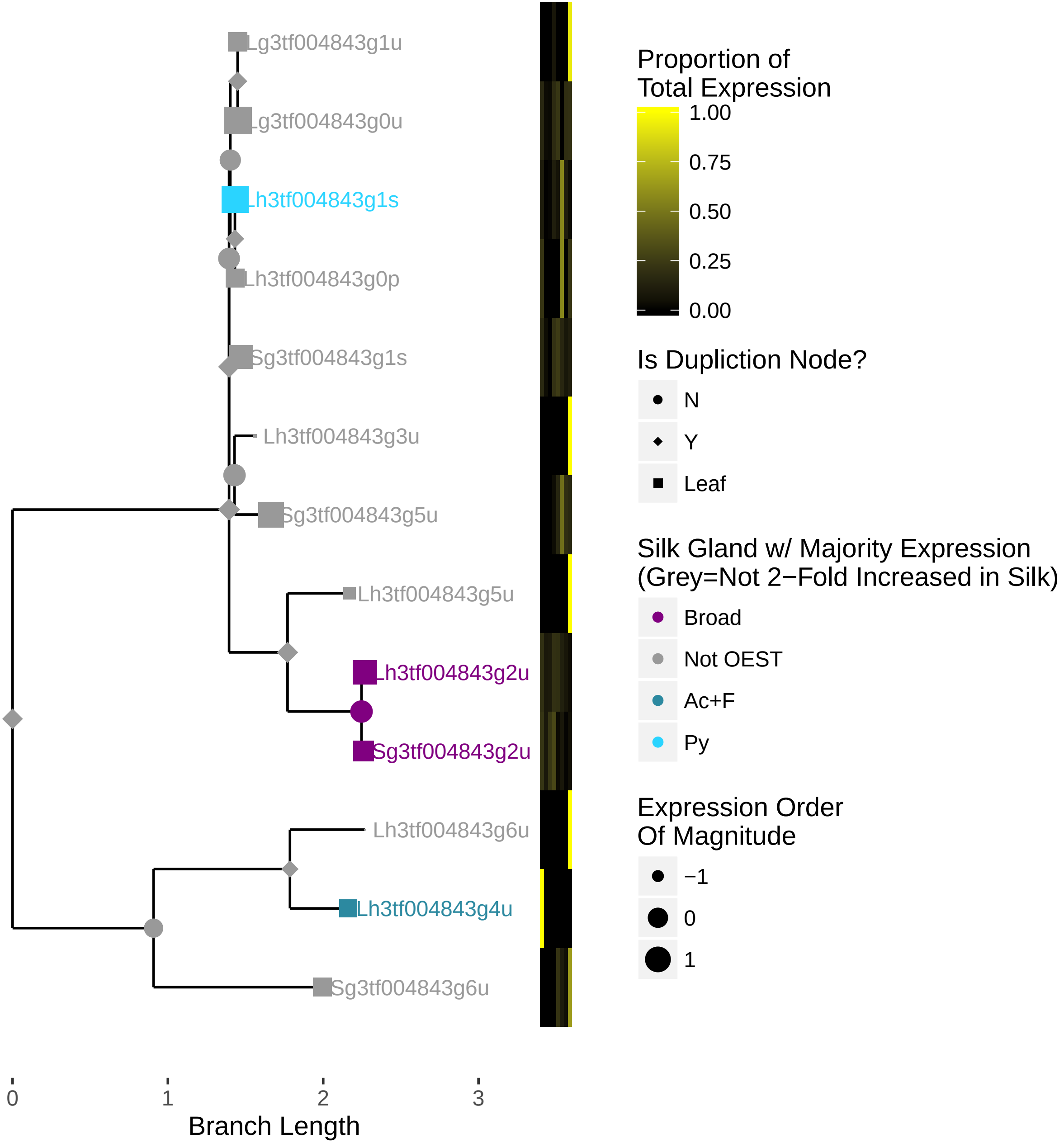

Lg3tf004843g1u
Lg3tf004843g0u
Lh3tf004843g1s
Lh3tf004843g0p
Sg3tf004843g1s
Lh3tf004843g3u
Sg3tf004843g5u
Lh3tf004843g5u
Lh3tf004843g2u
Sg3tf004843g2u
Lh3tf004843g6u
Lh3tf004843g4u
Sg3tf004843g6u
0
1
2
3
Branch Length
Proportion of Total Expression
1.00
0.75
0.50
0.25
0.00
Is Dupliction Node?
N
Y
Leaf
Silk Gland w/ Majority Expression (Grey=Not 2−Fold Increased in Silk)
Broad
Not OEST
Ac+F
Py
Expression Order Of Magnitude
−1
0
1

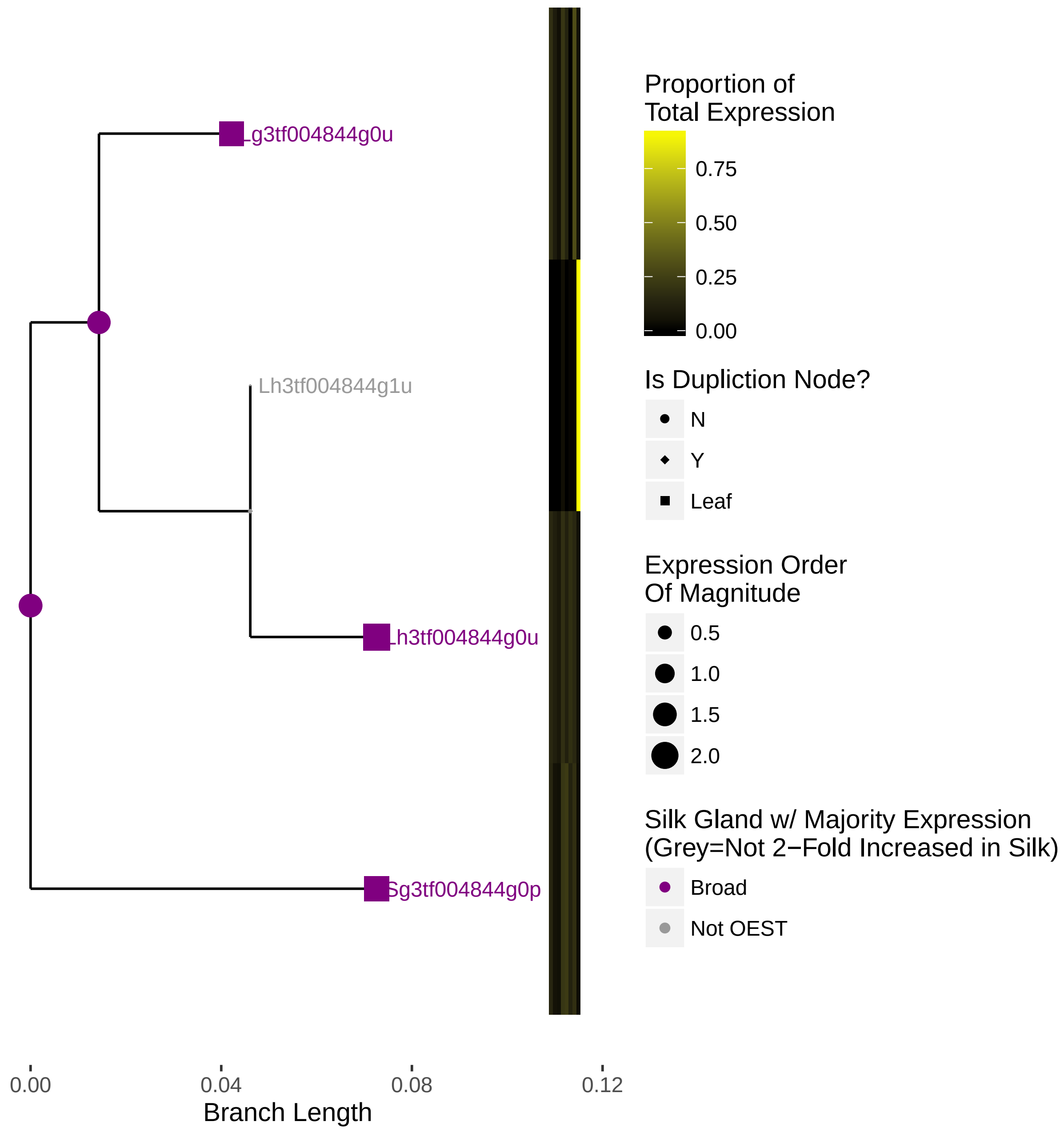

Lg3tf004844g0u
Lh3tf004844g1u
Lh3tf004844g0u
Sg3tf004844g0p
0.00
0.04
0.08
0.12
Branch Length
Proportion of
Total Expression
0.75
0.50
0.25
0.00
Is Dupliction Node?
N
Y
Leaf
Expression Order
Of Magnitude
0.5
1.0
1.5
2.0
Silk Gland w/ Majority Expression
(Grey=Not 2−Fold Increased in Silk)
Broad
Not OEST

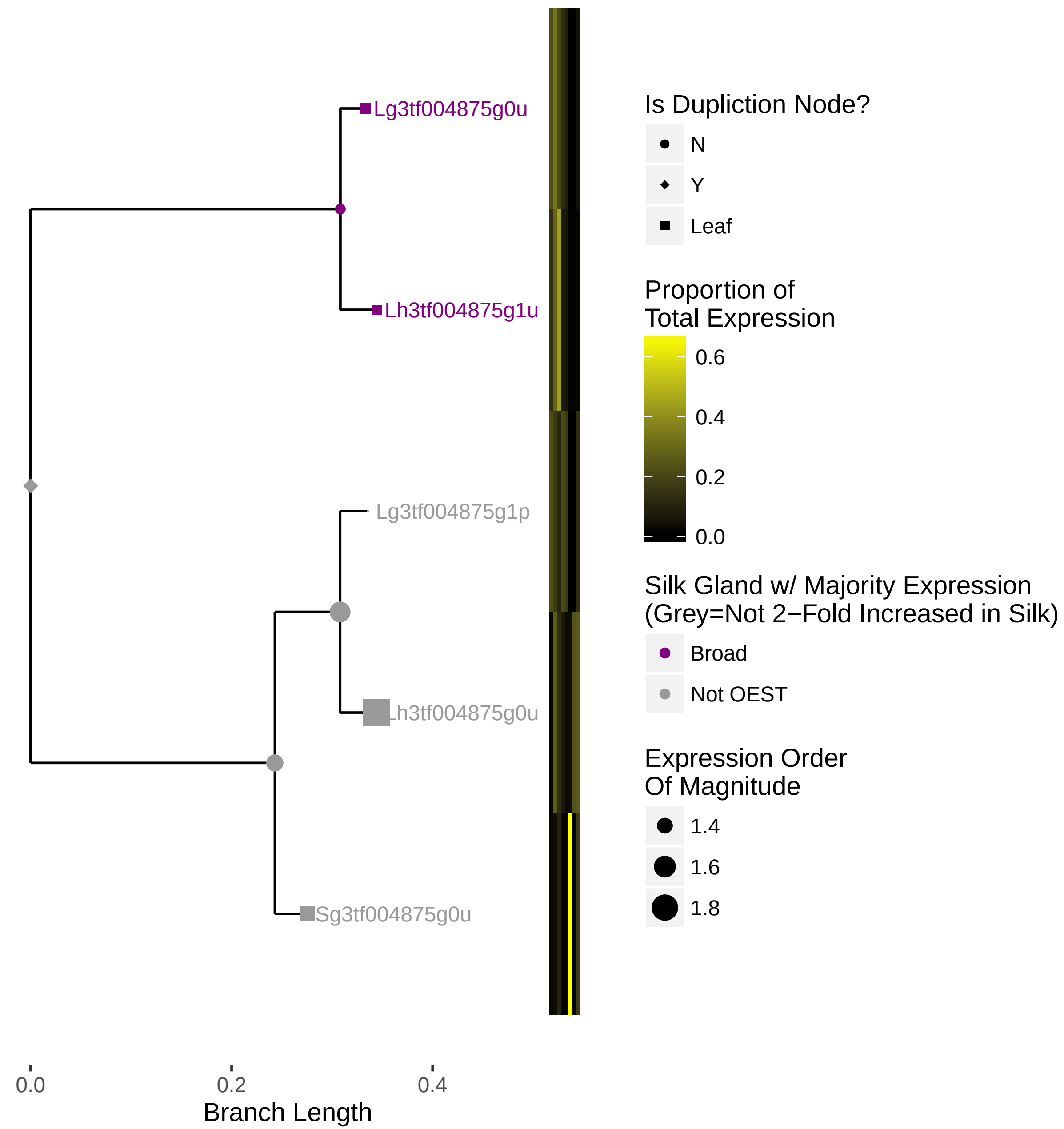

Lg3tf004875g0u
Lh3tf004875g1u
Lg3tf004875g1p
Lh3tf004875g0u
Sg3tf004875g0u
0.0
0.2
0.4
Branch Length
Is Dupliction Node?
N
Y
Leaf
Proportion of
Total Expression
0.6
0.4
0.2
0.0
Silk Gland w/ Majority Expression
(Grey=Not 2−Fold Increased in Silk)
Broad
Not OEST
Expression Order
Of Magnitude
1.4
1.6
1.8

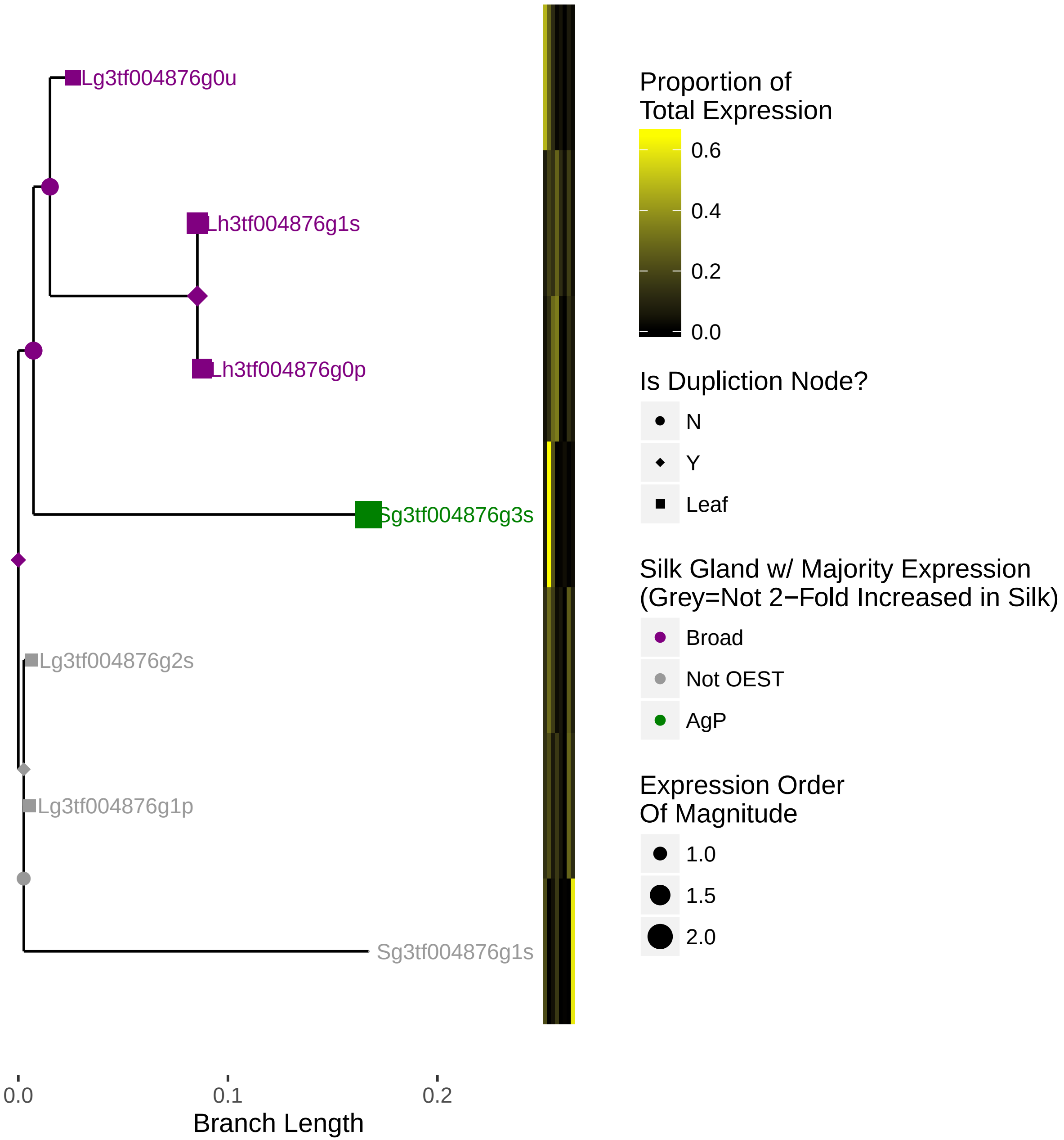

Lg3tf004876g0u
Lh3tf004876g1s
Lh3tf004876g0p
Sg3tf004876g3s
Lg3tf004876g2s
Lg3tf004876g1p
Sg3tf004876g1s
0.0
0.1
0.2
Branch Length
Proportion of
Total Expression
0.6
0.4
0.2
0.0
Is Dupliction Node?
N
Y
Leaf
Silk Gland w/ Majority Expression
(Grey=Not 2−Fold Increased in Silk)
Broad
Not OEST
AgP
Expression Order
Of Magnitude
1.0
1.5
2.0

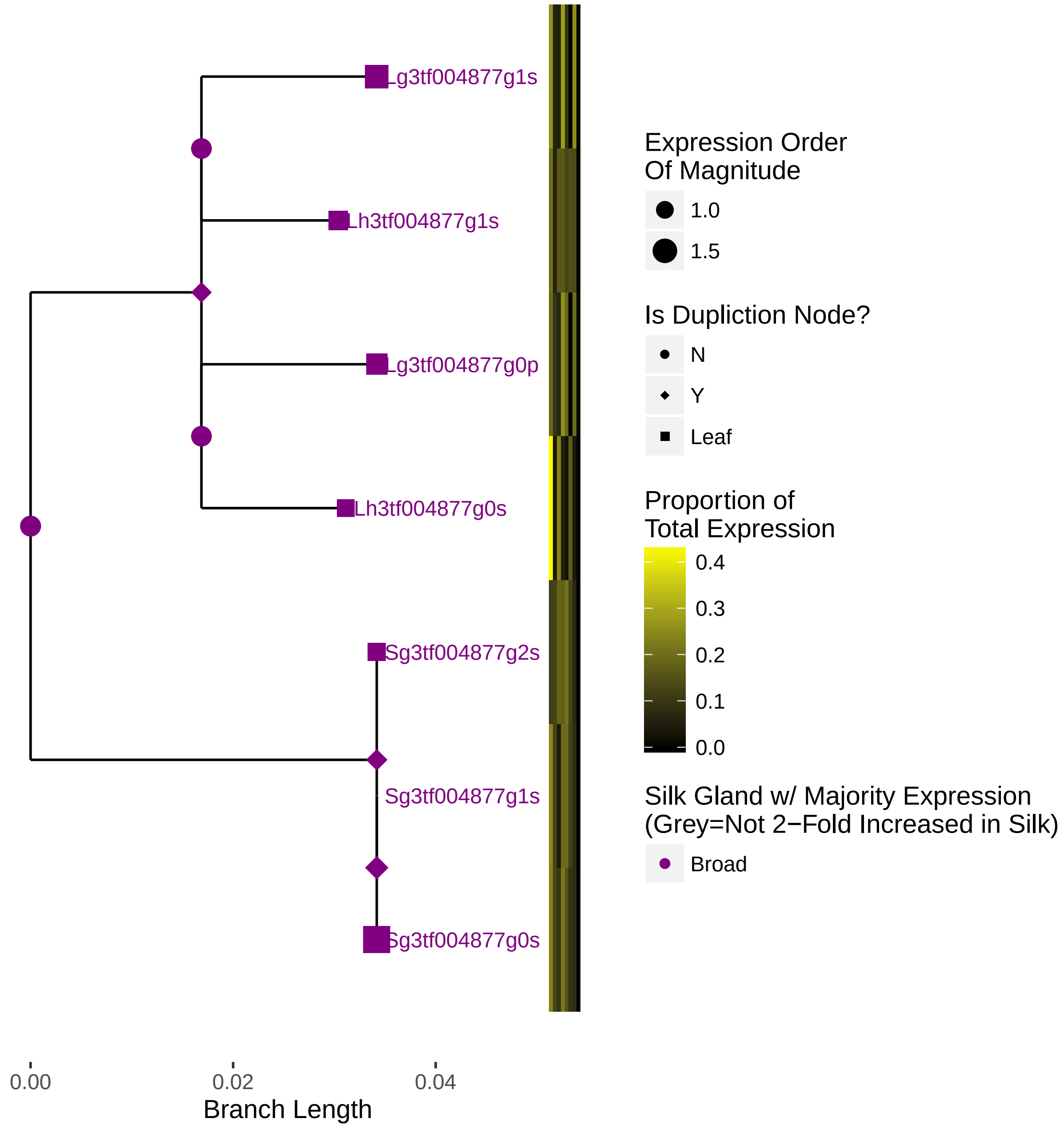

Lg3tf004877g1s
Lh3tf004877g1s
Lg3tf004877g0p
Lh3tf004877g0s
Sg3tf004877g2s
Sg3tf004877g1s
Sg3tf004877g0s
0.00
0.02
0.04
Branch Length
Expression Order Of Magnitude
1.0
1.5
Is Dupliction Node?
N
Y
Leaf
Proportion of Total Expression
0.4
0.3
0.2
0.1
0.0
Silk Gland w/ Majority Expression (Grey=Not 2−Fold Increased in Silk)
Broad

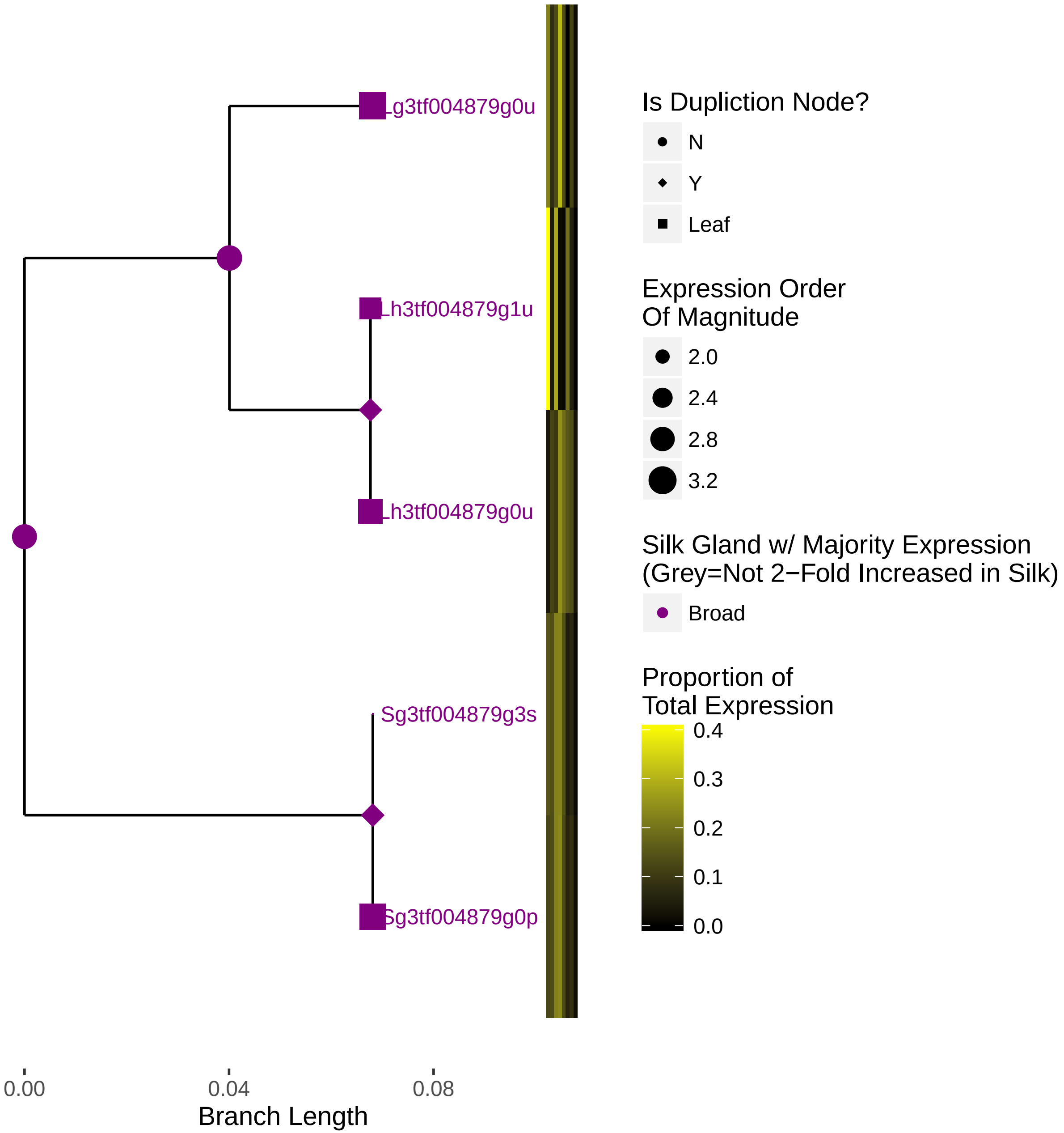

Lg3tf004879g0u
Lh3tf004879g1u
Lh3tf004879g0u
Sg3tf004879g3s
Sg3tf004879g0p
Is Dupliction Node?
N
Y
Leaf
Expression Order
Of Magnitude
2.0
2.4
2.8
3.2
Silk Gland w/ Majority Expression
(Grey=Not 2−Fold Increased in Silk)
Broad
Proportion of
Total Expression
0.4
0.3
0.2
0.1
0.0
0.00
0.04
0.08
Branch Length

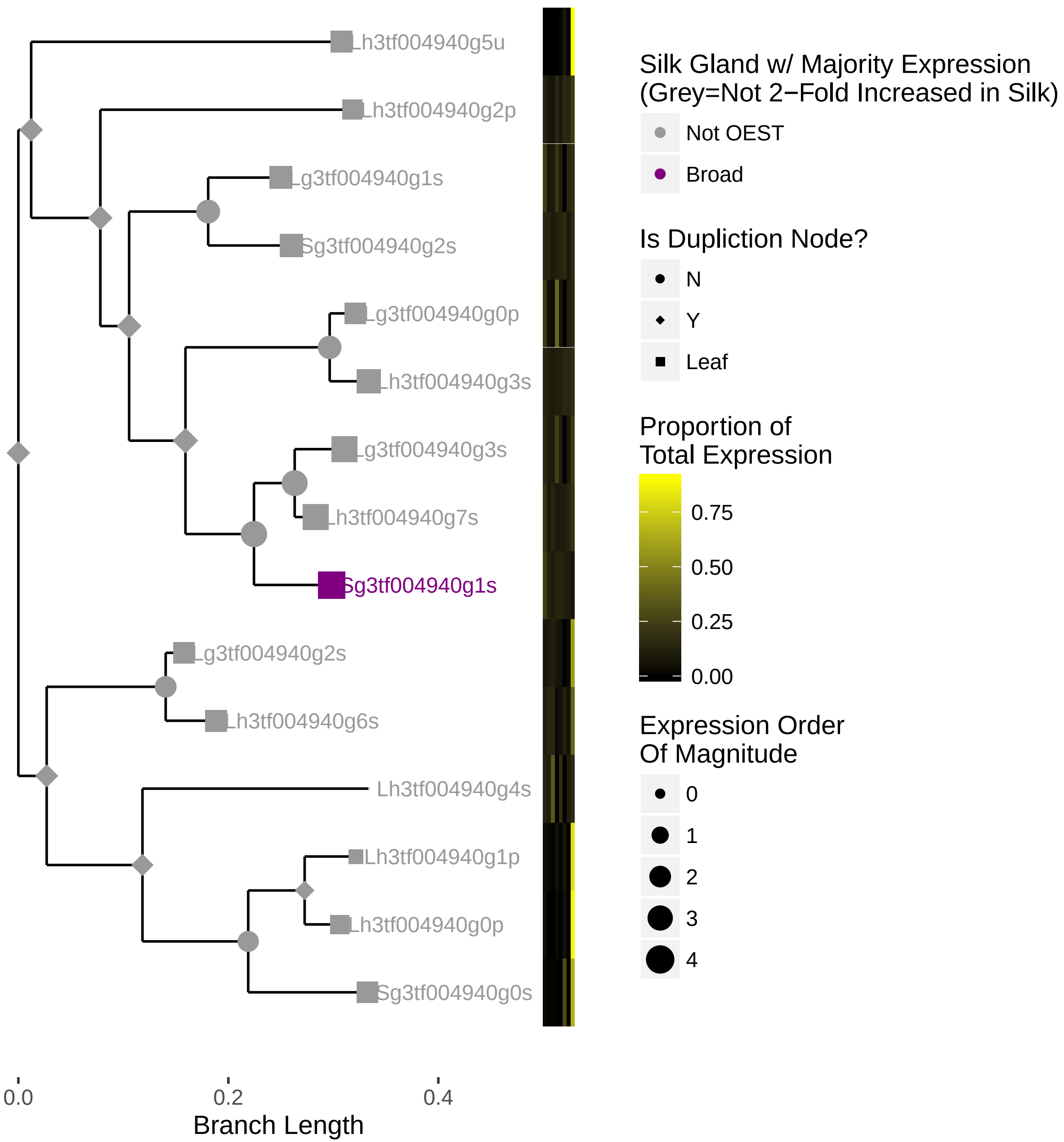

Lh3tf004940g5u
Lh3tf004940g2p
Lg3tf004940g1s
Sg3tf004940g2s
Lg3tf004940g0p
Lh3tf004940g3s
Lg3tf004940g3s
Lh3tf004940g7s
Sg3tf004940g1s
Lg3tf004940g2s
Lh3tf004940g6s
Lh3tf004940g4s
Lh3tf004940g1p
Lh3tf004940g0p
Sg3tf004940g0s
0.0
0.2
0.4
Branch Length
Silk Gland w/ Majority Expression
(Grey=Not 2−Fold Increased in Silk)
Not OEST
Broad
Is Dupliction Node?
N
Y
Leaf
Proportion of
Total Expression
0.75
0.50
0.25
0.00
Expression Order
Of Magnitude
0
1
2
3
4

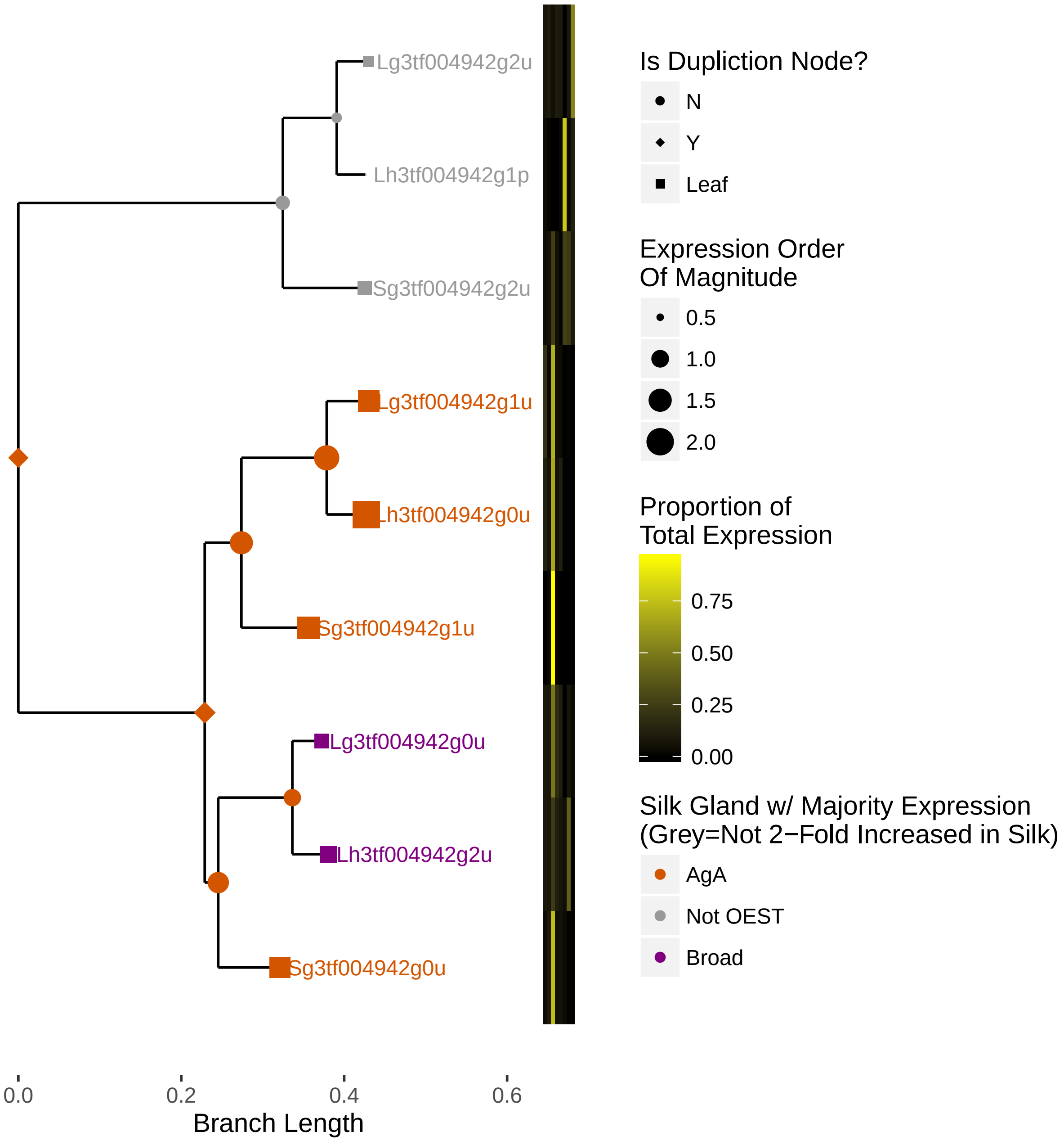

Lg3tf004942g2u
Lh3tf004942g1p
Sg3tf004942g2u
Lg3tf004942g1u
Lh3tf004942g0u
Sg3tf004942g1u
Lg3tf004942g0u
Lh3tf004942g2u
Sg3tf004942g0u
0.0
0.2
0.4
0.6
Branch Length
Is Dupliction Node?
N
Y
Leaf
Expression Order
Of Magnitude
0.5
1.0
1.5
2.0
Proportion of
Total Expression
0.75
0.50
0.25
0.00
Silk Gland w/ Majority Expression
(Grey=Not 2−Fold Increased in Silk)
AgA
Not OEST
Broad

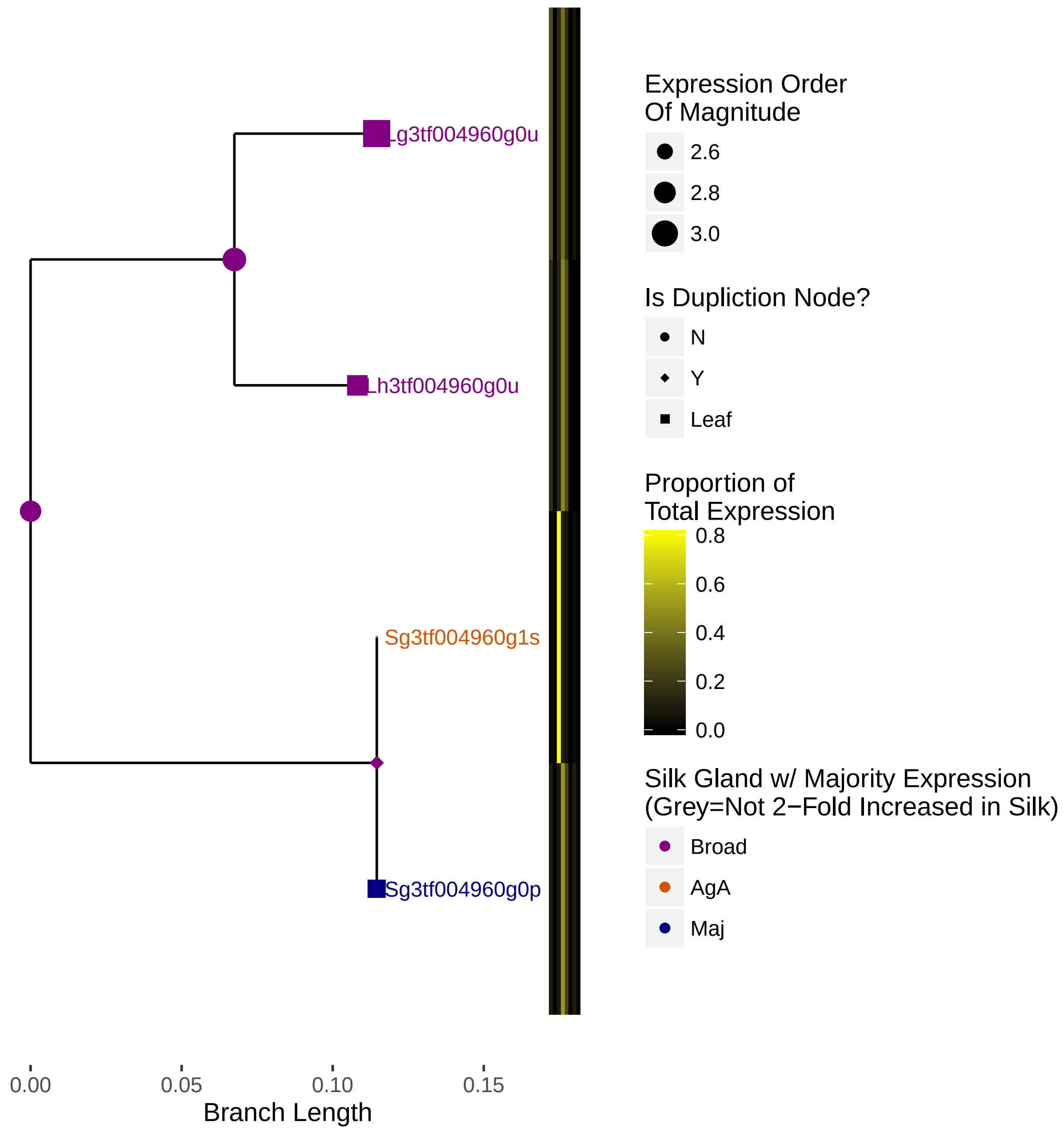

g3tf004960g0u
Lh3tf004960g0u
Sg3tf004960g1s
Sg3tf004960g0p
0.00
0.05
0.10
0.15
Branch Length
Expression Order
Of Magnitude
2.6
2.8
3.0
Is Dupliction Node?
N
Y
Leaf
Proportion of
Total Expression
0.8
0.6
0.4
0.2
0.0
Silk Gland w/ Majority Expression
(Grey=Not 2−Fold Increased in Silk)
Broad
AgA
Maj

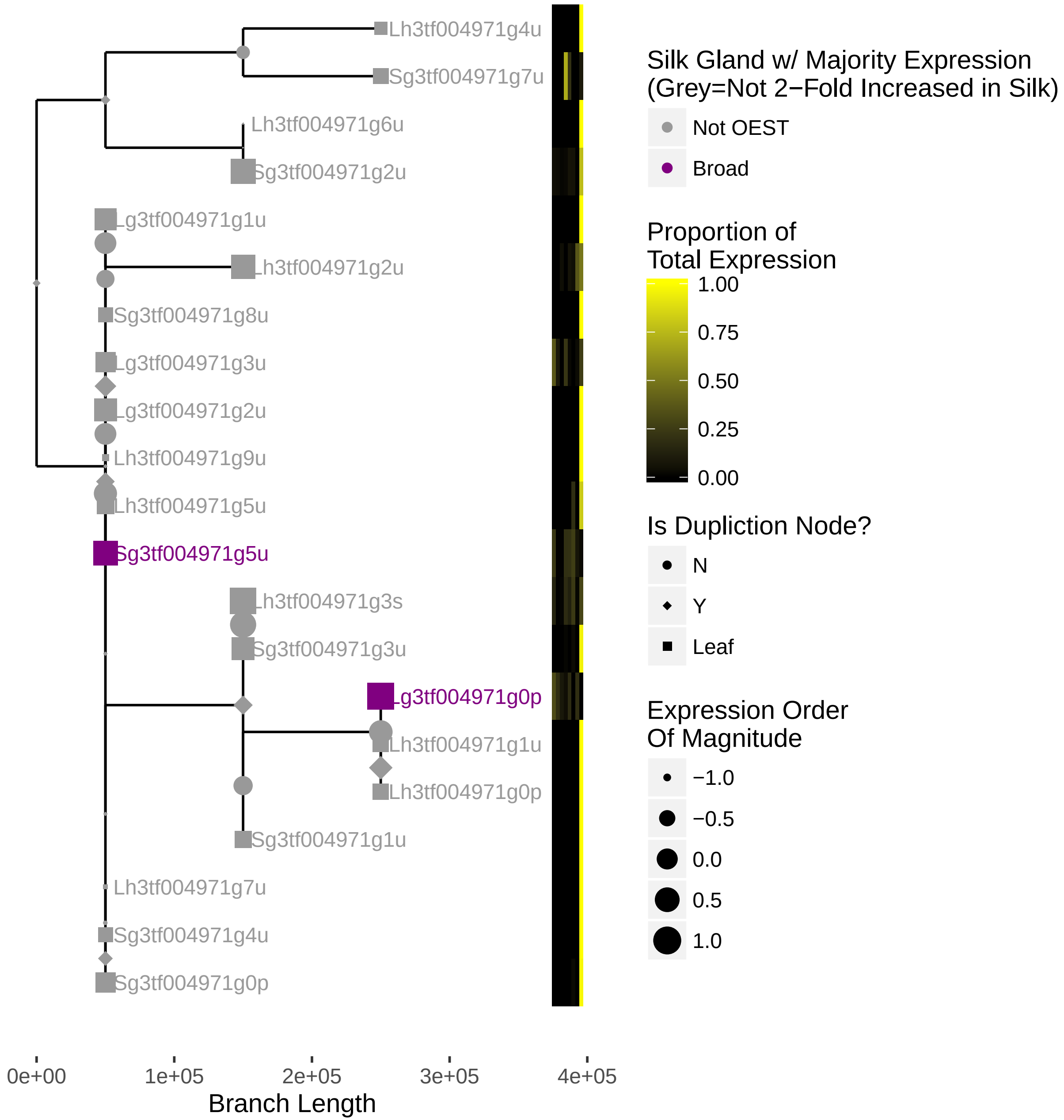

Lh3tf004971g4u
Sg3tf004971g7u
Lh3tf004971g6u
Sg3tf004971g2u
Lg3tf004971g1u
Lh3tf004971g2u
Sg3tf004971g8u
Lg3tf004971g3u
Lg3tf004971g2u
Lh3tf004971g9u
Lh3tf004971g5u
Sg3tf004971g5u
Lh3tf004971g3s
Sg3tf004971g3u
Lg3tf004971g0p
Lh3tf004971g1u
Lh3tf004971g0p
Sg3tf004971g1u
Lh3tf004971g7u
Sg3tf004971g4u
Sg3tf004971g0p
0e+00
1e+05
2e+05
3e+05
4e+05
Branch Length
Silk Gland w/ Majority Expression
(Grey=Not 2−Fold Increased in Silk)
Not OEST
Broad
Proportion of
Total Expression
1.00
0.75
0.50
0.25
0.00
Is Dupliction Node?
N
Y
Leaf
Expression Order
Of Magnitude
−1.0
−0.5
0.0
0.5
1.0

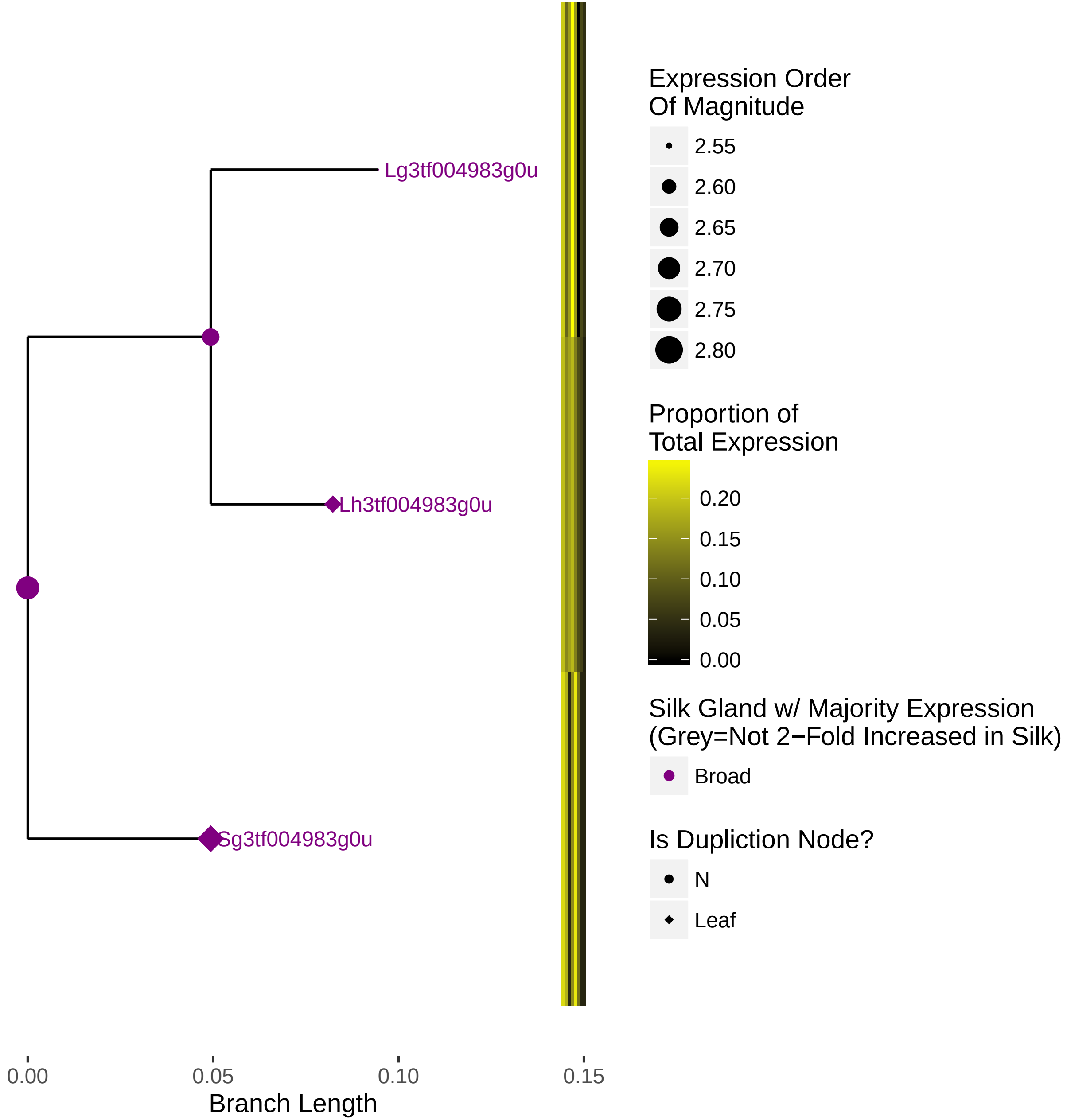

Lg3tf004983g0u
Lh3tf004983g0u
Sg3tf004983g0u
0.00
0.05
0.10
0.15
Branch Length
Expression Order Of Magnitude
2.55
2.60
2.65
2.70
2.75
2.80
Proportion of Total Expression
0.20
0.15
0.10
0.05
0.00
Silk Gland w/ Majority Expression (Grey=Not 2−Fold Increased in Silk)
Broad
Is Dupliction Node?
N
Leaf

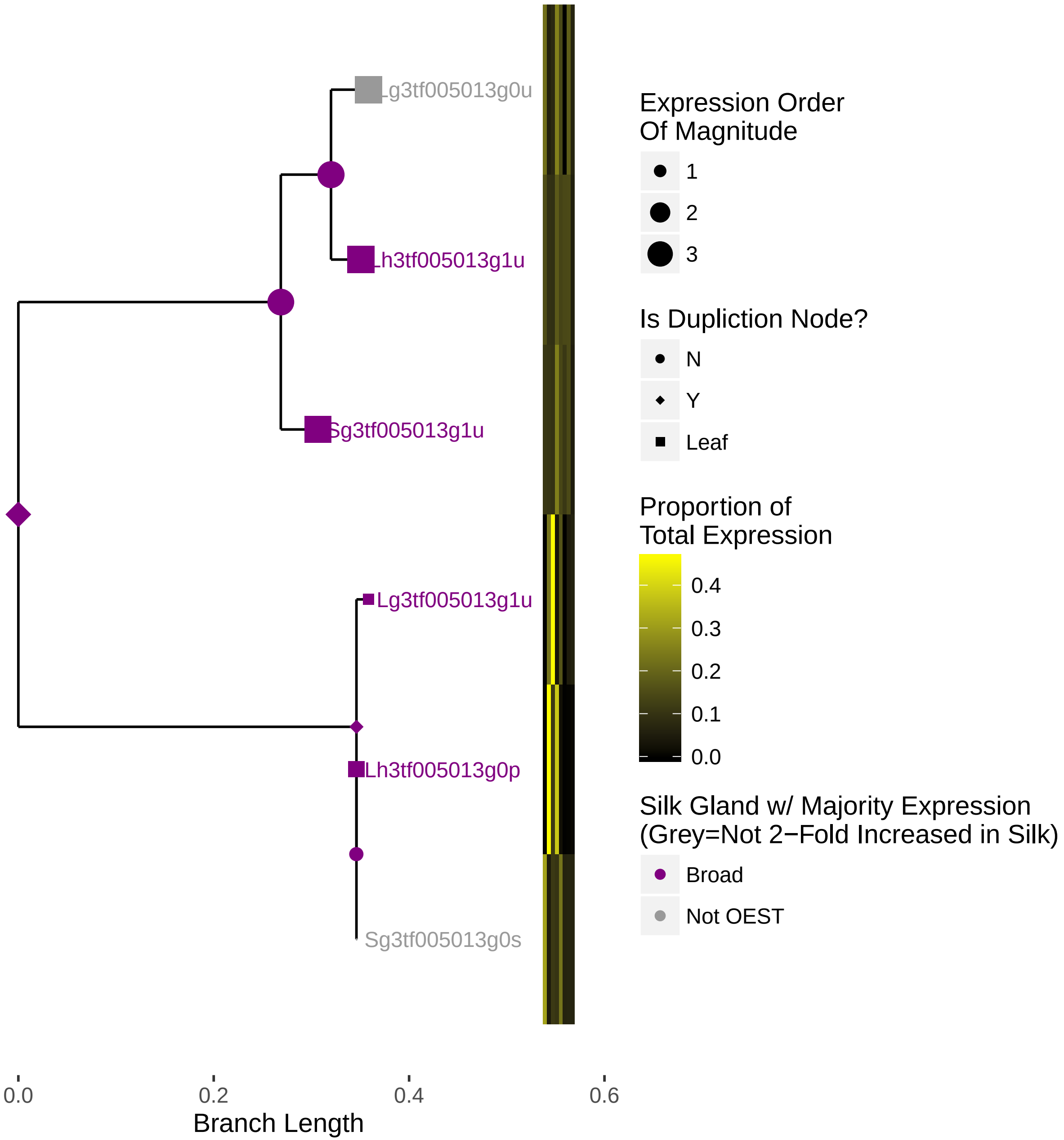

Lg3tf005013g0u
Lh3tf005013g1u
Sg3tf005013g1u
Lg3tf005013g1u
Lh3tf005013g0p
Sg3tf005013g0s
0.0
0.2
0.4
0.6
Branch Length
Expression Order
Of Magnitude
1
2
3
Is Dupliction Node?
N
Y
Leaf
Proportion of
Total Expression
0.4
0.3
0.2
0.1
0.0
Silk Gland w/ Majority Expression
(Grey=Not 2−Fold Increased in Silk)
Broad
Not OEST

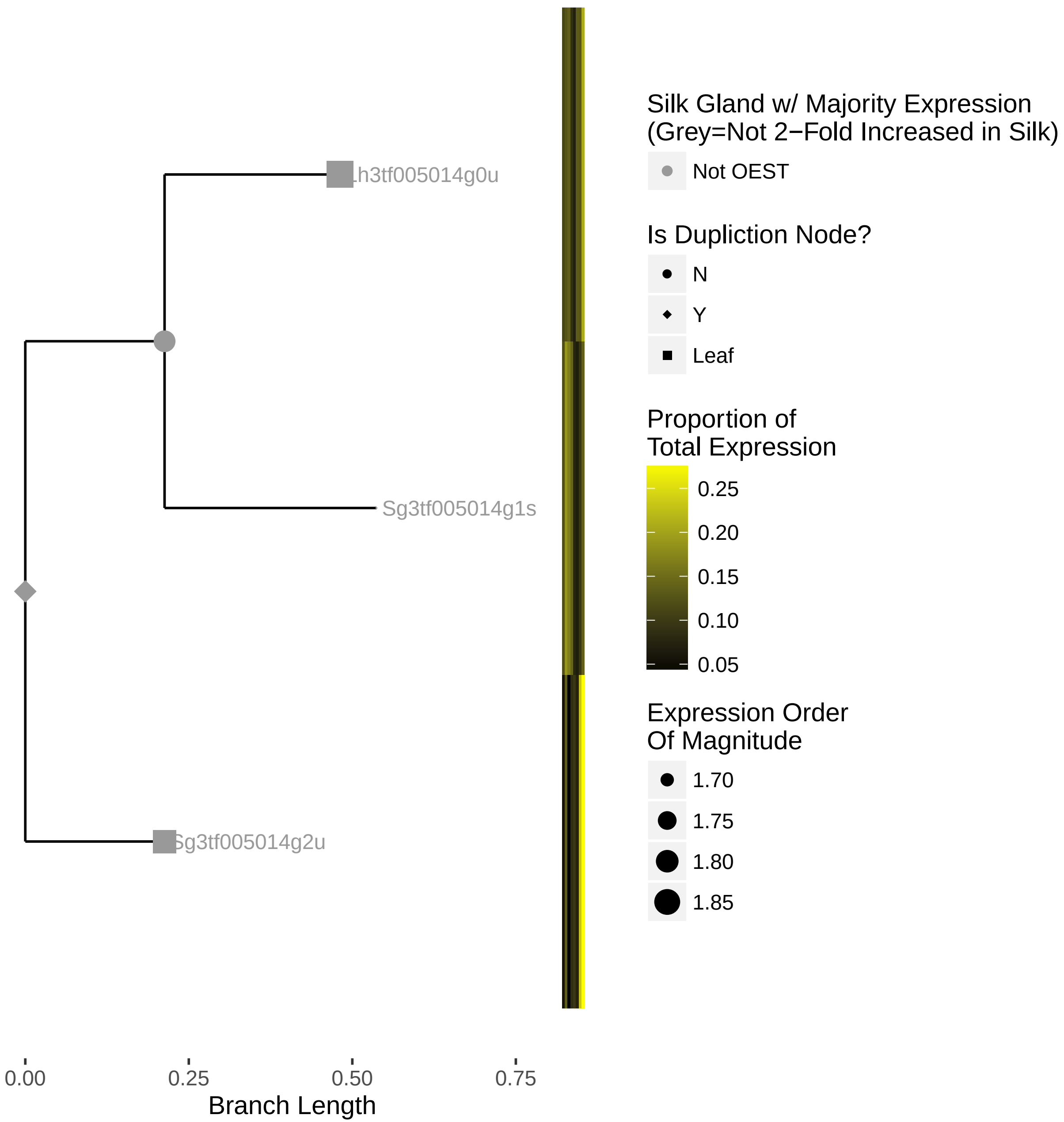
h3tf005014g0u
Sg3tf005014g1s
Sg3tf005014g2u
0.00
0.25
0.50
0.75
Branch Length
Silk Gland w/ Majority Expression
(Grey=Not 2−Fold Increased in Silk)
Not OEST
Is Dupliction Node?
N
Y
Leaf
Proportion of
Total Expression
0.25
0.20
0.15
0.10
0.05
Expression Order
Of Magnitude
1.70
1.75
1.80
1.85

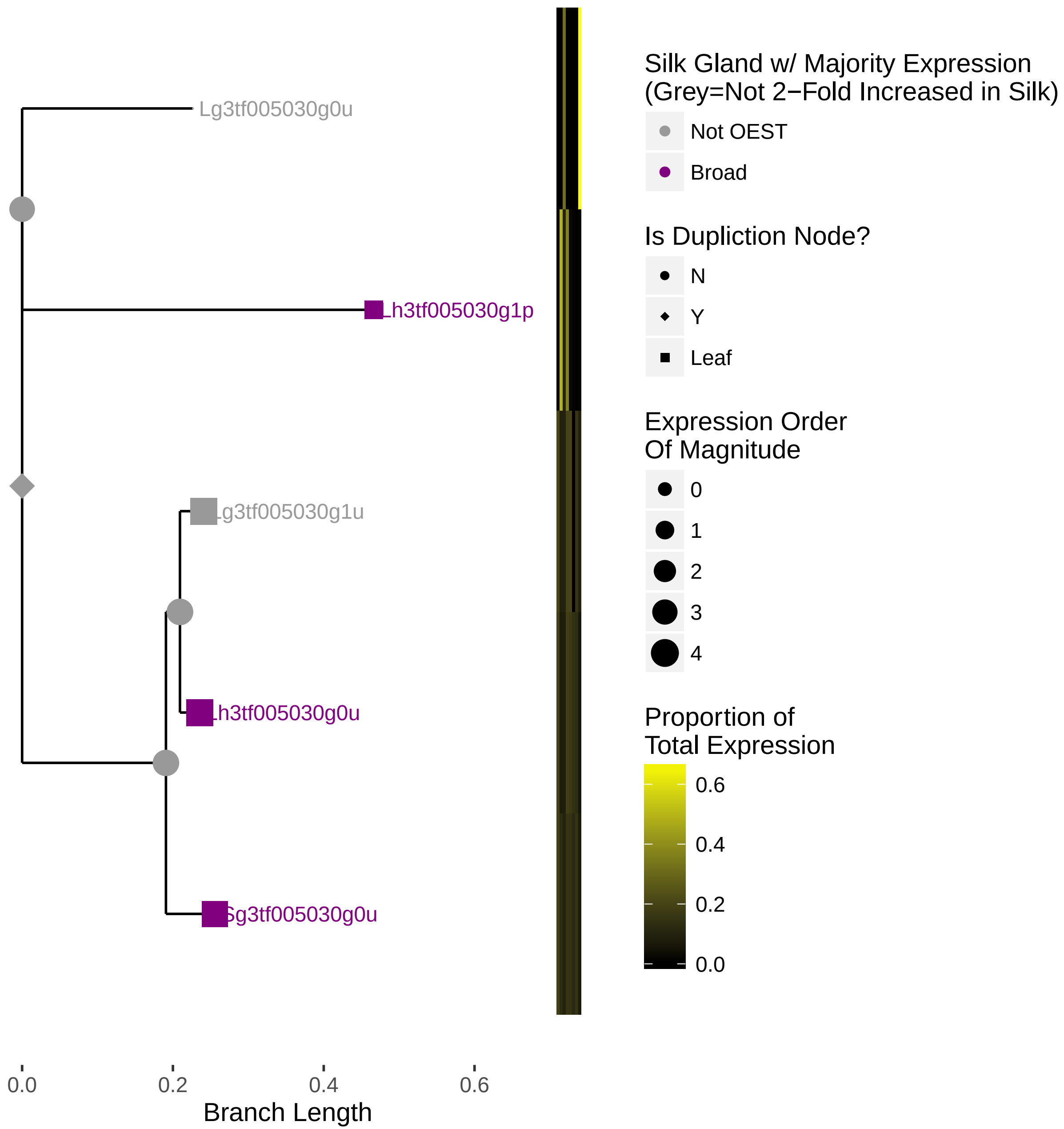

Lg3tf005030g0u
Lh3tf005030g1p
Lg3tf005030g1u
Lh3tf005030g0u
Sg3tf005030g0u
0.0
0.2
0.4
0.6
Branch Length
Silk Gland w/ Majority Expression
(Grey=Not 2−Fold Increased in Silk)
Not OEST
Broad
Is Dupliction Node?
N
Y
Leaf
Expression Order
Of Magnitude
0
1
2
3
4
Proportion of
Total Expression
0.6
0.4
0.2
0.0

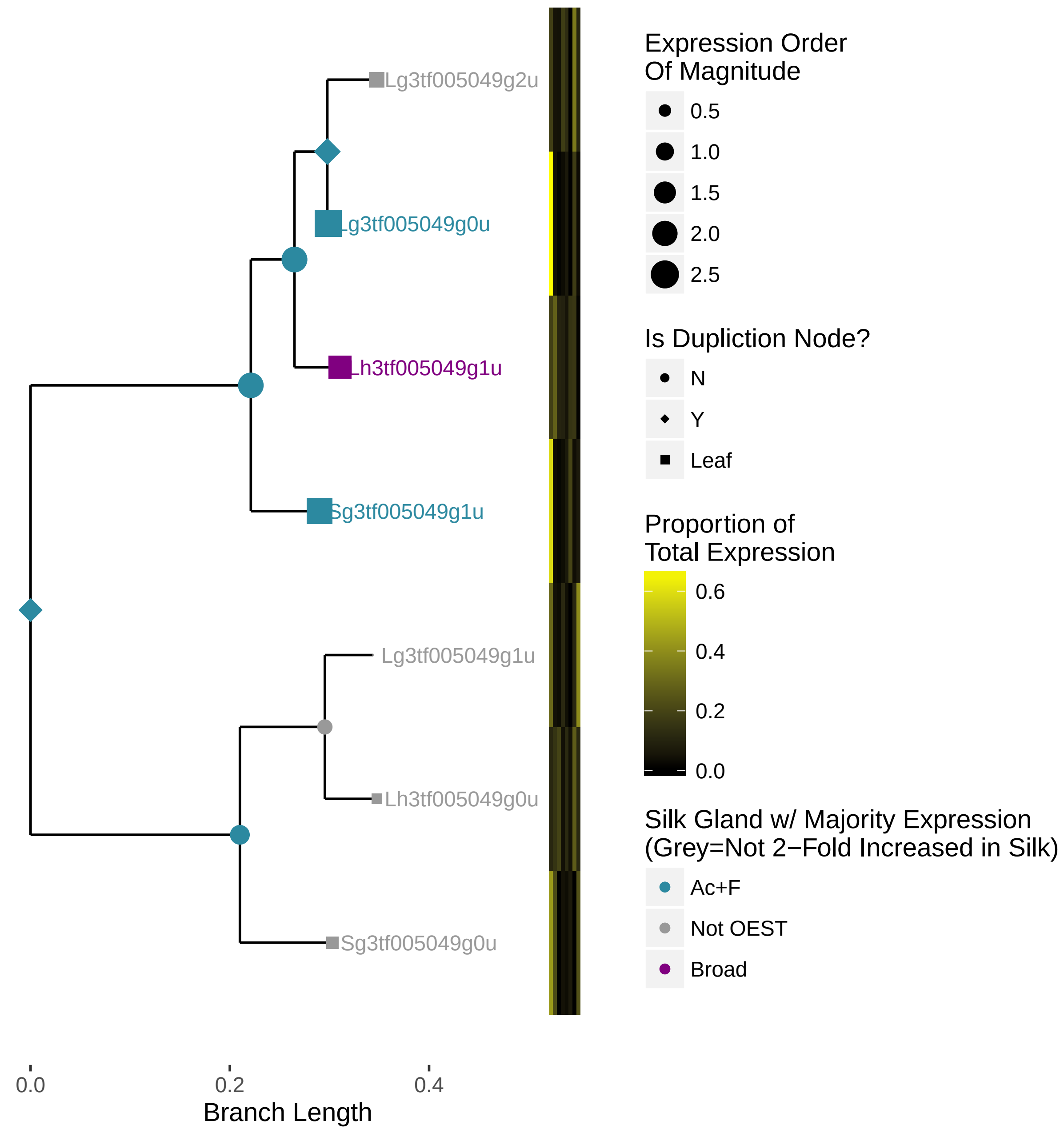

Lg3tf005049g2u
Lg3tf005049g0u
Lh3tf005049g1u
Sg3tf005049g1u
Lg3tf005049g1u
Lh3tf005049g0u
Sg3tf005049g0u
0.0
0.2
0.4
Branch Length
Expression Order
Of Magnitude
0.5
1.0
1.5
2.0
2.5
Is Dupliction Node?
N
Y
Leaf
Proportion of
Total Expression
0.6
0.4
0.2
0.0
Silk Gland w/ Majority Expression
(Grey=Not 2−Fold Increased in Silk)
Ac+F
Not OEST
Broad

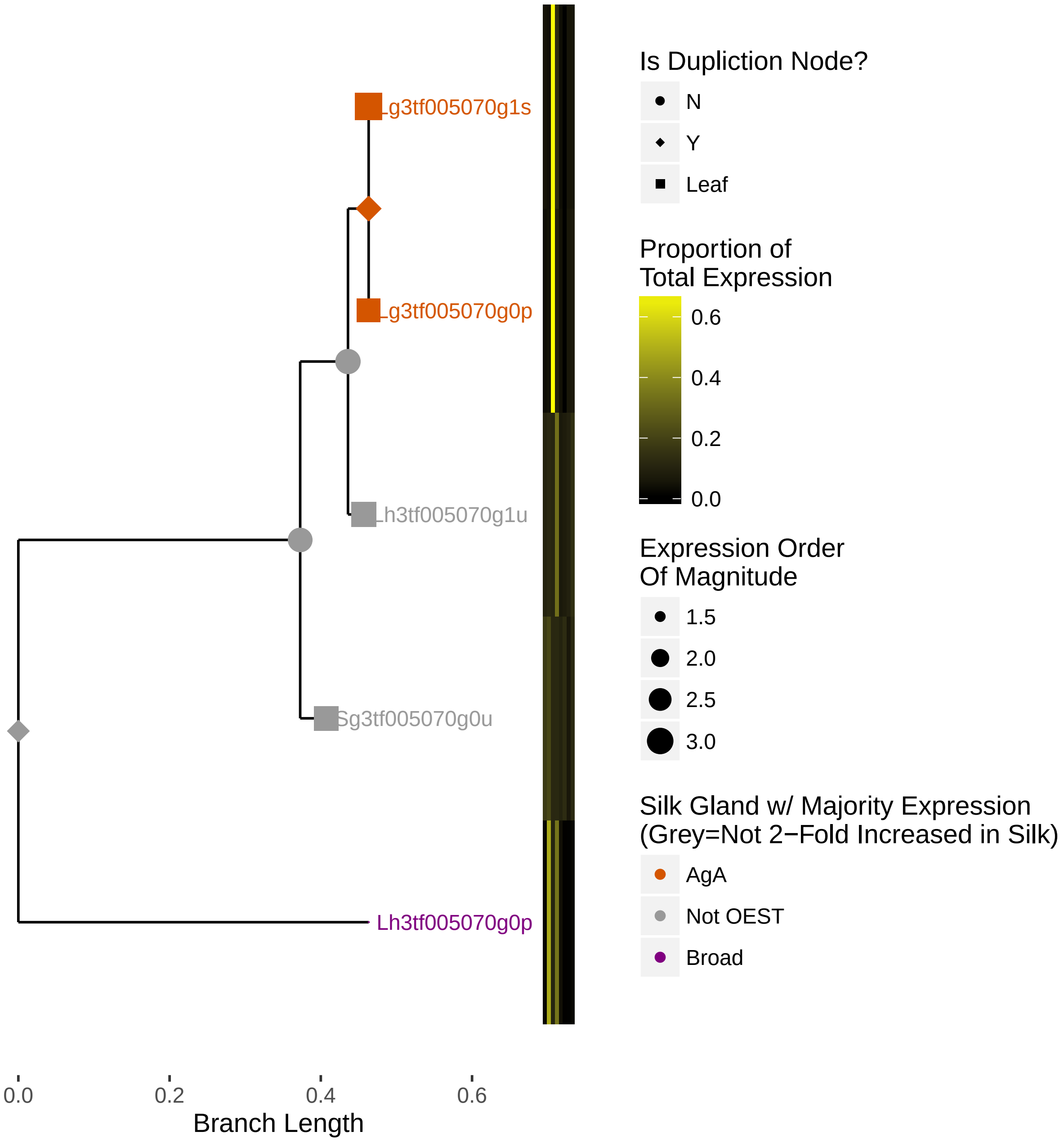

Lg3tf005070g1s
Lg3tf005070g0p
Lh3tf005070g1u
Sg3tf005070g0u
Lh3tf005070g0p
0.0
0.2
0.4
0.6
Branch Length
Is Dupliction Node?
N
Y
Leaf
Proportion of
Total Expression
0.6
0.4
0.2
0.0
Expression Order
Of Magnitude
1.5
2.0
2.5
3.0
Silk Gland w/ Majority Expression
(Grey=Not 2−Fold Increased in Silk)
AgA
Not OEST
Broad

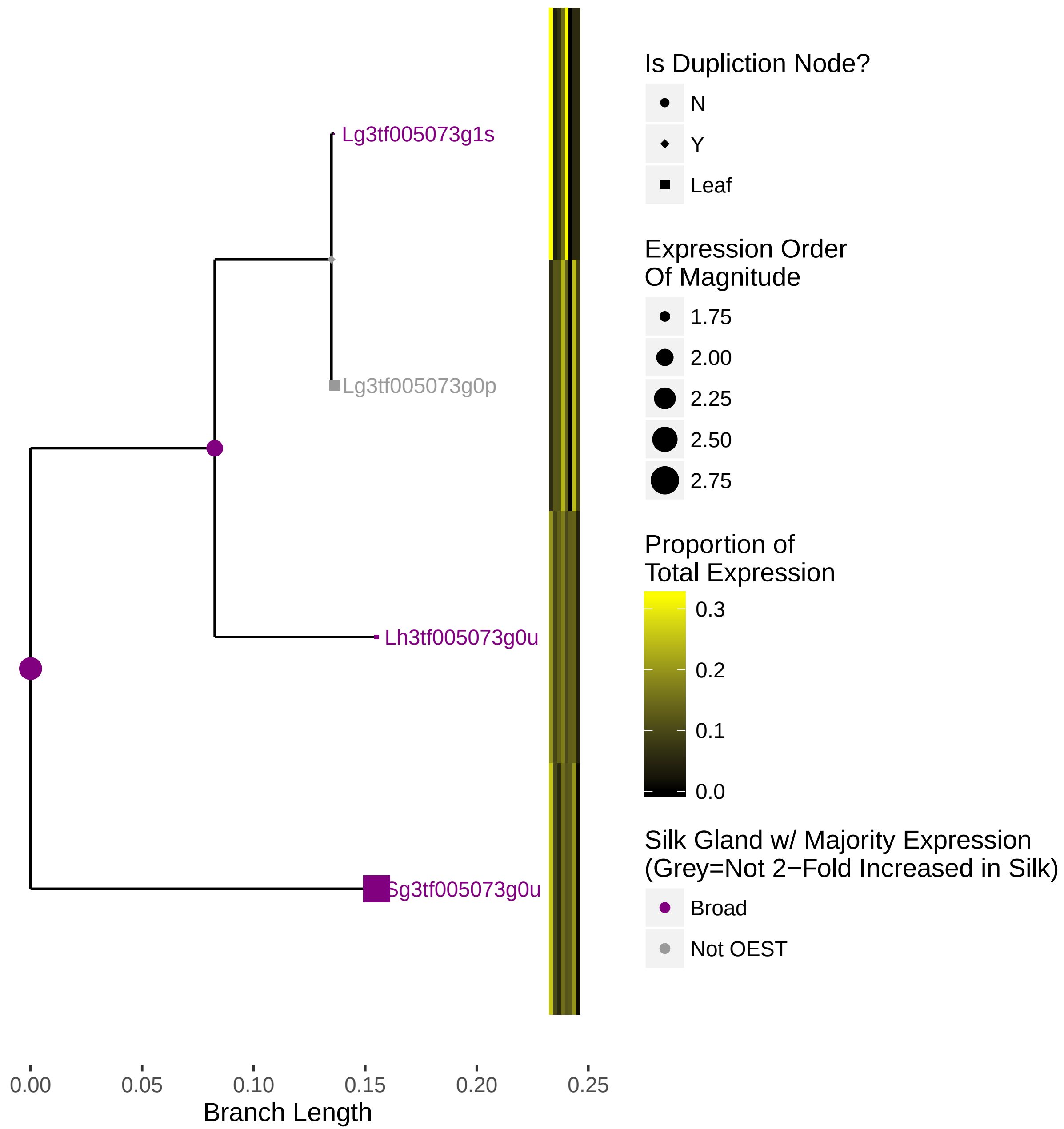

Lg3tf005073g1s
Lg3tf005073g0p
Lh3tf005073g0u
Sg3tf005073g0u
0.00
0.05
0.10
0.15
0.20
0.25
Branch Length
Is Dupliction Node?
N
Y
Leaf
Expression Order
Of Magnitude
1.75
2.00
2.25
2.50
2.75
Proportion of
Total Expression
0.3
0.2
0.1
0.0
Silk Gland w/ Majority Expression
(Grey=Not 2−Fold Increased in Silk)
Broad
Not OEST

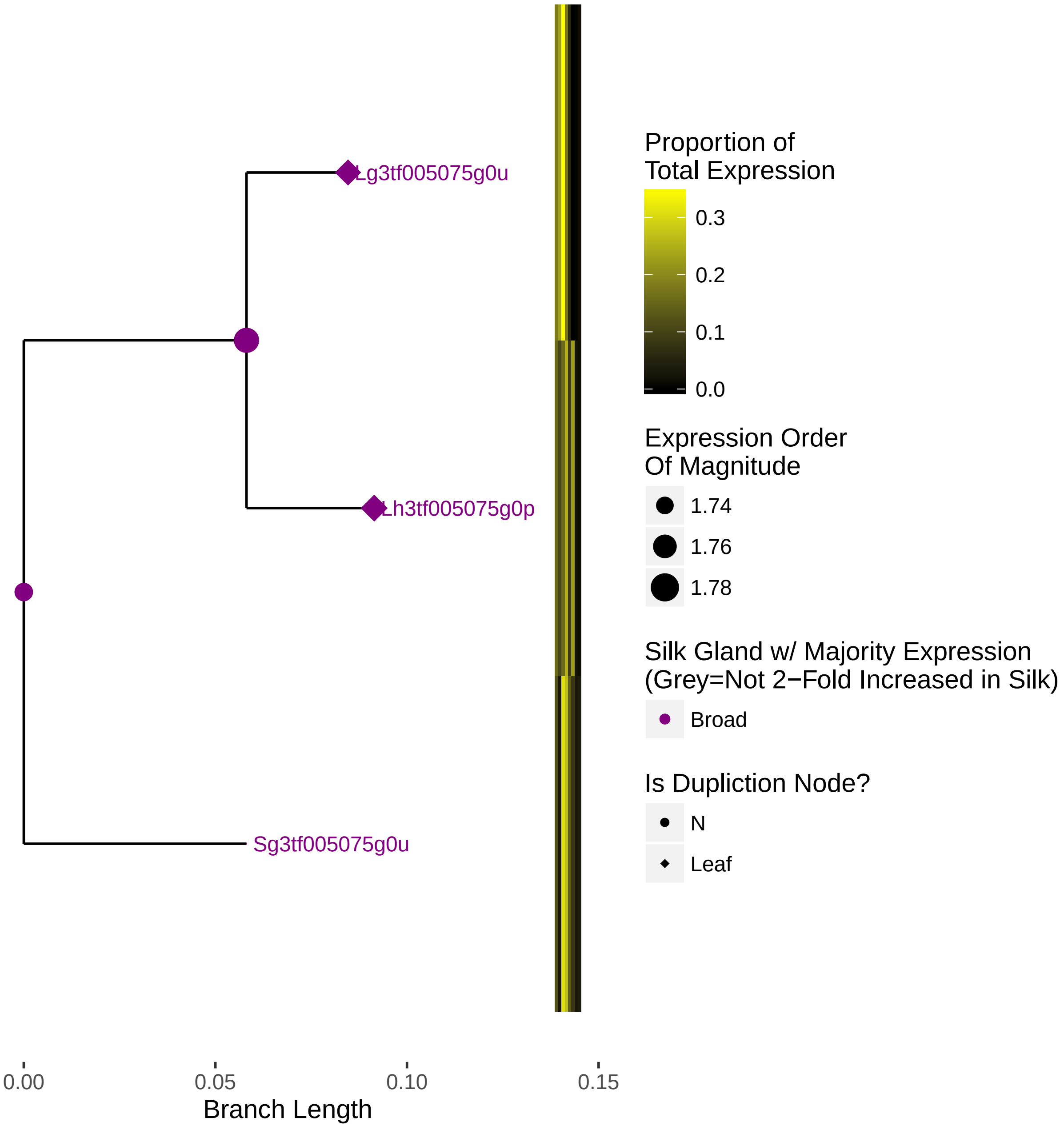

Lg3tf005075g0u
Lh3tf005075g0p
Sg3tf005075g0u
0.00
0.05
0.10
0.15
Branch Length
Proportion of
Total Expression
0.3
0.2
0.1
0.0
Expression Order
Of Magnitude
1.74
1.76
1.78
Silk Gland w/ Majority Expression
(Grey=Not 2−Fold Increased in Silk)
Broad
Is Dupliction Node?
N
Leaf

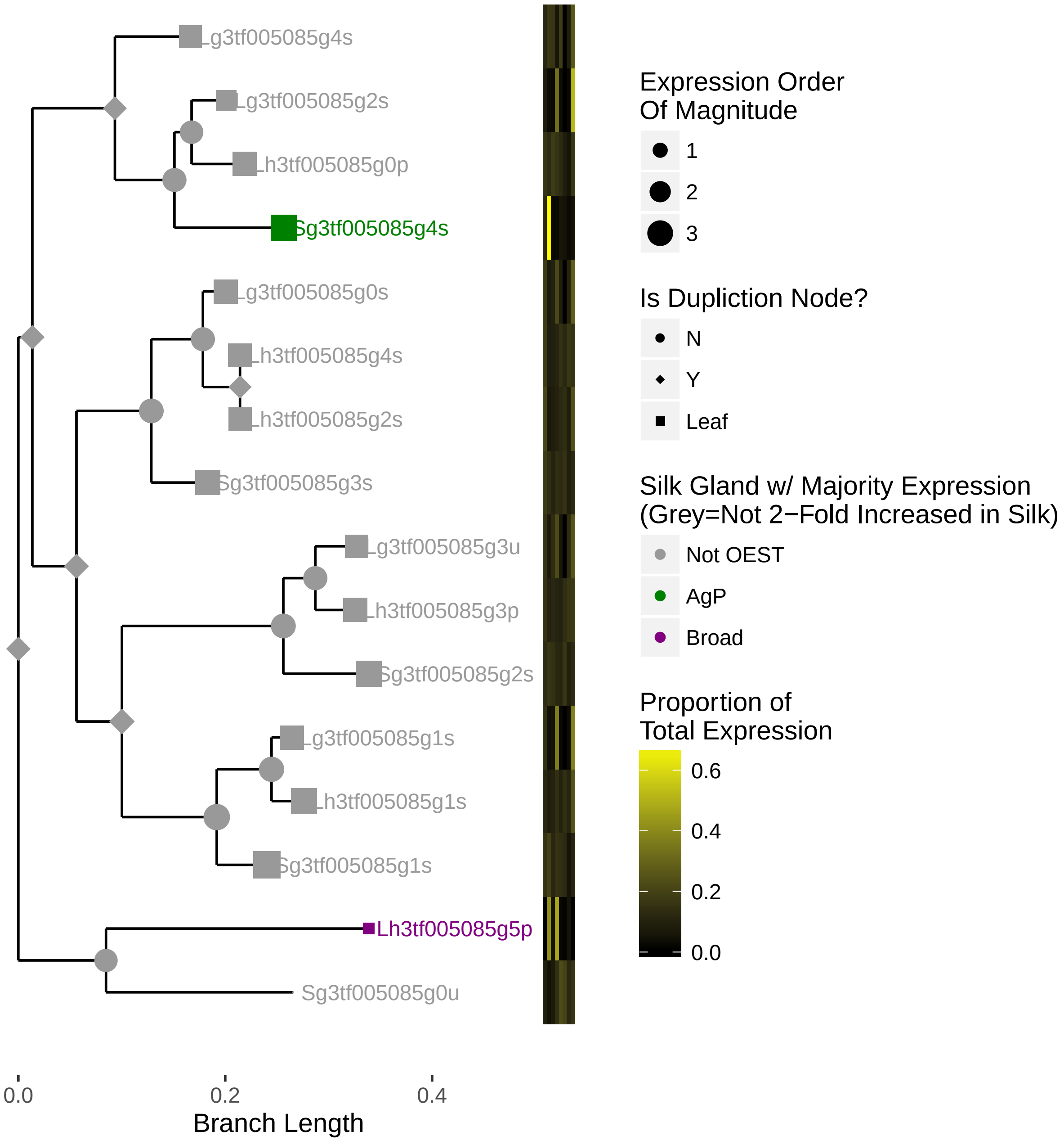

Lg3tf005085g4s
Lg3tf005085g2s
Lh3tf005085g0p
Sg3tf005085g4s
Lg3tf005085g0s
Lh3tf005085g4s
Lh3tf005085g2s
Sg3tf005085g3s
Lg3tf005085g3u
Lh3tf005085g3p
Sg3tf005085g2s
Lg3tf005085g1s
Lh3tf005085g1s
Sg3tf005085g1s
Lh3tf005085g5p
Sg3tf005085g0u
0.0
0.2
0.4
Branch Length
Expression Order Of Magnitude
1
2
3
Is Dupliction Node?
N
Y
Leaf
Silk Gland w/ Majority Expression (Grey=Not 2−Fold Increased in Silk)
Not OEST
AgP
Broad
Proportion of Total Expression
0.6
0.4
0.2
0.0

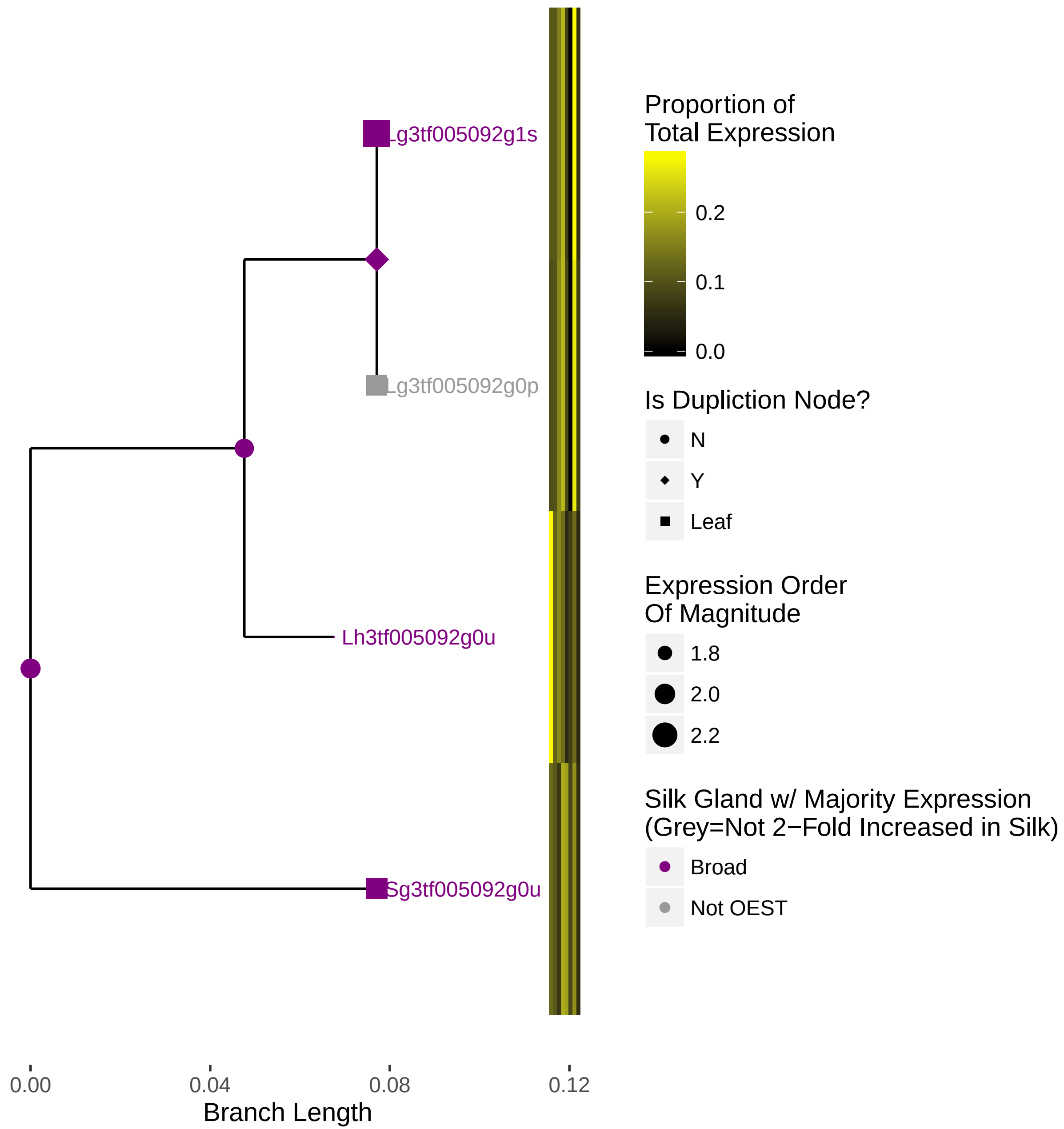

Lg3tf005092g1s
Lg3tf005092g0p
Lh3tf005092g0u
Sg3tf005092g0u
0.00
0.04
0.08
0.12
Branch Length
Proportion of
Total Expression
0.2
0.1
0.0
Is Dupliction Node?
N
Y
Leaf
Expression Order
Of Magnitude
1.8
2.0
2.2
Silk Gland w/ Majority Expression
(Grey=Not 2−Fold Increased in Silk)
Broad
Not OEST

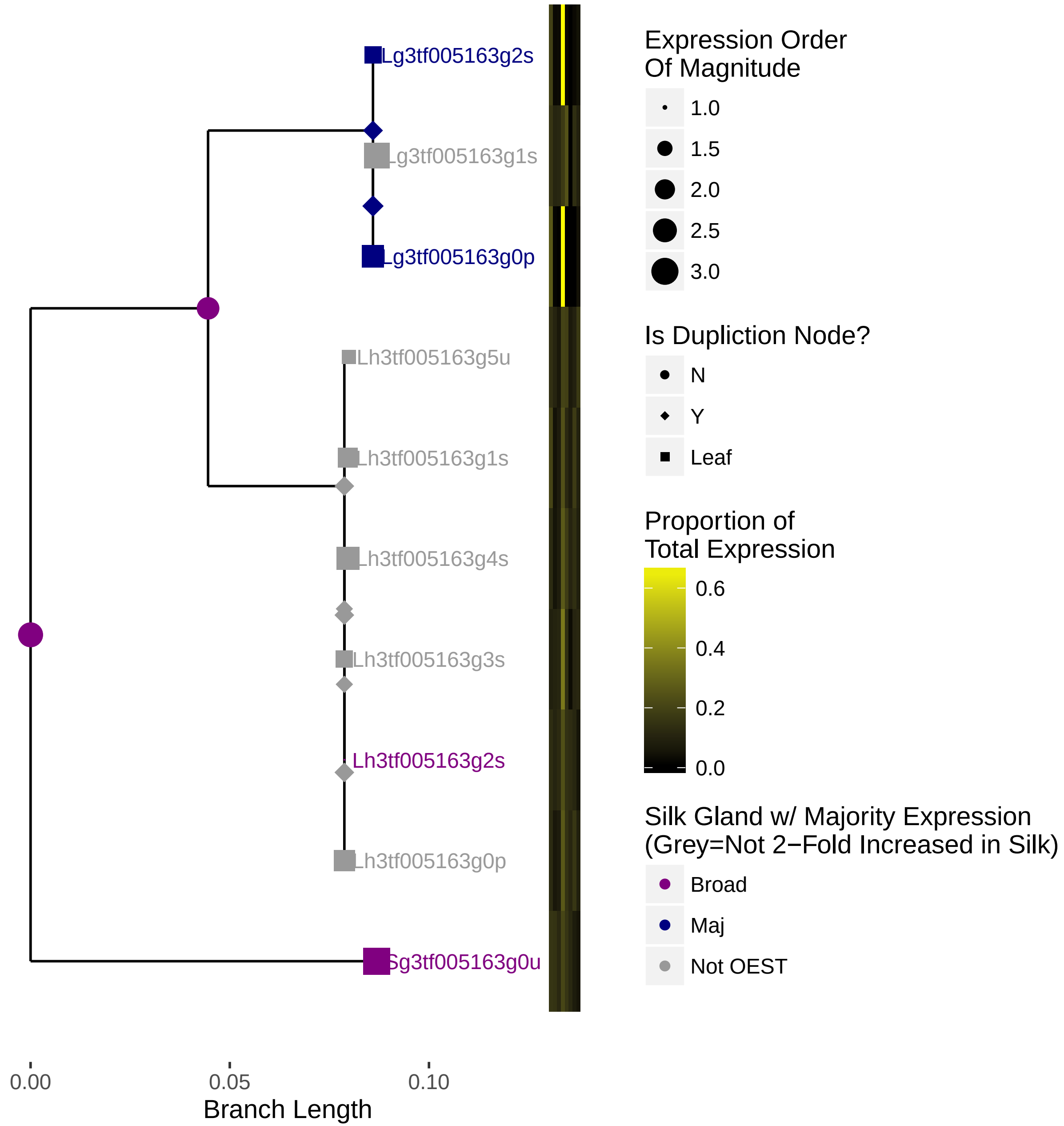

Lg3tf005163g2s
Lg3tf005163g1s
Lg3tf005163g0p
Lh3tf005163g5u
Lh3tf005163g1s
Lh3tf005163g4s
Lh3tf005163g3s
Lh3tf005163g2s
Lh3tf005163g0p
Sg3tf005163g0u
0.00
0.05
0.10
Branch Length
Expression Order Of Magnitude
1.0
1.5
2.0
2.5
3.0
Is Dupliction Node?
N
Y
Leaf
Proportion of Total Expression
0.6
0.4
0.2
0.0
Silk Gland w/ Majority Expression (Grey=Not 2−Fold Increased in Silk)
Broad
Maj
Not OEST

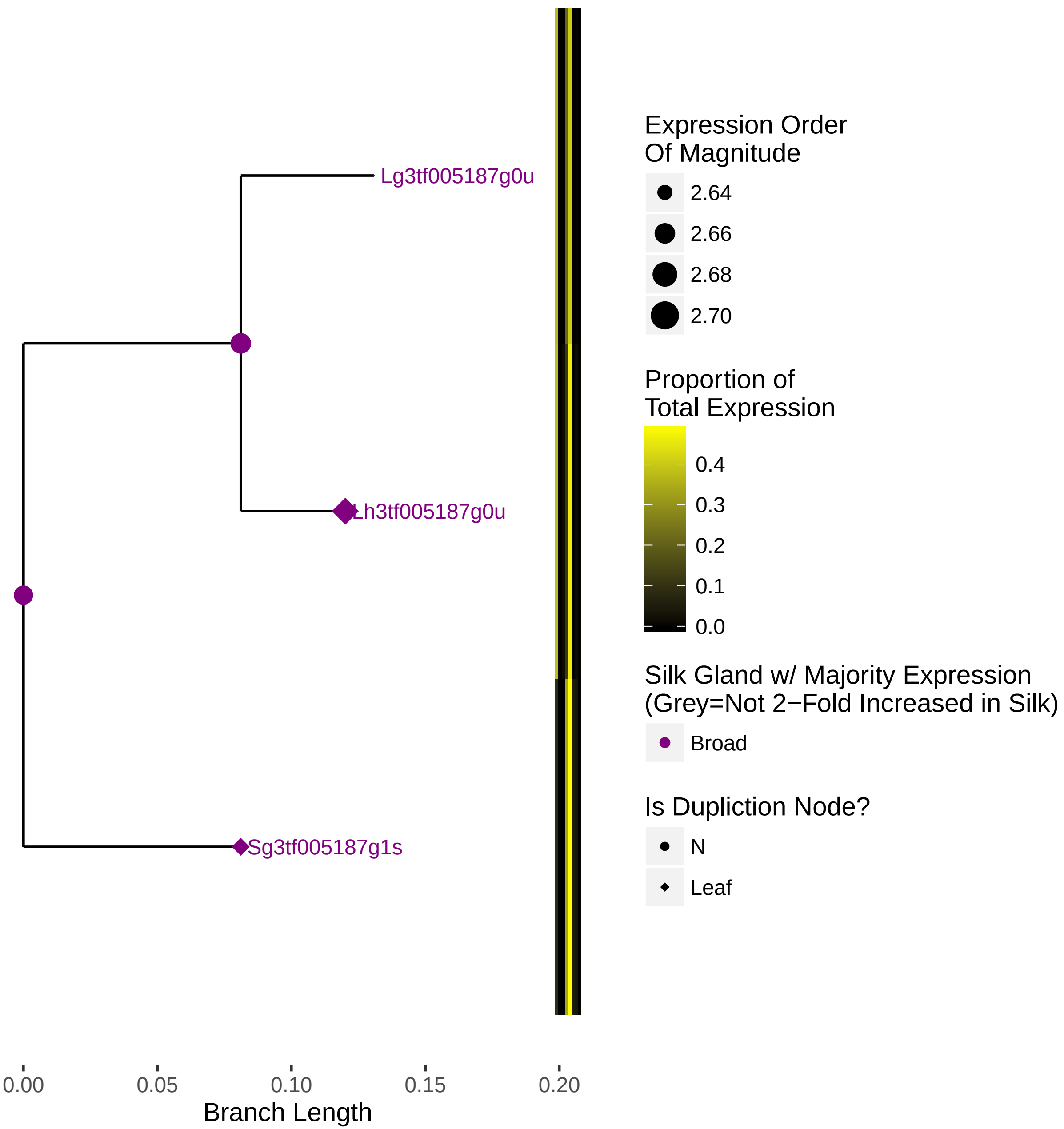

Lg3tf005187g0u
Lh3tf005187g0u
Sg3tf005187g1s
0.00
0.05
0.10
0.15
0.20
Branch Length
Expression Order
Of Magnitude
2.64
2.66
2.68
2.70
Proportion of
Total Expression
0.4
0.3
0.2
0.1
0.0
Silk Gland w/ Majority Expression
(Grey=Not 2−Fold Increased in Silk)
Broad
Is Dupliction Node?
N
Leaf

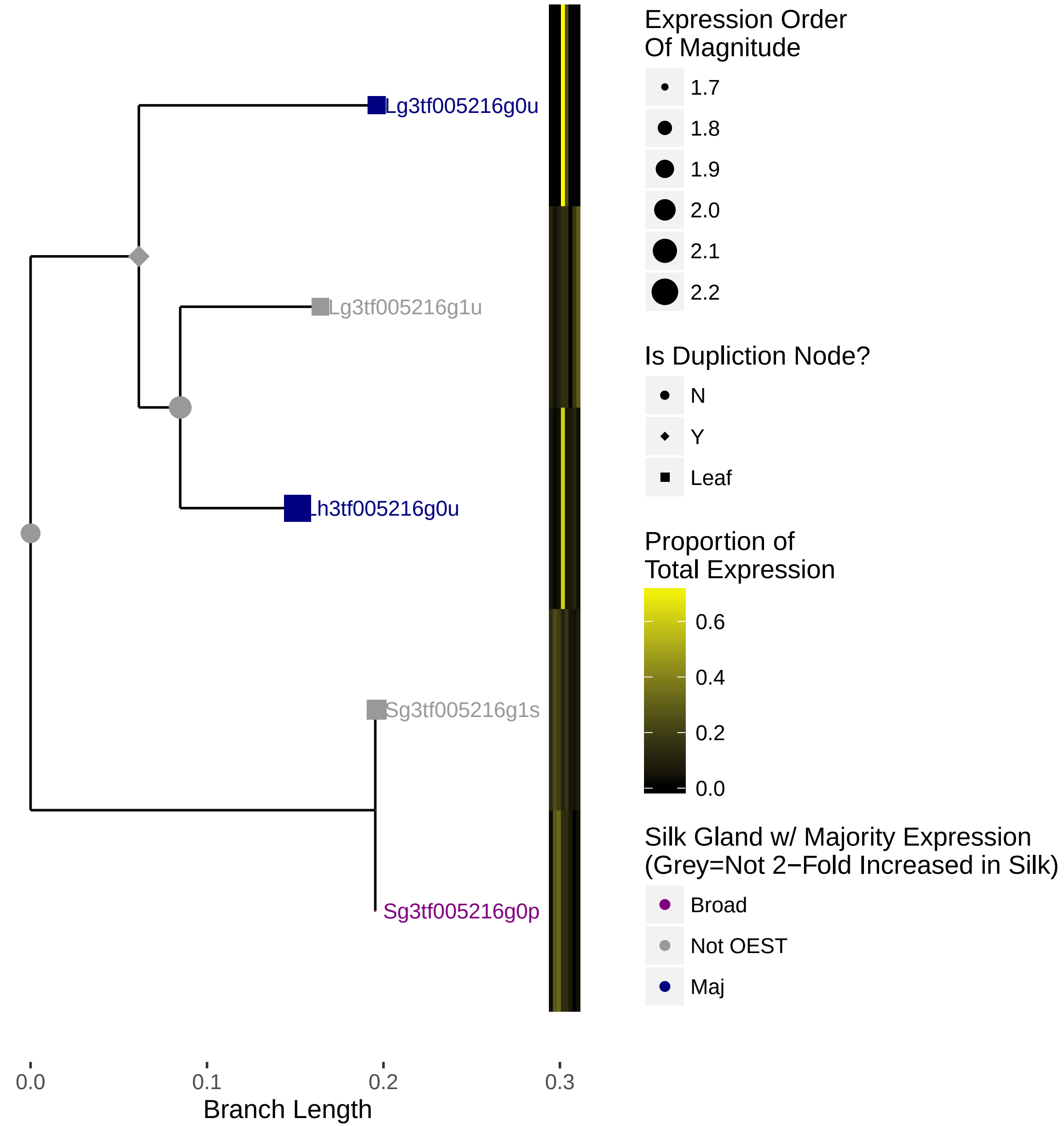
Lg3tf005216g0u
Lg3tf005216g1u
Lh3tf005216g0u
Sg3tf005216g1s
Sg3tf005216g0p
0.0
0.1
0.2
0.3
Branch Length
Expression Order
Of Magnitude
1.7
1.8
1.9
2.0
2.1
2.2
Is Dupliction Node?
N
Y
Leaf
Proportion of
Total Expression
0.6
0.4
0.2
0.0
Silk Gland w/ Majority Expression
(Grey=Not 2−Fold Increased in Silk)
Broad
Not OEST
Maj

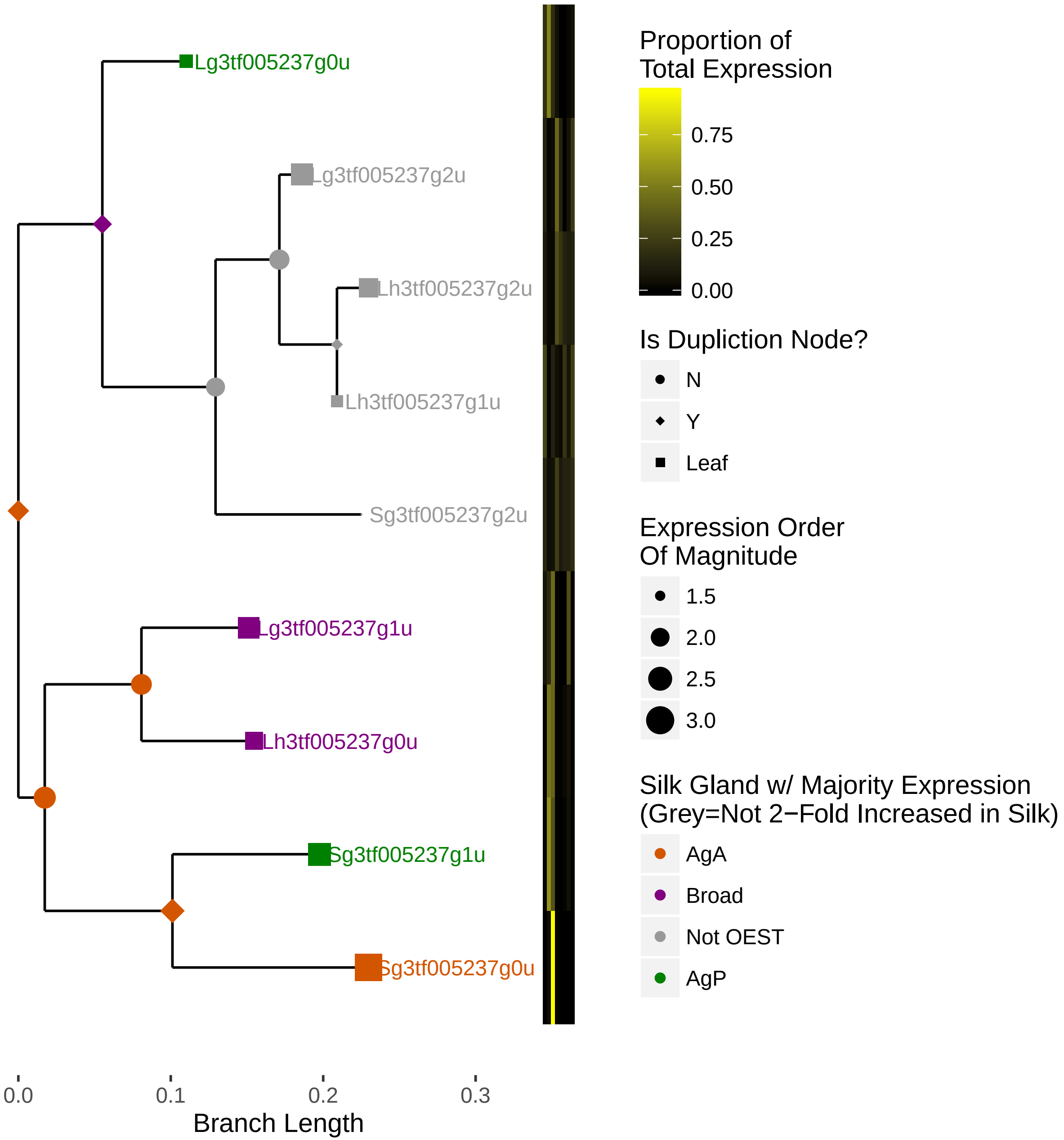

Lg3tf005237g0u
Lg3tf005237g2u
Lh3tf005237g2u
Lh3tf005237g1u
Sg3tf005237g2u
Lg3tf005237g1u
Lh3tf005237g0u
Sg3tf005237g1u
Sg3tf005237g0u
0.0
0.1
0.2
0.3
Branch Length
Proportion of Total Expression
0.75
0.50
0.25
0.00
Is Dupliction Node?
N
Y
Leaf
Expression Order Of Magnitude
1.5
2.0
2.5
3.0
Silk Gland w/ Majority Expression (Grey=Not 2−Fold Increased in Silk)
AgA
Broad
Not OEST
AgP

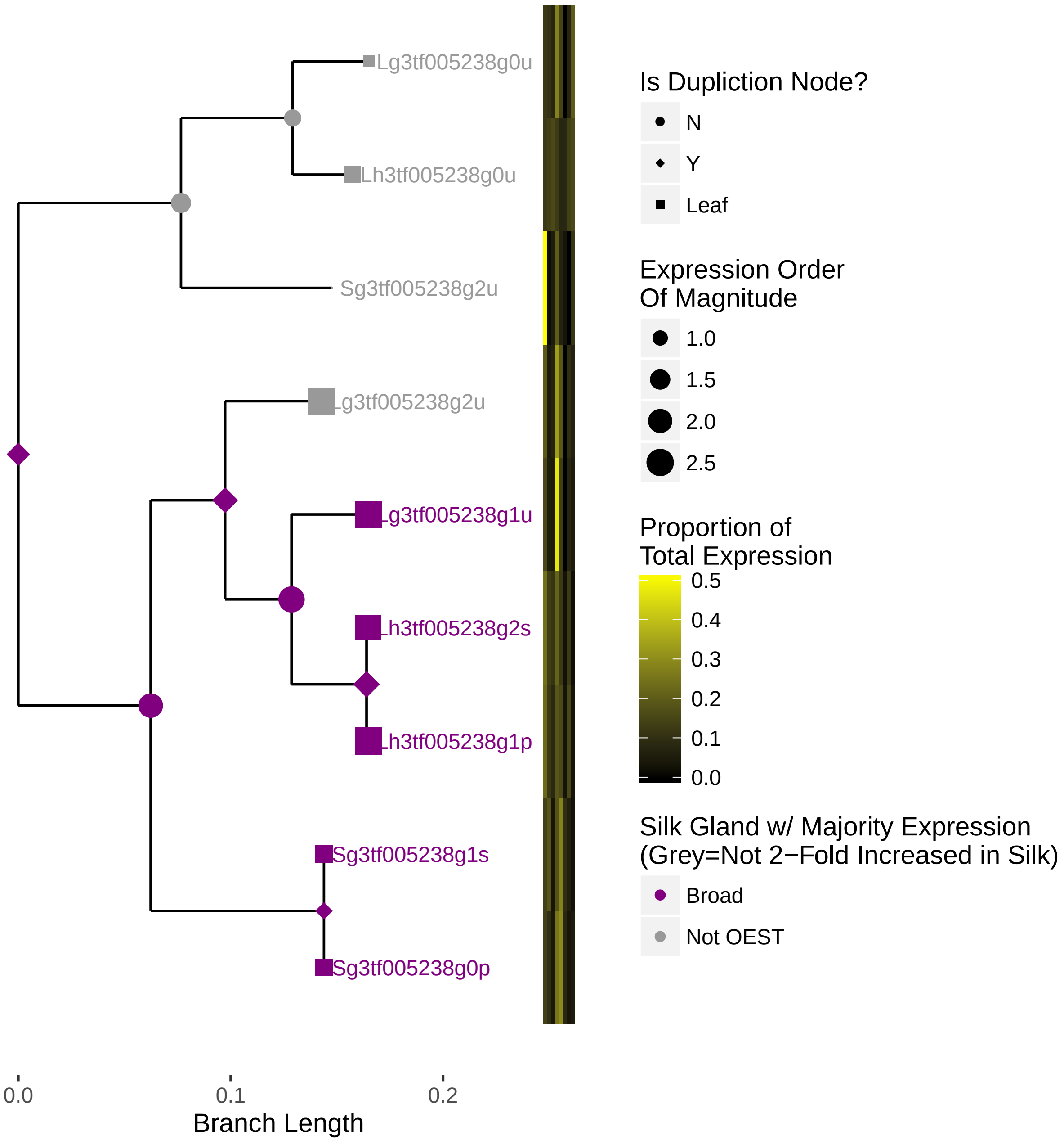
Lg3tf005238g0u
Lh3tf005238g0u
Sg3tf005238g2u
Lg3tf005238g2u
Lg3tf005238g1u
Lh3tf005238g2s
Lh3tf005238g1p
Sg3tf005238g1s
Sg3tf005238g0p
0.0
0.1
0.2
Branch Length
Is Dupliction Node?
N
Y
Leaf
Expression Order Of Magnitude
1.0
1.5
2.0
2.5
Proportion of Total Expression
0.5
0.4
0.3
0.2
0.1
0.0
Silk Gland w/ Majority Expression (Grey=Not 2−Fold Increased in Silk)
Broad
Not OEST

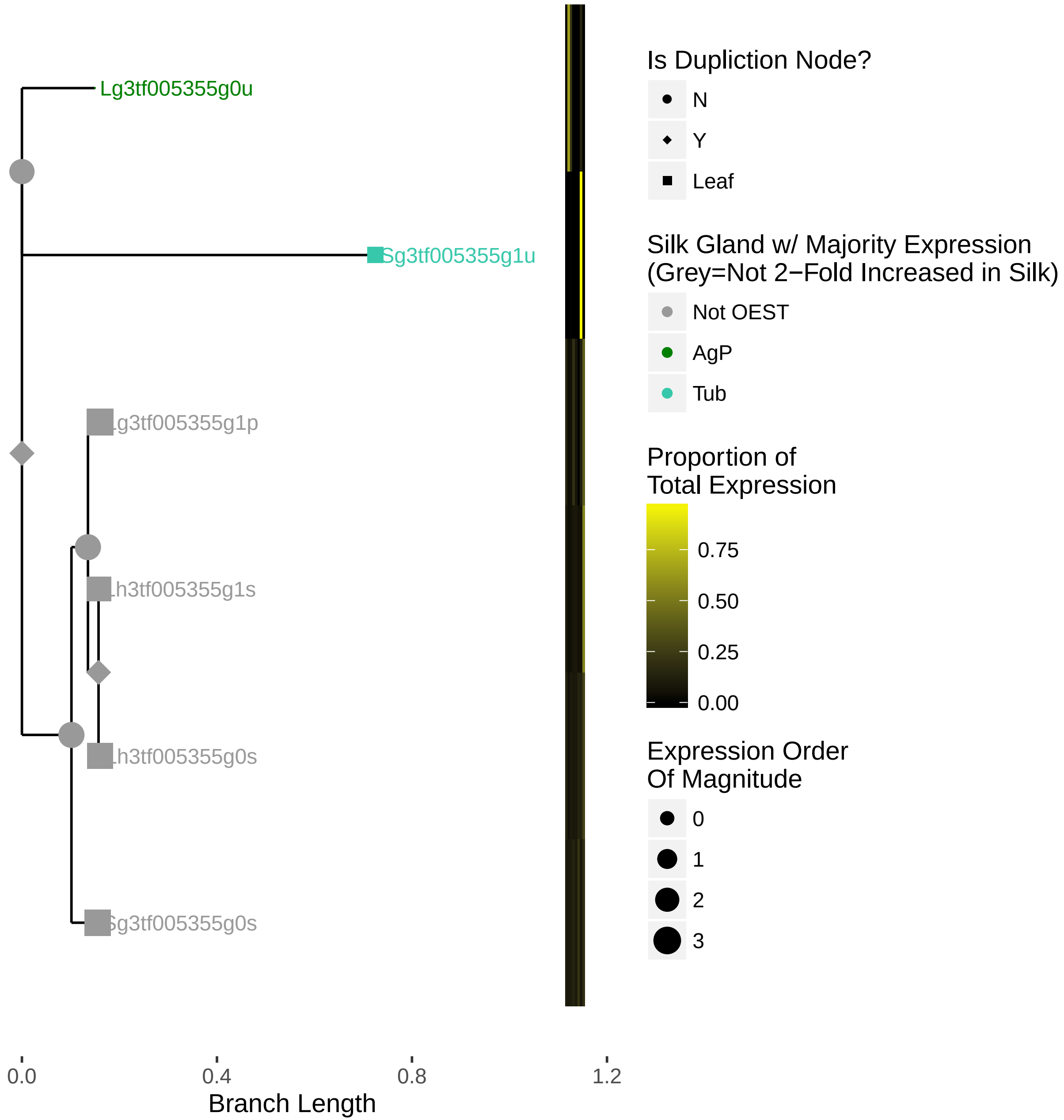

Lg3tf005355g0u
Sg3tf005355g1u
Lg3tf005355g1p
Lh3tf005355g1s
Lh3tf005355g0s
Sg3tf005355g0s
0.0
0.4
0.8
1.2
Branch Length
Is Dupliction Node?
N
Y
Leaf
Silk Gland w/ Majority Expression
(Grey=Not 2−Fold Increased in Silk)
Not OEST
AgP
Tub
Proportion of
Total Expression
0.75
0.50
0.25
0.00
Expression Order
Of Magnitude
0
1
2
3

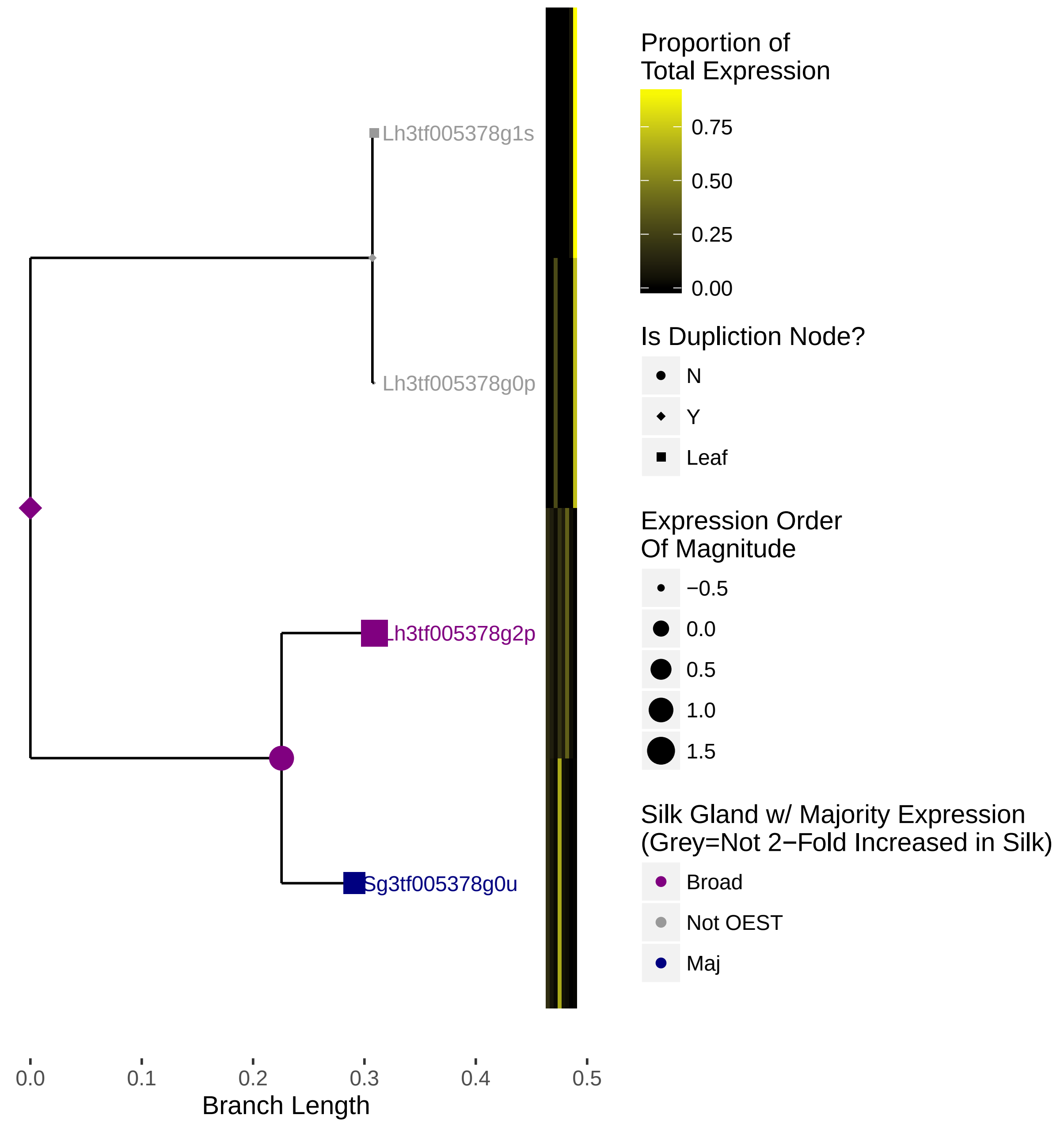

Lh3tf005378g1s
Lh3tf005378g0p
Lh3tf005378g2p
Sg3tf005378g0u
0.0
0.1
0.2
0.3
0.4
0.5
Branch Length
Proportion of
Total Expression
0.75
0.50
0.25
0.00
Is Dupliction Node?
N
Y
Leaf
Expression Order
Of Magnitude
−0.5
0.0
0.5
1.0
1.5
Silk Gland w/ Majority Expression
(Grey=Not 2−Fold Increased in Silk)
Broad
Not OEST
Maj

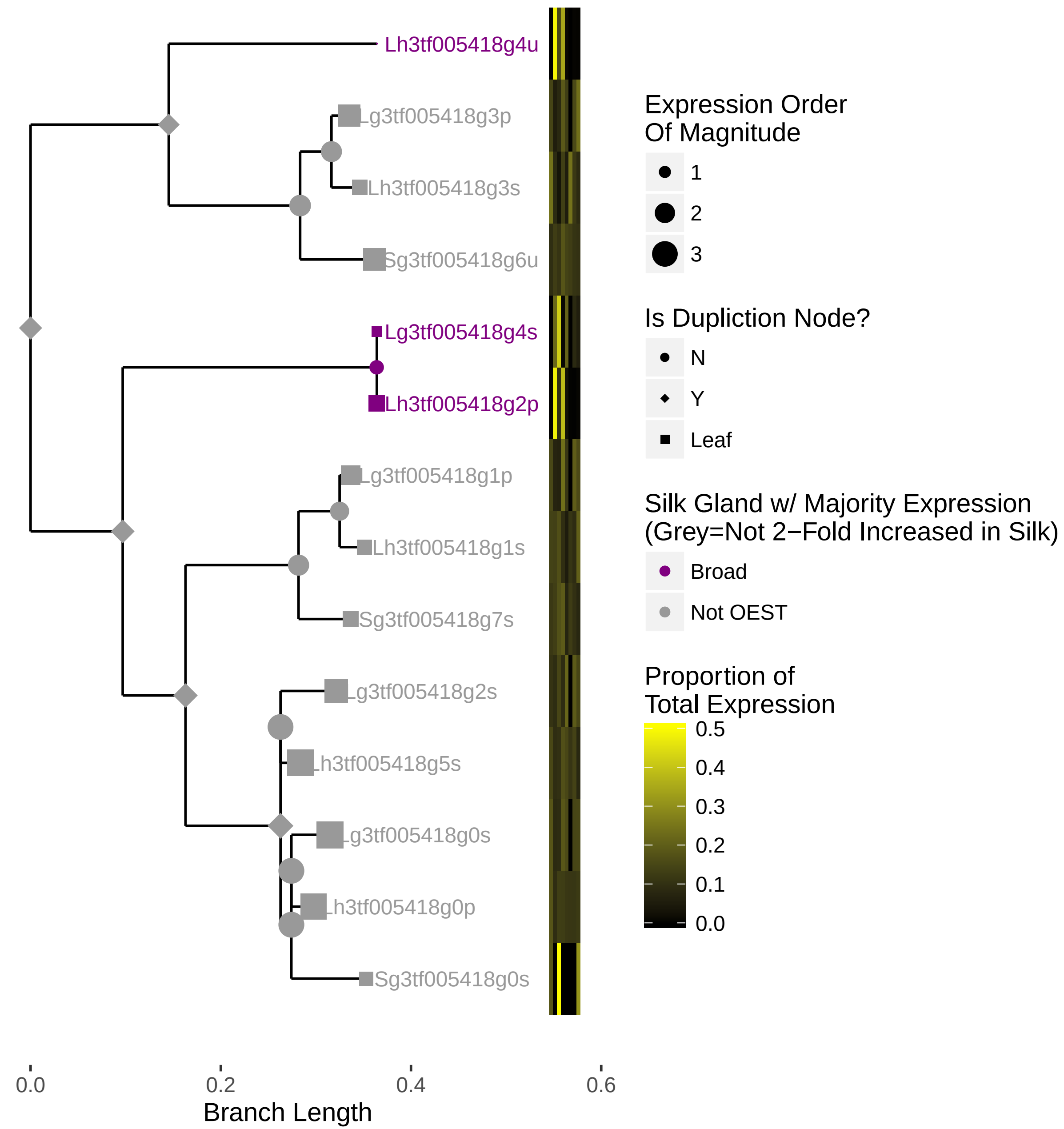

Lh3tf005418g4u
Lg3tf005418g3p
Lh3tf005418g3s
Sg3tf005418g6u
Lg3tf005418g4s
Lh3tf005418g2p
Lg3tf005418g1p
Lh3tf005418g1s
Sg3tf005418g7s
Lg3tf005418g2s
Lh3tf005418g5s
Lg3tf005418g0s
Lh3tf005418g0p
Sg3tf005418g0s
0.0
0.2
0.4
0.6
Branch Length
Expression Order Of Magnitude
1
2
3
Is Dupliction Node?
N
Y
Leaf
Silk Gland w/ Majority Expression (Grey=Not 2−Fold Increased in Silk)
Broad
Not OEST
Proportion of Total Expression
0.5
0.4
0.3
0.2
0.1
0.0

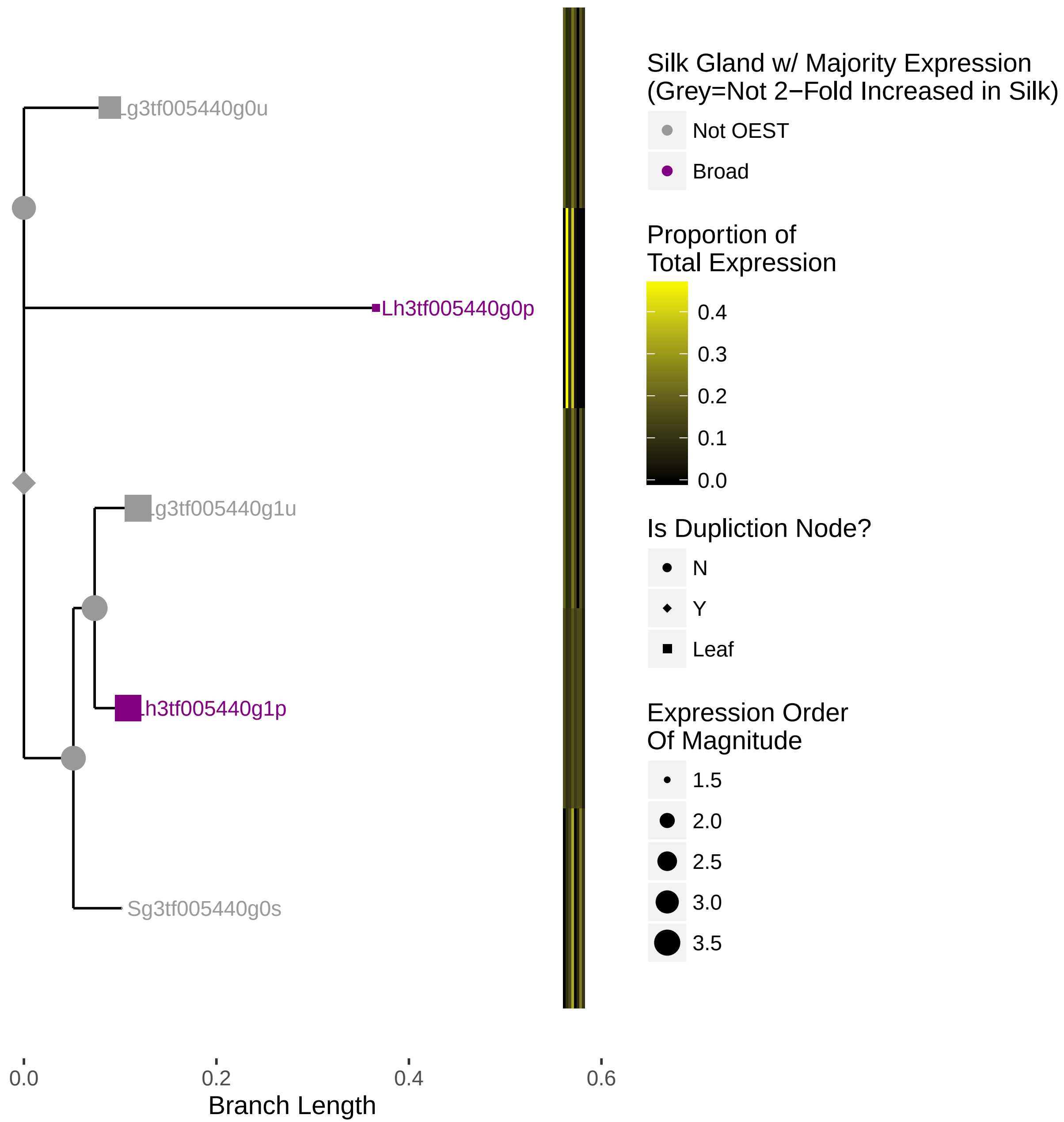

Lg3tf005440g0u
Lh3tf005440g0p
Lg3tf005440g1u
Lh3tf005440g1p
Sg3tf005440g0s
0.0
0.2
0.4
0.6
Branch Length
Silk Gland w/ Majority Expression
(Grey=Not 2−Fold Increased in Silk)
Not OEST
Broad
Proportion of
Total Expression
0.4
0.3
0.2
0.1
0.0
Is Dupliction Node?
N
Y
Leaf
Expression Order
Of Magnitude
1.5
2.0
2.5
3.0
3.5

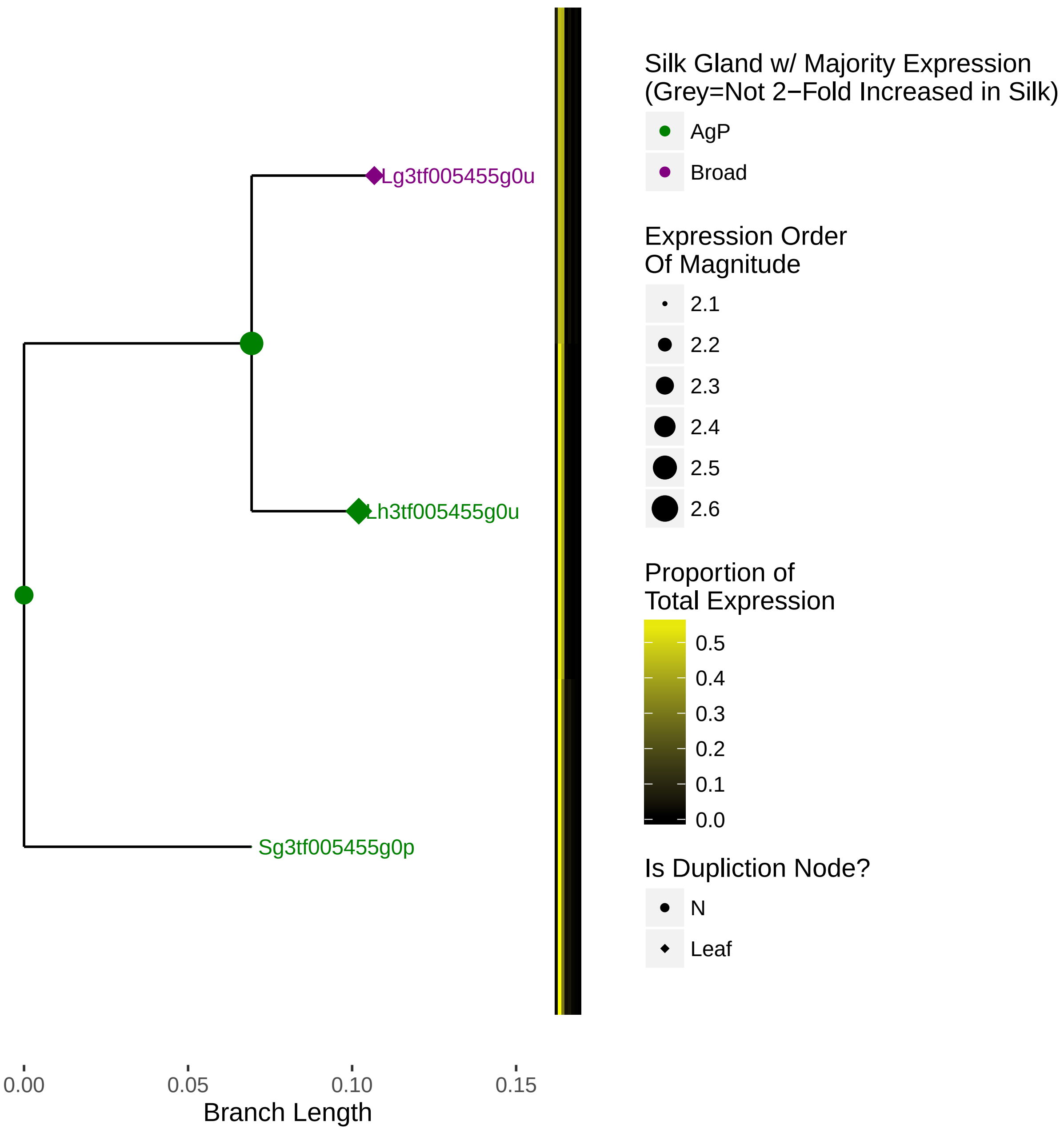

Lg3tf005455g0u
Lh3tf005455g0u
Sg3tf005455g0p
0.00
0.05
0.10
0.15
Branch Length
Silk Gland w/ Majority Expression
(Grey=Not 2−Fold Increased in Silk)
AgP
Broad
Expression Order
Of Magnitude
2.1
2.2
2.3
2.4
2.5
2.6
Proportion of
Total Expression
0.5
0.4
0.3
0.2
0.1
0.0
Is Dupliction Node?
N
Leaf

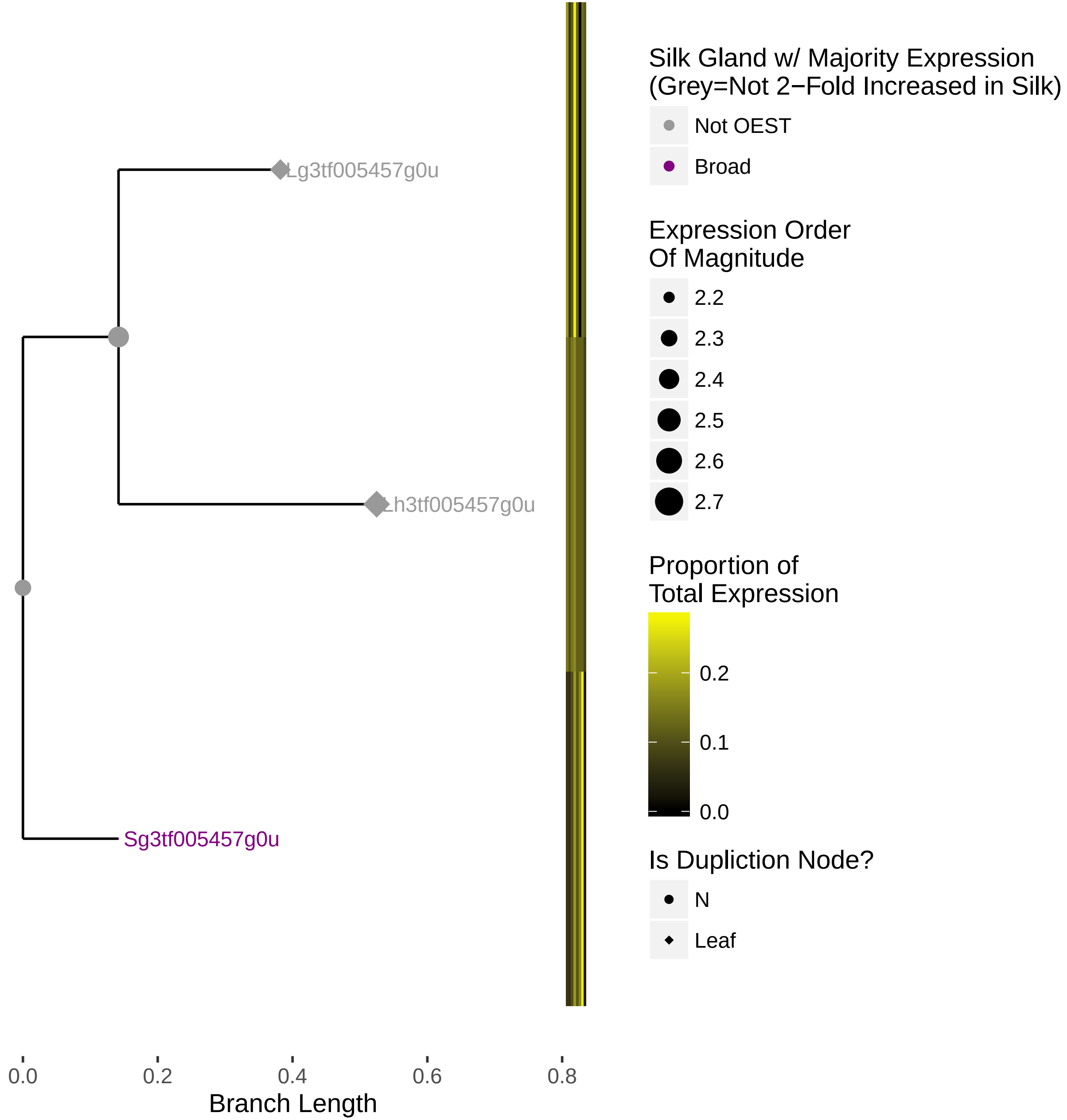

Silk Gland w/ Majority Expression
(Grey=Not 2−Fold Increased in Silk)
Not OEST
Broad
Expression Order
Of Magnitude
2.2
2.3
2.4
2.5
2.6
2.7
Proportion of
Total Expression
0.2
0.1
0.0
Is Dupliction Node?
N
Leaf
Lg3tf005457g0u
Lh3tf005457g0u
Sg3tf005457g0u
0.0
0.2
0.4
0.6
0.8
Branch Length

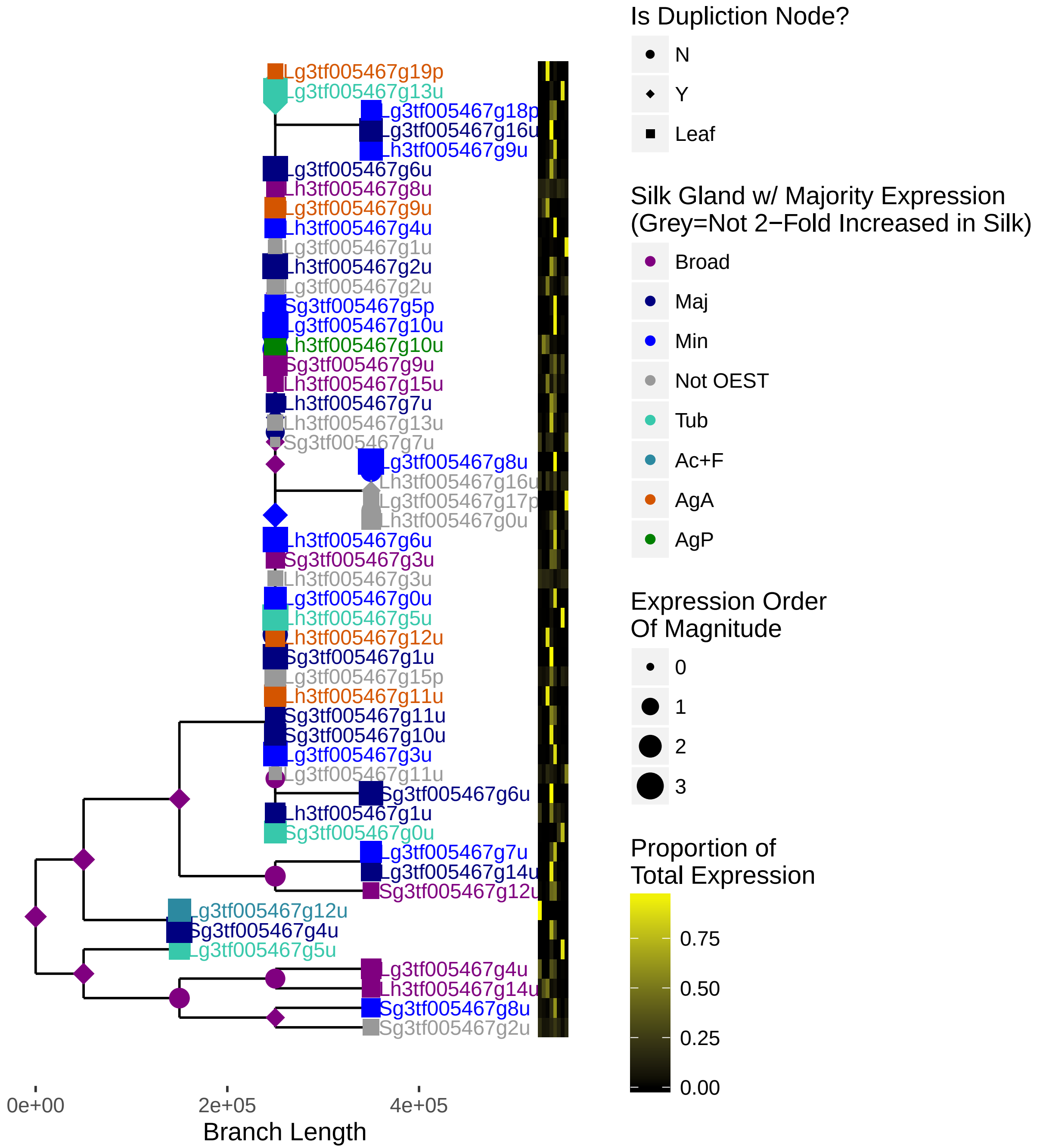

Lg3tf005467g19p
Lg3tf005467g13u
Lg3tf005467g18p
Lg3tf005467g16u
Lh3tf005467g9u
Lg3tf005467g6u
Lh3tf005467g8u
Lg3tf005467g9u
Lh3tf005467g4u
Lg3tf005467g1u
Lh3tf005467g2u
Lg3tf005467g2u
Sg3tf005467g5p
Lg3tf005467g10u
Lh3tf005467g10u
Sg3tf005467g9u
Lh3tf005467g15u
Lh3tf005467g7u
Lh3tf005467g13u
Sg3tf005467g7u
Lg3tf005467g8u
Lh3tf005467g16u
Lg3tf005467g17p
Lh3tf005467g0u
Lh3tf005467g6u
Sg3tf005467g3u
Lh3tf005467g3u
Lg3tf005467g0u
Lh3tf005467g5u
Lh3tf005467g12u
Sg3tf005467g1u
Lg3tf005467g15p
Lh3tf005467g11u
Sg3tf005467g11u
Sg3tf005467g10u
Lg3tf005467g3u
Lg3tf005467g11u
Sg3tf005467g6u
Lh3tf005467g1u
Sg3tf005467g0u
Lg3tf005467g7u
Lg3tf005467g14u
Sg3tf005467g12u
Lg3tf005467g12u
Sg3tf005467g4u
Lg3tf005467g5u
Lg3tf005467g4u
Lh3tf005467g14u
Sg3tf005467g8u
Sg3tf005467g2u
0e+00
2e+05
4e+05
Branch Length
Is Dupliction Node?
N
Y
Leaf
Silk Gland w/ Majority Expression
(Grey=Not 2−Fold Increased in Silk)
Broad
Maj
Min
Not OEST
Tub
Ac+F
AgA
AgP
Expression Order
Of Magnitude
0
1
2
3
Proportion of
Total Expression
0.75
0.50
0.25
0.00

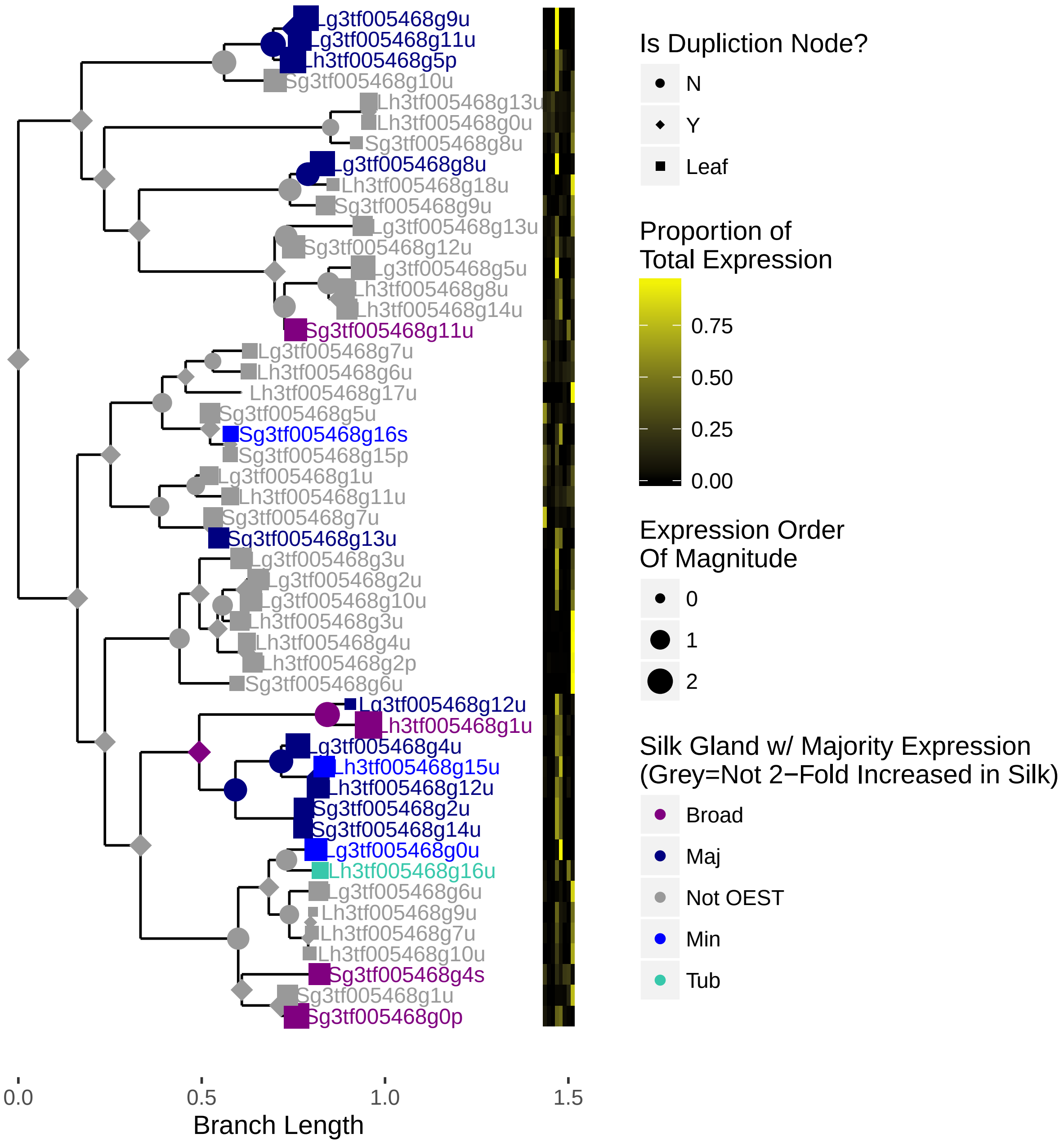

Lg3tf005468g9u
Lg3tf005468g11u
Lh3tf005468g5p
Sg3tf005468g10u
Lh3tf005468g13u
Lh3tf005468g0u
Sg3tf005468g8u
Lg3tf005468g8u
Lh3tf005468g18u
Sg3tf005468g9u
Lg3tf005468g13u
Sg3tf005468g12u
Lg3tf005468g5u
Lh3tf005468g8u
Lh3tf005468g14u
Sg3tf005468g11u
Lg3tf005468g7u
Lh3tf005468g6u
Lh3tf005468g17u
Sg3tf005468g5u
Sg3tf005468g16s
Sg3tf005468g15p
Lg3tf005468g1u
Lh3tf005468g11u
Sg3tf005468g7u
Sg3tf005468g13u
Lg3tf005468g3u
Lg3tf005468g2u
Lg3tf005468g10u
Lh3tf005468g3u
Lh3tf005468g4u
Lh3tf005468g2p
Sg3tf005468g6u
Lg3tf005468g12u
Lh3tf005468g1u
Lg3tf005468g4u
Lh3tf005468g15u
Lh3tf005468g12u
Sg3tf005468g2u
Sg3tf005468g14u
Lg3tf005468g0u
Lh3tf005468g16u
Lg3tf005468g6u
Lh3tf005468g9u
Lh3tf005468g7u
Lh3tf005468g10u
Sg3tf005468g4s
Sg3tf005468g1u
Sg3tf005468g0p
0.0
0.5
1.0
1.5
Branch Length
Is Dupliction Node?
N
Y
Leaf
Proportion of
Total Expression
0.75
0.50
0.25
0.00
Expression Order
Of Magnitude
0
1
2
Silk Gland w/ Majority Expression
(Grey=Not 2−Fold Increased in Silk)
Broad
Maj
Not OEST
Min
Tub

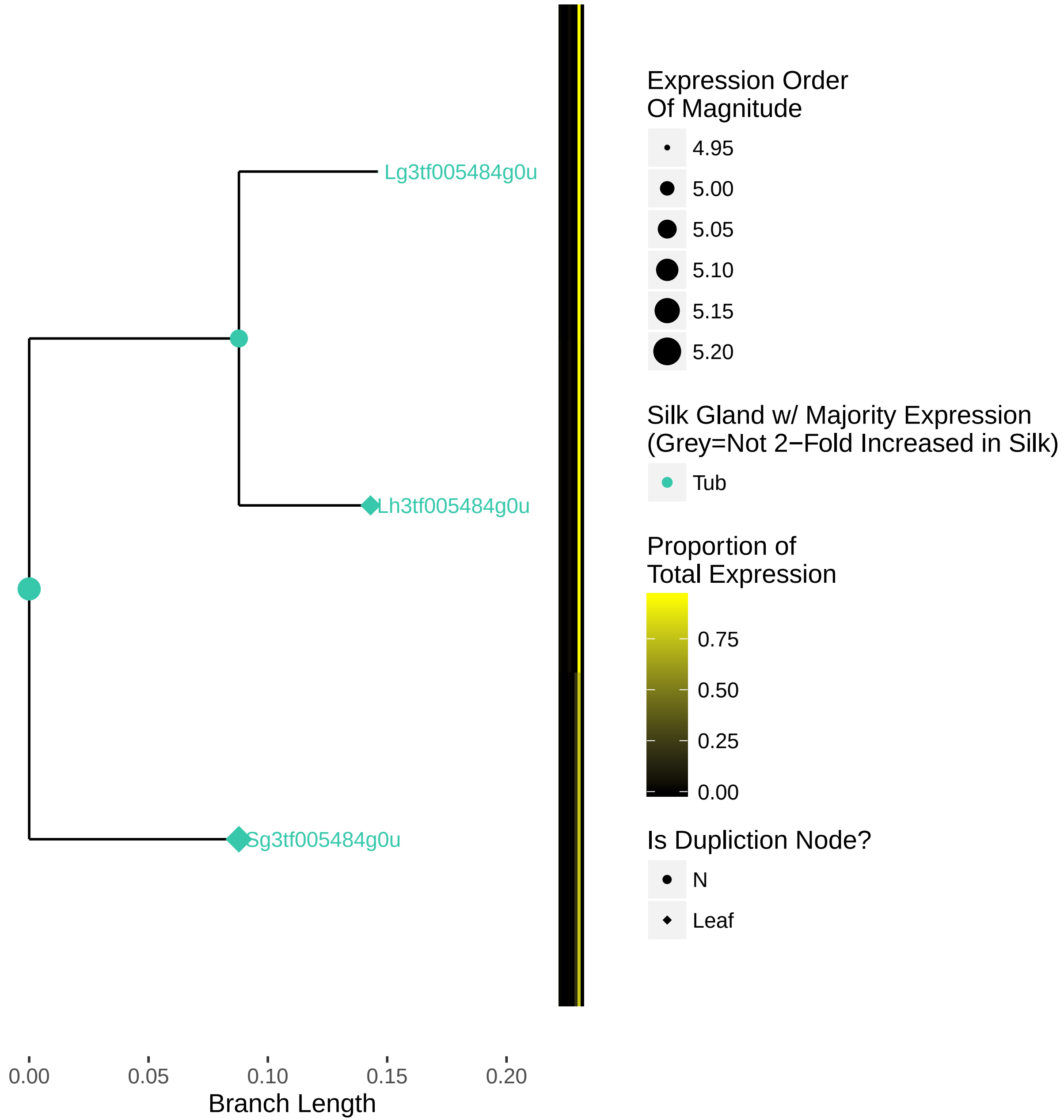
Lg3tf005484g0u
Lh3tf005484g0u
Sg3tf005484g0u
0.00
0.05
0.10
0.15
0.20
Branch Length
Expression Order
Of Magnitude
4.95
5.00
5.05
5.10
5.15
5.20
Silk Gland w/ Majority Expression
(Grey=Not 2−Fold Increased in Silk)
Tub
Proportion of
Total Expression
0.75
0.50
0.25
0.00
Is Dupliction Node?
N
Leaf

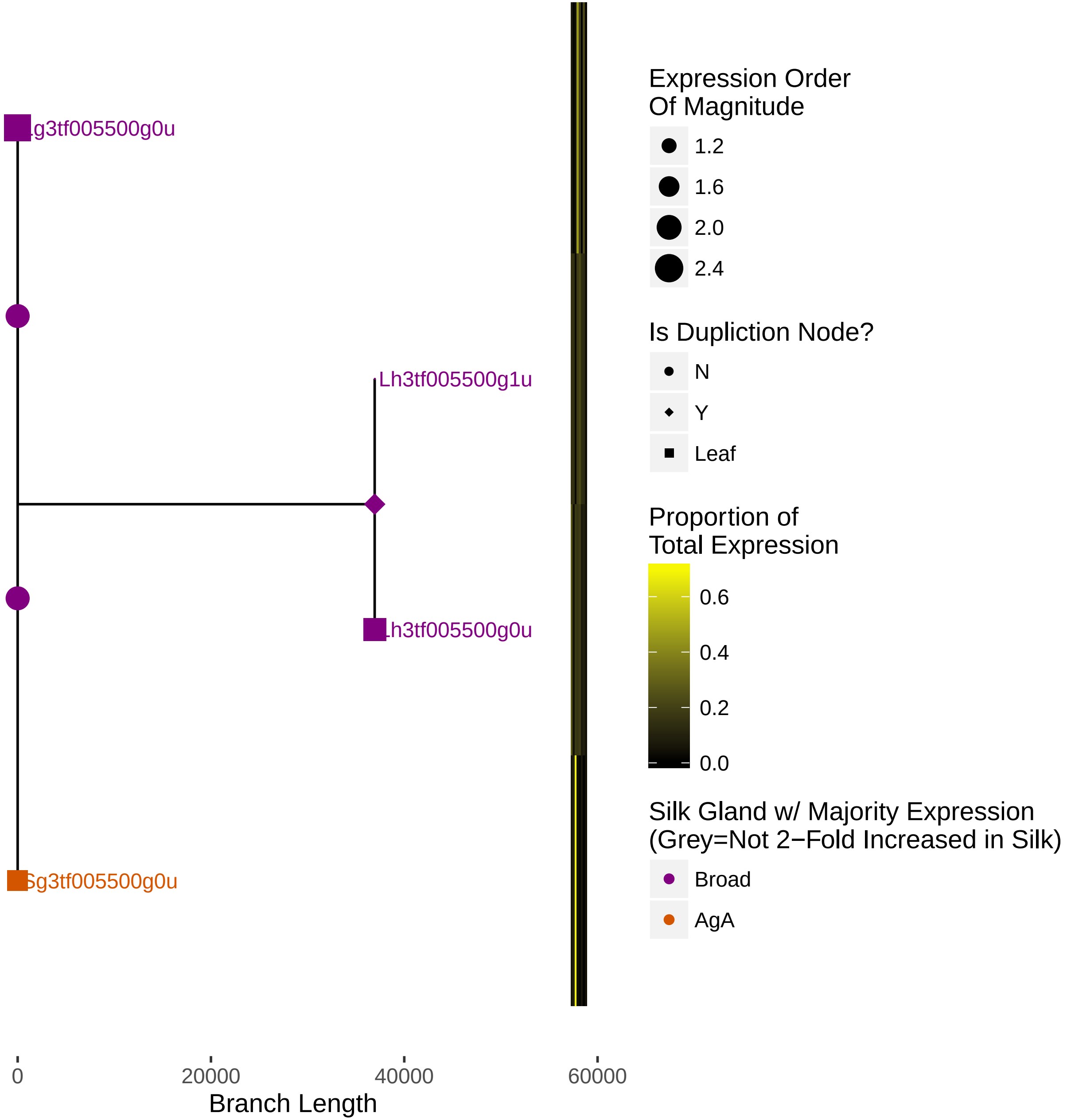

Lg3tf005500g0u
Lh3tf005500g1u
Lh3tf005500g0u
Sg3tf005500g0u
Expression Order
Of Magnitude
1.2
1.6
2.0
2.4
Is Dupliction Node?
N
Y
Leaf
Proportion of
Total Expression
0.6
0.4
0.2
0.0
Silk Gland w/ Majority Expression
(Grey=Not 2−Fold Increased in Silk)
Broad
AgA
0
20000
40000
60000
Branch Length

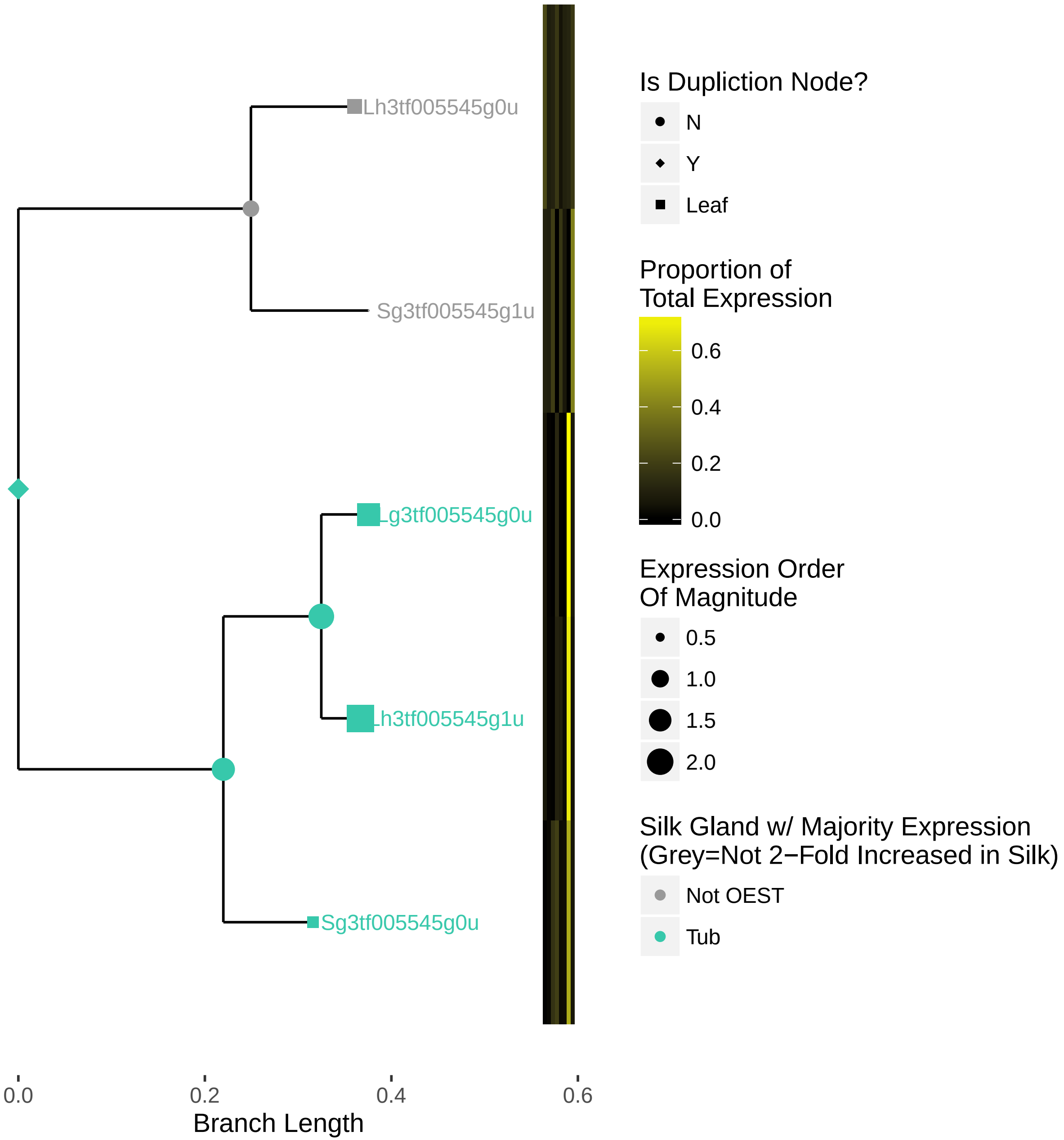

Lh3tf005545g0u
Sg3tf005545g1u
Lg3tf005545g0u
Lh3tf005545g1u
Sg3tf005545g0u
Is Dupliction Node?
N
Y
Leaf
Proportion of
Total Expression
0.6
0.4
0.2
0.0
Expression Order
Of Magnitude
0.5
1.0
1.5
2.0
Silk Gland w/ Majority Expression
(Grey=Not 2−Fold Increased in Silk)
Not OEST
Tub
0.0
0.2
0.4
0.6
Branch Length

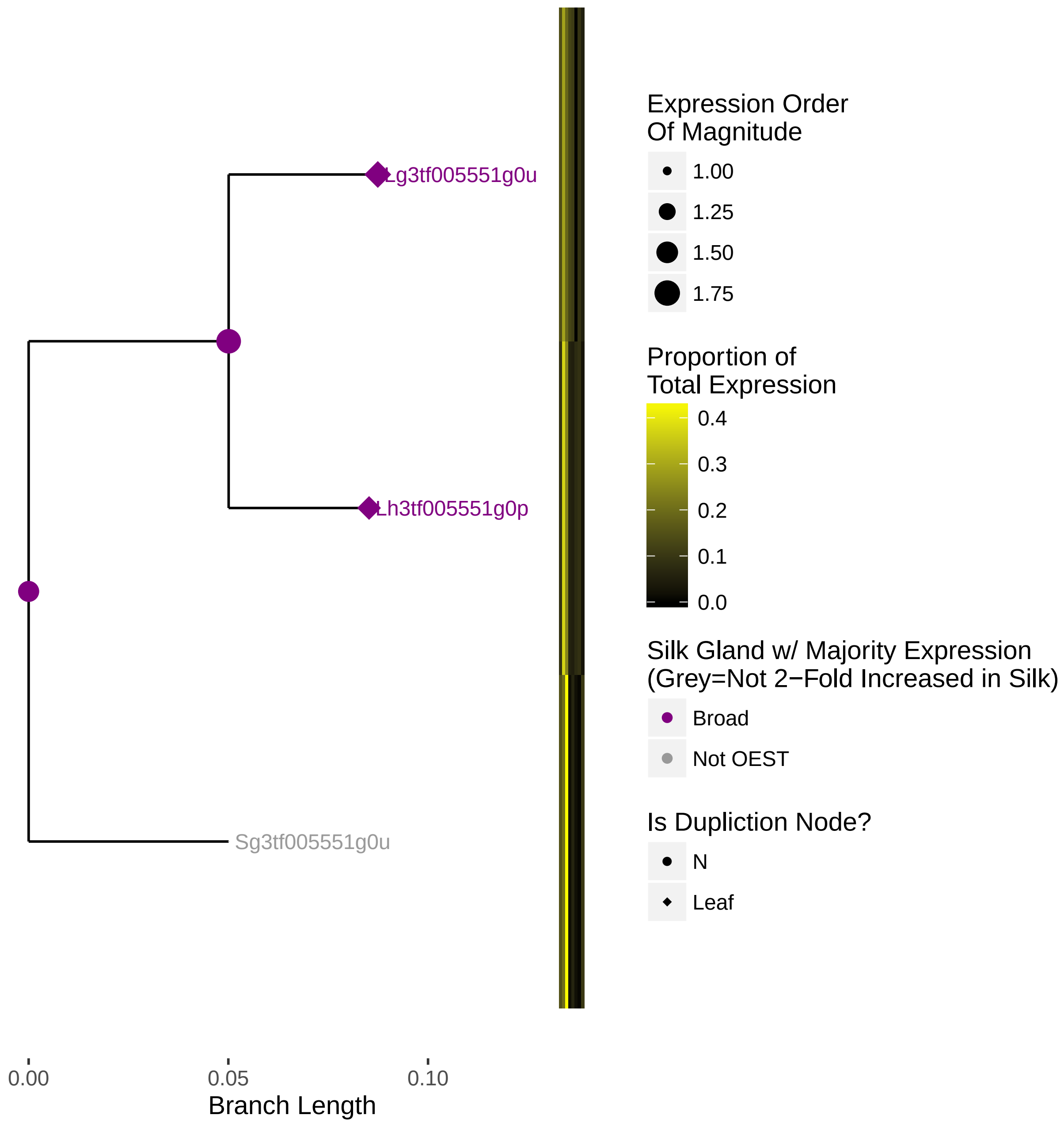

Lg3tf005551g0u
Lh3tf005551g0p
Sg3tf005551g0u
0.00
0.05
0.10
Branch Length
Expression Order
Of Magnitude
1.00
1.25
1.50
1.75
Proportion of
Total Expression
0.4
0.3
0.2
0.1
0.0
Silk Gland w/ Majority Expression
(Grey=Not 2−Fold Increased in Silk)
Broad
Not OEST
Is Dupliction Node?
N
Leaf

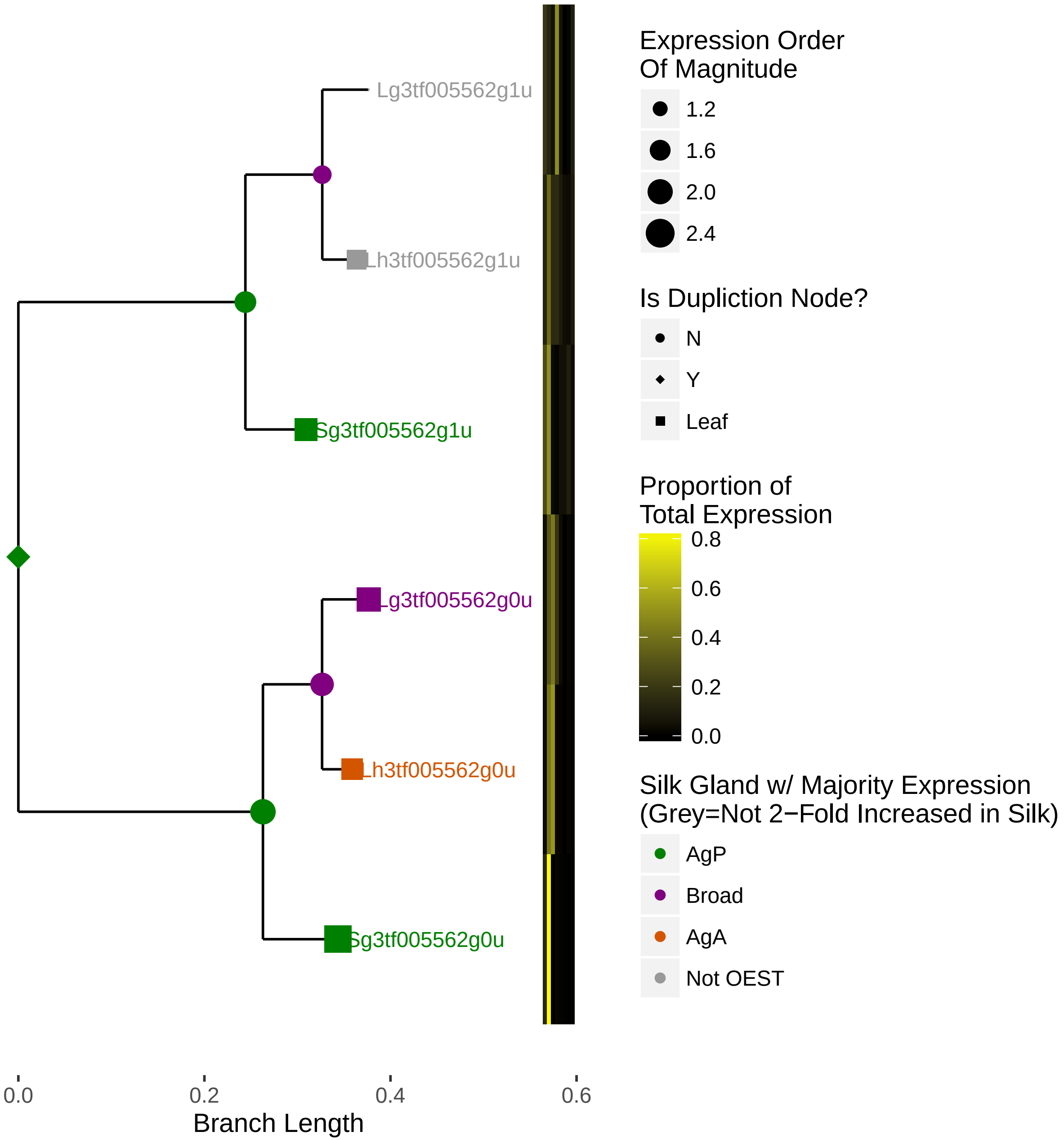
Lg3tf005562g1u
Lh3tf005562g1u
Sg3tf005562g1u
Lg3tf005562g0u
Lh3tf005562g0u
Sg3tf005562g0u
0.0
0.2
0.4
0.6
Branch Length
Expression Order
Of Magnitude
1.2
1.6
2.0
2.4
Is Dupliction Node?
N
Y
Leaf
Proportion of
Total Expression
0.8
0.6
0.4
0.2
0.0
Silk Gland w/ Majority Expression
(Grey=Not 2−Fold Increased in Silk)
AgP
Broad
AgA
Not OEST

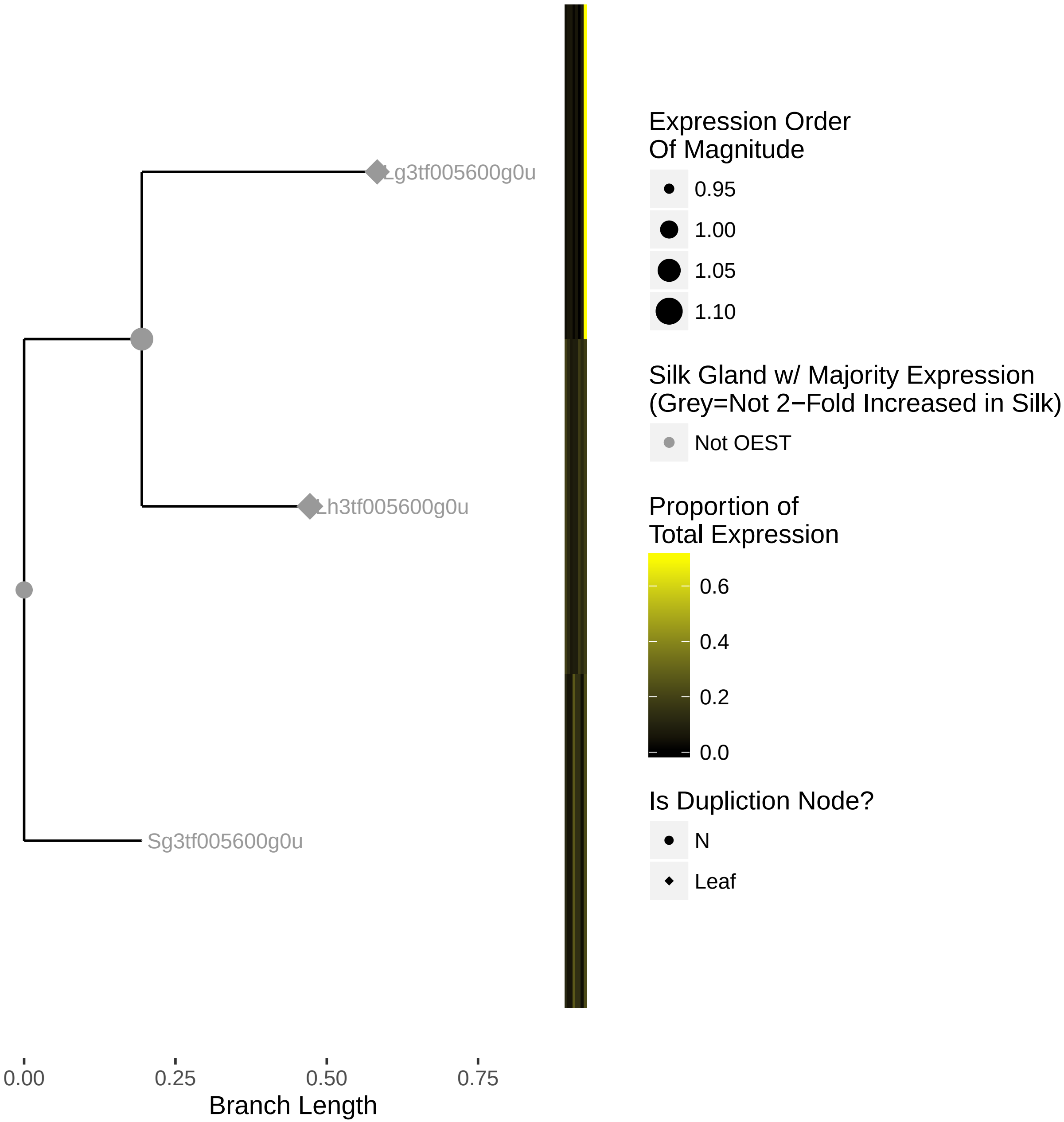

g3tf005600g0u
h3tf005600g0u
Sg3tf005600g0u
0.00
0.25
0.50
0.75
Branch Length
Expression Order
Of Magnitude
0.95
1.00
1.05
1.10
Silk Gland w/ Majority Expression
(Grey=Not 2−Fold Increased in Silk)
Not OEST
Proportion of
Total Expression
0.6
0.4
0.2
0.0
Is Dupliction Node?
N
Leaf

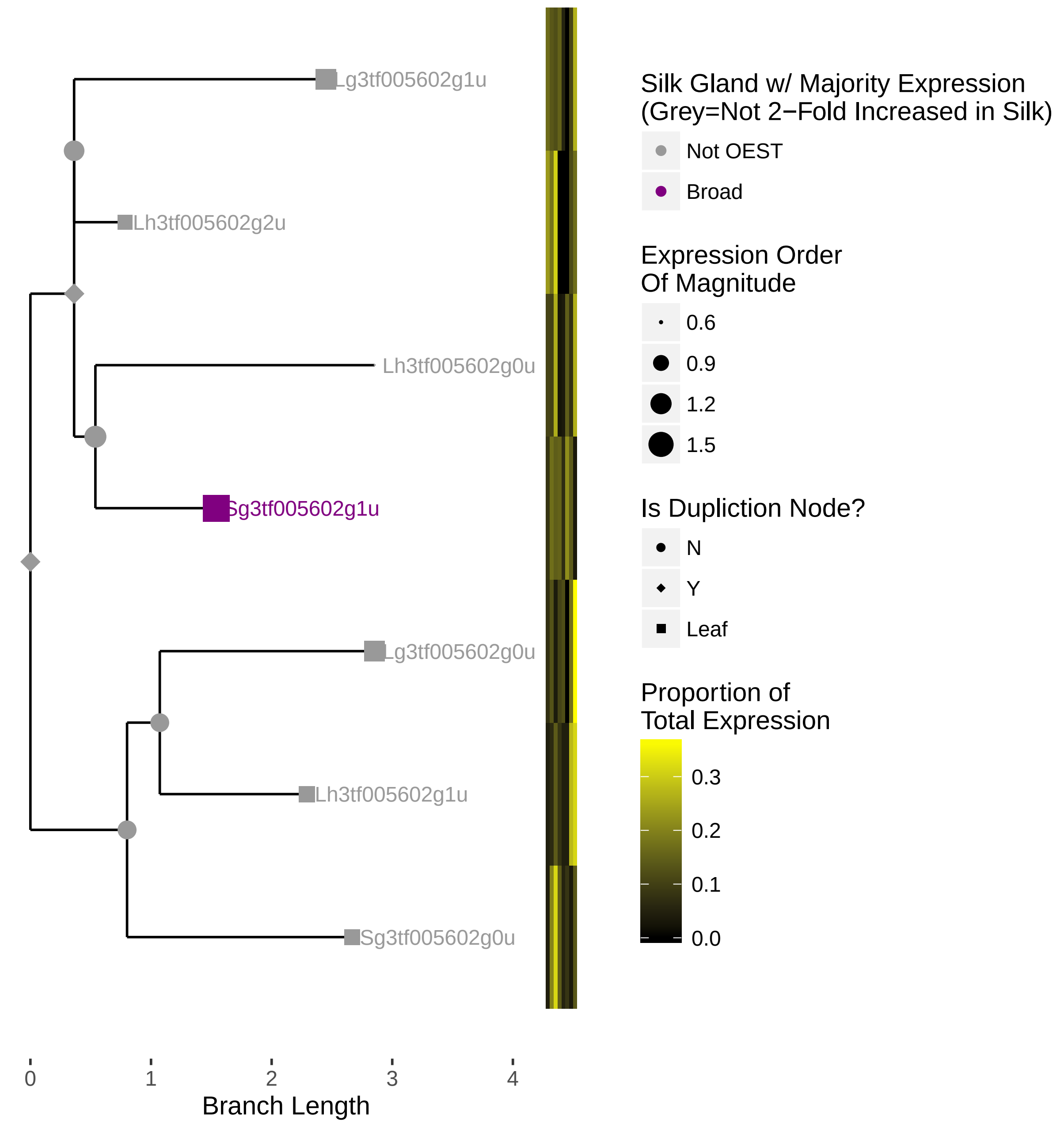

Lg3tf005602g1u
Lh3tf005602g2u
Lh3tf005602g0u
Sg3tf005602g1u
Lg3tf005602g0u
Lh3tf005602g1u
Sg3tf005602g0u
0
1
2
3
4
Branch Length
Silk Gland w/ Majority Expression
(Grey=Not 2−Fold Increased in Silk)
Not OEST
Broad
Expression Order
Of Magnitude
0.6
0.9
1.2
1.5
Is Dupliction Node?
N
Y
Leaf
Proportion of
Total Expression
0.3
0.2
0.1
0.0

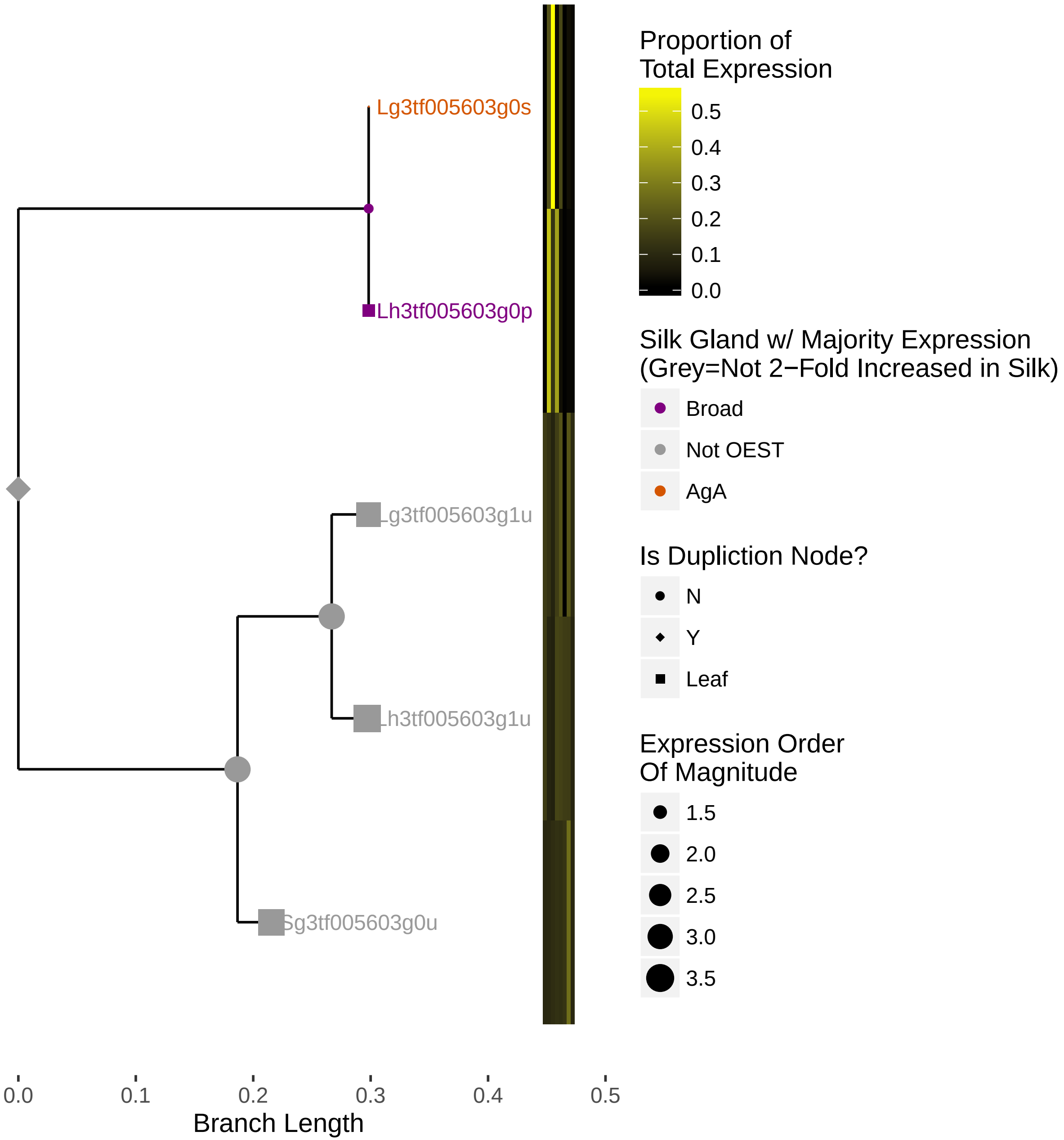

Lg3tf005603g0s
Lh3tf005603g0p
Lg3tf005603g1u
Lh3tf005603g1u
Sg3tf005603g0u
0.0
0.1
0.2
0.3
0.4
0.5
Branch Length
Proportion of
Total Expression
0.5
0.4
0.3
0.2
0.1
0.0
Silk Gland w/ Majority Expression
(Grey=Not 2−Fold Increased in Silk)
Broad
Not OEST
AgA
Is Dupliction Node?
N
Y
Leaf
Expression Order
Of Magnitude
1.5
2.0
2.5
3.0
3.5

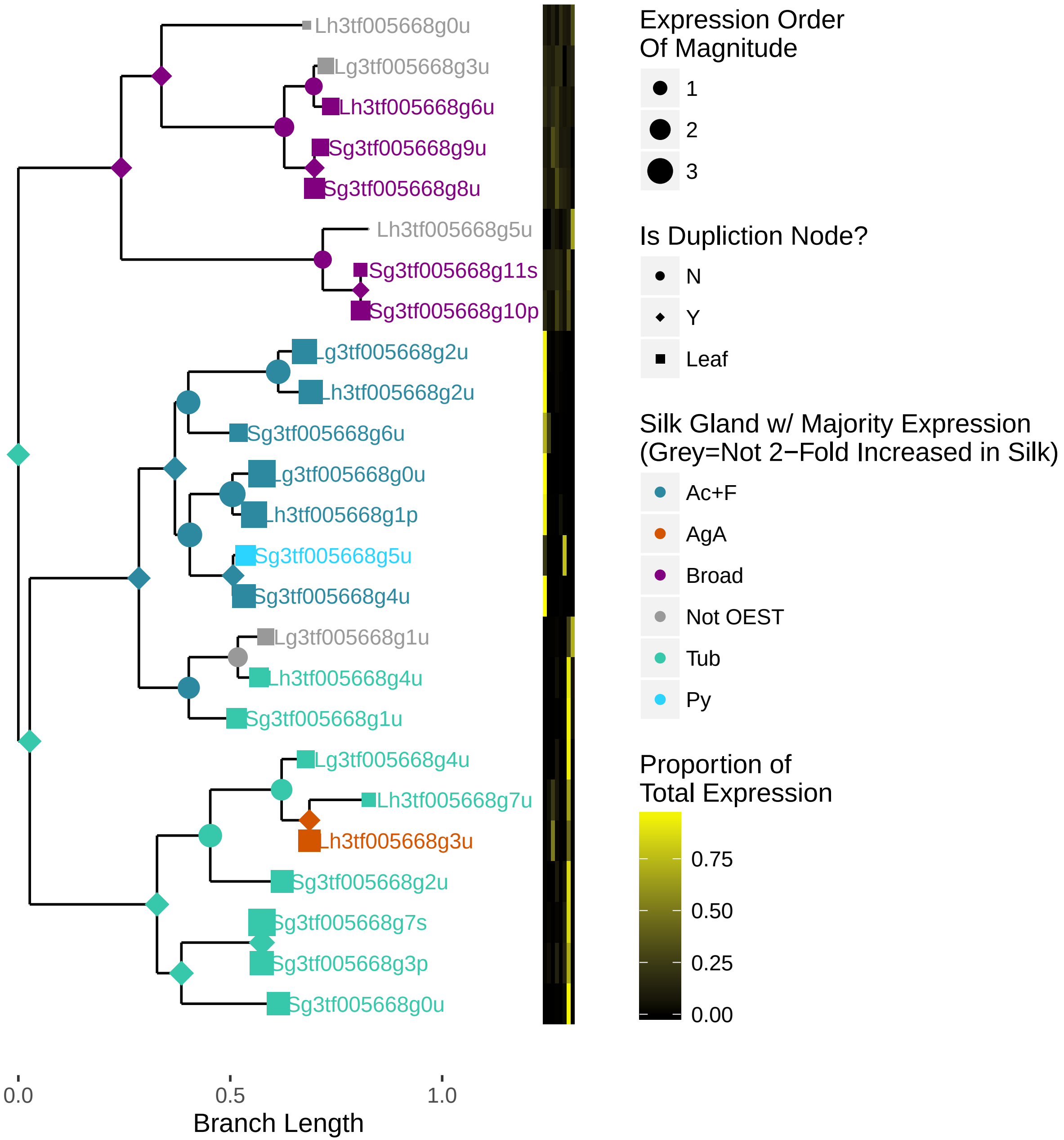

Lh3tf005668g0u
Lg3tf005668g3u
Lh3tf005668g6u
Sg3tf005668g9u
Sg3tf005668g8u
Lh3tf005668g5u
Sg3tf005668g11s
Sg3tf005668g10p
Lg3tf005668g2u
Lh3tf005668g2u
Sg3tf005668g6u
Lg3tf005668g0u
Lh3tf005668g1p
Sg3tf005668g5u
Sg3tf005668g4u
Lg3tf005668g1u
Lh3tf005668g4u
Sg3tf005668g1u
Lg3tf005668g4u
Lh3tf005668g7u
Lh3tf005668g3u
Sg3tf005668g2u
Sg3tf005668g7s
Sg3tf005668g3p
Sg3tf005668g0u
0.0
0.5
1.0
Branch Length
Expression Order Of Magnitude
1
2
3
Is Dupliction Node?
N
Y
Leaf
Silk Gland w/ Majority Expression (Grey=Not 2−Fold Increased in Silk)
Ac+F
AgA
Broad
Not OEST
Tub
Py
Proportion of Total Expression
0.75
0.50
0.25
0.00

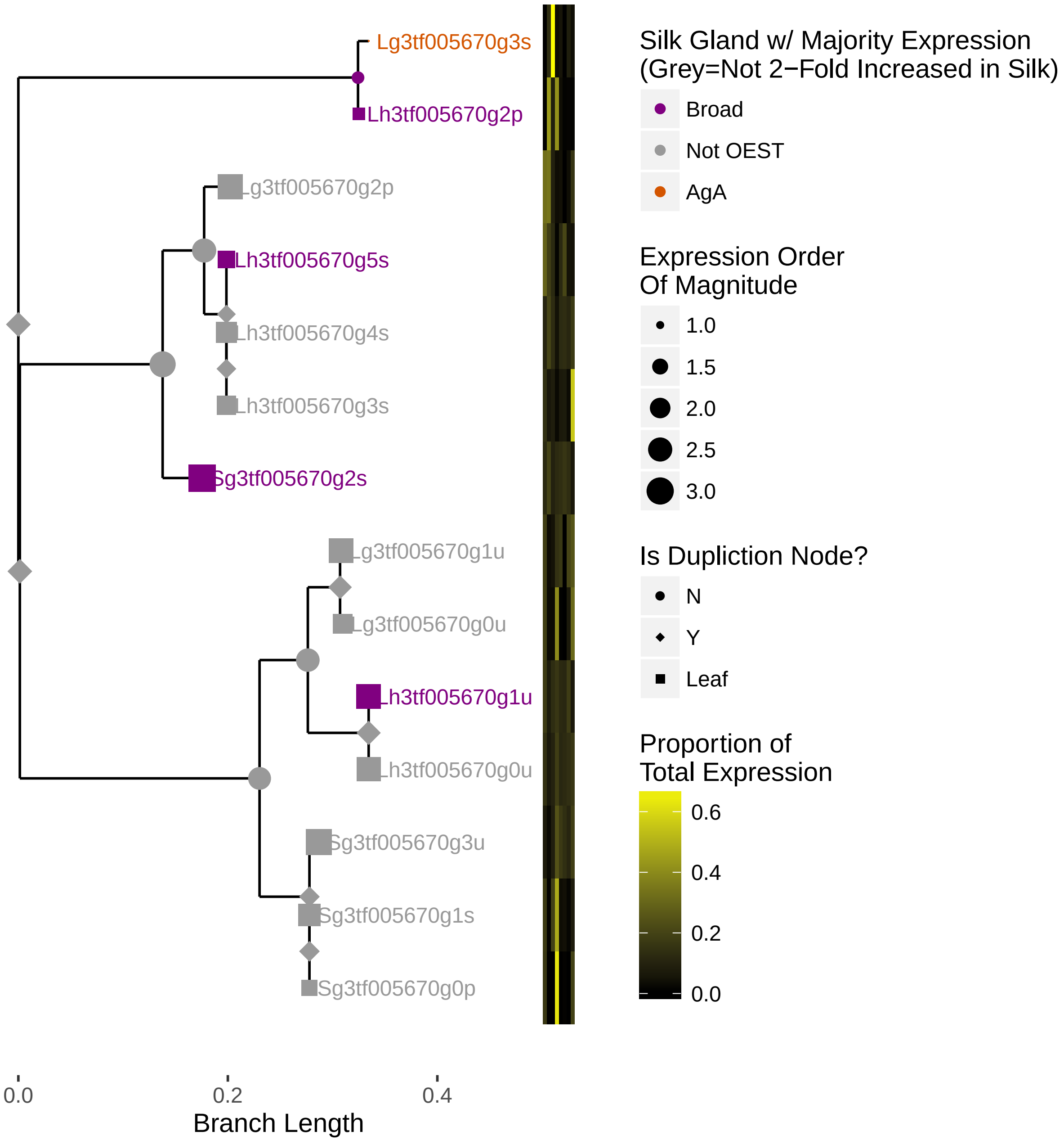

Lg3tf005670g3s
Lh3tf005670g2p
Lg3tf005670g2p
Lh3tf005670g5s
Lh3tf005670g4s
Lh3tf005670g3s
Sg3tf005670g2s
Lg3tf005670g1u
Lg3tf005670g0u
Lh3tf005670g1u
Lh3tf005670g0u
Sg3tf005670g3u
Sg3tf005670g1s
Sg3tf005670g0p
0.0
0.2
0.4
Branch Length
Silk Gland w/ Majority Expression
(Grey=Not 2−Fold Increased in Silk)
Broad
Not OEST
AgA
Expression Order
Of Magnitude
1.0
1.5
2.0
2.5
3.0
Is Dupliction Node?
N
Y
Leaf
Proportion of
Total Expression
0.6
0.4
0.2
0.0

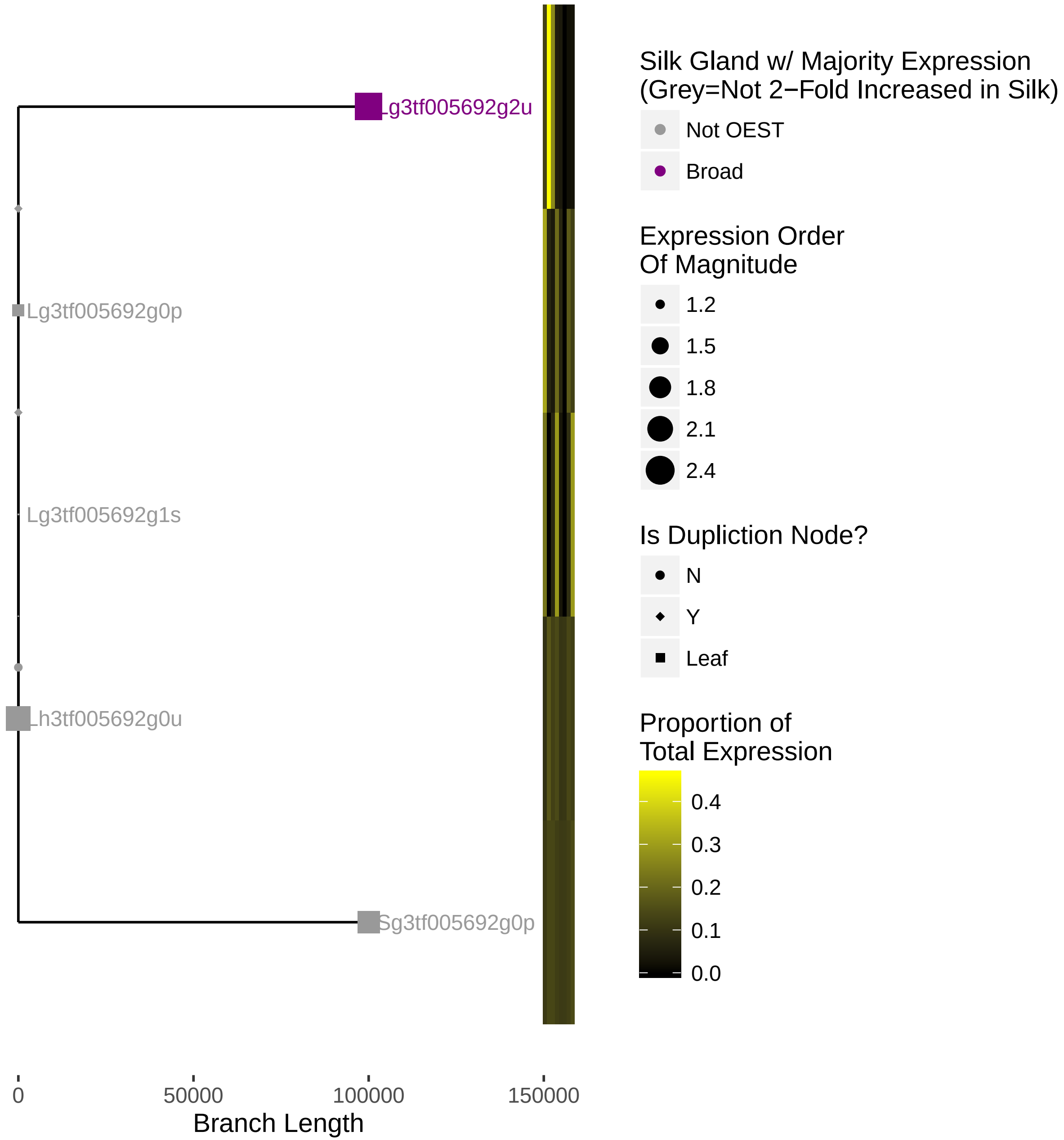

Lg3tf005692g2u
Lg3tf005692g0p
Lg3tf005692g1s
Lh3tf005692g0u
Sg3tf005692g0p
0
50000
100000
150000
Branch Length
Silk Gland w/ Majority Expression
(Grey=Not 2−Fold Increased in Silk)
Not OEST
Broad
Expression Order
Of Magnitude
1.2
1.5
1.8
2.1
2.4
Is Dupliction Node?
N
Y
Leaf
Proportion of
Total Expression
0.4
0.3
0.2
0.1
0.0

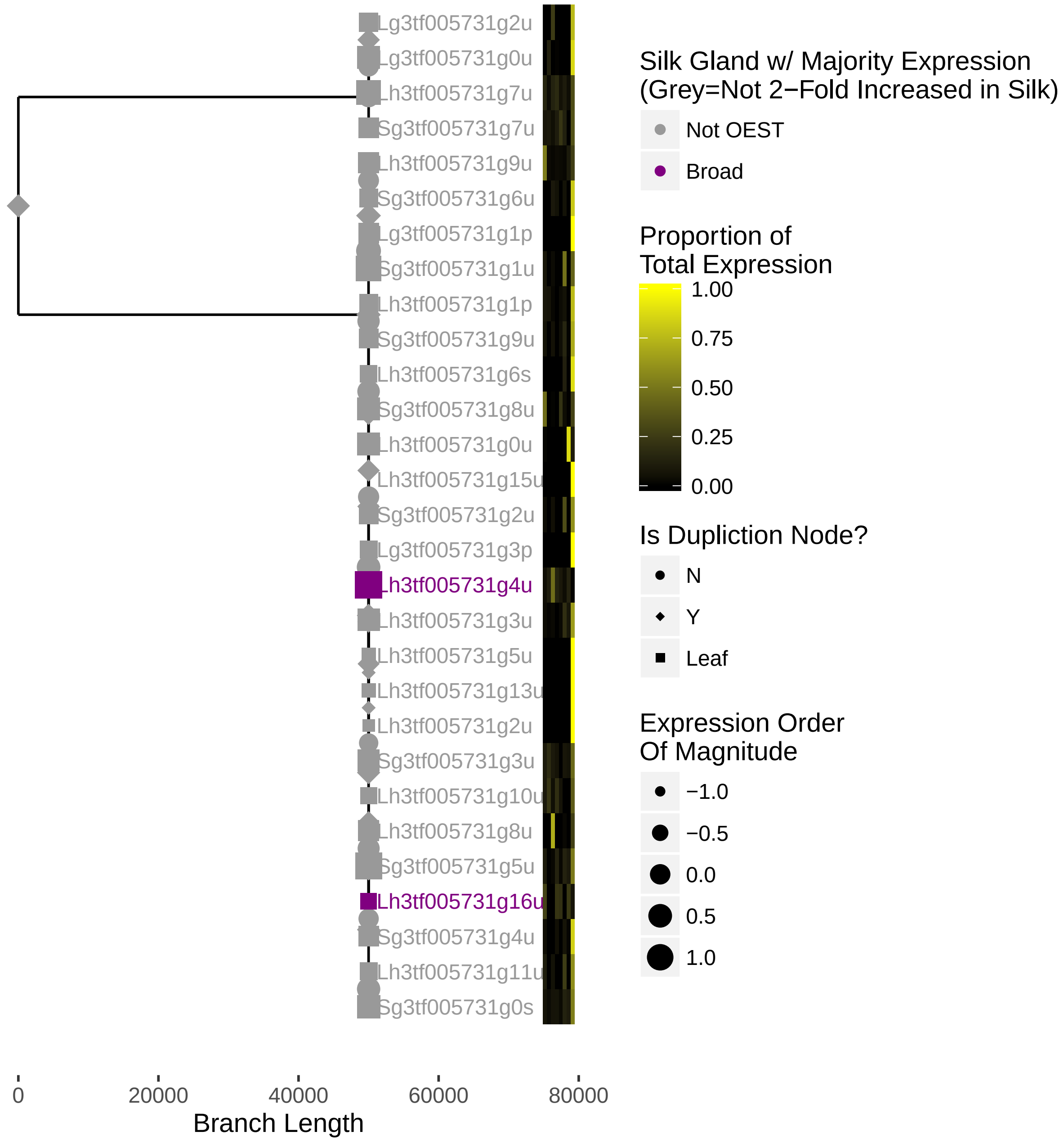

Lg3tf005731g2u
Lg3tf005731g0u
Lh3tf005731g7u
Sg3tf005731g7u
Lh3tf005731g9u
Sg3tf005731g6u
Lg3tf005731g1p
Sg3tf005731g1u
Lh3tf005731g1p
Sg3tf005731g9u
Lh3tf005731g6s
Sg3tf005731g8u
Lh3tf005731g0u
Lh3tf005731g15u
Sg3tf005731g2u
Lg3tf005731g3p
Lh3tf005731g4u
Lh3tf005731g3u
Lh3tf005731g5u
Lh3tf005731g13u
Lh3tf005731g2u
Sg3tf005731g3u
Lh3tf005731g10u
Lh3tf005731g8u
Sg3tf005731g5u
Lh3tf005731g16u
Sg3tf005731g4u
Lh3tf005731g11u
Sg3tf005731g0s
0
20000
40000
60000
80000
Branch Length
Silk Gland w/ Majority Expression
(Grey=Not 2−Fold Increased in Silk)
Not OEST
Broad
Proportion of
Total Expression
1.00
0.75
0.50
0.25
0.00
Is Dupliction Node?
N
Y
Leaf
Expression Order
Of Magnitude
−1.0
−0.5
0.0
0.5
1.0

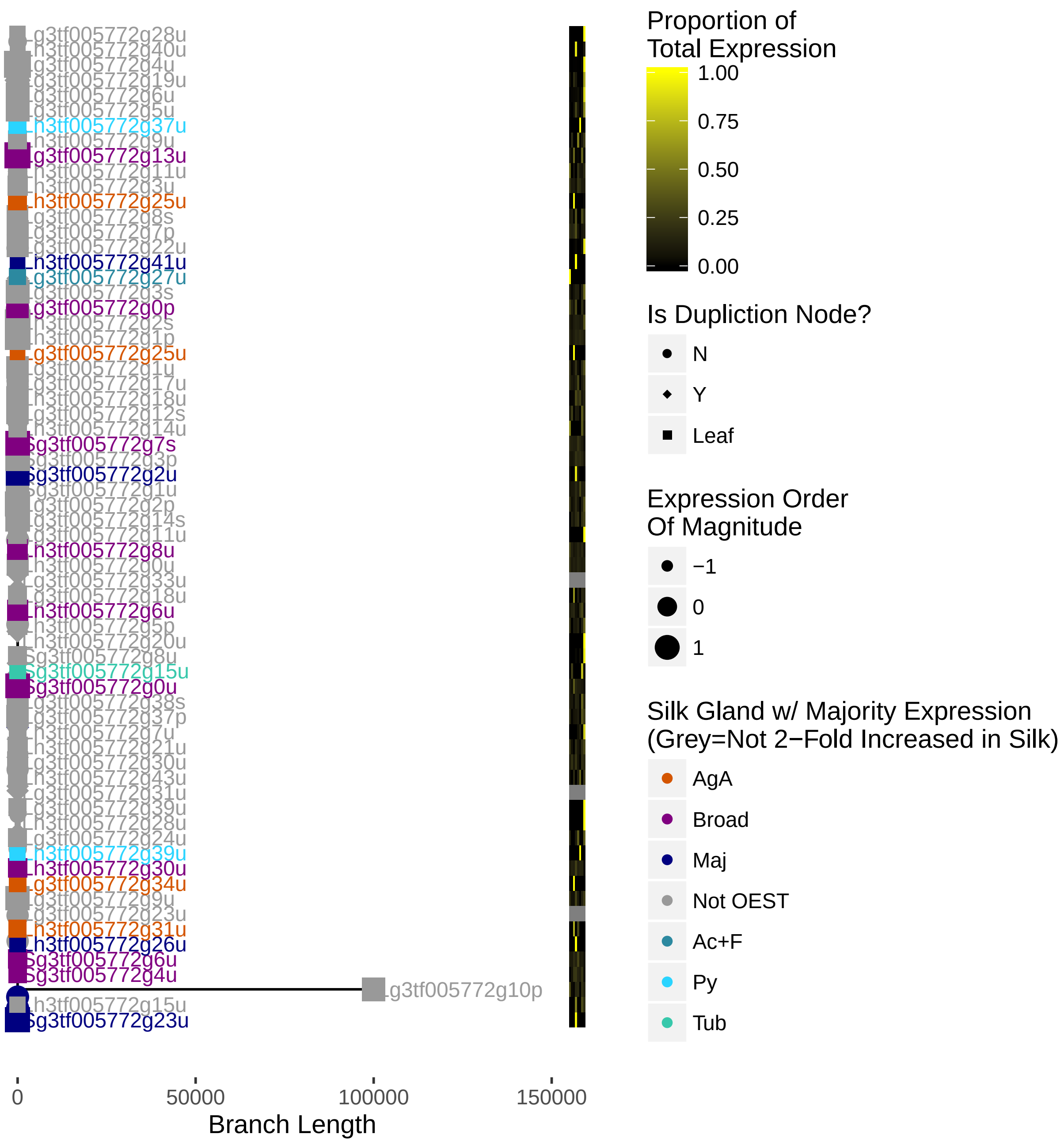

Lg3tf005772g28u
Lh3tf005772g40u
Lg3tf005772g4u
Lg3tf005772g19u
Lg3tf005772g6u
Lg3tf005772g5u
Lh3tf005772g37u
Lh3tf005772g9u
Lg3tf005772g13u
Lh3tf005772g11u
Lh3tf005772g3u
Lh3tf005772g25u
Lg3tf005772g8s
Lg3tf005772g7p
Lg3tf005772g22u
Lh3tf005772g41u
Lg3tf005772g27u
Lg3tf005772g3s
Lg3tf005772g0p
Lh3tf005772g2s
Lh3tf005772g1p
Lg3tf005772g25u
Lg3tf005772g1u
Lg3tf005772g17u
Lh3tf005772g18u
Lg3tf005772g12s
Lh3tf005772g14u
Sg3tf005772g7s
Sg3tf005772g3p
Sg3tf005772g2u
Sg3tf005772g1u
Lg3tf005772g2p
Lg3tf005772g14s
Lg3tf005772g11u
Lh3tf005772g8u
Lh3tf005772g0u
Lg3tf005772g33u
Lg3tf005772g18u
Lh3tf005772g6u
Lh3tf005772g5p
Lh3tf005772g20u
Sg3tf005772g8u
Sg3tf005772g15u
Sg3tf005772g0u
Lg3tf005772g38s
Lg3tf005772g37p
Lh3tf005772g7u
Lh3tf005772g21u
Lg3tf005772g30u
Lh3tf005772g43u
Lg3tf005772g31u
Lg3tf005772g39u
Lh3tf005772g28u
Lg3tf005772g24u
Lh3tf005772g39u
Lh3tf005772g30u
Lg3tf005772g34u
Lg3tf005772g9u
Lg3tf005772g23u
Lh3tf005772g31u
Lh3tf005772g26u
Sg3tf005772g6u
Sg3tf005772g4u
Lg3tf005772g10p
Lh3tf005772g15u
Sg3tf005772g23u
0
50000
100000
150000
Branch Length
Proportion of
Total Expression
1.00
0.75
0.50
0.25
0.00
Is Dupliction Node?
N
Y
Leaf
Expression Order
Of Magnitude
−1
0
1
Silk Gland w/ Majority Expression
(Grey=Not 2−Fold Increased in Silk)
AgA
Broad
Maj
Not OEST
Ac+F
Py
Tub

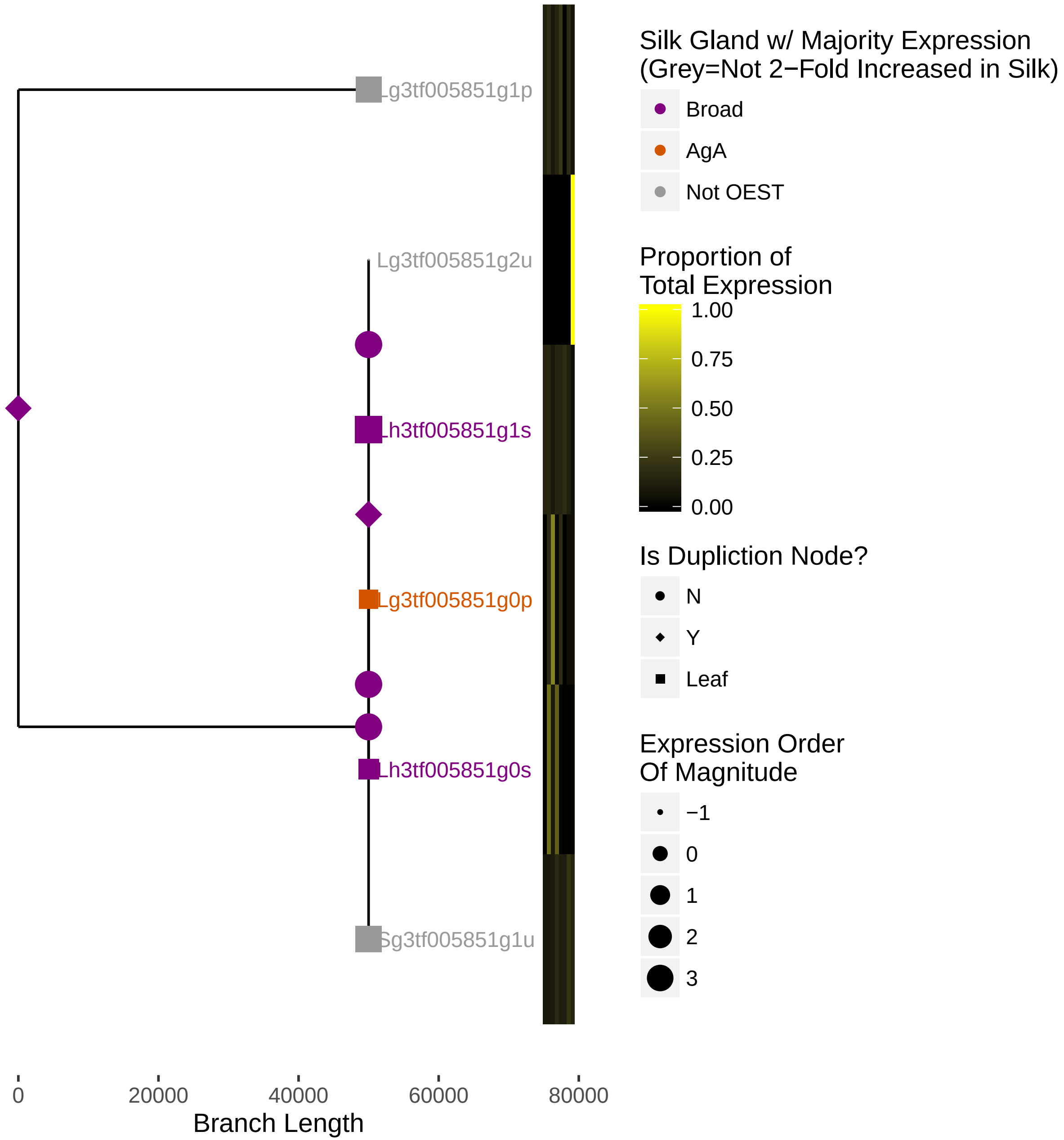

Lg3tf005851g1p
Lg3tf005851g2u
Lh3tf005851g1s
Lg3tf005851g0p
Lh3tf005851g0s
Sg3tf005851g1u
0
20000
40000
60000
80000
Branch Length
Silk Gland w/ Majority Expression
(Grey=Not 2−Fold Increased in Silk)
Broad
AgA
Not OEST
Proportion of
Total Expression
1.00
0.75
0.50
0.25
0.00
Is Dupliction Node?
N
Y
Leaf
Expression Order
Of Magnitude
−1
0
1
2
3

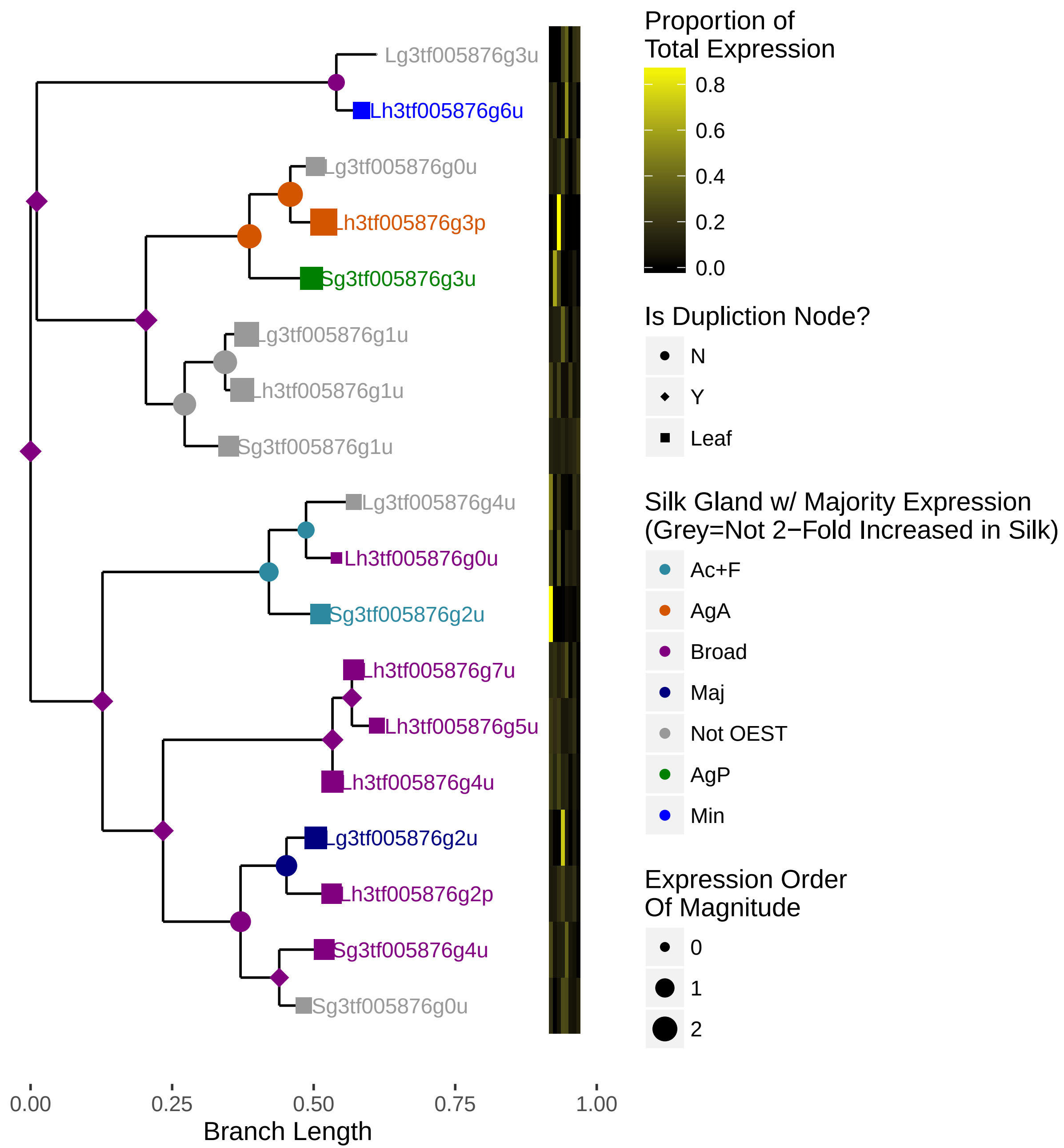

Lg3tf005876g3u
Lh3tf005876g6u
Lg3tf005876g0u
Lh3tf005876g3p
Sg3tf005876g3u
Lg3tf005876g1u
Lh3tf005876g1u
Sg3tf005876g1u
Lg3tf005876g4u
Lh3tf005876g0u
Sg3tf005876g2u
Lh3tf005876g7u
Lh3tf005876g5u
Lh3tf005876g4u
Lg3tf005876g2u
Lh3tf005876g2p
Sg3tf005876g4u
Sg3tf005876g0u
0.00
0.25
0.50
0.75
1.00
Branch Length
Proportion of Total Expression
0.8
0.6
0.4
0.2
0.0
Is Dupliction Node?
N
Y
Leaf
Silk Gland w/ Majority Expression (Grey=Not 2−Fold Increased in Silk)
Ac+F
AgA
Broad
Maj
Not OEST
AgP
Min
Expression Order Of Magnitude
0
1
2

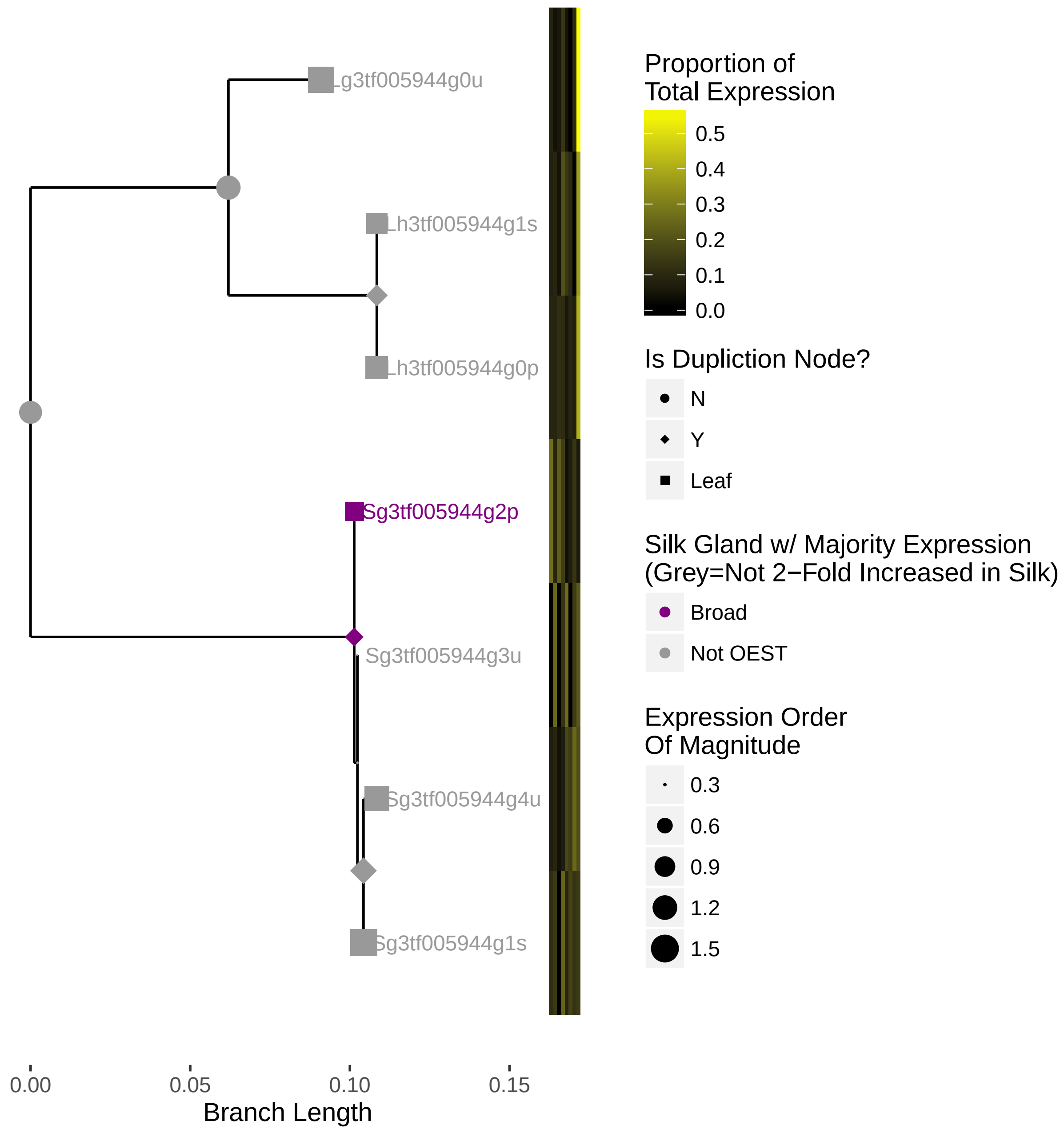

Lg3tf005944g0u
Lh3tf005944g1s
Lh3tf005944g0p
Sg3tf005944g2p
Sg3tf005944g3u
Sg3tf005944g4u
Sg3tf005944g1s
0.00
0.05
0.10
0.15
Branch Length
Proportion of
Total Expression
0.5
0.4
0.3
0.2
0.1
0.0
Is Dupliction Node?
N
Y
Leaf
Silk Gland w/ Majority Expression
(Grey=Not 2−Fold Increased in Silk)
Broad
Not OEST
Expression Order
Of Magnitude
0.3
0.6
0.9
1.2
1.5

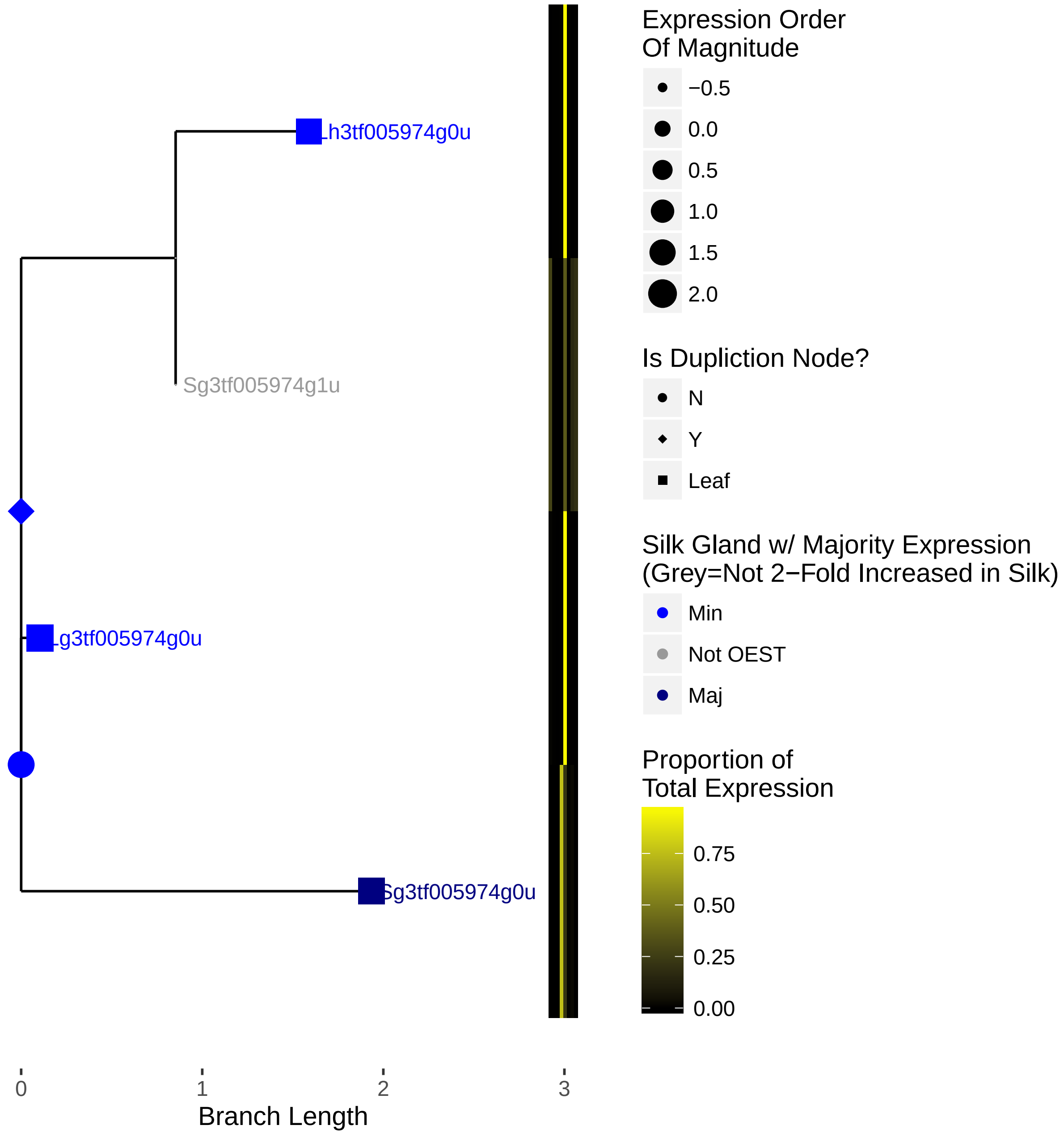

Lh3tf005974g0u
Sg3tf005974g1u
Lg3tf005974g0u
Sg3tf005974g0u
0
1
2
3
Branch Length
Expression Order Of Magnitude
−0.5
0.0
0.5
1.0
1.5
2.0
Is Dupliction Node?
N
Y
Leaf
Silk Gland w/ Majority Expression (Grey=Not 2−Fold Increased in Silk)
Min
Not OEST
Maj
Proportion of Total Expression
0.75
0.50
0.25
0.00

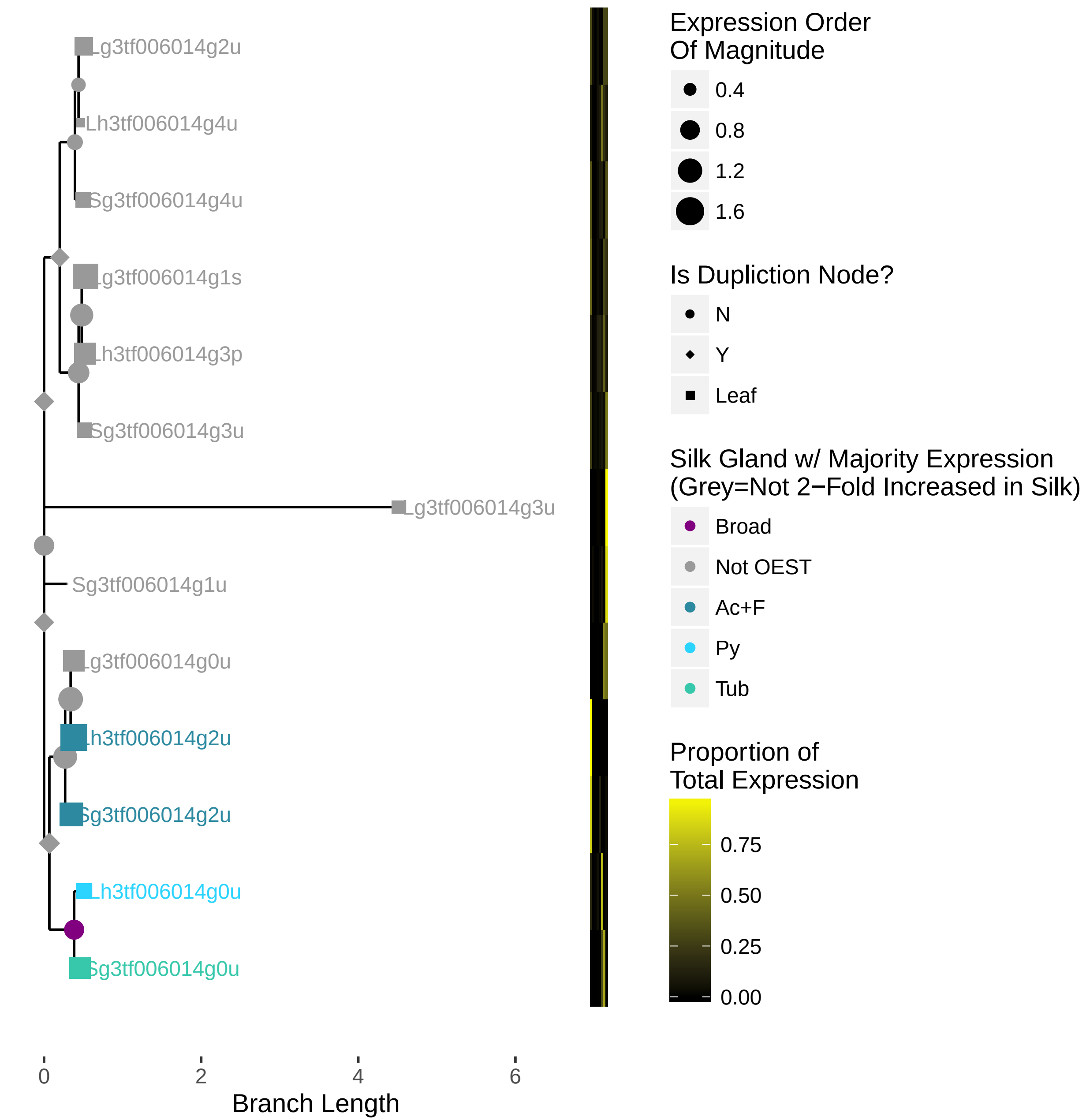
Lg3tf006014g2u
Lh3tf006014g4u
Sg3tf006014g4u
Lg3tf006014g1s
Lh3tf006014g3p
Sg3tf006014g3u
Lg3tf006014g3u
Sg3tf006014g1u
Lg3tf006014g0u
Lh3tf006014g2u
Sg3tf006014g2u
Lh3tf006014g0u
Sg3tf006014g0u
0
2
4
6
Branch Length
Expression Order Of Magnitude
0.4
0.8
1.2
1.6
Is Dupliction Node?
N
Y
Leaf
Silk Gland w/ Majority Expression (Grey=Not 2−Fold Increased in Silk)
Broad
Not OEST
Ac+F
Py
Tub
Proportion of Total Expression
0.75
0.50
0.25
0.00

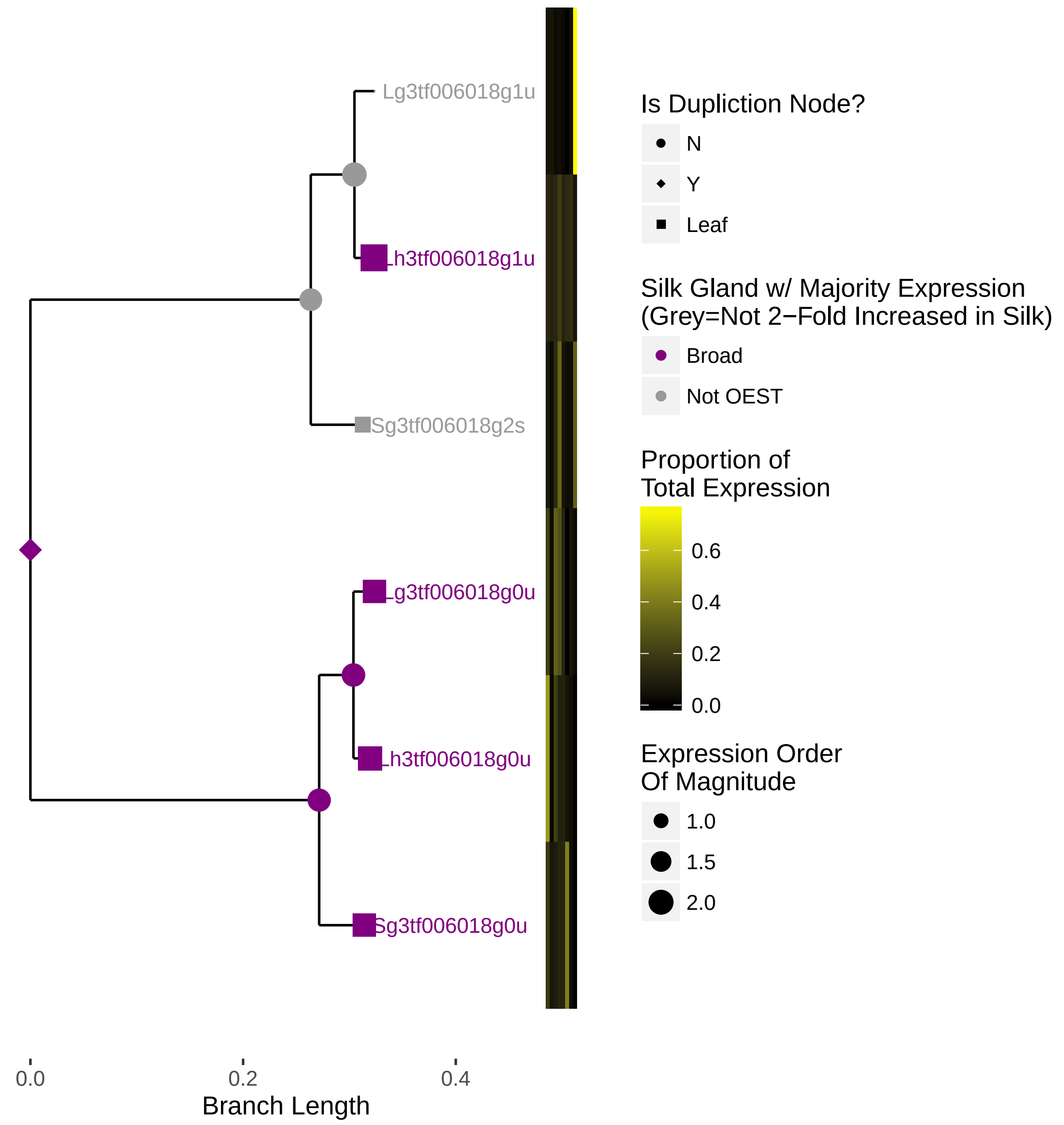
Lg3tf006018g1u
Lh3tf006018g1u
Sg3tf006018g2s
Lg3tf006018g0u
Lh3tf006018g0u
Sg3tf006018g0u
0.0
0.2
0.4
Branch Length
Is Dupliction Node?
N
Y
Leaf
Silk Gland w/ Majority Expression
(Grey=Not 2−Fold Increased in Silk)
Broad
Not OEST
Proportion of
Total Expression
0.6
0.4
0.2
0.0
Expression Order
Of Magnitude
1.0
1.5
2.0

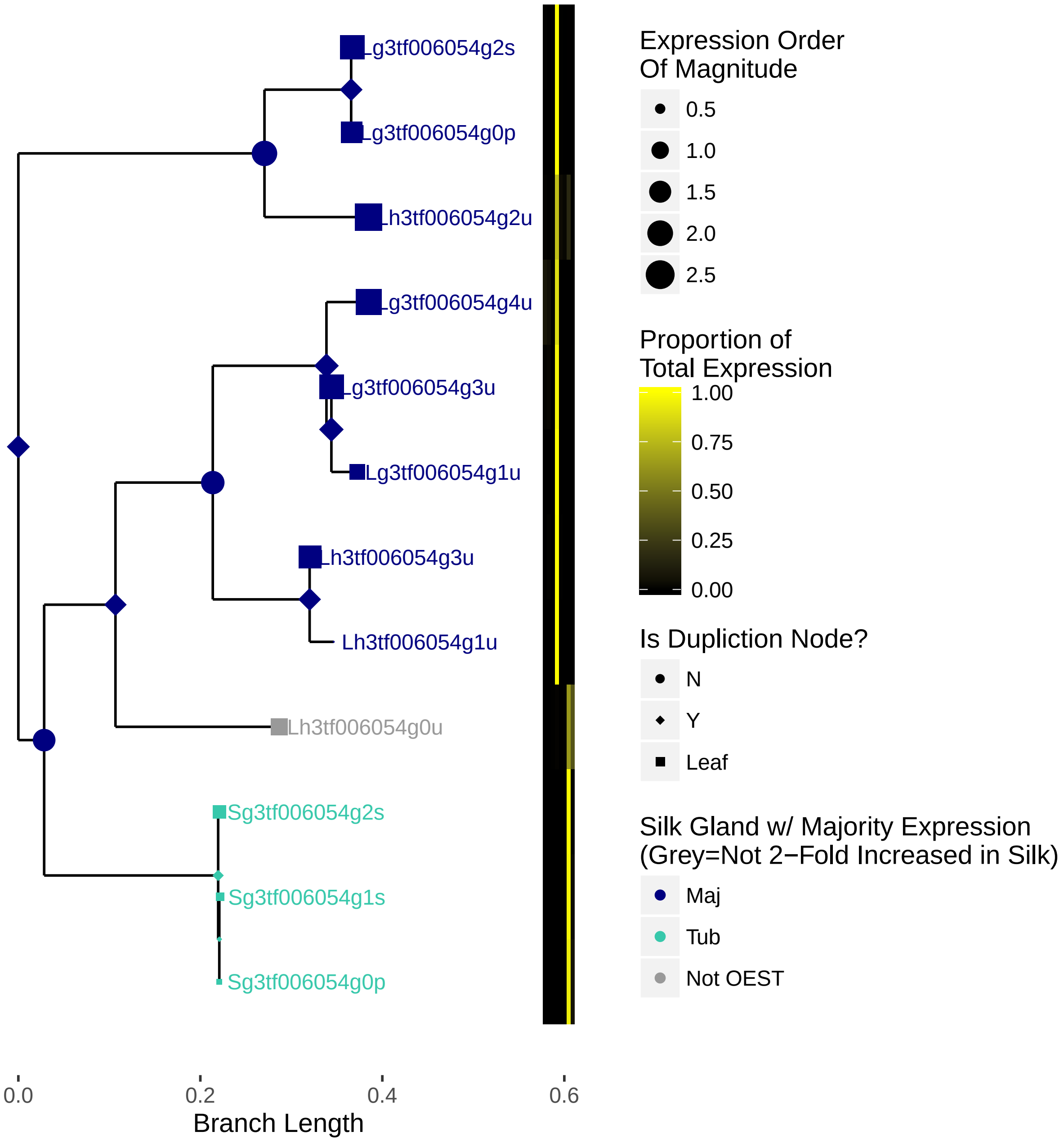

Lg3tf006054g2s
Lg3tf006054g0p
Lh3tf006054g2u
Lg3tf006054g4u
Lg3tf006054g3u
Lg3tf006054g1u
Lh3tf006054g3u
Lh3tf006054g1u
Lh3tf006054g0u
Sg3tf006054g2s
Sg3tf006054g1s
Sg3tf006054g0p
0.0
0.2
0.4
0.6
Branch Length
Expression Order Of Magnitude
0.5
1.0
1.5
2.0
2.5
Proportion of Total Expression
1.00
0.75
0.50
0.25
0.00
Is Dupliction Node?
N
Y
Leaf
Silk Gland w/ Majority Expression (Grey=Not 2−Fold Increased in Silk)
Maj
Tub
Not OEST

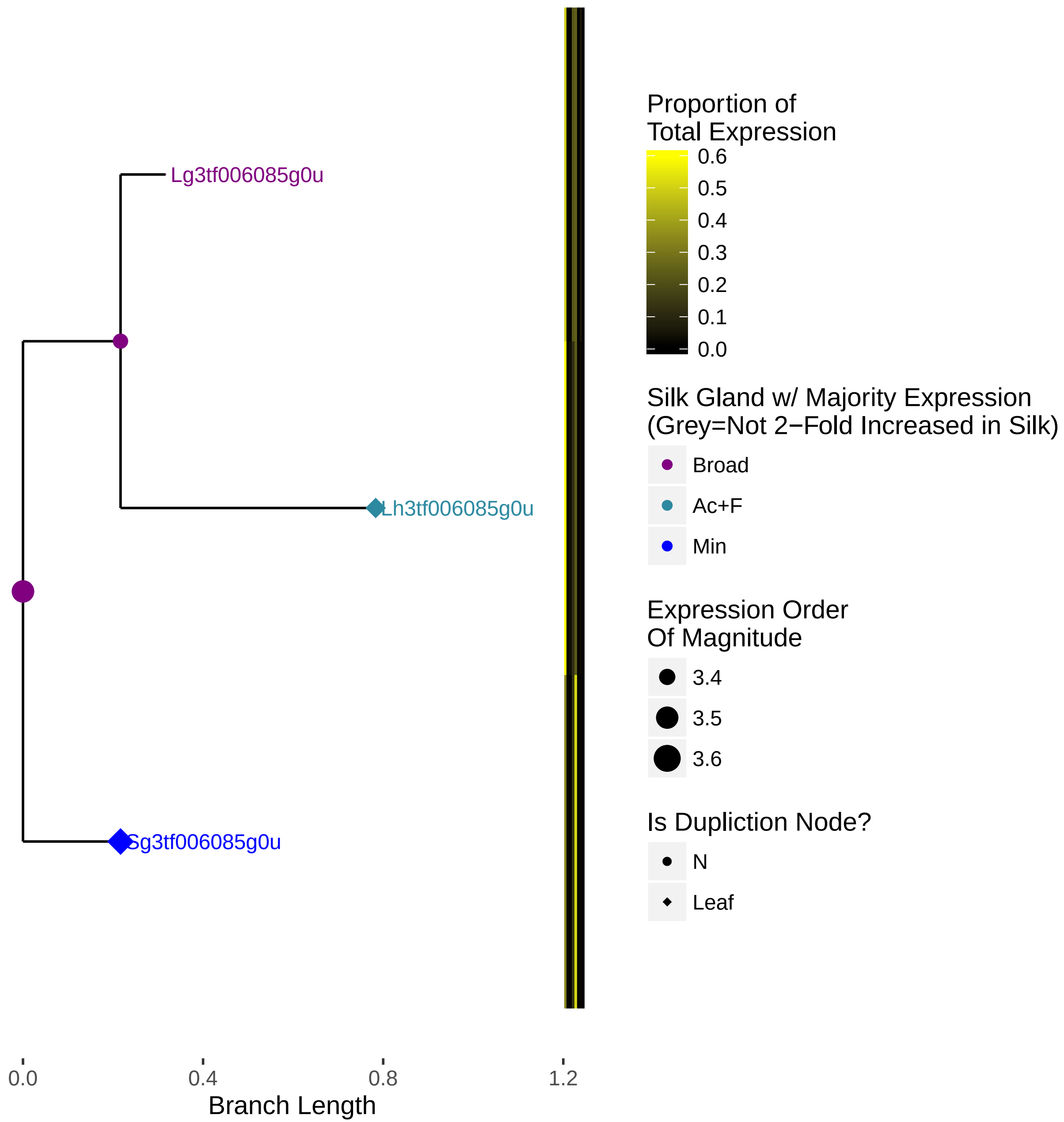

Lg3tf006085g0u
Lh3tf006085g0u
Sg3tf006085g0u
0.0
0.4
0.8
1.2
Branch Length
Proportion of
Total Expression
0.6
0.5
0.4
0.3
0.2
0.1
0.0
Silk Gland w/ Majority Expression
(Grey=Not 2−Fold Increased in Silk)
Broad
Ac+F
Min
Expression Order
Of Magnitude
3.4
3.5
3.6
Is Dupliction Node?
N
Leaf

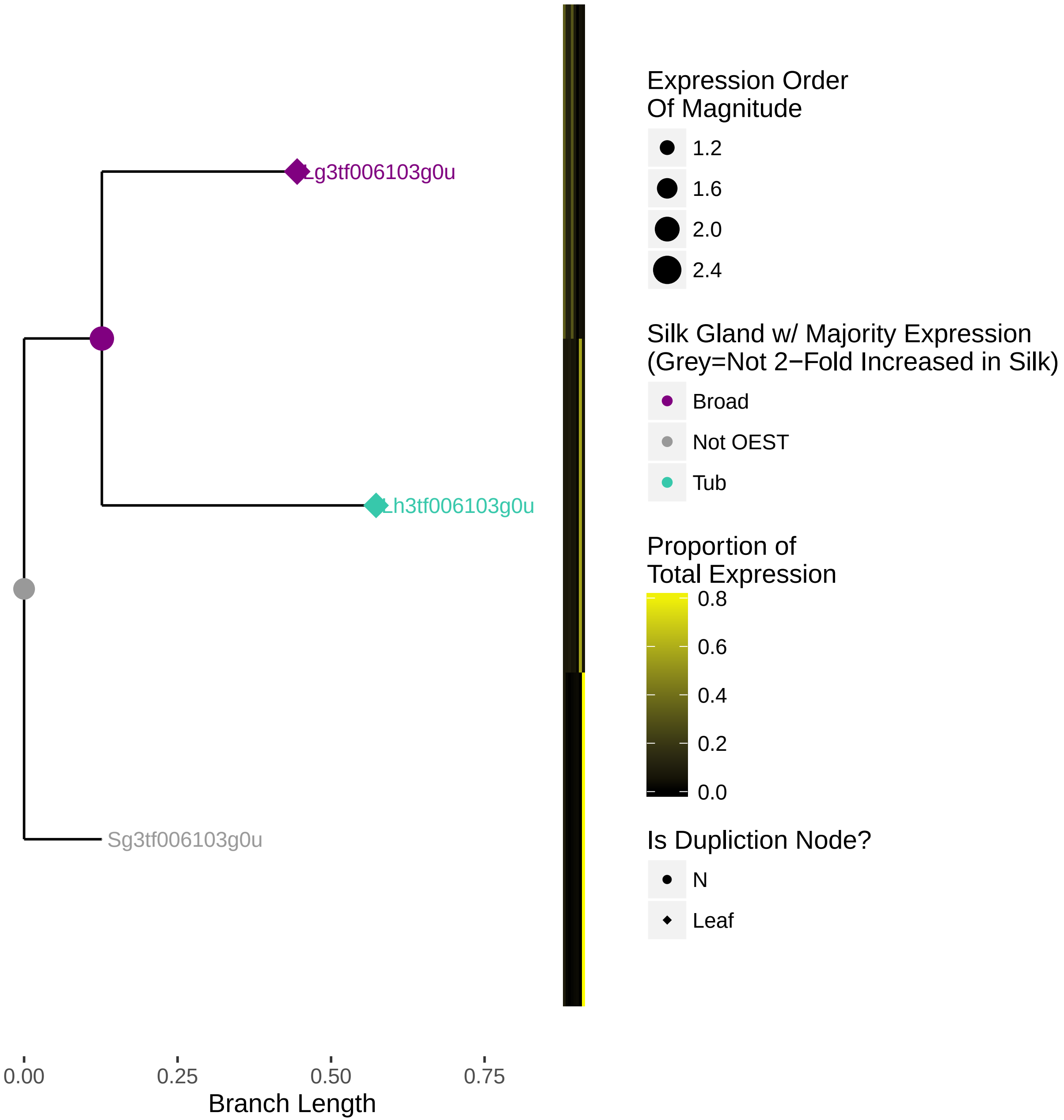

Lg3tf006103g0u
Lh3tf006103g0u
Sg3tf006103g0u
0.00
0.25
0.50
0.75
Branch Length
Expression Order
Of Magnitude
1.2
1.6
2.0
2.4
Silk Gland w/ Majority Expression
(Grey=Not 2−Fold Increased in Silk)
Broad
Not OEST
Tub
Proportion of
Total Expression
0.8
0.6
0.4
0.2
0.0
Is Dupliction Node?
N
Leaf

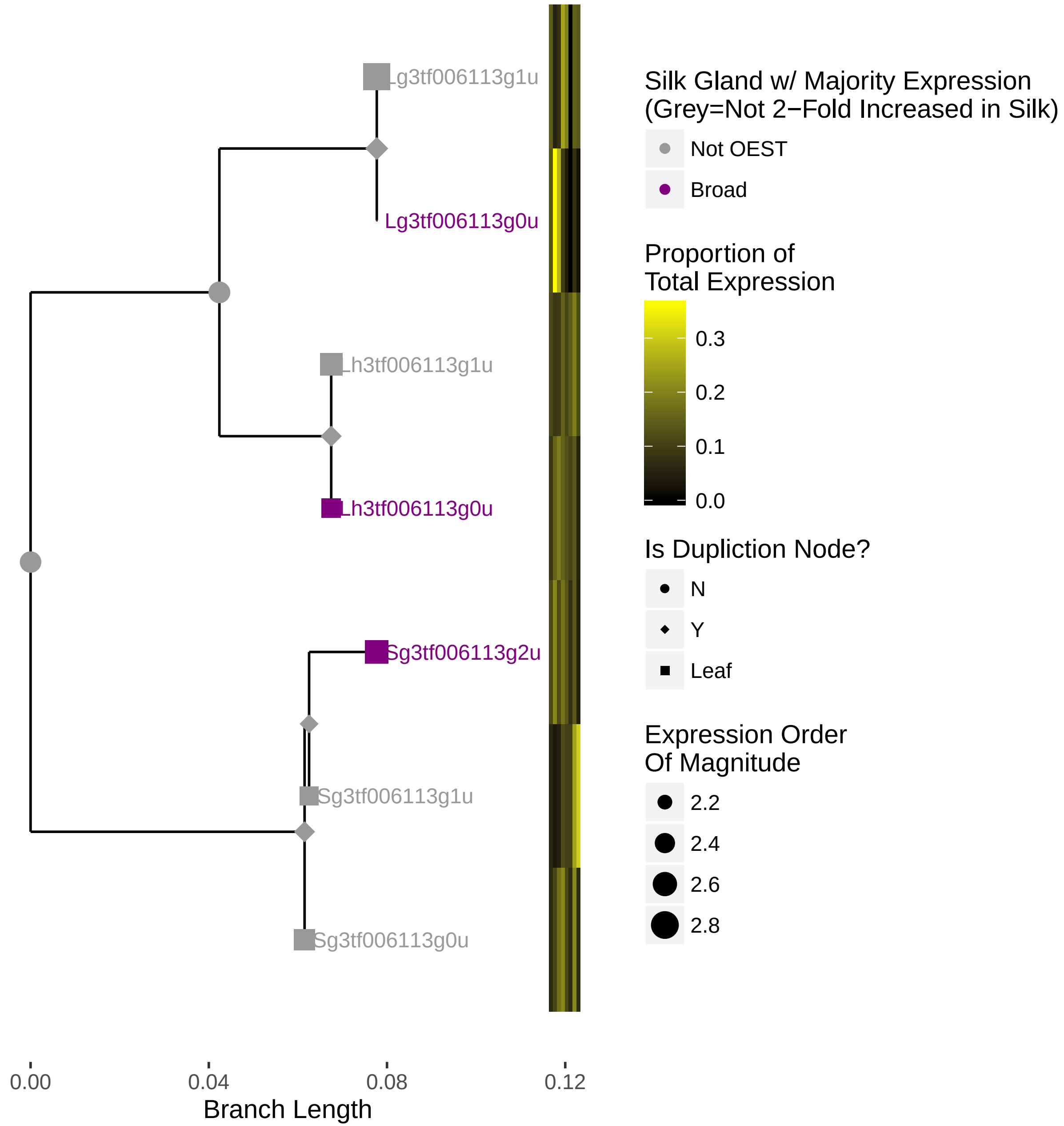

Lg3tf006113g1u
Lg3tf006113g0u
Lh3tf006113g1u
Lh3tf006113g0u
Sg3tf006113g2u
Sg3tf006113g1u
Sg3tf006113g0u
0.00
0.04
0.08
0.12
Branch Length
Silk Gland w/ Majority Expression
(Grey=Not 2−Fold Increased in Silk)
Not OEST
Broad
Proportion of
Total Expression
0.3
0.2
0.1
0.0
Is Dupliction Node?
N
Y
Leaf
Expression Order
Of Magnitude
2.2
2.4
2.6
2.8

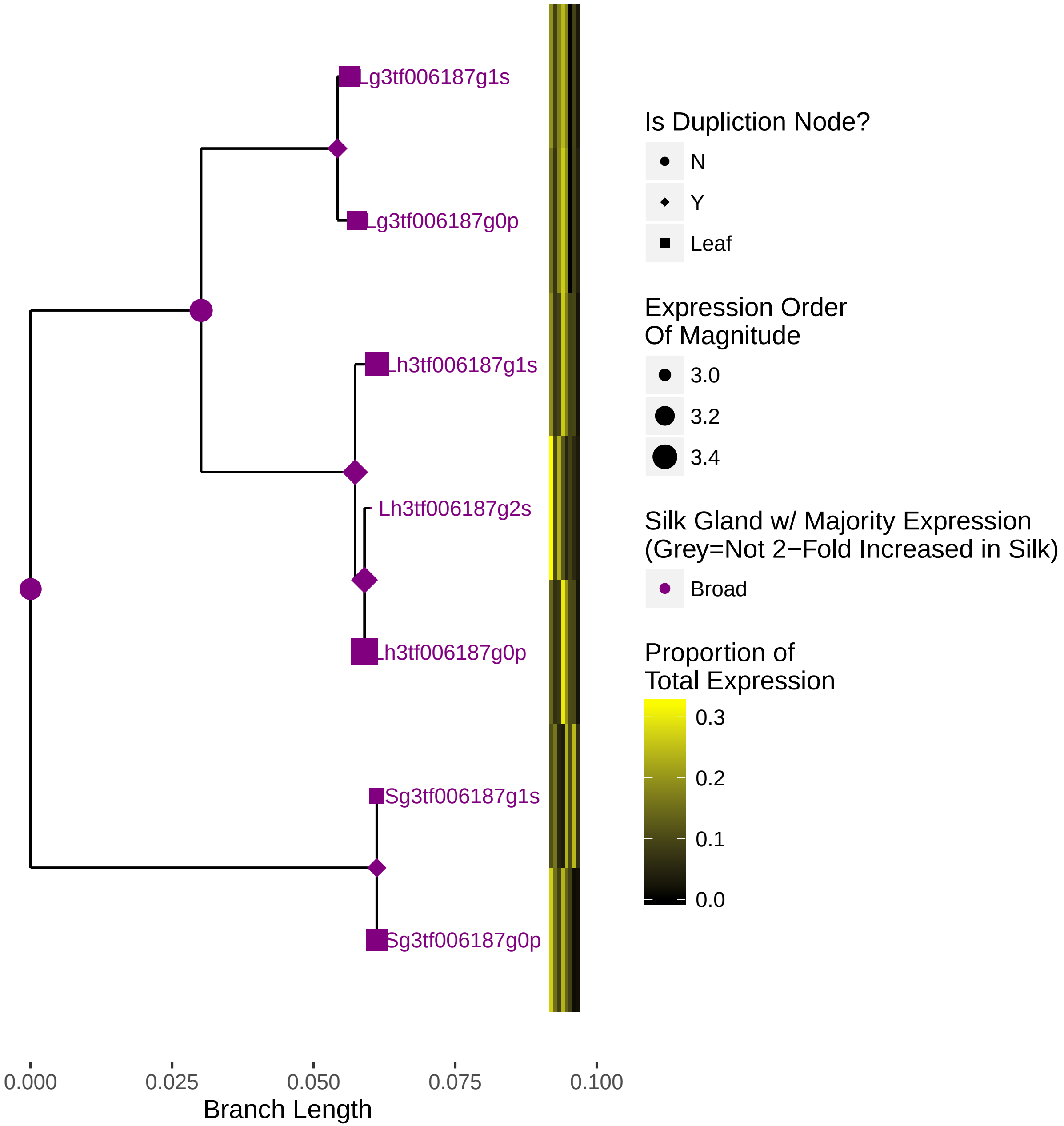
Lg3tf006187g1s
Lg3tf006187g0p
Lh3tf006187g1s
Lh3tf006187g2s
Lh3tf006187g0p
Sg3tf006187g1s
Sg3tf006187g0p
0.000
0.025
0.050
0.075
0.100
Branch Length
Is Dupliction Node?
N
Y
Leaf
Expression Order Of Magnitude
3.0
3.2
3.4
Silk Gland w/ Majority Expression (Grey=Not 2−Fold Increased in Silk)
Broad
Proportion of Total Expression
0.3
0.2
0.1
0.0

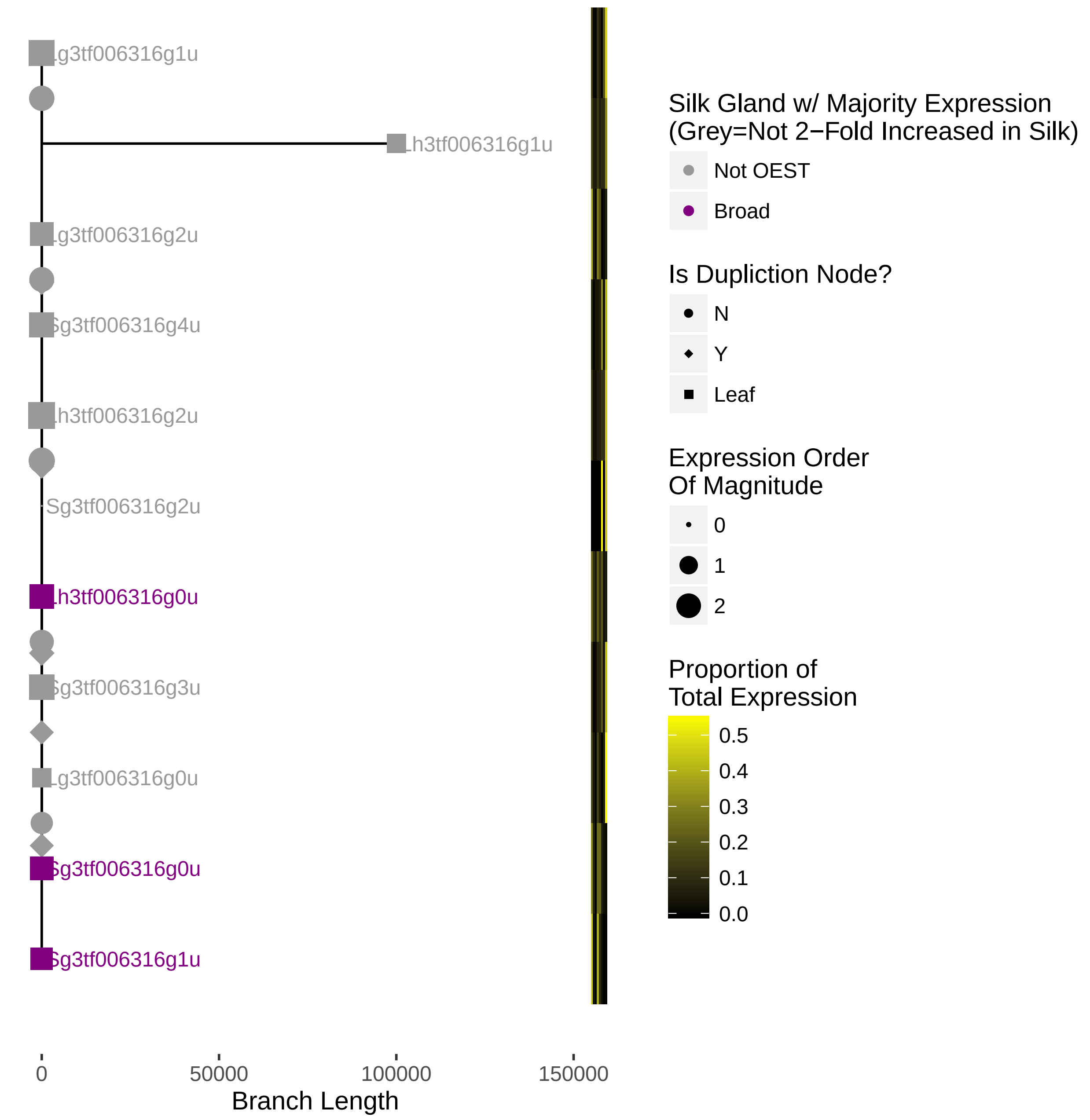

g3tf006316g1u
h3tf006316g1u
g3tf006316g2u
Sg3tf006316g4u
h3tf006316g2u
Sg3tf006316g2u
h3tf006316g0u
Sg3tf006316g3u
g3tf006316g0u
Sg3tf006316g0u
Sg3tf006316g1u
0
50000
100000
150000
Branch Length
Silk Gland w/ Majority Expression
(Grey=Not 2−Fold Increased in Silk)
Not OEST
Broad
Is Dupliction Node?
N
Y
Leaf
Expression Order
Of Magnitude
0
1
2
Proportion of
Total Expression
0.5
0.4
0.3
0.2
0.1
0.0

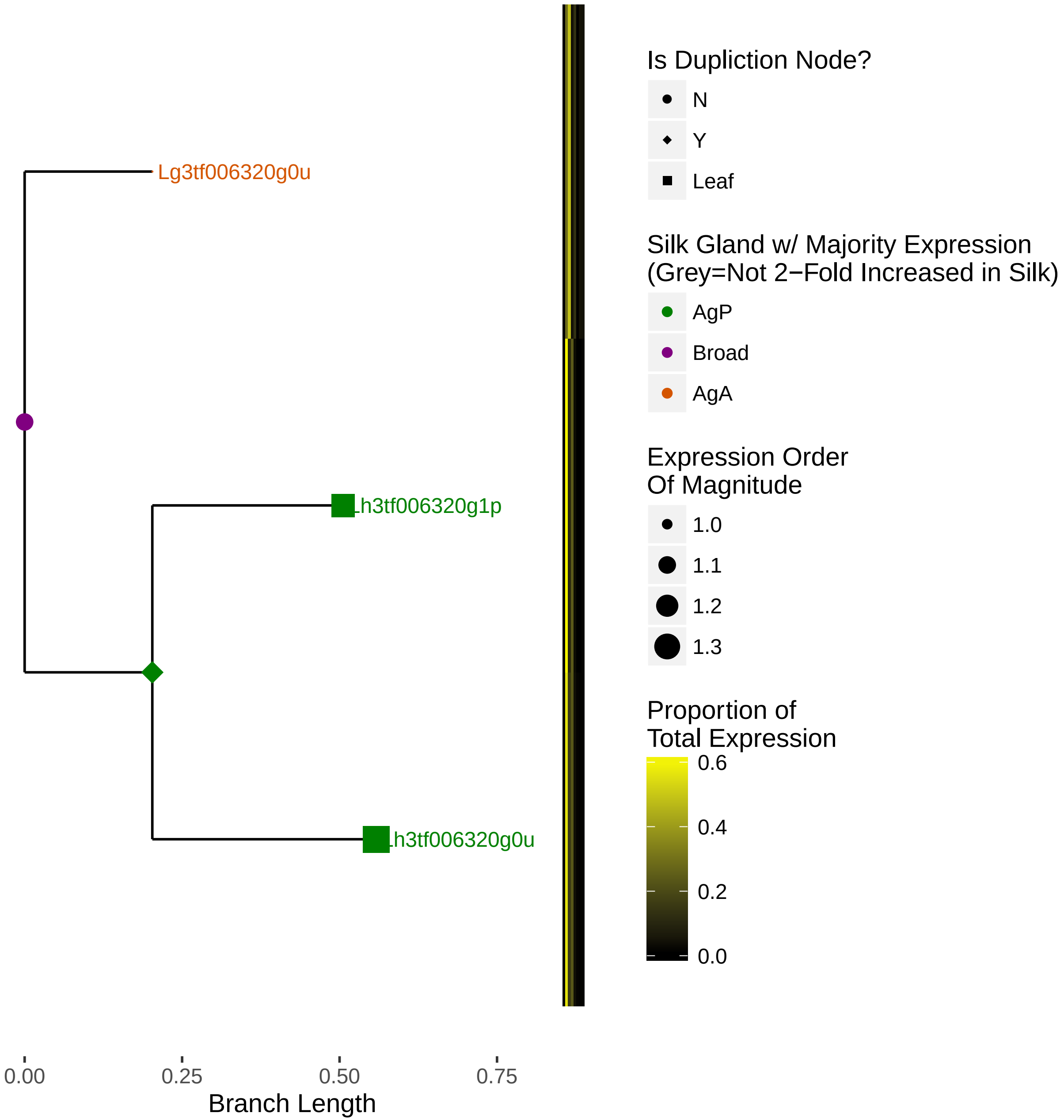

Lg3tf006320g0u
h3tf006320g1p
h3tf006320g0u
0.00
0.25
0.50
0.75
Branch Length
Is Dupliction Node?
N
Y
Leaf
Silk Gland w/ Majority Expression
(Grey=Not 2−Fold Increased in Silk)
AgP
Broad
AgA
Expression Order
Of Magnitude
1.0
1.1
1.2
1.3
Proportion of
Total Expression
0.6
0.4
0.2
0.0

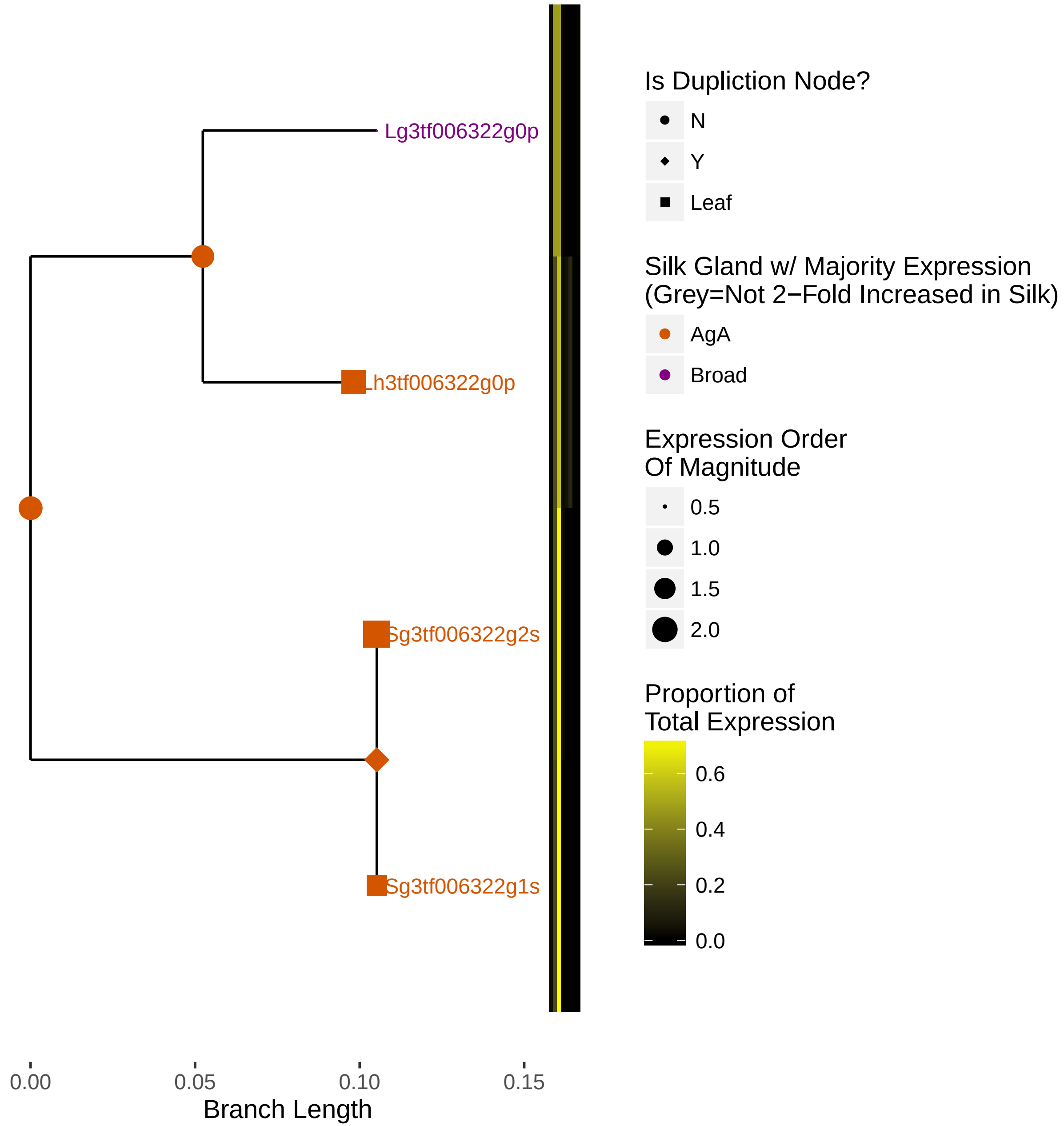
Lg3tf006322g0p
Lh3tf006322g0p
Sg3tf006322g2s
Sg3tf006322g1s
Is Dupliction Node?
N
Y
Leaf
Silk Gland w/ Majority Expression
(Grey=Not 2−Fold Increased in Silk)
AgA
Broad
Expression Order
Of Magnitude
0.5
1.0
1.5
2.0
Proportion of
Total Expression
0.6
0.4
0.2
0.0
0.00
0.05
0.10
0.15
Branch Length

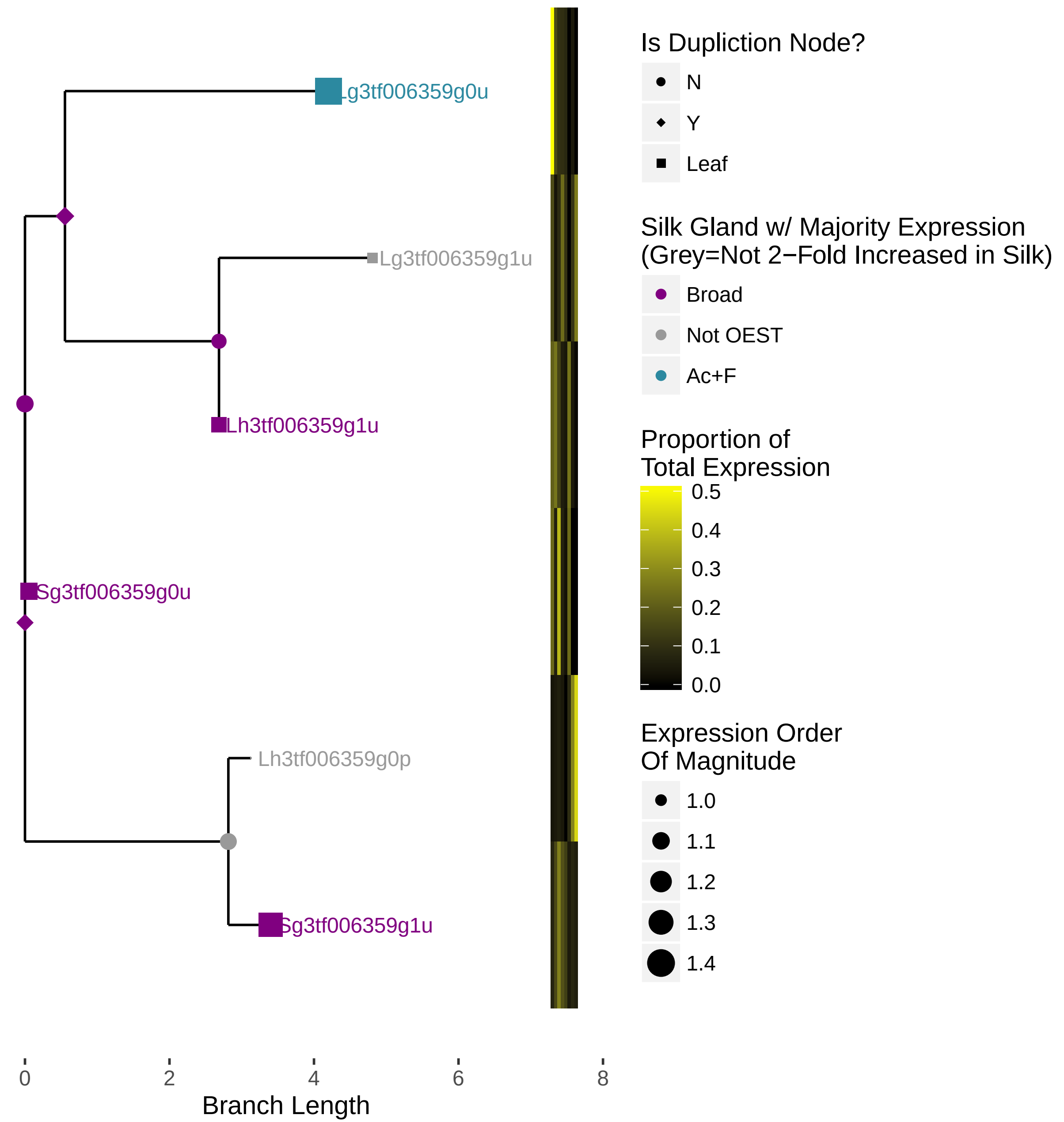
Lg3tf006359g0u
Lg3tf006359g1u
Lh3tf006359g1u
Sg3tf006359g0u
Lh3tf006359g0p
Sg3tf006359g1u
0
2
4
6
8
Branch Length
Is Dupliction Node?
N
Y
Leaf
Silk Gland w/ Majority Expression
(Grey=Not 2−Fold Increased in Silk)
Broad
Not OEST
Ac+F
Proportion of
Total Expression
0.5
0.4
0.3
0.2
0.1
0.0
Expression Order
Of Magnitude
1.0
1.1
1.2
1.3
1.4

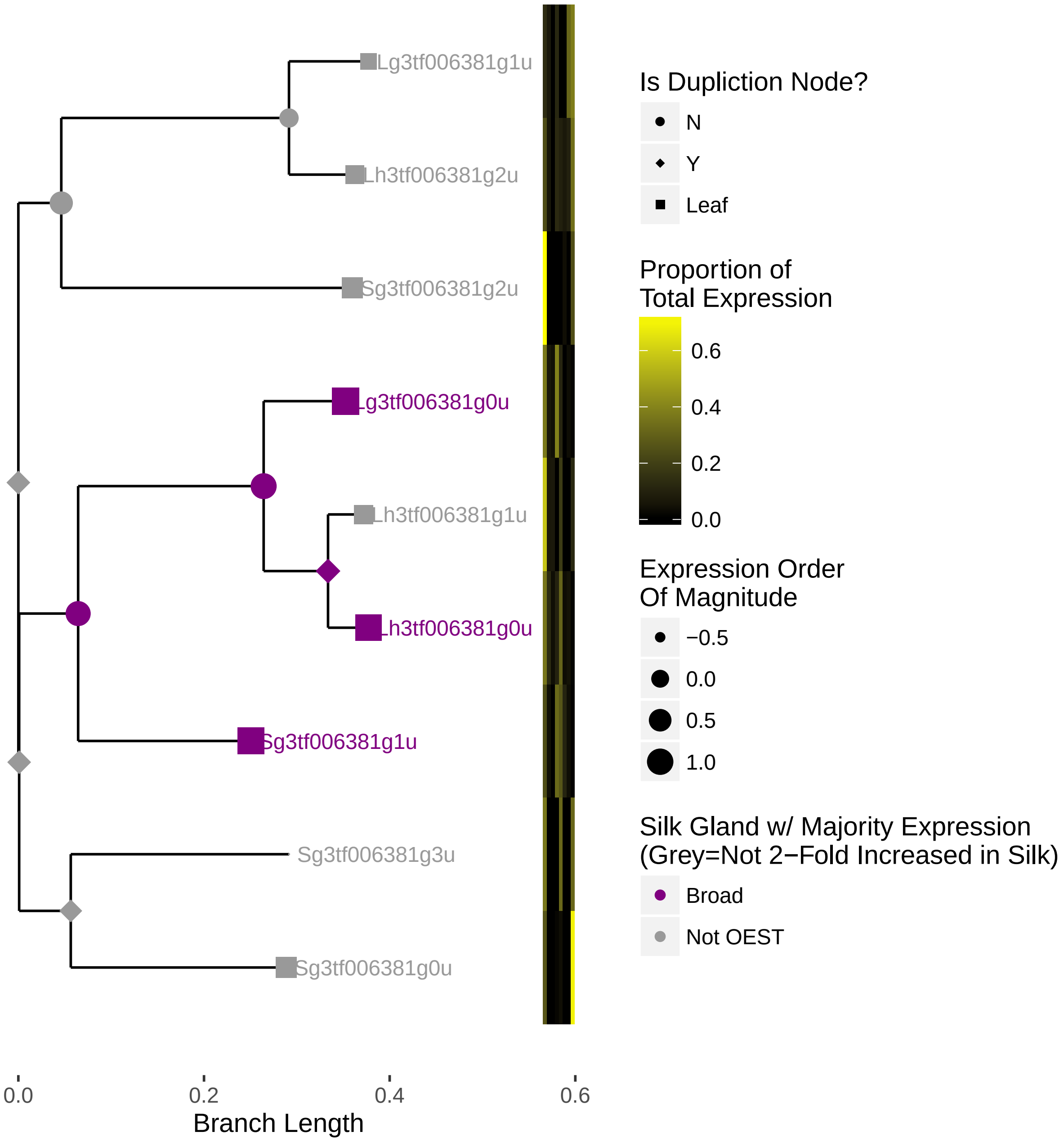

Lg3tf006381g1u
Lh3tf006381g2u
Sg3tf006381g2u
Lg3tf006381g0u
Lh3tf006381g1u
Lh3tf006381g0u
Sg3tf006381g1u
Sg3tf006381g3u
Sg3tf006381g0u
0.0
0.2
0.4
0.6
Branch Length
Is Dupliction Node?
N
Y
Leaf
Proportion of Total Expression
0.6
0.4
0.2
0.0
Expression Order Of Magnitude
−0.5
0.0
0.5
1.0
Silk Gland w/ Majority Expression (Grey=Not 2−Fold Increased in Silk)
Broad
Not OEST

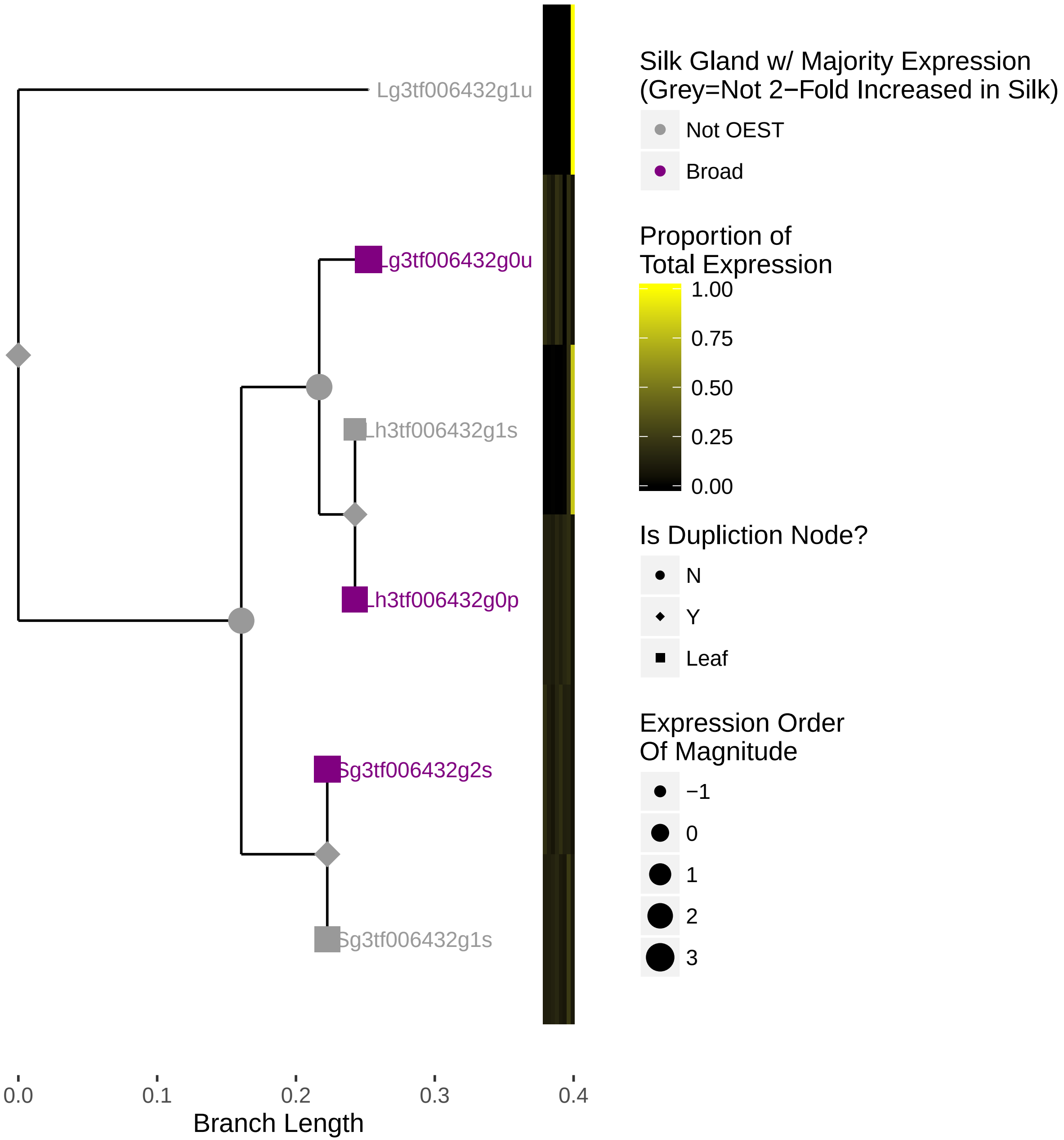
Lg3tf006432g1u
Lg3tf006432g0u
Lh3tf006432g1s
Lh3tf006432g0p
Sg3tf006432g2s
Sg3tf006432g1s
0.0
0.1
0.2
0.3
0.4
Branch Length
Silk Gland w/ Majority Expression
(Grey=Not 2−Fold Increased in Silk)
Not OEST
Broad
Proportion of
Total Expression
1.00
0.75
0.50
0.25
0.00
Is Dupliction Node?
N
Y
Leaf
Expression Order
Of Magnitude
−1
0
1
2
3

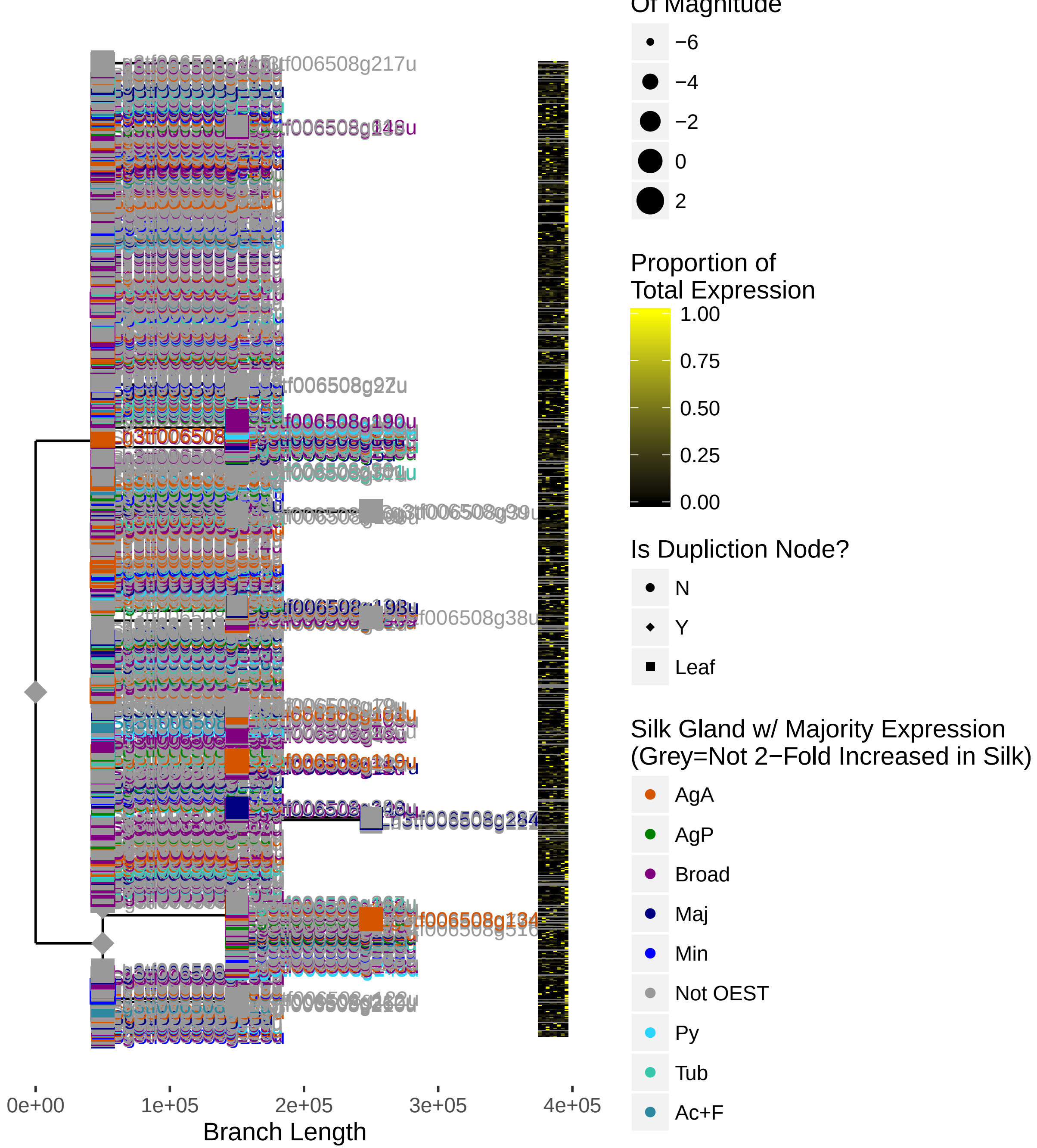

Of Magnitude
−6
−4
−2
0
2
Proportion of Total Expression
1.00
0.75
0.50
0.25
0.00
Is Dupliction Node?
N
Y
Leaf
Silk Gland w/ Majority Expression (Grey=Not 2−Fold Increased in Silk)
AgA
AgP
Broad
Maj
Min
Not OEST
Py
Tub
Ac+F
0e+00
1e+05
2e+05
3e+05
4e+05
Branch Length

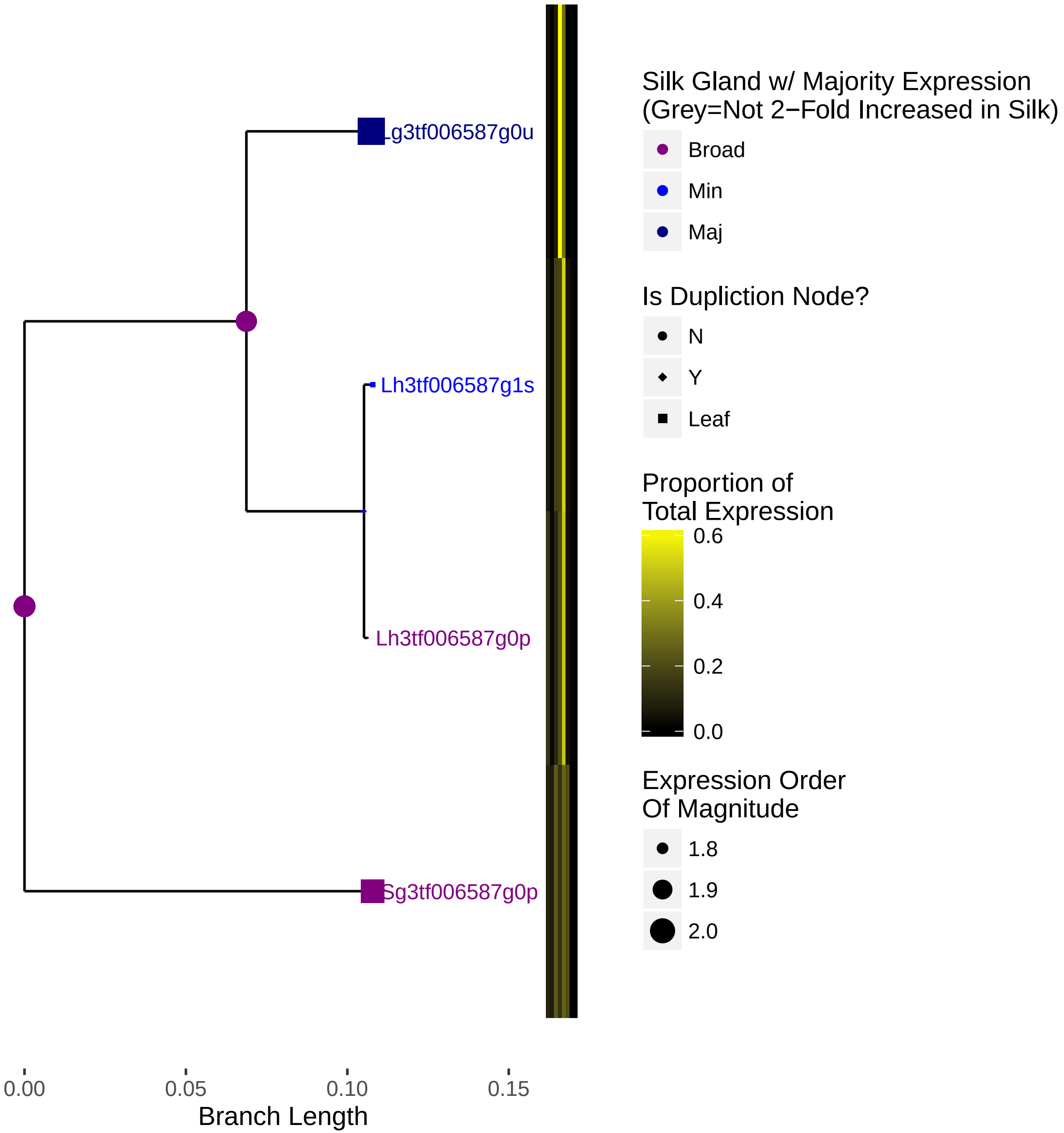

Lg3tf006587g0u
Lh3tf006587g1s
Lh3tf006587g0p
Sg3tf006587g0p
0.00
0.05
0.10
0.15
Branch Length
Silk Gland w/ Majority Expression
(Grey=Not 2−Fold Increased in Silk)
Broad
Min
Maj
Is Dupliction Node?
N
Y
Leaf
Proportion of
Total Expression
0.6
0.4
0.2
0.0
Expression Order
Of Magnitude
1.8
1.9
2.0

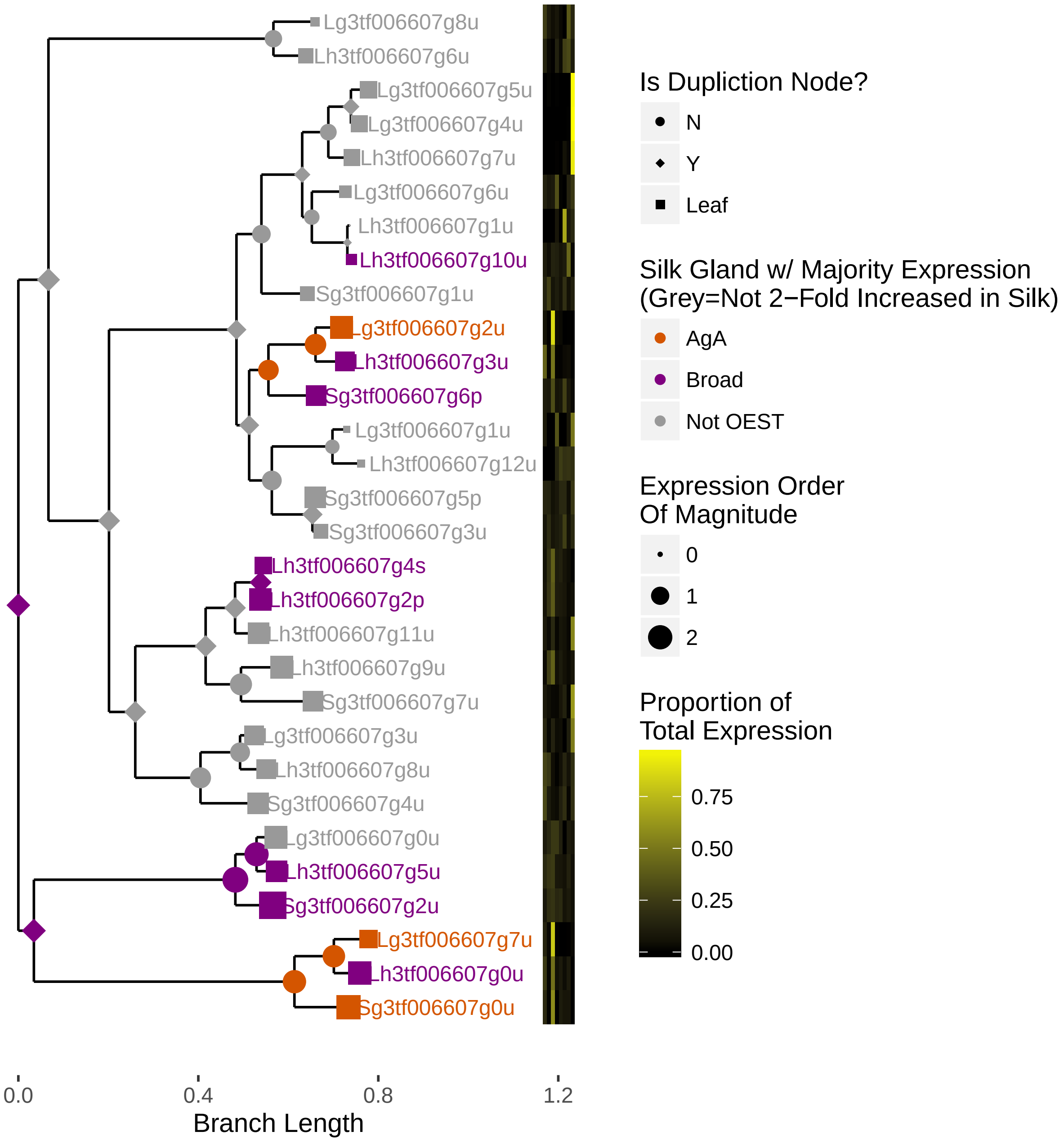

Lg3tf006607g8u
Lh3tf006607g6u
Lg3tf006607g5u
Lg3tf006607g4u
Lh3tf006607g7u
Lg3tf006607g6u
Lh3tf006607g1u
Lh3tf006607g10u
Sg3tf006607g1u
Lg3tf006607g2u
Lh3tf006607g3u
Sg3tf006607g6p
Lg3tf006607g1u
Lh3tf006607g12u
Sg3tf006607g5p
Sg3tf006607g3u
Lh3tf006607g4s
Lh3tf006607g2p
Lh3tf006607g11u
Lh3tf006607g9u
Sg3tf006607g7u
Lg3tf006607g3u
Lh3tf006607g8u
Sg3tf006607g4u
Lg3tf006607g0u
Lh3tf006607g5u
Sg3tf006607g2u
Lg3tf006607g7u
Lh3tf006607g0u
Sg3tf006607g0u
0.0
0.4
0.8
1.2
Branch Length
Is Dupliction Node?
N
Y
Leaf
Silk Gland w/ Majority Expression
(Grey=Not 2−Fold Increased in Silk)
AgA
Broad
Not OEST
Expression Order
Of Magnitude
0
1
2
Proportion of
Total Expression
0.75
0.50
0.25
0.00

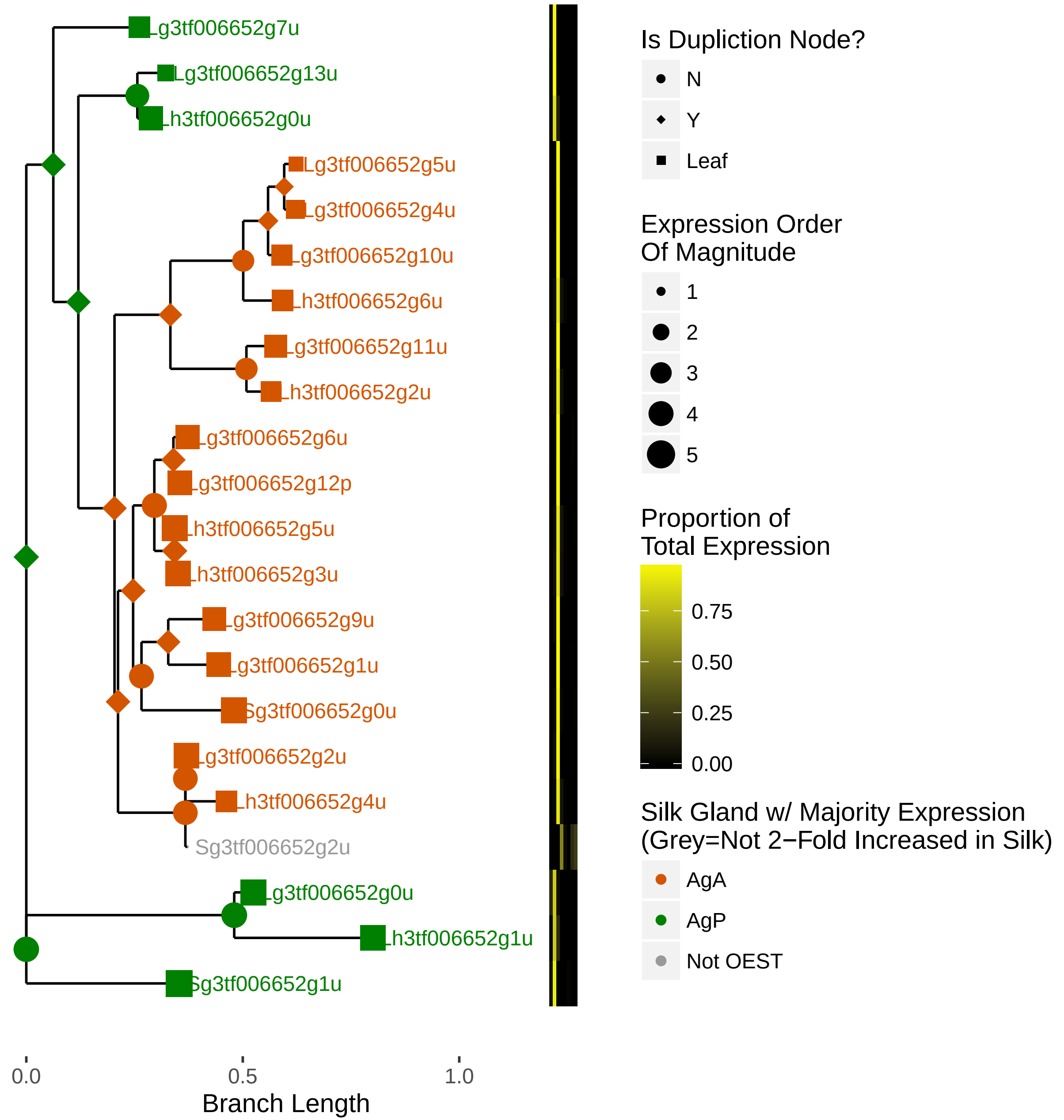

Lg3tf006652g7u
Lg3tf006652g13u
Lh3tf006652g0u
Lg3tf006652g5u
Lg3tf006652g4u
Lg3tf006652g10u
Lh3tf006652g6u
Lg3tf006652g11u
Lh3tf006652g2u
Lg3tf006652g6u
Lg3tf006652g12p
Lh3tf006652g5u
Lh3tf006652g3u
Lg3tf006652g9u
Lg3tf006652g1u
Sg3tf006652g0u
Lg3tf006652g2u
Lh3tf006652g4u
Sg3tf006652g2u
Lg3tf006652g0u
Lh3tf006652g1u
Sg3tf006652g1u
0.0
0.5
1.0
Branch Length
Is Dupliction Node?
N
Y
Leaf
Expression Order
Of Magnitude
1
2
3
4
5
Proportion of
Total Expression
0.75
0.50
0.25
0.00
Silk Gland w/ Majority Expression
(Grey=Not 2−Fold Increased in Silk)
AgA
AgP
Not OEST

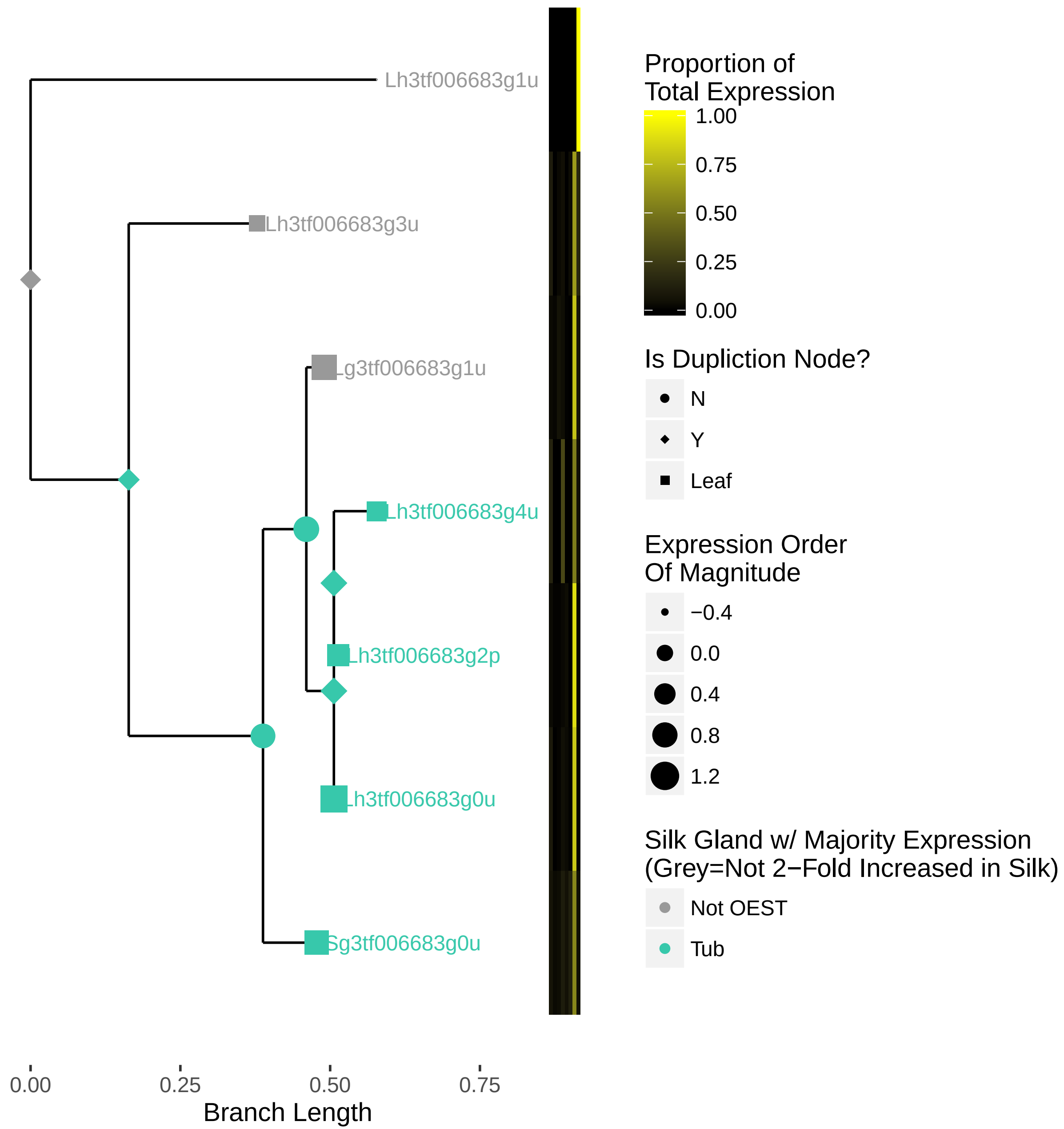

Lh3tf006683g1u
Lh3tf006683g3u
Lg3tf006683g1u
Lh3tf006683g4u
Lh3tf006683g2p
Lh3tf006683g0u
Sg3tf006683g0u
0.00
0.25
0.50
0.75
Branch Length
Proportion of
Total Expression
1.00
0.75
0.50
0.25
0.00
Is Dupliction Node?
N
Y
Leaf
Expression Order
Of Magnitude
−0.4
0.0
0.4
0.8
1.2
Silk Gland w/ Majority Expression
(Grey=Not 2−Fold Increased in Silk)
Not OEST
Tub

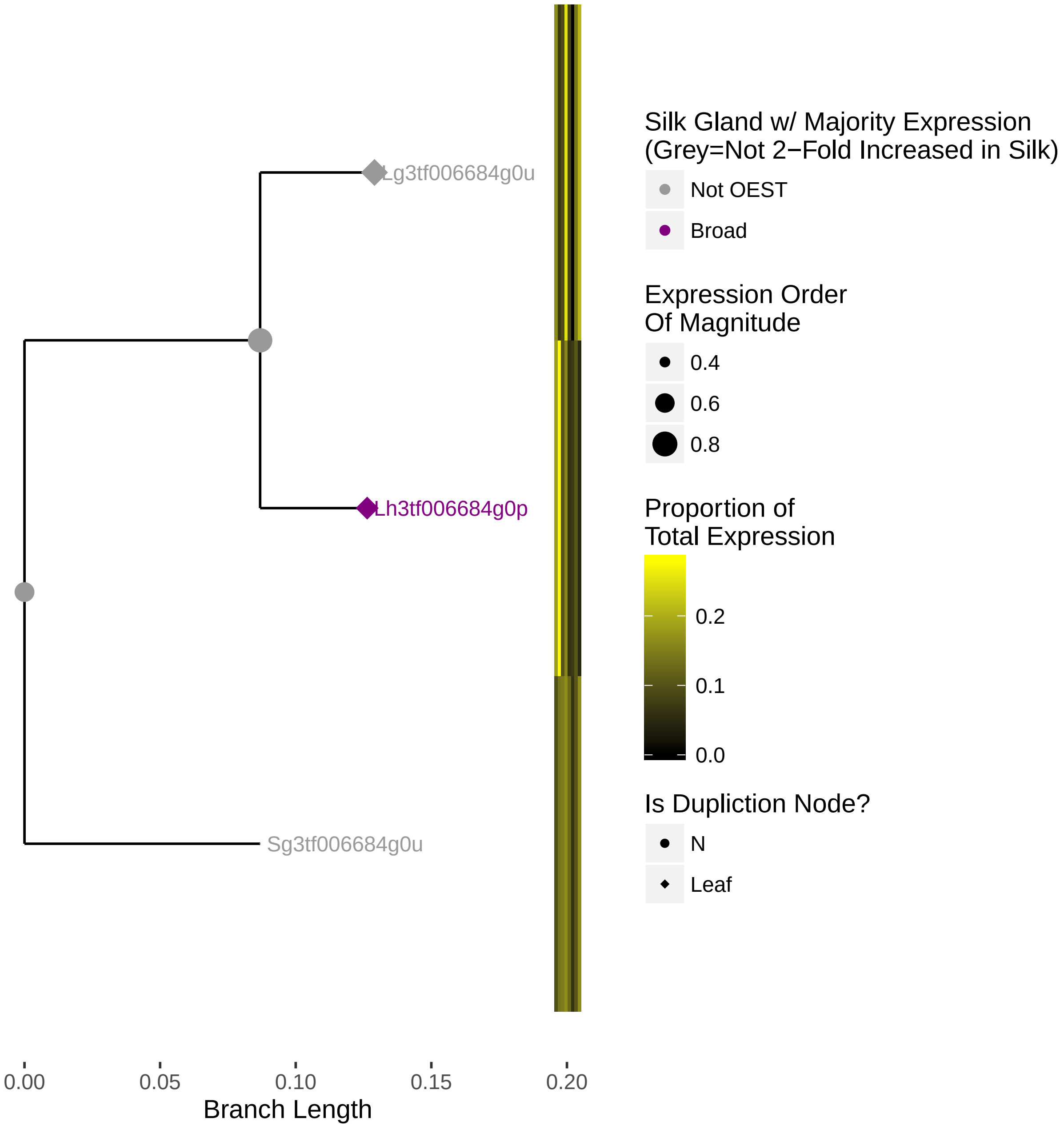

Lg3tf006684g0u
Lh3tf006684g0p
Sg3tf006684g0u
0.00
0.05
0.10
0.15
0.20
Branch Length
Silk Gland w/ Majority Expression
(Grey=Not 2−Fold Increased in Silk)
Not OEST
Broad
Expression Order
Of Magnitude
0.4
0.6
0.8
Proportion of
Total Expression
0.2
0.1
0.0
Is Dupliction Node?
N
Leaf

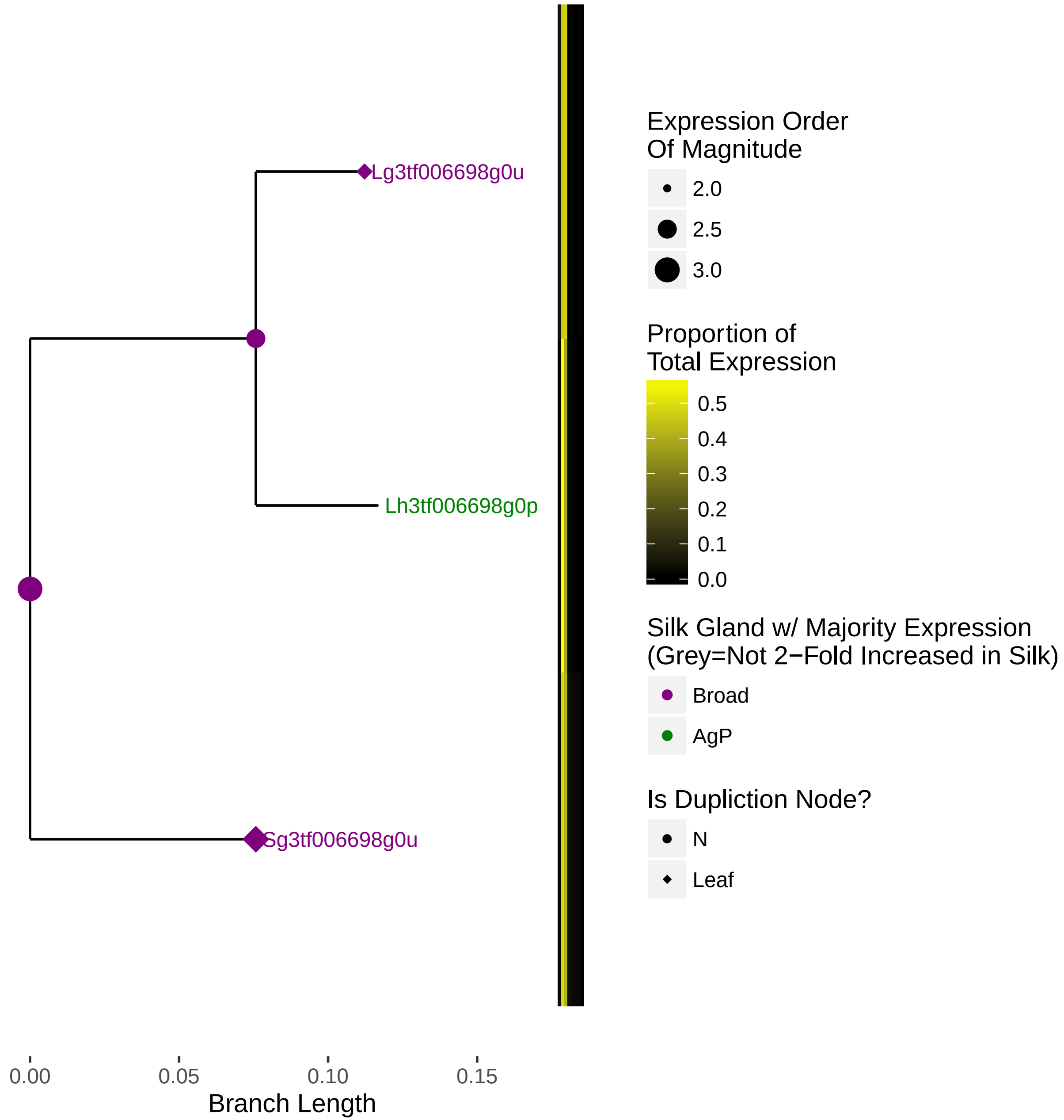

Lg3tf006698g0u
Lh3tf006698g0p
Sg3tf006698g0u
0.00
0.05
0.10
0.15
Branch Length
Expression Order Of Magnitude
2.0
2.5
3.0
Proportion of Total Expression
0.5
0.4
0.3
0.2
0.1
0.0
Silk Gland w/ Majority Expression (Grey=Not 2−Fold Increased in Silk)
Broad
AgP
Is Dupliction Node?
N
Leaf

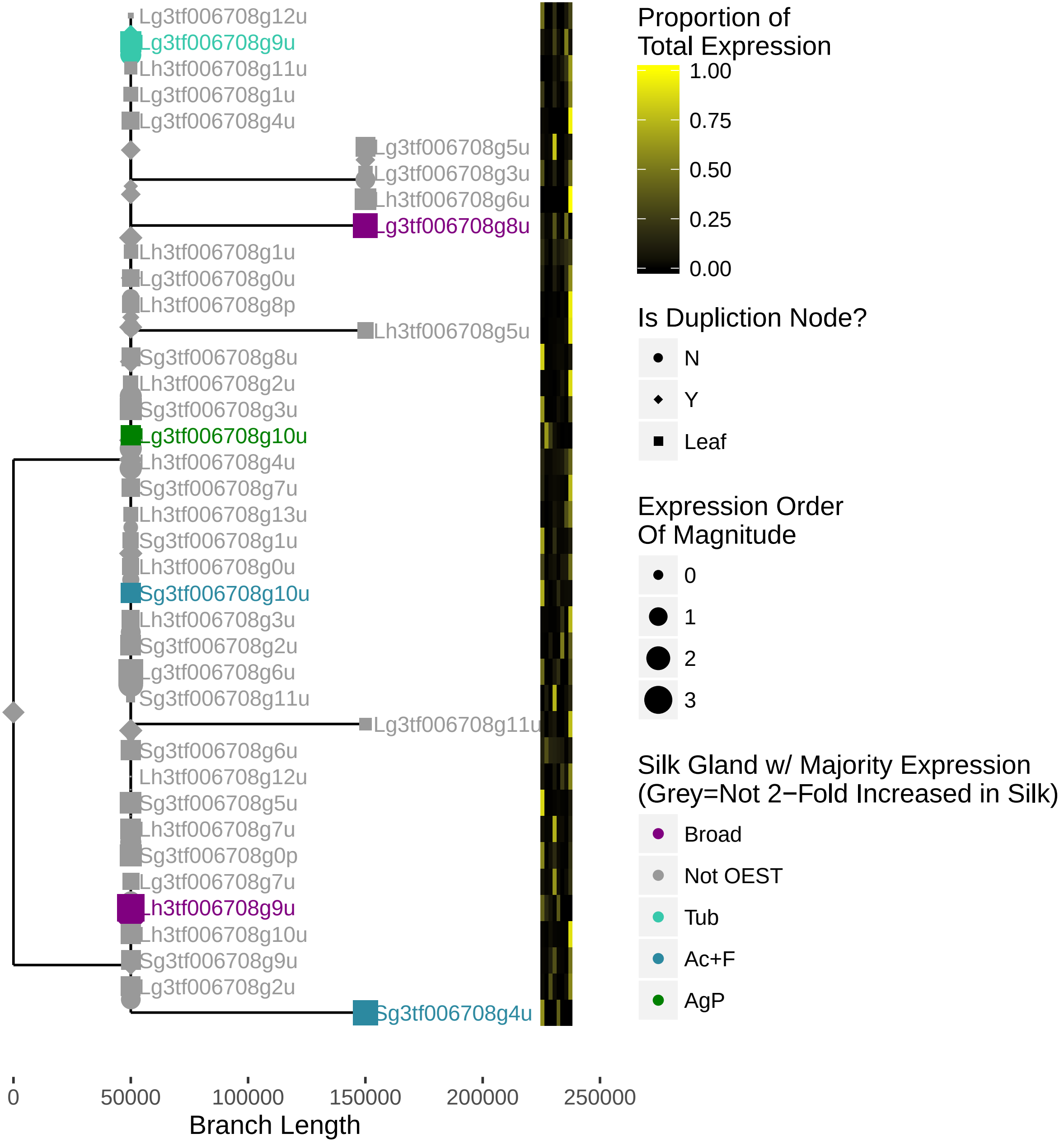

Lg3tf006708g12u
g3tf006708g9u
Lh3tf006708g11u
Lg3tf006708g1u
Lg3tf006708g4u
Lg3tf006708g5u
Lg3tf006708g3u
h3tf006708g6u
g3tf006708g8u
Lh3tf006708g1u
Lg3tf006708g0u
Lh3tf006708g8p
Lh3tf006708g5u
Sg3tf006708g8u
Lh3tf006708g2u
Sg3tf006708g3u
g3tf006708g10u
Lh3tf006708g4u
Sg3tf006708g7u
Lh3tf006708g13u
Sg3tf006708g1u
Lh3tf006708g0u
Sg3tf006708g10u
Lh3tf006708g3u
Sg3tf006708g2u
g3tf006708g6u
Sg3tf006708g11u
Lg3tf006708g11u
Sg3tf006708g6u
Lh3tf006708g12u
Sg3tf006708g5u
h3tf006708g7u
Sg3tf006708g0p
Lg3tf006708g7u
h3tf006708g9u
h3tf006708g10u
Sg3tf006708g9u
g3tf006708g2u
Sg3tf006708g4u
0
50000
100000
150000
200000
250000
Branch Length
Proportion of
Total Expression
1.00
0.75
0.50
0.25
0.00
Is Duplicion Node?
N
Y
Leaf
Expression Order
Of Magnitude
0
1
2
3
Silk Gland w/ Majority Expression
(Grey=Not 2−Fold Increased in Silk)
Broad
Not OEST
Tub
Ac+F
AgP

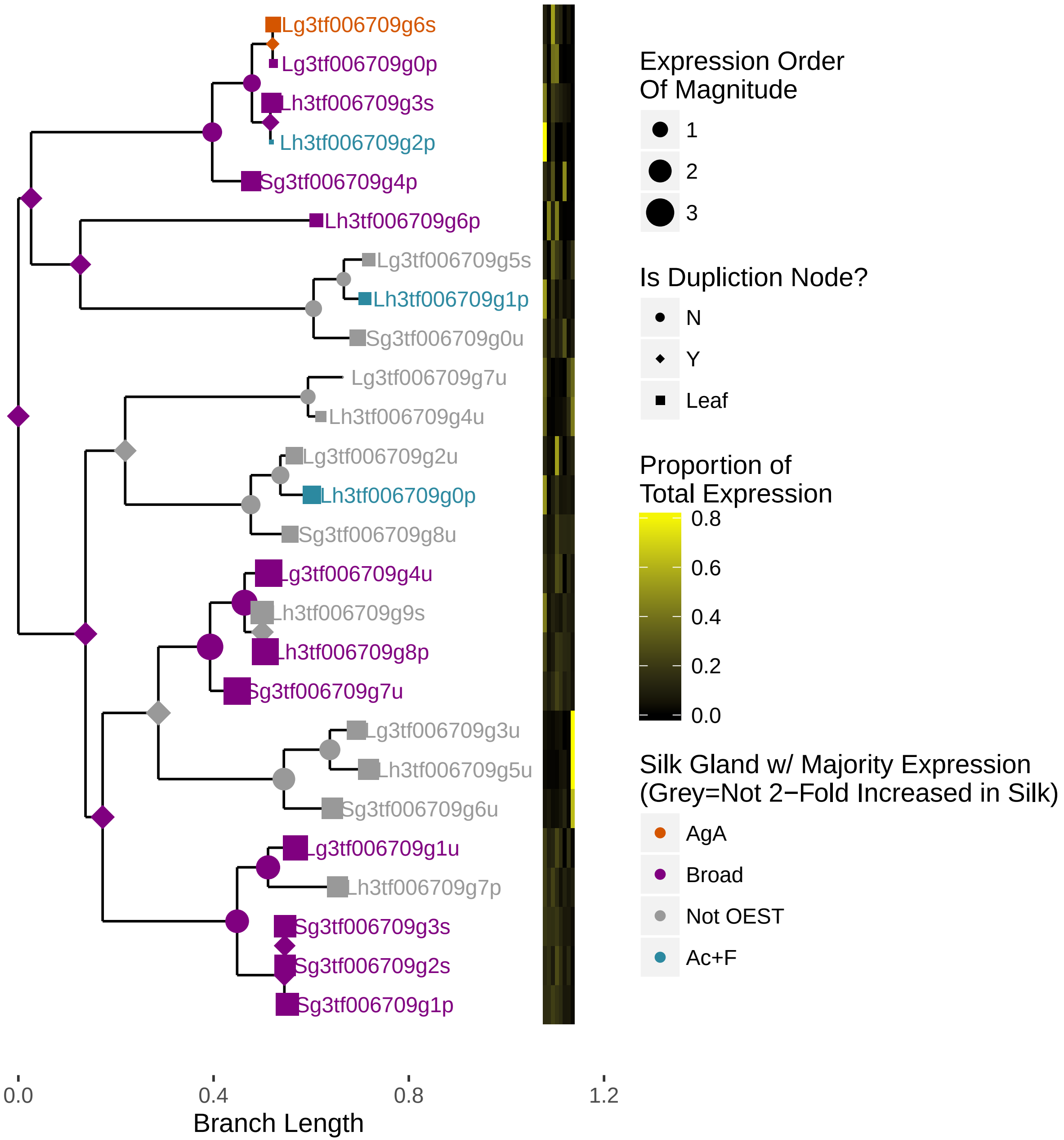

Lg3tf006709g6s
Lg3tf006709g0p
Lh3tf006709g3s
Lh3tf006709g2p
Sg3tf006709g4p
Lh3tf006709g6p
Lg3tf006709g5s
Lh3tf006709g1p
Sg3tf006709g0u
Lg3tf006709g7u
Lh3tf006709g4u
Lg3tf006709g2u
Lh3tf006709g0p
Sg3tf006709g8u
g3tf006709g4u
h3tf006709g9s
h3tf006709g8p
g3tf006709g7u
Lg3tf006709g3u
h3tf006709g5u
Sg3tf006709g6u
g3tf006709g1u
h3tf006709g7p
Sg3tf006709g3s
Sg3tf006709g2s
Sg3tf006709g1p
0.0
0.4
0.8
1.2
Branch Length
Expression Order Of Magnitude
1
2
3
Is Dupliction Node?
N
Y
Leaf
Proportion of Total Expression
0.8
0.6
0.4
0.2
0.0
Silk Gland w/ Majority Expression (Grey=Not 2−Fold Increased in Silk)
AgA
Broad
Not OEST
Ac+F

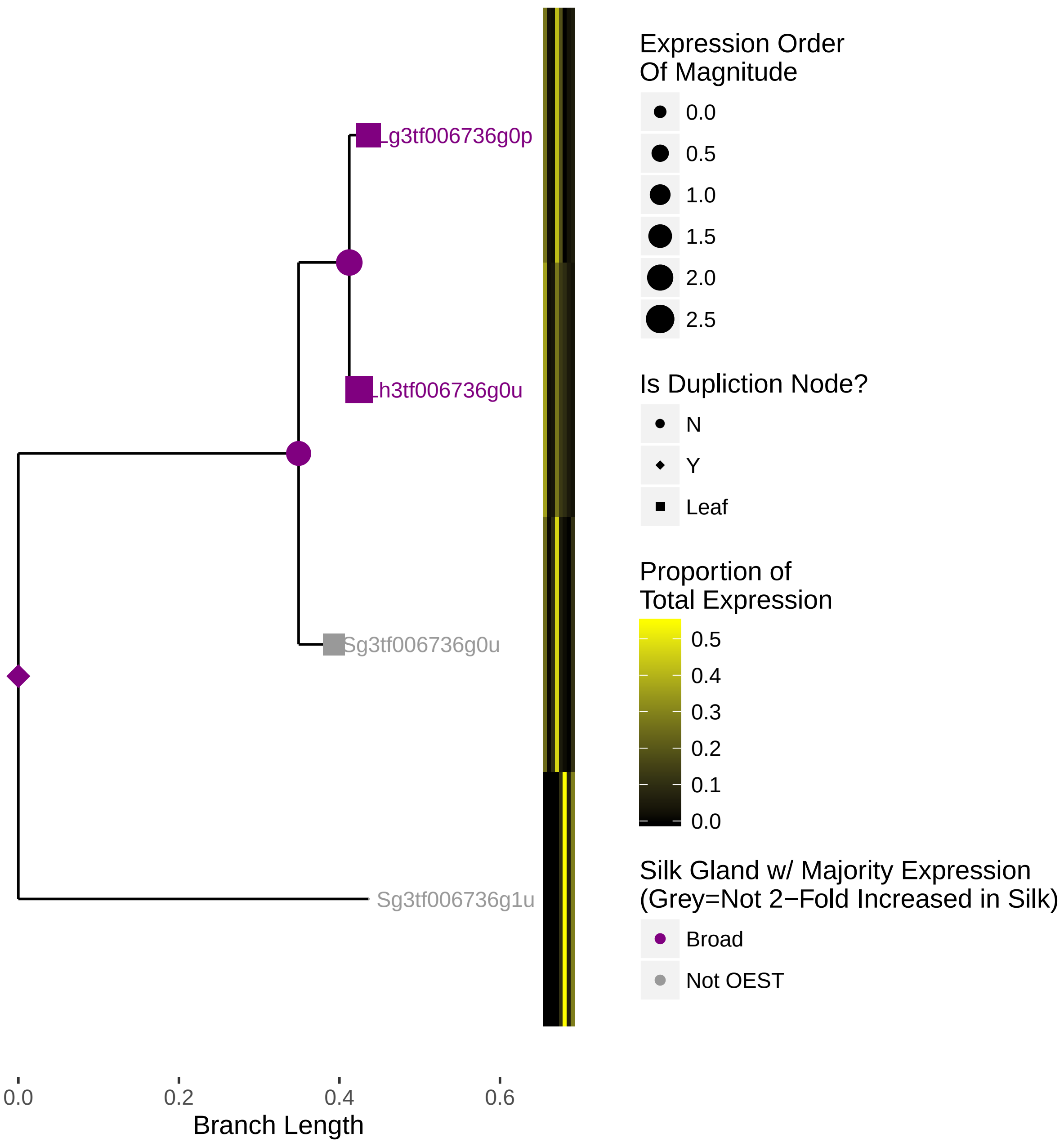
Lg3tf006736g0p
Lh3tf006736g0u
Sg3tf006736g0u
Sg3tf006736g1u
0.0
0.2
0.4
0.6
Branch Length
Expression Order
Of Magnitude
0.0
0.5
1.0
1.5
2.0
2.5
Is Dupliction Node?
N
Y
Leaf
Proportion of
Total Expression
0.5
0.4
0.3
0.2
0.1
0.0
Silk Gland w/ Majority Expression
(Grey=Not 2−Fold Increased in Silk)
Broad
Not OEST

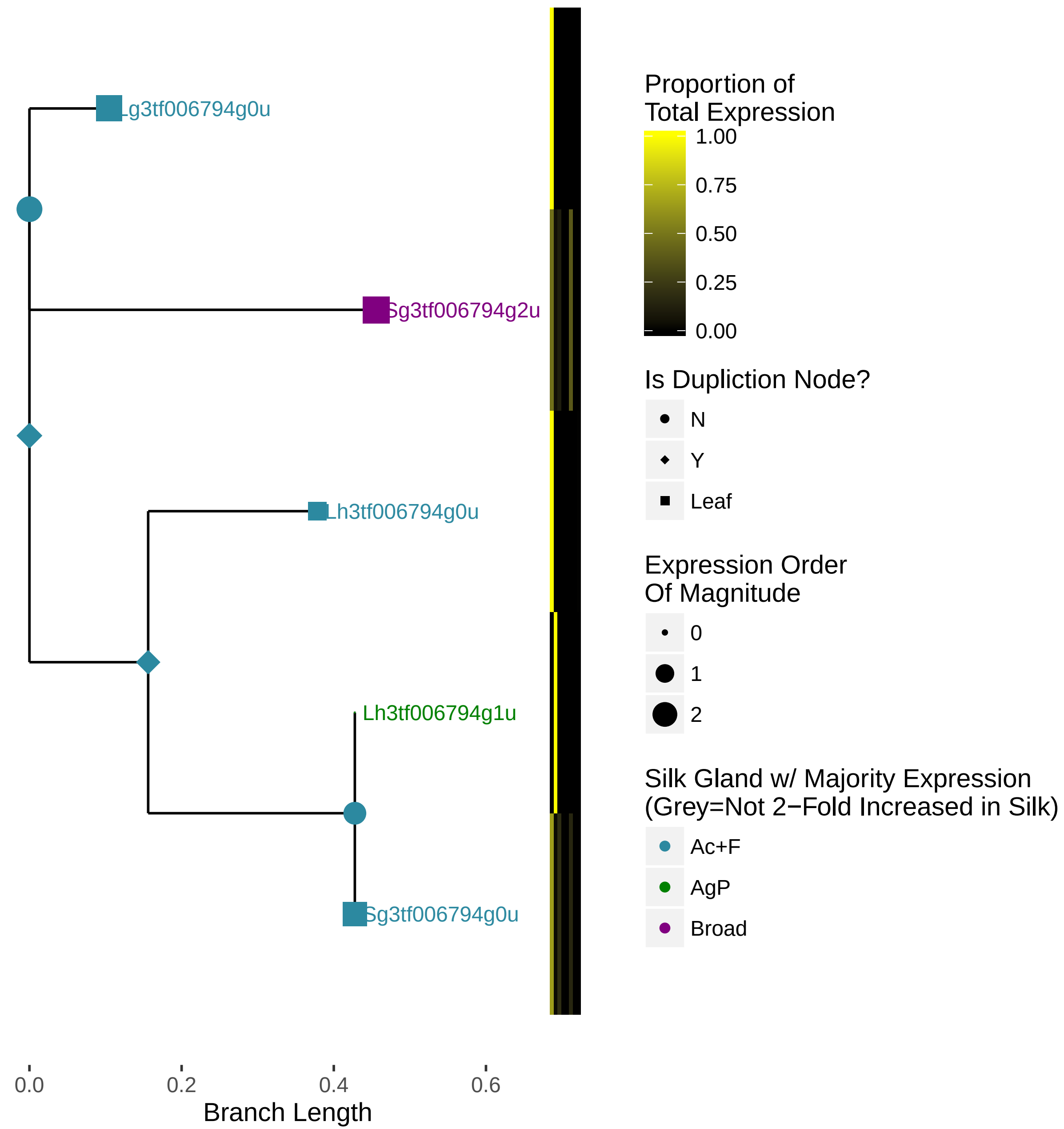

Lg3tf006794g0u
Sg3tf006794g2u
Lh3tf006794g0u
Lh3tf006794g1u
Sg3tf006794g0u
0.0
0.2
0.4
0.6
Branch Length
Proportion of Total Expression
1.00
0.75
0.50
0.25
0.00
Is Dupliction Node?
N
Y
Leaf
Expression Order Of Magnitude
0
1
2
Silk Gland w/ Majority Expression (Grey=Not 2−Fold Increased in Silk)
Ac+F
AgP
Broad

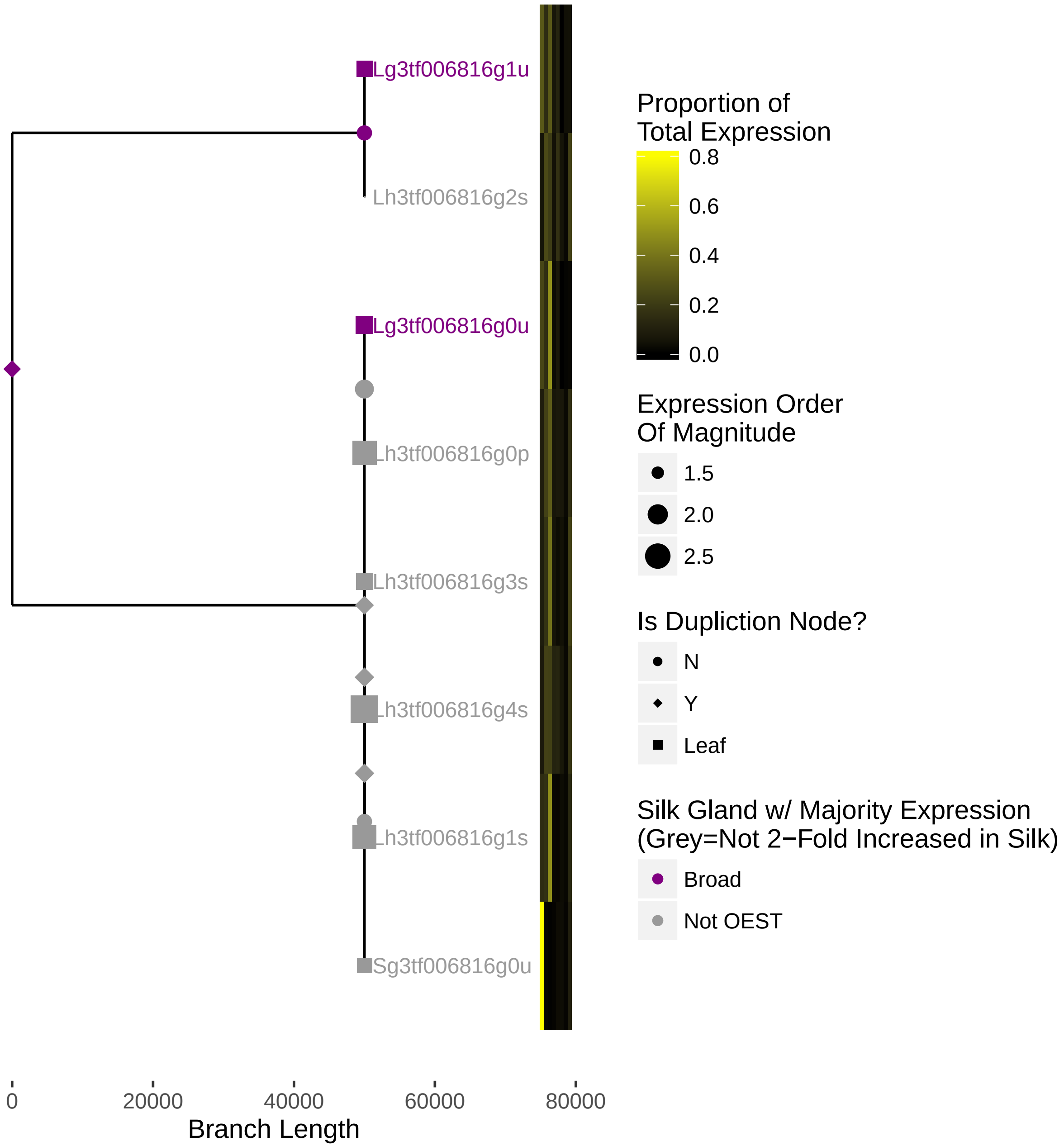
Lg3tf006816g1u
Lh3tf006816g2s
Lg3tf006816g0u
Lh3tf006816g0p
Lh3tf006816g3s
Lh3tf006816g4s
Lh3tf006816g1s
Sg3tf006816g0u
0
20000
40000
60000
80000
Branch Length
Proportion of
Total Expression
0.8
0.6
0.4
0.2
0.0
Expression Order
Of Magnitude
1.5
2.0
2.5
Is Dupliction Node?
N
Y
Leaf
Silk Gland w/ Majority Expression
(Grey=Not 2−Fold Increased in Silk)
Broad
Not OEST

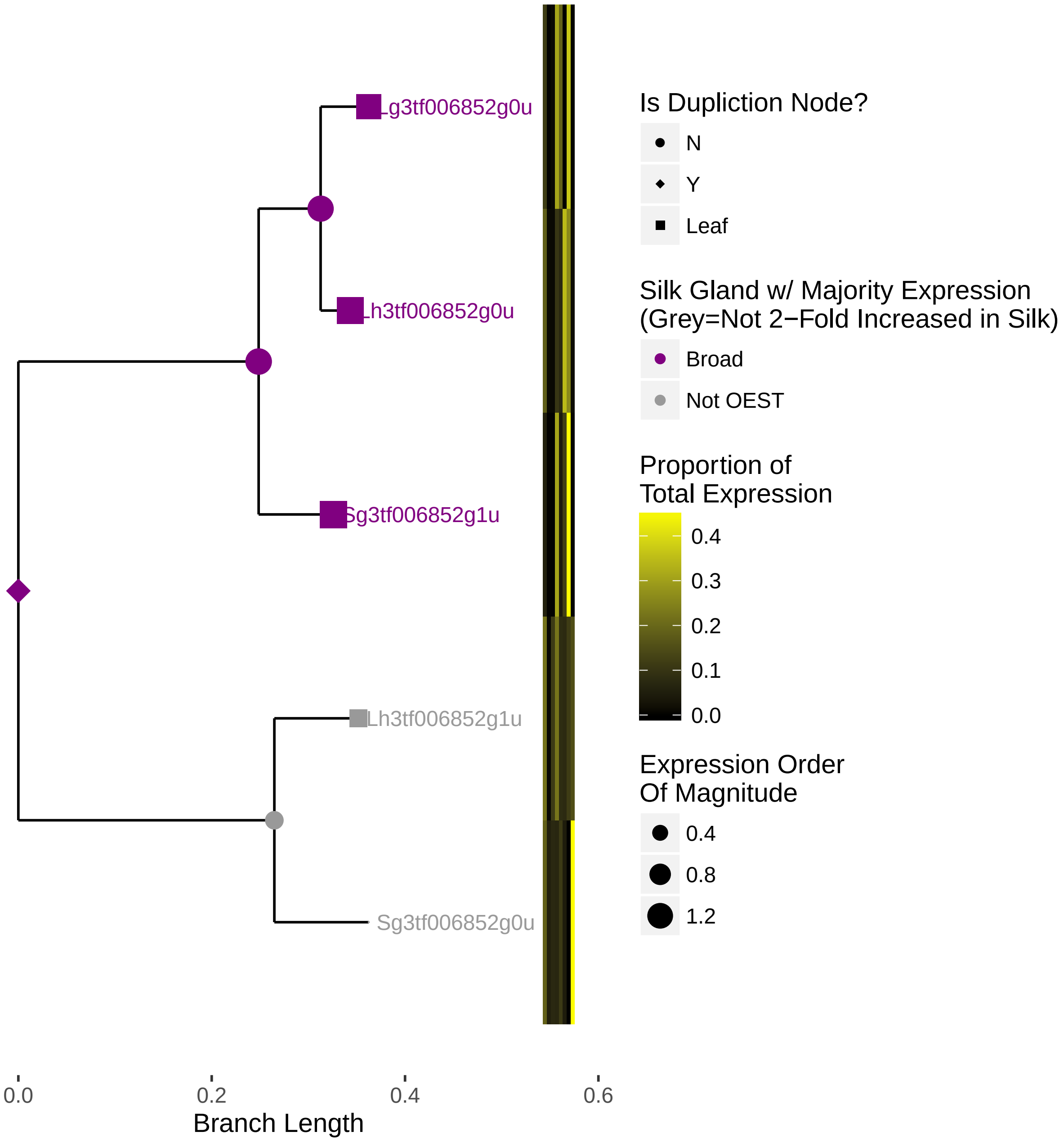
Lg3tf006852g0u
Lh3tf006852g0u
Sg3tf006852g1u
Lh3tf006852g1u
Sg3tf006852g0u
Is Dupliction Node?
N
Y
Leaf
Silk Gland w/ Majority Expression
(Grey=Not 2−Fold Increased in Silk)
Broad
Not OEST
Proportion of
Total Expression
0.4
0.3
0.2
0.1
0.0
Expression Order
Of Magnitude
0.4
0.8
1.2
0.0
0.2
0.4
0.6
Branch Length

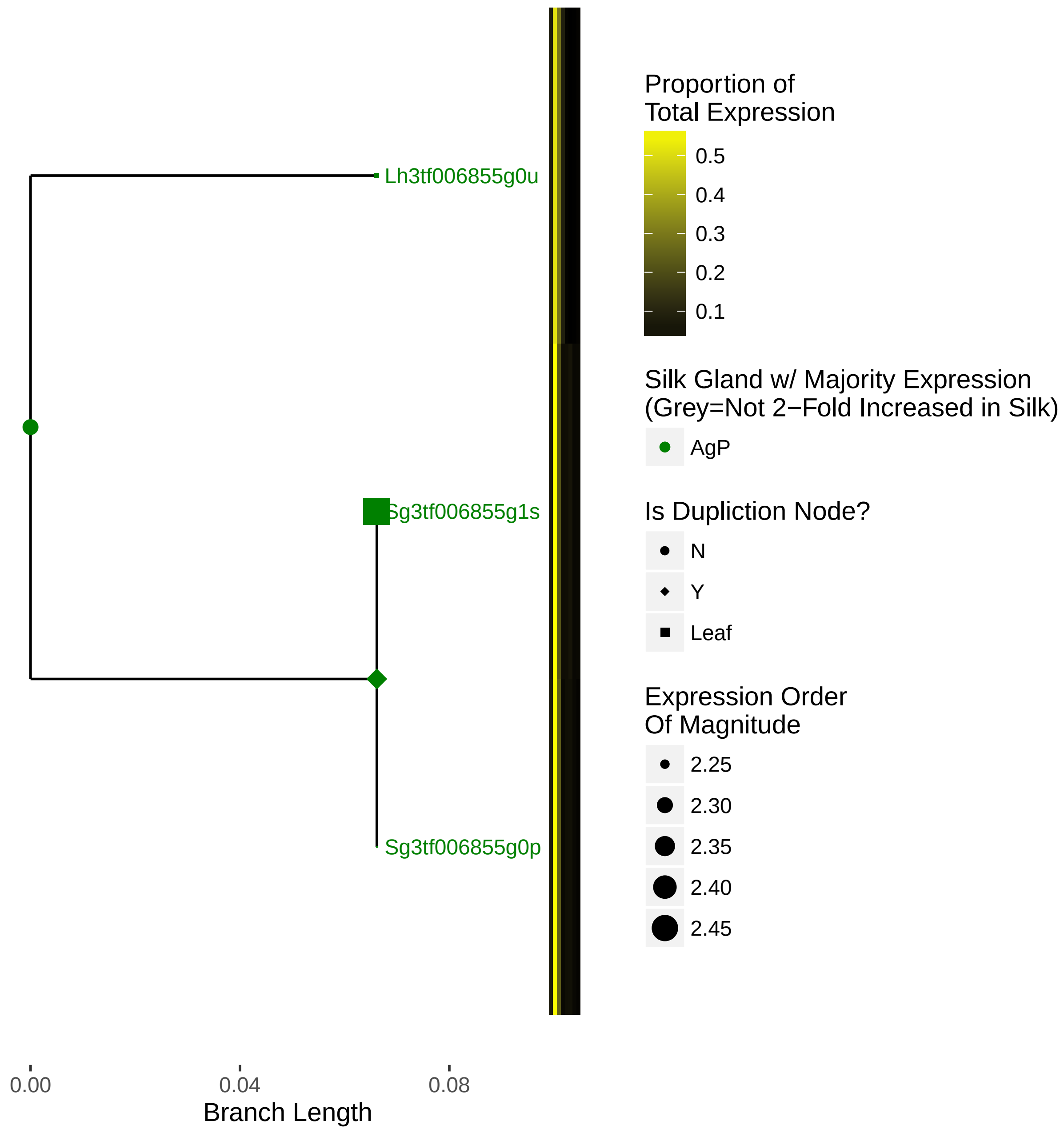
Lh3tf006855g0u
Sg3tf006855g1s
Sg3tf006855g0p
Proportion of Total Expression
0.5
0.4
0.3
0.2
0.1
Silk Gland w/ Majority Expression (Grey=Not 2−Fold Increased in Silk)
AgP
Is Dupliction Node?
N
Y
Leaf
Expression Order Of Magnitude
2.25
2.30
2.35
2.40
2.45
0.00
0.04
0.08
Branch Length

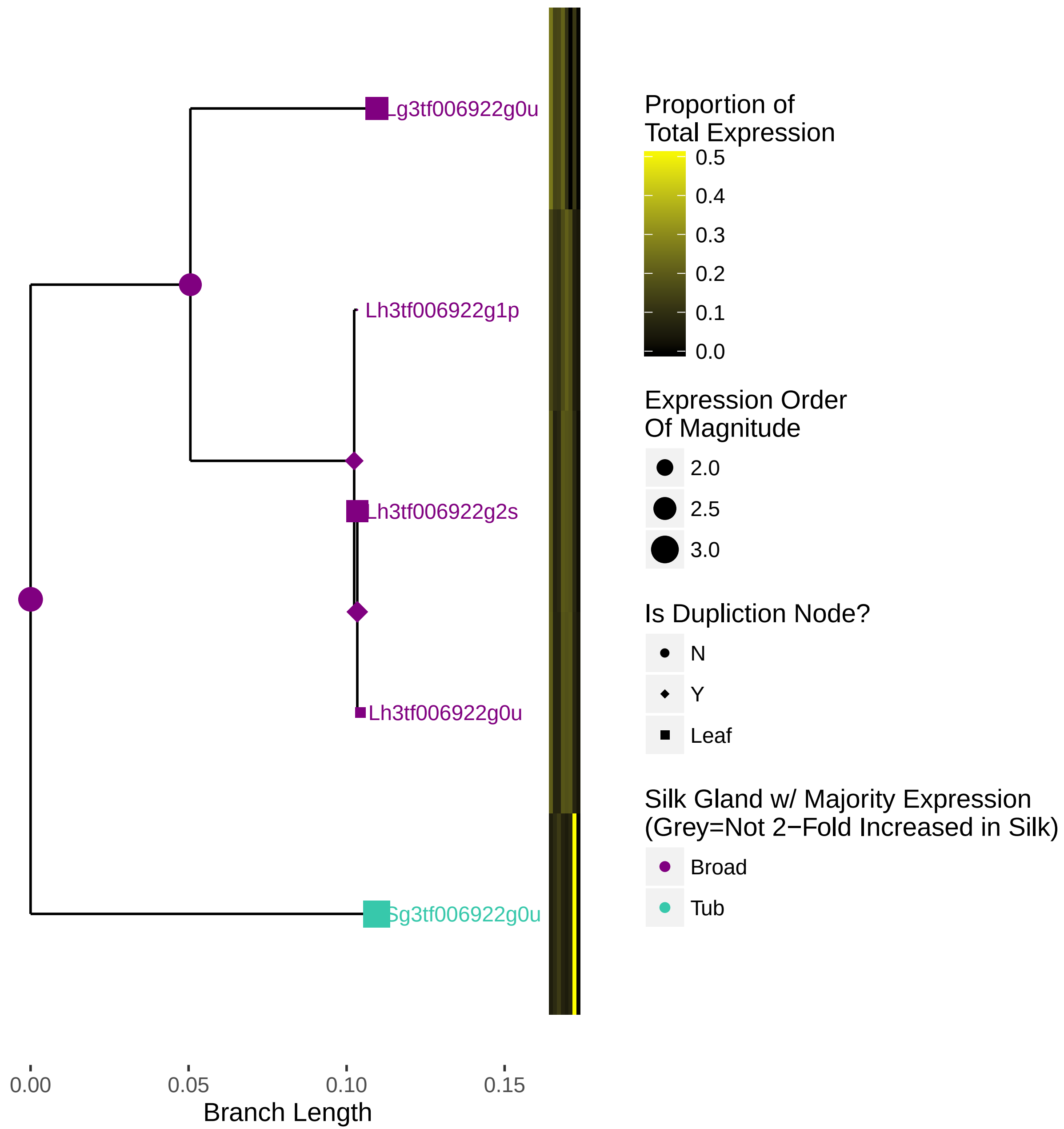
Lg3tf006922g0u
Lh3tf006922g1p
Lh3tf006922g2s
Lh3tf006922g0u
Sg3tf006922g0u
0.00
0.05
0.10
0.15
Branch Length
Proportion of
Total Expression
0.5
0.4
0.3
0.2
0.1
0.0
Expression Order
Of Magnitude
2.0
2.5
3.0
Is Dupliction Node?
N
Y
Leaf
Silk Gland w/ Majority Expression
(Grey=Not 2−Fold Increased in Silk)
Broad
Tub

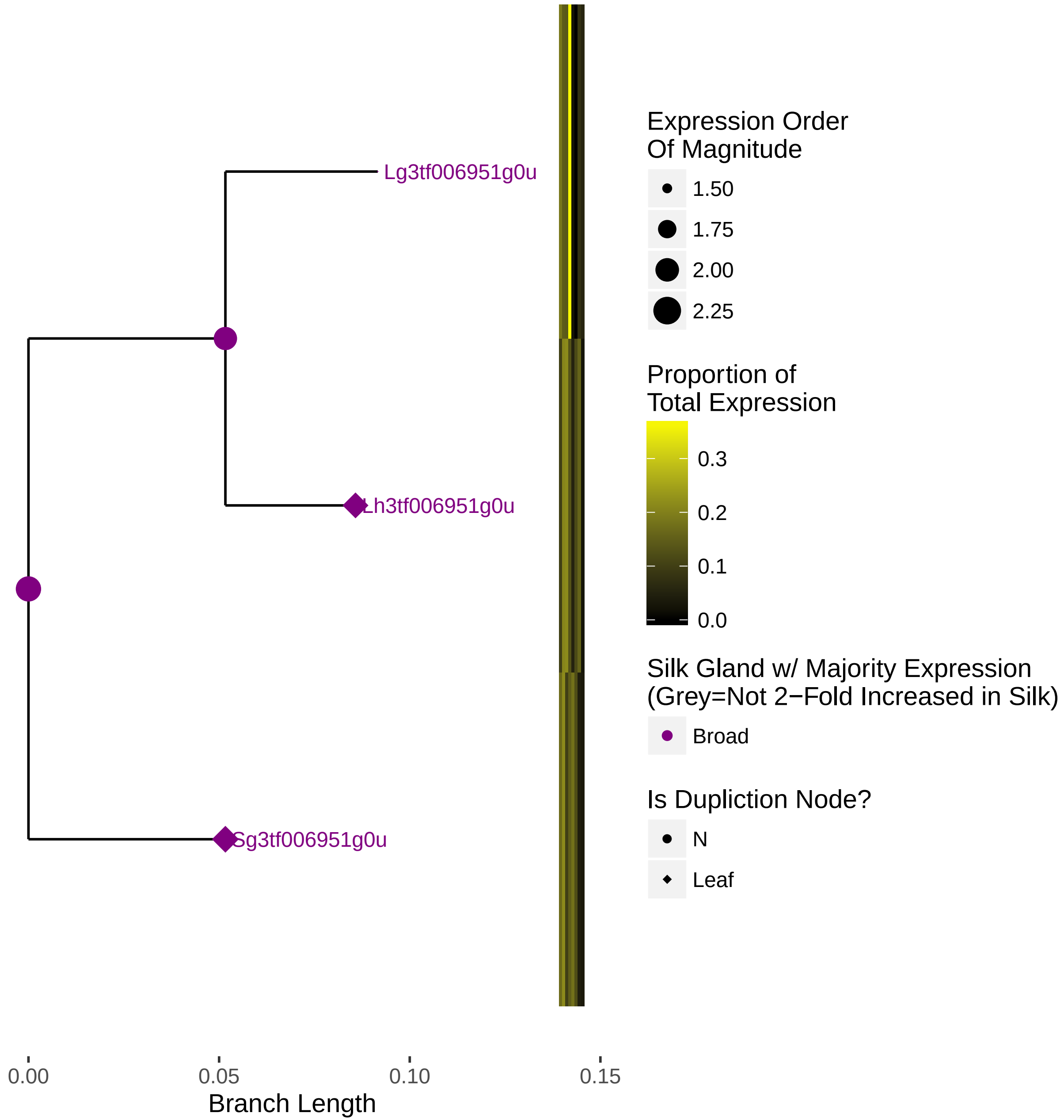

Lg3tf006951g0u
Lh3tf006951g0u
Sg3tf006951g0u
0.00
0.05
0.10
0.15
Branch Length
Expression Order
Of Magnitude
1.50
1.75
2.00
2.25
Proportion of
Total Expression
0.3
0.2
0.1
0.0
Silk Gland w/ Majority Expression
(Grey=Not 2−Fold Increased in Silk)
Broad
Is Dupliction Node?
N
Leaf

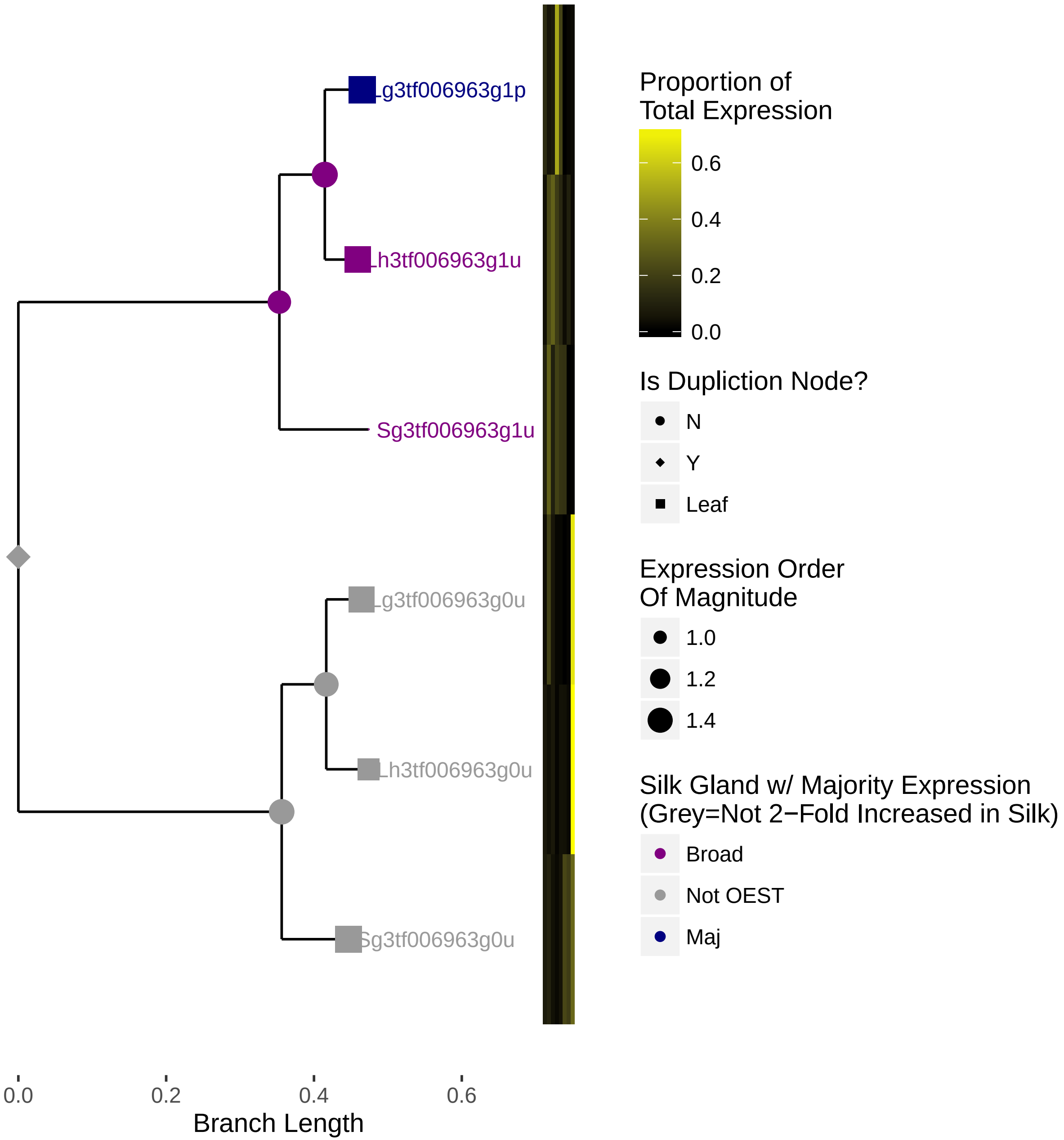

Lg3tf006963g1p
Lh3tf006963g1u
Sg3tf006963g1u
Lg3tf006963g0u
Lh3tf006963g0u
Sg3tf006963g0u
0.0
0.2
0.4
0.6
Branch Length
Proportion of
Total Expression
0.6
0.4
0.2
0.0
Is Dupliction Node?
N
Y
Leaf
Expression Order
Of Magnitude
1.0
1.2
1.4
Silk Gland w/ Majority Expression
(Grey=Not 2−Fold Increased in Silk)
Broad
Not OEST
Maj

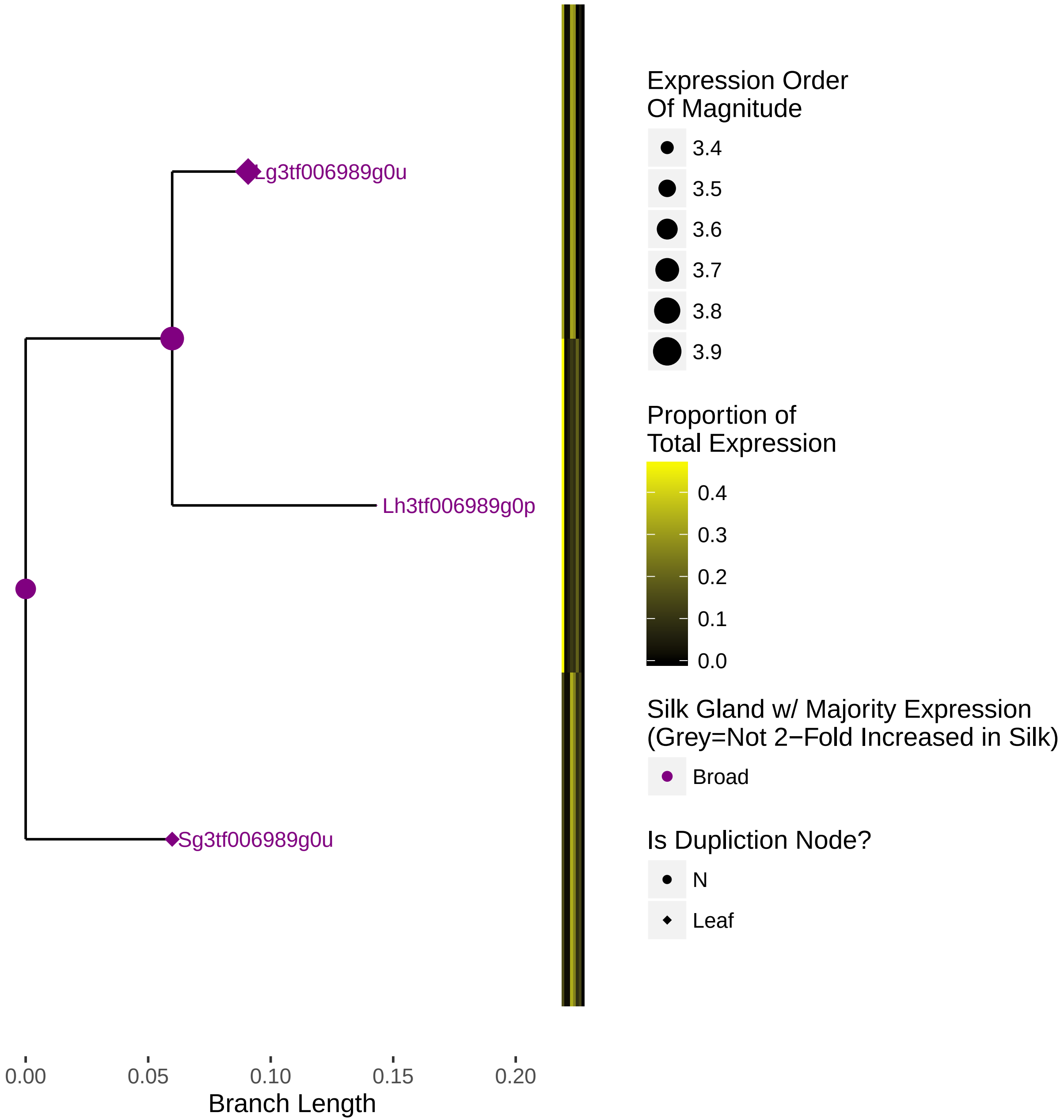

Lg3tf006989g0u
Lh3tf006989g0p
Sg3tf006989g0u
0.00
0.05
0.10
0.15
0.20
Branch Length
Expression Order
Of Magnitude
3.4
3.5
3.6
3.7
3.8
3.9
Proportion of
Total Expression
0.4
0.3
0.2
0.1
0.0
Silk Gland w/ Majority Expression
(Grey=Not 2−Fold Increased in Silk)
Broad
Is Duplication Node?
N
Leaf

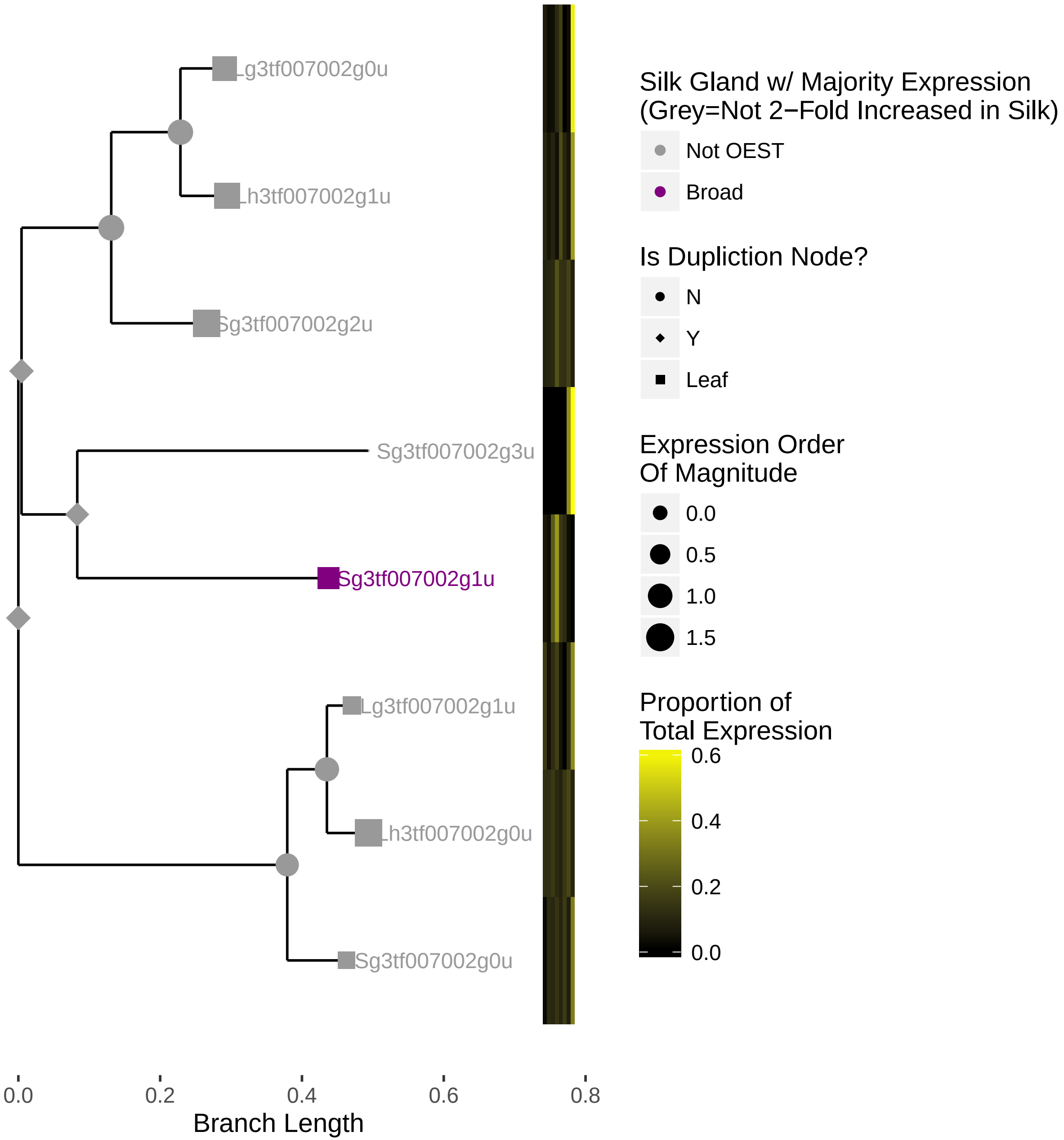

Lg3tf007002g0u
Lh3tf007002g1u
Sg3tf007002g2u
Sg3tf007002g3u
Sg3tf007002g1u
Lg3tf007002g1u
Lh3tf007002g0u
Sg3tf007002g0u
0.0
0.2
0.4
0.6
0.8
Branch Length
Silk Gland w/ Majority Expression (Grey=Not 2−Fold Increased in Silk)
Not OEST
Broad
Is Dupliction Node?
N
Y
Leaf
Expression Order Of Magnitude
0.0
0.5
1.0
1.5
Proportion of Total Expression
0.6
0.4
0.2
0.0

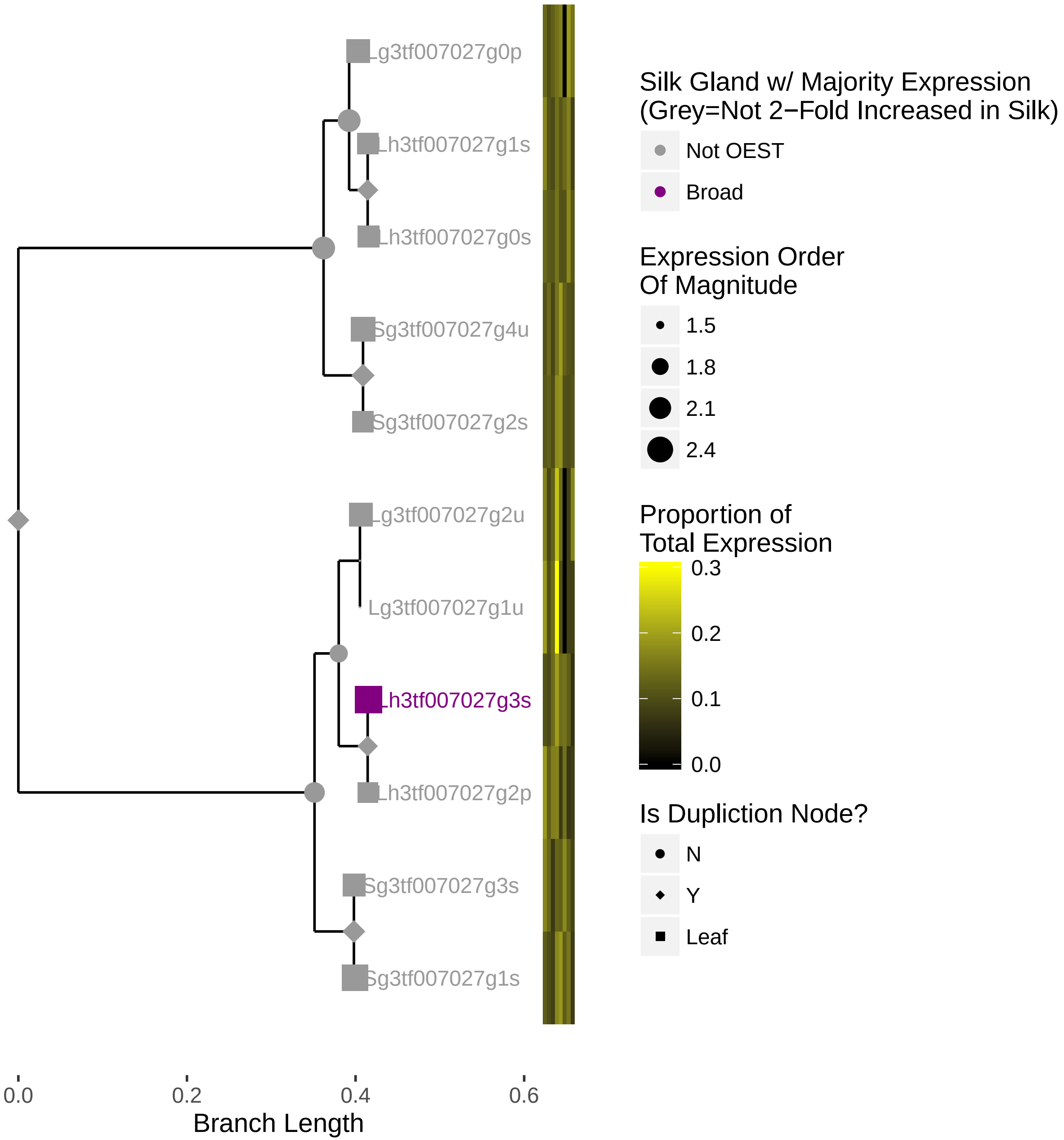

Lg3tf007027g0p
Lh3tf007027g1s
Lh3tf007027g0s
Sg3tf007027g4u
Sg3tf007027g2s
Lg3tf007027g2u
Lg3tf007027g1u
Lh3tf007027g3s
Lh3tf007027g2p
Sg3tf007027g3s
Sg3tf007027g1s
0.0
0.2
0.4
0.6
Branch Length
Silk Gland w/ Majority Expression (Grey=Not 2−Fold Increased in Silk)
Not OEST
Broad
Expression Order Of Magnitude
1.5
1.8
2.1
2.4
Proportion of Total Expression
0.3
0.2
0.1
0.0
Is Dupliction Node?
N
Y
Leaf

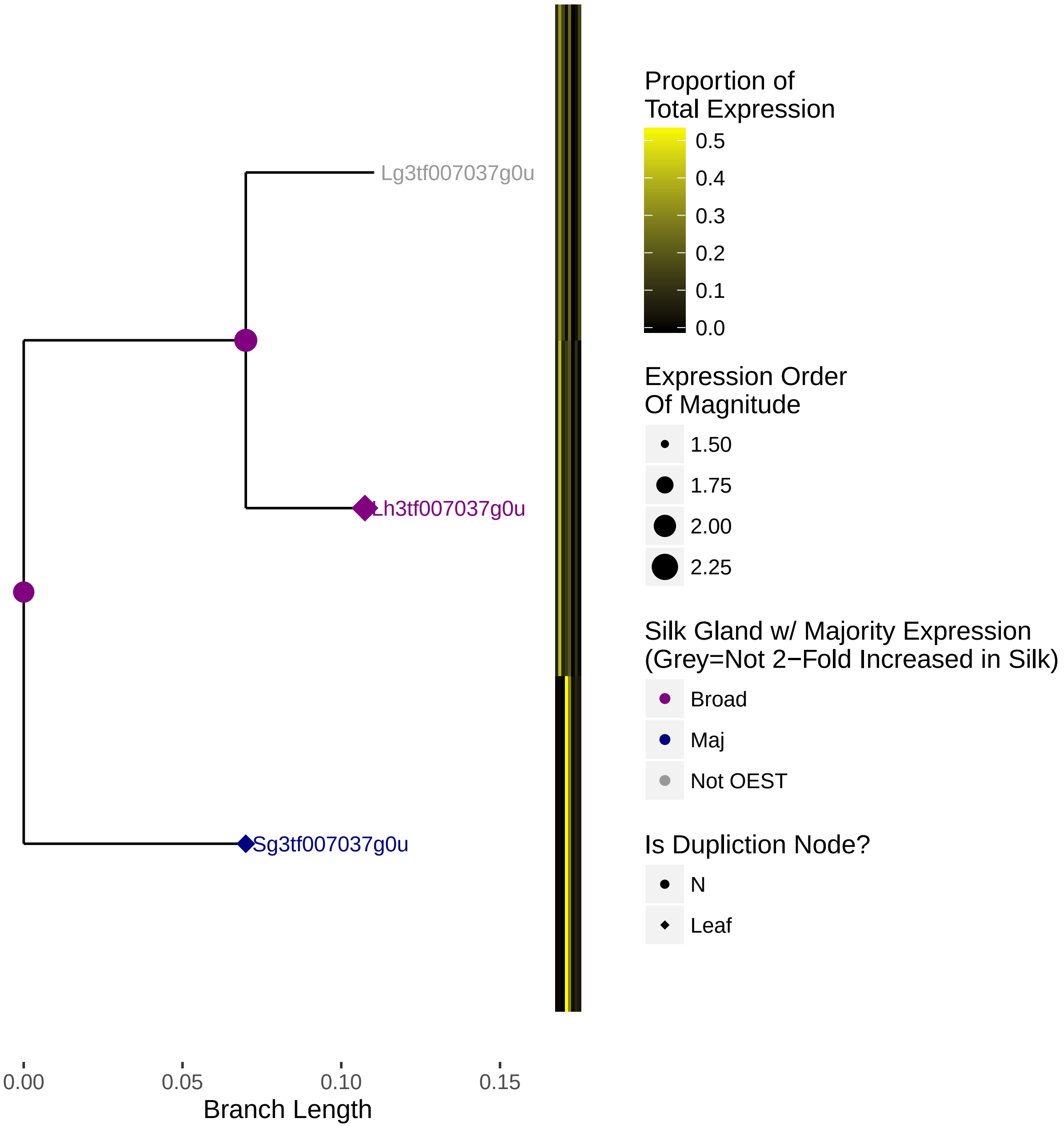

Lg3tf007037g0u
Lh3tf007037g0u
Sg3tf007037g0u
0.00
0.05
0.10
0.15
Branch Length
Proportion of
Total Expression
0.5
0.4
0.3
0.2
0.1
0.0
Expression Order
Of Magnitude
1.50
1.75
2.00
2.25
Silk Gland w/ Majority Expression
(Grey=Not 2−Fold Increased in Silk)
Broad
Maj
Not OEST
Is Dupliction Node?
N
Leaf

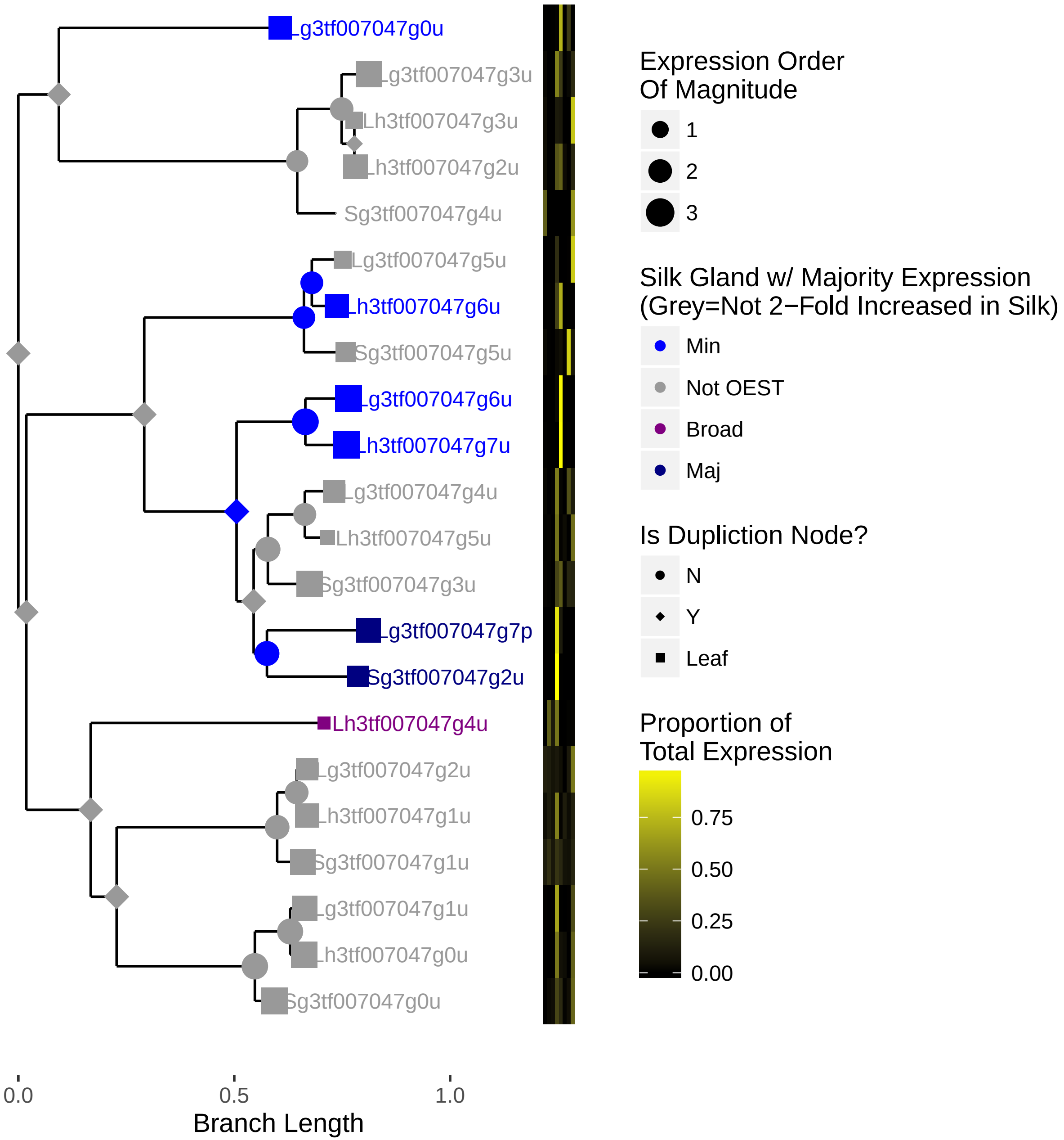

Lg3tf007047g0u
Lg3tf007047g3u
Lh3tf007047g3u
Lh3tf007047g2u
Sg3tf007047g4u
Lg3tf007047g5u
Lh3tf007047g6u
Sg3tf007047g5u
Lg3tf007047g6u
Lh3tf007047g7u
Lg3tf007047g4u
Lh3tf007047g5u
Sg3tf007047g3u
Lg3tf007047g7p
Sg3tf007047g2u
Lh3tf007047g4u
Lg3tf007047g2u
Lh3tf007047g1u
Sg3tf007047g1u
Lg3tf007047g1u
Lh3tf007047g0u
Sg3tf007047g0u
0.0
0.5
1.0
Branch Length
Expression Order
Of Magnitude
1
2
3
Silk Gland w/ Majority Expression
(Grey=Not 2−Fold Increased in Silk)
Min
Not OEST
Broad
Maj
Is Dupliction Node?
N
Y
Leaf
Proportion of
Total Expression
0.75
0.50
0.25
0.00

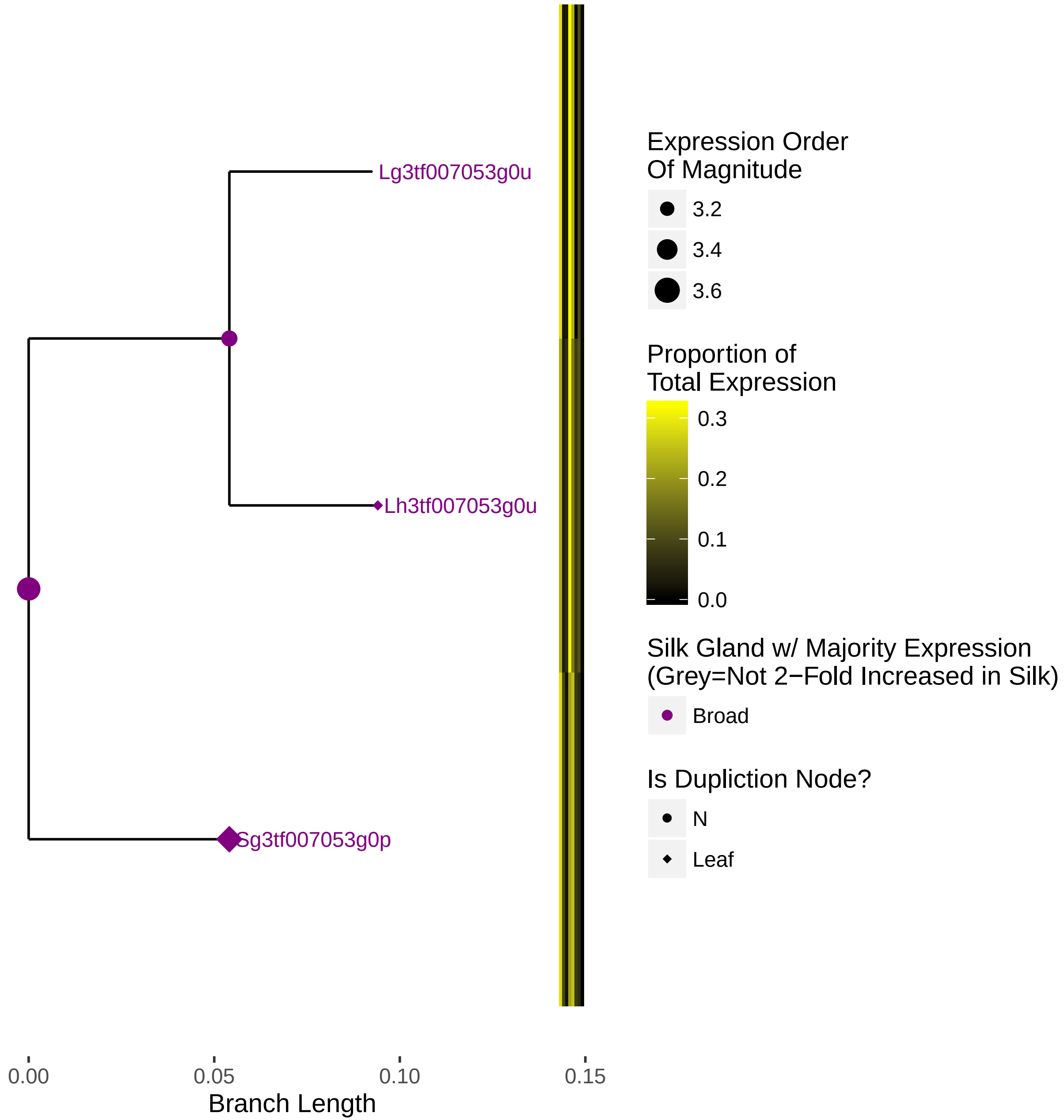

Lg3tf007053g0u
Lh3tf007053g0u
Sg3tf007053g0p
0.00
0.05
0.10
0.15
Branch Length
Expression Order
Of Magnitude
3.2
3.4
3.6
Proportion of
Total Expression
0.3
0.2
0.1
0.0
Silk Gland w/ Majority Expression
(Grey=Not 2−Fold Increased in Silk)
Broad
Is Dupliction Node?
N
Leaf

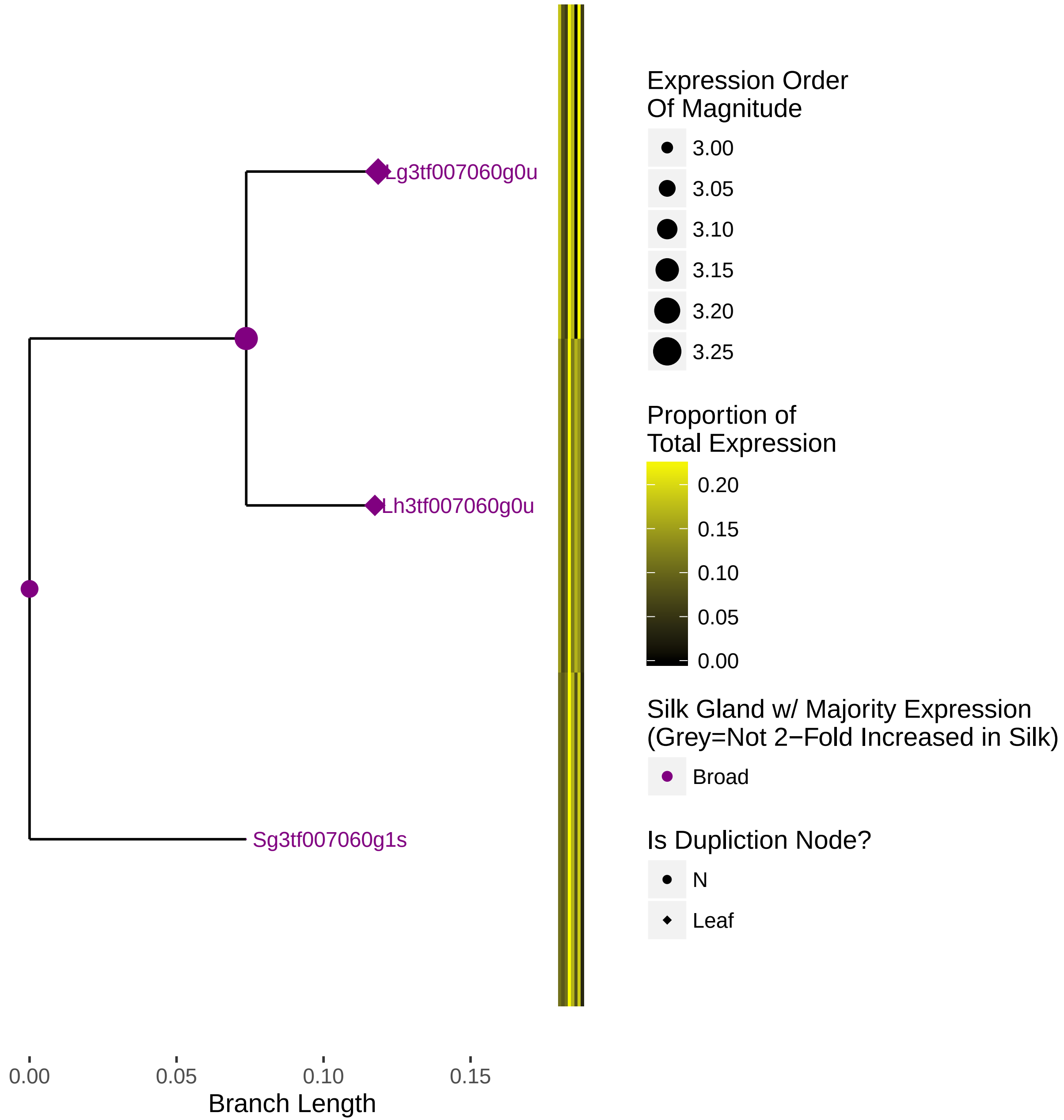

Lg3tf007060g0u
Lh3tf007060g0u
Sg3tf007060g1s
0.00
0.05
0.10
0.15
Branch Length
Expression Order
Of Magnitude
3.00
3.05
3.10
3.15
3.20
3.25
Proportion of
Total Expression
0.20
0.15
0.10
0.05
0.00
Silk Gland w/ Majority Expression
(Grey=Not 2−Fold Increased in Silk)
Broad
Is Dupliction Node?
N
Leaf

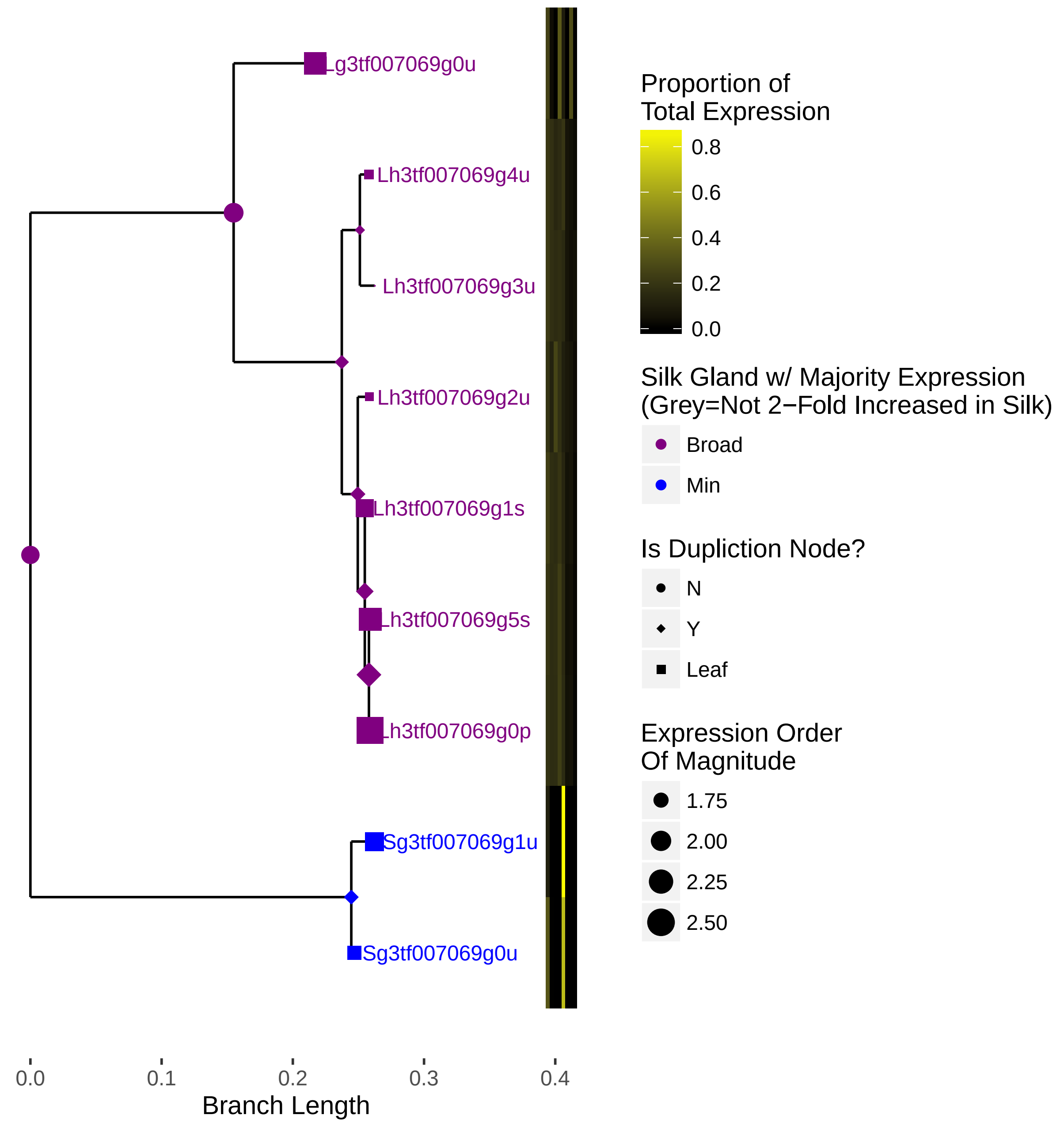

Lg3tf007069g0u
Lh3tf007069g4u
Lh3tf007069g3u
Lh3tf007069g2u
Lh3tf007069g1s
Lh3tf007069g5s
Lh3tf007069g0p
Sg3tf007069g1u
Sg3tf007069g0u
0.0
0.1
0.2
0.3
0.4
Branch Length
Proportion of Total Expression
0.8
0.6
0.4
0.2
0.0
Silk Gland w/ Majority Expression (Grey=Not 2−Fold Increased in Silk)
Broad
Min
Is Dupliction Node?
N
Y
Leaf
Expression Order Of Magnitude
1.75
2.00
2.25
2.50

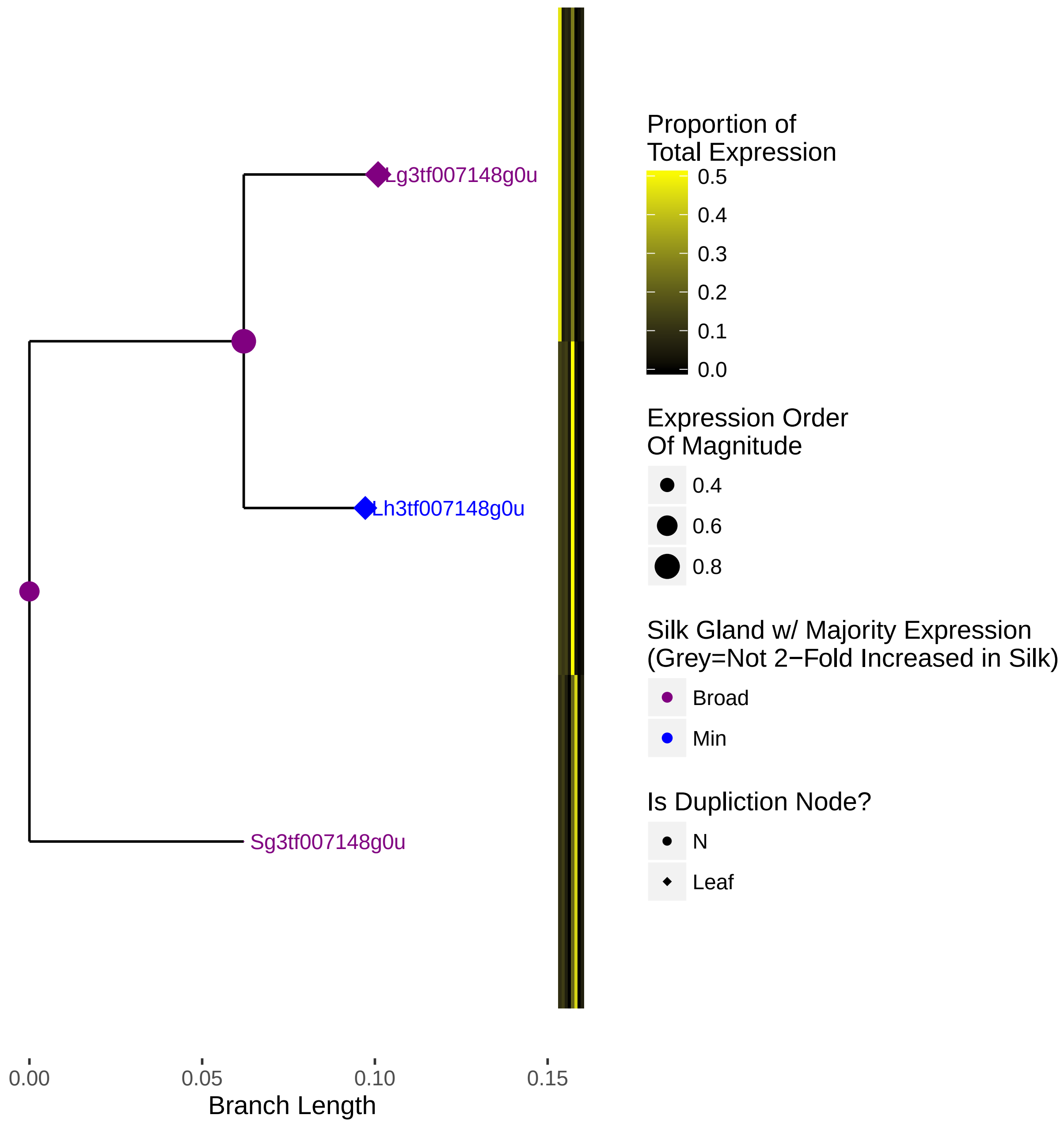

Lg3tf007148g0u
Lh3tf007148g0u
Sg3tf007148g0u
0.00
0.05
0.10
0.15
Branch Length
Proportion of
Total Expression
0.5
0.4
0.3
0.2
0.1
0.0
Expression Order
Of Magnitude
0.4
0.6
0.8
Silk Gland w/ Majority Expression
(Grey=Not 2−Fold Increased in Silk)
Broad
Min
Is Dupliction Node?
N
Leaf

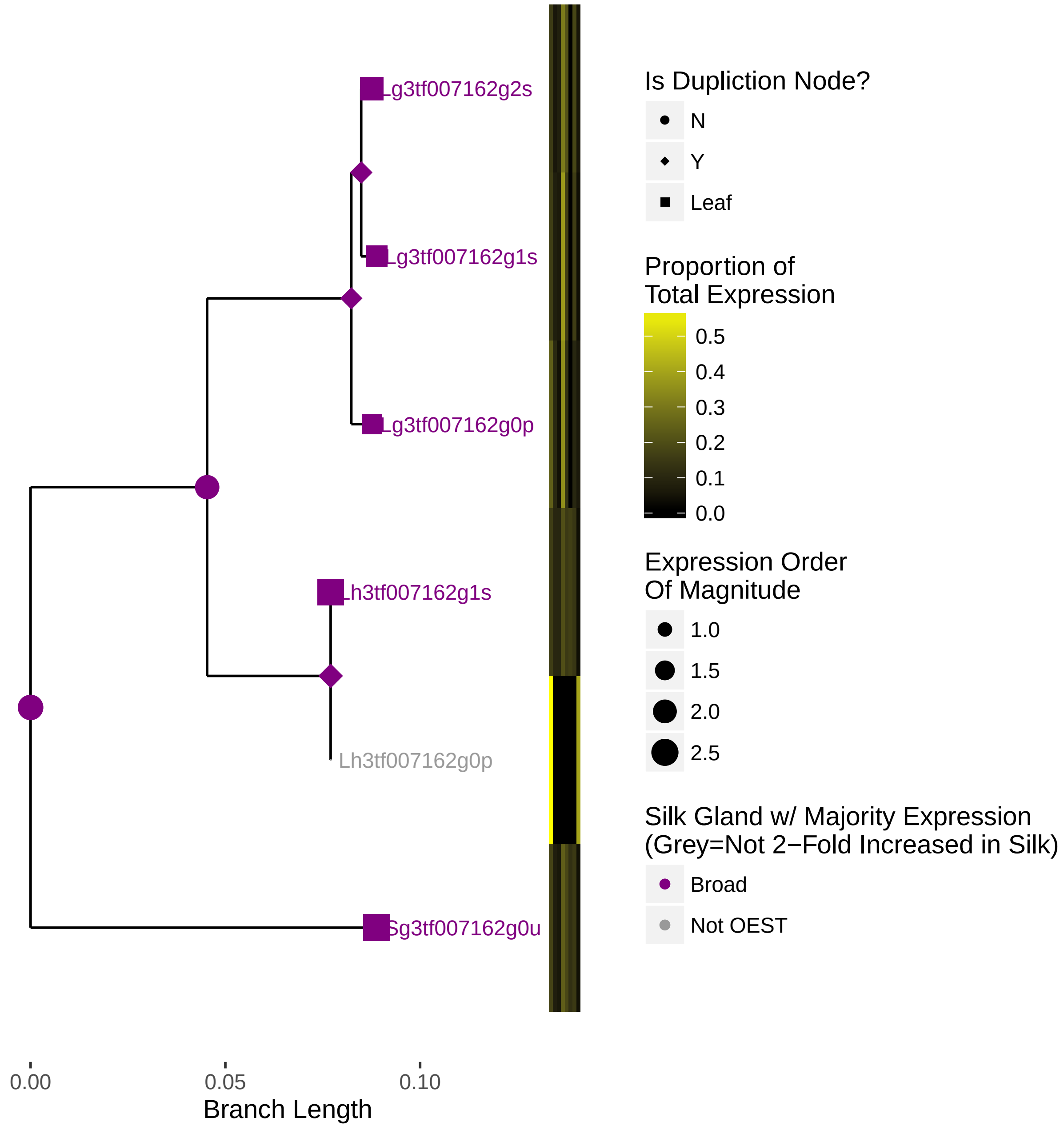

Lg3tf007162g2s
Lg3tf007162g1s
Lg3tf007162g0p
Lh3tf007162g1s
Lh3tf007162g0p
Sg3tf007162g0u
0.00
0.05
0.10
Branch Length
Is Dupliction Node?
N
Y
Leaf
Proportion of
Total Expression
0.5
0.4
0.3
0.2
0.1
0.0
Expression Order
Of Magnitude
1.0
1.5
2.0
2.5
Silk Gland w/ Majority Expression
(Grey=Not 2−Fold Increased in Silk)
Broad
Not OEST

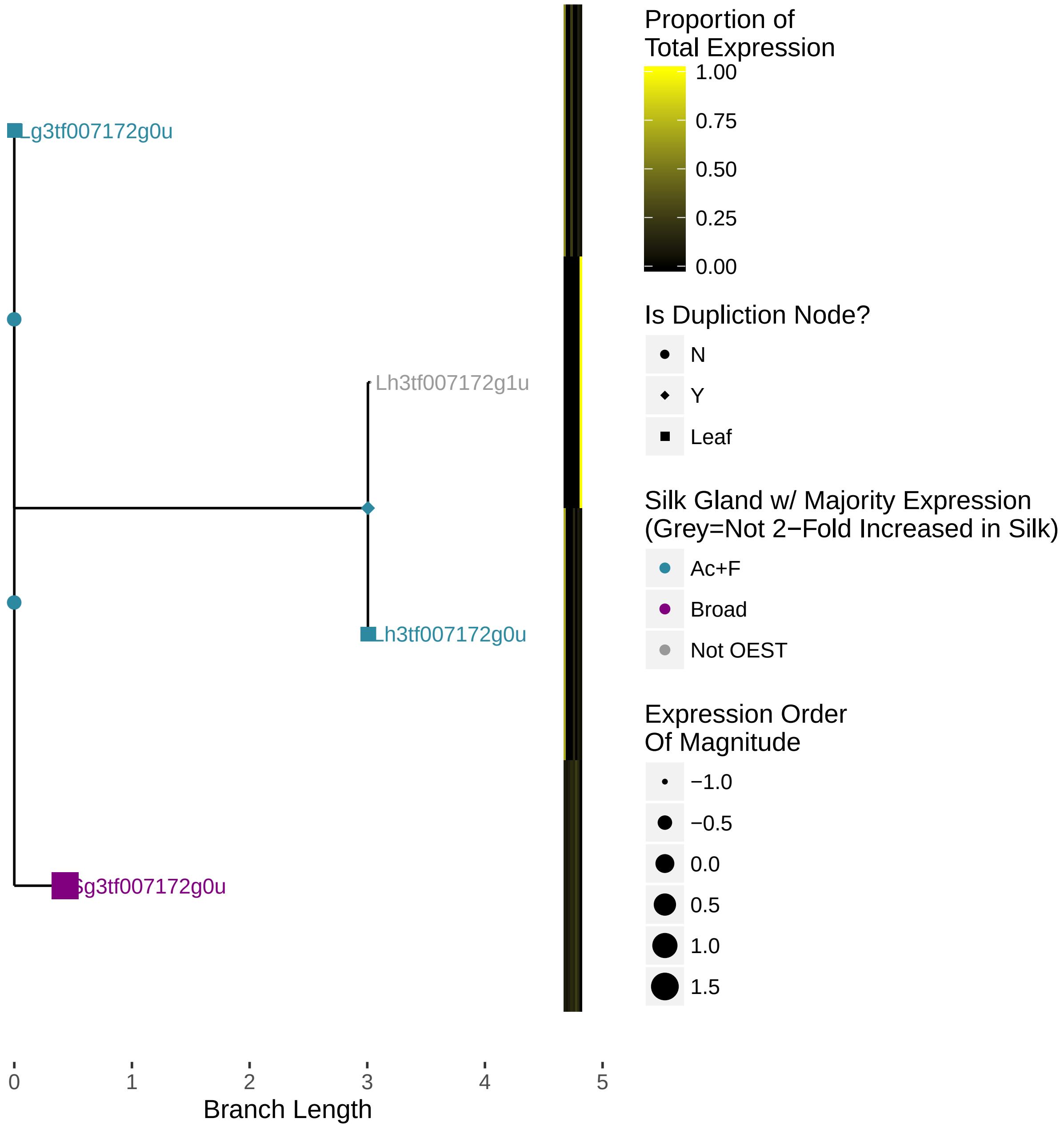

Lg3tf007172g0u
Lh3tf007172g1u
Lh3tf007172g0u
Sg3tf007172g0u
0
1
2
3
4
5
Branch Length
Proportion of
Total Expression
1.00
0.75
0.50
0.25
0.00
Is Dupliction Node?
N
Y
Leaf
Silk Gland w/ Majority Expression
(Grey=Not 2−Fold Increased in Silk)
Ac+F
Broad
Not OEST
Expression Order
Of Magnitude
−1.0
−0.5
0.0
0.5
1.0
1.5

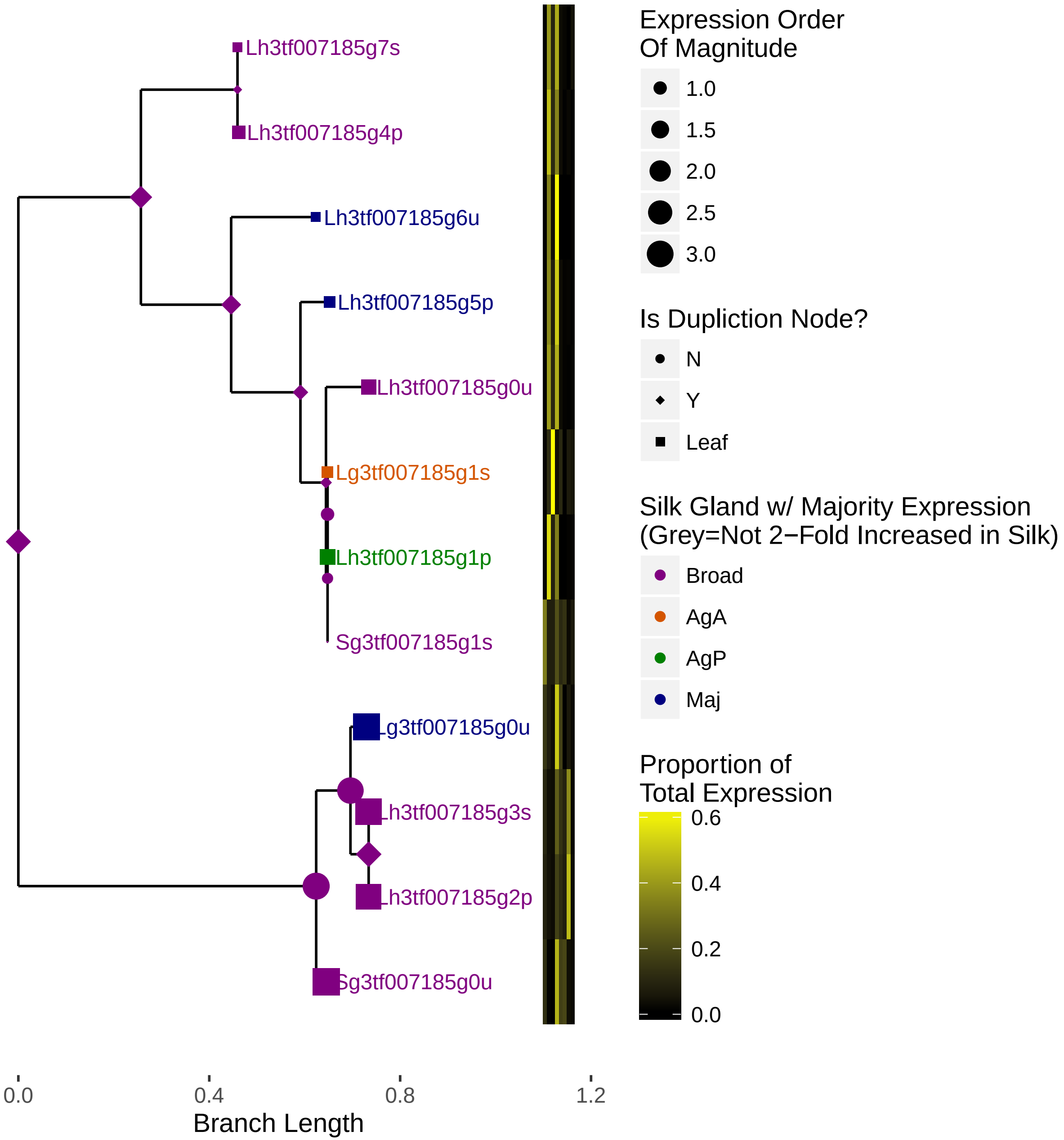

Lh3tf007185g7s
Lh3tf007185g4p
Lh3tf007185g6u
Lh3tf007185g5p
Lh3tf007185g0u
Lg3tf007185g1s
Lh3tf007185g1p
Sg3tf007185g1s
g3tf007185g0u
h3tf007185g3s
h3tf007185g2p
g3tf007185g0u
0.0
0.4
0.8
1.2
Branch Length
Expression Order
Of Magnitude
1.0
1.5
2.0
2.5
3.0
Is Dupliction Node?
N
Y
Leaf
Silk Gland w/ Majority Expression
(Grey=Not 2−Fold Increased in Silk)
Broad
AgA
AgP
Maj
Proportion of
Total Expression
0.6
0.4
0.2
0.0

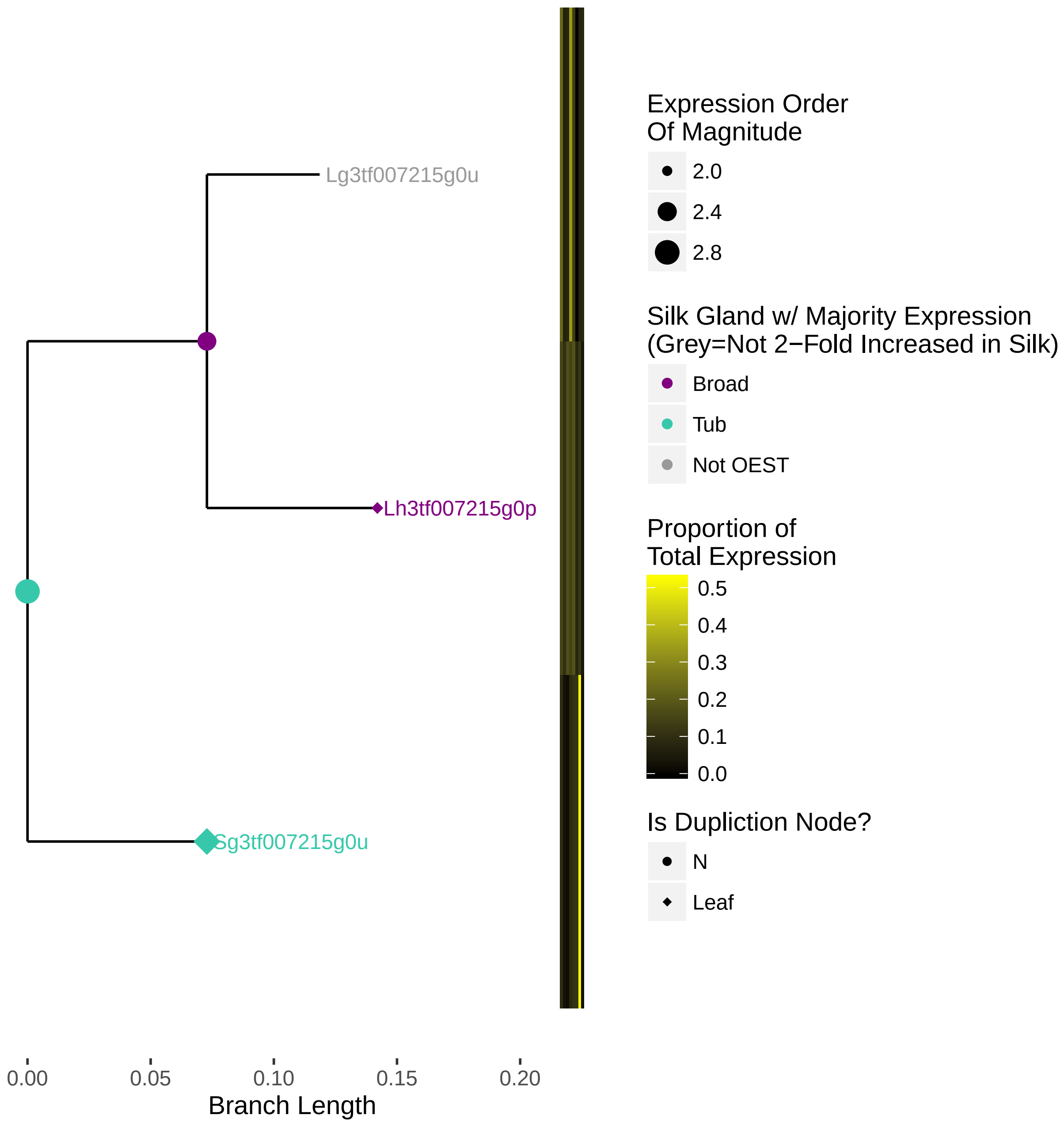

Lg3tf007215g0u
Lh3tf007215g0p
Sg3tf007215g0u
0.00
0.05
0.10
0.15
0.20
Branch Length
Expression Order
Of Magnitude
2.0
2.4
2.8
Silk Gland w/ Majority Expression
(Grey=Not 2−Fold Increased in Silk)
Broad
Tub
Not OEST
Proportion of
Total Expression
0.5
0.4
0.3
0.2
0.1
0.0
Is Dupliction Node?
N
Leaf

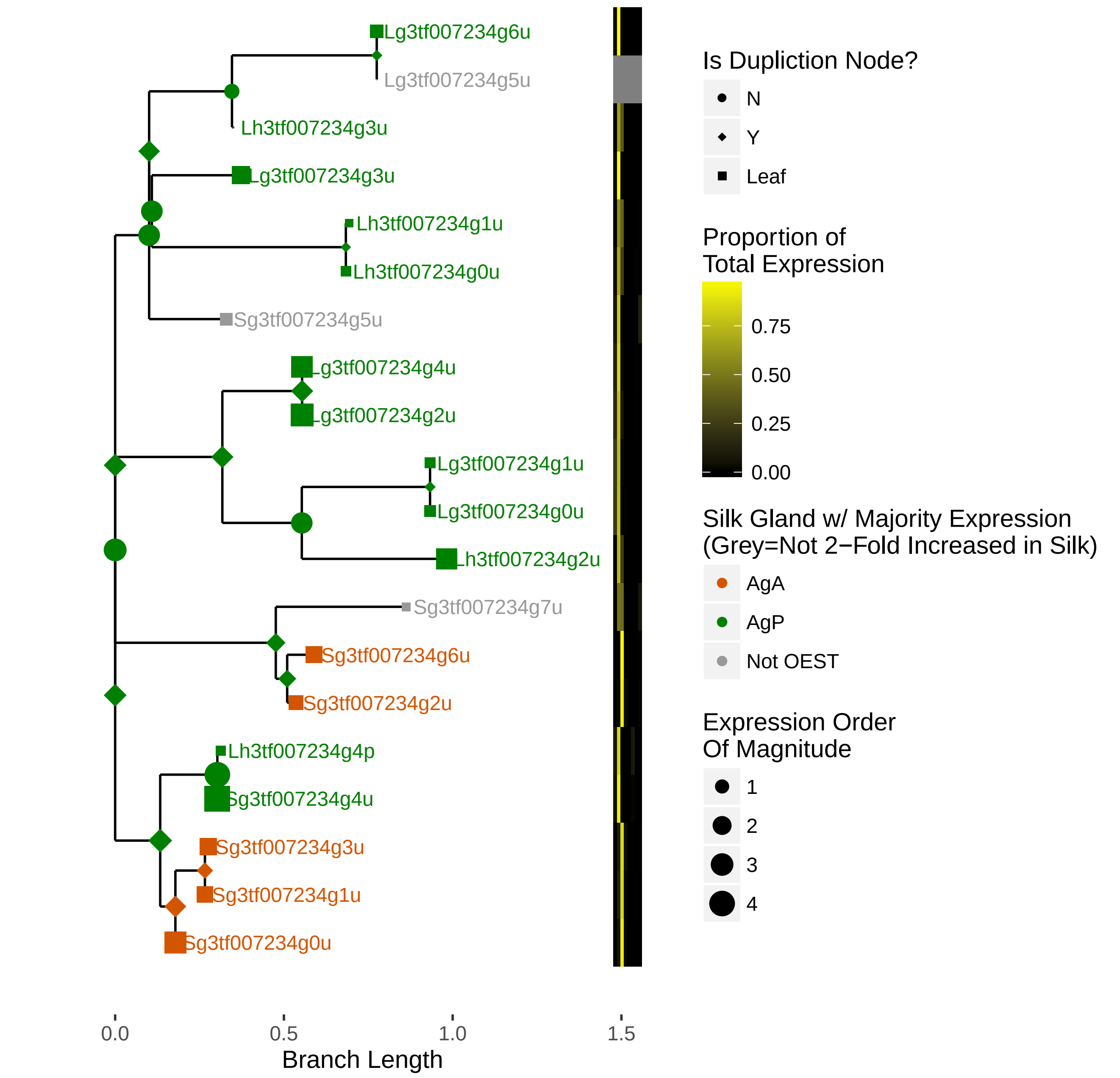
Lg3tf007234g6u
Lg3tf007234g5u
Lh3tf007234g3u
Lg3tf007234g3u
Lh3tf007234g1u
Lh3tf007234g0u
Sg3tf007234g5u
Lg3tf007234g4u
Lg3tf007234g2u
Lg3tf007234g1u
Lg3tf007234g0u
Lh3tf007234g2u
Sg3tf007234g7u
Sg3tf007234g6u
Sg3tf007234g2u
Lh3tf007234g4p
Sg3tf007234g4u
Sg3tf007234g3u
Sg3tf007234g1u
Sg3tf007234g0u
0.0
0.5
1.0
1.5
Branch Length
Is Dupliction Node?
N
Y
Leaf
Proportion of
Total Expression
0.75
0.50
0.25
0.00
Silk Gland w/ Majority Expression
(Grey=Not 2−Fold Increased in Silk)
AgA
AgP
Not OEST
Expression Order
Of Magnitude
1
2
3
4

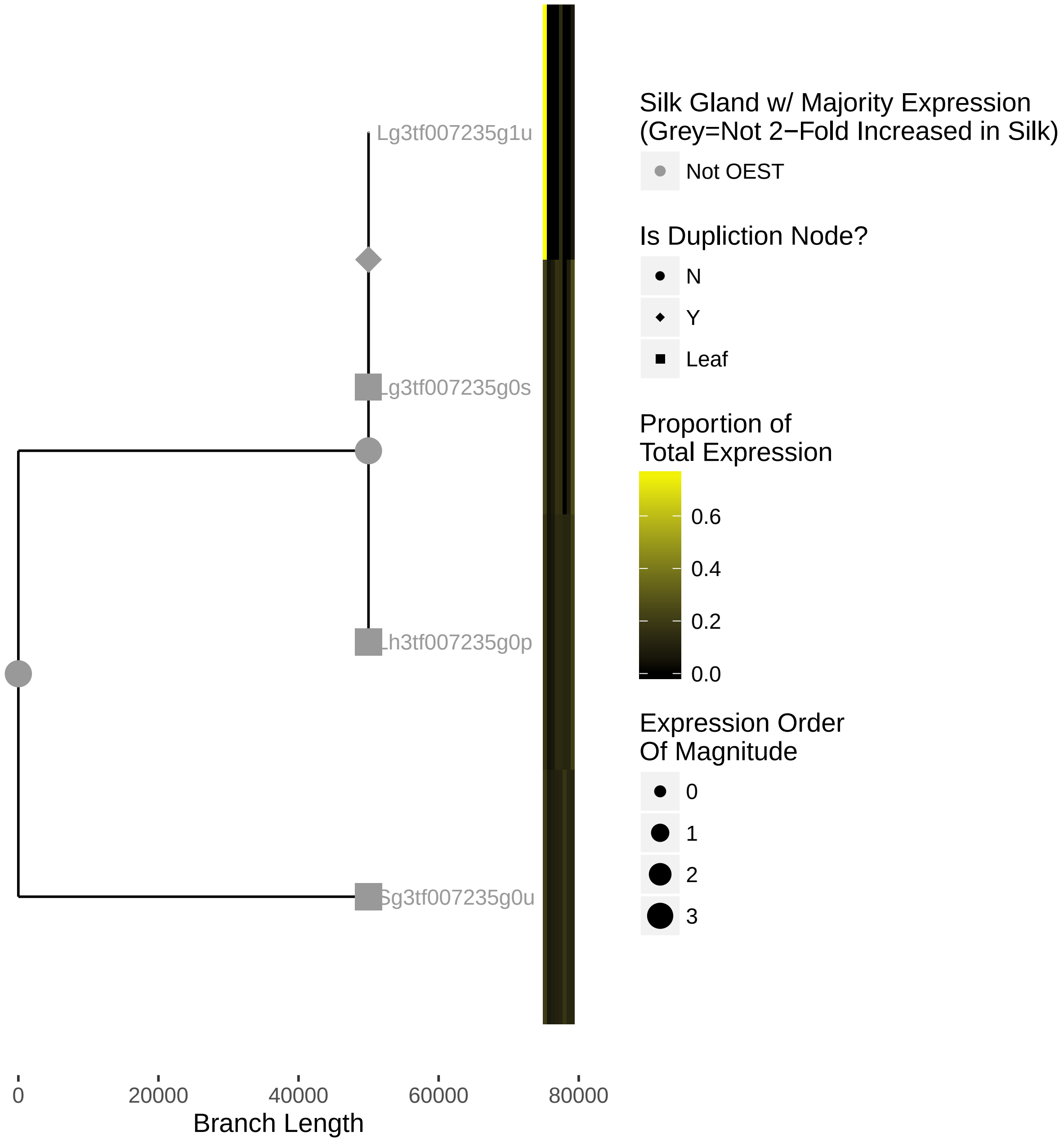

Lg3tf007235g1u
Lg3tf007235g0s
Lh3tf007235g0p
Sg3tf007235g0u
0
20000
40000
60000
80000
Branch Length
Silk Gland w/ Majority Expression
(Grey=Not 2−Fold Increased in Silk)
Not OEST
Is Dupliction Node?
N
Y
Leaf
Proportion of
Total Expression
0.6
0.4
0.2
0.0
Expression Order
Of Magnitude
0
1
2
3

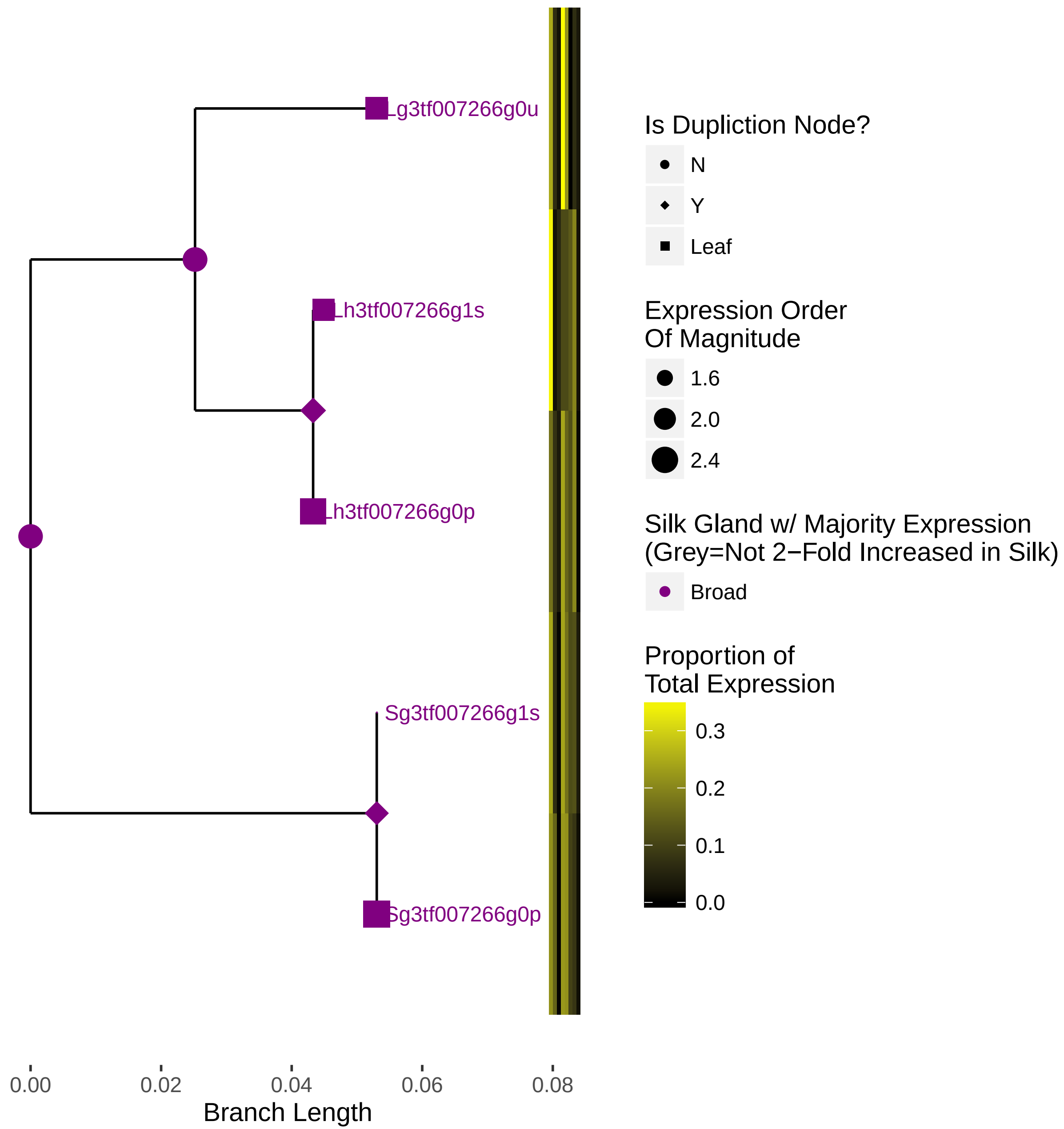

Lg3tf007266g0u
Lh3tf007266g1s
Lh3tf007266g0p
Sg3tf007266g1s
Sg3tf007266g0p
Is Dupliction Node?
N
Y
Leaf
Expression Order
Of Magnitude
1.6
2.0
2.4
Silk Gland w/ Majority Expression
(Grey=Not 2−Fold Increased in Silk)
Broad
Proportion of
Total Expression
0.3
0.2
0.1
0.0
0.00
0.02
0.04
0.06
0.08
Branch Length

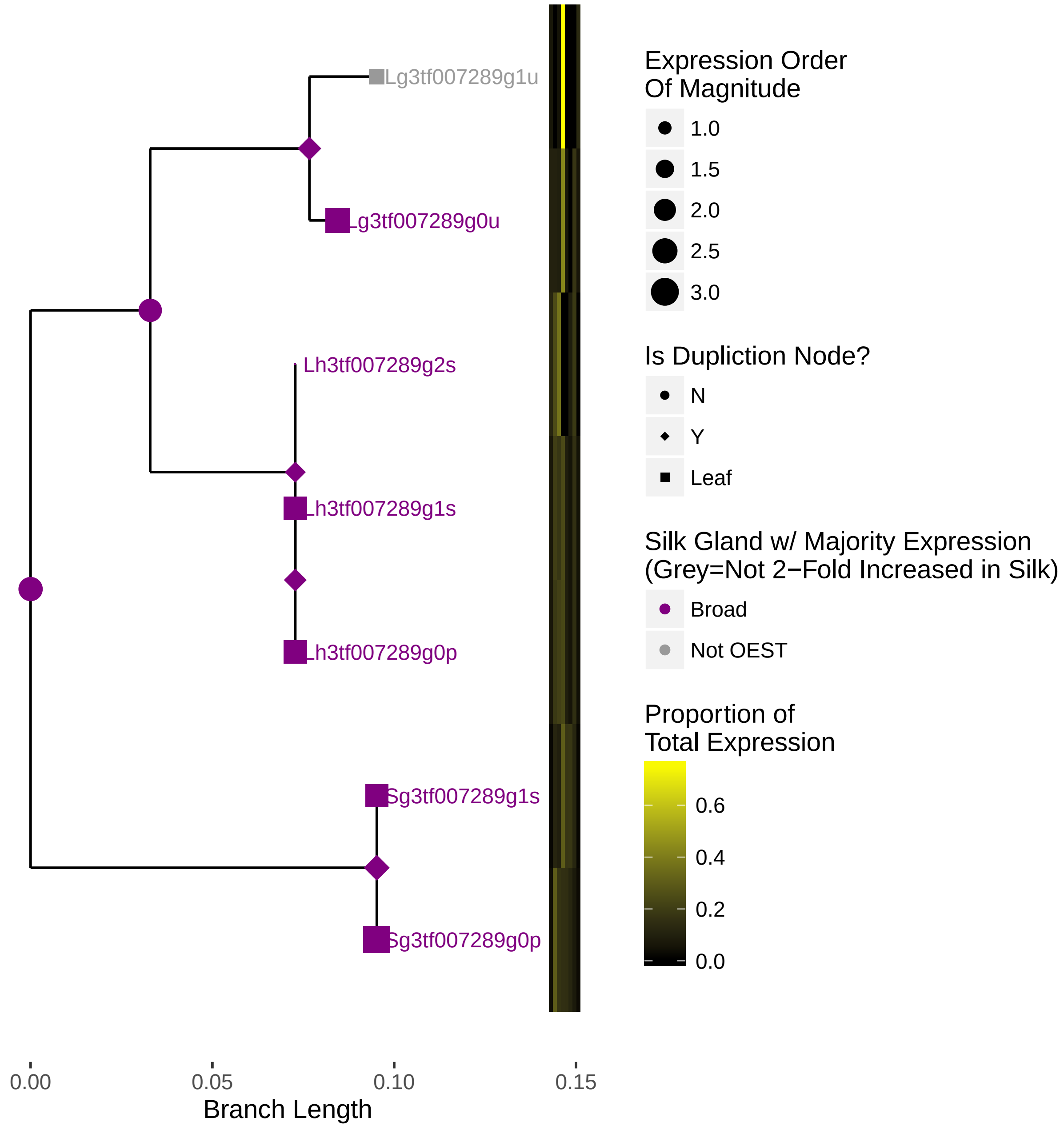

Lg3tf007289g1u
Lg3tf007289g0u
Lh3tf007289g2s
Lh3tf007289g1s
Lh3tf007289g0p
Sg3tf007289g1s
Sg3tf007289g0p
0.00
0.05
0.10
0.15
Branch Length
Expression Order Of Magnitude
1.0
1.5
2.0
2.5
3.0
Is Dupliction Node?
N
Y
Leaf
Silk Gland w/ Majority Expression (Grey=Not 2−Fold Increased in Silk)
Broad
Not OEST
Proportion of Total Expression
0.6
0.4
0.2
0.0

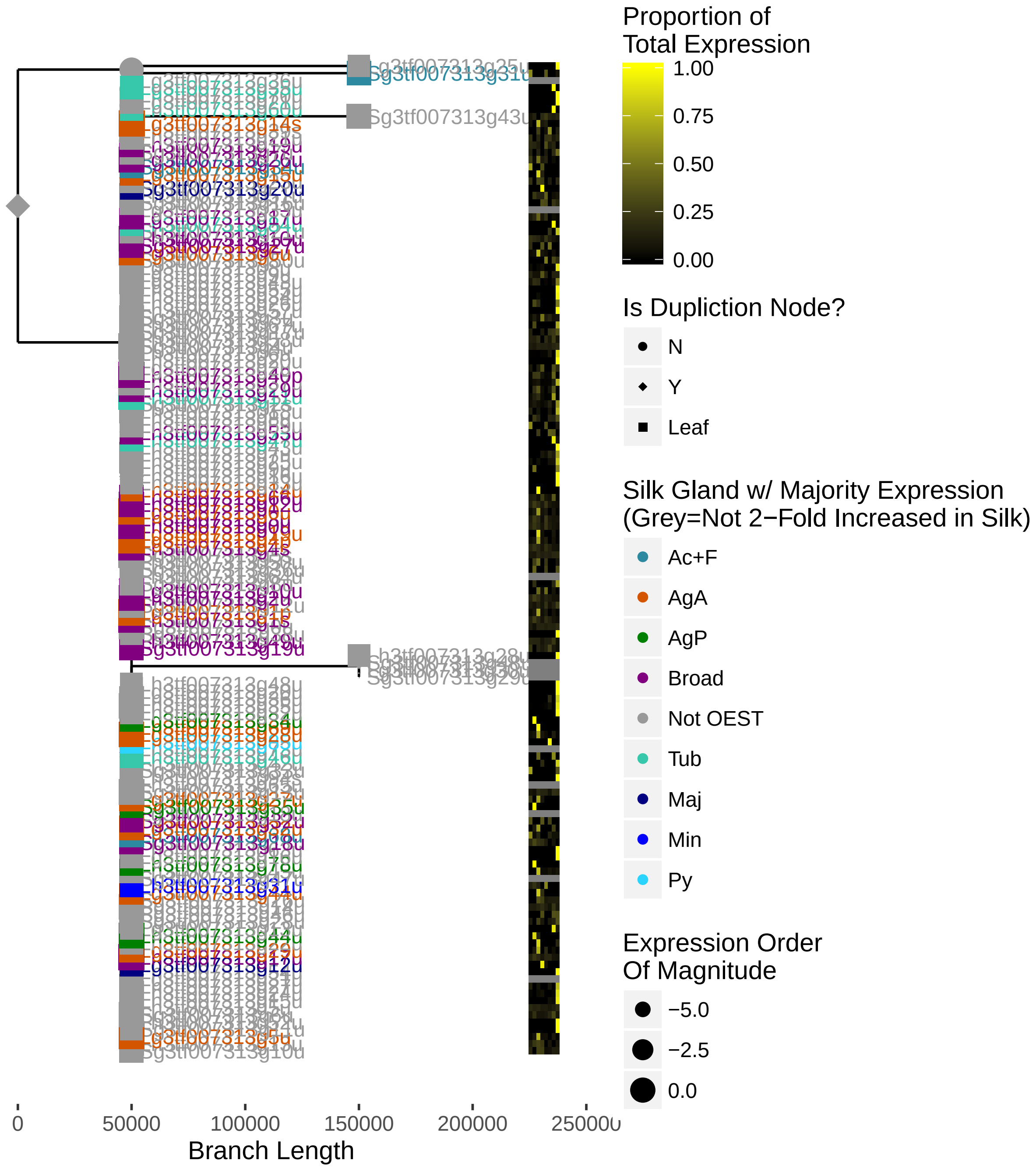

Proportion of Total Expression
1.00
0.75
0.50
0.25
0.00
Is Dupliction Node?
N
Y
Leaf
Silk Gland w/ Majority Expression (Grey=Not 2−Fold Increased in Silk)
Ac+F
AgA
AgP
Broad
Not OEST
Tub
Maj
Min
Py
Expression Order Of Magnitude
−5.0
−2.5
0.0
Sg3tf007313g31u
Sg3tf007313g43u
Sg3tf007313g20u
Sg3tf007313g19u
Sg3tf007313g10u
0
50000
100000
150000
200000
250000
Branch Length

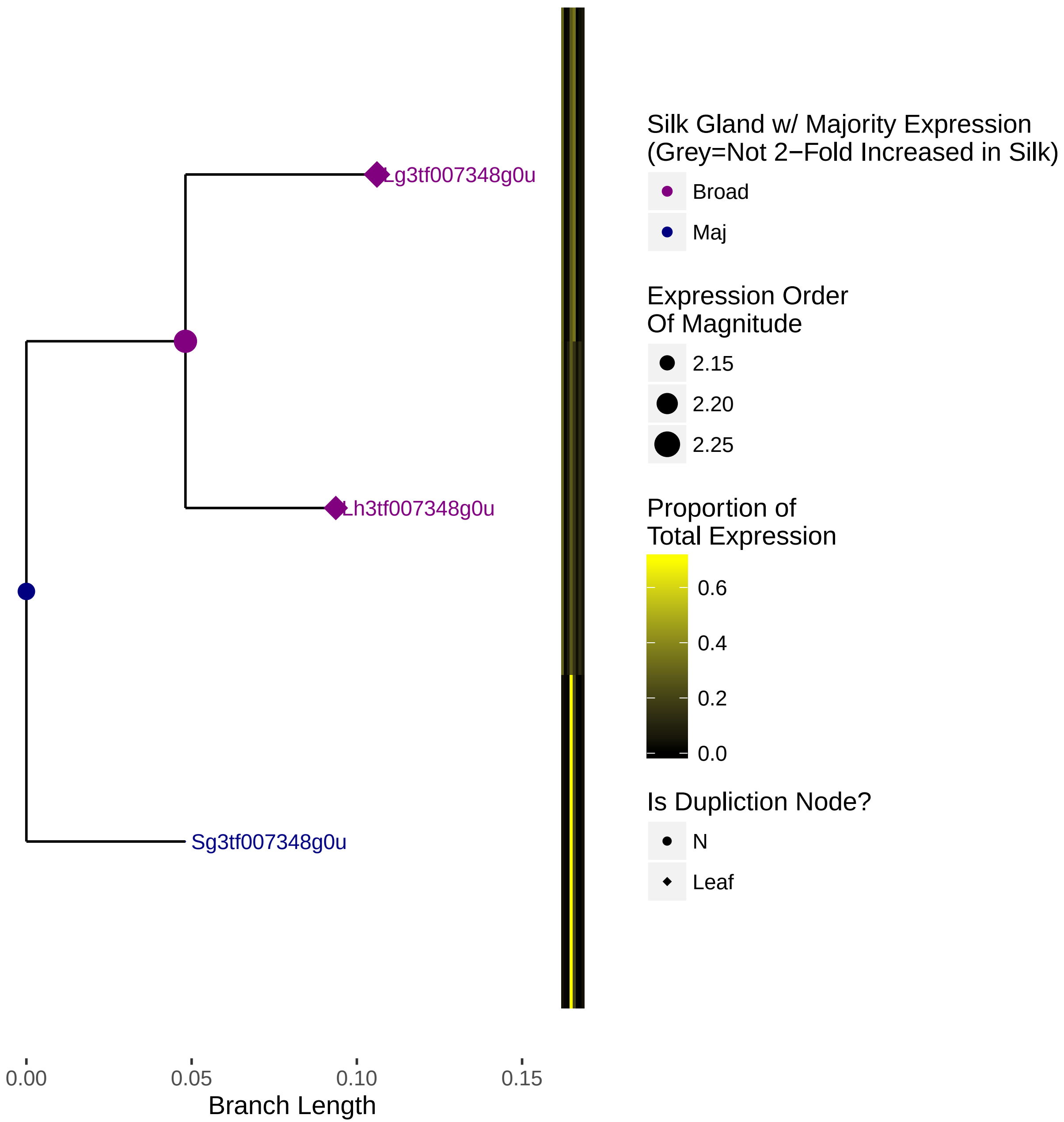
Lg3tf007348g0u
Lh3tf007348g0u
Sg3tf007348g0u
Silk Gland w/ Majority Expression
(Grey=Not 2−Fold Increased in Silk)
Broad
Maj
Expression Order
Of Magnitude
2.15
2.20
2.25
Proportion of
Total Expression
0.6
0.4
0.2
0.0
Is Dupliction Node?
N
Leaf
0.00
0.05
0.10
0.15
Branch Length

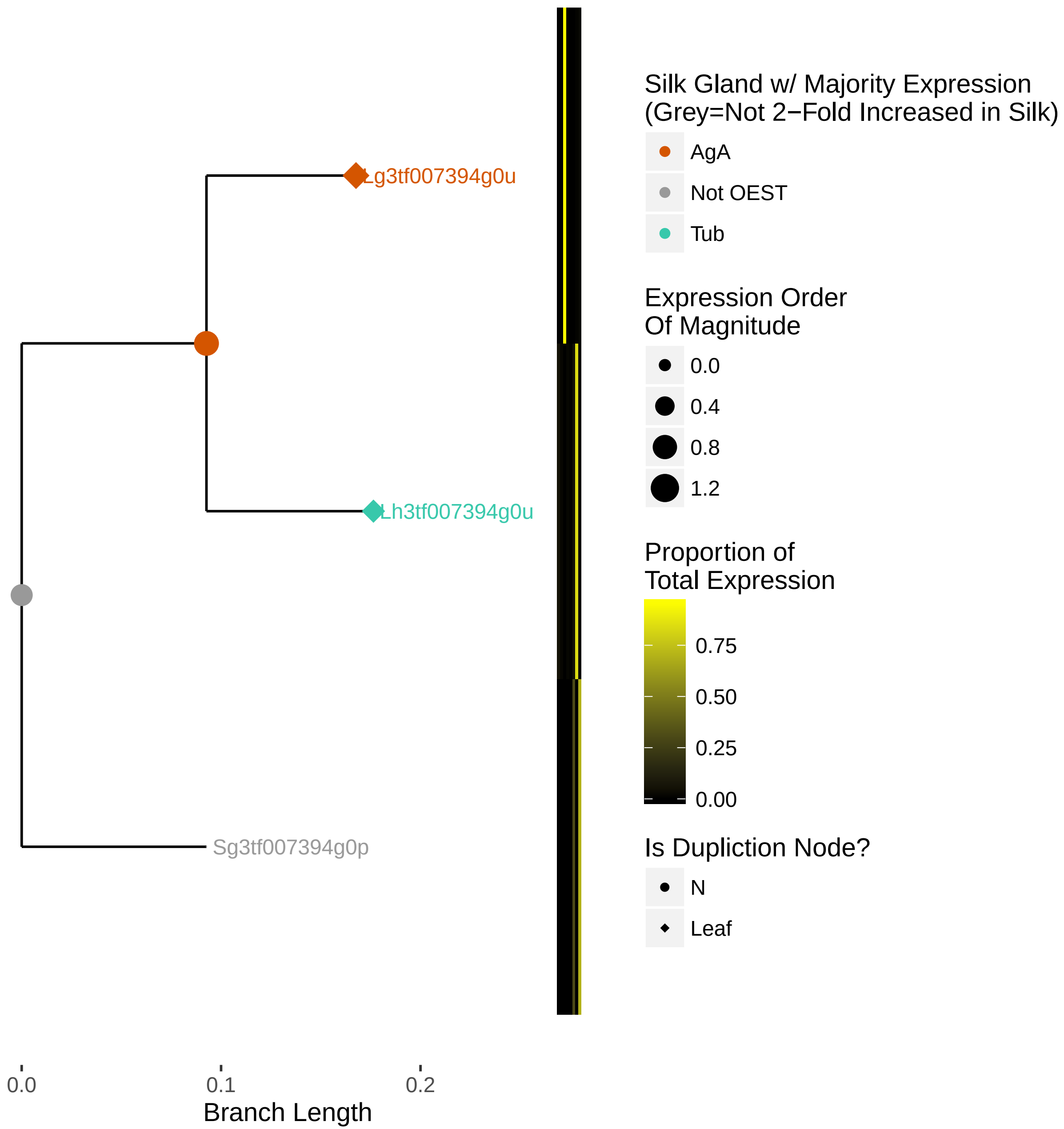
Lg3tf007394g0u
Lh3tf007394g0u
Sg3tf007394g0p
0.0
0.1
0.2
Branch Length
Silk Gland w/ Majority Expression
(Grey=Not 2−Fold Increased in Silk)
AgA
Not OEST
Tub
Expression Order
Of Magnitude
0.0
0.4
0.8
1.2
Proportion of
Total Expression
0.75
0.50
0.25
0.00
Is Dupliction Node?
N
Leaf

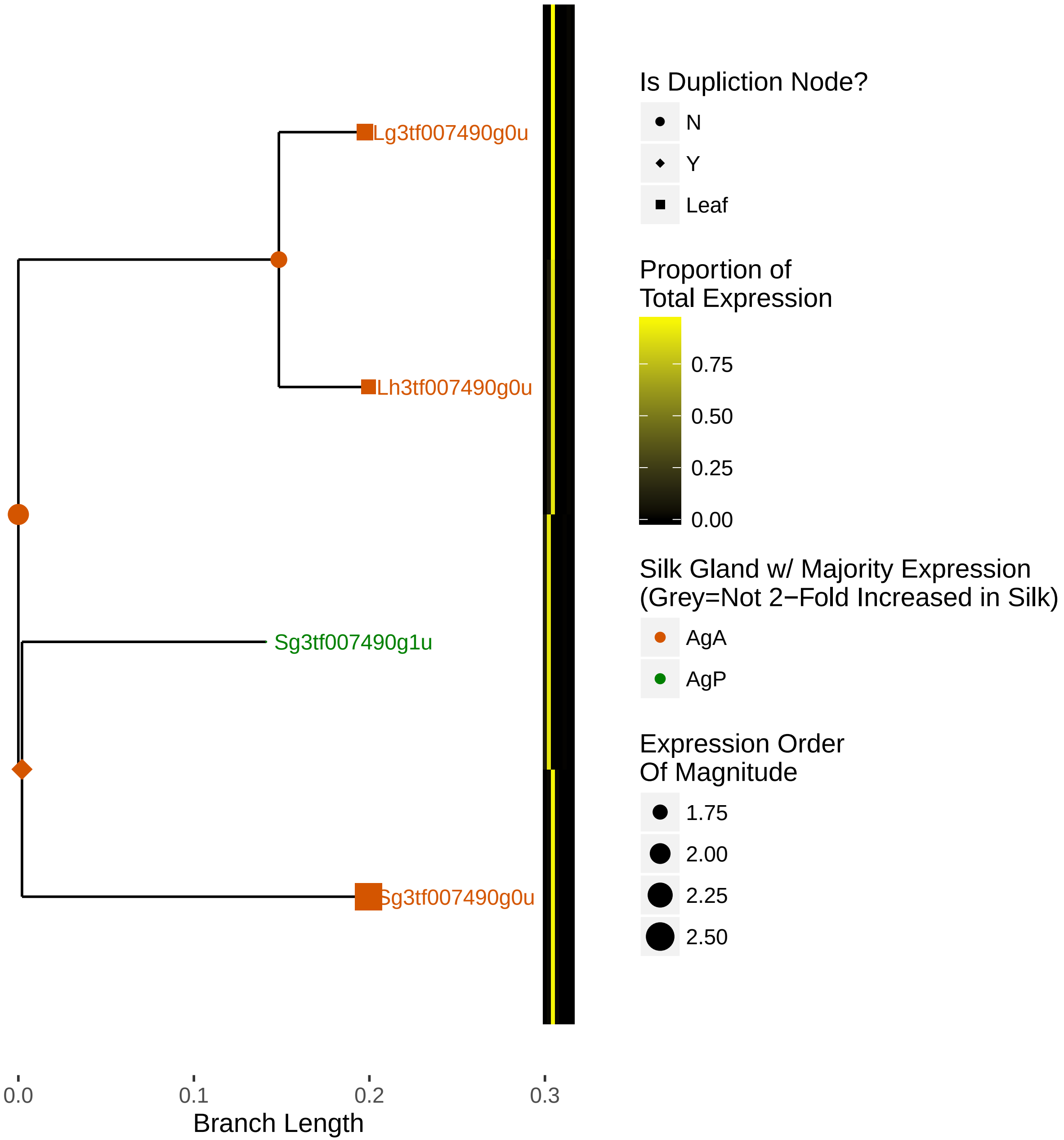

Lg3tf007490g0u
Lh3tf007490g0u
Sg3tf007490g1u
Sg3tf007490g0u
0.0
0.1
0.2
0.3
Branch Length
Is Dupliction Node?
N
Y
Leaf
Proportion of
Total Expression
0.75
0.50
0.25
0.00
Silk Gland w/ Majority Expression
(Grey=Not 2−Fold Increased in Silk)
AgA
AgP
Expression Order
Of Magnitude
1.75
2.00
2.25
2.50

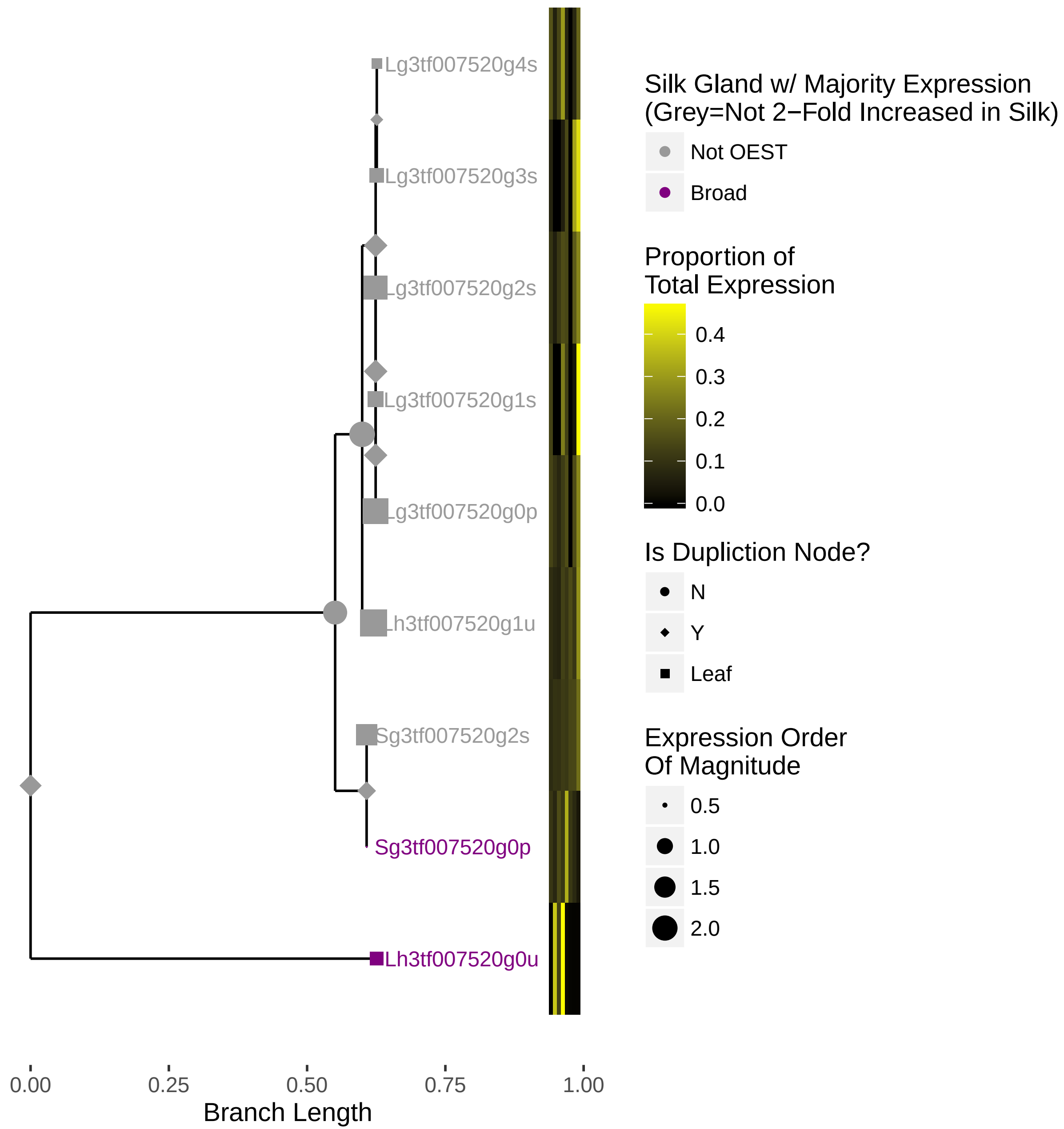

Lg3tf007520g4s
Lg3tf007520g3s
Lg3tf007520g2s
Lg3tf007520g1s
Lg3tf007520g0p
Lh3tf007520g1u
Sg3tf007520g2s
Sg3tf007520g0p
Lh3tf007520g0u
0.00
0.25
0.50
0.75
1.00
Branch Length
Silk Gland w/ Majority Expression
(Grey=Not 2−Fold Increased in Silk)
Not OEST
Broad
Proportion of
Total Expression
0.4
0.3
0.2
0.1
0.0
Is Dupliction Node?
N
Y
Leaf
Expression Order
Of Magnitude
0.5
1.0
1.5
2.0

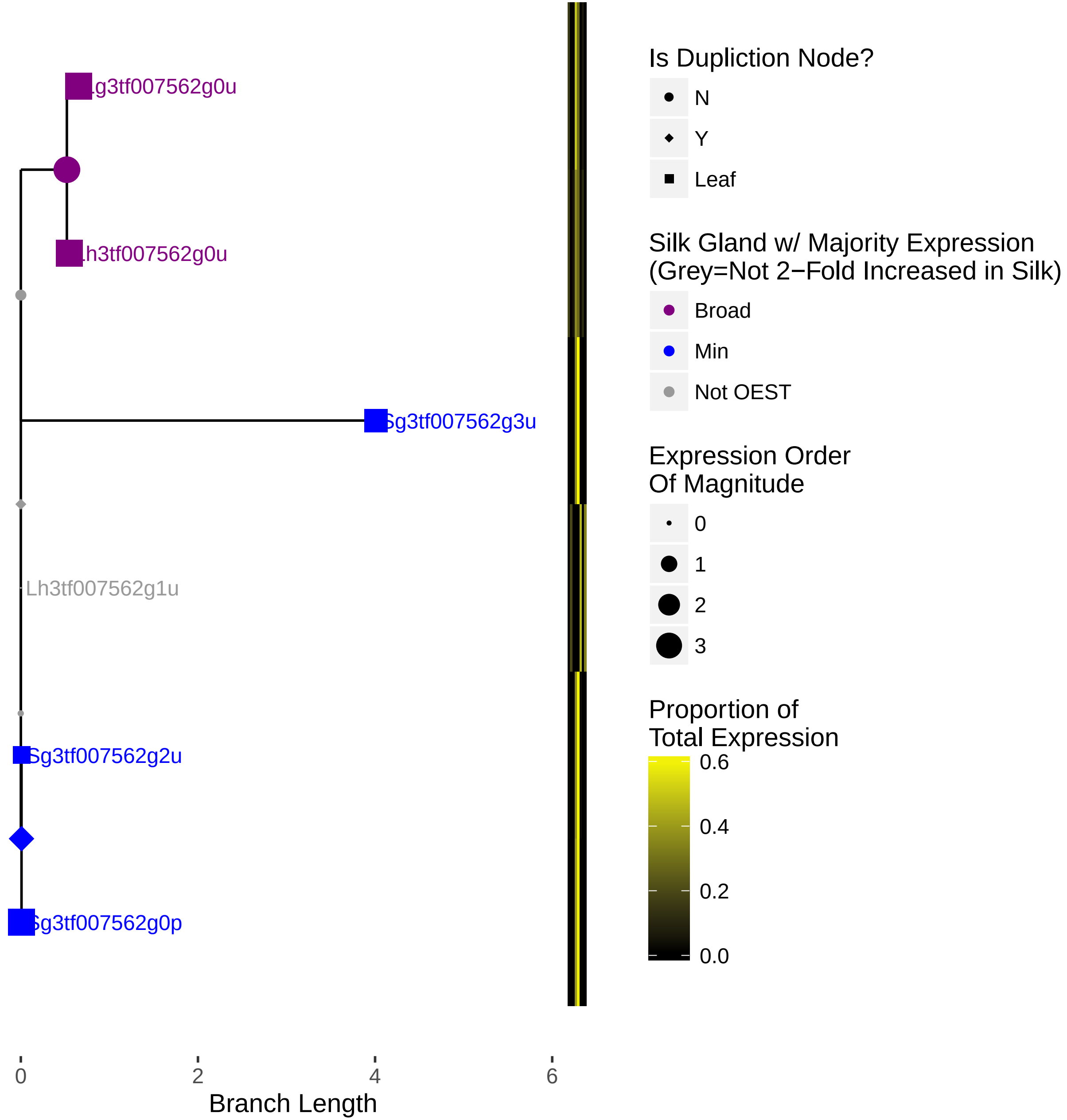

Lg3tf007562g0u
Lh3tf007562g0u
Sg3tf007562g3u
Lh3tf007562g1u
Sg3tf007562g2u
Sg3tf007562g0p
0
2
4
6
Branch Length
Is Dupliction Node?
N
Y
Leaf
Silk Gland w/ Majority Expression
(Grey=Not 2−Fold Increased in Silk)
Broad
Min
Not OEST
Expression Order
Of Magnitude
0
1
2
3
Proportion of
Total Expression
0.6
0.4
0.2
0.0

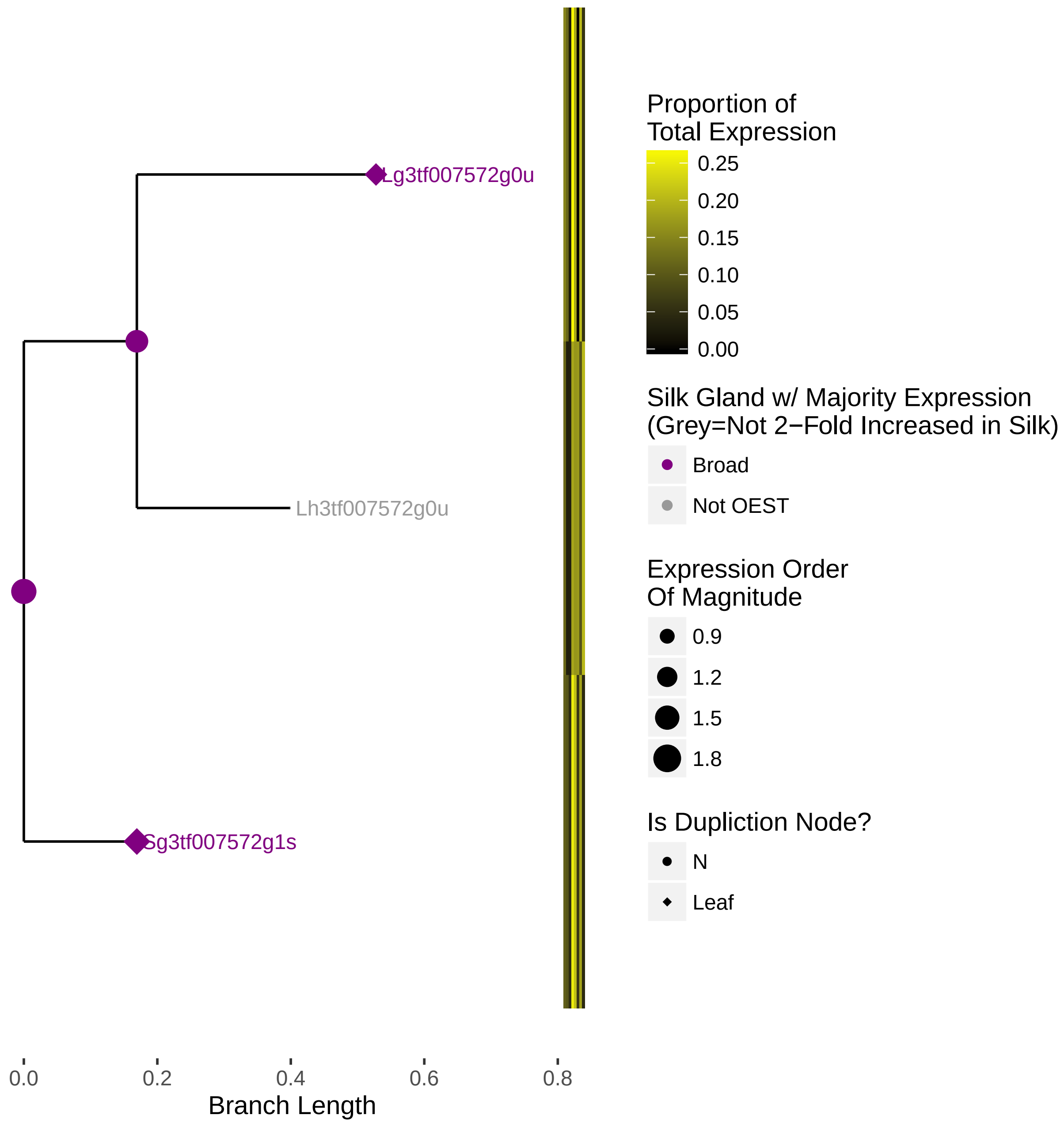

Lg3tf007572g0u
Lh3tf007572g0u
Sg3tf007572g1s
0.0
0.2
0.4
0.6
0.8
Branch Length
Proportion of
Total Expression
0.25
0.20
0.15
0.10
0.05
0.00
Silk Gland w/ Majority Expression
(Grey=Not 2−Fold Increased in Silk)
Broad
Not OEST
Expression Order
Of Magnitude
0.9
1.2
1.5
1.8
Is Dupliction Node?
N
Leaf

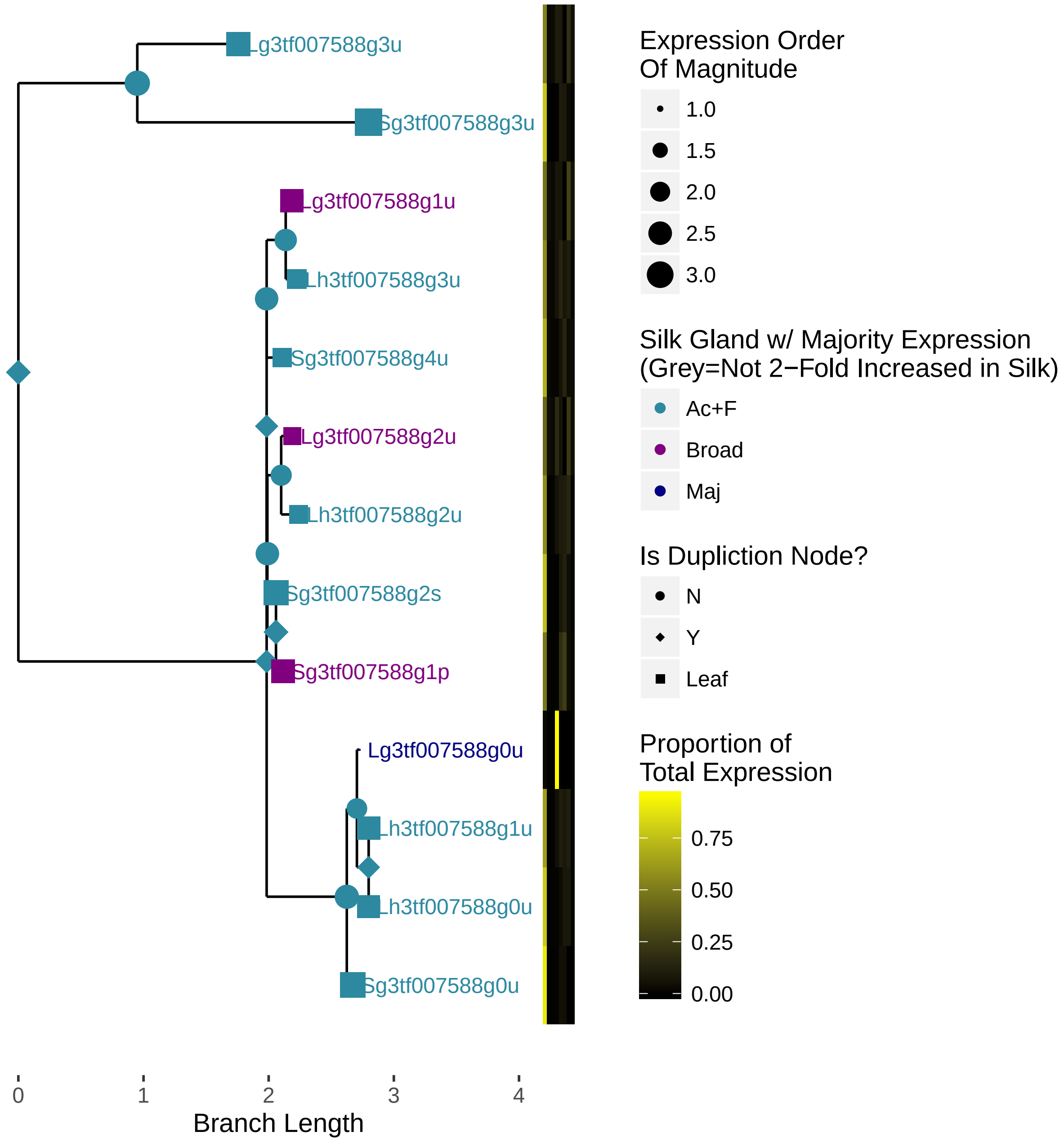

Lg3tf007588g3u
Sg3tf007588g3u
Lg3tf007588g1u
Lh3tf007588g3u
Sg3tf007588g4u
Lg3tf007588g2u
Lh3tf007588g2u
Sg3tf007588g2s
Sg3tf007588g1p
Lg3tf007588g0u
Lh3tf007588g1u
Lh3tf007588g0u
Sg3tf007588g0u
0
1
2
3
4
Branch Length
Expression Order Of Magnitude
1.0
1.5
2.0
2.5
3.0
Silk Gland w/ Majority Expression (Grey=Not 2−Fold Increased in Silk)
Ac+F
Broad
Maj
Is Dupliction Node?
N
Y
Leaf
Proportion of Total Expression
0.75
0.50
0.25
0.00

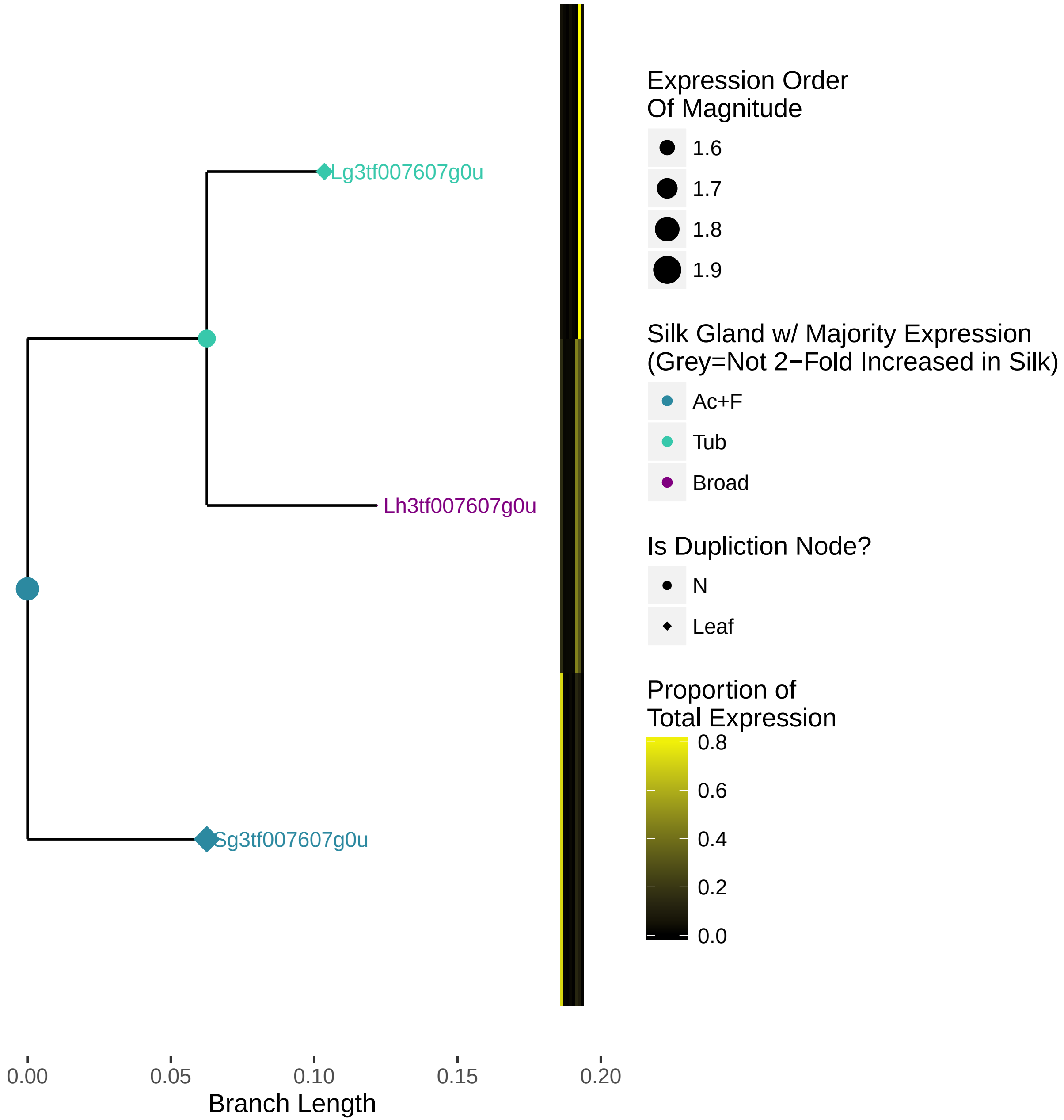

Lg3tf007607g0u
Lh3tf007607g0u
Sg3tf007607g0u
Expression Order
Of Magnitude
1.6
1.7
1.8
1.9
Silk Gland w/ Majority Expression
(Grey=Not 2−Fold Increased in Silk)
Ac+F
Tub
Broad
Is Dupliction Node?
N
Leaf
Proportion of
Total Expression
0.8
0.6
0.4
0.2
0.0
0.00
0.05
0.10
0.15
0.20
Branch Length

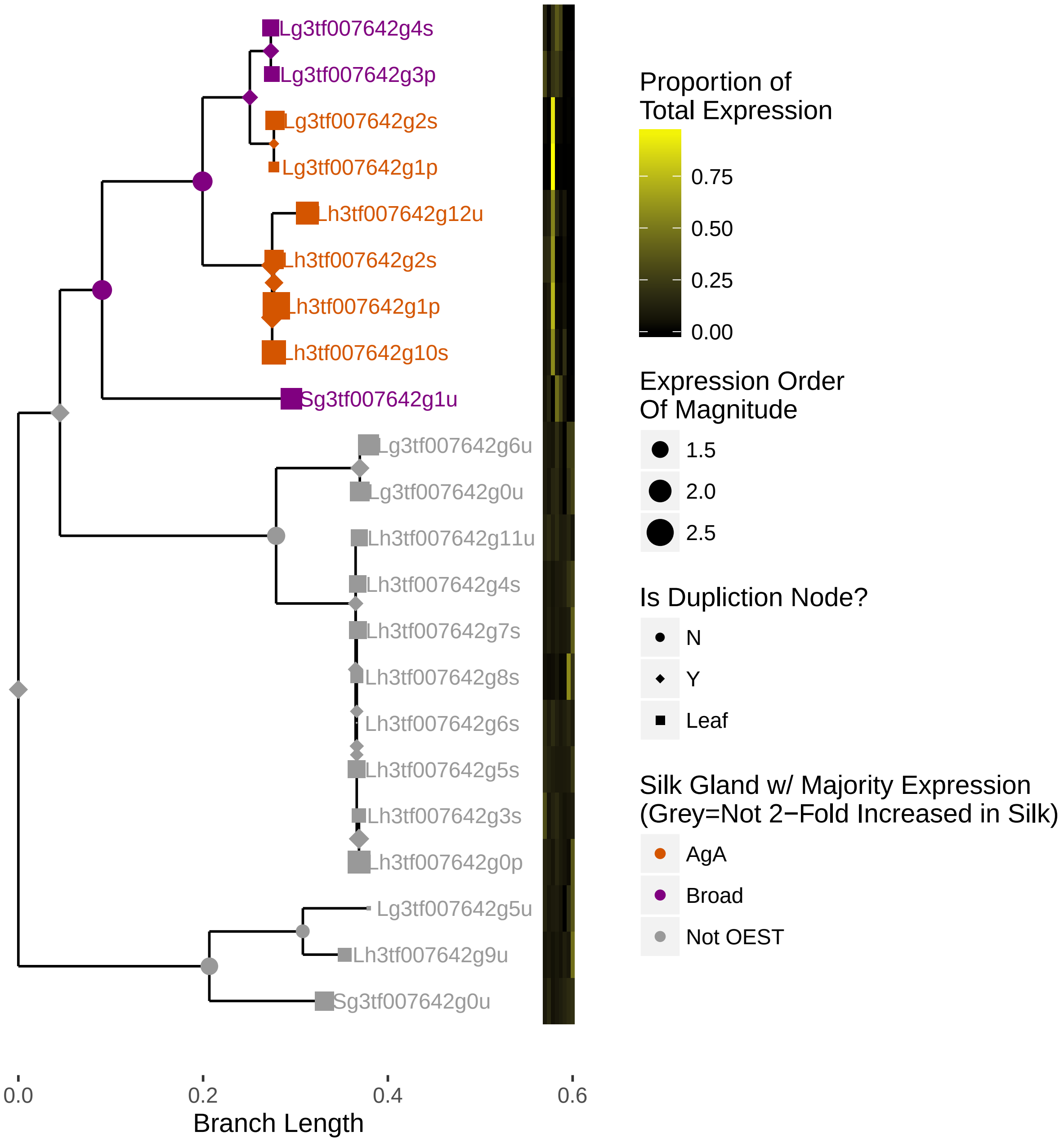

Lg3tf007642g4s
Lg3tf007642g3p
Lg3tf007642g2s
Lg3tf007642g1p
Lh3tf007642g12u
Lh3tf007642g2s
Lh3tf007642g1p
Lh3tf007642g10s
Sg3tf007642g1u
Lg3tf007642g6u
Lg3tf007642g0u
Lh3tf007642g11u
Lh3tf007642g4s
Lh3tf007642g7s
Lh3tf007642g8s
Lh3tf007642g6s
Lh3tf007642g5s
Lh3tf007642g3s
Lh3tf007642g0p
Lg3tf007642g5u
Lh3tf007642g9u
Sg3tf007642g0u
0.0
0.2
0.4
0.6
Branch Length
Proportion of Total Expression
0.75
0.50
0.25
0.00
Expression Order Of Magnitude
1.5
2.0
2.5
Is Dupliction Node?
N
Y
Leaf
Silk Gland w/ Majority Expression (Grey=Not 2−Fold Increased in Silk)
AgA
Broad
Not OEST

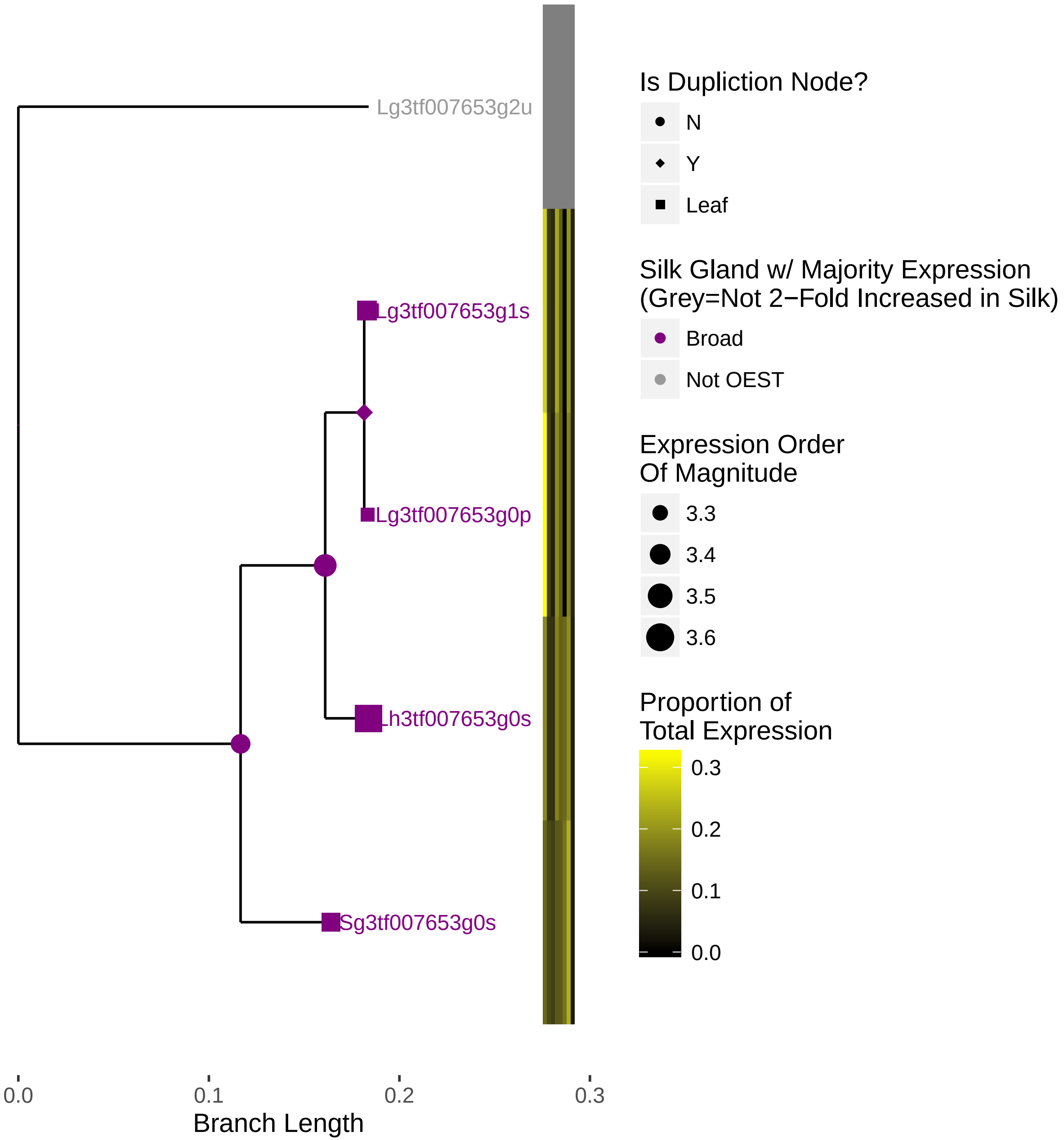

Lg3tf007653g2u
Lg3tf007653g1s
Lg3tf007653g0p
Lh3tf007653g0s
Sg3tf007653g0s
0.0
0.1
0.2
0.3
Branch Length
Is Dupliction Node?
N
Y
Leaf
Silk Gland w/ Majority Expression
(Grey=Not 2−Fold Increased in Silk)
Broad
Not OEST
Expression Order
Of Magnitude
3.3
3.4
3.5
3.6
Proportion of
Total Expression
0.3
0.2
0.1
0.0

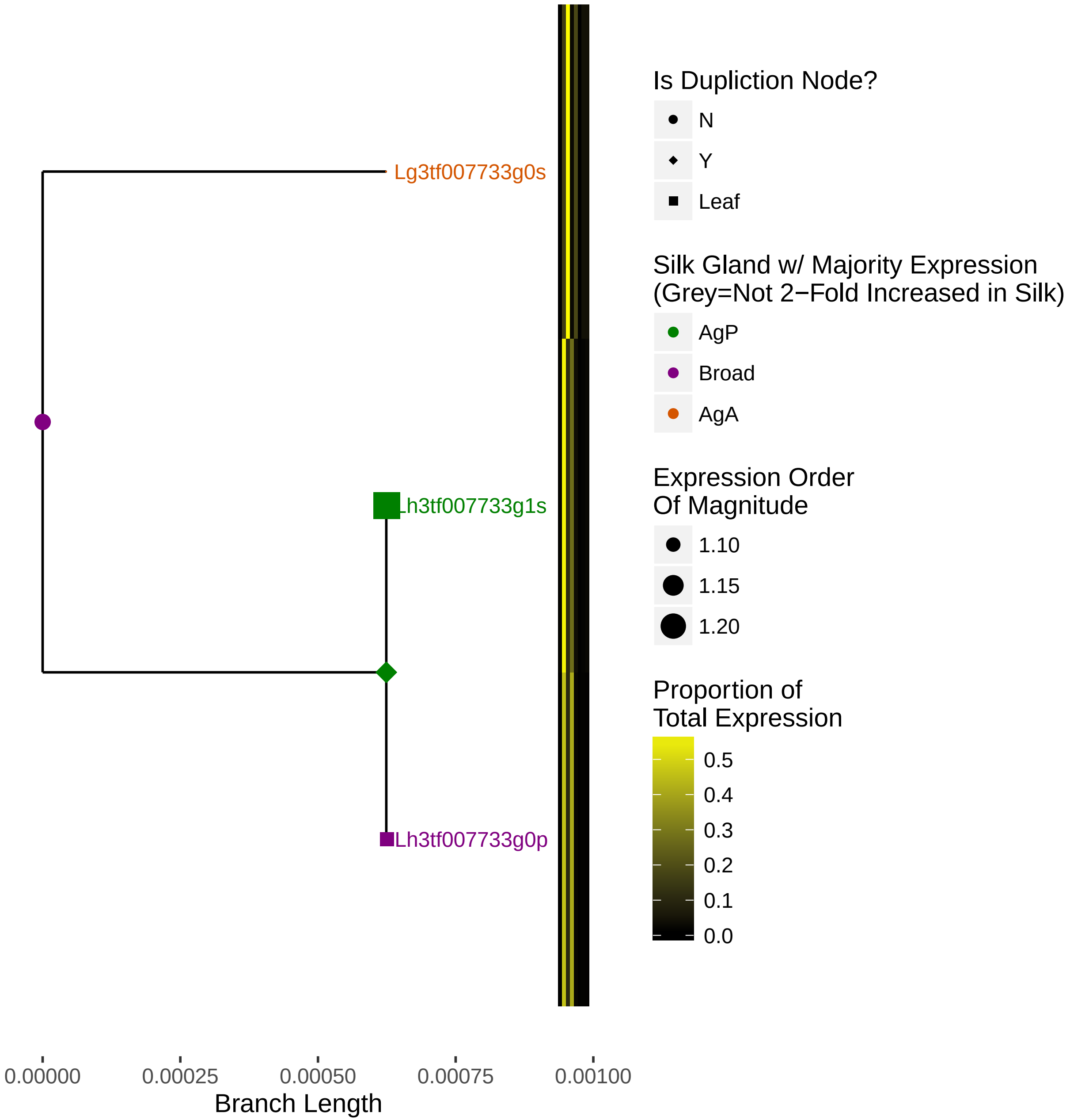

Lg3tf007733g0s
Lh3tf007733g1s
Lh3tf007733g0p
0.00000
0.00025
0.00050
0.00075
0.00100
Branch Length
Is Dupliction Node?
N
Y
Leaf
Silk Gland w/ Majority Expression
(Grey=Not 2−Fold Increased in Silk)
AgP
Broad
AgA
Expression Order
Of Magnitude
1.10
1.15
1.20
Proportion of
Total Expression
0.5
0.4
0.3
0.2
0.1
0.0

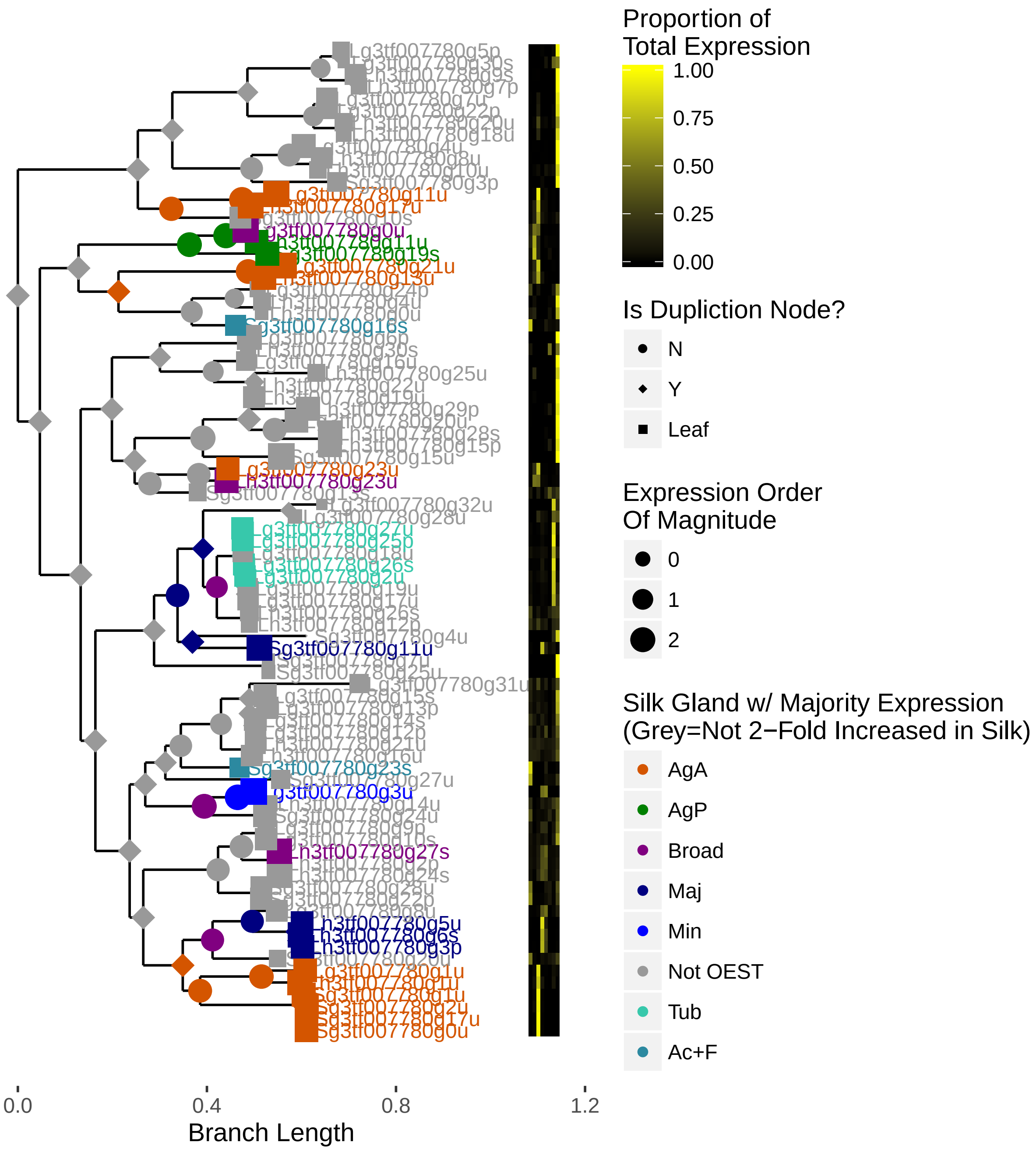

Proportion of Total Expression
1.00
0.75
0.50
0.25
0.00
Is Dupliction Node?
N
Y
Leaf
Expression Order Of Magnitude
0
1
2
Silk Gland w/ Majority Expression (Grey=Not 2−Fold Increased in Silk)
AgA
AgP
Broad
Maj
Min
Not OEST
Tub
Ac+F
0.0
0.4
0.8
1.2
Branch Length

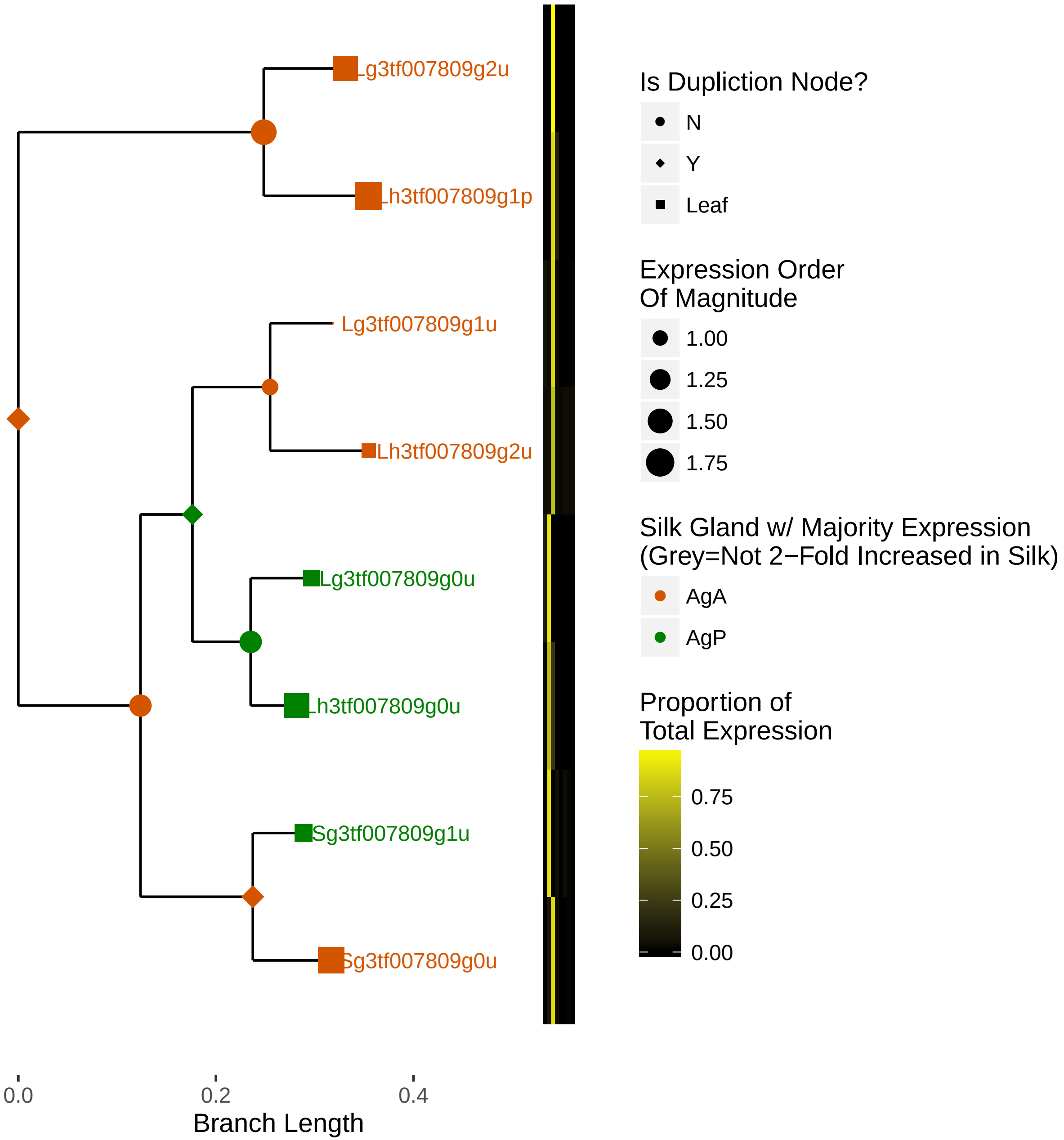

Lg3tf007809g2u
Lh3tf007809g1p
Lg3tf007809g1u
Lh3tf007809g2u
Lg3tf007809g0u
Lh3tf007809g0u
Sg3tf007809g1u
Sg3tf007809g0u
0.0
0.2
0.4
Branch Length
Is Dupliction Node?
N
Y
Leaf
Expression Order
Of Magnitude
1.00
1.25
1.50
1.75
Silk Gland w/ Majority Expression
(Grey=Not 2−Fold Increased in Silk)
AgA
AgP
Proportion of
Total Expression
0.75
0.50
0.25
0.00

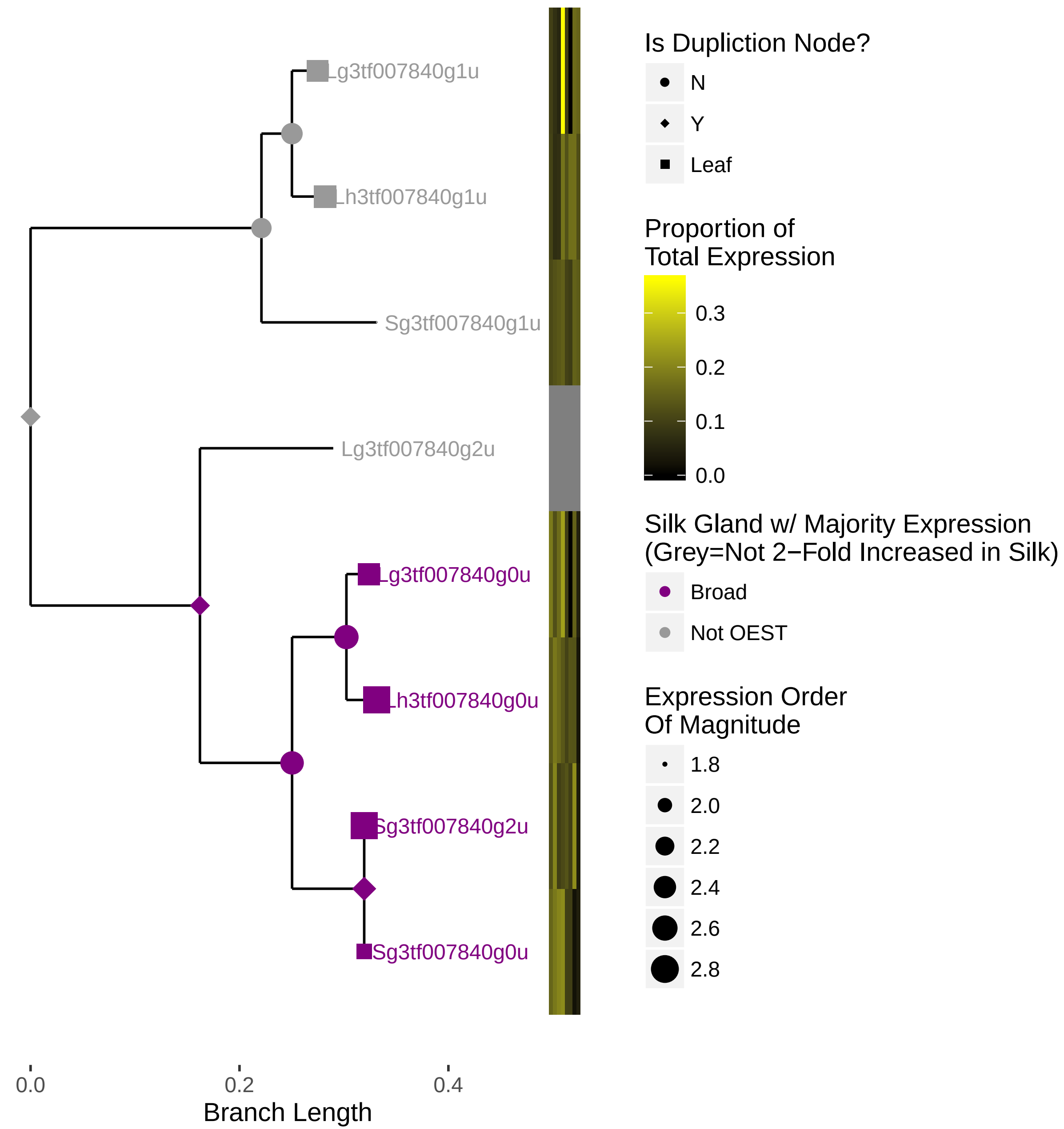

Lg3tf007840g1u
Lh3tf007840g1u
Sg3tf007840g1u
Lg3tf007840g2u
Lg3tf007840g0u
Lh3tf007840g0u
Sg3tf007840g2u
Sg3tf007840g0u
0.0
0.2
0.4
Branch Length
Is Dupliction Node?
N
Y
Leaf
Proportion of
Total Expression
0.3
0.2
0.1
0.0
Silk Gland w/ Majority Expression
(Grey=Not 2−Fold Increased in Silk)
Broad
Not OEST
Expression Order
Of Magnitude
1.8
2.0
2.2
2.4
2.6
2.8

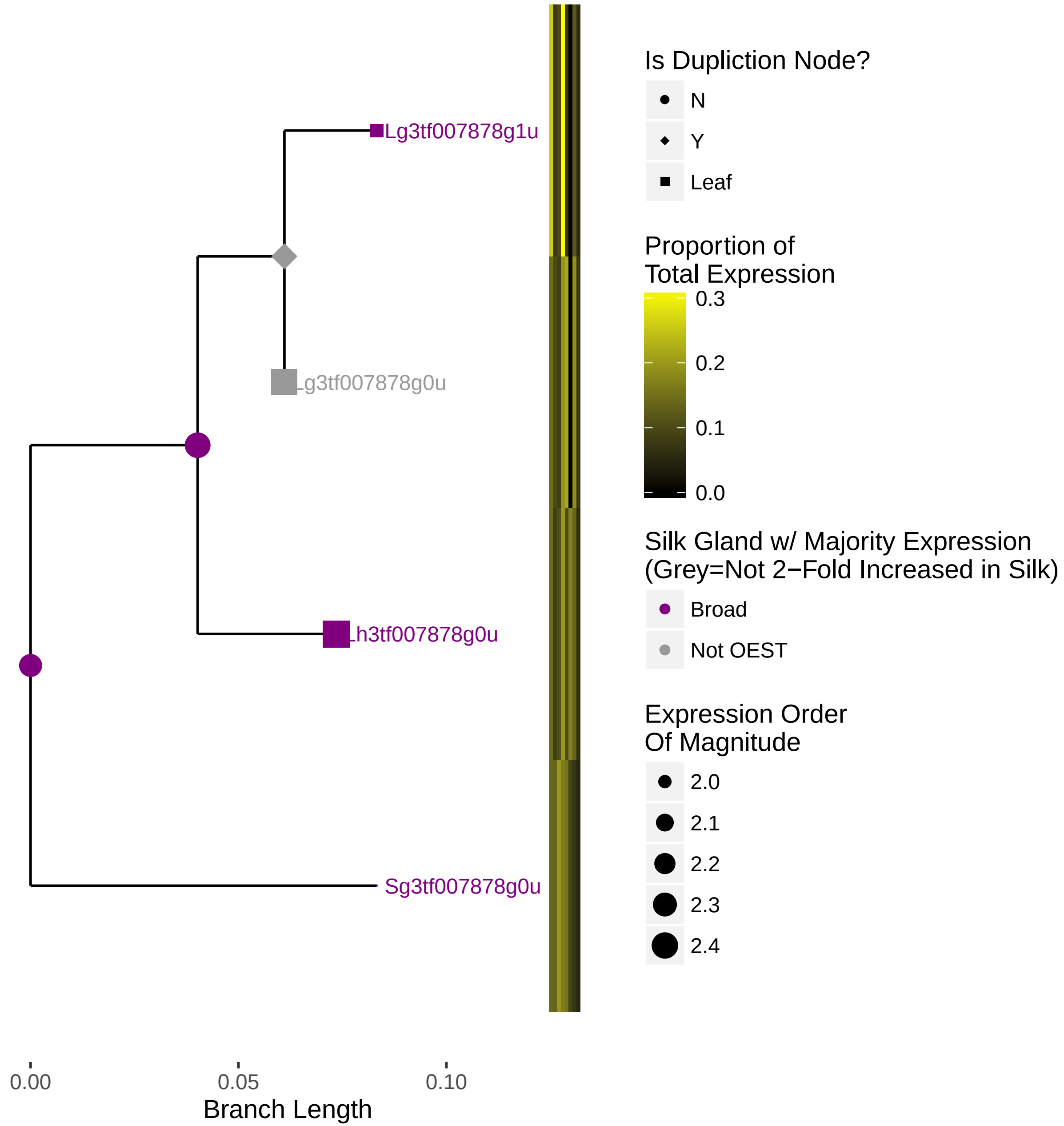

Lg3tf007878g1u
Lg3tf007878g0u
Lh3tf007878g0u
Sg3tf007878g0u
0.00
0.05
0.10
Branch Length
Is Dupliction Node?
N
Y
Leaf
Proportion of
Total Expression
0.3
0.2
0.1
0.0
Silk Gland w/ Majority Expression
(Grey=Not 2−Fold Increased in Silk)
Broad
Not OEST
Expression Order
Of Magnitude
2.0
2.1
2.2
2.3
2.4

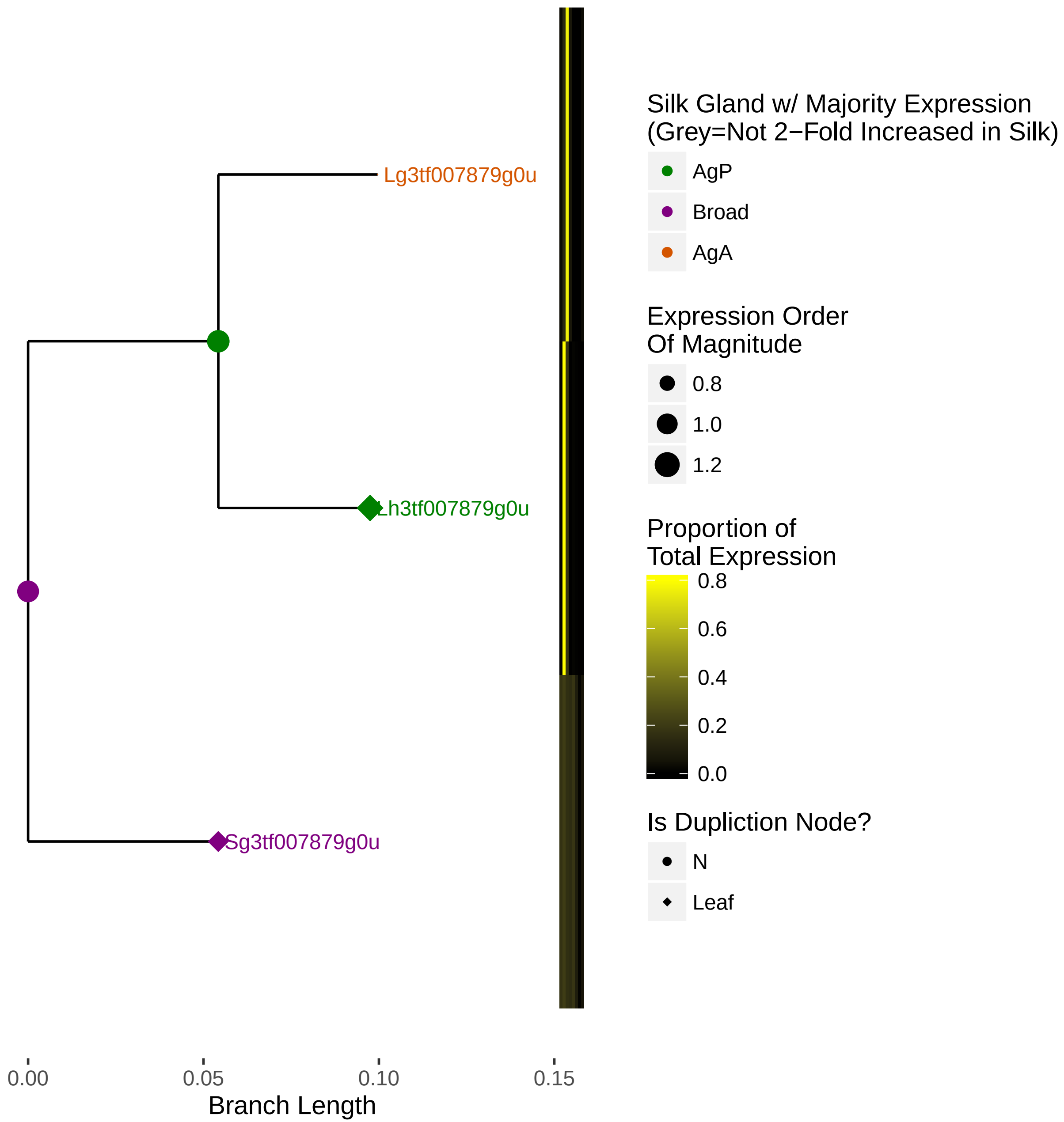
Lg3tf007879g0u
Lh3tf007879g0u
Sg3tf007879g0u
0.00
0.05
0.10
0.15
Branch Length
Silk Gland w/ Majority Expression
(Grey=Not 2−Fold Increased in Silk)
AgP
Broad
AgA
Expression Order
Of Magnitude
0.8
1.0
1.2
Proportion of
Total Expression
0.8
0.6
0.4
0.2
0.0
Is Dupliction Node?
N
Leaf

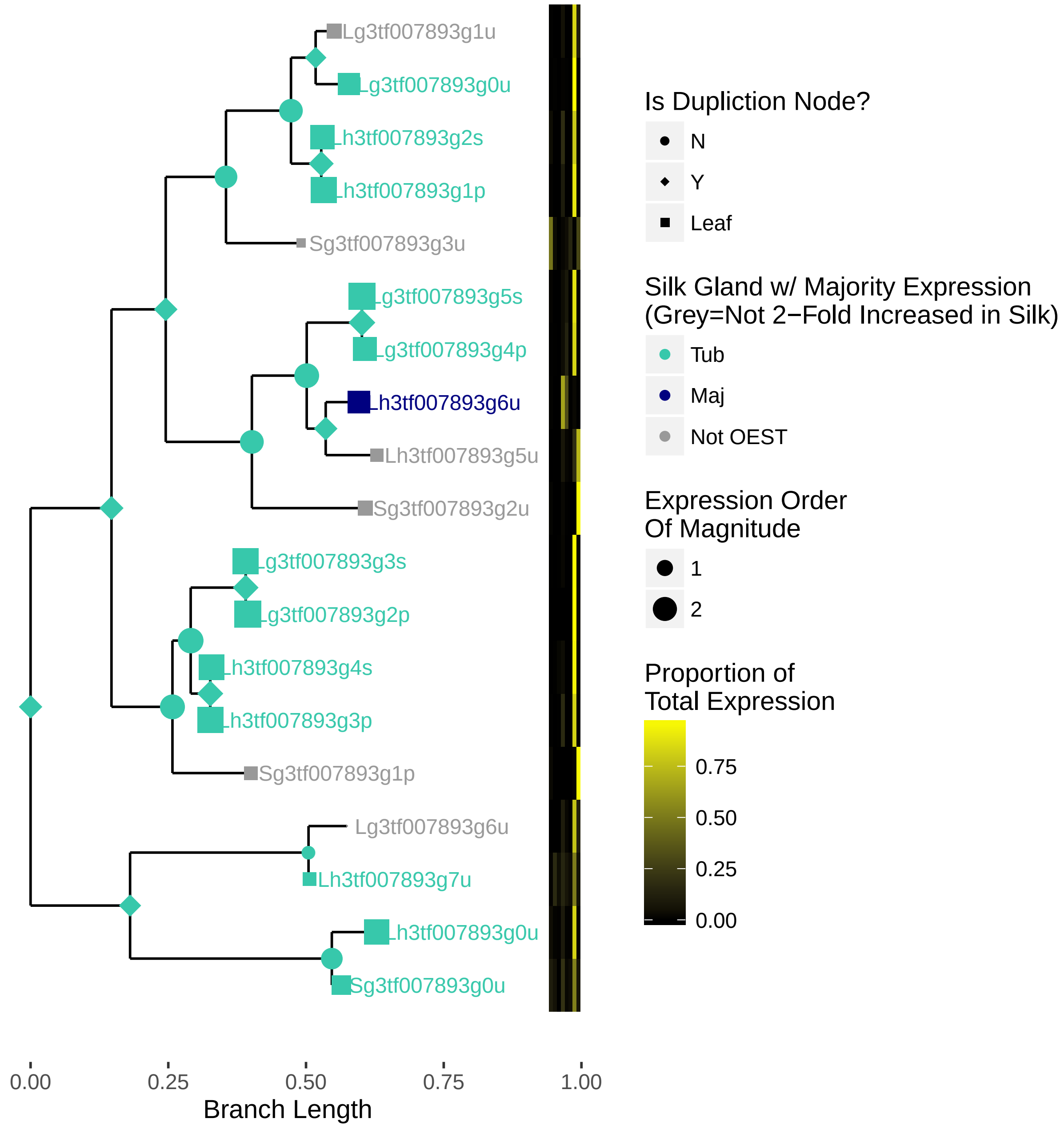

Lg3tf007893g1u
g3tf007893g0u
h3tf007893g2s
h3tf007893g1p
Sg3tf007893g3u
g3tf007893g5s
g3tf007893g4p
h3tf007893g6u
Lh3tf007893g5u
Sg3tf007893g2u
g3tf007893g3s
g3tf007893g2p
h3tf007893g4s
h3tf007893g3p
Sg3tf007893g1p
Lg3tf007893g6u
Lh3tf007893g7u
h3tf007893g0u
Sg3tf007893g0u
0.00
0.25
0.50
0.75
1.00
Branch Length
Is Dupliction Node?
N
Y
Leaf
Silk Gland w/ Majority Expression
(Grey=Not 2−Fold Increased in Silk)
Tub
Maj
Not OEST
Expression Order
Of Magnitude
1
2
Proportion of
Total Expression
0.75
0.50
0.25
0.00

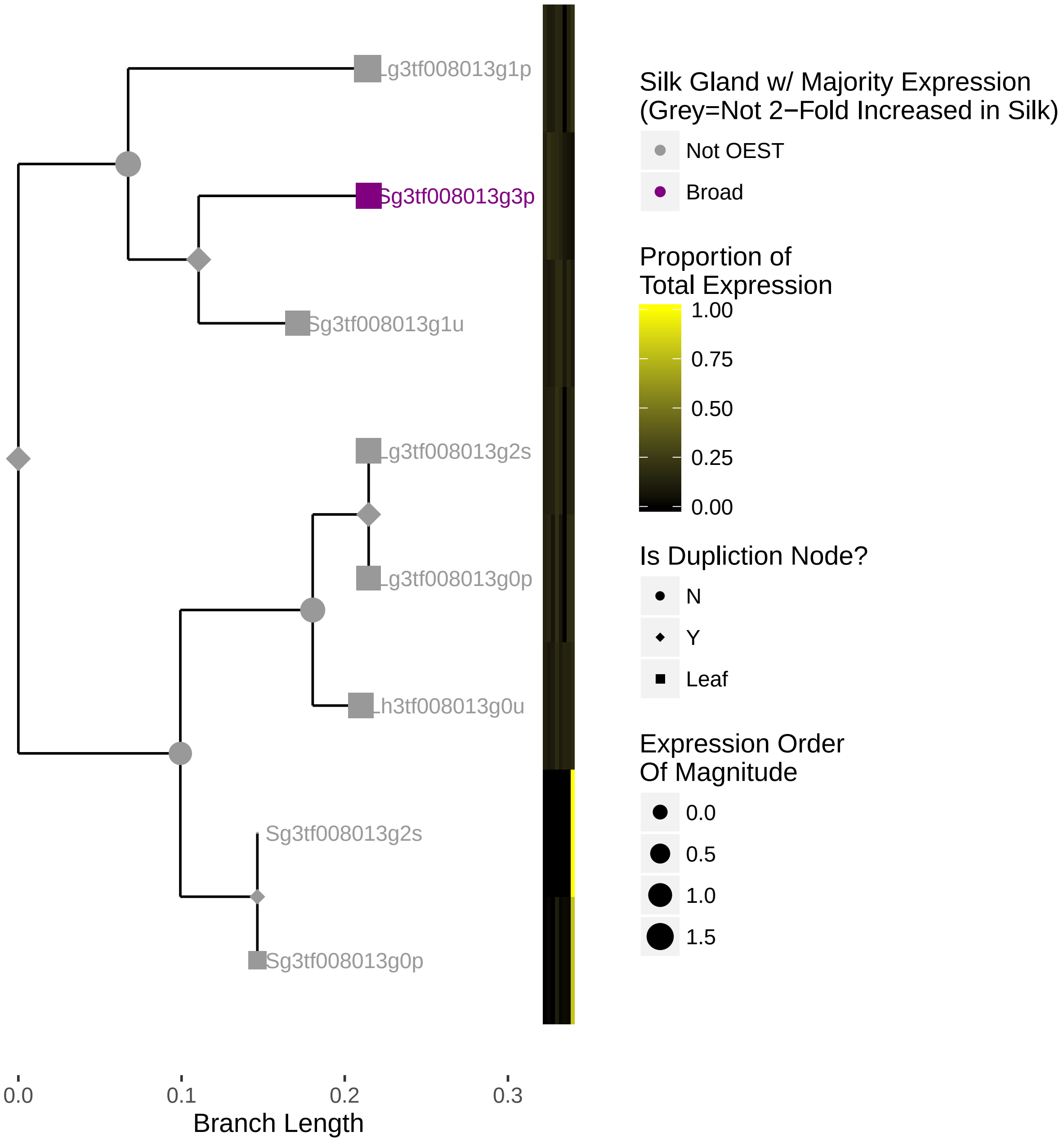

Lg3tf008013g1p
Sg3tf008013g3p
Sg3tf008013g1u
Lg3tf008013g2s
Lg3tf008013g0p
Lh3tf008013g0u
Sg3tf008013g2s
Sg3tf008013g0p
0.0
0.1
0.2
0.3
Branch Length
Silk Gland w/ Majority Expression
(Grey=Not 2−Fold Increased in Silk)
Not OEST
Broad
Proportion of
Total Expression
1.00
0.75
0.50
0.25
0.00
Is Dupliction Node?
N
Y
Leaf
Expression Order
Of Magnitude
0.0
0.5
1.0
1.5

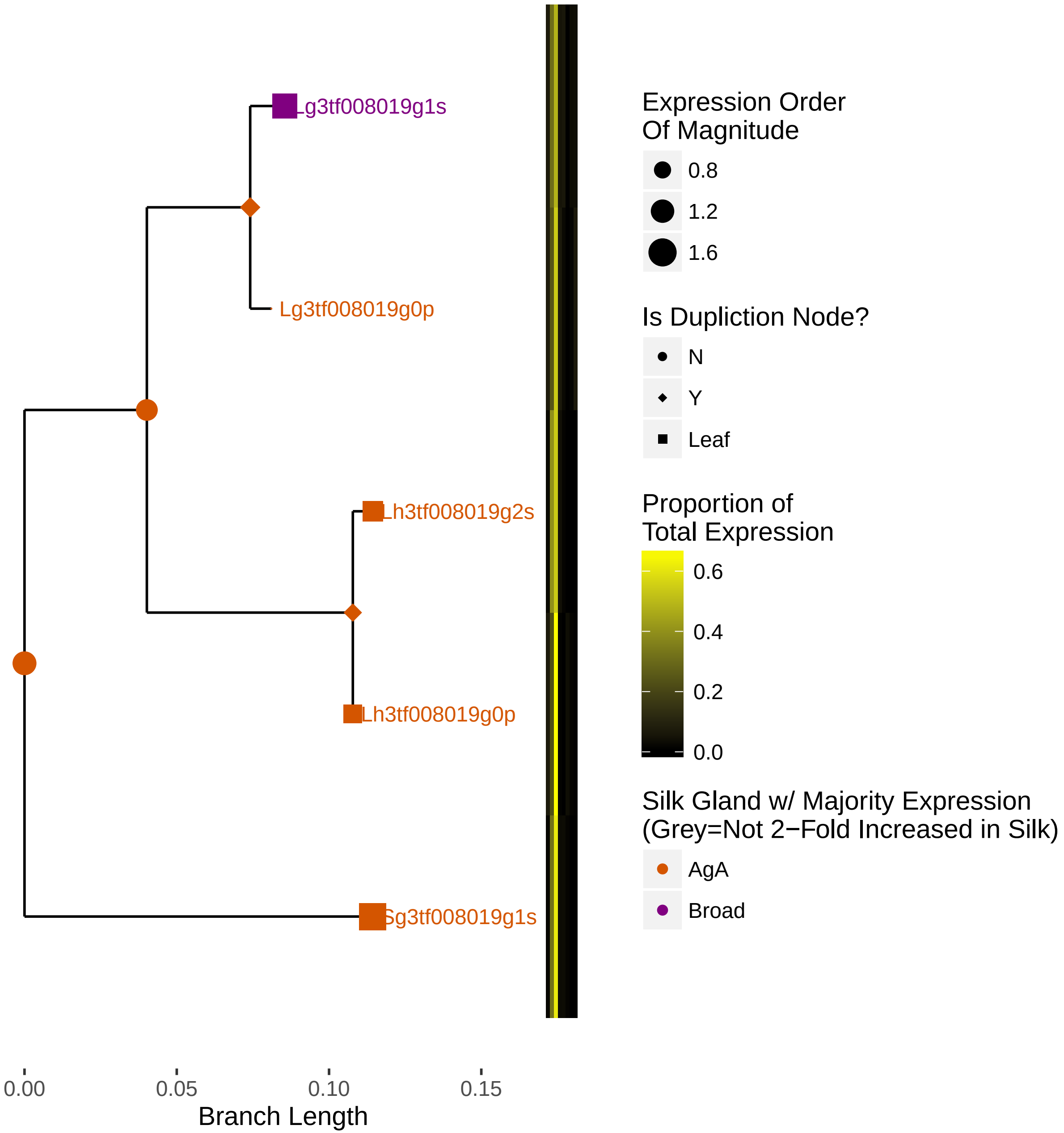

Lg3tf008019g1s
Lg3tf008019g0p
Lh3tf008019g2s
Lh3tf008019g0p
Sg3tf008019g1s
0.00
0.05
0.10
0.15
Branch Length
Expression Order
Of Magnitude
0.8
1.2
1.6
Is Dupliction Node?
N
Y
Leaf
Proportion of
Total Expression
0.6
0.4
0.2
0.0
Silk Gland w/ Majority Expression
(Grey=Not 2−Fold Increased in Silk)
AgA
Broad

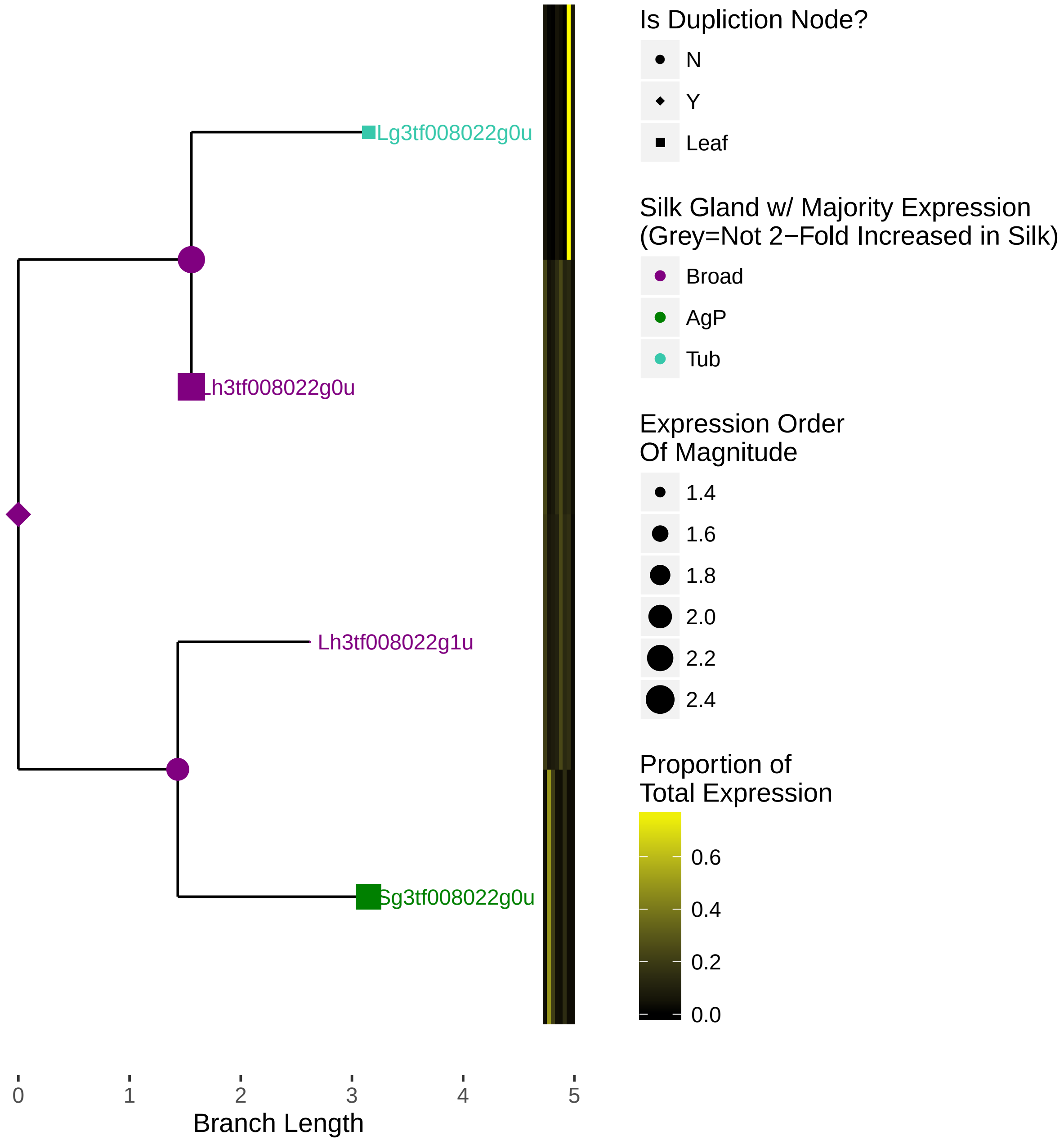
Is Dupliction Node?
N
Y
Leaf
Silk Gland w/ Majority Expression
(Grey=Not 2−Fold Increased in Silk)
Broad
AgP
Tub
Expression Order
Of Magnitude
1.4
1.6
1.8
2.0
2.2
2.4
Proportion of
Total Expression
0.6
0.4
0.2
0.0
Lg3tf008022g0u
Lh3tf008022g0u
Lh3tf008022g1u
Sg3tf008022g0u
0
1
2
3
4
5
Branch Length

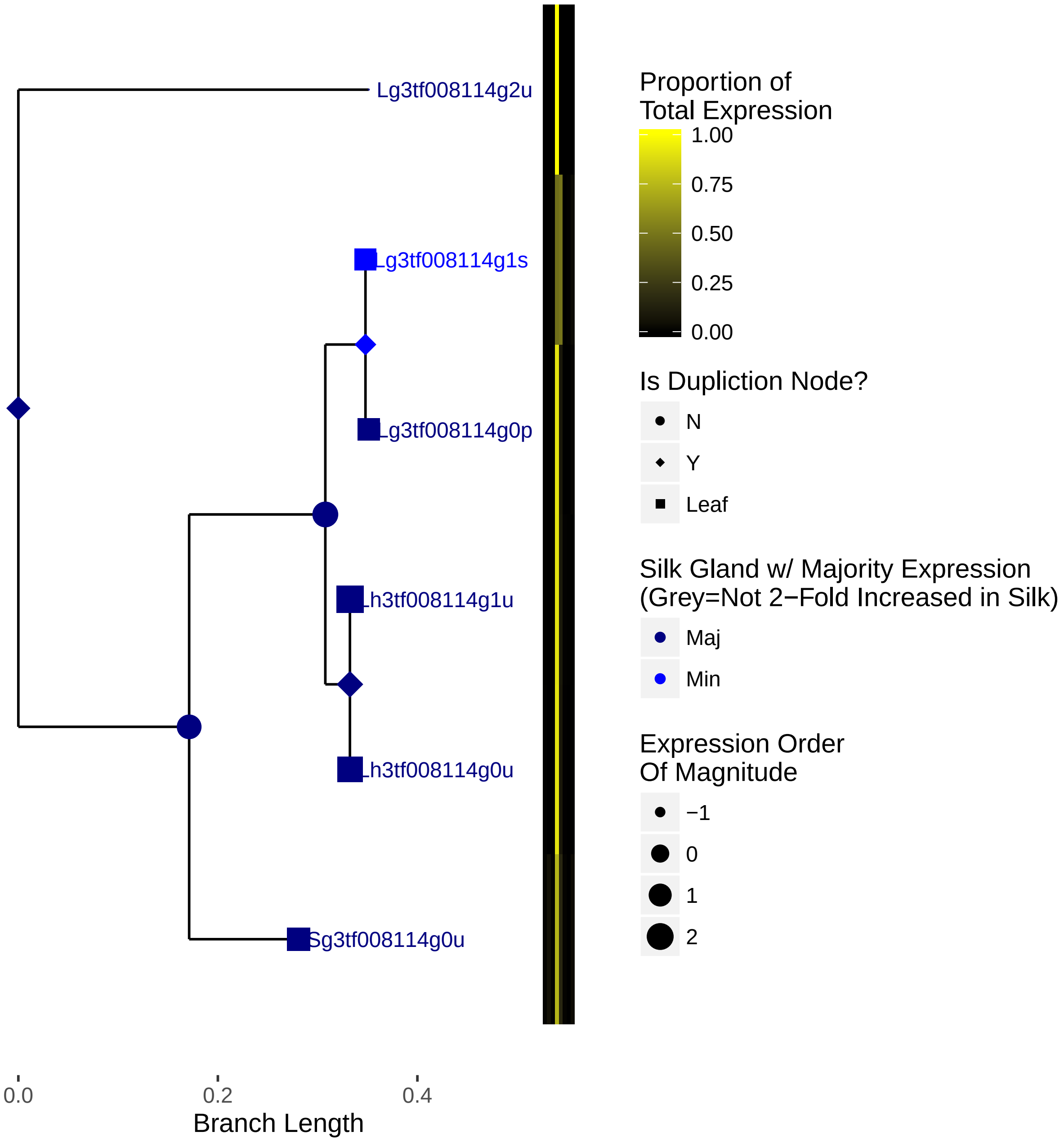
Lg3tf008114g2u
Lg3tf008114g1s
Lg3tf008114g0p
Lh3tf008114g1u
Lh3tf008114g0u
Sg3tf008114g0u
0.0
0.2
0.4
Branch Length
Proportion of Total Expression
1.00
0.75
0.50
0.25
0.00
Is Dupliction Node?
N
Y
Leaf
Silk Gland w/ Majority Expression (Grey=Not 2−Fold Increased in Silk)
Maj
Min
Expression Order Of Magnitude
−1
0
1
2

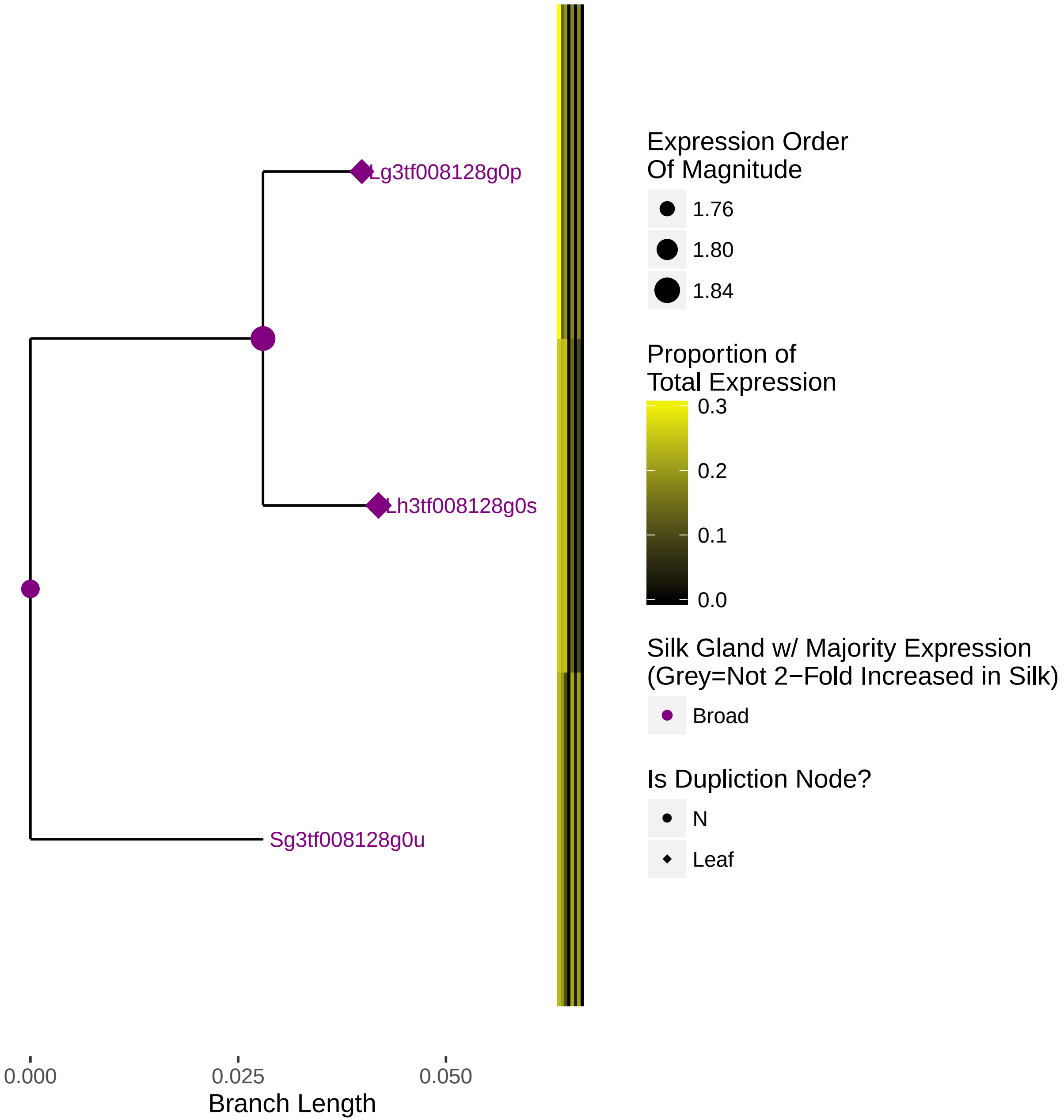
Lg3tf008128g0p
Lh3tf008128g0s
Sg3tf008128g0u
0.000
0.025
0.050
Branch Length
Expression Order
Of Magnitude
1.76
1.80
1.84
Proportion of
Total Expression
0.3
0.2
0.1
0.0
Silk Gland w/ Majority Expression
(Grey=Not 2−Fold Increased in Silk)
Broad
Is Dupliction Node?
N
Leaf

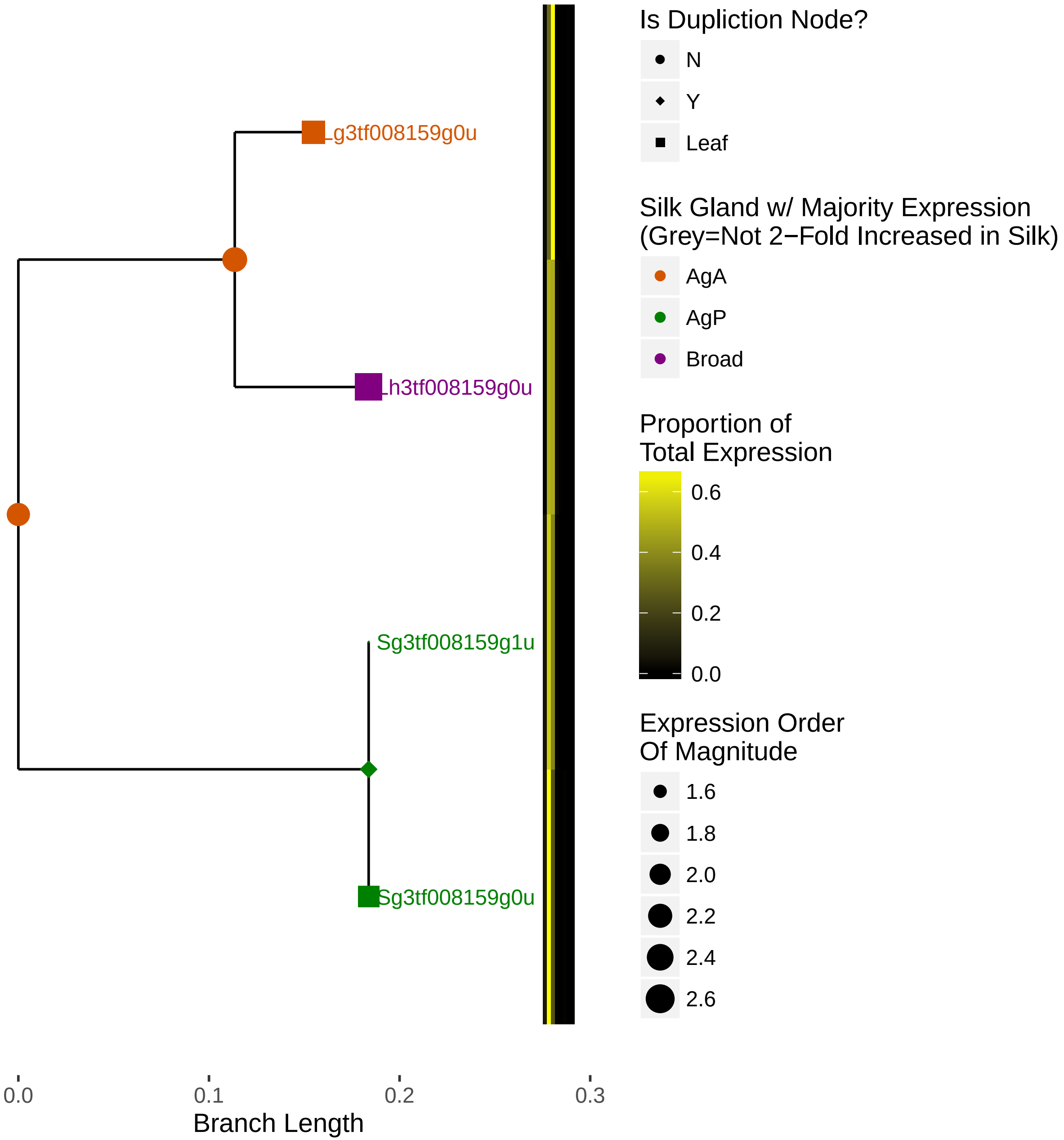

Lg3tf008159g0u
Lh3tf008159g0u
Sg3tf008159g1u
Sg3tf008159g0u
0.0
0.1
0.2
0.3
Branch Length
Is Dupliction Node?
N
Y
Leaf
Silk Gland w/ Majority Expression
(Grey=Not 2−Fold Increased in Silk)
AgA
AgP
Broad
Proportion of
Total Expression
0.6
0.4
0.2
0.0
Expression Order
Of Magnitude
1.6
1.8
2.0
2.2
2.4
2.6

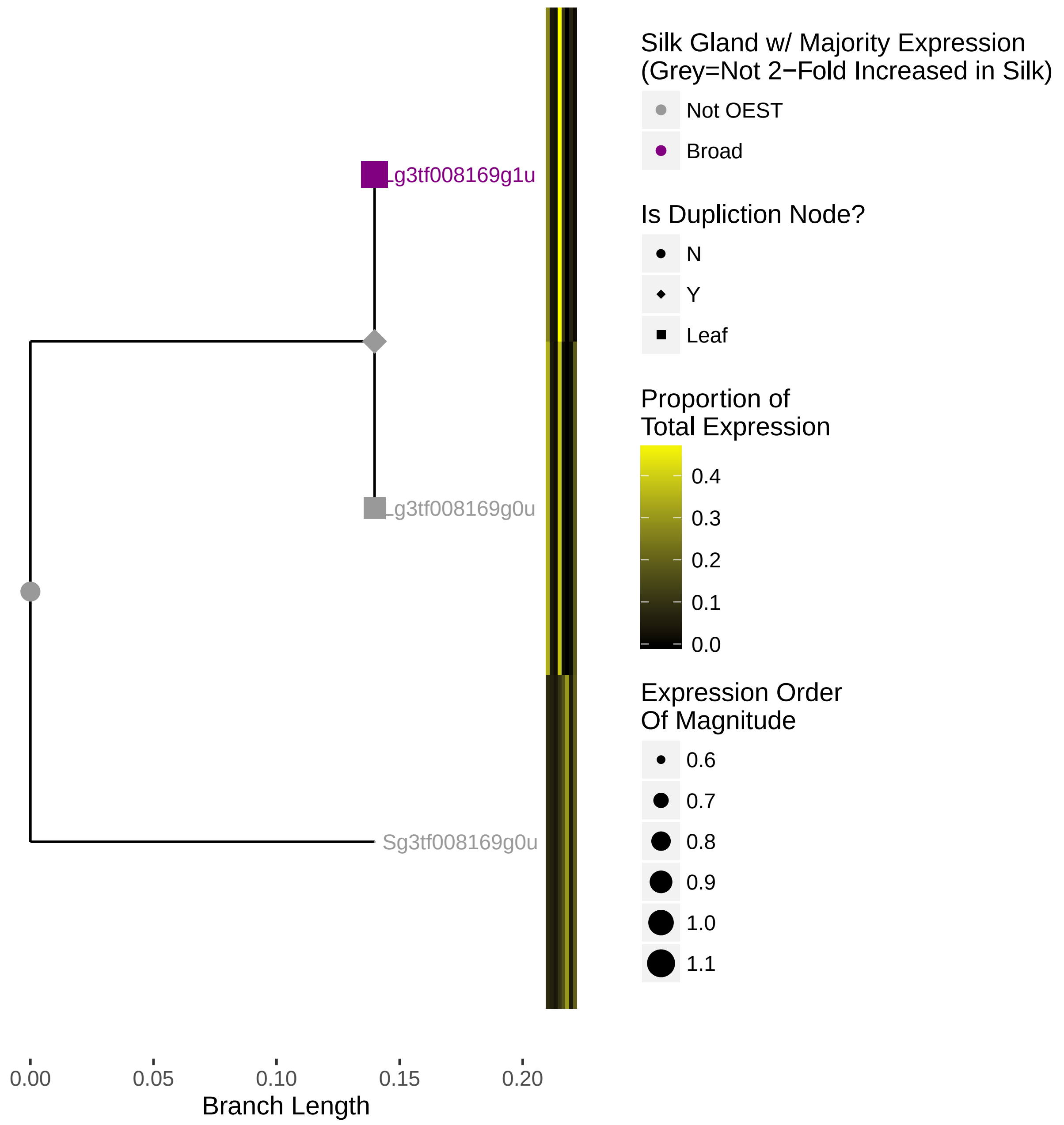

Silk Gland w/ Majority Expression
(Grey=Not 2−Fold Increased in Silk)
Not OEST
Broad
Is Dupliction Node?
N
Y
Leaf
Proportion of
Total Expression
0.4
0.3
0.2
0.1
0.0
Expression Order
Of Magnitude
0.6
0.7
0.8
0.9
1.0
1.1
Lg3tf008169g1u
Lg3tf008169g0u
Sg3tf008169g0u
0.00
0.05
0.10
0.15
0.20
Branch Length

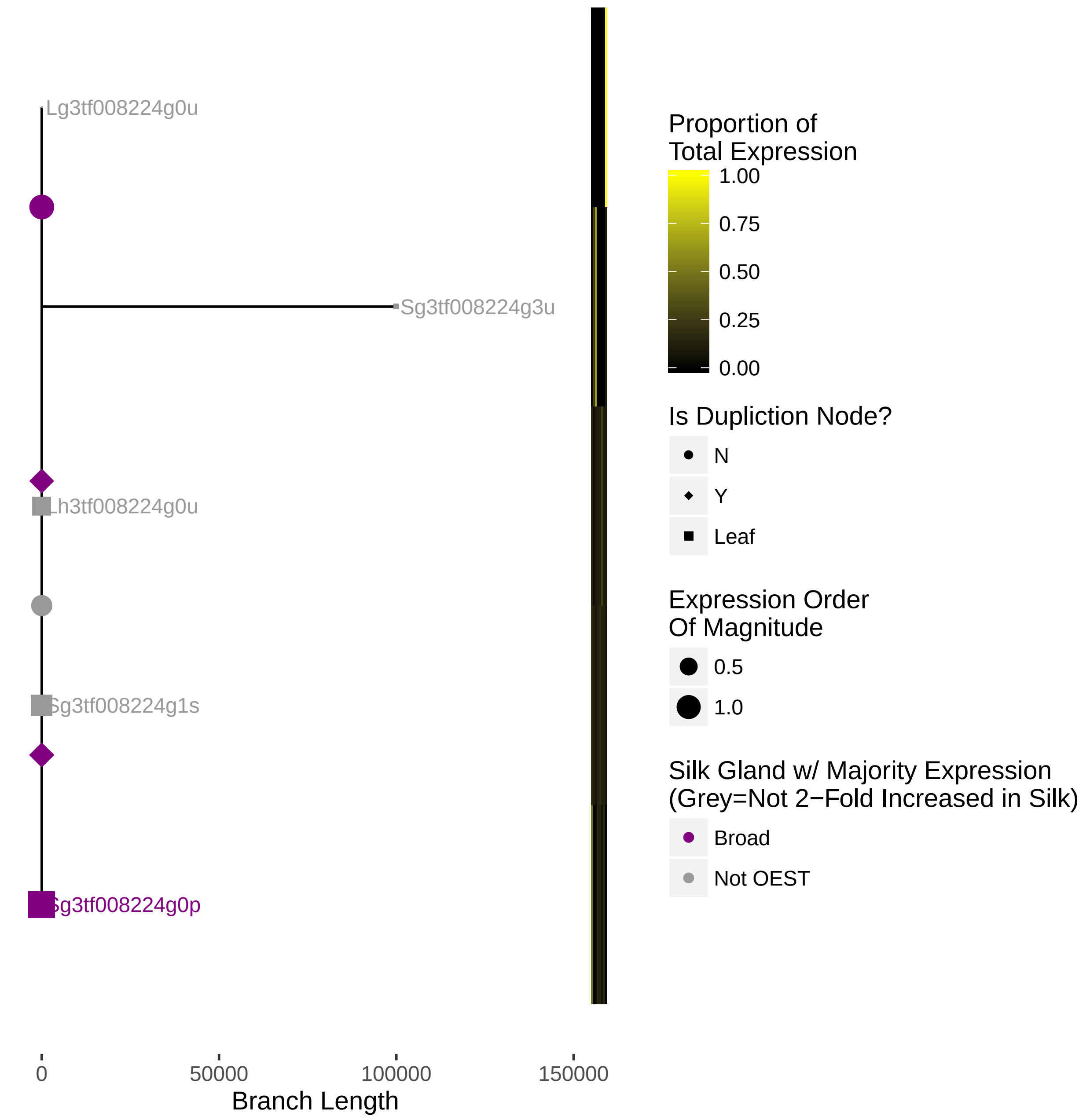

Lg3tf008224g0u
Sg3tf008224g3u
Lh3tf008224g0u
Sg3tf008224g1s
Sg3tf008224g0p
0
50000
100000
150000
Branch Length
Proportion of
Total Expression
1.00
0.75
0.50
0.25
0.00
Is Dupliction Node?
N
Y
Leaf
Expression Order
Of Magnitude
0.5
1.0
Silk Gland w/ Majority Expression
(Grey=Not 2−Fold Increased in Silk)
Broad
Not OEST

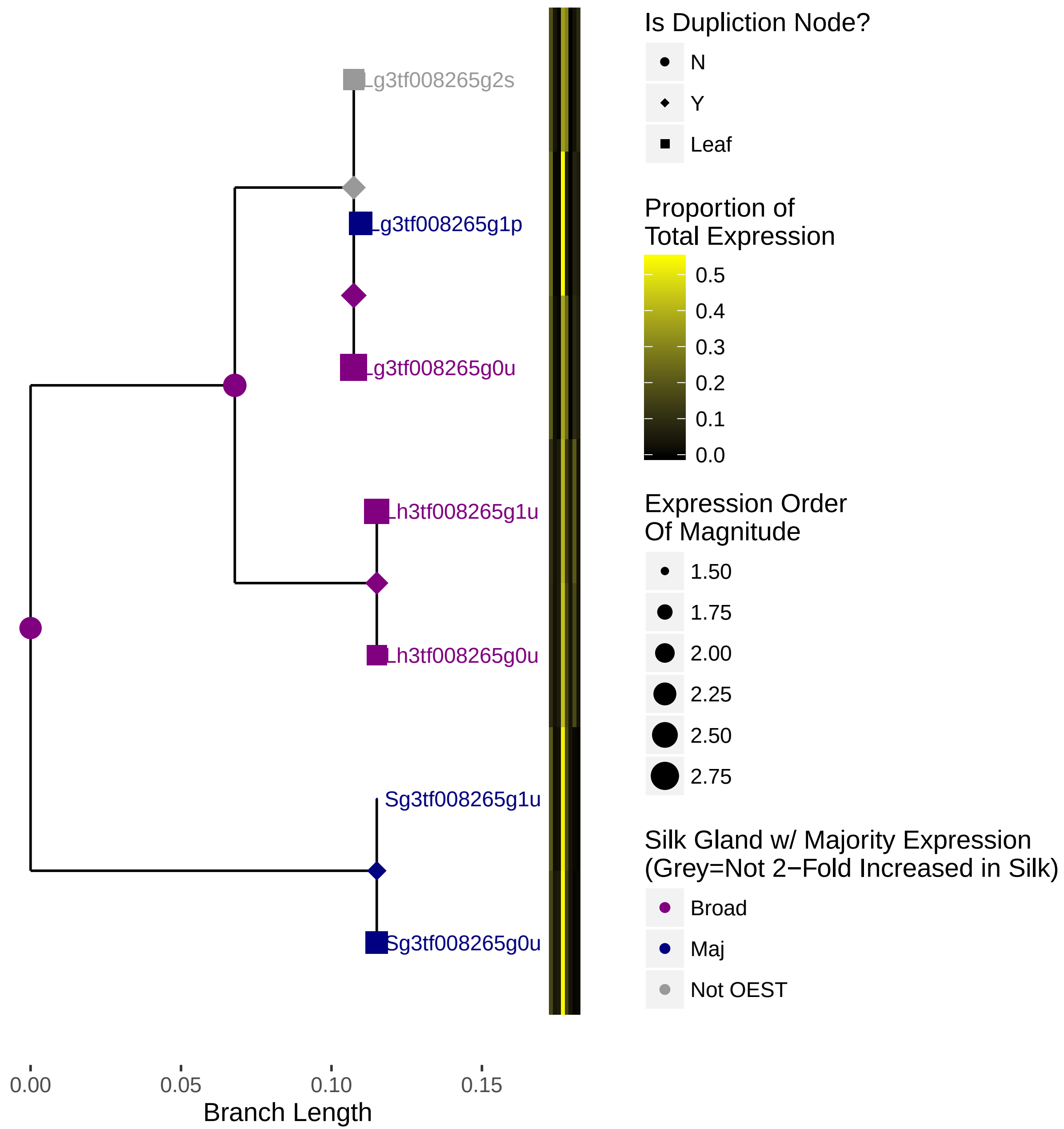
Lg3tf008265g2s
Lg3tf008265g1p
Lg3tf008265g0u
Lh3tf008265g1u
Lh3tf008265g0u
Sg3tf008265g1u
Sg3tf008265g0u
0.00
0.05
0.10
0.15
Branch Length
Is Dupliction Node?
N
Y
Leaf
Proportion of
Total Expression
0.5
0.4
0.3
0.2
0.1
0.0
Expression Order
Of Magnitude
1.50
1.75
2.00
2.25
2.50
2.75
Silk Gland w/ Majority Expression
(Grey=Not 2−Fold Increased in Silk)
Broad
Maj
Not OEST

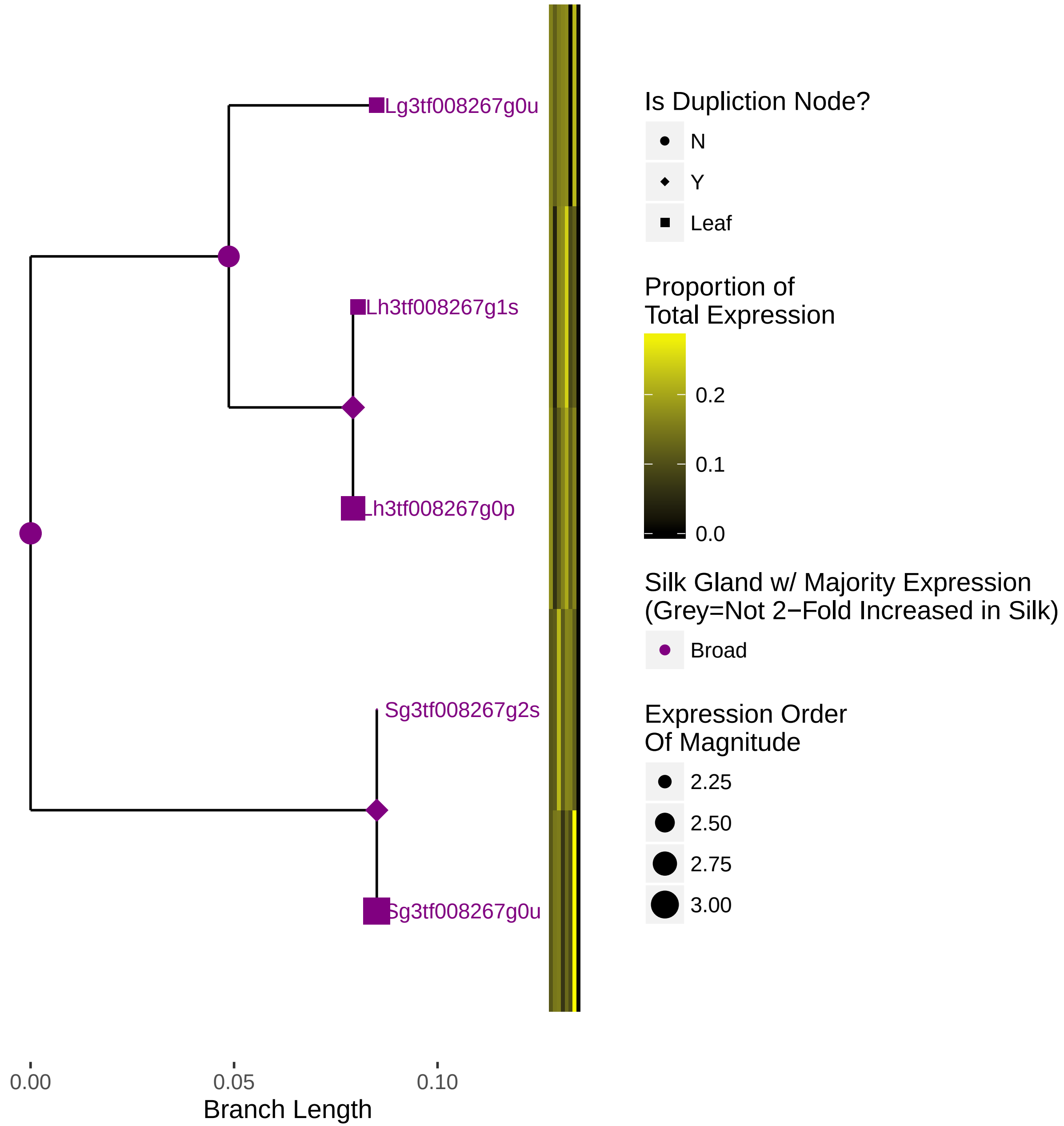

Lg3tf008267g0u
Lh3tf008267g1s
Lh3tf008267g0p
Sg3tf008267g2s
Sg3tf008267g0u
Is Dupliction Node?
N
Y
Leaf
Proportion of
Total Expression
0.2
0.1
0.0
Silk Gland w/ Majority Expression
(Grey=Not 2−Fold Increased in Silk)
Broad
Expression Order
Of Magnitude
2.25
2.50
2.75
3.00
0.00
0.05
0.10
Branch Length

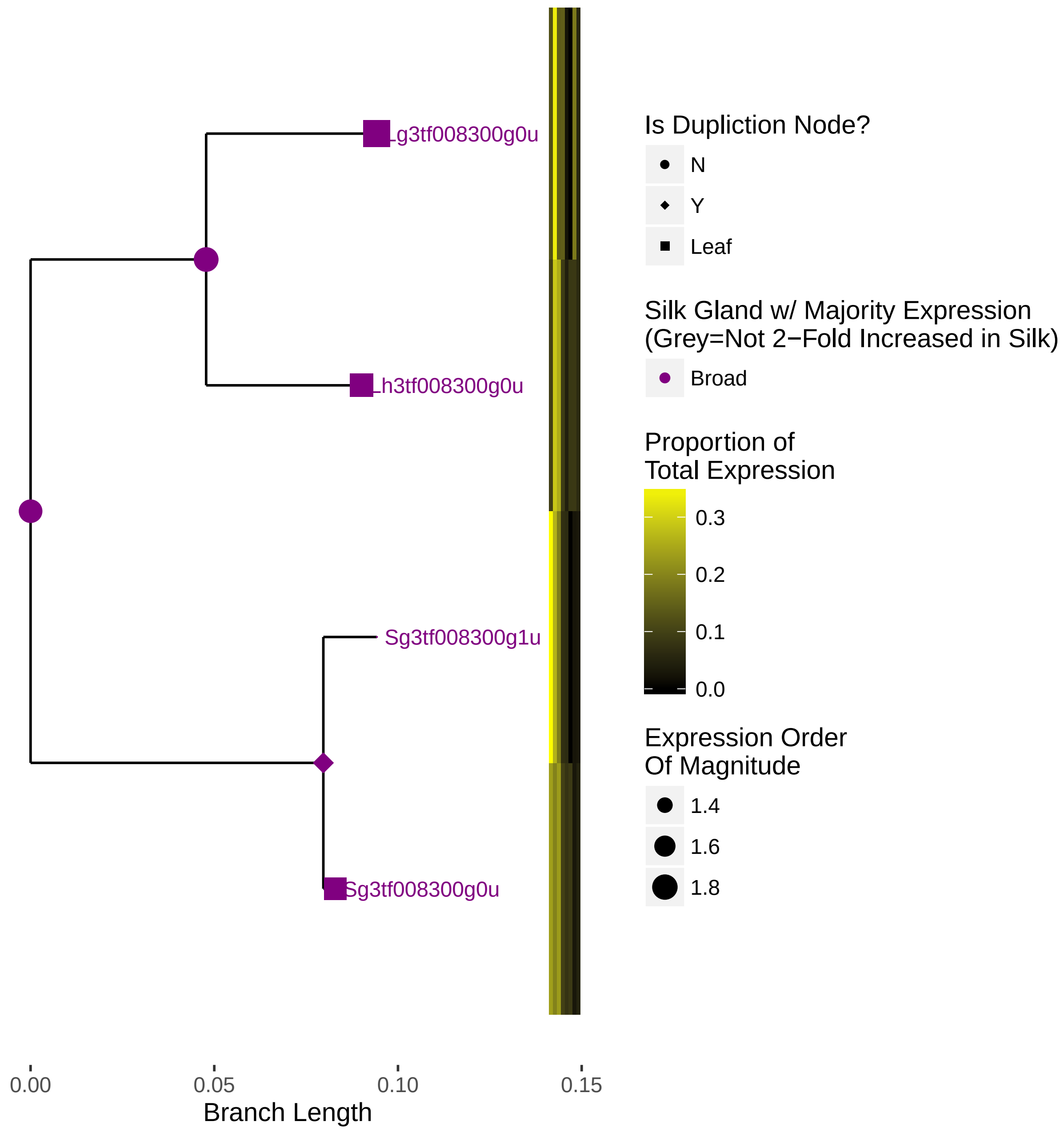

Lg3tf008300g0u
Lh3tf008300g0u
Sg3tf008300g1u
Sg3tf008300g0u
0.00
0.05
0.10
0.15
Branch Length
Is Dupliction Node?
N
Y
Leaf
Silk Gland w/ Majority Expression
(Grey=Not 2−Fold Increased in Silk)
Broad
Proportion of
Total Expression
0.3
0.2
0.1
0.0
Expression Order
Of Magnitude
1.4
1.6
1.8

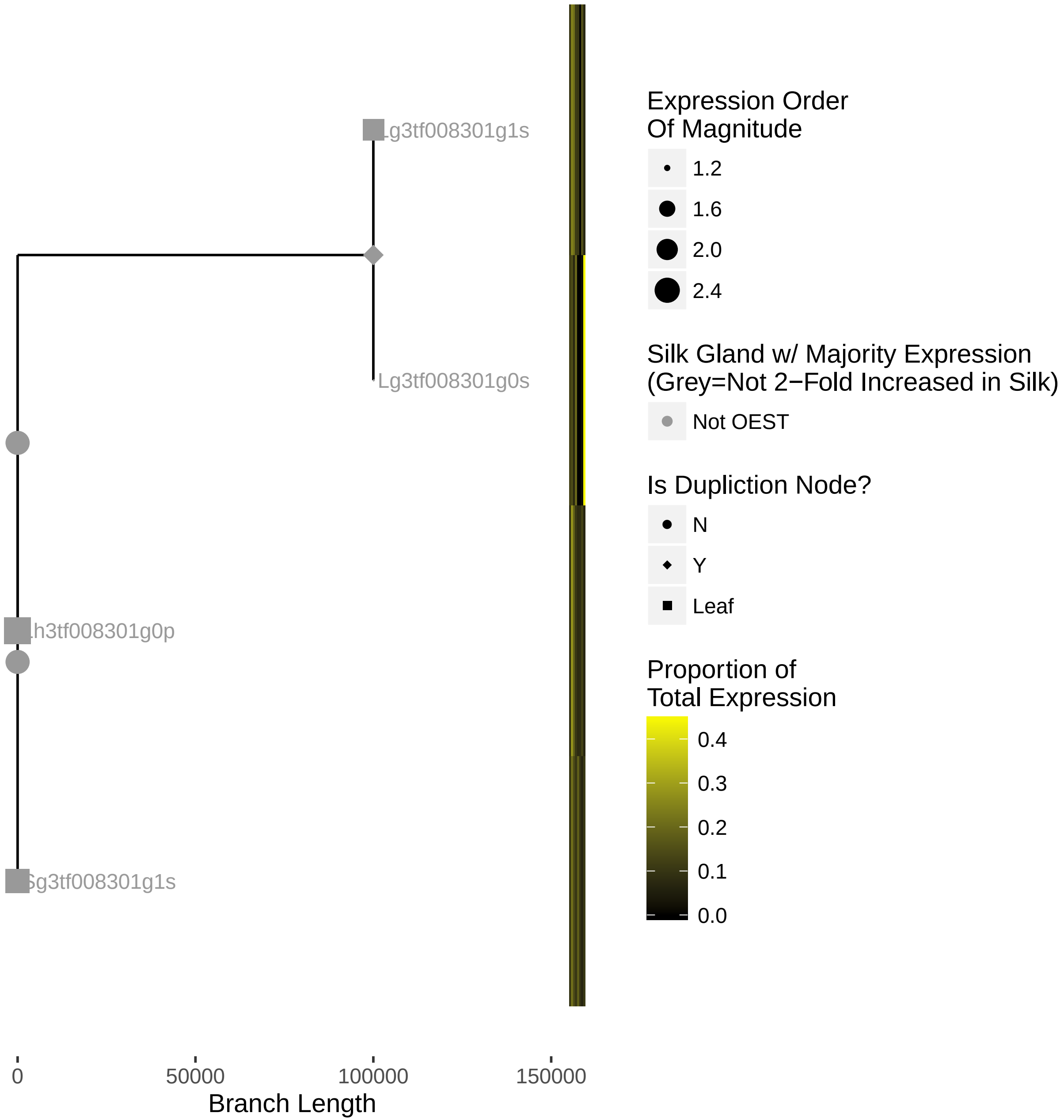

Lg3tf008301g1s
Lg3tf008301g0s
h3tf008301g0p
g3tf008301g1s
0
50000
100000
150000
Branch Length
Expression Order
Of Magnitude
1.2
1.6
2.0
2.4
Silk Gland w/ Majority Expression
(Grey=Not 2−Fold Increased in Silk)
Not OEST
Is Dupliction Node?
N
Y
Leaf
Proportion of
Total Expression
0.4
0.3
0.2
0.1
0.0

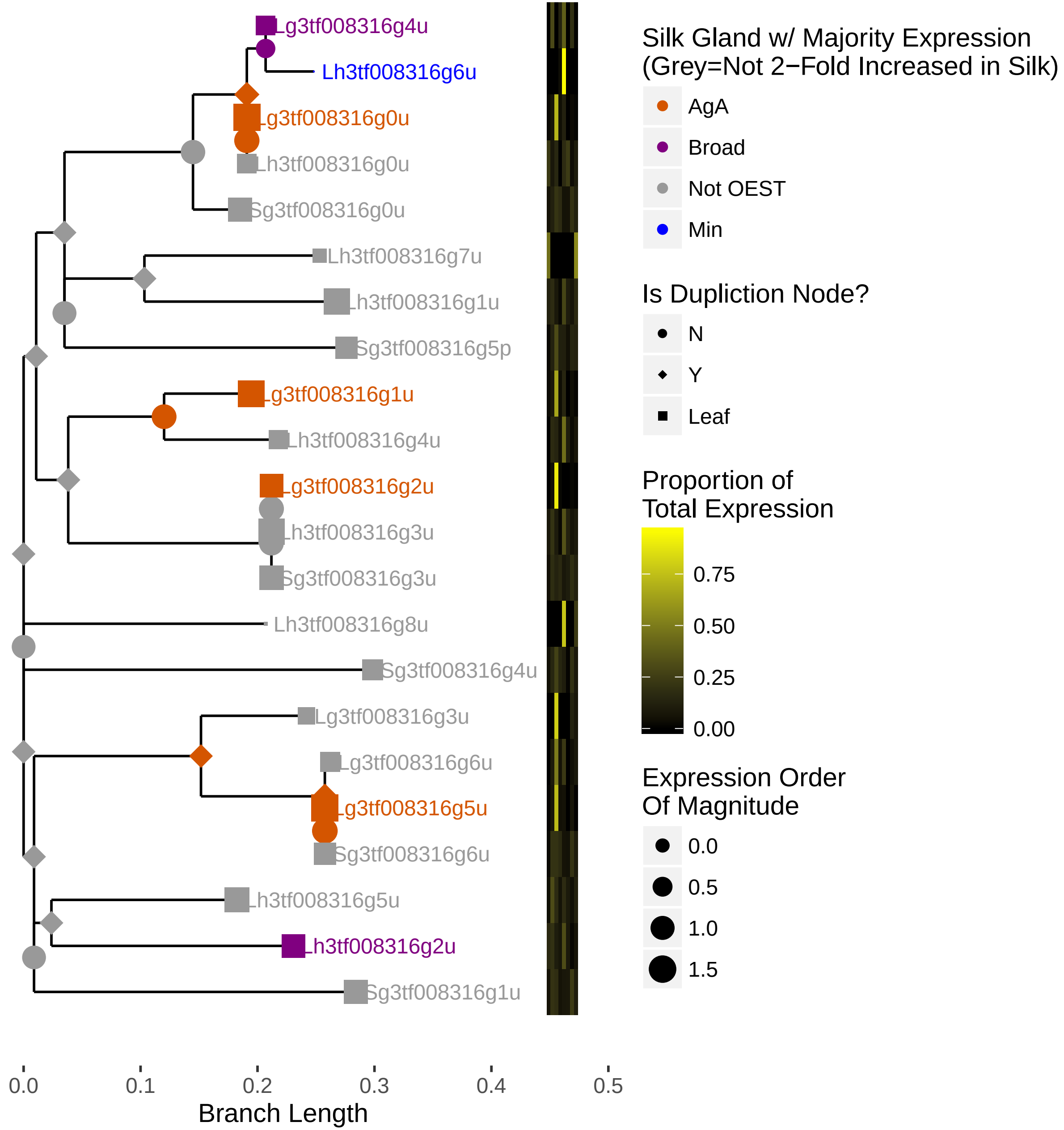

Lg3tf008316g4u
Lh3tf008316g6u
Lg3tf008316g0u
Lh3tf008316g0u
Sg3tf008316g0u
Lh3tf008316g7u
Lh3tf008316g1u
Sg3tf008316g5p
Lg3tf008316g1u
Lh3tf008316g4u
Lg3tf008316g2u
Lh3tf008316g3u
Sg3tf008316g3u
Lh3tf008316g8u
Sg3tf008316g4u
Lg3tf008316g3u
Lg3tf008316g6u
Lg3tf008316g5u
Sg3tf008316g6u
Lh3tf008316g5u
Lh3tf008316g2u
Sg3tf008316g1u
0.0
0.1
0.2
0.3
0.4
0.5
Branch Length
Silk Gland w/ Majority Expression
(Grey=Not 2−Fold Increased in Silk)
AgA
Broad
Not OEST
Min
Is Dupliction Node?
N
Y
Leaf
Proportion of
Total Expression
0.75
0.50
0.25
0.00
Expression Order
Of Magnitude
0.0
0.5
1.0
1.5

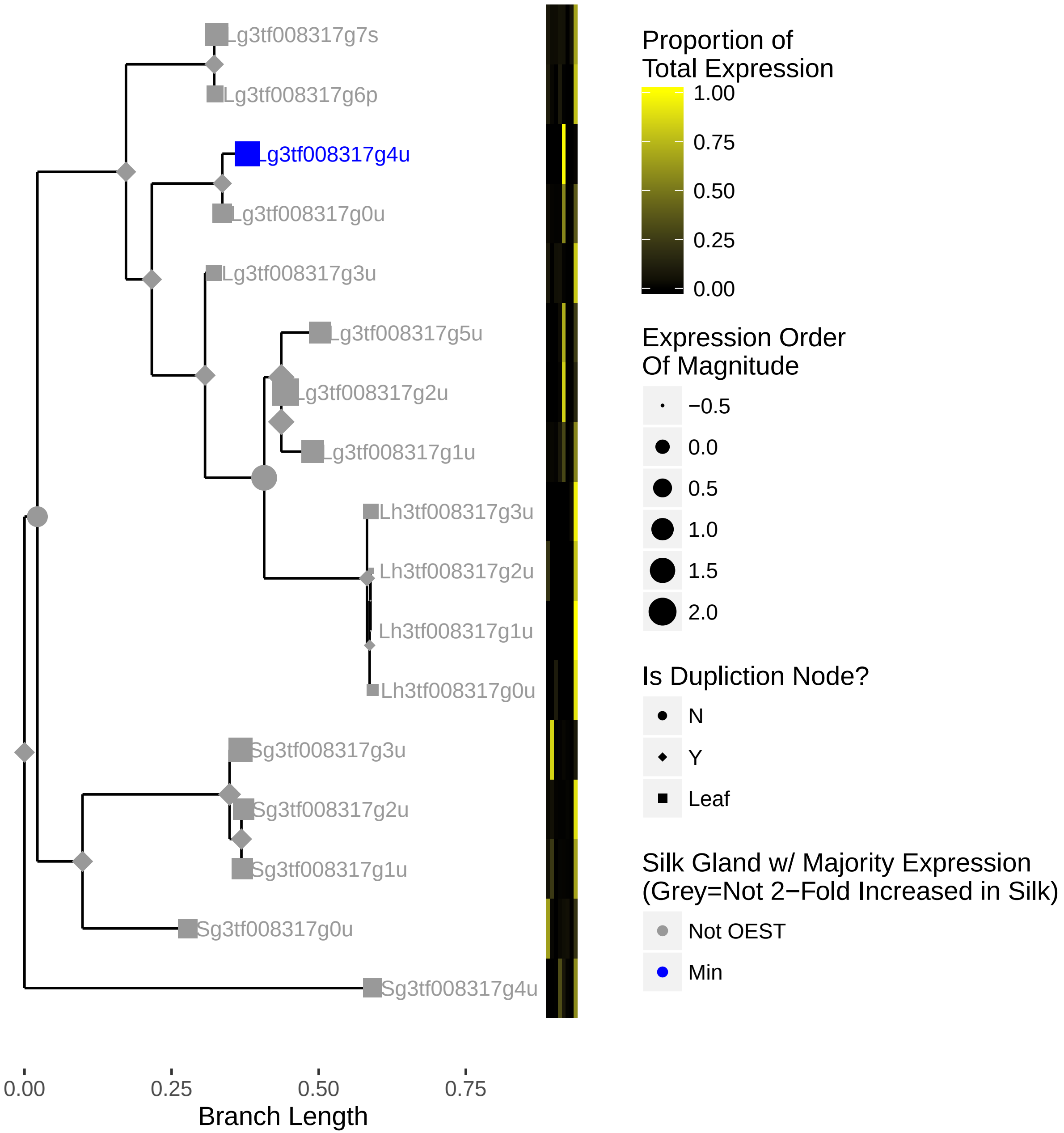

Lg3tf008317g7s
Lg3tf008317g6p
Lg3tf008317g4u
Lg3tf008317g0u
Lg3tf008317g3u
Lg3tf008317g5u
Lg3tf008317g2u
Lg3tf008317g1u
Lh3tf008317g3u
Lh3tf008317g2u
Lh3tf008317g1u
Lh3tf008317g0u
Sg3tf008317g3u
Sg3tf008317g2u
Sg3tf008317g1u
Sg3tf008317g0u
Sg3tf008317g4u
0.00
0.25
0.50
0.75
Branch Length
Proportion of Total Expression
1.00
0.75
0.50
0.25
0.00
Expression Order Of Magnitude
−0.5
0.0
0.5
1.0
1.5
2.0
Is Dupliction Node?
N
Y
Leaf
Silk Gland w/ Majority Expression (Grey=Not 2−Fold Increased in Silk)
Not OEST
Min

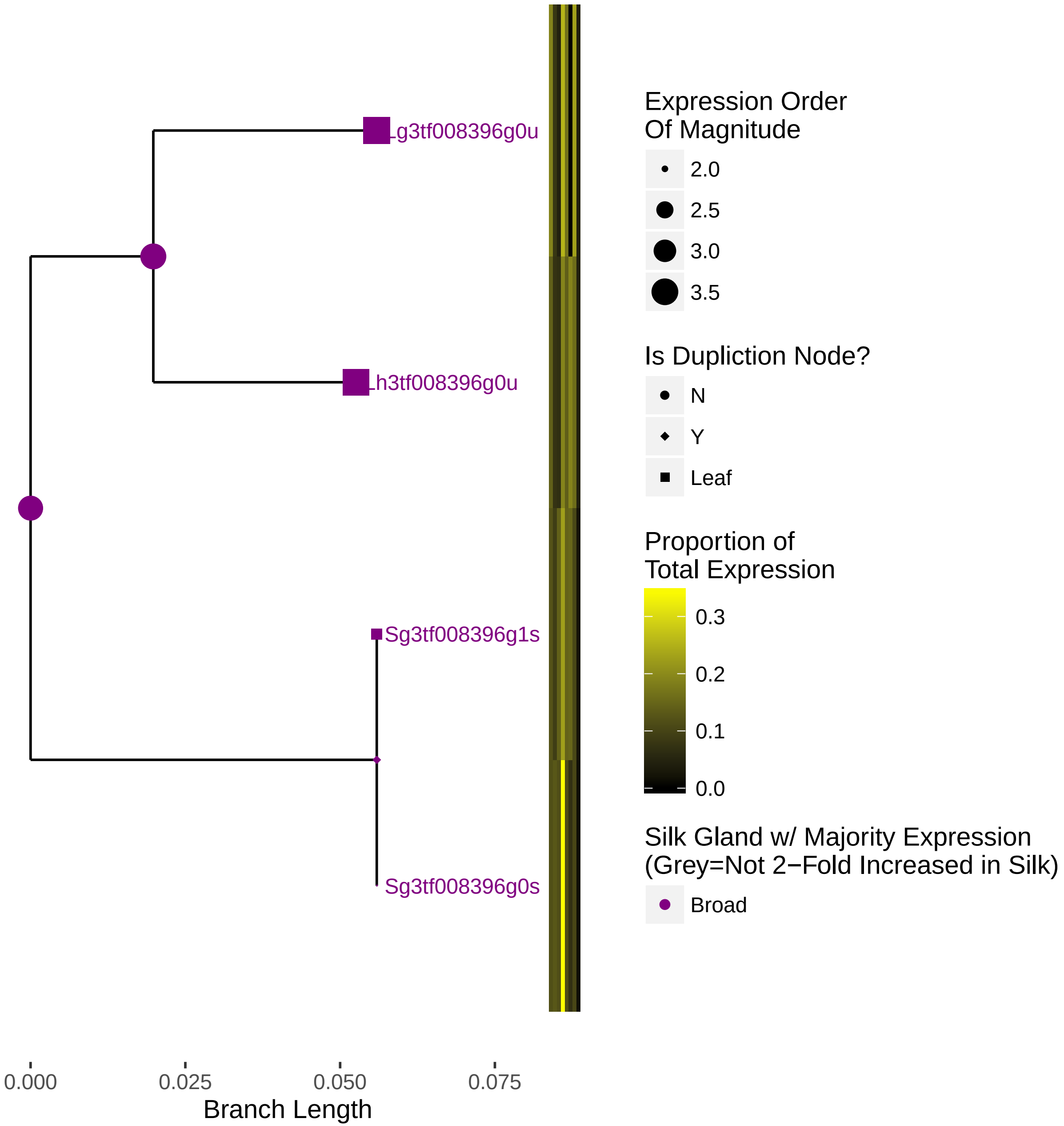

Lg3tf008396g0u
Lh3tf008396g0u
Sg3tf008396g1s
Sg3tf008396g0s
0.000
0.025
0.050
0.075
Branch Length
Expression Order
Of Magnitude
2.0
2.5
3.0
3.5
Is Dupliction Node?
N
Y
Leaf
Proportion of
Total Expression
0.3
0.2
0.1
0.0
Silk Gland w/ Majority Expression
(Grey=Not 2−Fold Increased in Silk)
Broad

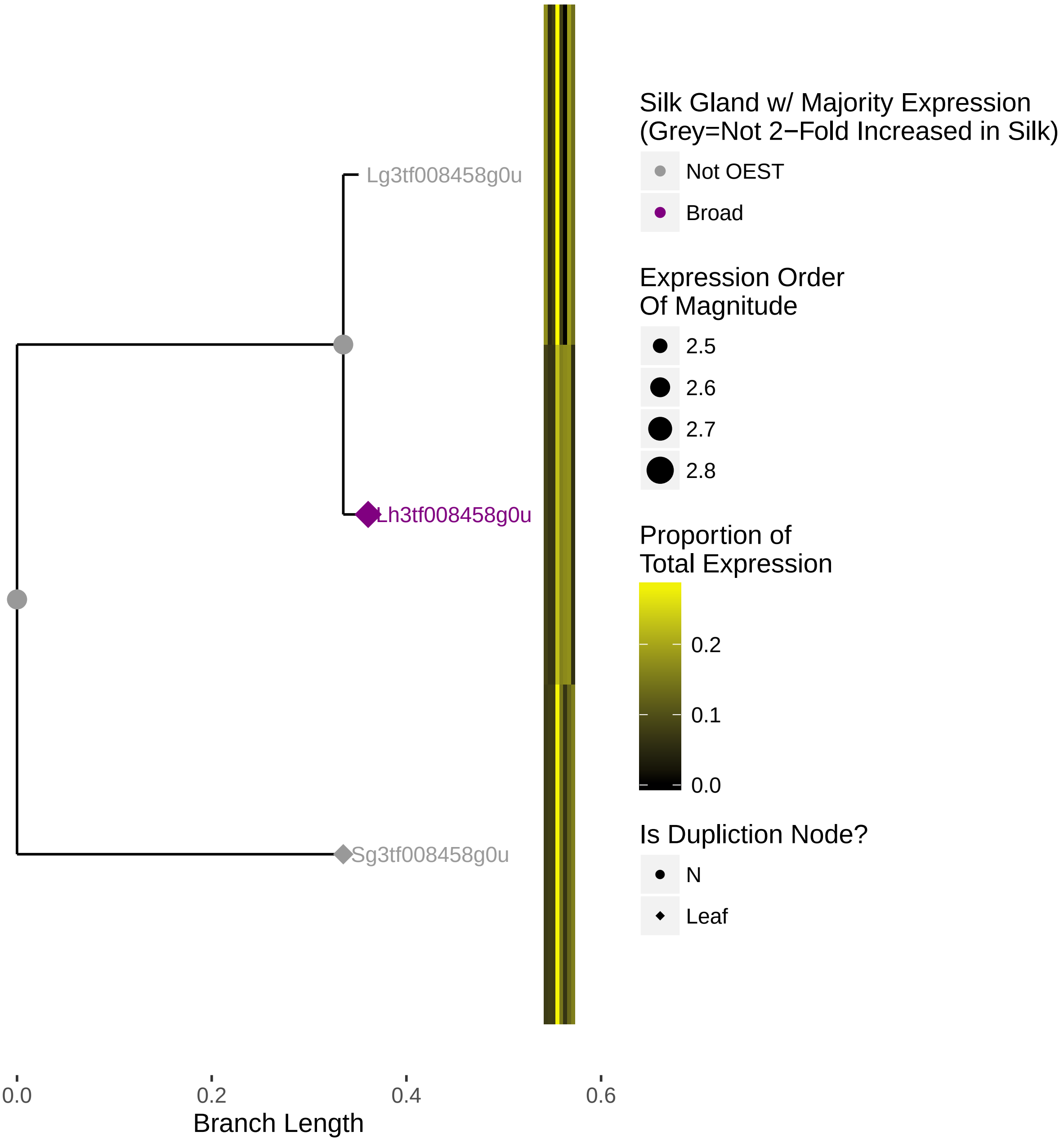
Lg3tf008458g0u
Lh3tf008458g0u
Sg3tf008458g0u
Silk Gland w/ Majority Expression
(Grey=Not 2−Fold Increased in Silk)
Not OEST
Broad
Expression Order
Of Magnitude
2.5
2.6
2.7
2.8
Proportion of
Total Expression
0.2
0.1
0.0
Is Dupliction Node?
N
Leaf
0.0
0.2
0.4
0.6
Branch Length

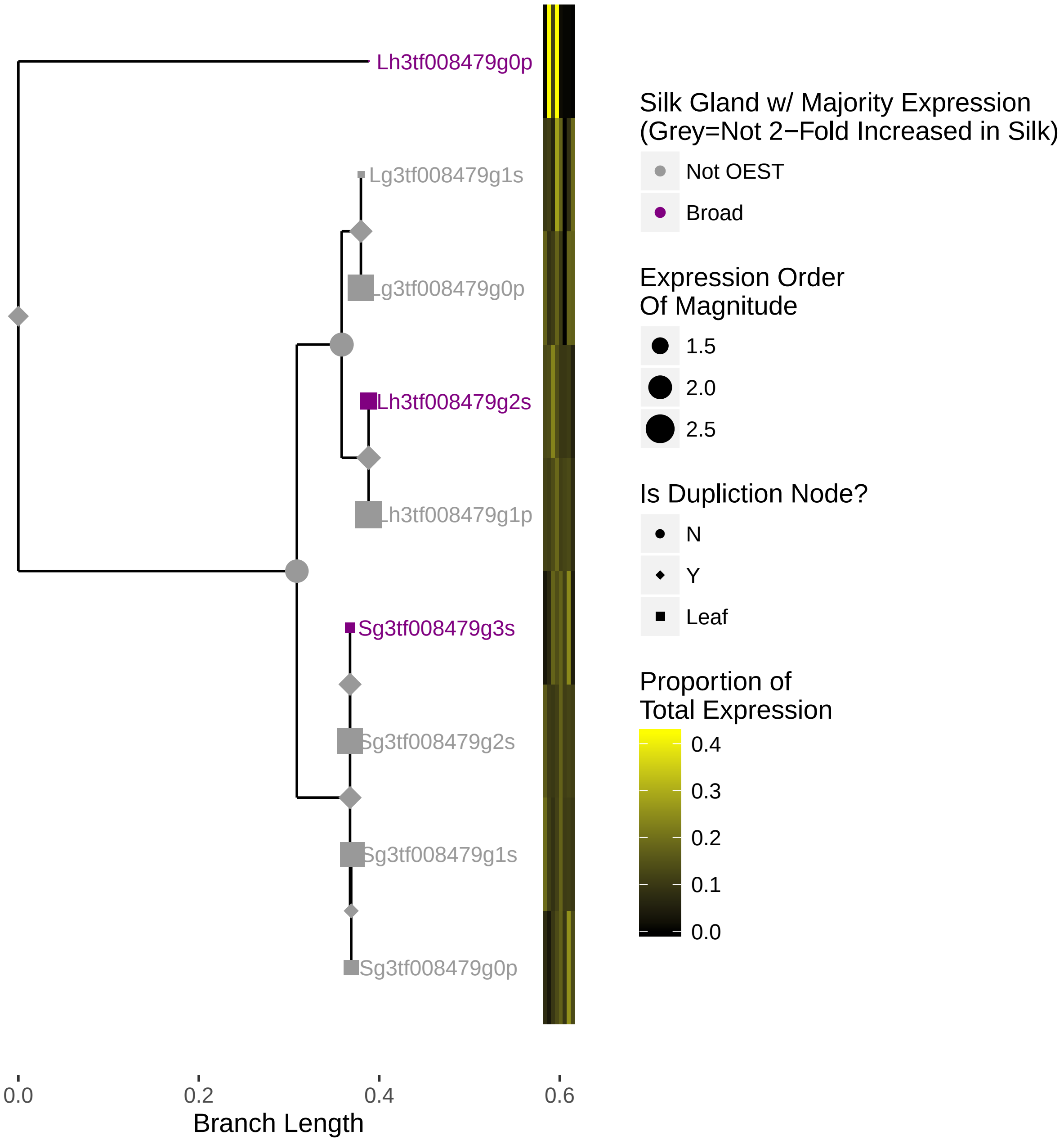

Lh3tf008479g0p
Lg3tf008479g1s
Lg3tf008479g0p
Lh3tf008479g2s
Lh3tf008479g1p
Sg3tf008479g3s
Sg3tf008479g2s
Sg3tf008479g1s
Sg3tf008479g0p
0.0
0.2
0.4
0.6
Branch Length
Silk Gland w/ Majority Expression
(Grey=Not 2−Fold Increased in Silk)
Not OEST
Broad
Expression Order
Of Magnitude
1.5
2.0
2.5
Is Dupliction Node?
N
Y
Leaf
Proportion of
Total Expression
0.4
0.3
0.2
0.1
0.0

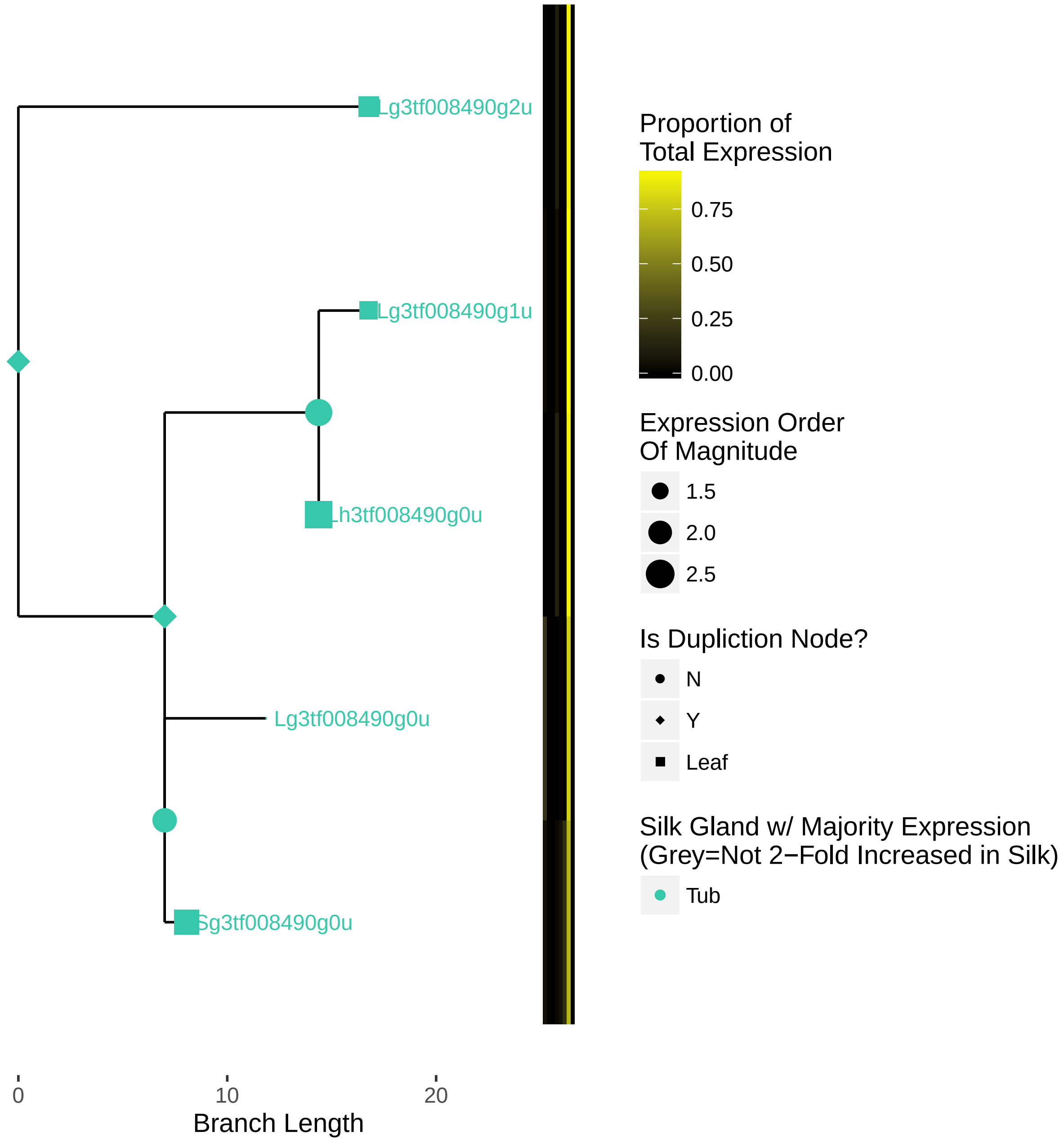

Lg3tf008490g2u
Lg3tf008490g1u
Lh3tf008490g0u
Lg3tf008490g0u
Sg3tf008490g0u
0
10
20
Branch Length
Proportion of
Total Expression
0.75
0.50
0.25
0.00
Expression Order
Of Magnitude
1.5
2.0
2.5
Is Dupliction Node?
N
Y
Leaf
Silk Gland w/ Majority Expression
(Grey=Not 2−Fold Increased in Silk)
Tub

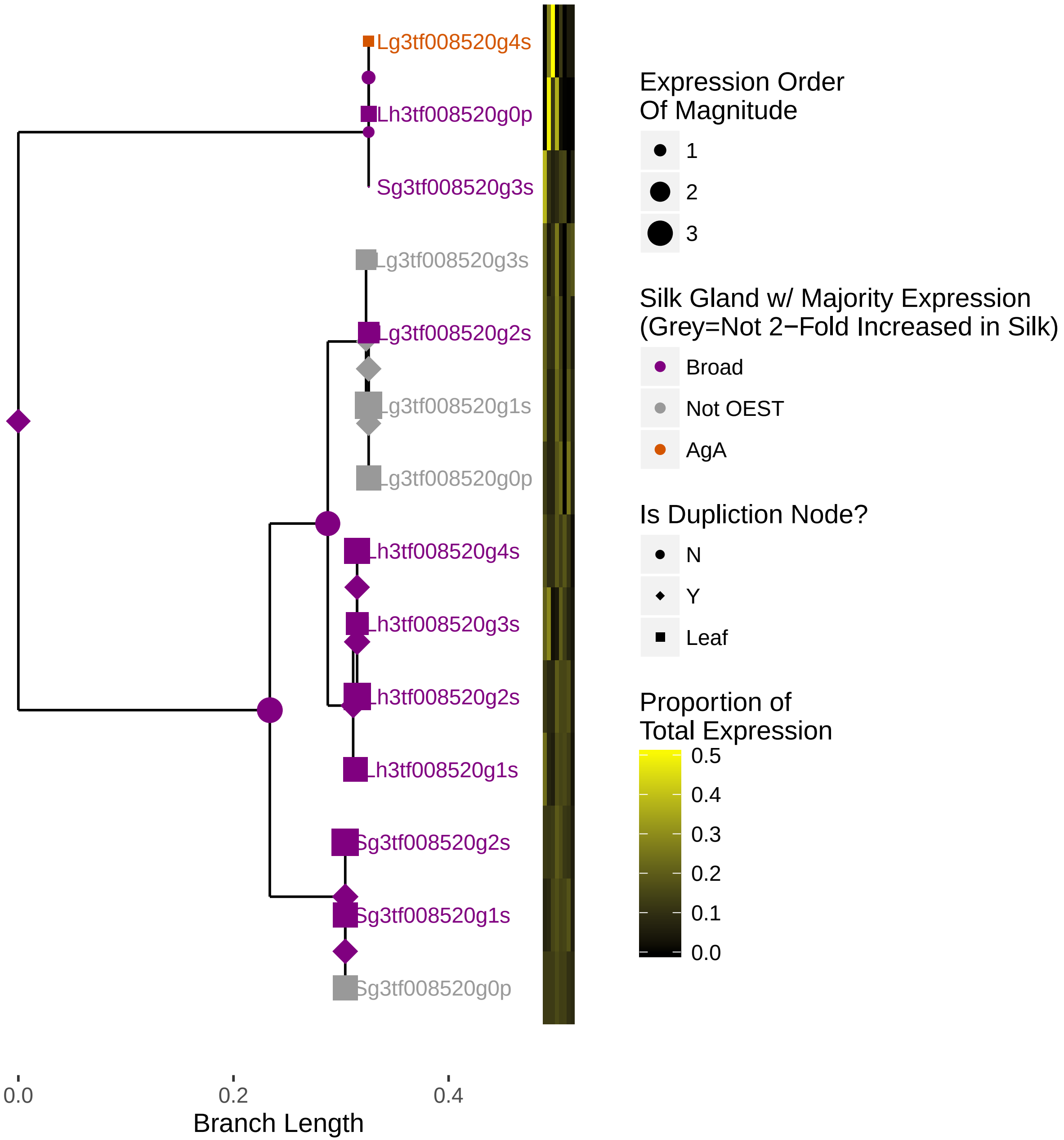

Lg3tf008520g4s
Lh3tf008520g0p
Sg3tf008520g3s
Lg3tf008520g3s
Lg3tf008520g2s
Lg3tf008520g1s
Lg3tf008520g0p
Lh3tf008520g4s
Lh3tf008520g3s
Lh3tf008520g2s
Lh3tf008520g1s
Sg3tf008520g2s
Sg3tf008520g1s
Sg3tf008520g0p
0.0
0.2
0.4
Branch Length
Expression Order Of Magnitude
1
2
3
Silk Gland w/ Majority Expression (Grey=Not 2−Fold Increased in Silk)
Broad
Not OEST
AgA
Is Dupliction Node?
N
Y
Leaf
Proportion of Total Expression
0.5
0.4
0.3
0.2
0.1
0.0

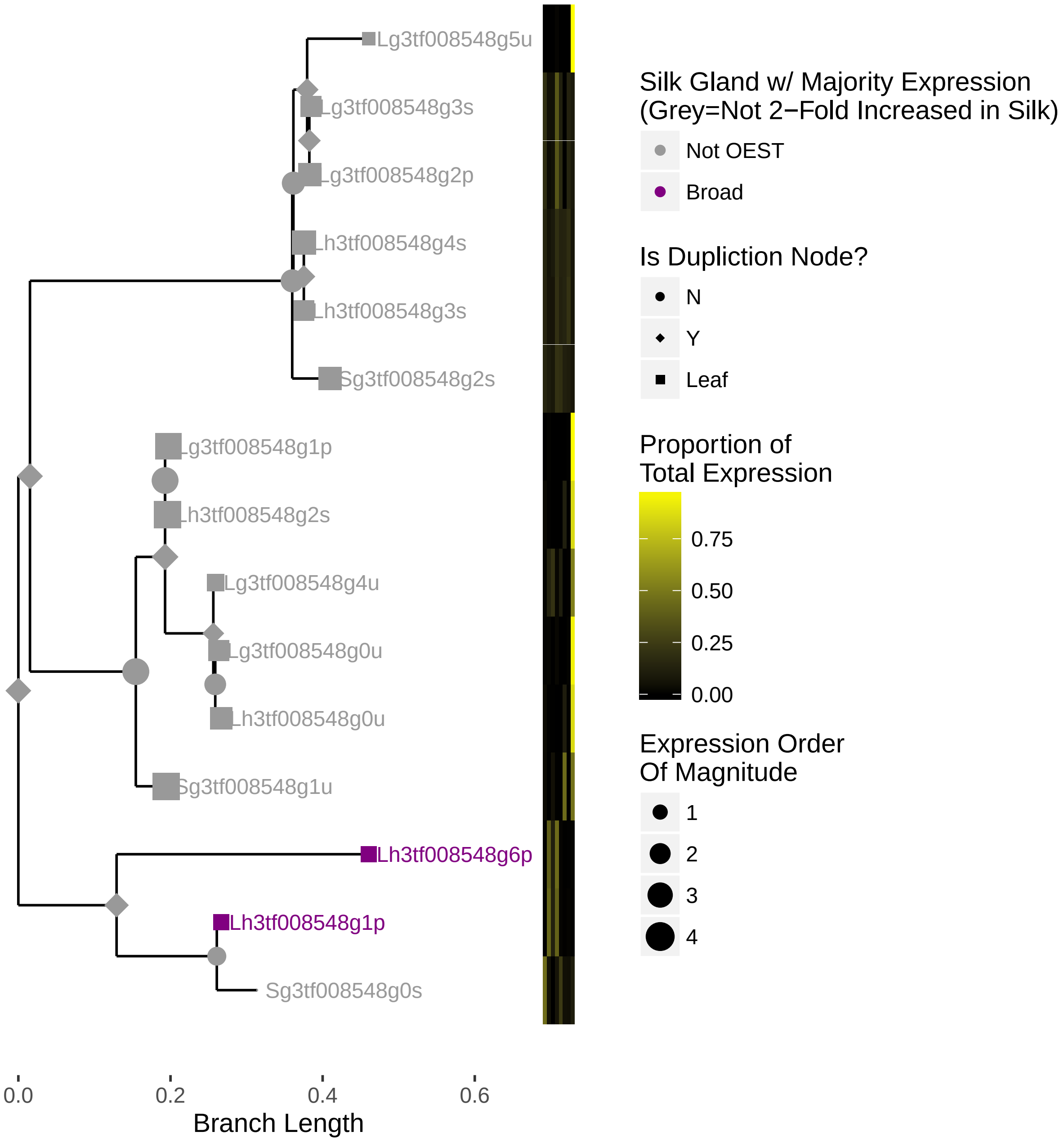

Lg3tf008548g5u
Lg3tf008548g3s
Lg3tf008548g2p
Lh3tf008548g4s
Lh3tf008548g3s
Sg3tf008548g2s
Lg3tf008548g1p
Lh3tf008548g2s
Lg3tf008548g4u
Lg3tf008548g0u
Lh3tf008548g0u
Sg3tf008548g1u
Lh3tf008548g6p
Lh3tf008548g1p
Sg3tf008548g0s
0.0
0.2
0.4
0.6
Branch Length
Silk Gland w/ Majority Expression
(Grey=Not 2−Fold Increased in Silk)
Not OEST
Broad
Is Dupliction Node?
N
Y
Leaf
Proportion of
Total Expression
0.75
0.50
0.25
0.00
Expression Order
Of Magnitude
1
2
3
4

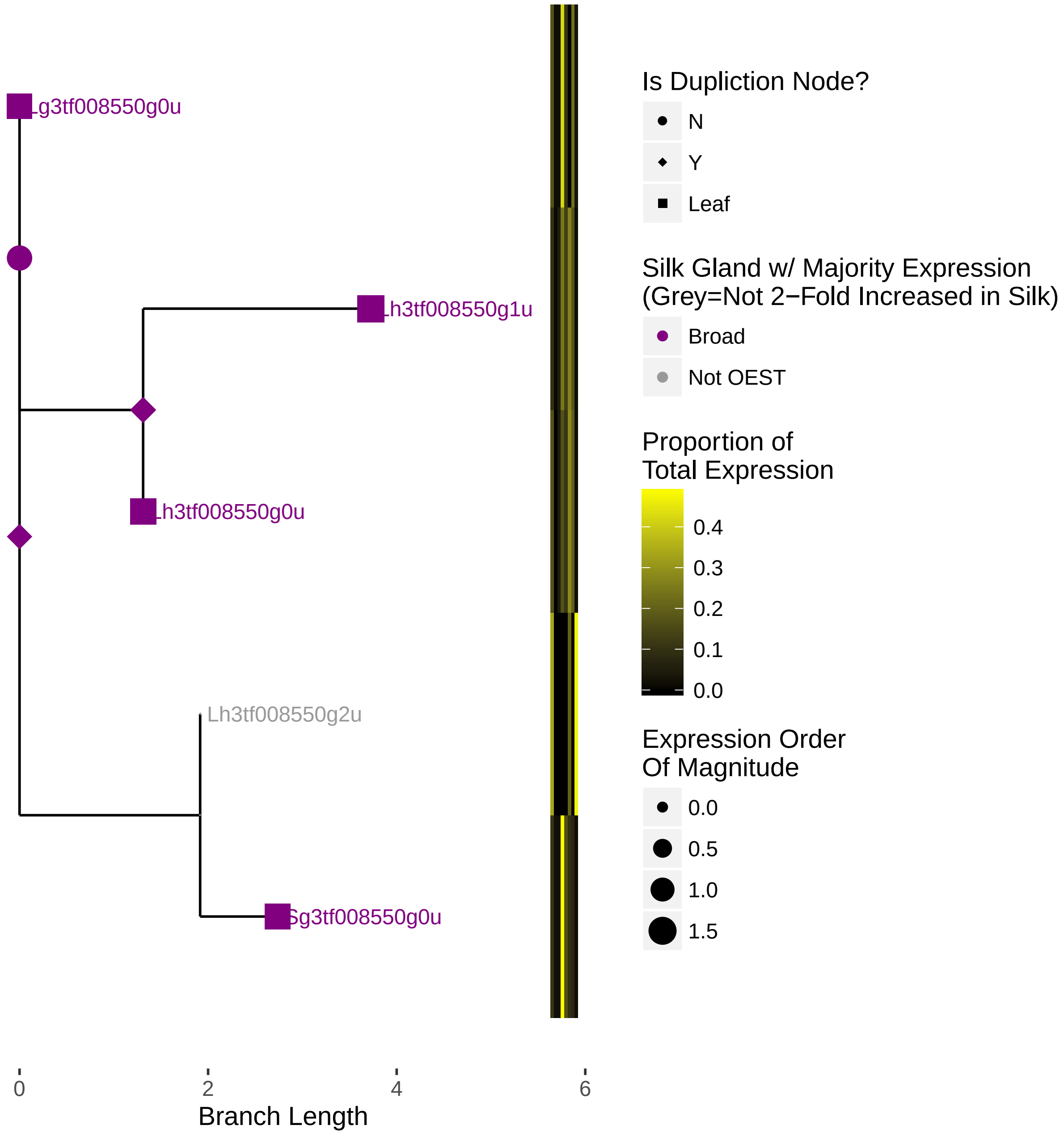

Lg3tf008550g0u
Lh3tf008550g1u
Lh3tf008550g0u
Lh3tf008550g2u
Sg3tf008550g0u
0
2
4
6
Branch Length
Is Dupliction Node?
N
Y
Leaf
Silk Gland w/ Majority Expression
(Grey=Not 2−Fold Increased in Silk)
Broad
Not OEST
Proportion of
Total Expression
0.4
0.3
0.2
0.1
0.0
Expression Order
Of Magnitude
0.0
0.5
1.0
1.5

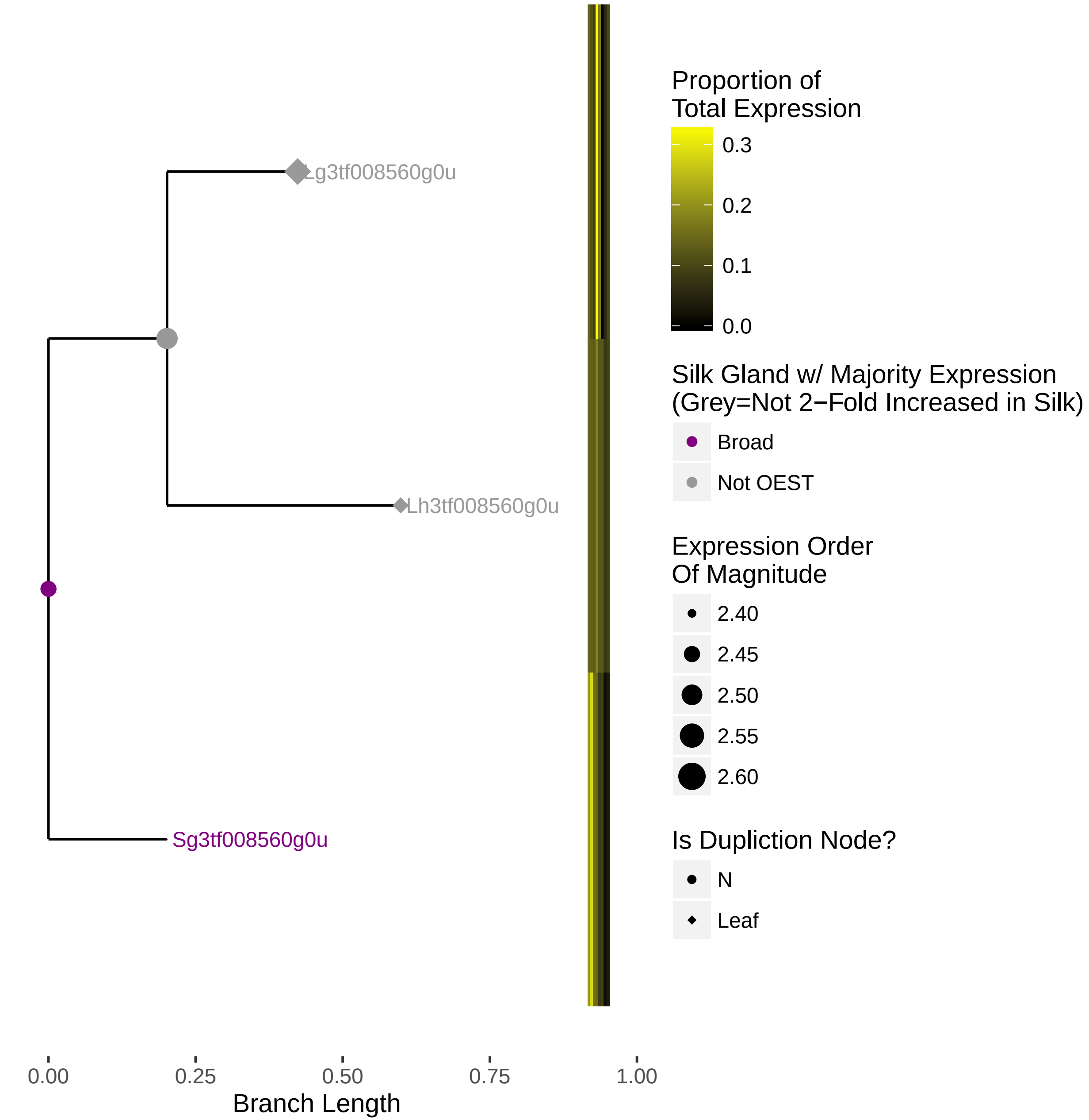
Lg3tf008560g0u
Lh3tf008560g0u
Sg3tf008560g0u
Proportion of Total Expression
0.3
0.2
0.1
0.0
Silk Gland w/ Majority Expression (Grey=Not 2−Fold Increased in Silk)
Broad
Not OEST
Expression Order Of Magnitude
2.40
2.45
2.50
2.55
2.60
Is Dupliction Node?
N
Leaf
0.00
0.25
0.50
0.75
1.00
Branch Length

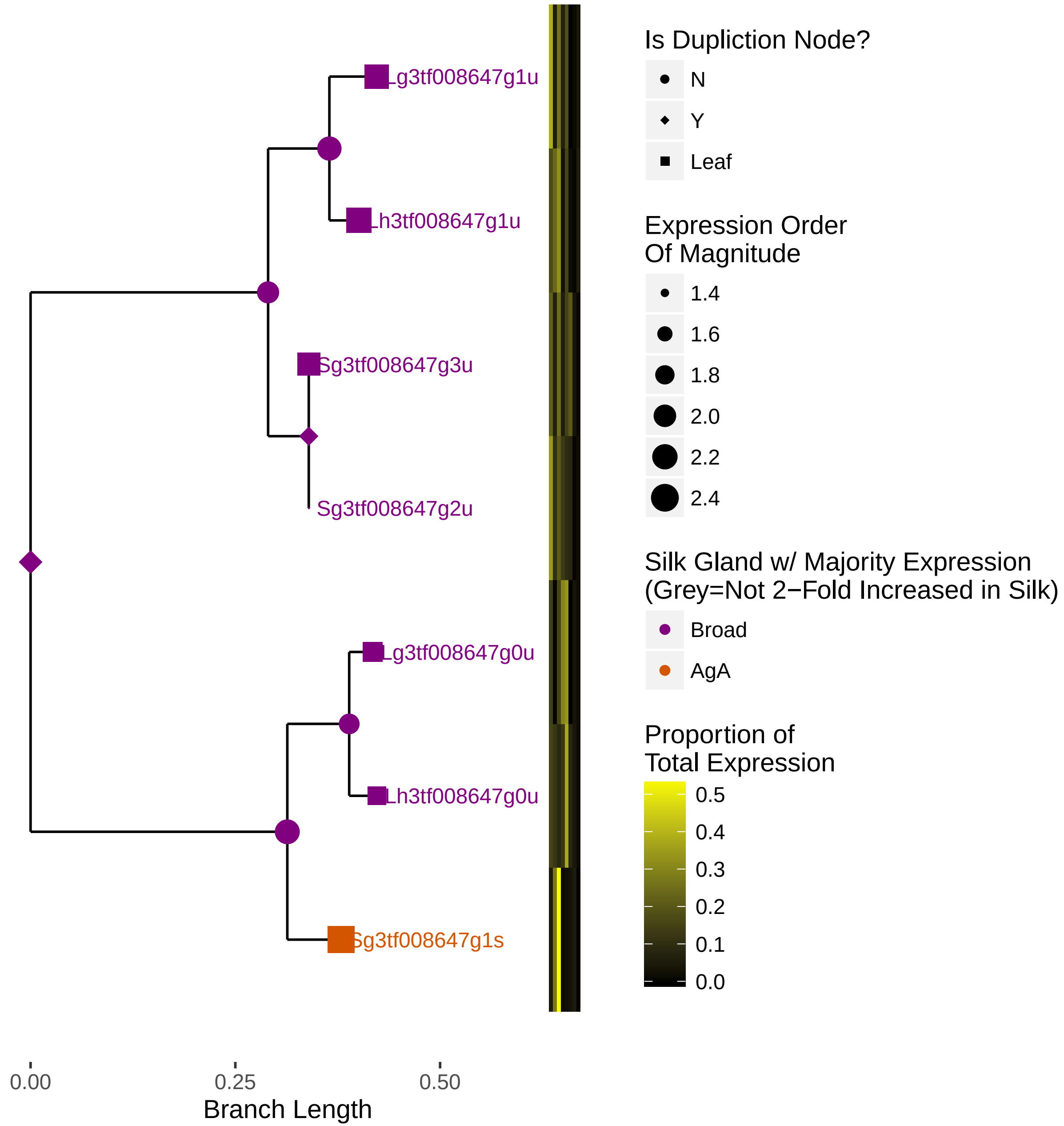

Lg3tf008647g1u
Lh3tf008647g1u
Sg3tf008647g3u
Sg3tf008647g2u
Lg3tf008647g0u
Lh3tf008647g0u
Sg3tf008647g1s
0.00
0.25
0.50
Branch Length
Is Dupliction Node?
N
Y
Leaf
Expression Order
Of Magnitude
1.4
1.6
1.8
2.0
2.2
2.4
Silk Gland w/ Majority Expression
(Grey=Not 2−Fold Increased in Silk)
Broad
AgA
Proportion of
Total Expression
0.5
0.4
0.3
0.2
0.1
0.0

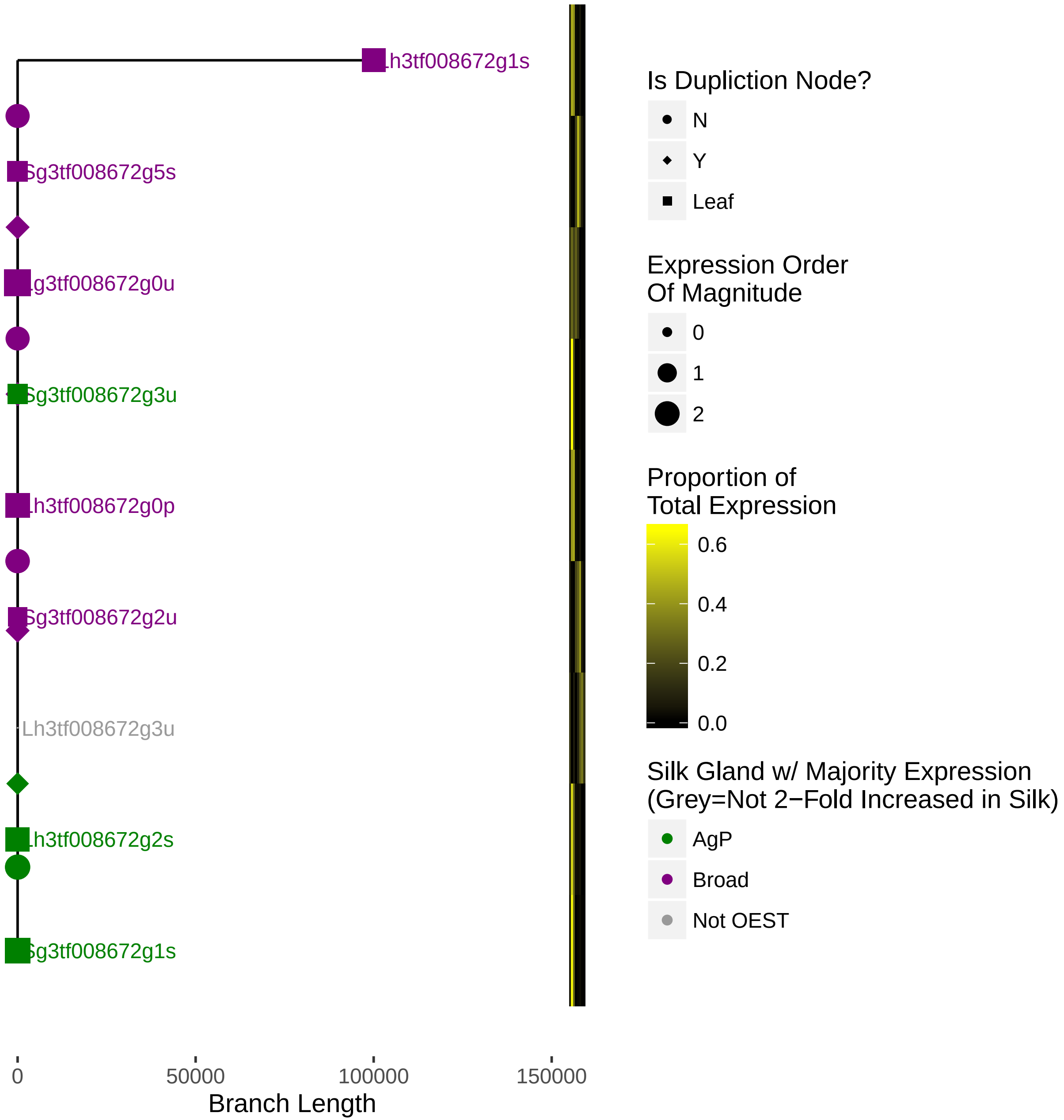

Lh3tf008672g1s
Sg3tf008672g5s
Lg3tf008672g0u
Sg3tf008672g3u
Lh3tf008672g0p
Sg3tf008672g2u
Lh3tf008672g3u
Lh3tf008672g2s
Sg3tf008672g1s
0
50000
100000
150000
Branch Length
Is Dupliction Node?
N
Y
Leaf
Expression Order Of Magnitude
0
1
2
Proportion of Total Expression
0.6
0.4
0.2
0.0
Silk Gland w/ Majority Expression (Grey=Not 2−Fold Increased in Silk)
AgP
Broad
Not OEST

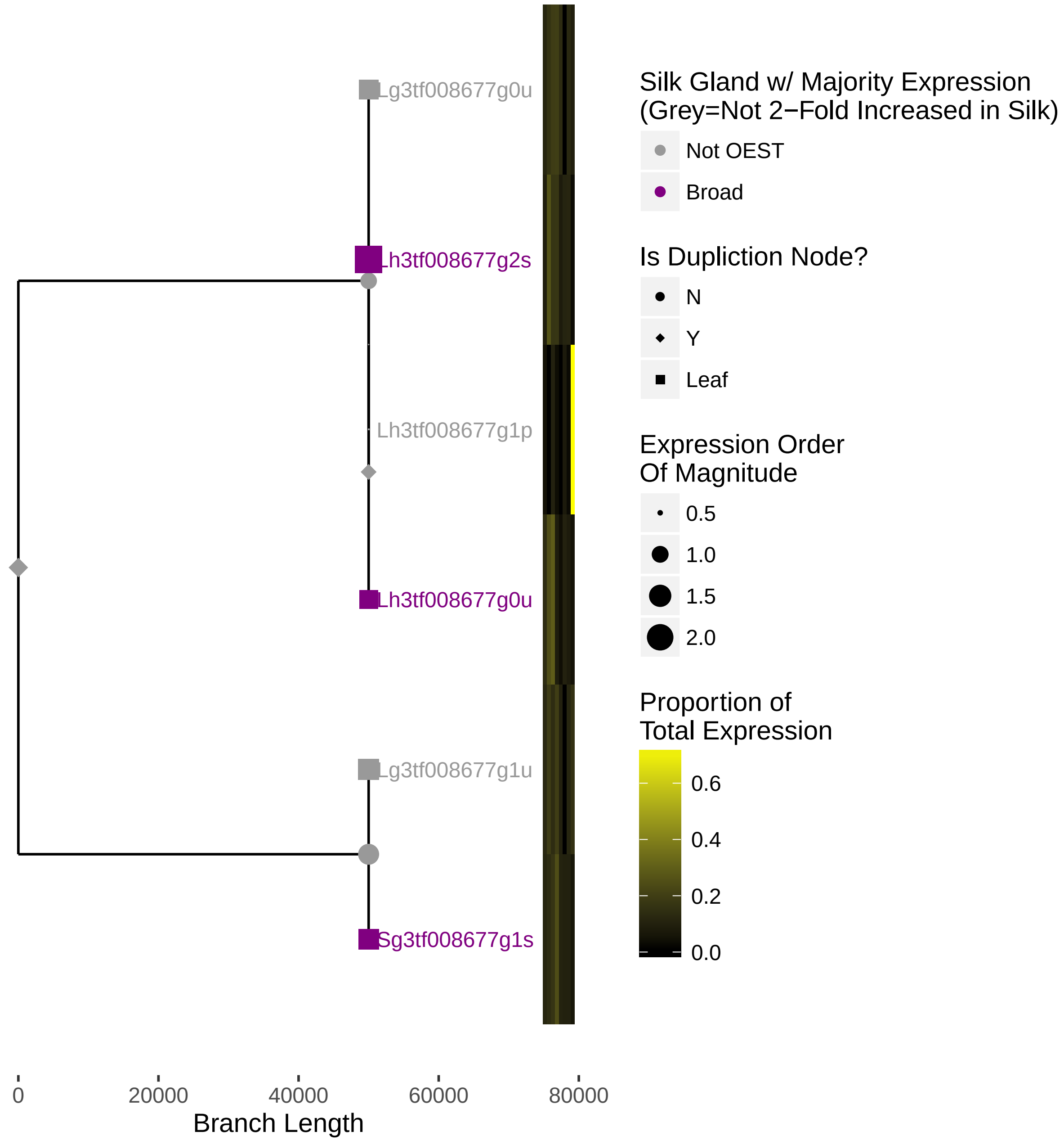

Lg3tf008677g0u
Lh3tf008677g2s
Lh3tf008677g1p
Lh3tf008677g0u
Lg3tf008677g1u
Sg3tf008677g1s
0
20000
40000
60000
80000
Branch Length
Silk Gland w/ Majority Expression
(Grey=Not 2−Fold Increased in Silk)
Not OEST
Broad
Is Dupliction Node?
N
Y
Leaf
Expression Order
Of Magnitude
0.5
1.0
1.5
2.0
Proportion of
Total Expression
0.6
0.4
0.2
0.0

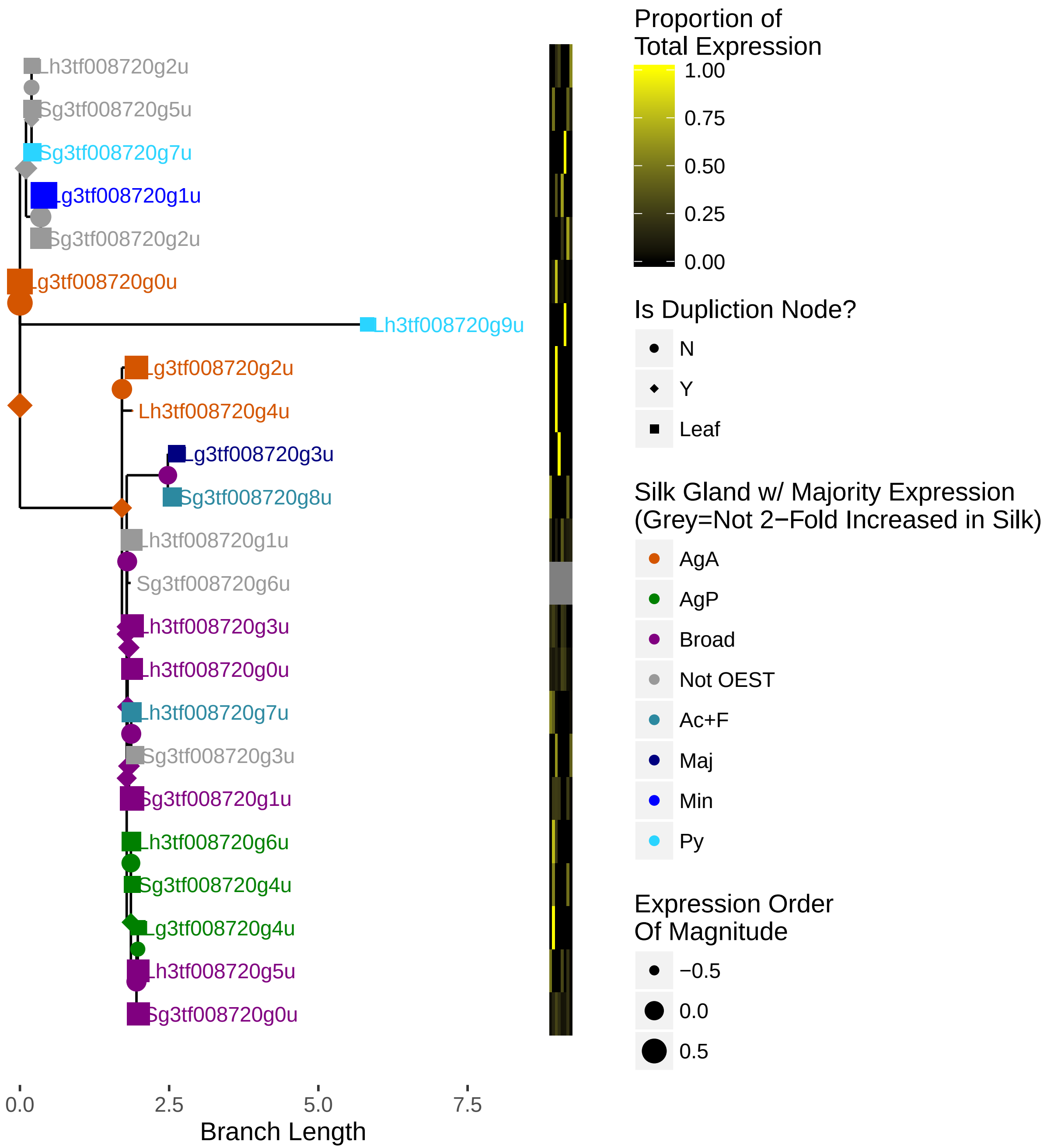

Lh3tf008720g2u
Sg3tf008720g5u
Sg3tf008720g7u
Lg3tf008720g1u
Sg3tf008720g2u
Lg3tf008720g0u
Lh3tf008720g9u
Lg3tf008720g2u
Lh3tf008720g4u
Lg3tf008720g3u
Sg3tf008720g8u
Lh3tf008720g1u
Sg3tf008720g6u
Lh3tf008720g3u
Lh3tf008720g0u
Lh3tf008720g7u
Sg3tf008720g3u
Sg3tf008720g1u
Lh3tf008720g6u
Sg3tf008720g4u
Lg3tf008720g4u
Lh3tf008720g5u
Sg3tf008720g0u
0.0
2.5
5.0
7.5
Branch Length
Proportion of Total Expression
1.00
0.75
0.50
0.25
0.00
Is Dupliction Node?
N
Y
Leaf
Silk Gland w/ Majority Expression (Grey=Not 2−Fold Increased in Silk)
AgA
AgP
Broad
Not OEST
Ac+F
Maj
Min
Py
Expression Order Of Magnitude
−0.5
0.0
0.5

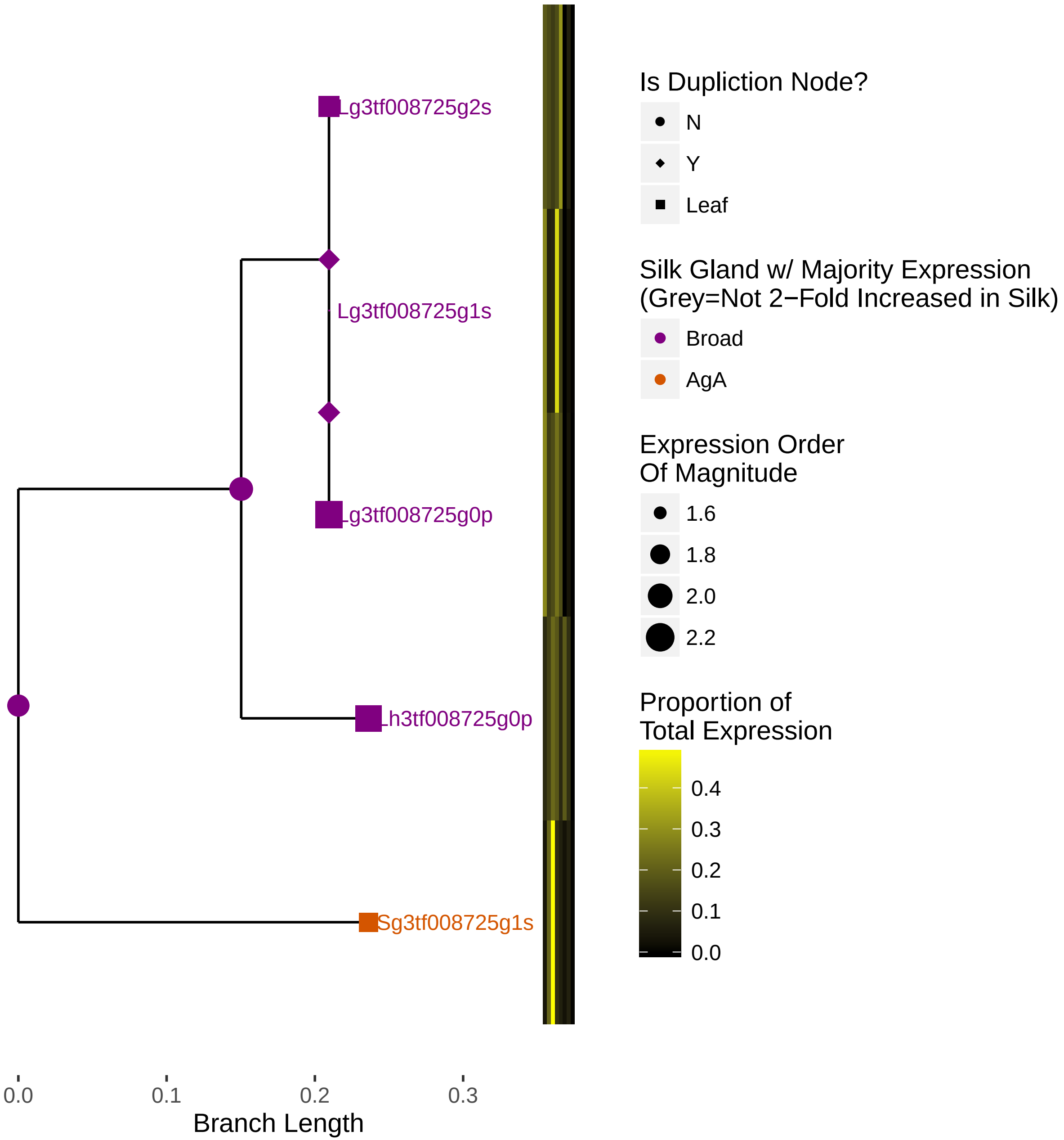

Lg3tf008725g2s
Lg3tf008725g1s
Lg3tf008725g0p
Lh3tf008725g0p
Sg3tf008725g1s
0.0
0.1
0.2
0.3
Branch Length
Is Dupliction Node?
N
Y
Leaf
Silk Gland w/ Majority Expression
(Grey=Not 2−Fold Increased in Silk)
Broad
AgA
Expression Order
Of Magnitude
1.6
1.8
2.0
2.2
Proportion of
Total Expression
0.4
0.3
0.2
0.1
0.0

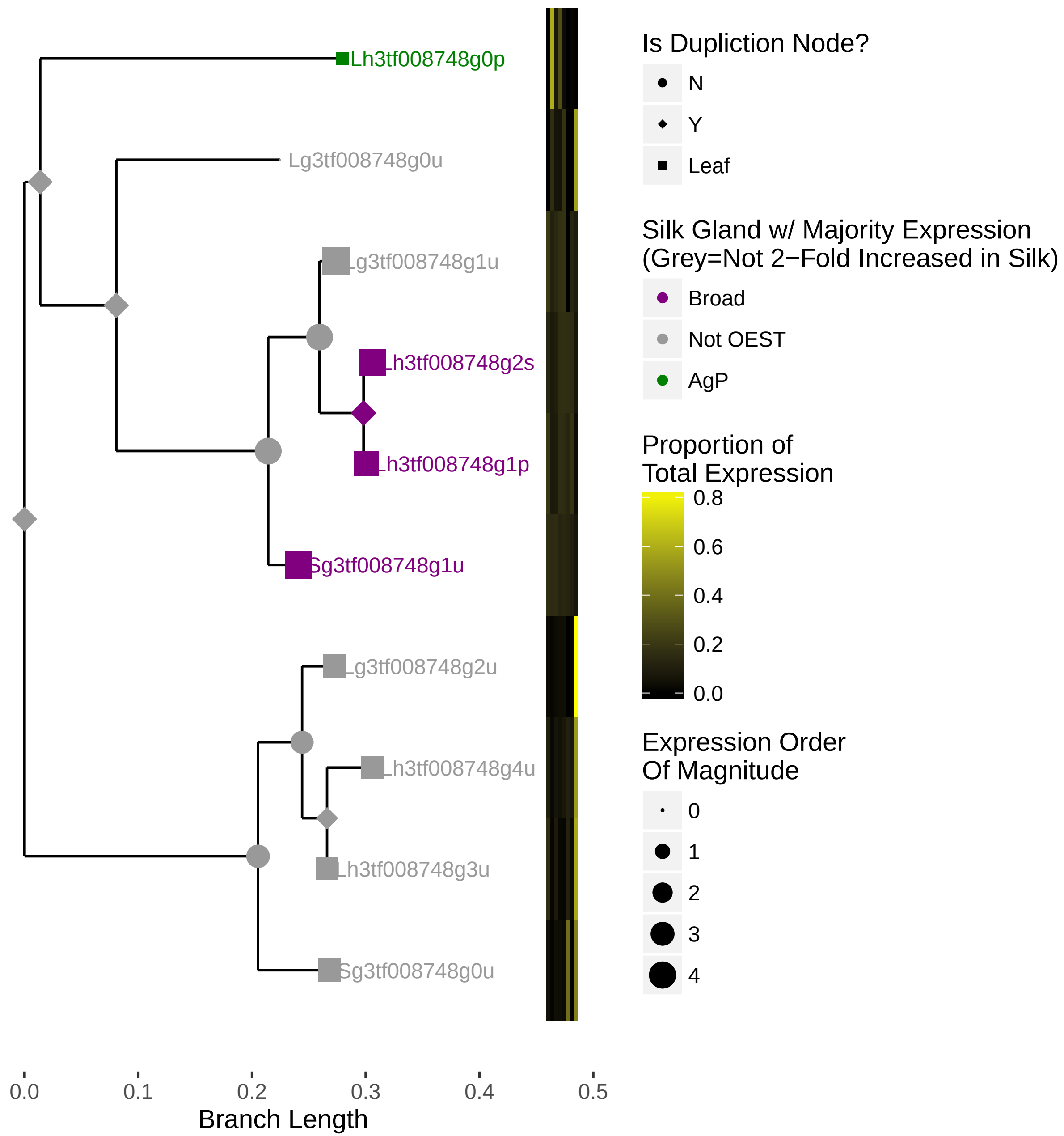
Lh3tf008748g0p
Lg3tf008748g0u
Lg3tf008748g1u
Lh3tf008748g2s
Lh3tf008748g1p
Sg3tf008748g1u
Lg3tf008748g2u
Lh3tf008748g4u
Lh3tf008748g3u
Sg3tf008748g0u
0.0
0.1
0.2
0.3
0.4
0.5
Branch Length
Is Dupliction Node?
N
Y
Leaf
Silk Gland w/ Majority Expression
(Grey=Not 2−Fold Increased in Silk)
Broad
Not OEST
AgP
Proportion of
Total Expression
0.8
0.6
0.4
0.2
0.0
Expression Order
Of Magnitude
0
1
2
3
4

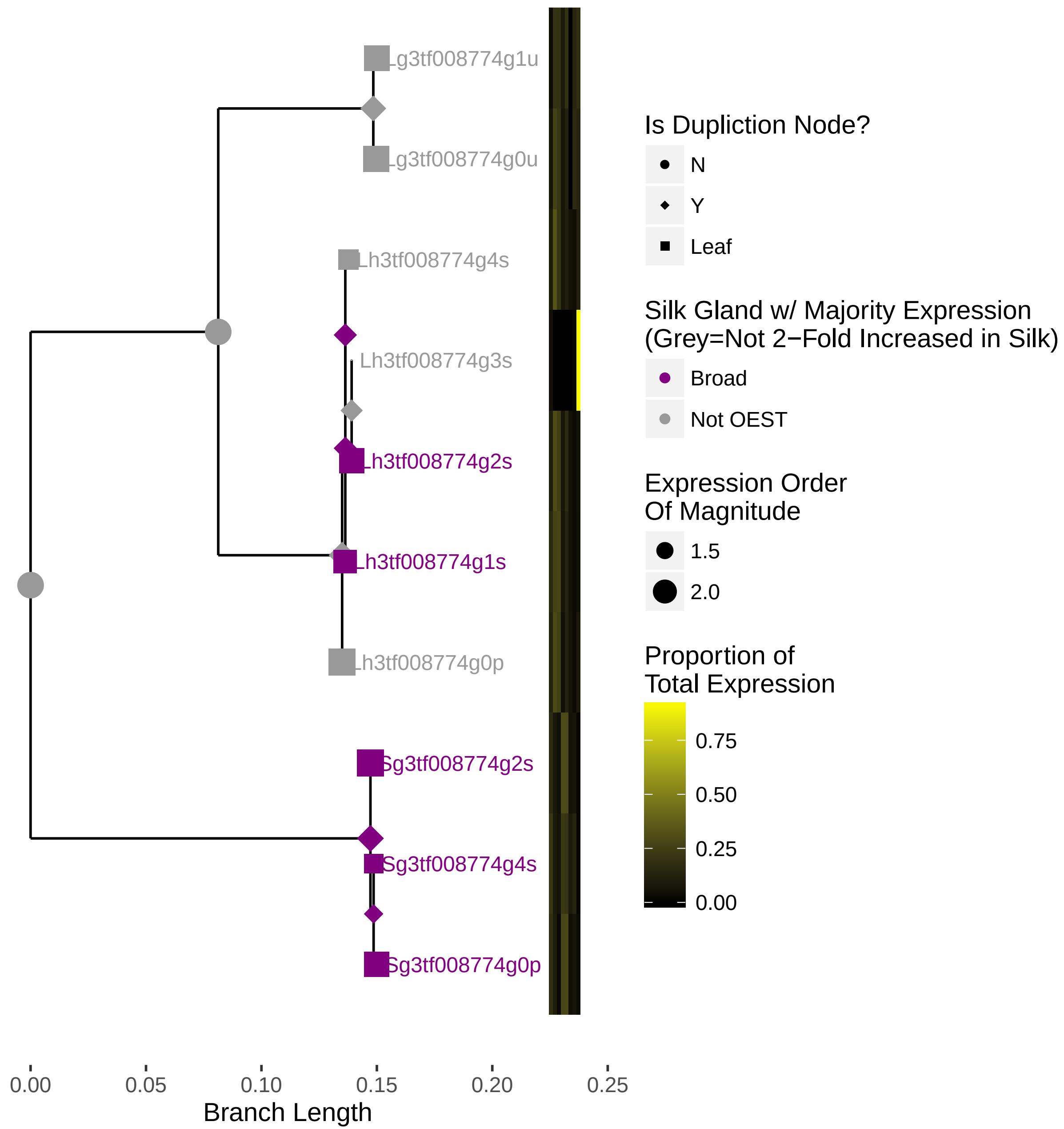

Lg3tf008774g1u
Lg3tf008774g0u
Lh3tf008774g4s
Lh3tf008774g3s
Lh3tf008774g2s
Lh3tf008774g1s
Lh3tf008774g0p
Sg3tf008774g2s
Sg3tf008774g4s
Sg3tf008774g0p
0.00
0.05
0.10
0.15
0.20
0.25
Branch Length
Is Dupliction Node?
N
Y
Leaf
Silk Gland w/ Majority Expression
(Grey=Not 2−Fold Increased in Silk)
Broad
Not OEST
Expression Order
Of Magnitude
1.5
2.0
Proportion of
Total Expression
0.75
0.50
0.25
0.00

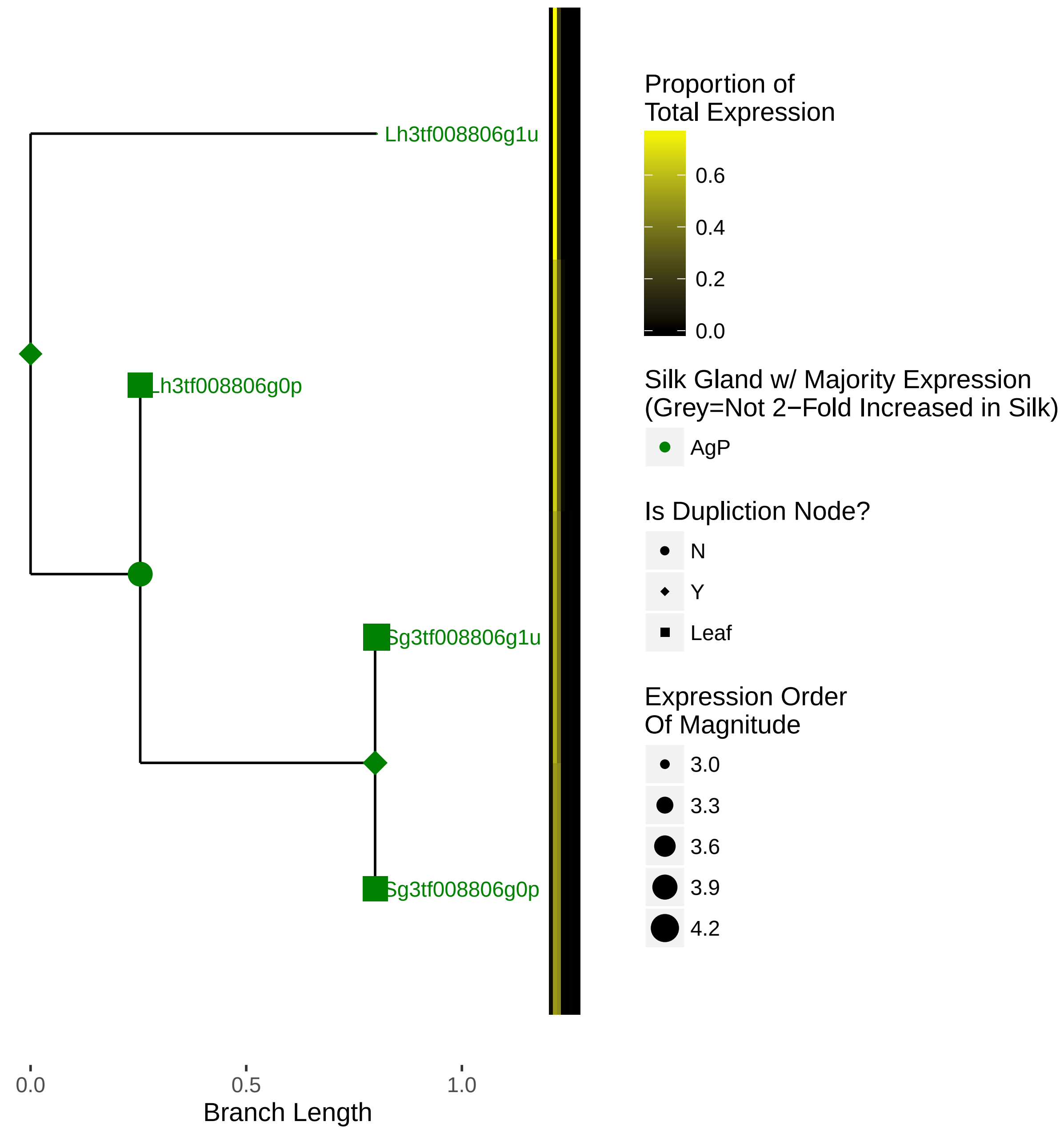
Lh3tf008806g1u
Lh3tf008806g0p
Sg3tf008806g1u
Sg3tf008806g0p
0.0
0.5
1.0
Branch Length
Proportion of
Total Expression
0.6
0.4
0.2
0.0
Silk Gland w/ Majority Expression
(Grey=Not 2−Fold Increased in Silk)
AgP
Is Dupliction Node?
N
Y
Leaf
Expression Order
Of Magnitude
3.0
3.3
3.6
3.9
4.2

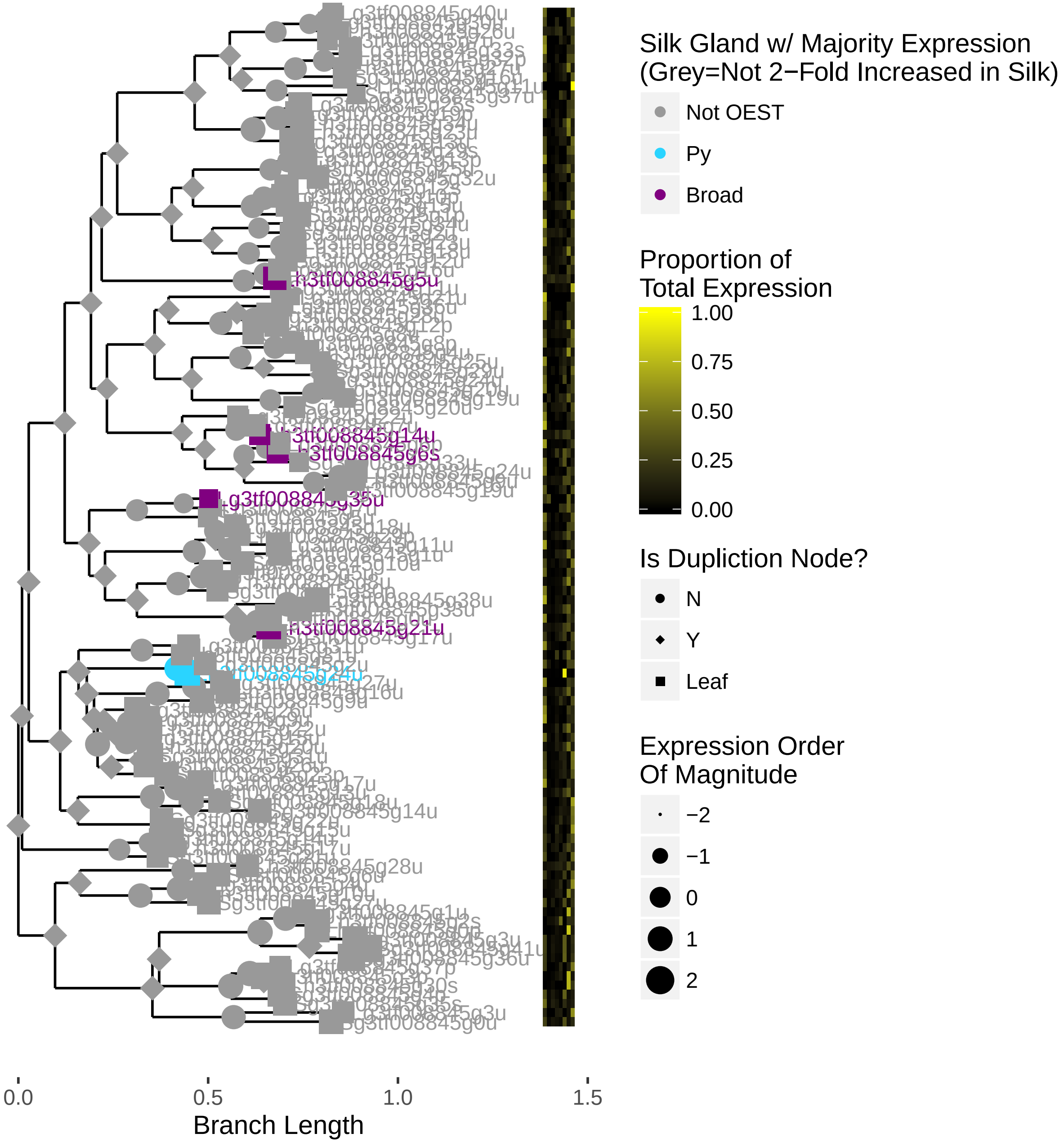

Silk Gland w/ Majority Expression
(Grey=Not 2−Fold Increased in Silk)
Not OEST
Py
Broad
Proportion of
Total Expression
1.00
0.75
0.50
0.25
0.00
Is Dupliction Node?
N
Y
Leaf
Expression Order
Of Magnitude
−2
−1
0
1
2
0.0
0.5
1.0
1.5
Branch Length

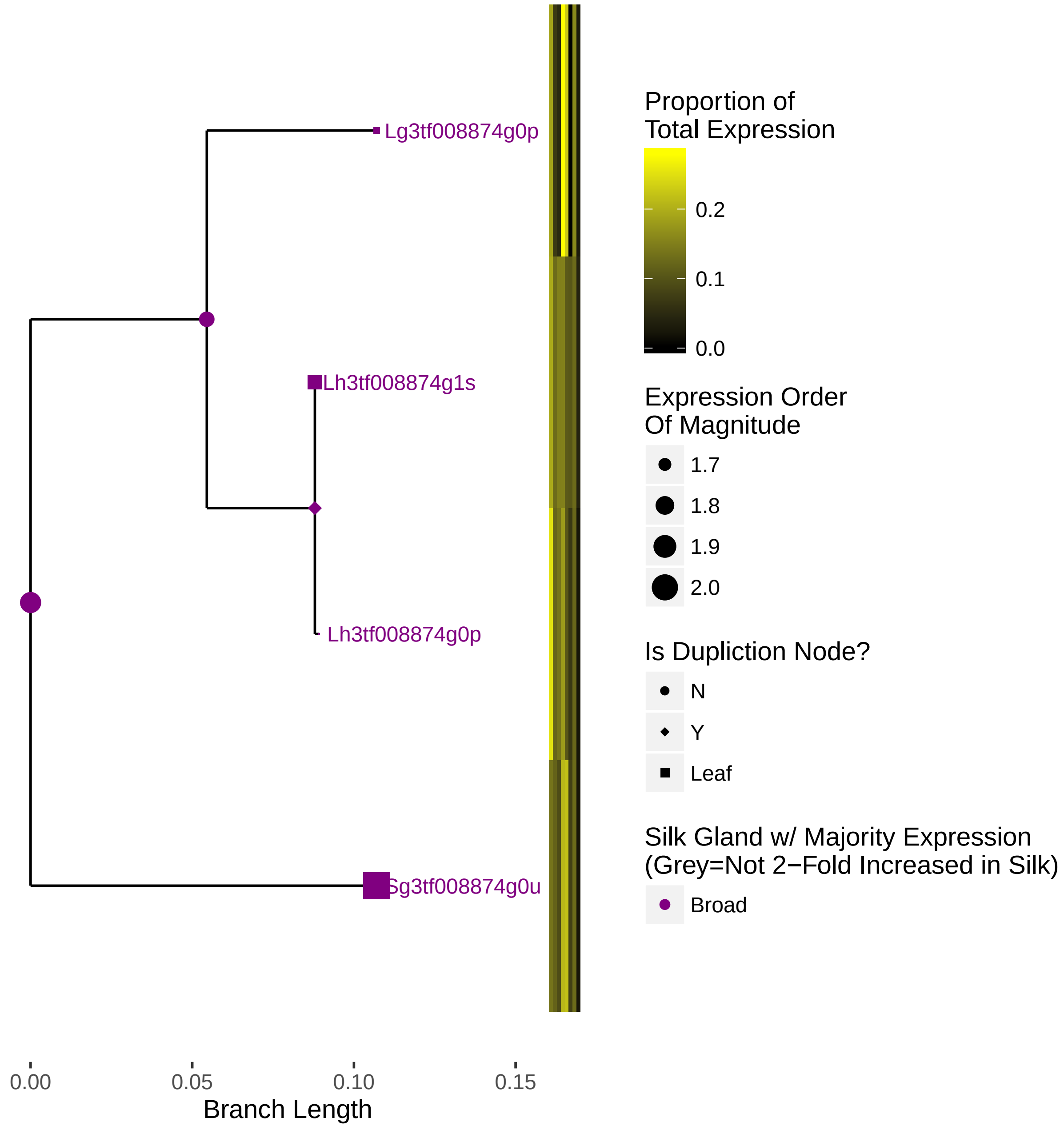

Lg3tf008874g0p
Lh3tf008874g1s
Lh3tf008874g0p
Sg3tf008874g0u
0.00
0.05
0.10
0.15
Branch Length
Proportion of
Total Expression
0.2
0.1
0.0
Expression Order
Of Magnitude
1.7
1.8
1.9
2.0
Is Dupliction Node?
N
Y
Leaf
Silk Gland w/ Majority Expression
(Grey=Not 2−Fold Increased in Silk)
Broad

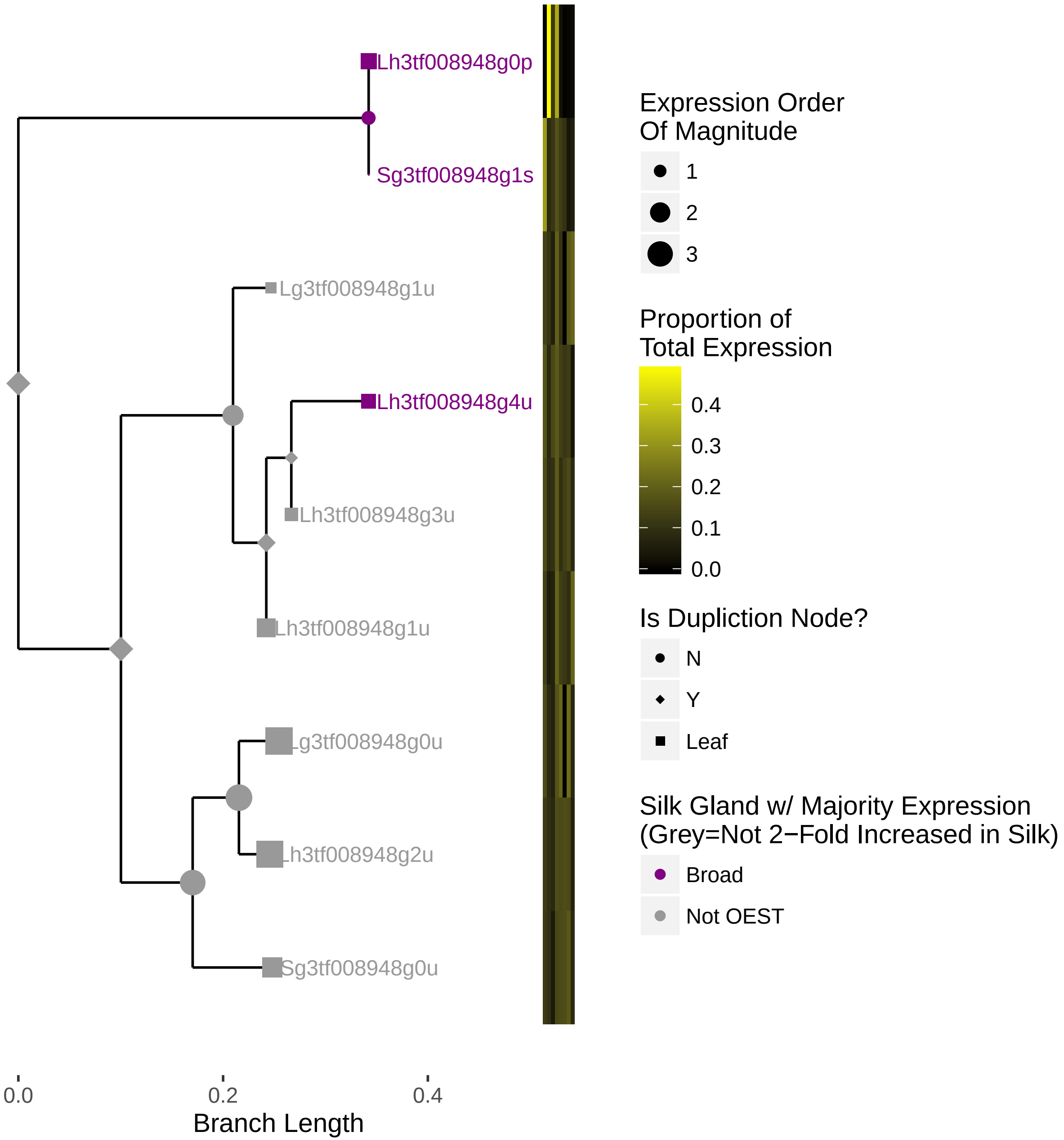

Lh3tf008948g0p
Sg3tf008948g1s
Lg3tf008948g1u
Lh3tf008948g4u
Lh3tf008948g3u
Lh3tf008948g1u
Lg3tf008948g0u
Lh3tf008948g2u
Sg3tf008948g0u
0.0
0.2
0.4
Branch Length
Expression Order
Of Magnitude
1
2
3
Proportion of
Total Expression
0.4
0.3
0.2
0.1
0.0
Is Dupliction Node?
N
Y
Leaf
Silk Gland w/ Majority Expression
(Grey=Not 2−Fold Increased in Silk)
Broad
Not OEST

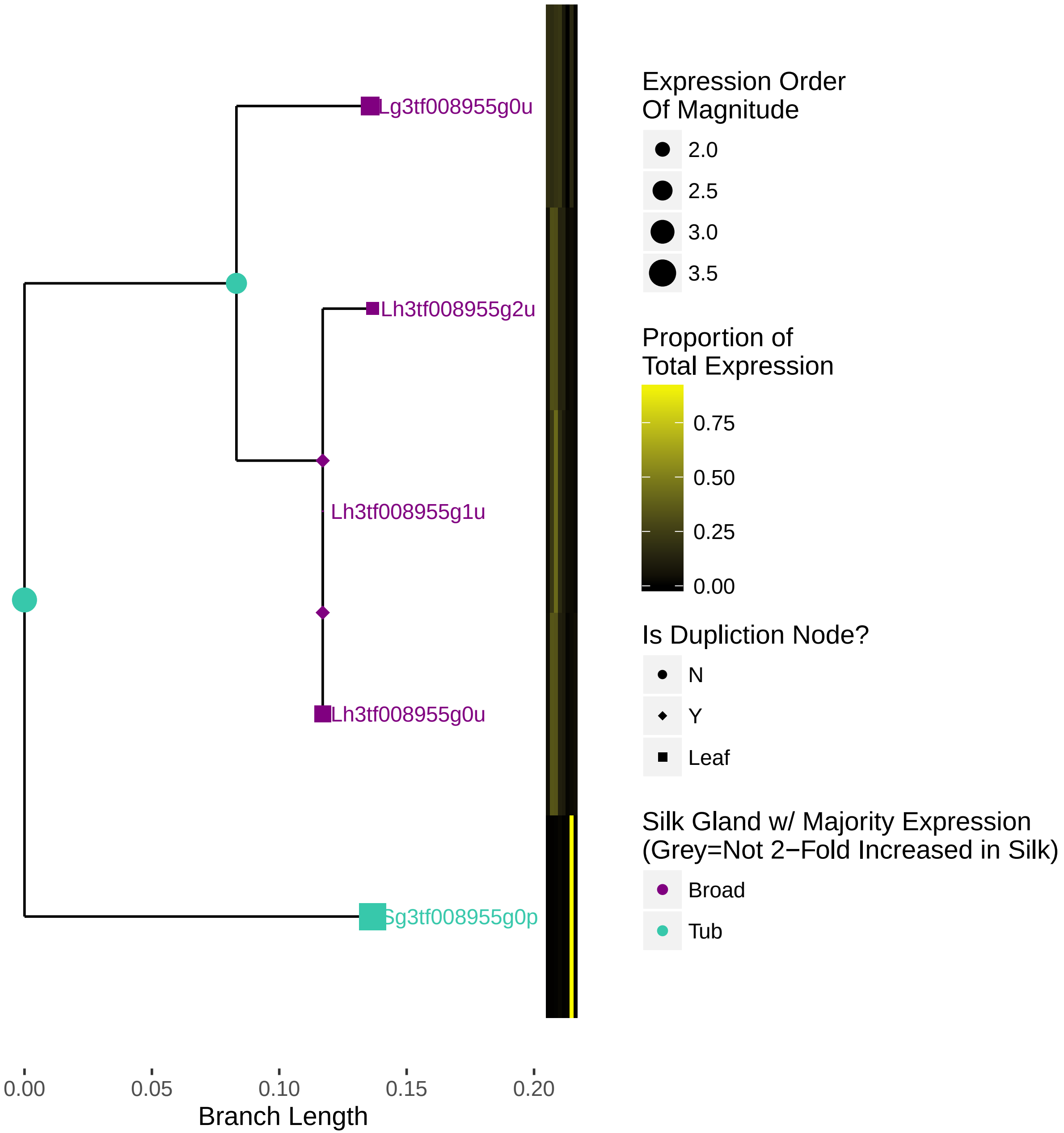
Lg3tf008955g0u
Lh3tf008955g2u
Lh3tf008955g1u
Lh3tf008955g0u
Sg3tf008955g0p
0.00
0.05
0.10
0.15
0.20
Branch Length
Expression Order
Of Magnitude
2.0
2.5
3.0
3.5
Proportion of
Total Expression
0.75
0.50
0.25
0.00
Is Dupliction Node?
N
Y
Leaf
Silk Gland w/ Majority Expression
(Grey=Not 2−Fold Increased in Silk)
Broad
Tub

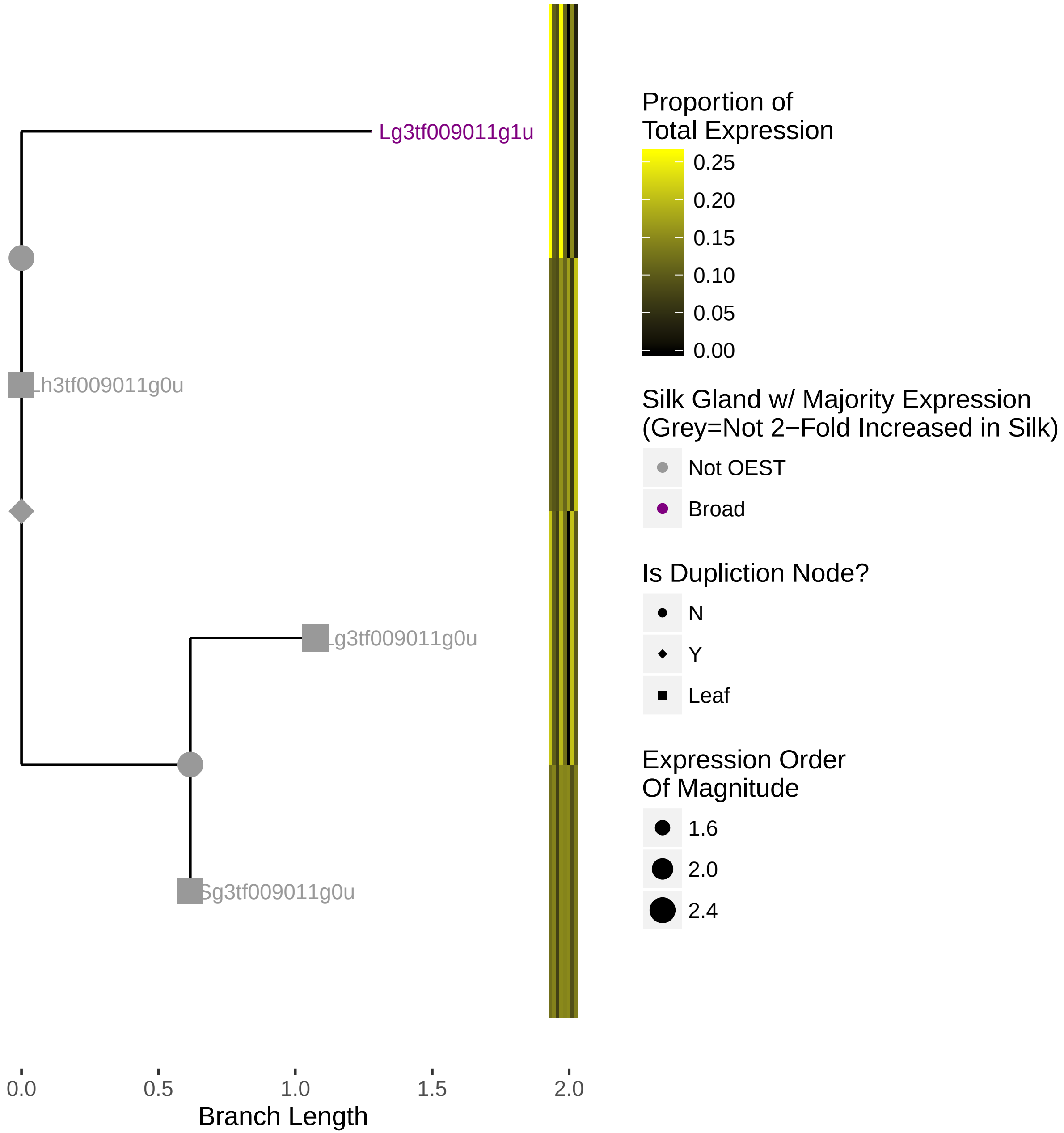

Lg3tf009011g1u
Lh3tf009011g0u
Lg3tf009011g0u
Sg3tf009011g0u
0.0
0.5
1.0
1.5
2.0
Branch Length
Proportion of
Total Expression
0.25
0.20
0.15
0.10
0.05
0.00
Silk Gland w/ Majority Expression
(Grey=Not 2−Fold Increased in Silk)
Not OEST
Broad
Is Dupliction Node?
N
Y
Leaf
Expression Order
Of Magnitude
1.6
2.0
2.4

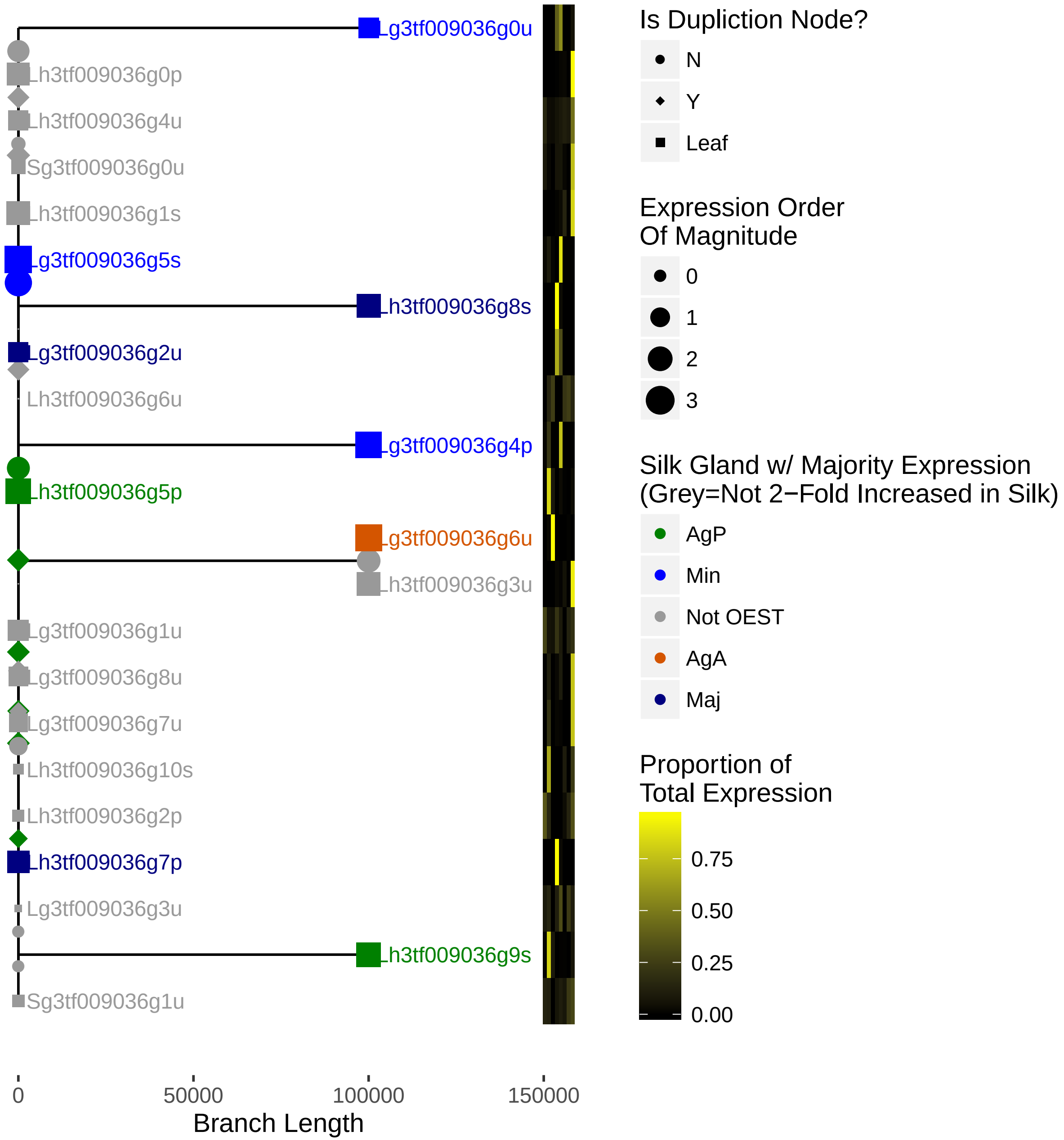
Lg3tf009036g0u
Lh3tf009036g0p
Lh3tf009036g4u
Sg3tf009036g0u
Lh3tf009036g1s
Lg3tf009036g5s
Lh3tf009036g8s
Lg3tf009036g2u
Lh3tf009036g6u
Lg3tf009036g4p
Lh3tf009036g5p
Lg3tf009036g6u
Lh3tf009036g3u
Lg3tf009036g1u
Lg3tf009036g8u
Lg3tf009036g7u
Lh3tf009036g10s
Lh3tf009036g2p
Lh3tf009036g7p
Lg3tf009036g3u
Lh3tf009036g9s
Sg3tf009036g1u
0
50000
100000
150000
Branch Length
Is Dupliction Node?
N
Y
Leaf
Expression Order
Of Magnitude
0
1
2
3
Silk Gland w/ Majority Expression
(Grey=Not 2−Fold Increased in Silk)
AgP
Min
Not OEST
AgA
Maj
Proportion of
Total Expression
0.75
0.50
0.25
0.00

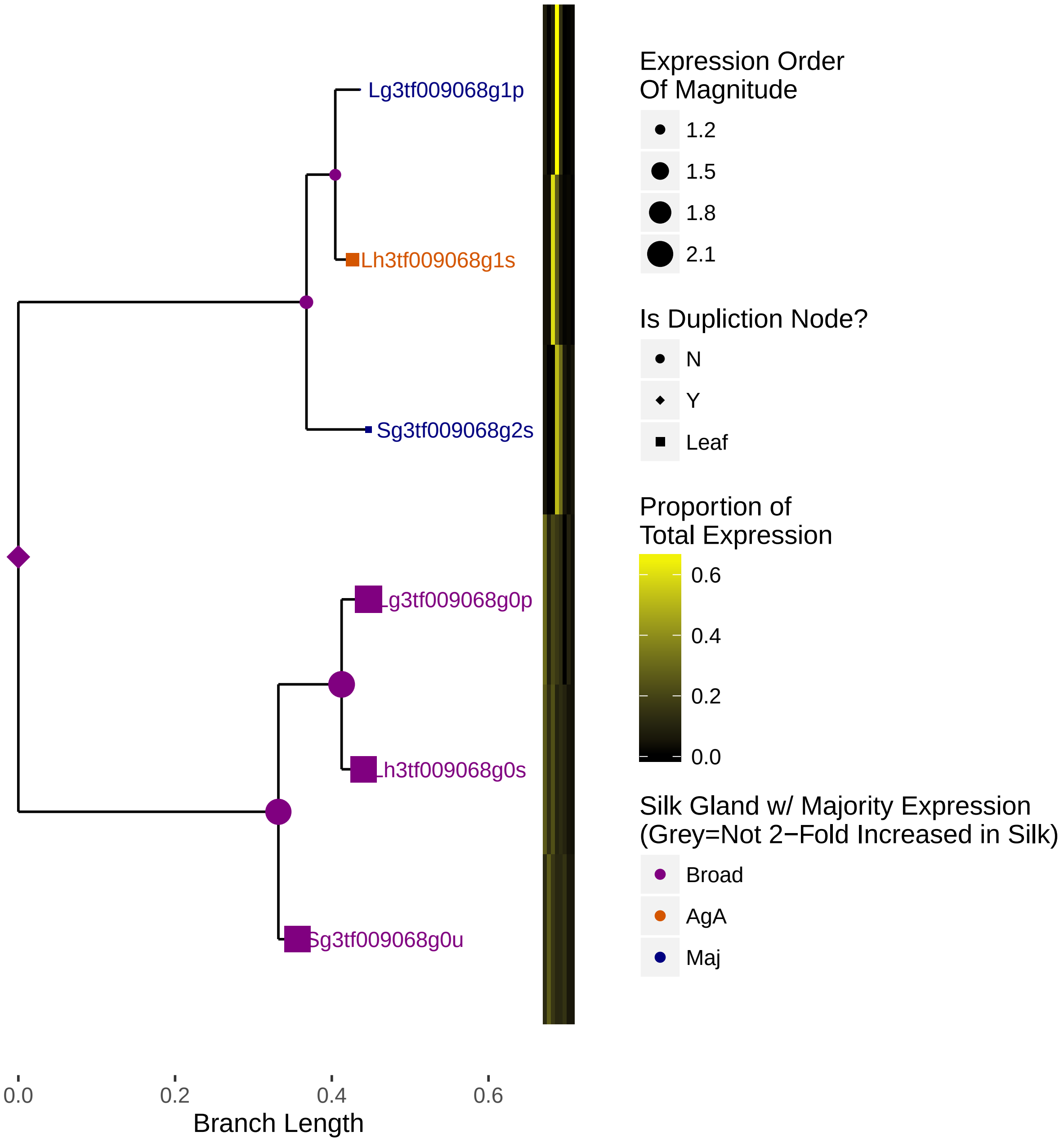

Lg3tf009068g1p
Lh3tf009068g1s
Sg3tf009068g2s
Lg3tf009068g0p
Lh3tf009068g0s
Sg3tf009068g0u
0.0
0.2
0.4
0.6
Branch Length
Expression Order Of Magnitude
1.2
1.5
1.8
2.1
Is Dupliction Node?
N
Y
Leaf
Proportion of Total Expression
0.6
0.4
0.2
0.0
Silk Gland w/ Majority Expression (Grey=Not 2−Fold Increased in Silk)
Broad
AgA
Maj

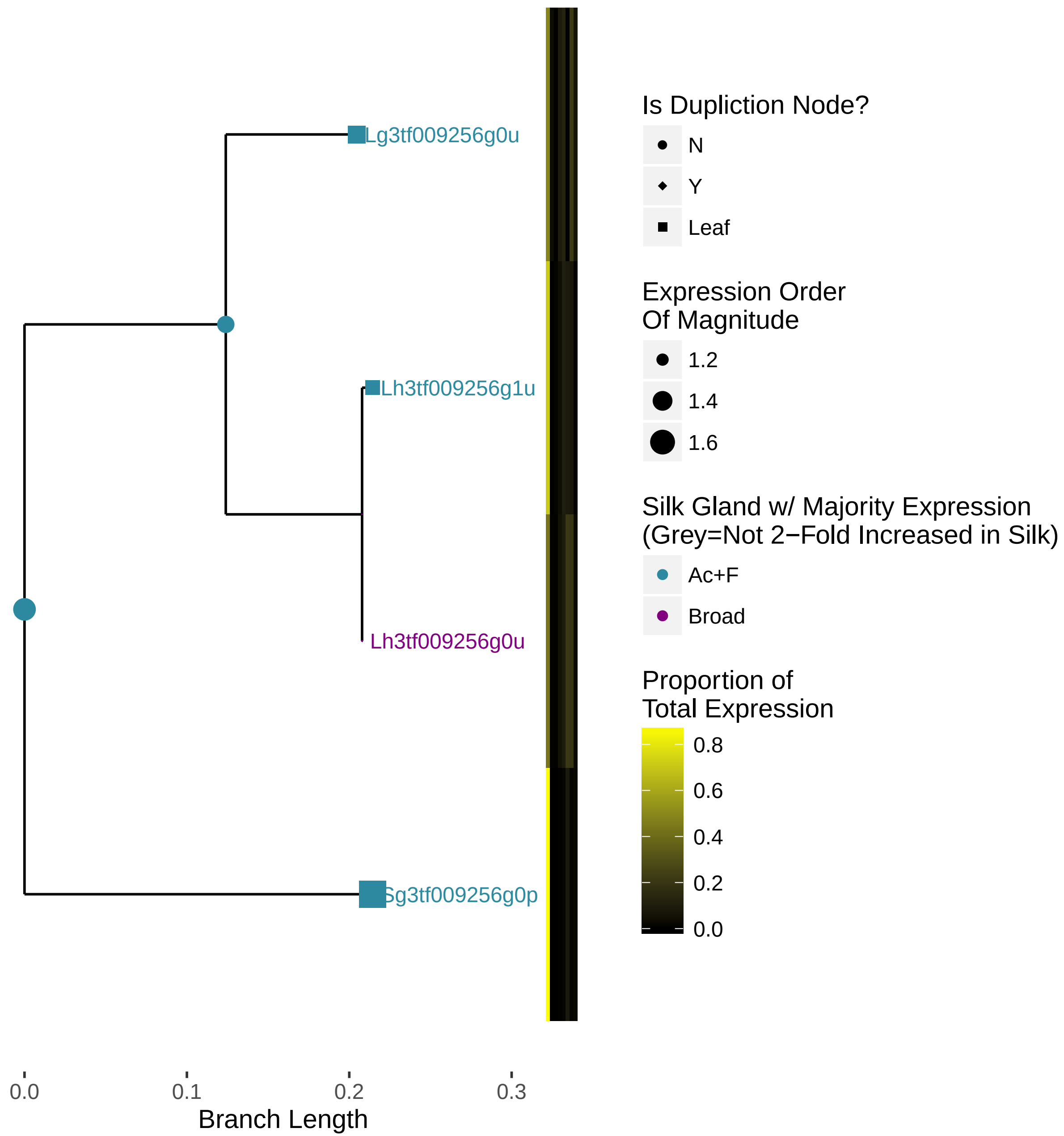

Lg3tf009256g0u
Lh3tf009256g1u
Lh3tf009256g0u
Sg3tf009256g0p
0.0
0.1
0.2
0.3
Branch Length
Is Dupliction Node?
N
Y
Leaf
Expression Order
Of Magnitude
1.2
1.4
1.6
Silk Gland w/ Majority Expression
(Grey=Not 2−Fold Increased in Silk)
Ac+F
Broad
Proportion of
Total Expression
0.8
0.6
0.4
0.2
0.0

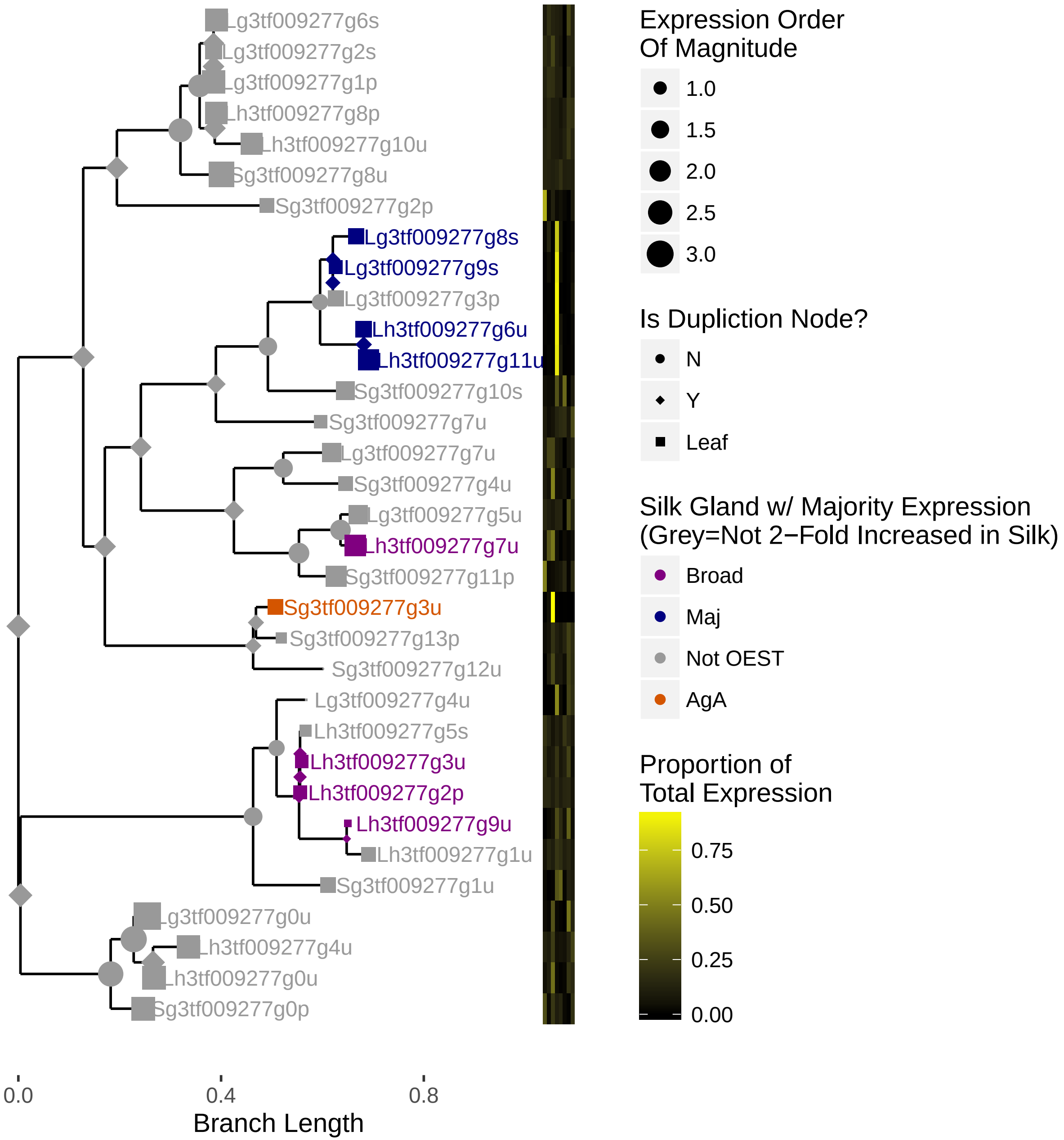

Lg3tf009277g6s
Lg3tf009277g2s
Lg3tf009277g1p
Lh3tf009277g8p
Lh3tf009277g10u
Sg3tf009277g8u
Sg3tf009277g2p
Lg3tf009277g8s
Lg3tf009277g9s
Lg3tf009277g3p
Lh3tf009277g6u
Lh3tf009277g11u
Sg3tf009277g10s
Sg3tf009277g7u
Lg3tf009277g7u
Sg3tf009277g4u
Lg3tf009277g5u
Lh3tf009277g7u
Sg3tf009277g11p
Sg3tf009277g3u
Sg3tf009277g13p
Sg3tf009277g12u
Lg3tf009277g4u
Lh3tf009277g5s
Lh3tf009277g3u
Lh3tf009277g2p
Lh3tf009277g9u
Lh3tf009277g1u
Sg3tf009277g1u
Lg3tf009277g0u
Lh3tf009277g4u
Lh3tf009277g0u
Sg3tf009277g0p
0.0
0.4
0.8
Branch Length
Expression Order Of Magnitude
1.0
1.5
2.0
2.5
3.0
Is Dupliction Node?
N
Y
Leaf
Silk Gland w/ Majority Expression (Grey=Not 2−Fold Increased in Silk)
Broad
Maj
Not OEST
AgA
Proportion of Total Expression
0.75
0.50
0.25
0.00

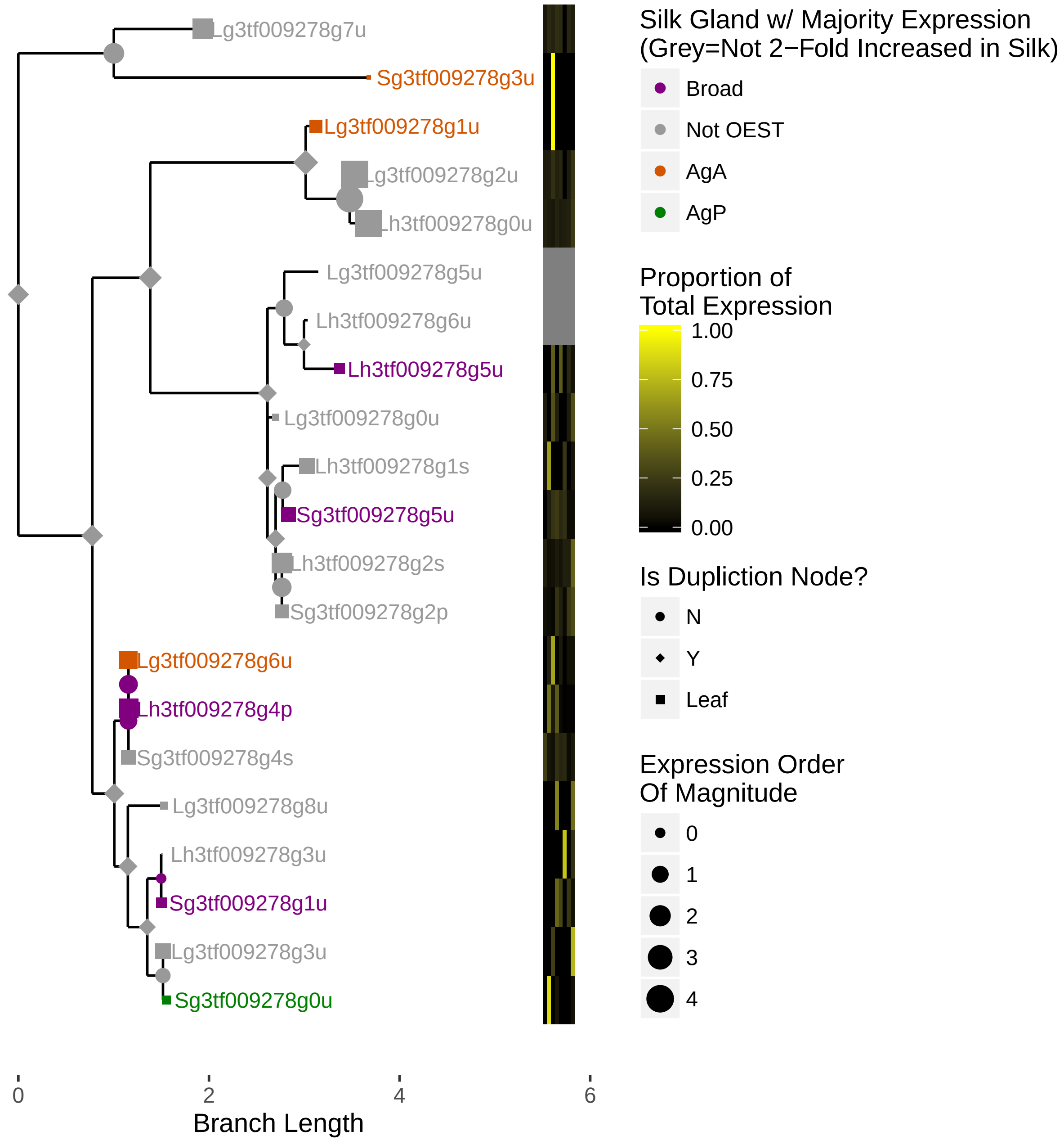
Lg3tf009278g7u
Sg3tf009278g3u
Lg3tf009278g1u
Lg3tf009278g2u
Lh3tf009278g0u
Lg3tf009278g5u
Lh3tf009278g6u
Lh3tf009278g5u
Lg3tf009278g0u
Lh3tf009278g1s
Sg3tf009278g5u
Lh3tf009278g2s
Sg3tf009278g2p
Lg3tf009278g6u
Lh3tf009278g4p
Sg3tf009278g4s
Lg3tf009278g8u
Lh3tf009278g3u
Sg3tf009278g1u
Lg3tf009278g3u
Sg3tf009278g0u
0
2
4
6
Branch Length
Silk Gland w/ Majority Expression
(Grey=Not 2−Fold Increased in Silk)
Broad
Not OEST
AgA
AgP
Proportion of
Total Expression
1.00
0.75
0.50
0.25
0.00
Is Dupliction Node?
N
Y
Leaf
Expression Order
Of Magnitude
0
1
2
3
4

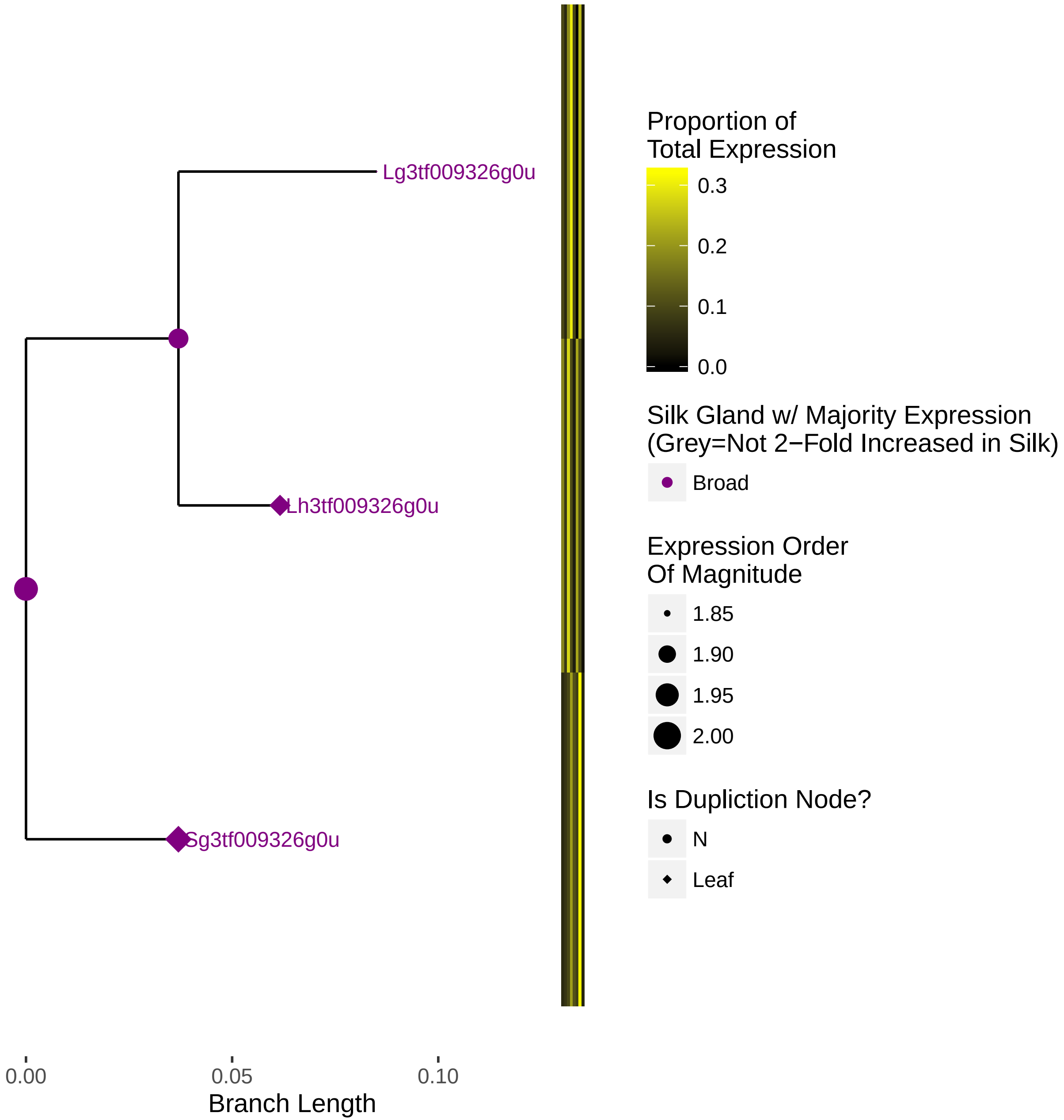

Lg3tf009326g0u
Lh3tf009326g0u
Sg3tf009326g0u
0.00
0.05
0.10
Branch Length
Proportion of
Total Expression
0.3
0.2
0.1
0.0
Silk Gland w/ Majority Expression
(Grey=Not 2−Fold Increased in Silk)
Broad
Expression Order
Of Magnitude
1.85
1.90
1.95
2.00
Is Dupliction Node?
N
Leaf

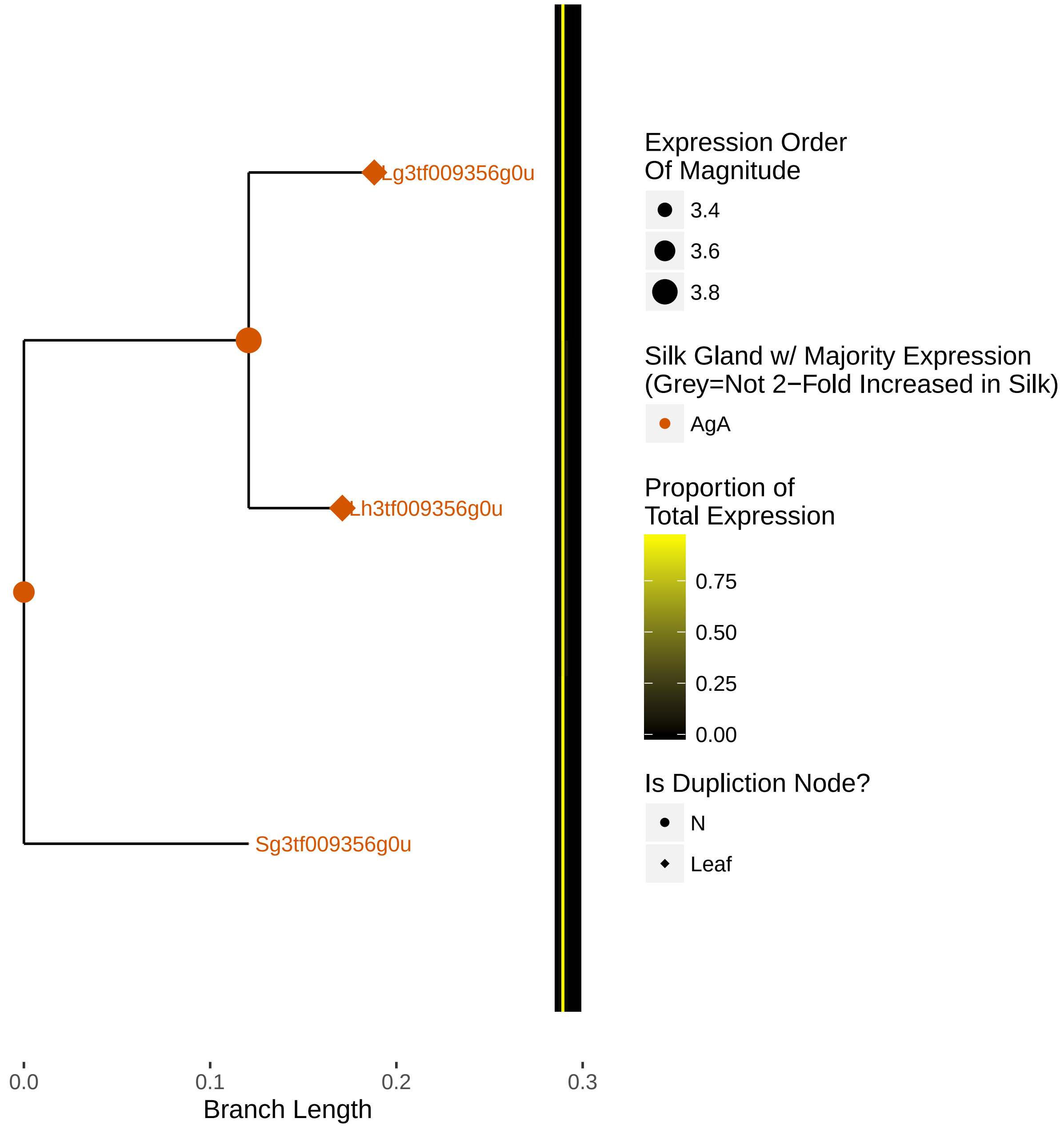

Lg3tf009356g0u
Lh3tf009356g0u
Sg3tf009356g0u
0.0
0.1
0.2
0.3
Branch Length
Expression Order
Of Magnitude
3.4
3.6
3.8
Silk Gland w/ Majority Expression
(Grey=Not 2−Fold Increased in Silk)
AgA
Proportion of
Total Expression
0.75
0.50
0.25
0.00
Is Dupliction Node?
N
Leaf

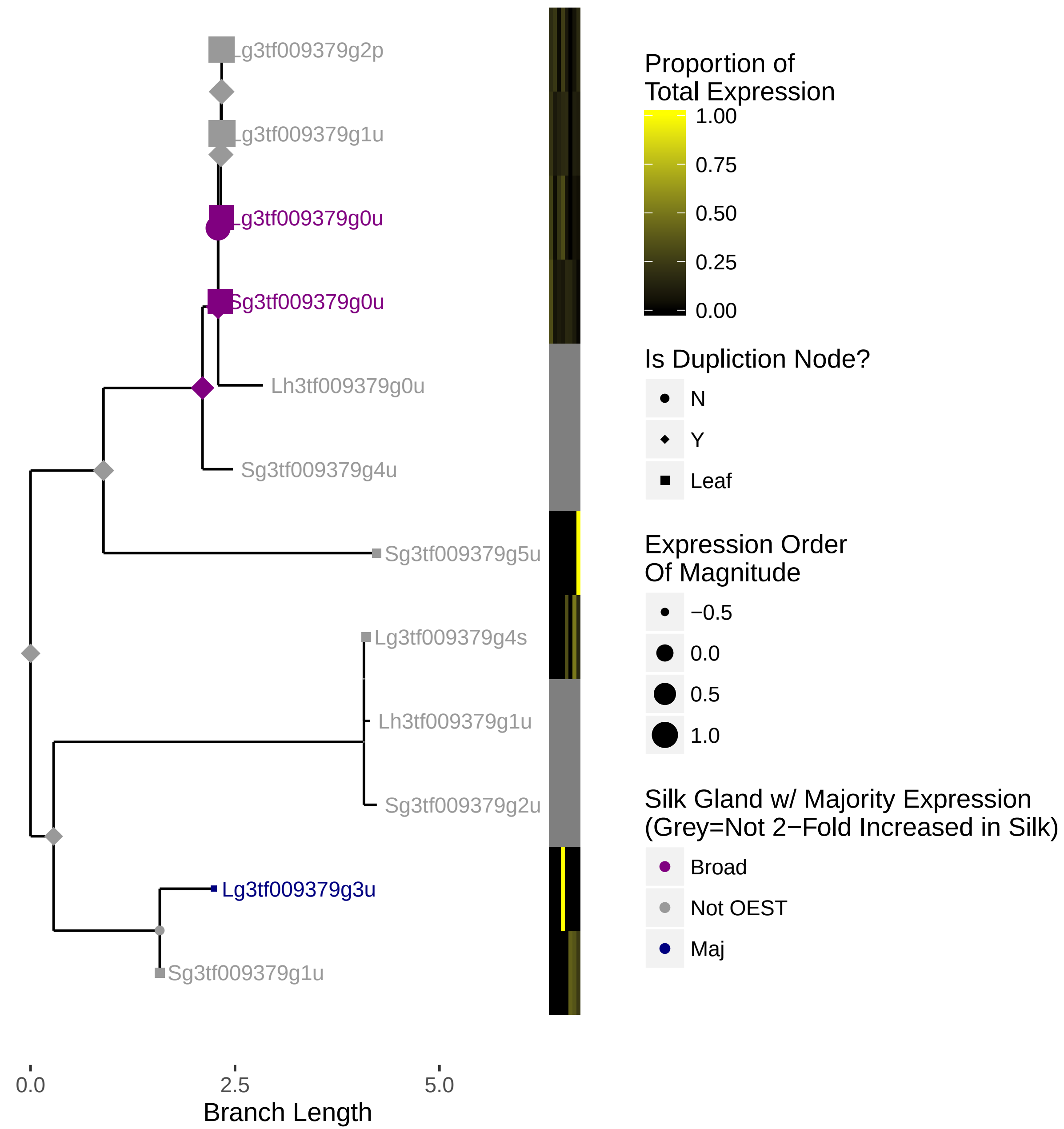

Lg3tf009379g2p
Lg3tf009379g1u
Lg3tf009379g0u
Sg3tf009379g0u
Lh3tf009379g0u
Sg3tf009379g4u
Sg3tf009379g5u
Lg3tf009379g4s
Lh3tf009379g1u
Sg3tf009379g2u
Lg3tf009379g3u
Sg3tf009379g1u
0.0
2.5
5.0
Branch Length
Proportion of
Total Expression
1.00
0.75
0.50
0.25
0.00
Is Dupliction Node?
N
Y
Leaf
Expression Order
Of Magnitude
−0.5
0.0
0.5
1.0
Silk Gland w/ Majority Expression
(Grey=Not 2−Fold Increased in Silk)
Broad
Not OEST
Maj

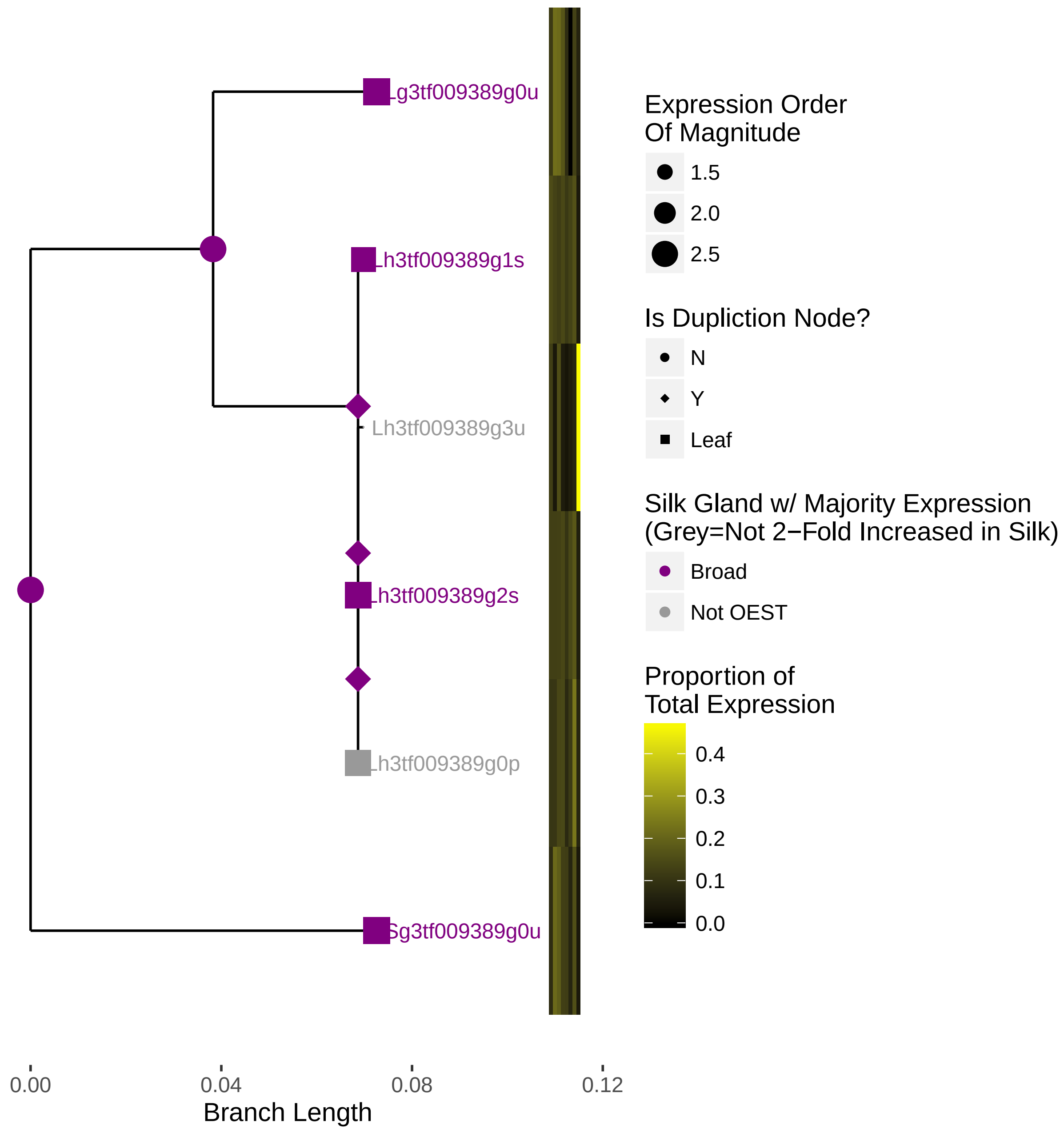

Lg3tf009389g0u
Lh3tf009389g1s
Lh3tf009389g3u
Lh3tf009389g2s
Lh3tf009389g0p
Sg3tf009389g0u
Expression Order
Of Magnitude
1.5
2.0
2.5
Is Dupliction Node?
N
Y
Leaf
Silk Gland w/ Majority Expression
(Grey=Not 2−Fold Increased in Silk)
Broad
Not OEST
Proportion of
Total Expression
0.4
0.3
0.2
0.1
0.0
0.00
0.04
0.08
0.12
Branch Length

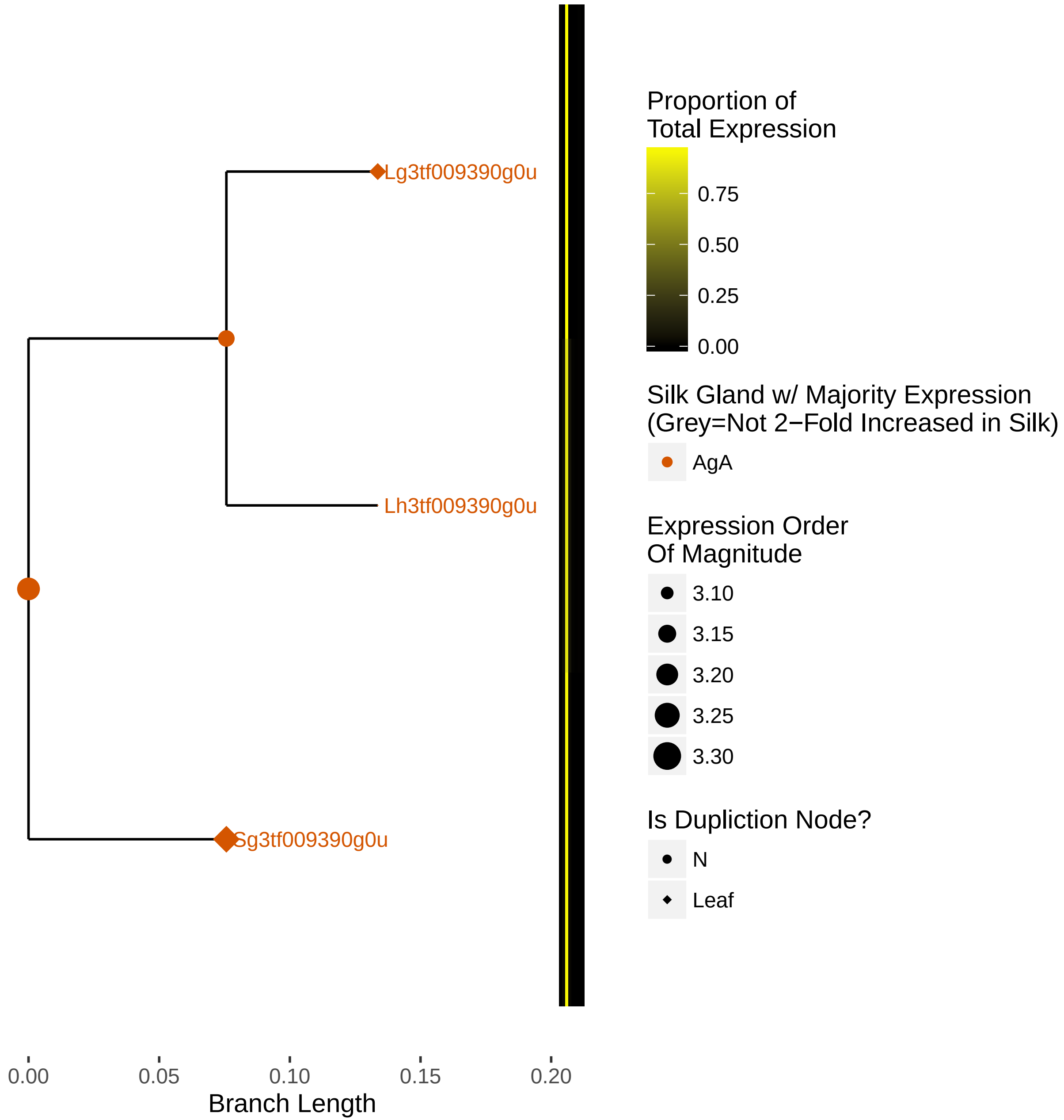
Lg3tf009390g0u
Lh3tf009390g0u
Sg3tf009390g0u
Proportion of
Total Expression
0.75
0.50
0.25
0.00
Silk Gland w/ Majority Expression
(Grey=Not 2−Fold Increased in Silk)
AgA
Expression Order
Of Magnitude
3.10
3.15
3.20
3.25
3.30
Is Dupliction Node?
N
Leaf
0.00
0.05
0.10
0.15
0.20
Branch Length

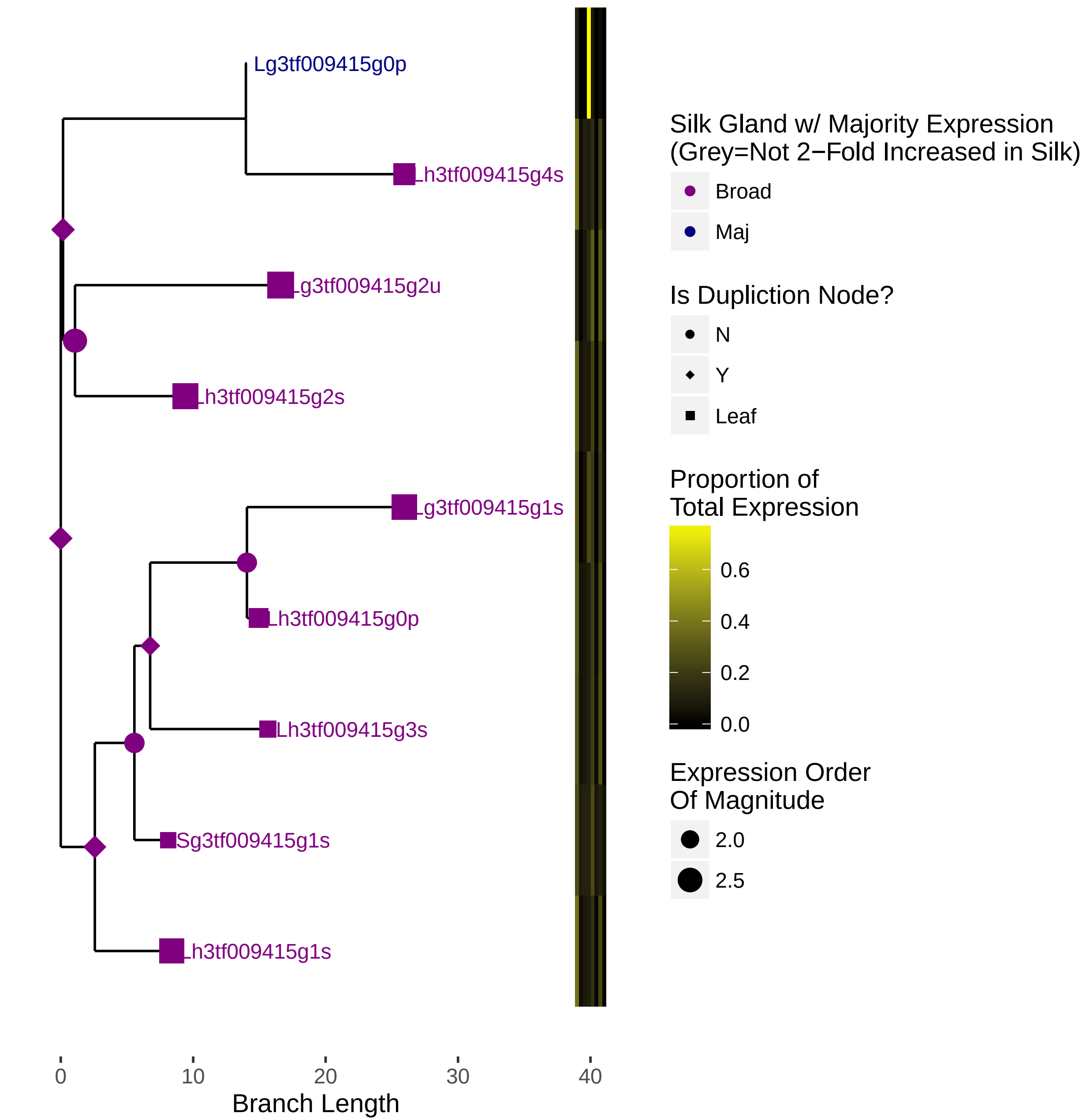

Lg3tf009415g0p
h3tf009415g4s
g3tf009415g2u
h3tf009415g2s
g3tf009415g1s
Lh3tf009415g0p
Lh3tf009415g3s
Sg3tf009415g1s
h3tf009415g1s
0
10
20
30
40
Branch Length
Silk Gland w/ Majority Expression
(Grey=Not 2−Fold Increased in Silk)
Broad
Maj
Is Dupliction Node?
N
Y
Leaf
Proportion of
Total Expression
0.6
0.4
0.2
0.0
Expression Order
Of Magnitude
2.0
2.5

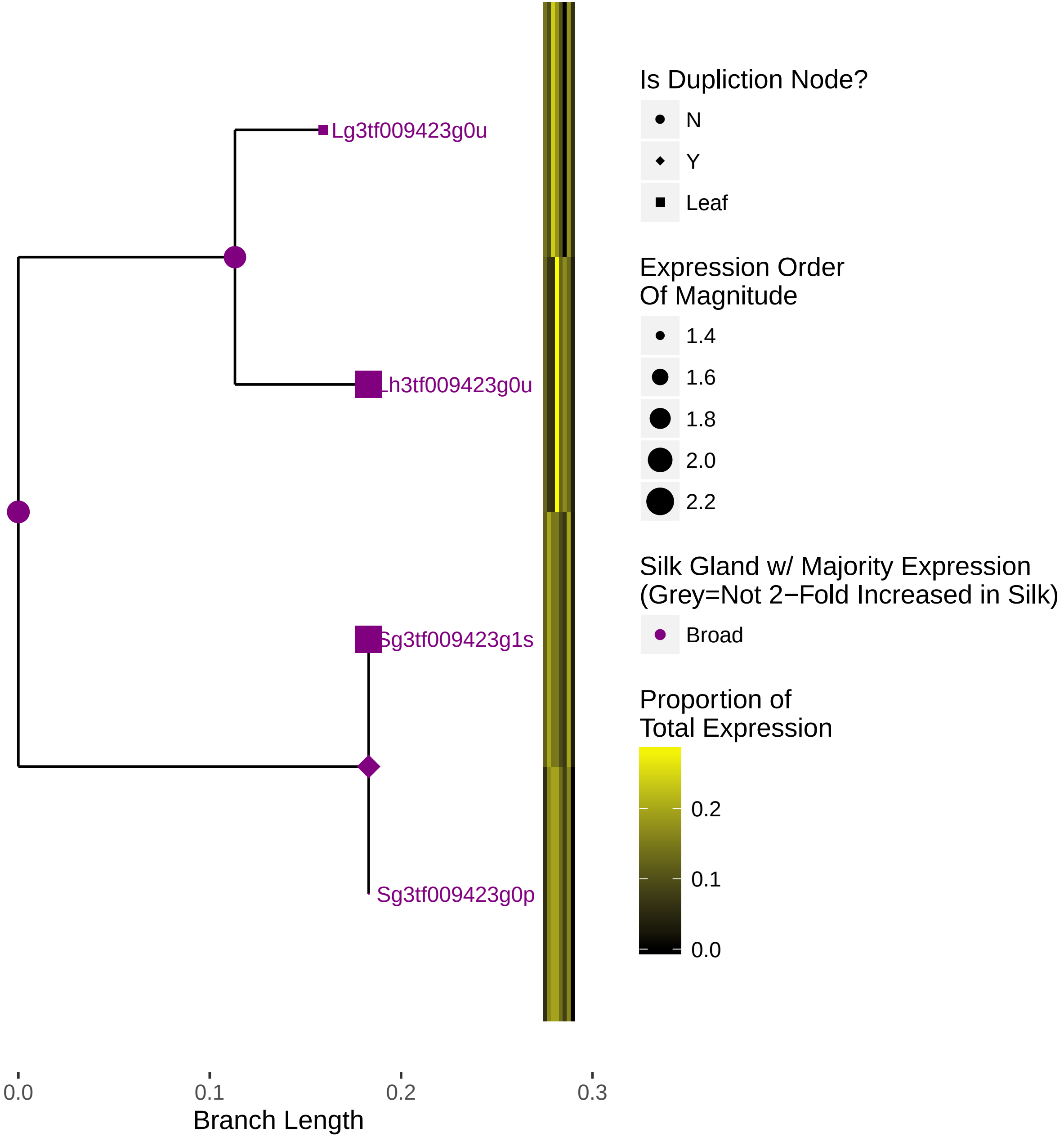

Lg3tf009423g0u
Lh3tf009423g0u
Sg3tf009423g1s
Sg3tf009423g0p
Is Dupliction Node?
N
Y
Leaf
Expression Order
Of Magnitude
1.4
1.6
1.8
2.0
2.2
Silk Gland w/ Majority Expression
(Grey=Not 2−Fold Increased in Silk)
Broad
Proportion of
Total Expression
0.2
0.1
0.0
0.0
0.1
0.2
0.3
Branch Length

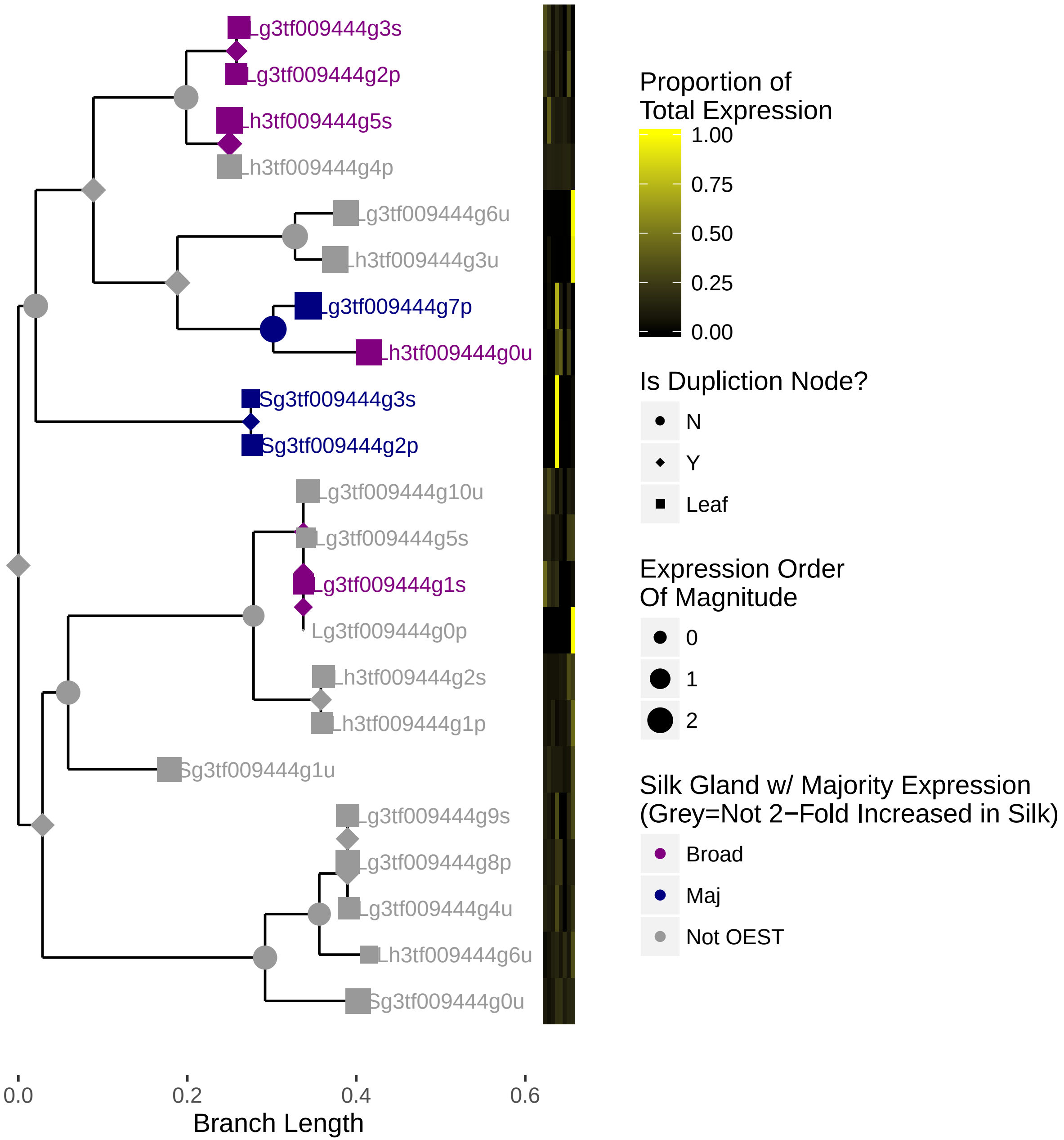

Lg3tf009444g3s
Lg3tf009444g2p
Lh3tf009444g5s
Lh3tf009444g4p
Lg3tf009444g6u
Lh3tf009444g3u
Lg3tf009444g7p
Lh3tf009444g0u
Sg3tf009444g3s
Sg3tf009444g2p
Lg3tf009444g10u
Lg3tf009444g5s
Lg3tf009444g1s
Lg3tf009444g0p
Lh3tf009444g2s
Lh3tf009444g1p
Sg3tf009444g1u
Lg3tf009444g9s
Lg3tf009444g8p
Lg3tf009444g4u
Lh3tf009444g6u
Sg3tf009444g0u
0.0
0.2
0.4
0.6
Branch Length
Proportion of Total Expression
1.00
0.75
0.50
0.25
0.00
Is Dupliction Node?
N
Y
Leaf
Expression Order Of Magnitude
0
1
2
Silk Gland w/ Majority Expression (Grey=Not 2−Fold Increased in Silk)
Broad
Maj
Not OEST

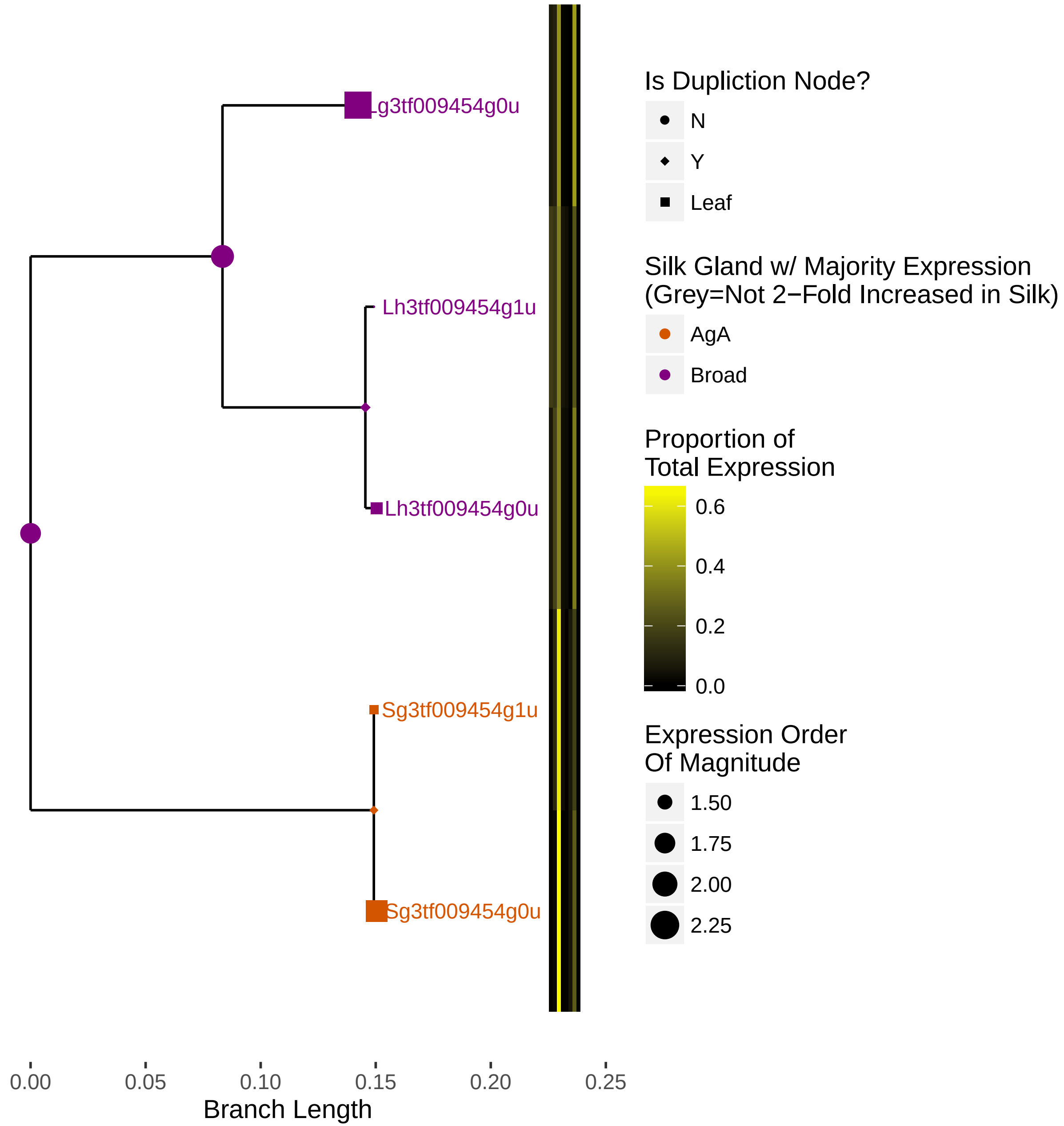

Lg3tf009454g0u
Lh3tf009454g1u
Lh3tf009454g0u
Sg3tf009454g1u
Sg3tf009454g0u
Is Dupliction Node?
N
Y
Leaf
Silk Gland w/ Majority Expression
(Grey=Not 2−Fold Increased in Silk)
AgA
Broad
Proportion of
Total Expression
0.6
0.4
0.2
0.0
Expression Order
Of Magnitude
1.50
1.75
2.00
2.25
0.00
0.05
0.10
0.15
0.20
0.25
Branch Length

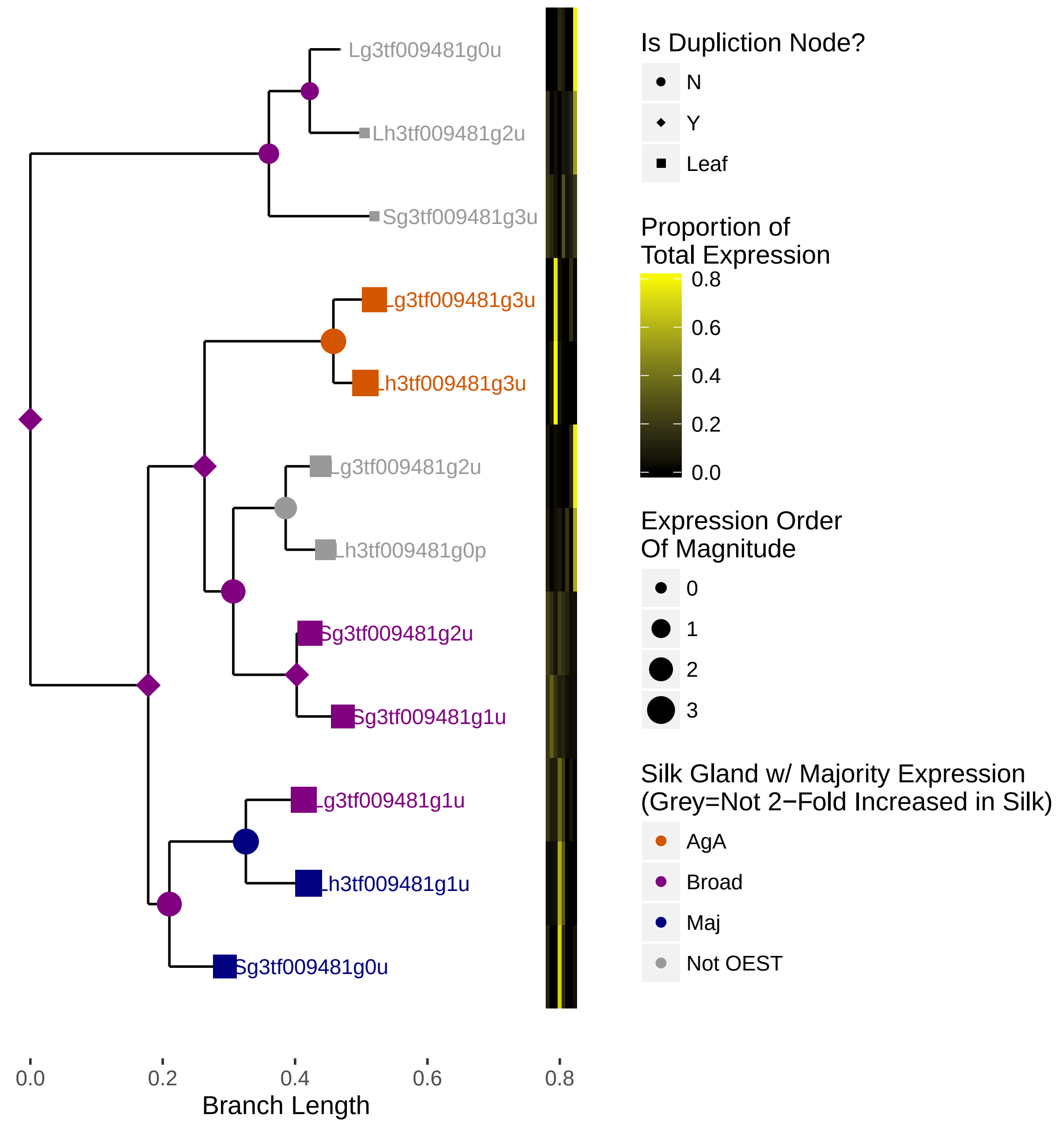

Lg3tf009481g0u
Lh3tf009481g2u
Sg3tf009481g3u
Lg3tf009481g3u
Lh3tf009481g3u
Lg3tf009481g2u
Lh3tf009481g0p
Sg3tf009481g2u
Sg3tf009481g1u
Lg3tf009481g1u
Lh3tf009481g1u
Sg3tf009481g0u
0.0
0.2
0.4
0.6
0.8
Branch Length
Is Dupliction Node?
N
Y
Leaf
Proportion of
Total Expression
0.8
0.6
0.4
0.2
0.0
Expression Order
Of Magnitude
0
1
2
3
Silk Gland w/ Majority Expression
(Grey=Not 2−Fold Increased in Silk)
AgA
Broad
Maj
Not OEST

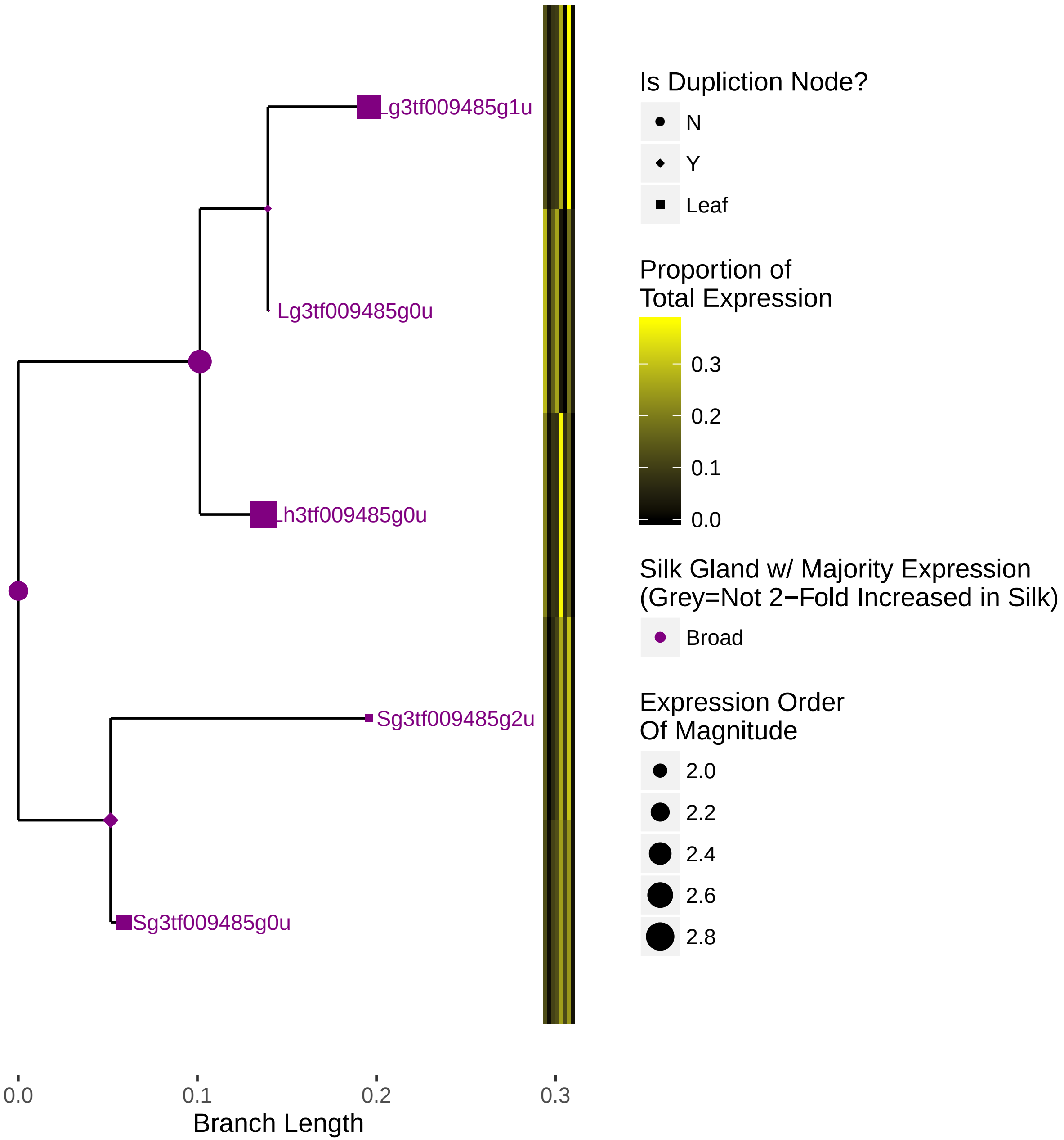
Lg3tf009485g1u
Lg3tf009485g0u
Lh3tf009485g0u
Sg3tf009485g2u
Sg3tf009485g0u
0.0
0.1
0.2
0.3
Branch Length
Is Dupliction Node?
N
Y
Leaf
Proportion of
Total Expression
0.3
0.2
0.1
0.0
Silk Gland w/ Majority Expression
(Grey=Not 2−Fold Increased in Silk)
Broad
Expression Order
Of Magnitude
2.0
2.2
2.4
2.6
2.8

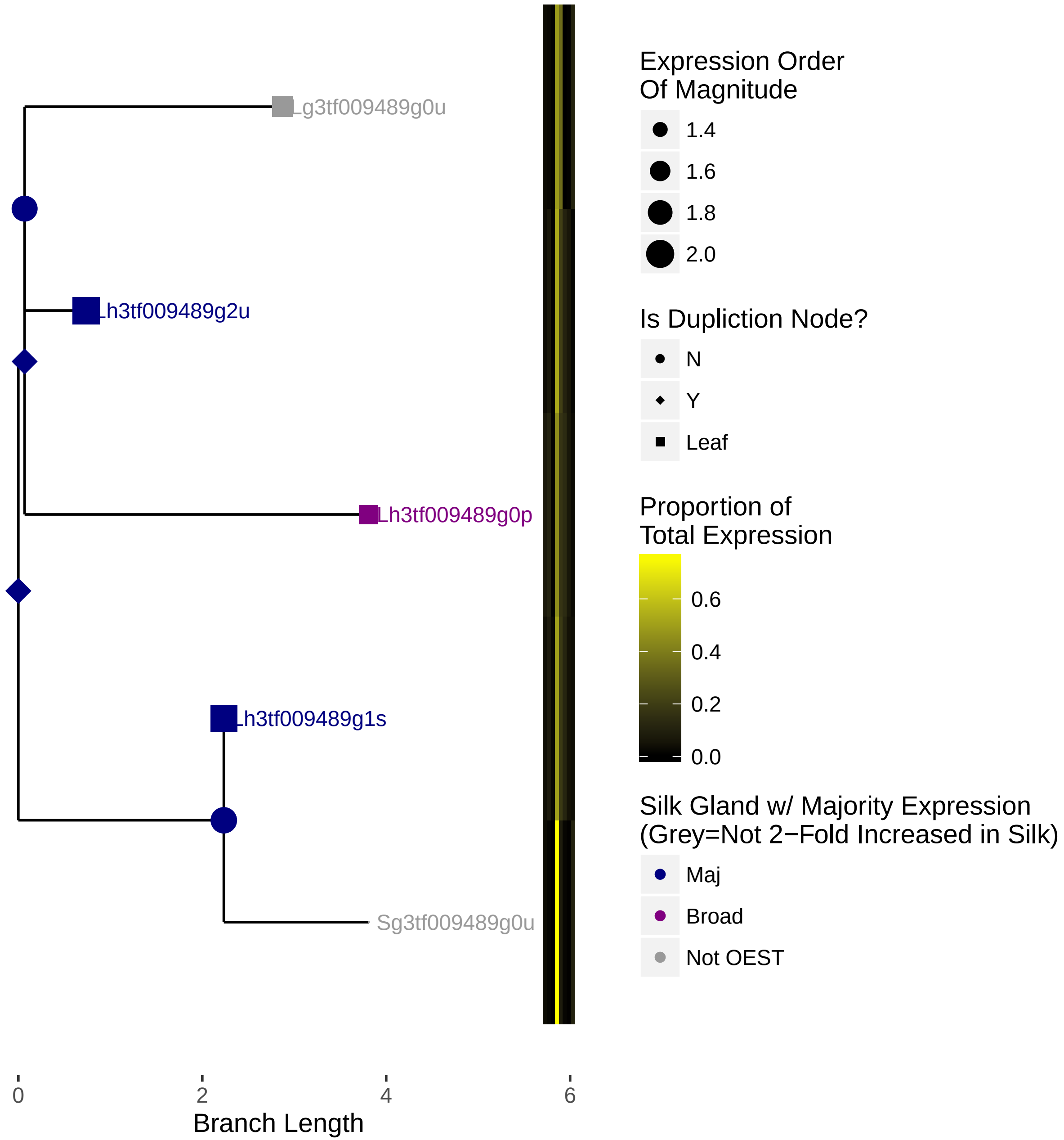

Lg3tf009489g0u
Lh3tf009489g2u
Lh3tf009489g0p
Lh3tf009489g1s
Sg3tf009489g0u
0
2
4
6
Branch Length
Expression Order
Of Magnitude
1.4
1.6
1.8
2.0
Is Dupliction Node?
N
Y
Leaf
Proportion of
Total Expression
0.6
0.4
0.2
0.0
Silk Gland w/ Majority Expression
(Grey=Not 2−Fold Increased in Silk)
Maj
Broad
Not OEST

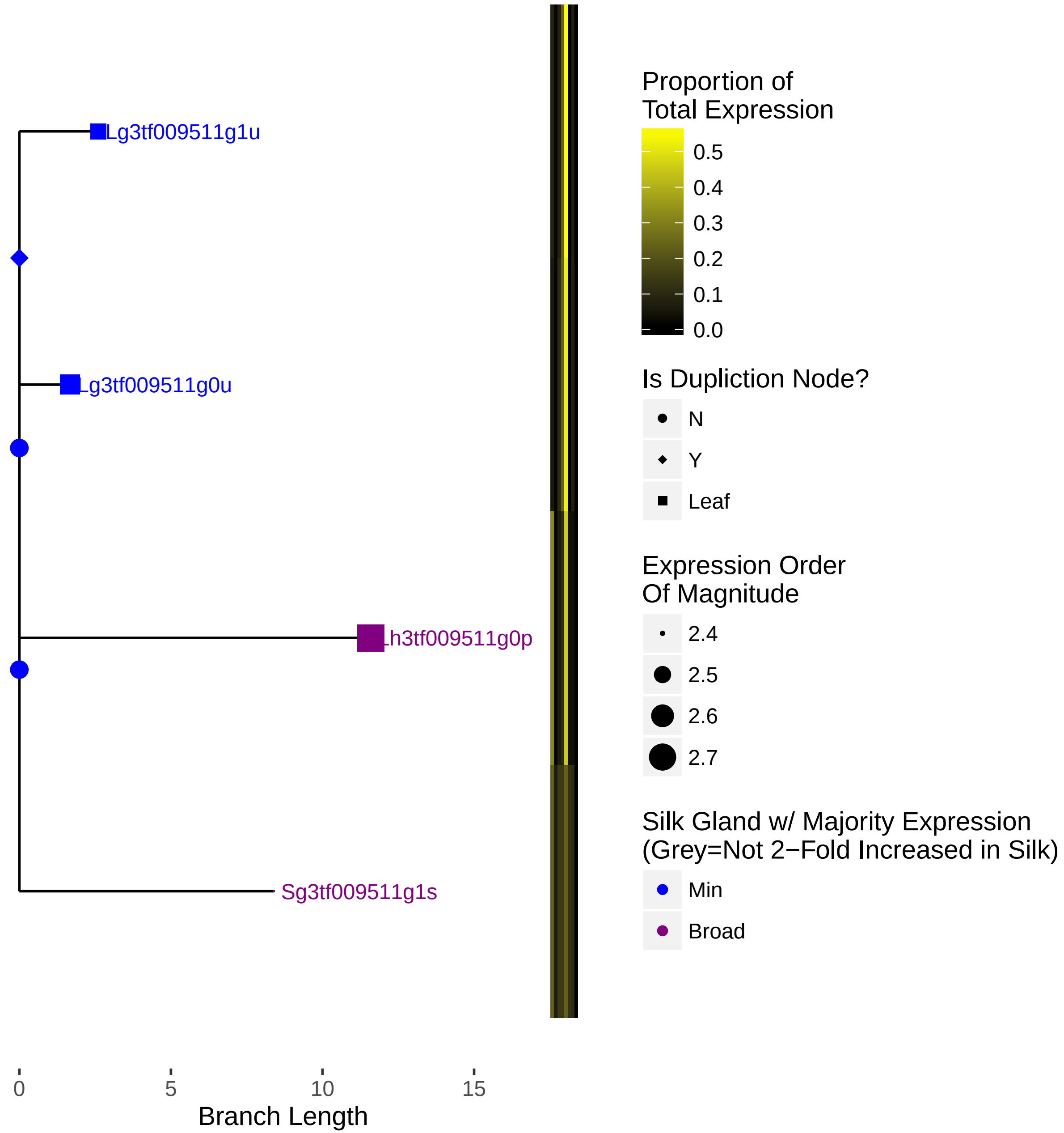

Lg3tf009511g1u
Lg3tf009511g0u
Lh3tf009511g0p
Sg3tf009511g1s
0
5
10
15
Branch Length
Proportion of
Total Expression
0.5
0.4
0.3
0.2
0.1
0.0
Is Dupliction Node?
N
Y
Leaf
Expression Order
Of Magnitude
2.4
2.5
2.6
2.7
Silk Gland w/ Majority Expression
(Grey=Not 2−Fold Increased in Silk)
Min
Broad

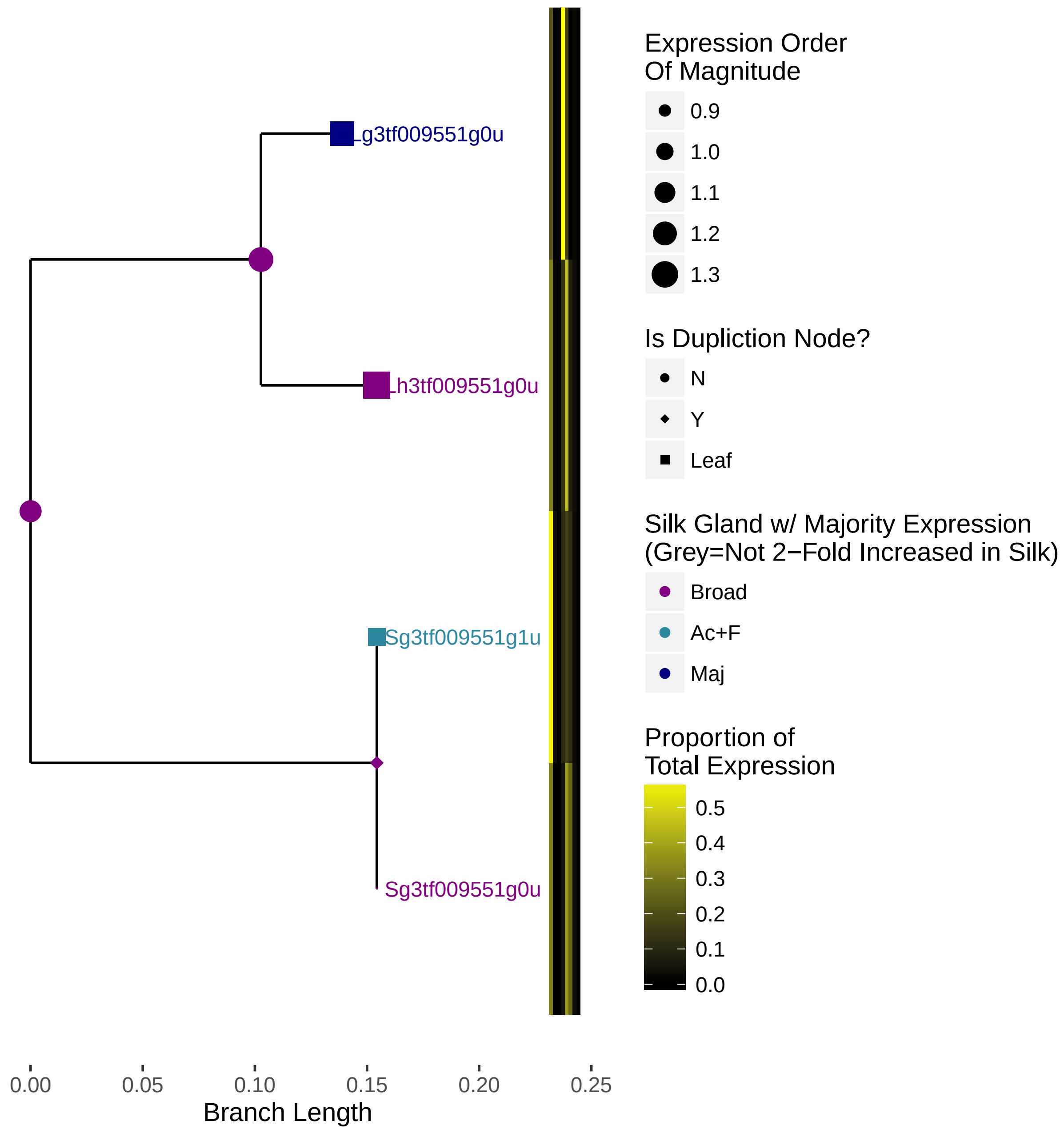

Lg3tf009551g0u
Lh3tf009551g0u
Sg3tf009551g1u
Sg3tf009551g0u
0.00
0.05
0.10
0.15
0.20
0.25
Branch Length
Expression Order
Of Magnitude
0.9
1.0
1.1
1.2
1.3
Is Dupliction Node?
N
Y
Leaf
Silk Gland w/ Majority Expression
(Grey=Not 2−Fold Increased in Silk)
Broad
Ac+F
Maj
Proportion of
Total Expression
0.5
0.4
0.3
0.2
0.1
0.0

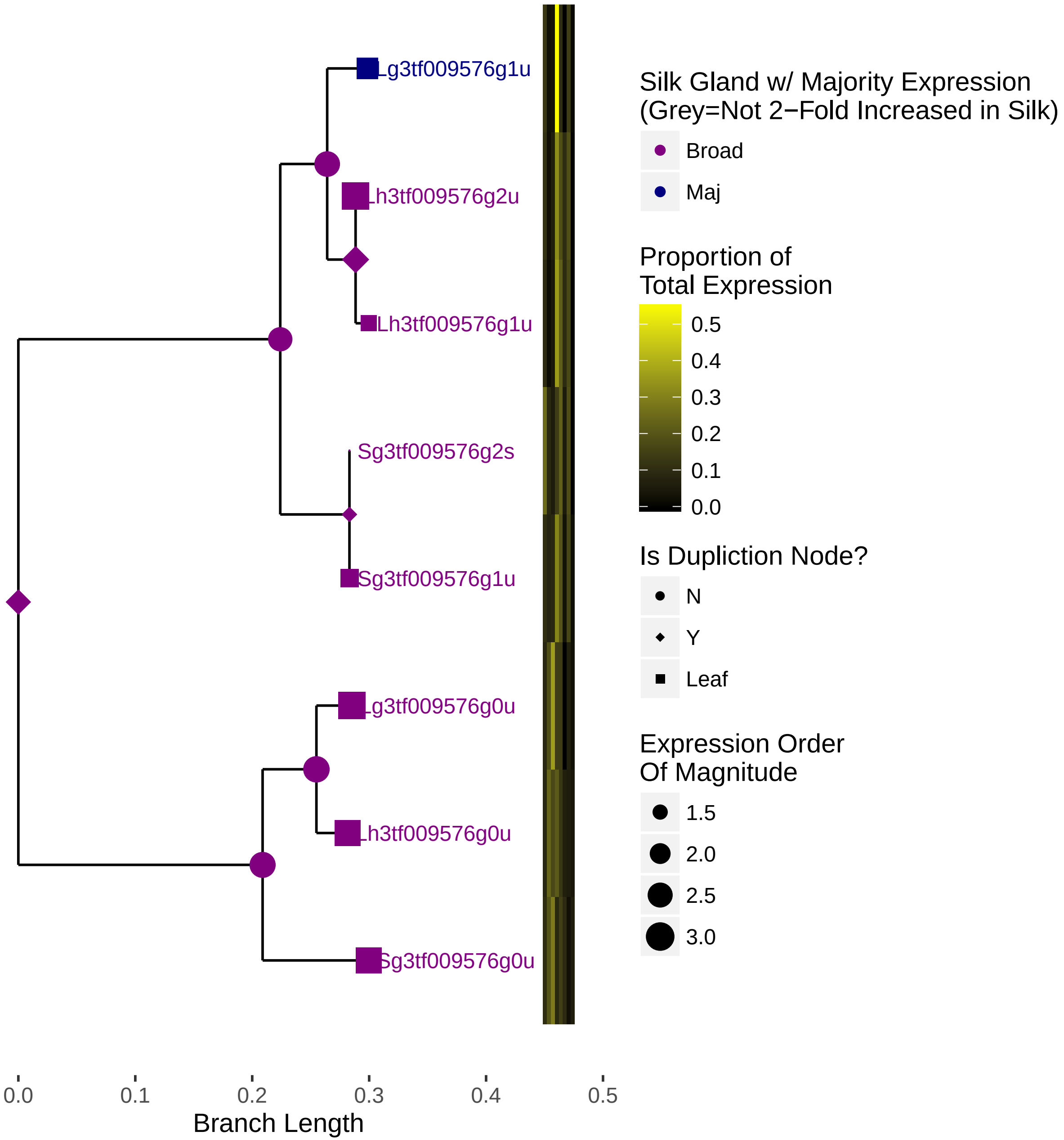

Lg3tf009576g1u
Lh3tf009576g2u
Lh3tf009576g1u
Sg3tf009576g2s
Sg3tf009576g1u
Lg3tf009576g0u
Lh3tf009576g0u
Sg3tf009576g0u
0.0
0.1
0.2
0.3
0.4
0.5
Branch Length
Silk Gland w/ Majority Expression
(Grey=Not 2−Fold Increased in Silk)
Broad
Maj
Proportion of
Total Expression
0.5
0.4
0.3
0.2
0.1
0.0
Is Dupliction Node?
N
Y
Leaf
Expression Order
Of Magnitude
1.5
2.0
2.5
3.0

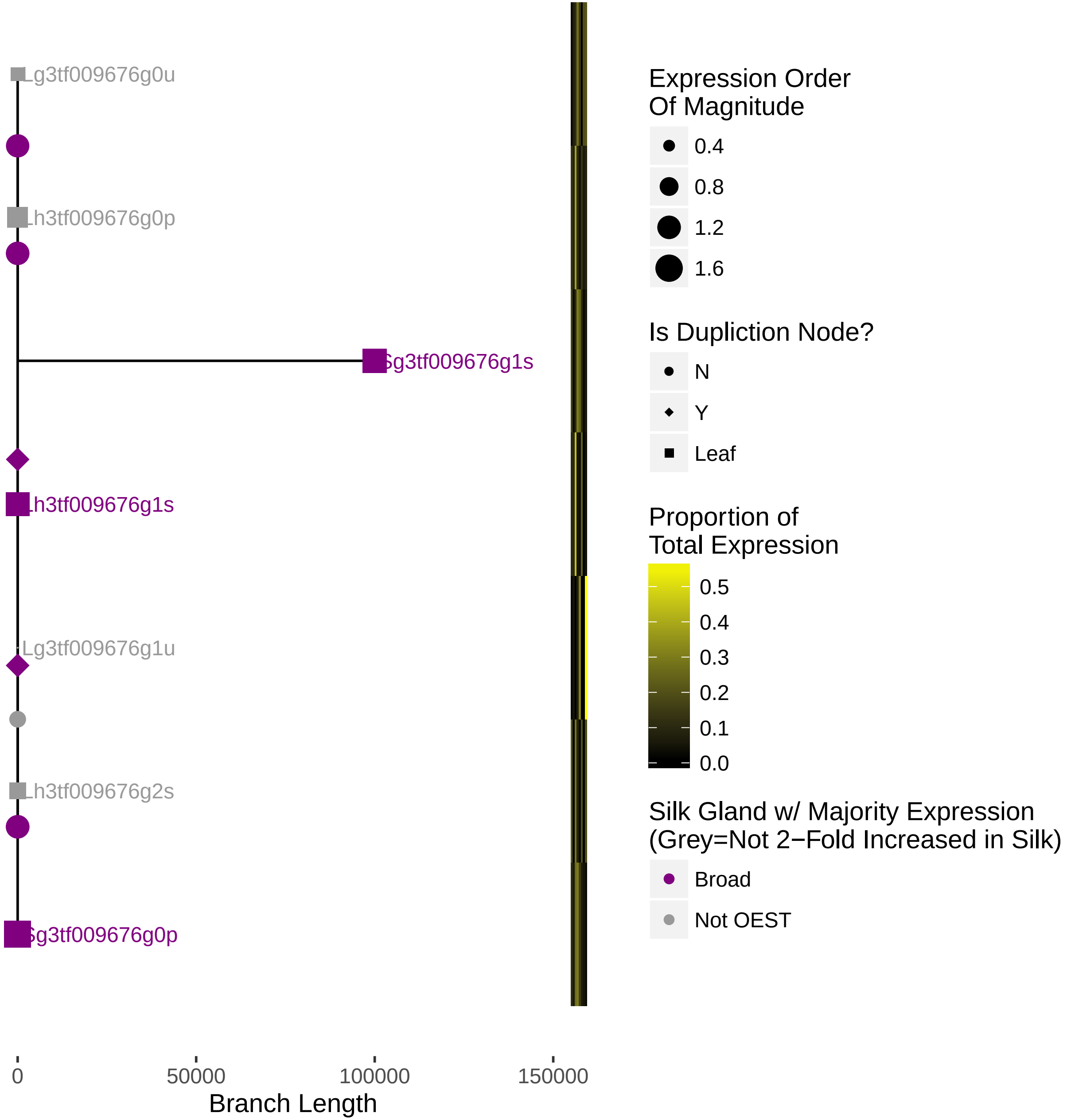

Lg3tf009676g0u
Lh3tf009676g0p
Sg3tf009676g1s
Lh3tf009676g1s
Lg3tf009676g1u
Lh3tf009676g2s
Sg3tf009676g0p
0
50000
100000
150000
Branch Length
Expression Order
Of Magnitude
0.4
0.8
1.2
1.6
Is Dupliction Node?
N
Y
Leaf
Proportion of
Total Expression
0.5
0.4
0.3
0.2
0.1
0.0
Silk Gland w/ Majority Expression
(Grey=Not 2−Fold Increased in Silk)
Broad
Not OEST

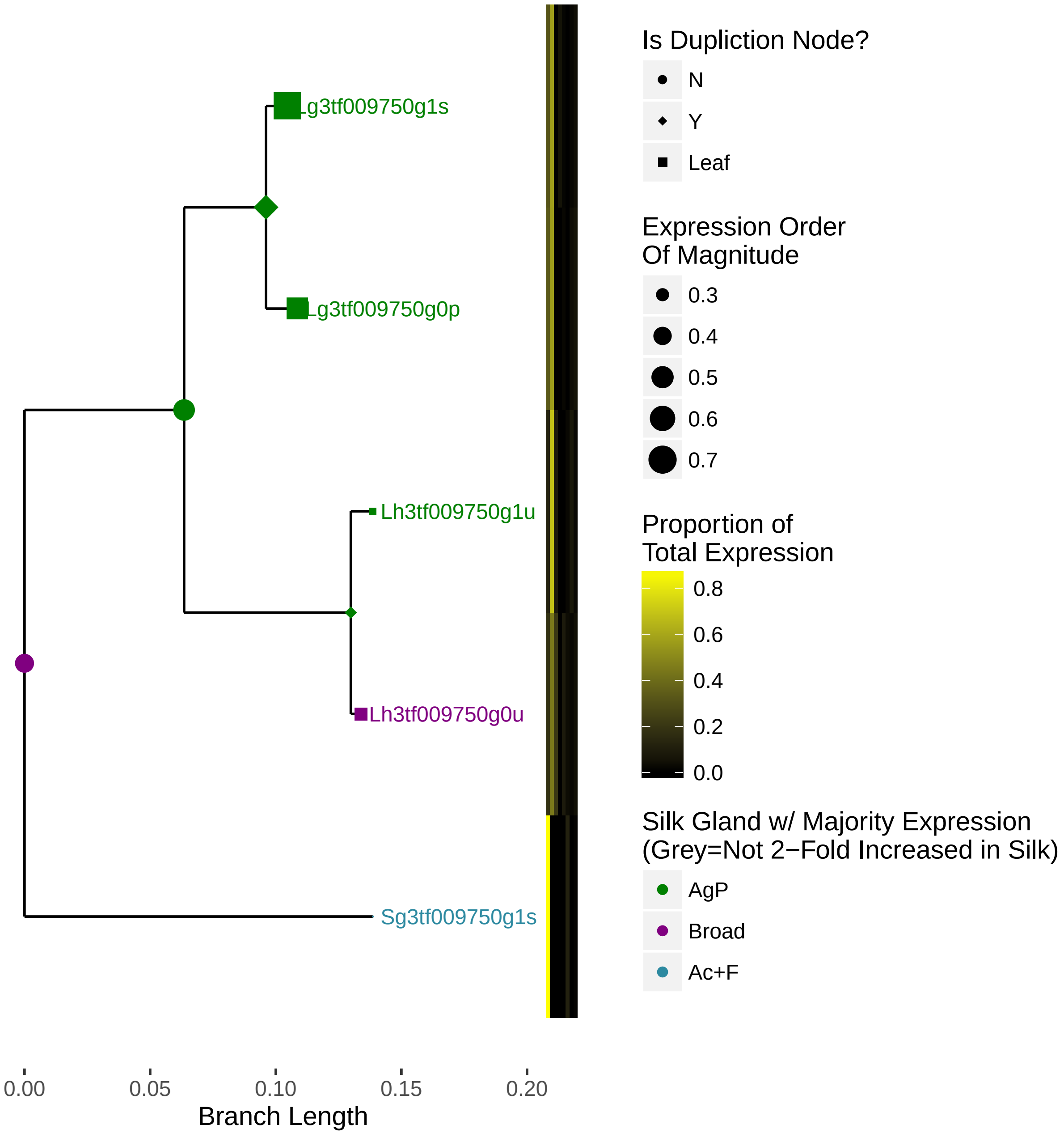
Lg3tf009750g1s
Lg3tf009750g0p
Lh3tf009750g1u
Lh3tf009750g0u
Sg3tf009750g1s
0.00
0.05
0.10
0.15
0.20
Branch Length
Is Dupliction Node?
N
Y
Leaf
Expression Order Of Magnitude
0.3
0.4
0.5
0.6
0.7
Proportion of Total Expression
0.8
0.6
0.4
0.2
0.0
Silk Gland w/ Majority Expression (Grey=Not 2−Fold Increased in Silk)
AgP
Broad
Ac+F

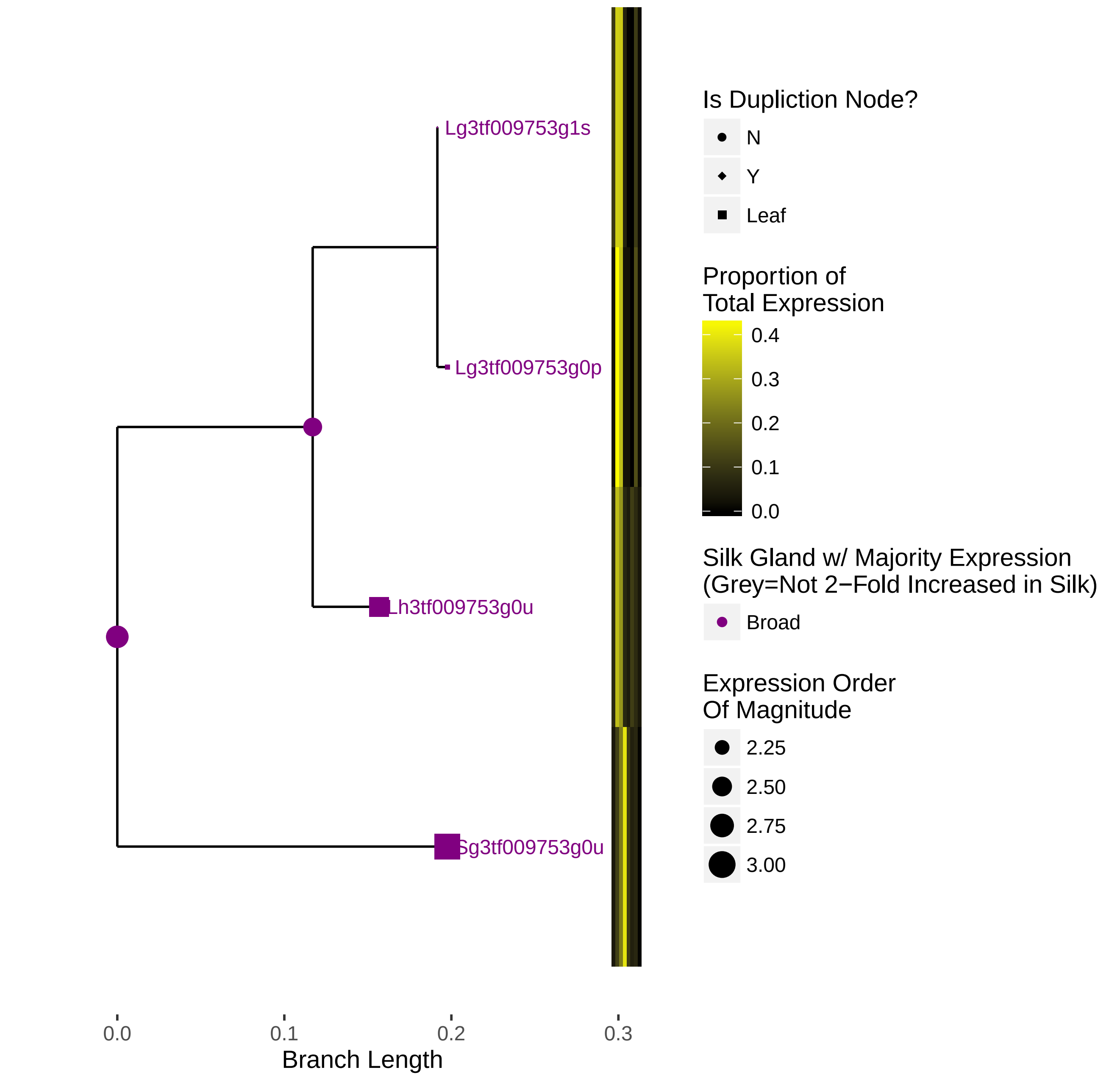

Lg3tf009753g1s
Lg3tf009753g0p
Lh3tf009753g0u
Sg3tf009753g0u
0.0
0.1
0.2
0.3
Branch Length
Is Dupliction Node?
N
Y
Leaf
Proportion of
Total Expression
0.4
0.3
0.2
0.1
0.0
Silk Gland w/ Majority Expression
(Grey=Not 2−Fold Increased in Silk)
Broad
Expression Order
Of Magnitude
2.25
2.50
2.75
3.00

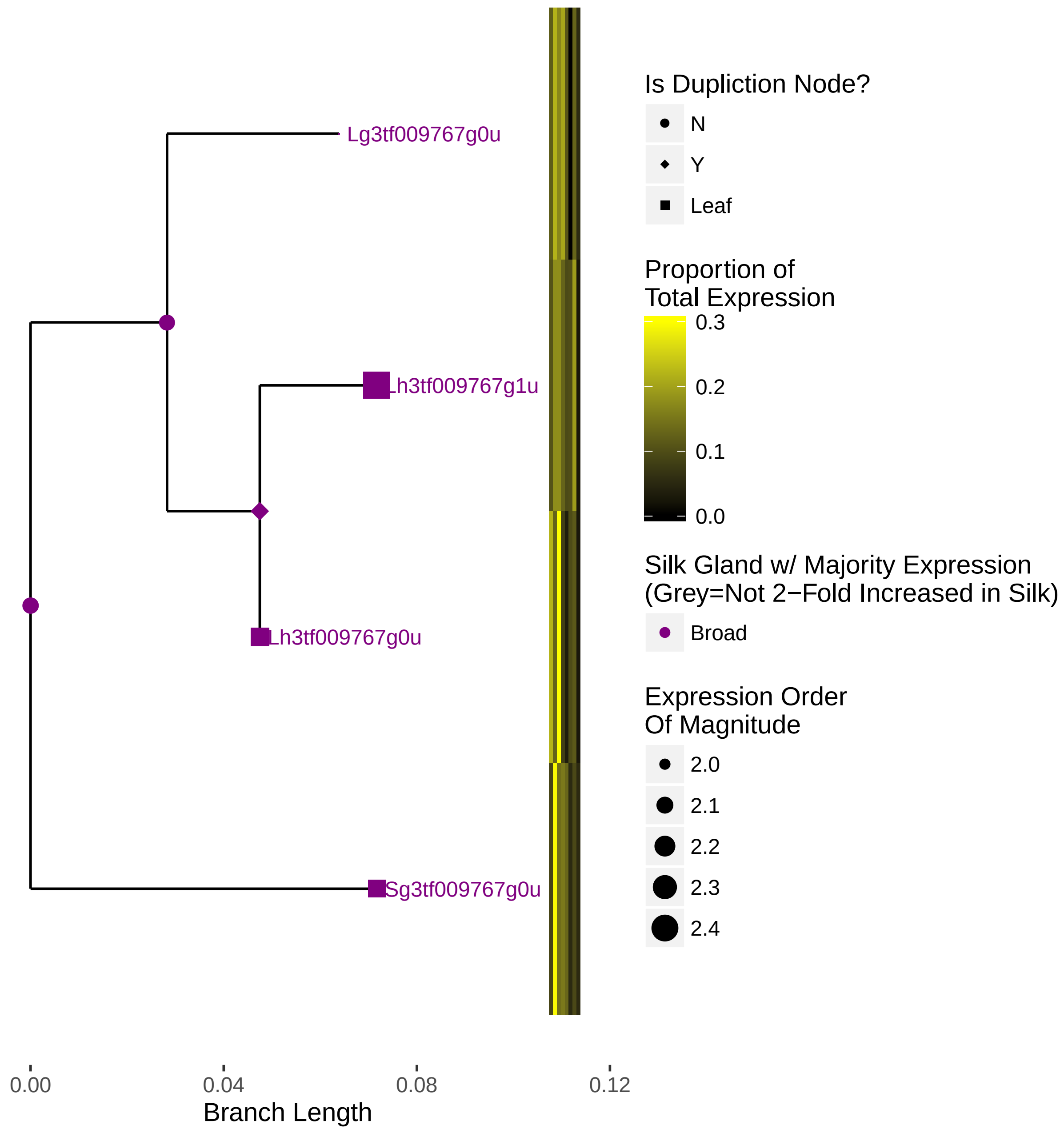

Lg3tf009767g0u
Lh3tf009767g1u
Lh3tf009767g0u
Sg3tf009767g0u
Is Dupliction Node?
N
Y
Leaf
Proportion of
Total Expression
0.3
0.2
0.1
0.0
Silk Gland w/ Majority Expression
(Grey=Not 2−Fold Increased in Silk)
Broad
Expression Order
Of Magnitude
2.0
2.1
2.2
2.3
2.4
0.00
0.04
0.08
0.12
Branch Length

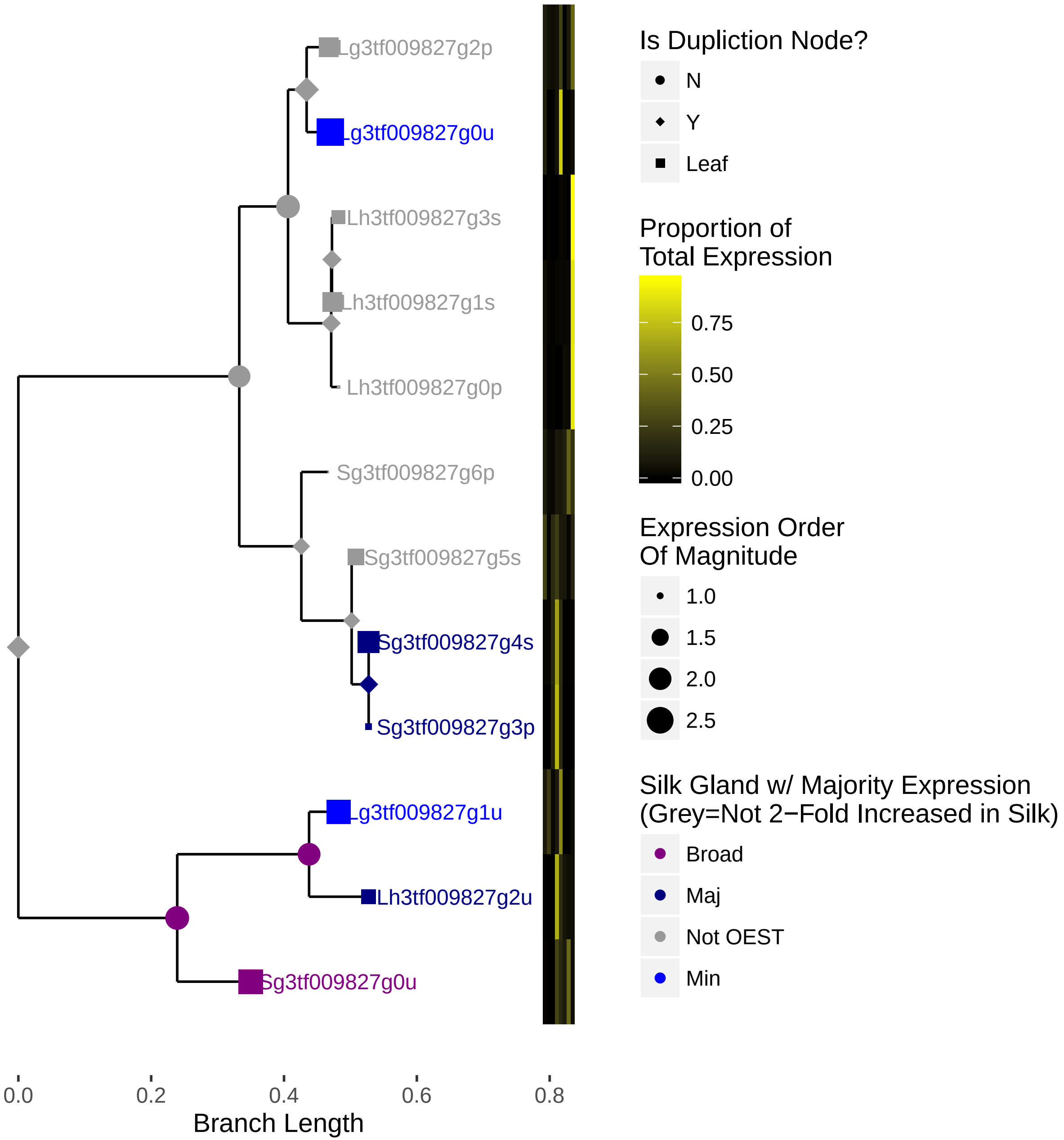

Lg3tf009827g2p
Lg3tf009827g0u
Lh3tf009827g3s
Lh3tf009827g1s
Lh3tf009827g0p
Sg3tf009827g6p
Sg3tf009827g5s
Sg3tf009827g4s
Sg3tf009827g3p
Lg3tf009827g1u
Lh3tf009827g2u
Sg3tf009827g0u
0.0
0.2
0.4
0.6
0.8
Branch Length
Is Dupliction Node?
N
Y
Leaf
Proportion of
Total Expression
0.75
0.50
0.25
0.00
Expression Order
Of Magnitude
1.0
1.5
2.0
2.5
Silk Gland w/ Majority Expression
(Grey=Not 2−Fold Increased in Silk)
Broad
Maj
Not OEST
Min

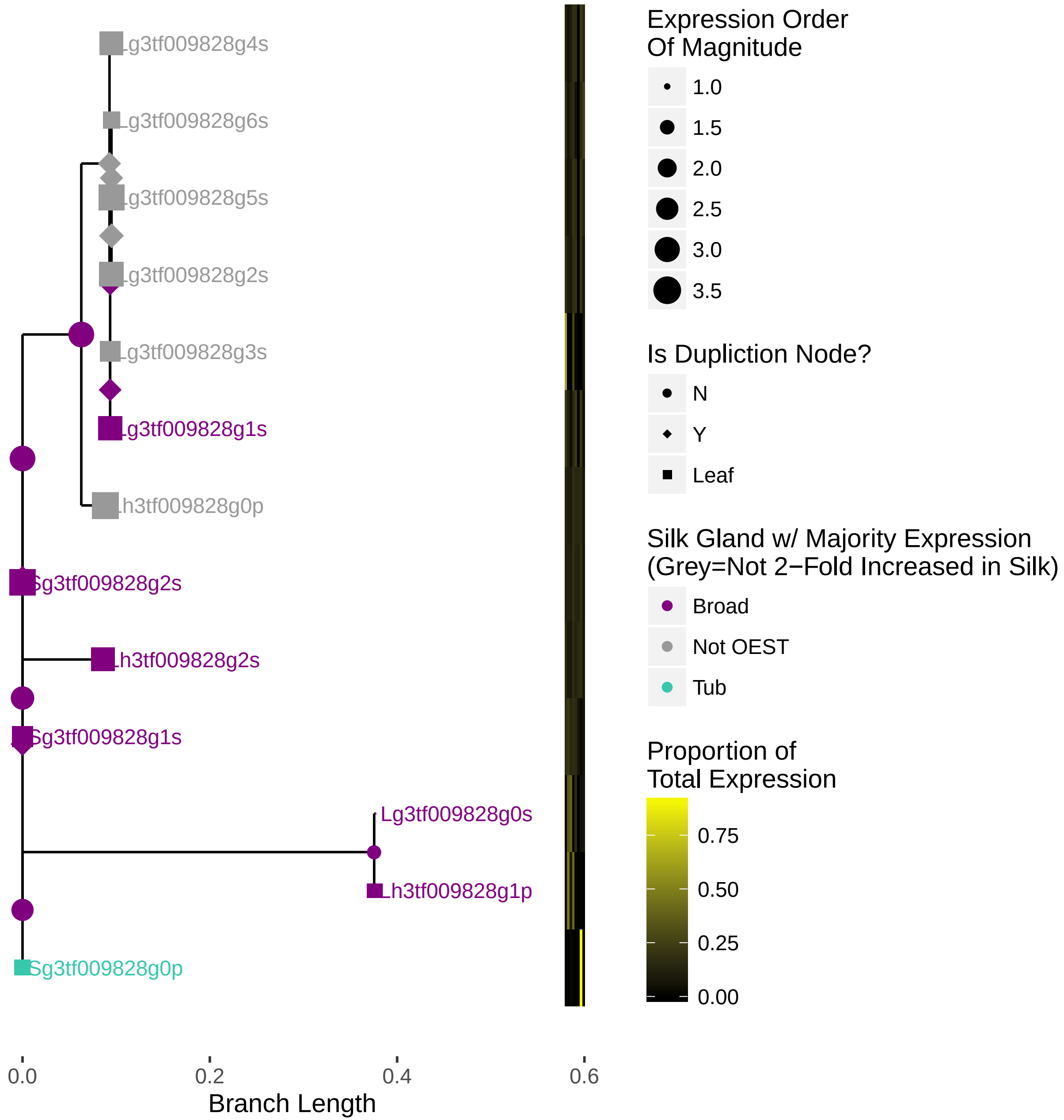

Lg3tf009828g4s
Lg3tf009828g6s
Lg3tf009828g5s
Lg3tf009828g2s
Lg3tf009828g3s
Lg3tf009828g1s
Lh3tf009828g0p
Sg3tf009828g2s
Lh3tf009828g2s
Sg3tf009828g1s
Lg3tf009828g0s
Lh3tf009828g1p
Sg3tf009828g0p
0.0
0.2
0.4
0.6
Branch Length
Expression Order
Of Magnitude
1.0
1.5
2.0
2.5
3.0
3.5
Is Dupliction Node?
N
Y
Leaf
Silk Gland w/ Majority Expression
(Grey=Not 2−Fold Increased in Silk)
Broad
Not OEST
Tub
Proportion of
Total Expression
0.75
0.50
0.25
0.00

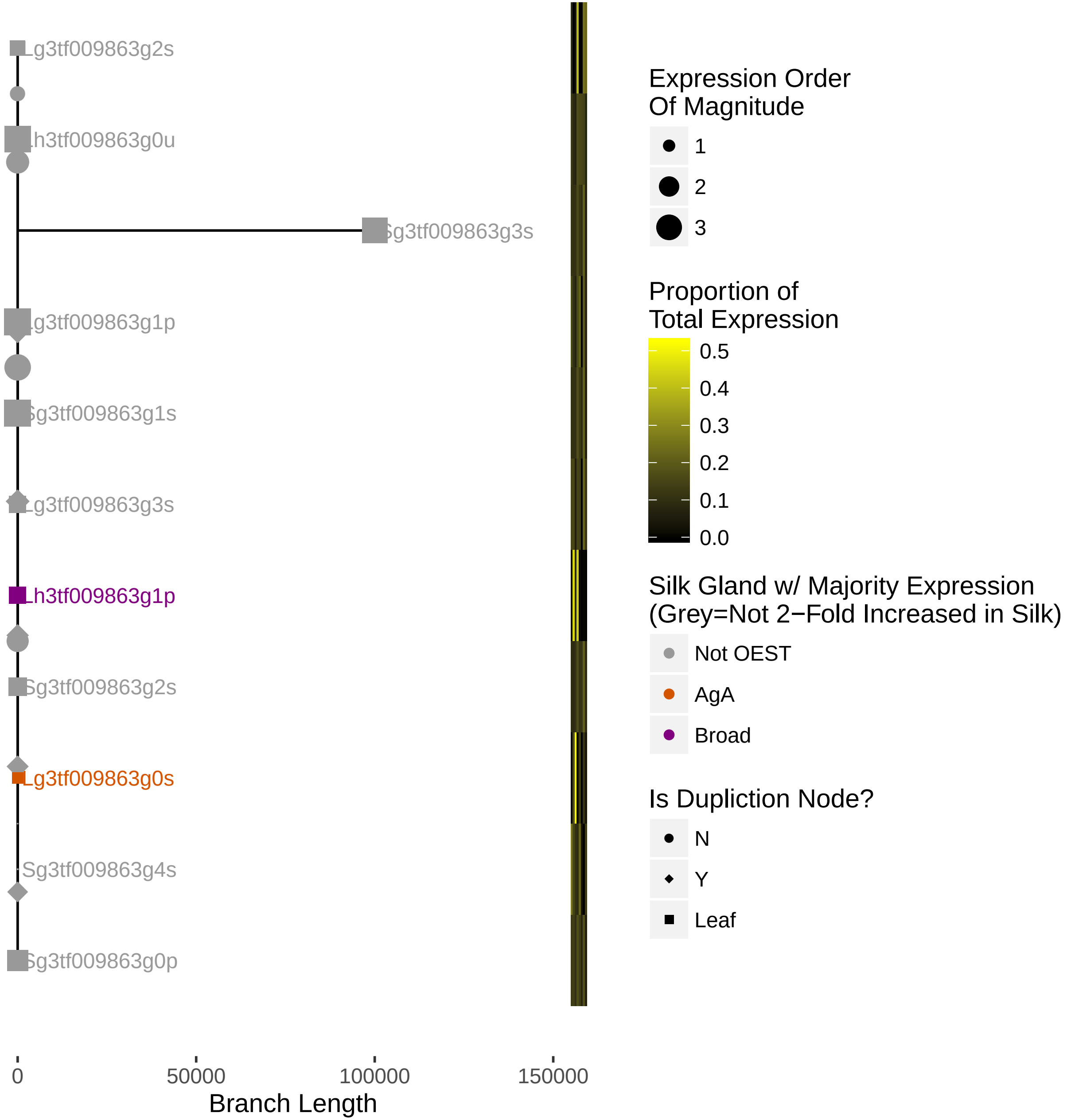

Lg3tf009863g2s
Lh3tf009863g0u
Sg3tf009863g3s
Lg3tf009863g1p
Sg3tf009863g1s
Lg3tf009863g3s
Lh3tf009863g1p
Sg3tf009863g2s
Lg3tf009863g0s
Sg3tf009863g4s
Sg3tf009863g0p
0
50000
100000
150000
Branch Length
Expression Order
Of Magnitude
1
2
3
Proportion of
Total Expression
0.5
0.4
0.3
0.2
0.1
0.0
Silk Gland w/ Majority Expression
(Grey=Not 2−Fold Increased in Silk)
Not OEST
AgA
Broad
Is Dupliction Node?
N
Y
Leaf

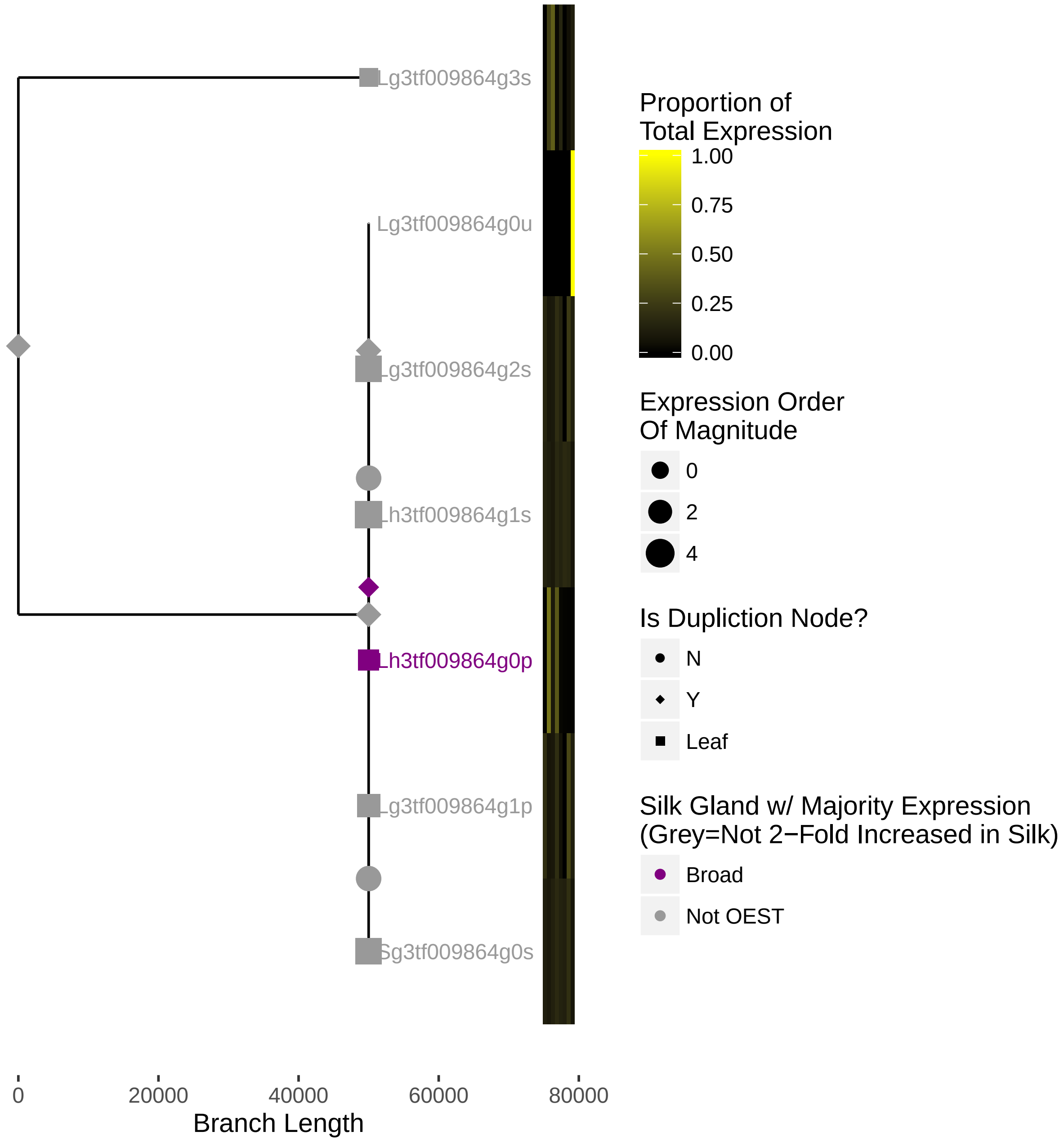

Lg3tf009864g3s
Lg3tf009864g0u
Lg3tf009864g2s
Lh3tf009864g1s
Lh3tf009864g0p
Lg3tf009864g1p
Sg3tf009864g0s
0
20000
40000
60000
80000
Branch Length
Proportion of
Total Expression
1.00
0.75
0.50
0.25
0.00
Expression Order
Of Magnitude
0
2
4
Is Dupliction Node?
N
Y
Leaf
Silk Gland w/ Majority Expression
(Grey=Not 2−Fold Increased in Silk)
Broad
Not OEST

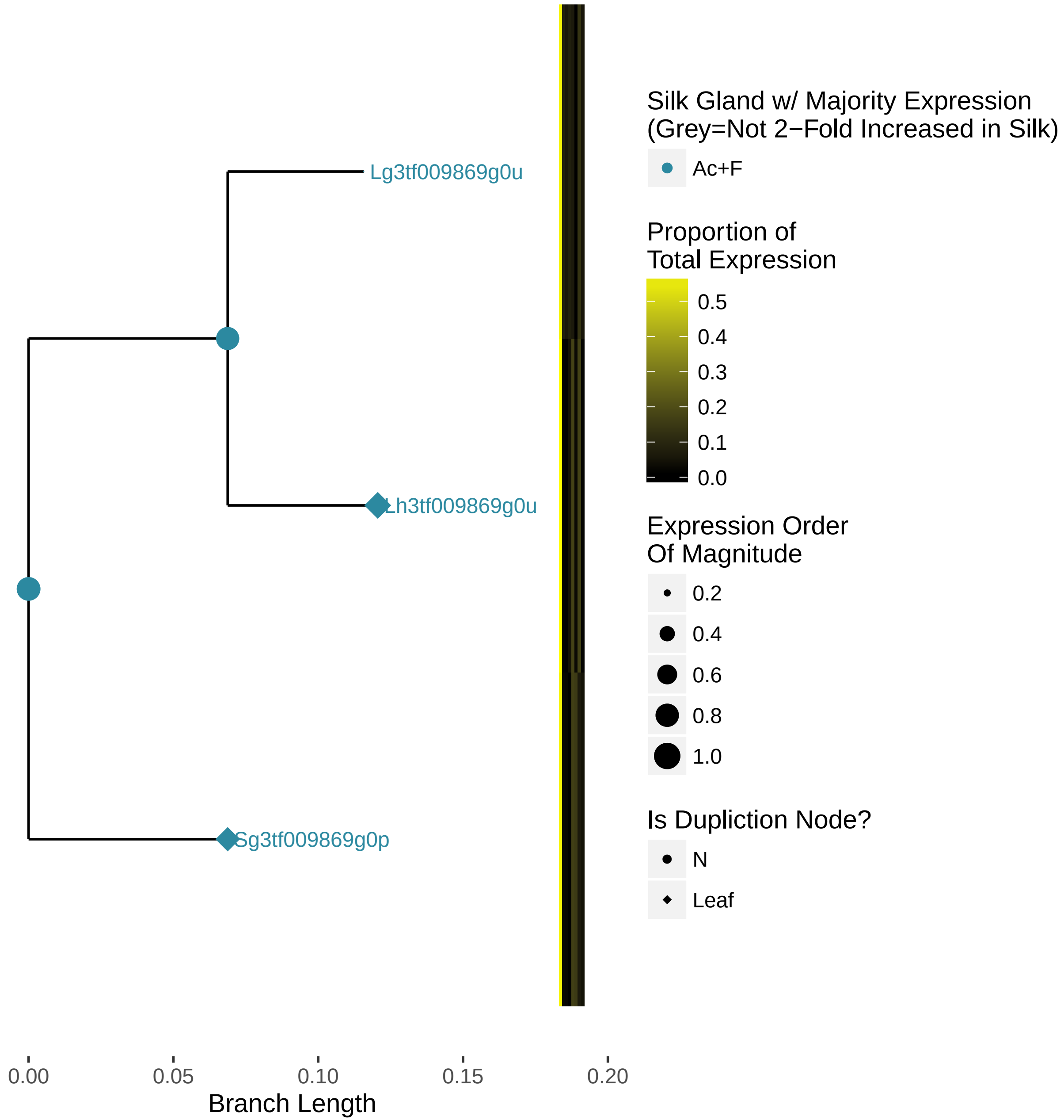

Lg3tf009869g0u
Lh3tf009869g0u
Sg3tf009869g0p
Silk Gland w/ Majority Expression
(Grey=Not 2−Fold Increased in Silk)
Ac+F
Proportion of
Total Expression
0.5
0.4
0.3
0.2
0.1
0.0
Expression Order
Of Magnitude
0.2
0.4
0.6
0.8
1.0
Is Dupliction Node?
N
Leaf
0.00
0.05
0.10
0.15
0.20
Branch Length

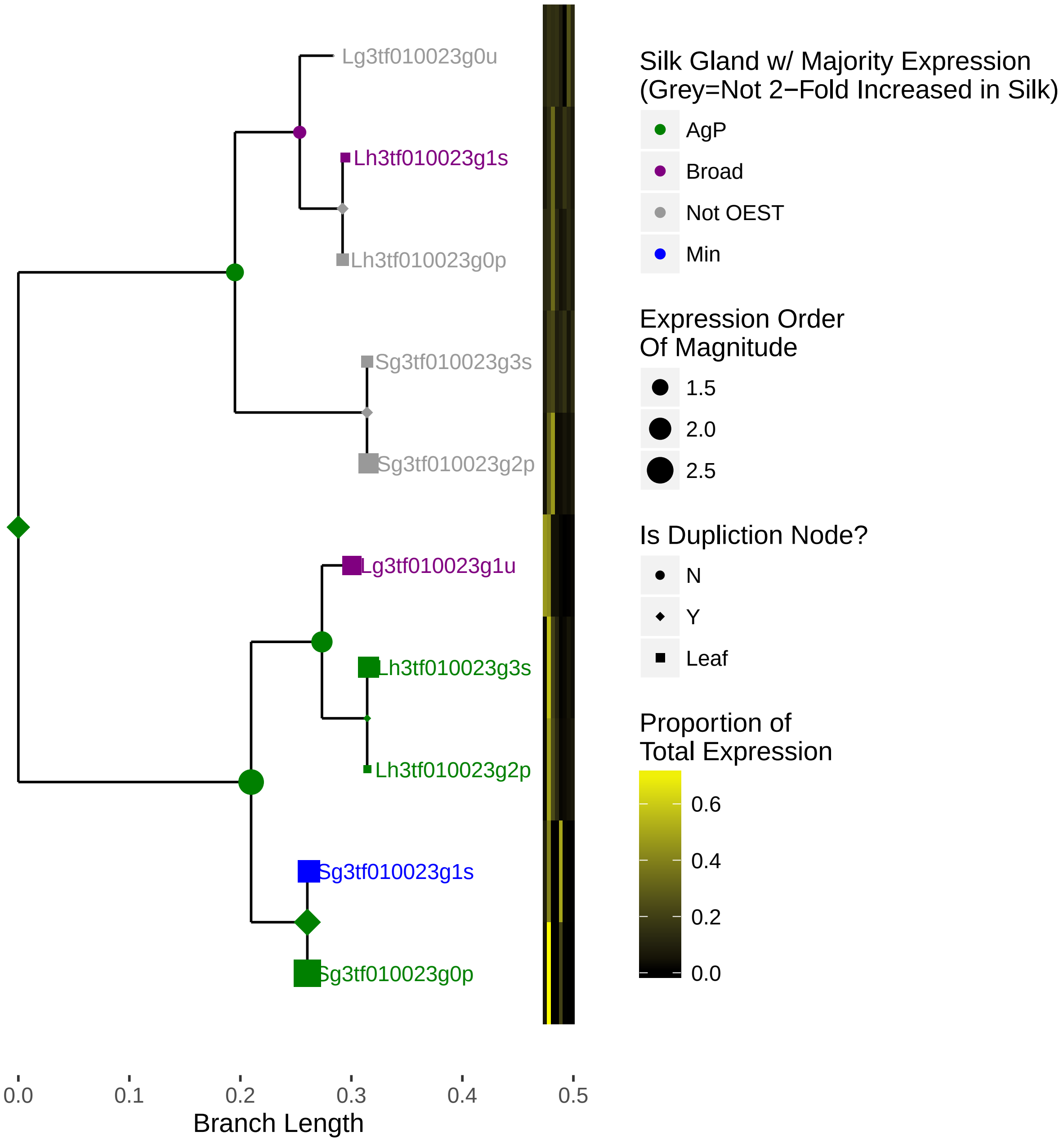

Lg3tf010023g0u
Lh3tf010023g1s
Lh3tf010023g0p
Sg3tf010023g3s
Sg3tf010023g2p
Lg3tf010023g1u
Lh3tf010023g3s
Lh3tf010023g2p
Sg3tf010023g1s
Sg3tf010023g0p
0.0
0.1
0.2
0.3
0.4
0.5
Branch Length
Silk Gland w/ Majority Expression
(Grey=Not 2−Fold Increased in Silk)
AgP
Broad
Not OEST
Min
Expression Order
Of Magnitude
1.5
2.0
2.5
Is Dupliction Node?
N
Y
Leaf
Proportion of
Total Expression
0.6
0.4
0.2
0.0

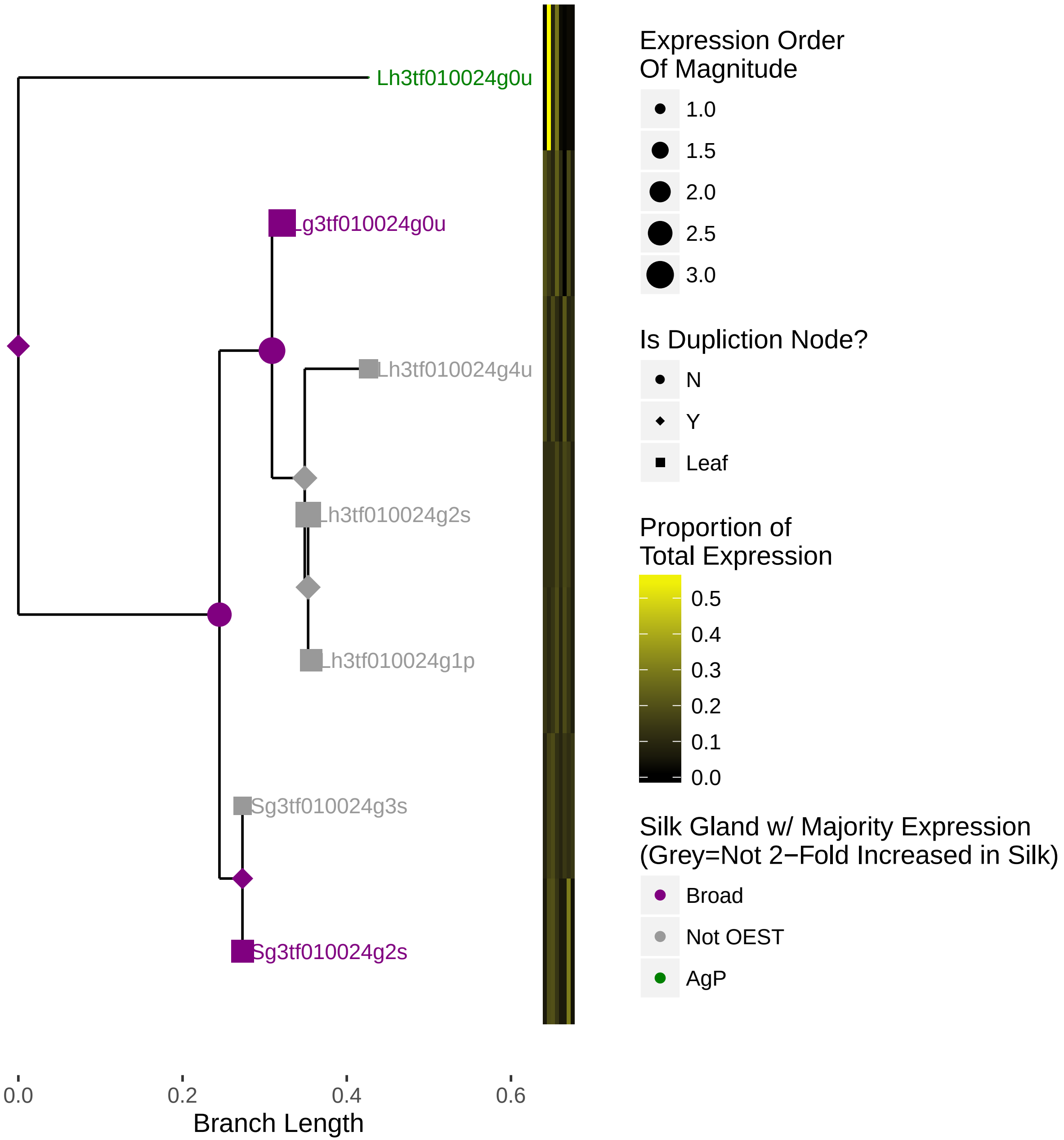

Lh3tf010024g0u
Lg3tf010024g0u
Lh3tf010024g4u
Lh3tf010024g2s
Lh3tf010024g1p
Sg3tf010024g3s
Sg3tf010024g2s
0.0
0.2
0.4
0.6
Branch Length
Expression Order
Of Magnitude
1.0
1.5
2.0
2.5
3.0
Is Dupliction Node?
N
Y
Leaf
Proportion of
Total Expression
0.5
0.4
0.3
0.2
0.1
0.0
Silk Gland w/ Majority Expression
(Grey=Not 2−Fold Increased in Silk)
Broad
Not OEST
AgP

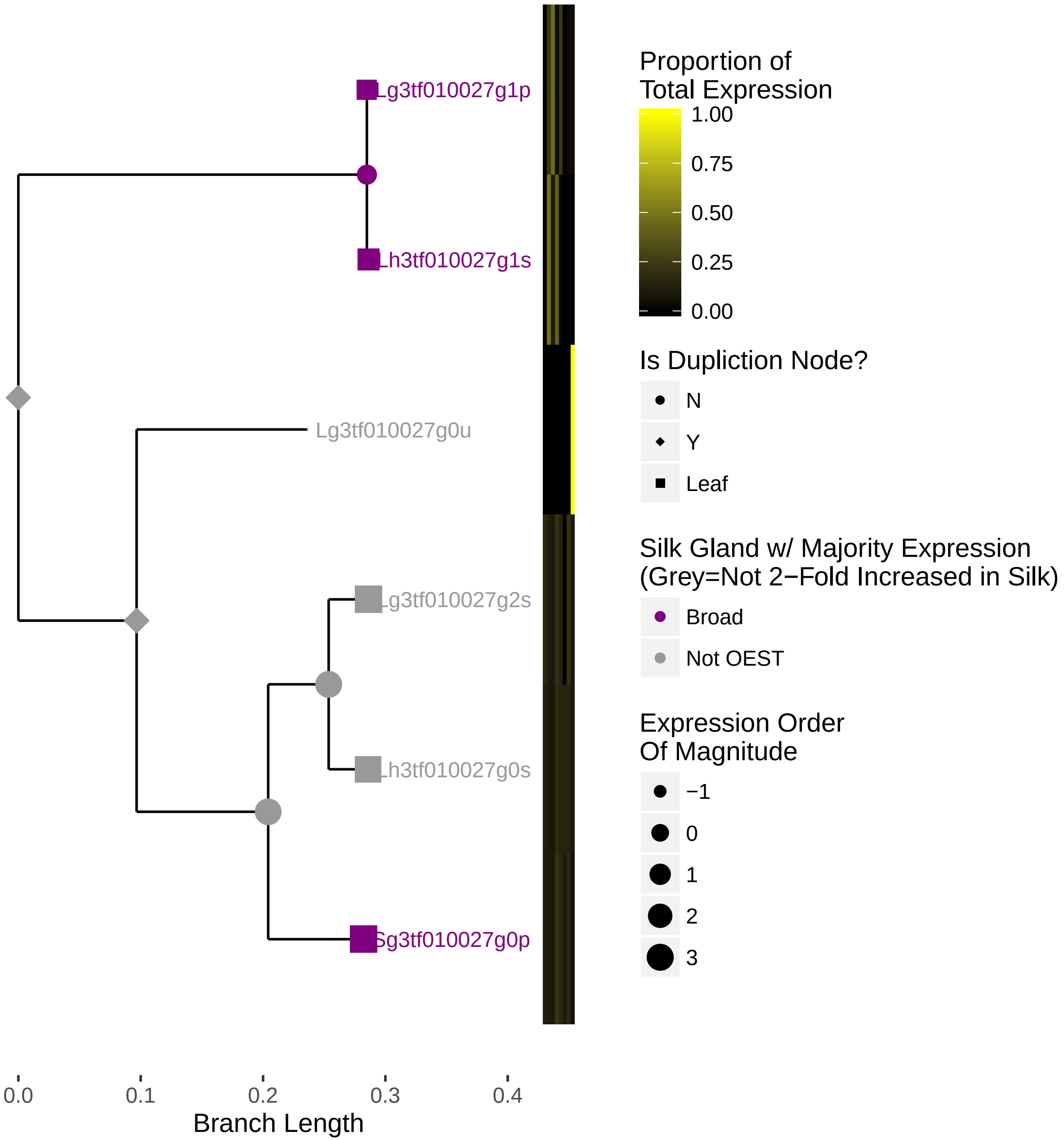

Lg3tf010027g1p
Lh3tf010027g1s
Lg3tf010027g0u
Lg3tf010027g2s
Lh3tf010027g0s
Sg3tf010027g0p
0.0
0.1
0.2
0.3
0.4
Branch Length
Proportion of
Total Expression
1.00
0.75
0.50
0.25
0.00
Is Dupliction Node?
N
Y
Leaf
Silk Gland w/ Majority Expression
(Grey=Not 2−Fold Increased in Silk)
Broad
Not OEST
Expression Order
Of Magnitude
−1
0
1
2
3

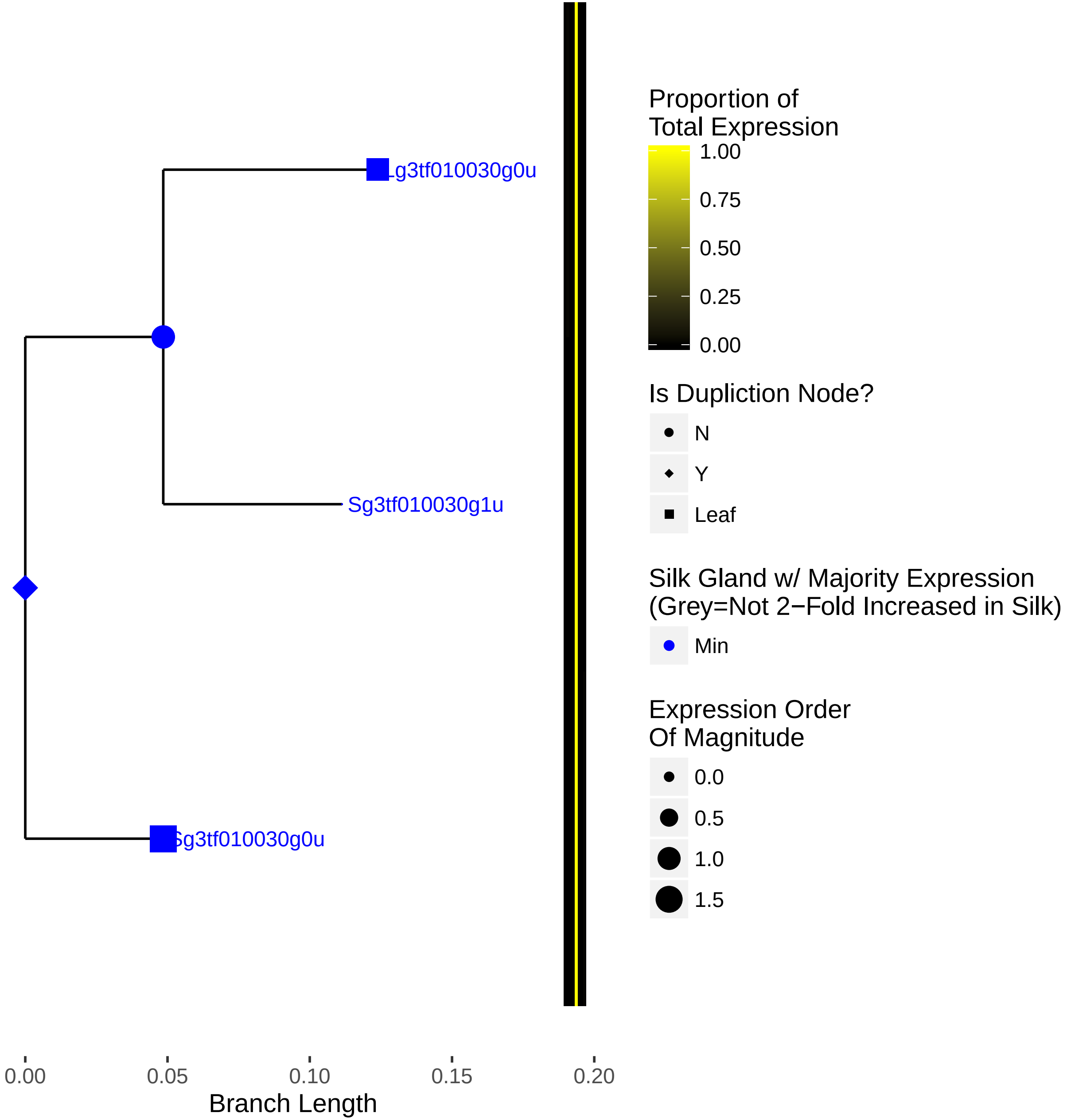

Lg3tf010030g0u
Sg3tf010030g1u
Sg3tf010030g0u
0.00
0.05
0.10
0.15
0.20
Branch Length
Proportion of
Total Expression
1.00
0.75
0.50
0.25
0.00
Is Dupliction Node?
N
Y
Leaf
Silk Gland w/ Majority Expression
(Grey=Not 2−Fold Increased in Silk)
Min
Expression Order
Of Magnitude
0.0
0.5
1.0
1.5

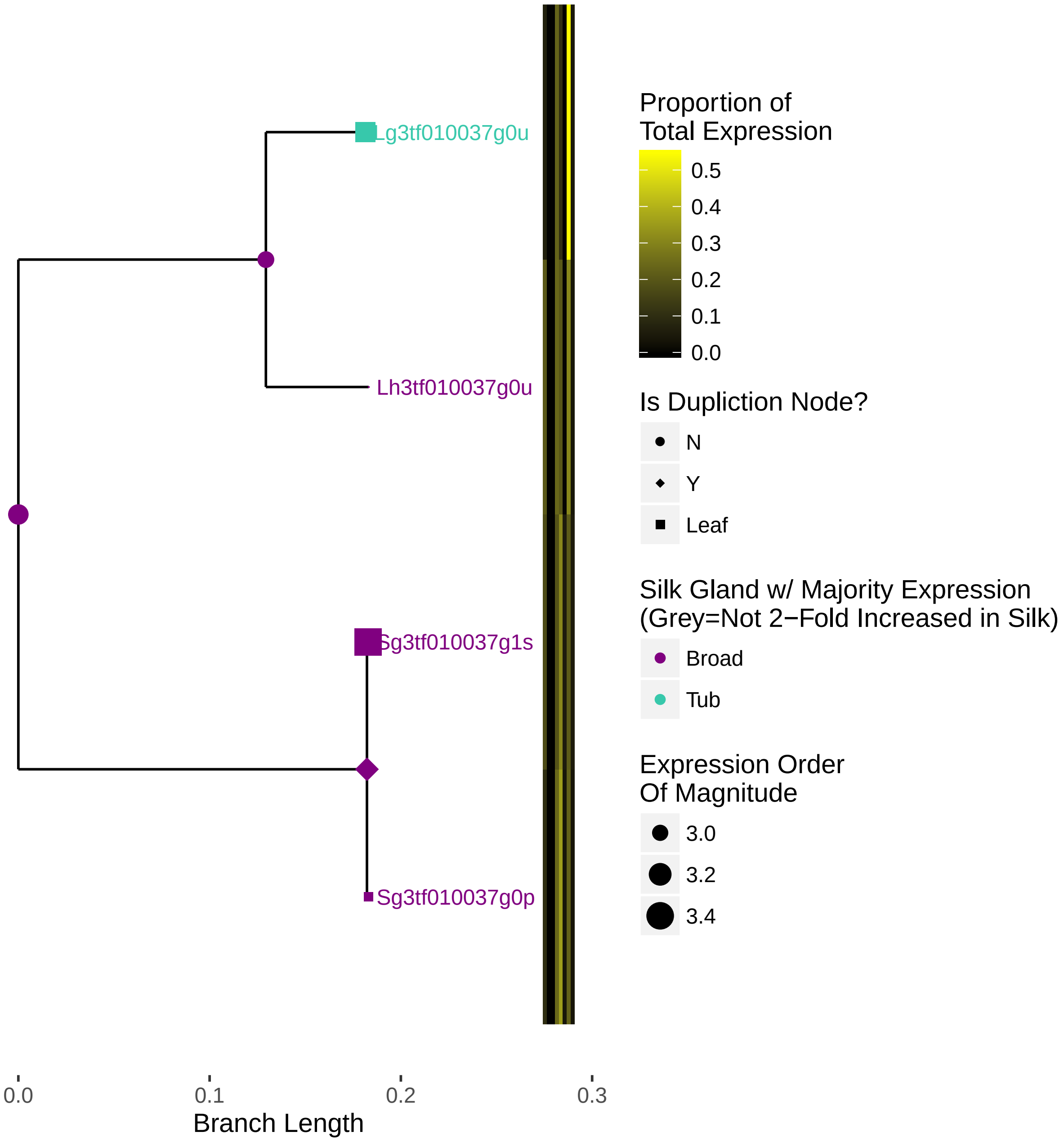

Lg3tf010037g0u
Lh3tf010037g0u
Sg3tf010037g1s
Sg3tf010037g0p
0.0
0.1
0.2
0.3
Branch Length
Proportion of
Total Expression
0.5
0.4
0.3
0.2
0.1
0.0
Is Dupliction Node?
N
Y
Leaf
Silk Gland w/ Majority Expression
(Grey=Not 2−Fold Increased in Silk)
Broad
Tub
Expression Order
Of Magnitude
3.0
3.2
3.4

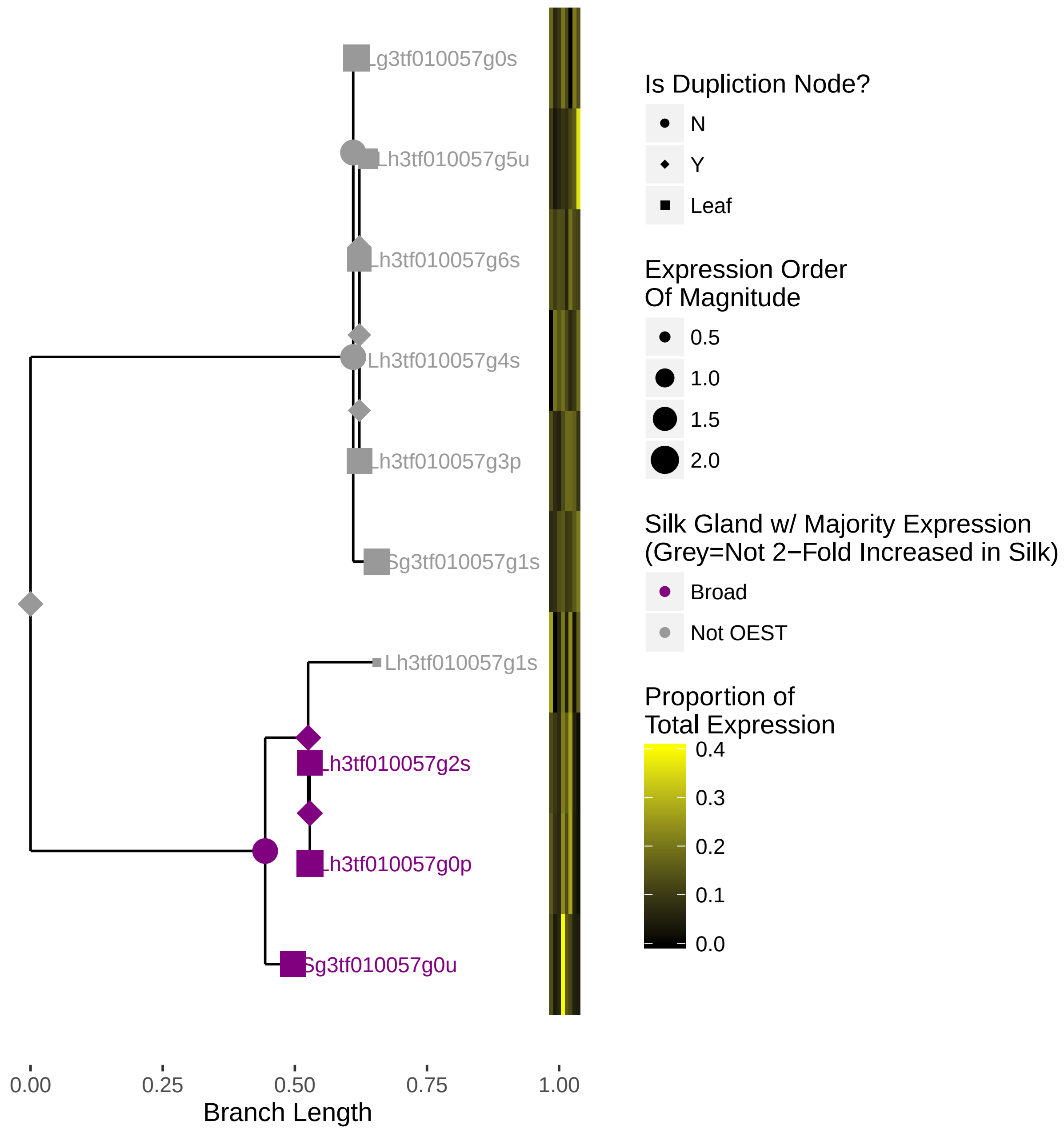
Lg3tf010057g0s
Lh3tf010057g5u
Lh3tf010057g6s
Lh3tf010057g4s
Lh3tf010057g3p
Sg3tf010057g1s
Lh3tf010057g1s
Lh3tf010057g2s
Lh3tf010057g0p
Sg3tf010057g0u
0.00
0.25
0.50
0.75
1.00
Branch Length
Is Dupliction Node?
N
Y
Leaf
Expression Order Of Magnitude
0.5
1.0
1.5
2.0
Silk Gland w/ Majority Expression (Grey=Not 2−Fold Increased in Silk)
Broad
Not OEST
Proportion of Total Expression
0.4
0.3
0.2
0.1
0.0

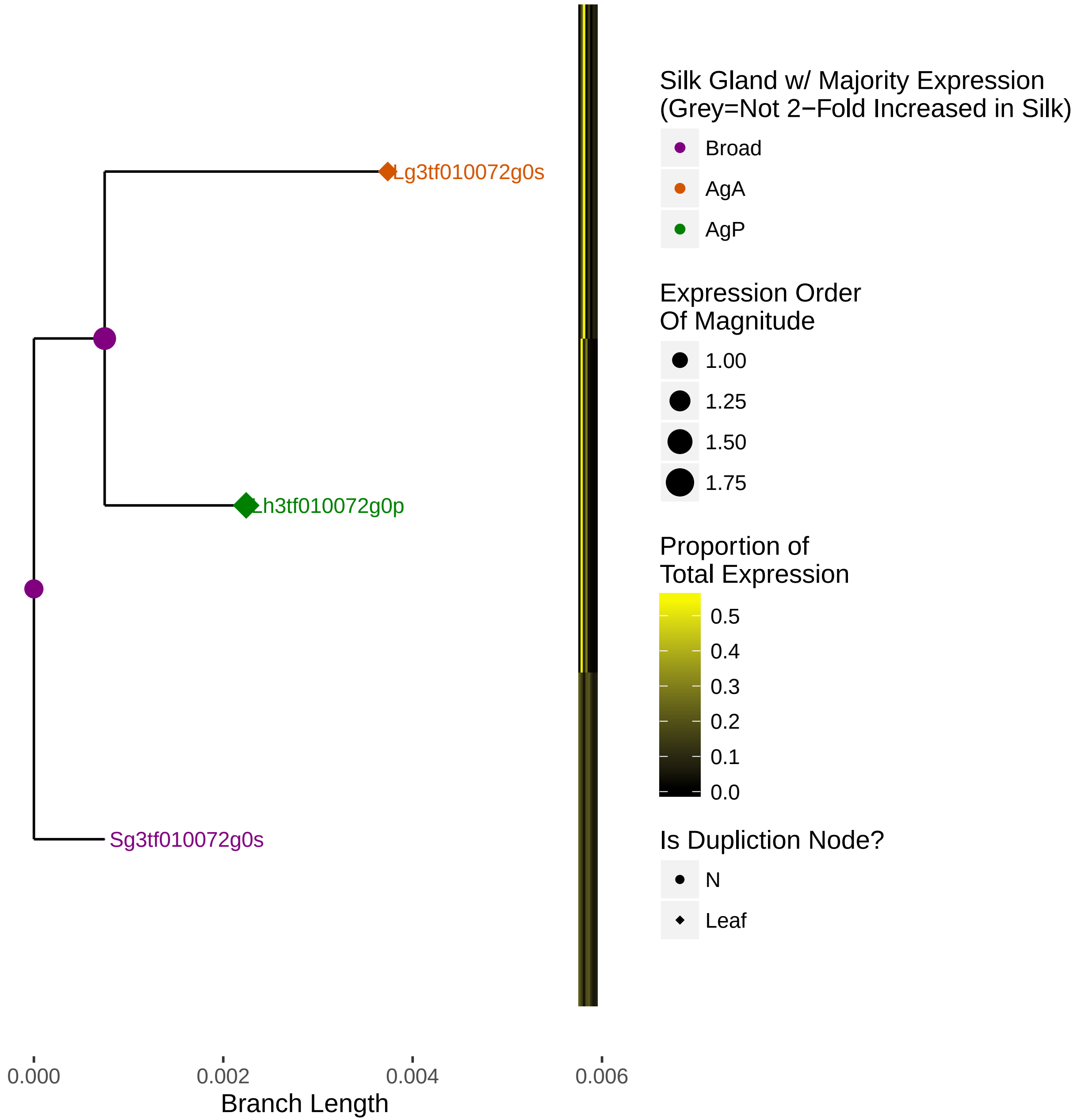
Lg3tf010072g0s
Lh3tf010072g0p
Sg3tf010072g0s
Silk Gland w/ Majority Expression
(Grey=Not 2−Fold Increased in Silk)
Broad
AgA
AgP
Expression Order
Of Magnitude
1.00
1.25
1.50
1.75
Proportion of
Total Expression
0.5
0.4
0.3
0.2
0.1
0.0
Is Dupliction Node?
N
Leaf
0.000
0.002
0.004
0.006
Branch Length

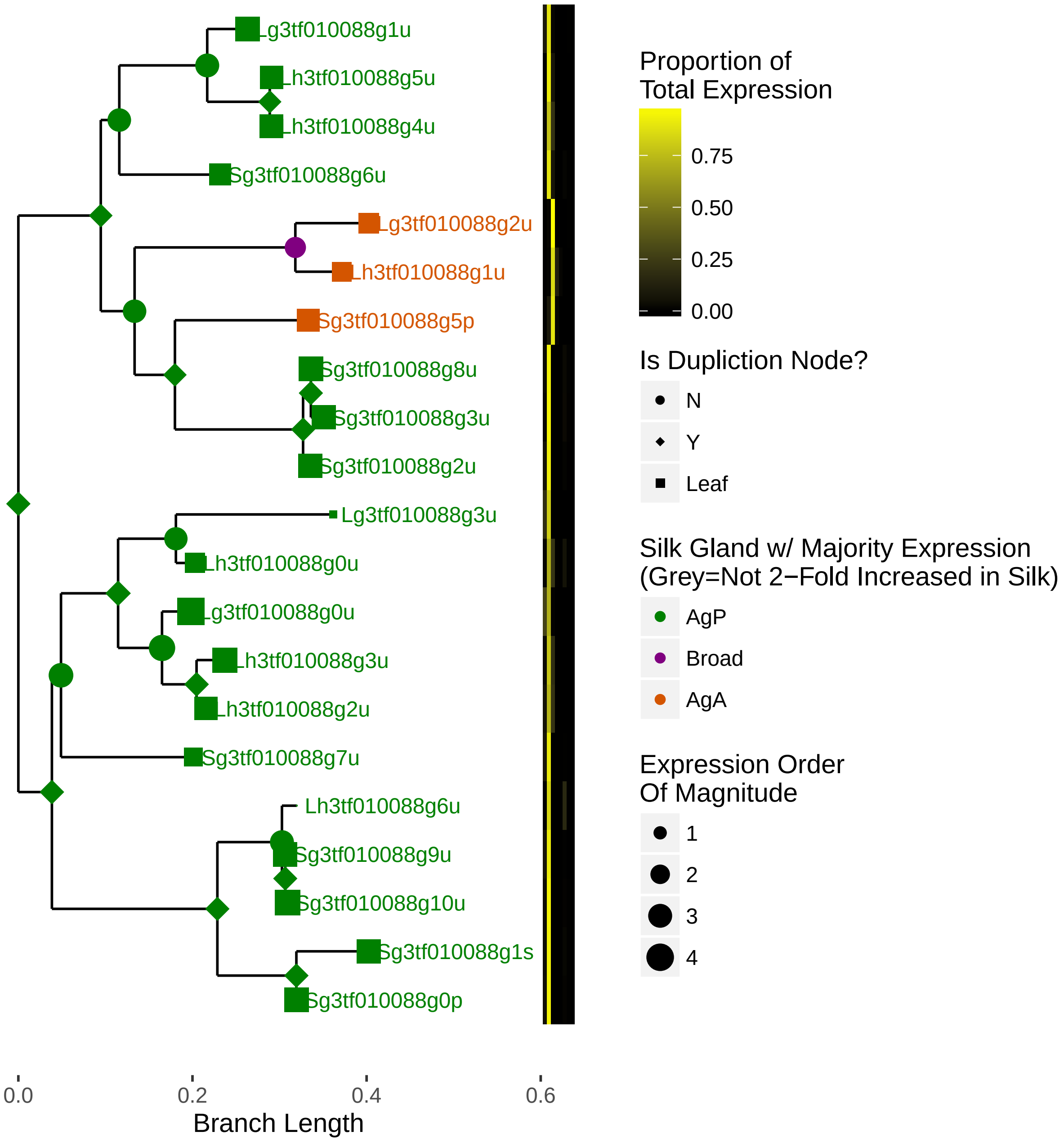
Lg3tf010088g1u
Lh3tf010088g5u
Lh3tf010088g4u
Sg3tf010088g6u
Lg3tf010088g2u
Lh3tf010088g1u
Sg3tf010088g5p
Sg3tf010088g8u
Sg3tf010088g3u
Sg3tf010088g2u
Lg3tf010088g3u
Lh3tf010088g0u
Lg3tf010088g0u
Lh3tf010088g3u
Lh3tf010088g2u
Sg3tf010088g7u
Lh3tf010088g6u
Sg3tf010088g9u
Sg3tf010088g10u
Sg3tf010088g1s
Sg3tf010088g0p
0.0
0.2
0.4
0.6
Branch Length
Proportion of Total Expression
0.75
0.50
0.25
0.00
Is Dupliction Node?
N
Y
Leaf
Silk Gland w/ Majority Expression (Grey=Not 2−Fold Increased in Silk)
AgP
Broad
AgA
Expression Order Of Magnitude
1
2
3
4

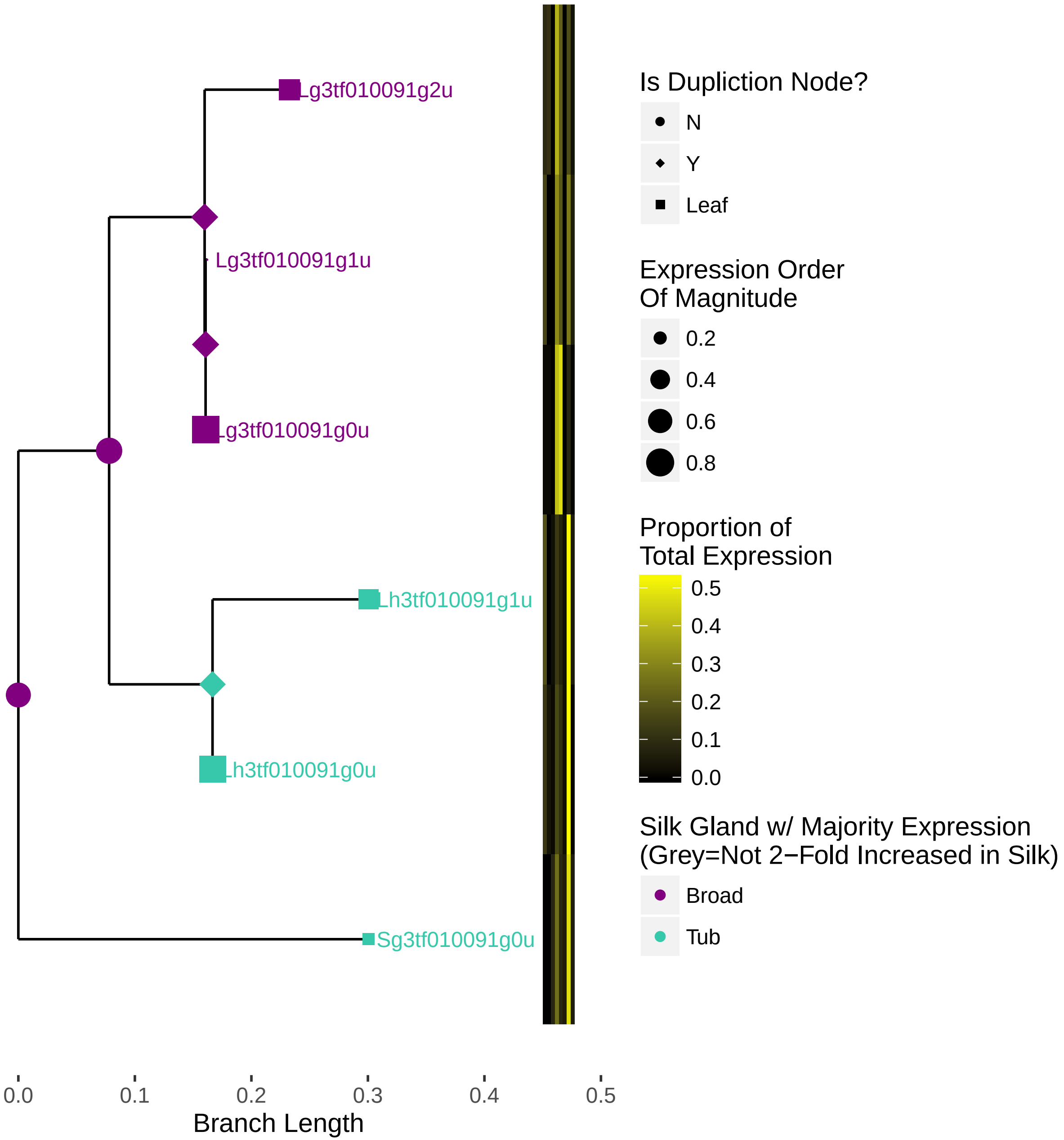

Lg3tf010091g2u
Lg3tf010091g1u
Lg3tf010091g0u
Lh3tf010091g1u
Lh3tf010091g0u
Sg3tf010091g0u
0.0
0.1
0.2
0.3
0.4
0.5
Branch Length
Is Dupliction Node?
N
Y
Leaf
Expression Order
Of Magnitude
0.2
0.4
0.6
0.8
Proportion of
Total Expression
0.5
0.4
0.3
0.2
0.1
0.0
Silk Gland w/ Majority Expression
(Grey=Not 2−Fold Increased in Silk)
Broad
Tub

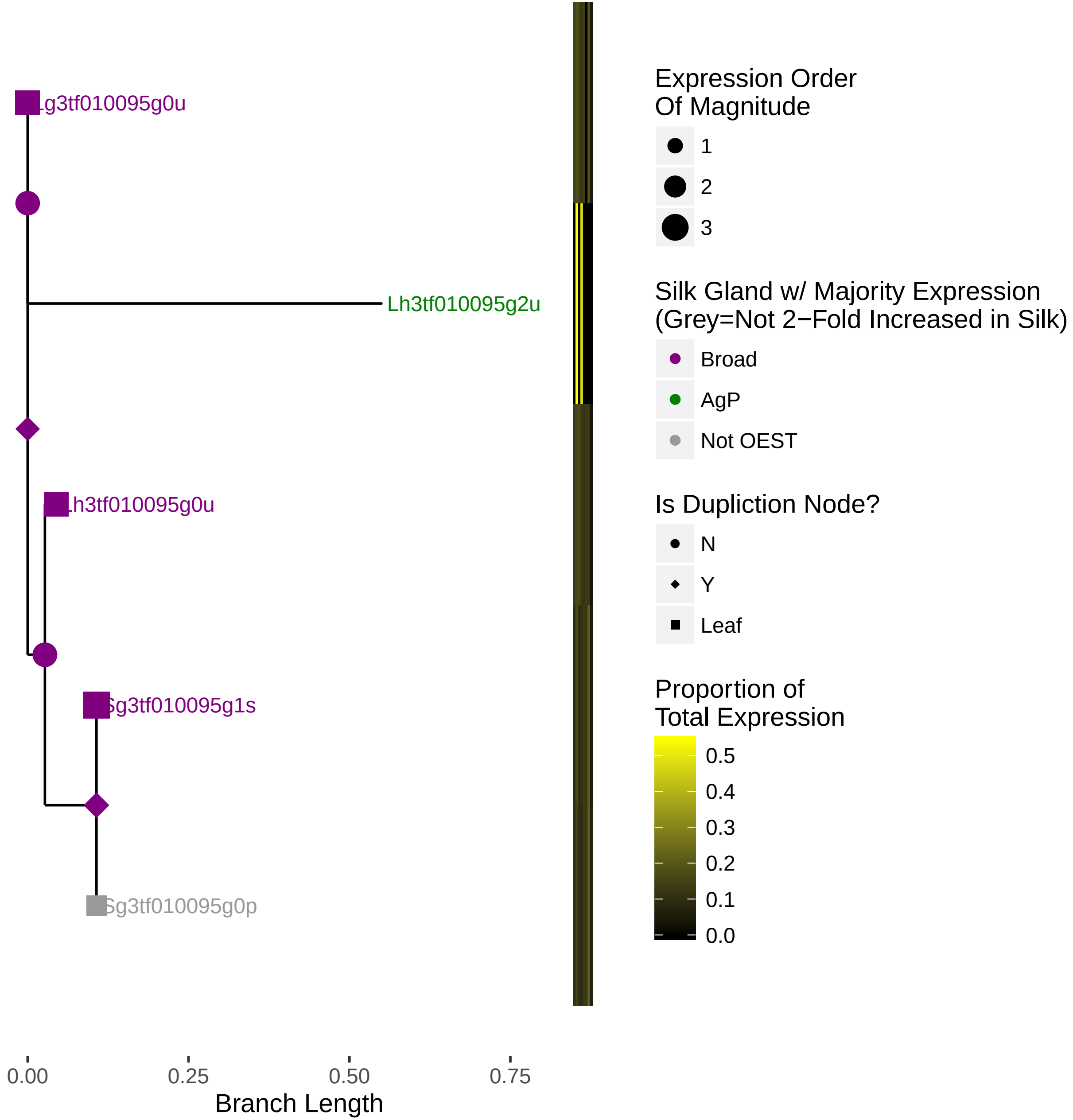

g3tf010095g0u
Lh3tf010095g2u
h3tf010095g0u
g3tf010095g1s
g3tf010095g0p
0.00
0.25
0.50
0.75
Branch Length
Expression Order
Of Magnitude
1
2
3
Silk Gland w/ Majority Expression
(Grey=Not 2−Fold Increased in Silk)
Broad
AgP
Not OEST
Is Dupliction Node?
N
Y
Leaf
Proportion of
Total Expression
0.5
0.4
0.3
0.2
0.1
0.0

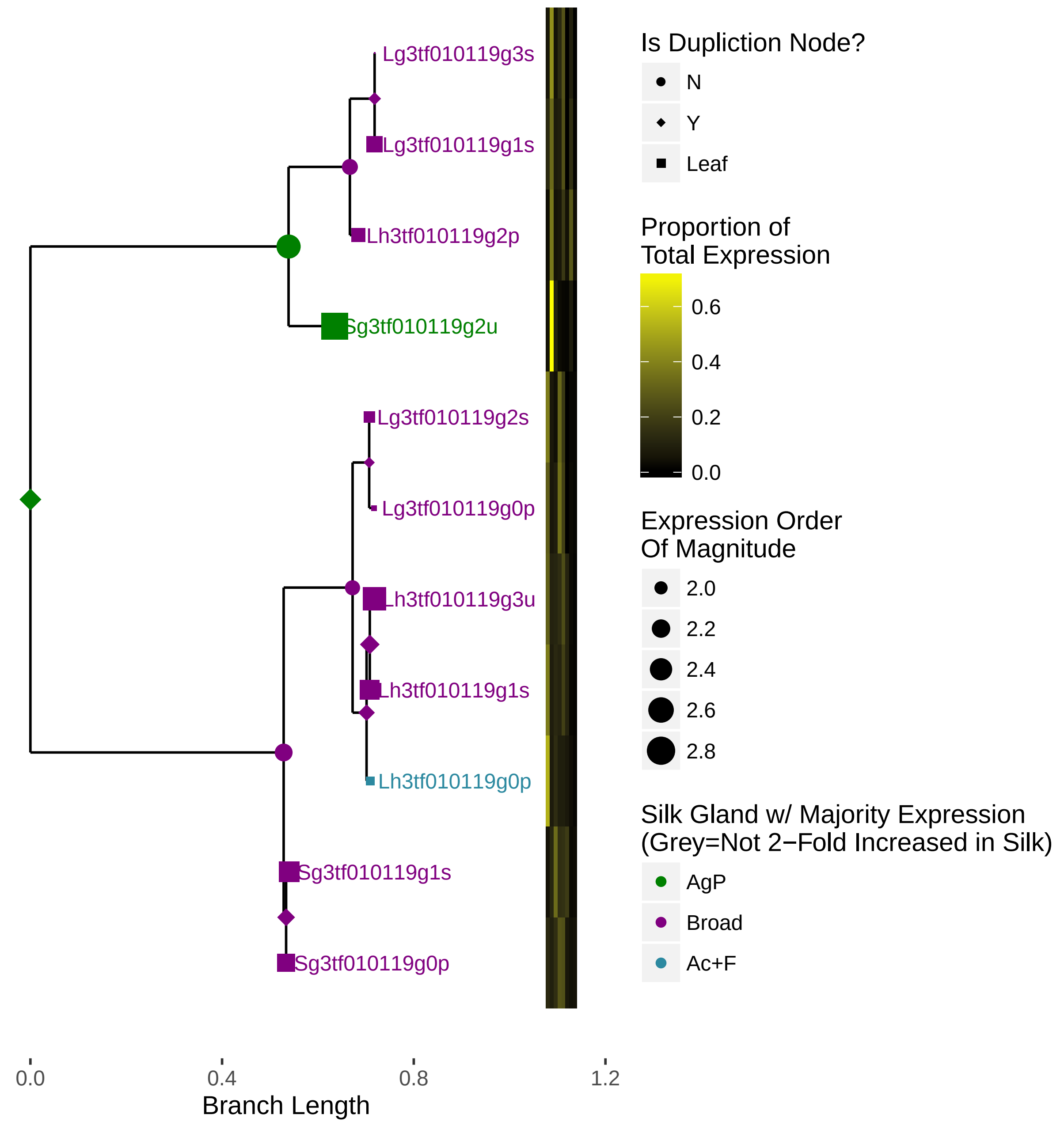

Lg3tf010119g3s
Lg3tf010119g1s
Lh3tf010119g2p
Sg3tf010119g2u
Lg3tf010119g2s
Lg3tf010119g0p
Lh3tf010119g3u
Lh3tf010119g1s
Lh3tf010119g0p
Sg3tf010119g1s
Sg3tf010119g0p
0.0
0.4
0.8
1.2
Branch Length
Is Dupliction Node?
N
Y
Leaf
Proportion of
Total Expression
0.6
0.4
0.2
0.0
Expression Order
Of Magnitude
2.0
2.2
2.4
2.6
2.8
Silk Gland w/ Majority Expression
(Grey=Not 2−Fold Increased in Silk)
AgP
Broad
Ac+F

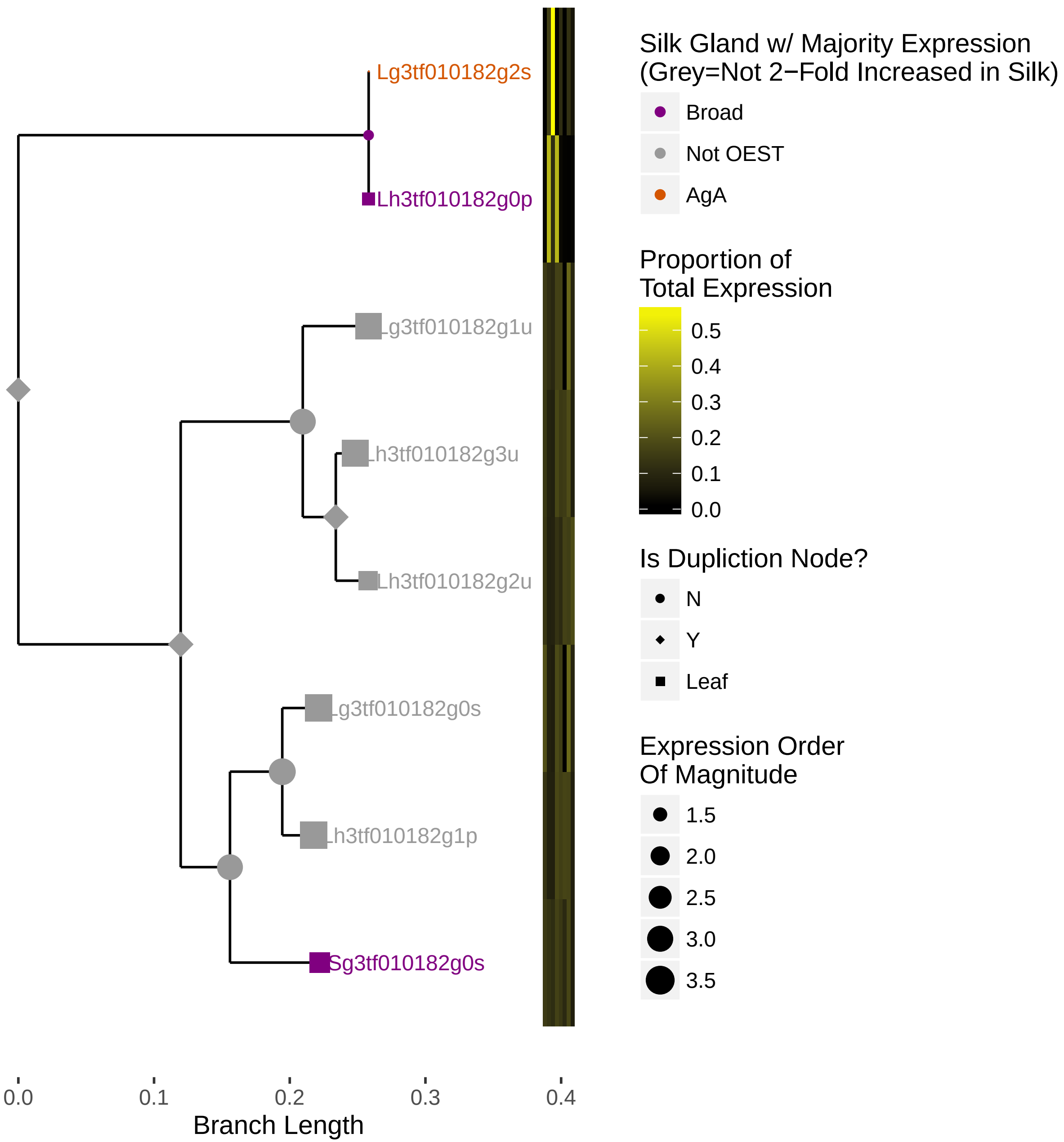
Lg3tf010182g2s
Lh3tf010182g0p
Lg3tf010182g1u
Lh3tf010182g3u
Lh3tf010182g2u
Lg3tf010182g0s
Lh3tf010182g1p
Sg3tf010182g0s
0.0
0.1
0.2
0.3
0.4
Branch Length
Silk Gland w/ Majority Expression
(Grey=Not 2−Fold Increased in Silk)
Broad
Not OEST
AgA
Proportion of
Total Expression
0.5
0.4
0.3
0.2
0.1
0.0
Is Dupliction Node?
N
Y
Leaf
Expression Order
Of Magnitude
1.5
2.0
2.5
3.0
3.5

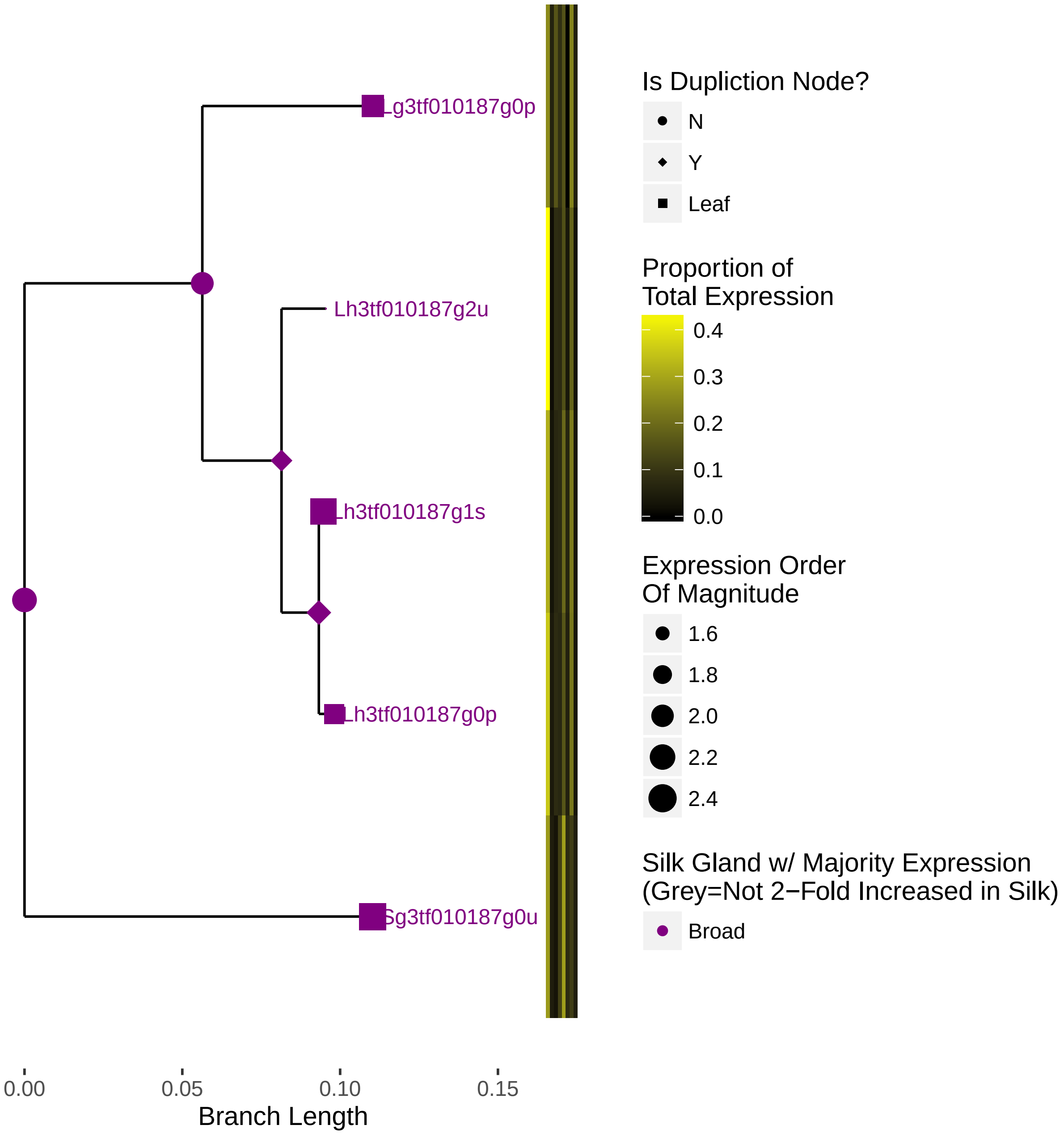

Lg3tf010187g0p
Lh3tf010187g2u
Lh3tf010187g1s
Lh3tf010187g0p
Sg3tf010187g0u
0.00
0.05
0.10
0.15
Branch Length
Is Dupliction Node?
N
Y
Leaf
Proportion of
Total Expression
0.4
0.3
0.2
0.1
0.0
Expression Order
Of Magnitude
1.6
1.8
2.0
2.2
2.4
Silk Gland w/ Majority Expression
(Grey=Not 2−Fold Increased in Silk)
Broad

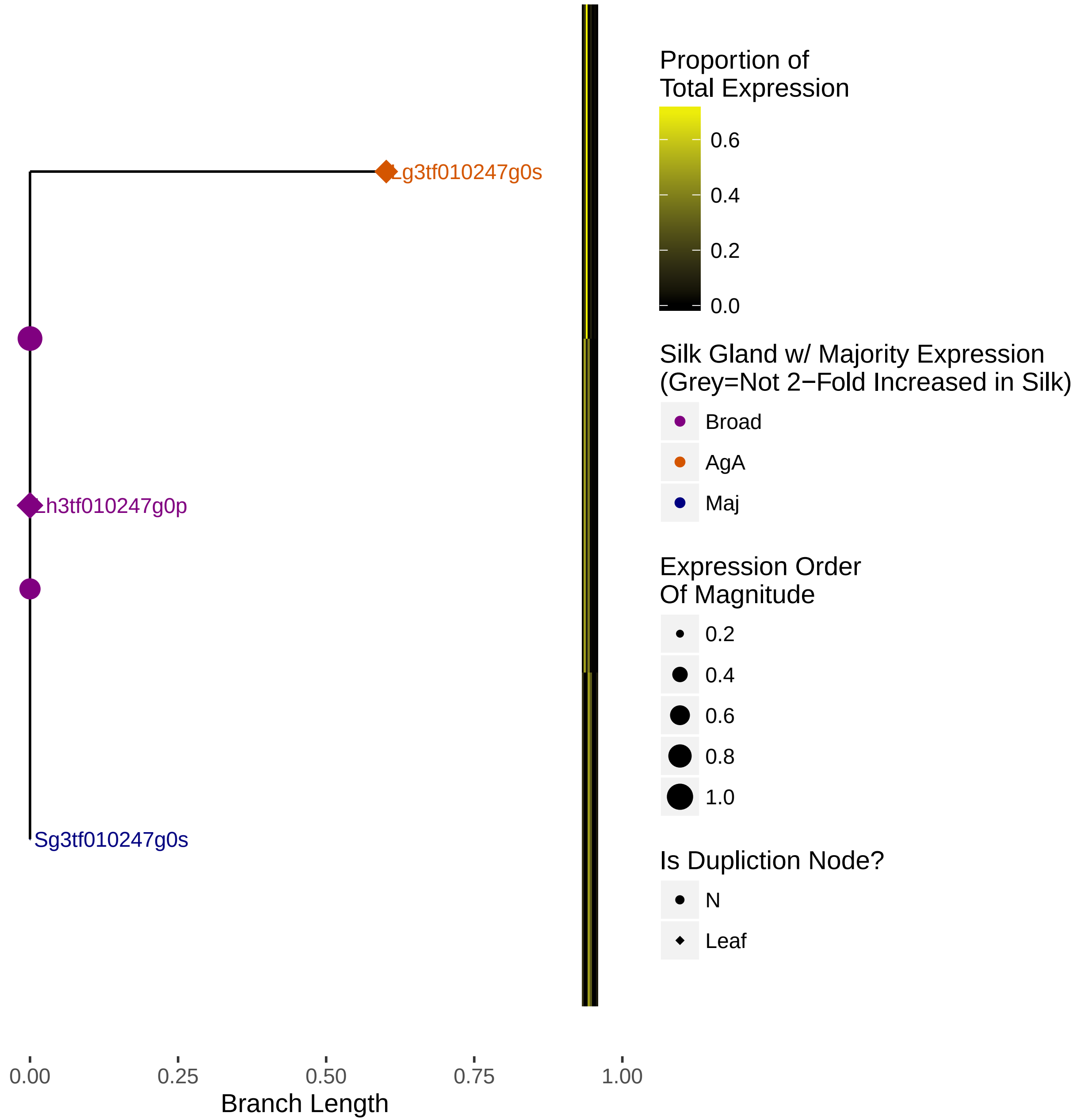

Lg3tf010247g0s
Lh3tf010247g0p
Sg3tf010247g0s
0.00
0.25
0.50
0.75
1.00
Branch Length
Proportion of
Total Expression
0.6
0.4
0.2
0.0
Silk Gland w/ Majority Expression
(Grey=Not 2−Fold Increased in Silk)
Broad
AgA
Maj
Expression Order
Of Magnitude
0.2
0.4
0.6
0.8
1.0
Is Dupliction Node?
N
Leaf

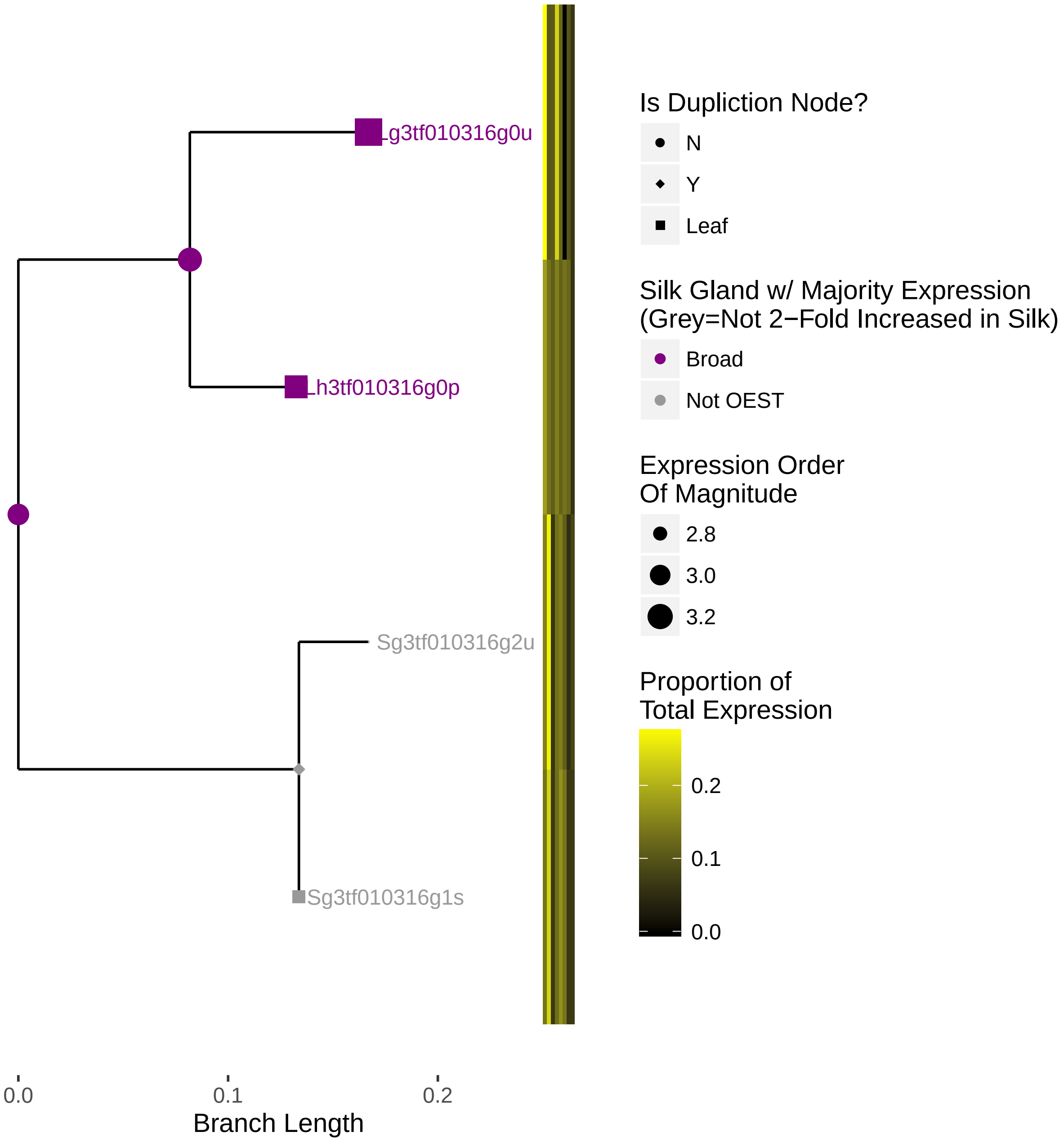

Lg3tf010316g0u
Lh3tf010316g0p
Sg3tf010316g2u
Sg3tf010316g1s
0.0
0.1
0.2
Branch Length
Is Dupliction Node?
N
Y
Leaf
Silk Gland w/ Majority Expression
(Grey=Not 2−Fold Increased in Silk)
Broad
Not OEST
Expression Order
Of Magnitude
2.8
3.0
3.2
Proportion of
Total Expression
0.2
0.1
0.0

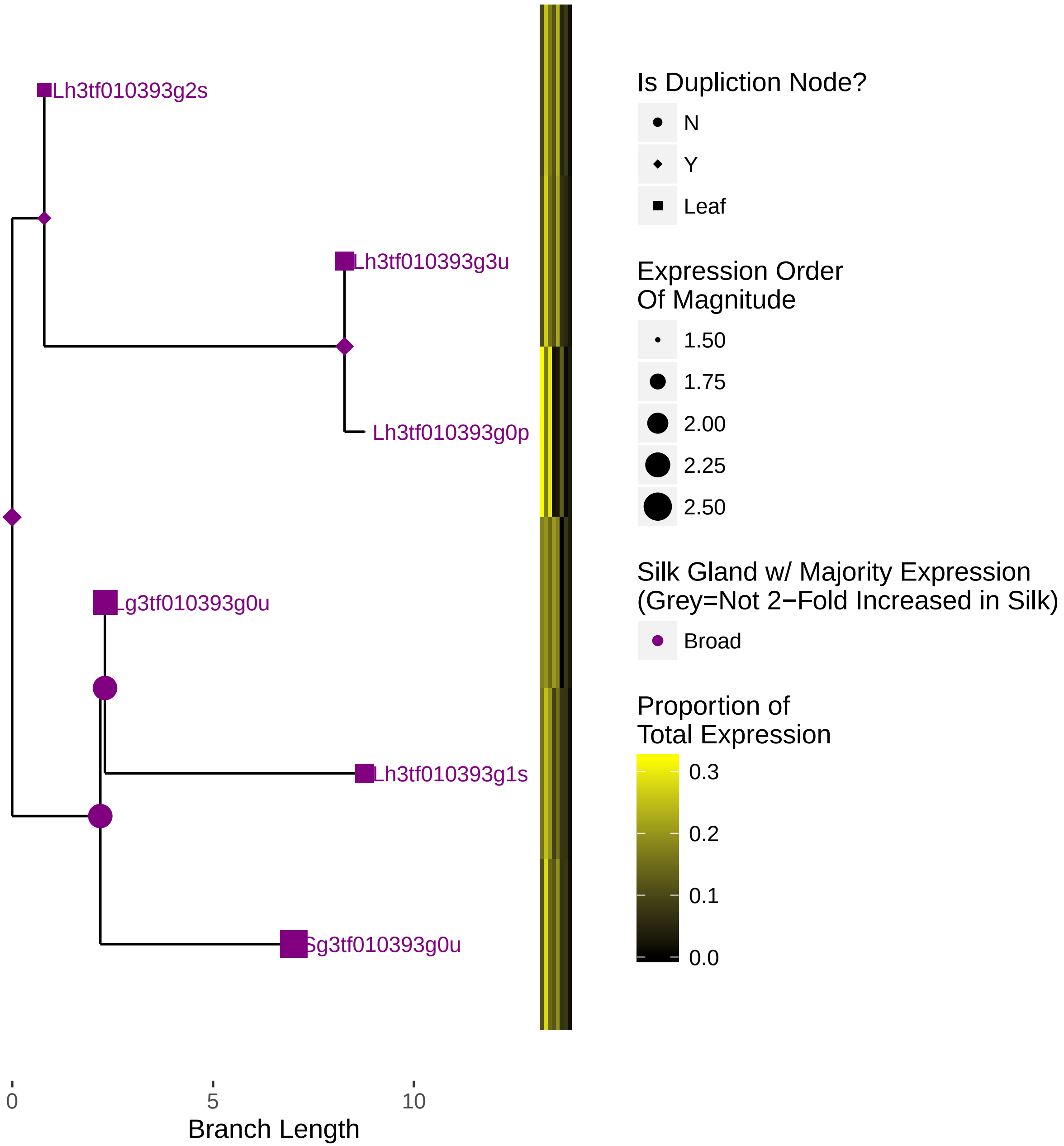

Lh3tf010393g2s
Lh3tf010393g3u
Lh3tf010393g0p
Lg3tf010393g0u
Lh3tf010393g1s
Sg3tf010393g0u
0
5
10
Branch Length
Is Dupliction Node?
N
Y
Leaf
Expression Order
Of Magnitude
1.50
1.75
2.00
2.25
2.50
Silk Gland w/ Majority Expression
(Grey=Not 2−Fold Increased in Silk)
Broad
Proportion of
Total Expression
0.3
0.2
0.1
0.0

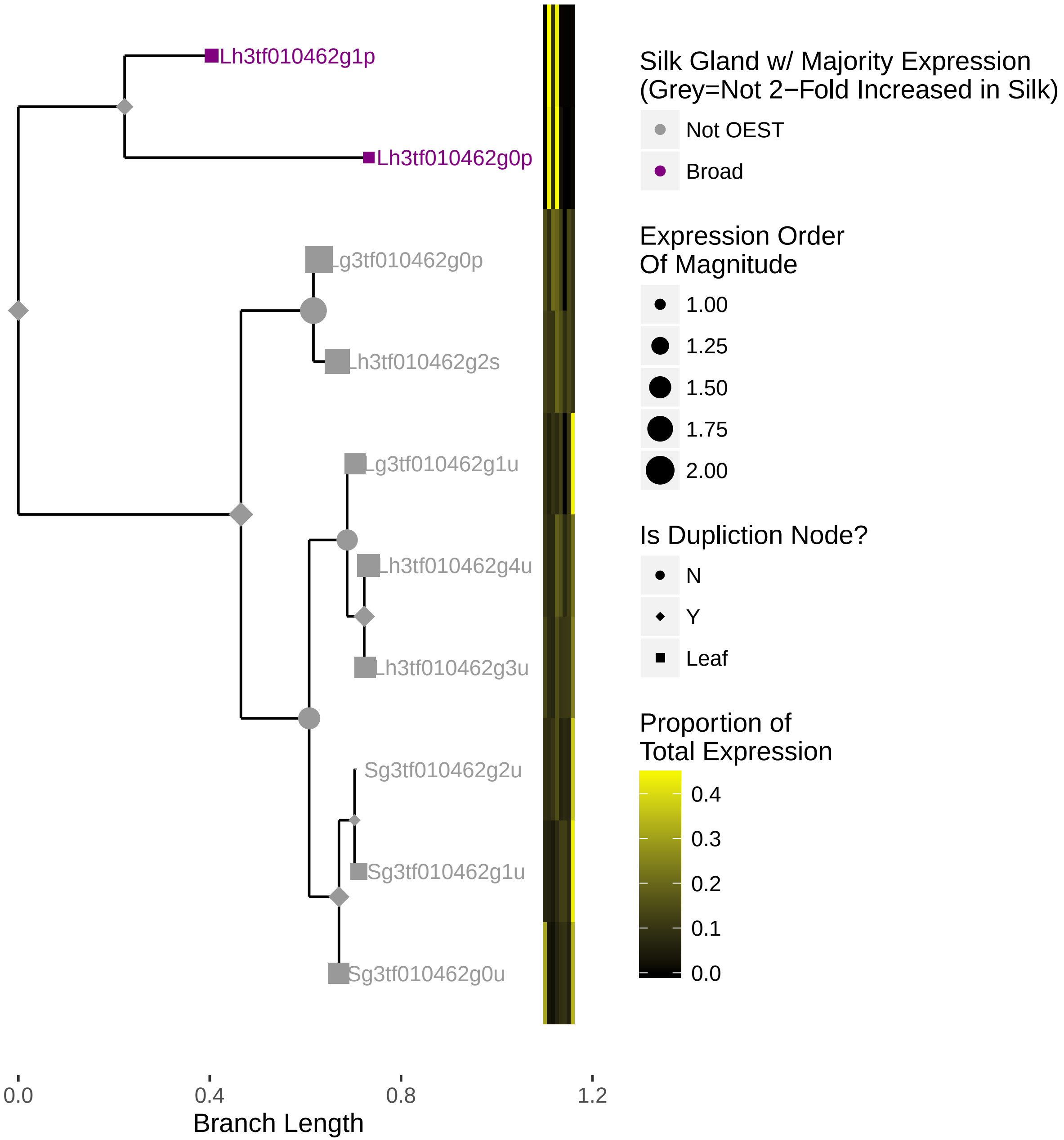

Lh3tf010462g1p
Lh3tf010462g0p
Lg3tf010462g0p
Lh3tf010462g2s
Lg3tf010462g1u
Lh3tf010462g4u
Lh3tf010462g3u
Sg3tf010462g2u
Sg3tf010462g1u
Sg3tf010462g0u
0.0
0.4
0.8
1.2
Branch Length
Silk Gland w/ Majority Expression
(Grey=Not 2−Fold Increased in Silk)
Not OEST
Broad
Expression Order
Of Magnitude
1.00
1.25
1.50
1.75
2.00
Is Dupliction Node?
N
Y
Leaf
Proportion of
Total Expression
0.4
0.3
0.2
0.1
0.0

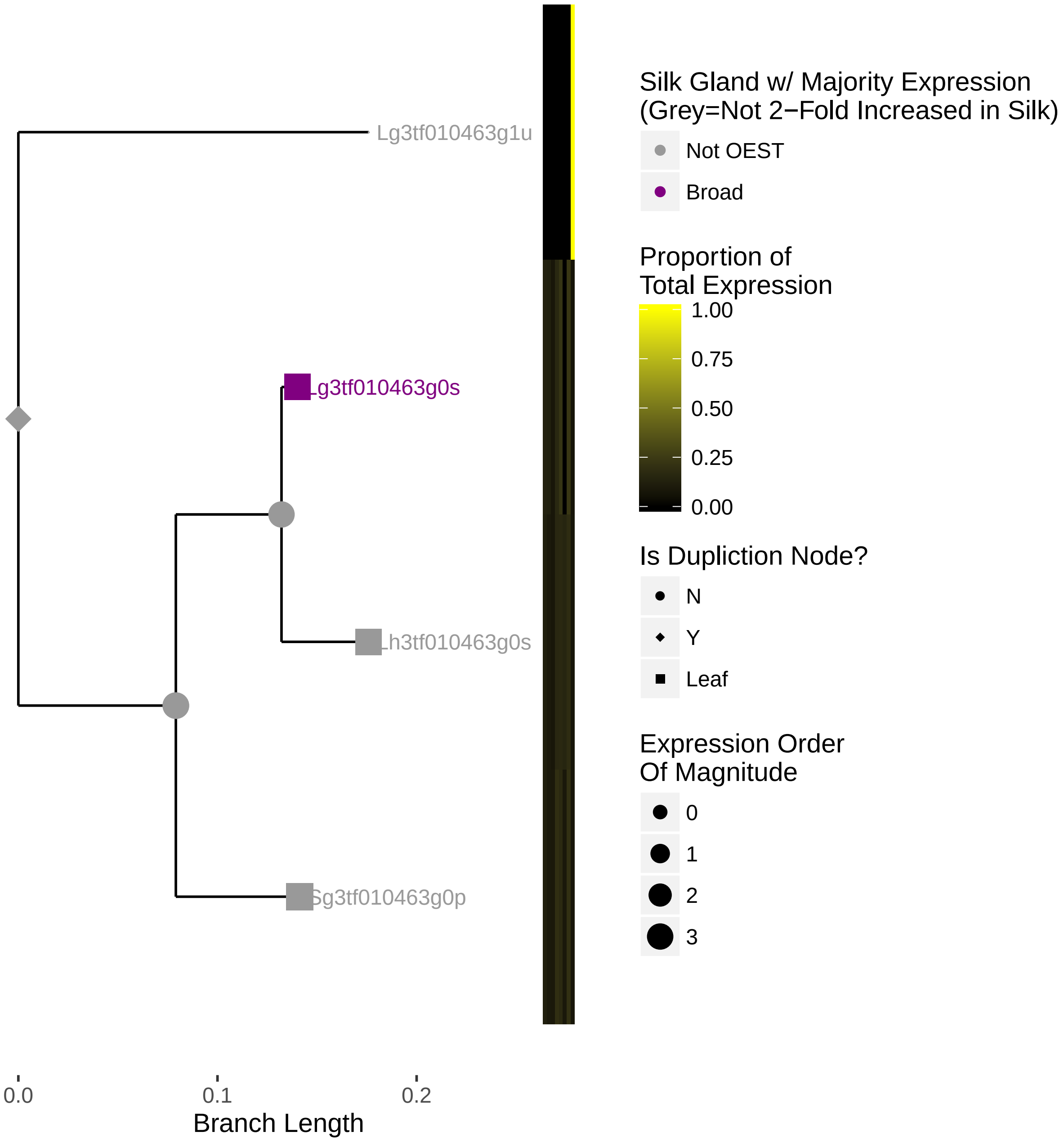

Lg3tf010463g1u
Lg3tf010463g0s
Lh3tf010463g0s
Sg3tf010463g0p
0.0
0.1
0.2
Branch Length
Silk Gland w/ Majority Expression
(Grey=Not 2−Fold Increased in Silk)
Not OEST
Broad
Proportion of
Total Expression
1.00
0.75
0.50
0.25
0.00
Is Dupliction Node?
N
Y
Leaf
Expression Order
Of Magnitude
0
1
2
3

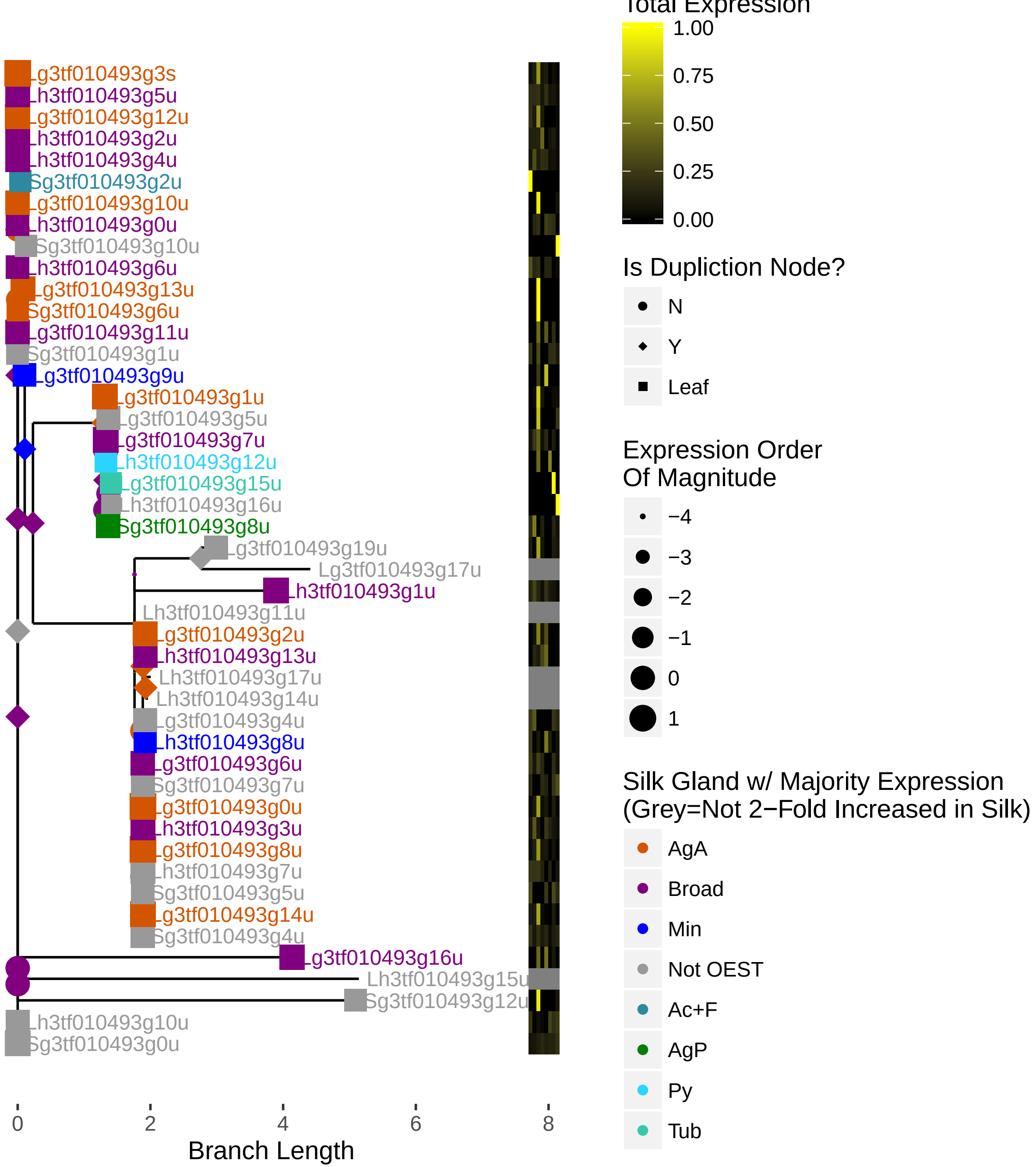
Total Expression
1.00
0.75
0.50
0.25
0.00
Is Dupliction Node?
N
Y
Leaf
Expression Order Of Magnitude
−4
−3
−2
−1
0
1
Silk Gland w/ Majority Expression (Grey=Not 2−Fold Increased in Silk)
AgA
Broad
Min
Not OEST
Ac+F
AgP
Py
Tub
Lg3tf010493g3s
Lh3tf010493g5u
Lg3tf010493g12u
Lh3tf010493g2u
Lh3tf010493g4u
Sg3tf010493g2u
Lg3tf010493g10u
Lh3tf010493g0u
Sg3tf010493g10u
Lh3tf010493g6u
Lg3tf010493g13u
Sg3tf010493g6u
Lg3tf010493g11u
Sg3tf010493g1u
Lg3tf010493g9u
Lg3tf010493g1u
Lg3tf010493g5u
Lg3tf010493g7u
Lh3tf010493g12u
Lg3tf010493g15u
Lh3tf010493g16u
Sg3tf010493g8u
Lg3tf010493g19u
Lg3tf010493g17u
Lh3tf010493g1u
Lh3tf010493g11u
Lg3tf010493g2u
Lh3tf010493g13u
Lh3tf010493g17u
Lh3tf010493g14u
Lg3tf010493g4u
Lh3tf010493g8u
Lg3tf010493g6u
Sg3tf010493g7u
Lg3tf010493g0u
Lh3tf010493g3u
Lg3tf010493g8u
Lh3tf010493g7u
Sg3tf010493g5u
Lg3tf010493g14u
Sg3tf010493g4u
Lg3tf010493g16u
Lh3tf010493g15u
Sg3tf010493g12u
Lh3tf010493g10u
Sg3tf010493g0u
0
2
4
6
8
Branch Length

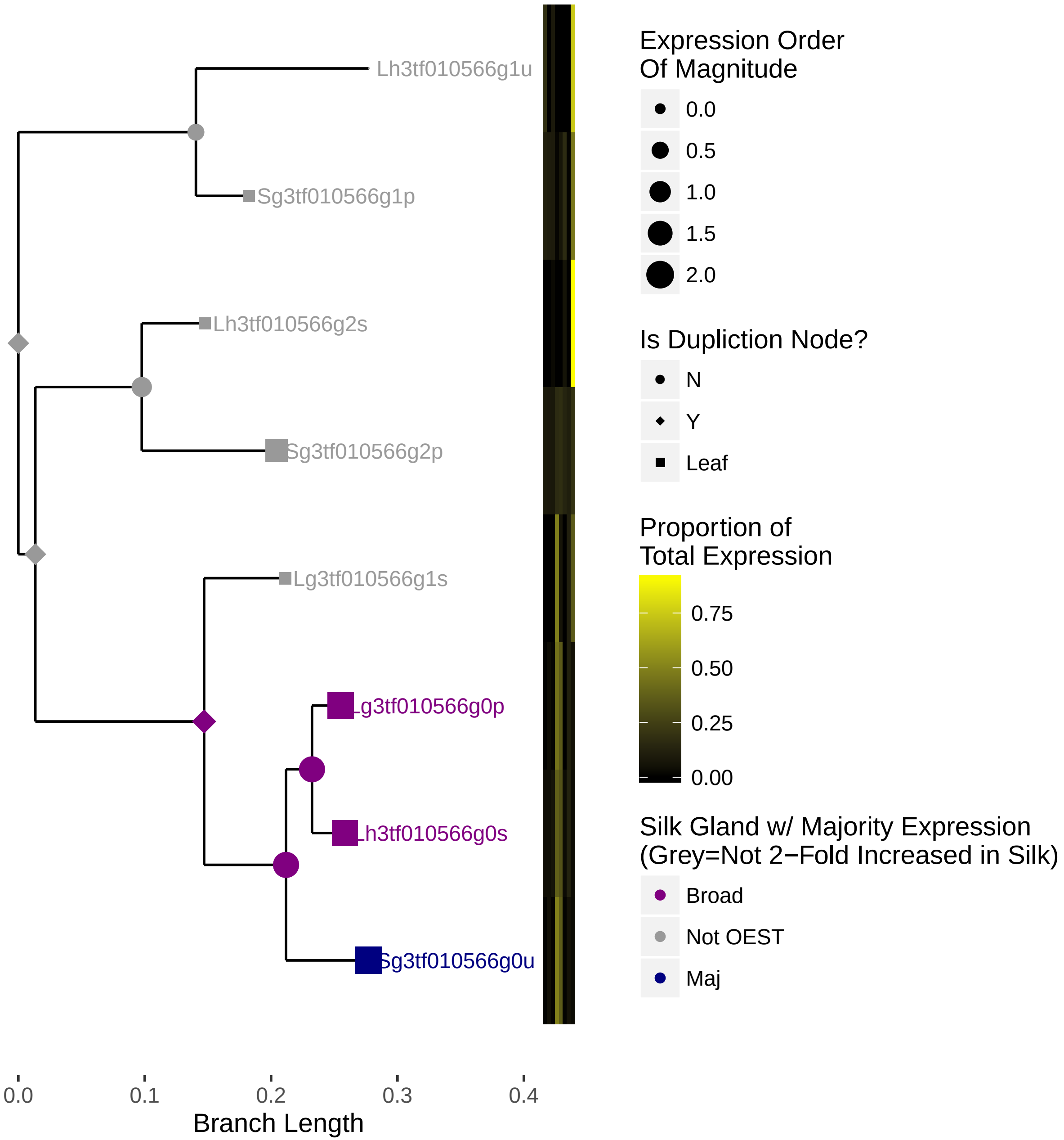
Lh3tf010566g1u
Sg3tf010566g1p
Lh3tf010566g2s
Sg3tf010566g2p
Lg3tf010566g1s
Lg3tf010566g0p
Lh3tf010566g0s
Sg3tf010566g0u
0.0
0.1
0.2
0.3
0.4
Branch Length
Expression Order
Of Magnitude
0.0
0.5
1.0
1.5
2.0
Is Dupliction Node?
N
Y
Leaf
Proportion of
Total Expression
0.75
0.50
0.25
0.00
Silk Gland w/ Majority Expression
(Grey=Not 2−Fold Increased in Silk)
Broad
Not OEST
Maj

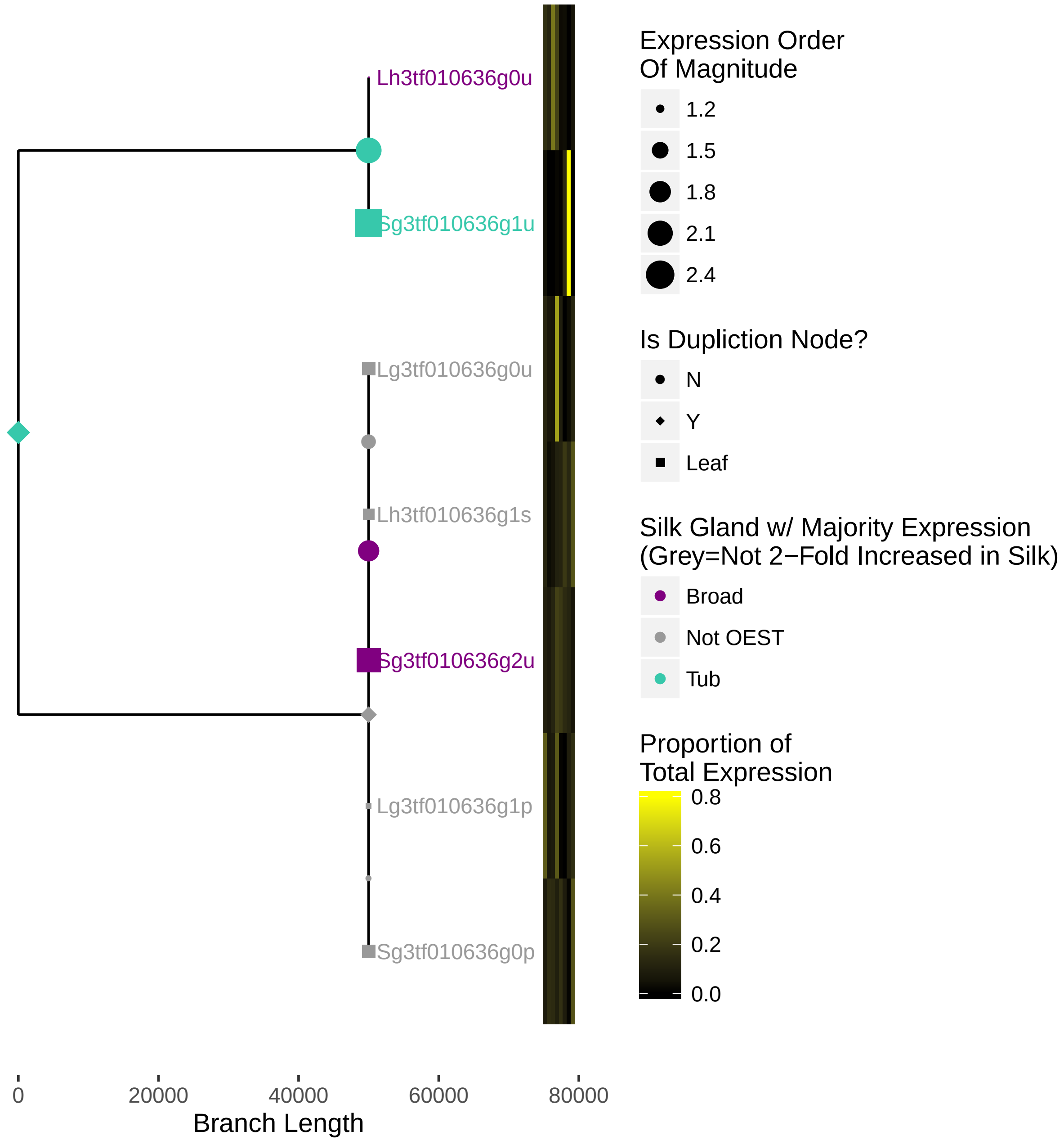

Lh3tf010636g0u
Sg3tf010636g1u
Lg3tf010636g0u
Lh3tf010636g1s
Sg3tf010636g2u
Lg3tf010636g1p
Sg3tf010636g0p
0
20000
40000
60000
80000
Branch Length
Expression Order Of Magnitude
1.2
1.5
1.8
2.1
2.4
Is Dupliction Node?
N
Y
Leaf
Silk Gland w/ Majority Expression (Grey=Not 2−Fold Increased in Silk)
Broad
Not OEST
Tub
Proportion of Total Expression
0.8
0.6
0.4
0.2
0.0

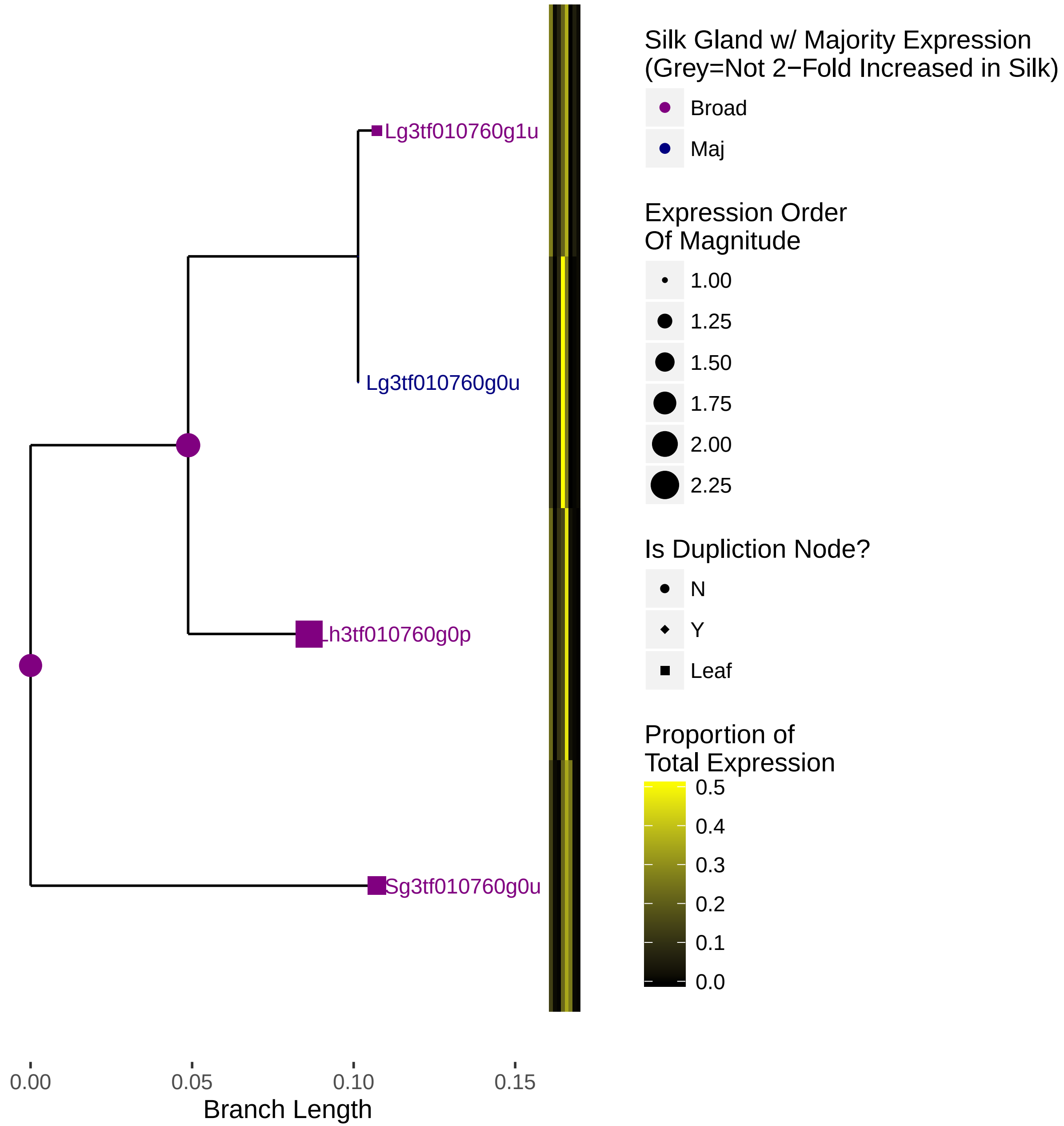

Lg3tf010760g1u
Lg3tf010760g0u
Lh3tf010760g0p
Sg3tf010760g0u
0.00
0.05
0.10
0.15
Branch Length
Silk Gland w/ Majority Expression
(Grey=Not 2−Fold Increased in Silk)
Broad
Maj
Expression Order
Of Magnitude
1.00
1.25
1.50
1.75
2.00
2.25
Is Dupliction Node?
N
Y
Leaf
Proportion of
Total Expression
0.5
0.4
0.3
0.2
0.1
0.0

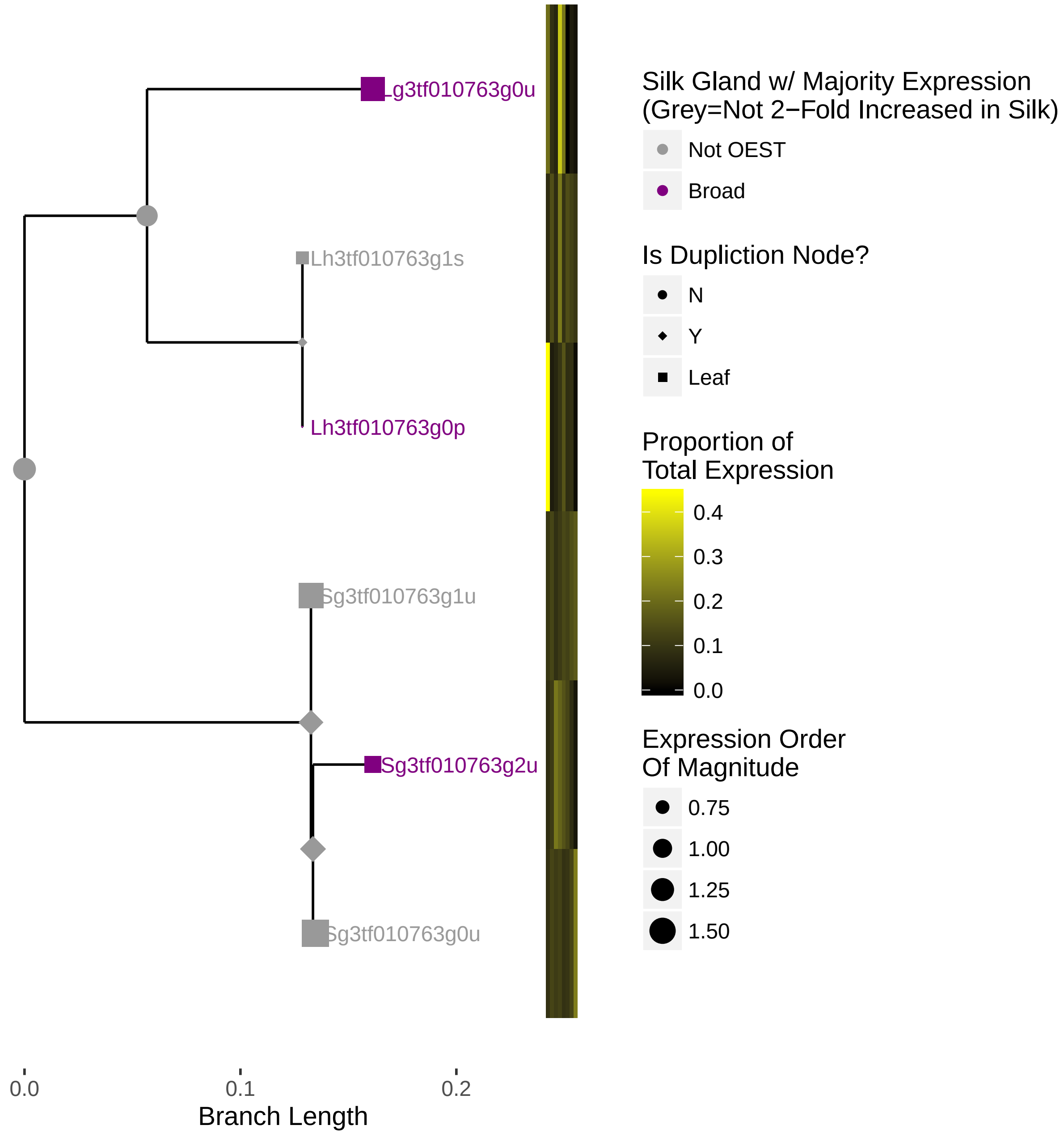
Lg3tf010763g0u
Lh3tf010763g1s
Lh3tf010763g0p
Sg3tf010763g1u
Sg3tf010763g2u
Sg3tf010763g0u
0.0
0.1
0.2
Branch Length
Silk Gland w/ Majority Expression
(Grey=Not 2−Fold Increased in Silk)
Not OEST
Broad
Is Dupliction Node?
N
Y
Leaf
Proportion of
Total Expression
0.4
0.3
0.2
0.1
0.0
Expression Order
Of Magnitude
0.75
1.00
1.25
1.50

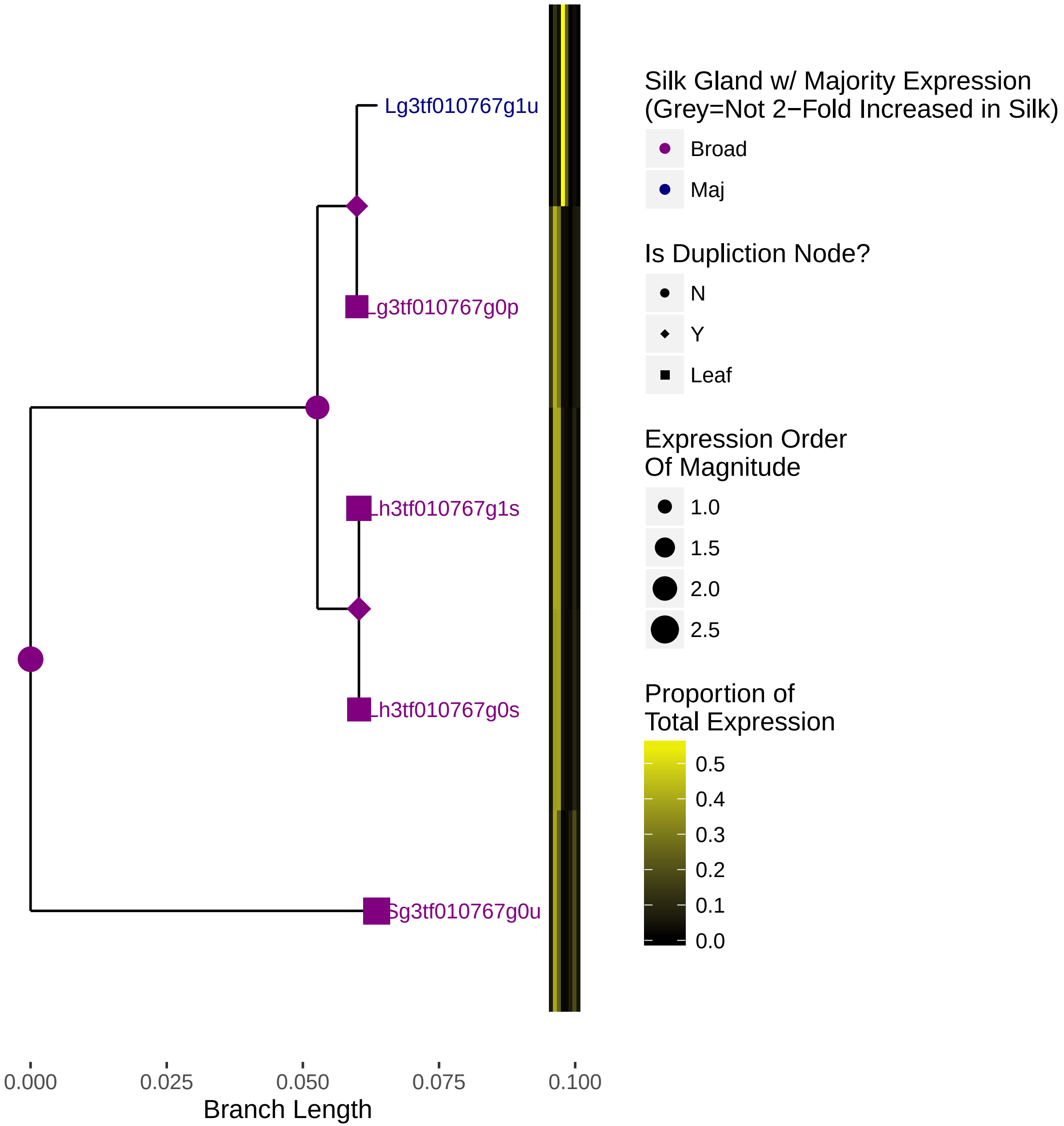
Lg3tf010767g1u
Lg3tf010767g0p
Lh3tf010767g1s
Lh3tf010767g0s
Sg3tf010767g0u
0.000
0.025
0.050
0.075
0.100
Branch Length
Silk Gland w/ Majority Expression
(Grey=Not 2−Fold Increased in Silk)
Broad
Maj
Is Dupliction Node?
N
Y
Leaf
Expression Order
Of Magnitude
1.0
1.5
2.0
2.5
Proportion of
Total Expression
0.5
0.4
0.3
0.2
0.1
0.0

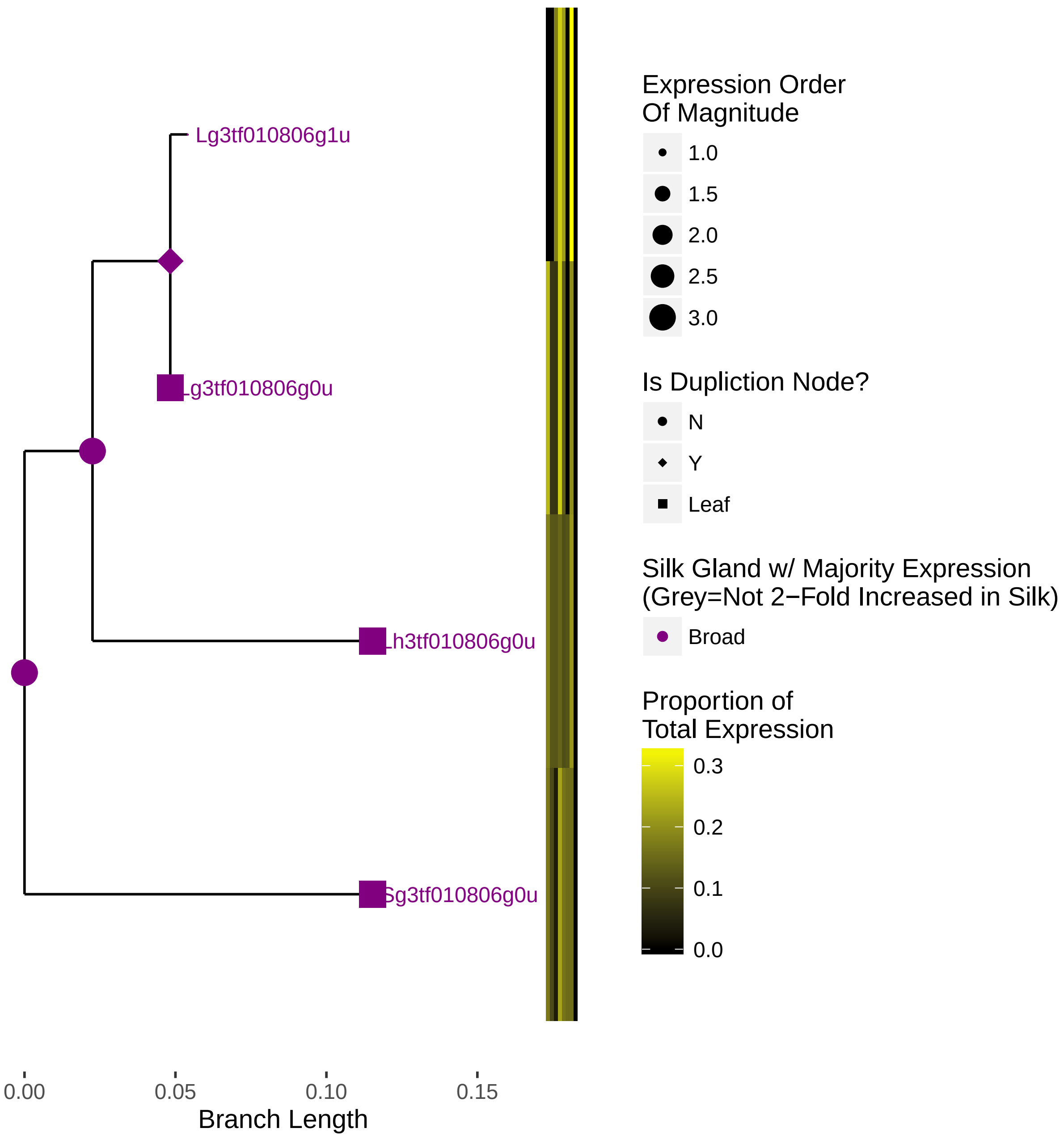

Lg3tf010806g1u
Lg3tf010806g0u
Lh3tf010806g0u
Sg3tf010806g0u
0.00
0.05
0.10
0.15
Branch Length
Expression Order
Of Magnitude
1.0
1.5
2.0
2.5
3.0
Is Dupliction Node?
N
Y
Leaf
Silk Gland w/ Majority Expression
(Grey=Not 2−Fold Increased in Silk)
Broad
Proportion of
Total Expression
0.3
0.2
0.1
0.0

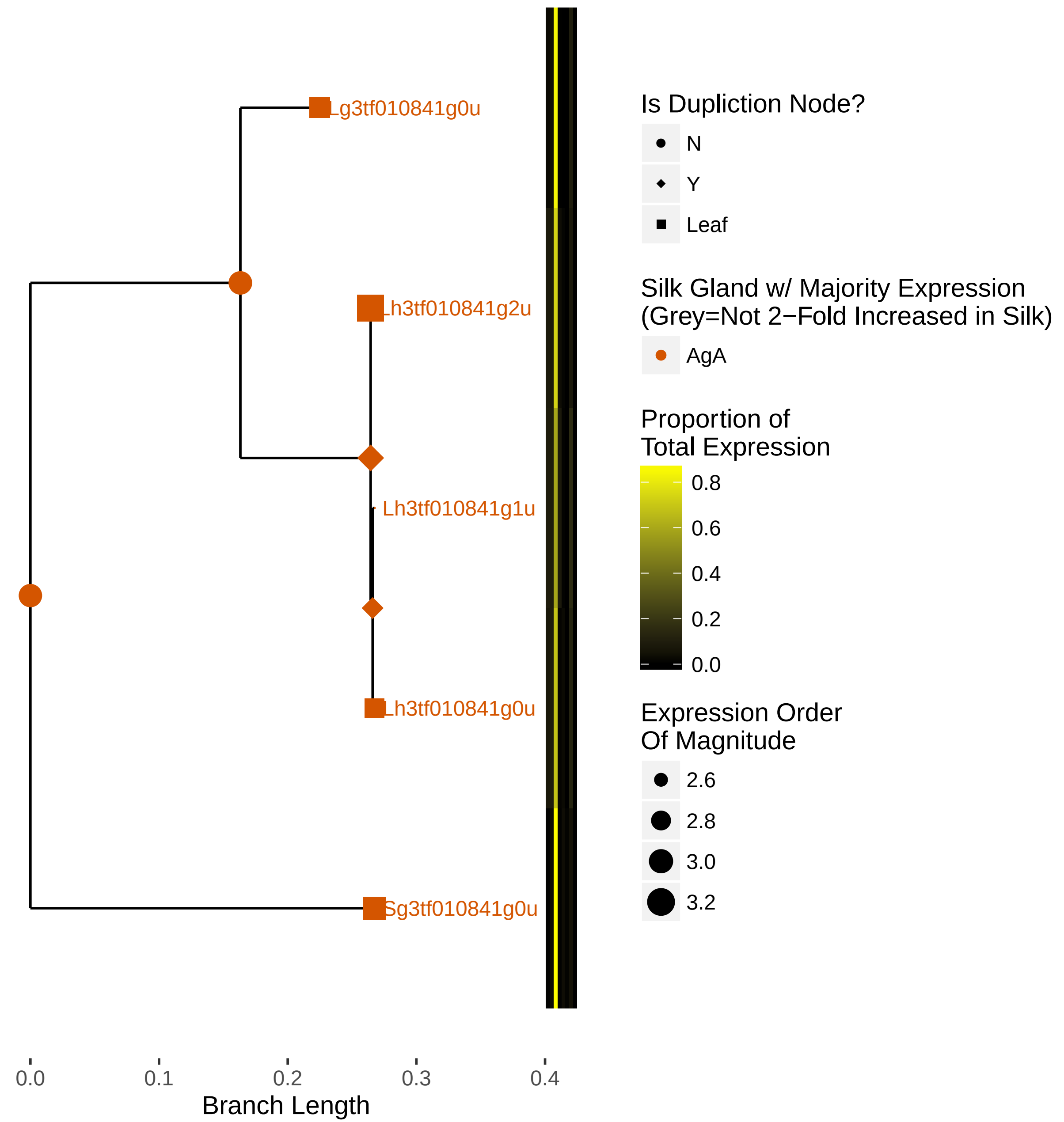

Lg3tf010841g0u
Lh3tf010841g2u
Lh3tf010841g1u
Lh3tf010841g0u
Sg3tf010841g0u
0.0
0.1
0.2
0.3
0.4
Branch Length
Is Dupliction Node?
N
Y
Leaf
Silk Gland w/ Majority Expression
(Grey=Not 2−Fold Increased in Silk)
AgA
Proportion of
Total Expression
0.8
0.6
0.4
0.2
0.0
Expression Order
Of Magnitude
2.6
2.8
3.0
3.2

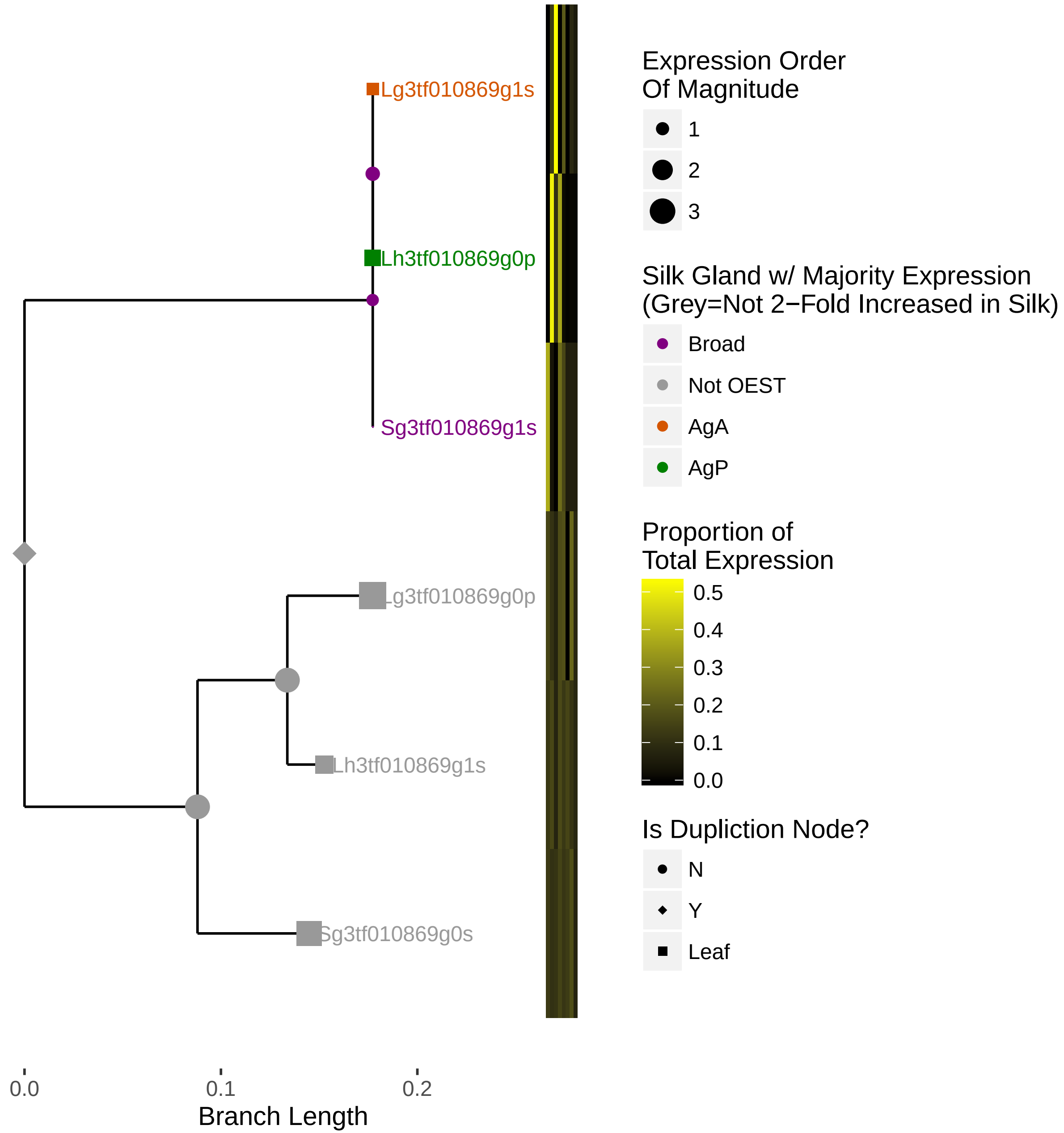
Lg3tf010869g1s
Lh3tf010869g0p
Sg3tf010869g1s
Lg3tf010869g0p
Lh3tf010869g1s
Sg3tf010869g0s
0.0
0.1
0.2
Branch Length
Expression Order
Of Magnitude
1
2
3
Silk Gland w/ Majority Expression
(Grey=Not 2−Fold Increased in Silk)
Broad
Not OEST
AgA
AgP
Proportion of
Total Expression
0.5
0.4
0.3
0.2
0.1
0.0
Is Dupliction Node?
N
Y
Leaf

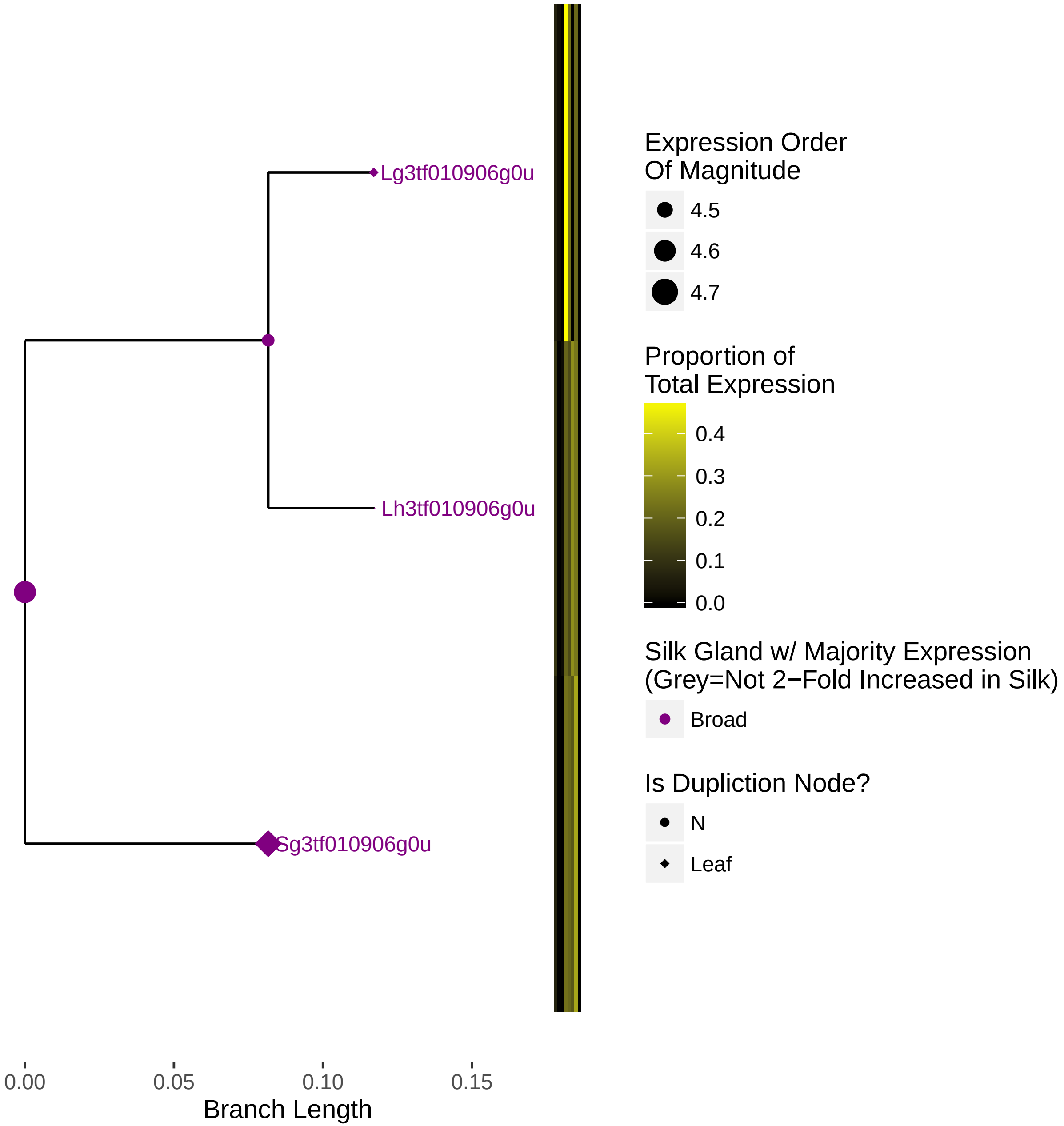

Lg3tf010906g0u
Lh3tf010906g0u
Sg3tf010906g0u
0.00
0.05
0.10
0.15
Branch Length
Expression Order Of Magnitude
4.5
4.6
4.7
Proportion of Total Expression
0.4
0.3
0.2
0.1
0.0
Silk Gland w/ Majority Expression (Grey=Not 2−Fold Increased in Silk)
Broad
Is Dupliction Node?
N
Leaf

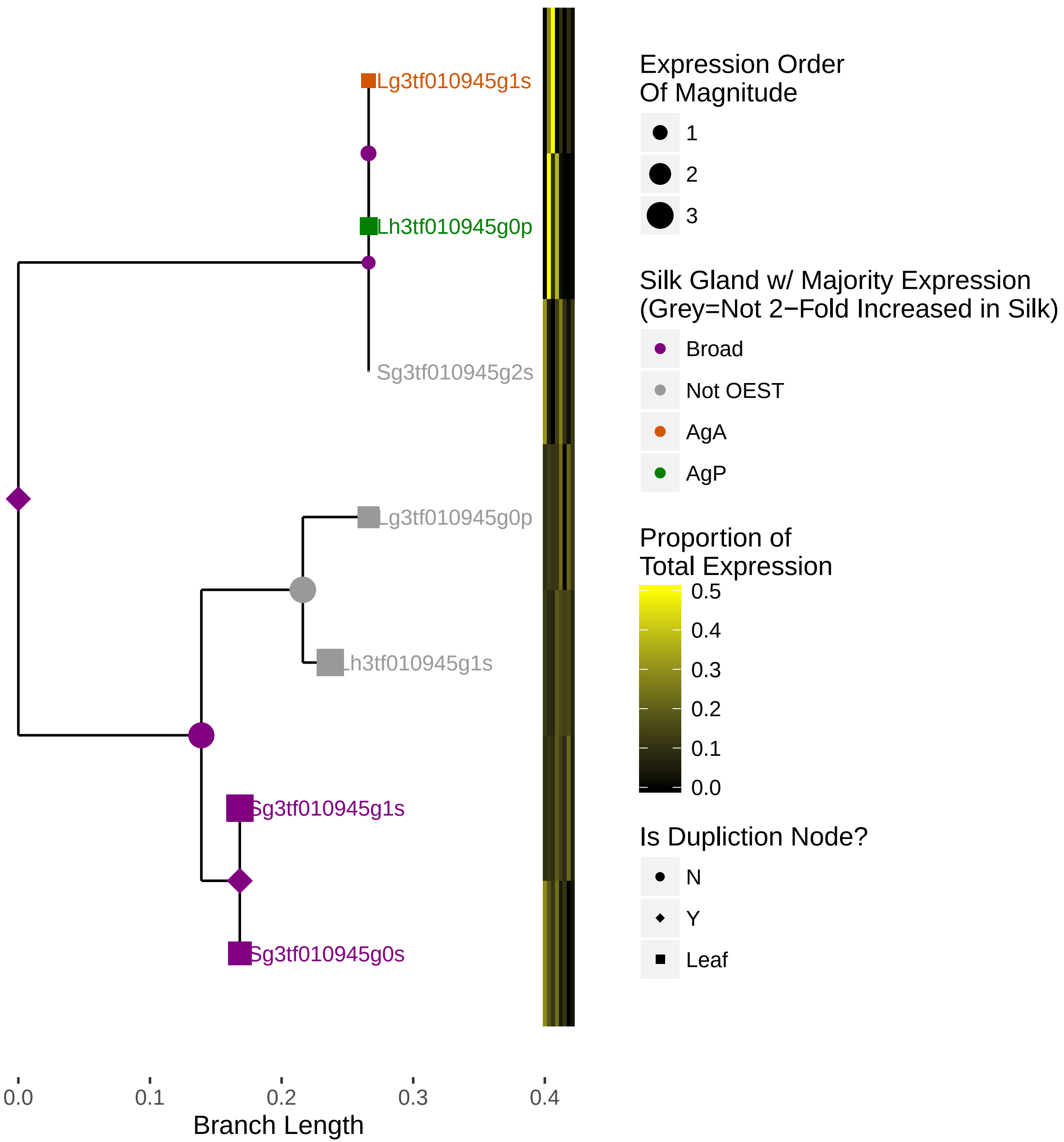

Lg3tf010945g1s
Lh3tf010945g0p
Sg3tf010945g2s
Lg3tf010945g0p
Lh3tf010945g1s
Sg3tf010945g1s
Sg3tf010945g0s
0.0
0.1
0.2
0.3
0.4
Branch Length
Expression Order
Of Magnitude
1
2
3
Silk Gland w/ Majority Expression
(Grey=Not 2−Fold Increased in Silk)
Broad
Not OEST
AgA
AgP
Proportion of
Total Expression
0.5
0.4
0.3
0.2
0.1
0.0
Is Dupliction Node?
N
Y
Leaf

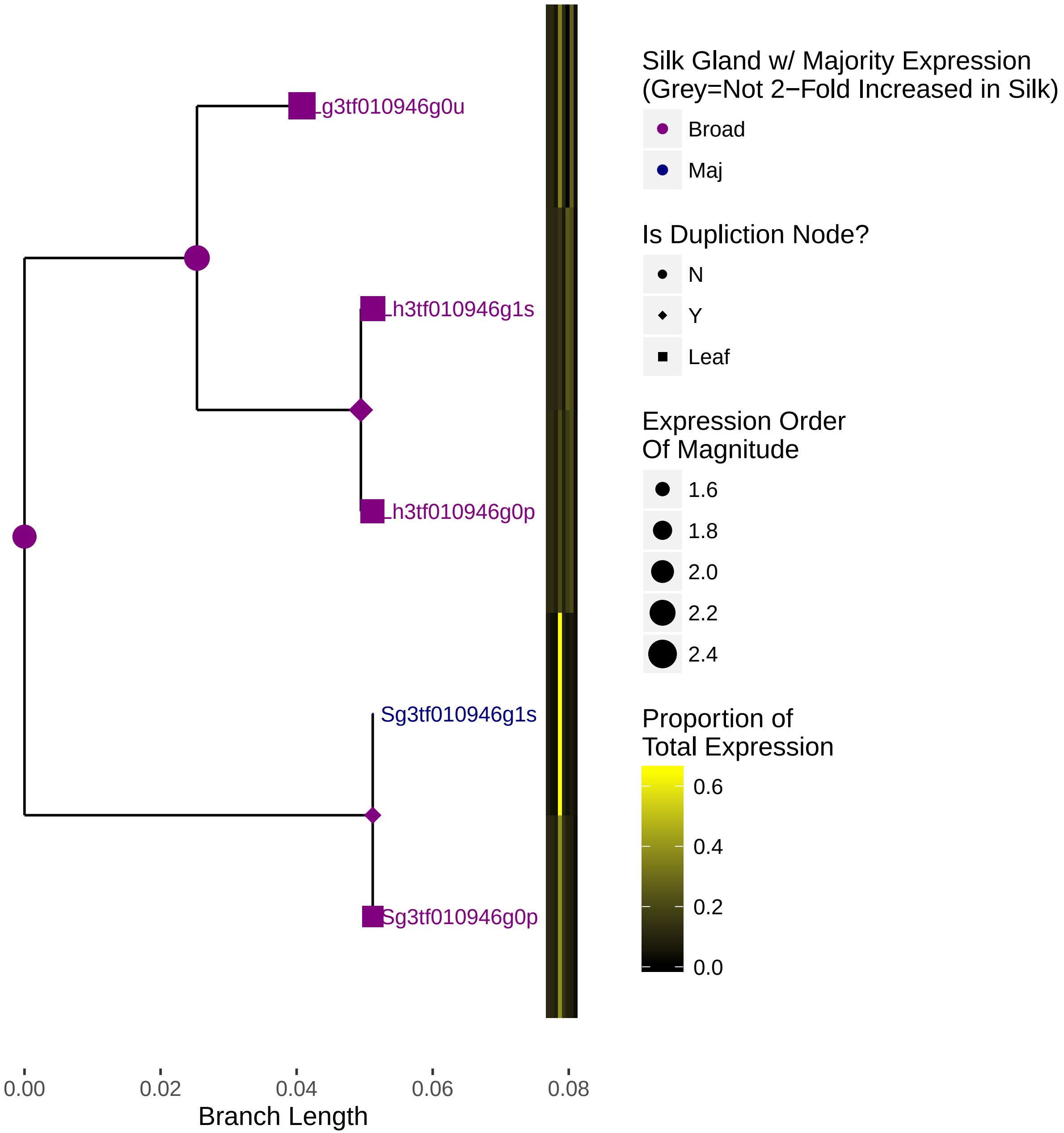

g3tf010946g0u
h3tf010946g1s
h3tf010946g0p
Sg3tf010946g1s
Sg3tf010946g0p
0.00
0.02
0.04
0.06
0.08
Branch Length
Silk Gland w/ Majority Expression
(Grey=Not 2−Fold Increased in Silk)
Broad
Maj
Is Dupliction Node?
N
Y
Leaf
Expression Order
Of Magnitude
1.6
1.8
2.0
2.2
2.4
Proportion of
Total Expression
0.6
0.4
0.2
0.0

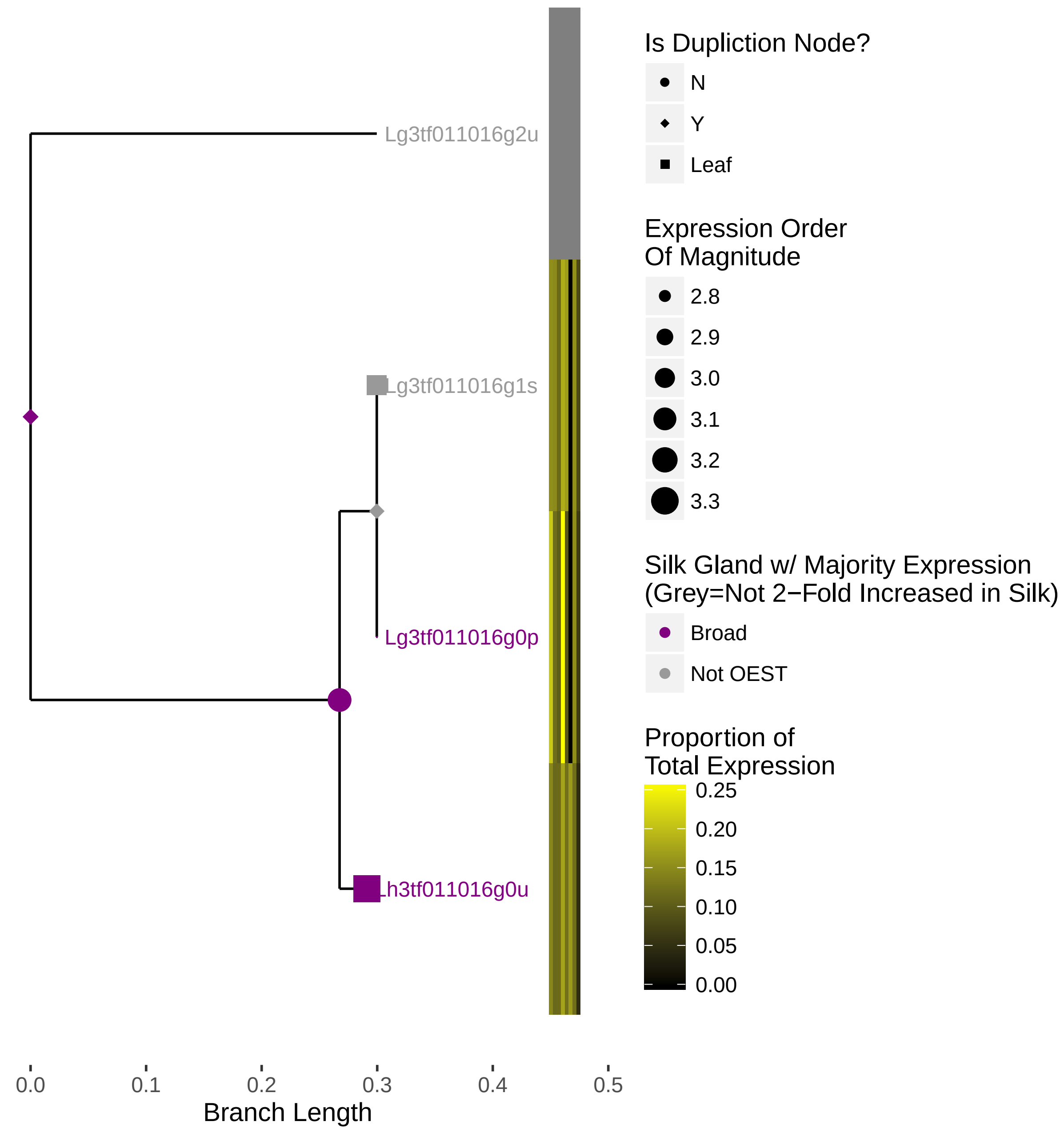

Is Dupliction Node?
N
Y
Leaf
Expression Order Of Magnitude
2.8
2.9
3.0
3.1
3.2
3.3
Silk Gland w/ Majority Expression (Grey=Not 2−Fold Increased in Silk)
Broad
Not OEST
Proportion of Total Expression
0.25
0.20
0.15
0.10
0.05
0.00
Lg3tf011016g2u
Lg3tf011016g1s
Lg3tf011016g0p
Lh3tf011016g0u
0.0
0.1
0.2
0.3
0.4
0.5
Branch Length

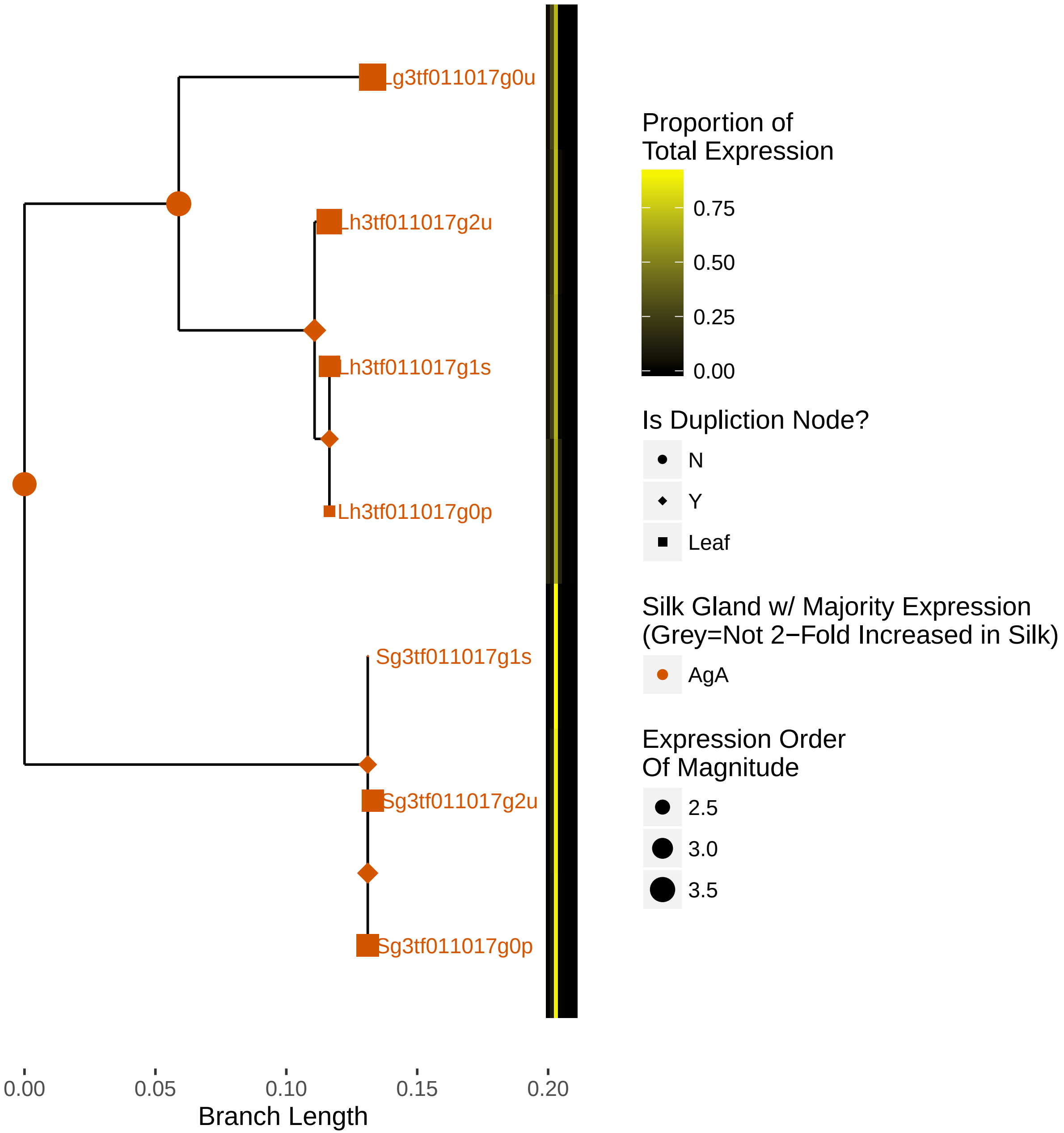

Lg3tf011017g0u
Lh3tf011017g2u
Lh3tf011017g1s
Lh3tf011017g0p
Sg3tf011017g1s
Sg3tf011017g2u
Sg3tf011017g0p
0.00
0.05
0.10
0.15
0.20
Branch Length
Proportion of
Total Expression
0.75
0.50
0.25
0.00
Is Dupliction Node?
N
Y
Leaf
Silk Gland w/ Majority Expression
(Grey=Not 2−Fold Increased in Silk)
AgA
Expression Order
Of Magnitude
2.5
3.0
3.5

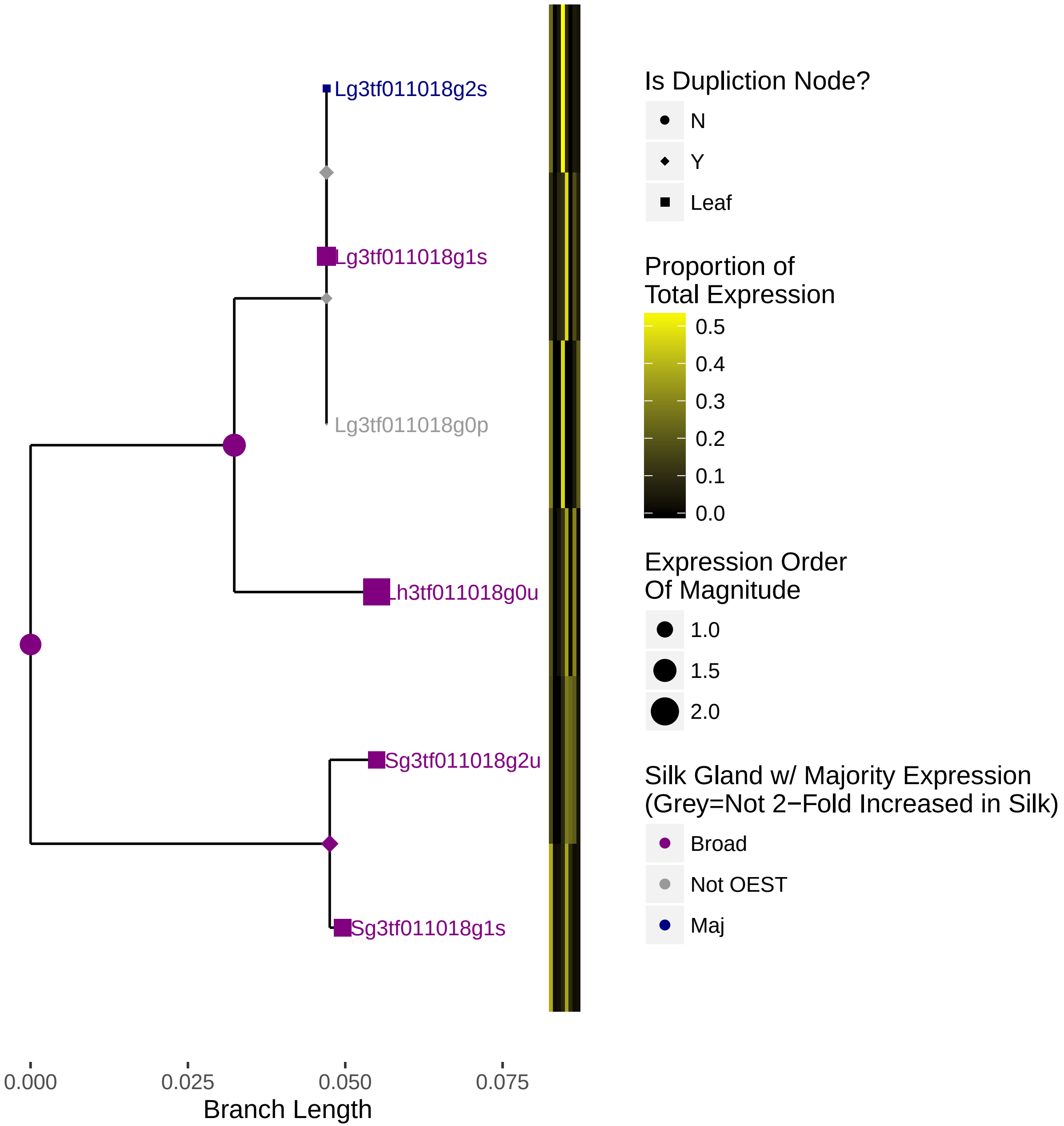

Lg3tf011018g2s
Lg3tf011018g1s
Lg3tf011018g0p
Lh3tf011018g0u
Sg3tf011018g2u
Sg3tf011018g1s
0.000
0.025
0.050
0.075
Branch Length
Is Dupliction Node?
N
Y
Leaf
Proportion of Total Expression
0.5
0.4
0.3
0.2
0.1
0.0
Expression Order Of Magnitude
1.0
1.5
2.0
Silk Gland w/ Majority Expression (Grey=Not 2−Fold Increased in Silk)
Broad
Not OEST
Maj

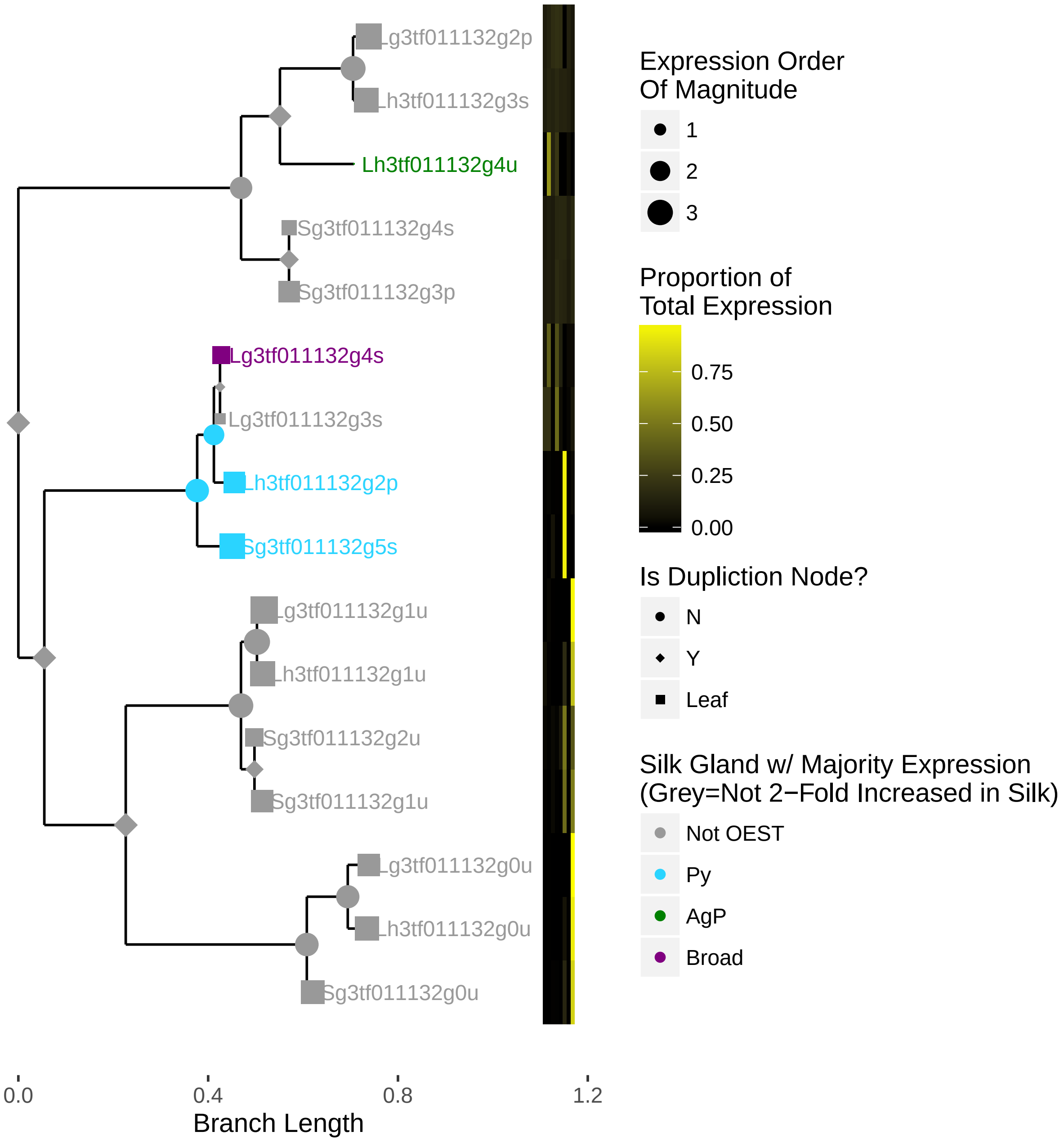

g3tf011132g2p
h3tf011132g3s
Lh3tf011132g4u
Sg3tf011132g4s
Sg3tf011132g3p
Lg3tf011132g4s
Lg3tf011132g3s
Lh3tf011132g2p
Sg3tf011132g5s
g3tf011132g1u
h3tf011132g1u
Sg3tf011132g2u
Sg3tf011132g1u
g3tf011132g0u
h3tf011132g0u
Sg3tf011132g0u
0.0
0.4
0.8
1.2
Branch Length
Expression Order Of Magnitude
1
2
3
Proportion of Total Expression
0.75
0.50
0.25
0.00
Is Dupliction Node?
N
Y
Leaf
Silk Gland w/ Majority Expression (Grey=Not 2−Fold Increased in Silk)
Not OEST
Py
AgP
Broad

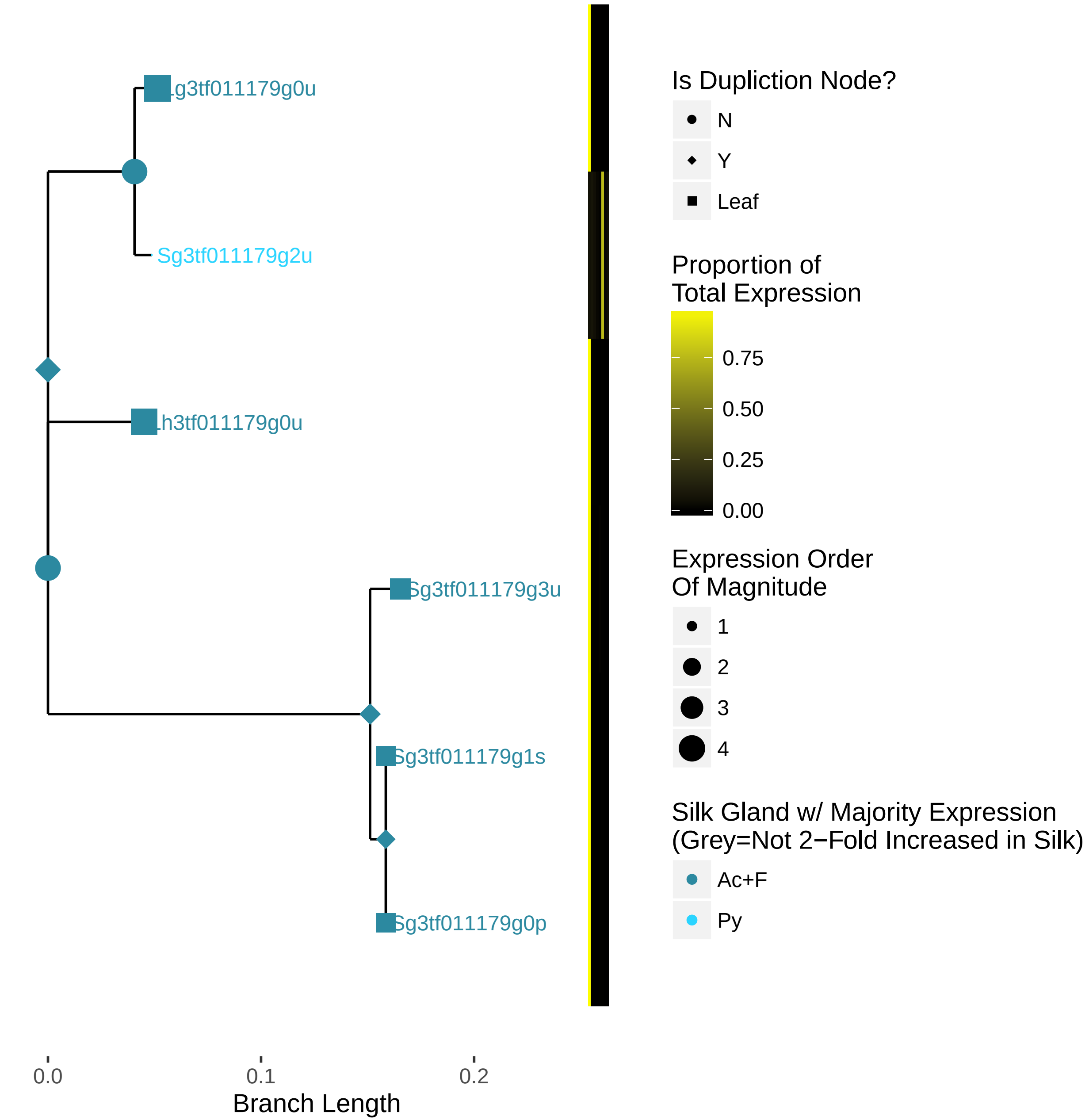

g3tf011179g0u
Sg3tf011179g2u
h3tf011179g0u
Sg3tf011179g3u
Sg3tf011179g1s
Sg3tf011179g0p
0.0
0.1
0.2
Branch Length
Is Dupliction Node?
N
Y
Leaf
Proportion of
Total Expression
0.75
0.50
0.25
0.00
Expression Order
Of Magnitude
1
2
3
4
Silk Gland w/ Majority Expression
(Grey=Not 2−Fold Increased in Silk)
Ac+F
Py

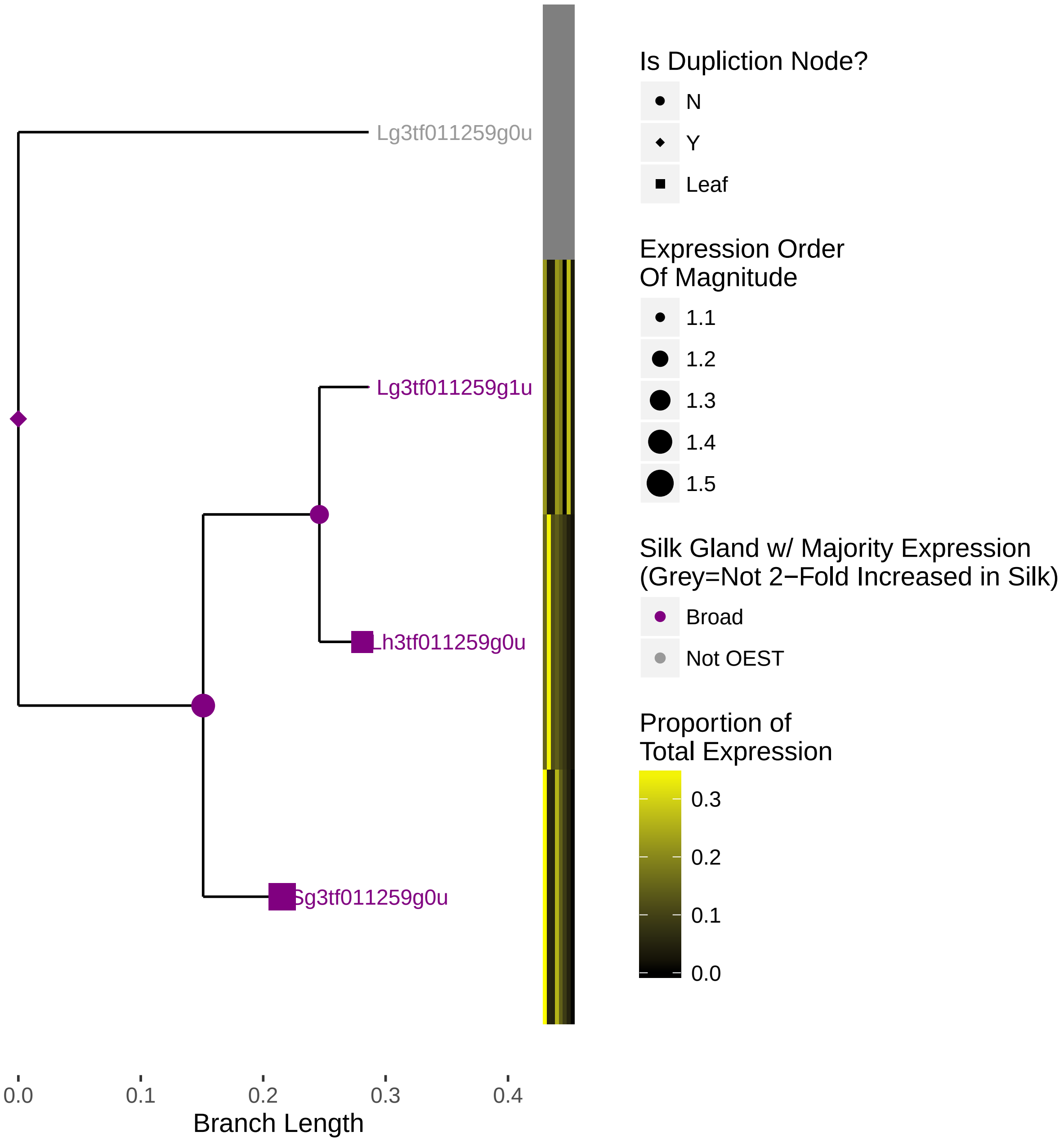

Lg3tf011259g0u
Lg3tf011259g1u
Lh3tf011259g0u
Sg3tf011259g0u
Is Dupliction Node?
N
Y
Leaf
Expression Order
Of Magnitude
1.1
1.2
1.3
1.4
1.5
Silk Gland w/ Majority Expression
(Grey=Not 2−Fold Increased in Silk)
Broad
Not OEST
Proportion of
Total Expression
0.3
0.2
0.1
0.0
0.0
0.1
0.2
0.3
0.4
Branch Length

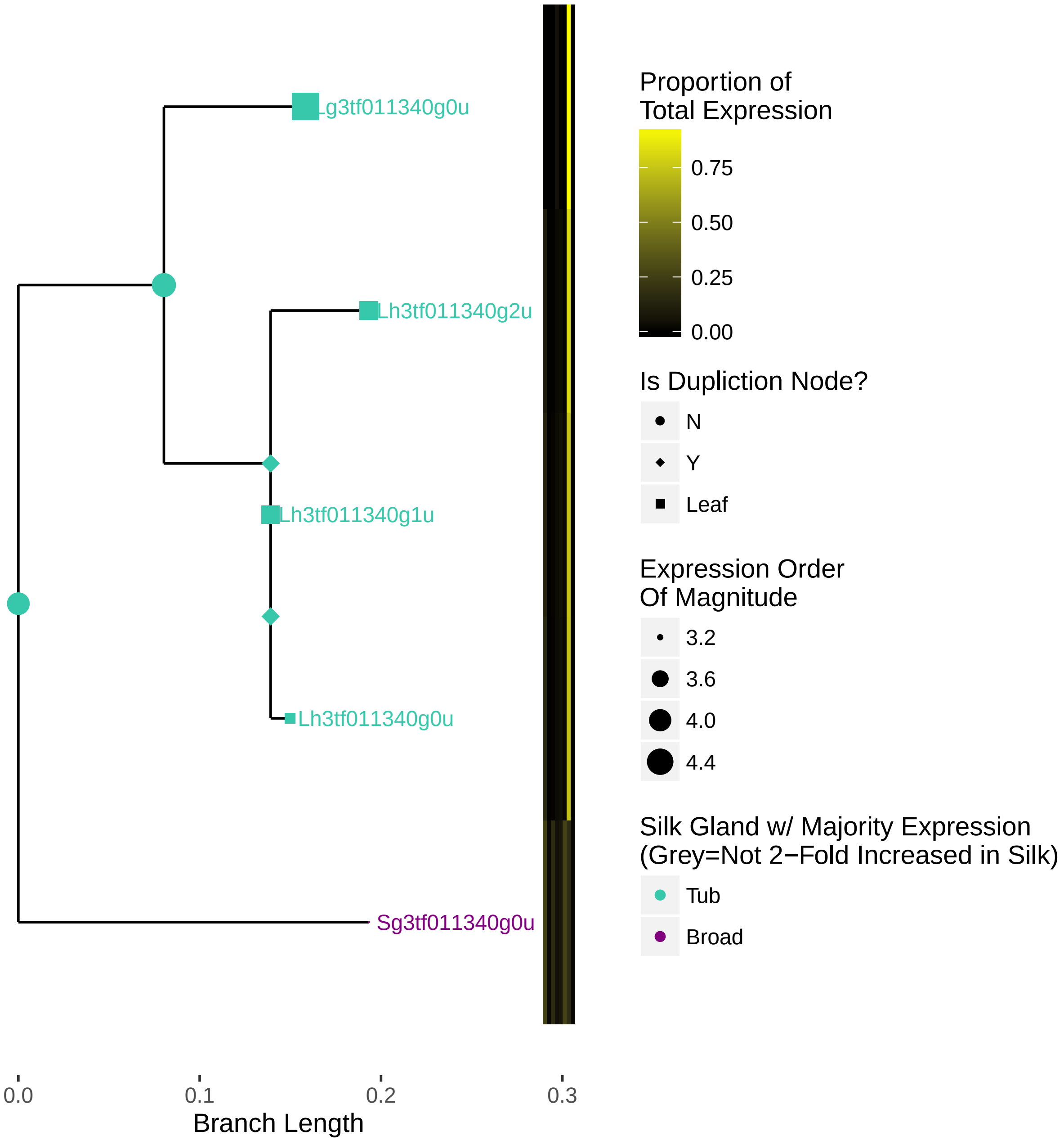

g3tf011340g0u
Lh3tf011340g2u
Lh3tf011340g1u
Lh3tf011340g0u
Sg3tf011340g0u
0.0
0.1
0.2
0.3
Branch Length
Proportion of
Total Expression
0.75
0.50
0.25
0.00
Is Dupliction Node?
N
Y
Leaf
Expression Order
Of Magnitude
3.2
3.6
4.0
4.4
Silk Gland w/ Majority Expression
(Grey=Not 2−Fold Increased in Silk)
Tub
Broad

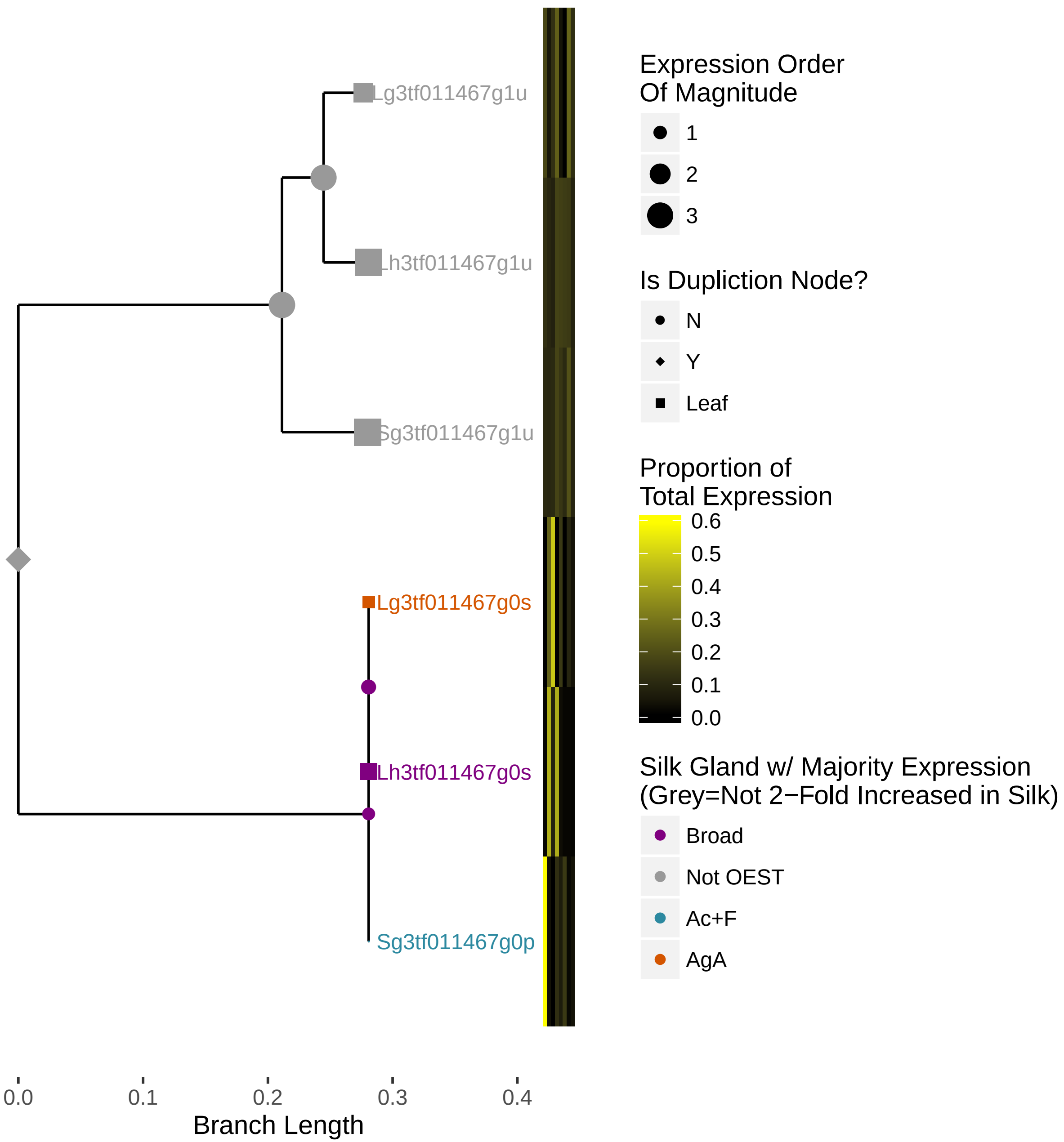

Lg3tf011467g1u
Lh3tf011467g1u
Sg3tf011467g1u
Lg3tf011467g0s
Lh3tf011467g0s
Sg3tf011467g0p
0.0
0.1
0.2
0.3
0.4
Branch Length
Expression Order Of Magnitude
1
2
3
Is Dupliction Node?
N
Y
Leaf
Proportion of Total Expression
0.6
0.5
0.4
0.3
0.2
0.1
0.0
Silk Gland w/ Majority Expression (Grey=Not 2−Fold Increased in Silk)
Broad
Not OEST
Ac+F
AgA

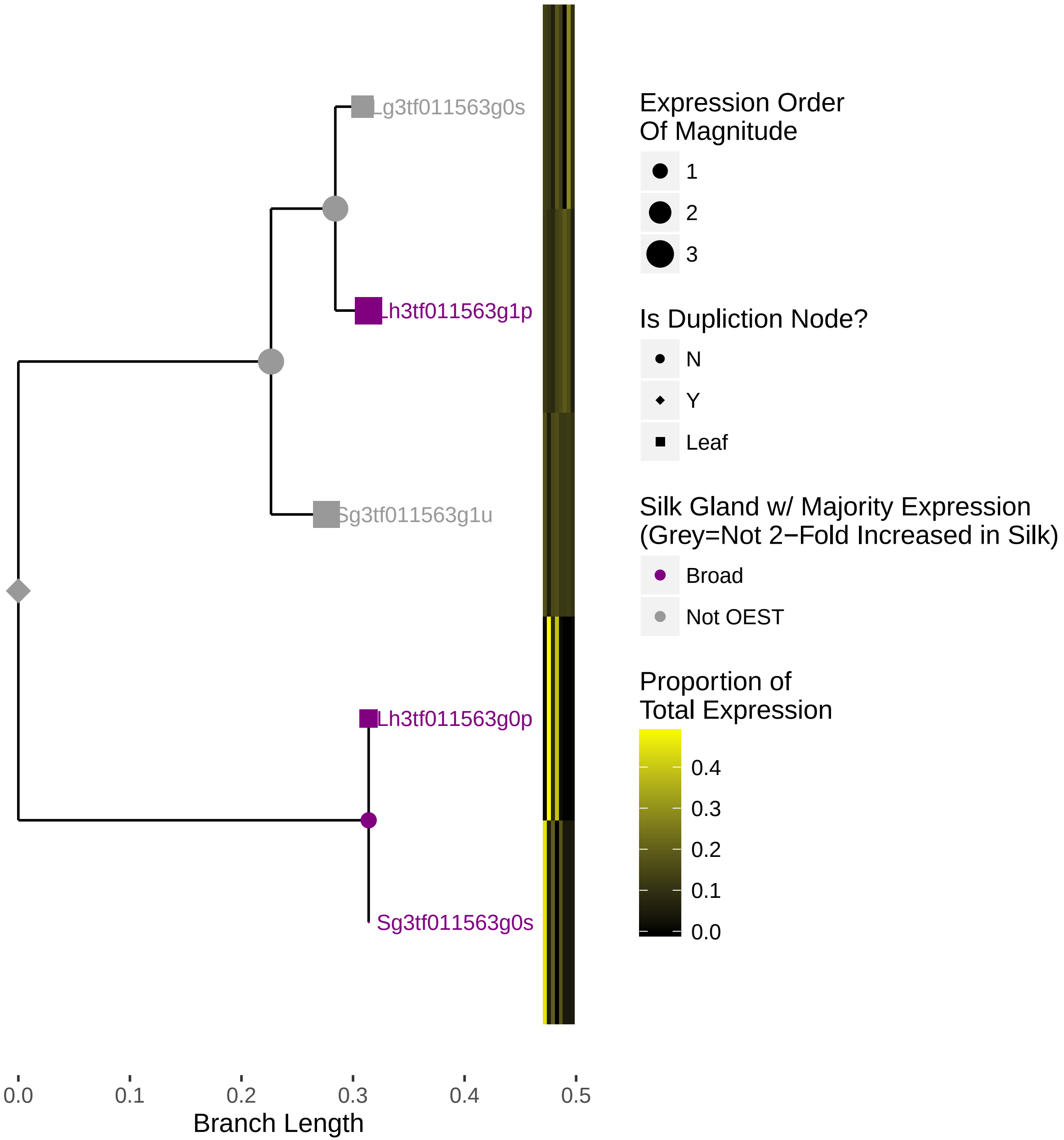

Lg3tf011563g0s
Lh3tf011563g1p
Sg3tf011563g1u
Lh3tf011563g0p
Sg3tf011563g0s
0.0
0.1
0.2
0.3
0.4
0.5
Branch Length
Expression Order Of Magnitude
1
2
3
Is Dupliction Node?
N
Y
Leaf
Silk Gland w/ Majority Expression (Grey=Not 2−Fold Increased in Silk)
Broad
Not OEST
Proportion of Total Expression
0.4
0.3
0.2
0.1
0.0

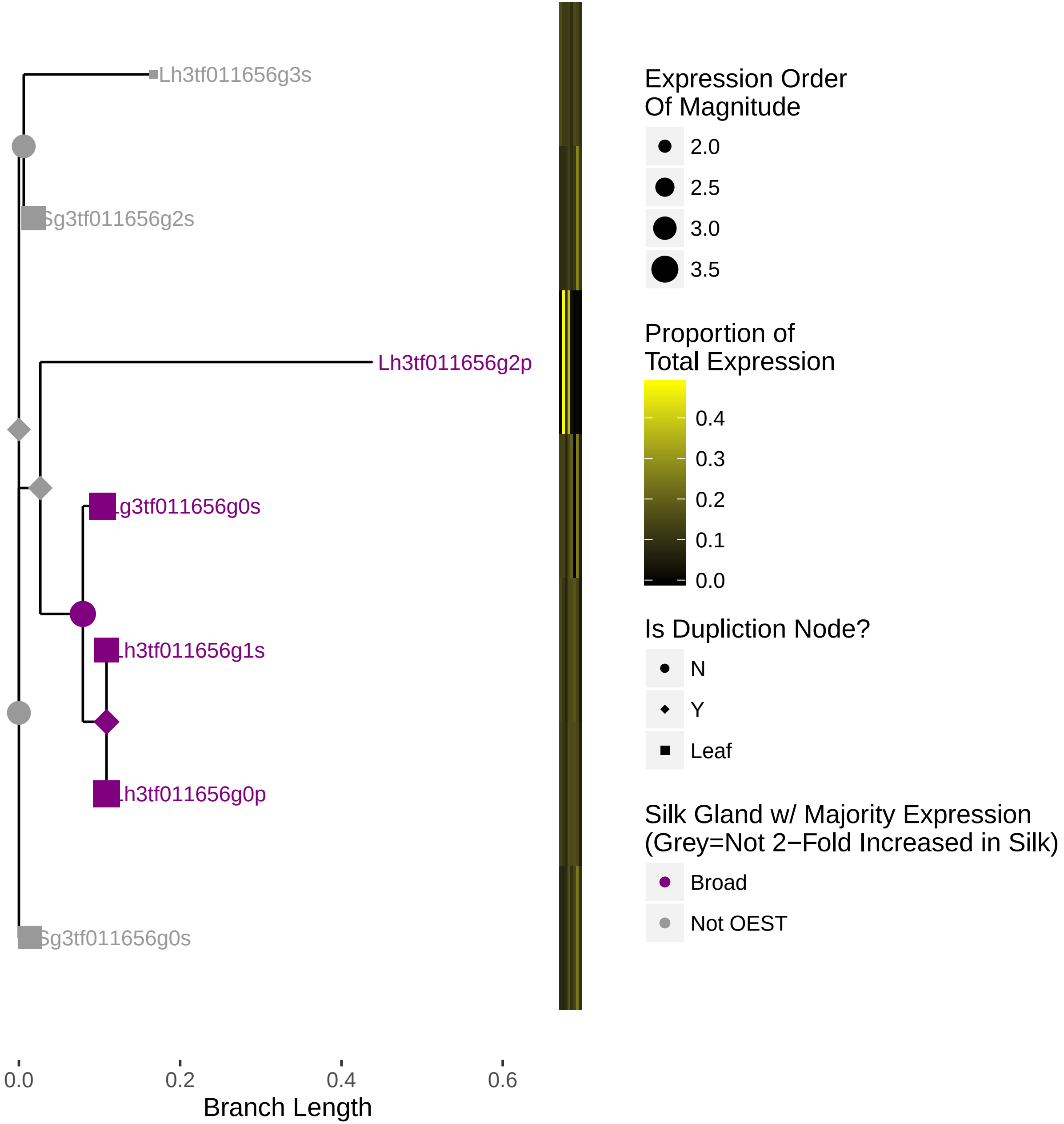

Lh3tf011656g3s
Sg3tf011656g2s
Lh3tf011656g2p
Lg3tf011656g0s
Lh3tf011656g1s
Lh3tf011656g0p
Sg3tf011656g0s
0.0
0.2
0.4
0.6
Branch Length
Expression Order Of Magnitude
2.0
2.5
3.0
3.5
Proportion of Total Expression
0.4
0.3
0.2
0.1
0.0
Is Dupliction Node?
N
Y
Leaf
Silk Gland w/ Majority Expression (Grey=Not 2−Fold Increased in Silk)
Broad
Not OEST

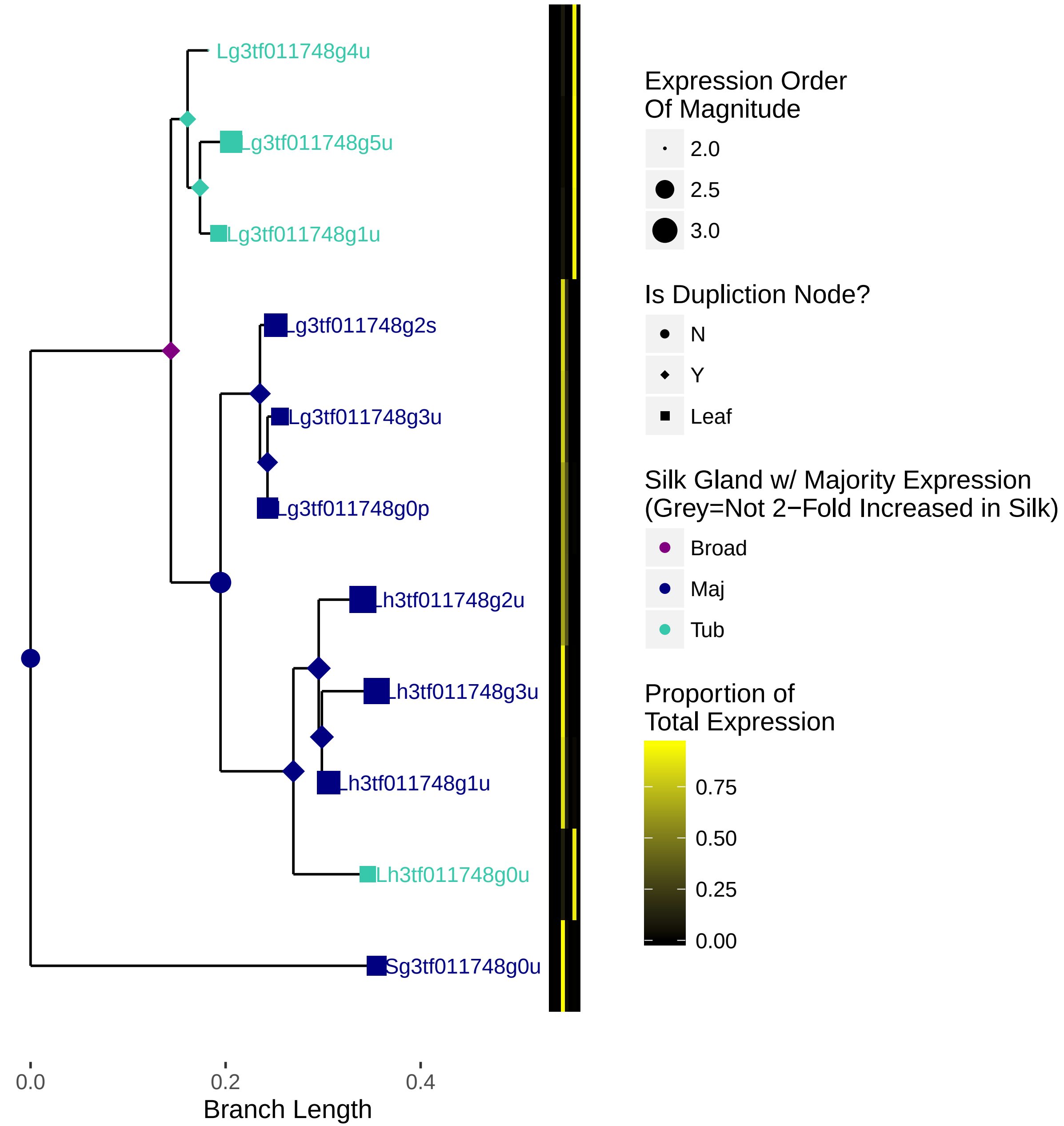

Lg3tf011748g4u
g3tf011748g5u
Lg3tf011748g1u
g3tf011748g2s
Lg3tf011748g3u
g3tf011748g0p
h3tf011748g2u
h3tf011748g3u
h3tf011748g1u
Lh3tf011748g0u
Sg3tf011748g0u
0.0
0.2
0.4
Branch Length
Expression Order
Of Magnitude
2.0
2.5
3.0
Is Dupliction Node?
N
Y
Leaf
Silk Gland w/ Majority Expression
(Grey=Not 2−Fold Increased in Silk)
Broad
Maj
Tub
Proportion of
Total Expression
0.75
0.50
0.25
0.00

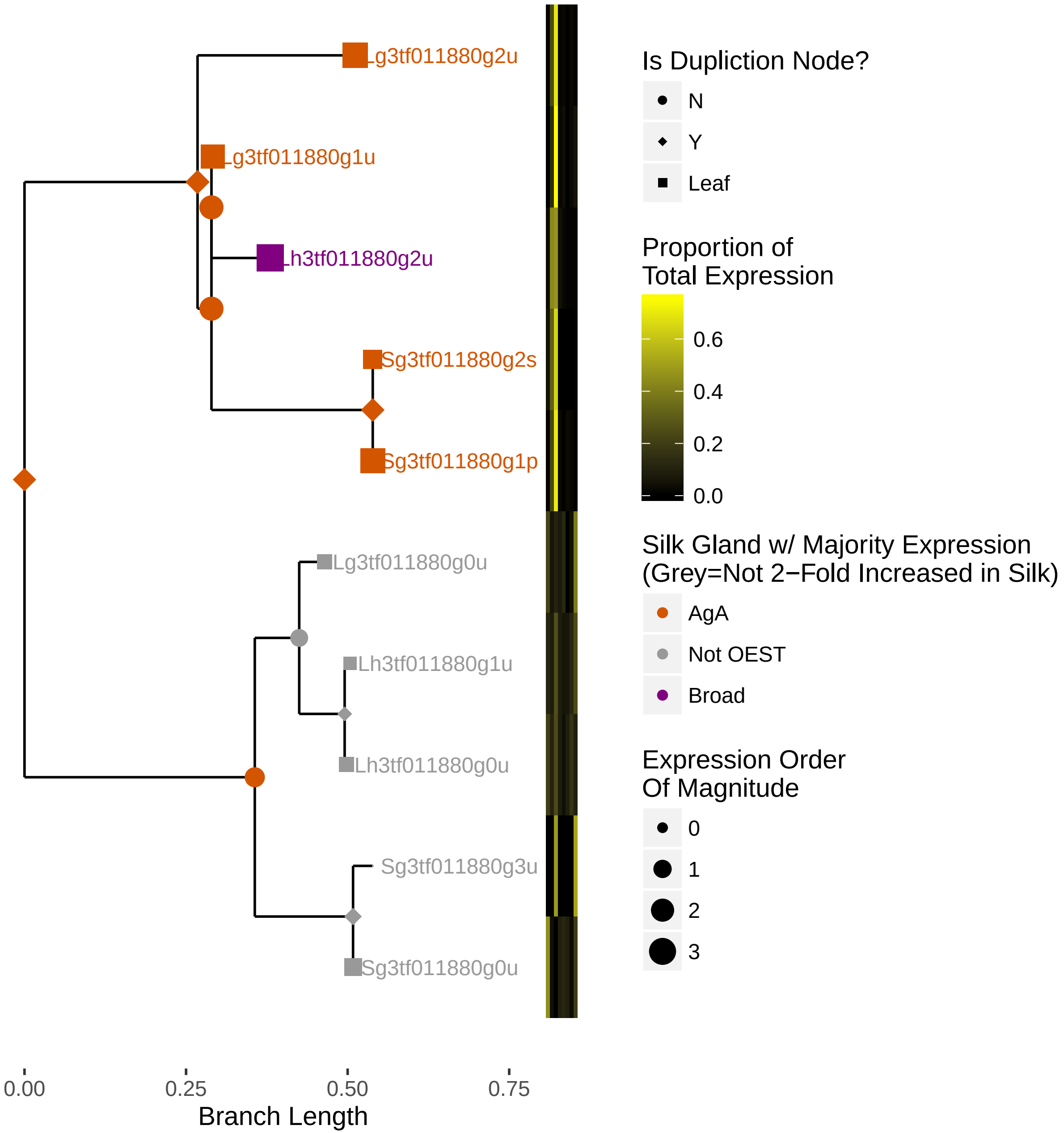

Lg3tf011880g2u
Lg3tf011880g1u
Lh3tf011880g2u
Sg3tf011880g2s
Sg3tf011880g1p
Lg3tf011880g0u
Lh3tf011880g1u
Lh3tf011880g0u
Sg3tf011880g3u
Sg3tf011880g0u
0.00
0.25
0.50
0.75
Branch Length
Is Dupliction Node?
N
Y
Leaf
Proportion of
Total Expression
0.6
0.4
0.2
0.0
Silk Gland w/ Majority Expression
(Grey=Not 2−Fold Increased in Silk)
AgA
Not OEST
Broad
Expression Order
Of Magnitude
0
1
2
3

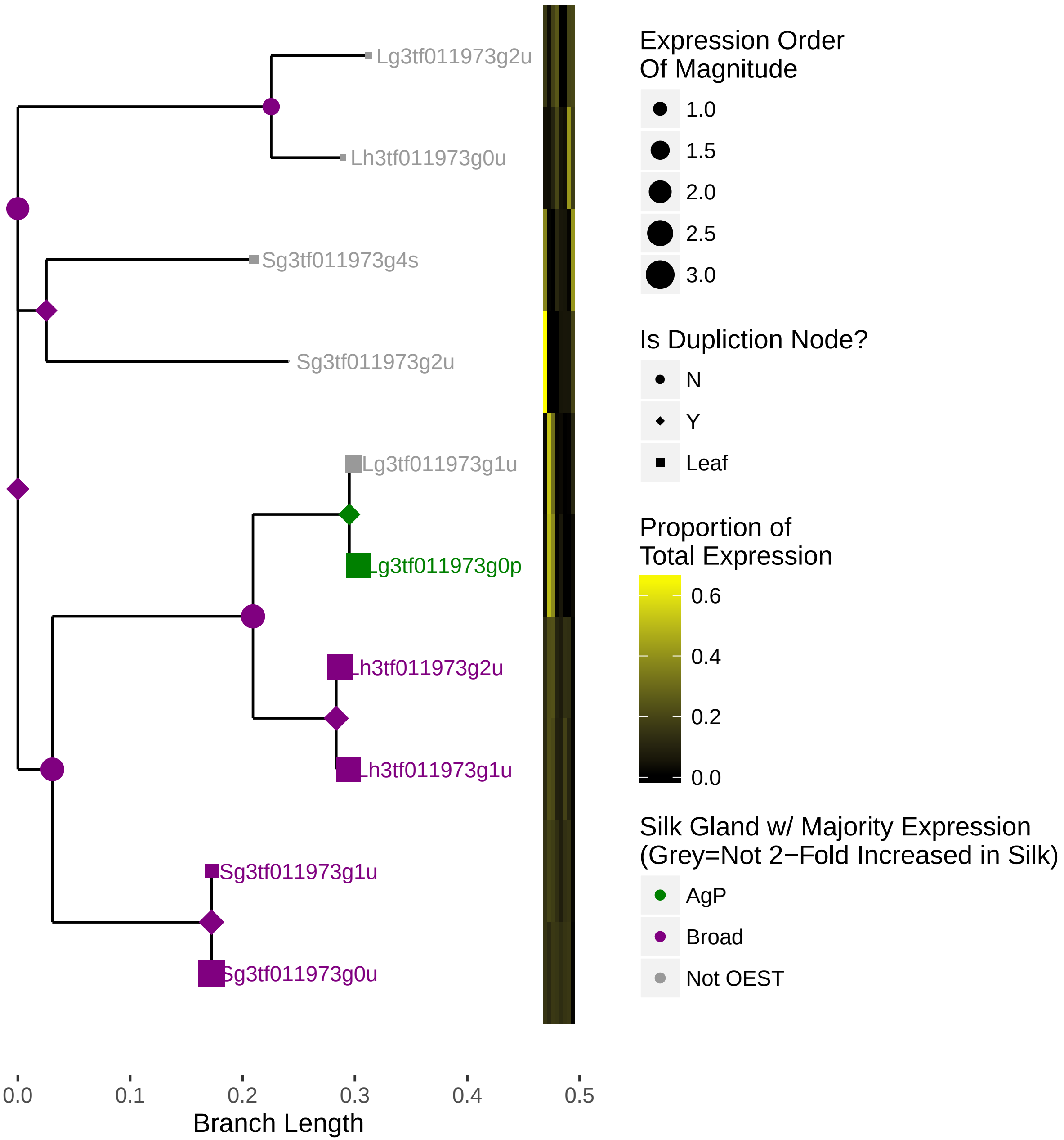
Lg3tf011973g2u
Lh3tf011973g0u
Sg3tf011973g4s
Sg3tf011973g2u
Lg3tf011973g1u
Lg3tf011973g0p
Lh3tf011973g2u
Lh3tf011973g1u
Sg3tf011973g1u
Sg3tf011973g0u
0.0
0.1
0.2
0.3
0.4
0.5
Branch Length
Expression Order Of Magnitude
1.0
1.5
2.0
2.5
3.0
Is Dupliction Node?
N
Y
Leaf
Proportion of Total Expression
0.6
0.4
0.2
0.0
Silk Gland w/ Majority Expression (Grey=Not 2−Fold Increased in Silk)
AgP
Broad
Not OEST

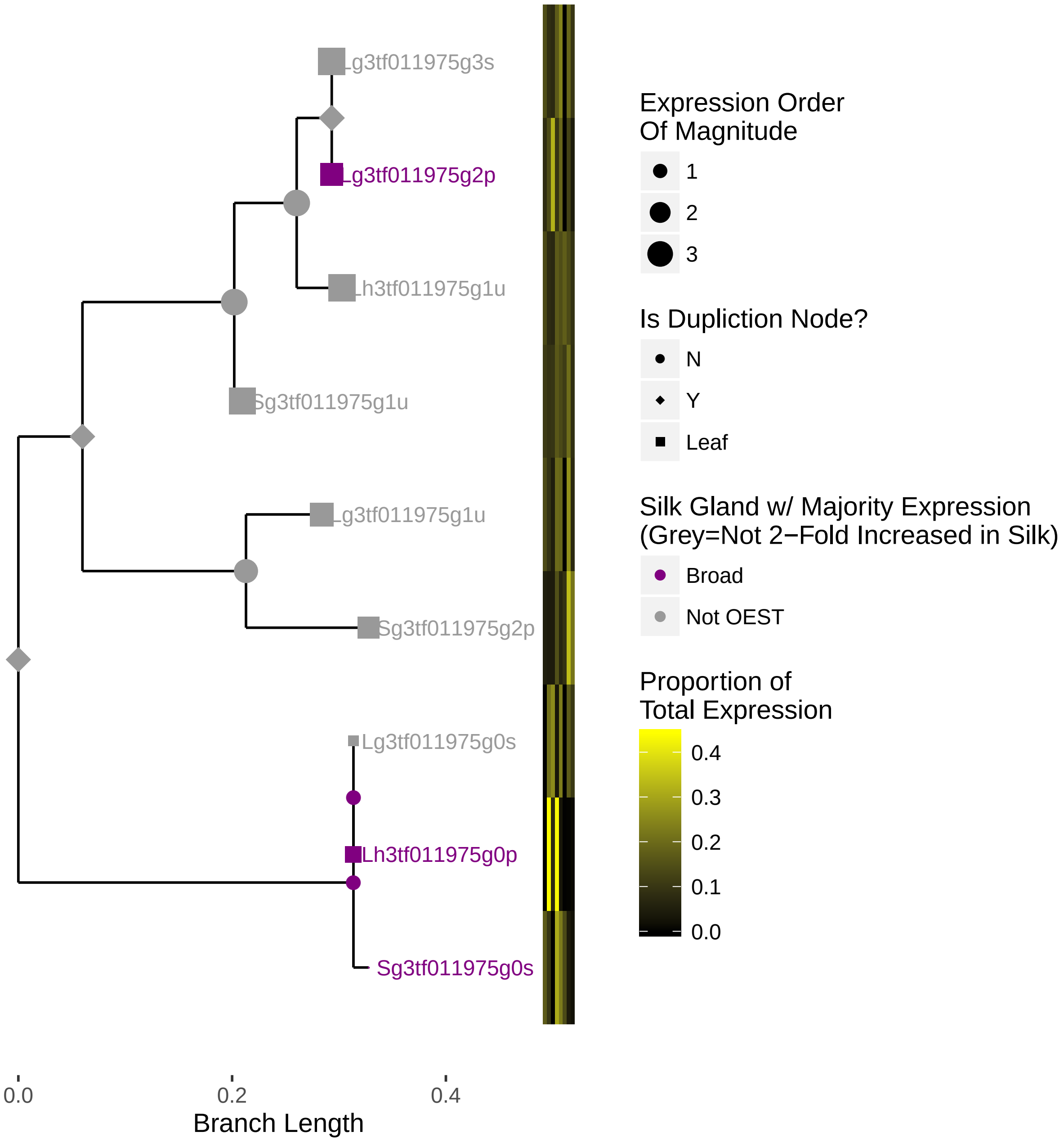

g3tf011975g3s
g3tf011975g2p
h3tf011975g1u
g3tf011975g1u
g3tf011975g1u
Sg3tf011975g2p
Lg3tf011975g0s
Lh3tf011975g0p
Sg3tf011975g0s
0.0
0.2
0.4
Branch Length
Expression Order
Of Magnitude
1
2
3
Is Dupliction Node?
N
Y
Leaf
Silk Gland w/ Majority Expression
(Grey=Not 2−Fold Increased in Silk)
Broad
Not OEST
Proportion of
Total Expression
0.4
0.3
0.2
0.1
0.0

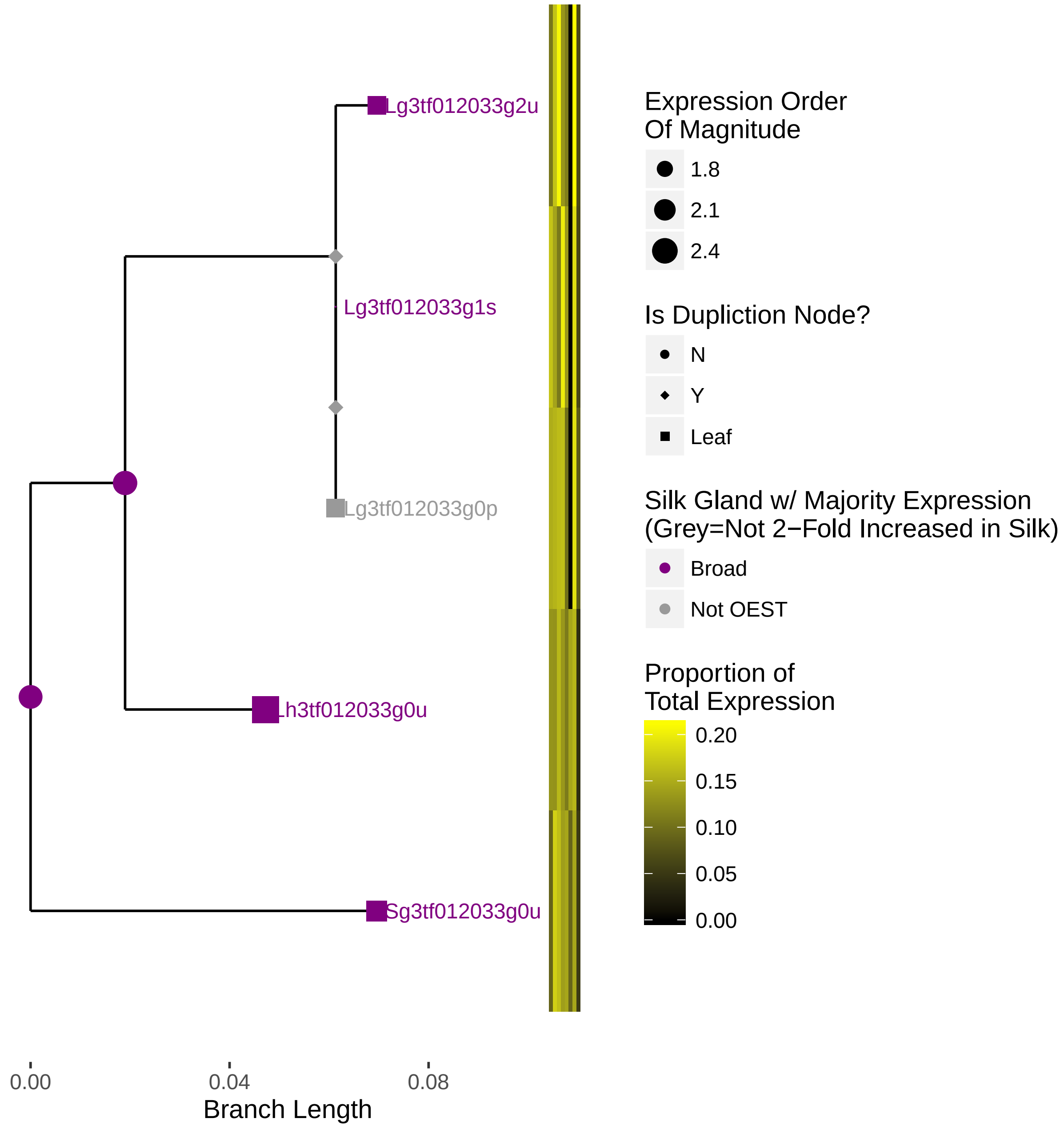

Lg3tf012033g2u
Lg3tf012033g1s
Lg3tf012033g0p
Lh3tf012033g0u
Sg3tf012033g0u
0.00
0.04
0.08
Branch Length
Expression Order
Of Magnitude
1.8
2.1
2.4
Is Dupliction Node?
N
Y
Leaf
Silk Gland w/ Majority Expression
(Grey=Not 2−Fold Increased in Silk)
Broad
Not OEST
Proportion of
Total Expression
0.20
0.15
0.10
0.05
0.00

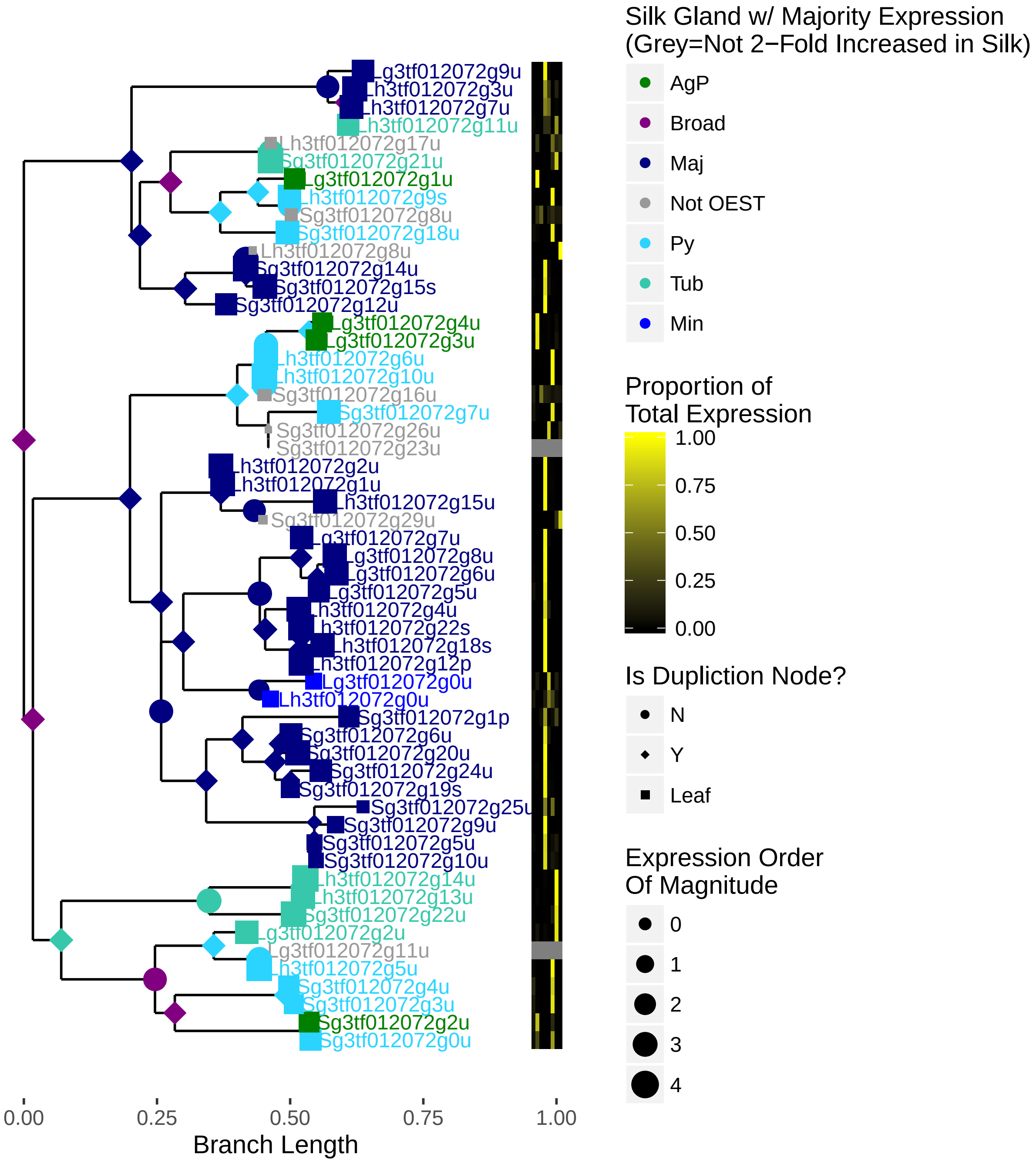

g3tf012072g9u
h3tf012072g3u
Lh3tf012072g7u
Lh3tf012072g11u
Lh3tf012072g17u
Sg3tf012072g21u
Lg3tf012072g1u
Lh3tf012072g9s
Sg3tf012072g8u
Sg3tf012072g18u
Lh3tf012072g8u
Sg3tf012072g14u
Sg3tf012072g15s
Sg3tf012072g12u
Lg3tf012072g4u
Lg3tf012072g3u
Lh3tf012072g6u
Lh3tf012072g10u
Sg3tf012072g16u
Sg3tf012072g7u
Sg3tf012072g26u
Sg3tf012072g23u
Lh3tf012072g2u
Lh3tf012072g1u
Lh3tf012072g15u
Sg3tf012072g29u
Lg3tf012072g7u
Lg3tf012072g8u
Lg3tf012072g6u
Lg3tf012072g5u
Lh3tf012072g4u
Lh3tf012072g22s
Lh3tf012072g18s
Lh3tf012072g12p
Lg3tf012072g0u
Lh3tf012072g0u
Sg3tf012072g1p
Sg3tf012072g6u
Sg3tf012072g20u
Sg3tf012072g24u
Sg3tf012072g19s
Sg3tf012072g25u
Sg3tf012072g9u
Sg3tf012072g5u
Sg3tf012072g10u
Lh3tf012072g14u
Lh3tf012072g13u
Sg3tf012072g22u
Lg3tf012072g2u
Lg3tf012072g11u
Lh3tf012072g5u
Sg3tf012072g4u
Sg3tf012072g3u
Sg3tf012072g2u
Sg3tf012072g0u
0.00
0.25
0.50
0.75
1.00
Branch Length
Silk Gland w/ Majority Expression
(Grey=Not 2−Fold Increased in Silk)
AgP
Broad
Maj
Not OEST
Py
Tub
Min
Proportion of
Total Expression
1.00
0.75
0.50
0.25
0.00
Is Dupliction Node?
N
Y
Leaf
Expression Order
Of Magnitude
0
1
2
3
4

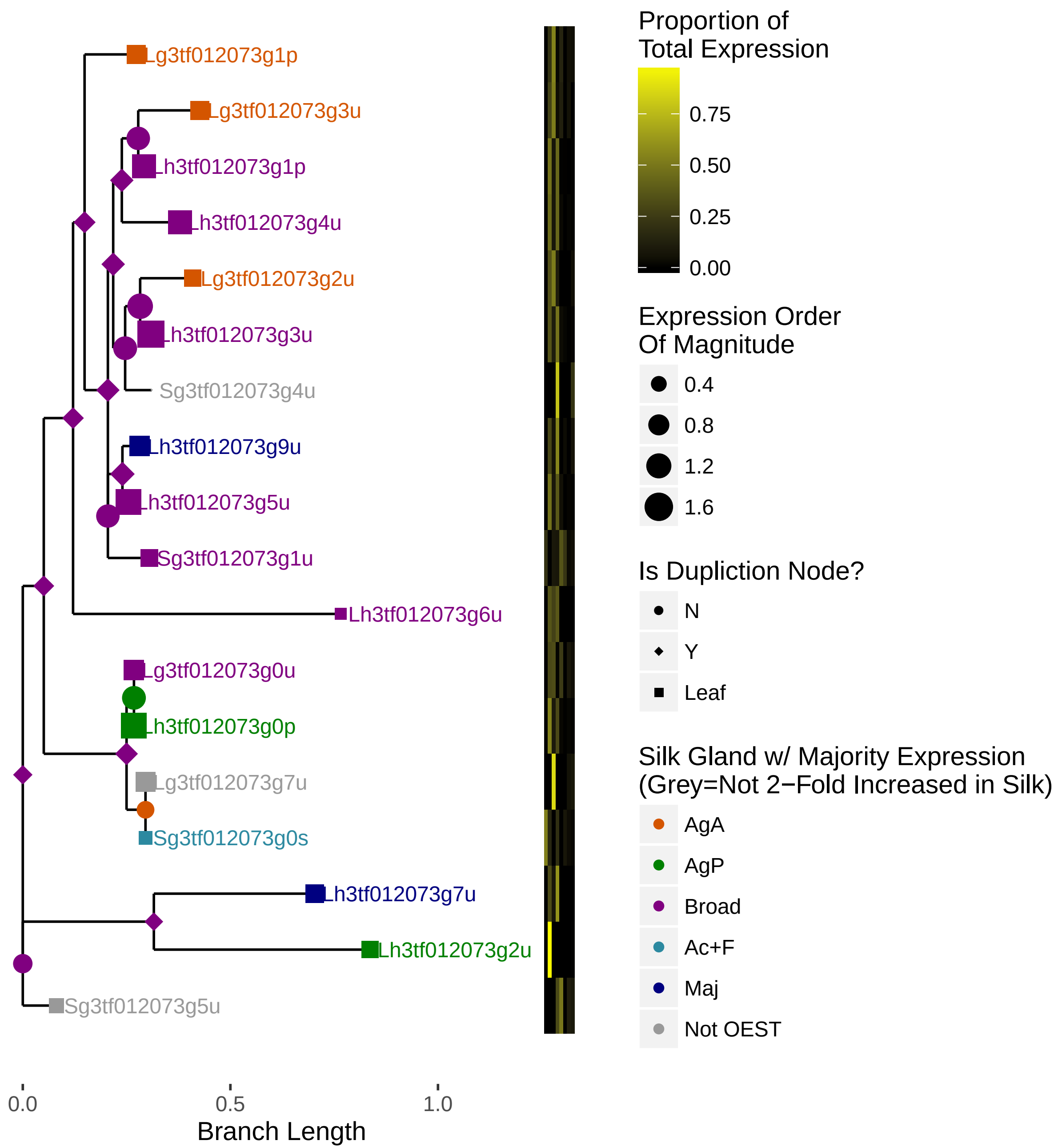

Lg3tf012073g1p
Lg3tf012073g3u
Lh3tf012073g1p
Lh3tf012073g4u
Lg3tf012073g2u
Lh3tf012073g3u
Sg3tf012073g4u
Lh3tf012073g9u
Lh3tf012073g5u
Sg3tf012073g1u
Lh3tf012073g6u
Lg3tf012073g0u
Lh3tf012073g0p
Lg3tf012073g7u
Sg3tf012073g0s
Lh3tf012073g7u
Lh3tf012073g2u
Sg3tf012073g5u
0.0
0.5
1.0
Branch Length
Proportion of Total Expression
0.75
0.50
0.25
0.00
Expression Order Of Magnitude
0.4
0.8
1.2
1.6
Is Dupliction Node?
N
Y
Leaf
Silk Gland w/ Majority Expression (Grey=Not 2−Fold Increased in Silk)
AgA
AgP
Broad
Ac+F
Maj
Not OEST

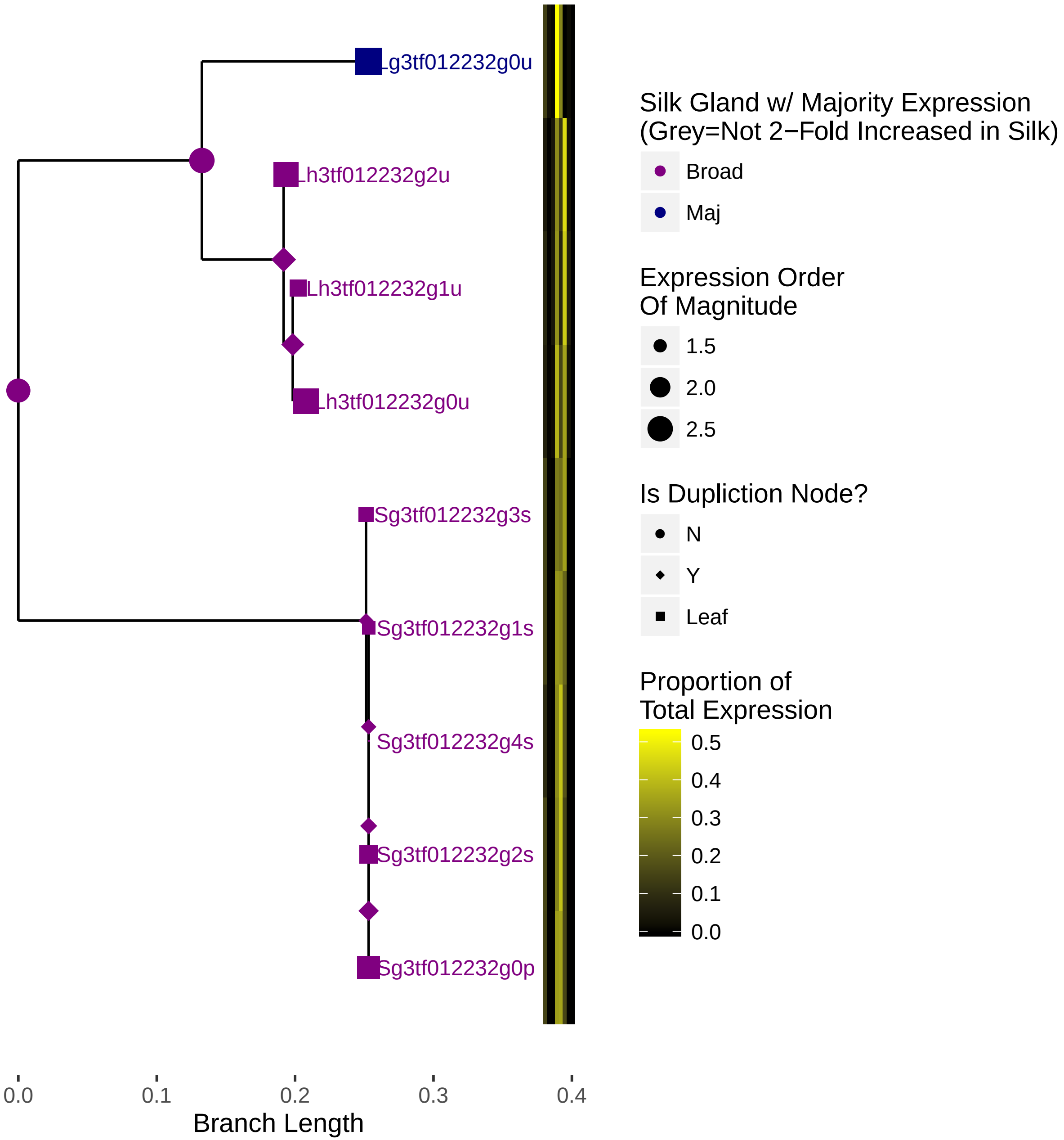

Lg3tf012232g0u
Lh3tf012232g2u
Lh3tf012232g1u
Lh3tf012232g0u
Sg3tf012232g3s
Sg3tf012232g1s
Sg3tf012232g4s
Sg3tf012232g2s
Sg3tf012232g0p
0.0
0.1
0.2
0.3
0.4
Branch Length
Silk Gland w/ Majority Expression
(Grey=Not 2−Fold Increased in Silk)
Broad
Maj
Expression Order
Of Magnitude
1.5
2.0
2.5
Is Dupliction Node?
N
Y
Leaf
Proportion of
Total Expression
0.5
0.4
0.3
0.2
0.1
0.0

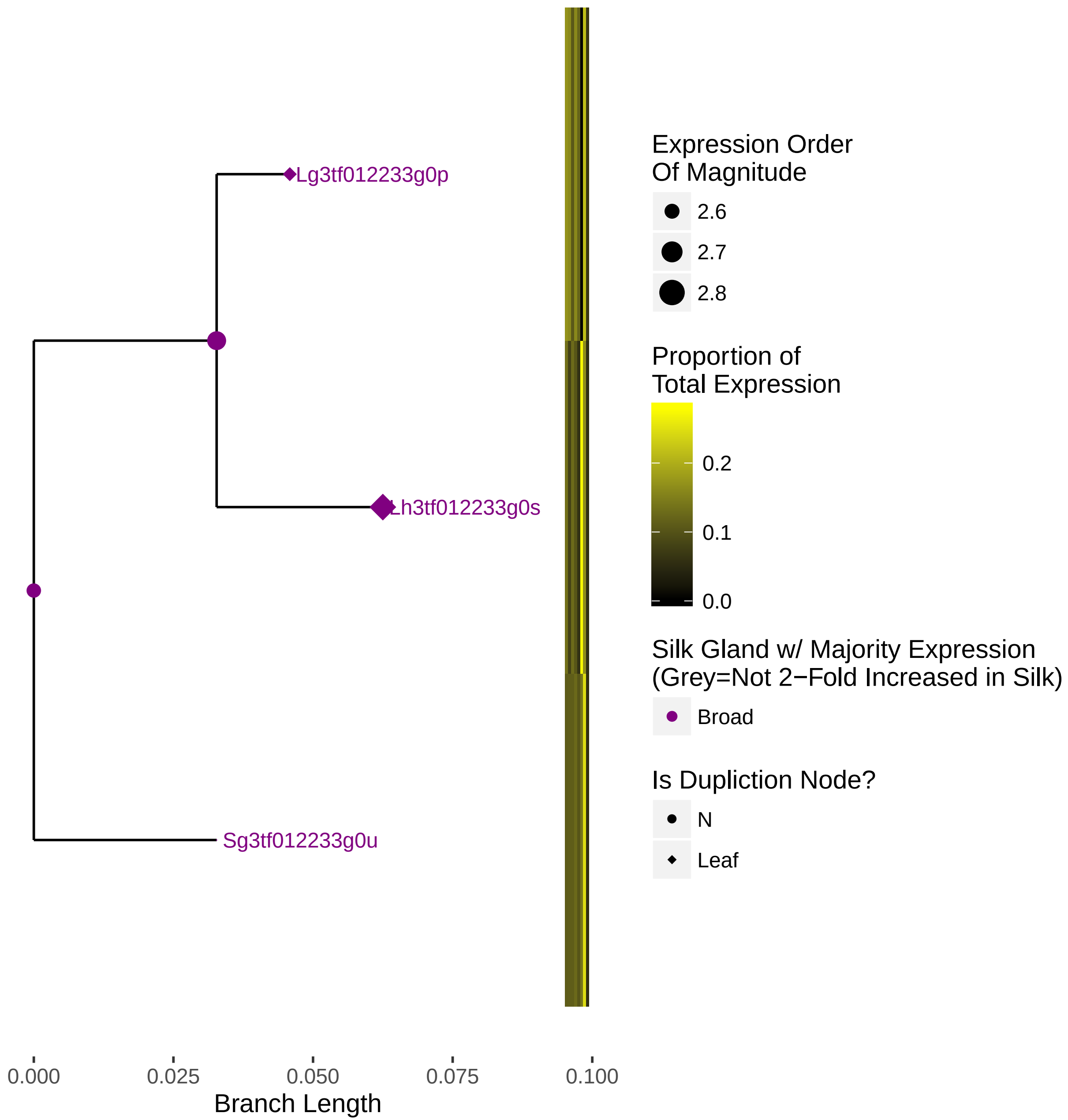

Lg3tf012233g0p
Lh3tf012233g0s
Sg3tf012233g0u
Expression Order
Of Magnitude
2.6
2.7
2.8
Proportion of
Total Expression
0.2
0.1
0.0
Silk Gland w/ Majority Expression
(Grey=Not 2−Fold Increased in Silk)
Broad
Is Dupliction Node?
N
Leaf
0.000
0.025
0.050
0.075
0.100
Branch Length

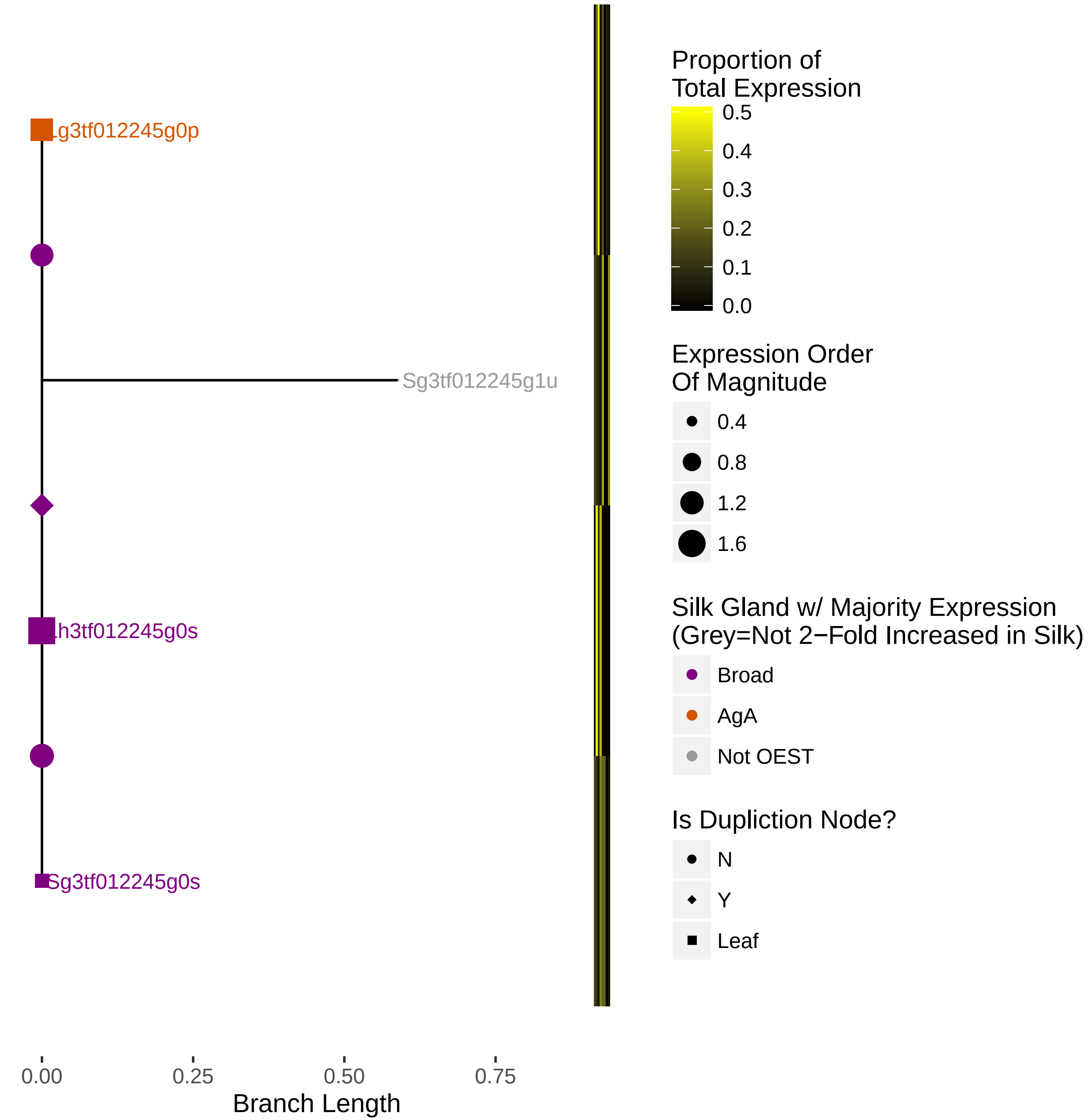

g3tf012245g0p
Sg3tf012245g1u
h3tf012245g0s
Sg3tf012245g0s
0.00
0.25
0.50
0.75
Branch Length
Proportion of Total Expression
0.5
0.4
0.3
0.2
0.1
0.0
Expression Order Of Magnitude
0.4
0.8
1.2
1.6
Silk Gland w/ Majority Expression (Grey=Not 2−Fold Increased in Silk)
Broad
AgA
Not OEST
Is Dupliction Node?
N
Y
Leaf

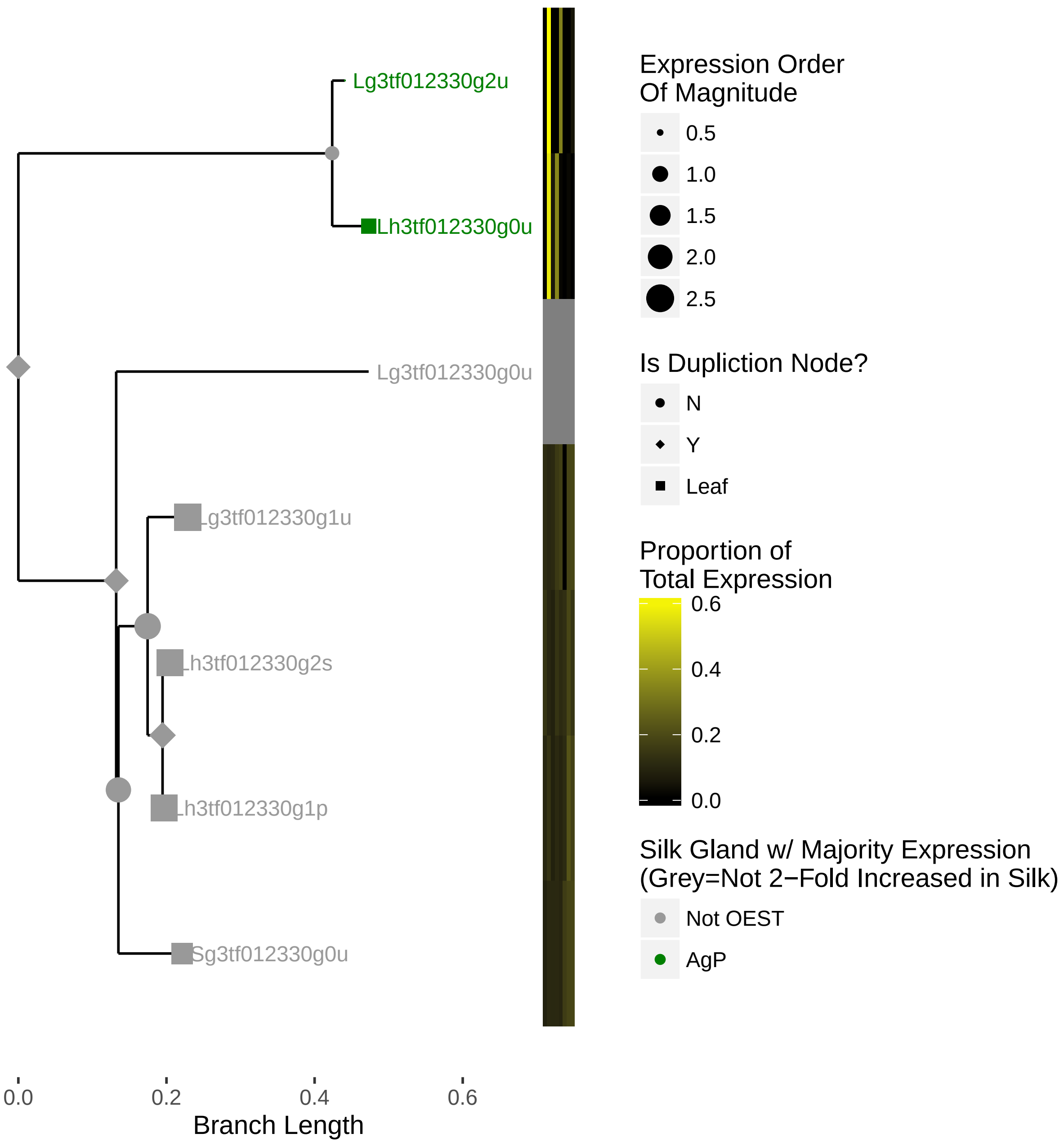
Lg3tf012330g2u
Lh3tf012330g0u
Lg3tf012330g0u
Lg3tf012330g1u
Lh3tf012330g2s
Lh3tf012330g1p
Sg3tf012330g0u
0.0
0.2
0.4
0.6
Branch Length
Expression Order
Of Magnitude
0.5
1.0
1.5
2.0
2.5
Is Dupliction Node?
N
Y
Leaf
Proportion of
Total Expression
0.6
0.4
0.2
0.0
Silk Gland w/ Majority Expression
(Grey=Not 2−Fold Increased in Silk)
Not OEST
AgP

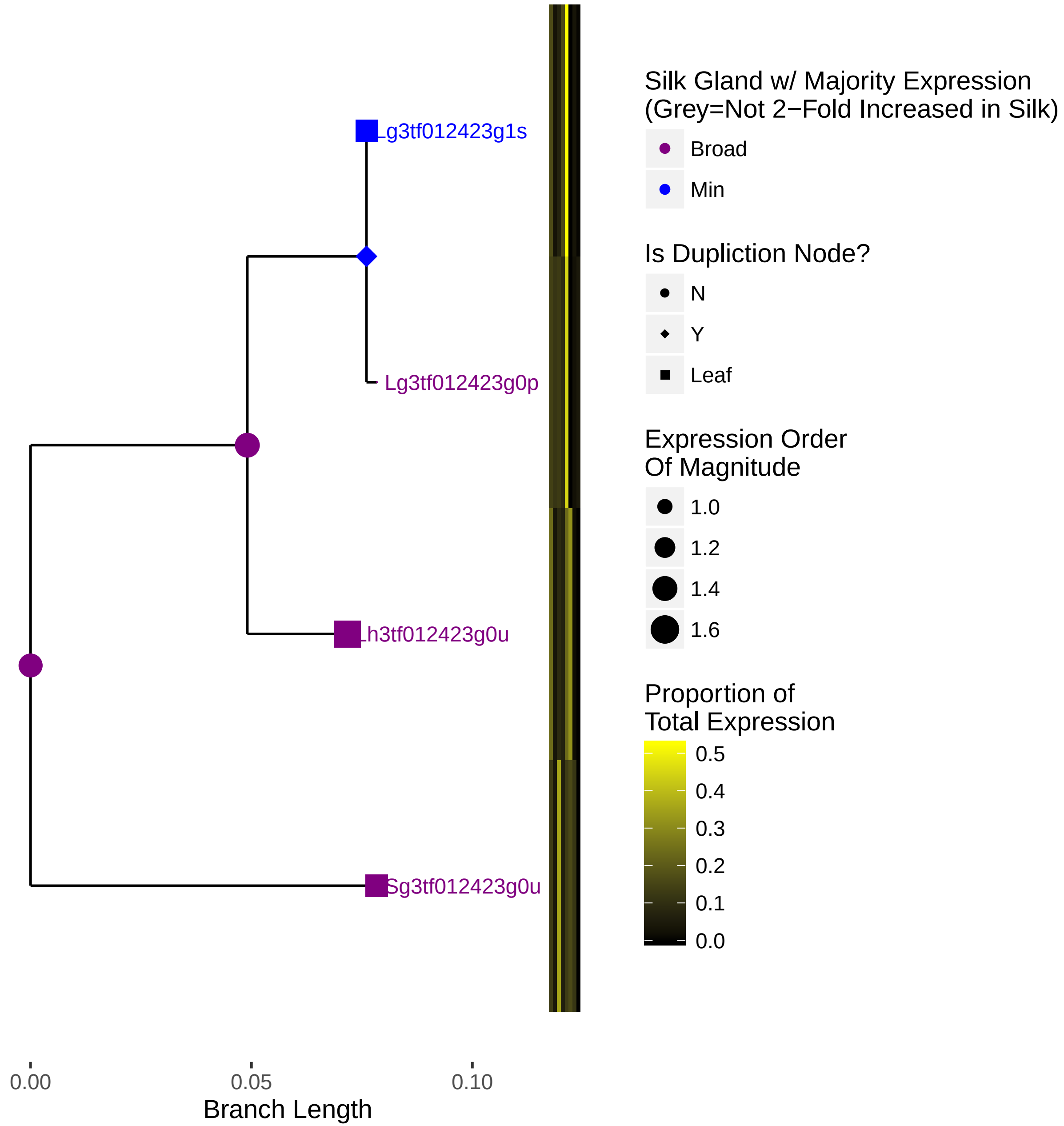
Lg3tf012423g1s
Lg3tf012423g0p
Lh3tf012423g0u
Sg3tf012423g0u
0.00
0.05
0.10
Branch Length
Silk Gland w/ Majority Expression
(Grey=Not 2−Fold Increased in Silk)
Broad
Min
Is Dupliction Node?
N
Y
Leaf
Expression Order
Of Magnitude
1.0
1.2
1.4
1.6
Proportion of
Total Expression
0.5
0.4
0.3
0.2
0.1
0.0

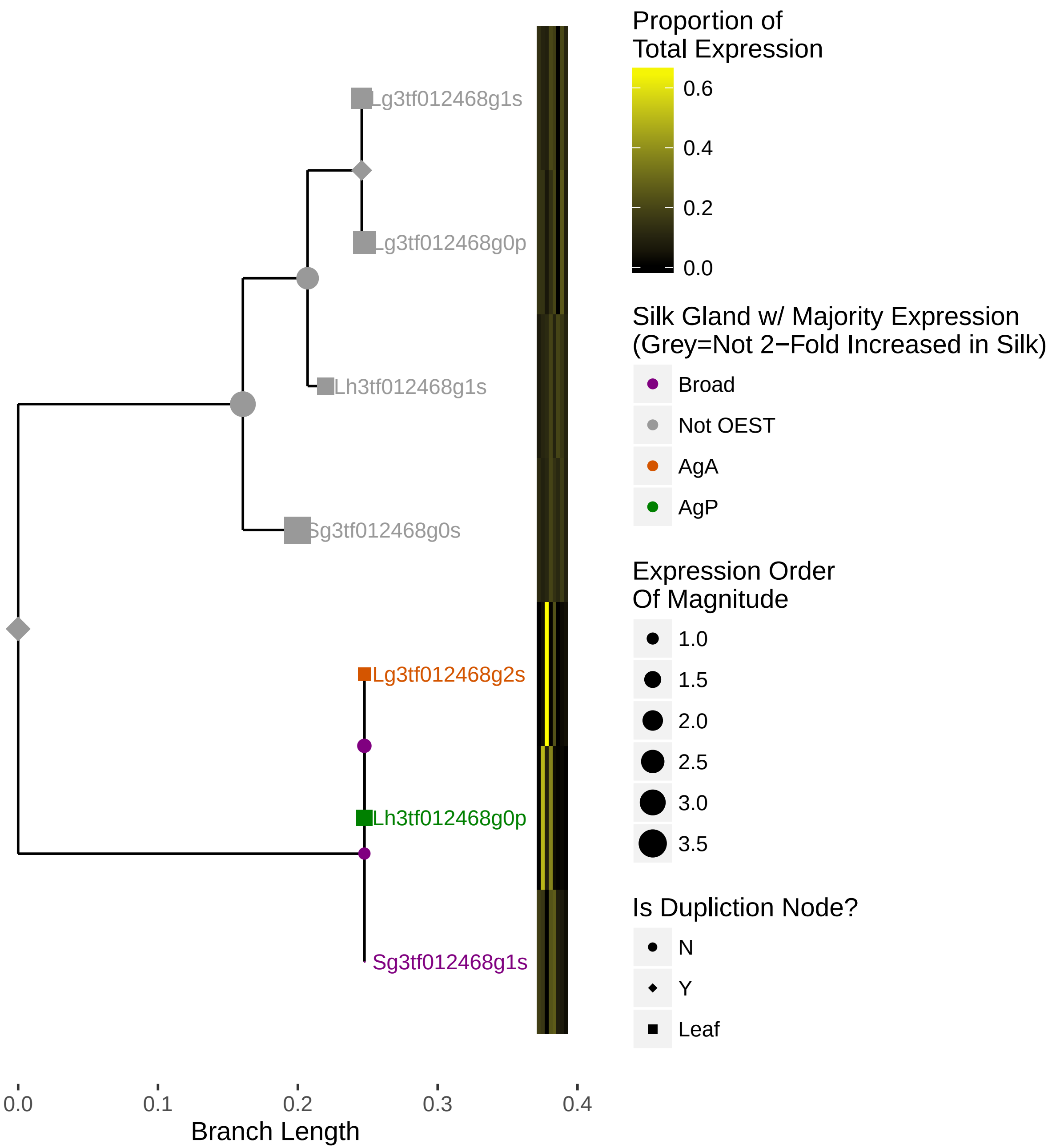
Proportion of
Total Expression
0.6
0.4
0.2
0.0
Silk Gland w/ Majority Expression
(Grey=Not 2−Fold Increased in Silk)
Broad
Not OEST
AgA
AgP
Expression Order
Of Magnitude
1.0
1.5
2.0
2.5
3.0
3.5
Is Dupliction Node?
N
Y
Leaf
Lg3tf012468g1s
Lg3tf012468g0p
Lh3tf012468g1s
Sg3tf012468g0s
Lg3tf012468g2s
Lh3tf012468g0p
Sg3tf012468g1s
0.0
0.1
0.2
0.3
0.4
Branch Length

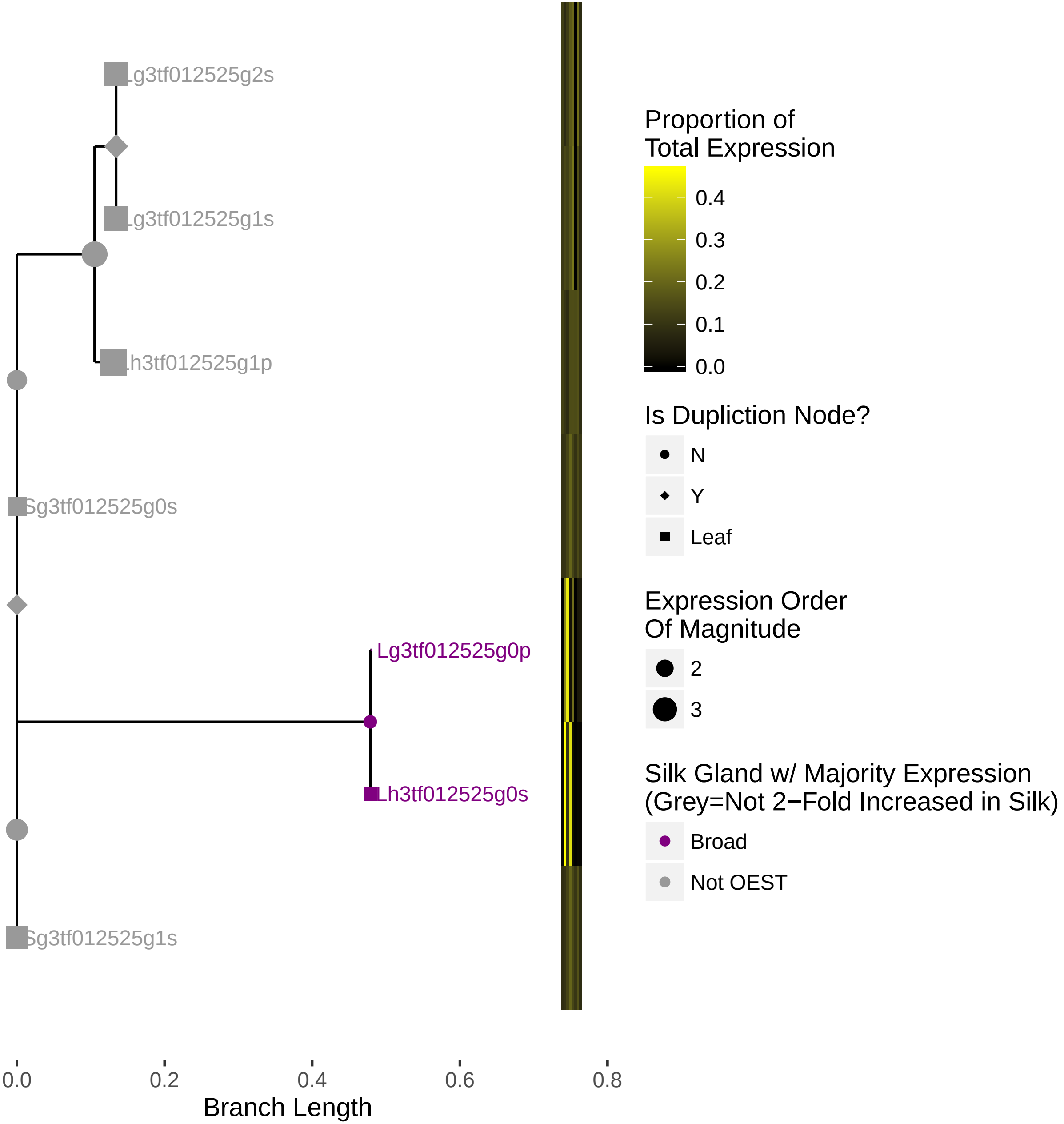

Lg3tf012525g2s
Lg3tf012525g1s
Lh3tf012525g1p
Sg3tf012525g0s
Lg3tf012525g0p
Lh3tf012525g0s
Sg3tf012525g1s
0.0
0.2
0.4
0.6
0.8
Branch Length
Proportion of
Total Expression
0.4
0.3
0.2
0.1
0.0
Is Dupliction Node?
N
Y
Leaf
Expression Order
Of Magnitude
2
3
Silk Gland w/ Majority Expression
(Grey=Not 2−Fold Increased in Silk)
Broad
Not OEST

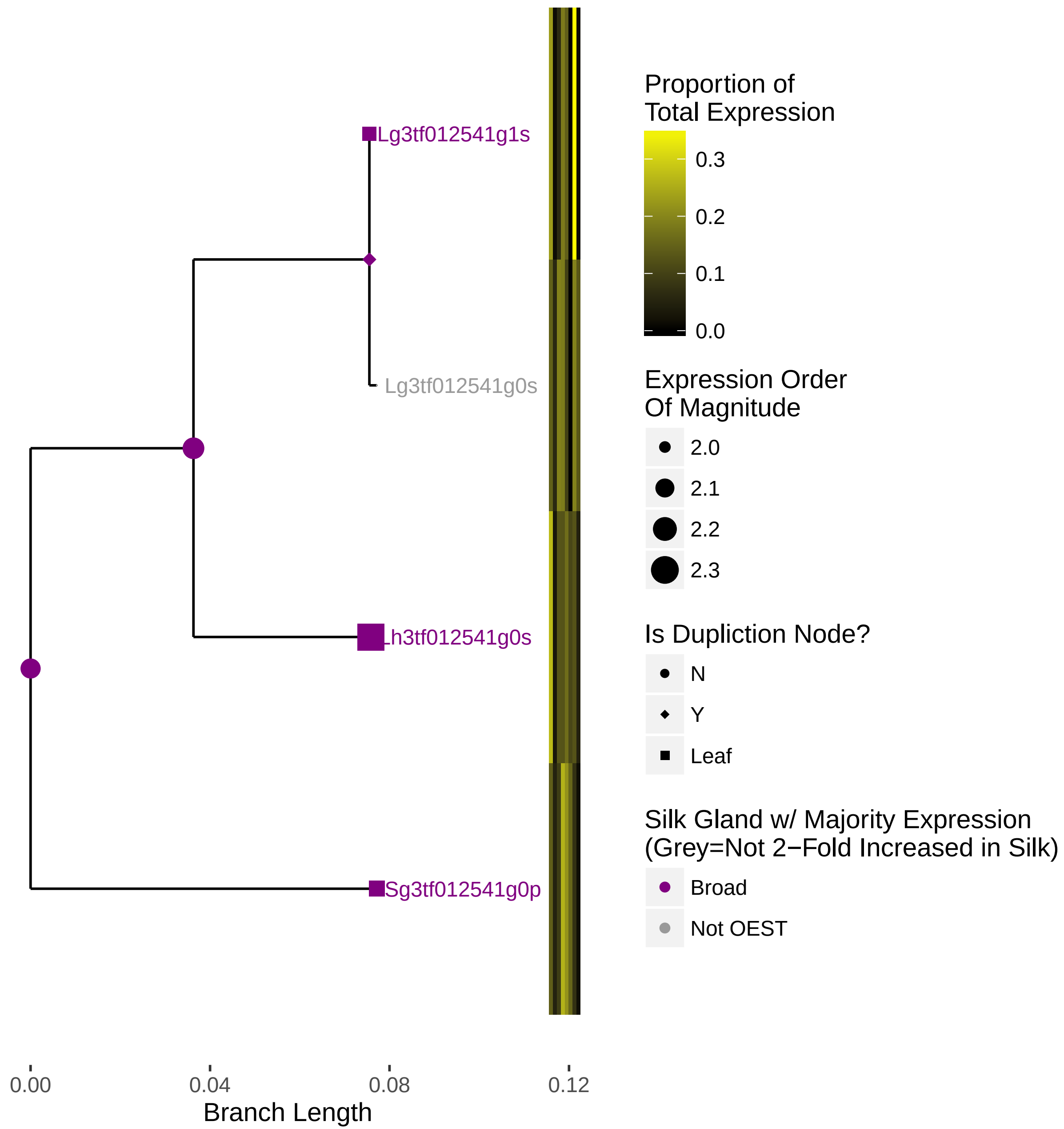

Lg3tf012541g1s
Lg3tf012541g0s
Lh3tf012541g0s
Sg3tf012541g0p
0.00
0.04
0.08
0.12
Branch Length
Proportion of
Total Expression
0.3
0.2
0.1
0.0
Expression Order
Of Magnitude
2.0
2.1
2.2
2.3
Is Dupliction Node?
N
Y
Leaf
Silk Gland w/ Majority Expression
(Grey=Not 2−Fold Increased in Silk)
Broad
Not OEST

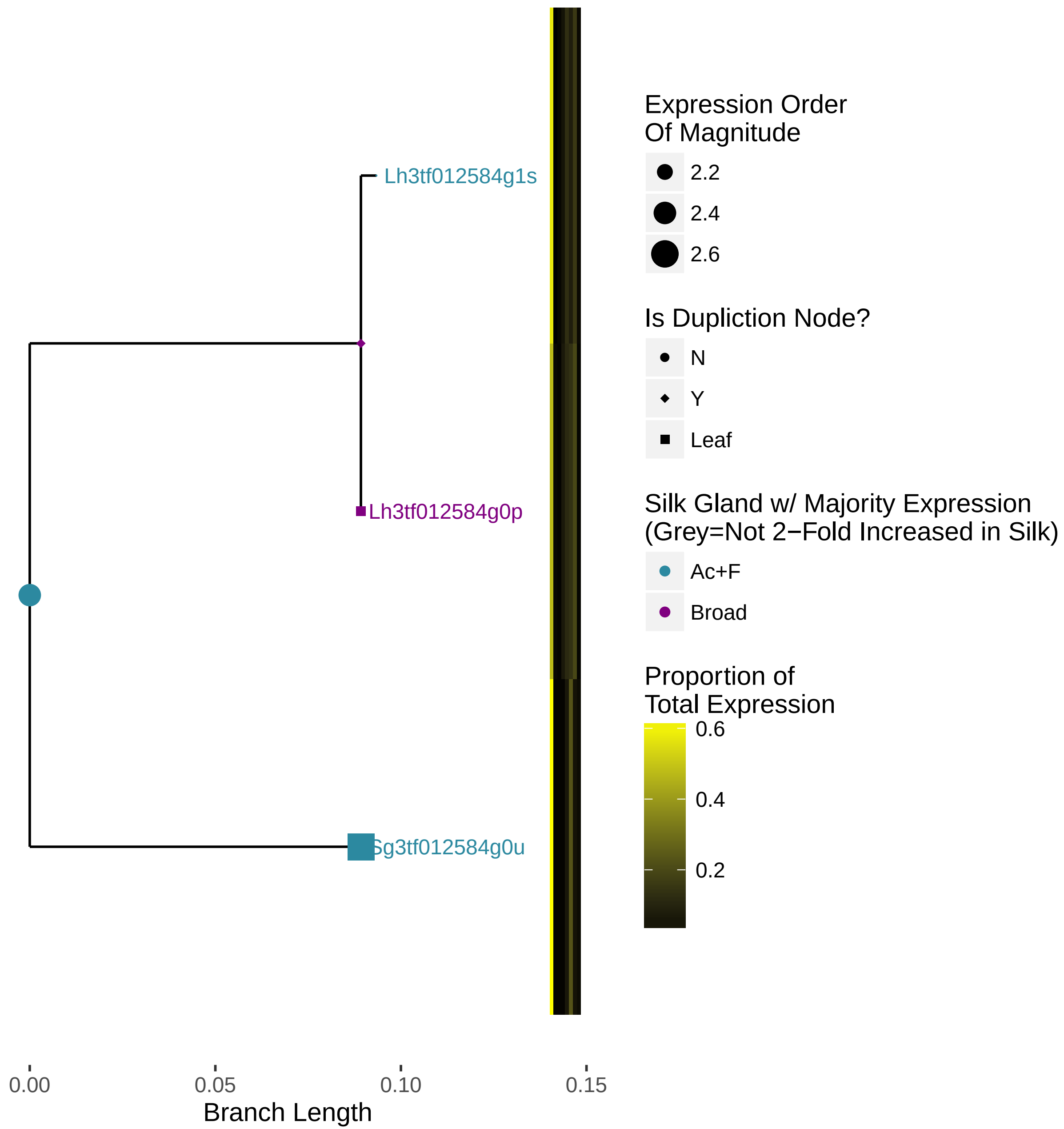

Lh3tf012584g1s
Lh3tf012584g0p
Sg3tf012584g0u
Expression Order
Of Magnitude
2.2
2.4
2.6
Is Dupliction Node?
N
Y
Leaf
Silk Gland w/ Majority Expression
(Grey=Not 2−Fold Increased in Silk)
Ac+F
Broad
Proportion of
Total Expression
0.6
0.4
0.2
0.00
0.05
0.10
0.15
Branch Length

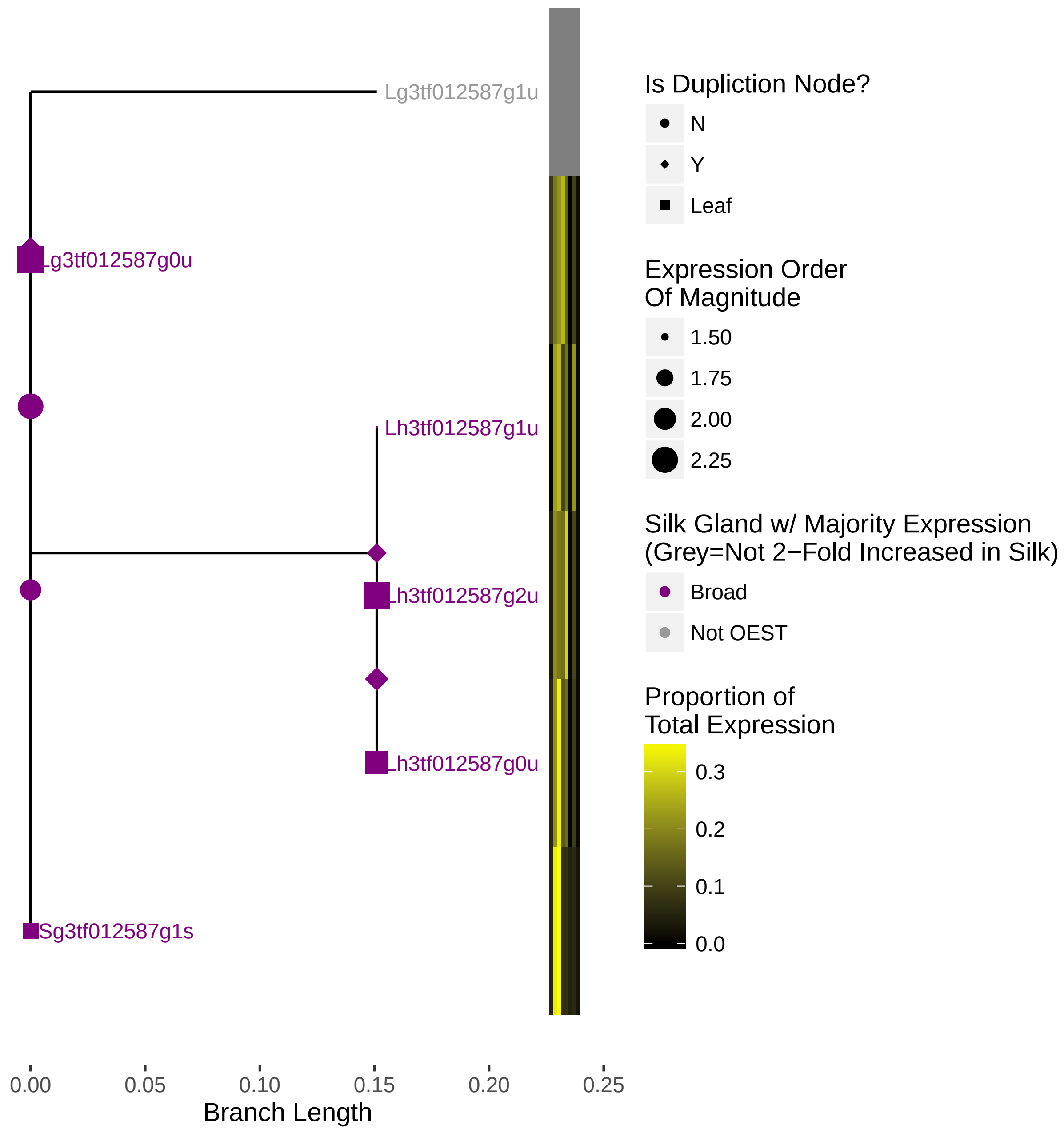
Lg3tf012587g1u
Lg3tf012587g0u
Lh3tf012587g1u
Lh3tf012587g2u
Lh3tf012587g0u
Sg3tf012587g1s
0.00
0.05
0.10
0.15
0.20
0.25
Branch Length
Is Dupliction Node?
N
Y
Leaf
Expression Order
Of Magnitude
1.50
1.75
2.00
2.25
Silk Gland w/ Majority Expression
(Grey=Not 2−Fold Increased in Silk)
Broad
Not OEST
Proportion of
Total Expression
0.3
0.2
0.1
0.0

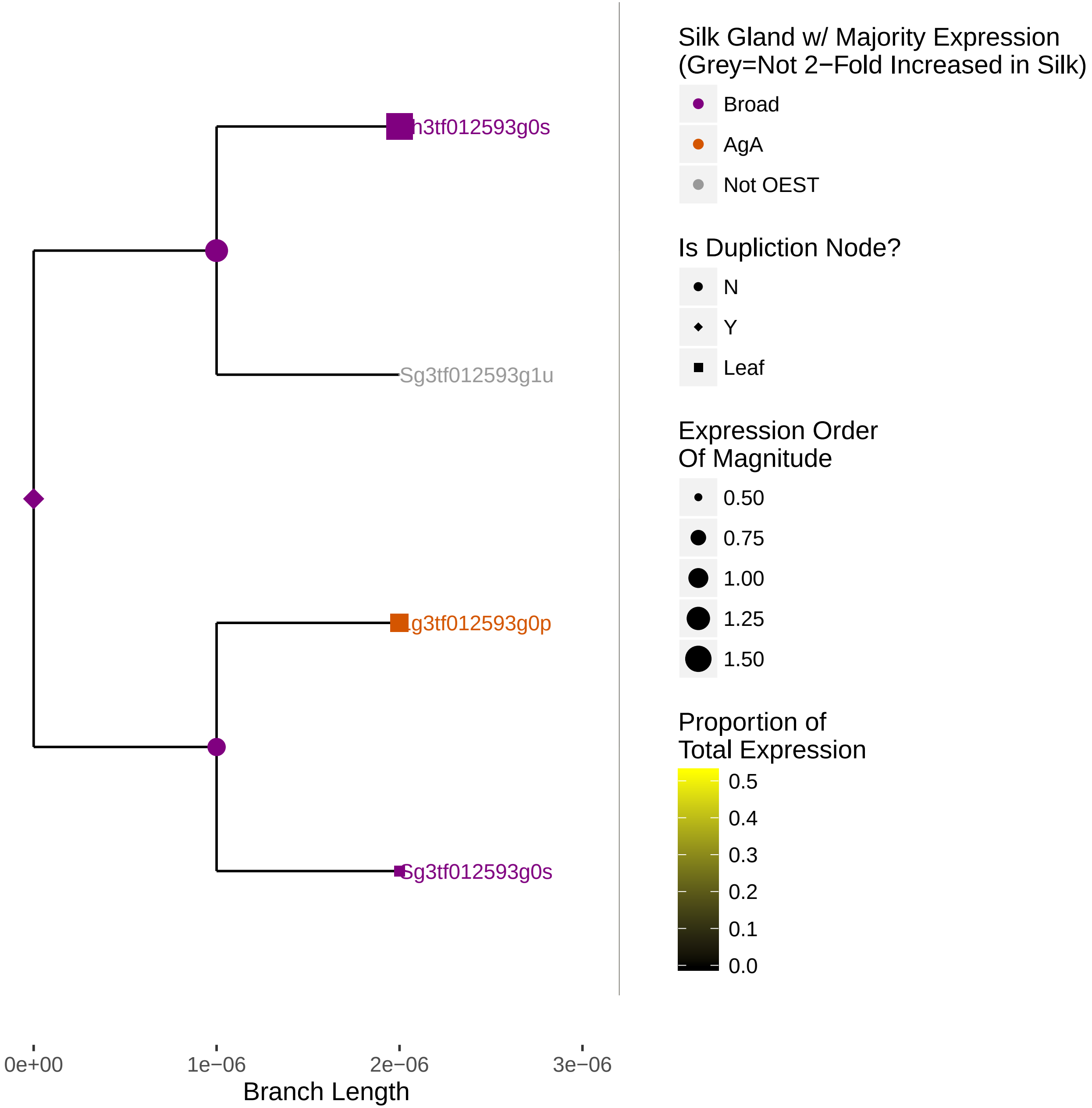
h3tf012593g0s
Sg3tf012593g1u
g3tf012593g0p
Sg3tf012593g0s
0e+00
1e−06
2e−06
3e−06
Branch Length
Silk Gland w/ Majority Expression
(Grey=Not 2−Fold Increased in Silk)
Broad
AgA
Not OEST
Is Dupliction Node?
N
Y
Leaf
Expression Order
Of Magnitude
0.50
0.75
1.00
1.25
1.50
Proportion of
Total Expression
0.5
0.4
0.3
0.2
0.1
0.0

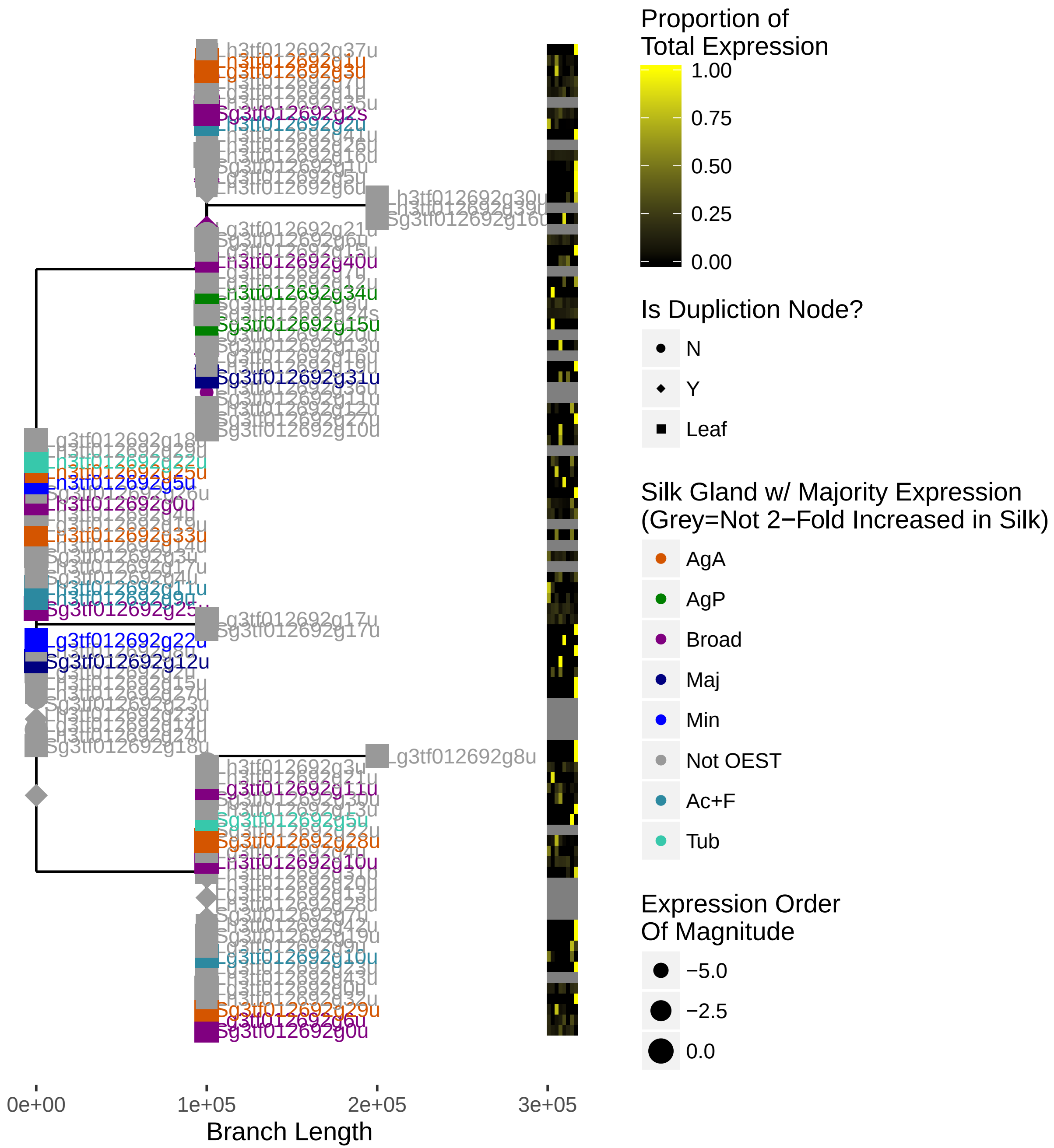

Proportion of Total Expression
1.00
0.75
0.50
0.25
0.00
Is Dupliction Node?
N
Y
Leaf
Silk Gland w/ Majority Expression (Grey=Not 2−Fold Increased in Silk)
AgA
AgP
Broad
Maj
Min
Not OEST
Ac+F
Tub
Expression Order Of Magnitude
−5.0
−2.5
0.0
h3tf012692g37u
Sg3tf012692g2s
h3tf012692g30u
Sg3tf012692g16u
h3tf012692g40u
h3tf012692g34u
Sg3tf012692g15u
Sg3tf012692g31u
Sg3tf012692g10u
g3tf012692g18u
h3tf012692g22u
h3tf012692g25u
h3tf012692g5u
h3tf012692g0u
h3tf012692g33u
Sg3tf012692g25u
g3tf012692g17u
Sg3tf012692g17u
g3tf012692g22u
Sg3tf012692g12u
Sg3tf012692g18u
g3tf012692g8u
h3tf012692g3u
g3tf012692g11u
Sg3tf012692g5u
Sg3tf012692g28u
h3tf012692g10u
g3tf012692g10u
Sg3tf012692g29u
Sg3tf012692g0u
0e+00
1e+05
2e+05
3e+05
Branch Length

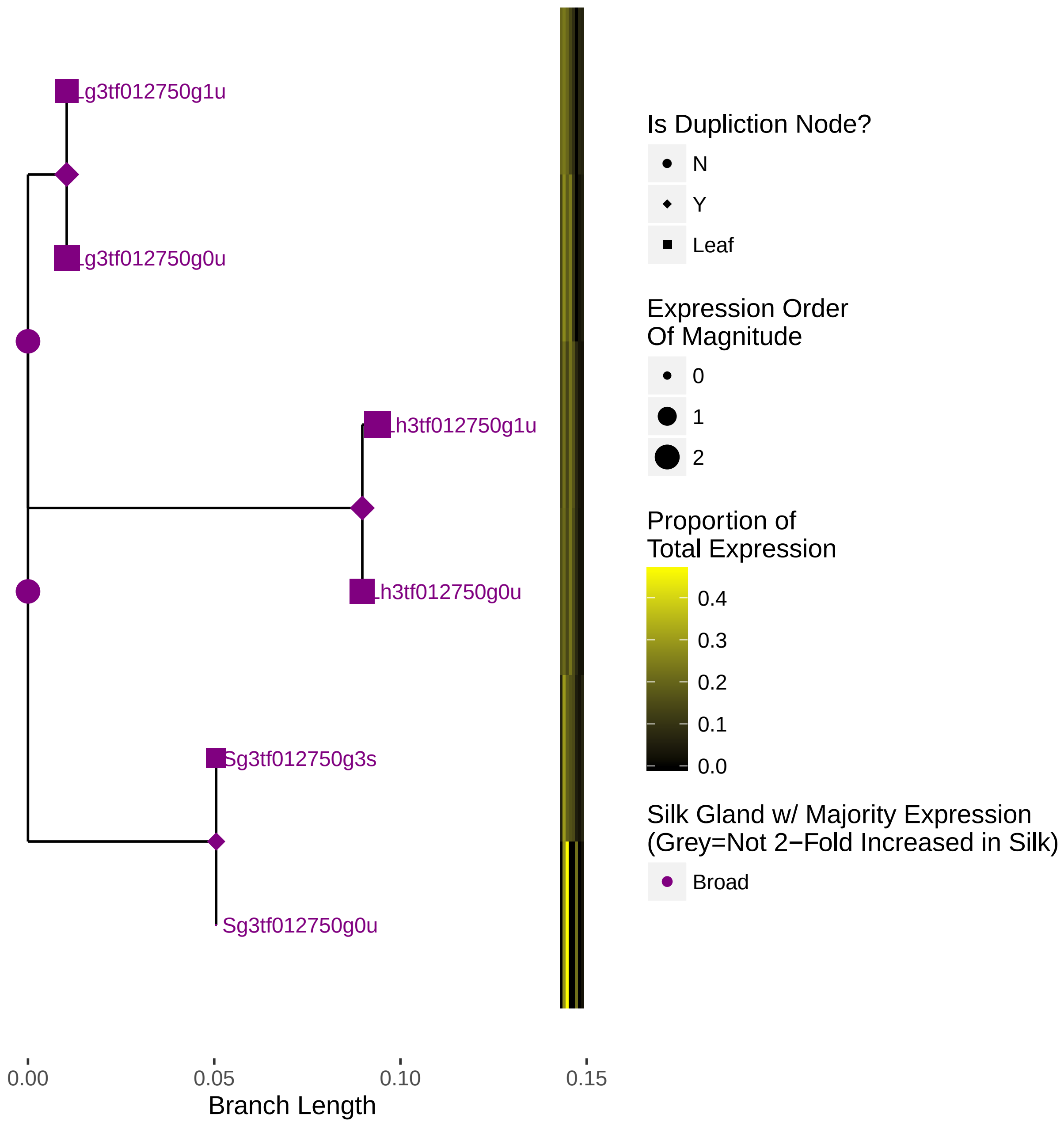

Lg3tf012750g1u
Lg3tf012750g0u
Lh3tf012750g1u
Lh3tf012750g0u
Sg3tf012750g3s
Sg3tf012750g0u
0.00
0.05
0.10
0.15
Branch Length
Is Dupliction Node?
N
Y
Leaf
Expression Order
Of Magnitude
0
1
2
Proportion of
Total Expression
0.4
0.3
0.2
0.1
0.0
Silk Gland w/ Majority Expression
(Grey=Not 2−Fold Increased in Silk)
Broad

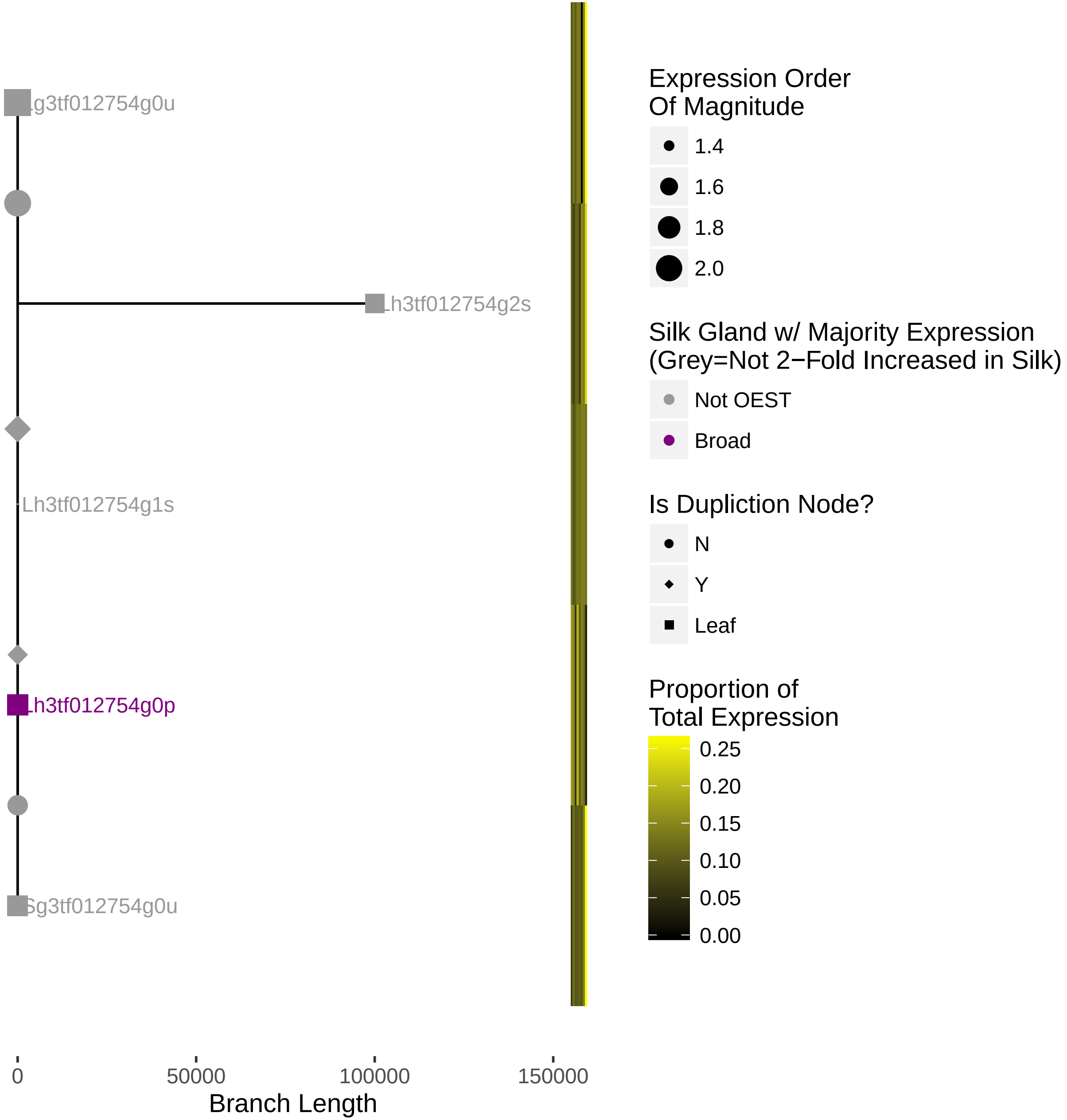

g3tf012754g0u
Lh3tf012754g2s
Lh3tf012754g1s
Lh3tf012754g0p
Sg3tf012754g0u
0
50000
100000
150000
Branch Length
Expression Order
Of Magnitude
1.4
1.6
1.8
2.0
Silk Gland w/ Majority Expression
(Grey=Not 2−Fold Increased in Silk)
Not OEST
Broad
Is Participation Node?
N
Y
Leaf
Proportion of
Total Expression
0.25
0.20
0.15
0.10
0.05
0.00

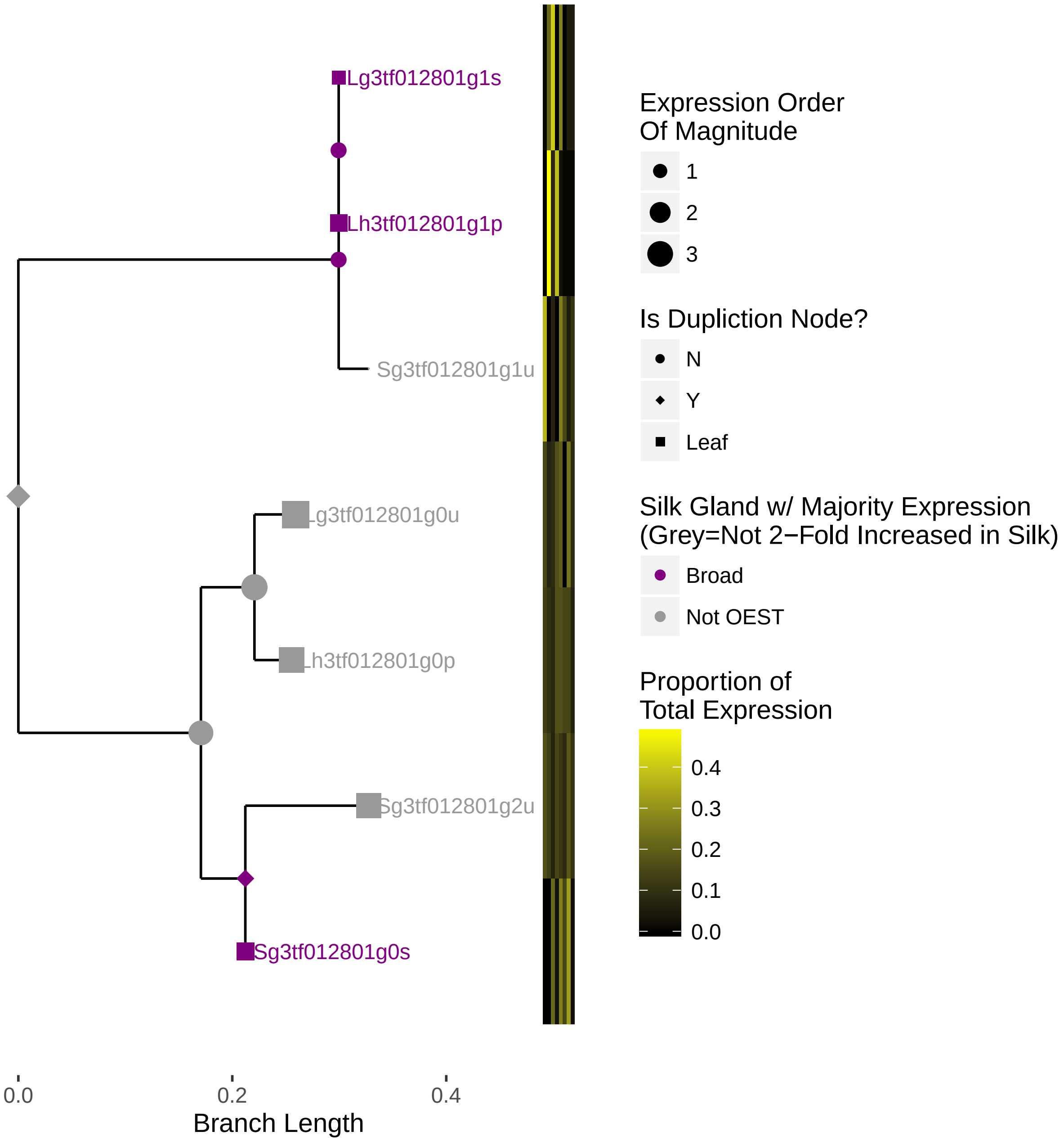

Lg3tf012801g1s
Lh3tf012801g1p
Sg3tf012801g1u
Lg3tf012801g0u
Lh3tf012801g0p
Sg3tf012801g2u
Sg3tf012801g0s
0.0
0.2
0.4
Branch Length
Expression Order Of Magnitude
1
2
3
Is Dupliction Node?
N
Y
Leaf
Silk Gland w/ Majority Expression (Grey=Not 2−Fold Increased in Silk)
Broad
Not OEST
Proportion of Total Expression
0.4
0.3
0.2
0.1
0.0

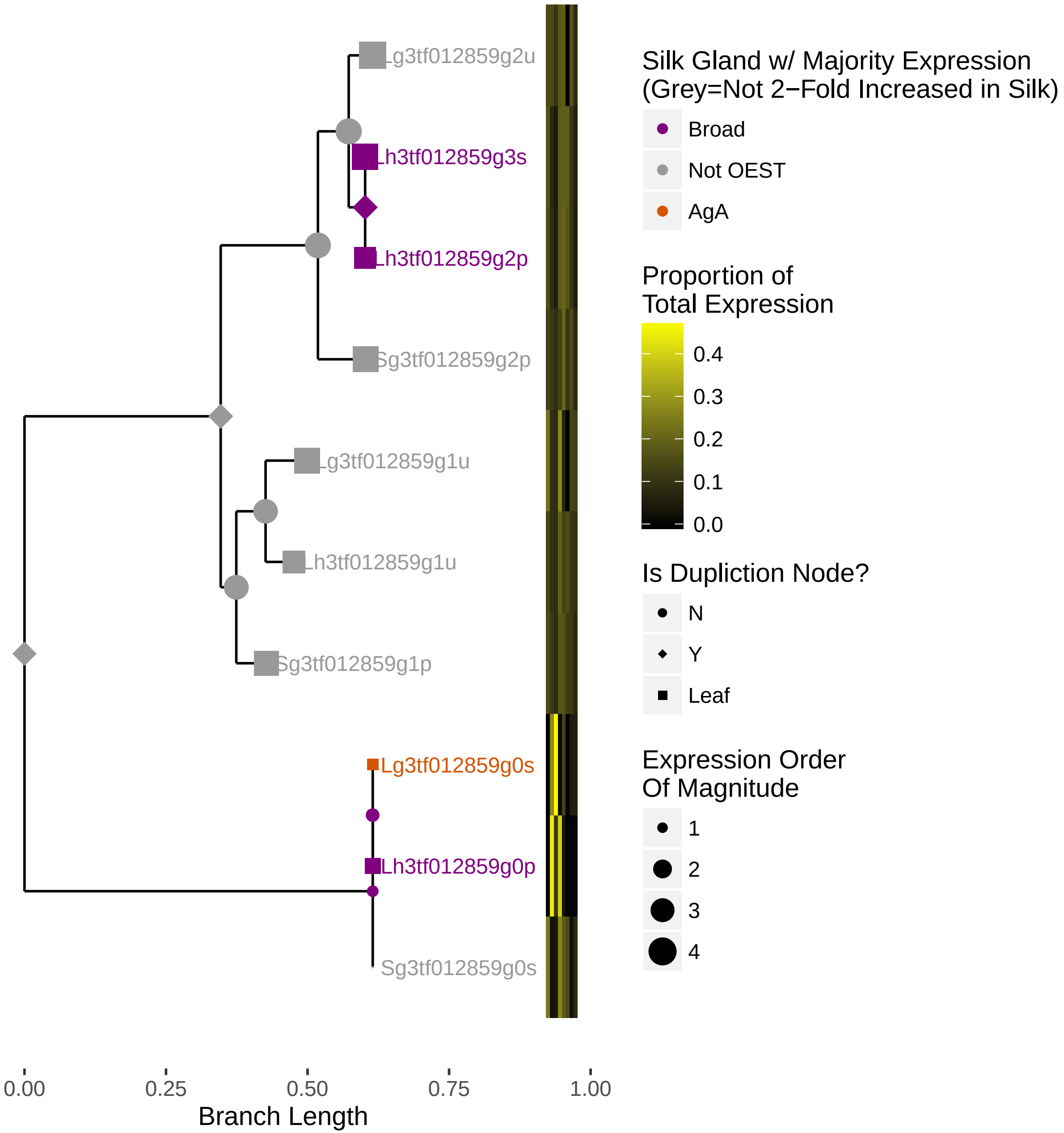

Lg3tf012859g2u
Lh3tf012859g3s
Lh3tf012859g2p
Sg3tf012859g2p
Lg3tf012859g1u
Lh3tf012859g1u
Sg3tf012859g1p
Lg3tf012859g0s
Lh3tf012859g0p
Sg3tf012859g0s
0.00
0.25
0.50
0.75
1.00
Branch Length
Silk Gland w/ Majority Expression (Grey=Not 2−Fold Increased in Silk)
Broad
Not OEST
AgA
Proportion of Total Expression
0.4
0.3
0.2
0.1
0.0
Is Dupliction Node?
N
Y
Leaf
Expression Order Of Magnitude
1
2
3
4

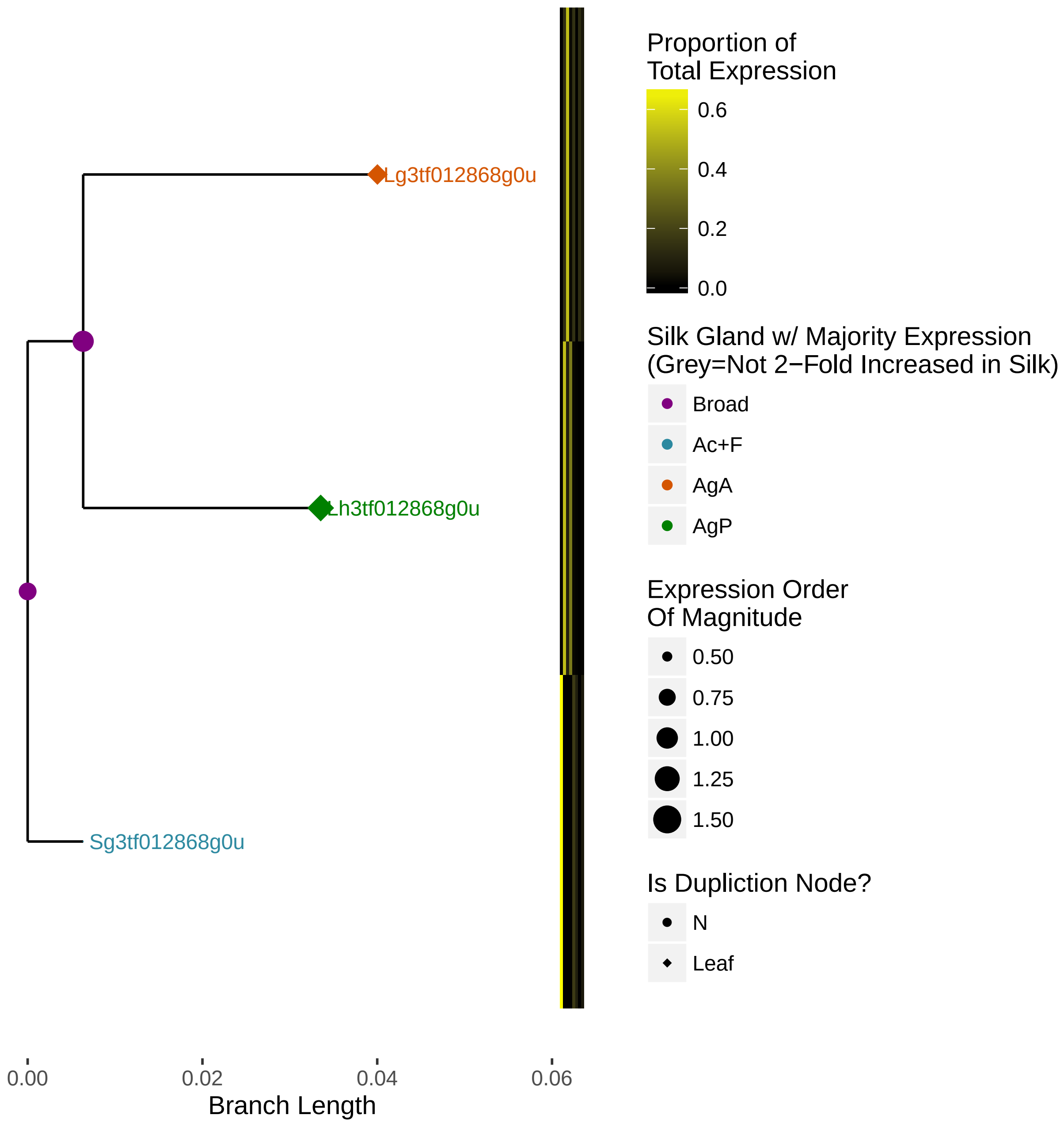

Lg3tf012868g0u
Lh3tf012868g0u
Sg3tf012868g0u
0.00
0.02
0.04
0.06
Branch Length
Proportion of Total Expression
0.6
0.4
0.2
0.0
Silk Gland w/ Majority Expression (Grey=Not 2−Fold Increased in Silk)
Broad
Ac+F
AgA
AgP
Expression Order Of Magnitude
0.50
0.75
1.00
1.25
1.50
Is Dupliction Node?
N
Leaf

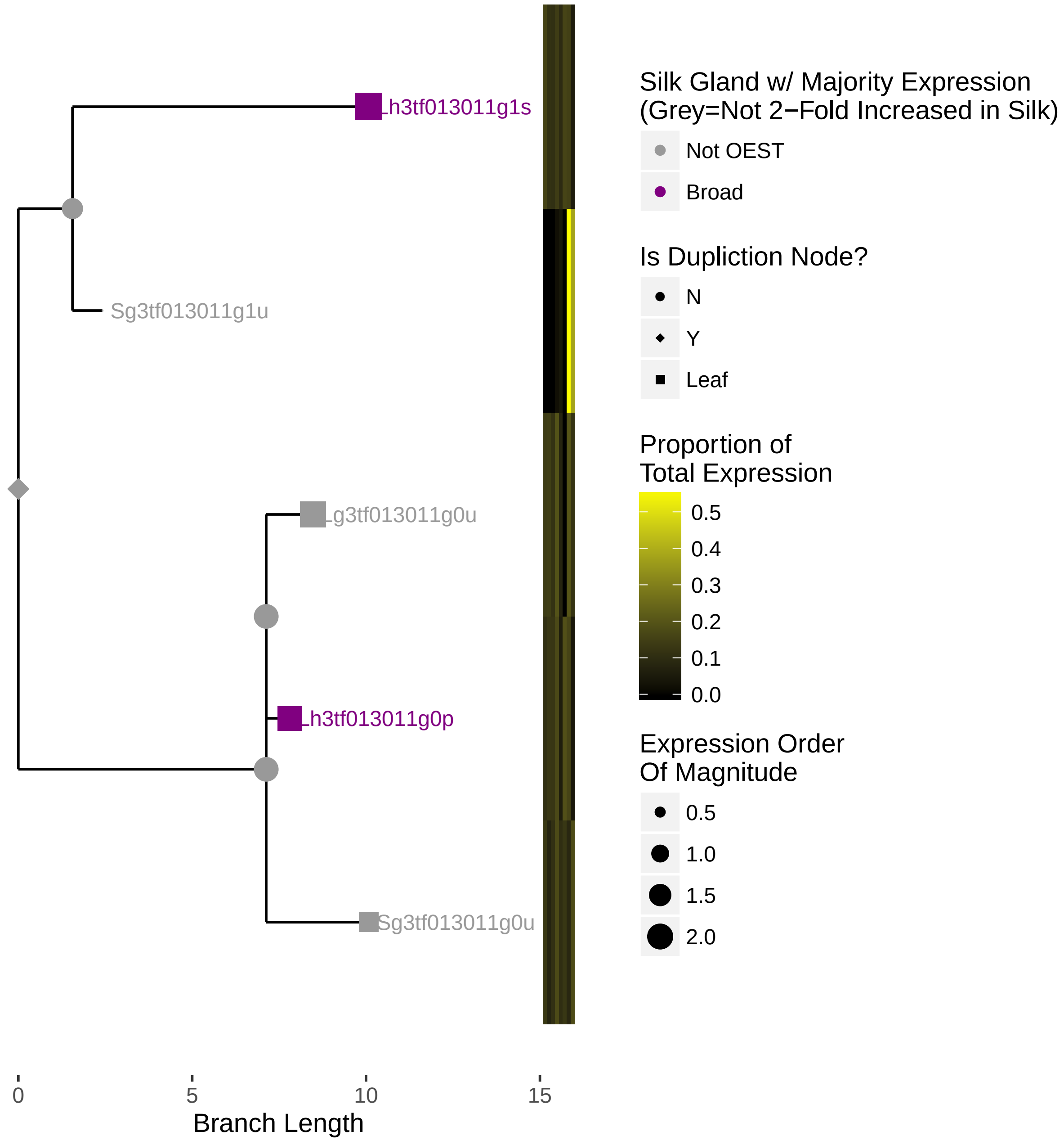

h3tf013011g1s
Sg3tf013011g1u
g3tf013011g0u
h3tf013011g0p
Sg3tf013011g0u
0
5
10
15
Branch Length
Silk Gland w/ Majority Expression
(Grey=Not 2−Fold Increased in Silk)
Not OEST
Broad
Is Dupliction Node?
N
Y
Leaf
Proportion of
Total Expression
0.5
0.4
0.3
0.2
0.1
0.0
Expression Order
Of Magnitude
0.5
1.0
1.5
2.0

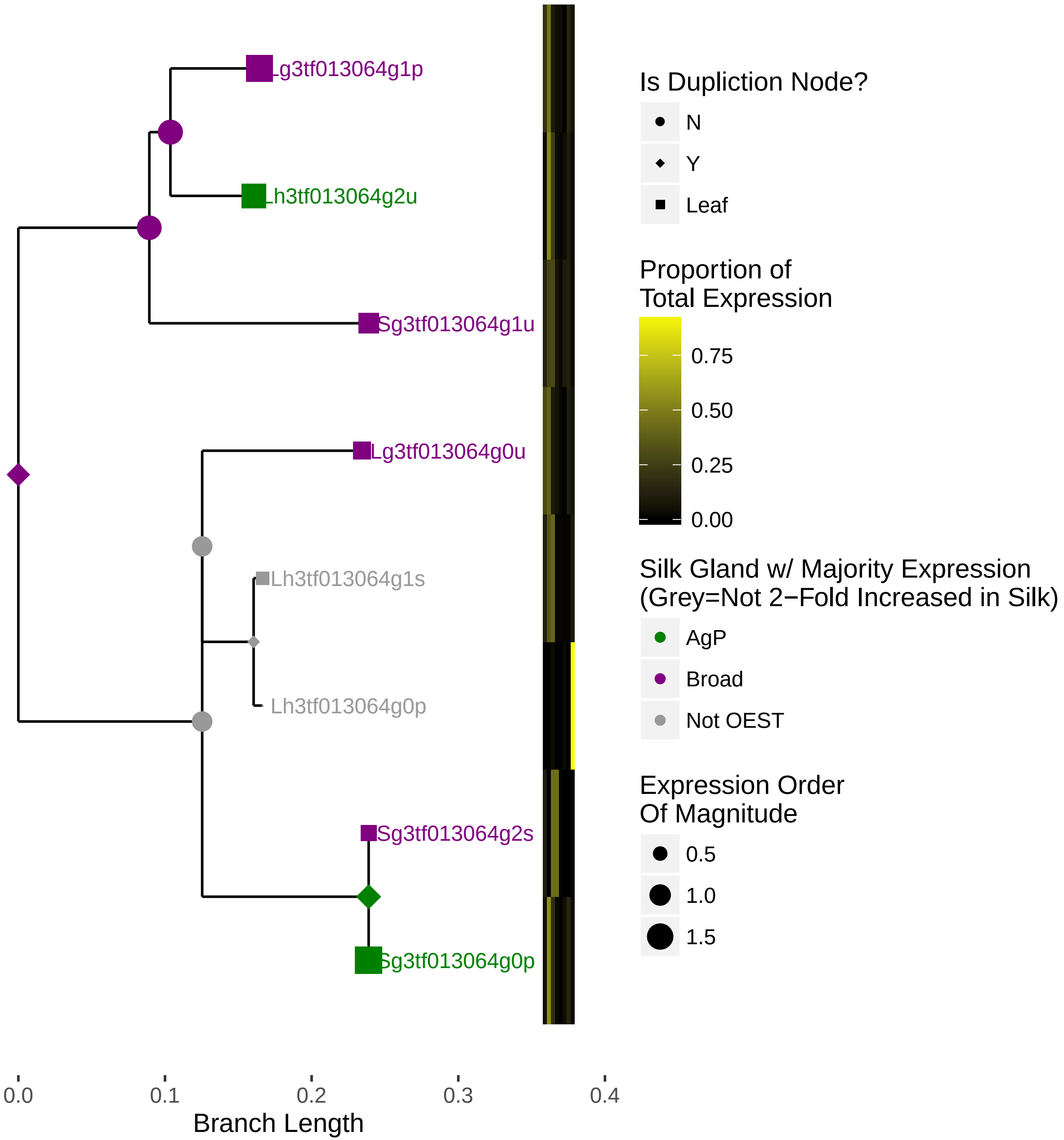

Lg3tf013064g1p
Lh3tf013064g2u
Sg3tf013064g1u
Lg3tf013064g0u
Lh3tf013064g1s
Lh3tf013064g0p
Sg3tf013064g2s
Sg3tf013064g0p
0.0
0.1
0.2
0.3
0.4
Branch Length
Is Dupliction Node?
N
Y
Leaf
Proportion of Total Expression
0.75
0.50
0.25
0.00
Silk Gland w/ Majority Expression (Grey=Not 2−Fold Increased in Silk)
AgP
Broad
Not OEST
Expression Order Of Magnitude
0.5
1.0
1.5

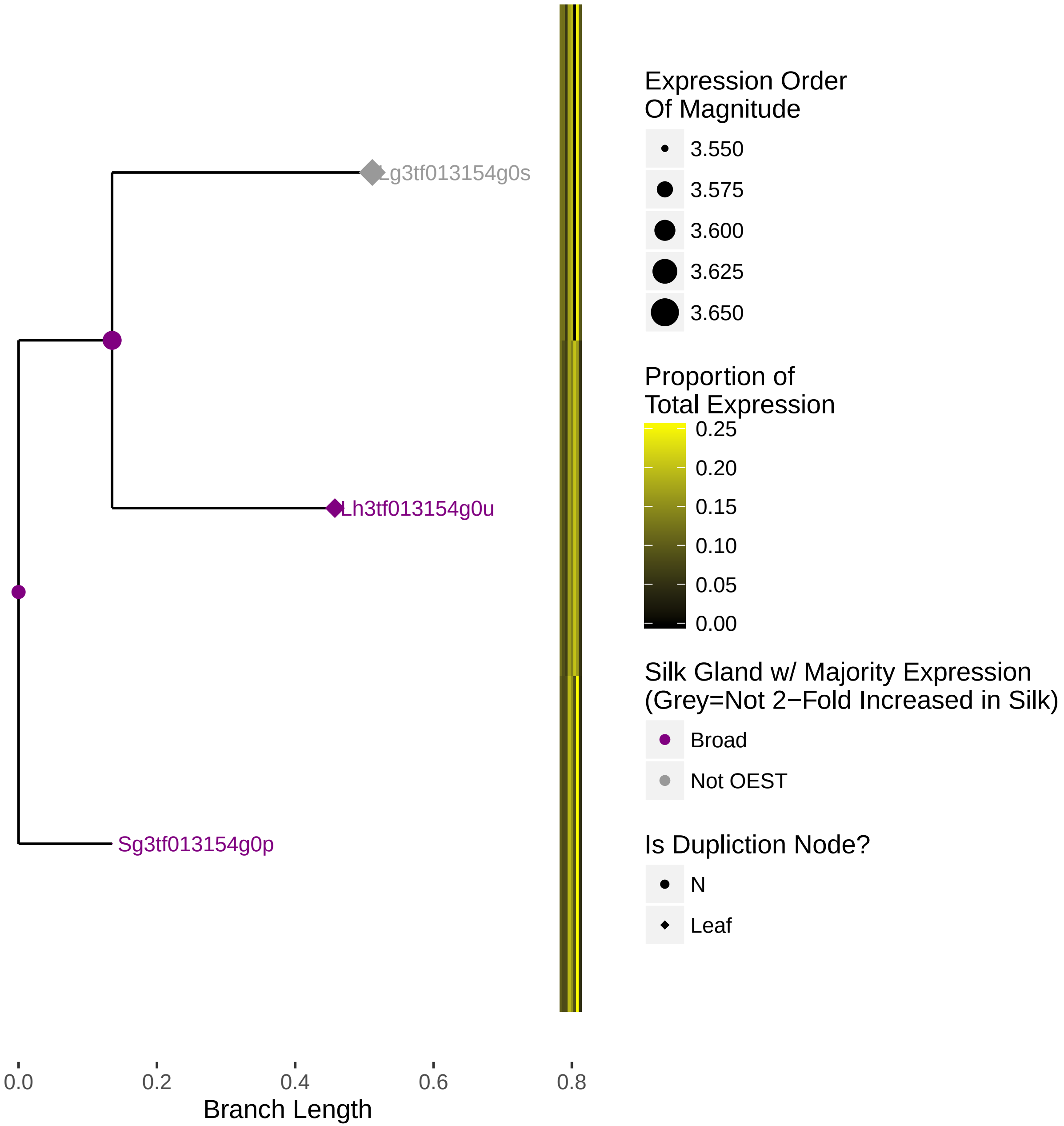
Lg3tf013154g0s
Lh3tf013154g0u
Sg3tf013154g0p
Expression Order
Of Magnitude
3.550
3.575
3.600
3.625
3.650
Proportion of
Total Expression
0.25
0.20
0.15
0.10
0.05
0.00
Silk Gland w/ Majority Expression
(Grey=Not 2−Fold Increased in Silk)
Broad
Not OEST
Is Duplication Node?
N
Leaf
0.0
0.2
0.4
0.6
0.8
Branch Length

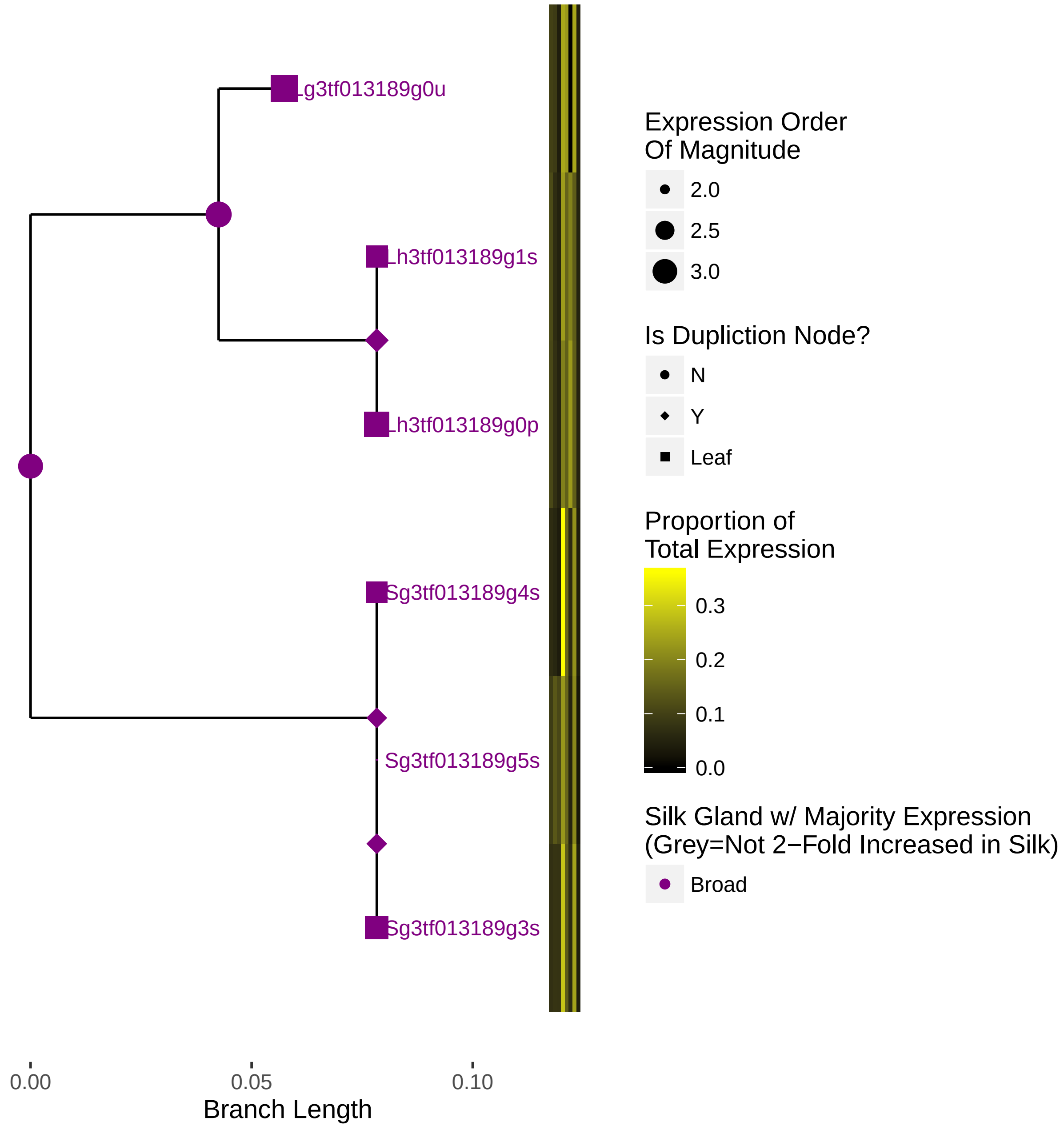

Lg3tf013189g0u
Lh3tf013189g1s
Lh3tf013189g0p
Sg3tf013189g4s
Sg3tf013189g5s
Sg3tf013189g3s
Expression Order
Of Magnitude
2.0
2.5
3.0
Is Dupliction Node?
N
Y
Leaf
Proportion of
Total Expression
0.3
0.2
0.1
0.0
Silk Gland w/ Majority Expression
(Grey=Not 2−Fold Increased in Silk)
Broad
0.00
0.05
0.10
Branch Length

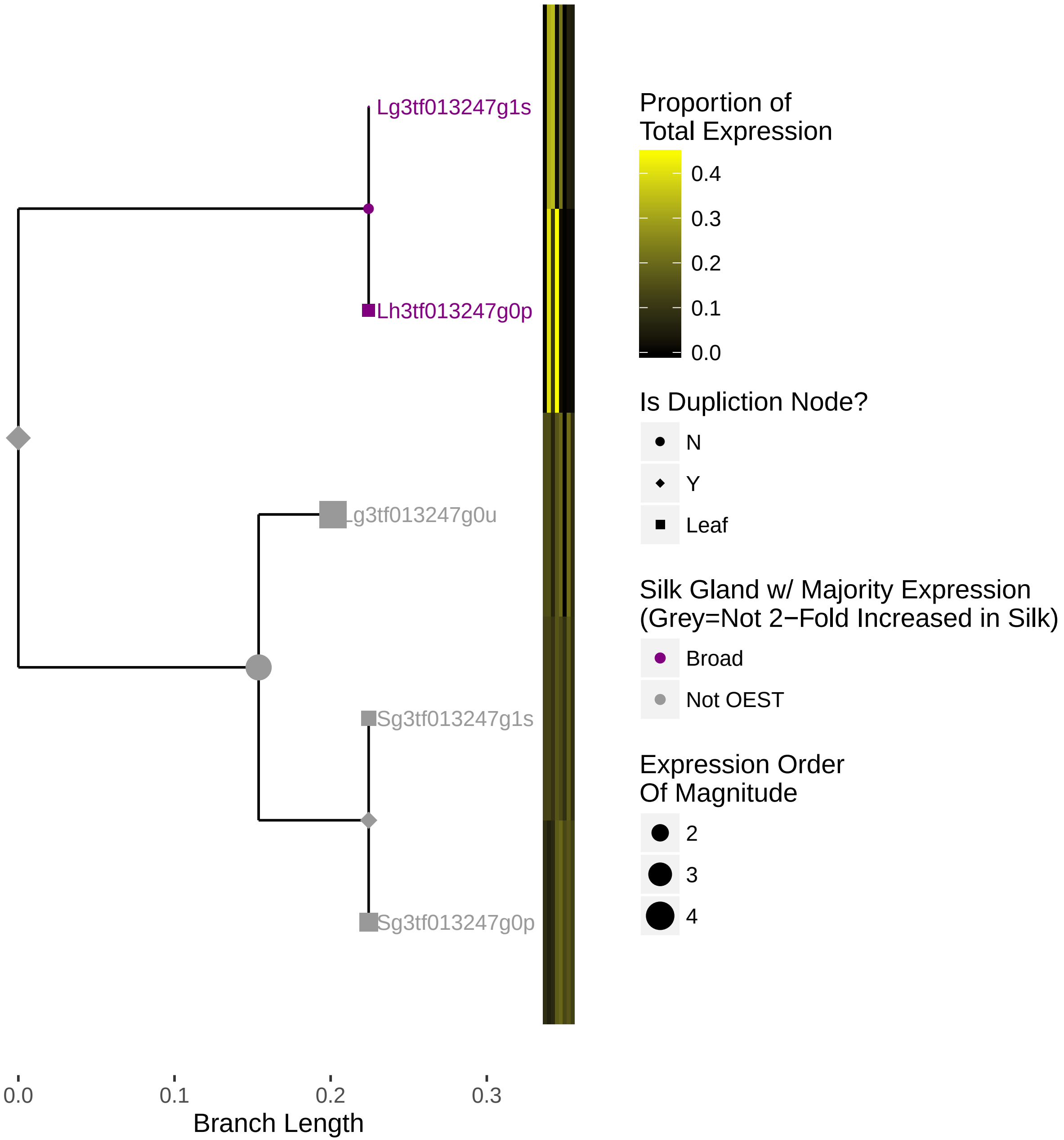
Lg3tf013247g1s
Lh3tf013247g0p
Lg3tf013247g0u
Sg3tf013247g1s
Sg3tf013247g0p
0.0
0.1
0.2
0.3
Branch Length
Proportion of
Total Expression
0.4
0.3
0.2
0.1
0.0
Is Dupliction Node?
N
Y
Leaf
Silk Gland w/ Majority Expression
(Grey=Not 2−Fold Increased in Silk)
Broad
Not OEST
Expression Order
Of Magnitude
2
3
4

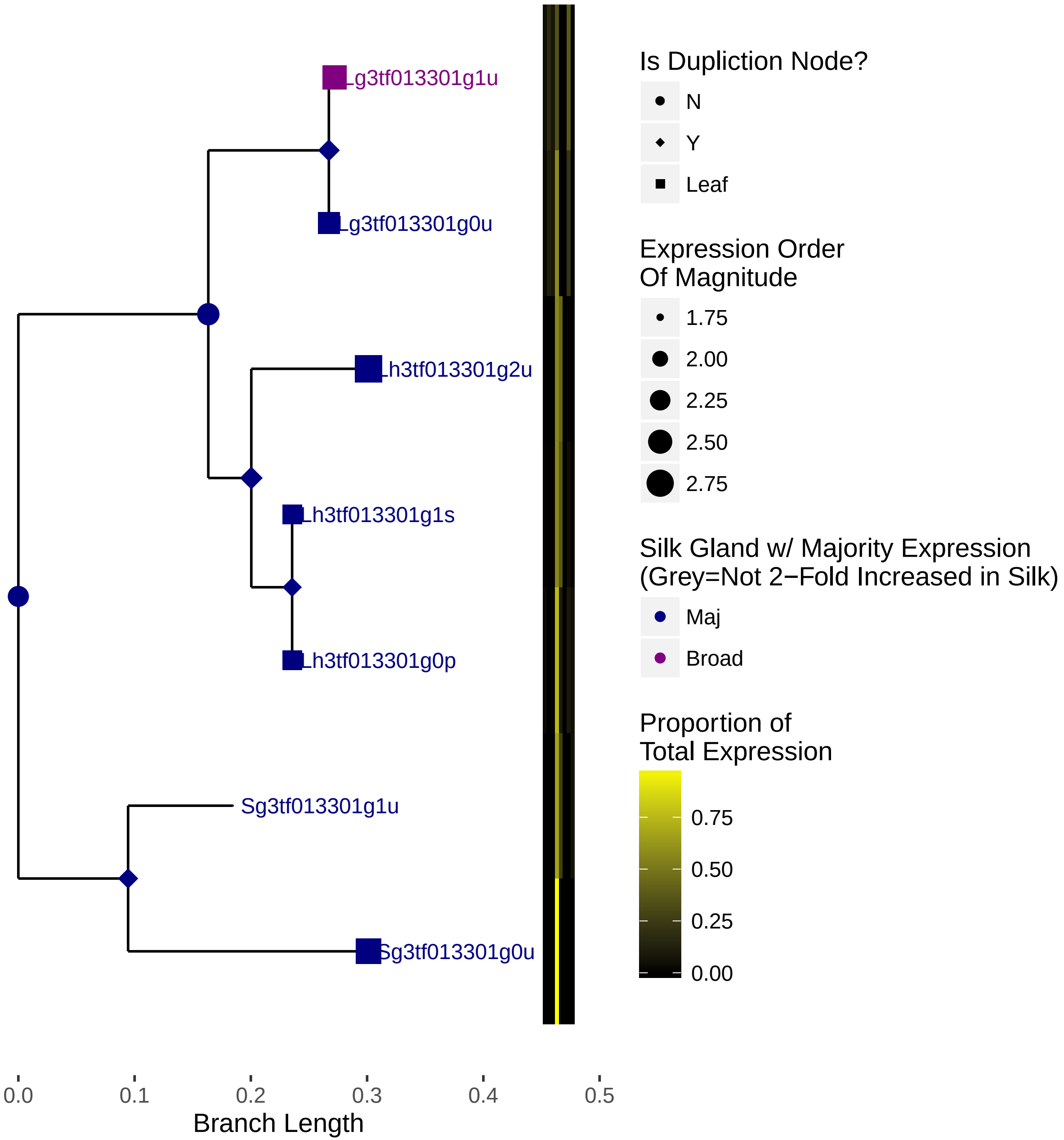
Lg3tf013301g1u
Lg3tf013301g0u
Lh3tf013301g2u
Lh3tf013301g1s
Lh3tf013301g0p
Sg3tf013301g1u
Sg3tf013301g0u
0.0
0.1
0.2
0.3
0.4
0.5
Branch Length
Is Dupliction Node?
N
Y
Leaf
Expression Order
Of Magnitude
1.75
2.00
2.25
2.50
2.75
Silk Gland w/ Majority Expression
(Grey=Not 2−Fold Increased in Silk)
Maj
Broad
Proportion of
Total Expression
0.75
0.50
0.25
0.00

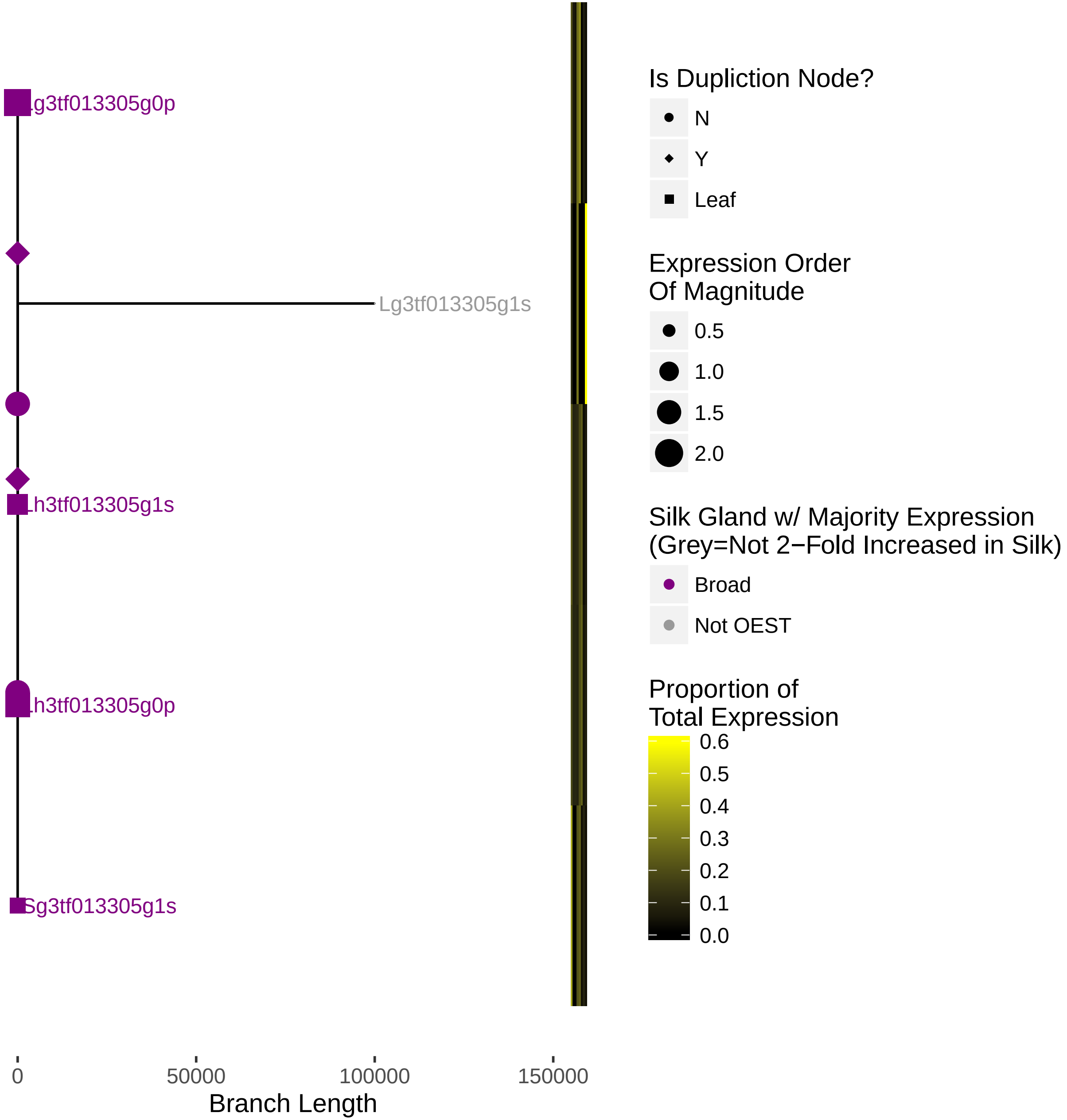

Lg3tf013305g0p
Lg3tf013305g1s
Lh3tf013305g1s
Lh3tf013305g0p
Sg3tf013305g1s
0
50000
100000
150000
Branch Length
Is Dupliction Node?
N
Y
Leaf
Expression Order
Of Magnitude
0.5
1.0
1.5
2.0
Silk Gland w/ Majority Expression
(Grey=Not 2−Fold Increased in Silk)
Broad
Not OEST
Proportion of
Total Expression
0.6
0.5
0.4
0.3
0.2
0.1
0.0

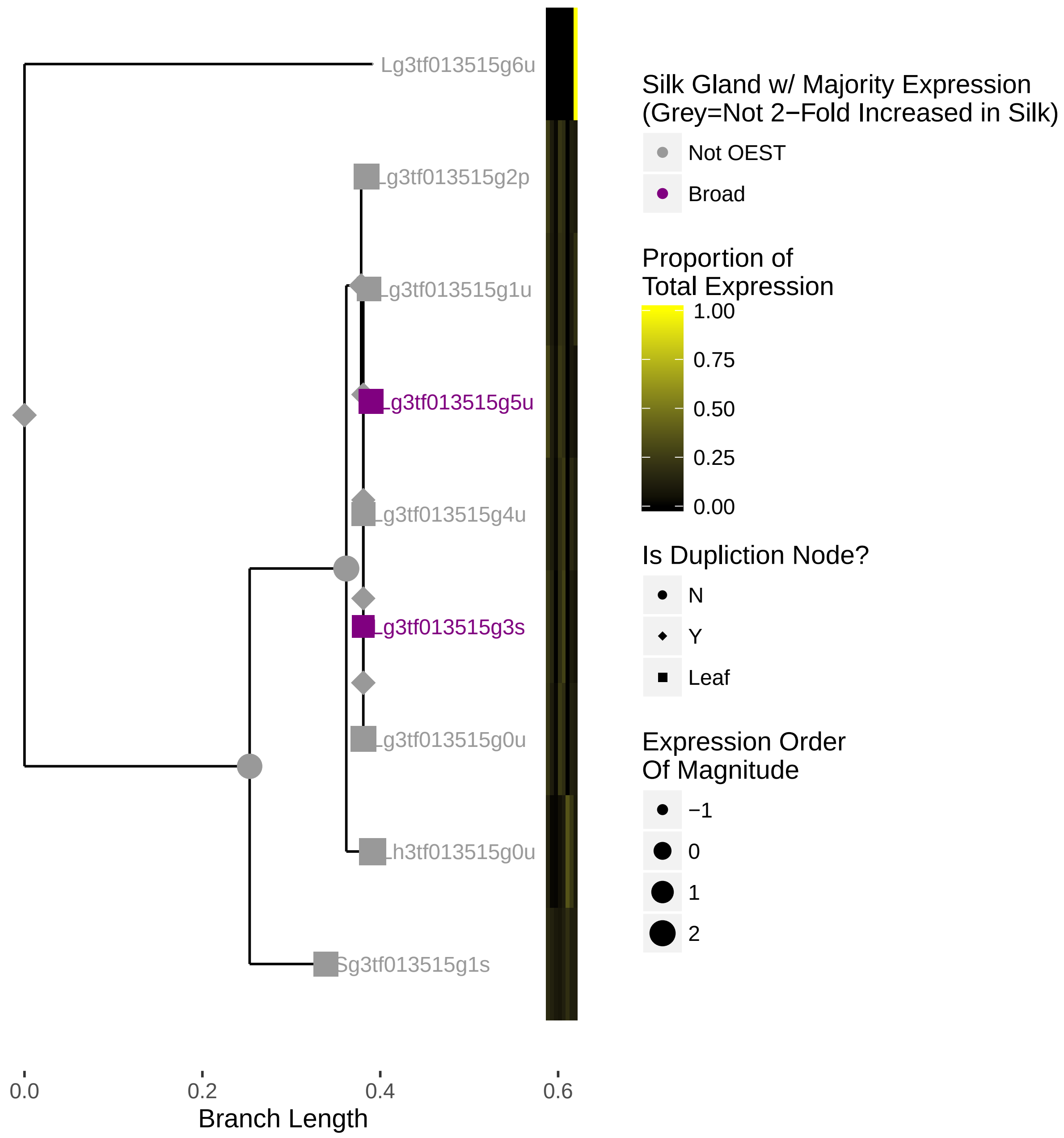
Lg3tf013515g6u
Lg3tf013515g2p
Lg3tf013515g1u
Lg3tf013515g5u
Lg3tf013515g4u
Lg3tf013515g3s
Lg3tf013515g0u
Lh3tf013515g0u
Sg3tf013515g1s
0.0
0.2
0.4
0.6
Branch Length
Silk Gland w/ Majority Expression
(Grey=Not 2−Fold Increased in Silk)
Not OEST
Broad
Proportion of
Total Expression
1.00
0.75
0.50
0.25
0.00
Is Dupliction Node?
N
Y
Leaf
Expression Order
Of Magnitude
−1
0
1
2

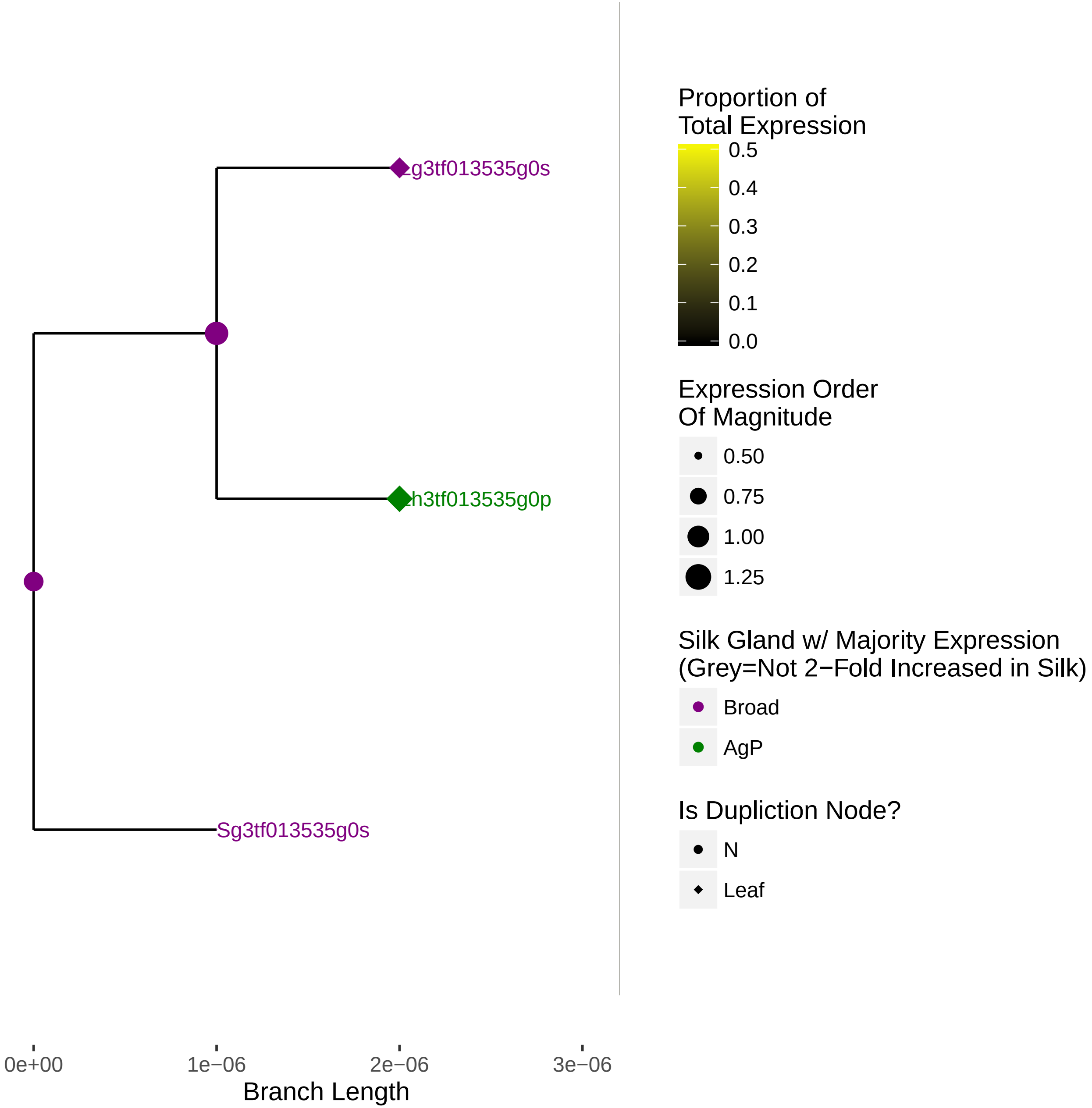

g3tf013535g0s
h3tf013535g0p
Sg3tf013535g0s
0e+00
1e−06
2e−06
3e−06
Branch Length
Proportion of
Total Expression
0.5
0.4
0.3
0.2
0.1
0.0
Expression Order
Of Magnitude
0.50
0.75
1.00
1.25
Silk Gland w/ Majority Expression
(Grey=Not 2−Fold Increased in Silk)
Broad
AgP
Is Dupliction Node?
N
Leaf

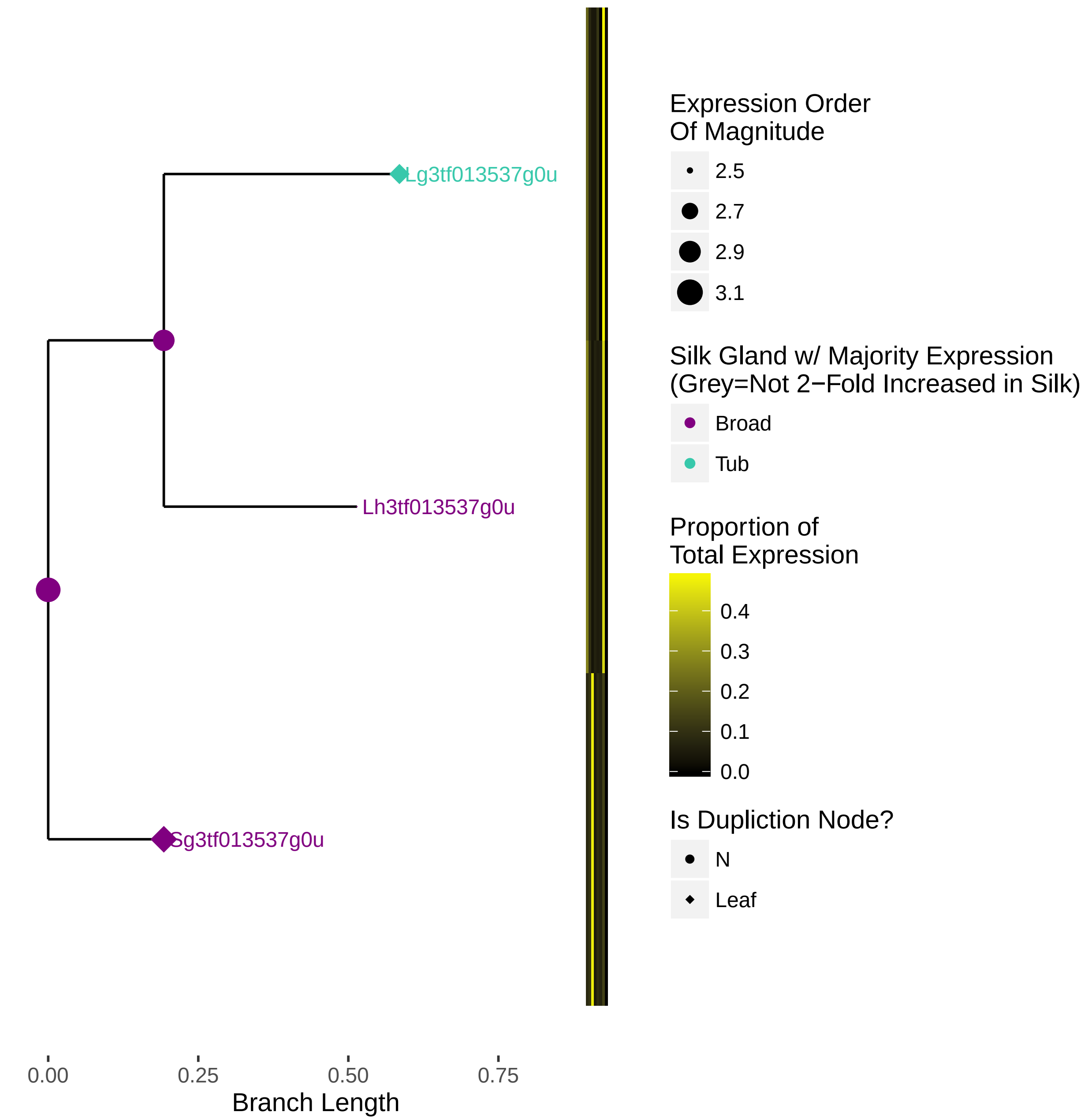
Lg3tf013537g0u
Lh3tf013537g0u
Sg3tf013537g0u
0.00
0.25
0.50
0.75
Branch Length
Expression Order
Of Magnitude
2.5
2.7
2.9
3.1
Silk Gland w/ Majority Expression
(Grey=Not 2−Fold Increased in Silk)
Broad
Tub
Proportion of
Total Expression
0.4
0.3
0.2
0.1
0.0
Is Dupliction Node?
N
Leaf

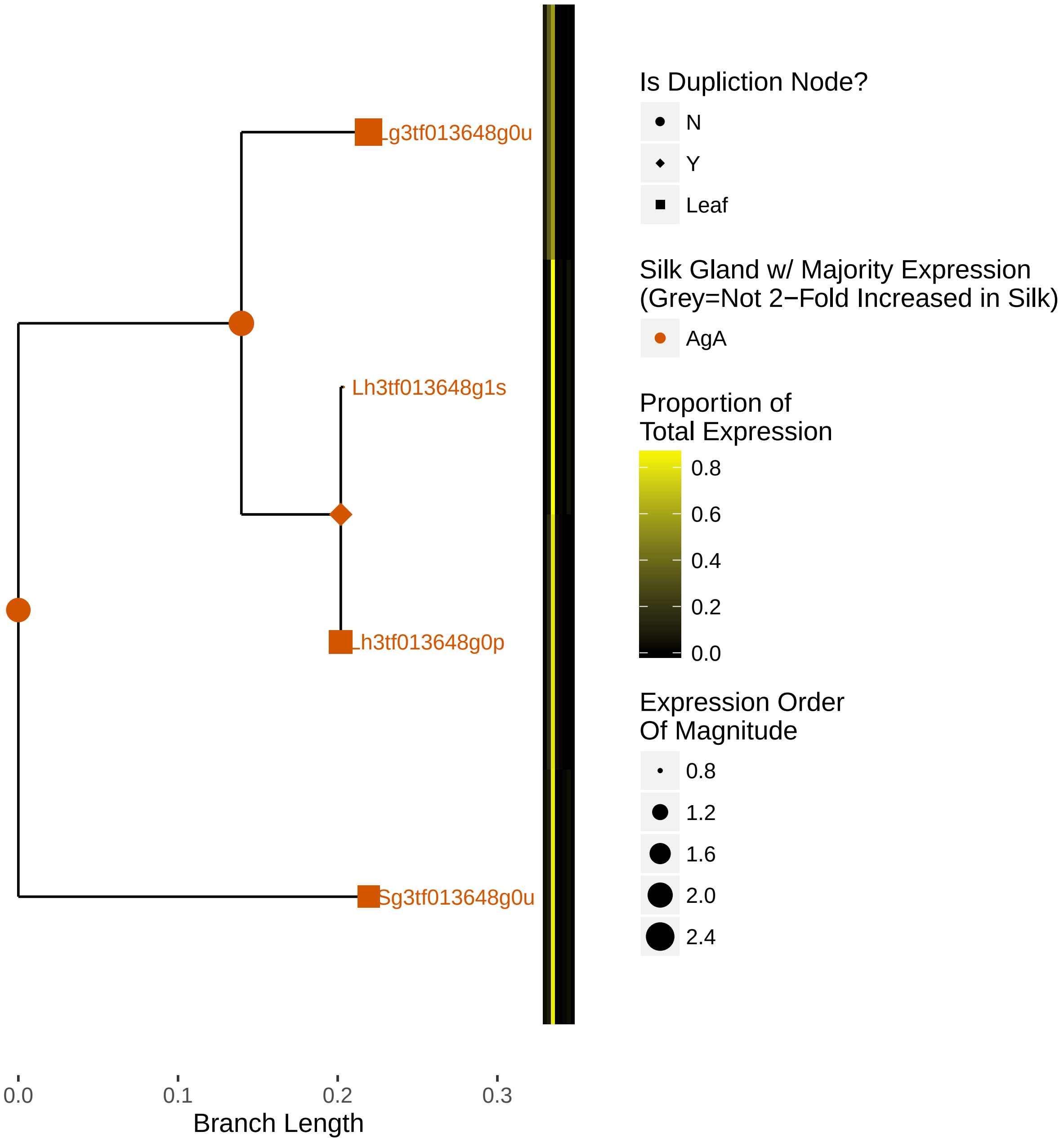

Lg3tf013648g0u
Lh3tf013648g1s
Lh3tf013648g0p
Sg3tf013648g0u
0.0
0.1
0.2
0.3
Branch Length
Is Dupliction Node?
N
Y
Leaf
Silk Gland w/ Majority Expression
(Grey=Not 2−Fold Increased in Silk)
AgA
Proportion of
Total Expression
0.8
0.6
0.4
0.2
0.0
Expression Order
Of Magnitude
0.8
1.2
1.6
2.0
2.4

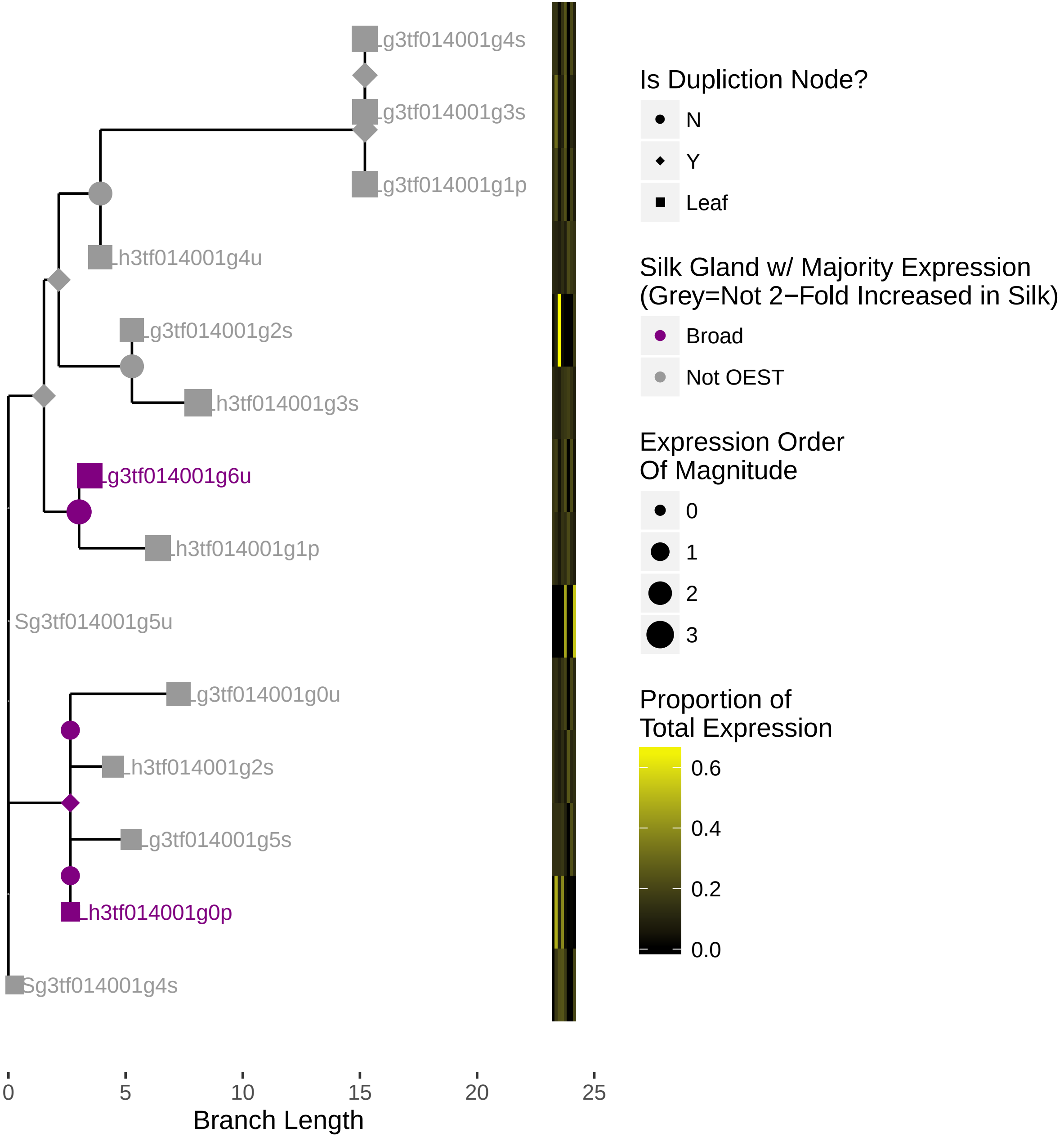

Lg3tf014001g4s
Lg3tf014001g3s
Lg3tf014001g1p
Lh3tf014001g4u
Lg3tf014001g2s
Lh3tf014001g3s
Lg3tf014001g6u
Lh3tf014001g1p
Sg3tf014001g5u
Lg3tf014001g0u
Lh3tf014001g2s
Lg3tf014001g5s
Lh3tf014001g0p
Sg3tf014001g4s
0
5
10
15
20
25
Branch Length
Is Dupliction Node?
N
Y
Leaf
Silk Gland w/ Majority Expression
(Grey=Not 2−Fold Increased in Silk)
Broad
Not OEST
Expression Order
Of Magnitude
0
1
2
3
Proportion of
Total Expression
0.6
0.4
0.2
0.0

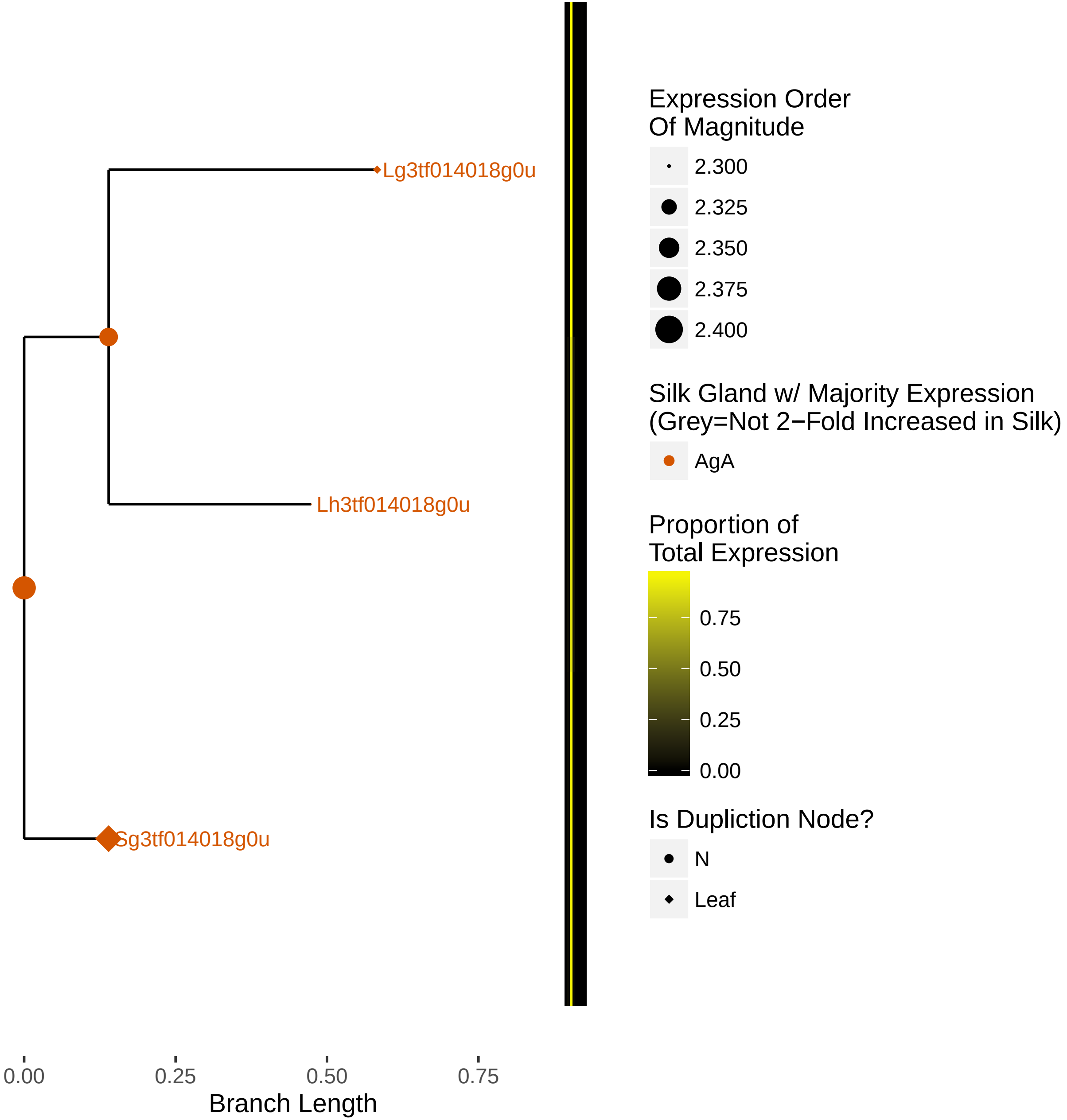

Lg3tf014018g0u
Lh3tf014018g0u
Sg3tf014018g0u
0.00
0.25
0.50
0.75
Branch Length
Expression Order Of Magnitude
2.300
2.325
2.350
2.375
2.400
Silk Gland w/ Majority Expression (Grey=Not 2−Fold Increased in Silk)
AgA
Proportion of Total Expression
0.75
0.50
0.25
0.00
Is Dupliction Node?
N
Leaf

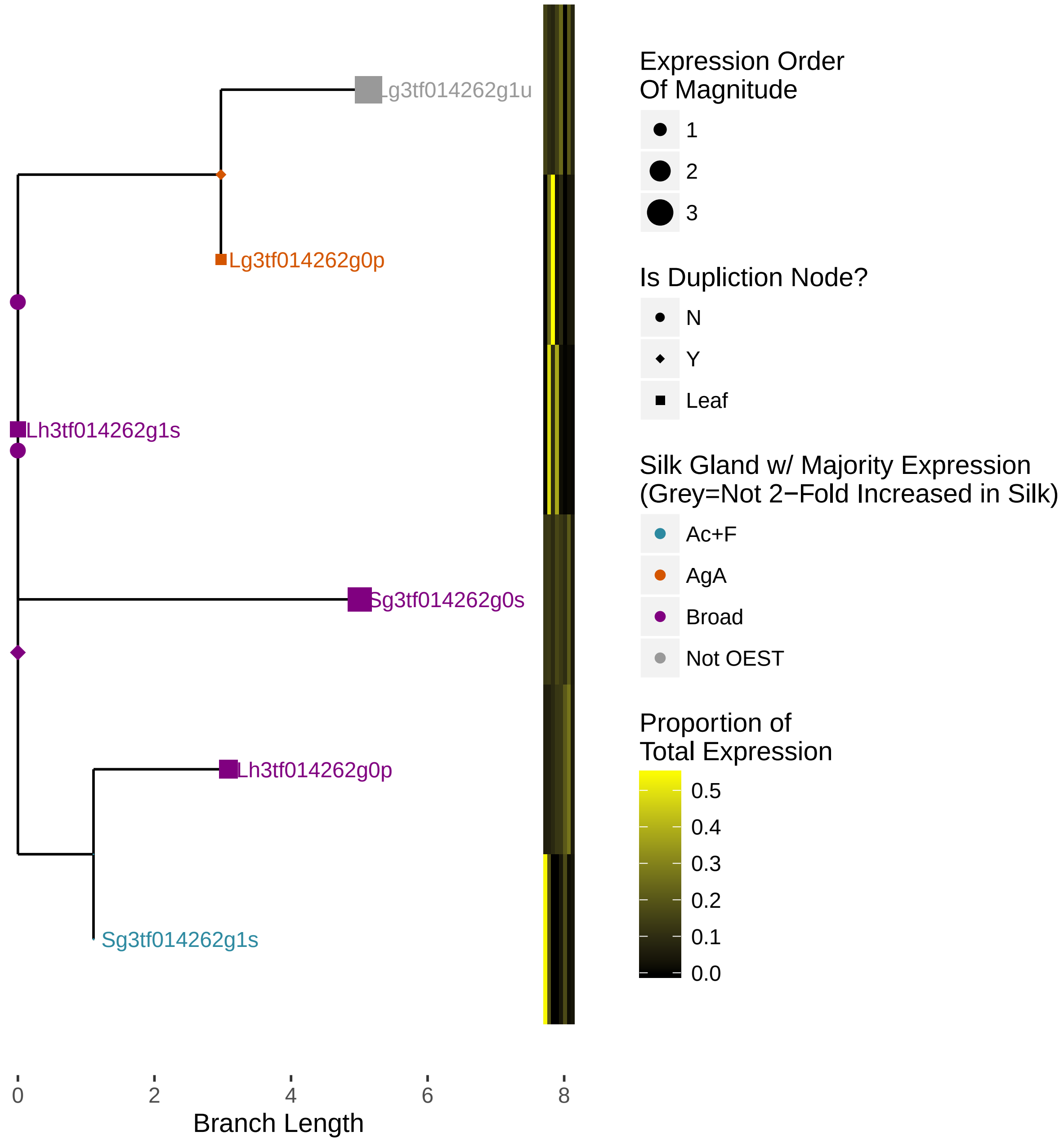

Lg3tf014262g1u
Lg3tf014262g0p
Lh3tf014262g1s
Sg3tf014262g0s
Lh3tf014262g0p
Sg3tf014262g1s
0
2
4
6
8
Branch Length
Expression Order
Of Magnitude
1
2
3
Is Dupliction Node?
N
Y
Leaf
Silk Gland w/ Majority Expression
(Grey=Not 2−Fold Increased in Silk)
Ac+F
AgA
Broad
Not OEST
Proportion of
Total Expression
0.5
0.4
0.3
0.2
0.1
0.0

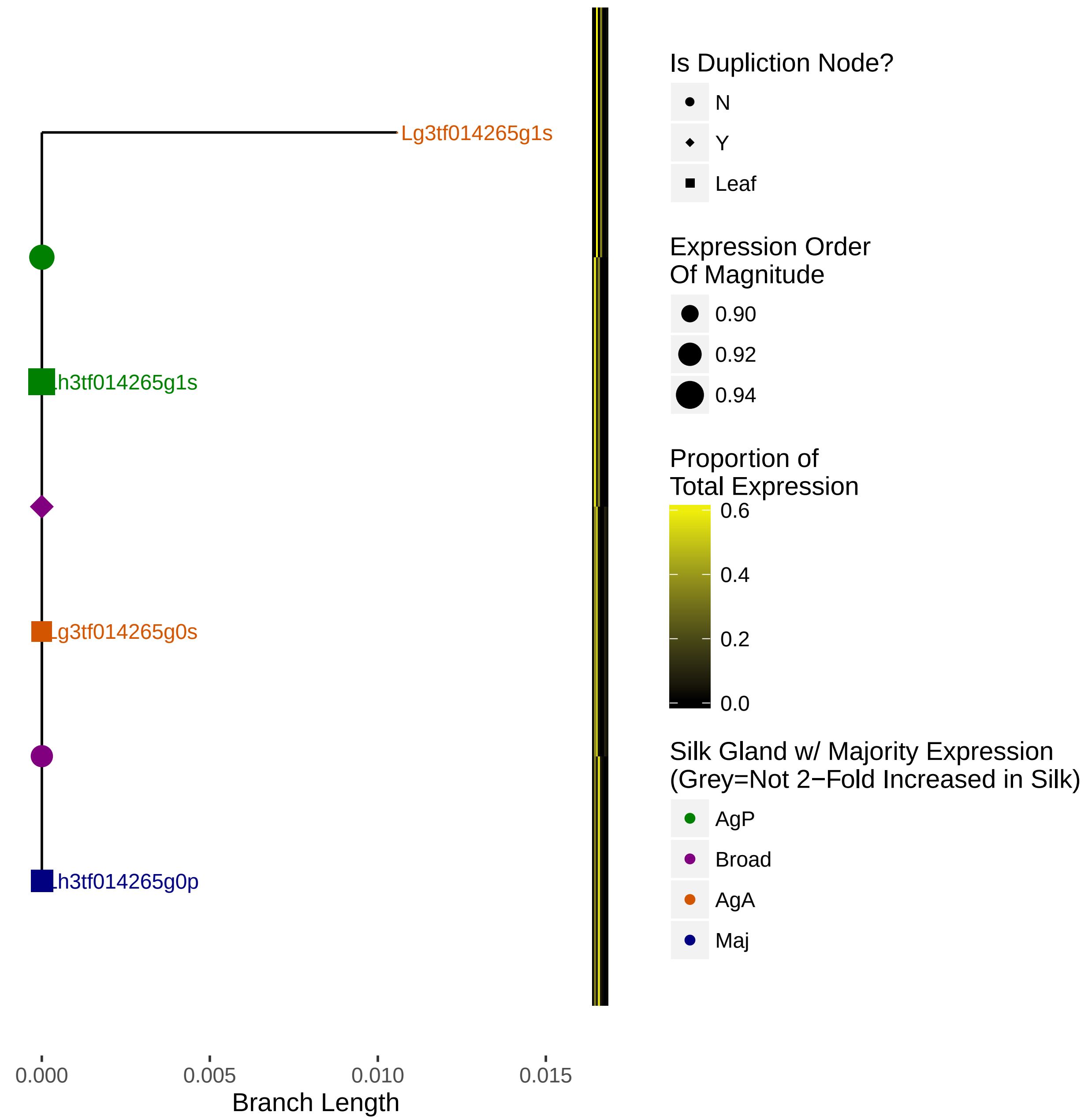

Lg3tf014265g1s
Lh3tf014265g1s
Lg3tf014265g0s
Lh3tf014265g0p
0.000
0.005
0.010
0.015
Branch Length
Is Dupliction Node?
N
Y
Leaf
Expression Order
Of Magnitude
0.90
0.92
0.94
Proportion of
Total Expression
0.6
0.4
0.2
0.0
Silk Gland w/ Majority Expression
(Grey=Not 2−Fold Increased in Silk)
AgP
Broad
AgA
Maj

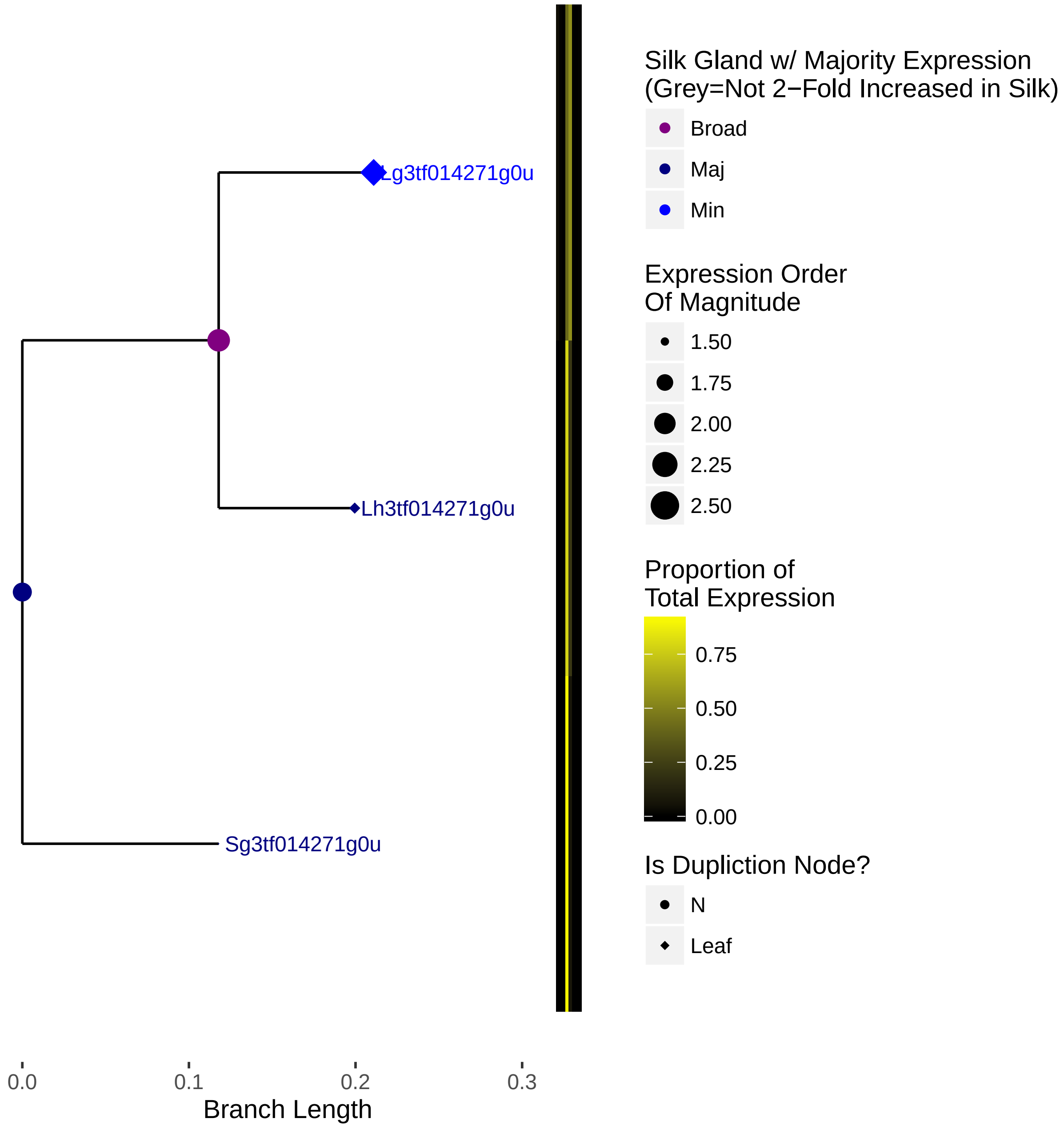
Lg3tf014271g0u
Lh3tf014271g0u
Sg3tf014271g0u
0.0
0.1
0.2
0.3
Branch Length
Silk Gland w/ Majority Expression
(Grey=Not 2−Fold Increased in Silk)
Broad
Maj
Min
Expression Order
Of Magnitude
1.50
1.75
2.00
2.25
2.50
Proportion of
Total Expression
0.75
0.50
0.25
0.00
Is Dupliction Node?
N
Leaf

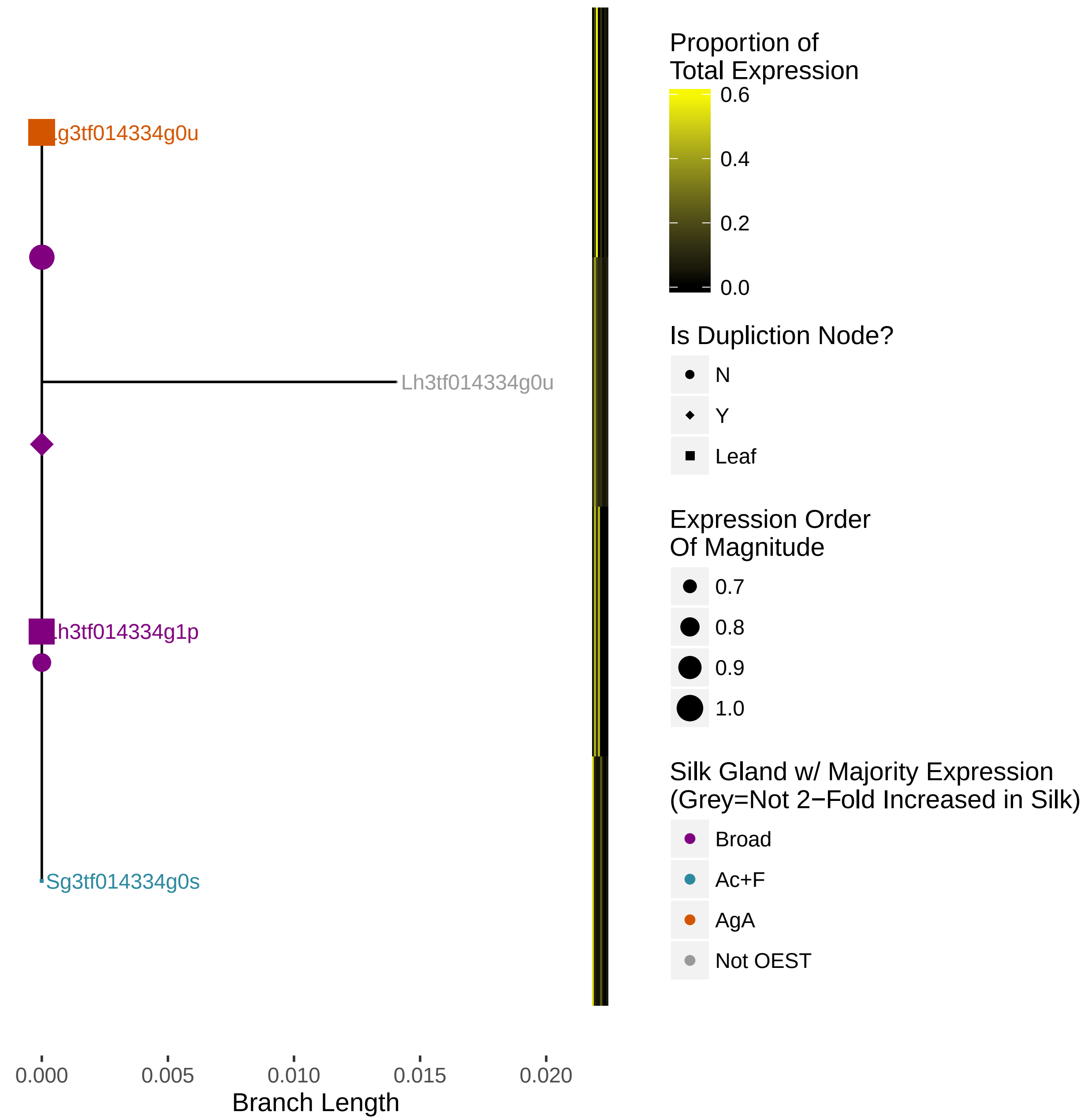

Proportion of Total Expression
0.6
0.4
0.2
0.0
Is Dupliction Node?
N
Y
Leaf
Expression Order Of Magnitude
0.7
0.8
0.9
1.0
Silk Gland w/ Majority Expression (Grey=Not 2−Fold Increased in Silk)
Broad
Ac+F
AgA
Not OEST
g3tf014334g0u
Lh3tf014334g0u
h3tf014334g1p
Sg3tf014334g0s
0.000
0.005
0.010
0.015
0.020
Branch Length

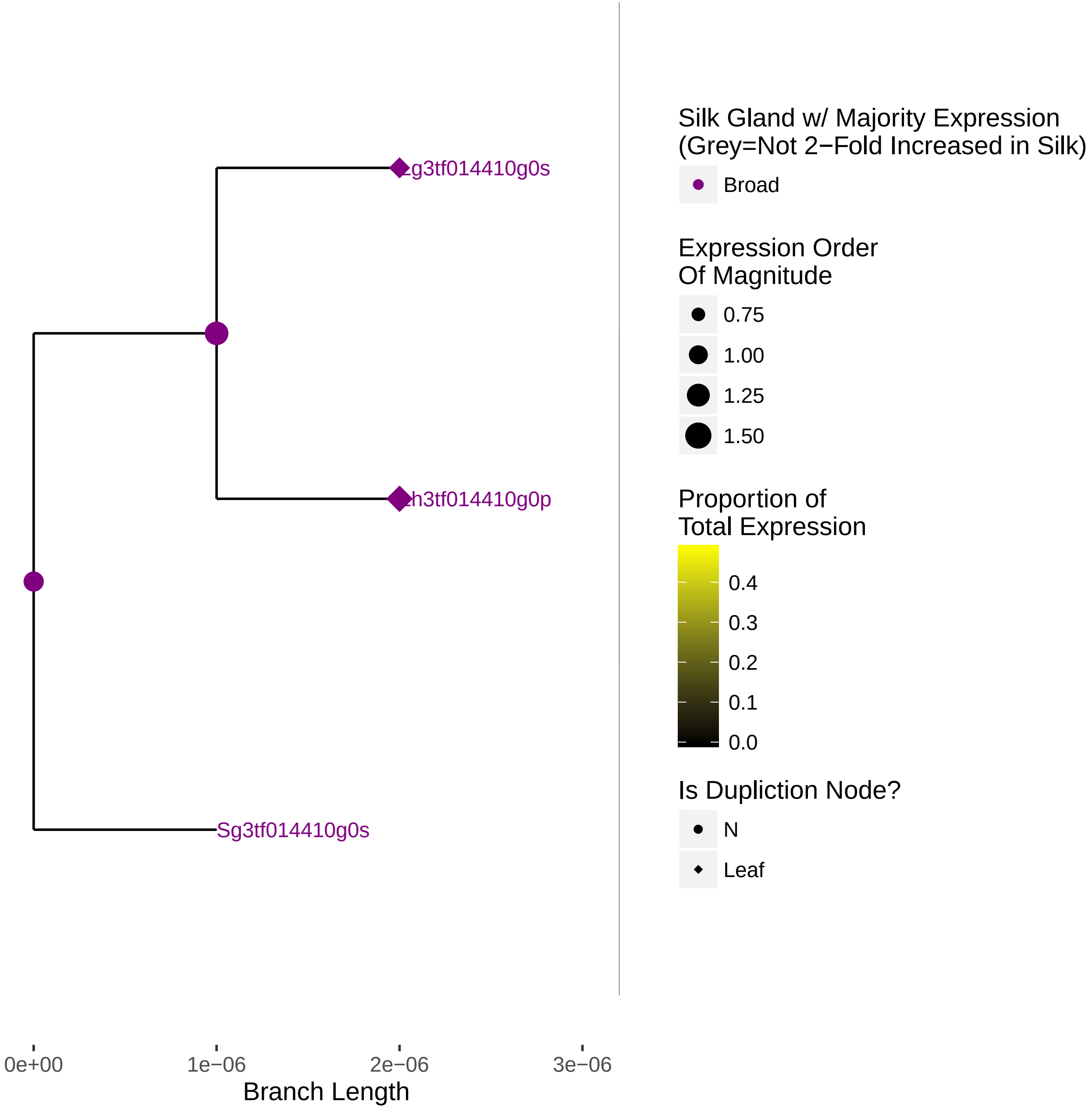

g3tf014410g0s
h3tf014410g0p
Sg3tf014410g0s
0e+00
1e−06
2e−06
3e−06
Branch Length
Silk Gland w/ Majority Expression
(Grey=Not 2−Fold Increased in Silk)
Broad
Expression Order
Of Magnitude
0.75
1.00
1.25
1.50
Proportion of
Total Expression
0.4
0.3
0.2
0.1
0.0
Is Dupliction Node?
N
Leaf

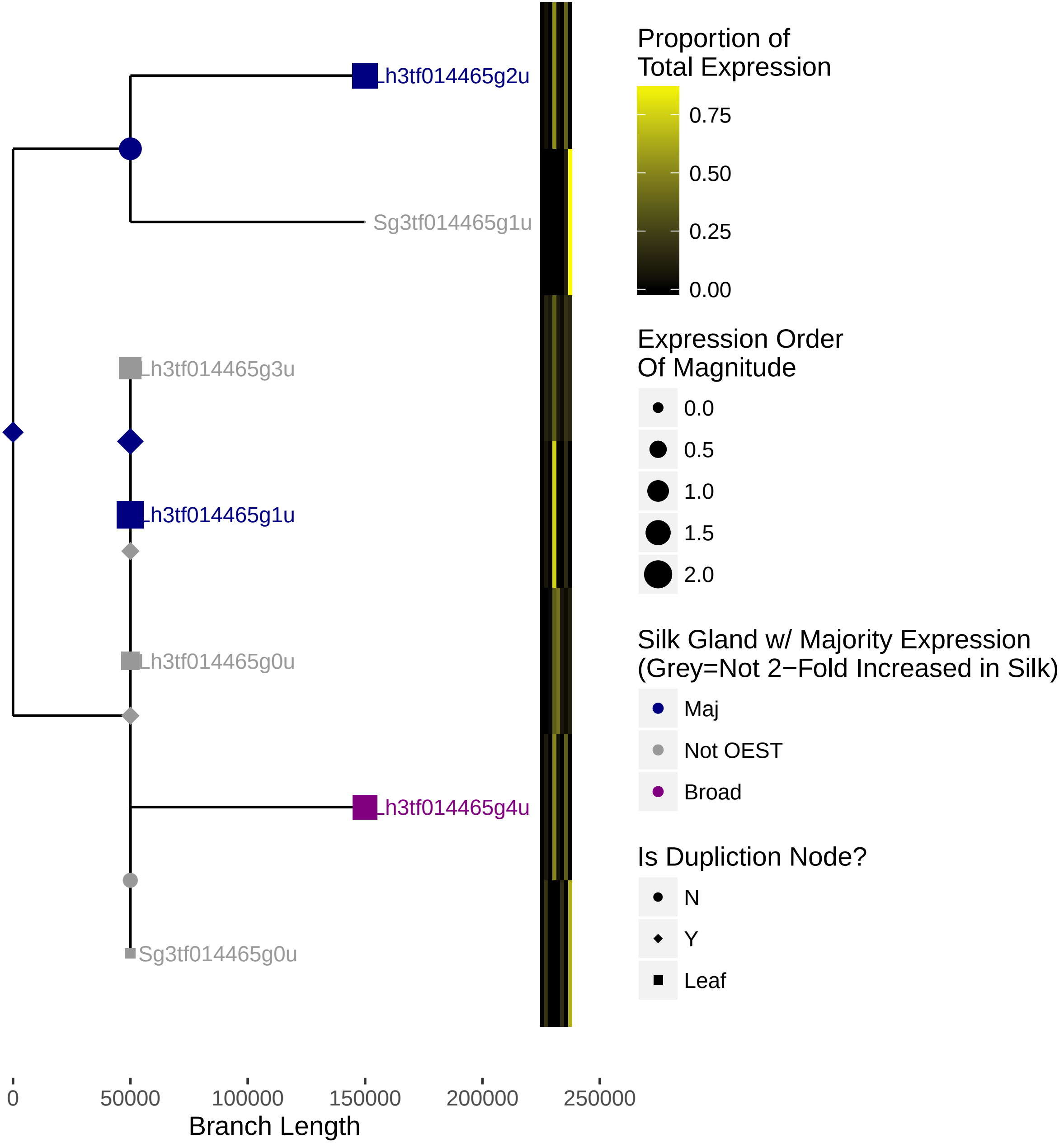

Lh3tf014465g2u
Sg3tf014465g1u
Lh3tf014465g3u
Lh3tf014465g1u
Lh3tf014465g0u
Lh3tf014465g4u
Sg3tf014465g0u
0
50000
100000
150000
200000
250000
Branch Length
Proportion of Total Expression
0.75
0.50
0.25
0.00
Expression Order Of Magnitude
0.0
0.5
1.0
1.5
2.0
Silk Gland w/ Majority Expression (Grey=Not 2−Fold Increased in Silk)
Maj
Not OEST
Broad
Is Dupliction Node?
N
Y
Leaf

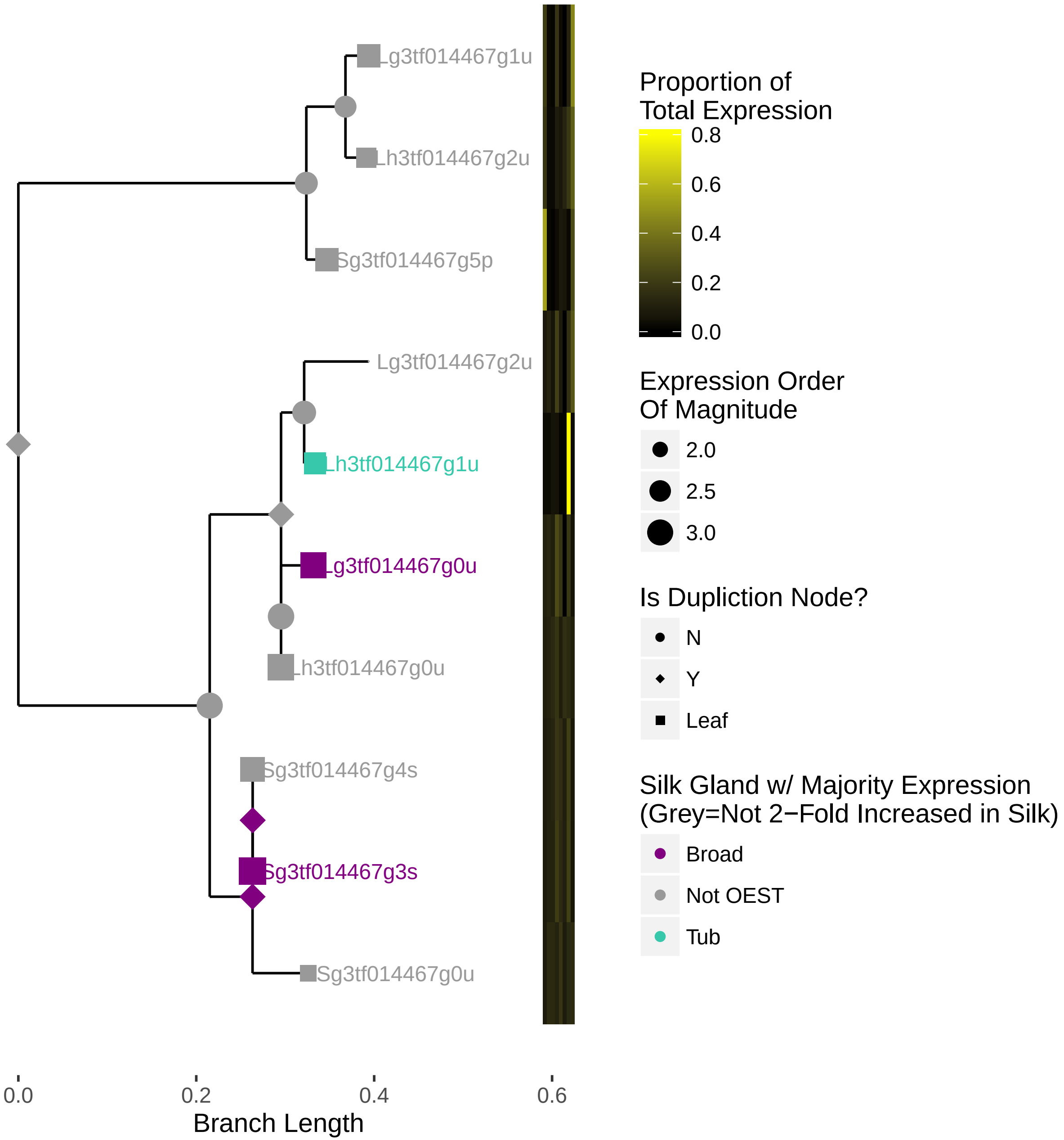

Lg3tf014467g1u
Lh3tf014467g2u
Sg3tf014467g5p
Lg3tf014467g2u
Lh3tf014467g1u
Lg3tf014467g0u
Lh3tf014467g0u
Sg3tf014467g4s
Sg3tf014467g3s
Sg3tf014467g0u
0.0
0.2
0.4
0.6
Branch Length
Proportion of Total Expression
0.8
0.6
0.4
0.2
0.0
Expression Order Of Magnitude
2.0
2.5
3.0
Is Dupliction Node?
N
Y
Leaf
Silk Gland w/ Majority Expression (Grey=Not 2−Fold Increased in Silk)
Broad
Not OEST
Tub

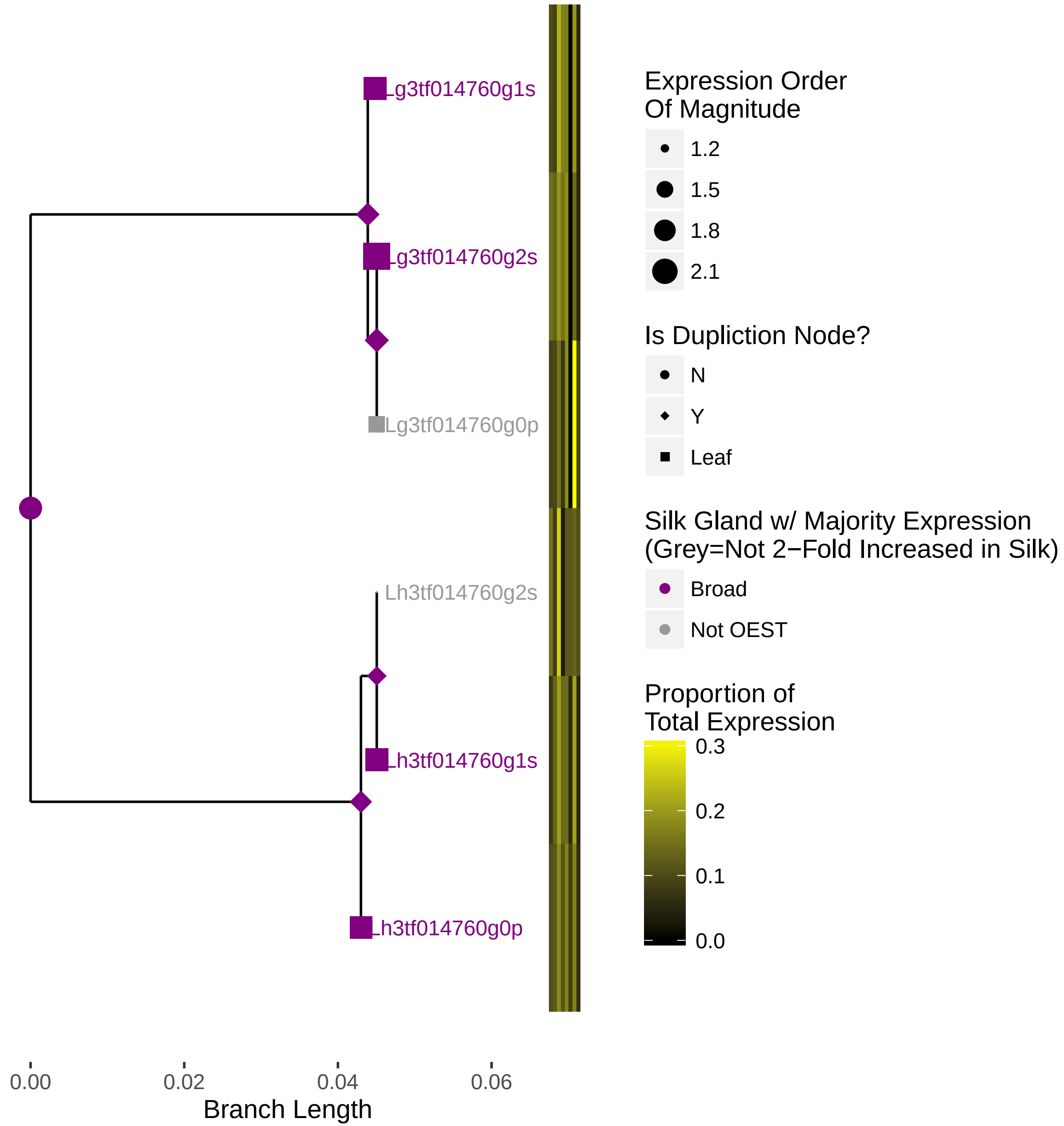

Lg3tf014760g1s
Lg3tf014760g2s
Lg3tf014760g0p
Lh3tf014760g2s
Lh3tf014760g1s
Lh3tf014760g0p
0.00
0.02
0.04
0.06
Branch Length
Expression Order
Of Magnitude
1.2
1.5
1.8
2.1
Is Dupliction Node?
N
Y
Leaf
Silk Gland w/ Majority Expression
(Grey=Not 2−Fold Increased in Silk)
Broad
Not OEST
Proportion of
Total Expression
0.3
0.2
0.1
0.0

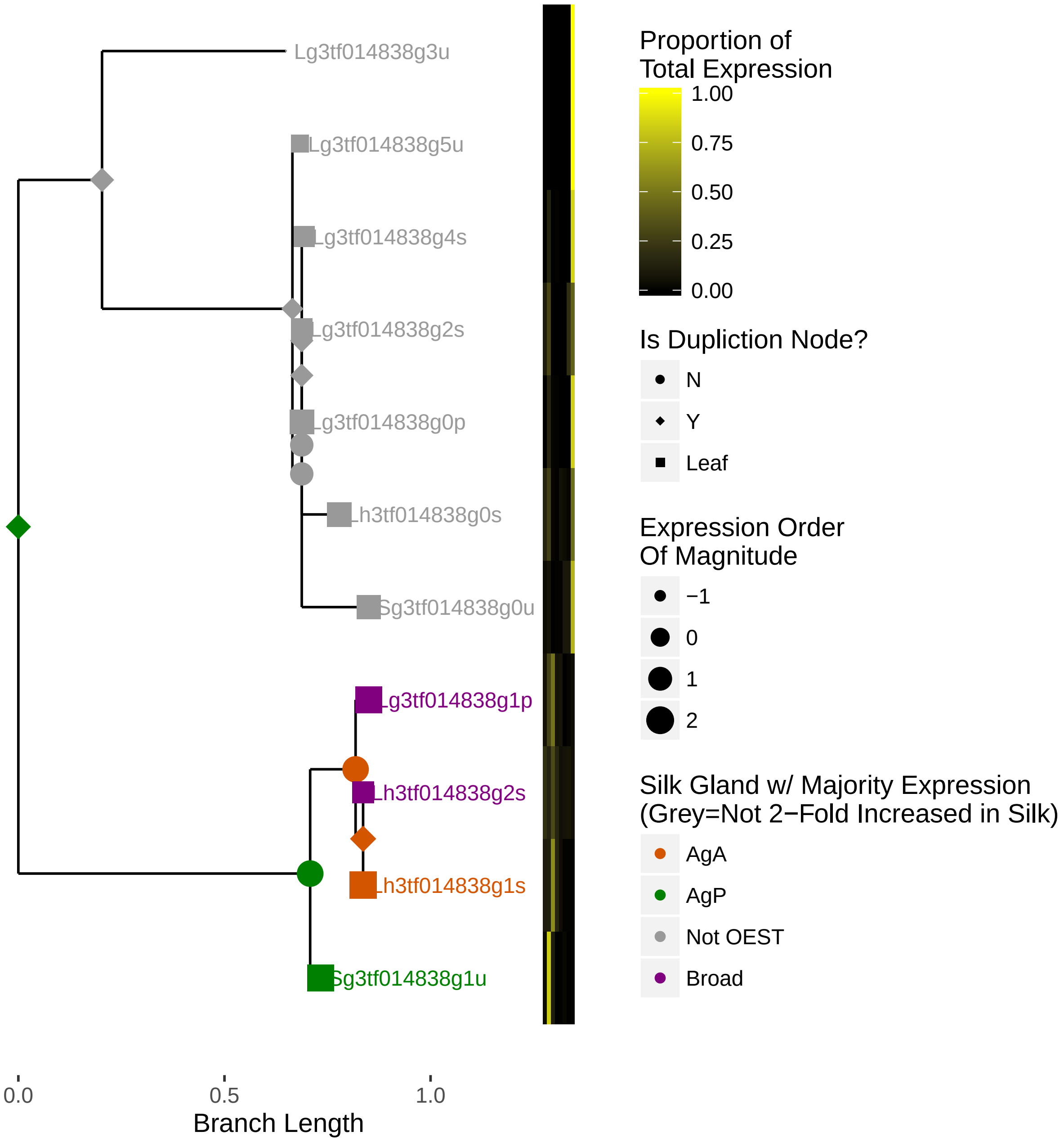

Lg3tf014838g3u
Lg3tf014838g5u
Lg3tf014838g4s
Lg3tf014838g2s
Lg3tf014838g0p
Lh3tf014838g0s
Sg3tf014838g0u
Lg3tf014838g1p
Lh3tf014838g2s
Lh3tf014838g1s
Sg3tf014838g1u
0.0
0.5
1.0
Branch Length
Proportion of
Total Expression
1.00
0.75
0.50
0.25
0.00
Is Dupliction Node?
N
Y
Leaf
Expression Order
Of Magnitude
−1
0
1
2
Silk Gland w/ Majority Expression
(Grey=Not 2−Fold Increased in Silk)
AgA
AgP
Not OEST
Broad

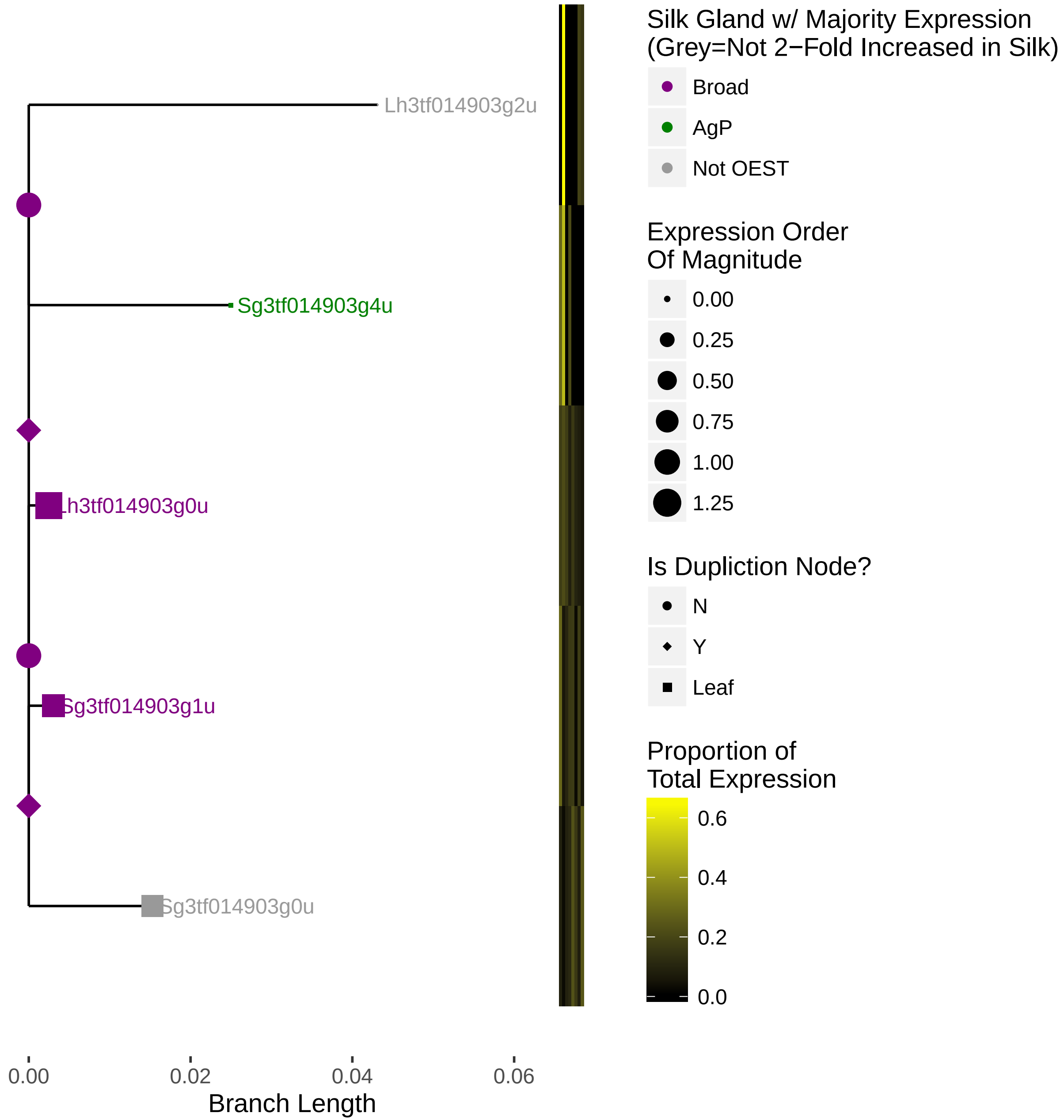

Lh3tf014903g2u
Sg3tf014903g4u
Lh3tf014903g0u
Sg3tf014903g1u
Sg3tf014903g0u
0.00
0.02
0.04
0.06
Branch Length
Silk Gland w/ Majority Expression
(Grey=Not 2−Fold Increased in Silk)
Broad
AgP
Not OEST
Expression Order
Of Magnitude
0.00
0.25
0.50
0.75
1.00
1.25
Is Dupliction Node?
N
Y
Leaf
Proportion of
Total Expression
0.6
0.4
0.2
0.0

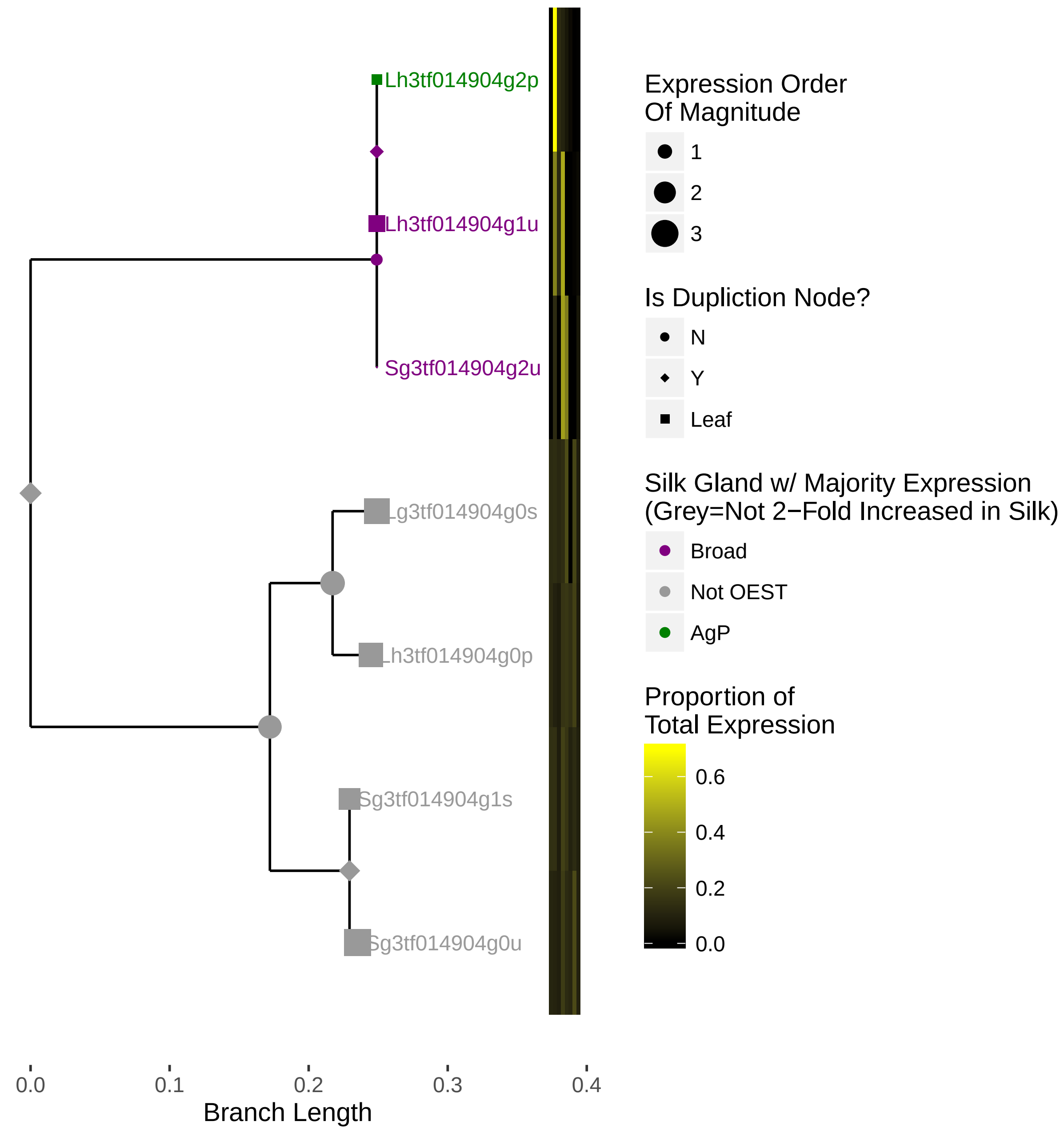
Lh3tf014904g2p
Lh3tf014904g1u
Sg3tf014904g2u
Lg3tf014904g0s
Lh3tf014904g0p
Sg3tf014904g1s
Sg3tf014904g0u
0.0
0.1
0.2
0.3
0.4
Branch Length
Expression Order Of Magnitude
1
2
3
Is Dupliction Node?
N
Y
Leaf
Silk Gland w/ Majority Expression (Grey=Not 2−Fold Increased in Silk)
Broad
Not OEST
AgP
Proportion of Total Expression
0.6
0.4
0.2
0.0

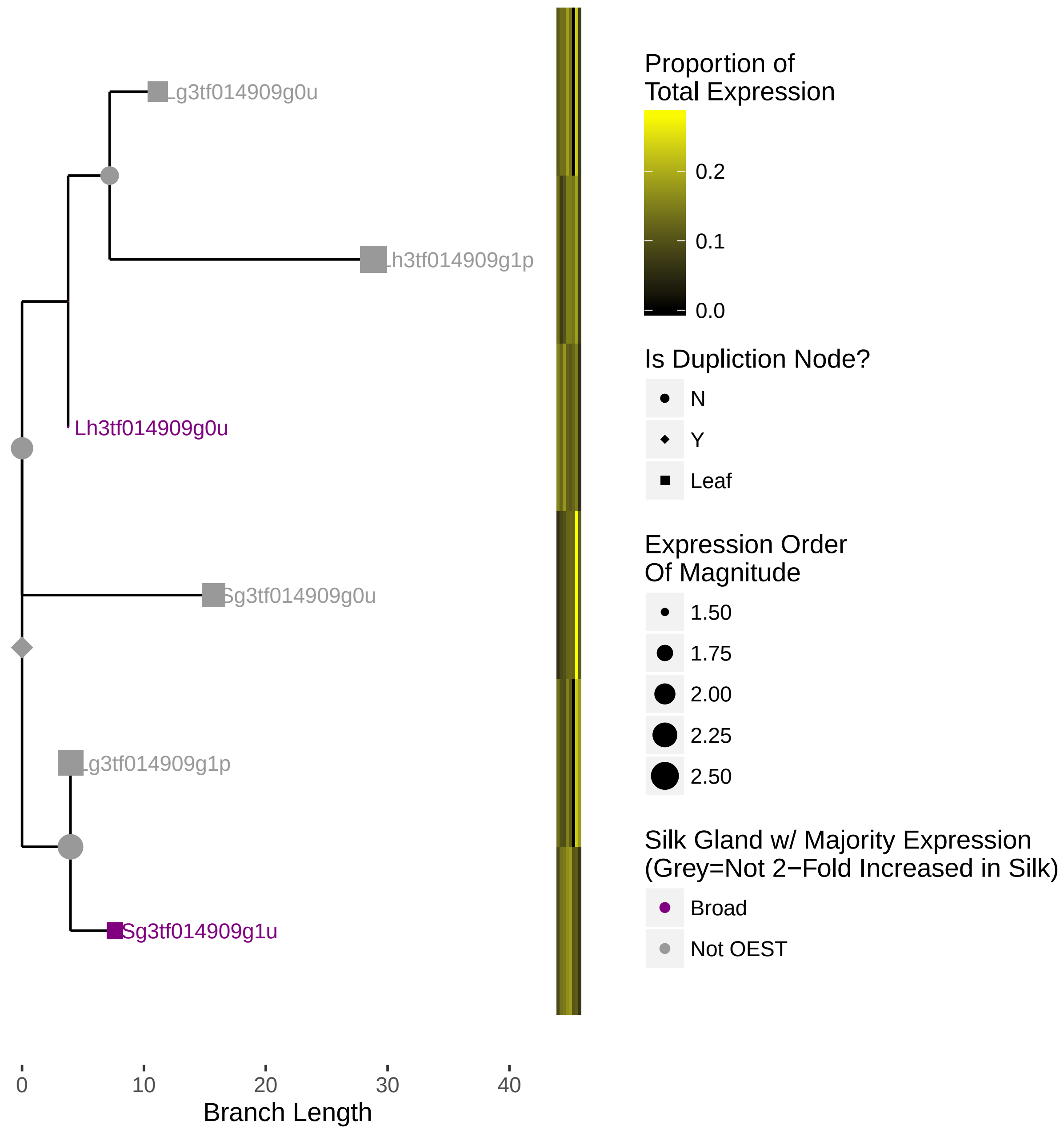

Lg3tf014909g0u
Lh3tf014909g1p
Lh3tf014909g0u
Sg3tf014909g0u
Lg3tf014909g1p
Sg3tf014909g1u
0
10
20
30
40
Branch Length
Proportion of
Total Expression
0.2
0.1
0.0
Is Dupliction Node?
N
Y
Leaf
Expression Order
Of Magnitude
1.50
1.75
2.00
2.25
2.50
Silk Gland w/ Majority Expression
(Grey=Not 2−Fold Increased in Silk)
Broad
Not OEST

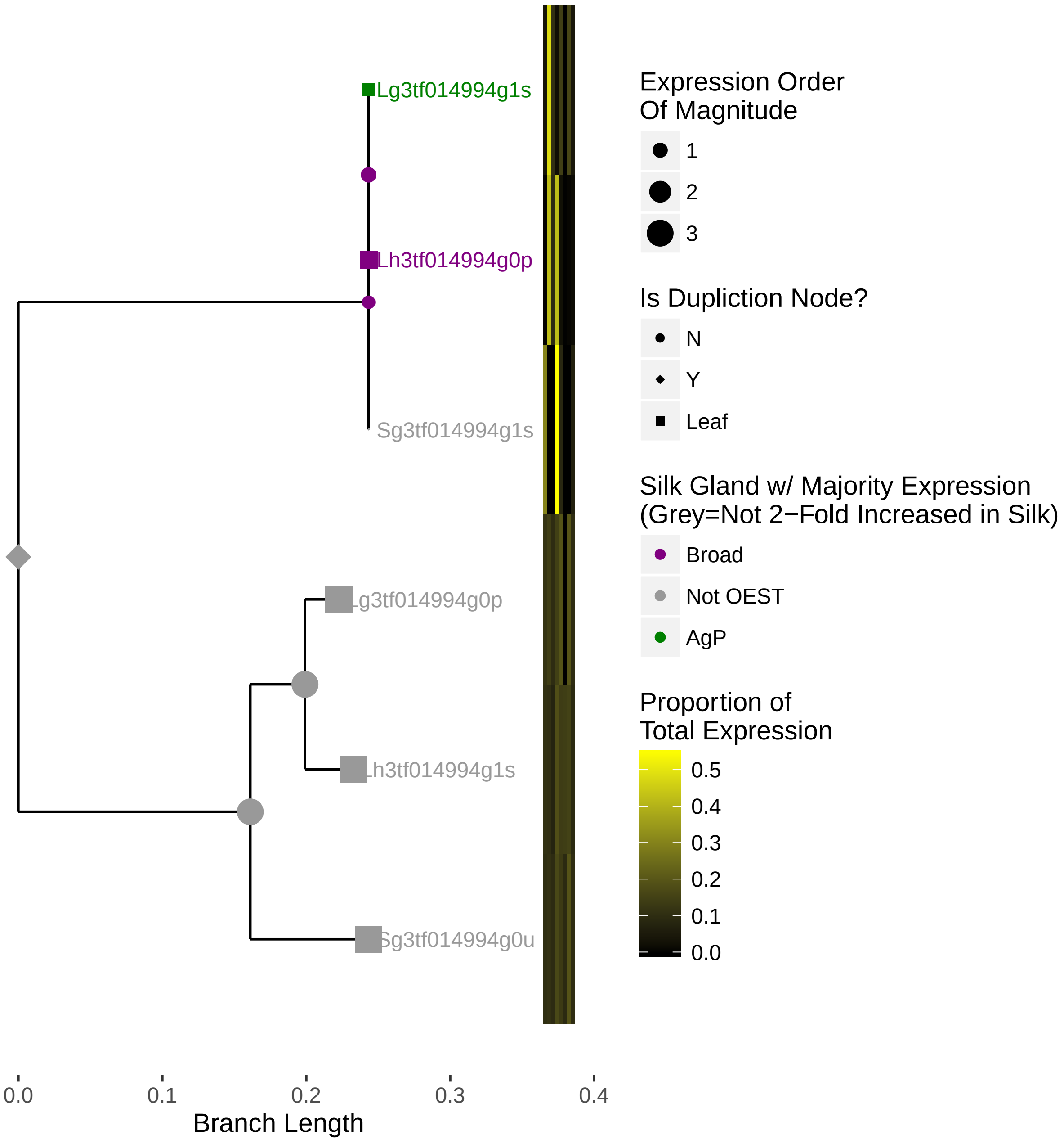
Lg3tf014994g1s
Lh3tf014994g0p
Sg3tf014994g1s
Lg3tf014994g0p
Lh3tf014994g1s
Sg3tf014994g0u
0.0
0.1
0.2
0.3
0.4
Branch Length
Expression Order
Of Magnitude
1
2
3
Is Dupliction Node?
N
Y
Leaf
Silk Gland w/ Majority Expression
(Grey=Not 2−Fold Increased in Silk)
Broad
Not OEST
AgP
Proportion of
Total Expression
0.5
0.4
0.3
0.2
0.1
0.0

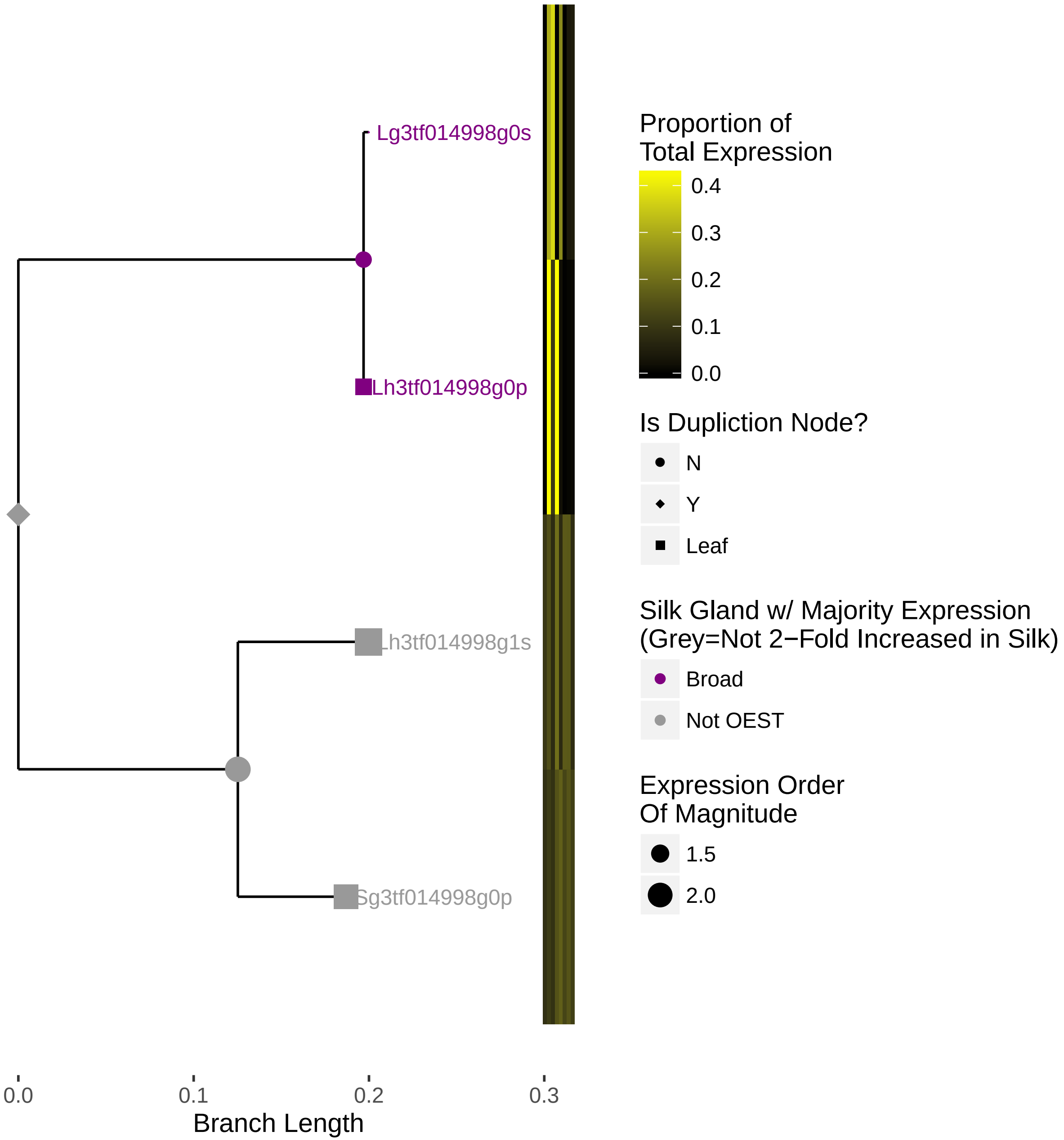

Lg3tf014998g0s
Lh3tf014998g0p
Lh3tf014998g1s
Sg3tf014998g0p
0.0
0.1
0.2
0.3
Branch Length
Proportion of
Total Expression
0.4
0.3
0.2
0.1
0.0
Is Dupliction Node?
N
Y
Leaf
Silk Gland w/ Majority Expression
(Grey=Not 2−Fold Increased in Silk)
Broad
Not OEST
Expression Order
Of Magnitude
1.5
2.0

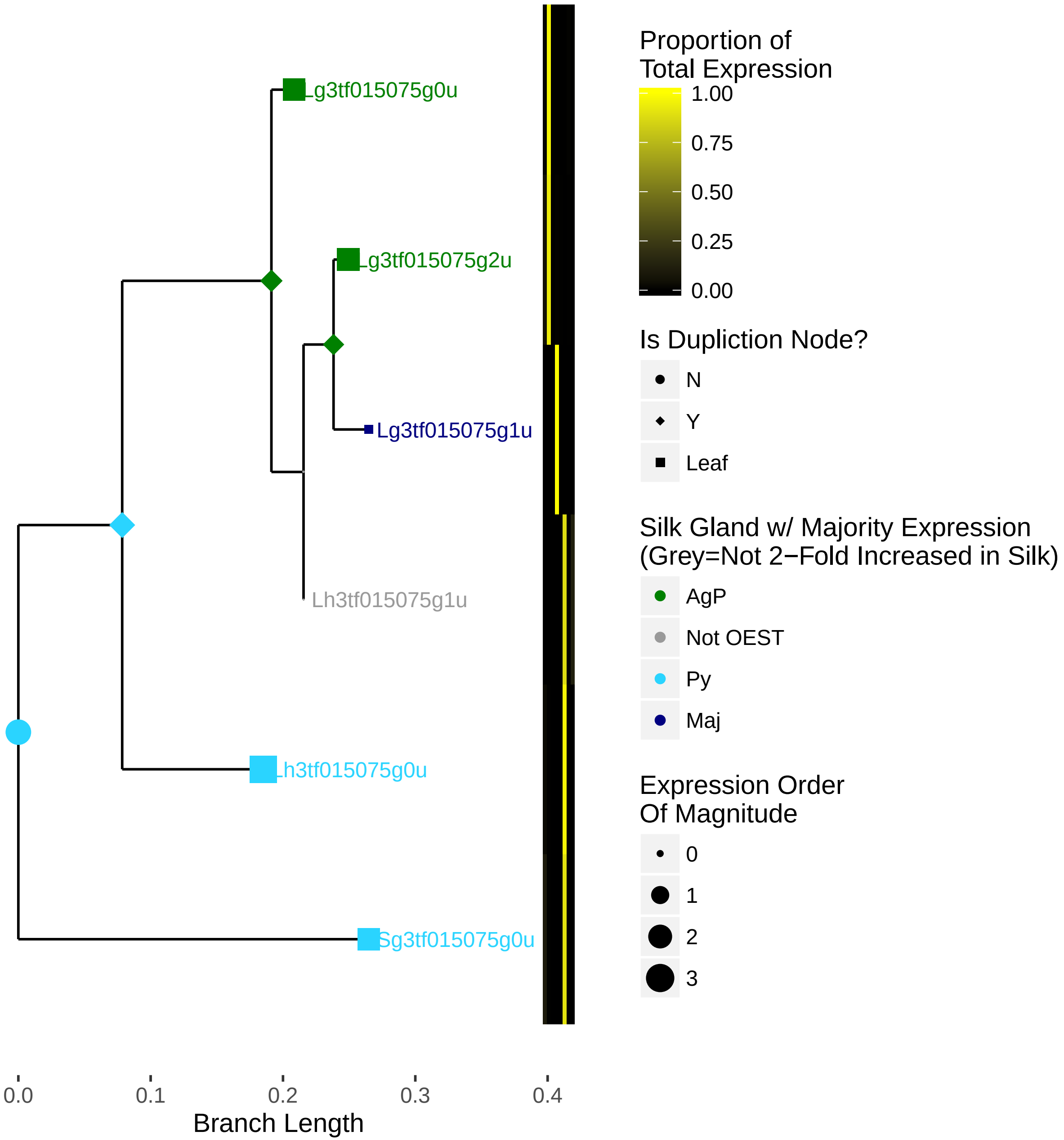

Lg3tf015075g0u
Lg3tf015075g2u
Lg3tf015075g1u
Lh3tf015075g1u
Lh3tf015075g0u
Sg3tf015075g0u
0.0
0.1
0.2
0.3
0.4
Branch Length
Proportion of
Total Expression
1.00
0.75
0.50
0.25
0.00
Is Dupliction Node?
N
Y
Leaf
Silk Gland w/ Majority Expression
(Grey=Not 2−Fold Increased in Silk)
AgP
Not OEST
Py
Maj
Expression Order
Of Magnitude
0
1
2
3

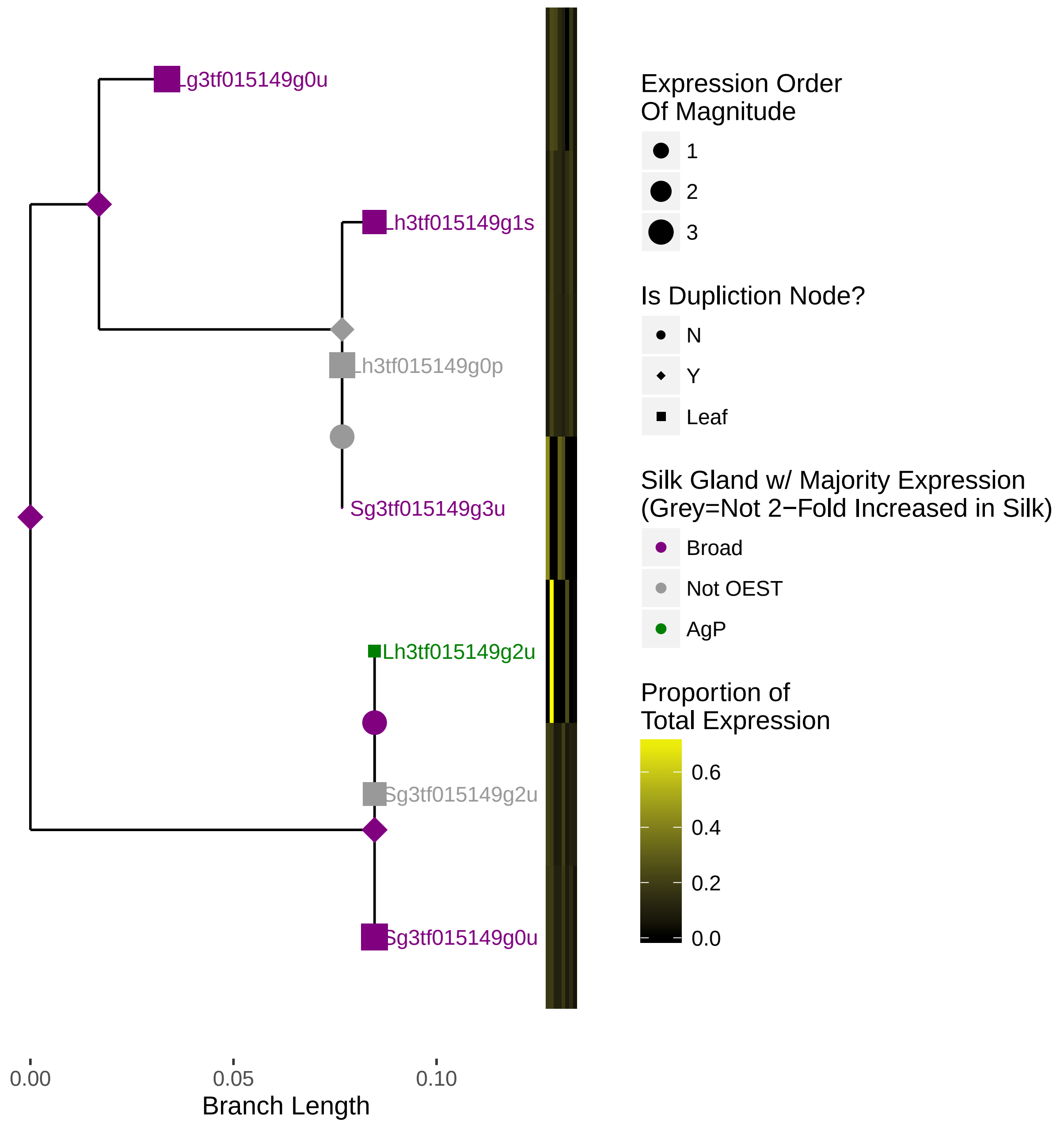

Lg3tf015149g0u
Lh3tf015149g1s
Lh3tf015149g0p
Sg3tf015149g3u
Lh3tf015149g2u
Sg3tf015149g2u
Sg3tf015149g0u
0.00
0.05
0.10
Branch Length
Expression Order
Of Magnitude
1
2
3
Is Dupliction Node?
N
Y
Leaf
Silk Gland w/ Majority Expression
(Grey=Not 2−Fold Increased in Silk)
Broad
Not OEST
AgP
Proportion of
Total Expression
0.6
0.4
0.2
0.0

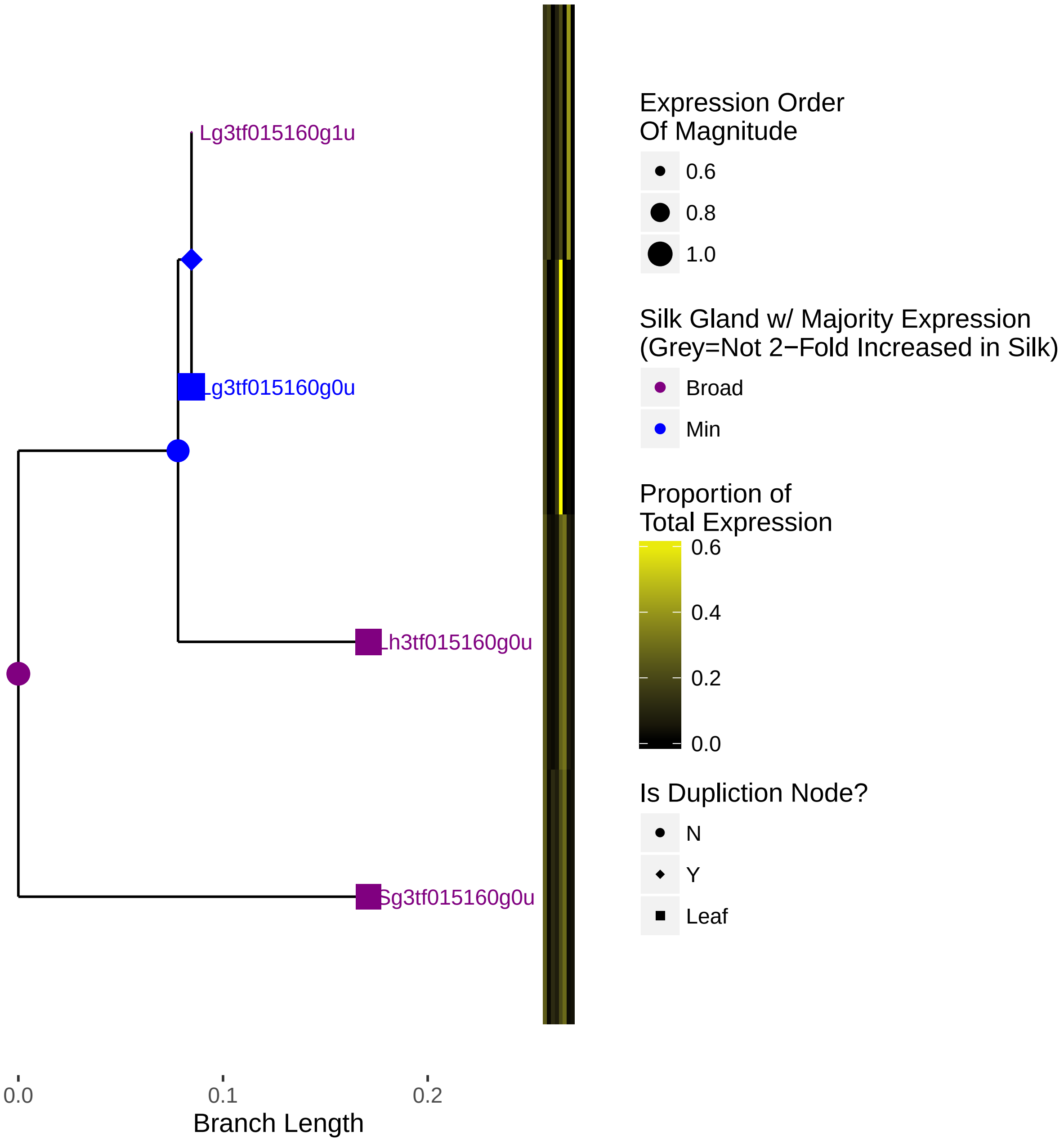

Lg3tf015160g1u
Lg3tf015160g0u
Lh3tf015160g0u
Sg3tf015160g0u
0.0
0.1
0.2
Branch Length
Expression Order
Of Magnitude
0.6
0.8
1.0
Silk Gland w/ Majority Expression
(Grey=Not 2−Fold Increased in Silk)
Broad
Min
Proportion of
Total Expression
0.6
0.4
0.2
0.0
Is Dupliction Node?
N
Y
Leaf

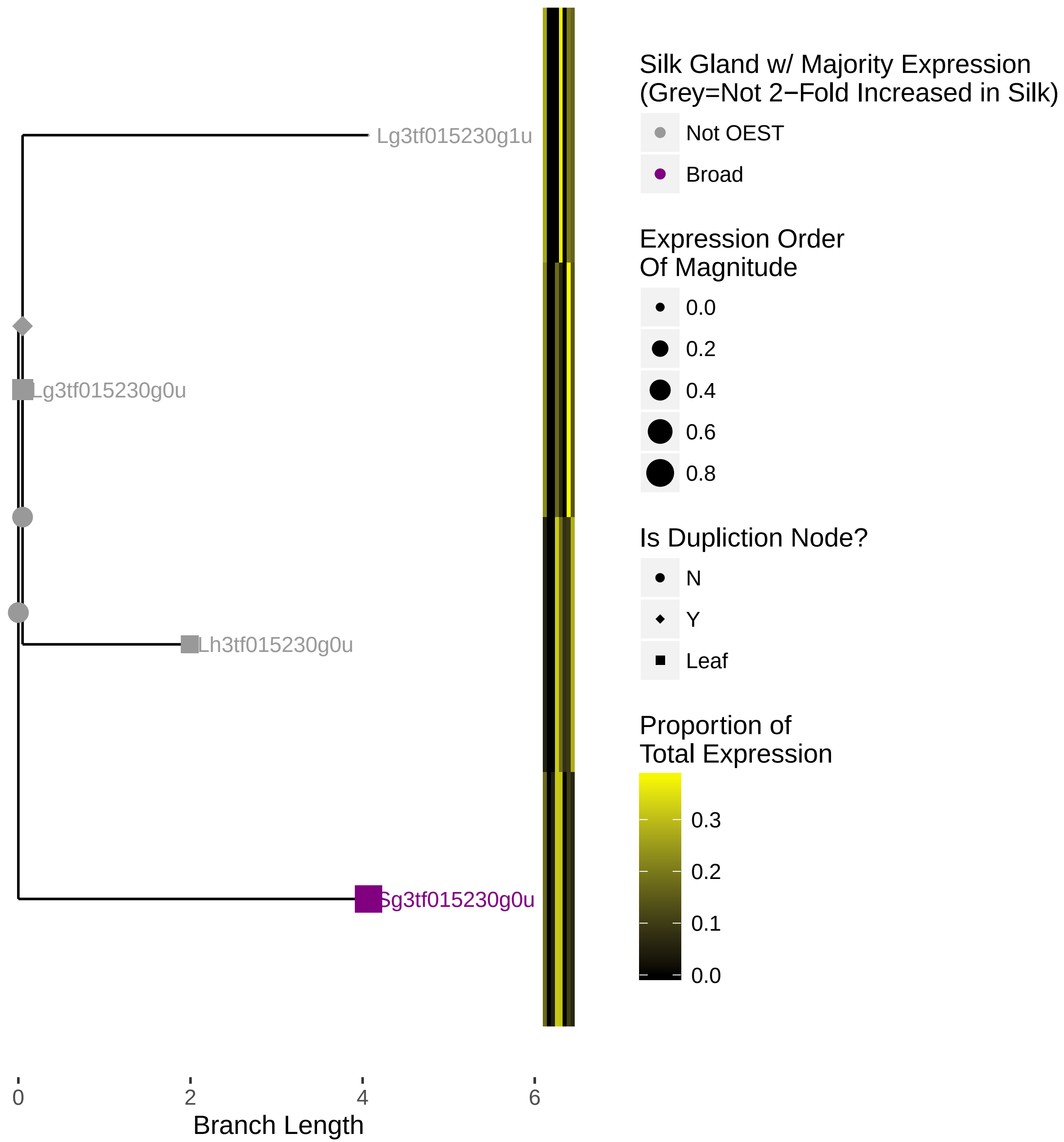
Lg3tf015230g1u
Lg3tf015230g0u
Lh3tf015230g0u
Sg3tf015230g0u
0
2
4
6
Branch Length
Silk Gland w/ Majority Expression
(Grey=Not 2−Fold Increased in Silk)
Not OEST
Broad
Expression Order
Of Magnitude
0.0
0.2
0.4
0.6
0.8
Is Dupliction Node?
N
Y
Leaf
Proportion of
Total Expression
0.3
0.2
0.1
0.0

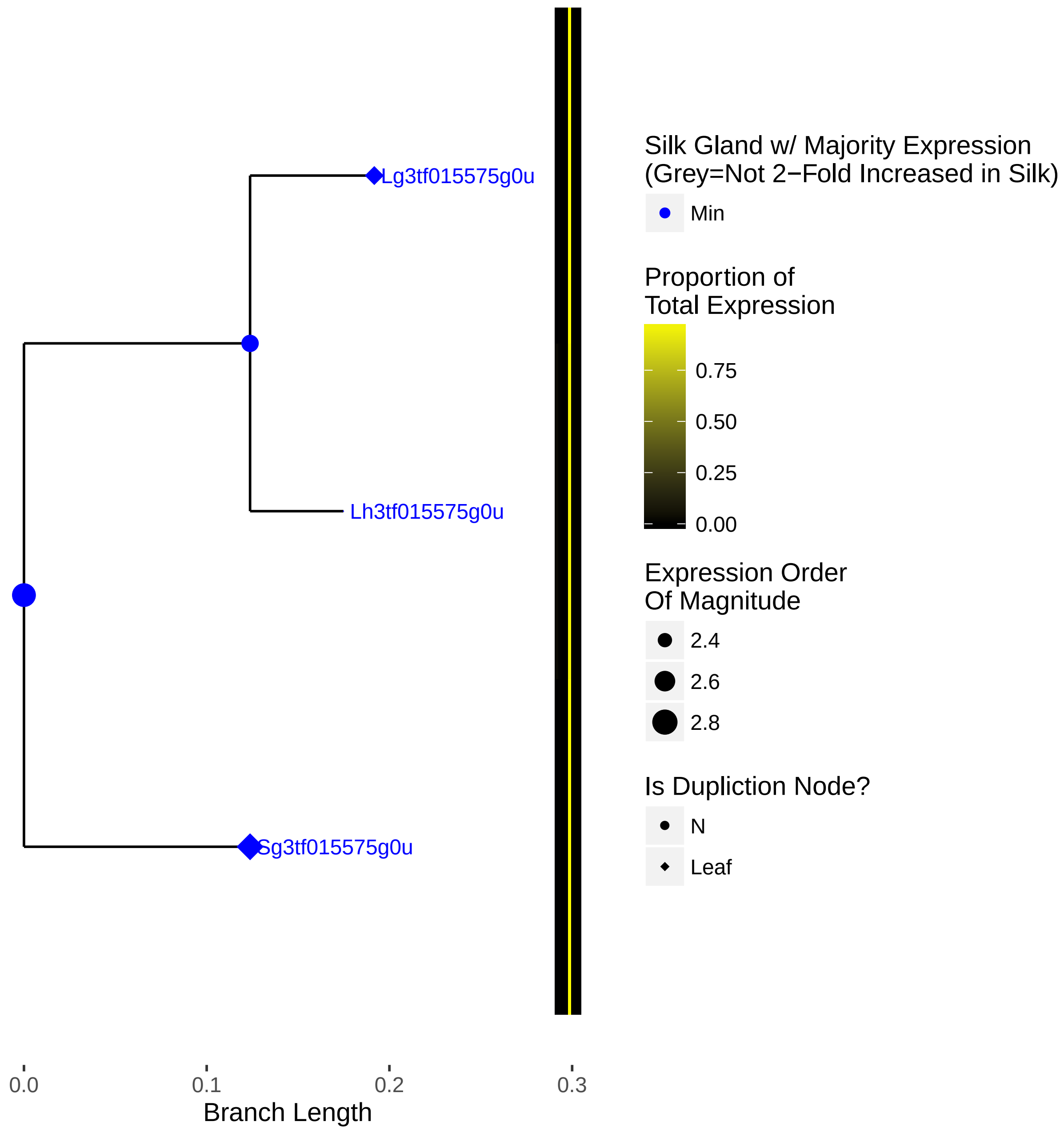
Lg3tf015575g0u
Lh3tf015575g0u
Sg3tf015575g0u
0.0
0.1
0.2
0.3
Branch Length
Silk Gland w/ Majority Expression
(Grey=Not 2−Fold Increased in Silk)
Min
Proportion of
Total Expression
0.75
0.50
0.25
0.00
Expression Order
Of Magnitude
2.4
2.6
2.8
Is Dupliction Node?
N
Leaf

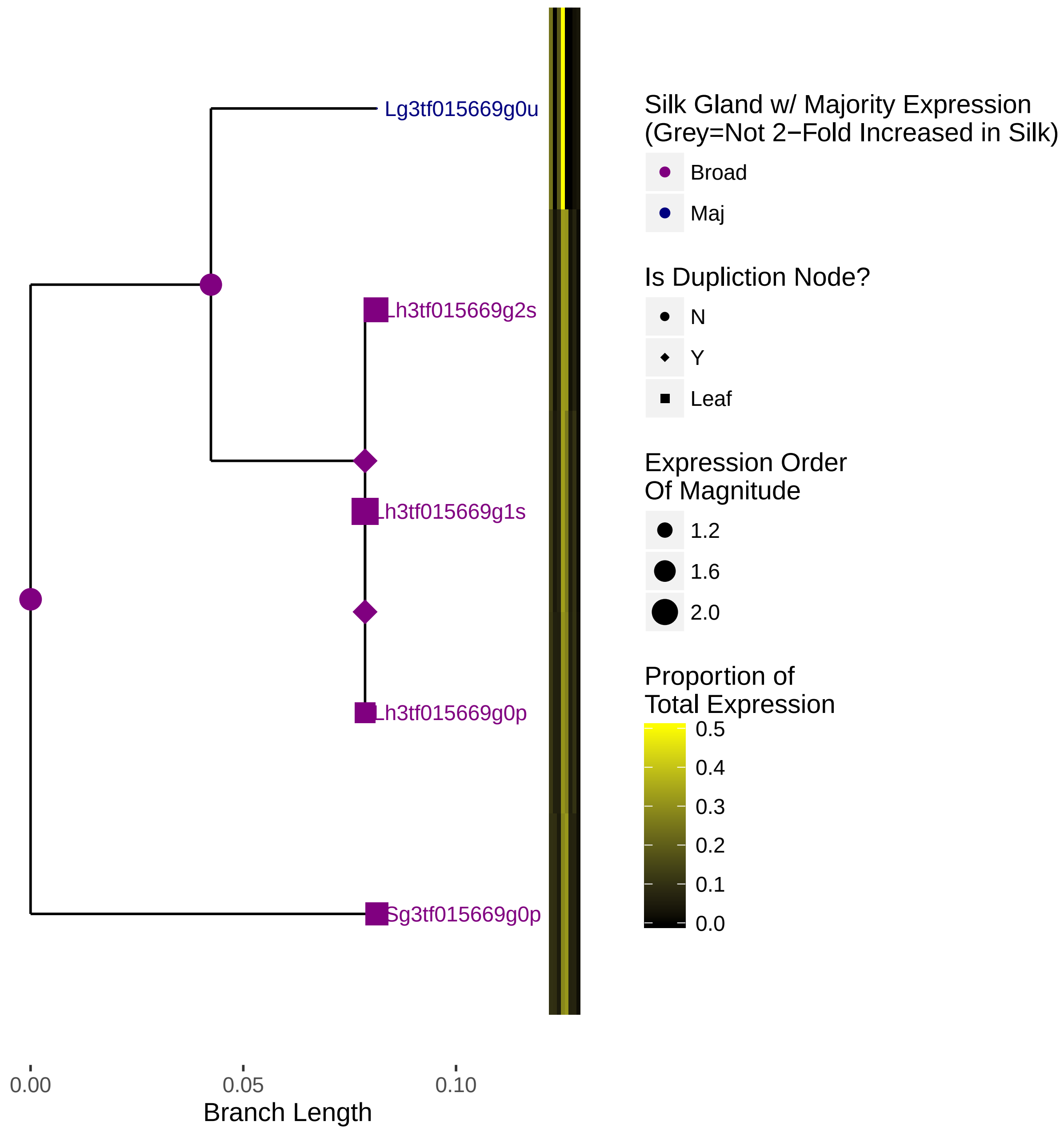

Lg3tf015669g0u
Lh3tf015669g2s
Lh3tf015669g1s
Lh3tf015669g0p
Sg3tf015669g0p
0.00
0.05
0.10
Branch Length
Silk Gland w/ Majority Expression
(Grey=Not 2−Fold Increased in Silk)
Broad
Maj
Is Dupliction Node?
N
Y
Leaf
Expression Order
Of Magnitude
1.2
1.6
2.0
Proportion of
Total Expression
0.5
0.4
0.3
0.2
0.1
0.0

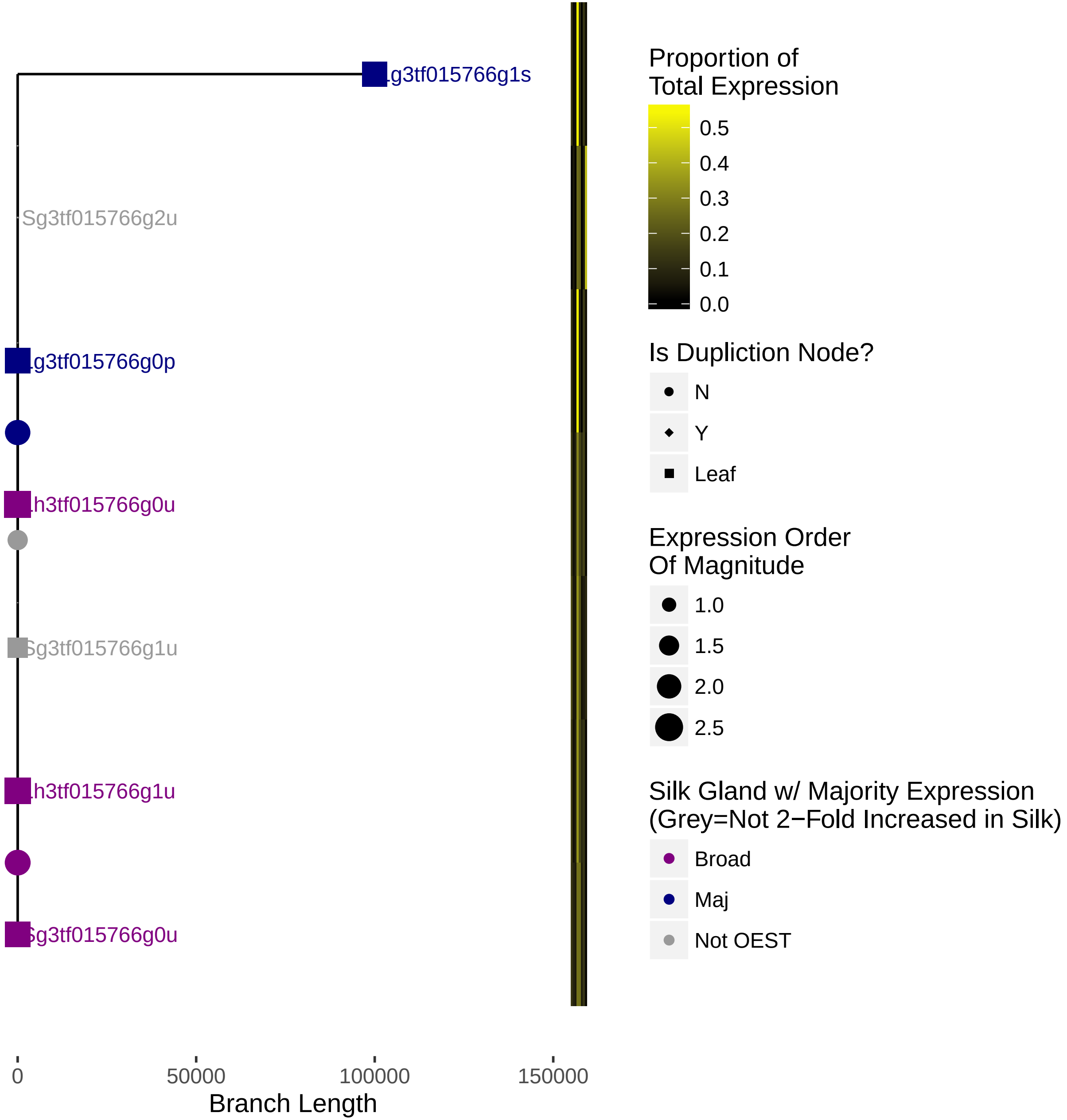

g3tf015766g1s
Sg3tf015766g2u
g3tf015766g0p
h3tf015766g0u
g3tf015766g1u
h3tf015766g1u
g3tf015766g0u
0
50000
100000
150000
Branch Length
Proportion of
Total Expression
0.5
0.4
0.3
0.2
0.1
0.0
Is Dupliction Node?
N
Y
Leaf
Expression Order
Of Magnitude
1.0
1.5
2.0
2.5
Silk Gland w/ Majority Expression
(Grey=Not 2−Fold Increased in Silk)
Broad
Maj
Not OEST

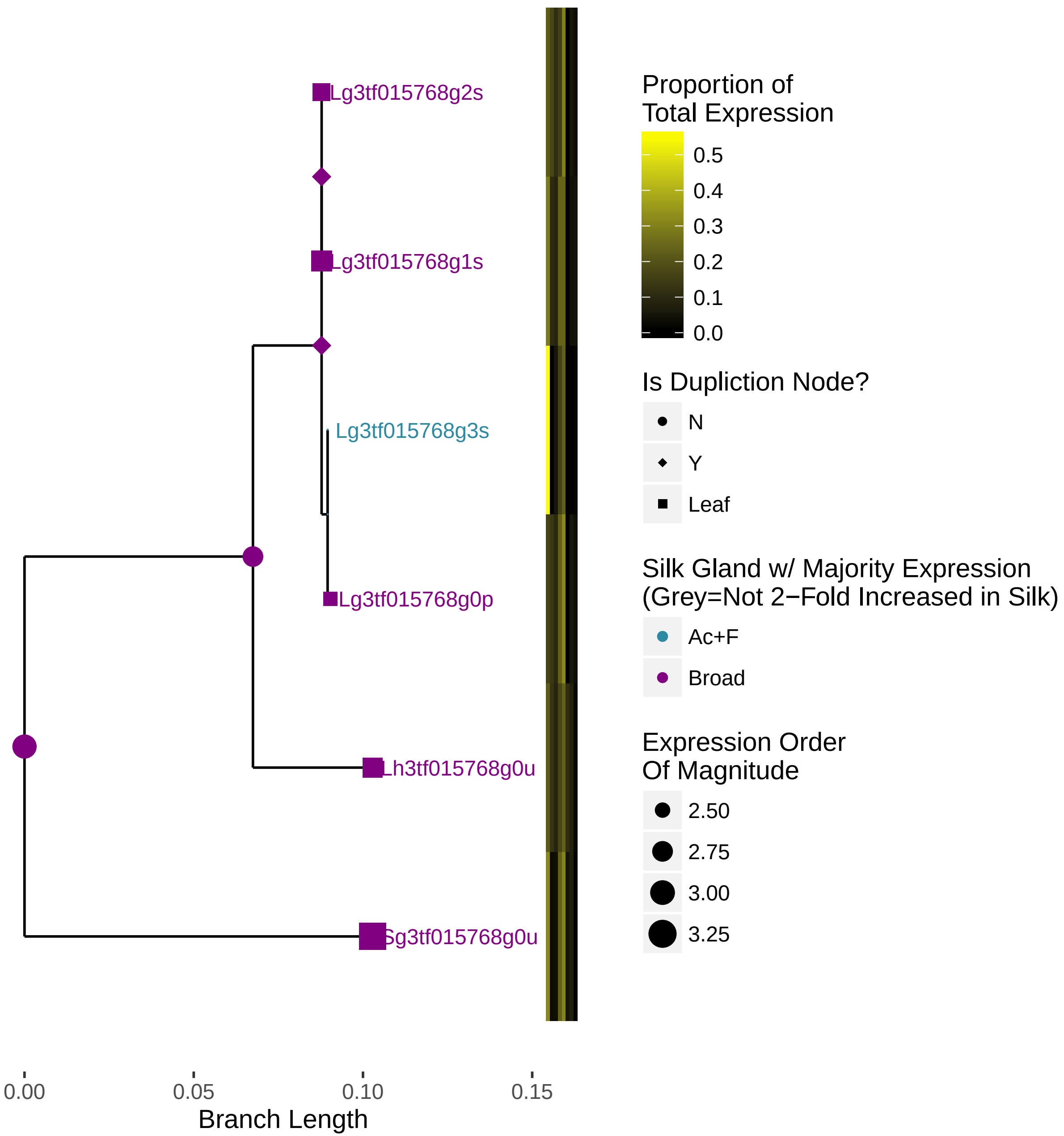

Lg3tf015768g2s
Lg3tf015768g1s
Lg3tf015768g3s
Lg3tf015768g0p
Lh3tf015768g0u
Sg3tf015768g0u
0.00
0.05
0.10
0.15
Branch Length
Proportion of
Total Expression
0.5
0.4
0.3
0.2
0.1
0.0
Is Dupliction Node?
N
Y
Leaf
Silk Gland w/ Majority Expression
(Grey=Not 2−Fold Increased in Silk)
Ac+F
Broad
Expression Order
Of Magnitude
2.50
2.75
3.00
3.25

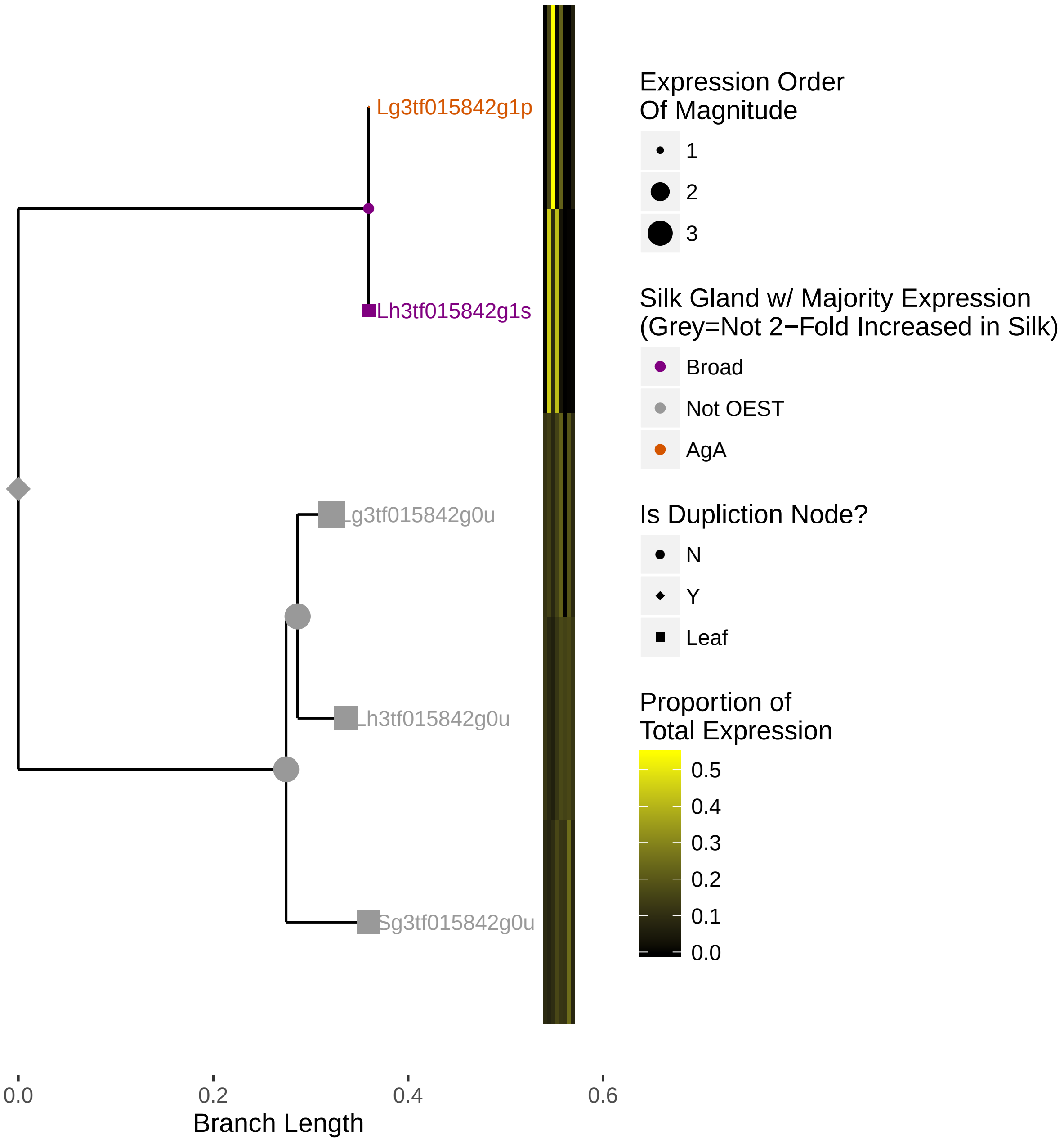
Lg3tf015842g1p
Lh3tf015842g1s
Lg3tf015842g0u
Lh3tf015842g0u
Sg3tf015842g0u
0.0
0.2
0.4
0.6
Branch Length
Expression Order
Of Magnitude
1
2
3
Silk Gland w/ Majority Expression
(Grey=Not 2−Fold Increased in Silk)
Broad
Not OEST
AgA
Is Dupliction Node?
N
Y
Leaf
Proportion of
Total Expression
0.5
0.4
0.3
0.2
0.1
0.0

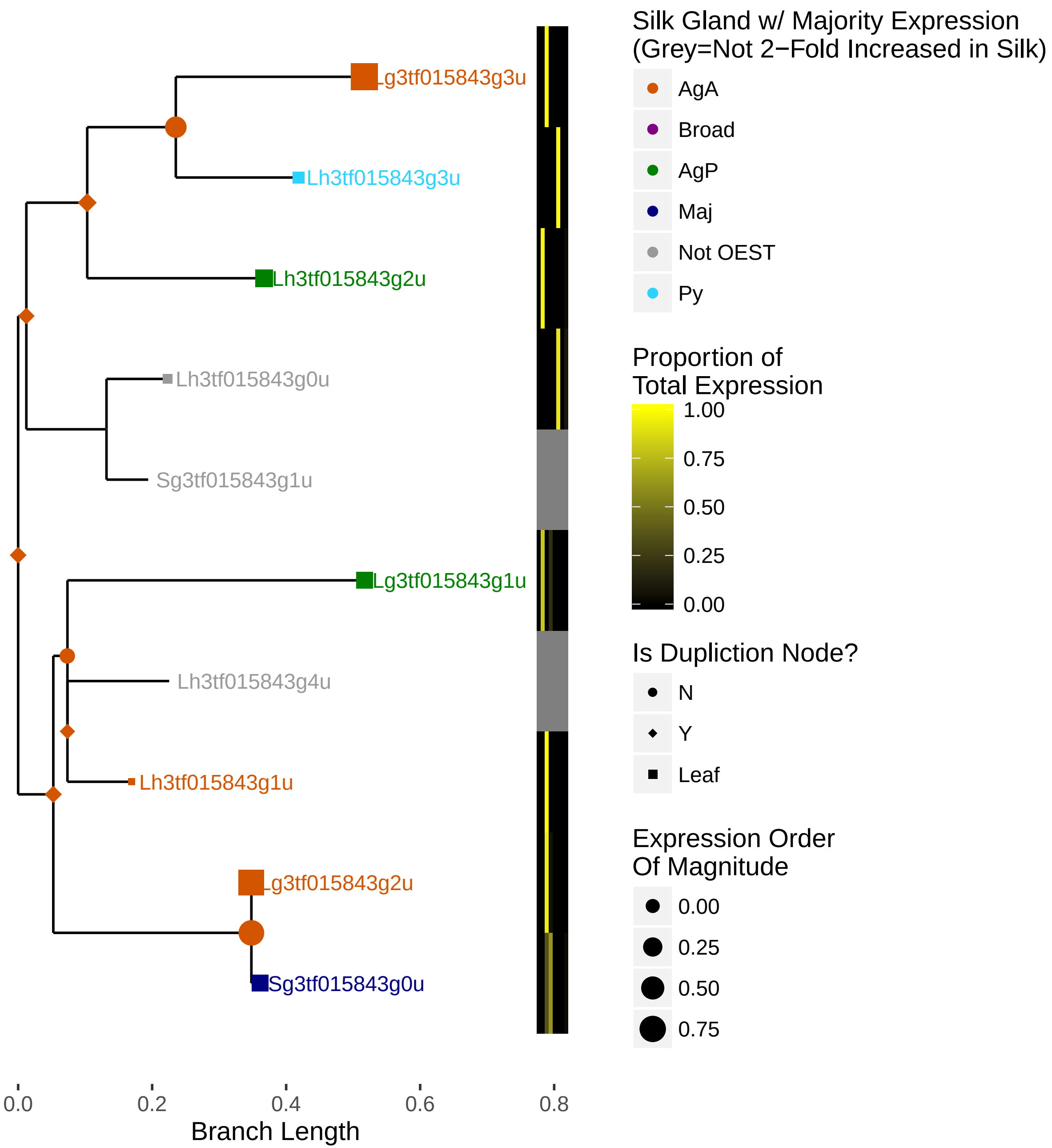
Lg3tf015843g3u
Lh3tf015843g3u
Lh3tf015843g2u
Lh3tf015843g0u
Sg3tf015843g1u
Lg3tf015843g1u
Lh3tf015843g4u
Lh3tf015843g1u
Lg3tf015843g2u
Sg3tf015843g0u
0.0
0.2
0.4
0.6
0.8
Branch Length
Silk Gland w/ Majority Expression
(Grey=Not 2−Fold Increased in Silk)
AgA
Broad
AgP
Maj
Not OEST
Py
Proportion of
Total Expression
1.00
0.75
0.50
0.25
0.00
Is Dupliction Node?
N
Y
Leaf
Expression Order
Of Magnitude
0.00
0.25
0.50
0.75

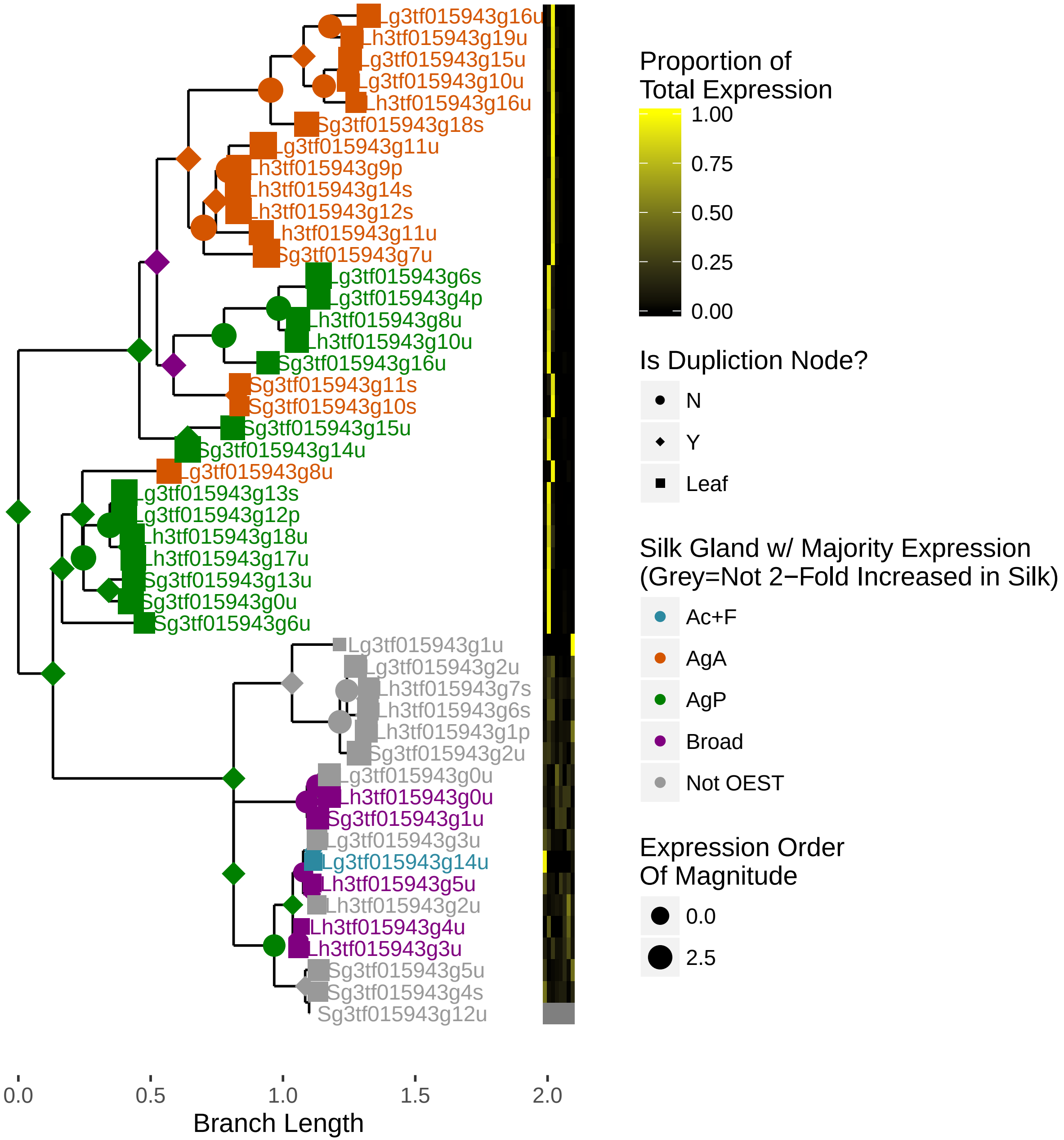

Lg3tf015943g16u
Lh3tf015943g19u
Lg3tf015943g15u
Lg3tf015943g10u
Lh3tf015943g16u
Sg3tf015943g18s
Lg3tf015943g11u
Lh3tf015943g9p
Lh3tf015943g14s
Lh3tf015943g12s
Lh3tf015943g11u
Sg3tf015943g7u
Lg3tf015943g6s
Lg3tf015943g4p
Lh3tf015943g8u
Lh3tf015943g10u
Sg3tf015943g16u
Sg3tf015943g11s
Sg3tf015943g10s
Sg3tf015943g15u
Sg3tf015943g14u
Lg3tf015943g8u
Lg3tf015943g13s
Lg3tf015943g12p
Lh3tf015943g18u
Lh3tf015943g17u
Sg3tf015943g13u
Sg3tf015943g0u
Sg3tf015943g6u
Lg3tf015943g1u
Lg3tf015943g2u
Lh3tf015943g7s
Lh3tf015943g6s
Lh3tf015943g1p
Sg3tf015943g2u
Lg3tf015943g0u
Lh3tf015943g0u
Sg3tf015943g1u
Lg3tf015943g3u
Lg3tf015943g14u
Lh3tf015943g5u
Lh3tf015943g2u
Lh3tf015943g4u
Lh3tf015943g3u
Sg3tf015943g5u
Sg3tf015943g4s
Sg3tf015943g12u
0.0
0.5
1.0
1.5
2.0
Branch Length
Proportion of Total Expression
1.00
0.75
0.50
0.25
0.00
Is Dupliction Node?
N
Y
Leaf
Silk Gland w/ Majority Expression (Grey=Not 2−Fold Increased in Silk)
Ac+F
AgA
AgP
Broad
Not OEST
Expression Order Of Magnitude
0.0
2.5

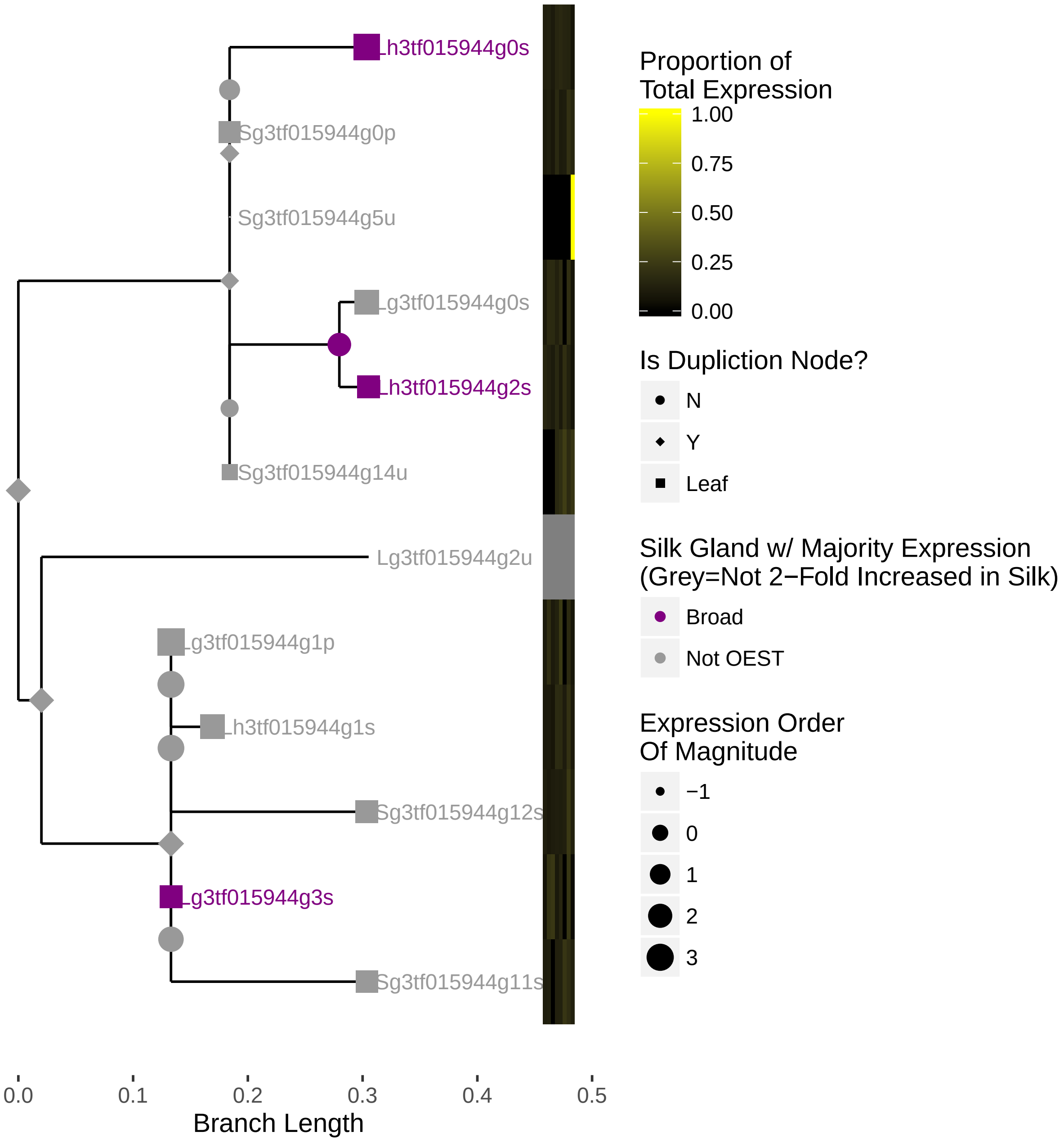

Lh3tf015944g0s
Sg3tf015944g0p
Sg3tf015944g5u
Lg3tf015944g0s
Lh3tf015944g2s
Sg3tf015944g14u
Lg3tf015944g2u
Lg3tf015944g1p
Lh3tf015944g1s
Sg3tf015944g12s
Lg3tf015944g3s
Sg3tf015944g11s
0.0
0.1
0.2
0.3
0.4
0.5
Branch Length
Proportion of Total Expression
1.00
0.75
0.50
0.25
0.00
Is Dupliction Node?
N
Y
Leaf
Silk Gland w/ Majority Expression (Grey=Not 2−Fold Increased in Silk)
Broad
Not OEST
Expression Order Of Magnitude
−1
0
1
2
3

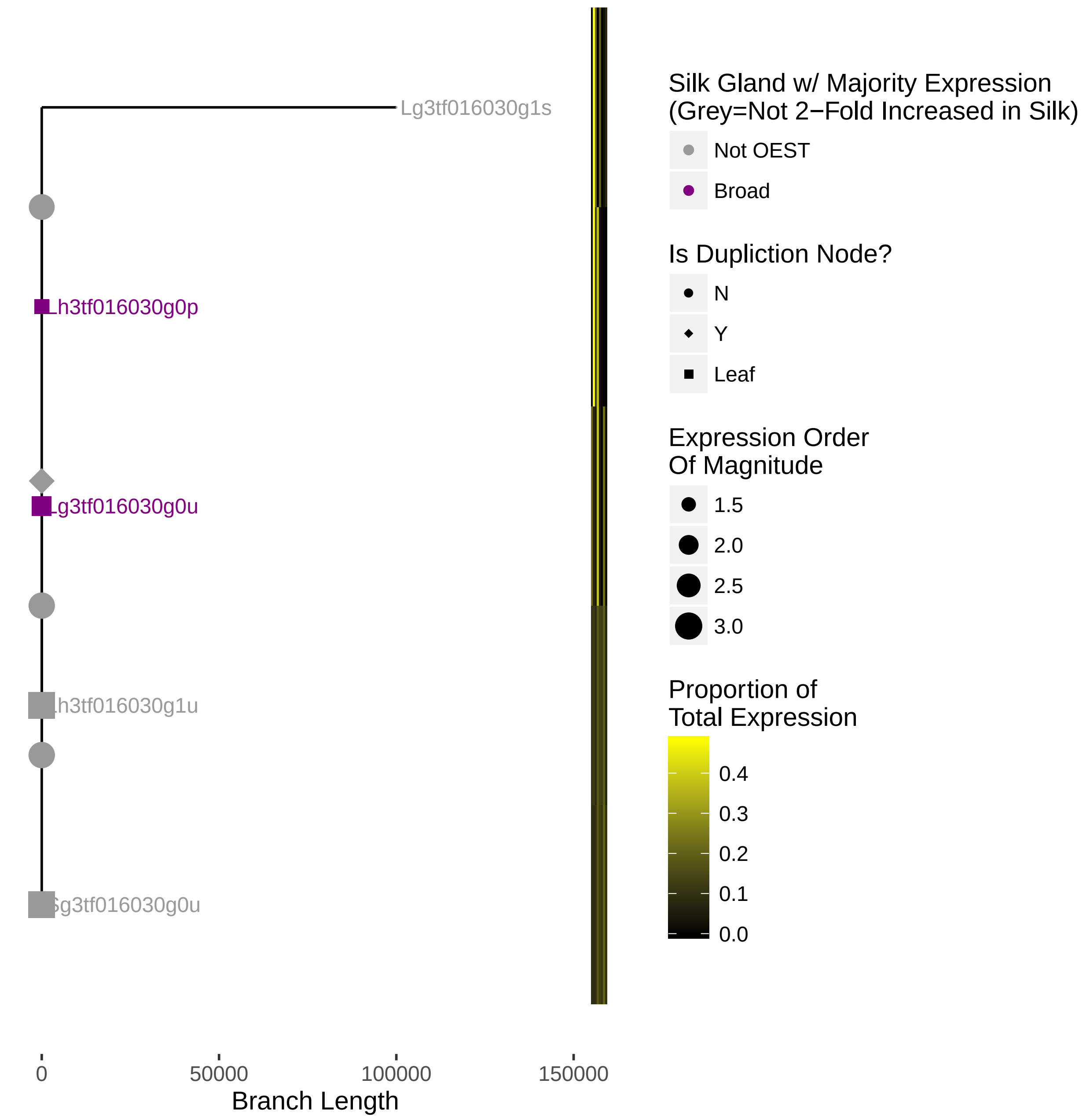

Lg3tf016030g1s
Lh3tf016030g0p
Lg3tf016030g0u
Lh3tf016030g1u
Sg3tf016030g0u
0
50000
100000
150000
Branch Length
Silk Gland w/ Majority Expression
(Grey=Not 2−Fold Increased in Silk)
Not OEST
Broad
Is Dupliction Node?
N
Y
Leaf
Expression Order
Of Magnitude
1.5
2.0
2.5
3.0
Proportion of
Total Expression
0.4
0.3
0.2
0.1
0.0

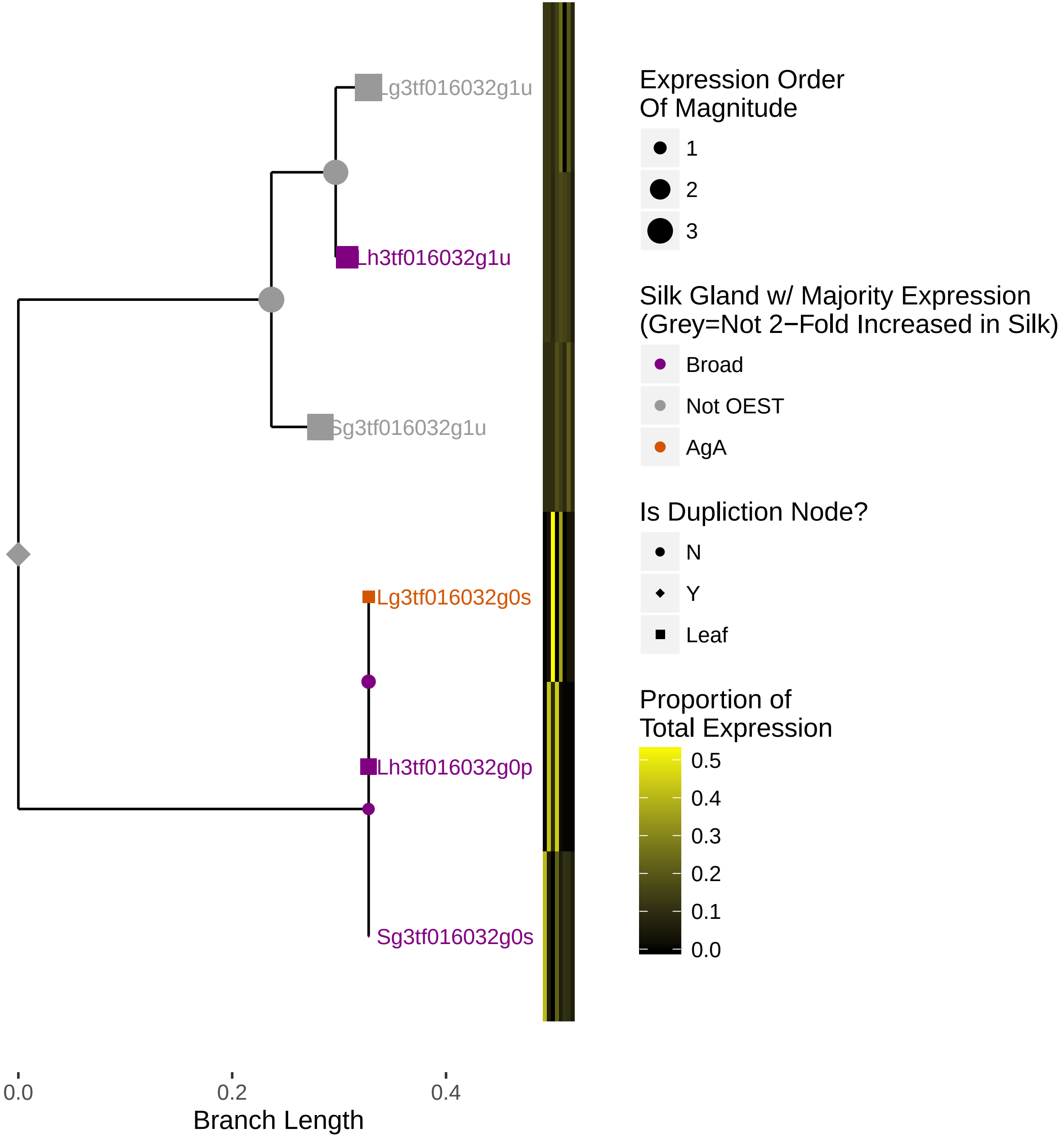

Lg3tf016032g1u
Lh3tf016032g1u
Sg3tf016032g1u
Lg3tf016032g0s
Lh3tf016032g0p
Sg3tf016032g0s
0.0
0.2
0.4
Branch Length
Expression Order Of Magnitude
1
2
3
Silk Gland w/ Majority Expression (Grey=Not 2−Fold Increased in Silk)
Broad
Not OEST
AgA
Is Dupliction Node?
N
Y
Leaf
Proportion of Total Expression
0.5
0.4
0.3
0.2
0.1
0.0

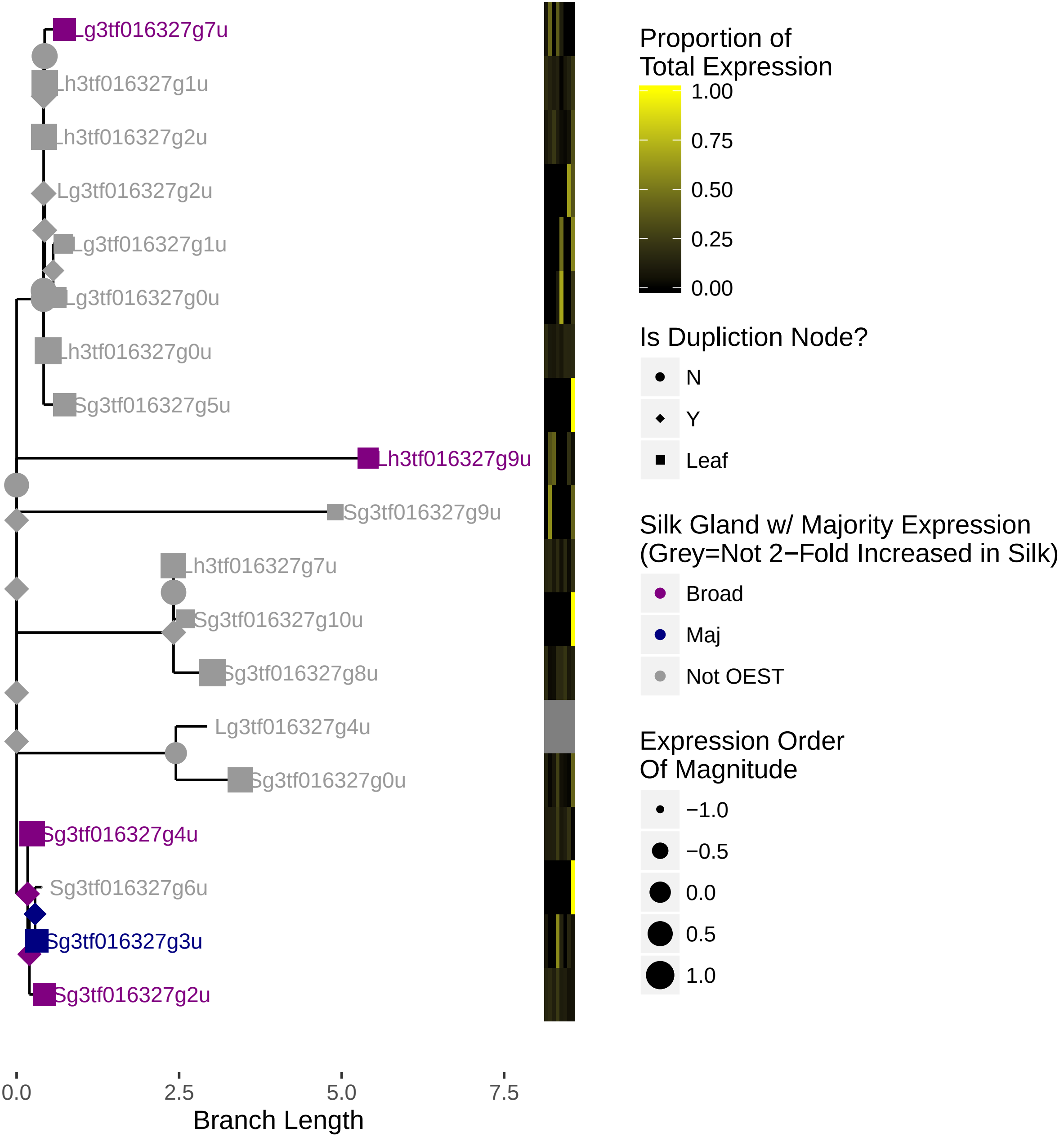

Lg3tf016327g7u
Lh3tf016327g1u
Lh3tf016327g2u
Lg3tf016327g2u
Lg3tf016327g1u
Lg3tf016327g0u
Lh3tf016327g0u
Sg3tf016327g5u
Lh3tf016327g9u
Sg3tf016327g9u
Lh3tf016327g7u
Sg3tf016327g10u
Sg3tf016327g8u
Lg3tf016327g4u
Sg3tf016327g0u
Sg3tf016327g4u
Sg3tf016327g6u
Sg3tf016327g3u
Sg3tf016327g2u
0.0
2.5
5.0
7.5
Branch Length
Proportion of
Total Expression
1.00
0.75
0.50
0.25
0.00
Is Dupliction Node?
N
Y
Leaf
Silk Gland w/ Majority Expression
(Grey=Not 2−Fold Increased in Silk)
Broad
Maj
Not OEST
Expression Order
Of Magnitude
−1.0
−0.5
0.0
0.5
1.0

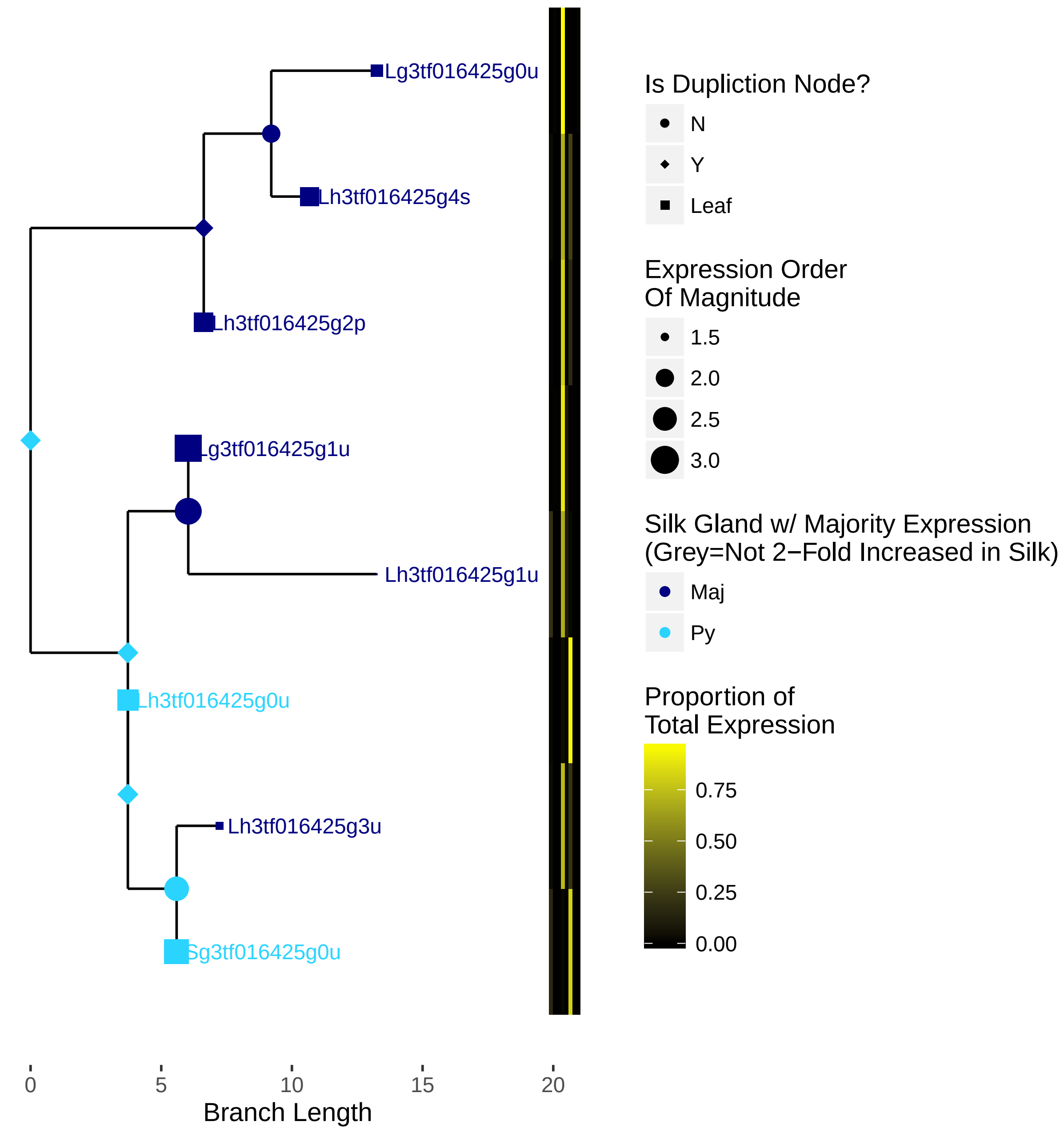

Lg3tf016425g0u
Lh3tf016425g4s
Lh3tf016425g2p
g3tf016425g1u
Lh3tf016425g1u
Lh3tf016425g0u
Lh3tf016425g3u
Sg3tf016425g0u
0
5
10
15
20
Branch Length
Is Dupliction Node?
N
Y
Leaf
Expression Order
Of Magnitude
1.5
2.0
2.5
3.0
Silk Gland w/ Majority Expression
(Grey=Not 2−Fold Increased in Silk)
Maj
Py
Proportion of
Total Expression
0.75
0.50
0.25
0.00

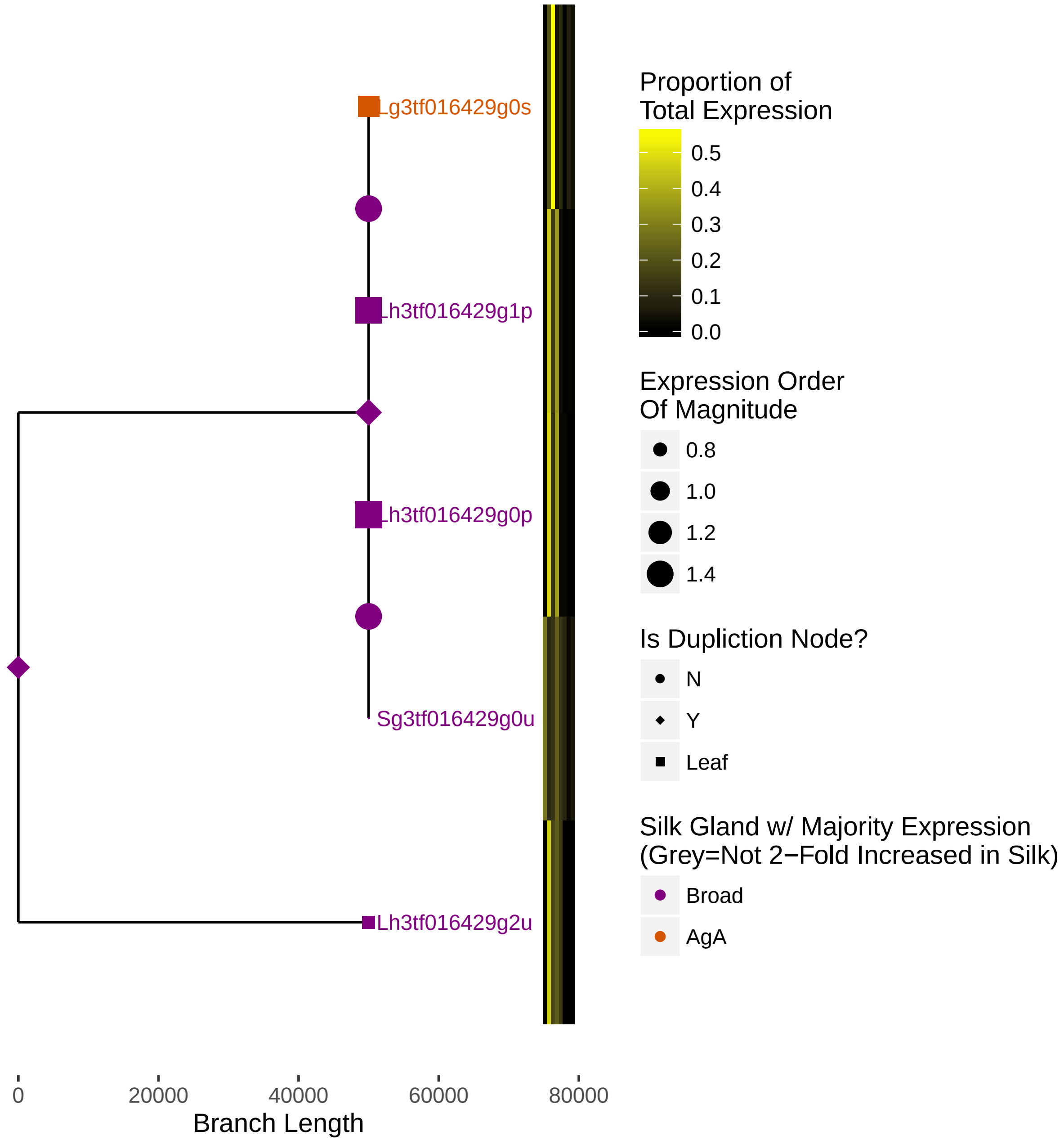

Lg3tf016429g0s
Lh3tf016429g1p
Lh3tf016429g0p
Sg3tf016429g0u
Lh3tf016429g2u
0
20000
40000
60000
80000
Branch Length
Proportion of
Total Expression
0.5
0.4
0.3
0.2
0.1
0.0
Expression Order
Of Magnitude
0.8
1.0
1.2
1.4
Is Dupliction Node?
N
Y
Leaf
Silk Gland w/ Majority Expression
(Grey=Not 2−Fold Increased in Silk)
Broad
AgA

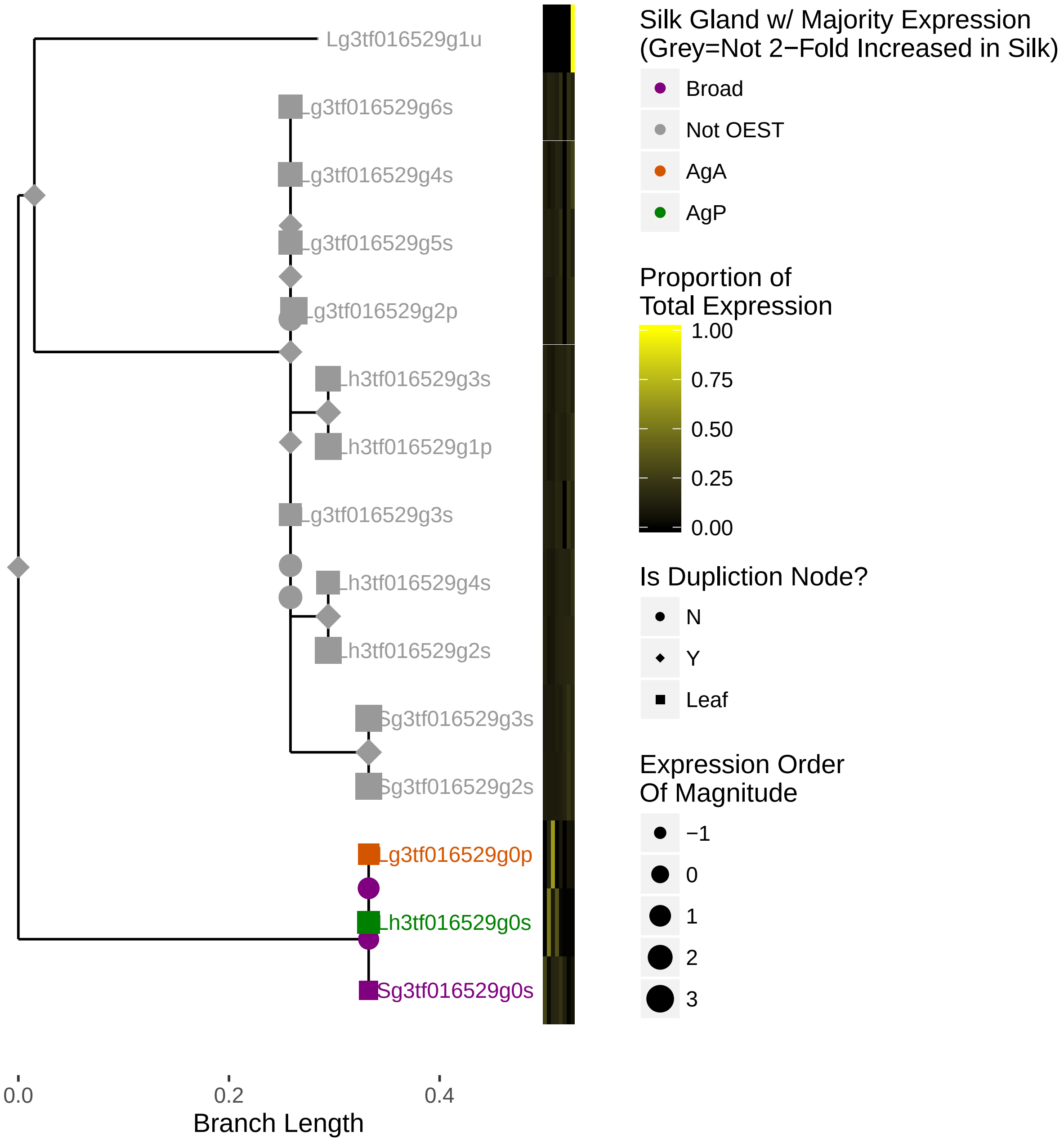

Lg3tf016529g1u
Lg3tf016529g6s
Lg3tf016529g4s
Lg3tf016529g5s
Lg3tf016529g2p
Lh3tf016529g3s
Lh3tf016529g1p
Lg3tf016529g3s
Lh3tf016529g4s
Lh3tf016529g2s
Sg3tf016529g3s
Sg3tf016529g2s
Lg3tf016529g0p
Lh3tf016529g0s
Sg3tf016529g0s
0.0
0.2
0.4
Branch Length
Silk Gland w/ Majority Expression
(Grey=Not 2−Fold Increased in Silk)
Broad
Not OEST
AgA
AgP
Proportion of
Total Expression
1.00
0.75
0.50
0.25
0.00
Is Dupliction Node?
N
Y
Leaf
Expression Order
Of Magnitude
−1
0
1
2
3

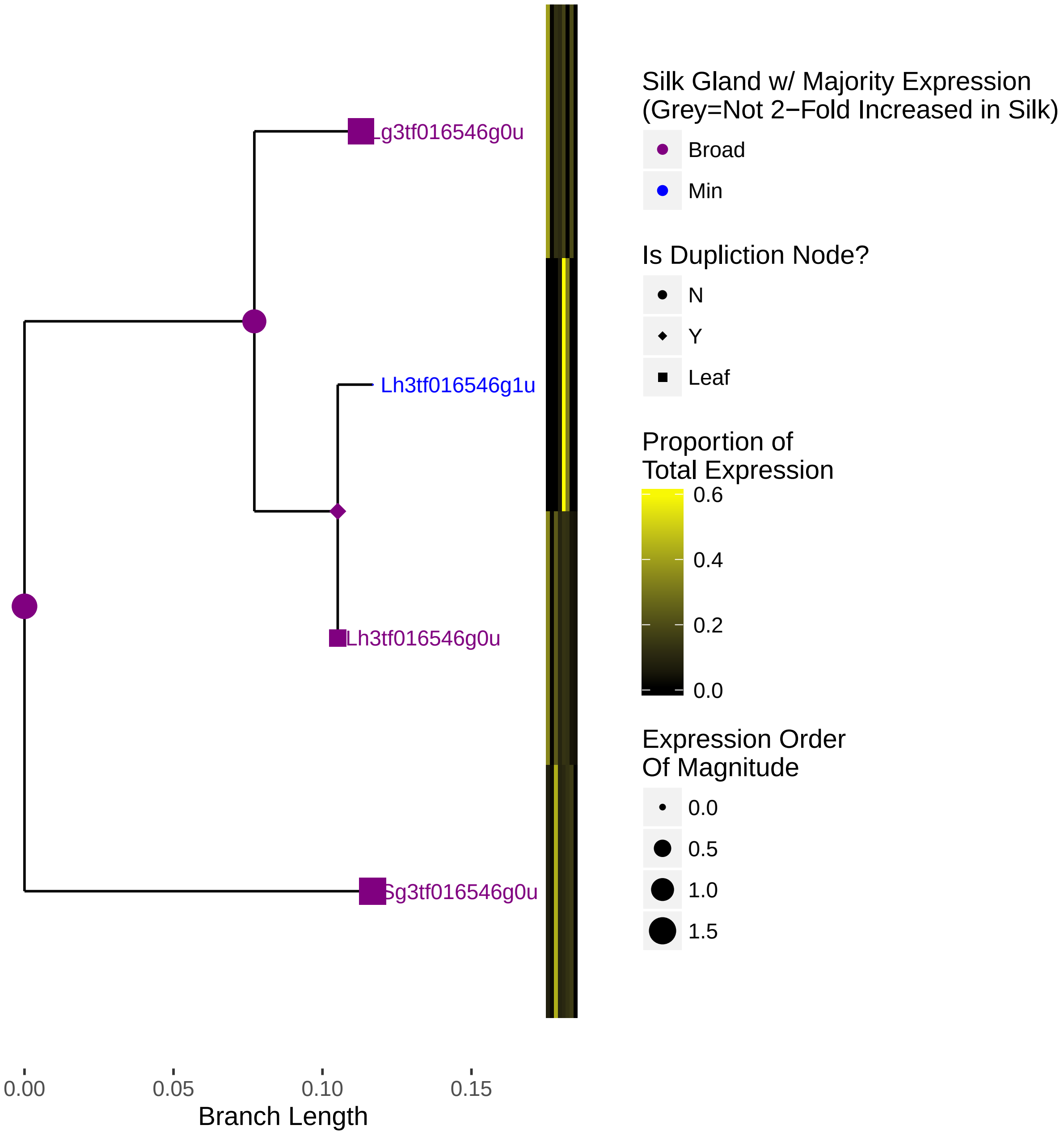

Lg3tf016546g0u
Lh3tf016546g1u
Lh3tf016546g0u
Sg3tf016546g0u
Silk Gland w/ Majority Expression
(Grey=Not 2−Fold Increased in Silk)
Broad
Min
Is Dupliction Node?
N
Y
Leaf
Proportion of
Total Expression
0.6
0.4
0.2
0.0
Expression Order
Of Magnitude
0.0
0.5
1.0
1.5
0.00
0.05
0.10
0.15
Branch Length

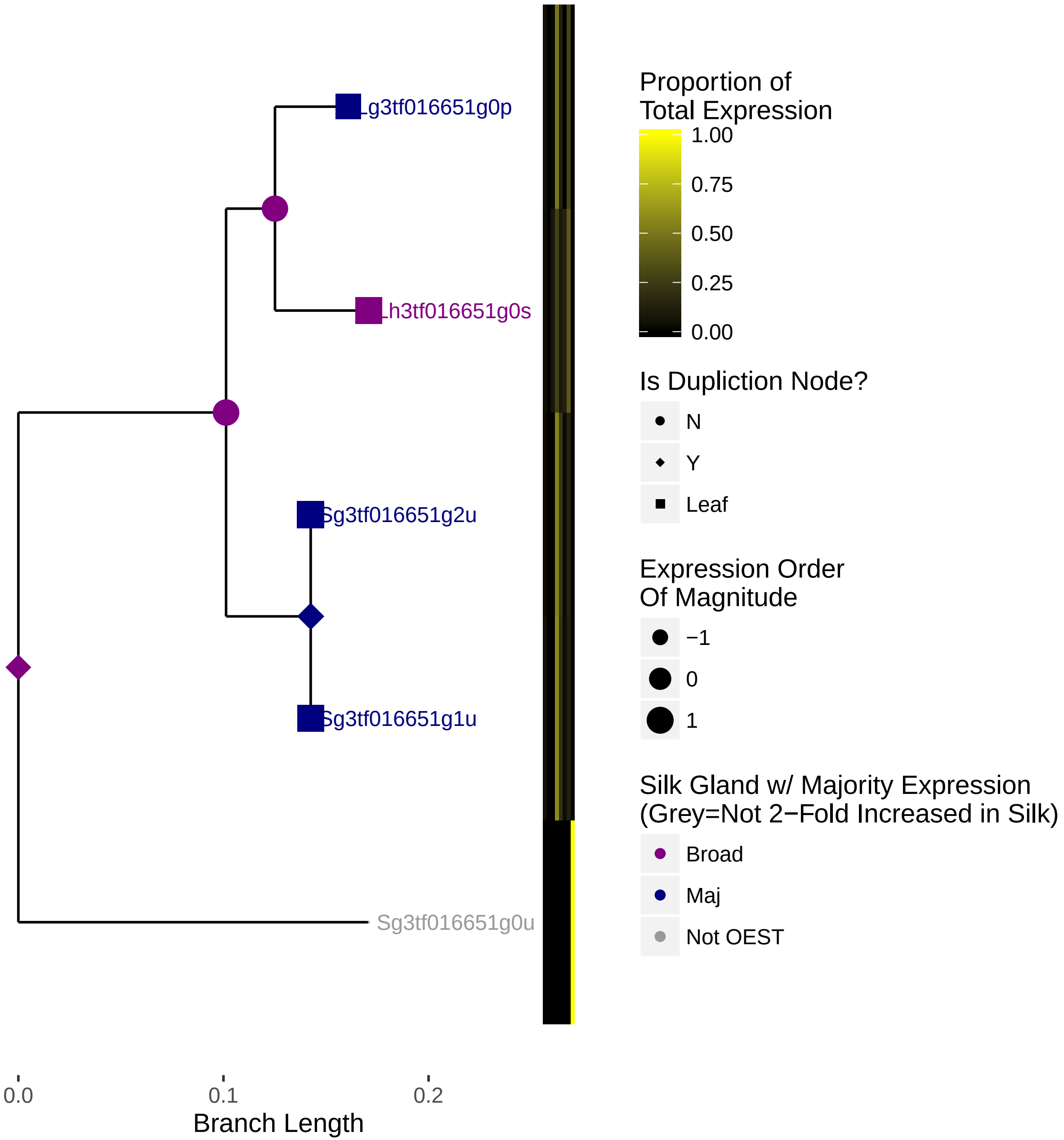

Lg3tf016651g0p
Lh3tf016651g0s
Sg3tf016651g2u
Sg3tf016651g1u
Sg3tf016651g0u
0.0
0.1
0.2
Branch Length
Proportion of Total Expression
1.00
0.75
0.50
0.25
0.00
Is Dupliction Node?
N
Y
Leaf
Expression Order Of Magnitude
−1
0
1
Silk Gland w/ Majority Expression (Grey=Not 2−Fold Increased in Silk)
Broad
Maj
Not OEST

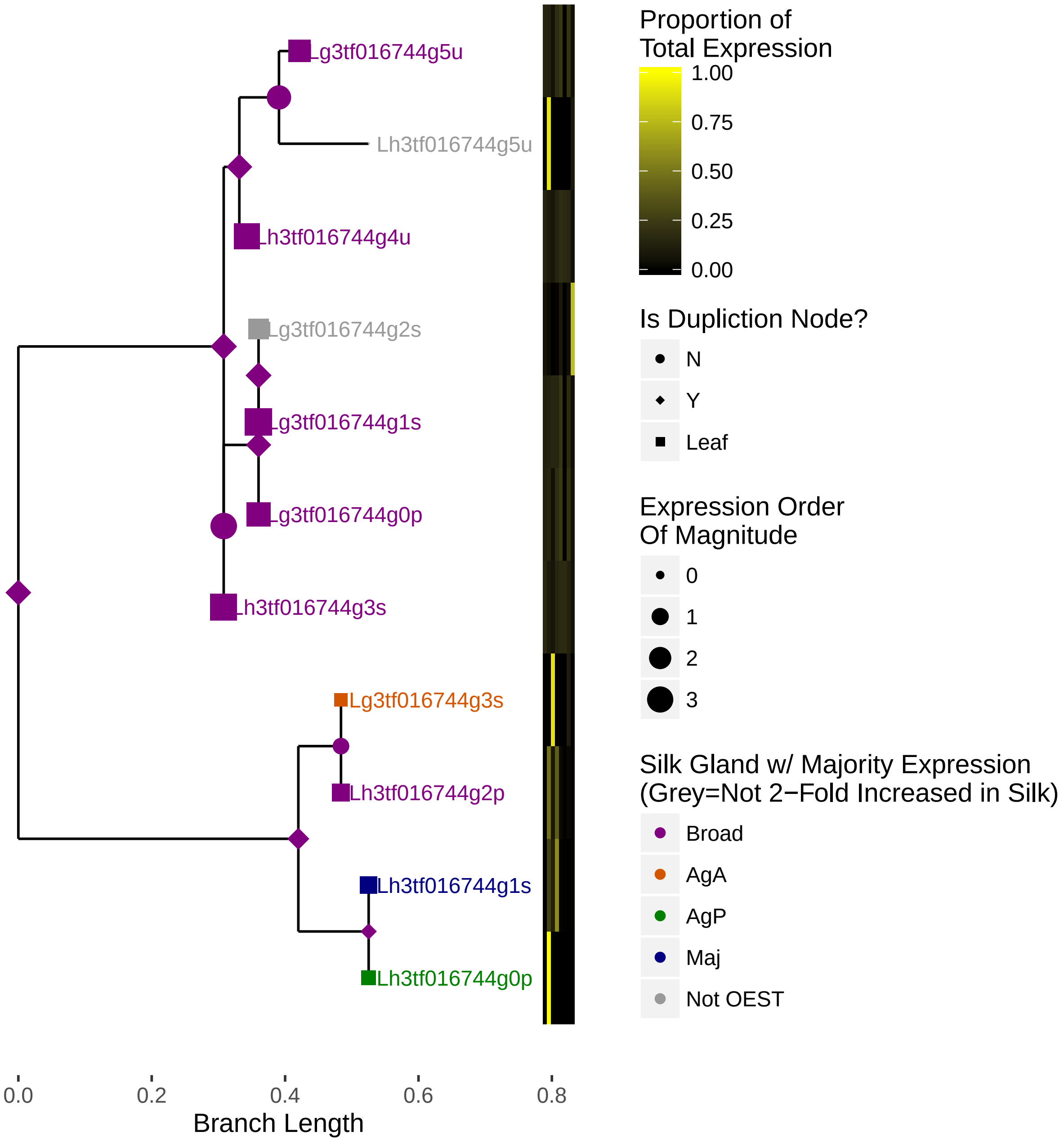
Lg3tf016744g5u
Lh3tf016744g5u
Lh3tf016744g4u
Lg3tf016744g2s
Lg3tf016744g1s
Lg3tf016744g0p
Lh3tf016744g3s
Lg3tf016744g3s
Lh3tf016744g2p
Lh3tf016744g1s
Lh3tf016744g0p
0.0
0.2
0.4
0.6
0.8
Branch Length
Proportion of
Total Expression
1.00
0.75
0.50
0.25
0.00
Is Dupliction Node?
N
Y
Leaf
Expression Order
Of Magnitude
0
1
2
3
Silk Gland w/ Majority Expression
(Grey=Not 2−Fold Increased in Silk)
Broad
AgA
AgP
Maj
Not OEST

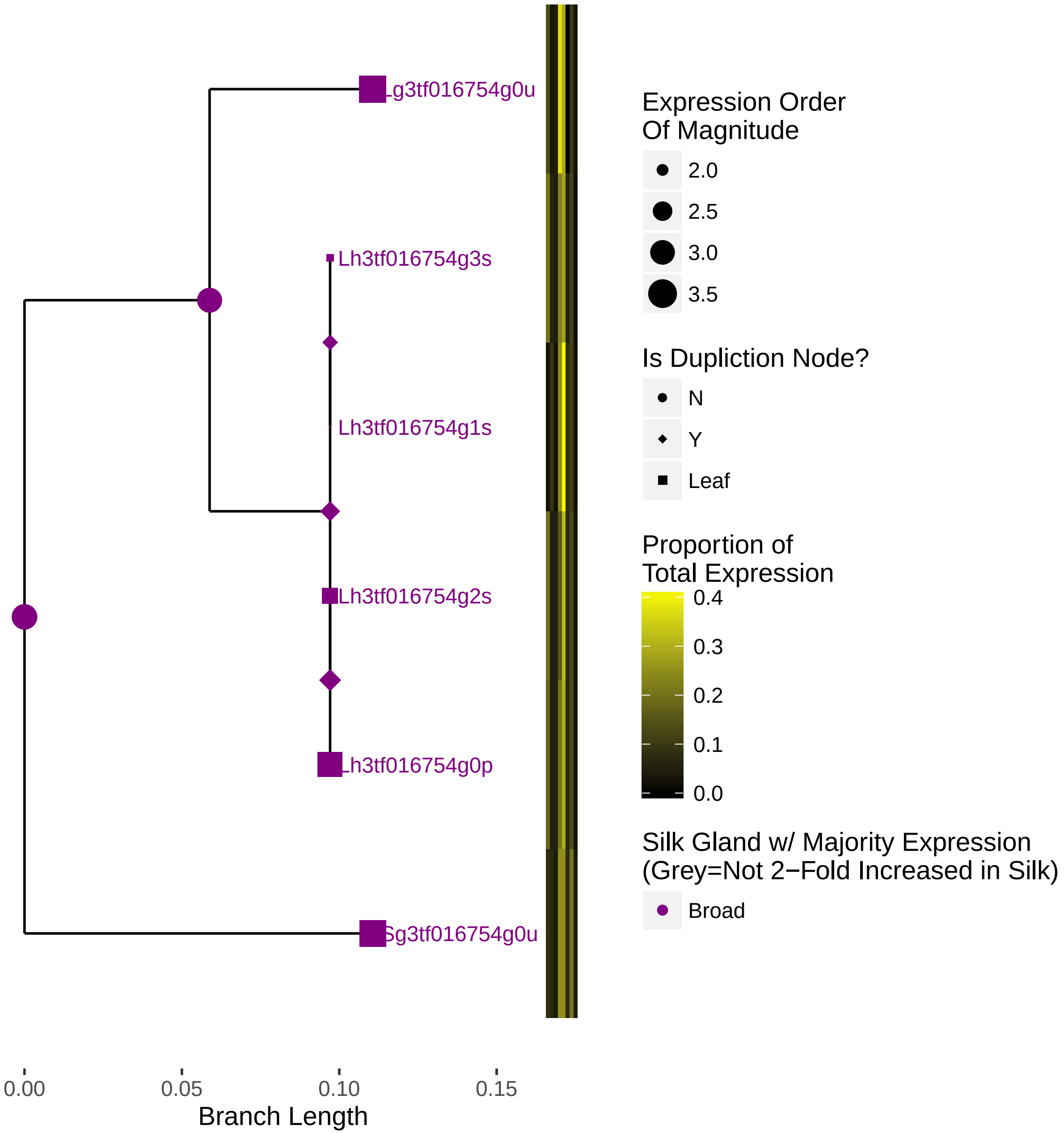

Lg3tf016754g0u
Lh3tf016754g3s
Lh3tf016754g1s
Lh3tf016754g2s
Lh3tf016754g0p
Sg3tf016754g0u
0.00
0.05
0.10
0.15
Branch Length
Expression Order
Of Magnitude
2.0
2.5
3.0
3.5
Is Dupliction Node?
N
Y
Leaf
Proportion of
Total Expression
0.4
0.3
0.2
0.1
0.0
Silk Gland w/ Majority Expression
(Grey=Not 2−Fold Increased in Silk)
Broad

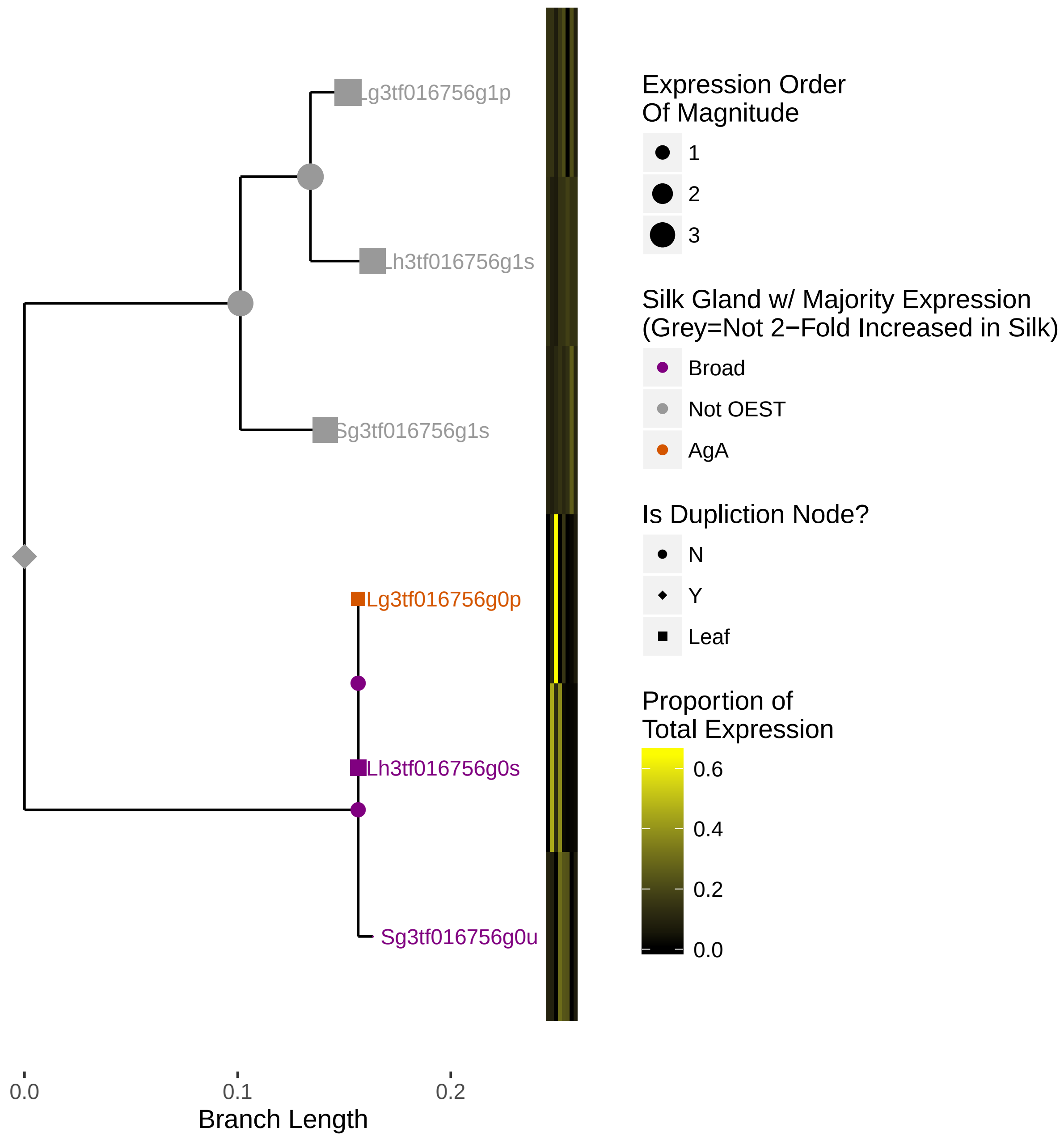

Lg3tf016756g1p
Lh3tf016756g1s
Sg3tf016756g1s
Lg3tf016756g0p
Lh3tf016756g0s
Sg3tf016756g0u
0.0
0.1
0.2
Branch Length
Expression Order
Of Magnitude
1
2
3
Silk Gland w/ Majority Expression
(Grey=Not 2−Fold Increased in Silk)
Broad
Not OEST
AgA
Is Dupliction Node?
N
Y
Leaf
Proportion of
Total Expression
0.6
0.4
0.2
0.0

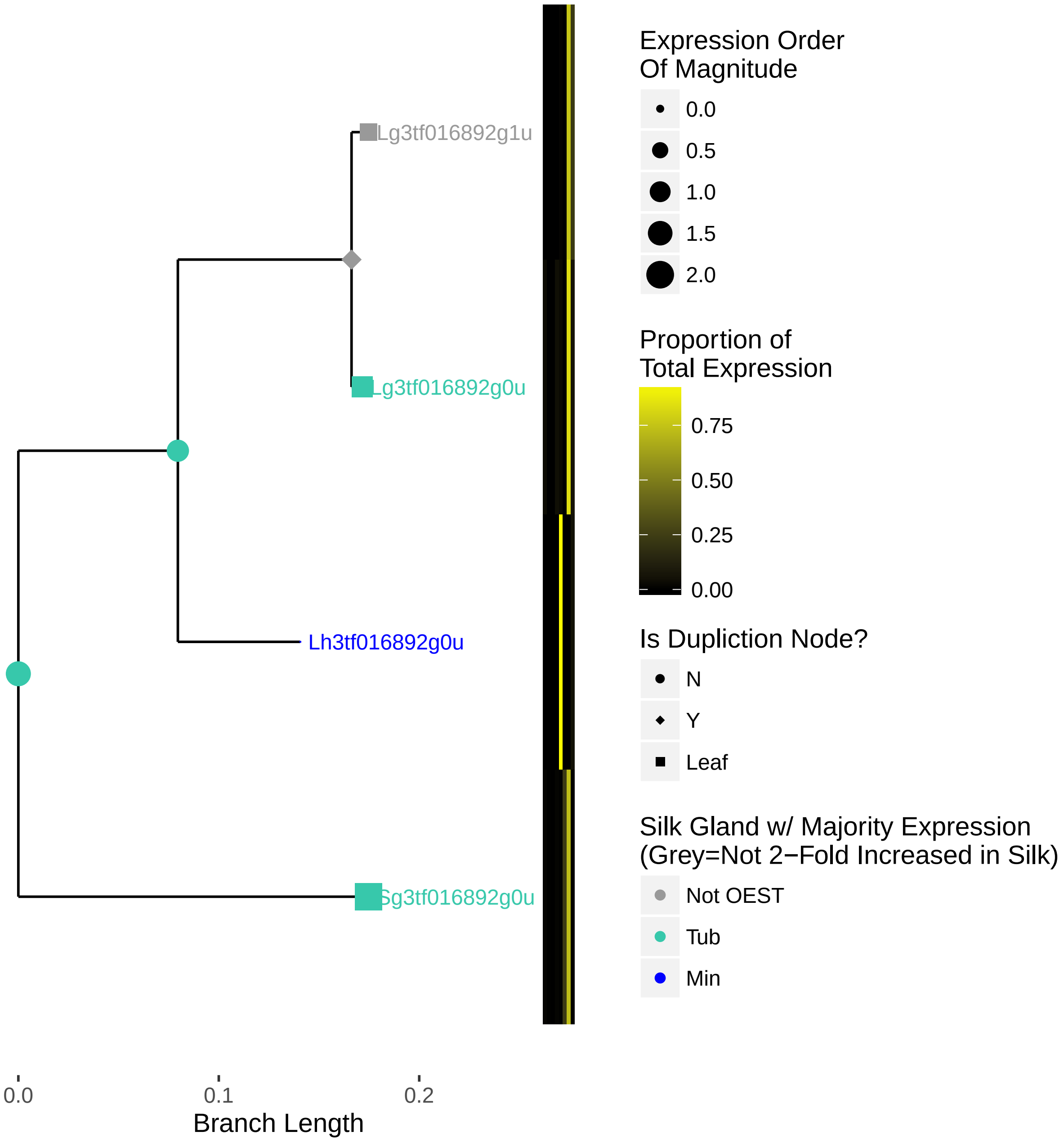

Lg3tf016892g1u
Lg3tf016892g0u
Lh3tf016892g0u
Sg3tf016892g0u
0.0
0.1
0.2
Branch Length
Expression Order
Of Magnitude
0.0
0.5
1.0
1.5
2.0
Proportion of
Total Expression
0.75
0.50
0.25
0.00
Is Dupliction Node?
N
Y
Leaf
Silk Gland w/ Majority Expression
(Grey=Not 2−Fold Increased in Silk)
Not OEST
Tub
Min

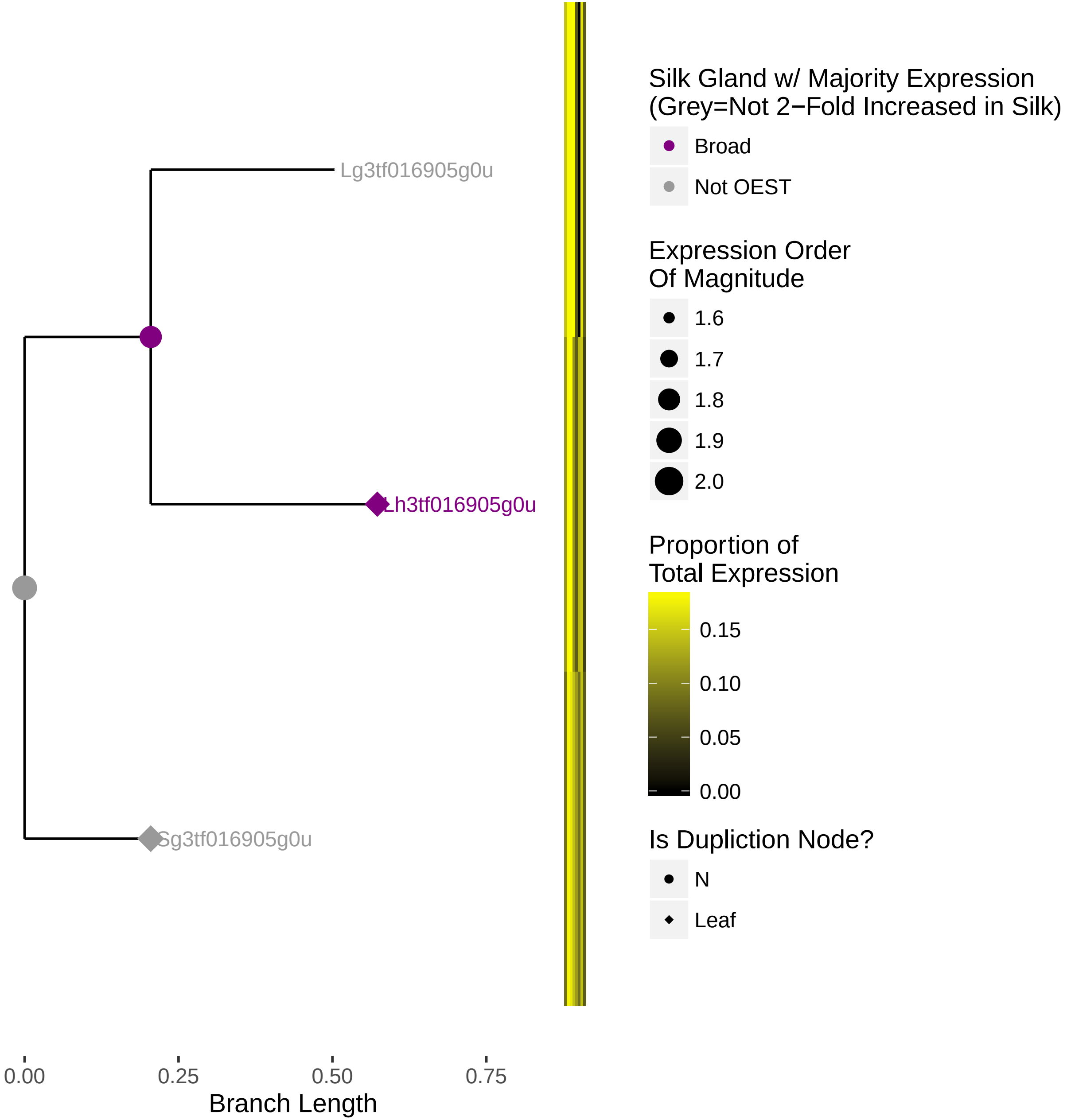
Lg3tf016905g0u
Lh3tf016905g0u
Sg3tf016905g0u
0.00
0.25
0.50
0.75
Branch Length
Silk Gland w/ Majority Expression
(Grey=Not 2−Fold Increased in Silk)
Broad
Not OEST
Expression Order
Of Magnitude
1.6
1.7
1.8
1.9
2.0
Proportion of
Total Expression
0.15
0.10
0.05
0.00
Is Dupliction Node?
N
Leaf

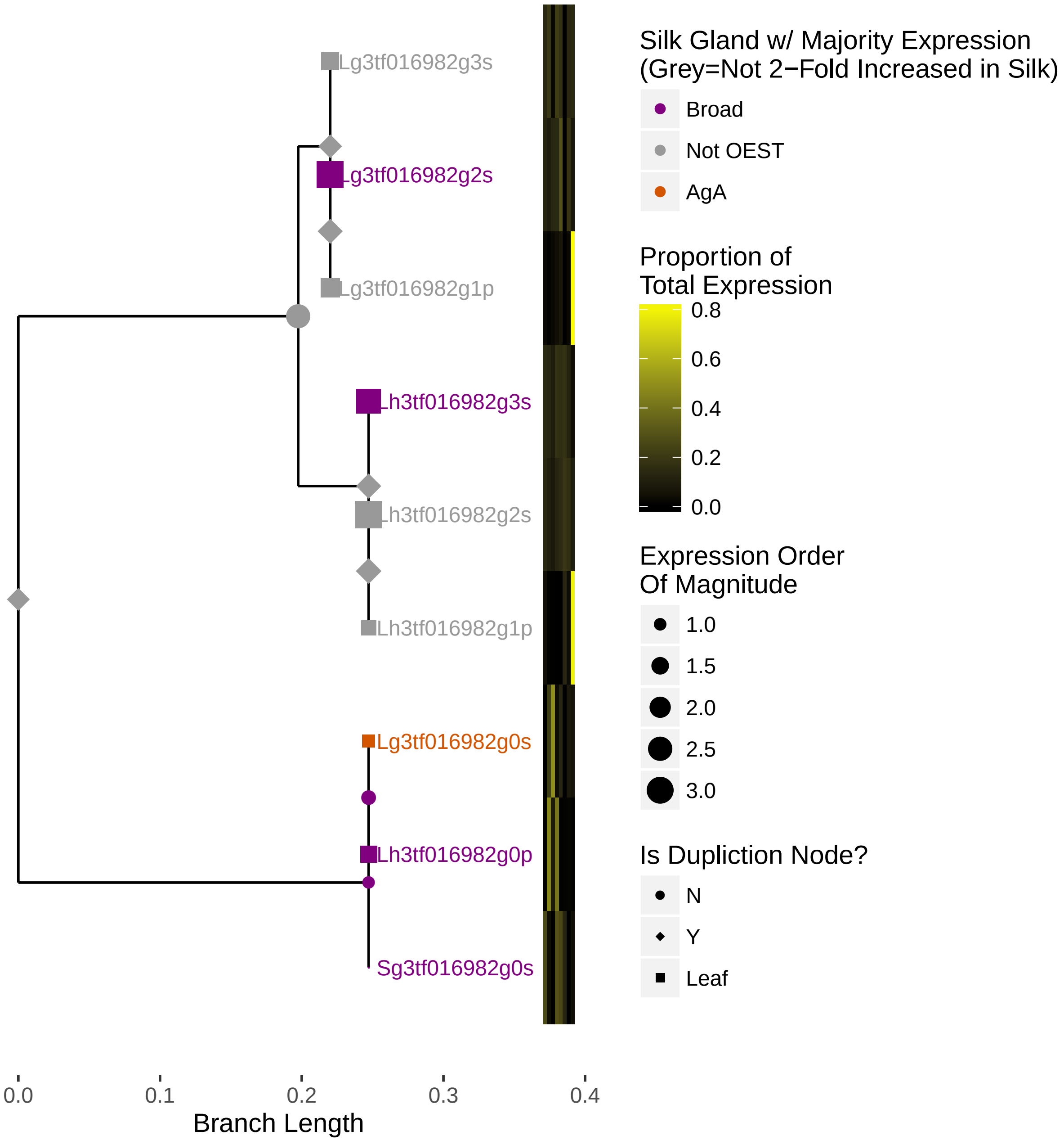
Lg3tf016982g3s
Lg3tf016982g2s
Lg3tf016982g1p
Lh3tf016982g3s
Lh3tf016982g2s
Lh3tf016982g1p
Lg3tf016982g0s
Lh3tf016982g0p
Sg3tf016982g0s
0.0
0.1
0.2
0.3
0.4
Branch Length
Silk Gland w/ Majority Expression
(Grey=Not 2−Fold Increased in Silk)
Broad
Not OEST
AgA
Proportion of
Total Expression
0.8
0.6
0.4
0.2
0.0
Expression Order
Of Magnitude
1.0
1.5
2.0
2.5
3.0
Is Dupliction Node?
N
Y
Leaf

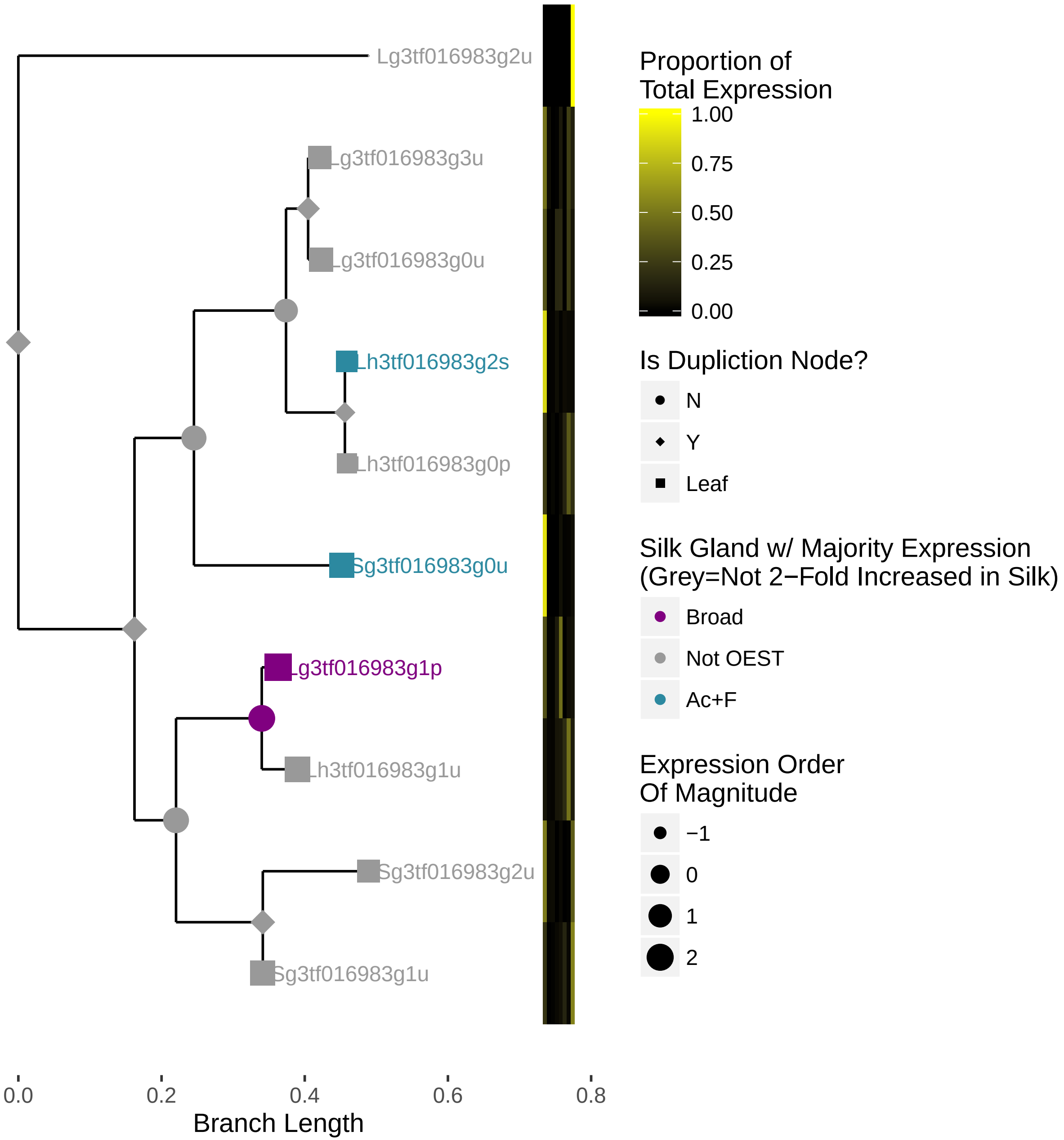

Lg3tf016983g2u
Lg3tf016983g3u
Lg3tf016983g0u
Lh3tf016983g2s
Lh3tf016983g0p
Sg3tf016983g0u
Lg3tf016983g1p
Lh3tf016983g1u
Sg3tf016983g2u
Sg3tf016983g1u
0.0
0.2
0.4
0.6
0.8
Branch Length
Proportion of
Total Expression
1.00
0.75
0.50
0.25
0.00
Is Dupliction Node?
N
Y
Leaf
Silk Gland w/ Majority Expression
(Grey=Not 2−Fold Increased in Silk)
Broad
Not OEST
Ac+F
Expression Order
Of Magnitude
−1
0
1
2

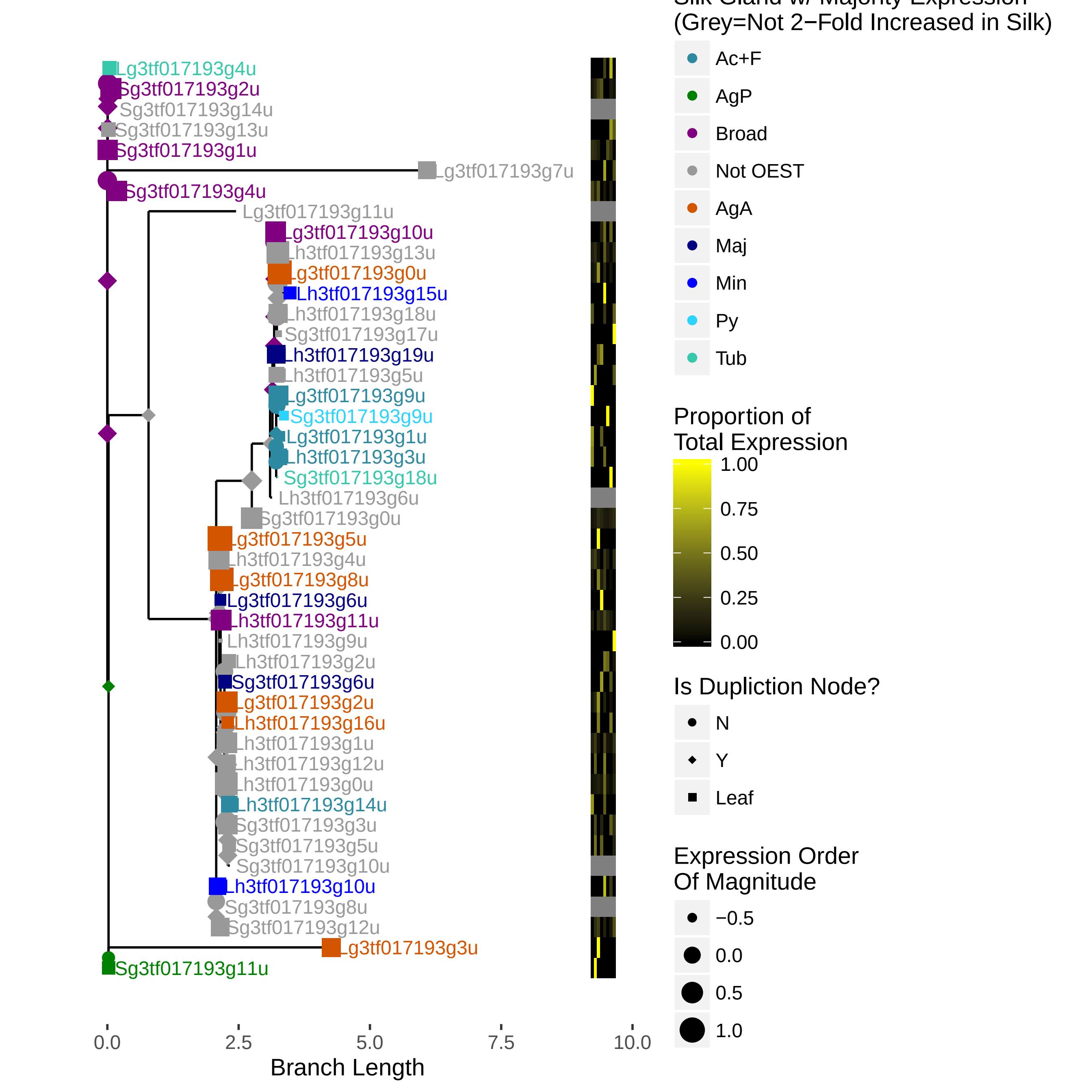

Lg3tf017193g4u
Sg3tf017193g2u
Sg3tf017193g14u
Sg3tf017193g13u
Sg3tf017193g1u
Lg3tf017193g7u
Sg3tf017193g4u
Lg3tf017193g11u
g3tf017193g10u
h3tf017193g13u
g3tf017193g0u
Lh3tf017193g15u
h3tf017193g18u
Sg3tf017193g17u
h3tf017193g19u
Lh3tf017193g5u
g3tf017193g9u
Sg3tf017193g9u
Lg3tf017193g1u
h3tf017193g3u
Sg3tf017193g18u
Lh3tf017193g6u
Sg3tf017193g0u
g3tf017193g5u
h3tf017193g4u
g3tf017193g8u
Lg3tf017193g6u
h3tf017193g11u
Lh3tf017193g9u
Lh3tf017193g2u
Sg3tf017193g6u
g3tf017193g2u
Lh3tf017193g16u
h3tf017193g1u
h3tf017193g12u
h3tf017193g0u
Lh3tf017193g14u
Sg3tf017193g3u
Sg3tf017193g5u
Sg3tf017193g10u
h3tf017193g10u
Sg3tf017193g8u
Sg3tf017193g12u
g3tf017193g3u
Sg3tf017193g11u
0.0
2.5
5.0
7.5
10.0
Branch Length
(Grey=Not 2−Fold Increased in Silk)
Ac+F
AgP
Broad
Not OEST
AgA
Maj
Min
Py
Tub
Proportion of Total Expression
1.00
0.75
0.50
0.25
0.00
Is Dupliction Node?
N
Y
Leaf
Expression Order Of Magnitude
−0.5
0.0
0.5
1.0

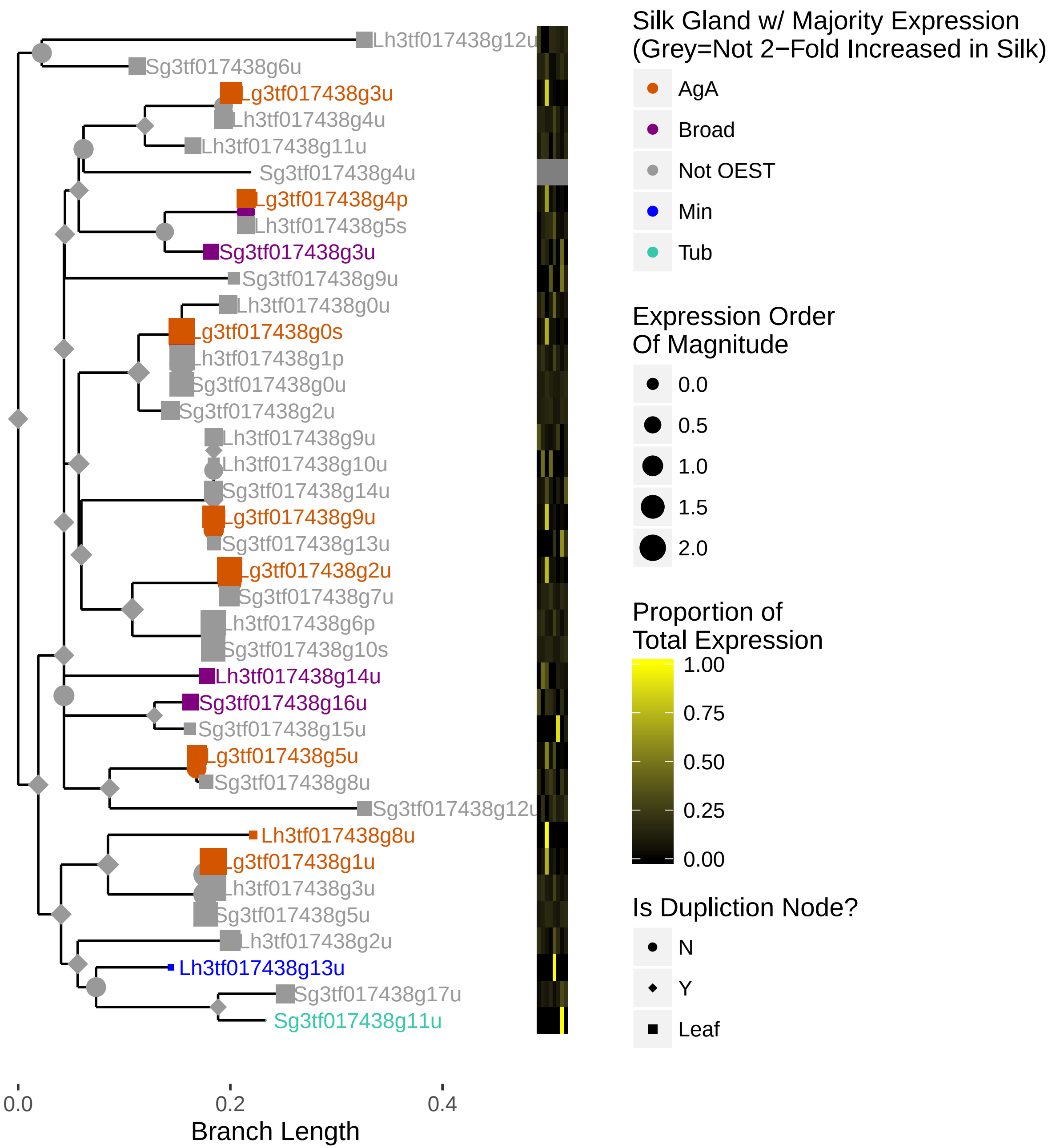
Lh3tf017438g12u
Sg3tf017438g6u
Lg3tf017438g3u
Lh3tf017438g4u
Lh3tf017438g11u
Sg3tf017438g4u
Lg3tf017438g4p
Lh3tf017438g5s
Sg3tf017438g3u
Sg3tf017438g9u
Lh3tf017438g0u
Lg3tf017438g0s
Lh3tf017438g1p
Sg3tf017438g0u
Sg3tf017438g2u
Lh3tf017438g9u
Lh3tf017438g10u
Sg3tf017438g14u
Lg3tf017438g9u
Sg3tf017438g13u
Lg3tf017438g2u
Sg3tf017438g7u
Lh3tf017438g6p
Sg3tf017438g10s
Lh3tf017438g14u
Sg3tf017438g16u
Sg3tf017438g15u
Lg3tf017438g5u
Sg3tf017438g8u
Sg3tf017438g12u
Lh3tf017438g8u
Lg3tf017438g1u
Lh3tf017438g3u
Sg3tf017438g5u
Lh3tf017438g2u
Lh3tf017438g13u
Sg3tf017438g17u
Sg3tf017438g11u
0.0
0.2
0.4
Branch Length
Silk Gland w/ Majority Expression
(Grey=Not 2−Fold Increased in Silk)
AgA
Broad
Not OEST
Min
Tub
Expression Order
Of Magnitude
0.0
0.5
1.0
1.5
2.0
Proportion of
Total Expression
1.00
0.75
0.50
0.25
0.00
Is Duplicion Node?
N
Y
Leaf

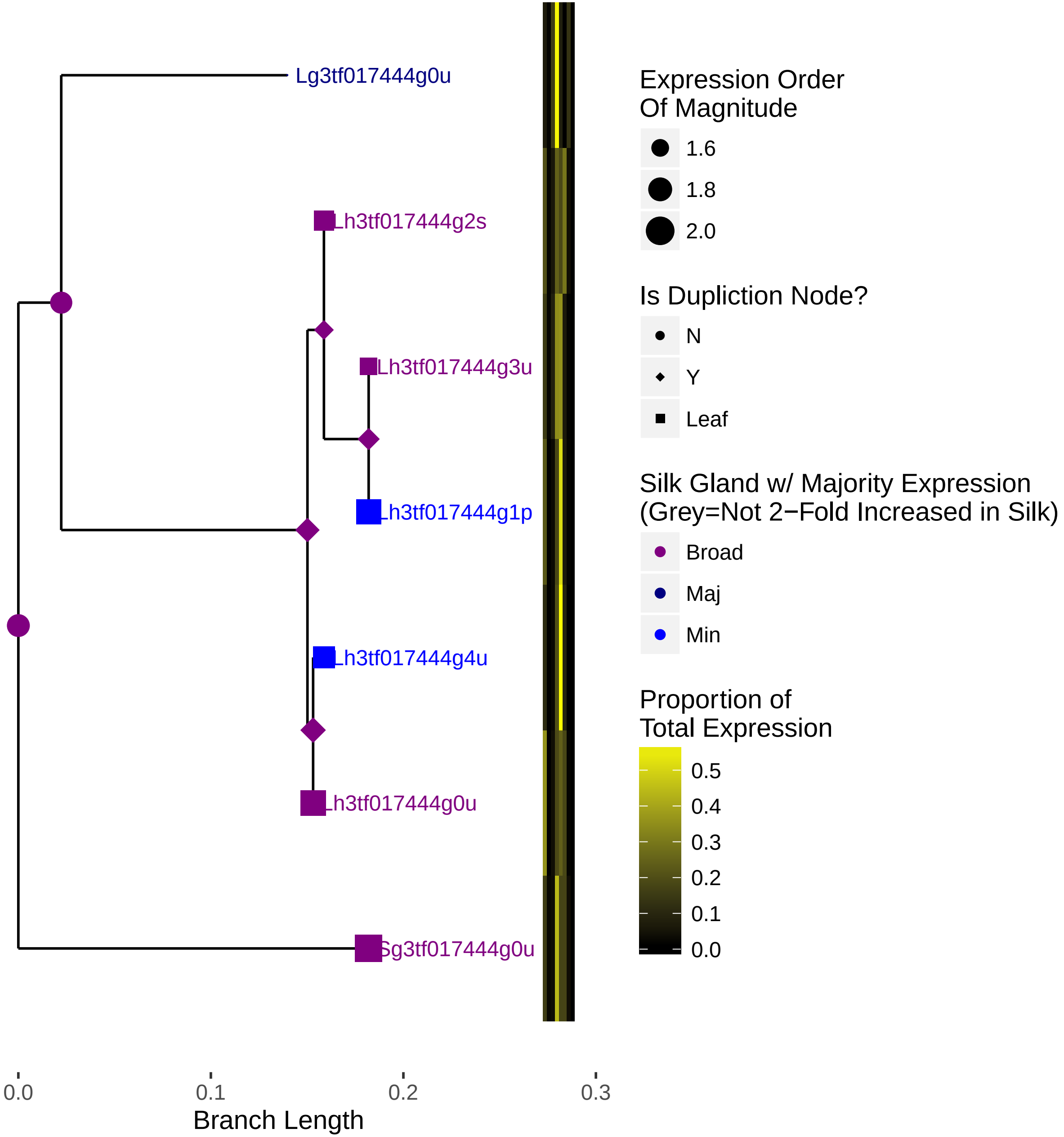
Lg3tf017444g0u
Lh3tf017444g2s
Lh3tf017444g3u
Lh3tf017444g1p
Lh3tf017444g4u
Lh3tf017444g0u
Sg3tf017444g0u
Expression Order Of Magnitude
1.6
1.8
2.0
Is Dupliction Node?
N
Y
Leaf
Silk Gland w/ Majority Expression (Grey=Not 2−Fold Increased in Silk)
Broad
Maj
Min
Proportion of Total Expression
0.5
0.4
0.3
0.2
0.1
0.0
0.0
0.1
0.2
0.3
Branch Length

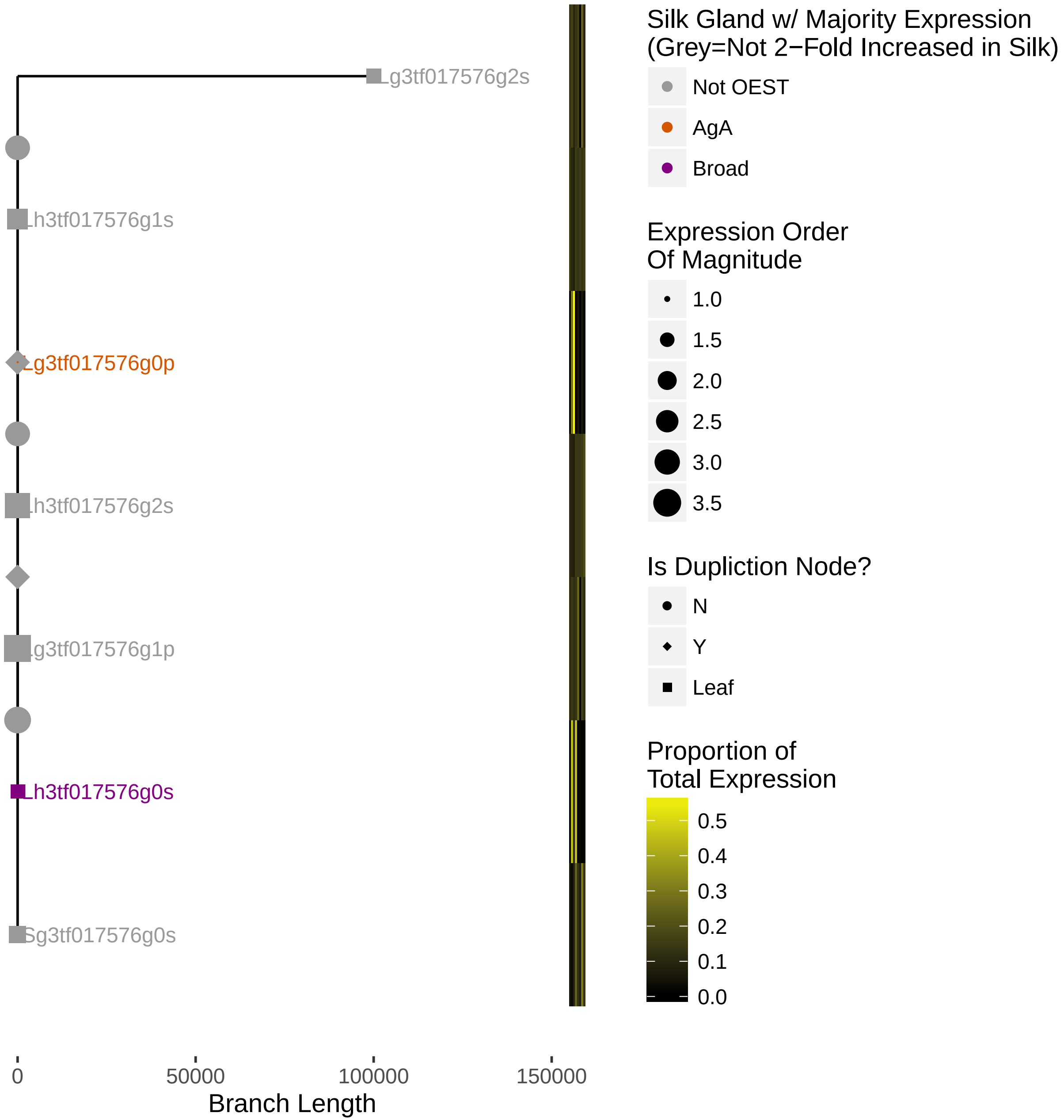

Lg3tf017576g2s
Lh3tf017576g1s
Lg3tf017576g0p
Lh3tf017576g2s
Lg3tf017576g1p
Lh3tf017576g0s
Sg3tf017576g0s
0
50000
100000
150000
Branch Length
Silk Gland w/ Majority Expression
(Grey=Not 2−Fold Increased in Silk)
Not OEST
AgA
Broad
Expression Order
Of Magnitude
1.0
1.5
2.0
2.5
3.0
3.5
Is Dupliction Node?
N
Y
Leaf
Proportion of
Total Expression
0.5
0.4
0.3
0.2
0.1
0.0

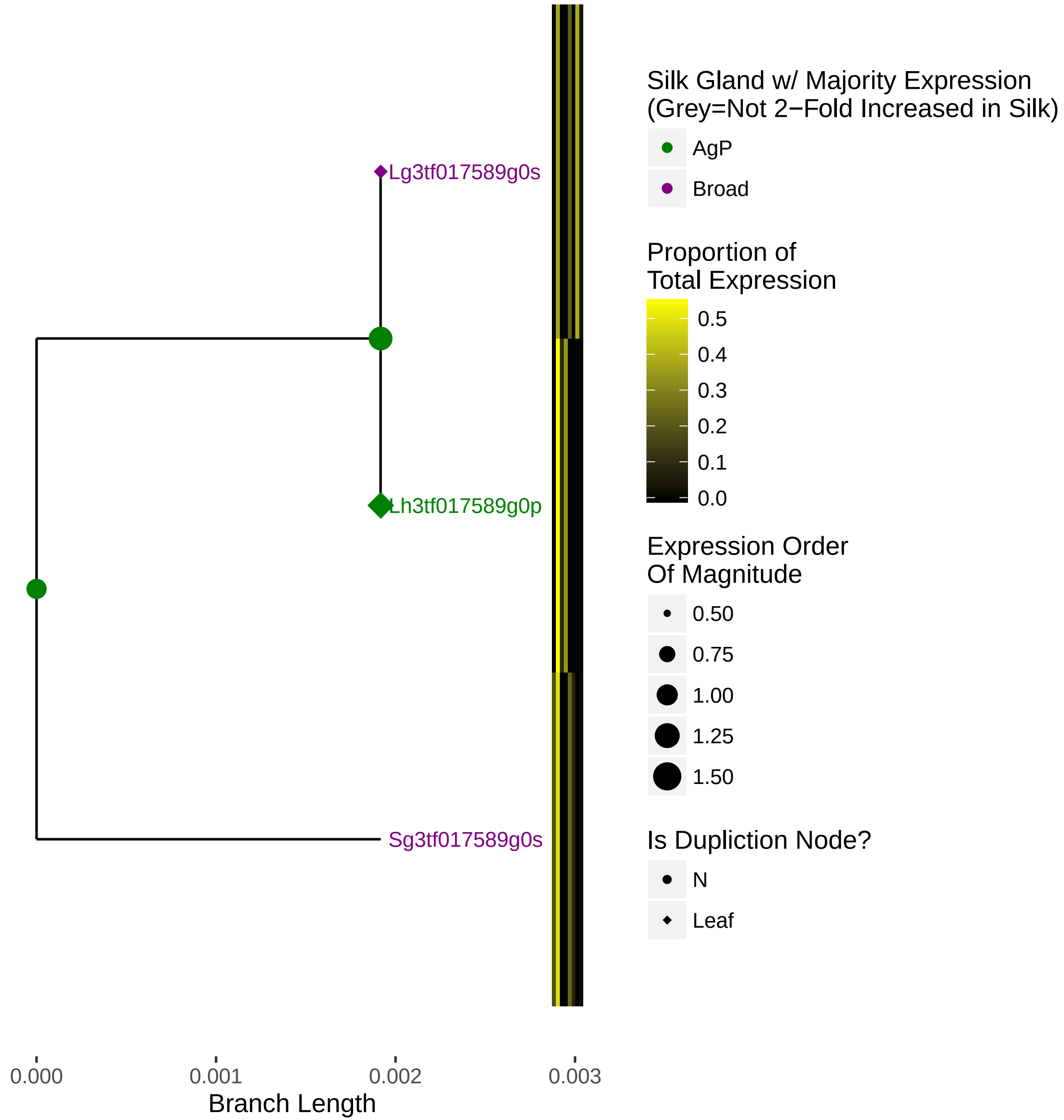
Lg3tf017589g0s
Lh3tf017589g0p
Sg3tf017589g0s
0.000
0.001
0.002
0.003
Branch Length
Silk Gland w/ Majority Expression
(Grey=Not 2−Fold Increased in Silk)
AgP
Broad
Proportion of
Total Expression
0.5
0.4
0.3
0.2
0.1
0.0
Expression Order
Of Magnitude
0.50
0.75
1.00
1.25
1.50
Is Dupliction Node?
N
Leaf

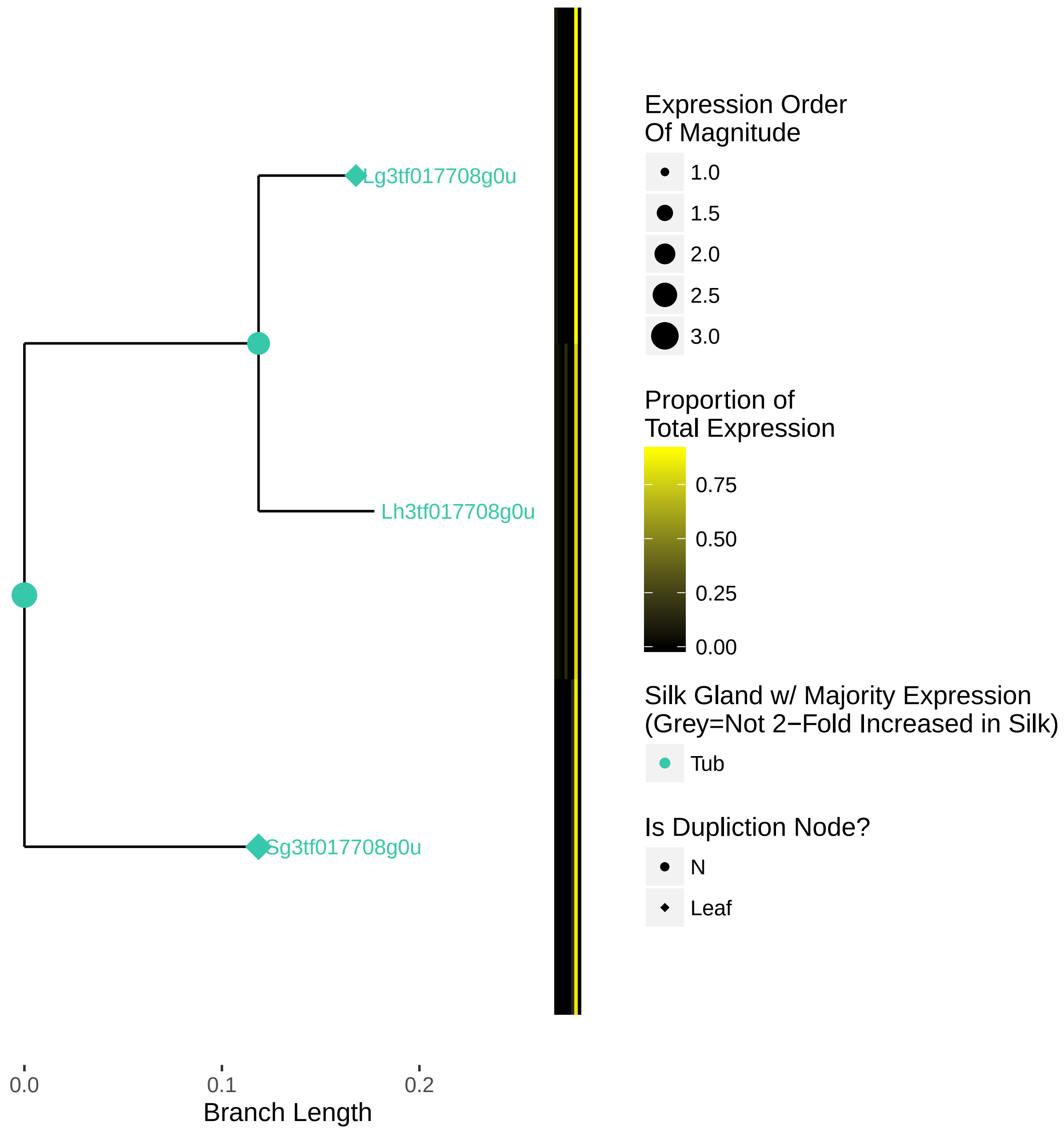

Lg3tf017708g0u
Lh3tf017708g0u
Sg3tf017708g0u
0.0
0.1
0.2
Branch Length
Expression Order Of Magnitude
1.0
1.5
2.0
2.5
3.0
Proportion of Total Expression
0.75
0.50
0.25
0.00
Silk Gland w/ Majority Expression (Grey=Not 2−Fold Increased in Silk)
Tub
Is Dupliction Node?
N
Leaf

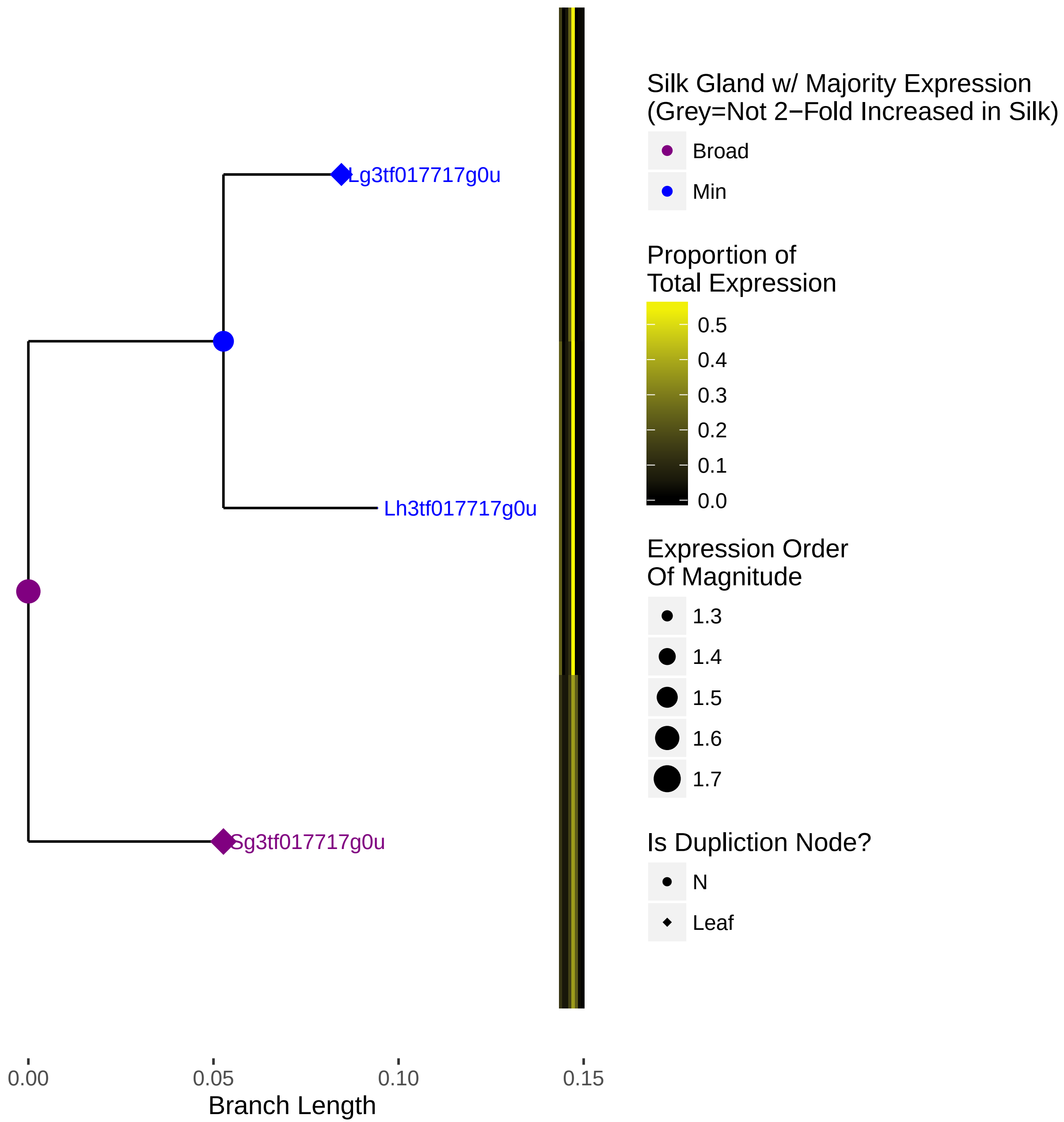
Lg3tf017717g0u
Lh3tf017717g0u
Sg3tf017717g0u
Silk Gland w/ Majority Expression (Grey=Not 2−Fold Increased in Silk)
Broad
Min
Proportion of Total Expression
0.5
0.4
0.3
0.2
0.1
0.0
Expression Order Of Magnitude
1.3
1.4
1.5
1.6
1.7
Is Dupliction Node?
N
Leaf
0.00
0.05
0.10
0.15
Branch Length

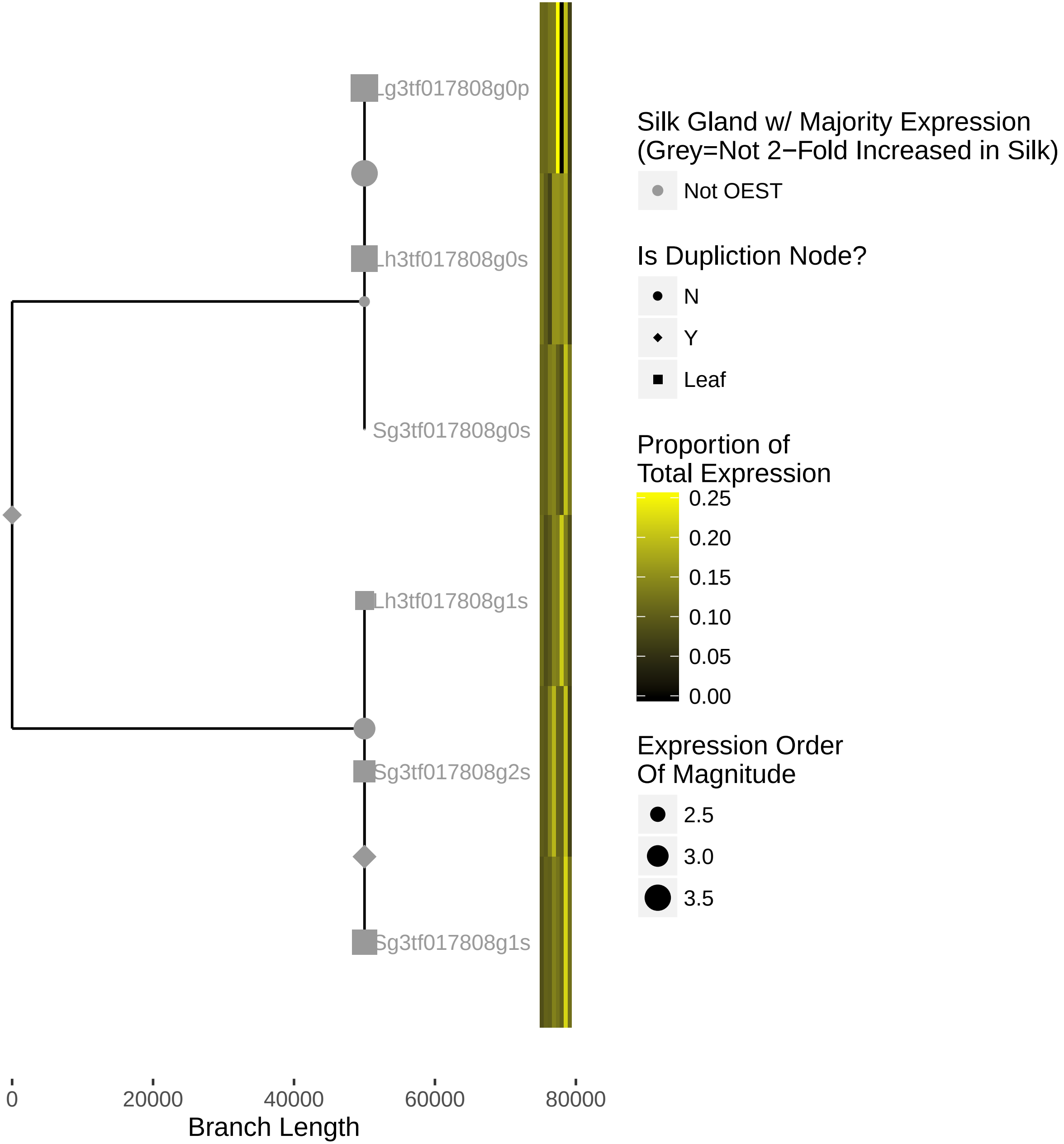
Lg3tf017808g0p
Lh3tf017808g0s
Sg3tf017808g0s
Lh3tf017808g1s
Sg3tf017808g2s
Sg3tf017808g1s
0
20000
40000
60000
80000
Branch Length
Silk Gland w/ Majority Expression (Grey=Not 2−Fold Increased in Silk)
Not OEST
Is Dupliction Node?
N
Y
Leaf
Proportion of Total Expression
0.25
0.20
0.15
0.10
0.05
0.00
Expression Order Of Magnitude
2.5
3.0
3.5

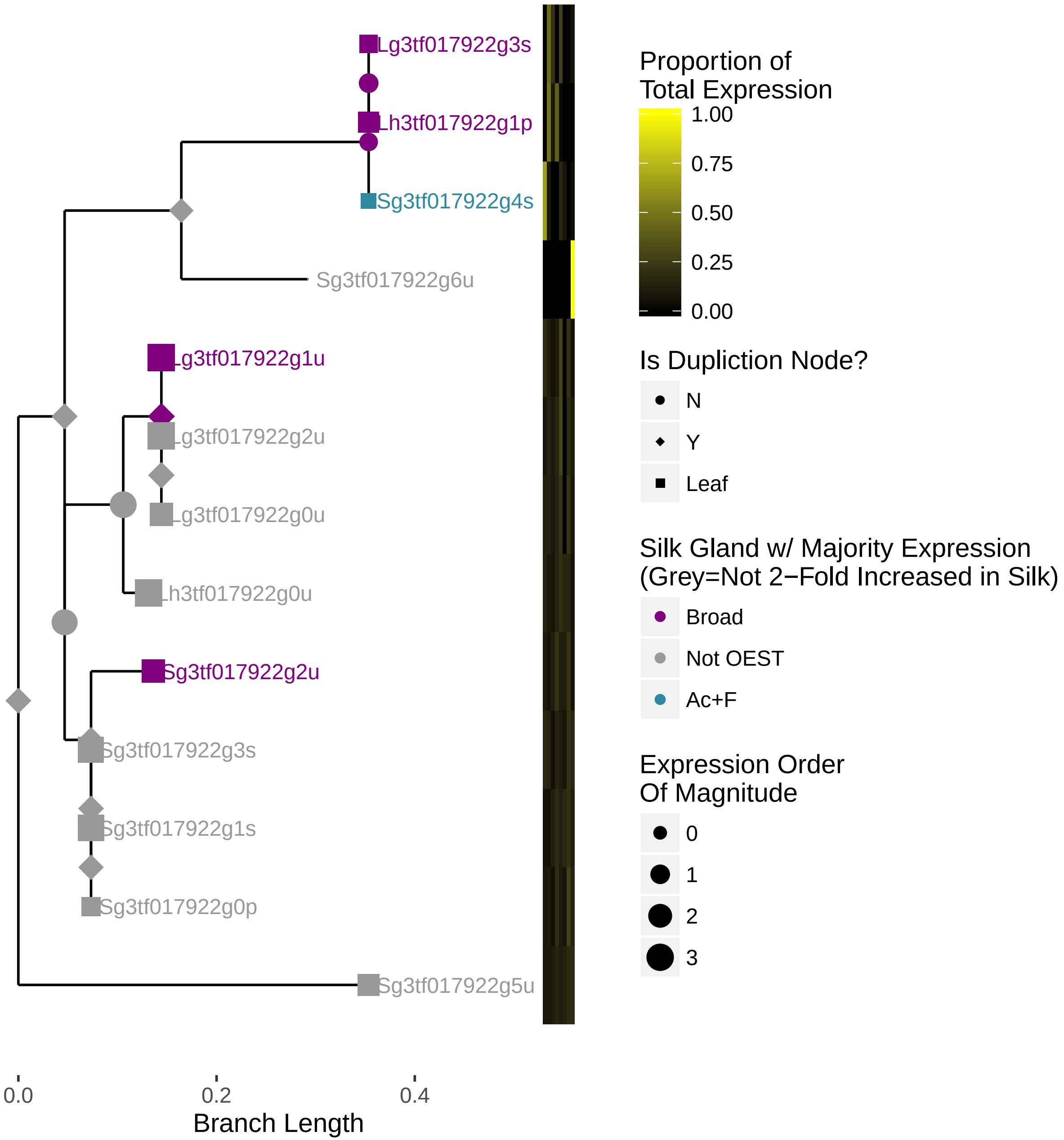

Lg3tf017922g3s
Lh3tf017922g1p
Sg3tf017922g4s
Sg3tf017922g6u
Lg3tf017922g1u
Lg3tf017922g2u
Lg3tf017922g0u
Lh3tf017922g0u
Sg3tf017922g2u
Sg3tf017922g3s
Sg3tf017922g1s
Sg3tf017922g0p
Sg3tf017922g5u
0.0
0.2
0.4
Branch Length
Proportion of
Total Expression
1.00
0.75
0.50
0.25
0.00
Is Dupliction Node?
N
Y
Leaf
Silk Gland w/ Majority Expression
(Grey=Not 2−Fold Increased in Silk)
Broad
Not OEST
Ac+F
Expression Order
Of Magnitude
0
1
2
3

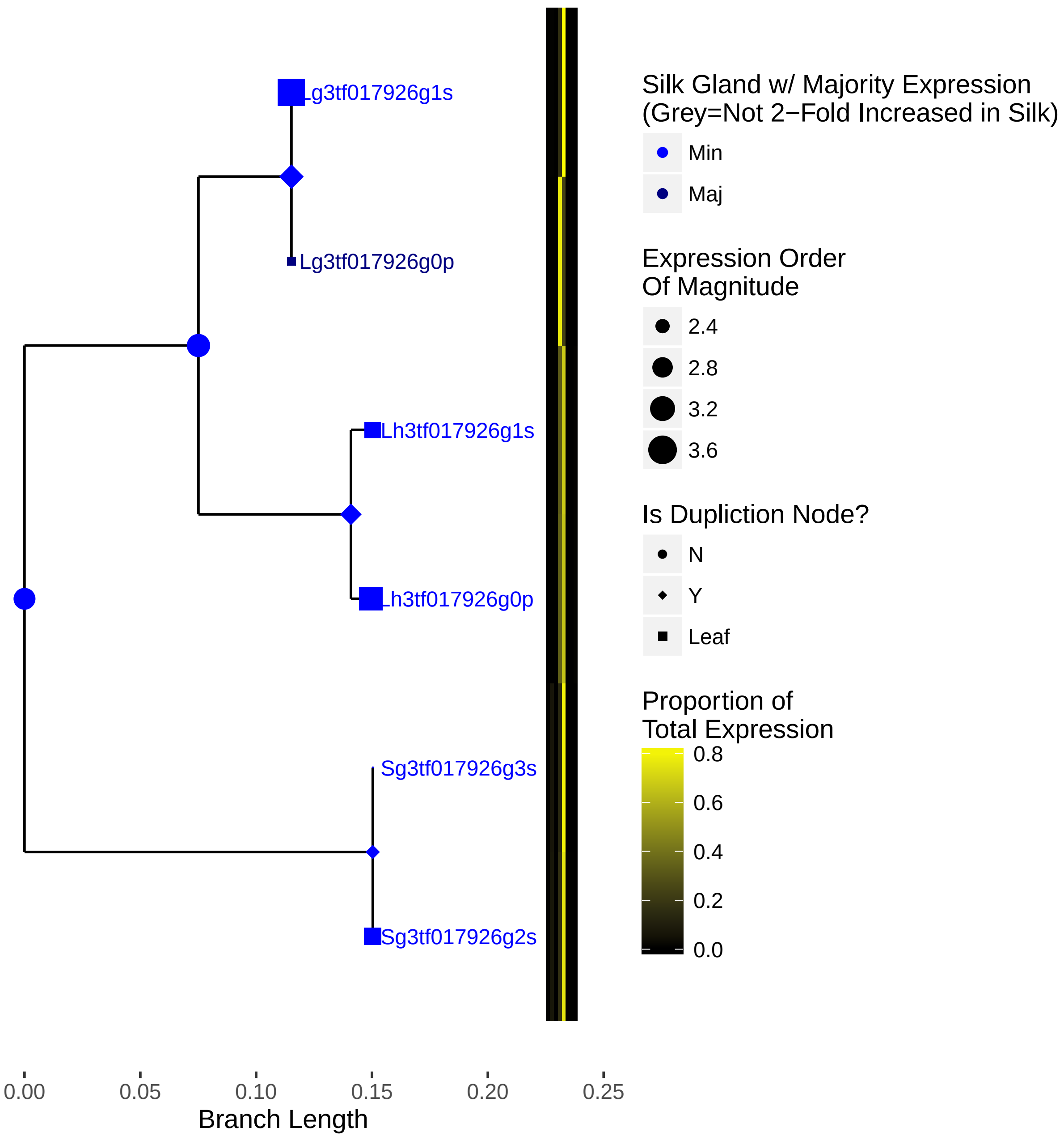
Lg3tf017926g1s
Lg3tf017926g0p
Lh3tf017926g1s
Lh3tf017926g0p
Sg3tf017926g3s
Sg3tf017926g2s
Silk Gland w/ Majority Expression
(Grey=Not 2−Fold Increased in Silk)
Min
Maj
Expression Order
Of Magnitude
2.4
2.8
3.2
3.6
Is Dupliction Node?
N
Y
Leaf
Proportion of
Total Expression
0.8
0.6
0.4
0.2
0.0
0.00
0.05
0.10
0.15
0.20
0.25
Branch Length

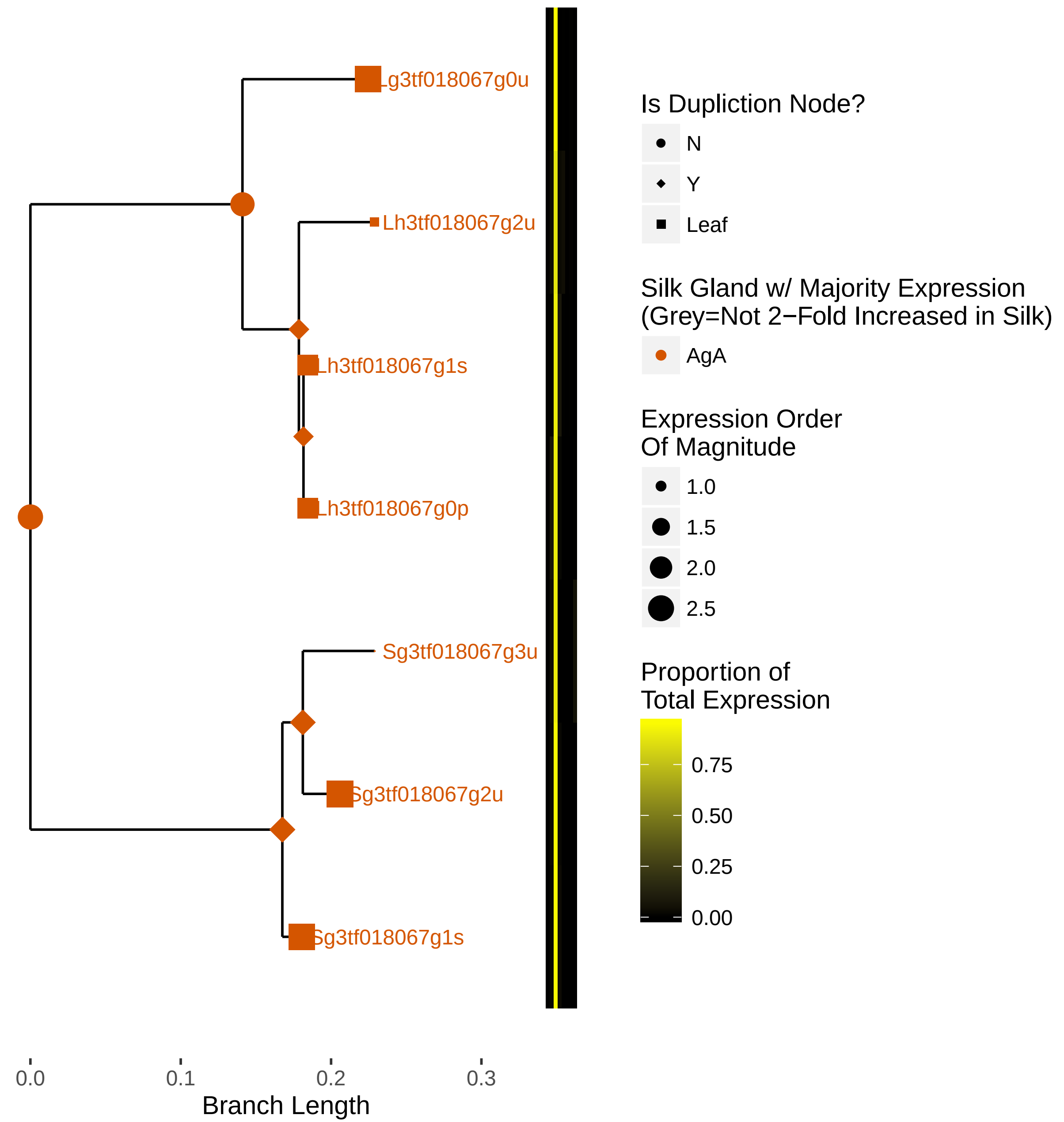
Lg3tf018067g0u
Lh3tf018067g2u
Lh3tf018067g1s
Lh3tf018067g0p
Sg3tf018067g3u
Sg3tf018067g2u
Sg3tf018067g1s
Is Dupliction Node?
N
Y
Leaf
Silk Gland w/ Majority Expression
(Grey=Not 2−Fold Increased in Silk)
AgA
Expression Order
Of Magnitude
1.0
1.5
2.0
2.5
Proportion of
Total Expression
0.75
0.50
0.25
0.00
0.0
0.1
0.2
0.3
Branch Length

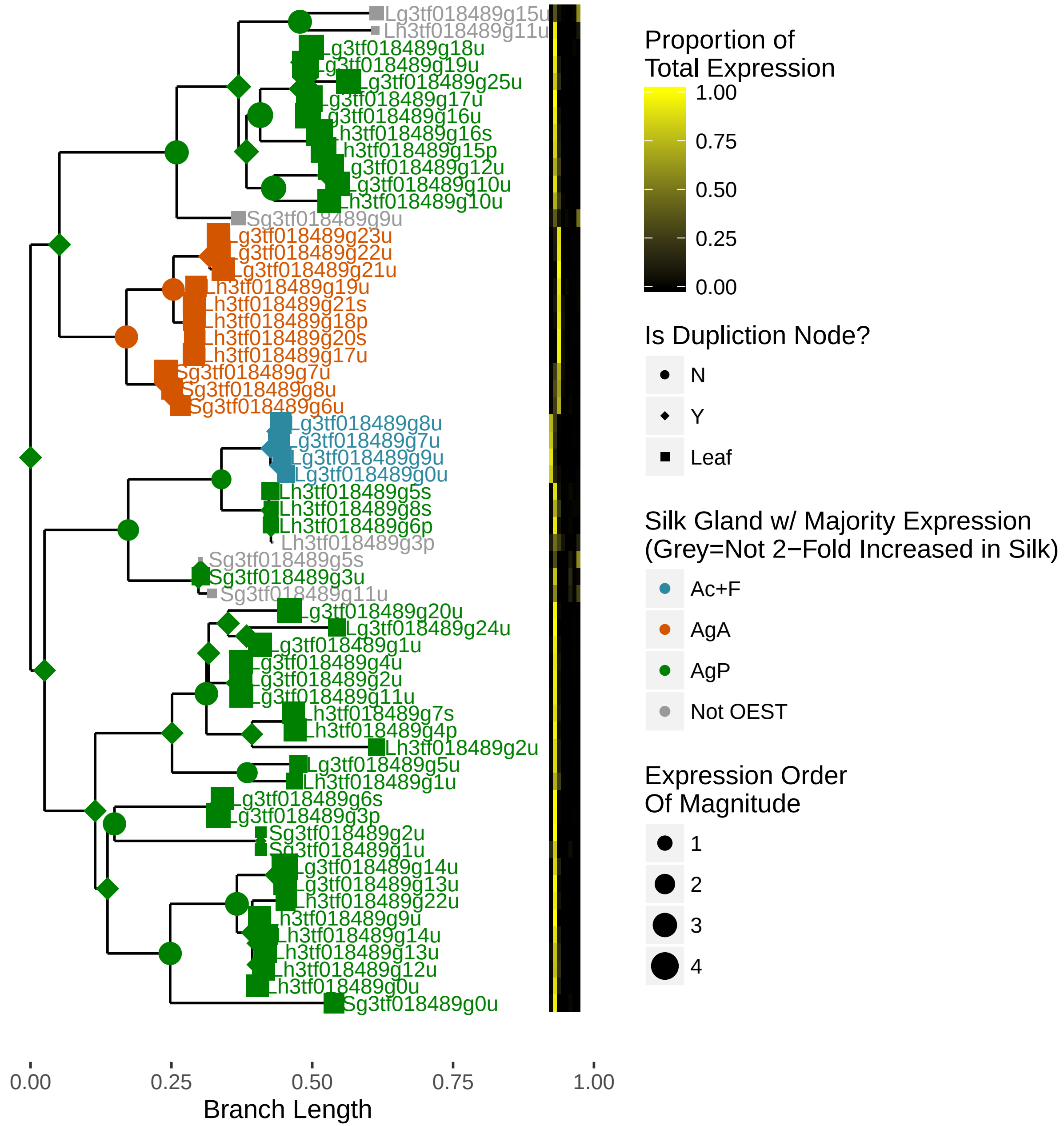
Lg3tf018489g15u
Lh3tf018489g11u
g3tf018489g18u
g3tf018489g19u
g3tf018489g25u
g3tf018489g17u
g3tf018489g16u
h3tf018489g16s
h3tf018489g15p
g3tf018489g12u
g3tf018489g10u
Lh3tf018489g10u
Sg3tf018489g9u
g3tf018489g23u
g3tf018489g22u
g3tf018489g21u
Lh3tf018489g19u
Lh3tf018489g21s
Lh3tf018489g18p
Lh3tf018489g20s
Lh3tf018489g17u
Sg3tf018489g7u
Sg3tf018489g8u
Sg3tf018489g6u
g3tf018489g8u
g3tf018489g7u
g3tf018489g9u
g3tf018489g0u
Lh3tf018489g5s
Lh3tf018489g8s
Lh3tf018489g6p
Lh3tf018489g3p
Sg3tf018489g5s
Sg3tf018489g3u
Sg3tf018489g11u
g3tf018489g20u
g3tf018489g24u
g3tf018489g1u
g3tf018489g4u
g3tf018489g2u
g3tf018489g11u
h3tf018489g7s
h3tf018489g4p
Lh3tf018489g2u
Lg3tf018489g5u
Lh3tf018489g1u
g3tf018489g6s
g3tf018489g3p
Sg3tf018489g2u
Sg3tf018489g1u
g3tf018489g14u
g3tf018489g13u
h3tf018489g22u
h3tf018489g9u
h3tf018489g14u
h3tf018489g13u
h3tf018489g12u
h3tf018489g0u
Sg3tf018489g0u
0.00
0.25
0.50
0.75
1.00
Branch Length
Proportion of
Total Expression
1.00
0.75
0.50
0.25
0.00
Is Dupliction Node?
N
Y
Leaf
Silk Gland w/ Majority Expression
(Grey=Not 2−Fold Increased in Silk)
Ac+F
AgA
AgP
Not OEST
Expression Order
Of Magnitude
1
2
3
4

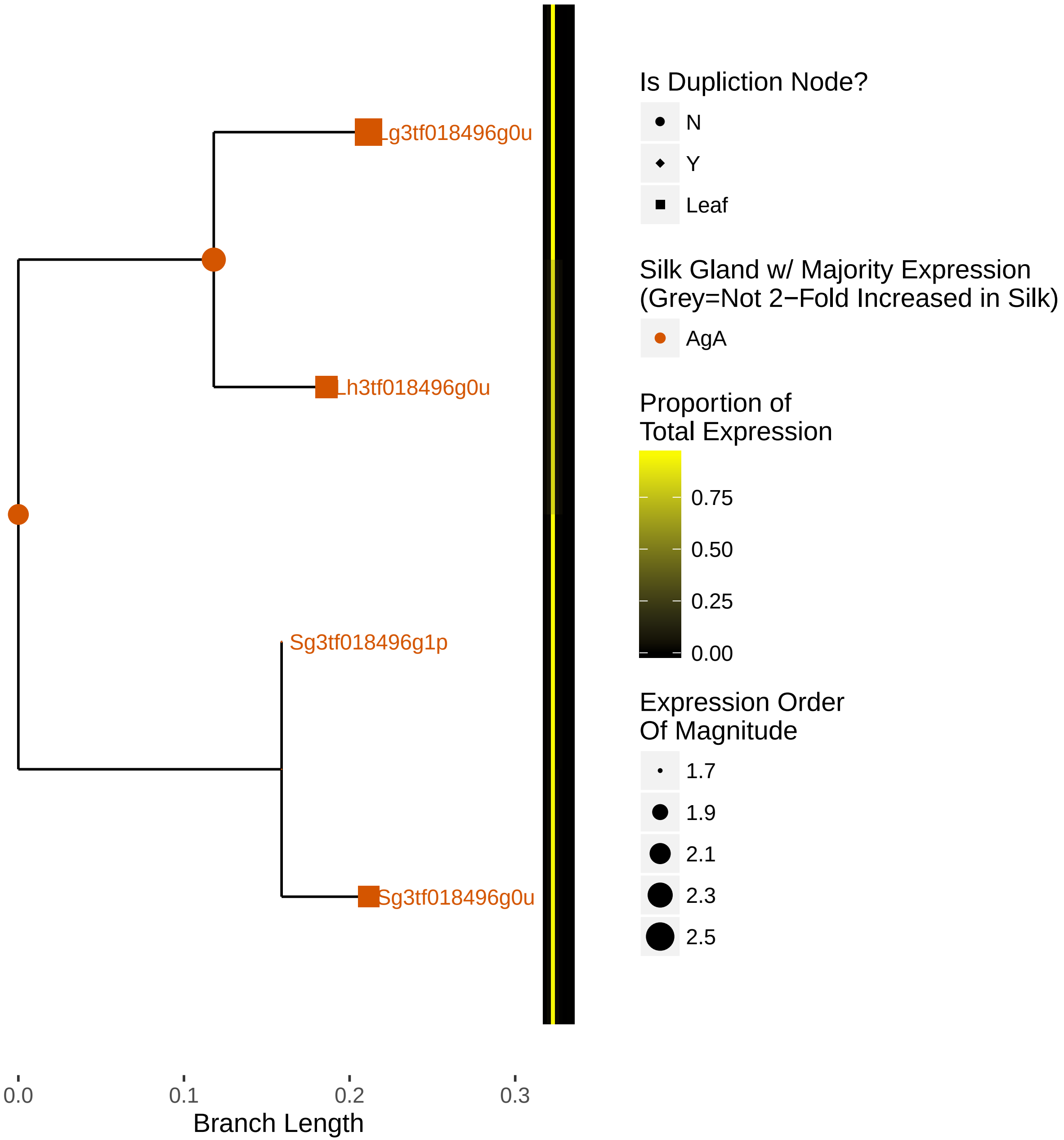

Lg3tf018496g0u
Lh3tf018496g0u
Sg3tf018496g1p
Sg3tf018496g0u
0.0
0.1
0.2
0.3
Branch Length
Is Dupliction Node?
N
Y
Leaf
Silk Gland w/ Majority Expression
(Grey=Not 2−Fold Increased in Silk)
AgA
Proportion of
Total Expression
0.75
0.50
0.25
0.00
Expression Order
Of Magnitude
1.7
1.9
2.1
2.3
2.5

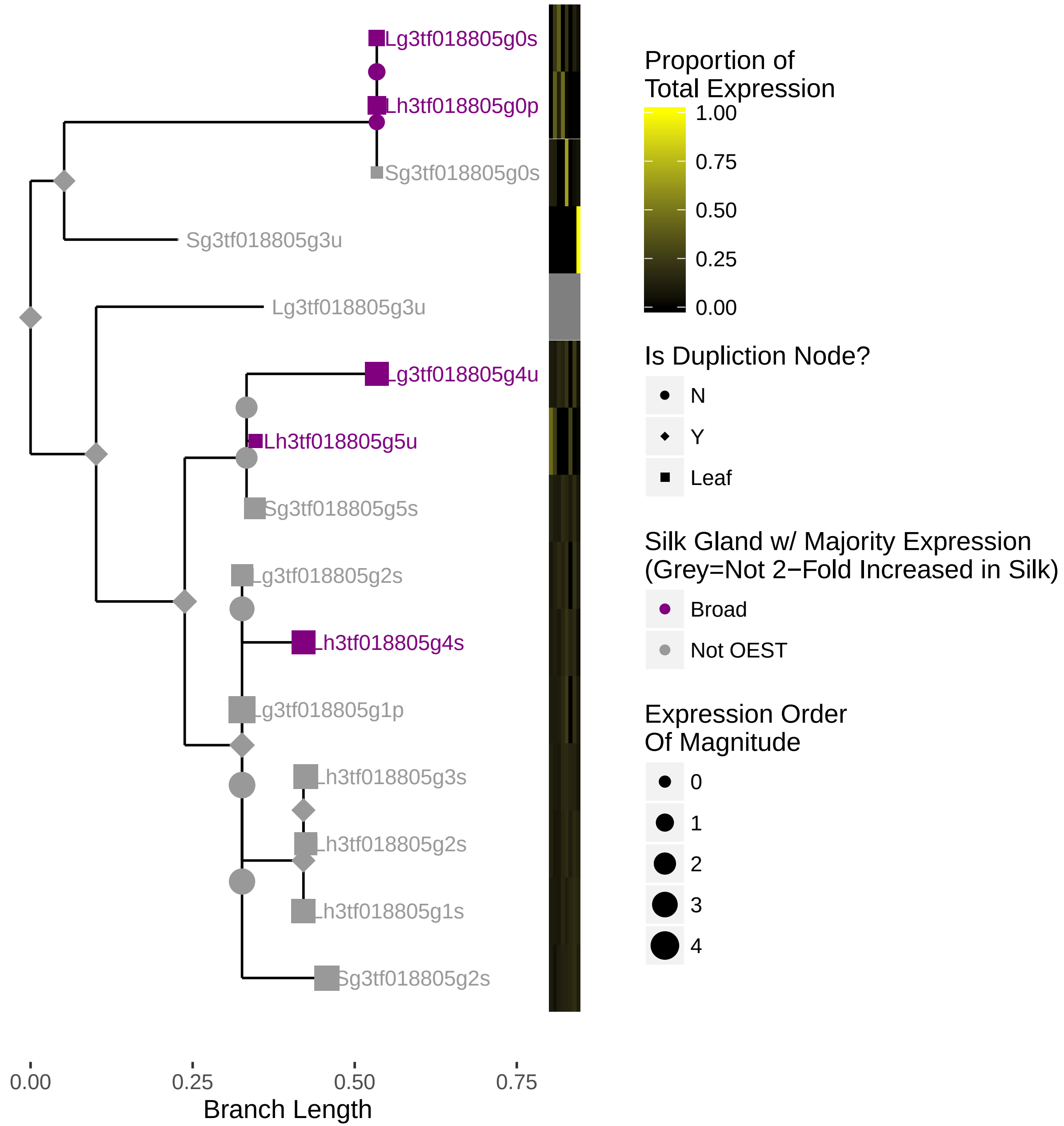

Lg3tf018805g0s
Lh3tf018805g0p
Sg3tf018805g0s
Sg3tf018805g3u
Lg3tf018805g3u
Lg3tf018805g4u
Lh3tf018805g5u
Sg3tf018805g5s
Lg3tf018805g2s
Lh3tf018805g4s
Lg3tf018805g1p
Lh3tf018805g3s
Lh3tf018805g2s
Lh3tf018805g1s
Sg3tf018805g2s
0.00
0.25
0.50
0.75
Branch Length
Proportion of Total Expression
1.00
0.75
0.50
0.25
0.00
Is Dupliction Node?
N
Y
Leaf
Silk Gland w/ Majority Expression (Grey=Not 2−Fold Increased in Silk)
Broad
Not OEST
Expression Order Of Magnitude
0
1
2
3
4

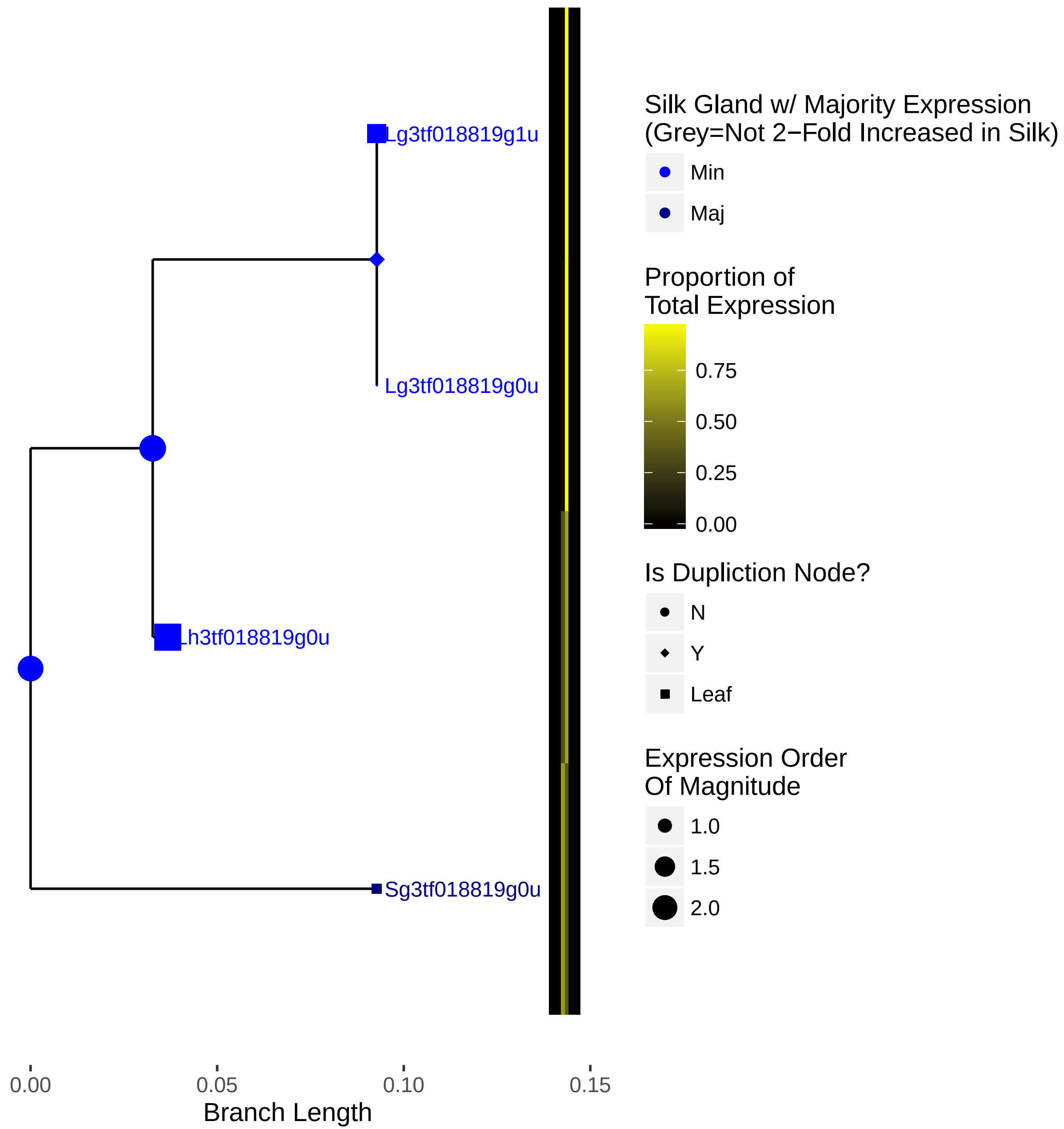

Lg3tf018819g1u
Lg3tf018819g0u
Lh3tf018819g0u
Sg3tf018819g0u
Silk Gland w/ Majority Expression
(Grey=Not 2−Fold Increased in Silk)
Min
Maj
Proportion of
Total Expression
0.75
0.50
0.25
0.00
Is Dupliction Node?
N
Y
Leaf
Expression Order
Of Magnitude
1.0
1.5
2.0
0.00
0.05
0.10
0.15
Branch Length

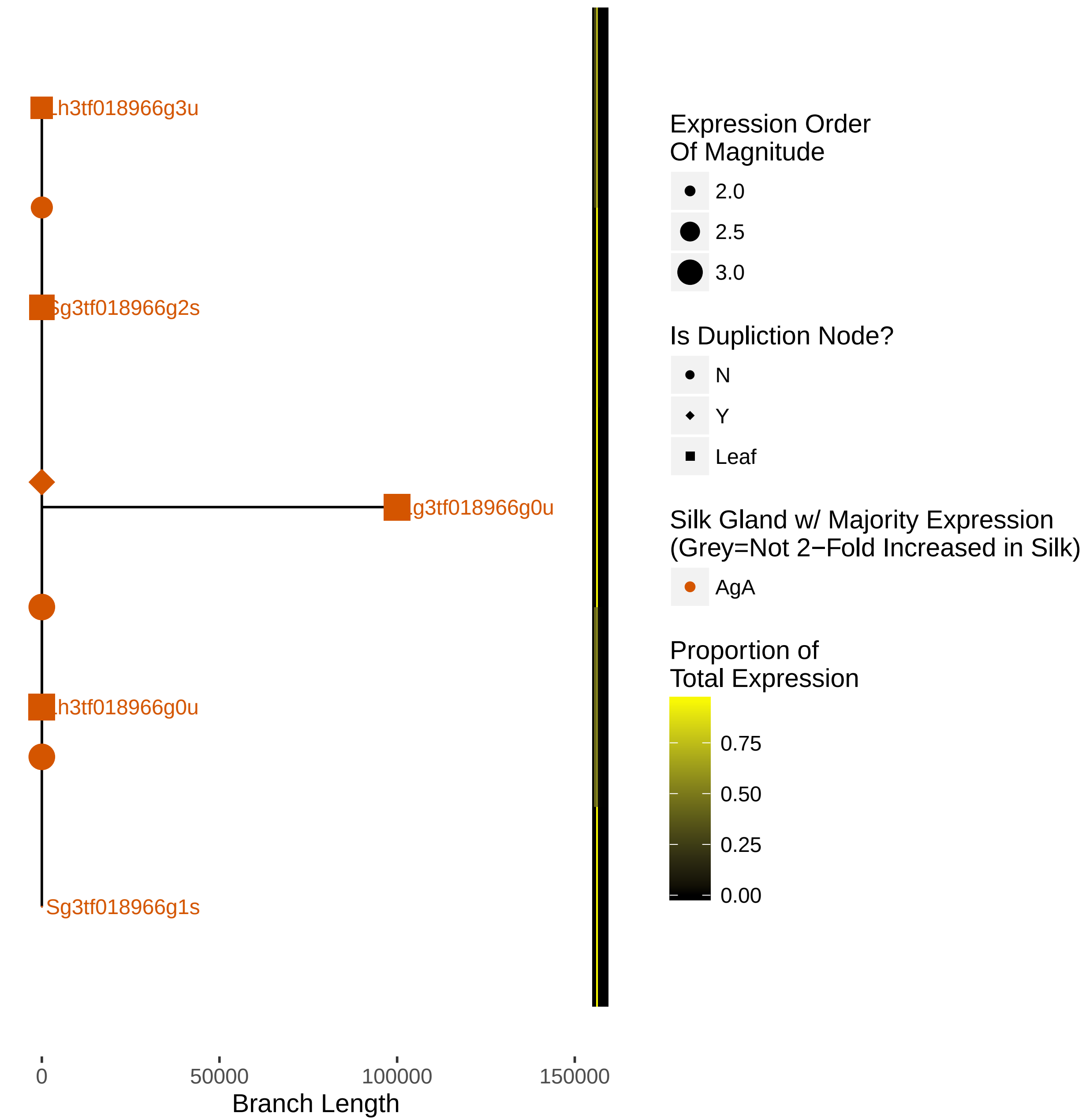

h3tf018966g3u
Sg3tf018966g2s
g3tf018966g0u
h3tf018966g0u
Sg3tf018966g1s
0
50000
100000
150000
Branch Length
Expression Order
Of Magnitude
2.0
2.5
3.0
Is Dupliction Node?
N
Y
Leaf
Silk Gland w/ Majority Expression
(Grey=Not 2−Fold Increased in Silk)
AgA
Proportion of
Total Expression
0.75
0.50
0.25
0.00

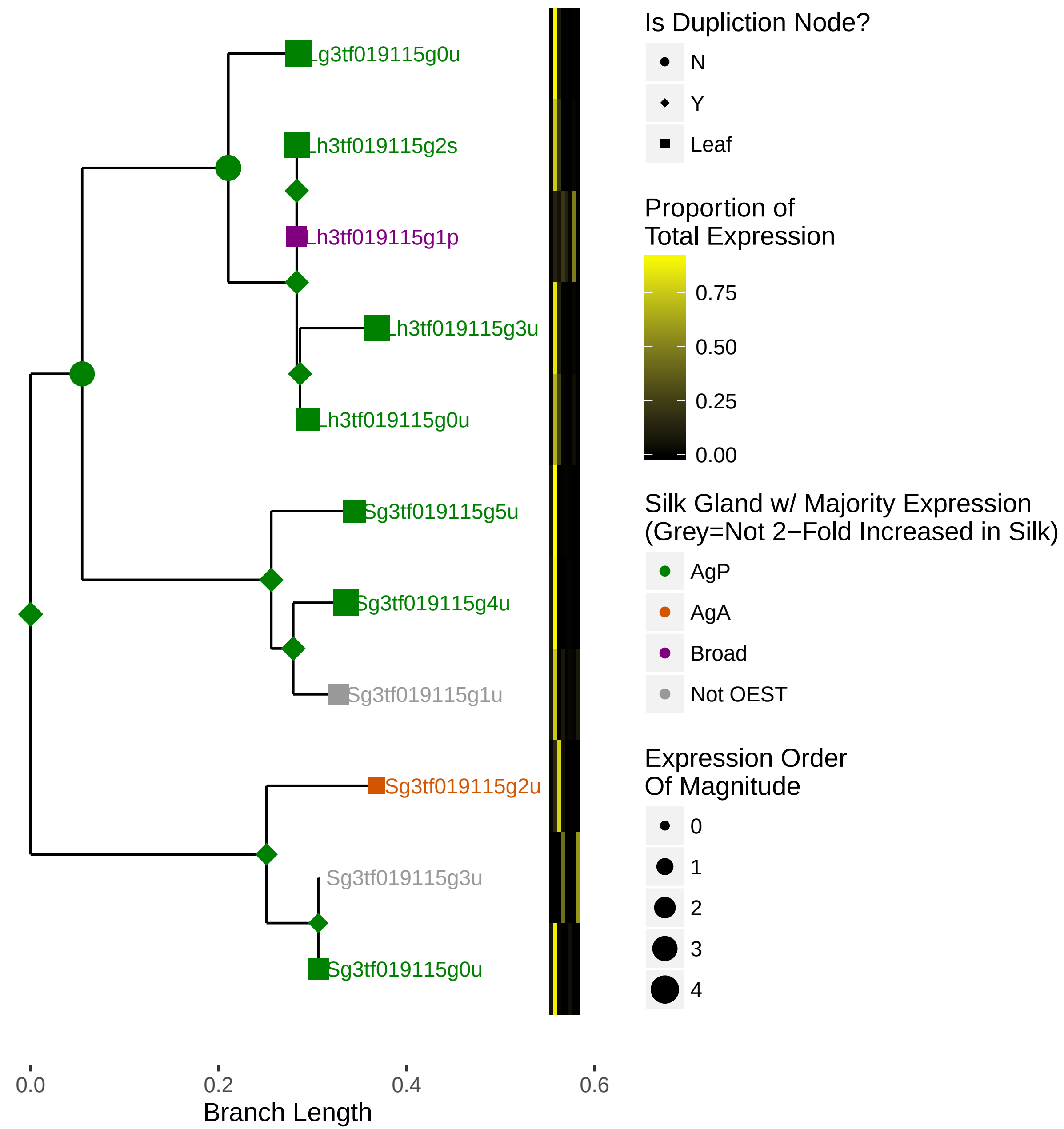

Lg3tf019115g0u
Lh3tf019115g2s
Lh3tf019115g1p
Lh3tf019115g3u
Lh3tf019115g0u
Sg3tf019115g5u
Sg3tf019115g4u
Sg3tf019115g1u
Sg3tf019115g2u
Sg3tf019115g3u
Sg3tf019115g0u
0.0
0.2
0.4
0.6
Branch Length
Is Dupliction Node?
N
Y
Leaf
Proportion of
Total Expression
0.75
0.50
0.25
0.00
Silk Gland w/ Majority Expression
(Grey=Not 2−Fold Increased in Silk)
AgP
AgA
Broad
Not OEST
Expression Order
Of Magnitude
0
1
2
3
4

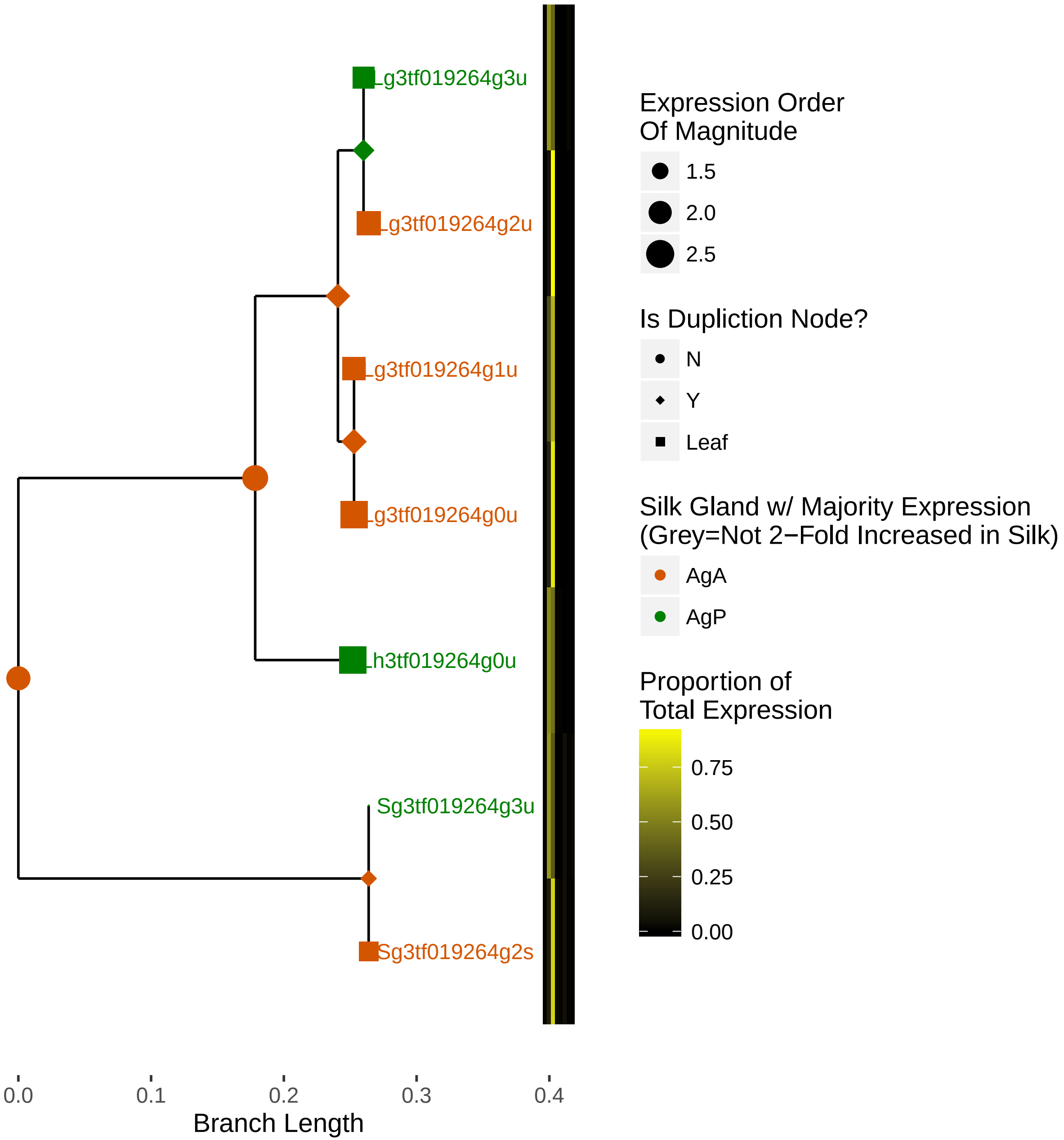

Lg3tf019264g3u
Lg3tf019264g2u
Lg3tf019264g1u
Lg3tf019264g0u
Lh3tf019264g0u
Sg3tf019264g3u
Sg3tf019264g2s
0.0
0.1
0.2
0.3
0.4
Branch Length
Expression Order Of Magnitude
1.5
2.0
2.5
Is Dupliction Node?
N
Y
Leaf
Silk Gland w/ Majority Expression (Grey=Not 2−Fold Increased in Silk)
AgA
AgP
Proportion of Total Expression
0.75
0.50
0.25
0.00

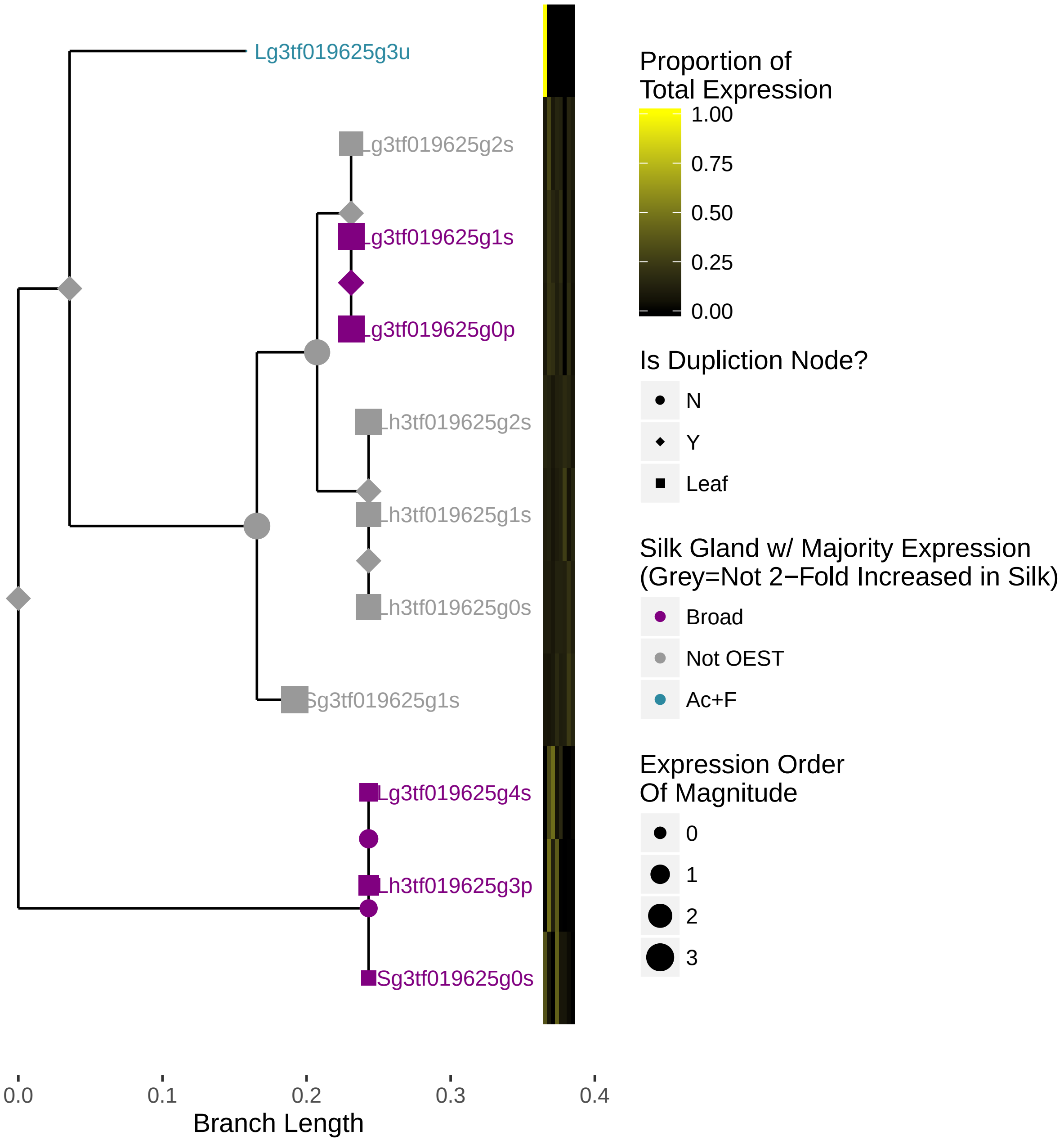
Lg3tf019625g3u
Lg3tf019625g2s
Lg3tf019625g1s
Lg3tf019625g0p
Lh3tf019625g2s
Lh3tf019625g1s
Lh3tf019625g0s
Sg3tf019625g1s
Lg3tf019625g4s
Lh3tf019625g3p
Sg3tf019625g0s
Proportion of
Total Expression
1.00
0.75
0.50
0.25
0.00
Is Dupliction Node?
N
Y
Leaf
Silk Gland w/ Majority Expression
(Grey=Not 2−Fold Increased in Silk)
Broad
Not OEST
Ac+F
Expression Order
Of Magnitude
0
1
2
3
0.0
0.1
0.2
0.3
0.4
Branch Length

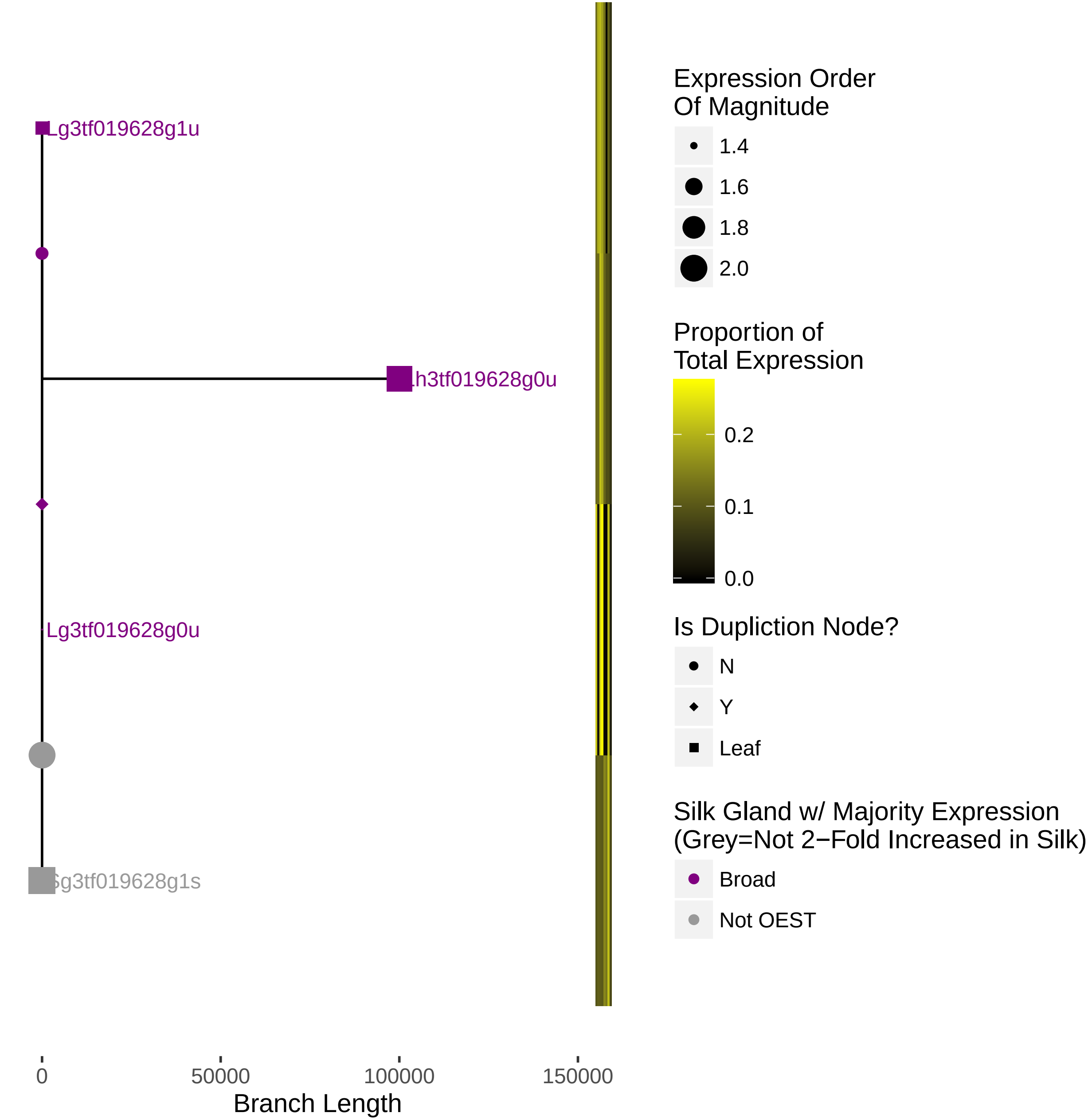

Lg3tf019628g1u
Lh3tf019628g0u
Lg3tf019628g0u
Sg3tf019628g1s
0
50000
100000
150000
Branch Length
Expression Order
Of Magnitude
1.4
1.6
1.8
2.0
Proportion of
Total Expression
0.2
0.1
0.0
Is Dupliction Node?
N
Y
Leaf
Silk Gland w/ Majority Expression
(Grey=Not 2−Fold Increased in Silk)
Broad
Not OEST

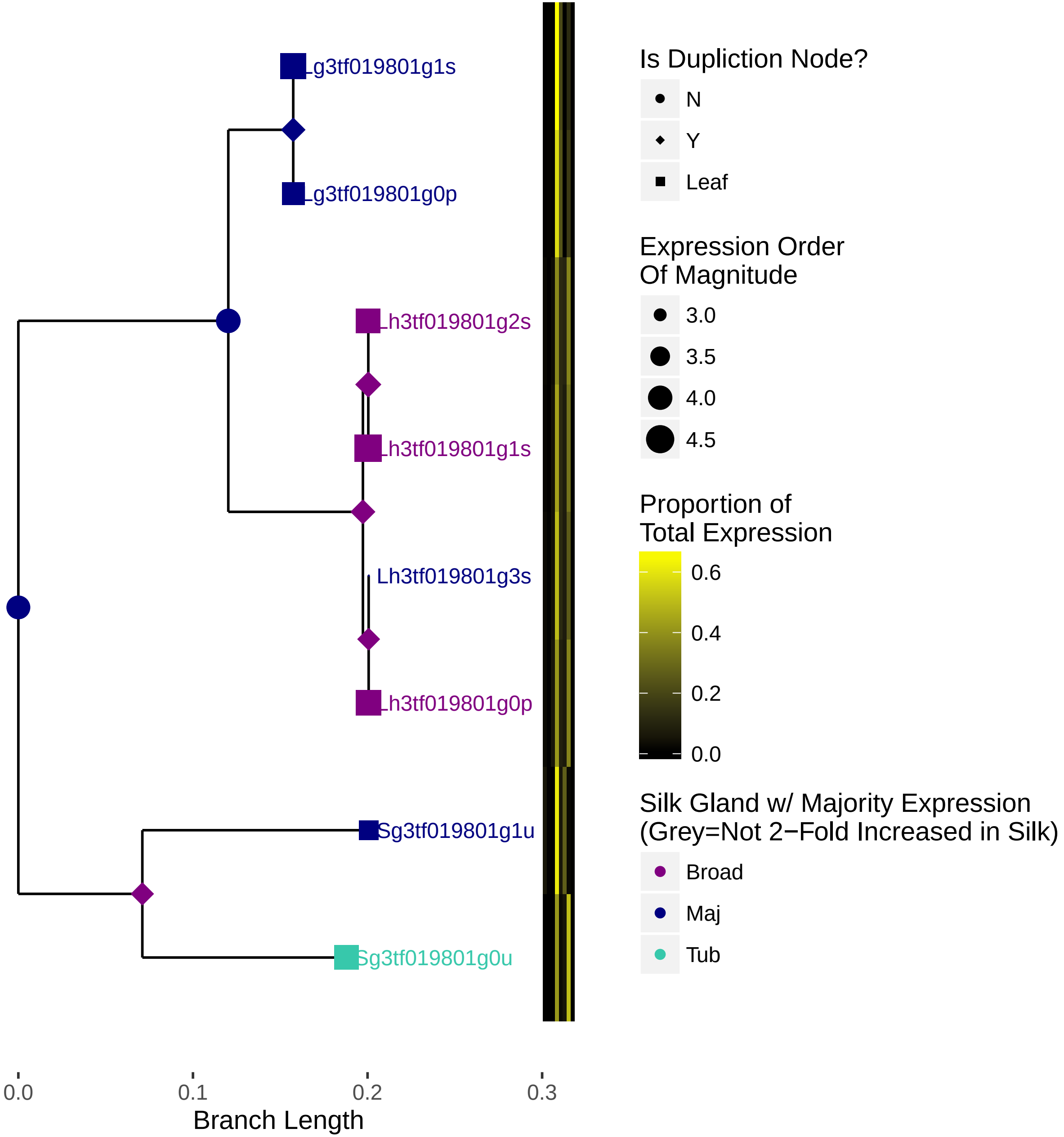

Lg3tf019801g1s
Lg3tf019801g0p
Lh3tf019801g2s
Lh3tf019801g1s
Lh3tf019801g3s
Lh3tf019801g0p
Sg3tf019801g1u
Sg3tf019801g0u
0.0
0.1
0.2
0.3
Branch Length
Is Dupliction Node?
N
Y
Leaf
Expression Order Of Magnitude
3.0
3.5
4.0
4.5
Proportion of Total Expression
0.6
0.4
0.2
0.0
Silk Gland w/ Majority Expression (Grey=Not 2−Fold Increased in Silk)
Broad
Maj
Tub

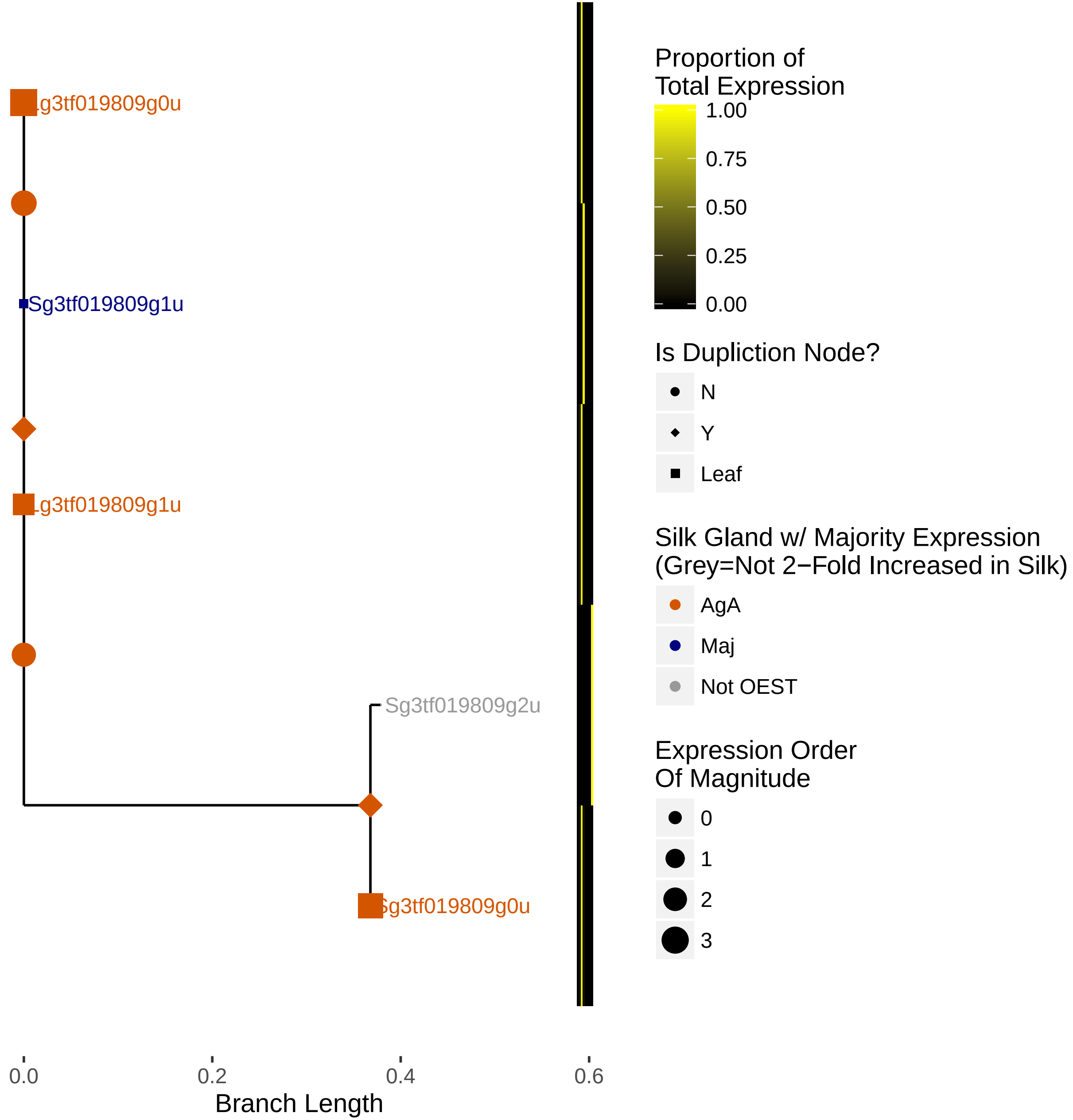

.g3tf019809g0u
Sg3tf019809g1u
.g3tf019809g1u
Sg3tf019809g2u
Sg3tf019809g0u
0.0
0.2
0.4
0.6
Branch Length
Proportion of
Total Expression
1.00
0.75
0.50
0.25
0.00
Is Dupliction Node?
N
Y
Leaf
Silk Gland w/ Majority Expression
(Grey=Not 2−Fold Increased in Silk)
AgA
Maj
Not OEST
Expression Order
Of Magnitude
0
1
2
3

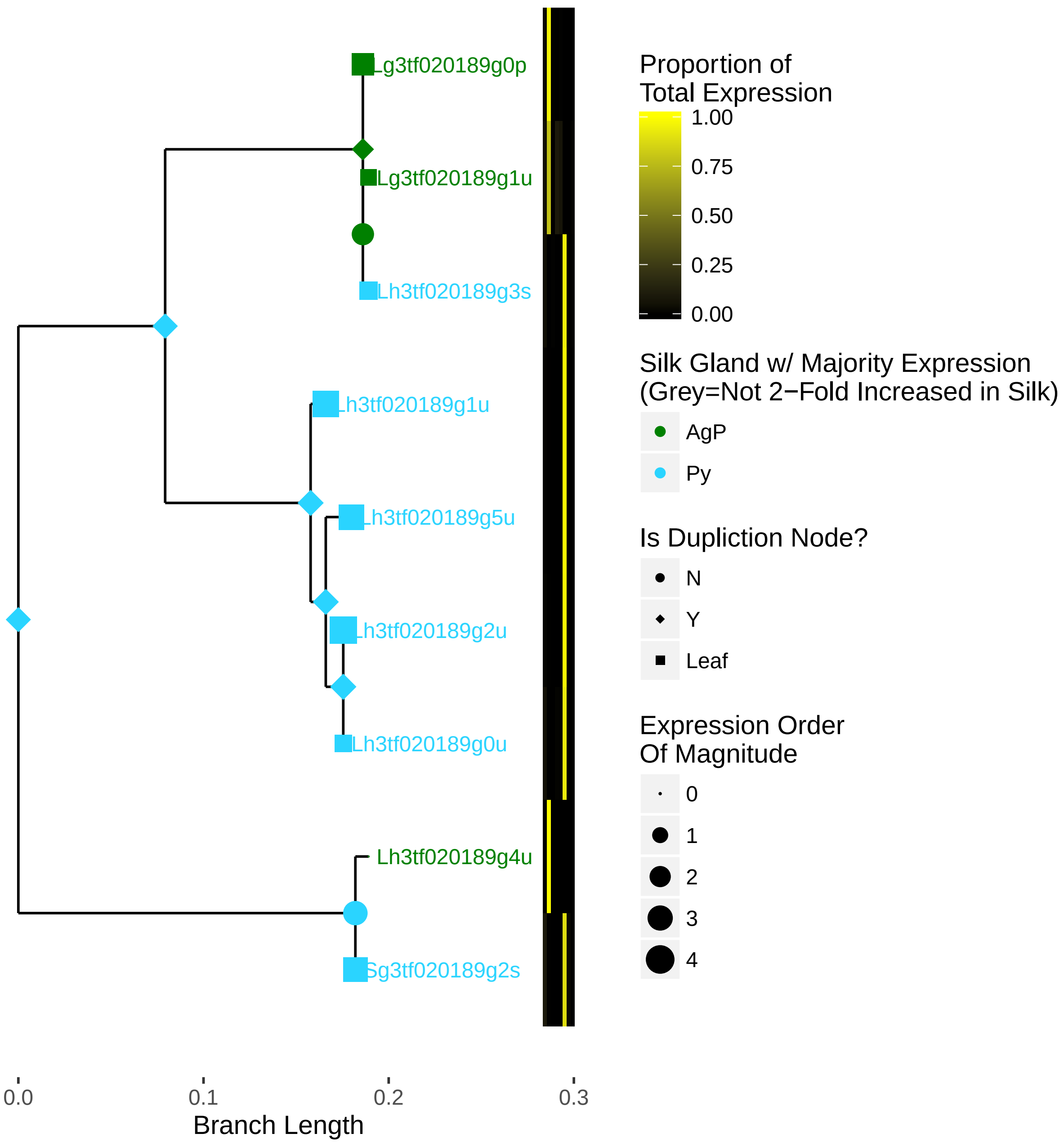
Lg3tf020189g0p
Lg3tf020189g1u
Lh3tf020189g3s
Lh3tf020189g1u
Lh3tf020189g5u
Lh3tf020189g2u
Lh3tf020189g0u
Lh3tf020189g4u
Sg3tf020189g2s
0.0
0.1
0.2
0.3
Branch Length
Proportion of Total Expression
1.00
0.75
0.50
0.25
0.00
Silk Gland w/ Majority Expression (Grey=Not 2−Fold Increased in Silk)
AgP
Py
Is Dupliction Node?
N
Y
Leaf
Expression Order Of Magnitude
0
1
2
3
4

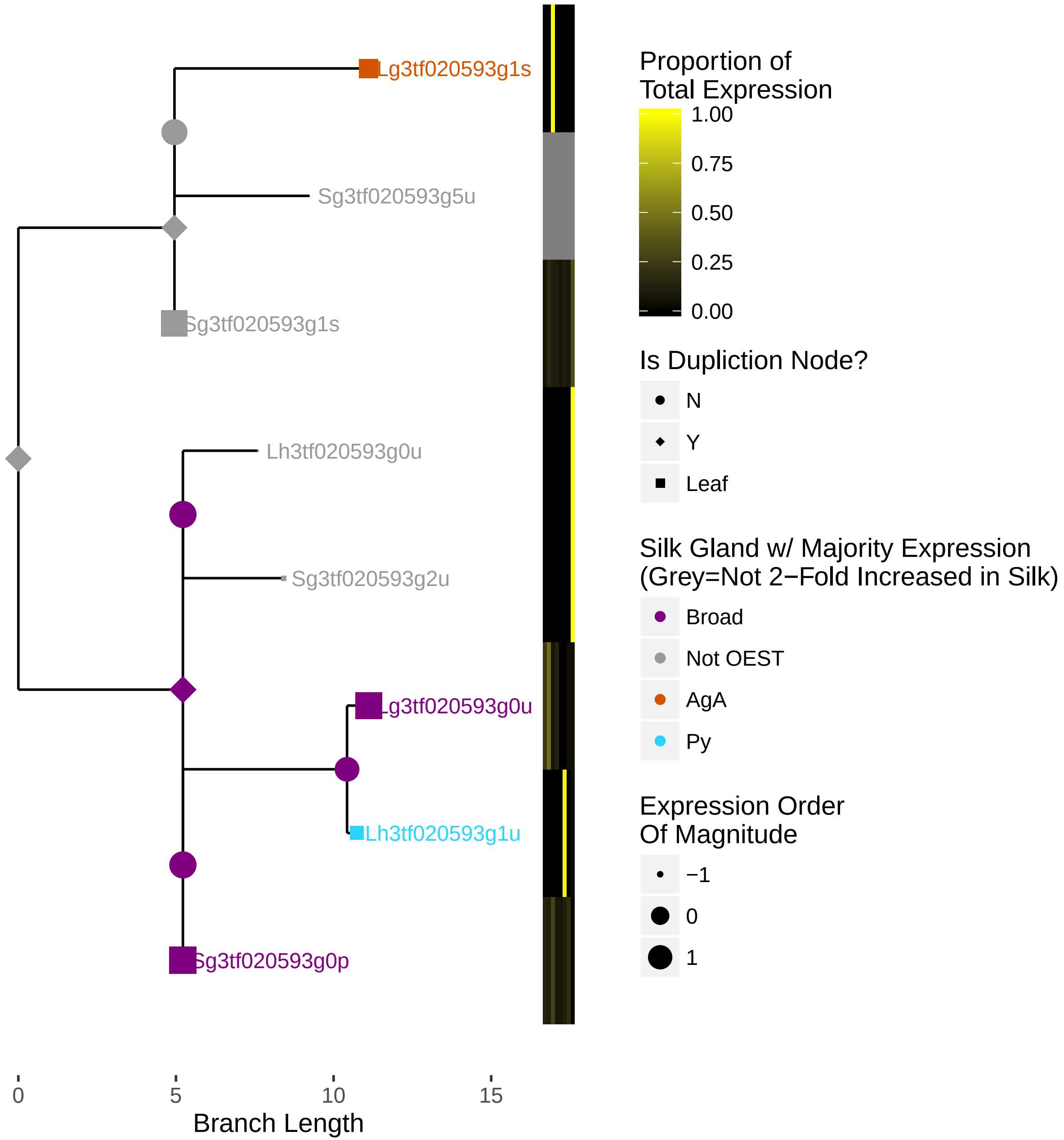

Lg3tf020593g1s
Sg3tf020593g5u
Sg3tf020593g1s
Lh3tf020593g0u
Sg3tf020593g2u
Lg3tf020593g0u
Lh3tf020593g1u
Sg3tf020593g0p
0
5
10
15
Branch Length
Proportion of
Total Expression
1.00
0.75
0.50
0.25
0.00
Is Dupliction Node?
N
Y
Leaf
Silk Gland w/ Majority Expression
(Grey=Not 2−Fold Increased in Silk)
Broad
Not OEST
AgA
Py
Expression Order
Of Magnitude
−1
0
1

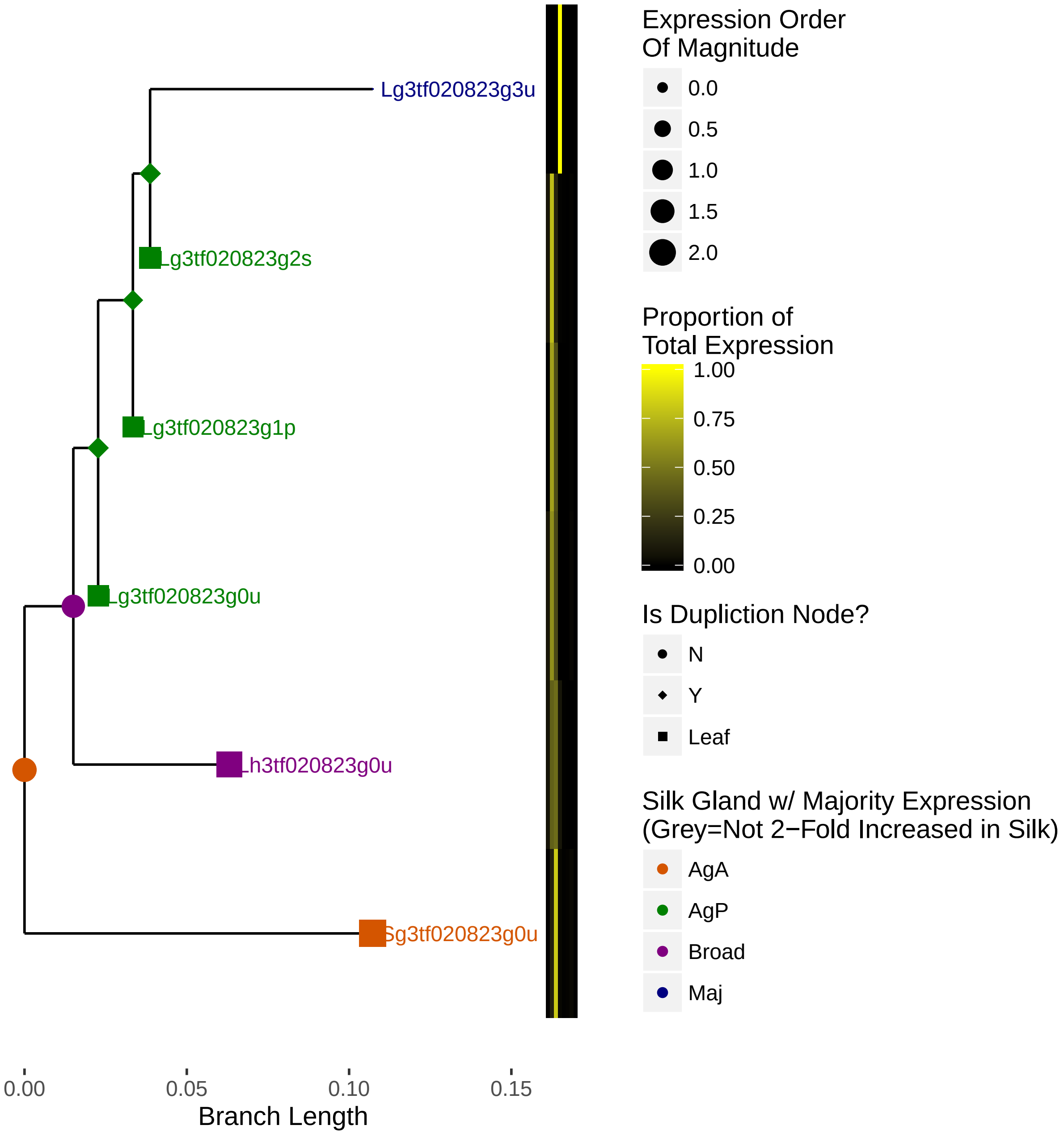

Lg3tf020823g3u
Lg3tf020823g2s
Lg3tf020823g1p
Lg3tf020823g0u
Lh3tf020823g0u
Sg3tf020823g0u
0.00
0.05
0.10
0.15
Branch Length
Expression Order
Of Magnitude
0.0
0.5
1.0
1.5
2.0
Proportion of
Total Expression
1.00
0.75
0.50
0.25
0.00
Is Dupliction Node?
N
Y
Leaf
Silk Gland w/ Majority Expression
(Grey=Not 2−Fold Increased in Silk)
AgA
AgP
Broad
Maj

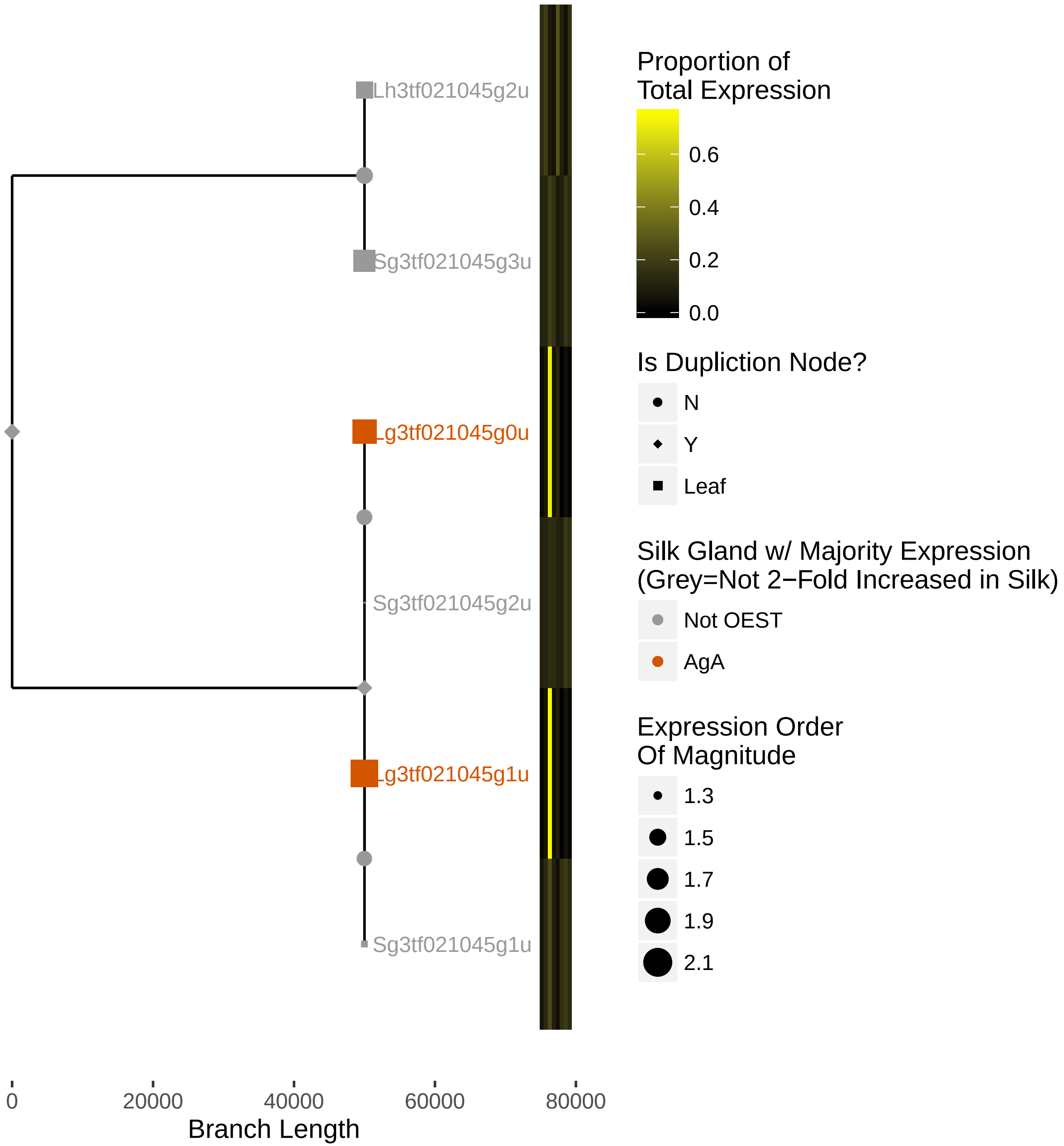

Lh3tf021045g2u
Sg3tf021045g3u
Lg3tf021045g0u
Sg3tf021045g2u
Lg3tf021045g1u
Sg3tf021045g1u
0
20000
40000
60000
80000
Branch Length
Proportion of Total Expression
0.6
0.4
0.2
0.0
Is Dupliction Node?
N
Y
Leaf
Silk Gland w/ Majority Expression (Grey=Not 2−Fold Increased in Silk)
Not OEST
AgA
Expression Order Of Magnitude
1.3
1.5
1.7
1.9
2.1

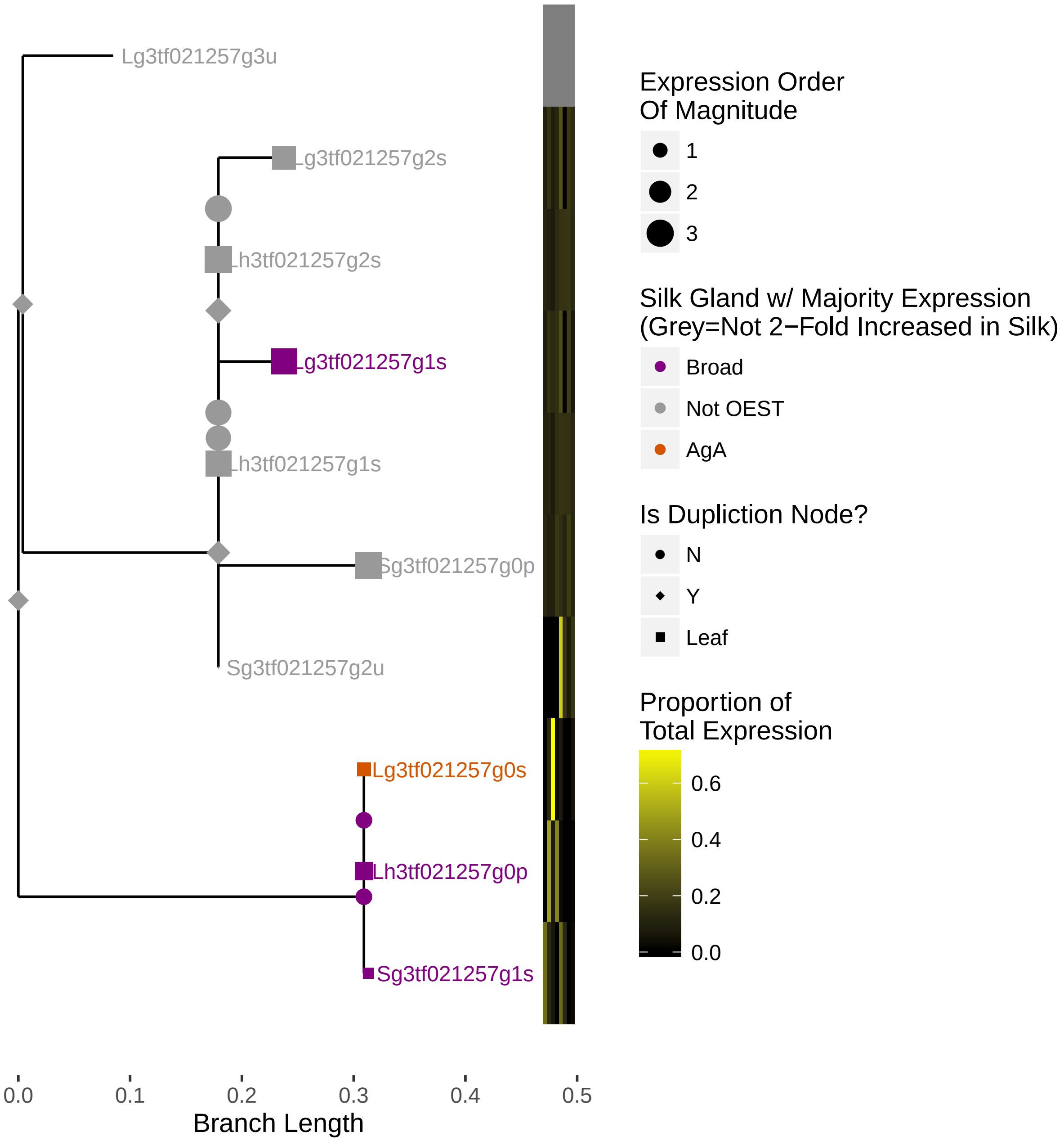
Lg3tf021257g3u
Lg3tf021257g2s
Lh3tf021257g2s
Lg3tf021257g1s
Lh3tf021257g1s
Sg3tf021257g0p
Sg3tf021257g2u
Lg3tf021257g0s
Lh3tf021257g0p
Sg3tf021257g1s
0.0
0.1
0.2
0.3
0.4
0.5
Branch Length
Expression Order Of Magnitude
1
2
3
Silk Gland w/ Majority Expression (Grey=Not 2−Fold Increased in Silk)
Broad
Not OEST
AgA
Is Dupliction Node?
N
Y
Leaf
Proportion of Total Expression
0.6
0.4
0.2
0.0

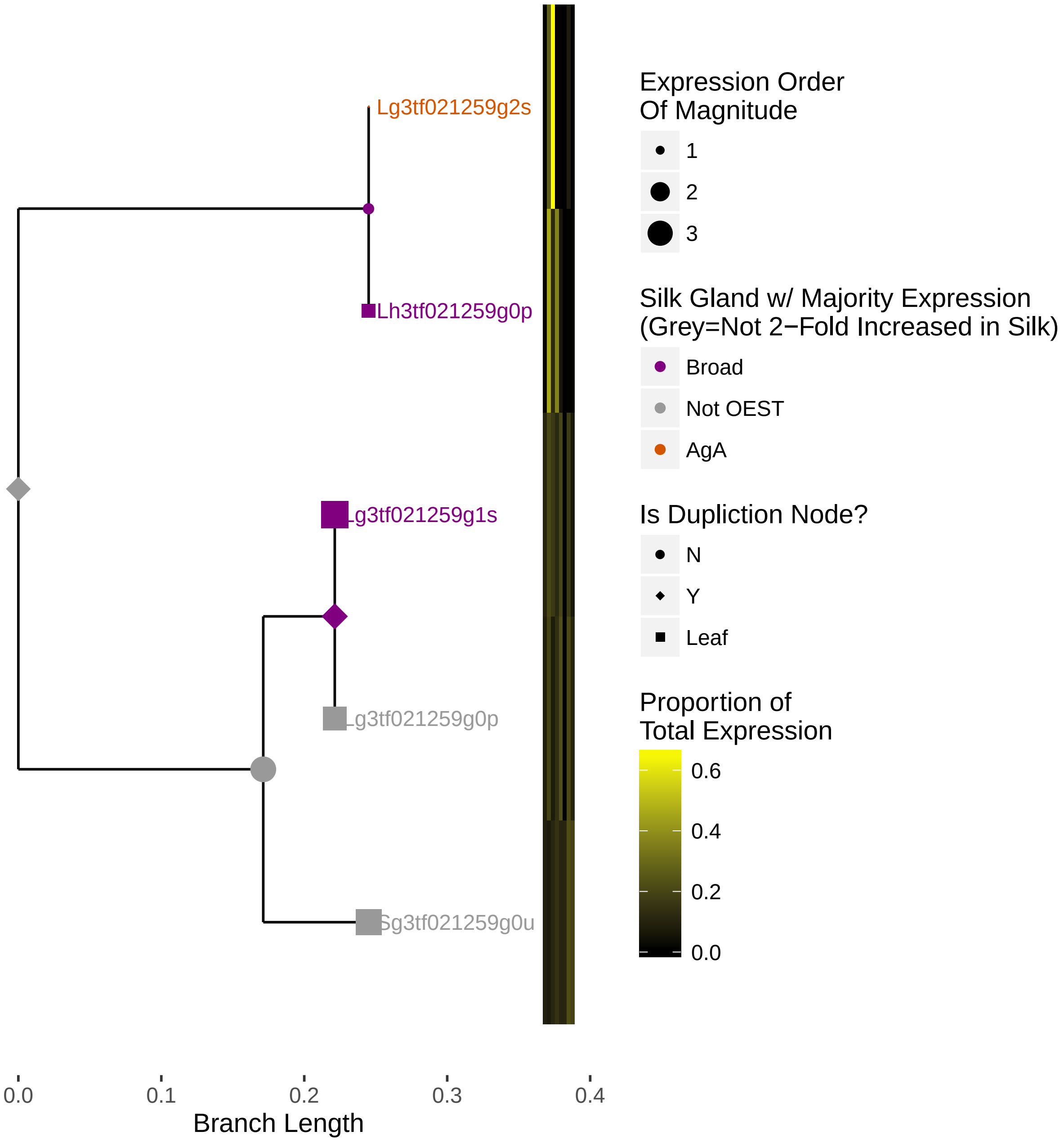
Lg3tf021259g2s
Lh3tf021259g0p
Lg3tf021259g1s
Lg3tf021259g0p
Sg3tf021259g0u
0.0
0.1
0.2
0.3
0.4
Branch Length
Expression Order
Of Magnitude
1
2
3
Silk Gland w/ Majority Expression
(Grey=Not 2−Fold Increased in Silk)
Broad
Not OEST
AgA
Is Dupliction Node?
N
Y
Leaf
Proportion of
Total Expression
0.6
0.4
0.2
0.0

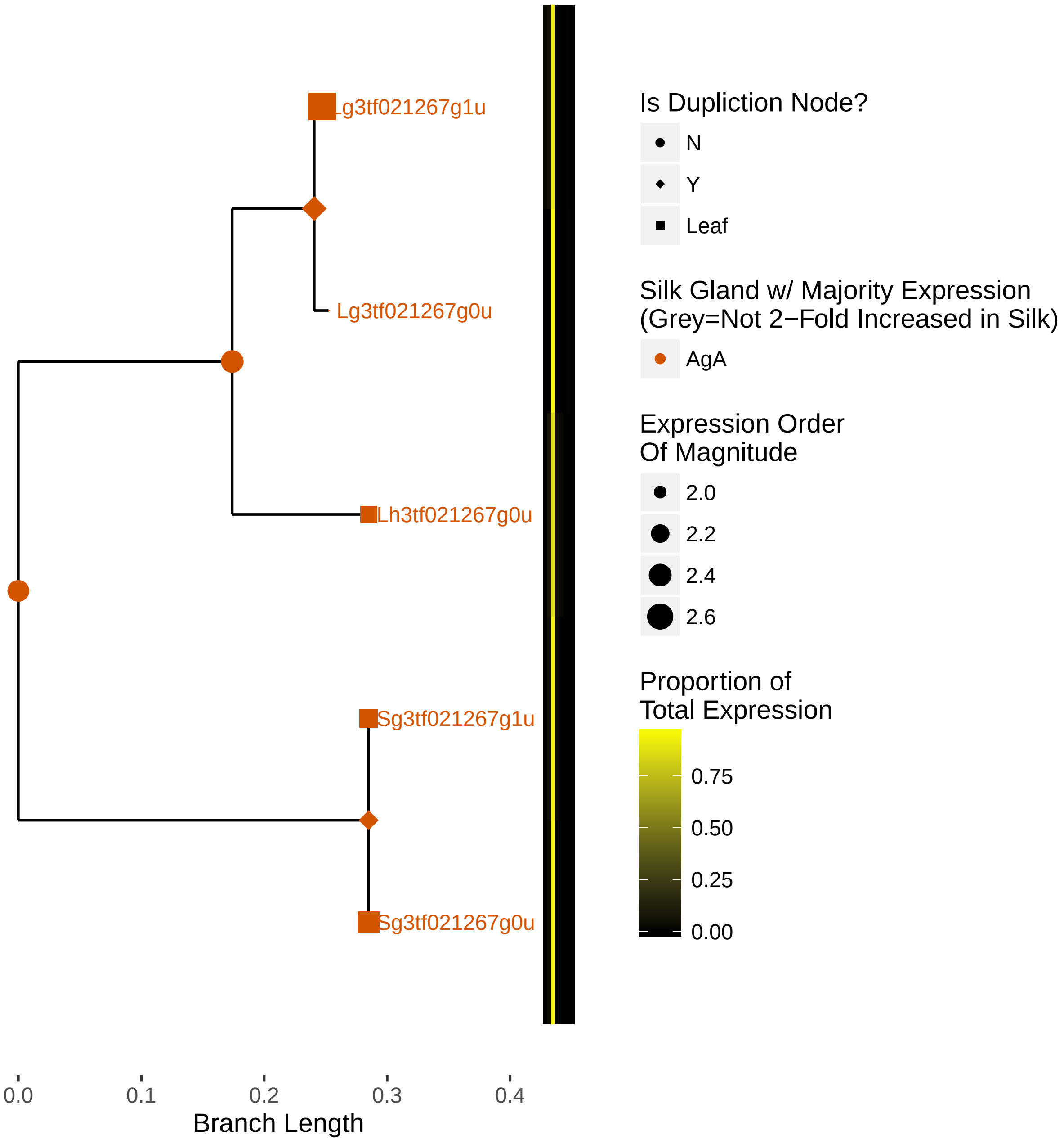

Lg3tf021267g1u
Lg3tf021267g0u
Lh3tf021267g0u
Sg3tf021267g1u
Sg3tf021267g0u
0.0
0.1
0.2
0.3
0.4
Branch Length
Is Dupliction Node?
N
Y
Leaf
Silk Gland w/ Majority Expression
(Grey=Not 2−Fold Increased in Silk)
AgA
Expression Order
Of Magnitude
2.0
2.2
2.4
2.6
Proportion of
Total Expression
0.75
0.50
0.25
0.00

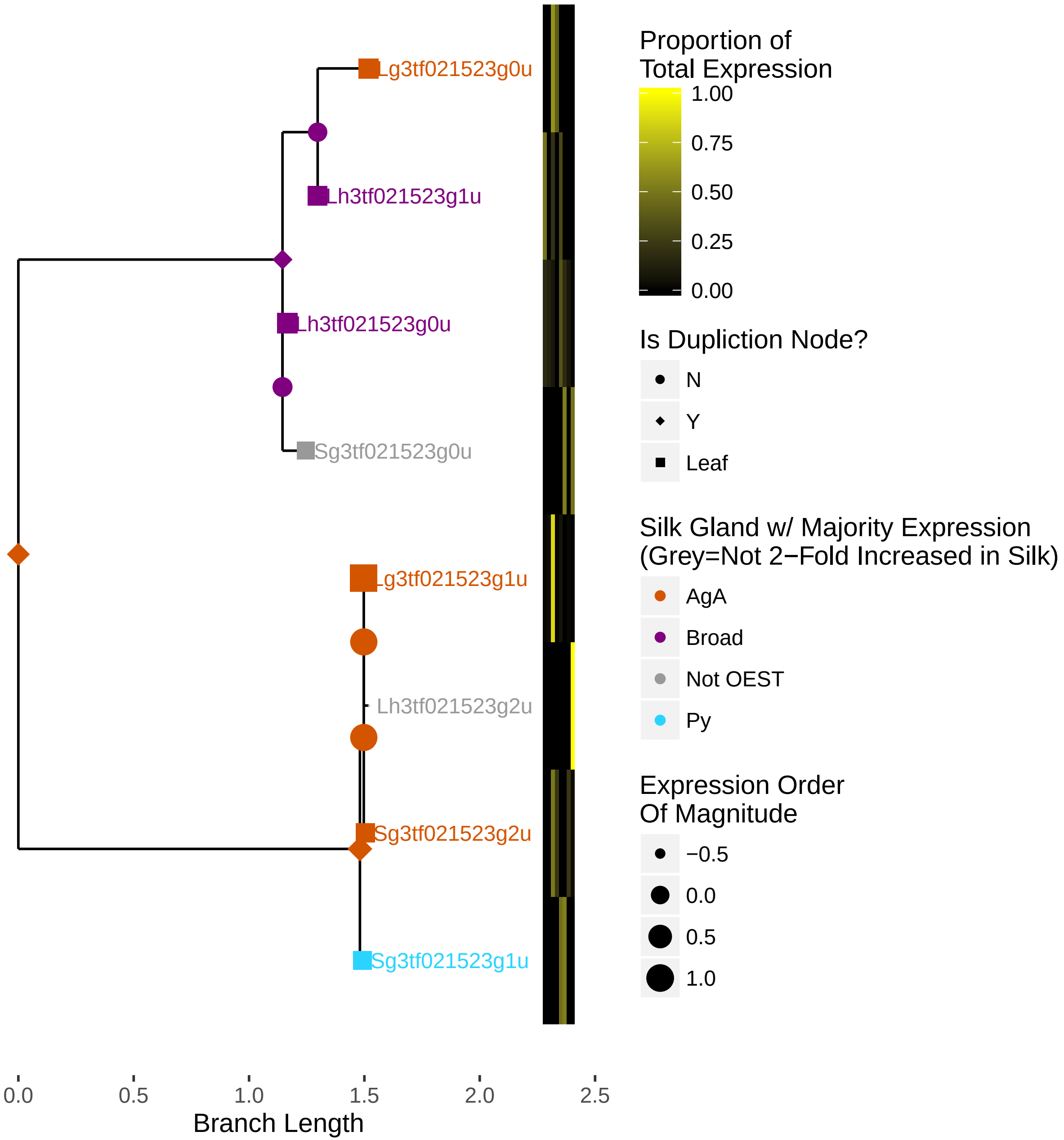

Lg3tf021523g0u
Lh3tf021523g1u
Lh3tf021523g0u
Sg3tf021523g0u
Lg3tf021523g1u
Lh3tf021523g2u
Sg3tf021523g2u
Sg3tf021523g1u
0.0
0.5
1.0
1.5
2.0
2.5
Branch Length
Proportion of Total Expression
1.00
0.75
0.50
0.25
0.00
Is Dupliction Node?
N
Y
Leaf
Silk Gland w/ Majority Expression (Grey=Not 2−Fold Increased in Silk)
AgA
Broad
Not OEST
Py
Expression Order Of Magnitude
−0.5
0.0
0.5
1.0

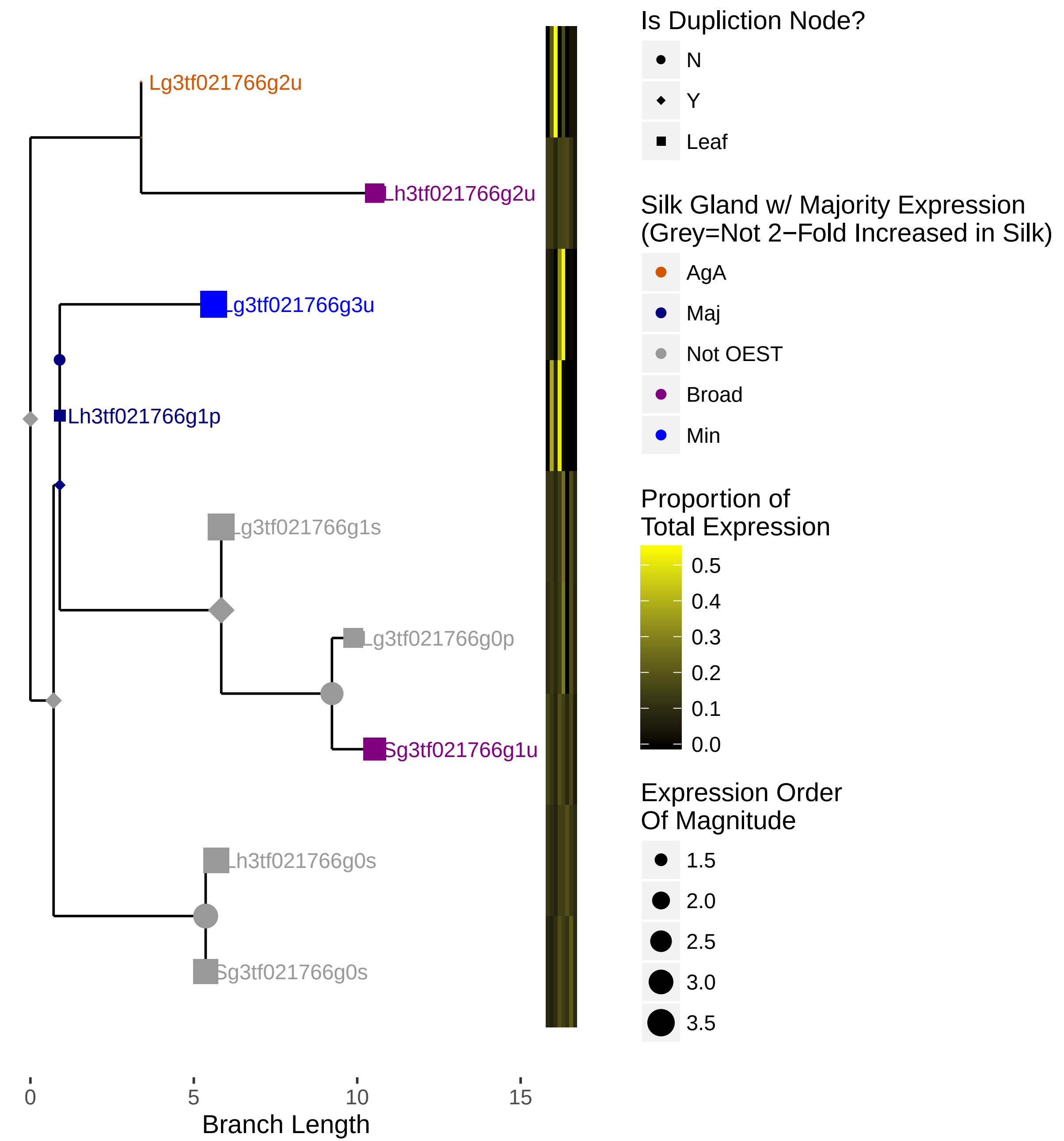

Lg3tf021766g2u
Lh3tf021766g2u
Lg3tf021766g3u
Lh3tf021766g1p
Lg3tf021766g1s
Lg3tf021766g0p
Sg3tf021766g1u
Lh3tf021766g0s
Sg3tf021766g0s
0
5
10
15
Branch Length
Is Dupliction Node?
N
Y
Leaf
Silk Gland w/ Majority Expression
(Grey=Not 2−Fold Increased in Silk)
AgA
Maj
Not OEST
Broad
Min
Proportion of
Total Expression
0.5
0.4
0.3
0.2
0.1
0.0
Expression Order
Of Magnitude
1.5
2.0
2.5
3.0
3.5

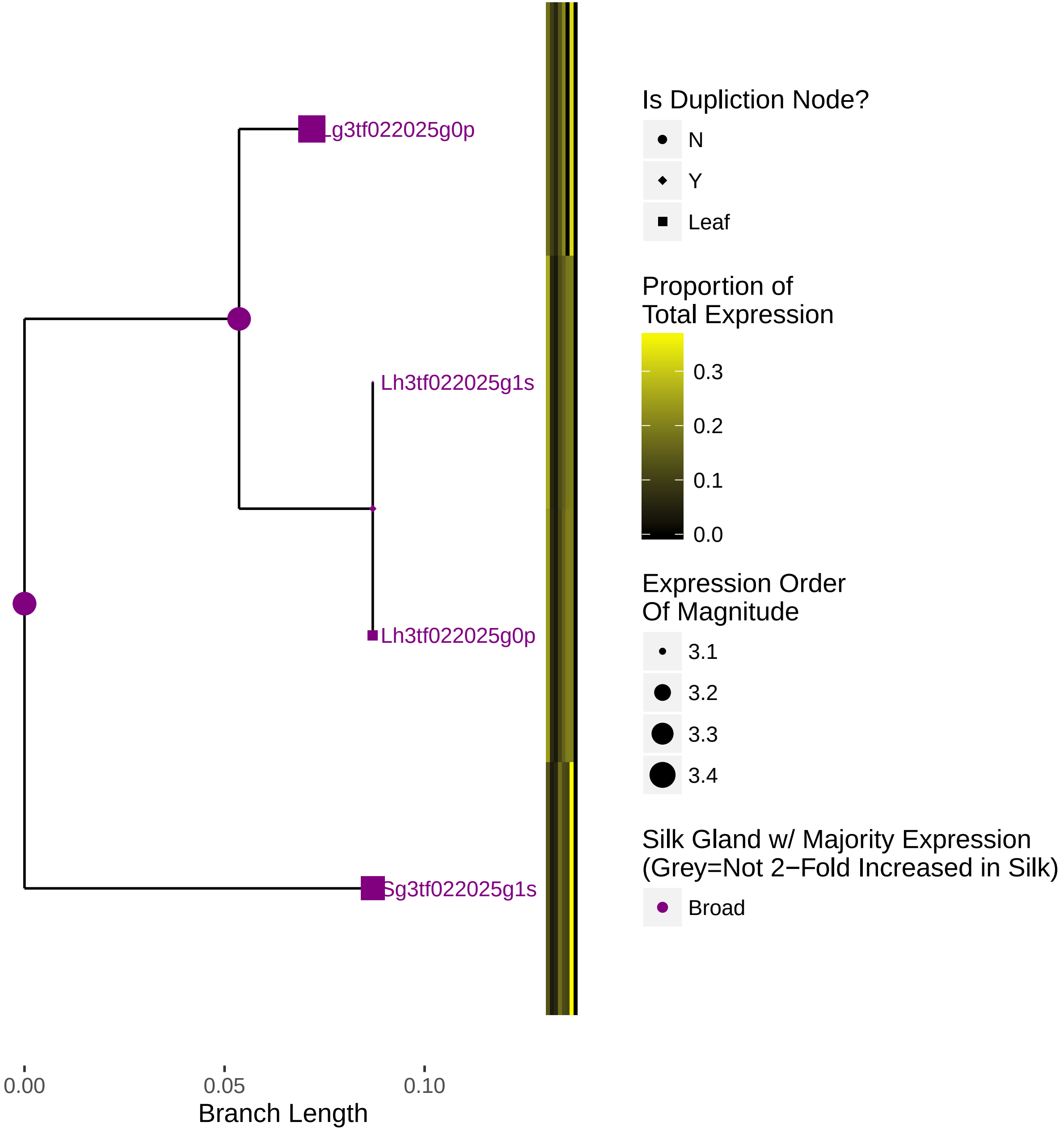

Lg3tf022025g0p
Lh3tf022025g1s
Lh3tf022025g0p
Sg3tf022025g1s
0.00
0.05
0.10
Branch Length
Is Dupliction Node?
N
Y
Leaf
Proportion of
Total Expression
0.3
0.2
0.1
0.0
Expression Order
Of Magnitude
3.1
3.2
3.3
3.4
Silk Gland w/ Majority Expression
(Grey=Not 2−Fold Increased in Silk)
Broad

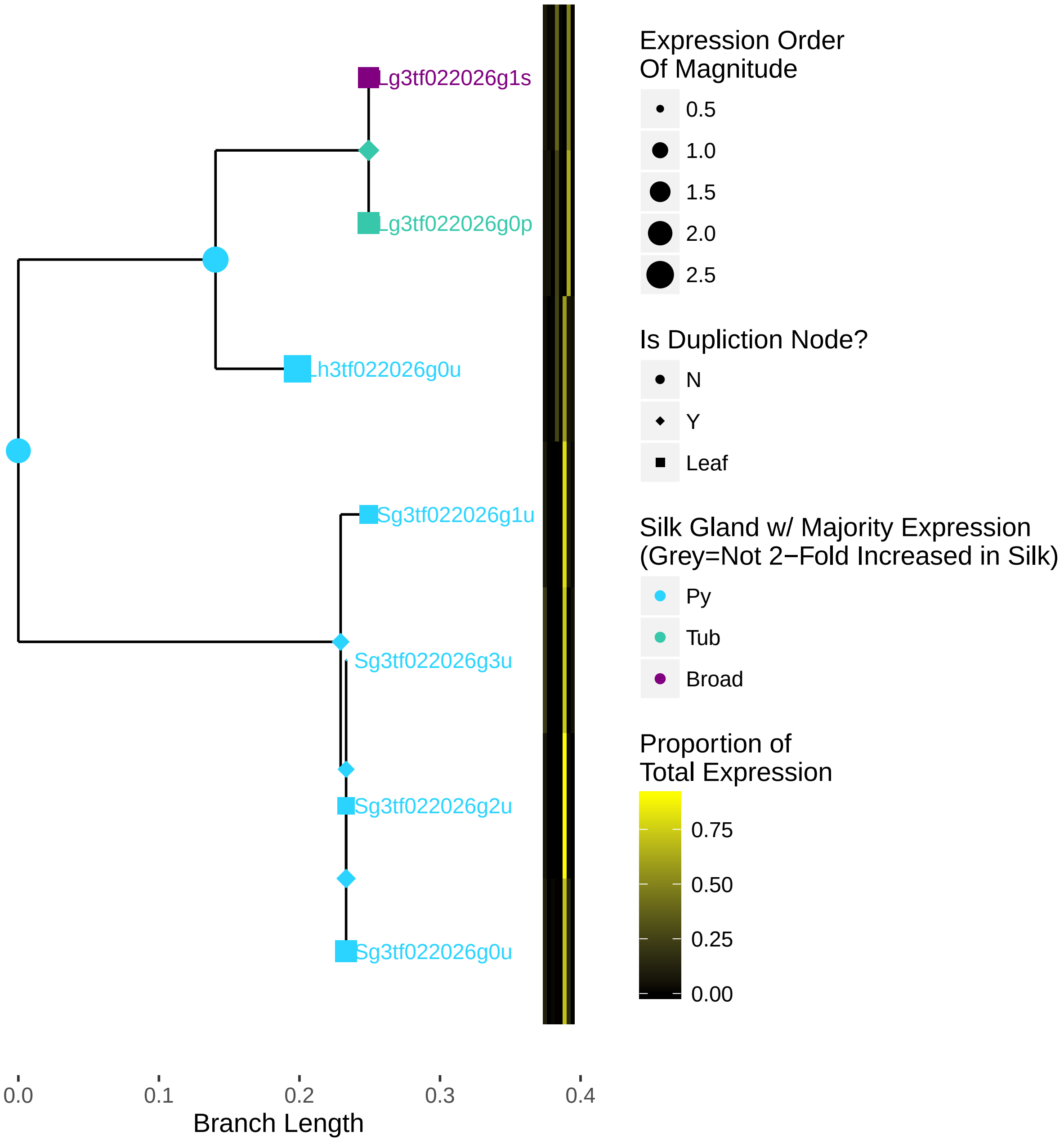
Lg3tf022026g1s
Lg3tf022026g0p
Lh3tf022026g0u
Sg3tf022026g1u
Sg3tf022026g3u
Sg3tf022026g2u
Sg3tf022026g0u
0.0
0.1
0.2
0.3
0.4
Branch Length
Expression Order Of Magnitude
0.5
1.0
1.5
2.0
2.5
Is Dupliction Node?
N
Y
Leaf
Silk Gland w/ Majority Expression (Grey=Not 2−Fold Increased in Silk)
Py
Tub
Broad
Proportion of Total Expression
0.75
0.50
0.25
0.00

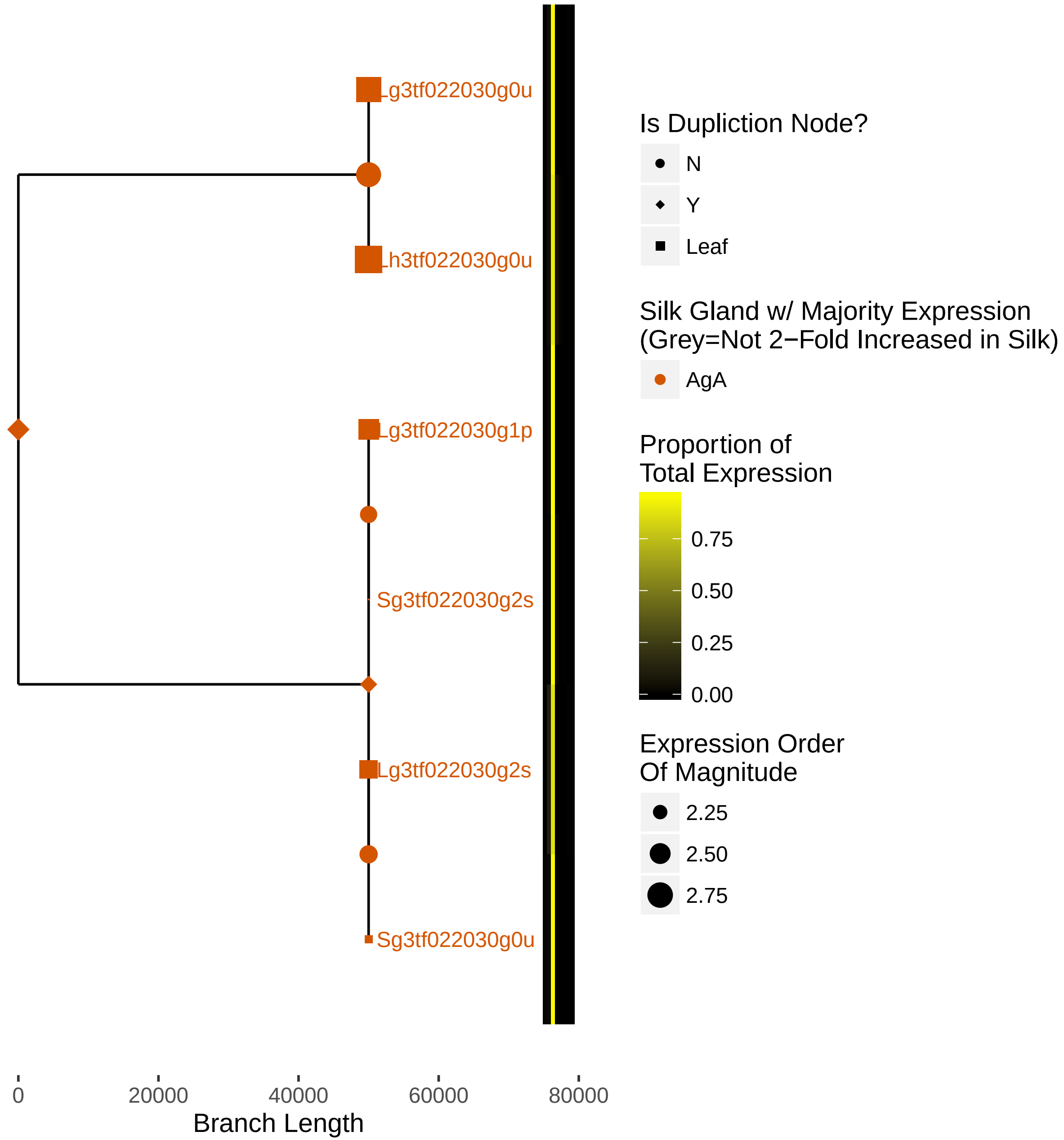

Lg3tf022030g0u
Lh3tf022030g0u
Lg3tf022030g1p
Sg3tf022030g2s
Lg3tf022030g2s
Sg3tf022030g0u
Is Dupliction Node?
N
Y
Leaf
Silk Gland w/ Majority Expression
(Grey=Not 2−Fold Increased in Silk)
AgA
Proportion of
Total Expression
0.75
0.50
0.25
0.00
Expression Order
Of Magnitude
2.25
2.50
2.75
0
20000
40000
60000
80000
Branch Length

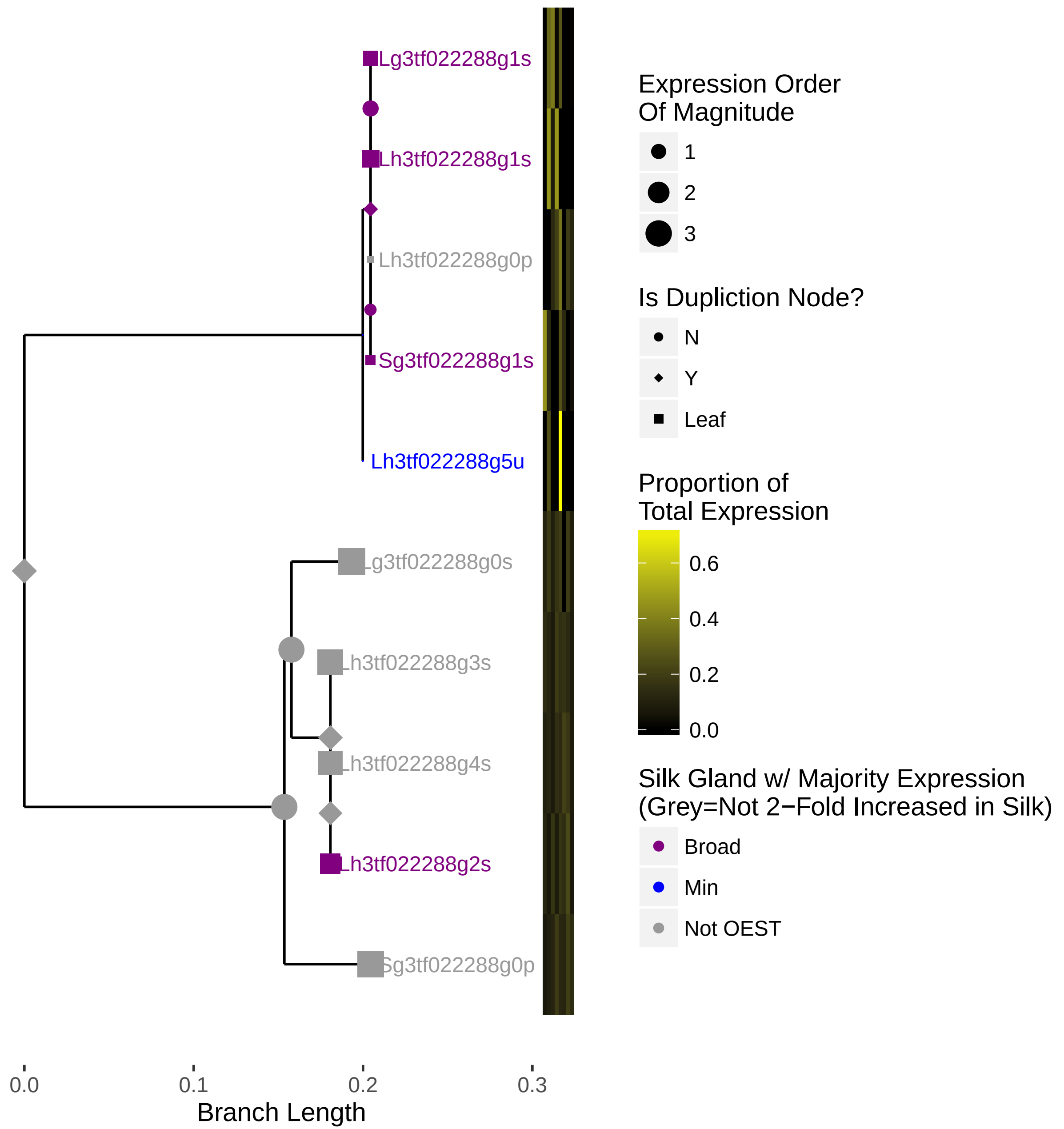

Lg3tf022288g1s
Lh3tf022288g1s
Lh3tf022288g0p
Sg3tf022288g1s
Lh3tf022288g5u
Lg3tf022288g0s
Lh3tf022288g3s
Lh3tf022288g4s
Lh3tf022288g2s
Sg3tf022288g0p
0.0
0.1
0.2
0.3
Branch Length
Expression Order Of Magnitude
1
2
3
Is Dupliction Node?
N
Y
Leaf
Proportion of Total Expression
0.6
0.4
0.2
0.0
Silk Gland w/ Majority Expression (Grey=Not 2−Fold Increased in Silk)
Broad
Min
Not OEST

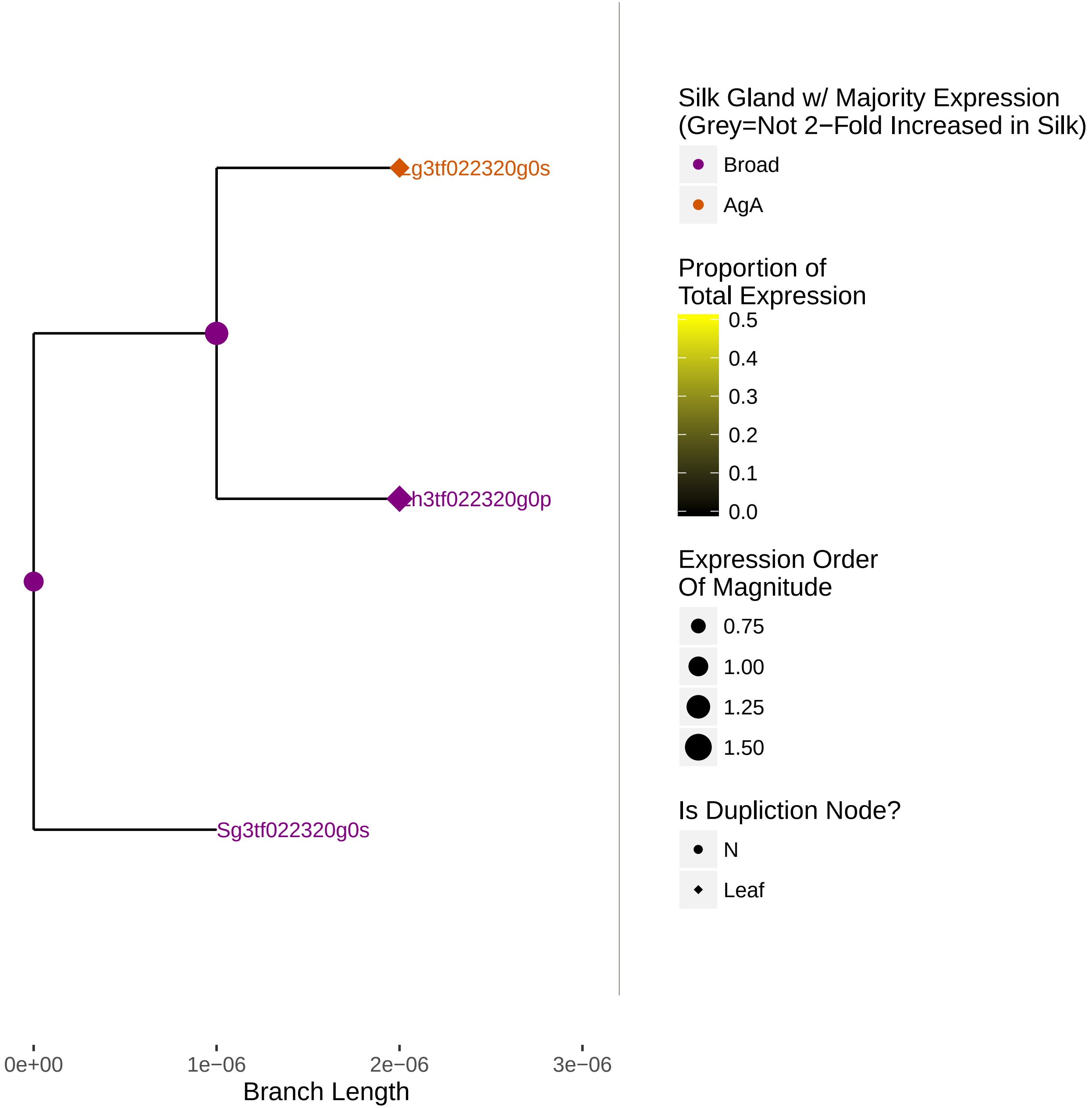

g3tf022320g0s
h3tf022320g0p
Sg3tf022320g0s
0e+00
1e−06
2e−06
3e−06
Branch Length
Silk Gland w/ Majority Expression
(Grey=Not 2−Fold Increased in Silk)
Broad
AgA
Proportion of
Total Expression
0.5
0.4
0.3
0.2
0.1
0.0
Expression Order
Of Magnitude
0.75
1.00
1.25
1.50
Is Dupliction Node?
N
Leaf

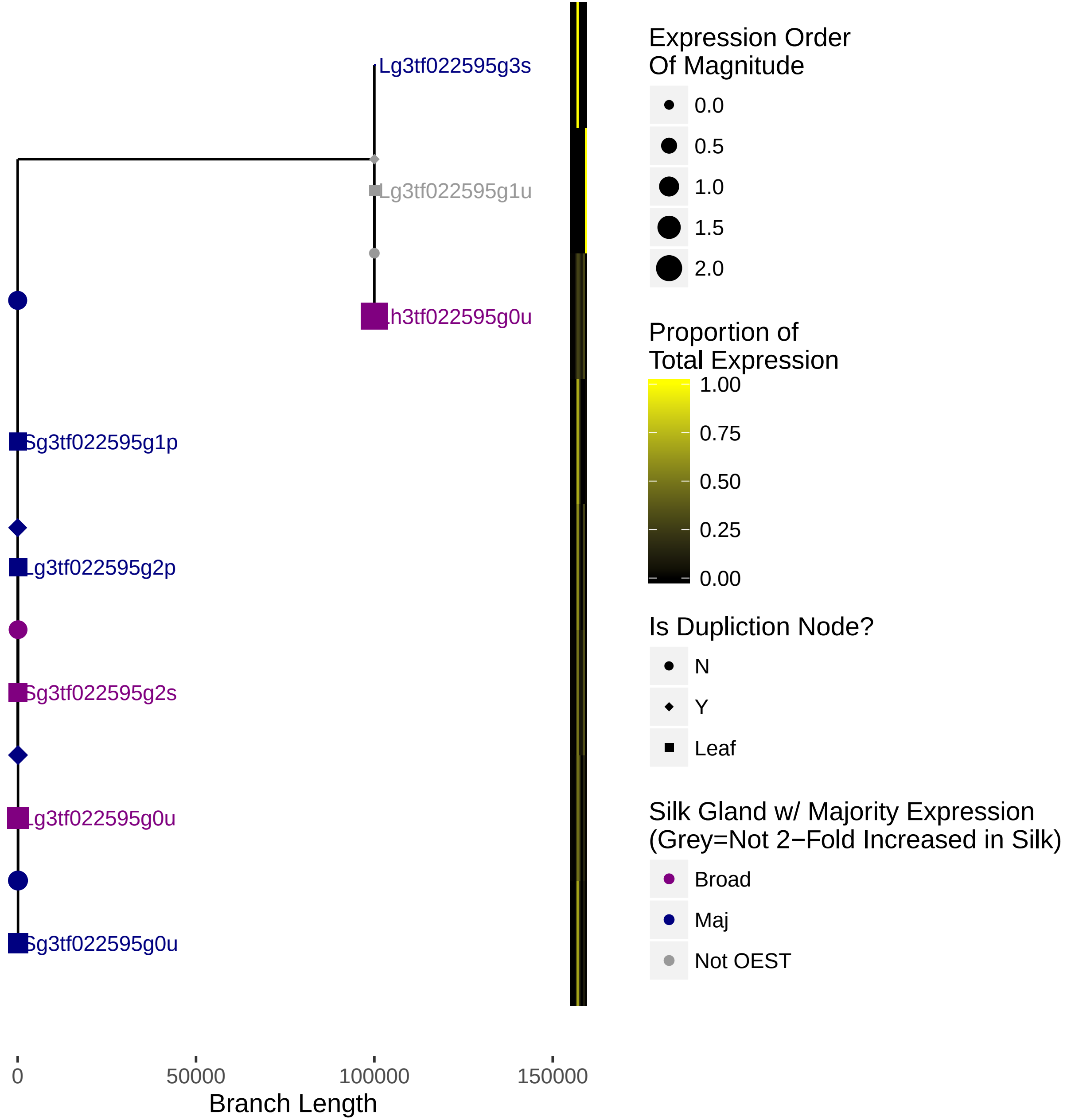

Lg3tf022595g3s
Lg3tf022595g1u
Lh3tf022595g0u
Sg3tf022595g1p
Lg3tf022595g2p
Sg3tf022595g2s
Lg3tf022595g0u
Sg3tf022595g0u
0
50000
100000
150000
Branch Length
Expression Order
Of Magnitude
0.0
0.5
1.0
1.5
2.0
Proportion of
Total Expression
1.00
0.75
0.50
0.25
0.00
Is Dupliction Node?
N
Y
Leaf
Silk Gland w/ Majority Expression
(Grey=Not 2−Fold Increased in Silk)
Broad
Maj
Not OEST

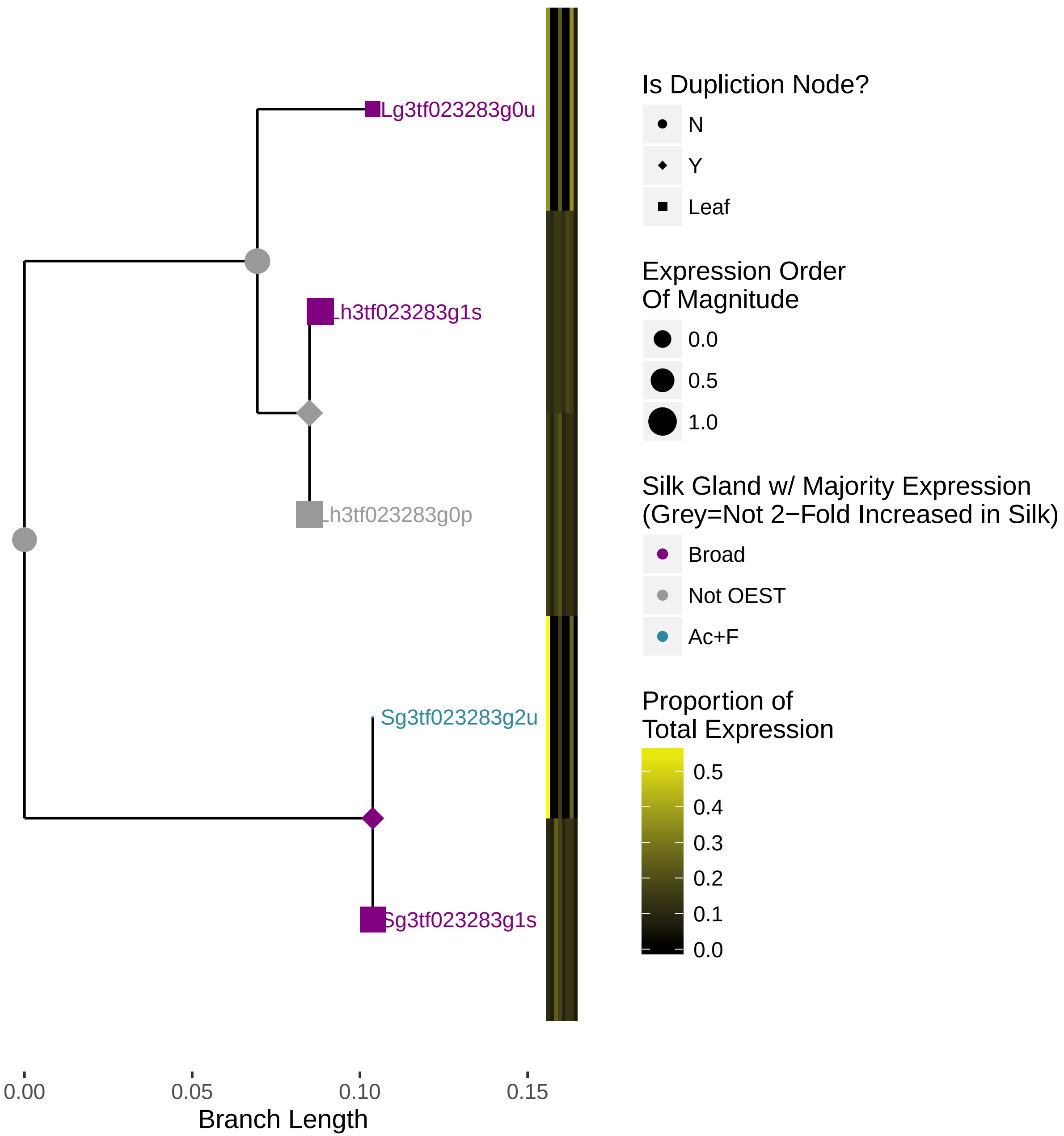

Lg3tf023283g0u
Lh3tf023283g1s
Lh3tf023283g0p
Sg3tf023283g2u
Sg3tf023283g1s
0.00
0.05
0.10
0.15
Branch Length
Is Dupliction Node?
N
Y
Leaf
Expression Order Of Magnitude
0.0
0.5
1.0
Silk Gland w/ Majority Expression (Grey=Not 2−Fold Increased in Silk)
Broad
Not OEST
Ac+F
Proportion of Total Expression
0.5
0.4
0.3
0.2
0.1
0.0

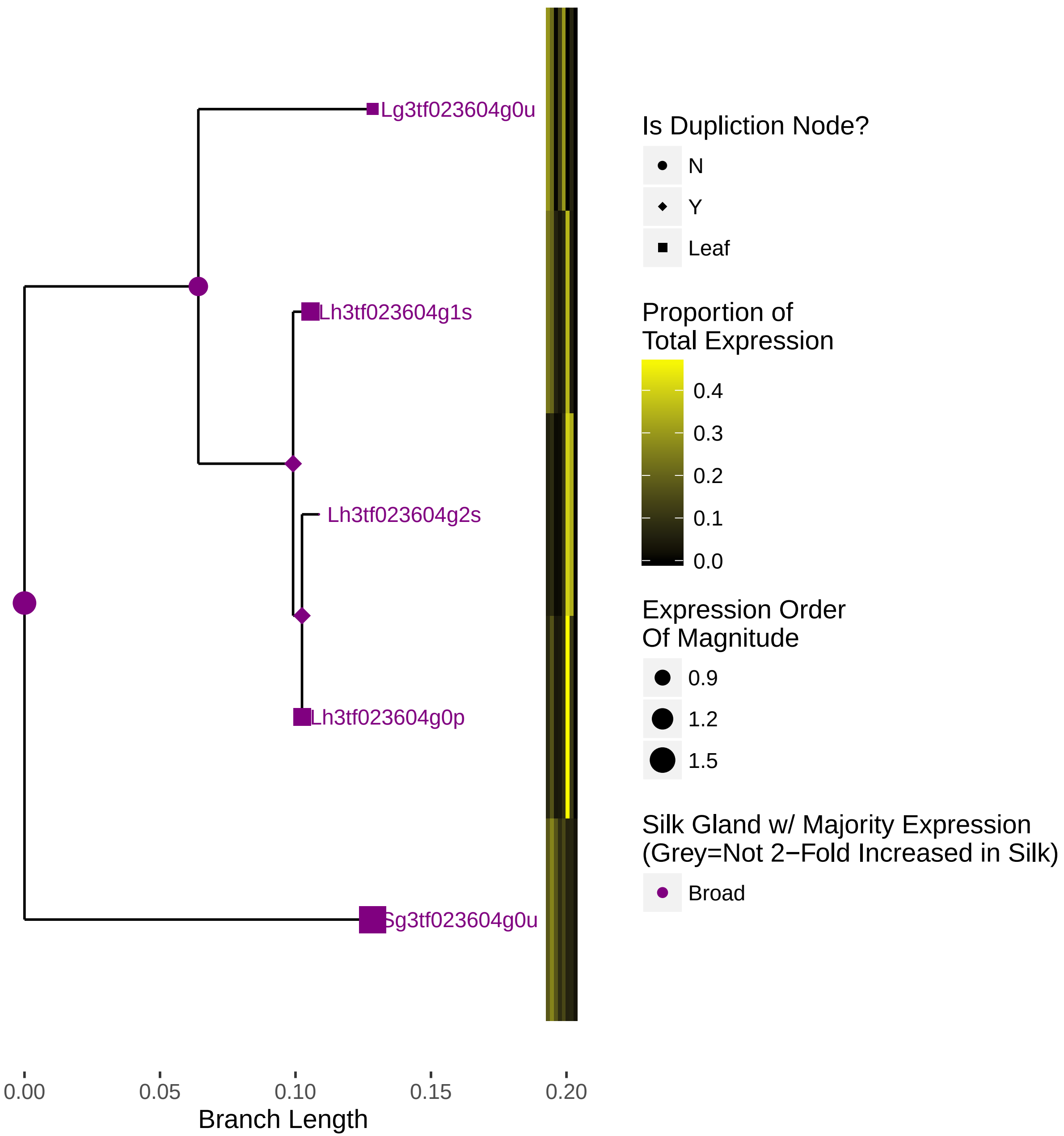
Lg3tf023604g0u
Lh3tf023604g1s
Lh3tf023604g2s
Lh3tf023604g0p
Sg3tf023604g0u
0.00
0.05
0.10
0.15
0.20
Branch Length
Is Dupliction Node?
N
Y
Leaf
Proportion of
Total Expression
0.4
0.3
0.2
0.1
0.0
Expression Order
Of Magnitude
0.9
1.2
1.5
Silk Gland w/ Majority Expression
(Grey=Not 2−Fold Increased in Silk)
Broad

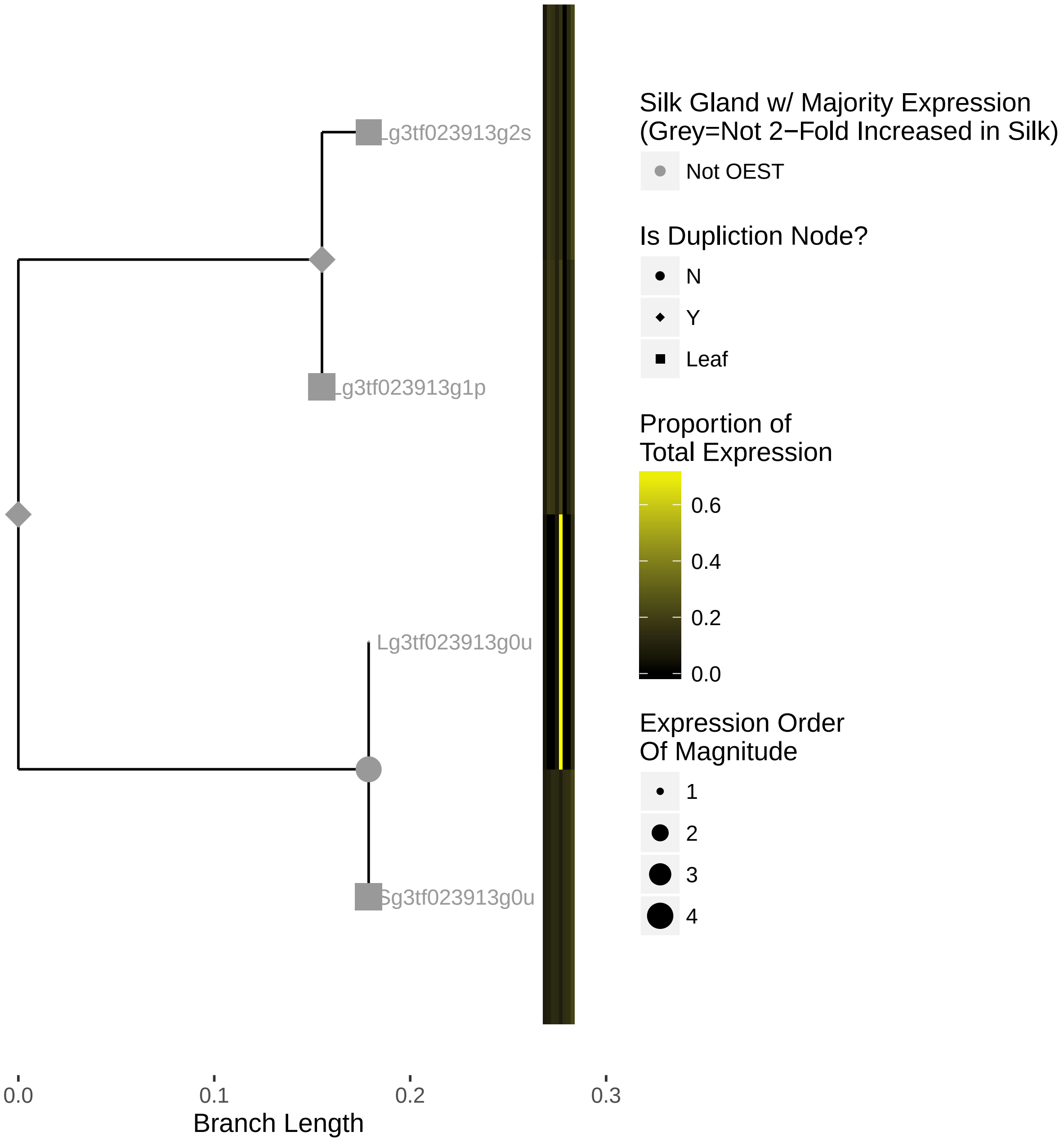

Lg3tf023913g2s
Lg3tf023913g1p
Lg3tf023913g0u
Sg3tf023913g0u
0.0
0.1
0.2
0.3
Branch Length
Silk Gland w/ Majority Expression
(Grey=Not 2−Fold Increased in Silk)
Not OEST
Is Dupliction Node?
N
Y
Leaf
Proportion of
Total Expression
0.6
0.4
0.2
0.0
Expression Order
Of Magnitude
1
2
3
4

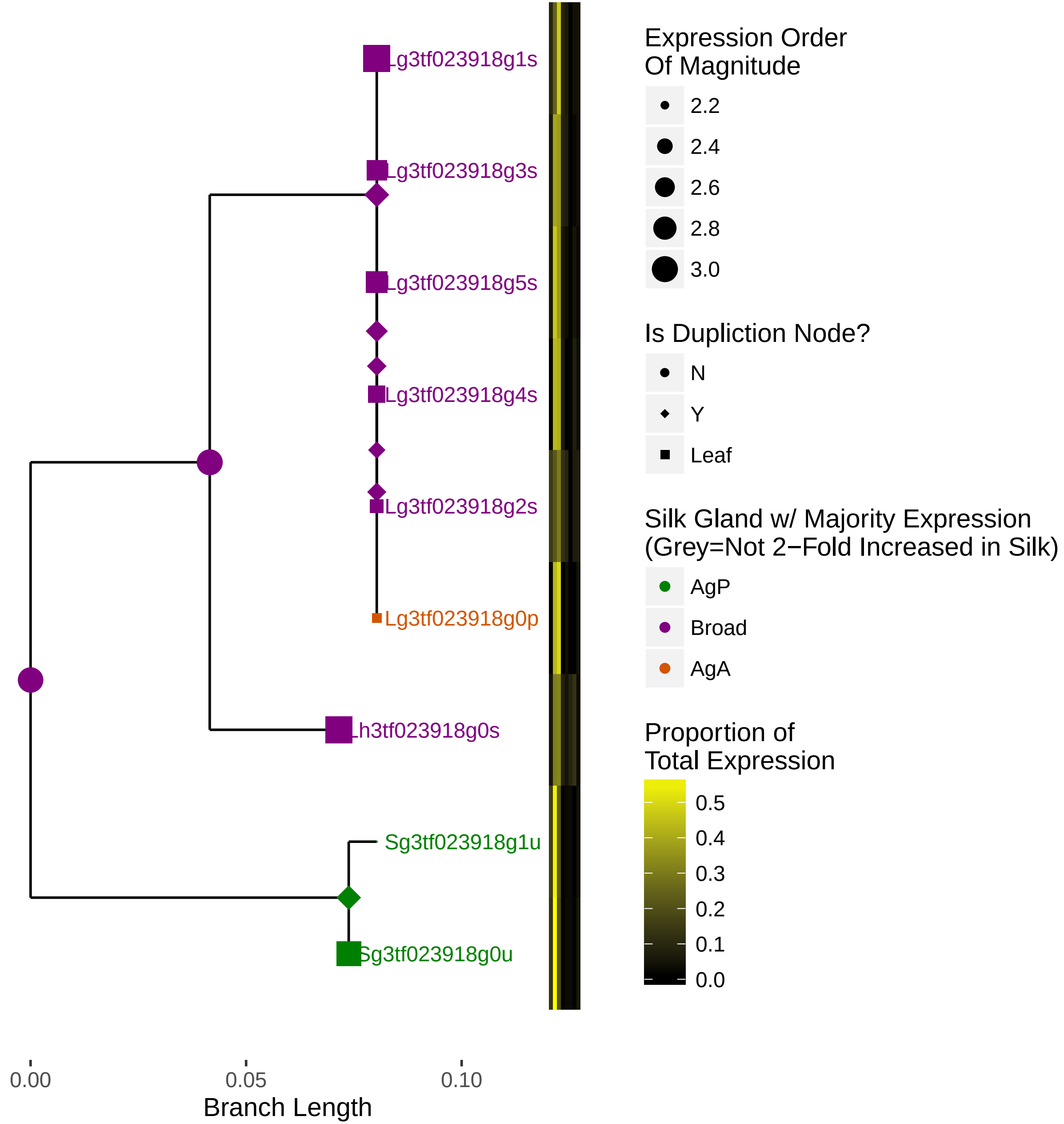
Lg3tf023918g1s
Lg3tf023918g3s
Lg3tf023918g5s
Lg3tf023918g4s
Lg3tf023918g2s
Lg3tf023918g0p
Lh3tf023918g0s
Sg3tf023918g1u
Sg3tf023918g0u
0.00
0.05
0.10
Branch Length
Expression Order Of Magnitude
2.2
2.4
2.6
2.8
3.0
Is Dupliction Node?
N
Y
Leaf
Silk Gland w/ Majority Expression (Grey=Not 2−Fold Increased in Silk)
AgP
Broad
AgA
Proportion of Total Expression
0.5
0.4
0.3
0.2
0.1
0.0

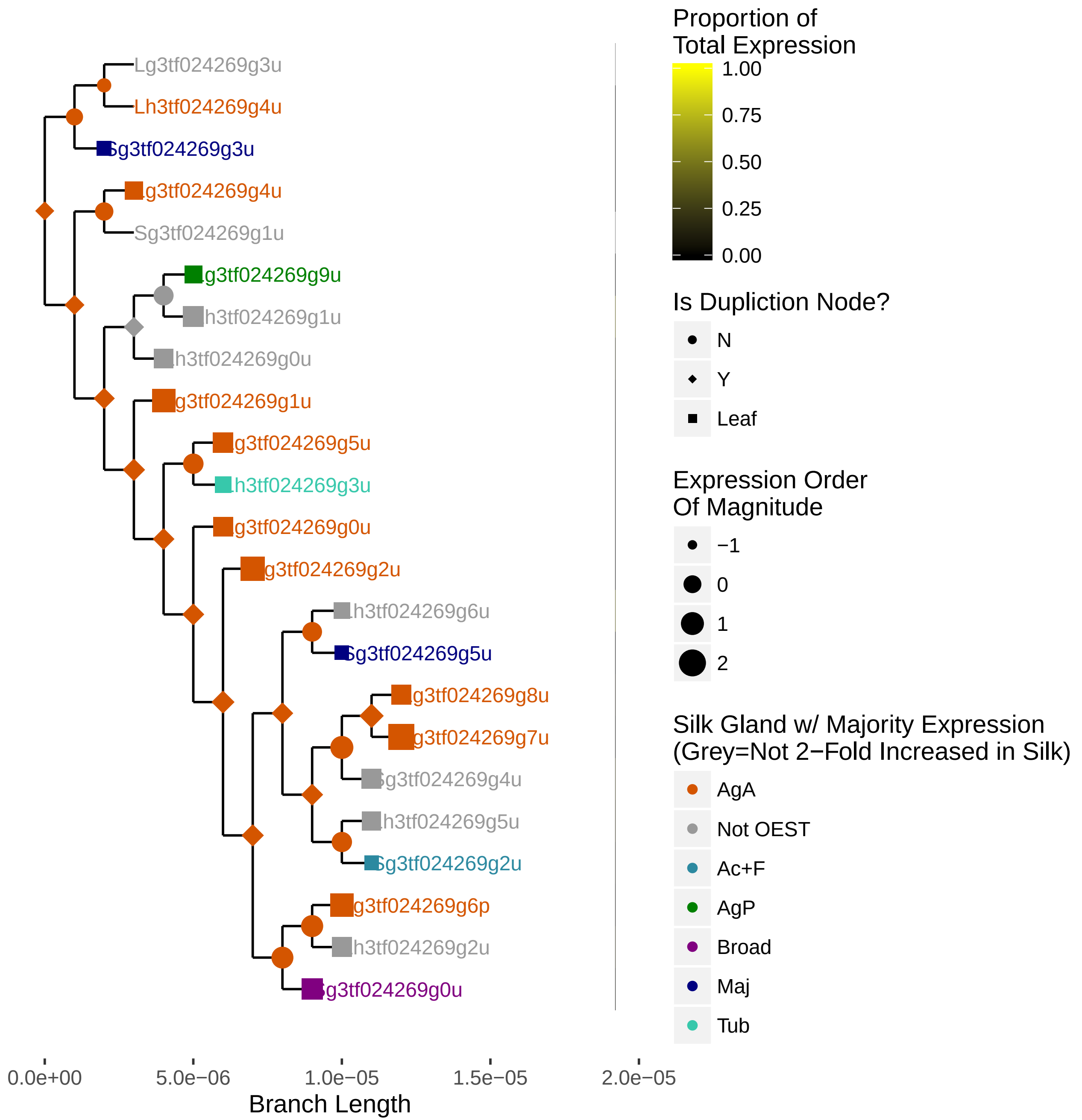

Lg3tf024269g3u
Lh3tf024269g4u
Sg3tf024269g3u
g3tf024269g4u
Sg3tf024269g1u
g3tf024269g9u
h3tf024269g1u
h3tf024269g0u
g3tf024269g1u
g3tf024269g5u
h3tf024269g3u
g3tf024269g0u
g3tf024269g2u
h3tf024269g6u
Sg3tf024269g5u
g3tf024269g8u
g3tf024269g7u
g3tf024269g4u
h3tf024269g5u
Sg3tf024269g2u
g3tf024269g6p
h3tf024269g2u
g3tf024269g0u
0.0e+00
5.0e−06
1.0e−05
1.5e−05
2.0e−05
Branch Length
Proportion of
Total Expression
1.00
0.75
0.50
0.25
0.00
Is Dupliction Node?
N
Y
Leaf
Expression Order
Of Magnitude
−1
0
1
2
Silk Gland w/ Majority Expression
(Grey=Not 2−Fold Increased in Silk)
AgA
Not OEST
Ac+F
AgP
Broad
Maj
Tub

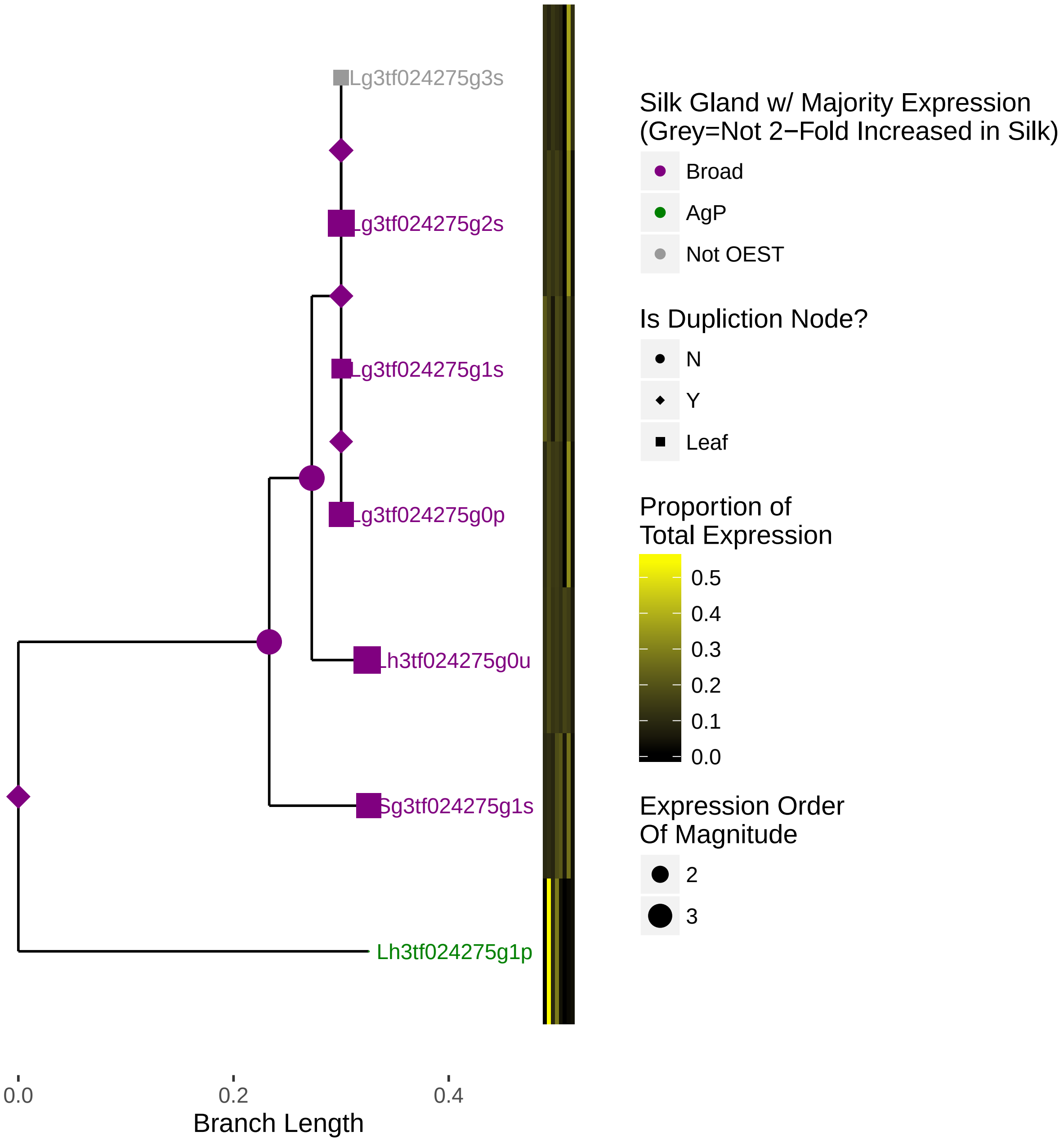

Lg3tf024275g3s
Lg3tf024275g2s
Lg3tf024275g1s
Lg3tf024275g0p
Lh3tf024275g0u
Sg3tf024275g1s
Lh3tf024275g1p
0.0
0.2
0.4
Branch Length
Silk Gland w/ Majority Expression
(Grey=Not 2−Fold Increased in Silk)
Broad
AgP
Not OEST
Is Dupliction Node?
N
Y
Leaf
Proportion of
Total Expression
0.5
0.4
0.3
0.2
0.1
0.0
Expression Order
Of Magnitude
2
3

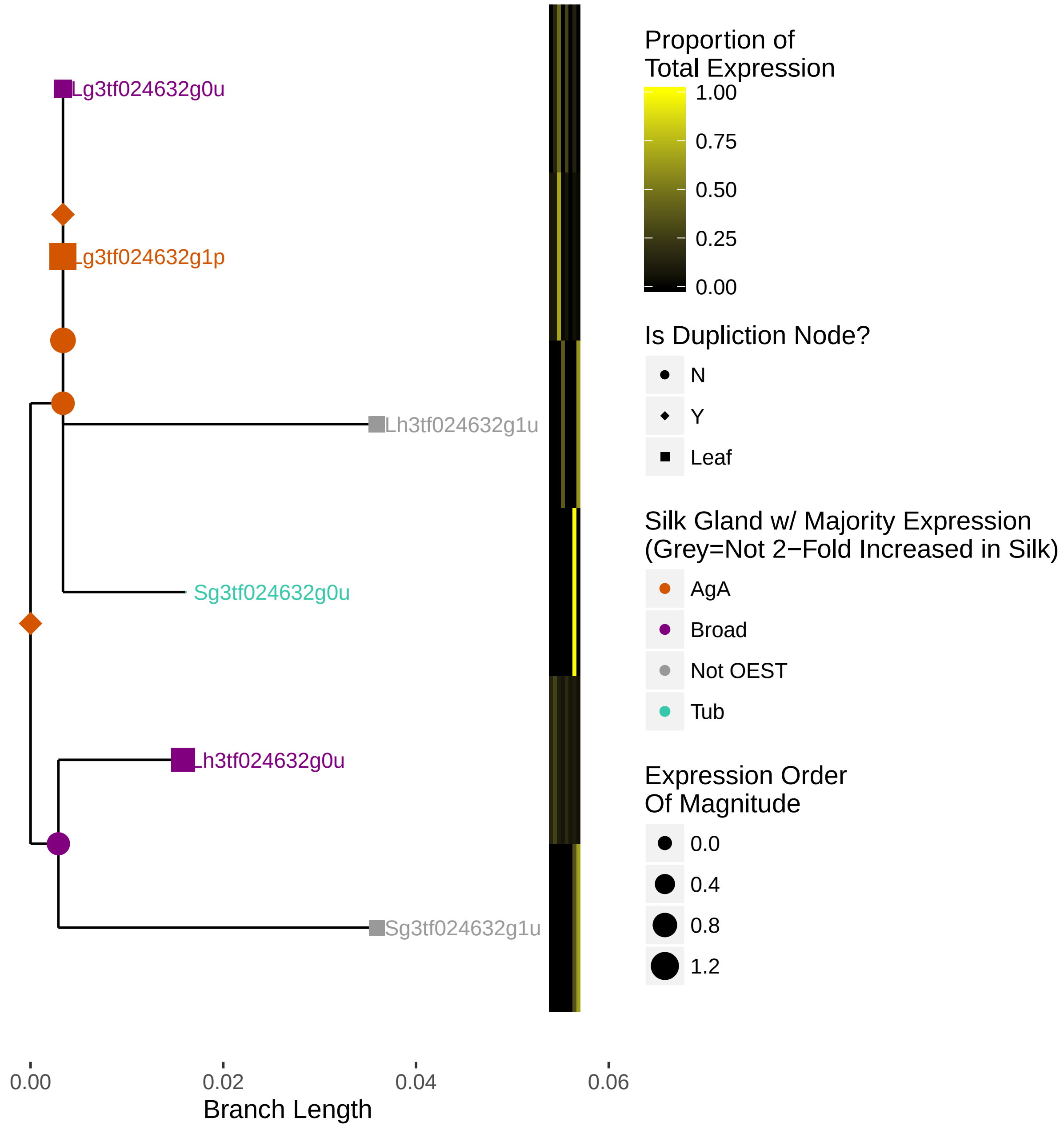

Lg3tf024632g0u
Lg3tf024632g1p
Lh3tf024632g1u
Sg3tf024632g0u
Lh3tf024632g0u
Sg3tf024632g1u
0.00
0.02
0.04
0.06
Branch Length
Proportion of
Total Expression
1.00
0.75
0.50
0.25
0.00
Is Dupliction Node?
N
Y
Leaf
Silk Gland w/ Majority Expression
(Grey=Not 2−Fold Increased in Silk)
AgA
Broad
Not OEST
Tub
Expression Order
Of Magnitude
0.0
0.4
0.8
1.2

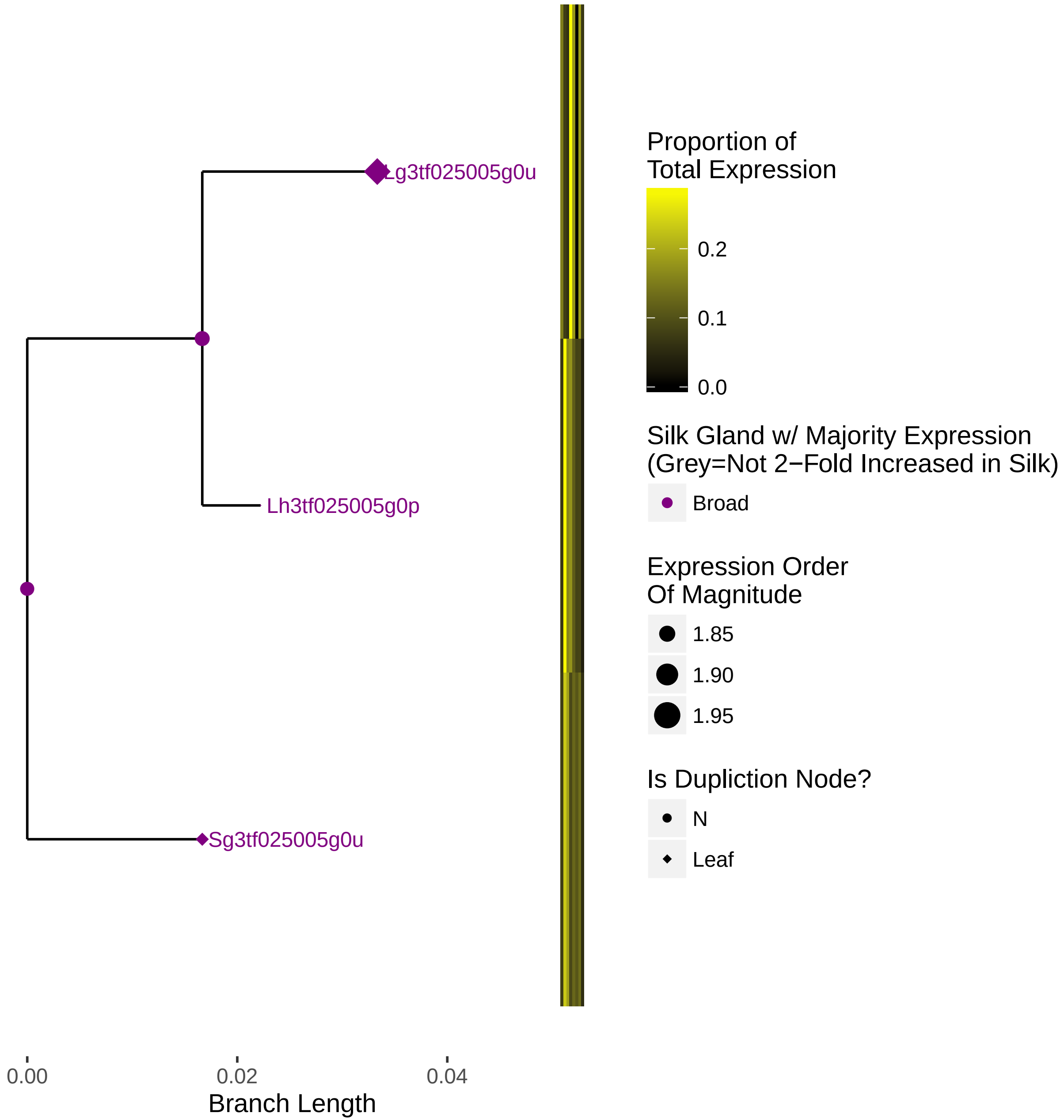

Lg3tf025005g0u
Lh3tf025005g0p
Sg3tf025005g0u
0.00
0.02
0.04
Branch Length
Proportion of
Total Expression
0.2
0.1
0.0
Silk Gland w/ Majority Expression
(Grey=Not 2−Fold Increased in Silk)
Broad
Expression Order
Of Magnitude
1.85
1.90
1.95
Is Dupliction Node?
N
Leaf

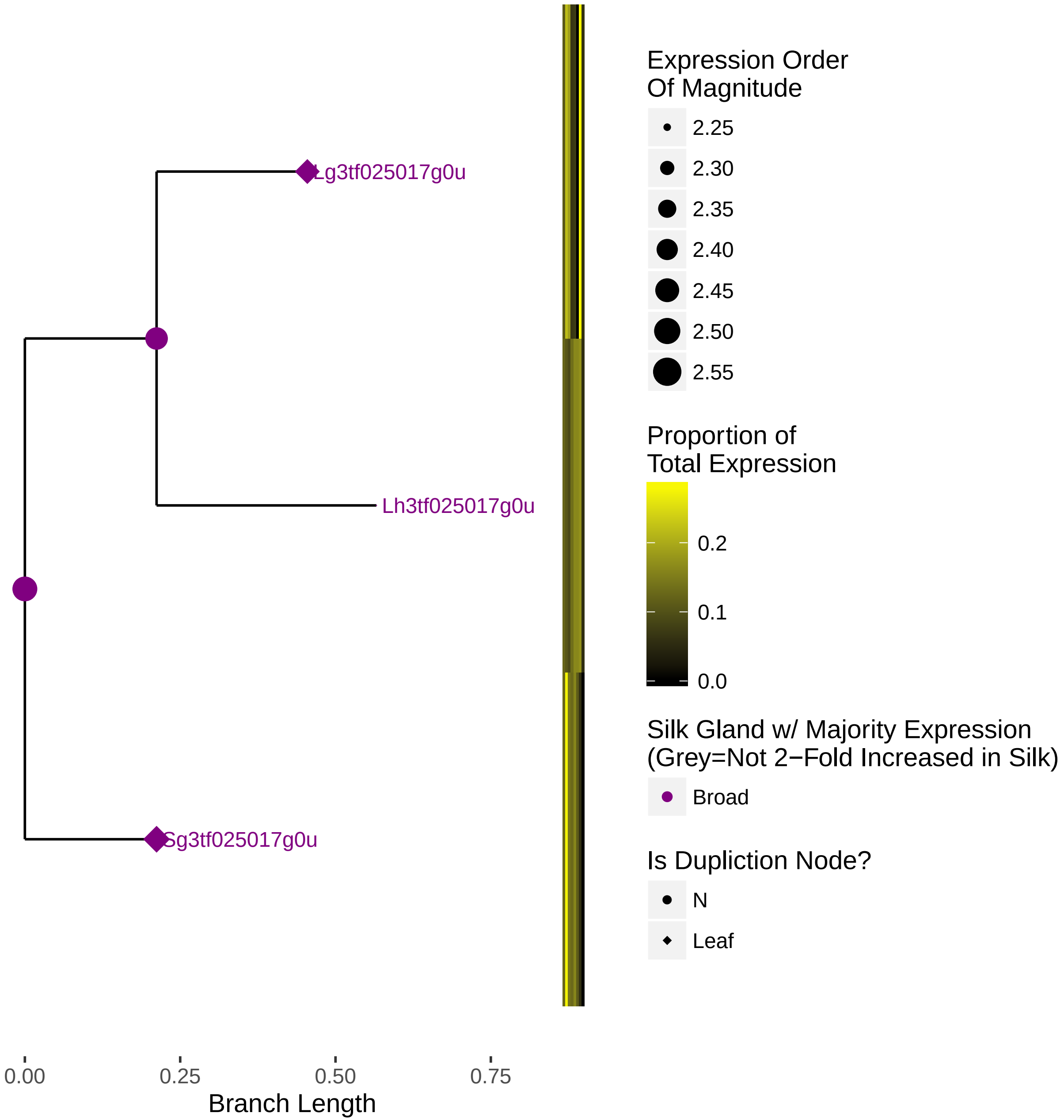

Lg3tf025017g0u
Lh3tf025017g0u
Sg3tf025017g0u
0.00
0.25
0.50
0.75
Branch Length
Expression Order
Of Magnitude
2.25
2.30
2.35
2.40
2.45
2.50
2.55
Proportion of
Total Expression
0.2
0.1
0.0
Silk Gland w/ Majority Expression
(Grey=Not 2−Fold Increased in Silk)
Broad
Is Dupliction Node?
N
Leaf

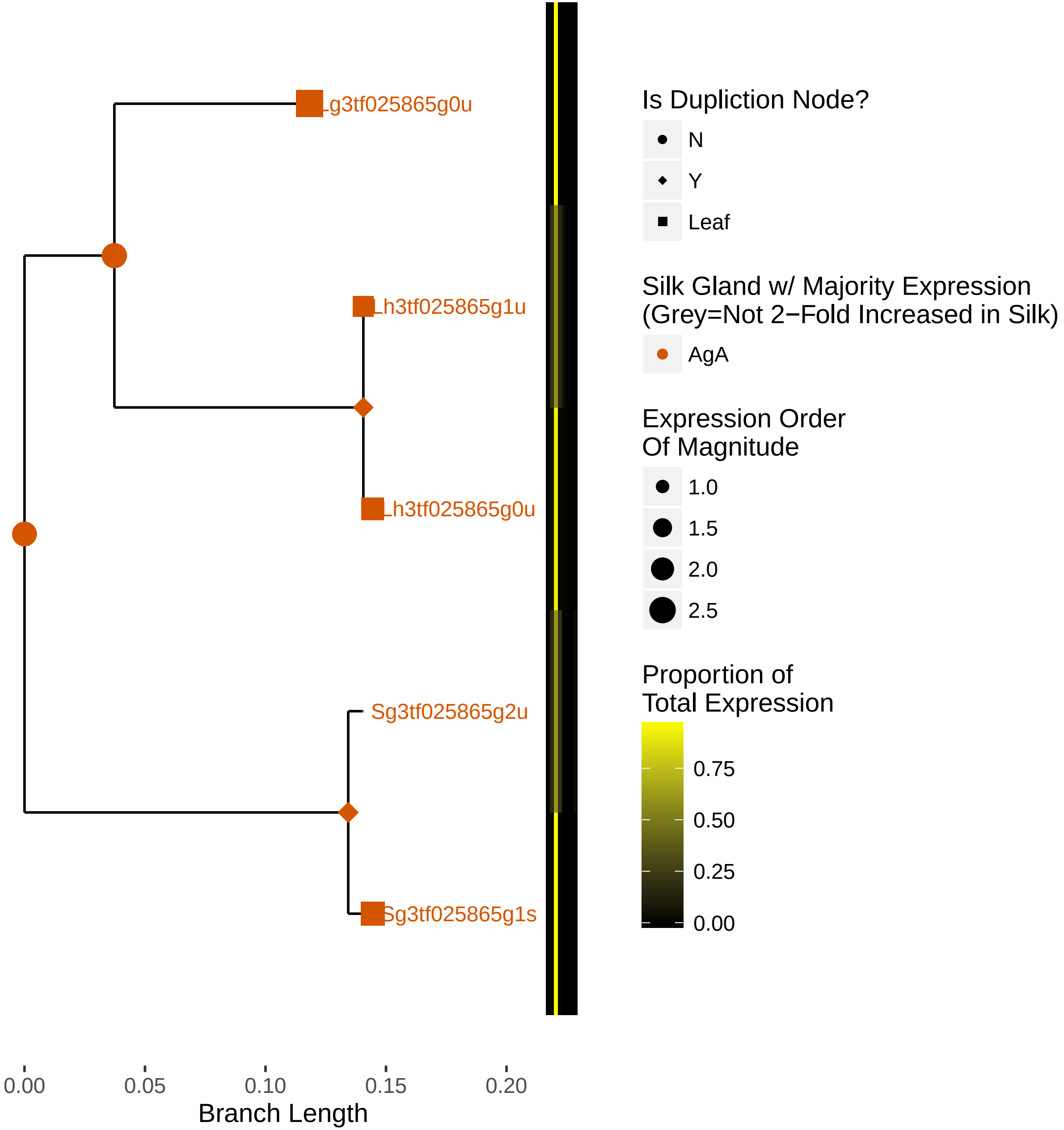

Lg3tf025865g0u
Lh3tf025865g1u
Lh3tf025865g0u
Sg3tf025865g2u
Sg3tf025865g1s
0.00
0.05
0.10
0.15
0.20
Branch Length
Is Dupliction Node?
N
Y
Leaf
Silk Gland w/ Majority Expression
(Grey=Not 2−Fold Increased in Silk)
AgA
Expression Order
Of Magnitude
1.0
1.5
2.0
2.5
Proportion of
Total Expression
0.75
0.50
0.25
0.00

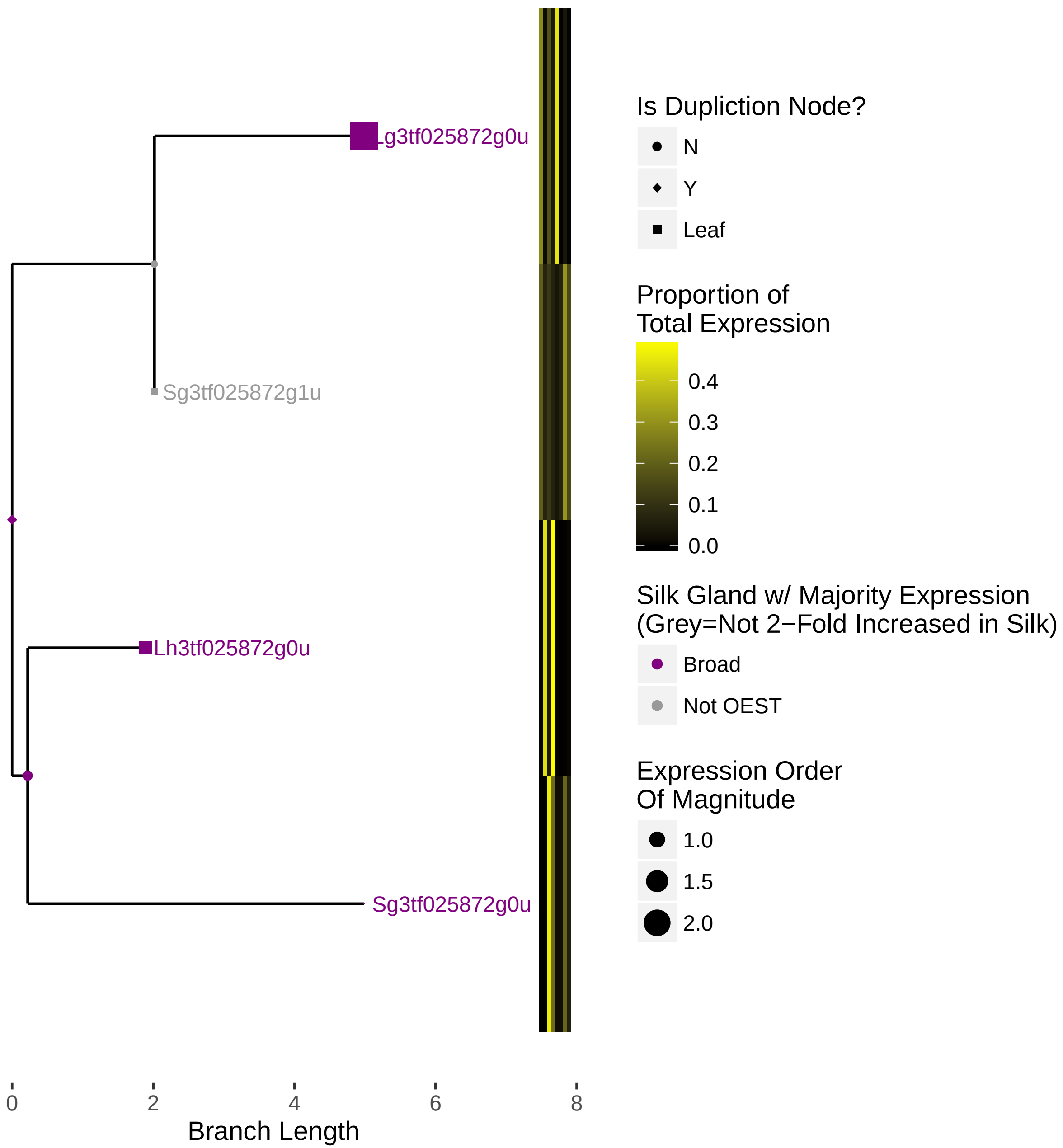

Lg3tf025872g0u
Sg3tf025872g1u
Lh3tf025872g0u
Sg3tf025872g0u
0
2
4
6
8
Branch Length
Is Dupliction Node?
N
Y
Leaf
Proportion of
Total Expression
0.4
0.3
0.2
0.1
0.0
Silk Gland w/ Majority Expression
(Grey=Not 2−Fold Increased in Silk)
Broad
Not OEST
Expression Order
Of Magnitude
1.0
1.5
2.0

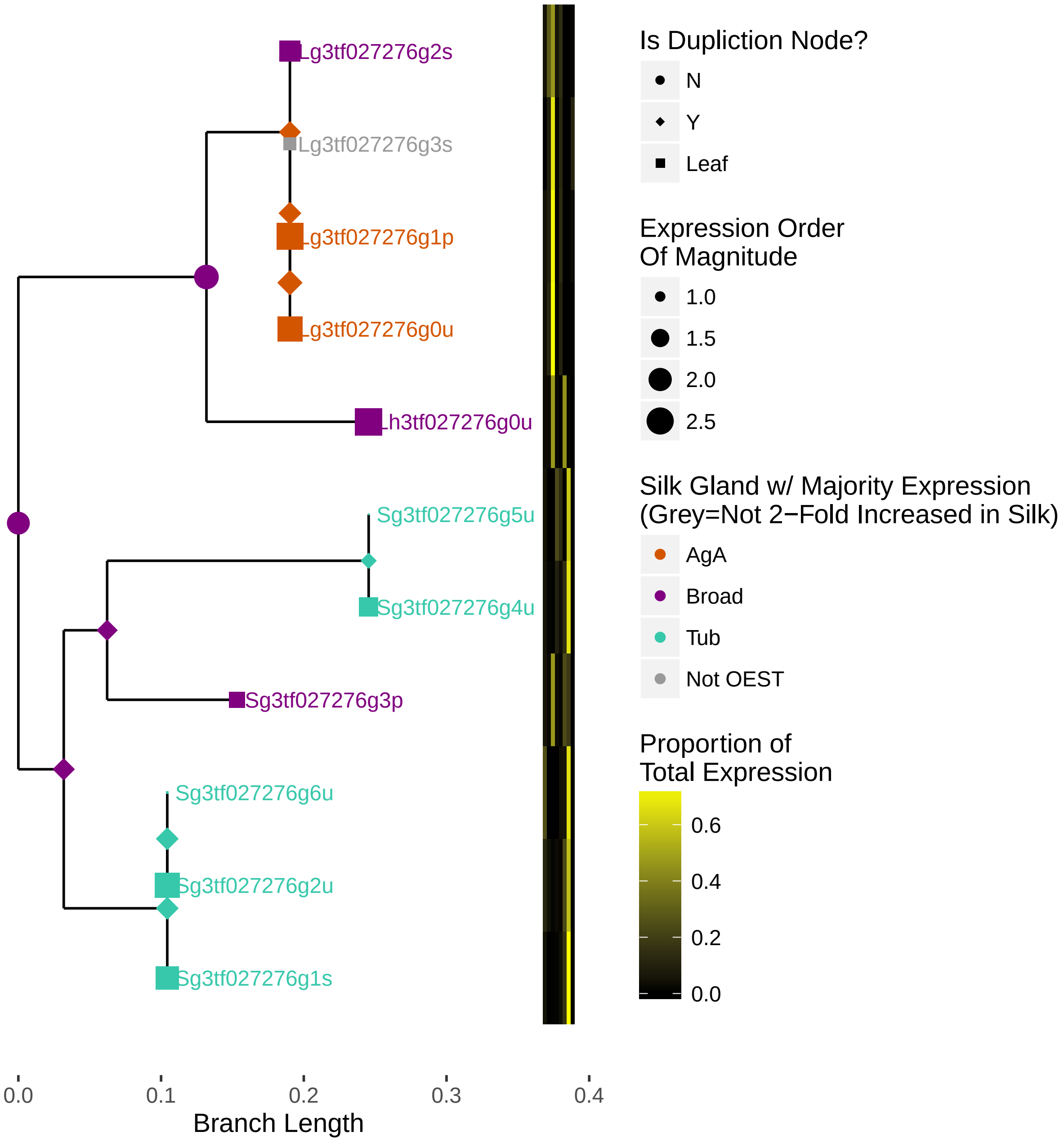

Lg3tf027276g2s
Lg3tf027276g3s
Lg3tf027276g1p
Lg3tf027276g0u
Lh3tf027276g0u
Sg3tf027276g5u
Sg3tf027276g4u
Sg3tf027276g3p
Sg3tf027276g6u
Sg3tf027276g2u
Sg3tf027276g1s
0.0
0.1
0.2
0.3
0.4
Branch Length
Is Dupliction Node?
N
Y
Leaf
Expression Order Of Magnitude
1.0
1.5
2.0
2.5
Silk Gland w/ Majority Expression (Grey=Not 2−Fold Increased in Silk)
AgA
Broad
Tub
Not OEST
Proportion of Total Expression
0.6
0.4
0.2
0.0

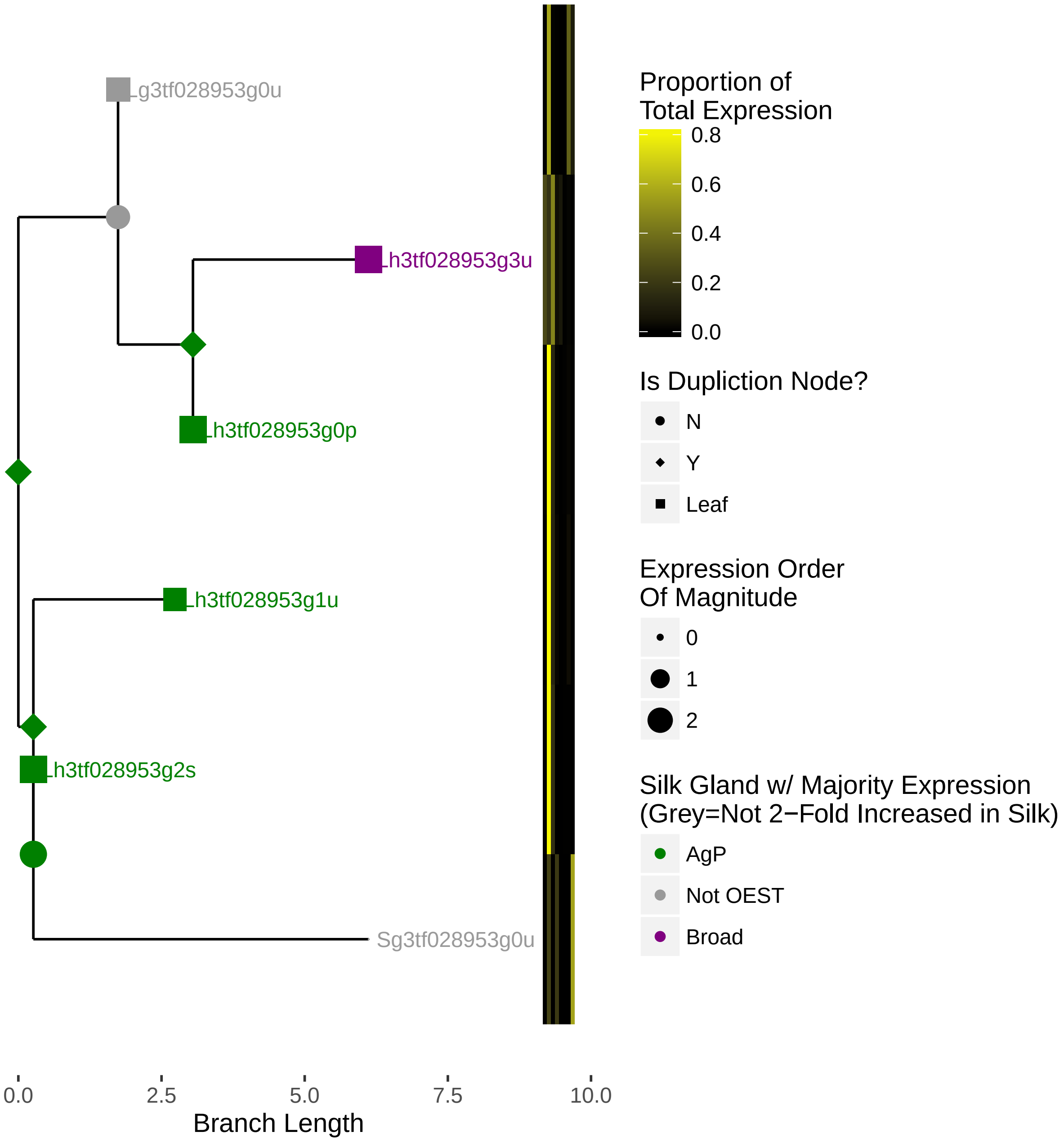

g3tf028953g0u
h3tf028953g3u
h3tf028953g0p
h3tf028953g1u
h3tf028953g2s
Sg3tf028953g0u
0.0
2.5
5.0
7.5
10.0
Branch Length
Proportion of Total Expression
0.8
0.6
0.4
0.2
0.0
Is Dupliction Node?
N
Y
Leaf
Expression Order Of Magnitude
0
1
2
Silk Gland w/ Majority Expression (Grey=Not 2−Fold Increased in Silk)
AgP
Not OEST
Broad

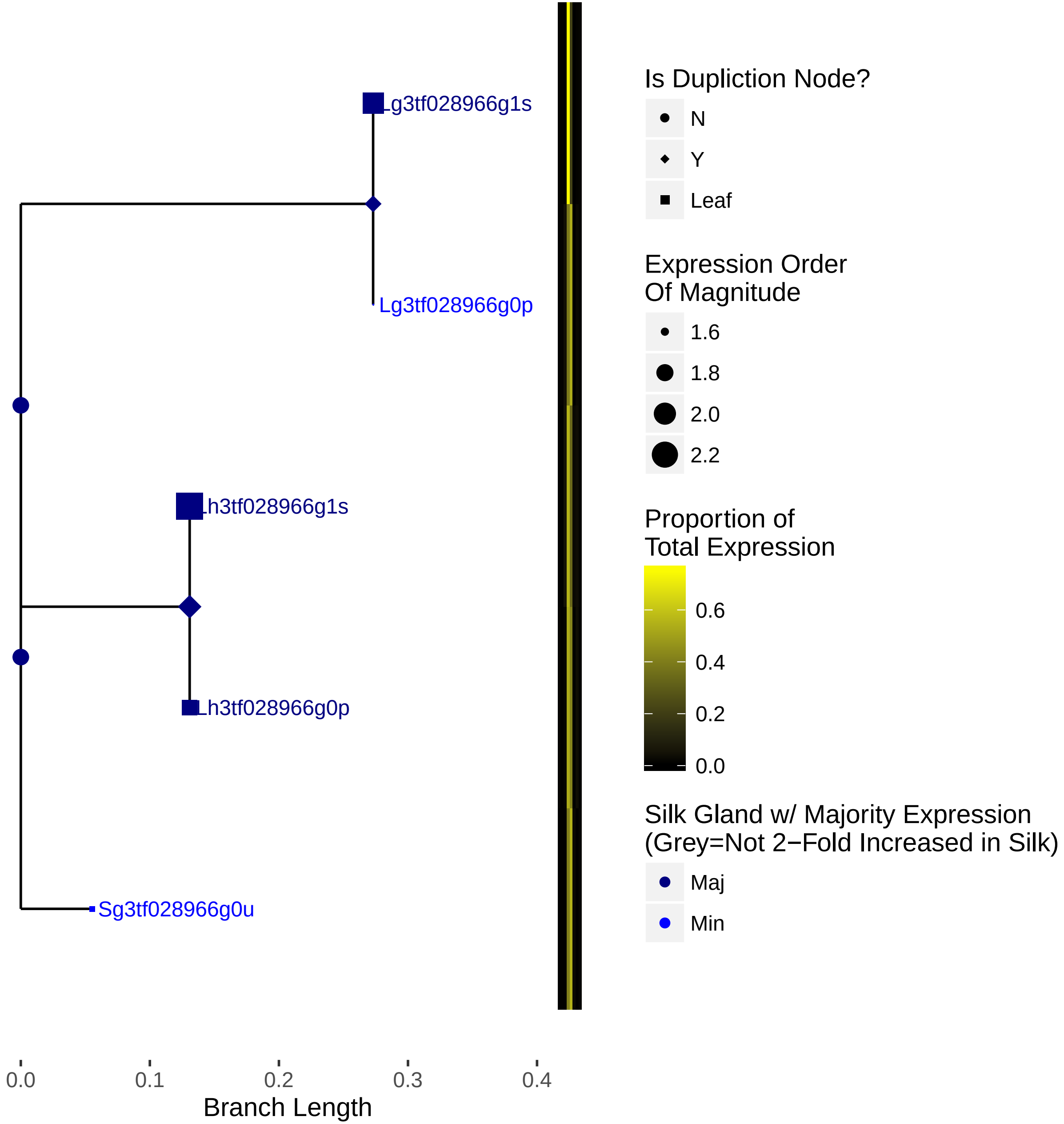

Lg3tf028966g1s
Lg3tf028966g0p
Lh3tf028966g1s
Lh3tf028966g0p
Sg3tf028966g0u
0.0
0.1
0.2
0.3
0.4
Branch Length
Is Dupliction Node?
N
Y
Leaf
Expression Order
Of Magnitude
1.6
1.8
2.0
2.2
Proportion of
Total Expression
0.6
0.4
0.2
0.0
Silk Gland w/ Majority Expression
(Grey=Not 2−Fold Increased in Silk)
Maj
Min

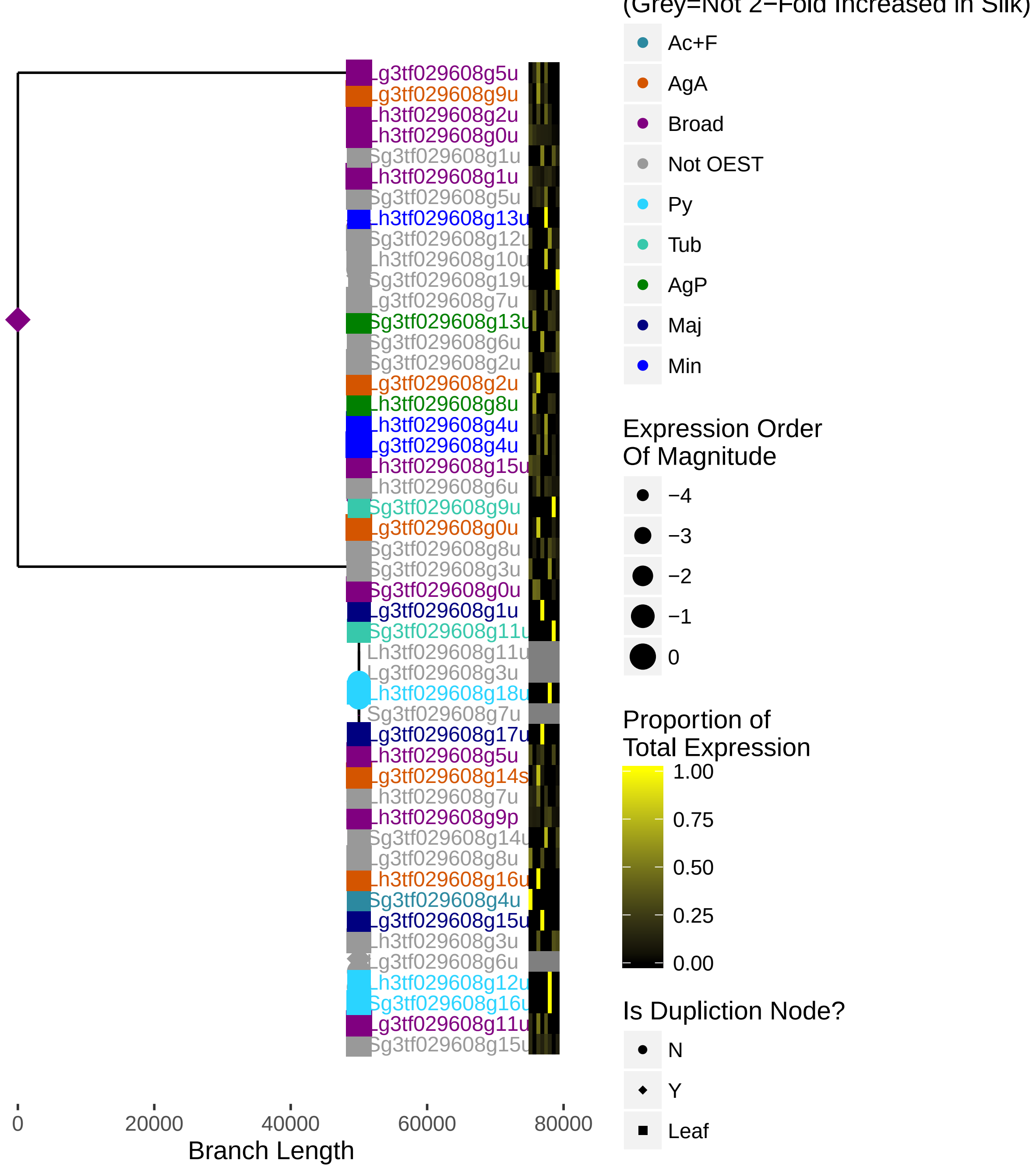
(Grey=Not 2−Fold Increased in Silk)
Ac+F
AgA
Broad
Not OEST
Py
Tub
AgP
Maj
Min
Expression Order Of Magnitude
−4
−3
−2
−1
0
Proportion of Total Expression
1.00
0.75
0.50
0.25
0.00
Is Dupliction Node?
N
Y
Leaf
Lg3tf029608g5u
Lg3tf029608g9u
Lh3tf029608g2u
Lh3tf029608g0u
Sg3tf029608g1u
Lh3tf029608g1u
Sg3tf029608g5u
Lh3tf029608g13u
Sg3tf029608g12u
Lh3tf029608g10u
Sg3tf029608g19u
Lg3tf029608g7u
Sg3tf029608g13u
Sg3tf029608g6u
Sg3tf029608g2u
Lg3tf029608g2u
Lh3tf029608g8u
Lh3tf029608g4u
Lg3tf029608g4u
Lh3tf029608g15u
Lh3tf029608g6u
Sg3tf029608g9u
Lg3tf029608g0u
Sg3tf029608g8u
Sg3tf029608g3u
Sg3tf029608g0u
Lg3tf029608g1u
Sg3tf029608g11u
Lh3tf029608g11u
Lg3tf029608g3u
Lh3tf029608g18u
Sg3tf029608g7u
Lg3tf029608g17u
Lh3tf029608g5u
Lg3tf029608g14s
Lh3tf029608g7u
Lh3tf029608g9p
Sg3tf029608g14u
Lg3tf029608g8u
Lh3tf029608g16u
Sg3tf029608g4u
Lg3tf029608g15u
Lh3tf029608g3u
Lg3tf029608g6u
Lh3tf029608g12u
Sg3tf029608g16u
Lg3tf029608g11u
Sg3tf029608g15u
0
20000
40000
60000
80000
Branch Length

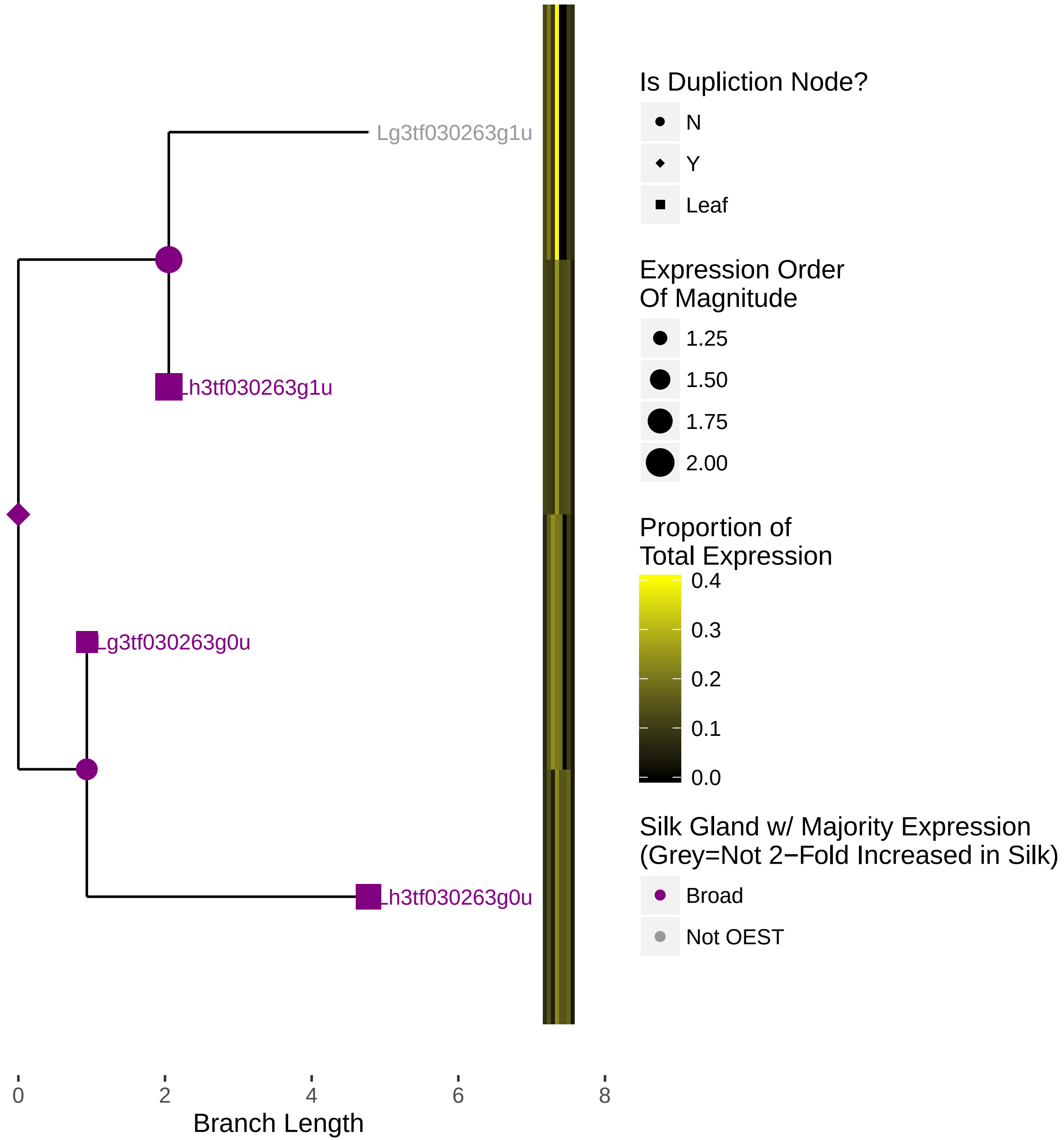

Lg3tf030263g1u
Lh3tf030263g1u
Lg3tf030263g0u
Lh3tf030263g0u
0
2
4
6
8
Branch Length
Is Dupliction Node?
N
Y
Leaf
Expression Order Of Magnitude
1.25
1.50
1.75
2.00
Proportion of Total Expression
0.4
0.3
0.2
0.1
0.0
Silk Gland w/ Majority Expression (Grey=Not 2−Fold Increased in Silk)
Broad
Not OEST

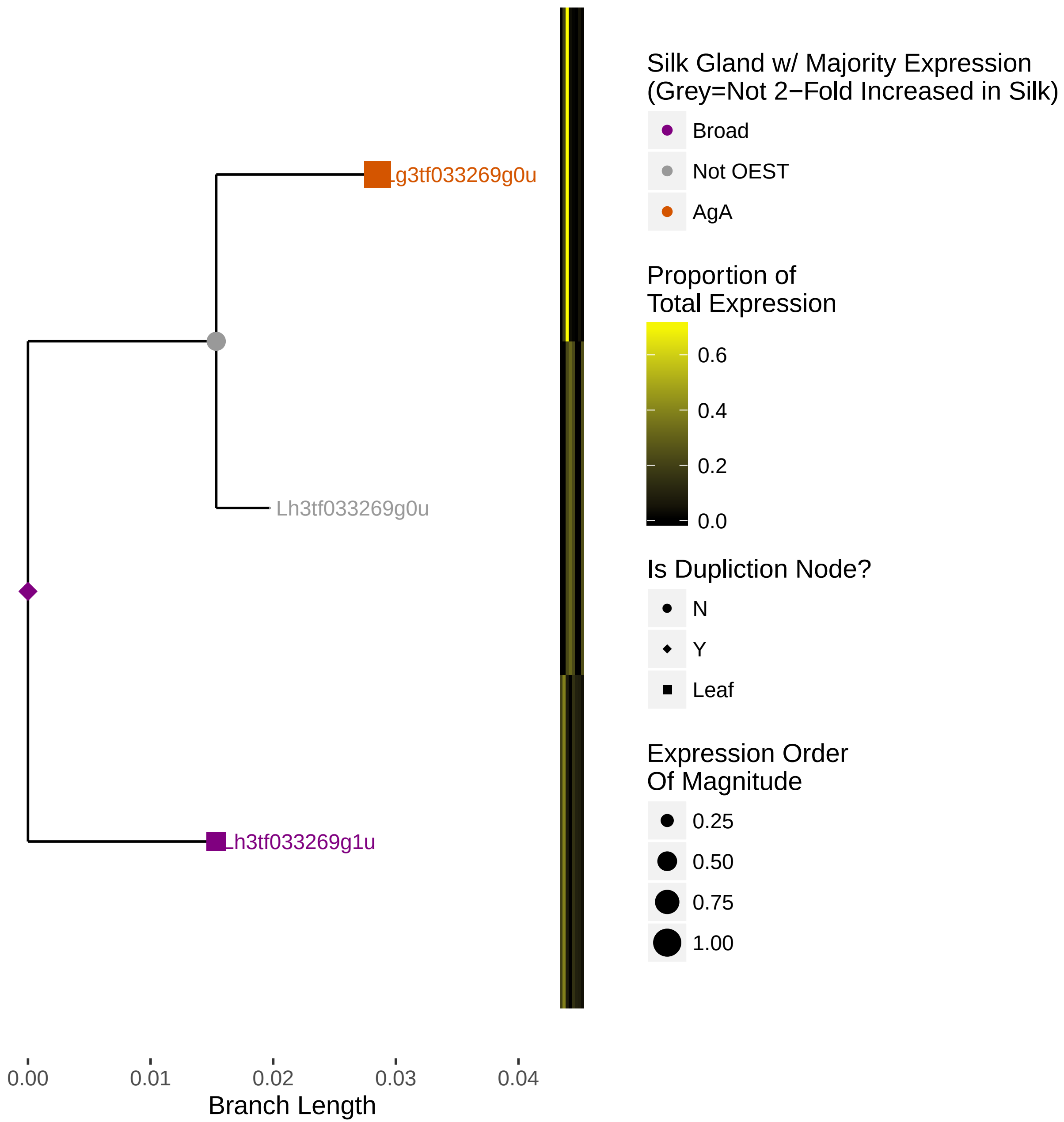

Lg3tf033269g0u
Lh3tf033269g0u
Lh3tf033269g1u
0.00
0.01
0.02
0.03
0.04
Branch Length
Silk Gland w/ Majority Expression
(Grey=Not 2−Fold Increased in Silk)
Broad
Not OEST
AgA
Proportion of
Total Expression
0.6
0.4
0.2
0.0
Is Dupliction Node?
N
Y
Leaf
Expression Order
Of Magnitude
0.25
0.50
0.75
1.00

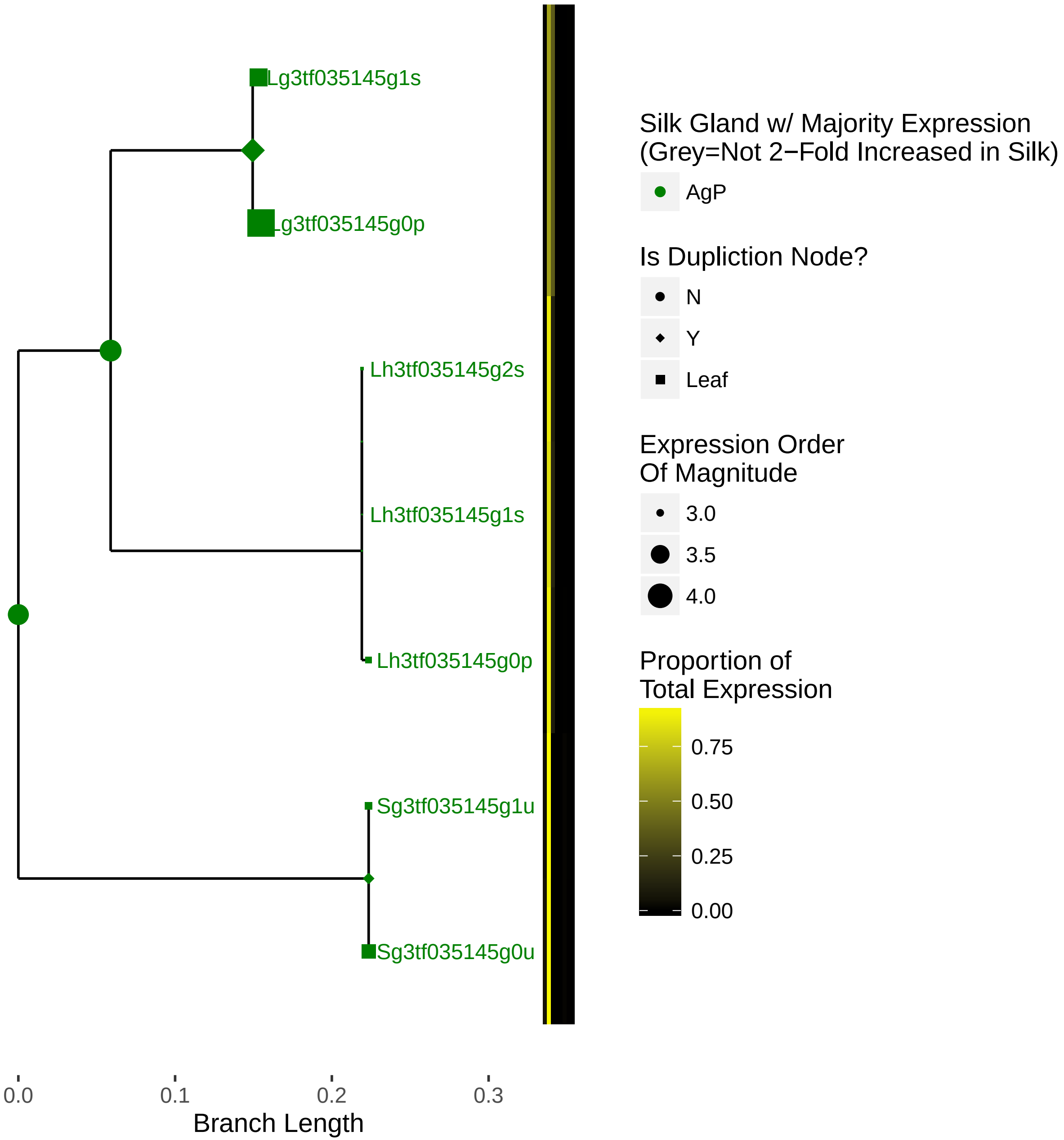

Lg3tf035145g1s
Lg3tf035145g0p
Lh3tf035145g2s
Lh3tf035145g1s
Lh3tf035145g0p
Sg3tf035145g1u
Sg3tf035145g0u
0.0
0.1
0.2
0.3
Branch Length
Silk Gland w/ Majority Expression
(Grey=Not 2−Fold Increased in Silk)
AgP
Is Dupliction Node?
N
Y
Leaf
Expression Order
Of Magnitude
3.0
3.5
4.0
Proportion of
Total Expression
0.75
0.50
0.25
0.00

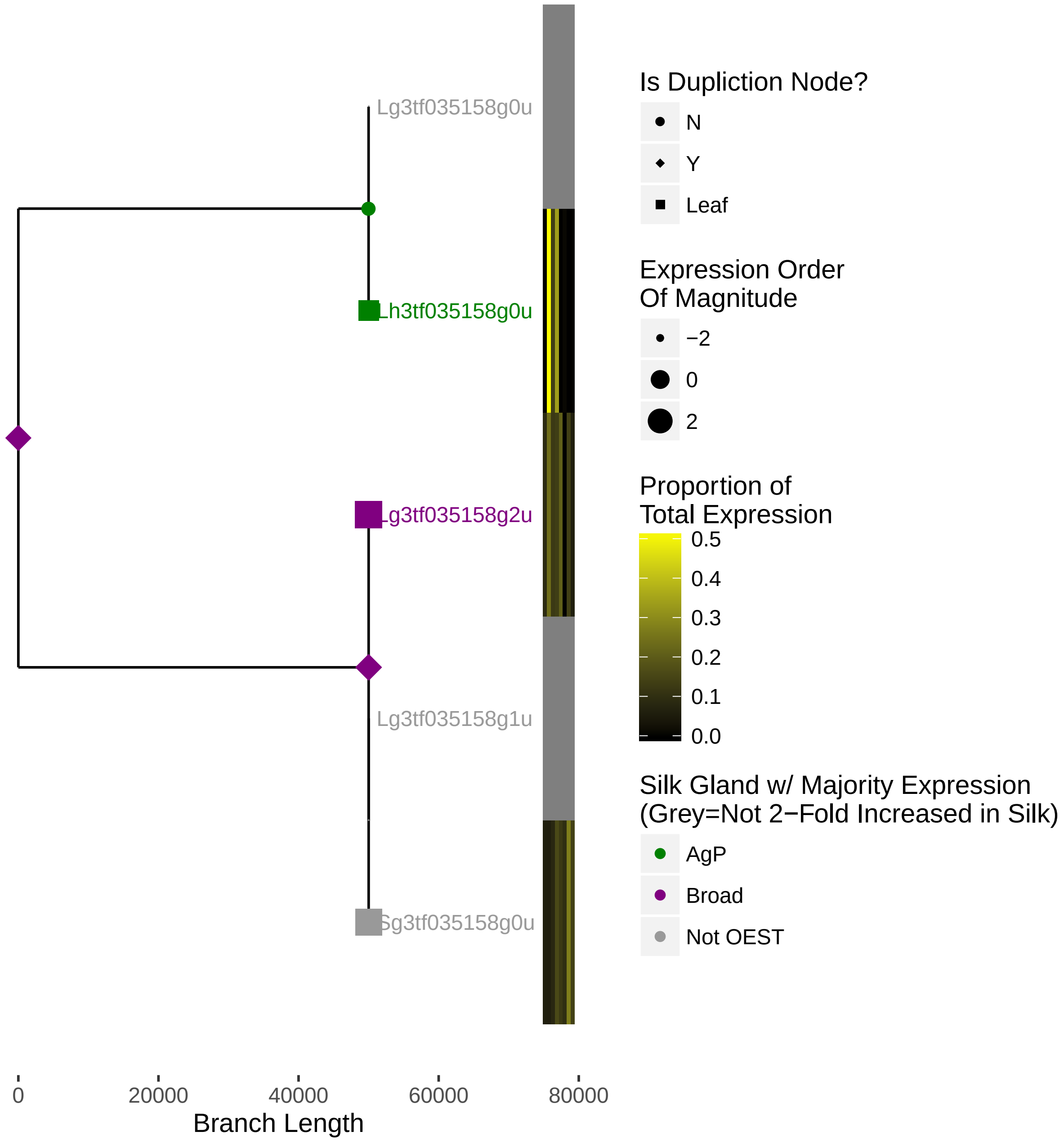

Lg3tf035158g0u
Lh3tf035158g0u
Lg3tf035158g2u
Lg3tf035158g1u
Sg3tf035158g0u
Is Dupliction Node?
N
Y
Leaf
Expression Order
Of Magnitude
−2
0
2
Proportion of
Total Expression
0.5
0.4
0.3
0.2
0.1
0.0
Silk Gland w/ Majority Expression
(Grey=Not 2−Fold Increased in Silk)
AgP
Broad
Not OEST
0
20000
40000
60000
80000
Branch Length

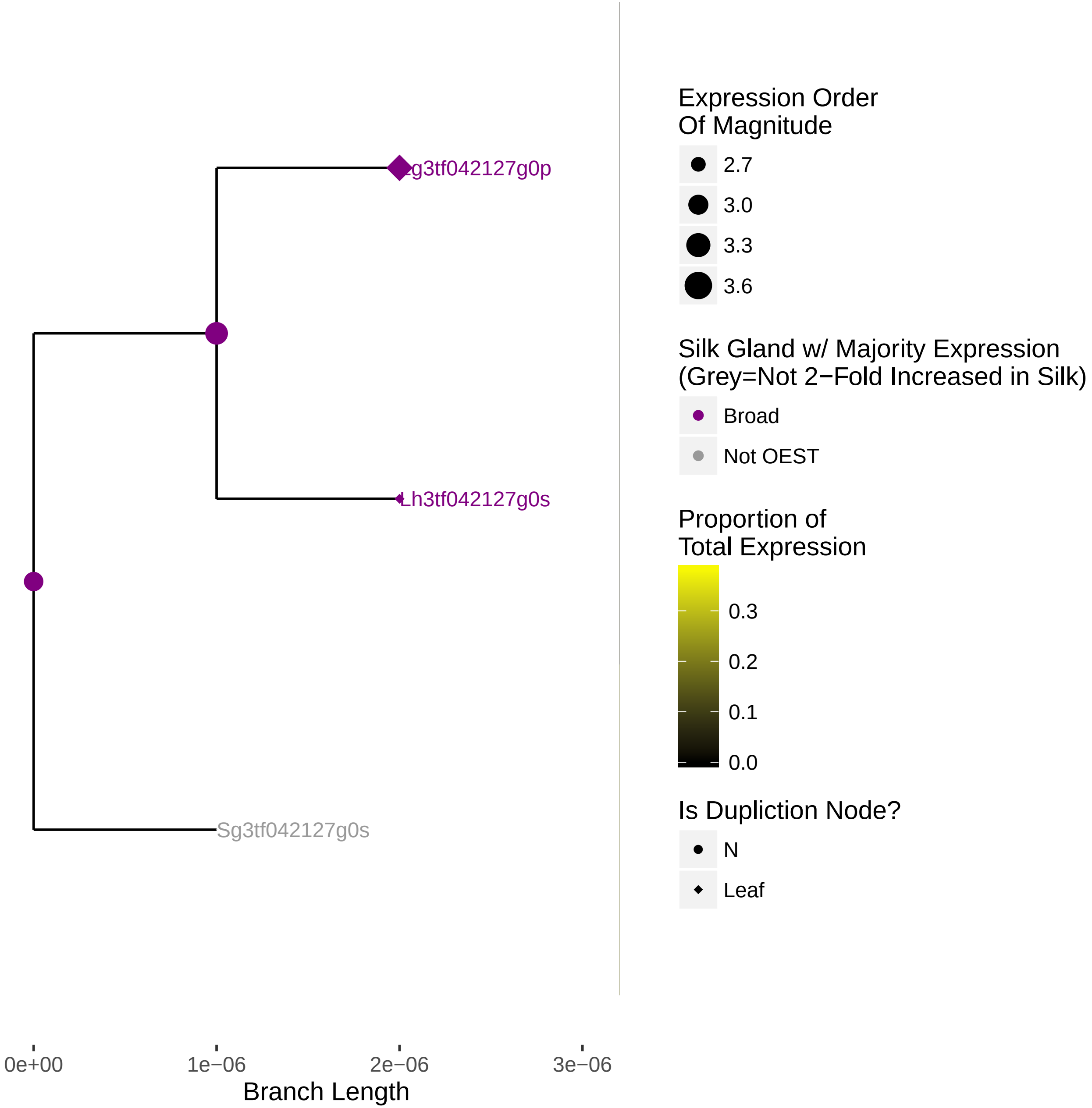

Sg3tf042127g0p
Lh3tf042127g0s
Sg3tf042127g0s
0e+00
1e−06
2e−06
3e−06
Branch Length
Expression Order
Of Magnitude
2.7
3.0
3.3
3.6
Silk Gland w/ Majority Expression
(Grey=Not 2−Fold Increased in Silk)
Broad
Not OEST
Proportion of
Total Expression
0.3
0.2
0.1
0.0
Is Dupliction Node?
N
Leaf

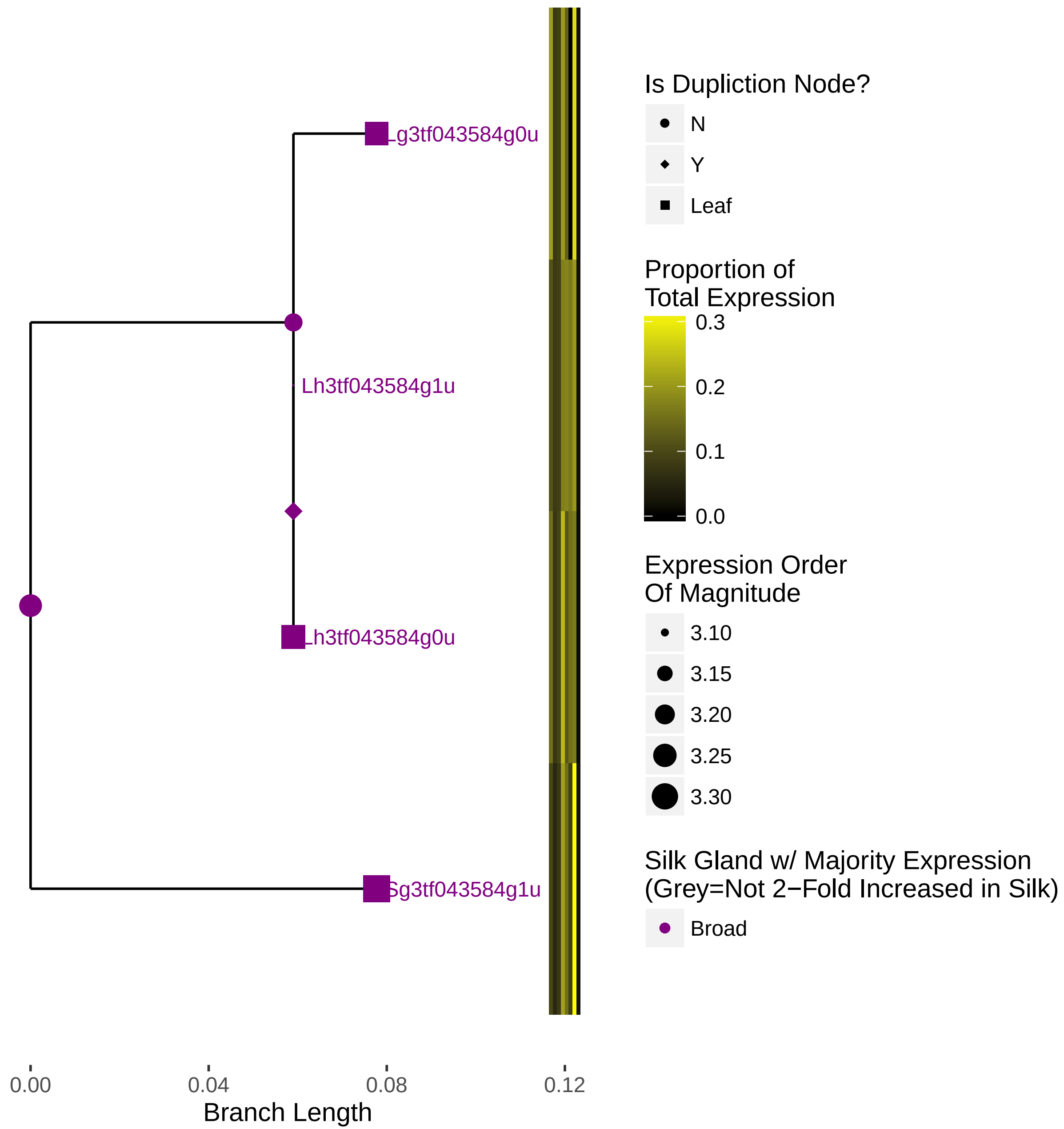

Lg3tf043584g0u
Lh3tf043584g1u
Lh3tf043584g0u
Sg3tf043584g1u
0.00
0.04
0.08
0.12
Branch Length
Is Dupliction Node?
N
Y
Leaf
Proportion of
Total Expression
0.3
0.2
0.1
0.0
Expression Order
Of Magnitude
3.10
3.15
3.20
3.25
3.30
Silk Gland w/ Majority Expression
(Grey=Not 2−Fold Increased in Silk)
Broad

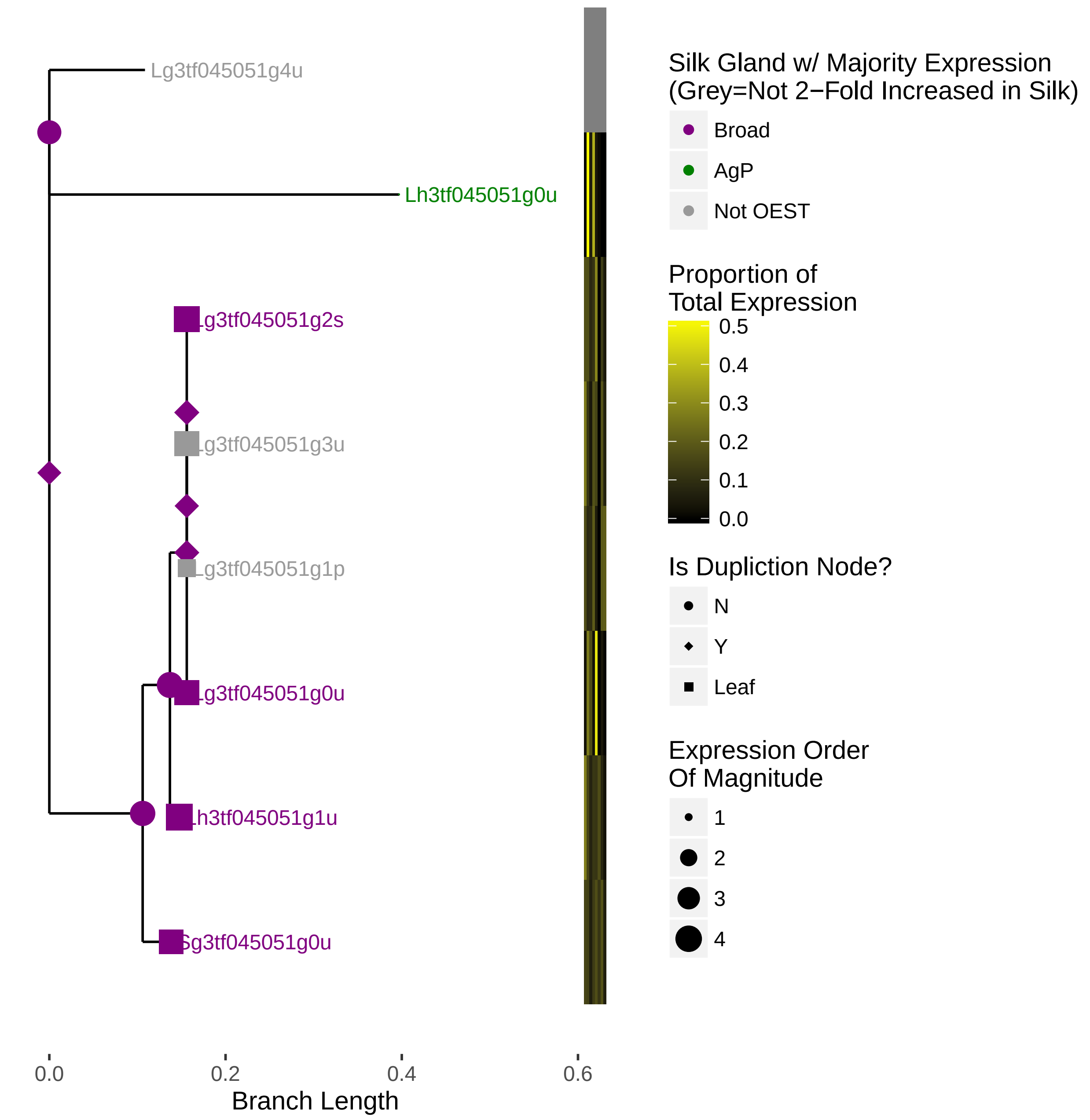

Lg3tf045051g4u
Lh3tf045051g0u
Lg3tf045051g2s
Lg3tf045051g3u
Lg3tf045051g1p
Lg3tf045051g0u
Lh3tf045051g1u
Sg3tf045051g0u
0.0
0.2
0.4
0.6
Branch Length
Silk Gland w/ Majority Expression
(Grey=Not 2−Fold Increased in Silk)
Broad
AgP
Not OEST
Proportion of
Total Expression
0.5
0.4
0.3
0.2
0.1
0.0
Is Dupliction Node?
N
Y
Leaf
Expression Order
Of Magnitude
1
2
3
4

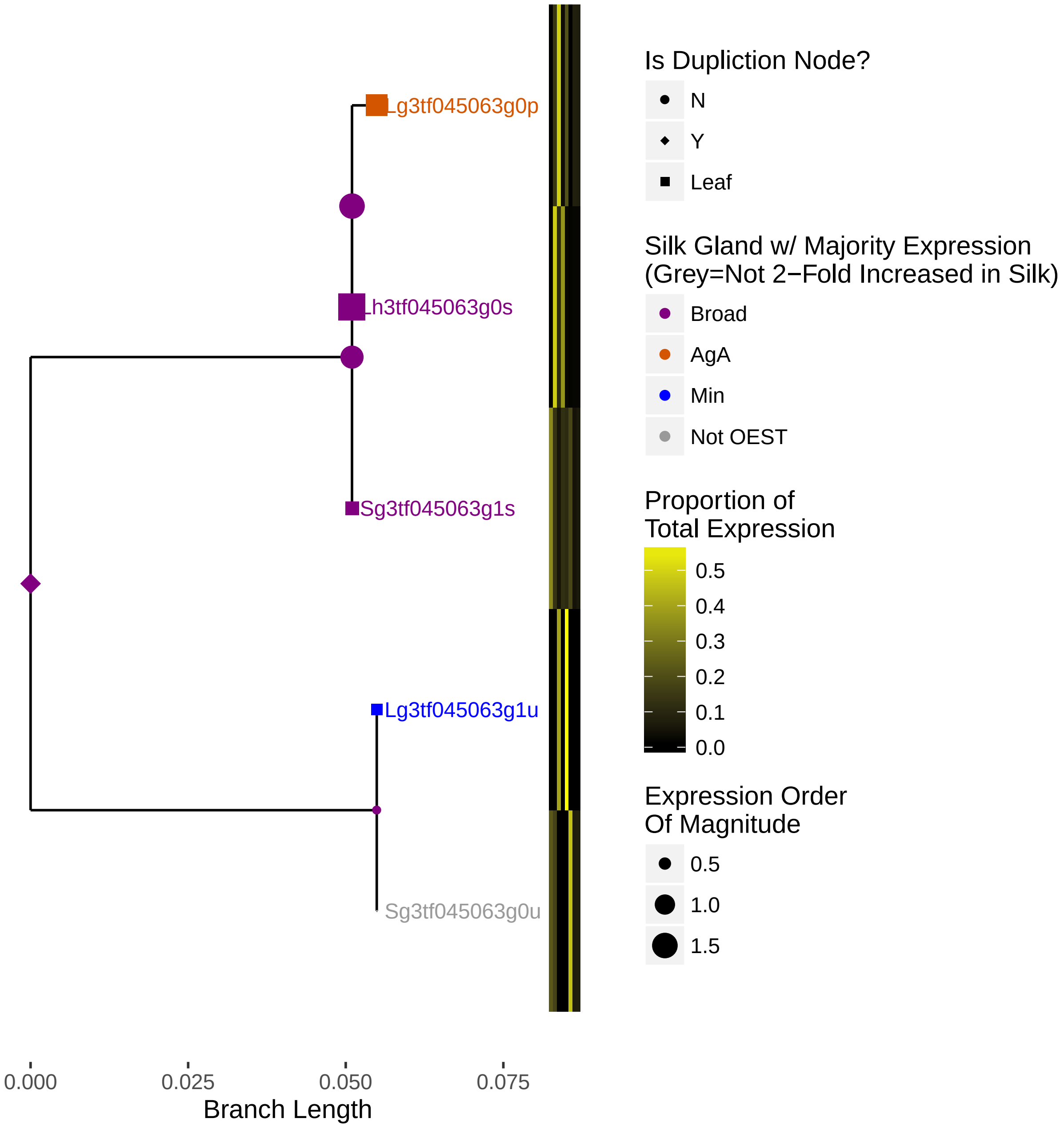
Lg3tf045063g0p
Lh3tf045063g0s
Sg3tf045063g1s
Lg3tf045063g1u
Sg3tf045063g0u
0.000
0.025
0.050
0.075
Branch Length
Is Dupliction Node?
N
Y
Leaf
Silk Gland w/ Majority Expression
(Grey=Not 2−Fold Increased in Silk)
Broad
AgA
Min
Not OEST
Proportion of
Total Expression
0.5
0.4
0.3
0.2
0.1
0.0
Expression Order
Of Magnitude
0.5
1.0
1.5

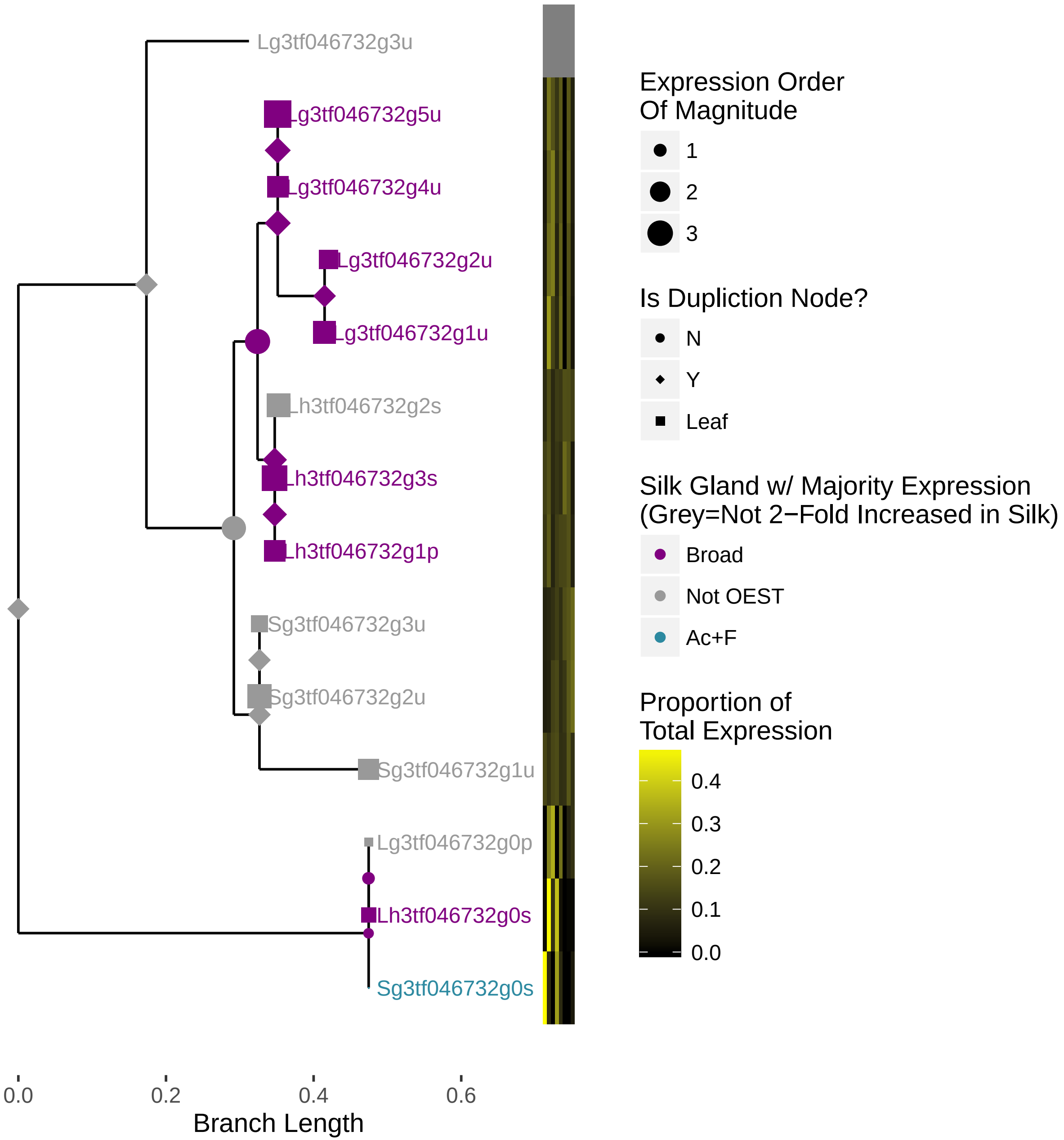
Lg3tf046732g3u
Lg3tf046732g5u
Lg3tf046732g4u
Lg3tf046732g2u
Lg3tf046732g1u
Lh3tf046732g2s
Lh3tf046732g3s
Lh3tf046732g1p
Sg3tf046732g3u
Sg3tf046732g2u
Sg3tf046732g1u
Lg3tf046732g0p
Lh3tf046732g0s
Sg3tf046732g0s
0.0
0.2
0.4
0.6
Branch Length
Expression Order
Of Magnitude
1
2
3
Is Dupliction Node?
N
Y
Leaf
Silk Gland w/ Majority Expression
(Grey=Not 2−Fold Increased in Silk)
Broad
Not OEST
Ac+F
Proportion of
Total Expression
0.4
0.3
0.2
0.1
0.0

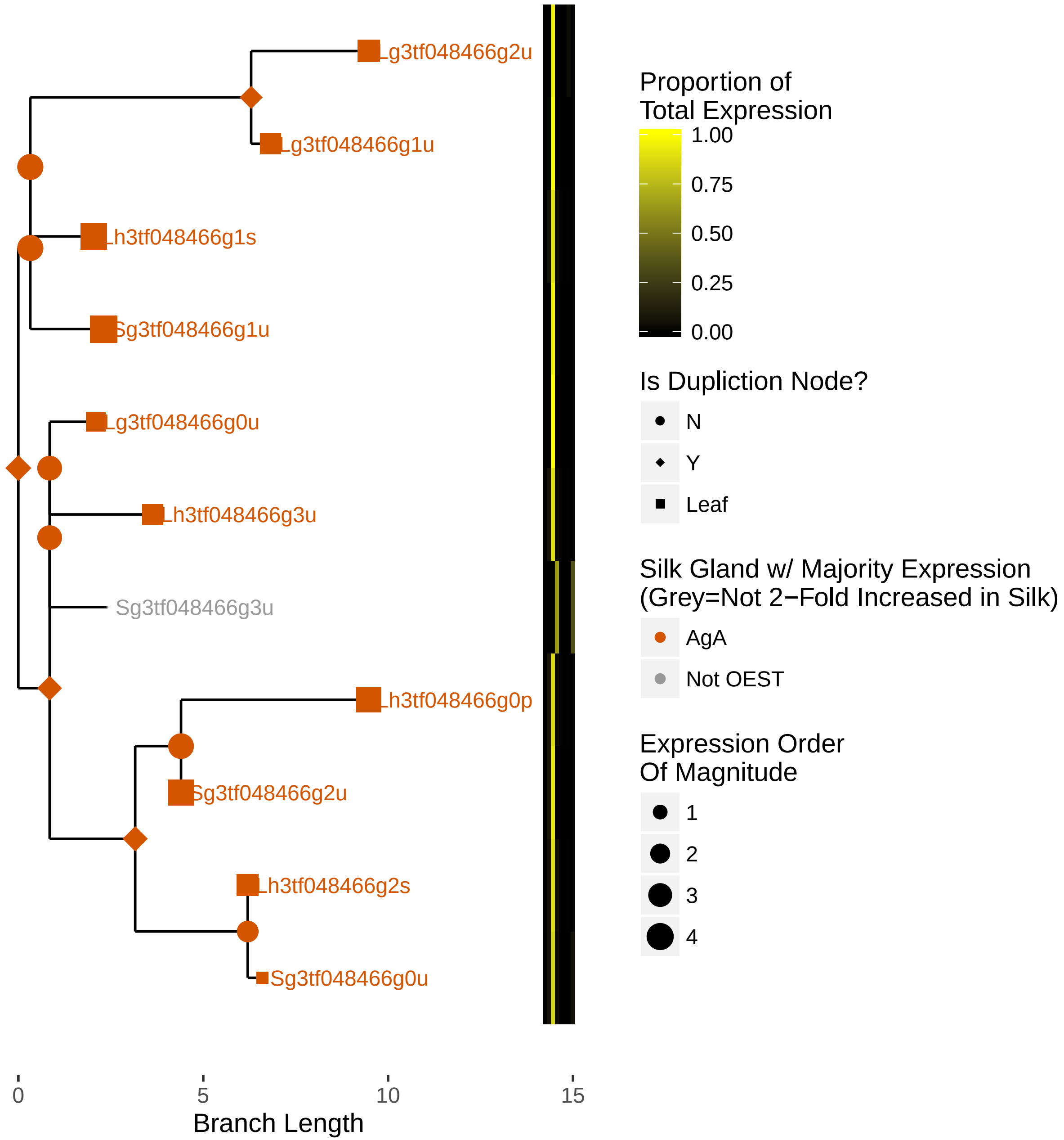

Lg3tf048466g2u
Lg3tf048466g1u
Lh3tf048466g1s
Sg3tf048466g1u
Lg3tf048466g0u
Lh3tf048466g3u
Sg3tf048466g3u
Lh3tf048466g0p
Sg3tf048466g2u
Lh3tf048466g2s
Sg3tf048466g0u
0
5
10
15
Branch Length
Proportion of
Total Expression
1.00
0.75
0.50
0.25
0.00
Is Dupliction Node?
N
Y
Leaf
Silk Gland w/ Majority Expression
(Grey=Not 2−Fold Increased in Silk)
AgA
Not OEST
Expression Order
Of Magnitude
1
2
3
4

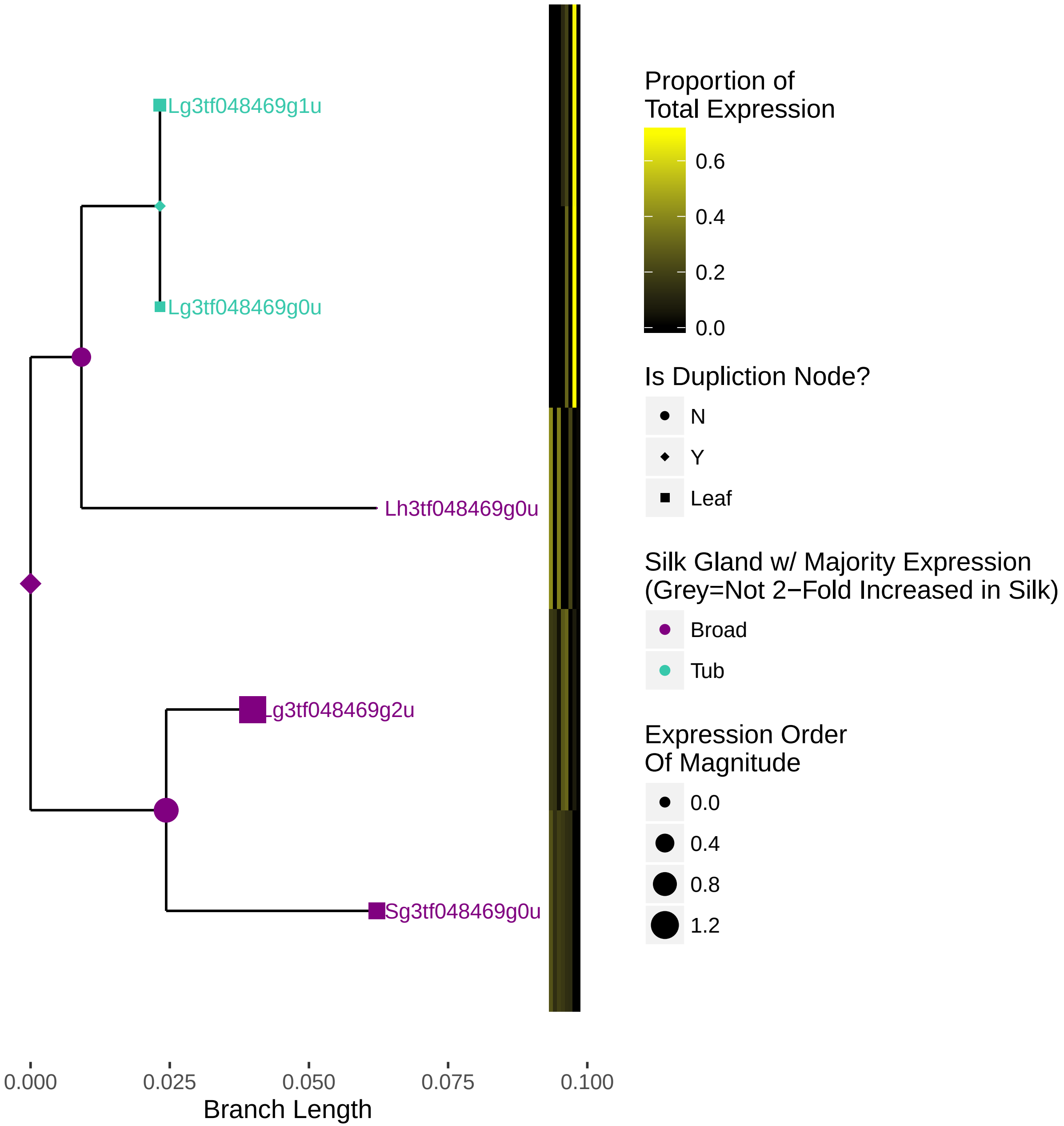

Lg3tf048469g1u
Lg3tf048469g0u
Lh3tf048469g0u
Lg3tf048469g2u
Sg3tf048469g0u
Proportion of
Total Expression
0.6
0.4
0.2
0.0
Is Dupliction Node?
N
Y
Leaf
Silk Gland w/ Majority Expression
(Grey=Not 2−Fold Increased in Silk)
Broad
Tub
Expression Order
Of Magnitude
0.0
0.4
0.8
1.2
0.000
0.025
0.050
0.075
0.100
Branch Length

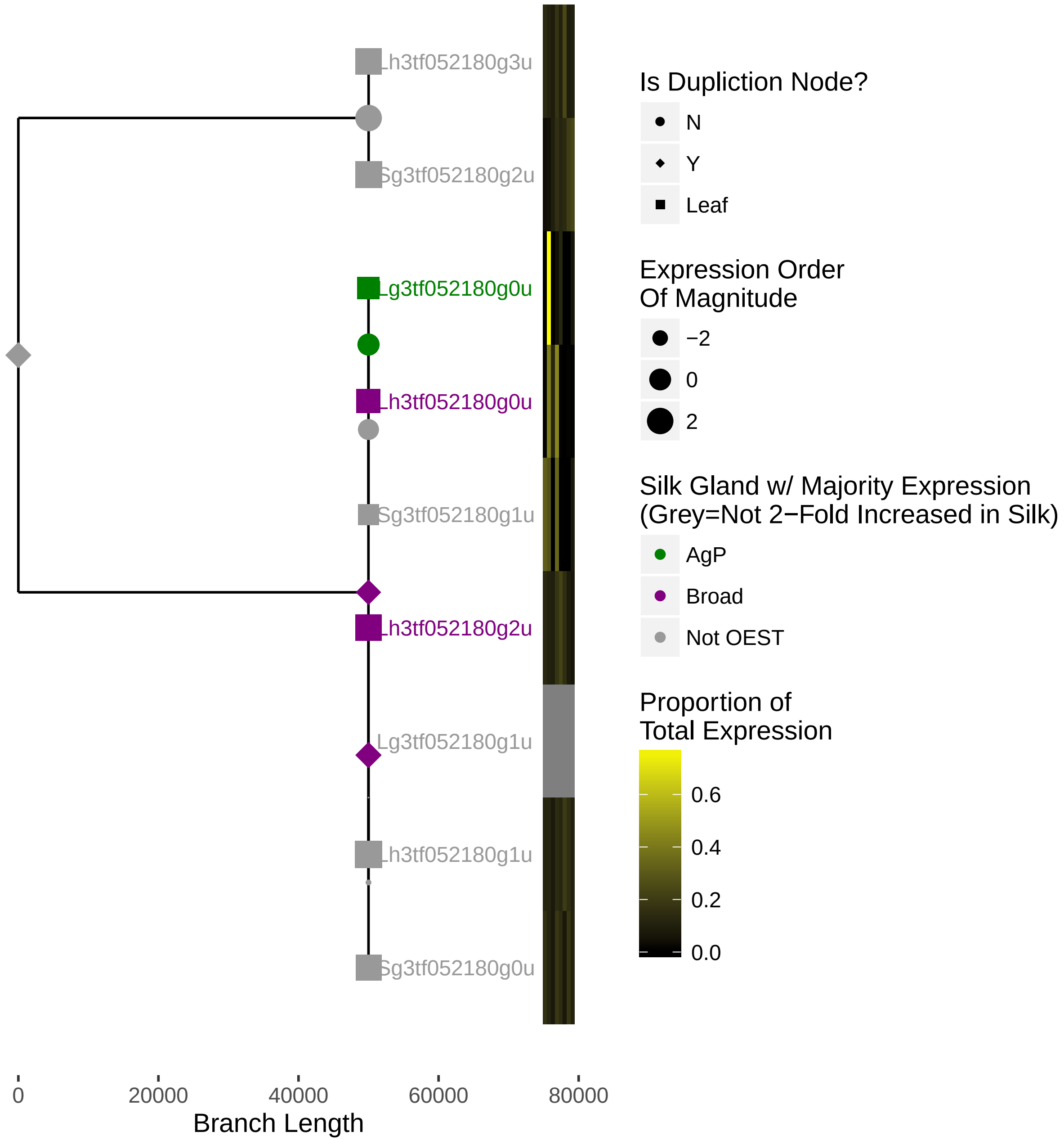

Lh3tf052180g3u
Sg3tf052180g2u
Lg3tf052180g0u
Lh3tf052180g0u
Sg3tf052180g1u
Lh3tf052180g2u
Lg3tf052180g1u
Lh3tf052180g1u
Sg3tf052180g0u
0
20000
40000
60000
80000
Branch Length
Is Dupliction Node?
N
Y
Leaf
Expression Order
Of Magnitude
−2
0
2
Silk Gland w/ Majority Expression
(Grey=Not 2−Fold Increased in Silk)
AgP
Broad
Not OEST
Proportion of
Total Expression
0.6
0.4
0.2
0.0

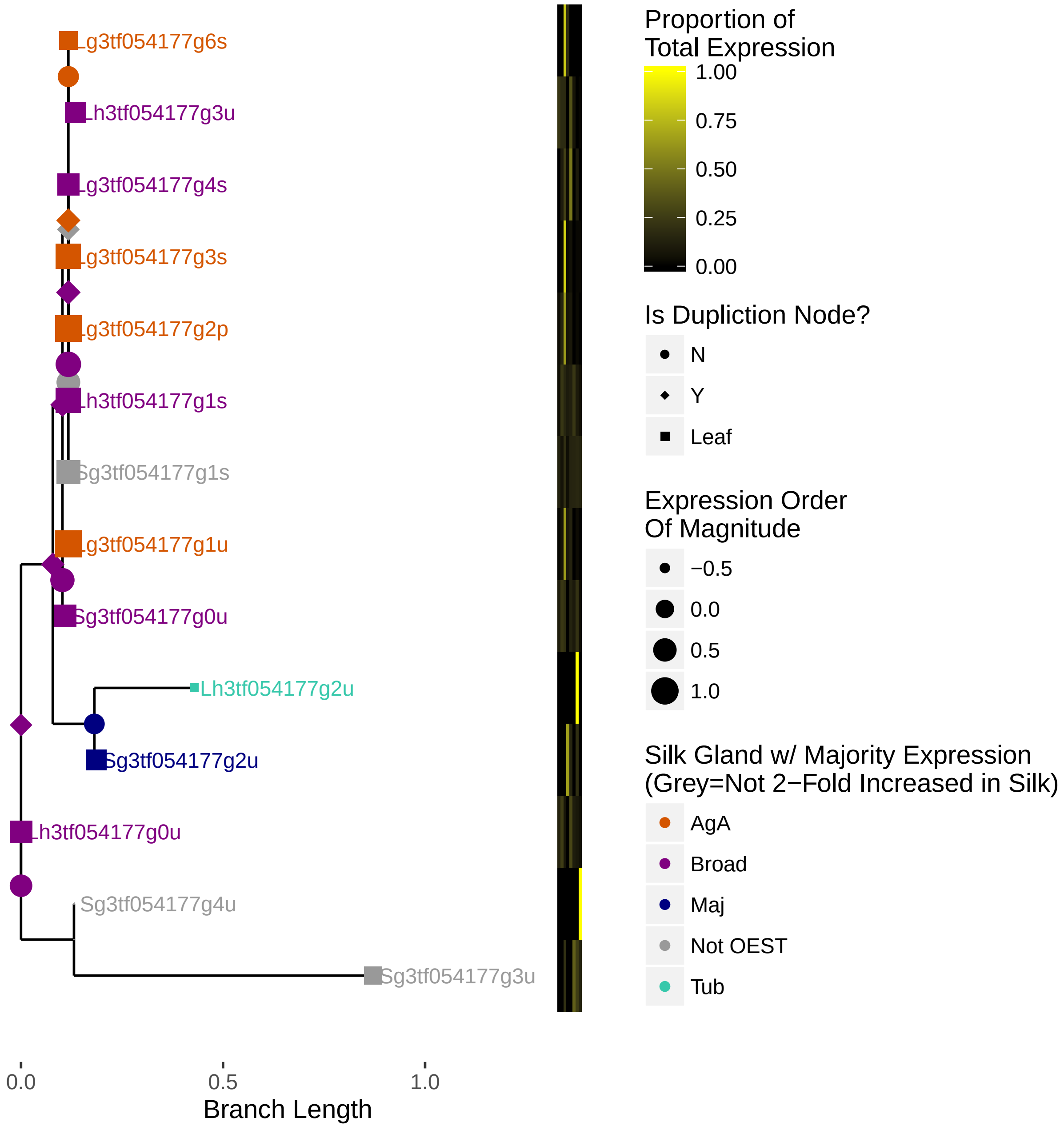

Lg3tf054177g6s
Lh3tf054177g3u
Lg3tf054177g4s
Lg3tf054177g3s
Lg3tf054177g2p
Lh3tf054177g1s
Sg3tf054177g1s
Lg3tf054177g1u
Sg3tf054177g0u
Lh3tf054177g2u
Sg3tf054177g2u
Lh3tf054177g0u
Sg3tf054177g4u
Sg3tf054177g3u
0.0
0.5
1.0
Branch Length
Proportion of
Total Expression
1.00
0.75
0.50
0.25
0.00
Is Dupliction Node?
N
Y
Leaf
Expression Order
Of Magnitude
−0.5
0.0
0.5
1.0
Silk Gland w/ Majority Expression
(Grey=Not 2−Fold Increased in Silk)
AgA
Broad
Maj
Not OEST
Tub

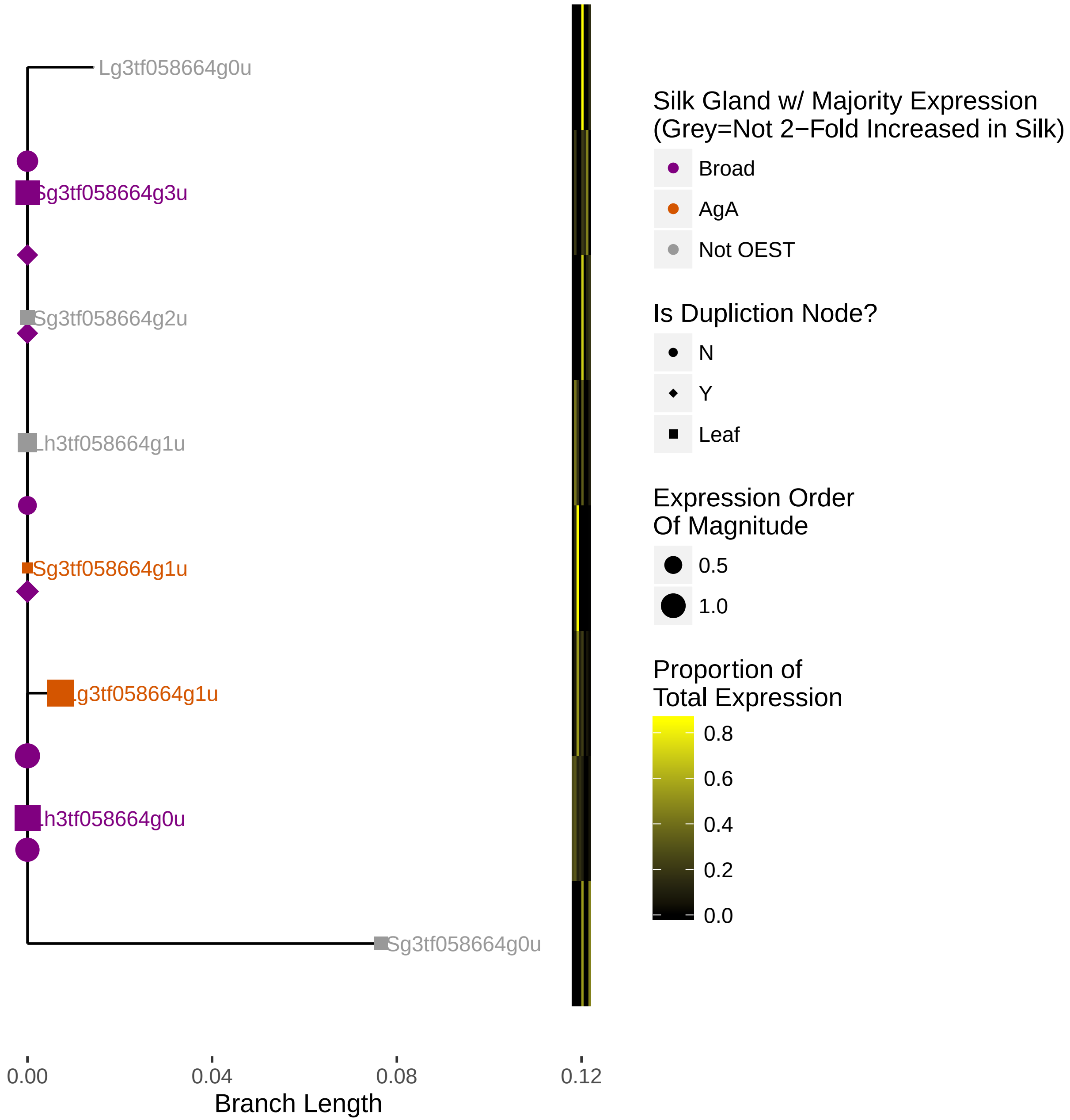

Lg3tf058664g0u
Sg3tf058664g3u
Sg3tf058664g2u
Lh3tf058664g1u
Sg3tf058664g1u
Lg3tf058664g1u
Lh3tf058664g0u
Sg3tf058664g0u
0.00
0.04
0.08
0.12
Branch Length
Silk Gland w/ Majority Expression
(Grey=Not 2−Fold Increased in Silk)
Broad
AgA
Not OEST
Is Dupliction Node?
N
Y
Leaf
Expression Order
Of Magnitude
0.5
1.0
Proportion of
Total Expression
0.8
0.6
0.4
0.2
0.0

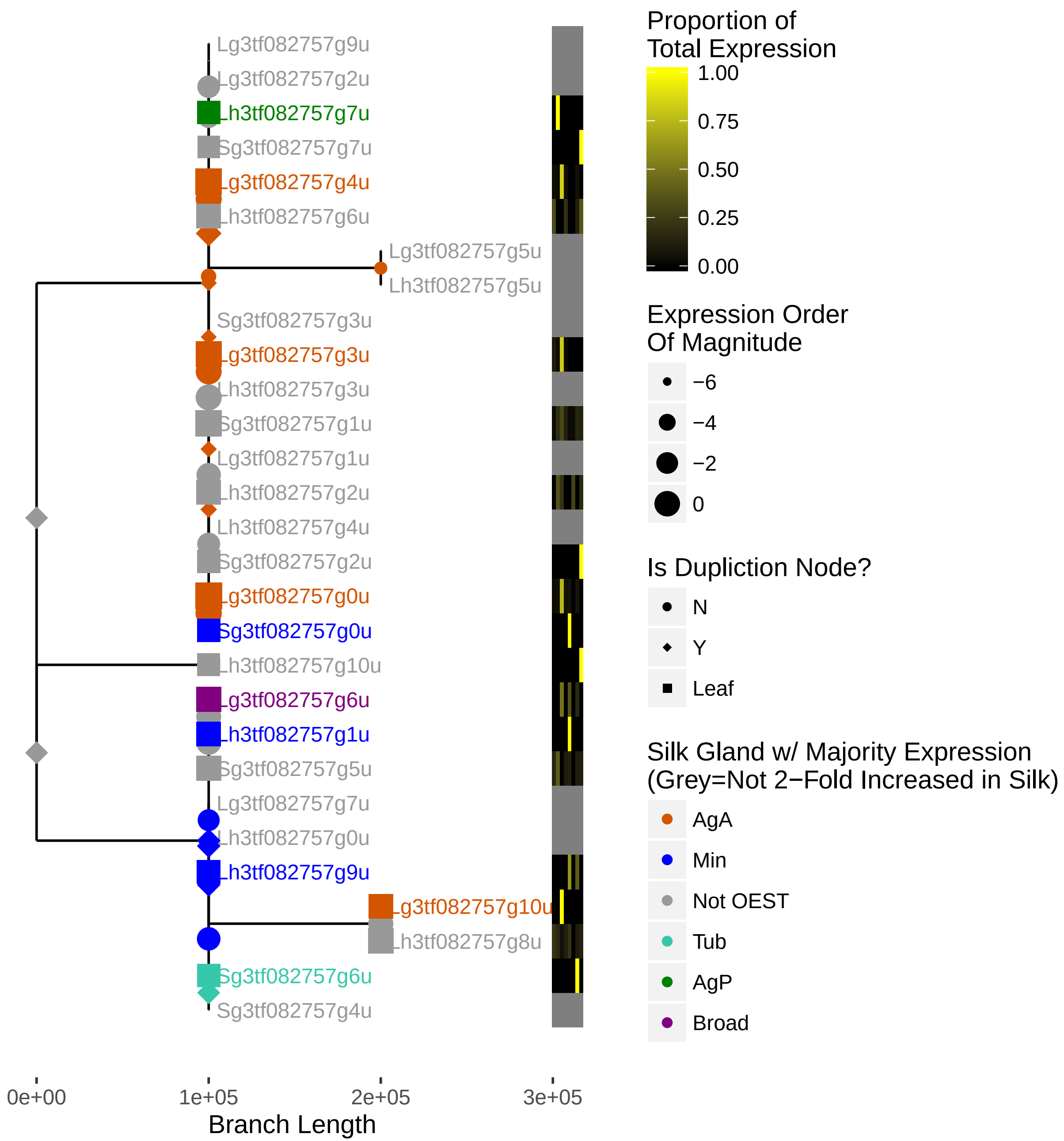

Lg3tf082757g9u
Lg3tf082757g2u
h3tf082757g7u
Sg3tf082757g7u
g3tf082757g4u
Lh3tf082757g6u
Lg3tf082757g5u
Lh3tf082757g5u
Sg3tf082757g3u
g3tf082757g3u
Lh3tf082757g3u
Sg3tf082757g1u
Lg3tf082757g1u
Lh3tf082757g2u
Lh3tf082757g4u
Sg3tf082757g2u
g3tf082757g0u
Sg3tf082757g0u
Lh3tf082757g10u
g3tf082757g6u
h3tf082757g1u
Sg3tf082757g5u
Lg3tf082757g7u
Lh3tf082757g0u
h3tf082757g9u
g3tf082757g10u
h3tf082757g8u
Sg3tf082757g6u
Sg3tf082757g4u
0e+00
1e+05
2e+05
3e+05
Branch Length
Proportion of
Total Expression
1.00
0.75
0.50
0.25
0.00
Expression Order
Of Magnitude
−6
−4
−2
0
Is Dupliction Node?
N
Y
Leaf
Silk Gland w/ Majority Expression
(Grey=Not 2−Fold Increased in Silk)
AgA
Min
Not OEST
Tub
AgP
Broad

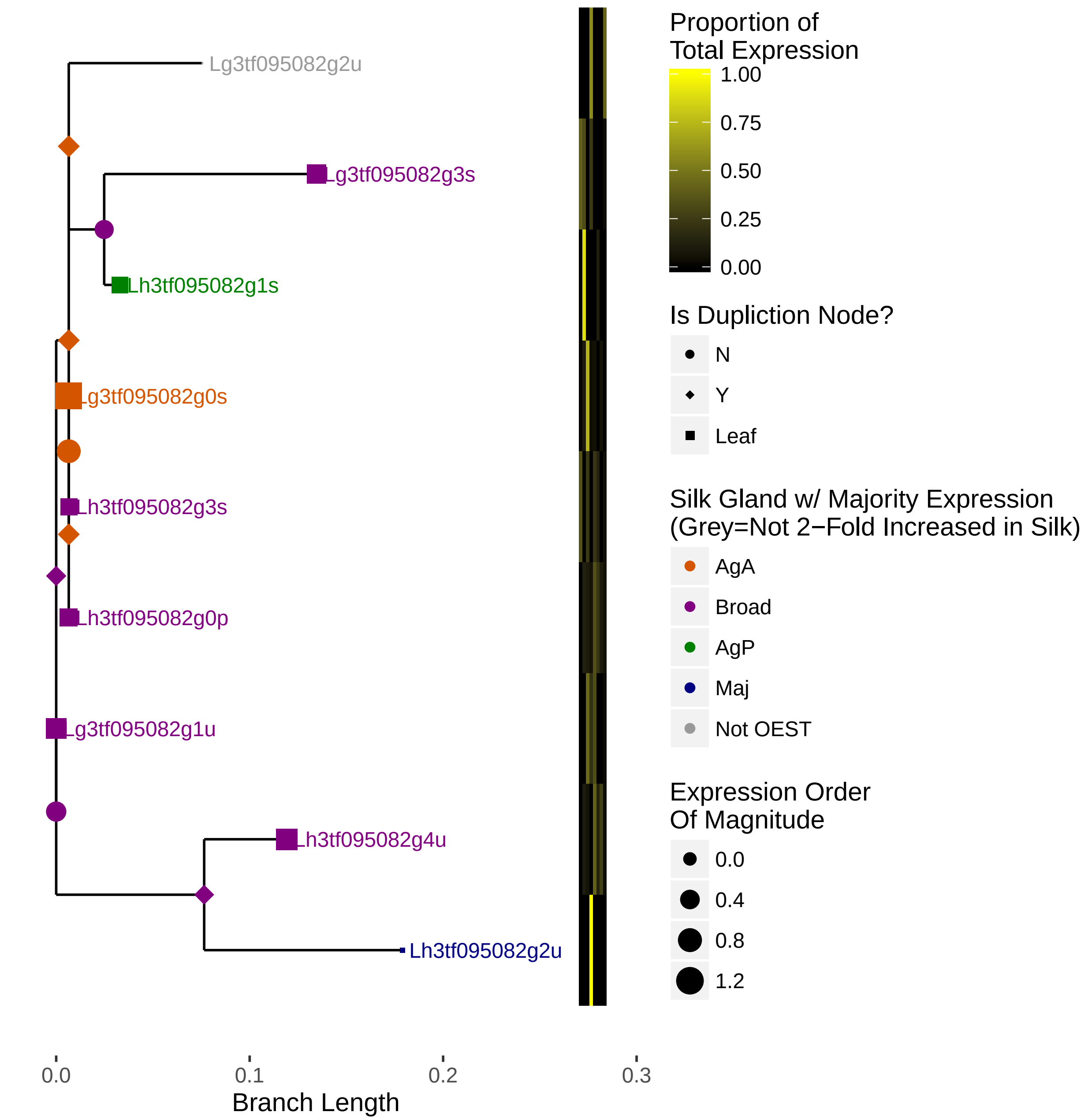
Lg3tf095082g2u
Lg3tf095082g3s
Lh3tf095082g1s
Lg3tf095082g0s
Lh3tf095082g3s
Lh3tf095082g0p
Lg3tf095082g1u
Lh3tf095082g4u
Lh3tf095082g2u
0.0
0.1
0.2
0.3
Branch Length
Proportion of
Total Expression
1.00
0.75
0.50
0.25
0.00
Is Dupliction Node?
N
Y
Leaf
Silk Gland w/ Majority Expression
(Grey=Not 2−Fold Increased in Silk)
AgA
Broad
AgP
Maj
Not OEST
Expression Order
Of Magnitude
0.0
0.4
0.8
1.2

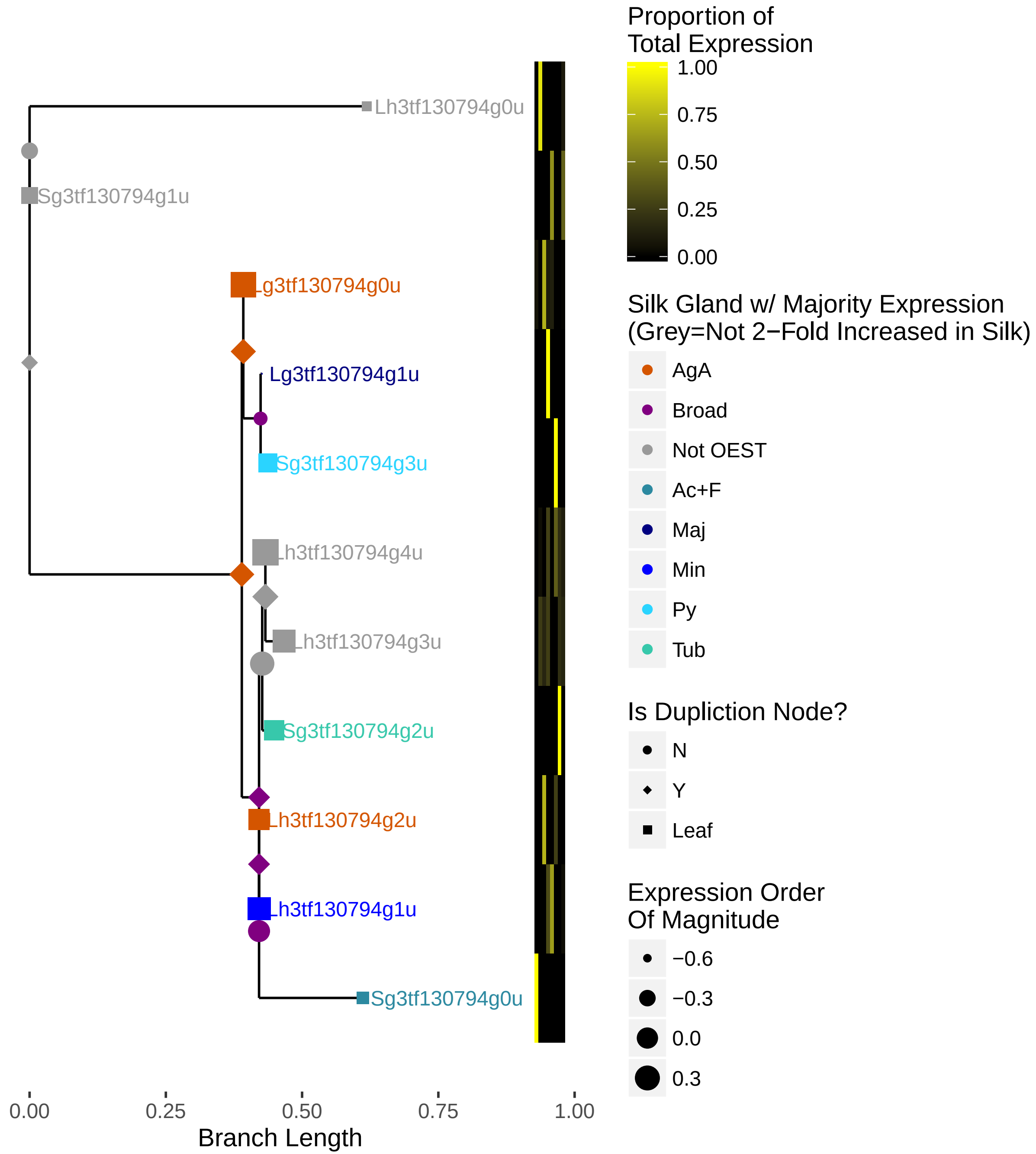
Lh3tf130794g0u
Sg3tf130794g1u
Lg3tf130794g0u
Lg3tf130794g1u
Sg3tf130794g3u
Lh3tf130794g4u
Lh3tf130794g3u
Sg3tf130794g2u
Lh3tf130794g2u
Lh3tf130794g1u
Sg3tf130794g0u
0.00
0.25
0.50
0.75
1.00
Branch Length
Proportion of Total Expression
1.00
0.75
0.50
0.25
0.00
Silk Gland w/ Majority Expression (Grey=Not 2−Fold Increased in Silk)
AgA
Broad
Not OEST
Ac+F
Maj
Min
Py
Tub
Is Dupliction Node?
N
Y
Leaf
Expression Order Of Magnitude
−0.6
−0.3
0.0
0.3

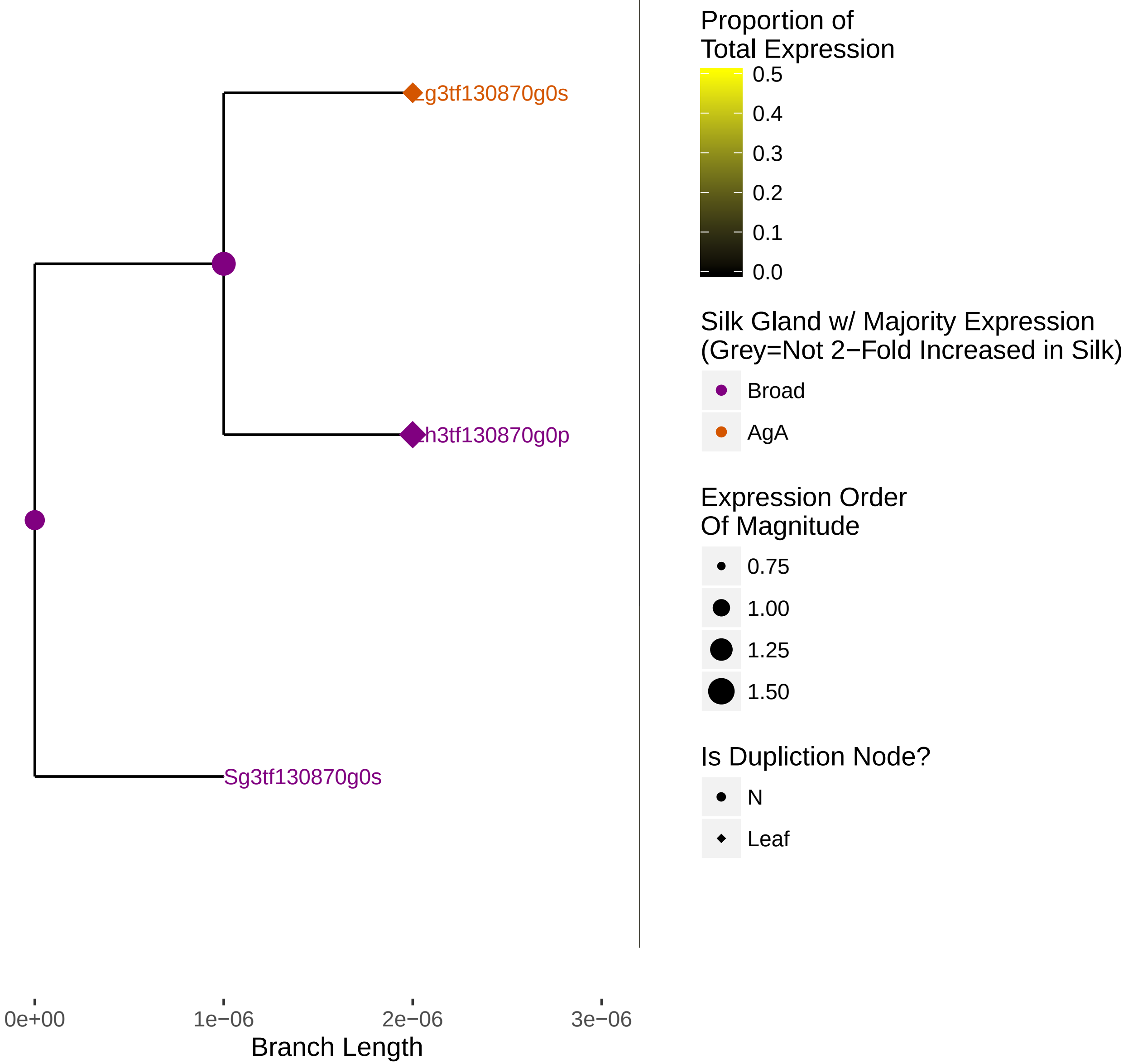

Zg3tf130870g0s
Zh3tf130870g0p
Sg3tf130870g0s
0e+00
1e−06
2e−06
3e−06
Branch Length
Proportion of
Total Expression
0.5
0.4
0.3
0.2
0.1
0.0
Silk Gland w/ Majority Expression
(Grey=Not 2−Fold Increased in Silk)
Broad
AgA
Expression Order
Of Magnitude
0.75
1.00
1.25
1.50
Is Dupliction Node?
N
Leaf

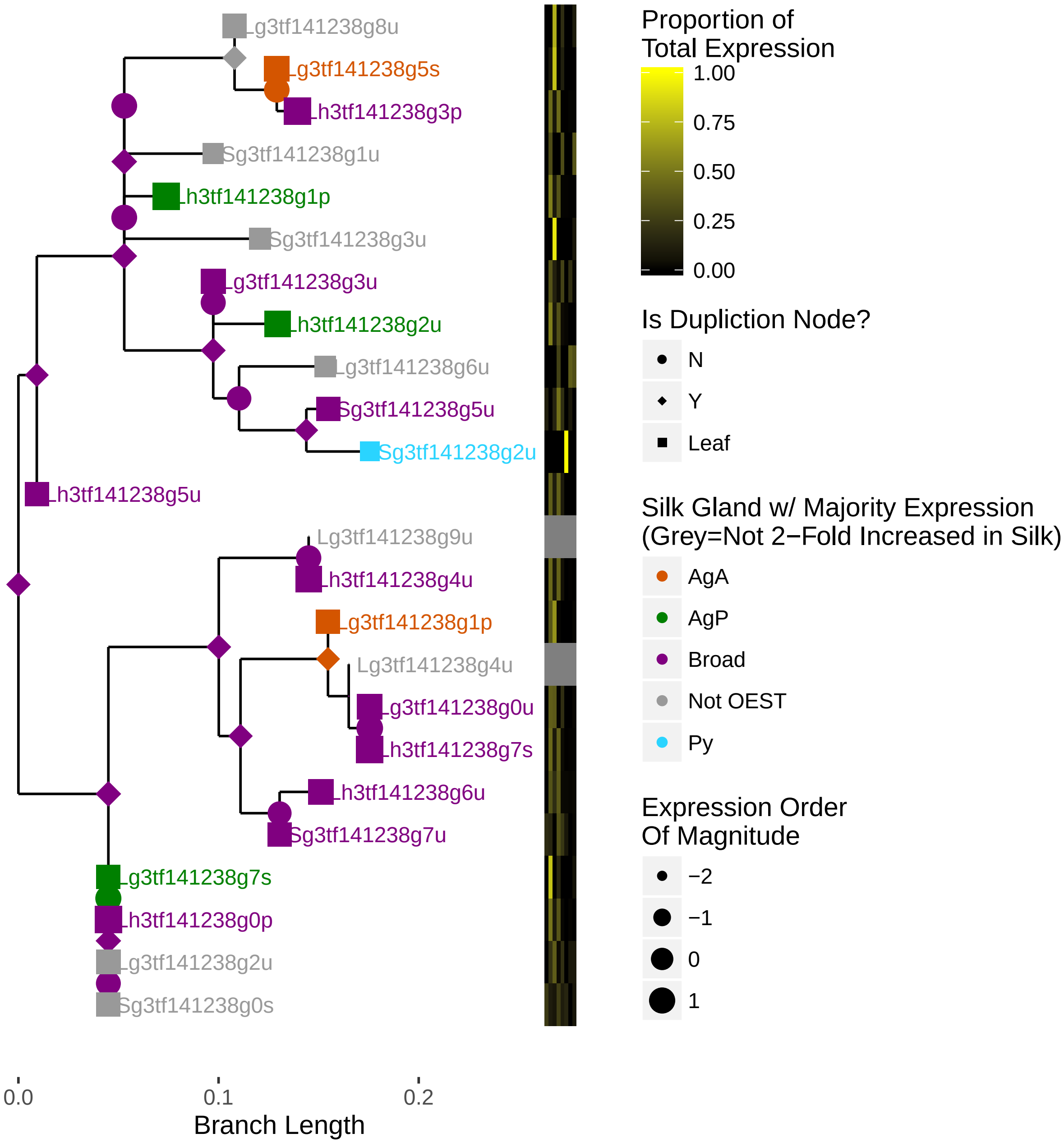

Lg3tf141238g8u
g3tf141238g5s
h3tf141238g3p
Sg3tf141238g1u
h3tf141238g1p
Sg3tf141238g3u
g3tf141238g3u
h3tf141238g2u
Lg3tf141238g6u
Sg3tf141238g5u
Sg3tf141238g2u
h3tf141238g5u
Lg3tf141238g9u
h3tf141238g4u
g3tf141238g1p
Lg3tf141238g4u
g3tf141238g0u
h3tf141238g7s
h3tf141238g6u
Sg3tf141238g7u
g3tf141238g7s
h3tf141238g0p
g3tf141238g2u
Sg3tf141238g0s
0.0
0.1
0.2
Branch Length
Proportion of
Total Expression
1.00
0.75
0.50
0.25
0.00
Is Dupliction Node?
N
Y
Leaf
Silk Gland w/ Majority Expression
(Grey=Not 2−Fold Increased in Silk)
AgA
AgP
Broad
Not OEST
Py
Expression Order
Of Magnitude
−2
−1
0
1

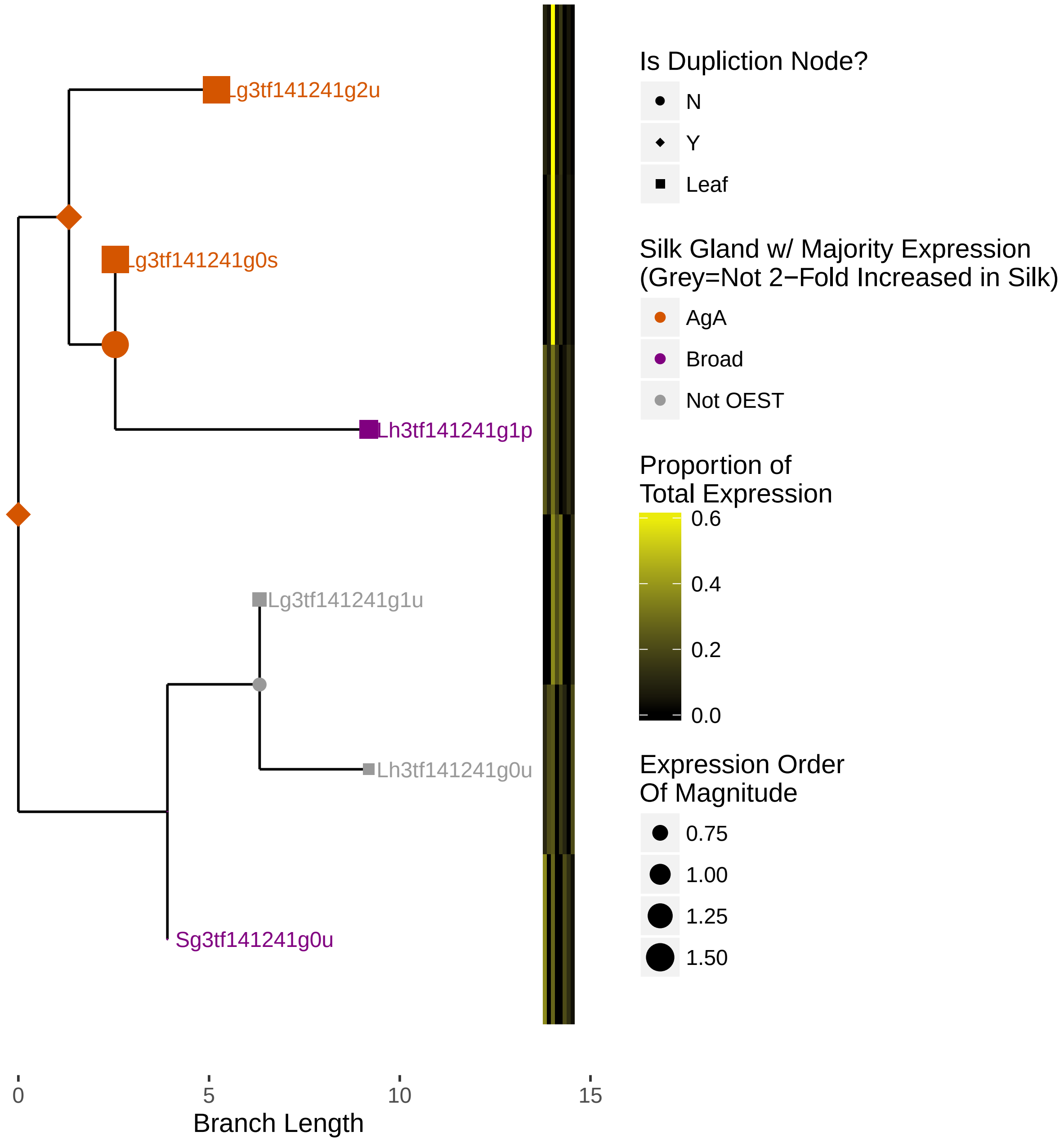
Lg3tf141241g2u
Lg3tf141241g0s
Lh3tf141241g1p
Lg3tf141241g1u
Lh3tf141241g0u
Sg3tf141241g0u
0
5
10
15
Branch Length
Is Dupliction Node?
N
Y
Leaf
Silk Gland w/ Majority Expression
(Grey=Not 2−Fold Increased in Silk)
AgA
Broad
Not OEST
Proportion of
Total Expression
0.6
0.4
0.2
0.0
Expression Order
Of Magnitude
0.75
1.00
1.25
1.50

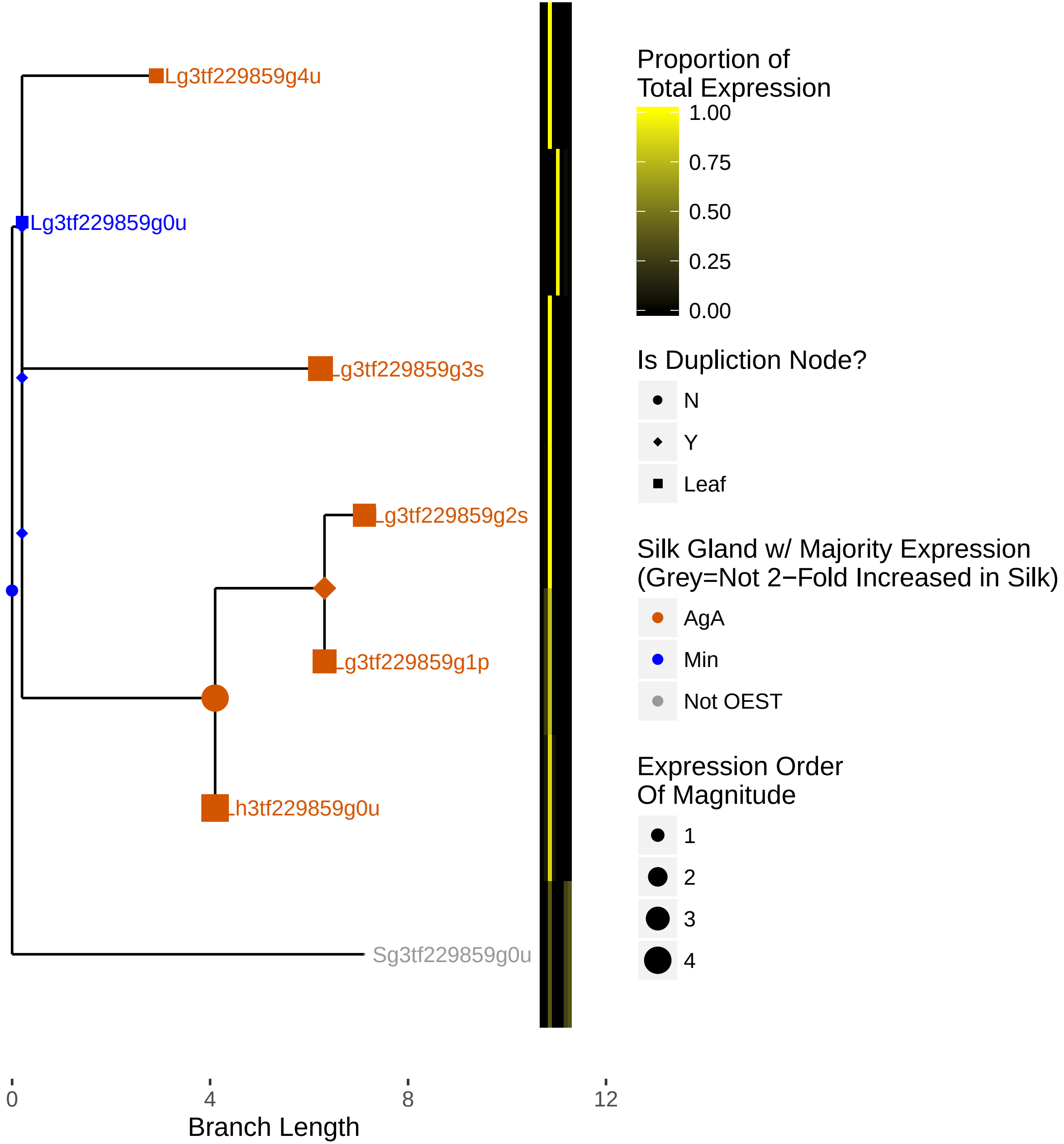
Lg3tf229859g4u
Lg3tf229859g0u
Lg3tf229859g3s
Lg3tf229859g2s
Lg3tf229859g1p
Lh3tf229859g0u
Sg3tf229859g0u
0
4
8
12
Branch Length
Proportion of Total Expression
1.00
0.75
0.50
0.25
0.00
Is Dupliction Node?
N
Y
Leaf
Silk Gland w/ Majority Expression (Grey=Not 2−Fold Increased in Silk)
AgA
Min
Not OEST
Expression Order Of Magnitude
1
2
3
4

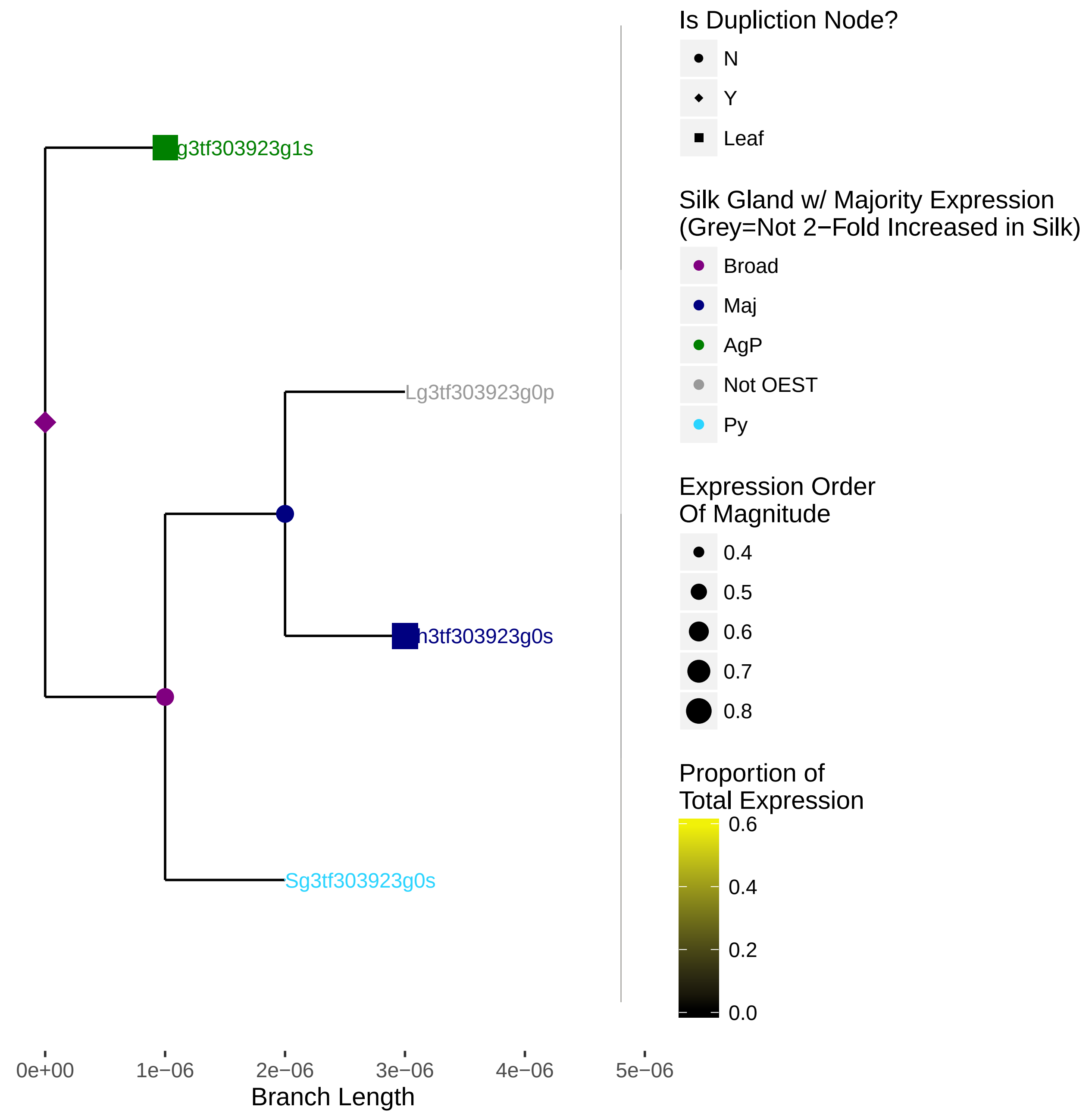

g3tf303923g1s
Lg3tf303923g0p
h3tf303923g0s
Sg3tf303923g0s
0e+00
1e−06
2e−06
3e−06
4e−06
5e−06
Branch Length
Is Dupliction Node?
N
Y
Leaf
Silk Gland w/ Majority Expression
(Grey=Not 2−Fold Increased in Silk)
Broad
Maj
AgP
Not OEST
Py
Expression Order
Of Magnitude
0.4
0.5
0.6
0.7
0.8
Proportion of
Total Expression
0.6
0.4
0.2
0.0
