# Supplementary material for: Evolutionary shifts in gene expression decoupled from gene duplication across functionally distinct spider silk glands
Source: Sci Rep. 2017 Aug 21;7:8393. doi: 10.1038/s41598-017-07388-1 (PMC5566633; doi:10.1038/s41598-017-07388-1)
Supplement: Supplementary file 8 — Supplementary File 7 [file 41598_2017_7388_MOESM8_ESM.pdf]

Supplementary file 7 for

Evolutionary shifts in gene expression decoupled from gene duplication across functionally distinct spider silk glands

Thomas H. Clarke, Jessica E. Garb, Robert A. Haney, R. Crystal Chaw, Cheryl Y. Hayashi, Nadia A. Ayoub

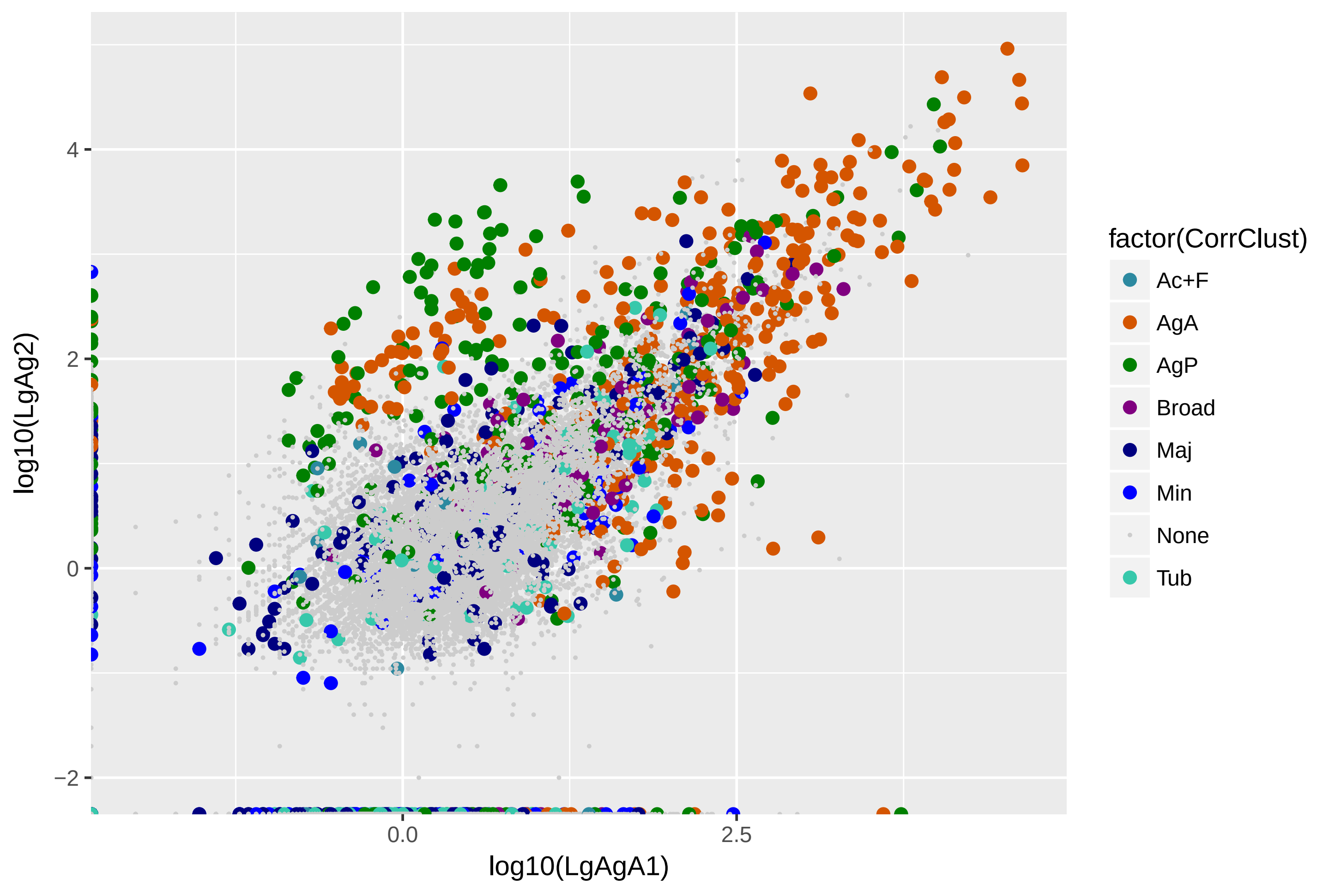

4
2
0
−2
0.0
2.5
log10(LgAg2)
log10(LgAgA1)
factor(CorrClust)
Ac+F
AgA
AgP
Broad
Maj
Min
None
Tub

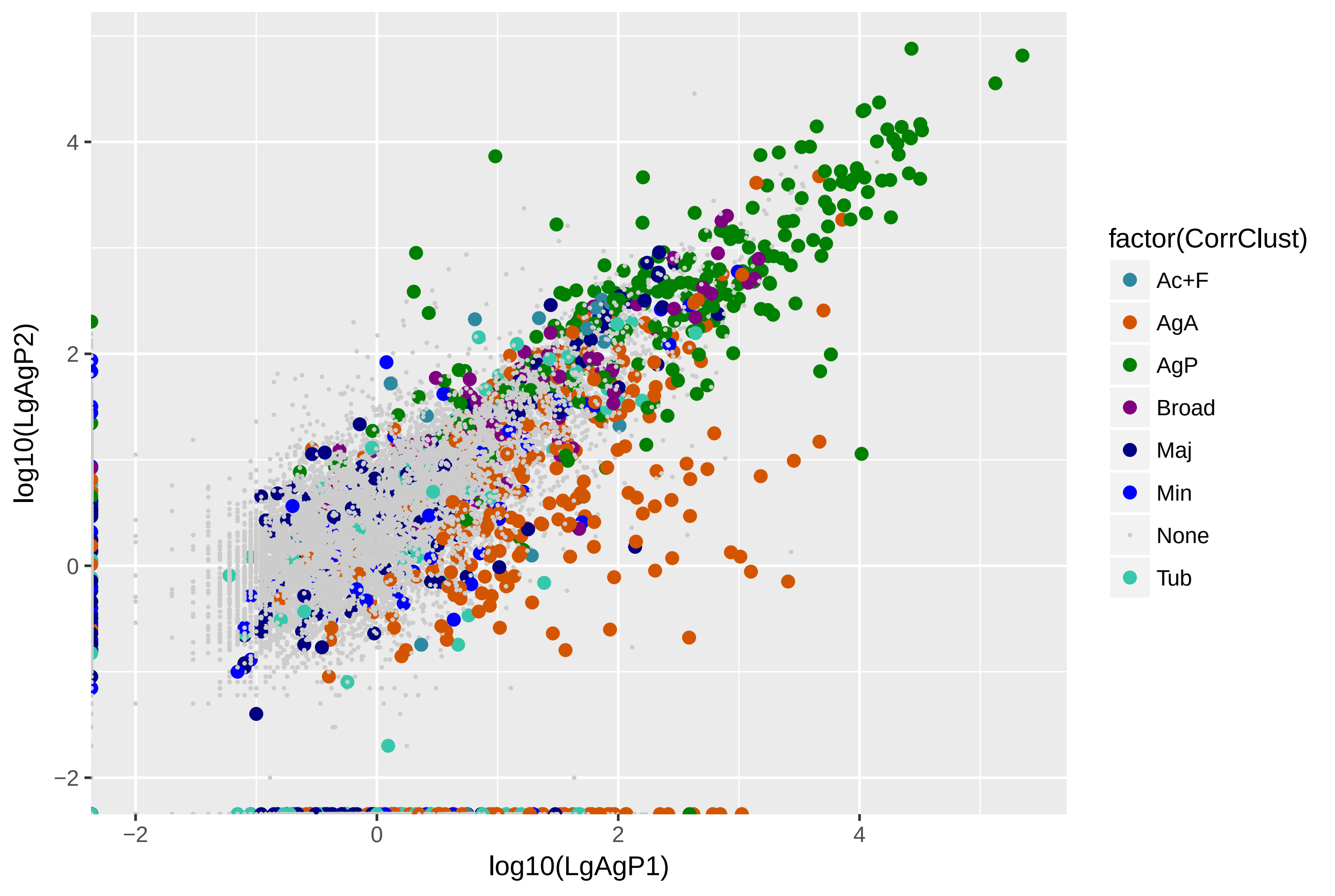

4
2
0
−2
log10(LgAgP2)
−2
0
2
4
log10(LgAgP1)
factor(CorrClust)
Ac+F
AgA
AgP
Broad
Maj
Min
None
Tub

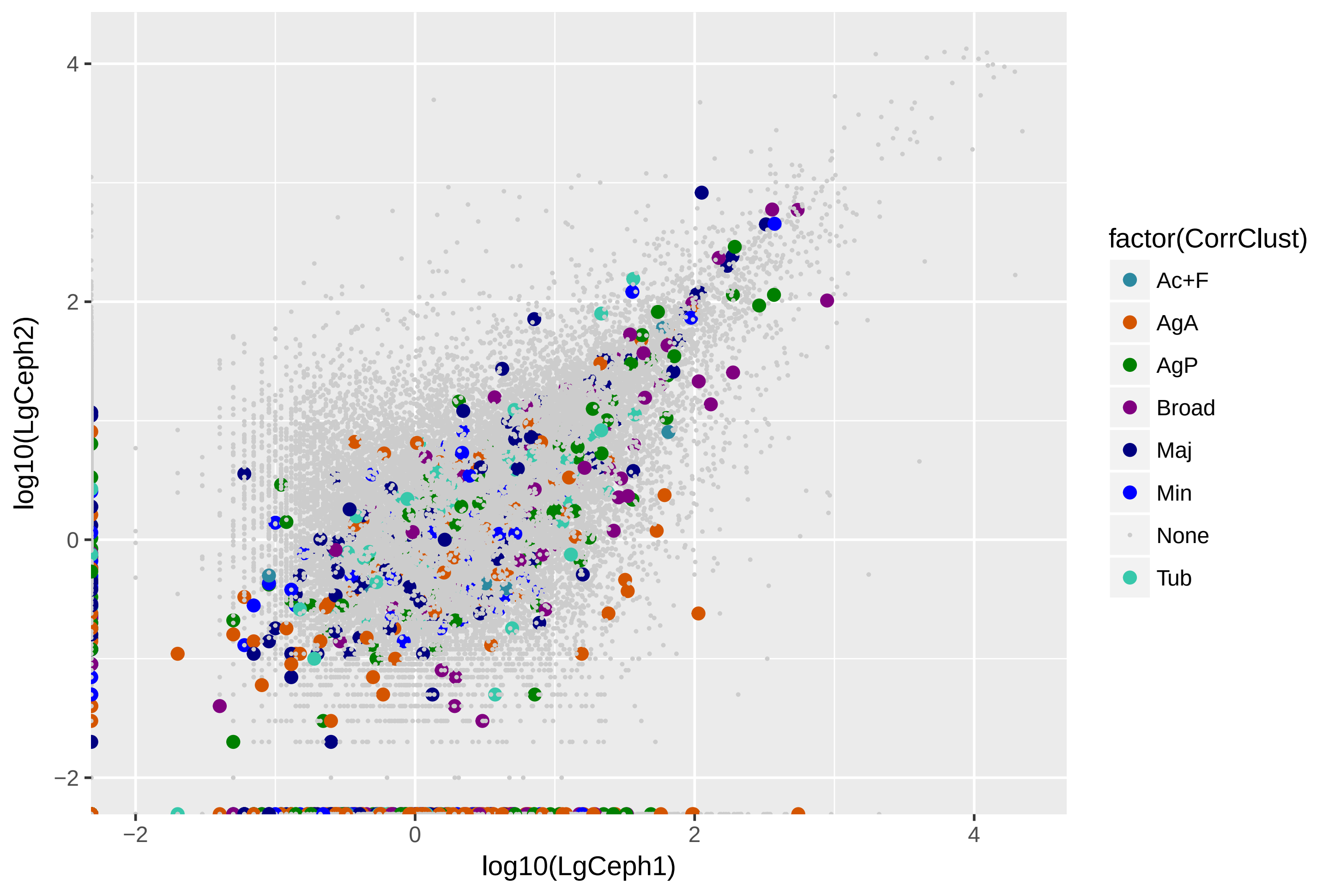

log10(LgCeph2)
log10(LgCeph1)
4
2
0
−2
−2
0
2
4
factor(CorrClust)
Ac+F
AgA
AgP
Broad
Maj
Min
None
Tub

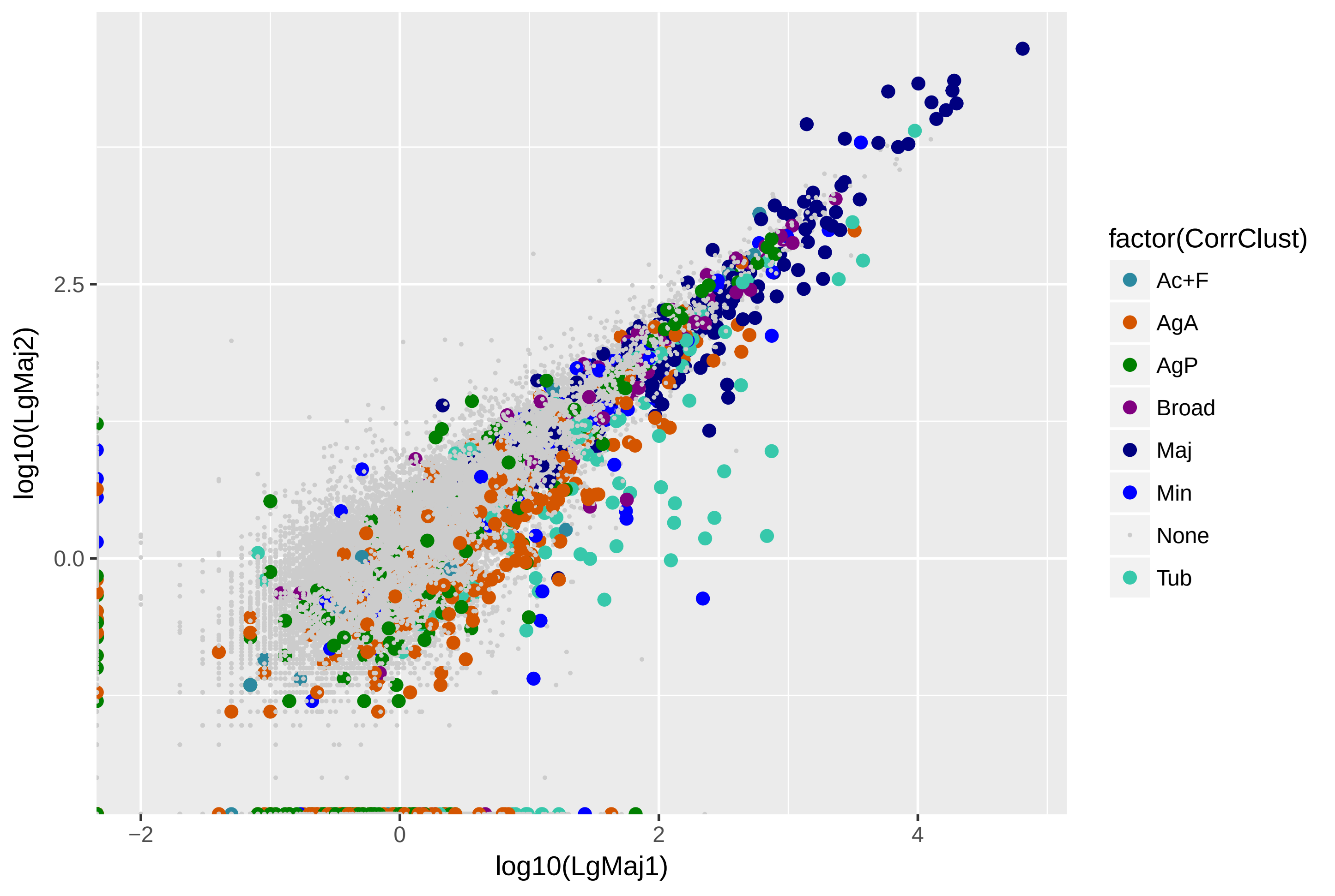

log10(LgMaj2)
2.5
0.0
−2
0
2
4
log10(LgMaj1)
factor(CorrClust)
Ac+F
AgA
AgP
Broad
Maj
Min
None
Tub

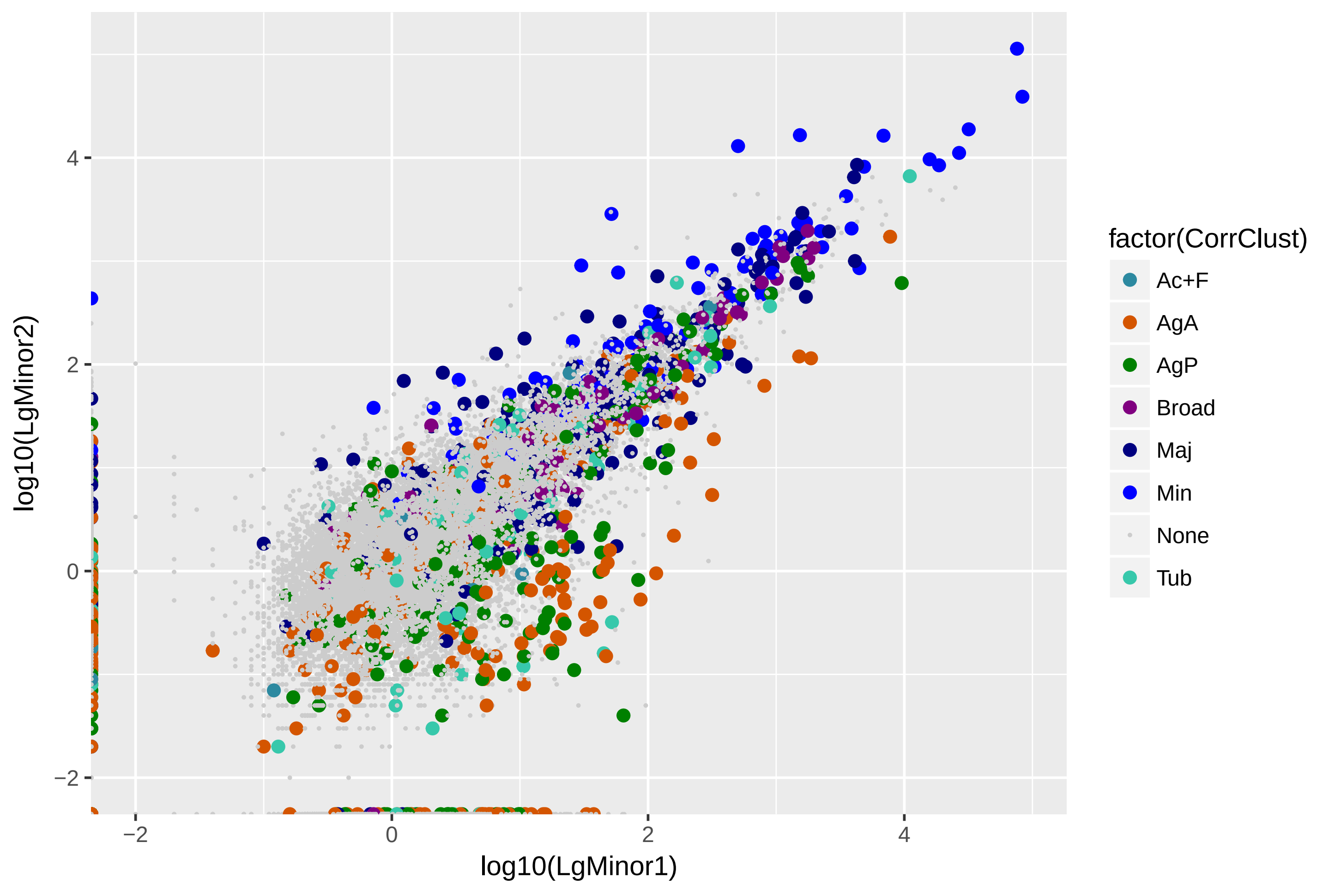

factor(CorrClust)
Ac+F
AgA
AgP
Broad
Maj
Min
None
Tub
log10(LgMinor2)
log10(LgMinor1)
4
2
0
−2
−2
0
2
4

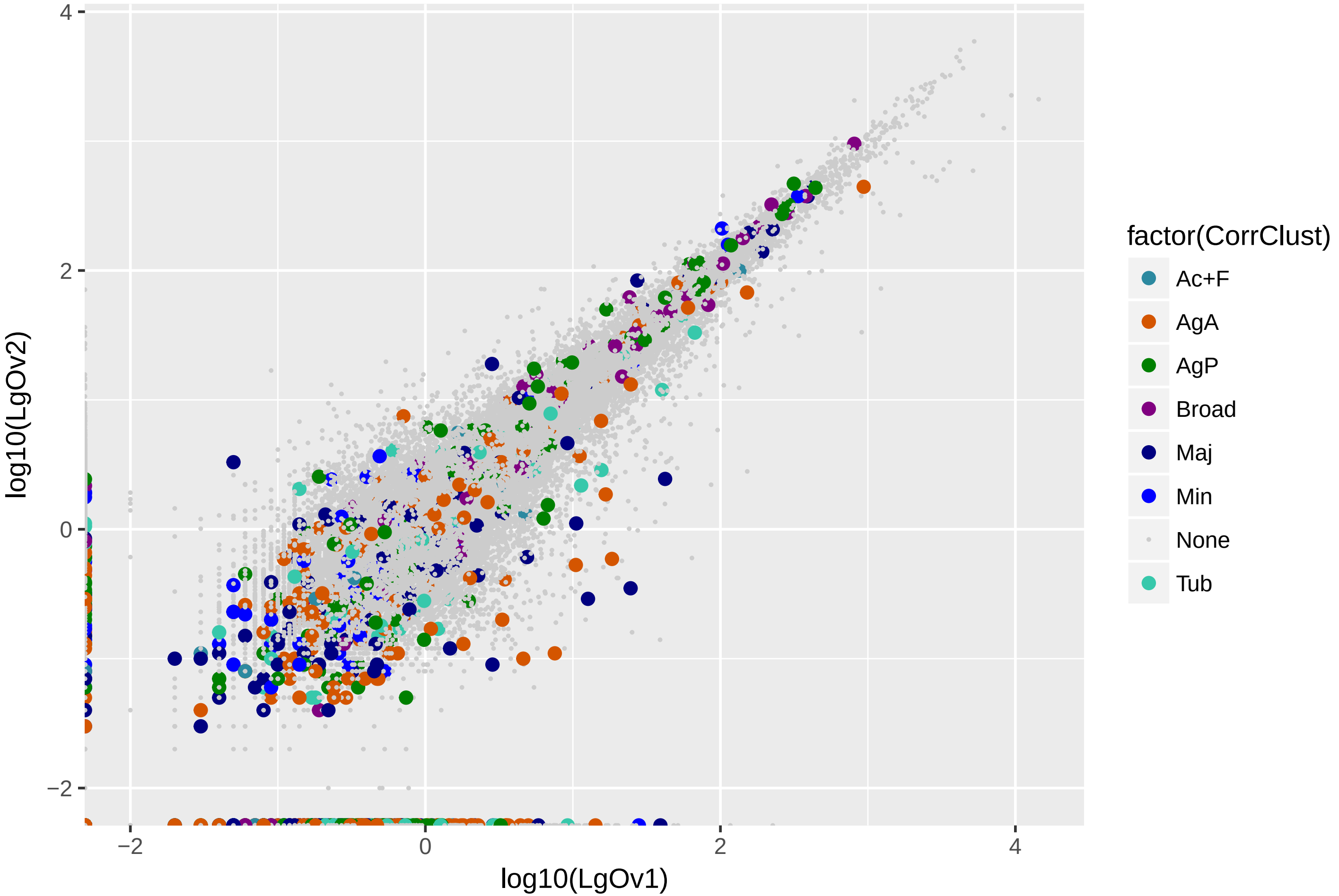
4
2
0
−2
−2
0
2
4
log10(LgOv2)
log10(LgOv1)
factor(CorrClust)
Ac+F
AgA
AgP
Broad
Maj
Min
None
Tub

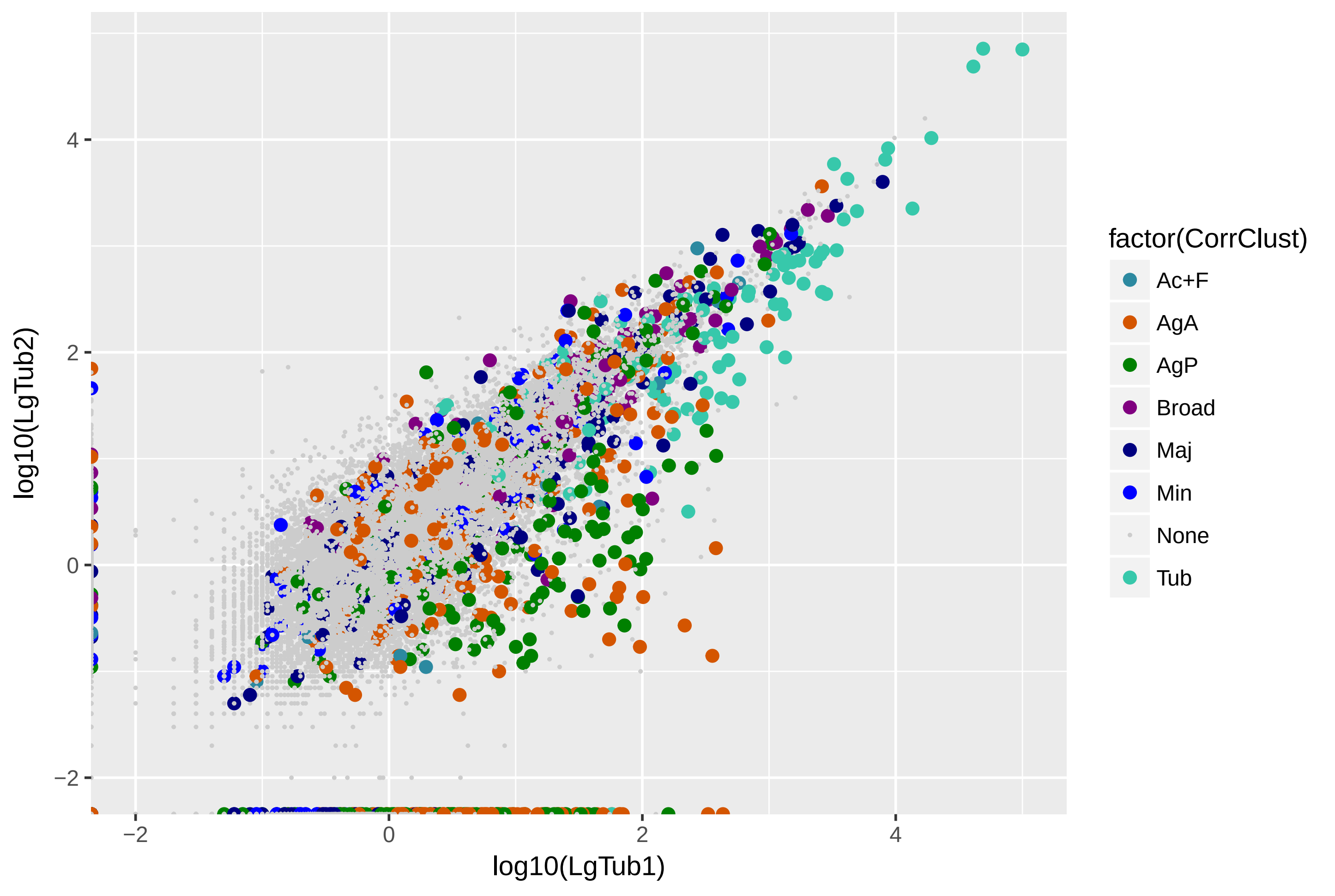
log10(LgTub2)
log10(LgTub1)
4
2
0
−2
−2
0
2
4
factor(CorrClust)
Ac+F
AgA
AgP
Broad
Maj
Min
None
Tub

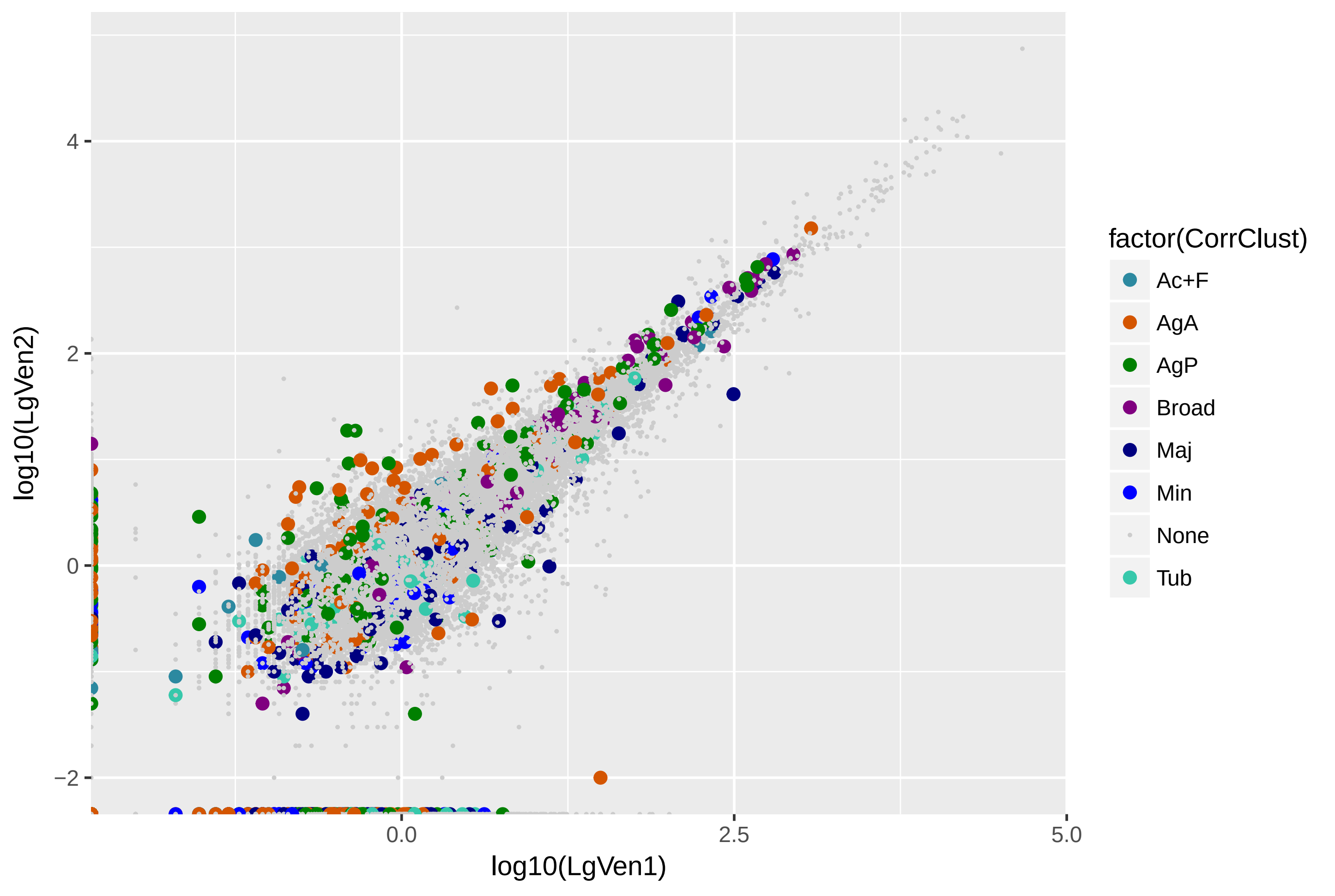

factor(CorrClust)
Ac+F
AgA
AgP
Broad
Maj
Min
None
Tub
log10(LgVen2)
log10(LgVen1)
4
2
0
−2
0.0
2.5
5.0

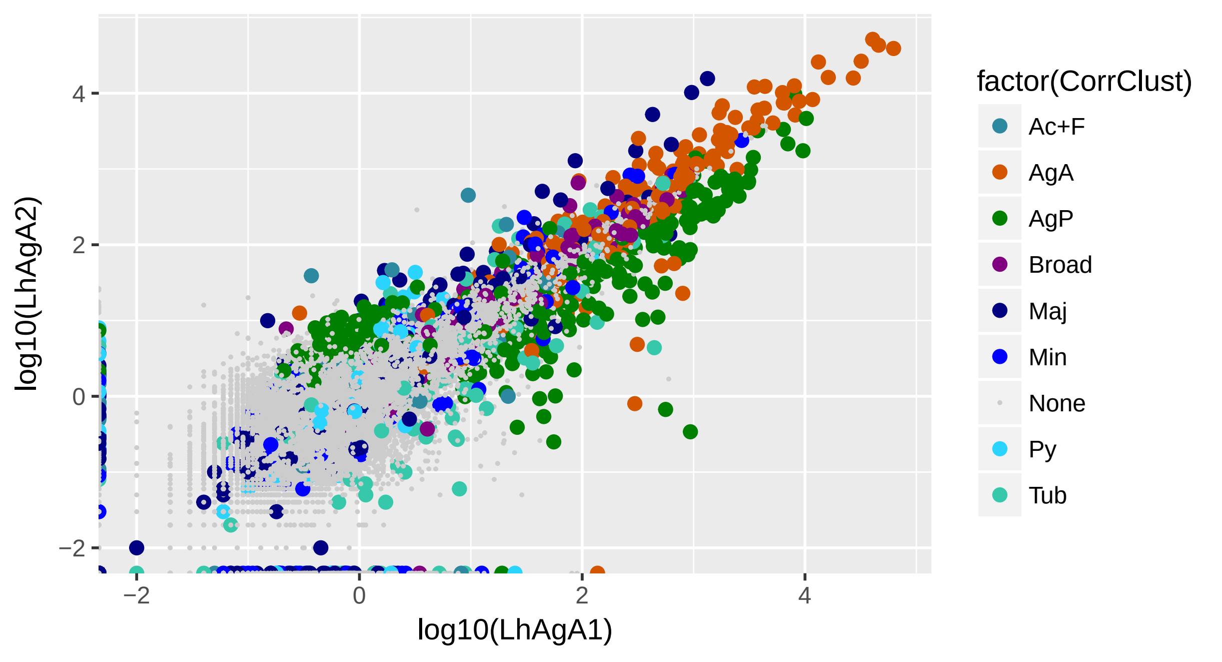

log10(LhAgA2)
log10(LhAgA1)
4
2
0
−2
−2
0
2
4
factor(CorrClust)
Ac+F
AgA
AgP
Broad
Maj
Min
None
Py
Tub

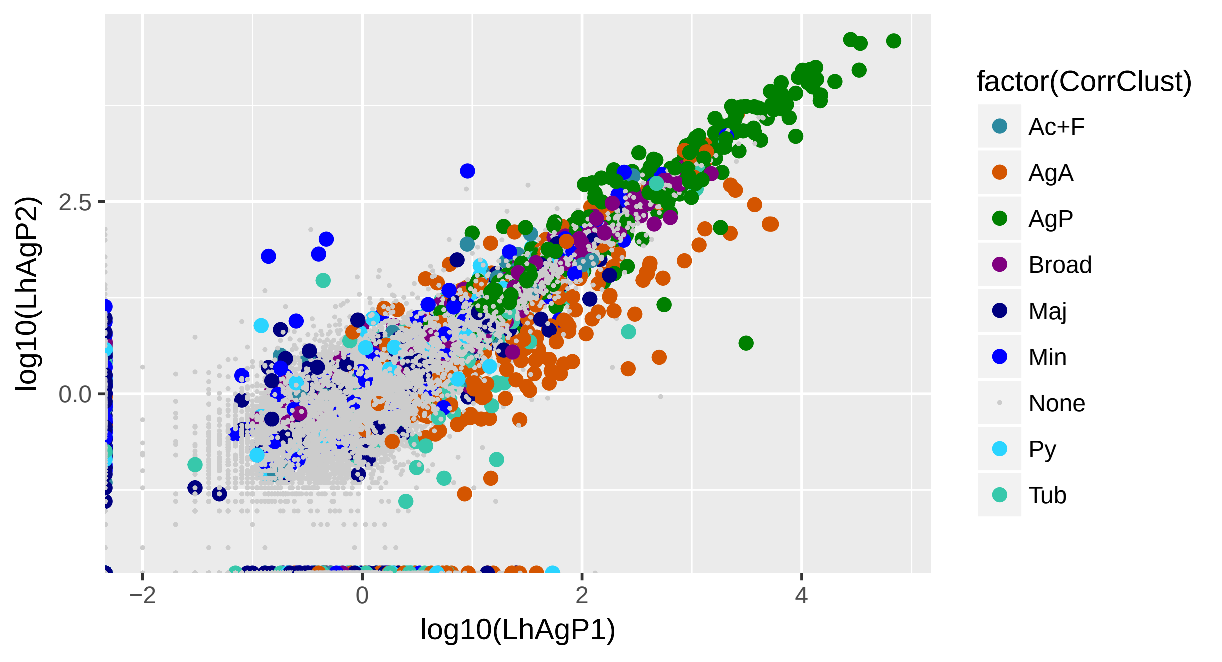
log10(LhAgP2)
2.5
0.0
−2
0
2
4
log10(LhAgP1)
factor(CorrClust)
Ac+F
AgA
AgP
Broad
Maj
Min
None
Py
Tub

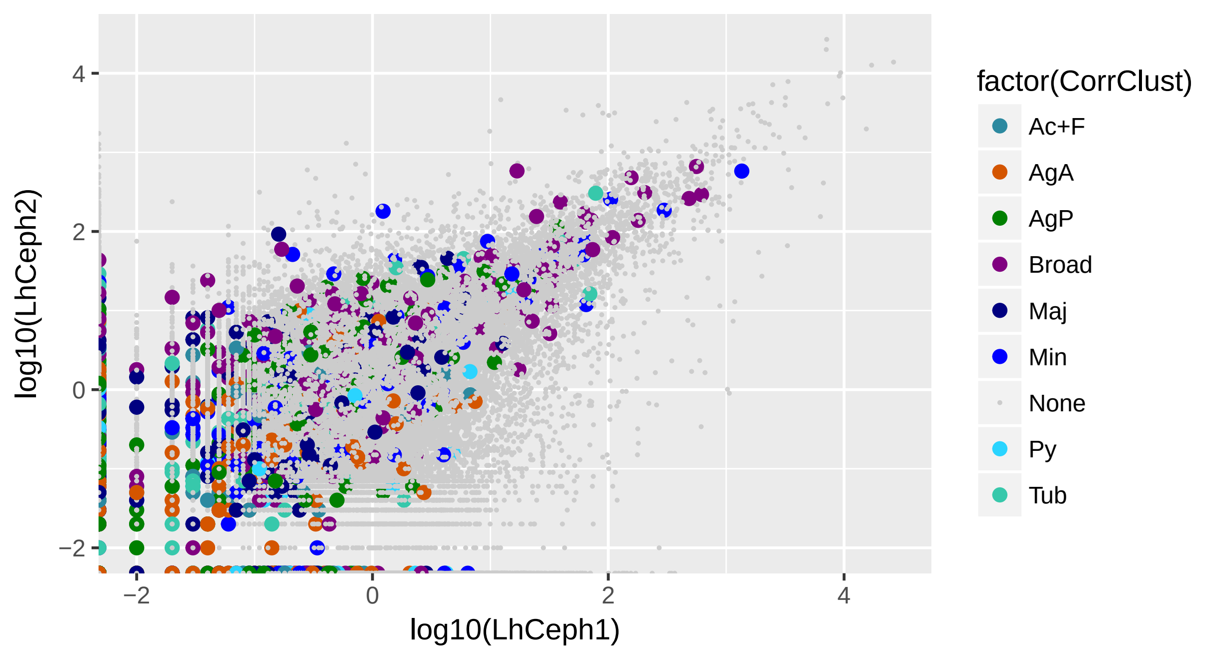
factor(CorrClust)
Ac+F
AgA
AgP
Broad
Maj
Min
None
Py
Tub
log10(LhCeph2)
log10(LhCeph1)
4
2
0
−2
−2
0
2
4

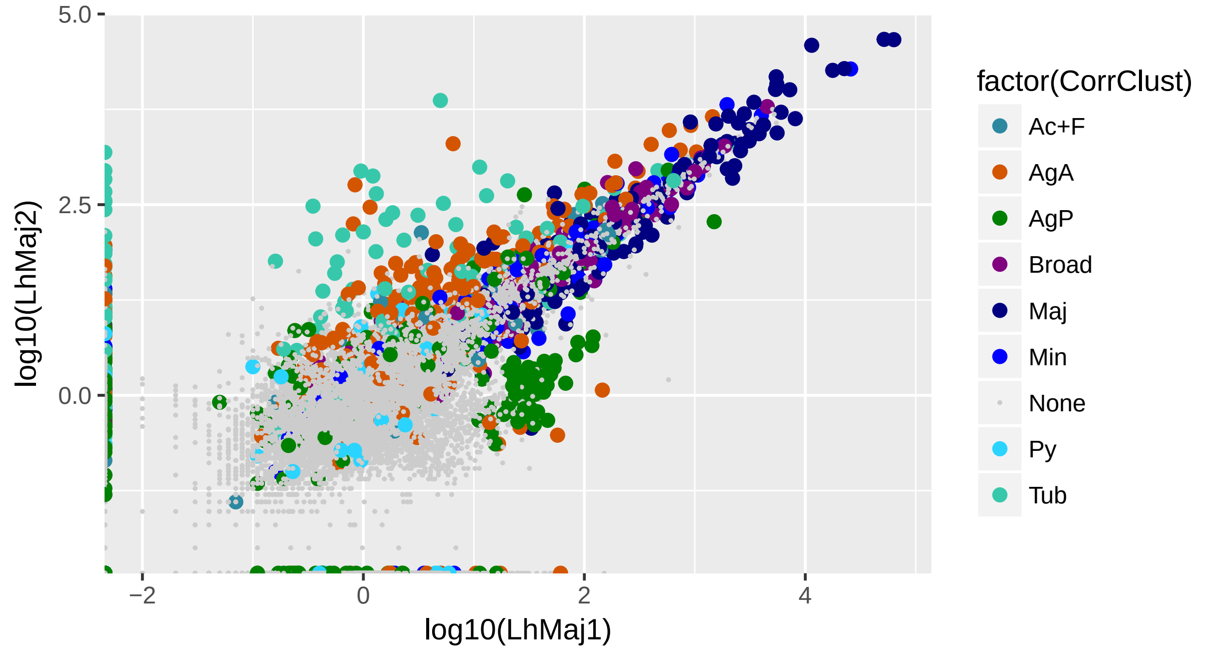

log10(LhMaj2)
5.0
2.5
0.0
−2
0
2
4
log10(LhMaj1)
factor(CorrClust)
Ac+F
AgA
AgP
Broad
Maj
Min
None
Py
Tub

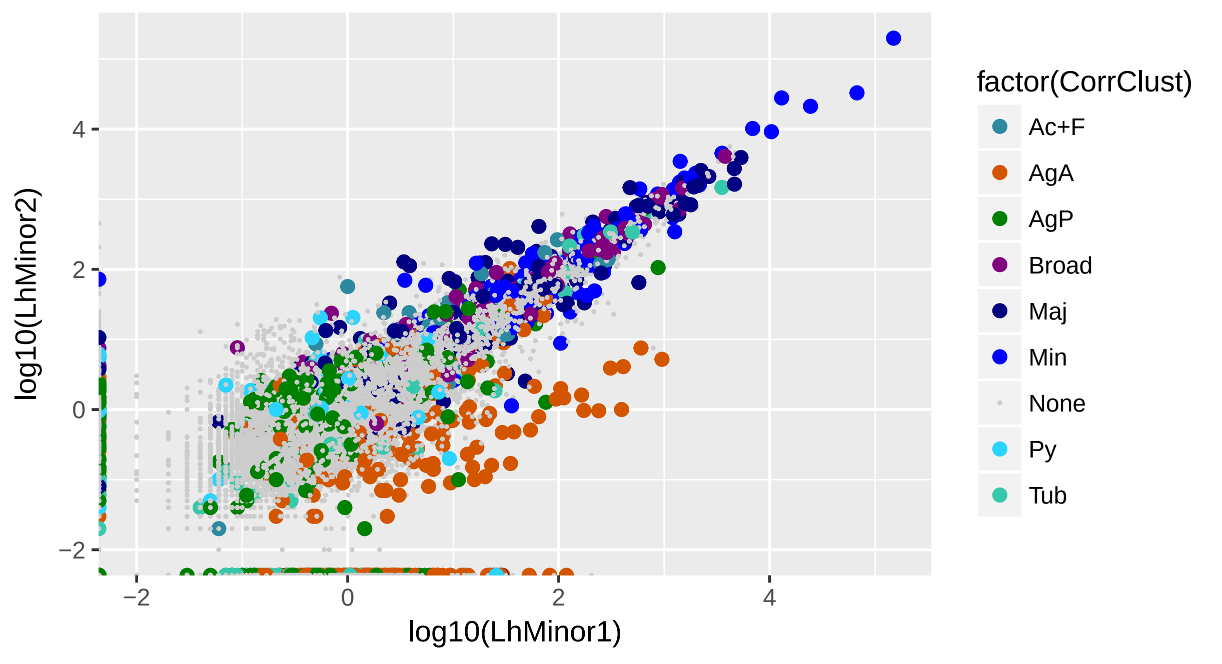

factor(CorrClust)
Ac+F
AgA
AgP
Broad
Maj
Min
None
Py
Tub
log10(LhMinor2)
log10(LhMinor1)
4
2
0
−2
−2
0
2
4

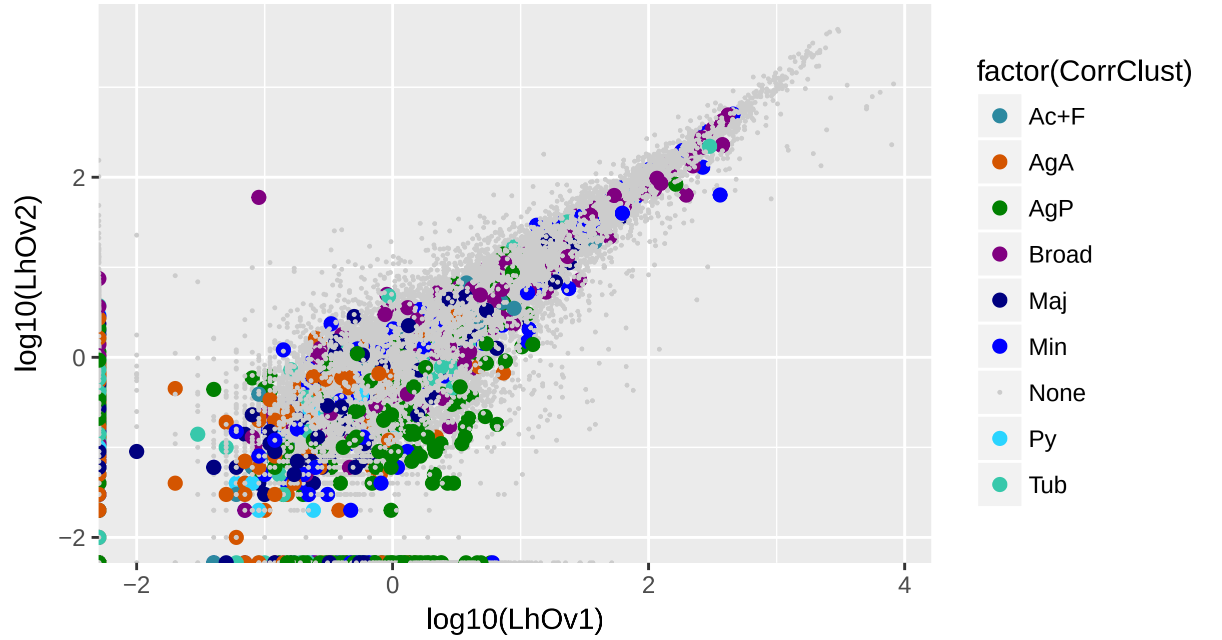

factor(CorrClust)
Ac+F
AgA
AgP
Broad
Maj
Min
None
Py
Tub
log10(LhOv2)
log10(LhOv1)
2
0
−2
−2
0
2
4

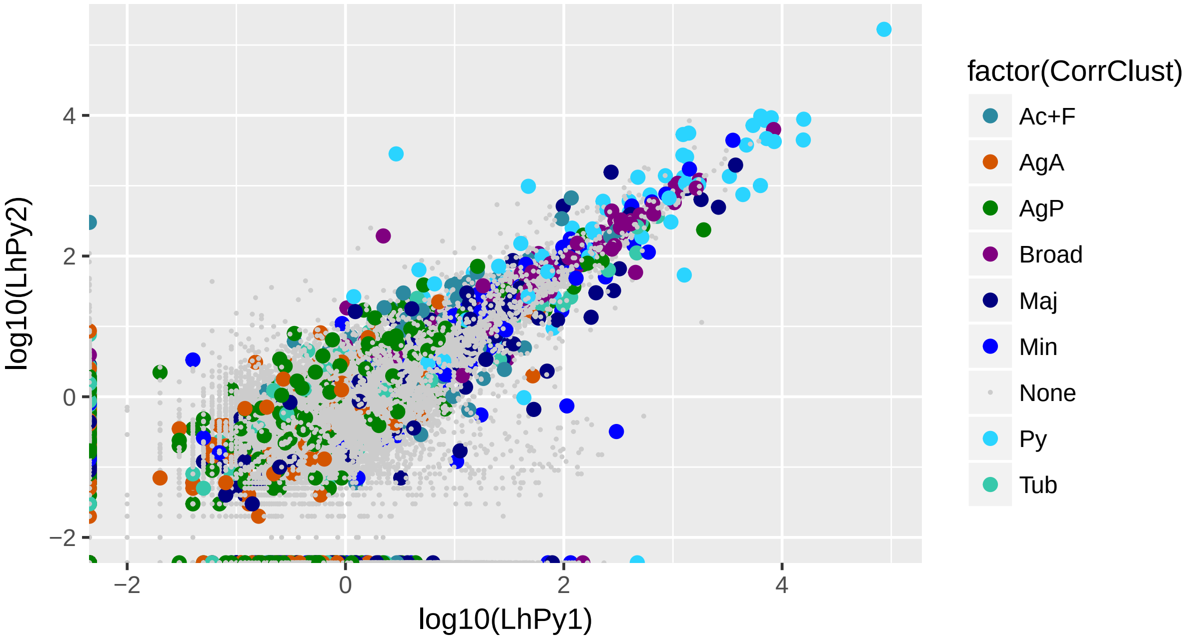

factor(CorrClust)
Ac+F
AgA
AgP
Broad
Maj
Min
None
Py
Tub
log10(LhPy2)
log10(LhPy1)
4
2
0
−2
−2
0
2
4

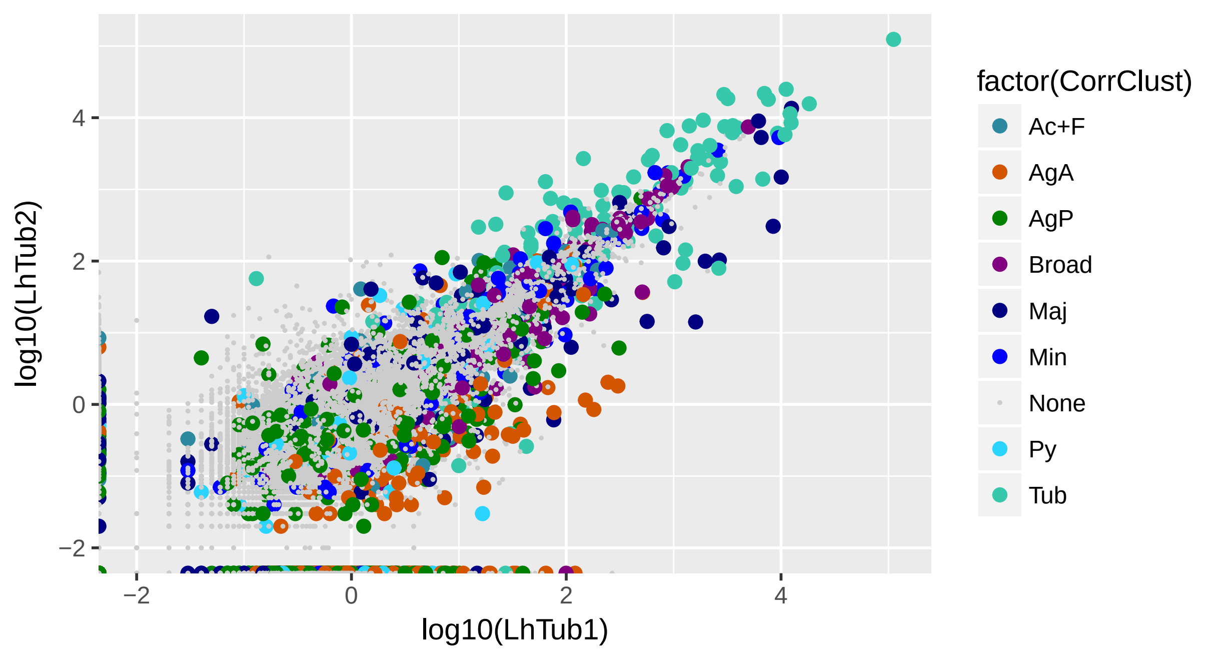

factor(CorrClust)
Ac+F
AgA
AgP
Broad
Maj
Min
None
Py
Tub
log10(LhTub2)
log10(LhTub1)
4
2
0
−2
−2
0
2
4

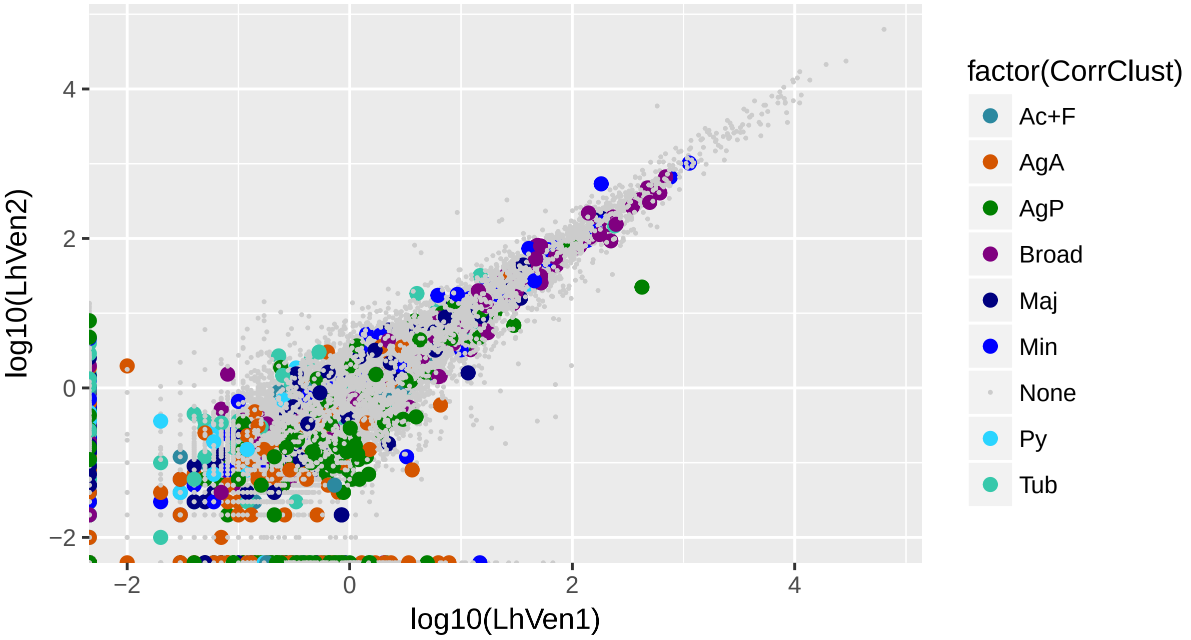
factor(CorrClust)
Ac+F
AgA
AgP
Broad
Maj
Min
None
Py
Tub
log10(LhVen2)
log10(LhVen1)
4
2
0
−2
−2
0
2
4

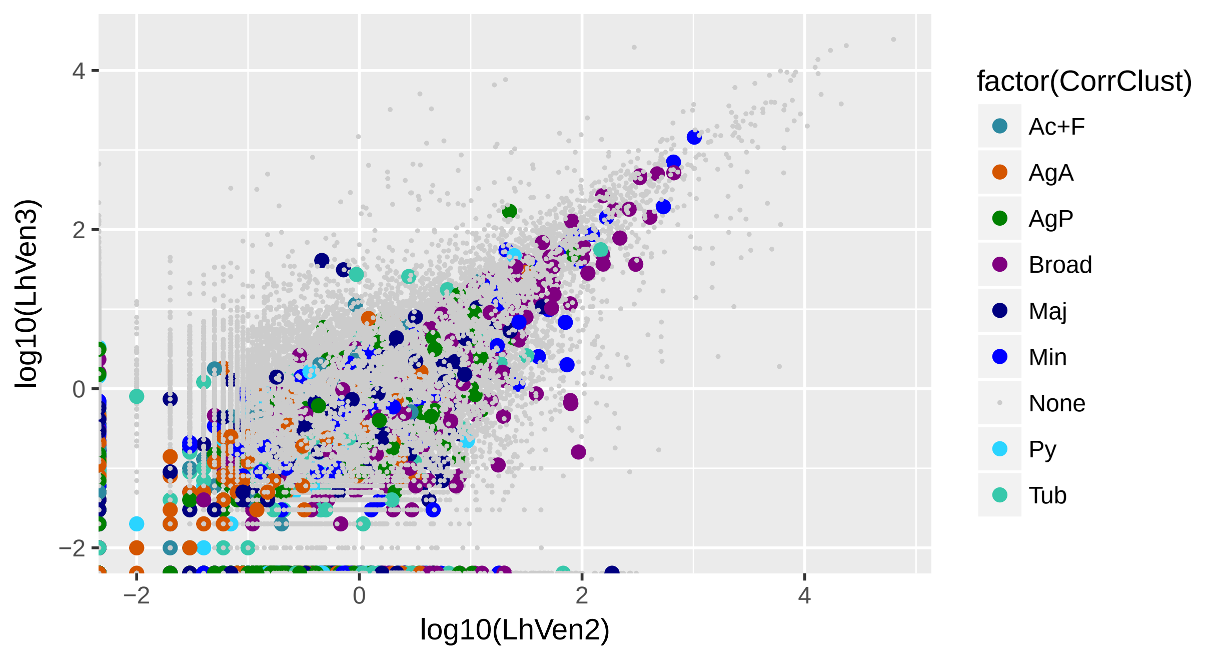
factor(CorrClust)
Ac+F
AgA
AgP
Broad
Maj
Min
None
Py
Tub
log10(LhVen3)
log10(LhVen2)
4
2
0
−2
−2
0
2
4

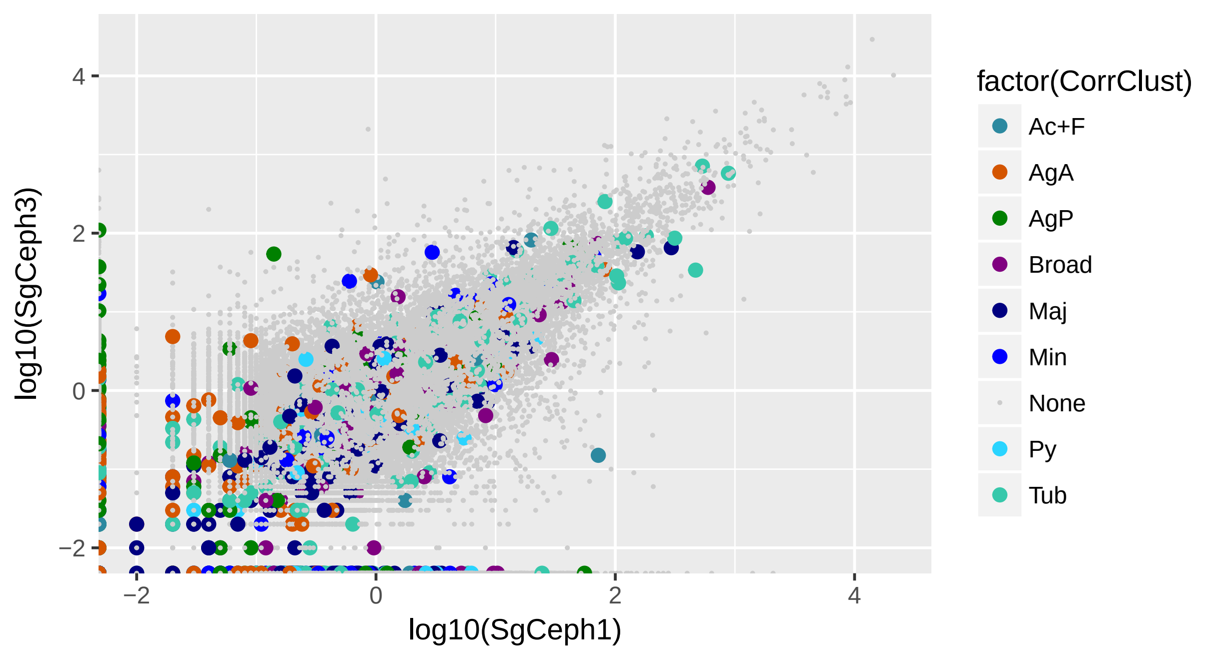

log10(SgCeph3)
4
2
0
−2
−2
0
2
4
log10(SgCeph1)
factor(CorrClust)
Ac+F
AgA
AgP
Broad
Maj
Min
None
Py
Tub

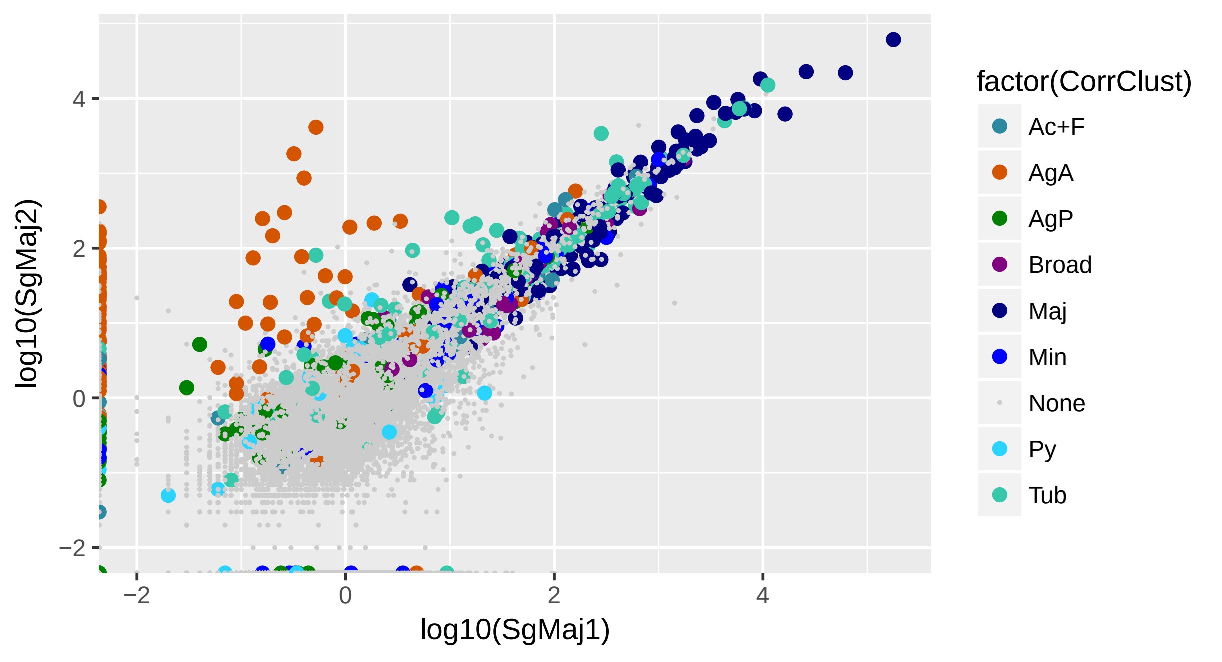

log10(SgMaj2)
4
2
0
−2
−2
0
2
4
log10(SgMaj1)
factor(CorrClust)
Ac+F
AgA
AgP
Broad
Maj
Min
None
Py
Tub

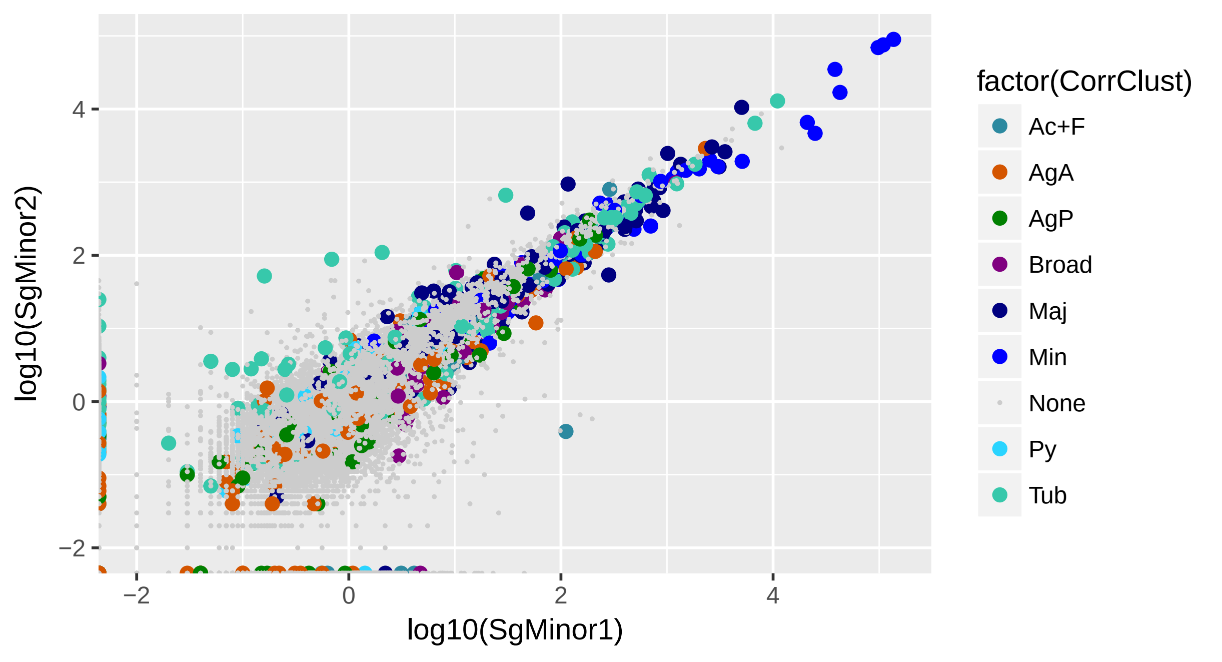
factor(CorrClust)
Ac+F
AgA
AgP
Broad
Maj
Min
None
Py
Tub
log10(SgMinor2)
log10(SgMinor1)
4
2
0
−2
−2
0
2
4

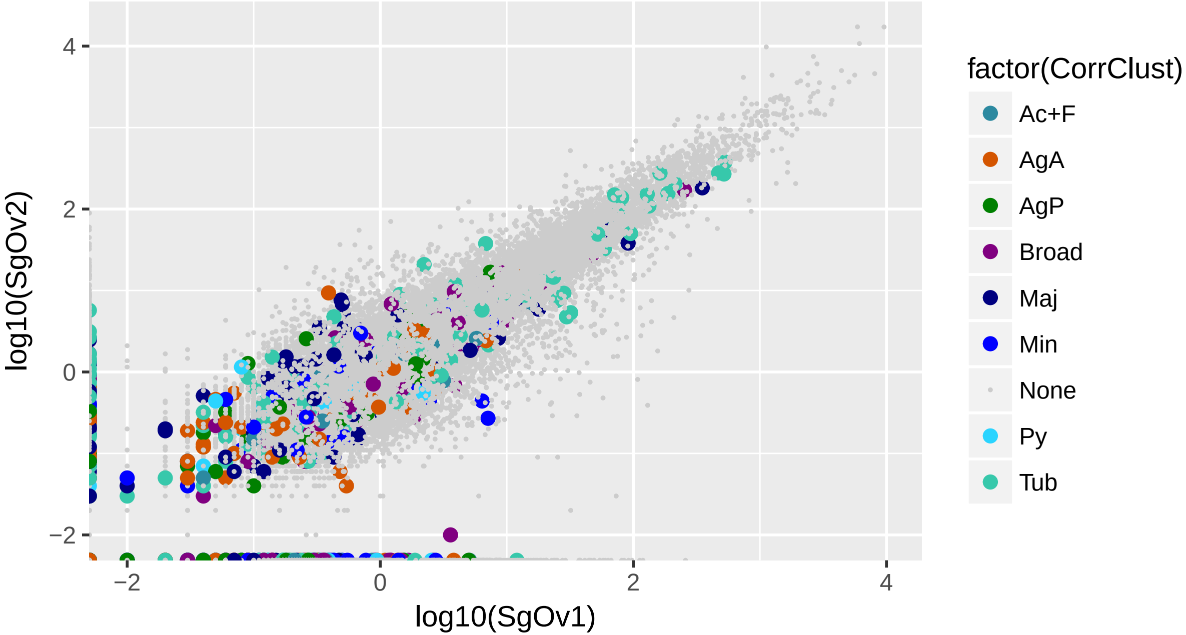

log10(SgOv2)
4
2
0
−2
−2
0
2
4
log10(SgOv1)
factor(CorrClust)
Ac+F
AgA
AgP
Broad
Maj
Min
None
Py
Tub

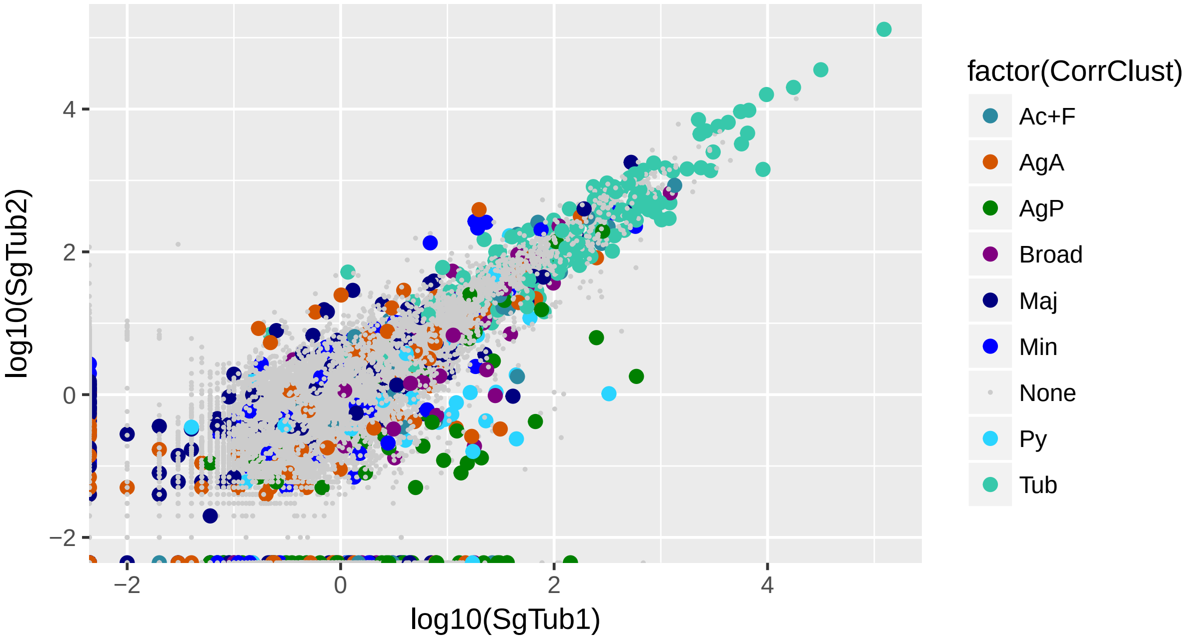
factor(CorrClust)
Ac+F
AgA
AgP
Broad
Maj
Min
None
Py
Tub
log10(SgTub2)
log10(SgTub1)
4
2
0
−2
−2
0
2
4

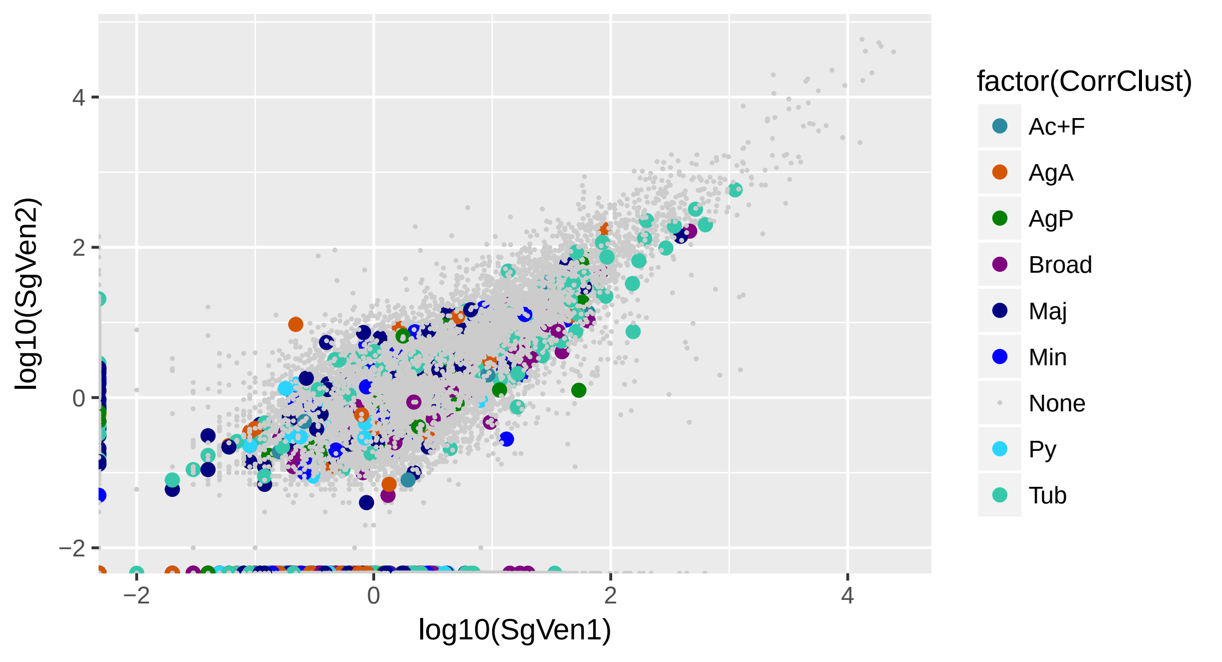
log10(SgVen2)
4
2
0
−2
−2
0
2
4
log10(SgVen1)
factor(CorrClust)
Ac+F
AgA
AgP
Broad
Maj
Min
None
Py
Tub

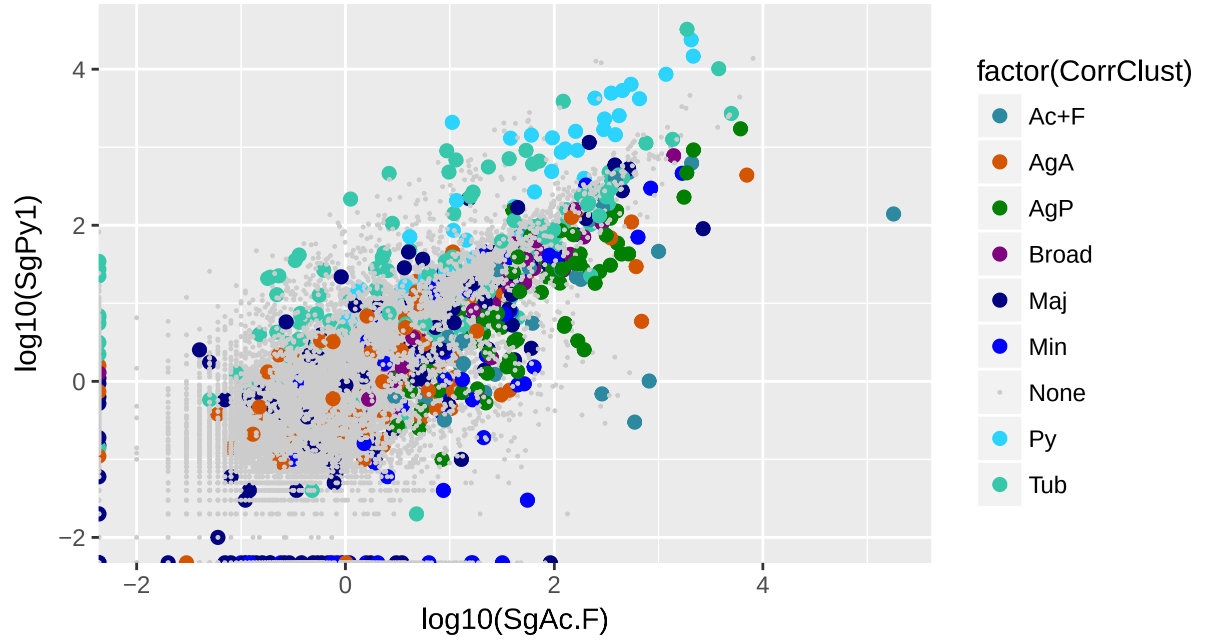
4
2
0
−2
log10(SgPy1)
−2
0
2
4
log10(SgAc.F)
factor(CorrClust)
Ac+F
AgA
AgP
Broad
Maj
Min
None
Py
Tub

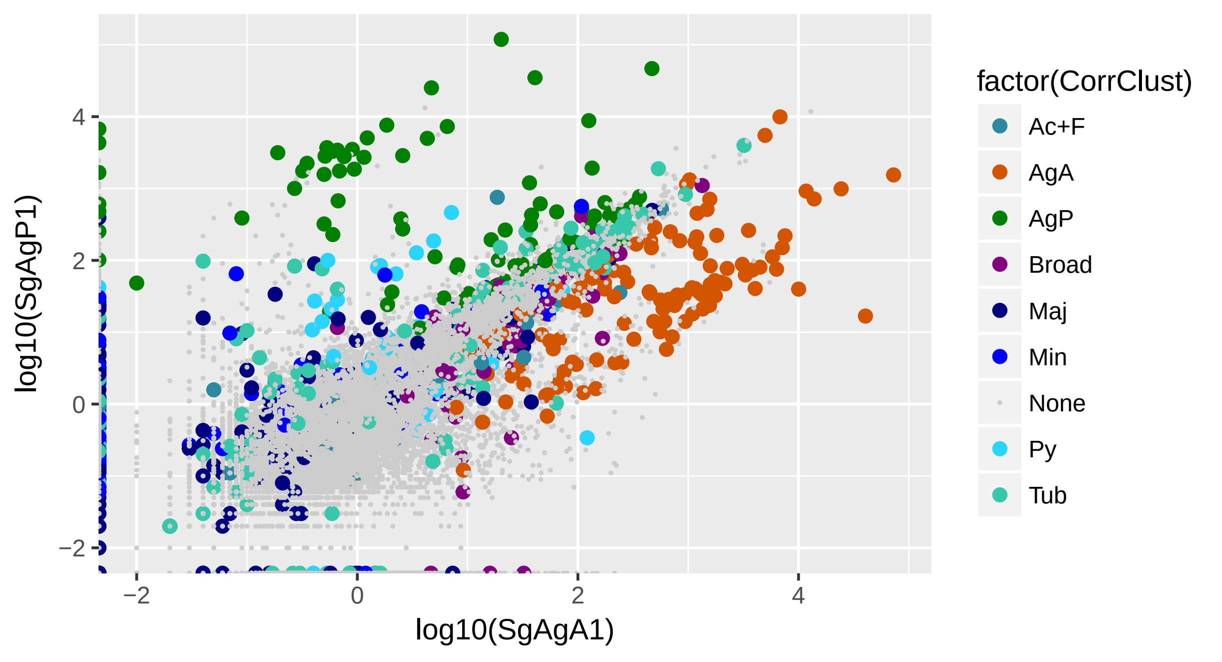

4
2
0
−2
−2
0
2
4
log10(SgAgP1)
log10(SgAgA1)
factor(CorrClust)
Ac+F
AgA
AgP
Broad
Maj
Min
None
Py
Tub
