# Supplementary material for: Evolutionary shifts in gene expression decoupled from gene duplication across functionally distinct spider silk glands
Source: Sci Rep. 2017 Aug 21;7:8393. doi: 10.1038/s41598-017-07388-1 (PMC5566633; doi:10.1038/s41598-017-07388-1)
Supplement: Supplementary file 9 — Supplementary File 8 [file 41598_2017_7388_MOESM9_ESM.docx]

**Supplementary Information**

Supplementary Figures. **A Microsoft Word document containing seven supplementary figures.**

Supplementary File 1. **Transcripts predicted to encode** **known** **structural components of silk fibers or glues**. Microsoft Excel spreadsheets containing all the transcripts that either have a BlastX match to a known silk structural protein (sheet 1) in UniProt or a CRP from Pham et al.^40^ (sheet 2), or are in a BlastClust 3-species cluster with a transcript with such a match. Included in the tables are: best BlastX match information including UniProt ID number, e-value, and description; the protein descriptor used for silk structural annotation in Fig 1, which was either the best UniProt Blastx match or was based on the BlastClust 3-species cluster annotation if there was a not UniProt hit; and the FPKM values for each tissue.

Supplementary File 2. **List of single-gland and multiple gland differentially expressed transcripts.** Tab-delimited text file with a list of all the transcripts with at least one RSEM mapped read showing the mean FPKM in the different silk gland types and non-silk gland tissues, and their inclusion in the set of transcripts deemed over-expressed in all silk glands or in a single gland type, as identified by edgeR. Also shown are the correlation cluster assigned in Figure 4, the presence of a signal peptide, the best UniProt hit, and the orthoMCL group membership.

Supplementary File 3. **Observed and estimated ancestral expression states of OEST gene families.** An Excel spreadsheet listing the gene families and their ancestral states. Shown on the table are the BlastClust 3-species cluster ID, the node identification (transcript ID for the leaf, Yn for a duplication node, Nn for a speciation node), whether there is a 2-fold increase in silk gland expression over non-silk gland expression, associated cluster from Figure 4, whether there has been any change in the silk gland specific expression state over the branch, and the observed or estimated expression levels. Also shown are GO Terms assigned to transcripts (e.g. leaves).

Supplementary File 4. **Ancestral reconstruction of expression states.** A PDF of 605 gene trees. Gene trees were inferred with TreeBest for BlastClust 3-species clusters that contained at least one transcript 2-fold higher in silk glands than non-silk glands and at least three transcripts after pruning low-quality alignments. See Methods for ancestral reconstruction methods; Supplementary File 3 for summary of ancestral states for each gene tree; and Supplementary File 5 for D-values associated with each gene tree. The middle six digits in the transcript names indicate gene tree numbers. Expression level (log10 FPKM) indicated by horizontal width of symbols. Panels to right of transcript names indicate the proportion of total expression in different tissue types in the following order: aciniform and flagelliform (Ac+F), posterior aggregate (AgP), anterior aggregate (AgA), major ampullate (Maj), minor ampullate (Min), pyriform (Py), tubuliform (Tub), and non-silk gland tissues.

Supplementary File 5. **Summary of D-statistics for 605 gene trees.** A Excel spreadsheet containing D-values and associated p-values for 605 gene trees. These gene trees were built for BlastClust 3-species clusters that contained at least one transcript 2-fold higher in silk glands than non-silk gland tissues.

Supplementary File 6. **Counts of OESTs with GO Term assignments.** Excel spreadsheets containing each GO Term with at least one transcript match, the number of OESTs and non-OESTs assigned that GO Term, and whether counts of OESTs assigned to each Go Term differs from the expectation based on counts of all transcripts using a hypergeometric test implemented with GoSeq. Also shown are the counts of OESTs annotated with the GO Term that were assigned to different silk gland types based on correlation clusters (see Fig 4). Separate sheets for each species and all species combined.

Supplementary File 7. **Comparisons of replicate RNA-seq libraries.** A PDF of dot plots comparing normalized FPKM of all transcripts between replicates. OESTs are indicated by the color of the correlation cluster identified in Fig 4. Tissue and species abbreviations as in Fig 1 & 3.
